# Global, regional, and national age-specific progress towards the 2020 milestones of the WHO End TB Strategy: a systematic analysis for the Global Burden of Disease Study 2021

**DOI:** 10.1016/S1473-3099(24)00007-0

**Published:** 2024-07

**Authors:** GBD 2021 Tuberculosis Collaborators, Jorge R Ledesma, Jianing Ma, Meixin Zhang, Ann V L Basting, Huong Thi Chu, Avina Vongpradith, Amanda Novotney, Kate E LeGrand, Yvonne Yiru Xu, Xiaochen Dai, Sneha Ingle Nicholson, Lauryn K Stafford, Austin Carter, Jennifer M Ross, Hedayat Abbastabar, Meriem Abdoun, Deldar Morad Abdulah, Richard Gyan Aboagye, Hassan Abolhassani, Woldu Aberhe Abrha, Hiwa Abubaker Ali, Eman Abu-Gharbieh, Salahdein Aburuz, Isaac Yeboah Addo, Abiola Victor Adepoju, Kishor Adhikari, Qorinah Estiningtyas Sakilah Adnani, Saryia Adra, Abel Afework, Shahin Aghamiri, Williams Agyemang-Duah, Bright Opoku Ahinkorah, Danish Ahmad, Sajjad Ahmad, Amir Mahmoud Ahmadzade, Haroon Ahmed, Mohammed Ahmed, Ayman Ahmed, Karolina Akinosoglou, Tareq Mohammed Ali AL-Ahdal, Nazmul Alam, Mohammed Albashtawy, Mohammad T AlBataineh, Adel Ali Saeed Al-Gheethi, Abid Ali, Endale Alemayehu Ali, Liaqat Ali, Zahid Ali, Syed Shujait Shujait Ali, Kasim Allel, Awais Altaf, Jaffar A Al-Tawfiq, Nelson Alvis-Guzman, Nelson J. Alvis-Zakzuk, Reza Amani, Ganiyu Adeniyi Amusa, Jimoh Amzat, Jason R Andrews, Abhishek Anil, Razique Anwer, Aleksandr Y Aravkin, Damelash Areda, Anton A Artamonov, Raphael Taiwo Aruleba, Mulusew A Asemahagn, Sachin R Atre, Avinash Aujayeb, Davood Azadi, Sina Azadnajafabad, Ahmed Y Azzam, Muhammad Badar, Ashish D Badiye, Sara Bagherieh, Saeed Bahadorikhalili, Atif Amin Baig, Maciej Banach, Biswajit Banik, Mainak Bardhan, Hiba Jawdat Barqawi, Zarrin Basharat, Pritish Baskaran, Saurav Basu, Maryam Beiranvand, Melaku Ashagrie Belete, Makda Abate Belew, Uzma Iqbal Belgaumi, Apostolos Beloukas, Paulo J G Bettencourt, Akshaya Srikanth Bhagavathula, Nikha Bhardwaj, Pankaj Bhardwaj, Ashish Bhargava, Vivek Bhat, Jasvinder Singh Bhatti, Gurjit Kaur Bhatti, Boris Bikbov, Veera R Bitra, Vesna Bjegovic-Mikanovic, Danilo Buonsenso, Katrin Burkart, Yasser Bustanji, Zahid A Butt, Paulo Camargos, Yu Cao, Sinclair Carr, Felix Carvalho, Luca Cegolon, Muthia Cenderadewi, Muge Cevik, Yaacoub Chahine, Vijay Kumar Chattu, Patrick R Ching, Hitesh Chopra, Eunice Chung, Mareli M Claassens, Kaleb Coberly, Natália Cruz-Martins, Bashir Dabo, Sriharsha Dadana, Omid Dadras, Isaac Darban, Jiregna Darega Gela, Aso Mohammad Darwesh, Mahmood Dashti, Berecha Hundessa Demessa, Biniyam Demisse, Solomon Demissie, Awoke Masrie Asrat Derese, Kebede Deribe, Hardik Dineshbhai Desai, Vinoth Gnana Chellaiyan Devanbu, Arkadeep Dhali, Kuldeep Dhama, Sameer Dhingra, Thao Huynh Phuong Do, Deepa Dongarwar, Haneil Larson Dsouza, John Dube, Arkadiusz Marian Dziedzic, Abdelaziz Ed-Dra, Ferry Efendi, Diyan Ermawan Effendi, Aziz Eftekharimehrabad, Nopryan Ekadinata, Temitope Cyrus Ekundayo, Muhammed Elhadi, Legesse Tesfaye Elilo, Theophilus I Emeto, Luchuo Engelbert Bain, Adeniyi Francis Fagbamigbe, Ayesha Fahim, Alireza Feizkhah, Getahun Fetensa, Florian Fischer, Abduzhappar Gaipov, Aravind P Gandhi, Rupesh K Gautam, Miglas W Gebregergis, Mesfin Gebrehiwot, Kahsu Gebrekirstos Gebrekidan, Kazem Ghaffari, Fariba Ghassemi, Ramy Mohamed Ghazy, Amador Goodridge, Anmol Goyal, Shi-Yang Guan, Mesay Dechasa Gudeta, Rashid Abdi Guled, Novianti Br Gultom, Veer Bala Gupta, Vivek Kumar Gupta, Sapna Gupta, Hailey Hagins, Semira Goitom Hailu, Wase Benti Hailu, Samer Hamidi, Asif Hanif, Harapan Harapan, Rumina Syeda Hasan, Shoaib Hassan, Johannes Haubold, Kamal Hezam, Sung Hwi Hong, Nobuyuki Horita, Md. Belal Hossain, Mehdi Hosseinzadeh, Mihaela Hostiuc, Sorin Hostiuc, Hong-Han Huynh, Segun Emmanuel Ibitoye, Kevin S Ikuta, Irena M. Ilic, Milena D. Ilic, Md. Rabiul Islam, Nahlah Elkudssiah Ismail, Faisal Ismail, Abdollah Jafarzadeh, Mihajlo Jakovljevic, Mahsa Jalili, Manthan Dilipkumar Janodia, Nabi Jomehzadeh, Jost B Jonas, Nitin Joseph, Charity Ehimwenma Joshua, Zubair Kabir, Bhushan Dattatray Kamble, Tanuj Kanchan, Himal Kandel, Kehinde Kazeem Kanmodi, Rami S Kantar, Ibraheem M Karaye, Arman Karimi Behnagh, Gebrehiwot G Kassa, Rimple Jeet Kaur, Navjot Kaur, Himanshu Khajuria, Faham Khamesipour, Yusra H Khan, M Nuruzzaman Khan, Mahammed Ziauddin Khan Suheb, Khaled Khatab, Fatemeh Khatami, Min Seo Kim, Soewarta Kosen, Parvaiz A Koul, Sindhura Lakshmi Koulmane Laxminarayana, Kewal Krishan, Burcu Kucuk Bicer, Md Abdul Kuddus, Mukhtar Kulimbet, Nithin Kumar, Dharmesh Kumar Lal, Iván Landires, Kamaluddin Latief, Trang Diep Thanh Le, Thao Thi Thu Le, Caterina Ledda, Munjae Lee, Seung Won Lee, Temesgen L Lerango, Stephen S Lim, Chaojie Liu, Xuefeng Liu, Platon D Lopukhov, Hong Luo, Hengliang Lv, Preetam Bhalchandra Mahajan, Amir Ali Mahboobipour, Azeem Majeed, Elaheh Malakan Rad, Kashish Malhotra, Muhammad Sajeel Ahmed Malik, Lesibana Anthony Malinga, Tauqeer Hussain Mallhi, Aseer Manilal, Bernardo Alfonso Martinez-Guerra, Francisco Rogerlândio Martins-Melo, Roy Rillera Marzo, Hossein Masoumi-Asl, Vasundhara Mathur, Richard James Maude, Ravi Mehrotra, Ziad A Memish, Walter Mendoza, Ritesh G Menezes, Muayad Aghali Merza, Tomislav Mestrovic, Laurette Mhlanga, Sanjeev Misra, Arup Kumar Misra, Prasanna Mithra, Babak Moazen, Hussen Mohammed, Ali H Mokdad, Lorenzo Monasta, Catrin E Moore, Parsa Mousavi, Francesk Mulita, Fungai Musaigwa, Raman Muthusamy, Ahamarshan Jayaraman Nagarajan, Pirouz Naghavi, Ganesh R Naik, Gurudatta Naik, Sanjeev Nair, Tapas Sadasivan Nair, Zuhair S Natto, Biswa Prakash Nayak, Hadush Negash, Dang H Nguyen, Van Thanh Nguyen, Robina Khan Niazi, Chukwudi A Nnaji, Lawrence Achilles Nnyanzi, Efaq Ali Noman, Shuhei Nomura, Bogdan Oancea, Kehinde O Obamiro, Ismail A Odetokun, Daniel Bogale Odo Odo, Oluwakemi Ololade Odukoya, In-Hwan Oh, Chukwuma O Okereke, Osaretin Christabel Okonji, Eyal Oren, Edgar Ortiz-Brizuela, Uchechukwu Levi Osuagwu, Amel Ouyahia, Mahesh Padukudru P A, Pragyan Paramita Parija, Romil R Parikh, Seoyeon Park, Ashwaghosha Parthasarathi, Shankargouda Patil, Shrikant Pawar, Minjin Peng, Veincent Christian Filipino Pepito, Prince Peprah, João Perdigão, Norberto Perico, Hoang Tran Pham, Maarten J Postma, Attur Ravindra Attur Prabhu, Manya Prasad, Akila Prashant, Elton Junio Sady Prates, Fakher Rahim, Mosiur Rahman, Muhammad Aziz Rahman, Masoud Rahmati, Sathish Rajaa, Shakthi Kumaran Ramasamy, Indu Ramachandra Rao, Sowmya J Rao, Deepthi Rapaka, Ahmed Mustafa Rashid, Zubair Ahmed Ratan, Nakul Ravikumar, Salman Rawaf, Murali Mohan Rama Krishna Reddy, Elrashdy Moustafa Mohamed Redwan, Giuseppe Remuzzi, Luis Felipe Reyes, Nazila Rezaei, Mohsen Rezaeian, Omid Rezahosseini, Mónica Rodrigues, Priyanka Roy, Guilherme de Andrade Ruela, Siamak Sabour, Basema Saddik, Umar Saeed, Sher Zaman Safi, Narjes Saheb Sharif-Askari, Fatemeh Saheb Sharif-Askari, Amirhossein Sahebkar, Biniyam Sahiledengle, Soumya Swaroop Sahoo, Nasir Salam, Afeez Abolarinwa Salami, Samreen Saleem, Mohamed A Saleh, Hossein Samadi Kafil, Sara Samadzadeh, Yoseph Leonardo Samodra, Rama Krishna Sanjeev, Aswini Saravanan, Susan M Sawyer, Siddharthan Selvaraj, Sabyasachi Senapati, Subramanian Senthilkumaran, Pritik A Shah, Samiah Shahid, Masood Ali Shaikh, Sunder Sham, Mohammad Ali Shamshirgaran, Mohd Shanawaz, Medha Sharath, Samendra P Sherchan, Ranjitha S Shetty, Hesamaddin Shirzad-Aski, Aminu Shittu, Emmanuel Edwar Siddig, João Pedro Silva, Surjit Singh, Paramdeep Singh, Harpreet Singh, Jasvinder A Singh, Md Shahjahan Siraj, Siswanto Siswanto, Ranjan Solanki, Yonatan Solomon, Joan B Soriano, Chandrashekhar T Sreeramareddy, Vijay Kumar Srivastava, Paschalis Steiropoulos, Chandan Kumar Swain, Takahiro Tabuchi, Mircea Tampa, Jacques JL Lukenze Tamuzi, Nathan Y Tat, Razieh Tavakoli Oliaee, Gebrehiwot Teklay, Edosa Geta Tesfaye, Belay Tessema, Pugazhenthan Thangaraju, Rekha Thapar, Chern Choong Chern Thum, Jansje Henny Vera Ticoalu, Imad M Tleyjeh, Ruoyan Tobe-Gai, Temesgen Mohammed Toma, Khai Hoan Tram, Aniefiok John Udoakang, Tungki Pratama Umar, Chukwuma David Umeokonkwo, Seyed Mohammad Vahabi, Asokan Govindaraj Vaithinathan, Job F M van Boven, Shoban Babu Varthya, Ziyue Wang, Muktar S A Warsame, Ronny Westerman, Tewodros Eshete Wonde, Sajad Yaghoubi, Siyan Yi, Vahit Yiğit, Dong Keon Yon, Naohiro Yonemoto, Chuanhua Yu, Fathiah Zakham, Moein Zangiabadian, Francis Zeukeng, Haijun Zhang, Yang Zhao, Peng Zheng, Magdalena Zielińska, Joshua A Salomon, Robert C Reiner Jr, Mohsen Naghavi, Theo Vos, Simon I Hay, Christopher J L Murray, Hmwe Hmwe Kyu

## Abstract

**Background:**

Global evaluations of the progress towards the WHO End TB Strategy 2020 interim milestones on mortality (35% reduction) and incidence (20% reduction) have not been age specific. We aimed to assess global, regional, and national-level burdens of and trends in tuberculosis and its risk factors across five separate age groups, from 1990 to 2021, and to report on age-specific progress between 2015 and 2020.

**Methods:**

We used the Global Burden of Diseases, Injuries, and Risk Factors Study 2021 (GBD 2021) analytical framework to compute age-specific tuberculosis mortality and incidence estimates for 204 countries and territories (1990–2021 inclusive). We quantified tuberculosis mortality among individuals without HIV co-infection using 22 603 site-years of vital registration data, 1718 site-years of verbal autopsy data, 825 site-years of sample-based vital registration data, 680 site-years of mortality surveillance data, and 9 site-years of minimally invasive tissue sample (MITS) diagnoses data as inputs into the Cause of Death Ensemble modelling platform. Age-specific HIV and tuberculosis deaths were established with a population attributable fraction approach. We analysed all available population-based data sources, including prevalence surveys, annual case notifications, tuberculin surveys, and tuberculosis mortality, in DisMod-MR 2.1 to produce internally consistent age-specific estimates of tuberculosis incidence, prevalence, and mortality. We also estimated age-specific tuberculosis mortality without HIV co-infection that is attributable to the independent and combined effects of three risk factors (smoking, alcohol use, and diabetes). As a secondary analysis, we examined the potential impact of the COVID-19 pandemic on tuberculosis mortality without HIV co-infection by comparing expected tuberculosis deaths, modelled with trends in tuberculosis deaths from 2015 to 2019 in vital registration data, with observed tuberculosis deaths in 2020 and 2021 for countries with available cause-specific mortality data.

**Findings:**

We estimated 9·40 million (95% uncertainty interval [UI] 8·36 to 10·5) tuberculosis incident cases and 1·35 million (1·23 to 1·52) deaths due to tuberculosis in 2021. At the global level, the all-age tuberculosis incidence rate declined by 6·26% (5·27 to 7·25) between 2015 and 2020 (the WHO End TB strategy evaluation period). 15 of 204 countries achieved a 20% decrease in all-age tuberculosis incidence between 2015 and 2020, eight of which were in western sub-Saharan Africa. When stratified by age, global tuberculosis incidence rates decreased by 16·5% (14·8 to 18·4) in children younger than 5 years, 16·2% (14·2 to 17·9) in those aged 5–14 years, 6·29% (5·05 to 7·70) in those aged 15–49 years, 5·72% (4·02 to 7·39) in those aged 50–69 years, and 8·48% (6·74 to 10·4) in those aged 70 years and older, from 2015 to 2020. Global tuberculosis deaths decreased by 11·9% (5·77 to 17·0) from 2015 to 2020. 17 countries attained a 35% reduction in deaths due to tuberculosis between 2015 and 2020, most of which were in eastern Europe (six countries) and central Europe (four countries). There was variable progress by age: a 35·3% (26·7 to 41·7) decrease in tuberculosis deaths in children younger than 5 years, a 29·5% (25·5 to 34·1) decrease in those aged 5–14 years, a 15·2% (10·0 to 20·2) decrease in those aged 15–49 years, a 7·97% (0·472 to 14·1) decrease in those aged 50–69 years, and a 3·29% (–5·56 to 9·07) decrease in those aged 70 years and older. Removing the combined effects of the three attributable risk factors would have reduced the number of all-age tuberculosis deaths from 1·39 million (1·28 to 1·54) to 1·00 million (0·703 to 1·23) in 2020, representing a 36·5% (21·5 to 54·8) reduction in tuberculosis deaths compared to those observed in 2015. 41 countries were included in our analysis of the impact of the COVID-19 pandemic on tuberculosis deaths without HIV co-infection in 2020, and 20 countries were included in the analysis for 2021. In 2020, 50 900 (95% CI 49 700 to 52 400) deaths were expected across all ages, compared to an observed 45 500 deaths, corresponding to 5340 (4070 to 6920) fewer deaths; in 2021, 39 600 (38 300 to 41 100) deaths were expected across all ages compared to an observed 39 000 deaths, corresponding to 657 (–713 to 2180) fewer deaths.

**Interpretation:**

Despite accelerated progress in reducing the global burden of tuberculosis in the past decade, the world did not attain the first interim milestones of the WHO End TB Strategy in 2020. The pace of decline has been unequal with respect to age, with older adults (ie, those aged >50 years) having the slowest progress. As countries refine their national tuberculosis programmes and recalibrate for achieving the 2035 targets, they could consider learning from the strategies of countries that achieved the 2020 milestones, as well as consider targeted interventions to improve outcomes in older age groups.

**Funding:**

Bill & Melinda Gates Foundation.

## Introduction

Despite being a preventable and largely curable disease, tuberculosis remains a major contributor to the global burden of disease. Tuberculosis causes more than 1 million deaths annually and was the leading cause of death due to a single infectious agent in 2019.^1^ Since the early 1990s, global initiatives to address tuberculosis have grown more prominent, resulting in declines in the global burden of this disease.^2^ Recent progress has been unimpressive, however, as global annual rates of decline in tuberculosis incidence over the past 10 years have ranged between 1% and 2%.^3^, ^4^ The WHO End TB Strategy represents a renewed global resolve to accelerate progress by aiming to reduce deaths due to tuberculosis by 95% and cut tuberculosis incidence by 90% between 2015 and 2035, while also seeking to have zero tuberculosis-affected households experiencing catastrophic financial costs due to the disease by 2035.^5^ Rigorous evaluations of the trends in the global burden of tuberculosis are therefore crucial for assessing progress towards achieving the End TB Strategy targets and identifying specific drivers of marked national progress.

**Research in context**

**Evidence before this study**

In the context of the WHO End TB Strategy, several groups have generated estimates of tuberculosis incidence and mortality, including the WHO Global Tuberculosis Programme and the Global Burden of Diseases, Injuries, and Risk Factors Study (GBD), to monitor global and national-level progress towards eliminating tuberculosis. 2020 marked a crucial year for evaluating progress as the first quantitative milestones of the End TB Strategy passed. We searched PubMed with the search string “(“tuberculosis” OR “TB”) AND (“burden” OR “estimates”) AND (“End TB”) AND (“target*” OR “milestone*”)”, with no language restrictions, for publications from Jan 1, 2015, to Dec 1, 2023. Our search identified seven studies presenting population-based tuberculosis burden estimates to assess progress towards the End TB targets for subsets of countries; of these, only one study examined differences in progress by age. The single study analysing progress by age used GBD 2019 tuberculosis estimates to evaluate temporal trends in Cambodia. Additionally, the 2022 Global Tuberculosis Report from WHO illustrated that tuberculosis deaths decreased by only 5·9%, while the tuberculosis incidence rate dropped by 10%, between 2015 and 2021, falling well short of the targeted milestones. However, as yet, no global, systematic, age-specific study has been done to investigate progress towards the 2020 End TB Strategy mortality and incidence milestones, which outlined a 35% reduction in tuberculosis deaths and 20% reduction in the tuberculosis incidence rate between 2015 and 2020, with additional assessments of the role of key risk factors in achieving these milestones.

**Added value of this study**

We comprehensively examined the burden of tuberculosis for five separate age groups in 204 countries and territories from 1990 to 2021. We focused on examining temporal trends in tuberculosis incidence and mortality from 2015 to 2020 to assess global progress towards the End TB Strategy milestones, with an emphasis on exploring progress by age for the first time. We also present age-specific, risk-deleted mortality estimates that represent deaths due to tuberculosis that would have been observed if the combined effects of all evaluated risk factors were removed, to provide insights into the role of addressing risk factors in achieving the End TB targets. Last, we analysed vital registration data only (2015–21 inclusive) to assess the potential impact of the COVID-19 pandemic on age-specific tuberculosis mortality in countries with available cause-specific data.

**Implications of all the available evidence**

Global tuberculosis control programmes could consider closely examining the 15 countries that achieved the 2020 incidence milestone and the 17 countries that achieved the 2020 mortality milestone, many of which were in western sub-Saharan Africa and eastern Europe, to better understand drivers of their marked progress. Many of these countries have implemented innovative approaches to active case finding while implementing social protection interventions such as advanced tuberculosis surveillance in high-risk areas and economic incentives for patients. We observed unequal progress by age groups and found particularly slow progress in reducing the tuberculosis burden in older adults (aged >50 years), indicating that tuberculosis in this age group should be more widely recognised and monitored to achieve WHO's 2035 End TB Strategy targets. One approach for reducing the tuberculosis burden in older adults and achieving the 2035 End TB targets is addressing risk factors for tuberculosis. Our assessment of risk-deleted mortality suggests that the world would have achieved the 2020 End TB mortality milestone, with a 36% reduction in global tuberculosis deaths and the largest benefits in older adults, if the risk factors of smoking, alcohol use, and diabetes were removed. Although complete elimination of such risk factors is highly unlikely, this assessment illustrates the magnitude of potential improvement. Finally, our secondary analysis of countries with complete vital registration data showed variable impacts of the COVID-19 pandemic on tuberculosis mortality in 2020 and 2021; some countries and age groups reported fewer than expected deaths due to tuberculosis, whereas others reported more deaths than expected. Additional data from countries with a high tuberculosis burden are urgently needed to understand the generalisability of these findings.

The year 2020 marked a crucial timepoint for evaluating progress towards eliminating tuberculosis. The first quantitative milestones in the End TB Strategy called for a 35% reduction in deaths due to tuberculosis and a 20% reduction in the tuberculosis incidence rate by 2020.^5^ The 2022 Global Tuberculosis Report from WHO illustrated that tuberculosis deaths decreased by only 5·9%, while the tuberculosis incidence rate decreased by 10%, between 2015 and 2021, falling well short of the 2020 milestones.^6^ Moreover, studies examining global trends towards WHO targets have yet to fully consider age-specific differences in progress. The current evidence suggests that although the tuberculosis burden remains substantially high in children,^7^, ^8^ older age groups generally have the highest rates of tuberculosis mortality and incidence.^9^, ^10^ A report of the Global Burden of Diseases, Injuries, and Risk Factors Study 2019 (GBD 2019) based on data from Cambodia also indicated that temporal trends of reductions in the tuberculosis burden become slower with increasing age.^11^ Several factors could explain these marked age-related differences, including age-specific diagnostic challenges,^12^, ^13^ age-specific mixing patterns,^14^ immune senescence^15^ and other age-related immune dysfunctions,^16^ and comorbidities common in older patients masking tuberculosis symptoms.^17^ Although largely unexplored at the global level, age-specific differences in risk factor prevalence might further augment these age-related discrepancies. Identification of age-specific progress towards the End TB Strategy milestones, combined with assessments of the role of key risk factors, could therefore help inform targeted interventions to accelerate progress towards eliminating tuberculosis.

Leveraging the GBD 2021 framework, we aimed to examine the levels and trends in the global burden of tuberculosis to investigate age-specific attainment of the 2020 WHO End TB Strategy mortality and incidence milestones for 204 countries and territories. Considering that modifiable tuberculosis risk factors, such as alcohol consumption, smoking, and diabetes, can augment preventive interventions, we supplemented our analysis by examining age-specific risk-deleted tuberculosis mortality (ie, the tuberculosis mortality rate that would have been observed if risk factors for tuberculosis mortality were removed) to highlight the need to address reductions in risk factor exposure as part of any holistic response to achieving the End TB Strategy targets. Last, we examined the potential impact of the COVID-19 pandemic on tuberculosis mortality by drawing on vital registration data from those countries with available data. This manuscript was produced as part of the GBD Collaborator Network and in accordance with the GBD Protocol.

## Methods

### Overview

We have previously published detailed methods of the GBD analytical framework^18^, ^19^ and tuberculosis burden estimation in GBD.^1^, ^3^, ^11^ Here, we summarise the methodology for estimating tuberculosis mortality and morbidity, as well as key risk factors, with more detailed descriptions of the modelling strategy provided in appendix 1 (pp 3–26). In compliance with the Guidelines for Accurate and Transparent Health Estimates Reporting, input data sources and codes for each step of the estimation process are available on the Global Health Data Exchange.

### Tuberculosis mortality

The GBD 2021 Cause of Death database contained all available vital registration, surveillance system, and verbal autopsy data from 1980 to 2020. Leveraging the database, we included 22 603 site-years of vital registration data, 825 site-years of sample-based vital registration data, 680 site-years of mortality surveillance data, and 9 site-years of minimally invasive tissue sample (MITS) diagnoses data for modelling tuberculosis mortality among individuals without HIV co-infection. Country-specific data sources and citations are included in the Global Health Data Exchange. We processed raw cause of death data to account for completeness and differences in coding schemes, to redistribute deaths from unspecified codes to more specific underlying causes of death,^20^ and to reassign misclassified HIV deaths.

We used the Cause of Death Ensemble modelling (CODEm) strategy to generate tuberculosis mortality estimates in individuals without HIV co-infection by location, year, age, and sex. CODEm is a hierarchical modelling platform that uses an ensemble of different modelling methods for rates or cause fractions with varying choices of covariates (eg, smoking prevalence, alcohol consumption, and Healthcare Access and Quality [HAQ] Index) that perform best with out-of-sample predictive validity testing.^21^ appendix 1 (p 13) provides example countries illustrating that final tuberculosis mortality estimates presented throughout this analysis largely follow trends in input data. Finally, we established age-specific tuberculosis deaths among individuals with HIV infection using a population attributable fraction (PAF) approach (appendix 1 pp 14–15).

### Tuberculosis morbidity

We simultaneously modelled age-sex-specific tuberculosis incidence, prevalence, and cause-specific mortality using the DisMod-MR 2.1 (disease-model-Bayesian meta-regression) modelling tool. DisMod-MR 2.1 is a Bayesian disease modelling tool that leverages all available morbidity and mortality data, the epidemiological relationships between disease parameters, and spatial relationships to output internally consistent disease burden estimates.^22^ We provide details of case definitions, input data sources, and data processing strategies in appendix 1 (pp 2–3, 16–26).

Briefly, we identified all population-based tuberculosis prevalence surveys via comprehensive reviews of the literature. Similarly to previous GBD iterations, we used a Bayesian meta-regression tool^23^ to adjust prevalence surveys that used smear-positive tuberculosis as the case definition rather than bacteriologically confirmed tuberculosis. We further recalibrated surveys that used symptoms only as the screening method over both symptoms and chest X-ray (appendix 1 pp 22–23). Next, we maximised data informing DisMod-MR by predicting age-sex-specific incidence for countries with low-quality data ratings for cause of death data. We estimated incidence for these countries through a mortality-to-incidence ratio approach using countries with high-quality data ratings on cause of death data as inputs into a Bayesian meta-regression analysis^23^ where the HAQ Index^24^ was the primary covariate. We then linked location-specific predicted mortality-to-incidence ratios with tuberculosis death estimates to obtain estimated age-sex-specific incidence (appendix 1 p 20).

We subsequently modelled these data, together with age-sex-specific case notifications for locations with high-quality data ratings on causes of death data, population-based tuberculin surveys, and estimates of tuberculosis excess mortality rate, remission, and cause-specific mortality rate, in DisMod-MR 2.1 to generate all-form tuberculosis morbidity estimates that were internally consistent. To further improve internal consistency of modelling in DisMod, we computed all-age and both-sexes priors of tuberculosis duration using a combination of tuberculosis duration data from a systematic review of studies during the pre-chemotherapy era^25^ and the HAQ Index. We then used DisMod's statistical triangulation approach, using the estimated tuberculosis duration priors and age-sex-specific tuberculosis mortality, prevalence, and incidence data, to derive final age-sex-specific tuberculosis duration estimates (appendix 1 pp 27–28). Finally, we disaggregated tuberculosis incidence by HIV status by applying the fraction of new all-form tuberculosis cases that were HIV and tuberculosis co-infections to our all-form tuberculosis cases estimated from DisMod-MR.

### Risk factor analysis

GBD has previously published detailed methodology for risk factor estimation.^26^ In summary, age-sex-specific PAFs in adults were computed within the comparative risk assessments framework of GBD with the following inputs: prevalence estimates for exposure to risk factors; the relative risk of tuberculosis mortality as an outcome of exposure to each risk factor; and the theoretical minimum risk exposure level (TMREL), defined as the level of exposure that would minimise risk for each individual in a population. We computed tuberculosis mortality attributable in adults (aged ≥15 years) to each risk factor by multiplying the PAF by the number of tuberculosis deaths for each risk–outcome pair. We subsequently derived risk-deleted mortality rates to quantify tuberculosis deaths that would have been observed had the risk factors been set to their corresponding TMRELs. The risk-deleted tuberculosis mortality rates are therefore computed by multiplying the observed tuberculosis mortality by one minus the PAF for a risk factor or combination of risk factors. The objective of the risk-deleted analysis is to illustrate the potential magnitude of addressing tuberculosis risk factors across countries and age groups. For this analysis, we quantified tuberculosis PAFs for alcohol consumption,^27^ smoking,^28^ and diabetes^29^ among adults without HIV co-infection. We provide complete details of the methods for estimation of the tuberculosis risk factors in appendix 1 (pp 39–67).

### Impact of the COVID-19 pandemic on tuberculosis mortality

We evaluated the potential age-specific impact of the COVID-19 pandemic on tuberculosis mortality without HIV co-infection using vital registration data for all countries with available cause-specific mortality data in 2020 and 2021. This secondary analysis included data from 41 countries that reported at least ten tuberculosis deaths in 2019 for each age group of interest, representing four of the seven GBD super-regions (appendix 2 pp 300–04), with 15 countries from the high-income super-region, 14 in central Europe, eastern Europe, and central Asia, ten in Latin America and the Caribbean, and two in southeast Asia, east Asia, and Oceania that had sufficient cause-specific mortality data. Using these data as inputs, we fitted quasi-Poission regression models to data from 2015 to 2019 with population size as an offset to estimate the expected number of tuberculosis deaths in 2020–21 for each country. The quasi-likelihood was selected to account for overdispersion in count data. This analysis was stratified by adults and children combined (aged <65 years), elderly individuals (≥65 years), and all ages combined. We selected these age groups on the basis of evidence illustrating that global tuberculosis diagnoses during the pandemic declined relatively more in individuals aged 65 years and older than in those younger than 65 years.^30^ We subsequently compared age-specific observed tuberculosis deaths to predicted tuberculosis deaths in 2020 and 2021 to identify potential excess tuberculosis deaths. Comparisons were made by computing the difference in observed to expected tuberculosis deaths (excess tuberculosis deaths) and the respective ratio.

### Data presentation

We aggregated tuberculosis incidence and mortality estimates by HIV status to present all-form tuberculosis burden estimates for five age groups (<5 years, 5–14 years, 15–49 years, 50–69 years, and ≥70 years) throughout this analysis. Next, we used the GBD world population age standard to derive age-standardised rates for all-form tuberculosis incidence and mortality. For changes over time, we provide annualised rates of change (ARC) as the difference in the natural log of the incidence and mortality rates at the start and end of the time interval divided by the number of years in the interval. We present tuberculosis incidence-specific and mortality-specific ARCs for 1990–2010 and 2010–21. To evaluate attainment of the 2020 WHO End TB interim milestones, we derived percentage changes in all-form tuberculosis rates and counts of both incidence and mortality from 2015 to 2020. At each modelling step described, parameter uncertainty was incorporated by randomly drawing 500 samples from each age-sex-location-year-specific parameter distribution and propagating this uncertainty forward across each step of the analysis. Consistent with the GBD framework, we computed 95% uncertainty intervals (UIs) for all estimates based on the 2·5th and 97·5th percentiles of the final 500 draws. More detailed tuberculosis burden results by HIV status, age, and sex across locations and years are available in the GBD Results Tool. Counts are rounded to three significant figures while rates and percentages are rounded to one decimal place, except where greater precision was needed to differentiate between values.

### Role of the funding source

The funder of the study had no role in study design, data collection, data analysis, data interpretation, writing of the report, or the decision to submit the manuscript for publication.

## Results

### Overview

In 2021, we estimated that there were 9·40 million (95% UI 8·36–10·5) tuberculosis incident cases and 1·35 million (1·23–1·52) deaths due to tuberculosis globally (table 1). HIV and tuberculosis co-infection constituted 1·00 million (0·90–1·13; 10·7% [10·3–11·0]) of the 9·40 million all-form tuberculosis incident cases and 205 000 (158 000–248 000; 15·1% [11·9–17·7]) of the 1·35 million all-form global tuberculosis deaths. The age-standardised incidence rate for all-form tuberculosis was 115 (102–128) per 100 000 population and the age-standardised mortality rate was 16·2 (14·8–18·2) per 100 000 population in 2021 (table 1). From 1990 to 2021, the global age-standardised tuberculosis incidence rate decreased by 37·0% (34·5–39·1), while the global age-standardised tuberculosis mortality rate decreased by 61·1% (52·6–66·3).

**Table 1:** Age-specific all-form tuberculosis incident cases and deaths with corresponding rates per 100 000 population by GBD super-region (2021)

	**Number of cases**	**Cases per 100 000 population**	**Annualised rate of change in tuberculosis incidence (1990–2010), %**	**Annualised rate of change in tuberculosis incidence (2010–21), %**	**Number of deaths**	**Deaths per 100 000 population**	**Annualised rate of change in tuberculosis deaths (1990–2010), %**	**Annualised rate of change in tuberculosis deaths (2010–21), %**
**Global**								
All ages	9 400 000 (8 360 000 to 10 500 000)	115 (102 to 128)	−0·734% (−0·914 to −0·523)	−1·94% (−2·09 to −1·78)	1 350 000 (1 230 000 to 1 520 000)	16·2 (14·8 to 18·2)	−1·40% (−1·82 to −0·928)	−4·01% (−4·84 to −2·96)
<5 years	354 000 (298 000 to 436 000)	53·8 (45·3 to 66·3)	−1·56% (−1·91 to −1·29)	−4·05% (−4·32 to −3·77)	60 300 (43 500 to 76 700)	9·16 (6·61 to 11·7)	−3·09% (−3·68 to −2·52)	−7·85% (−9·63 to −5·84)
5–14 years	446 000 (315 000 to 623 000)	33·0 (23·3 to 46·0)	−1·48% (−1·79 to −1·10)	−3·71% (−4·03 to −3·41)	20 900 (18 300 to 24 000)	1·55 (1·36 to 1·78)	−1·22% (−1·80 to −0·547)	−6·60% (−7·71 to −5·57)
15–49 years	5160 000 (4380 000 to 6180 000)	131 (111 to 157)	−0·596% (−0·842 to −0·336)	−1·90% (−2·09 to −1·74)	491 000 (439 000 to 546 000)	12·4 (11·1 to 13·8)	−0·400% (−0·873 to 0·0442)	−4·72% (−5·50 to −3·87)
50–69 years	2490 000 (1960 000 to 3100 000)	173 (137 to 216)	−1·56% (−1·81 to −1·28)	−2·12% (−2·33 to −1·96)	448 000 (404 000 to 510 000)	31·2 (28·1 to 35·5)	−2·79% (−3·23 to −2·22)	−4·36% (−5·23 to −2·99)
≥70 years	949 000 (770 000 to 1150 000)	192 (156 to 234)	−1·63% (−1·92 to −1·37)	−2·29% (−2·52 to −2·06)	331 000 (302 000 to 379 000)	67·0 (61·1 to 76·6)	−2·88% (−3·41 to −2·25)	−3·96% (−4·80 to −2·63)
**Central Europe, eastern Europe, and central Asia**								
All ages	219 000 (188 000 to 262 000)	46·9 (40·3 to 56·2)	−0·0580% (−0·374 to 0·249)	−4·28% (−4·79 to −3·80)	15 900 (14 600 to 17 300)	3·01 (2·77 to 3·29)	1·60% (1·40 to 1·78)	−8·65% (−9·32 to −8·08)
<5 years	2260 (1860 to 2900)	8·78 (7·25 to 11·3)	−2·07% (−2·44 to −1·68)	−4·94% (−5·50 to −4·43)	334 (267 to 441)	1·30 (1·04 to 1·72)	−3·07% (−3·77 to −2·42)	−6·90% (−8·85 to −4·68)
5–14 years	6020 (4140 to 8540)	10·9 (7·52 to 15·5)	−1·13% (−1·58 to −0·659)	−4·43% (−5·09 to −3·52)	66·2 (59·4 to 75·1)	0·120 (0·108 to 0·136)	−0·0316% (−0·505 to 0·385)	−7·86% (−8·68 to −6·90)
15–49 years	140 000 (113 000 to 175 000)	70·9 (57·3 to 88·6)	−0·0144% (−0·432 to 0·363)	−4·12% (−4·80 to −3·46)	6900 (6260 to 7800)	3·49 (3·17 to 3·94)	3·40% (3·19 to 3·67)	−9·40% (−10·0 to −8·89)
50–69 years	55 900 (42 100 to 72 900)	56·0 (42·2 to 73·1)	−0·807% (−1·27 to −0·285)	−3·77% (−4·39 to −3·10)	6410 (5850 to 7030)	6·42 (5·86 to 7·05)	0·238% (0·0892 to 0·388)	−8·83% (−9·67 to −7·94)
≥70 years	14 300 (11 200 to 18 000)	36·0 (28·2 to 45·5)	−1·76% (−2·14 to −1·40)	−3·61% (−4·19 to −3·08)	2150 (2040 to 2270)	5·42 (5·16 to 5·73)	−2·68% (−2·81 to −2·53)	−5·32% (−5·74 to −4·90)
**High income**								
All ages	99 300 (87 900 to 114 000)	7·29 (6·36 to 8·56)	−2·94% (−3·18 to −2·69)	−1·93% (−2·16 to −1·69)	12 700 (11 200 to 13 900)	0·576 (0·519 to 0·630)	−4·45% (−4·86 to −4·22)	−2·11% (−2·42 to −1·76)
<5 years	937 (727 to 1190)	1·73 (1·34 to 2·20)	−2·33% (−2·74 to −1·94)	−3·42% (−3·70 to −2·94)	24·2 (20·0 to 28·4)	0·0445 (0·0368 to 0·0524)	−8·40% (−8·93 to −7·92)	−6·05% (−6·79 to −5·35)
5–14 years	2370 (1590 to 3440)	1·94 (1·30 to 2·81)	−2·92% (−3·37 to −2·49)	−2·41% (−2·79 to −1·98)	19·2 (16·3 to 22·1)	0·0157 (0·0133 to 0·0181)	−9·11% (−9·80 to −8·34)	−4·29% (−4·85 to −3·71)
15–49 years	40 900 (33 500 to 50 900)	8·45 (6·91 to 10·5)	−3·04% (−3·44 to −2·69)	−2·52% (−2·74 to −2·30)	1470 (1170 to 1780)	0·303 (0·241 to 0·367)	−6·50% (−6·80 to −6·24)	−3·86% (−4·23 to −3·47)
50–69 years	27 600 (21 000 to 34 300)	9·94 (7·58 to 12·4)	−3·84% (−4·37 to −3·32)	−2·05% (−2·45 to −1·72)	2330 (2090 to 2520)	0·839 (0·754 to 0·908)	−7·28% (−7·68 to −6·91)	−3·83% (−4·38 to −3·44)
≥70 years	27 500 (22 600 to 33 900)	17·9 (14·8 to 22·1)	−3·20% (−3·57 to −2·91)	−2·13% (−2·59 to −1·66)	8820 (7290 to 9740)	5·76 (4·76 to 6·36)	−4·14% (−4·63 to −3·86)	−3·24% (−3·65 to −2·79)
**Latin America and Caribbean**								
All ages	207 000 (181 000 to 239 000)	33·1 (29·0 to 38·0)	−2·25% (−2·51 to −1·94)	−0·870% (−1·17 to −0·506)	24 200 (20 600 to 29 100)	3·87 (3·30 to 4·65)	−4·17% (−4·59 to −3·70)	−2·28% (−2·96 to −1·45)
<5 years	4040 (3320 to 5020)	8·54 (7·01 to 10·6)	−4·95% (−5·26 to −4·56)	−2·46% (−2·90 to −2·04)	552 (442 to 709)	1·17 (0·934 to 1·50)	−8·14% (−8·96 to −7·42)	−5·60% (−7·12 to −3·88)
5–14 years	7520 (5280 to 10 600)	7·84 (5·50 to 11·0)	−4·30% (−4·74 to −3·71)	−1·37% (−1·99 to −0·826)	266 (219 to 352)	0·277 (0·228 to 0·367)	−6·62% (−7·35 to −5·91)	−4·35% (−5·41 to −3·14)
15–49 years	125 000 (104 000 to 153 000)	40·0 (33·5 to 49·0)	−2·47% (−2·79 to −2·12)	−1·01% (−1·47 to −0·566)	10 100 (8190 to 12 500)	3·23 (2·62 to 4·01)	−4·17% (−4·77 to −3·48)	−2·89% (−3·46 to −2·17)
50–69 years	51 800 (39 400 to 66 000)	49·6 (37·7 to 63·2)	−2·56% (−2·88 to −2·23)	−1·70% (−2·25 to −1·16)	8040 (6850 to 9630)	7·69 (6·56 to 9·22)	−5·25% (−5·60 to −4·84)	−3·10% (−3·85 to −2·23)
≥70 years	18 600 (14 900 to 23 400)	54·0 (43·1 to 67·7)	−3·62% (−3·90 to −3·34)	−2·82% (−3·15 to −2·39)	5230 (4650 to 6090)	15·2 (13·5 to 17·7)	−5·67% (−5·94 to −5·35)	−4·09% (−4·99 to −3·05)
**North Africa and Middle East**								
All ages	169 000 (149 000 to 195 000)	29·7 (26·4 to 33·8)	−2·36% (−2·59 to −2·05)	−2·87% (−3·31 to −2·49)	19 600 (15 600 to 26 900)	4·13 (3·27 to 5·76)	−3·94% (−4·58 to −3·09)	−3·81% (−5·23 to −2·18)
<5 years	7310 (5740 to 9170)	12·0 (9·39 to 15·0)	−3·20% (−3·70 to −2·74)	−5·47% (−6·35 to −4·72)	1030 (767 to 1310)	1·68 (1·26 to 2·15)	−5·51% (−6·26 to −4·74)	−6·94% (−8·62 to −4·38)
5–14 years	14 800 (10 500 to 20 500)	12·1 (8·62 to 16·8)	−2·21% (−2·54 to −1·75)	−6·22% (−7·21 to −5·34)	424 (337 to 582)	0·347 (0·276 to 0·477)	−3·66% (−4·41 to −2·85)	−6·80% (−8·29 to −4·95)
15–49 years	88 700 (72 700 to 108 000)	26·5 (21·7 to 32·3)	−2·59% (−2·93 to −2·13)	−2·58% (−2·96 to −2·18)	6740 (5370 to 8920)	2·02 (1·61 to 2·67)	−3·56% (−4·39 to −2·74)	−3·77% (−5·18 to −1·91)
50–69 years	39 800 (30 300 to 49 600)	46·8 (35·6 to 58·4)	−2·80% (−3·33 to −2·29)	−2·95% (−3·46 to −2·42)	5790 (4500 to 8260)	6·81 (5·29 to 9·72)	−5·20% (−6·04 to −4·14)	−4·92% (−6·60 to −3·24)
≥70 years	18 100 (14 500 to 22 500)	88·8 (71·3 to 111)	−3·24% (−3·57 to −2·89)	−3·09% (−3·49 to −2·73)	5660 (4280 to 8480)	27·9 (21·1 to 41·7)	−4·70% (−5·79 to −3·65)	−4·55% (−6·00 to −2·82)
**South Asia**								
All ages	3640 000 (3140 000 to 4190 000)	208 (180 to 239)	−1·58% (−1·98 to −1·18)	−1·50% (−1·76 to −1·21)	509 000 (458 000 to 591 000)	33·4 (30·0 to 38·8)	−2·61% (−3·04 to −2·06)	−3·70% (−4·88 to −2·17)
<5 years	62 600 (50 000 to 77 800)	39·5 (31·5 to 49·0)	−3·20% (−3·78 to −2·65)	−4·26% (−4·86 to −3·71)	9970 (7950 to 12 300)	6·29 (5·01 to 7·76)	−5·78% (−6·76 to −4·74)	−7·12% (−8·90 to −4·81)
5–14 years	134 000 (90 800 to 189 000)	38·3 (26·1 to 54·1)	−3·17% (−3·90 to −2·45)	−3·62% (−4·28 to −3·04)	5740 (4900 to 6740)	1·65 (1·41 to 1·93)	−4·53% (−5·23 to −3·60)	−5·88% (−7·19 to −4·56)
15–49 years	2020 000 (1700 000 to 2540 000)	200 (169 to 252)	−1·79% (−2·31 to −1·32)	−2·09% (−2·42 to −1·73)	166 000 (149 000 to 192 000)	16·5 (14·8 to 19·1)	−2·63% (−3·13 to −2·08)	−5·17% (−6·38 to −3·70)
50–69 years	1040 000 (786 000 to 1320 000)	400 (303 to 507)	−2·05% (−2·55 to −1·49)	−2·33% (−2·66 to −2·03)	181 000 (159 000 to 216 000)	69·8 (61·2 to 83·3)	−3·62% (−4·09 to −2·93)	−5·03% (−6·31 to −3·17)
≥70 years	389 000 (304 000 to 481 000)	532 (415 to 657)	−2·33% (−2·86 to −1·89)	−2·09% (−2·51 to −1·68)	147 000 (129 000 to 171 000)	200 (176 to 233)	−3·32% (−3·91 to −2·50)	−4·16% (−5·48 to −2·55)
**Southeast Asia, east Asia, and Oceania**								
All ages	2060 000 (1870 000 to 2290 000)	84·5 (76·8 to 92·2)	−1·44% (−1·68 to −1·14)	−1·23% (−1·42 to −1·04)	237 000 (215 000 to 281 000)	9·09 (8·26 to 10·7)	−2·75% (−3·33 to −2·00)	−2·98% (−4·01 to −1·59)
<5 years	46 700 (39 100 to 58 000)	33·8 (28·3 to 41·9)	−3·52% (−3·88 to −3·14)	−2·04% (−2·29 to −1·77)	3520 (2790 to 4370)	2·55 (2·01 to 3·16)	−6·66% (−7·38 to −6·07)	−8·28% (−9·90 to −6·17)
5–14 years	81 100 (57 400 to 110 000)	26·4 (18·7 to 36·0)	−3·12% (−3·47 to −2·71)	−2·33% (−2·73 to −1·93)	1790 (1570 to 2040)	0·582 (0·512 to 0·664)	−4·04% (−4·56 to −3·48)	−8·29% (−9·26 to −7·21)
15–49 years	875 000 (739 000 to 1020 000)	82·1 (69·3 to 95·3)	−1·72% (−2·09 to −1·33)	−1·30% (−1·53 to −1·09)	59 700 (53 700 to 68 700)	5·59 (5·04 to 6·44)	−2·45% (−3·01 to −1·75)	−3·47% (−4·59 to −2·36)
50–69 years	740 000 (607 000 to 903 000)	142 (117 to 174)	−2·38% (−2·69 to −2·08)	−2·17% (−2·44 to −1·94)	90 400 (81 200 to 108 000)	17·4 (15·6 to 20·9)	−4·69% (−5·37 to −3·79)	−4·34% (−5·63 to −2·78)
≥70 years	318 000 (267 000 to 380 000)	207 (173 to 247)	−2·23% (−2·53 to −1·87)	−3·13% (−3·48 to −2·88)	81 500 (73 200 to 97 300)	52·9 (47·6 to 63·2)	−4·37% (−5·08 to −3·52)	−5·40% (−6·34 to −3·90)
**Sub-Saharan Africa**								
All ages	3010 000 (2670 000 to 3370 000)	351 (315 to 391)	−0·418% (−0·564 to −0·242)	−4·06% (−4·23 to −3·82)	533 000 (456 000 to 609 000)	83·2 (72·3 to 94·5)	−0·814% (−1·39 to −0·363)	−6·10% (−7·24 to −5·08)
<5 years	230 000 (191 000 to 282 000)	133 (111 to 163)	−2·05% (−2·34 to −1·73)	−5·46% (−5·83 to −5·02)	44 800 (31 200 to 57 800)	25·9 (18·1 to 33·5)	−3·36% (−3·98 to −2·68)	−9·23% (−11·2 to −7·03)
5–14 years	201 000 (139 000 to 281 000)	66·4 (45·9 to 92·6)	−0·203% (−0·487 to 0·195)	−5·18% (−5·51 to −4·80)	12 600 (10 300 to 15 100)	4·17 (3·40 to 4·97)	1·39% (0·555 to 2·28)	−7·88% (−9·22 to −6·83)
15–49 years	1870 000 (1580 000 to 2220 000)	342 (289 to 406)	−0·0102% (−0·185 to 0·188)	−4·28% (−4·50 to −4·01)	240 000 (204 000 to 280 000)	43·9 (37·3 to 51·2)	0·784% (0·0659 to 1·40)	−7·15% (−8·18 to −6·20)
50–69 years	538 000 (424 000 to 671 000)	595 (468 to 741)	−0·697% (−0·929 to −0·491)	−3·60% (−3·92 to −3·21)	154 000 (130 000 to 178 000)	170 (144 to 197)	−1·03% (−1·71 to −0·413)	−4·90% (−6·00 to −3·64)
≥70 years	163 000 (135 000 to 194 000)	831 (689 to 992)	−0·744% (−0·970 to −0·503)	−3·20% (−3·51 to −2·88)	81 400 (70 200 to 91 700)	416 (358 to 468)	−1·55% (−2·16 to −0·981)	−3·60% (−4·61 to −2·07)

Data are presented to three significant figures. Data in parentheses are 95% uncertainty intervals. In the all-ages rows, the rates of all-form tuberculosis incidence and deaths are age standardised. GBD=Global Burden of Diseases, Injuries, and Risk Factors Study.

### Age-specific tuberculosis burden

The global age distribution for the number of all-form tuberculosis incident cases in 2021 demonstrated a drop-off in cases after age 5 years, but a rapid spike in cases at age 15–24 years, followed by steady decreases until age 70–74 years, after which there was a rapid drop in cases (appendix 2 p 4). The corresponding age distribution of tuberculosis incidence rates showed a similar pattern until age 20–24 years, when the rate levelled off until a rapid increase from age 50–69 years, followed by a small drop (appendix 2 p 5). In 2021, 3·8% (95% UI 3·6–4·2) of incident cases were in those younger than 5 years, 4·7% (3·8–5·9) were in those aged 5–14 years, 54·9% (52·4–58·9) were in those aged 15–49 years, 26·5% (23·4–29·5) were in those aged 50–69 years, and 10·1% (9·2–11·0) were in those aged 70 years and older (table 1).

The age distribution for tuberculosis deaths in 2021 showed a similar drop-off in deaths after age 5 years, with numbers rapidly increasing after age 19 years before levelling off at age 55–69 years and then sharply decreasing (appendix 2 p 4). For the tuberculosis mortality rate distribution, the rate dropped after age 5 years before steadily increasing for the remaining age groups until age 80–84 years when it levelled off (appendix 2 p 5). In 2021, 4·5% (95% UI 3·5–5·0) of global tuberculosis deaths were in children younger than 5 years, 1·5% (1·5–1·6) were in those aged 5–14 years, 36·4% (35·7–35·9) were in those aged 15–49 years, 33·2% (32·8–33·6) were in those aged 50–69 years, and 24·5% (24·6–24·9) were in those aged 70 years and older (table 1).

In 2021, tuberculosis incidence rates were greater than 100 per 100 000 population in children younger than 5 years in 34 countries, and in children aged 5–14 years in 14 countries (appendix 2 pp 6, 8, 20–85). Tuberculosis incidence rates exceeded 500 per 100 000 population in those aged 15–49 years in nine countries, in those aged 50–69 years in 33 countries, and in those aged 70 years and older in 50 countries (appendix 2 pp 10, 12, 14, 20–85).

In 2021, tuberculosis mortality rates exceeded 25 per 100 000 population in children younger than 5 years in 20 countries, in those aged 5–14 years in one country, and in those aged 15–49 years in 37 countries (appendix 2 pp 7, 9, 11, 20–85). Among older adults, tuberculosis mortality rates exceeded 300 per 100 000 in those aged 50–69 years in eight countries and in those aged 70 years and older in 44 countries (appendix 2 pp 13, 15, 20–85).

Our assessment of the global ARC in tuberculosis incidence showed that children younger than 5 years and those aged 5–14 years had the largest ARCs, decreasing by 4·1% (95% UI 3·8–4·3) and 3·7% (3·4–4·0), respectively, between 2010 and 2021, while ARCs in the adult age groups decreased by around 2% in the same period (table 1). Similarly, children younger than 5 years and those aged 5–14 years had the largest ARCs for tuberculosis mortality rates, decreasing by 7·9% (5·8–9·6) and 6·6% (5·6–7·7), respectively, between 2010 and 2021, while ARCs in the adult age groups decreased by 4·0–4·7% in that period (table 1).

At the super-region level, central Europe, eastern Europe, and central Asia (incidence ARC –4·3% [95% UI –4·8 to –3·8]; mortality ARC –8·7% [–9·3 to –8·1]) and sub-Saharan Africa (incidence ARC –4·1% [–4·2 to –3·8]; mortality ARC –6·1% [–7·2 to –5·1]) had the largest age-standardised ARCs in 2010–21 (table 1). We found that 23 countries had ARCs for age-standardised tuberculosis incidence rates that exceeded 4·5% in 2010–21, 11 of which were in sub-Saharan Africa and nine were in central Europe, eastern Europe, and central Asia (appendix 2 pp 20–85). Similarly, 23 countries had ARCs for age-standardised tuberculosis mortality rates that exceeded 7·0%; of these, 13 were in central Europe, eastern Europe, and central Asia and six were in sub-Saharan Africa.

### Progress towards the End TB Strategy 2020 milestones

**Figure 1:**
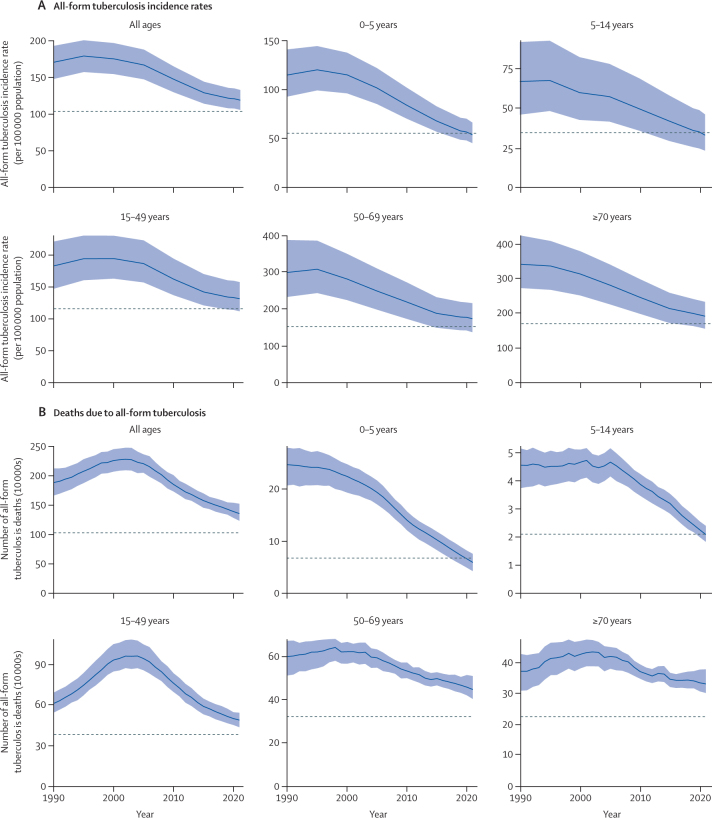
Global age-specific all-form tuberculosis incidence rates (A) and deaths (B), 1990–2021 The dashed grey lines indicate WHO End TB Strategy 2020 milestones (20% reduction in incidence rates and 35% reduction in deaths compared with 2015). The shaded areas represent 95% uncertainty intervals.

At the global level, the all-age tuberculosis incidence rate declined by 6·26% (95% UI 5·27 to 7·25) between 2015 and 2020 (table 2; figure 1). When stratified by sex, the tuberculosis incidence rate decreased by 7·9% (6·5 to 9·2) in females and by 4·9% (4·1 to 5·9) in males (appendix 2 p 18). Among both sexes, children younger than 5 years (16·5% [14·8 to 18·4]) and those aged 5–14 years (16·2% [14·2 to 17·9]) had the largest declines in incidence rate, while the percentage decline ranged between 5·72% and 8·48% for the other age groups (table 2).

**Table 2:** Age-specific all-form tuberculosis percentage change from 2015 to 2020 for incidence rates and deaths by GBD super-region

	**2015 incidence rate per 100 000 population**	**2020 incidence rate per 100 000 population**	**Percentage change in incidence rate**	**Incidence target per 100 000 population**	**2015 deaths**	**2020 deaths**	**Percentage change in mortality, %**	**Mortality target**
**Global**								
All ages	129 (114 to 144)	121 (108 to 135)	−6·26% (−7·25 to −5·27)	103 (91·5 to 115)	1 570 000 (1 450 000 to 1 700 000)	1 390 000 (1 280 000 to 1 540 000)	−11·9% (−17·0 to −5·77)	1 020 000 (943 000 to 1 100 000)
<5 years	67·9 (57·0 to 82·9)	56·7 (48·0 to 70·2)	−16·5% (−18·4 to −14·8)	54·3 (45·6 to 66·3)	104 000 (84 000 to 119 000)	67 200 (50 200 to 83 000)	−35·3% (−41·7 to −26·7)	67 300 (54 600 to 77 600)
5–14 years	41·9 (29·4 to 58·0)	35·1 (24·7 to 48·8)	−16·2% (−17·9 to −14·2)	33·5 (23·5 to 46·4)	32 000 (28 400 to 35 500)	22 600 (20 000 to 25 600)	−29·5% (−34·1 to −25·5)	20 800 (18 500 to 23 100)
15–49 years	141 (120 to 169)	132 (113 to 159)	−6·29% (−7·70 to −5·05)	113 (96·1 to 135)	593 000 (534 000 to 650 000)	503 000 (455 000 to 549 000)	−15·2% (−20·2 to −10·0)	385 000 (347 000 to 422 000)
50–69 years	188 (149 to 232)	177 (141 to 218)	−5·72% (−7·39 to −4·02)	150 (119 to 186)	499 000 (467 000 to 528 000)	459 000 (421 000 to 515 000)	−7·97% (−14·1 to −0·472)	324 000 (304 000 to 343 000)
≥70 years	214 (172 to 260)	196 (160 to 238)	−8·48% (−10·4 to −6·74)	171 (138 to 208)	347 000 (322 000 to 367 000)	335 000 (307 000 to 378 000)	−3·29% (−9·07 to 5·56)	225 000 (209 000 to 239 000)
**Central Europe, eastern Europe, and central Asia**								
All ages	64·2 (55·9 to 76·0)	53·7 (46·5 to 63·9)	−16·5% (−19·4 to −13·9)	51·4 (44·7 to 60·8)	26 900 (25 900 to 28 600)	16 400 (15 600 to 17 600)	−38·8% (−40·6 to −36·5)	17 500 (16 800 to 18 600)
<5 years	11·7 (9·76 to 15·1)	9·43 (7·82 to 12·2)	−19·6% (−24·4 to −16·8)	9·39 (7·81 to 12·1)	602 (507 to 705)	351 (287 to 454)	−41·6% (−50·0 to −33·7)	391 (330 to 458)
5–14 years	13·9 (9·46 to 19·2)	11·7 (8·00 to 16·4)	−15·7% (−20·1 to −11·3)	11·1 (7·57 to 15·4)	103 (93·6 to 113)	70·1 (63·0 to 78·8)	−31·9% (−35·5 to −27·7)	66·9 (60·9 to 73·5)
15–49 years	86·1 (69·6 to 106)	72·8 (58·9 to 92·3)	−15·5% (−19·5 to −12·3)	68·8 (55·7 to 85·1)	12 400 (11 600 to 13 900)	7160 (6570 to 8070)	−42·4% (−43·9 to −40·3)	8090 (7560 to 9060)
50–69 years	66·7 (50·0 to 84·6)	56·9 (42·7 to 73·3)	−14·7% (−18·3 to −10·9)	53·4 (40·0 to 67·7)	11 000 (10 800 to 11 300)	6610 (6340 to 7040)	−40·0% (−42·5 to −37·2)	7170 (7010 to 7350)
≥70 years	43·2 (33·5 to 54·6)	36·6 (28·9 to 46·3)	−15·3% (−19·6 to −11·3)	34·6 (26·8 to 43·7)	2680 (2550 to 2760)	2240 (2100 to 2330)	−16·5% (−18·6 to −14·4)	1740 (1660 to 1790)
**High income**								
All ages	10·3 (9·20 to 11·7)	9·40 (8·37 to 10·8)	−8·37% (−10·2 to −6·76)	8·21 (7·36 to 9·37)	13 200 (11 800 to 14 300)	12 500 (11 000 to 13 600)	−5·23% (−7·26 to −3·09)	8590 (7690 to 9300)
<5 years	2·19 (1·73 to 2·81)	1·81 (1·40 to 2·33)	−17·6% (−20·4 to −14·6)	1·76 (1·38 to 2·25)	39·6 (33·6 to 45·4)	26·0 (21·6 to 30·7)	−34·4% (−37·6 to −30·7)	25·7 (21·9 to 29·5)
5–14 years	2·23 (1·52 to 3·24)	2·15 (1·46 to 3·15)	−3·77% (−6·41 to −1·03)	1·78 (1·22 to 2·59)	24·6 (20·7 to 28·1)	19·7 (16·8 to 22·7)	−19·7% (−23·5 to −15·7)	16·0 (13·5 to 18·3)
15–49 years	9·96 (8·10 to 12·4)	8·86 (7·21 to 11·0)	−11·0% (−12·7 to −8·89)	7·96 (6·48 to 9·88)	1800 (1440 to 2120)	1500 (1200 to 1810)	−16·3% (−18·8 to −13·8)	1170 (935 to 1380)
50–69 years	11·3 (8·67 to 13·9)	10·3 (7·81 to 12·7)	−9·40% (−13·6 to −6·49)	9·07 (6·93 to 11·2)	2700 (2490 to 2920)	2350 (2120 to 2540)	−13·1% (−15·6 to −11·0)	1750 (1620 to 1900)
≥70 years	20·4 (17·0 to 24·7)	18·3 (15·1 to 22·3)	−10·3% (−15·2 to −6·76)	16·3 (13·6 to 19·8)	8660 (7320 to 9370)	8630 (7120 to 9510)	−0·334% (−3·03 to 2·71)	5630 (4760 to 6090)
**Latin America and Caribbean**								
All ages	36·0 (31·7 to 41·0)	34·7 (30·4 to 39·8)	−3·42% (−5·77 to −0·669)	28·8 (25·3 to 32·8)	25 800 (22 600 to 31 200)	24 700 (21 200 to 29 200)	−4·32% (−9·16 to 1·35)	16 800 (14 700 to 20 300)
<5 years	9·50 (7·94 to 11·7)	8·65 (7·29 to 10·8)	−8·93% (−12·4 to −5·44)	7·60 (6·35 to 9·33)	785 (640 to 986)	590 (460 to 750)	−25·0% (−32·9 to −15·0)	511 (416 to 641)
5–14 years	7·91 (5·54 to 10·8)	7·87 (5·64 to 11·0)	−0·467% (−5·06 to 5·26)	6·32 (4·43 to 8·65)	347 (289 to 468)	285 (236 to 371)	−17·7% (−23·4 to −9·85)	225 (188 to 304)
15–49 years	41·3 (34·8 to 50·1)	39·9 (33·5 to 48·5)	−3·55% (−7·06 to −0·0735)	33·1 (27·8 to 40·1)	11 400 (9440 to 14 200)	10 300 (8370 to 12 800)	−10·0% (−14·0 to −5·73)	7420 (6140 to 9250)
50–69 years	54·3 (42·5 to 67·5)	49·9 (38·3 to 63·1)	−8·13% (−12·0 to −3·12)	43·4 (34·0 to 54·0)	7960 (7130 to 9610)	8140 (7040 to 9660)	2·29% (−4·33 to 9·20)	5170 (4630 to 6250)
≥70 years	64·0 (52·0 to 79·1)	55·0 (44·6 to 68·5)	−14·0% (−16·7 to −11·5)	51·2 (41·6 to 63·3)	5290 (4810 to 5960)	5390 (4830 to 6100)	1·98% (−4·94 to 10·3)	3440 (3130 to 3870)
**North Africa and Middle East**								
All ages	31·5 (27·7 to 35·8)	28·1 (24·9 to 32·5)	−10·7% (−13·2 to −8·66)	25·2 (22·2 to 28·6)	22 600 (18 700 to 31 700)	20 200 (16 300 to 28 300)	−10·5% (−17·6 to −3·06)	14 700 (12 200 to 20 600)
<5 years	15·1 (12·5 to 18·5)	12·6 (9·82 to 15·4)	−16·8% (−21·8 to −11·8)	12·1 (9·97 to 14·8)	1660 (1260 to 2080)	1140 (849 to 1470)	−30·9% (−38·8 to −19·3)	1080 (821 to 1350)
5–14 years	15·6 (11·4 to 21·9)	12·7 (9·02 to 17·6)	−18·5% (−24·5 to −13·0)	12·4 (9·11 to 17·5)	612 (481 to 829)	454 (369 to 623)	−25·6% (−33·9 to −15·9)	398 (313 to 539)
15–49 years	31·3 (25·8 to 37·7)	27·7 (22·7 to 33·6)	−11·5% (−14·0 to −9·18)	25·1 (20·7 to 30·2)	7960 (6400 to 10 200)	6930 (5530 to 9100)	−12·9% (−20·8 to −4·60)	5180 (4160 to 6630)
50–69 years	54·5 (42·0 to 68·1)	48·5 (36·8 to 59·5)	−10·9% (−13·4 to −7·58)	43·6 (33·6 to 54·5)	5900 (4870 to 8700)	5790 (4590 to 8450)	−1·93% (−10·3 to 9·05)	3840 (3170 to 5650)
≥70 years	109 (87·6 to 134)	92·8 (75·2 to 115)	−14·6% (−18·1 to −12·1)	86·9 (70·1 to 108)	6410 (5020 to 10 800)	5870 (4500 to 8980)	−8·28% (−17·0 to 3·08)	4170 (3260 to 7010)
**South Asia**								
All ages	205 (177 to 235)	199 (172 to 228)	−3·06% (−5·10 to −1·25)	164 (142 to 188)	573 000 (536 000 to 611 000)	523 000 (460 000 to 598 000)	−8·71% (−16·4 to 1·24)	373 000 (348 000 to 397 000)
<5 years	48·2 (39·0 to 59·7)	43·1 (34·0 to 53·7)	−10·7% (−14·6 to −7·34)	38·6 (31·2 to 47·8)	17 800 (15 300 to 20 200)	11 200 (9140 to 13 800)	−36·9% (−46·9 to −26·0)	11 600 (9920 to 13 100)
5–14 years	47·7 (33·0 to 67·5)	40·6 (27·9 to 56·7)	−14·9% (−19·4 to −11·2)	38·2 (26·4 to 54·0)	8230 (7250 to 9190)	6090 (5150 to 7050)	−26·1% (−32·7 to −17·4)	5350 (4710 to 5970)
15–49 years	216 (181 to 273)	201 (171 to 254)	−6·59% (−9·20 to −4·10)	173 (145 to 218)	186 000 (172 000 to 202 000)	169 000 (148 000 to 188 000)	−9·39% (−19·3 to −0·412)	121 000 (112 000 to 131 000)
50–69 years	440 (331 to 561)	410 (315 to 516)	−6·61% (−9·71 to −4·01)	352 (265 to 449)	213 000 (198 000 to 229 000)	189 000 (164 000 to 224 000)	−11·0% (−19·3 to 0·197)	138 000 (129 000 to 149 000)
≥70 years	560 (436 to 707)	534 (418 to 660)	−4·52% (−7·94 to −1·36)	448 (349 to 566)	148 000 (137 000 to 160 000)	148 000 (130 000 to 170 000)	−0·103% (−9·08 to 13·1)	96 100 (89 100 to 104 000)
**Southeast Asia, east Asia, and Oceania**								
All ages	97·5 (88·6 to 106)	95·9 (87·5 to 105)	−1·72% (−3·32 to −0·442)	78·0 (70·9 to 85·1)	265 000 (248 000 to 286 000)	240 000 (219 000 to 273 000)	−9·45% (−16·9 to −0·0543)	173 000 (161 000 to 186 000)
<5 years	39·1 (32·6 to 48·3)	34·6 (28·9 to 42·7)	−11·4% (−13·6 to −9·52)	31·3 (26·1 to 38·6)	6020 (5020 to 6970)	3860 (3090 to 4750)	−35·9% (−43·1 to −28·5)	3910 (3260 to 4530)
5–14 years	30·4 (21·7 to 41·4)	27·8 (19·9 to 37·4)	−8·39% (−11·0 to −6·19)	24·3 (17·3 to 33·1)	2810 (2540 to 3070)	1950 (1720 to 2210)	−30·7% (−36·6 to −25·2)	1830 (1650 to 2000)
15–49 years	84·6 (72·5 to 97·1)	83·6 (71·3 to 97·2)	−1·21% (−3·31 to 1·25)	67·7 (58·0 to 77·7)	77 800 (72 200 to 84 400)	62 000 (56 500 to 70 500)	−20·2% (−29·0 to −10·5)	50 500 (46 900 to 54 900)
50–69 years	154 (127 to 184)	145 (119 to 175)	−5·56% (−8·08 to −3·40)	123 (102 to 148)	92 300 (85 400 to 99 900)	90 700 (80 000 to 104 000)	−1·65% (−10·9 to 10·4)	60 000 (55 500 to 64 900)
≥70 years	244 (205 to 285)	215 (181 to 256)	−12·0% (−14·5 to −9·23)	195 (164 to 228)	86 500 (78 700 to 95 000)	81 700 (74 000 to 93 800)	−5·59% (−12·2 to 4·66)	56 300 (51 100 to 61 800)
**Sub-Saharan Africa**								
All ages	333 (297 to 370)	276 (246 to 306)	−17·1% (−18·2 to −15·6)	266 (238 to 296)	646 000 (557 000 to 727 000)	549 000 (476 000 to 624 000)	−15·1% (−20·1 to −8·55)	420 000 (362 000 to 473 000)
<5 years	187 (156 to 227)	143 (121 to 177)	−23·6% (−26·2 to −21·2)	149 (125 to 182)	76 700 (59 000 to 90 300)	50 000 (35 400 to 62 600)	−35·0% (−42·7 to −26·7)	49 800 (38 300 to 58 700)
5–14 years	93·0 (64·9 to 128)	71·6 (49·3 to 99·6)	−23·0% (−25·6 to −21·0)	74·4 (51·9 to 102)	19 900 (16 700 to 22 800)	13 700 (11 400 to 16 100)	−31·1% (−36·7 to −26·3)	12 900 (10 900 to 14 800)
15–49 years	435 (366 to 515)	356 (303 to 427)	−18·3% (−20·0 to −16·7)	348 (293 to 412)	295 000 (255 000 to 341 000)	246 000 (210 000 to 288 000)	−16·6% (−21·7 to −11·0)	192 000 (166 000 to 221 000)
50–69 years	724 (581 to 897)	617 (491 to 760)	−14·8% (−16·8 to −12·5)	579 (465 to 717)	166 000 (144 000 to 187 000)	156 000 (133 000 to 179 000)	−6·04% (−12·2 to 2·10)	108 000 (93 300 to 122 000)
≥70 years	1010 (836 to 1200)	866 (722 to 1030)	−14·3% (−15·9 to −12·6)	808 (669 to 961)	89 000 (77 800 to 97 200)	83 500 (72 400 to 92 600)	−6·15% (−11·4 to 2·64)	57 900 (50 600 to 63 200)

Data are presented to three significant figures. Data in parentheses are 95% uncertainty intervals. GBD=Global Burden of Diseases, Injuries, and Risk Factors Study.

The number of global tuberculosis deaths decreased by 11·9% (95% UI 5·77 to 17·0) from 2015 to 2020 (table 2). The percentage change in the number of global tuberculosis deaths was greater in females (13·8% [8·4 to 18·0]) than in males (10·5% [0·1 to 18·1]; appendix 2 p 18). Among both sexes, children younger than 5 years (35·3% [26·7 to 41·7]) and those aged 5–14 years (29·5% [25·5 to 34·1]) had the largest declines in the number of deaths, while individuals aged 70 years and older (3·29% [–5·56 to 9·07]) had the lowest decline (figure 1).

**Figure 2:**
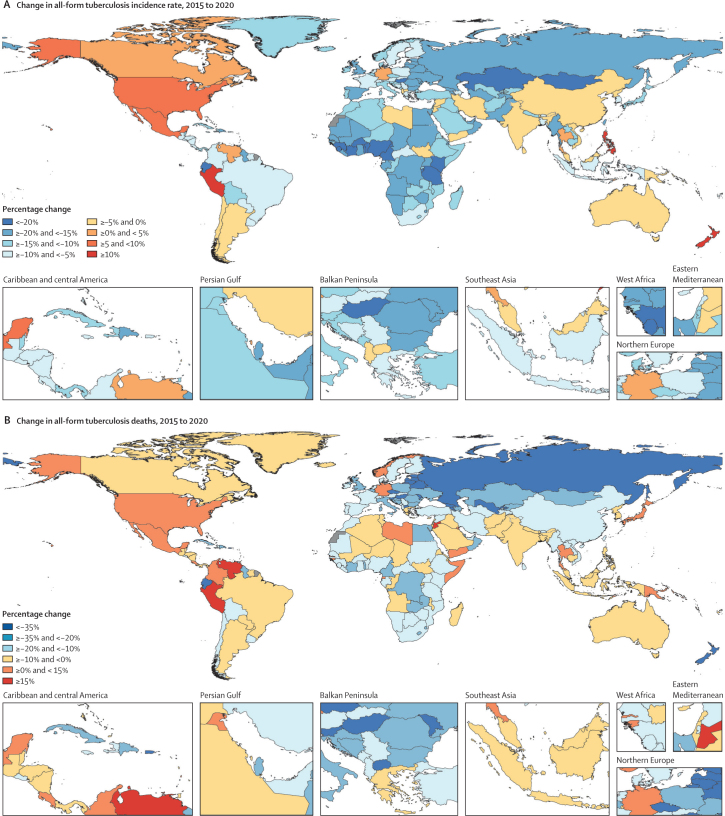
Percentage change from 2015 to 2020 in all-age, all-form tuberculosis incidence rates per 100 000 population (A) and deaths (B)

Among all countries, 15 achieved a 20% decrease in the all-age tuberculosis incidence rate between 2015 and 2020: eight of these were in western sub-Saharan Africa, three were in eastern sub-Saharan Africa, one was in central Europe, two were in central Asia, and one was in Andean Latin America (figure 2A; appendix 2 pp 86–149). When stratified by age groups, 66 countries achieved a 20% reduction in the all-age tuberculosis incidence rate in children younger than 5 years, as did 50 countries in those aged 5–14 years, 22 countries in those aged 15–49 years, 12 countries in those aged 50–69 years, and ten countries in those aged 70 years and older (appendix 2 pp 86–149). The only region that achieved a 20% decrease in the incidence rate was western sub-Saharan Africa (21·6% [95% UI 19·7 to 23·3]; appendix 2 pp 86–149). By 2021, 38 countries had achieved a 20% reduction in the all-age tuberculosis incidence rate (21 in sub-Saharan Africa; 11 in central Europe, eastern Europe, and central Asia; three in high-income countries; and three in the remaining GBD super-regions), with the sub-Saharan Africa super-region becoming the only super-region to achieve a 20% reduction (appendix 2 pp 150–213). Among the 20 high tuberculosis burden countries in GBD 2021, three countries (Tanzania, Kenya, and Nigeria) achieved a 20% decrease in the all-age incidence rate in 2020, with an additional three high tuberculosis burden countries (Myanmar, Uganda, and Zimbabwe) meeting the target in 2021 (appendix 2 p 16).

Eastern Europe (43·8% [95% UI 40·7 to 45·9]) was the only region that achieved a 35% decline in the number of tuberculosis deaths between 2015 and 2020 (appendix 2 pp 86–149). 19 countries and territories achieved this milestone: six in eastern Europe, four in central Europe, four in central Asia, two in the Caribbean, one in Andean Latin America, one in Australasia, and one in western sub-Saharan Africa (figure 2B; appendix 2 pp 86–149). Central Europe, eastern Europe, and central Asia was the only GBD super-region to achieve a 35% reduction in the number of tuberculosis deaths by 2020 (38·8% [36·5–40·6]; table 2). Regarding age groups, 95 countries achieved a 35% decrease in the number of tuberculosis deaths in children younger than 5 years, as did 55 countries in those aged 5–14 years, 26 countries in those aged 15–49 years, 20 countries in those aged 50–69 years, and six countries in those aged 70 years and older. In 2021, two additional countries (ie, 19 in total) achieved a 35% decrease in tuberculosis deaths (appendix 2 pp 150–213). None of the countries with a high tuberculosis burden achieved a 35% decrease in tuberculosis deaths in 2020 or 2021, but the Democratic Republic of the Congo (23·7% [11·9–34·9]), Myanmar (22·9% [6·94–33·0]), and Tanzania (22·5% [10·7–34·0]) had the largest reductions between 2015 and 2021 (appendix 2 p 17).

After removing the combined effects of three tuberculosis risk factors (smoking, alcohol use, and diabetes), the number of global all-form tuberculosis deaths would decrease from 1·39 million (95% UI 1·28–1·54) to 1·00 million (0·703–1·23) in 2020 (table 3). Comparing the observed number of tuberculosis deaths in 2015 to the risk-deleted number of tuberculosis deaths in 2020 represents a 36·5% (21·5–54·8) decrease. The corresponding percentage declines would be 31·0% (15·9–48·8) in those aged 15–49 years, 40·5% (22·3–62·6) in those aged 50–69 years, and 41·0% (25·4–62·0) in those aged 70 years and older. If the effects of risk factors were removed, 13 additional regions would have joined eastern Europe in achieving a 35% decline in the number of tuberculosis deaths. Furthermore, the cumulative number of countries achieving a 35% decrease would have increased from 15 to 132 in 2020 and 143 in 2021 (appendix 2 pp 214–99). A 35% reduction would have been achieved in 104 countries for individuals aged 15–49 years, in 144 countries for those aged 50–69 years, and in 169 countries for those aged 70 years and older in 2020.

**Table 3:** Age-specific risk-deleted tuberculosis deaths by GBD super-region, 2020

	**Observed number of deaths for 2015**	**Observed number of deaths for 2020**	**Smoking-deleted counts for 2020**	**Alcohol-deleted counts for 2020**	**Diabetes-deleted counts for 2020**	**All risk-deleted number of deaths**	**Percentage change in 2020 risk-deleted number of deaths *vs* 2015 observed number of deaths, %**
**Global**							
All ages	1 570 000 (1 450 000 to 1 700 000)	1 390 000 (1 280 000 to 1 540 000)	1 220 000 (1 050 000 to 1 390 000)	1 250 000 (889 000 to 1 550 000)	1 240 000 (1 130 000 to 1 390 000)	999 000 (703 000 to 1 230 000)	−36·5% (−54·8 to −21·5)
15–49 years	593 000 (534 000 to 650 000)	503 000 (455 000 to 549 000)	457 000 (396 000 to 510 000)	453 000 (323 000 to 566 000)	492 000 (444 000 to 538 000)	408 000 (297 000 to 499 000)	−31·0% (−48·8 to −15·9)
50–69 years	499 000 (467 000 to 528 000)	459 000 (421 000 to 515 000)	382 000 (313 000 to 456 000)	403 000 (255 000 to 523 000)	399 000 (361 000 to 462 000)	297 000 (185 000 to 389 000)	−40·5% (−62·6 to −22·3)
≥70 years	347 000 (322 000 to 367 000)	335 000 (307 000 to 378 000)	294 000 (253 000 to 336 000)	303 000 (207 000 to 379 000)	257 000 (230 000 to 309 000)	204 000 (129 000 to 260 000)	−41·0% (−62·0 to −25·4)
**Central Europe, eastern Europe, and central Asia**							
All ages	26 900 (25 900 to 28 600)	16 400 (15 600 to 17 600)	11 900 (9140 to 15 200)	13 000 (6220 to 20 800)	15 100 (14 200 to 16 500)	9040 (4520 to 14 500)	−66·3% (−83·0 to −45·4)
15–49 years	12 400 (11 600 to 13 900)	7160 (6570 to 8070)	5220 (4090 to 6710)	5670 (2500 to 9260)	7000 (6410 to 7930)	4230 (2090 to 6690)	−66·0% (−83·2 to −45·2)
50–69 years	11 000 (10 800 to 11 300)	6610 (6340 to 7040)	4310 (3060 to 5900)	5000 (1750 to 8580)	5870 (5520 to 6320)	2970 (989 to 5560)	−73·1% (−91·0 to −49·4)
≥70 years	2680 (2550 to 2760)	2240 (2100 to 2330)	1950 (1680 to 2190)	1940 (1190 to 2560)	1850 (1710 to 2020)	1420 (882 to 1920)	−47·1% (−67·5 to −30·2)
**High income**							
All ages	13 200 (11 800 to 14 300)	12 500 (11 000 to 13 600)	10 900 (8970 to 12 400)	10 200 (4570 to 15 200)	10 400 (9190 to 11 700)	7520 (3320 to 11 200)	−43·2% (−74·0 to −15·3)
15–49 years	1800 (1440 to 2120)	1500 (1200 to 1810)	1330 (1030 to 1610)	1320 (805 to 1860)	1480 (1180 to 1780)	1180 (763 to 1560)	−34·4% (−53·1 to −16·1)
50–69 years	2700 (2490 to 2920)	2350 (2120 to 2540)	1850 (1450 to 2240)	1880 (841 to 2910)	2050 (1810 to 2300)	1360 (640 to 2090)	−49·7% (−75·5 to −22·1)
≥70 years	8660 (7320 to 9370)	8630 (7120 to 9510)	7660 (5910 to 8830)	6950 (2920 to 10 500)	6860 (5680 to 7980)	4930 (1890 to 7650)	−43·1% (−77·7 to −14·4)
**Latin America and Caribbean**							
All ages	25 800 (22 600 to 31 200)	24 700 (21 200 to 29 200)	22 400 (18 700 to 27 200)	21 300 (12 900 to 29 600)	22 100 (18 500 to 26 800)	17 600 (10 700 to 24 500)	−31·6% (−56·4 to −9·35)
15–49 years	11 400 (9440 to 14 200)	10 300 (8370 to 12 800)	9550 (7610 to 12 100)	8820 (5350 to 12 900)	10 100 (8180 to 12 500)	8170 (5190 to 11 700)	−28·4% (−54·5 to −4·74)
50–69 years	7960 (7130 to 9610)	8140 (7040 to 9660)	7070 (5800 to 8740)	6890 (3990 to 9720)	7050 (5930 to 8510)	5320 (3080 to 7570)	−33·2% (−59·6 to −9·66)
≥70 years	5290 (4810 to 5960)	5390 (4830 to 6100)	4930 (4230 to 5690)	4700 (2780 to 6300)	4090 (3530 to 4810)	3280 (1830 to 4510)	−38·1% (−63·9 to −19·3)
**North Africa and Middle East**							
All ages	22 600 (18 700 to 31 700)	20 200 (16 300 to 28 300)	17 700 (13 800 to 23 900)	19 900 (16 000 to 28 200)	16 500 (12 800 to 23 500)	14 300 (11 100 to 19 500)	−36·4% (−45·9 to −26·9)
15–49 years	7960 (6400 to 10 200)	6930 (5530 to 9100)	6020 (4840 to 7730)	6800 (5350 to 8950)	6560 (5260 to 8660)	5610 (4440 to 7270)	−29·4% (−39·1 to −18·0)
50–69 years	5900 (4870 to 8700)	5790 (4590 to 8450)	4790 (3540 to 6700)	5700 (4490 to 8320)	4330 (3250 to 6440)	3500 (2460 to 5130)	−40·6% (−53·4 to −26·5)
≥70 years	6410 (5020 to 10 800)	5870 (4500 to 8980)	5290 (3910 to 8130)	5820 (4480 to 8970)	4050 (2980 to 6550)	3620 (2600 to 5800)	−43·7% (−54·2 to −33·2)
**South Asia**							
All ages	573 000 (536 000 to 611 000)	523 000 (460 000 to 598 000)	448 000 (367 000 to 534 000)	471 000 (314 000 to 594 000)	448 000 (388 000 to 525 000)	351 000 (223 000 to 454 000)	−38·8% (−60·2 to −21·2)
15–49 years	186 000 (172 000 to 202 000)	169 000 (148 000 to 188 000)	149 000 (127 000 to 172 000)	150 000 (97 900 to 191 000)	163 000 (143 000 to 181 000)	129 000 (84 500 to 168 000)	−30·7% (−54·7 to −10·0)
50–69 years	213 000 (198 000 to 229 000)	189 000 (164 000 to 224 000)	154 000 (120 000 to 193 000)	168 000 (102 000 to 218 000)	158 000 (134 000 to 193 000)	116 000 (65 900 to 157 000)	−45·7% (−67·7 to −27·3)
≥70 years	148 000 (137 000 to 160 000)	148 000 (130 000 to 170 000)	128 000 (104 000 to 152 000)	137 000 (96 600 to 168 000)	110 000 (92 400 to 136 000)	88 800 (56 400 to 116 000)	−40·0% (−60·6 to −22·7)
**Southeast Asia, east Asia, and Oceania**							
All ages	265 000 (248 000 to 286 000)	240 000 (219 000 to 273 000)	191 000 (159 000 to 230 000)	215 000 (144 000 to 276 000)	209 000 (185 000 to 241 000)	151 000 (95 900 to 203 000)	−42·9% (−63·0 to −24·4)
15–49 years	77 800 (72 200 to 84 400)	62 000 (56 500 to 70 500)	51 200 (45 600 to 60 000)	55 400 (38 000 to 70 400)	60 800 (55 000 to 69 200)	45 700 (31 900 to 60 900)	−41·1% (−60·1 to −24·7)
50–69 years	92 300 (85 400 to 99 900)	90 700 (80 000 to 104 000)	67 100 (52 500 to 85 800)	80 100 (49 600 to 106 000)	79 000 (68 100 to 92 500)	52 800 (31 300 to 74 500)	−42·8% (−66·0 to −18·8)
≥70 years	86 500 (78 700 to 95 000)	81 700 (74 000 to 93 800)	67 000 (55 000 to 78 500)	73 500 (50 100 to 95 700)	63 000 (55 400 to 73 600)	47 000 (28 700 to 62 300)	−45·7% (−65·8 to −26·8)
**Sub-Saharan Africa**							
All ages	646 000 (557 000 to 727 000)	549 000 (476 000 to 624 000)	520 000 (446 000 to 589 000)	498 000 (359 000 to 626 000)	516 000 (442 000 to 590 000)	449 000 (323 000 to 553 000)	−30·6% (−49·1 to −18·3)
15–49 years	295 000 (255 000 to 341 000)	246 000 (210 000 to 288 000)	234 000 (199 000 to 270 000)	225 000 (165 000 to 284 000)	243 000 (208 000 to 285 000)	214 000 (160 000 to 263 000)	−27·2% (−45·0 to −14·8)
50–69 years	166 000 (144 000 to 187 000)	156 000 (133 000 to 179 000)	142 000 (118 000 to 163 000)	136 000 (86 600 to 179 000)	142 000 (120 000 to 165 000)	115 000 (72 400 to 149 000)	−30·6% (−56·7 to −10·6)
≥70 years	89 000 (77 800 to 97 200)	83 500 (72 400 to 92 600)	79 300 (68 000 to 88 300)	73 200 (44 000 to 92 800)	66 400 (57 500 to 78 600)	55 500 (32 100 to 71 700)	−37·7% (−63·1 to −21·3)

Data are presented to three significant figures. Data in parentheses are 95% uncertainty intervals. The total number of deaths presented in this table is for all-form tuberculosis. However, the risk factors are for HIV-negative tuberculosis, and accordingly, risk-factor death deletion only occurred for individuals without HIV co-infection. GBD=Global Burden of Diseases, Injuries, and Risk Factors Study.

### Impact of the COVID-19 pandemic on tuberculosis mortality

**Figure 3:**
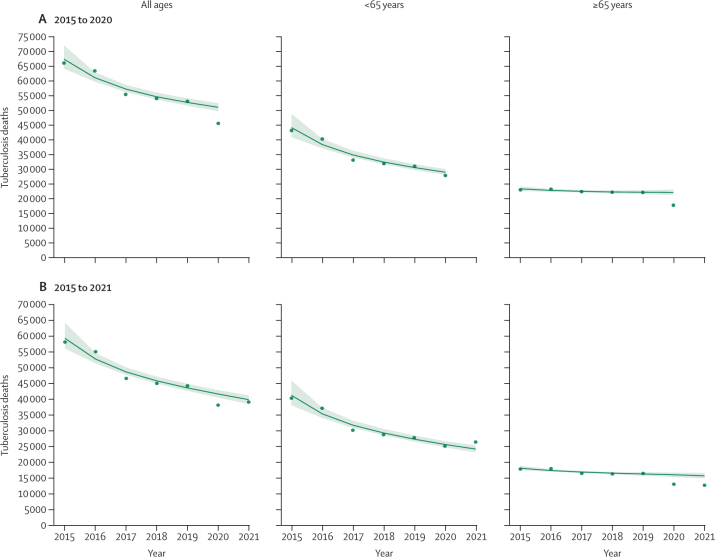
Observed versus predicted tuberculosis deaths for all countries with available cause-specific vital registration data combined from 2015 to 2020 (A) and 2021 (B) Observed tuberculosis deaths represent tuberculosis deaths without HIV co-infection from vital registration data. 41 countries had data up to 2020 and 20 countries had data up to 2021. Green circles represent observed tuberculosis deaths aggregated across included countries. Solid lines are aggregated predicted values from country-specific and age-specific quasi-Poisson regressions, with vital registration data from 2015 to 2019 used as inputs; the shaded areas represent the corresponding 95% CIs. Complete country-specific results are included in appendix 2 (pp 300–04).

For the 41 countries included in our analysis of COVID-19 impact, 50 900 (95% CI 49 700 to 52 400) tuberculosis deaths without HIV co-infection were expected across all ages in 2020, compared to an observed 45 500 tuberculosis deaths (figure 3A; appendix 2 pp 300–04). This corresponded to 5340 (4070 to 6920) fewer tuberculosis deaths than expected (negative excess tuberculosis deaths) and an observed to expected (OE) ratio of 0·90 (0·87 to 0·92). Individuals aged 65 years and older contributed more to the negative excess deaths (–4330 [–5310 to –3370], OE ratio 0·80 [0·77 to 0·84]) than did those younger than 65 years (–1000 [–2150 to –127], OE ratio 0·97 [0·93 to 1·00]). We estimated that 28 of 41 countries had negative excess tuberculosis deaths, with a combined contribution of 6000 (4770 to 7400) fewer tuberculosis deaths and an OE ratio of 0·86 (0·83 to 0·89). Those aged 65 years and older also contributed more negative excess deaths (–4340 [–5310 to –3370], OE ratio 0·78 [0·75 to 0·82]) than did those younger than 65 years (–1660 [–2580 to –817], OE ratio 0·93 [0·89 to 0·96]) in these 28 countries. 13 countries had excess tuberculosis deaths, with a combined contribution of 669 (–63 to 1030) excess tuberculosis deaths and an OE ratio of 1·08 (0·99 to 1·13). However, in these countries individuals younger than 65 years contributed almost all the excess tuberculosis deaths (660 [–60 to 1010], OE ratio 1·11 [0·99 to 1·17] *vs* eight [–160 to 126], OE ratio 1·00 [0·92 to 1·07], in those aged ≥65 years).

There were 20 countries reporting data through the year 2021 (figure 3B; appendix 2 pp 300–04). In these countries, 39 600 (95% CI 38 300 to 41 100) tuberculosis deaths without HIV co-infection were expected compared to an observed 39 000 tuberculosis deaths (appendix 2 pp 300–04). This corresponded to 657 (–713 to 2180) fewer tuberculosis deaths than expected, with an OE ratio of 0·98 (0·95–1·02). There were marked differences by age as people younger than 65 years contributed excess tuberculosis deaths (2290 [1100 to 3370], OE ratio 1·10 [1·04 to 1·15]), whereas those aged 65 years and older had negative excess tuberculosis deaths (–2950 [–3920 to –2100], OE ratio 0·81 [0·76 to 0·86]). Only five countries contributed negative excess deaths, with a combined contribution of 2310 (1100 to 3680) fewer tuberculosis deaths and an OE ratio of 0·90 (0·85 to 0·95). In those countries, people aged 65 years and older contributed negative excess deaths (–2880 [–3860 to –2090], OE ratio 0·72 [0·66 to 0·78]), whereas those younger than 65 years did not (574 [–467 to 1602], OE ratio 1·04 [0·97 to 1·13]). The remaining 15 countries had a combined contribution of 1650 (737 to 2100) excess tuberculosis deaths and an OE ratio of 1·10 (1·04 to 1·13). Among these 15 countries, people younger than 65 years contributed almost all excess tuberculosis deaths (1720 [824 to 2097], OE ratio 1·16 [1·07 to 1·20]), whereas those aged 65 years and older contributed 71 (–213 to 395) fewer deaths than expected (OE ratio 0·99 [0·93 to 1·04]).

## Discussion

Despite accelerated global progress in reducing the burden of tuberculosis in the past decade, we observed that the first WHO End TB interim milestones in 2020 were not achieved at the global level: global tuberculosis incidence decreased by only 6% and global tuberculosis deaths decreased by 12% between 2015 and 2020. The substantial gap in reaching the global milestones is in keeping with observations from other studies.^31^, ^32^, ^33^, ^34^ We further observed that progress was differential with respect to age such that individuals younger than 15 years had the sharpest declines while the older adult age groups had only minimal reductions. Our analysis of risk-deleted mortality suggests that reducing tuberculosis risk factors will be important in any holistic strategy for reducing tuberculosis mortality among adults and achieving the optimistic End TB milestones.

The finding that only 15 countries achieved the 2020 tuberculosis incidence milestone in 2020 underscores that substantial challenges remain in identifying all individuals with tuberculosis in communities and initiating treatment. When individuals with tuberculosis remain undiagnosed, they might unknowingly propagate infections to healthy contacts.^35^ Tuberculosis control programmes could consider enhancing interventions that have previously been shown to improve detection and mitigate delays in treatment initiation, including active case finding,^36^ tuberculosis outreach screening with communication campaigns,^37^ and the use of new rapid tuberculosis diagnostic tools.^38^ Tuberculosis control programmes could also consider exploring the programmes running in sub-Saharan Africa, since 11 of the 15 countries that achieved the incidence milestone in 2020 were in this region. For example, Nigeria achieved a 22% decrease (appendix 2 pp 86–149), indicating that its novel active case-finding interventions with advanced surveillance in tuberculosis hotspots,^39^ increase in diagnostic equipment and partnering with the private sector,^40^ and economic incentives for patients^41^ were effective in helping to achieve the milestone. In Tanzania, which achieved a 26% reduction (appendix 2 pp 86–149), the national tuberculosis programme scaled up community-based interventions aiming to link individuals to tuberculosis services with proven effectiveness^42^ while introducing an effective nationwide quality-improvement initiative in tuberculosis case finding.^43^ The marked progress in Cameroon^44^ (30% reduction in incidence) and Kenya^45^ (23% reduction in incidence; appendix 2 pp 86–149) might also point to the success of intensified tuberculosis case-finding programmes that were reported to increase case detection in these respective countries . In addition to these innovative case-finding programmes, many countries in sub-Saharan Africa saw marked increases in antiretroviral therapy coverage for people with HIV between 2015 and 2020 that might have further contributed to the substantial reductions in tuberculosis incidence in the region.^46^

We observed that only 17 countries achieved the End TB mortality milestone in 2020, indicating continued suboptimal treatment outcomes globally, potentially owing to poor early linkage to treatment, inadequate support for people with tuberculosis to ensure treatment adherence, unidentified drug resistance, and inadequate prevention of advanced HIV disease. Previous research has indicated that treatment adherence interventions (eg, patient counselling and education, reminders, and digital health technologies)^47^, ^48^ and social protection interventions (eg, cash transfers and economic incentives)^49^, ^50^ can increase treatment adherence and prevent mortality. A new tuberculosis treatment regimen for drug-resistant tuberculosis, endorsed by WHO, that is efficacious, safe, and shorter than previous regimens could have a role in reducing tuberculosis deaths.^51^ Additional research should examine the marked progress in reducing tuberculosis deaths in the 17 countries that achieved the End TB mortality milestone in 2020. For instance, national social protection interventions in Moldova^52^ (which achieved a 48% reduction) and Ecuador^53^ (which achieved a 41% reduction; appendix 2 pp 86–149) markedly increased treatment adherence, potentially prevented drug resistance, and might have played a role in the countries' progress.

Most importantly, our results showed differential progress with respect to age in reducing the burden of tuberculosis. We found that children (aged <15 years) had the largest reductions in tuberculosis burden, with a 16% reduction in the incidence rate and 34% decrease in deaths between 2015 and 2020. This finding is in keeping with a recent study done in Cambodia, which used GBD 2019 tuberculosis estimates to show greater reductions in the tuberculosis burden in children than in older adults.^11^ The substantial progress in reducing the tuberculosis burden in children might reflect recent trends showing marked decreases in child mortality,^54^ global improvements in child nutrition outcomes,^55^, ^56^ increasing tuberculosis case finding and services for children,^57^, ^58^, ^59^ and continued reductions in vertical HIV transmission.^60^, ^61^ Despite these improvements, the burden of tuberculosis in children remains high, with an estimated 89 800 deaths in 2020. With recent evidence suggesting that most tuberculosis transmission among children occurs outside the household,^62^ integrating effective household contact-tracing programmes^63^, ^64^ with community-based strategies (eg, routine or mass screening, targeted preventive therapy, and environmental interventions) will be particularly important for continued progress.

While individuals aged 50 years and older constituted 37% of all tuberculosis incident cases and 58% of all tuberculosis deaths in 2020, these age groups have shown slow progress in reducing the tuberculosis burden since 2015. These results suggest that tuberculosis in older adults poses a major challenge to tuberculosis control, and achieving the End TB targets will therefore require targeting these age groups. Previous studies have shown several challenges in controlling tuberculosis in older individuals. For example, older individuals are more likely to progress to active tuberculosis, they do not have classical clinical features,^65^ which has been shown to delay diagnosis and treatment,^17^ and they are more likely to have adverse reactions to treatment.^66^ Studies show that screening and treating older adults for latent tuberculosis is essential for meeting the 2025 interim End TB milestones.^67^ However, the high probability of adverse reactions to treatment, potentially contributing to higher likelihoods of interruptions to tuberculosis preventive therapy in this age group,^68^ underscores the importance of developing shorter, less toxic treatments, along with early diagnosis, for achieving the End TB targets.^69^

Our study highlights that one opportunity for reducing the tuberculosis burden among older adults and achieving the End TB targets is by better addressing tuberculosis risk factors. We found that eliminating tuberculosis risk factors would reduce global tuberculosis deaths by 37% in 2020 compared with 2015, with the largest reductions expected in individuals aged 50 years and older. Implementation of WHO's recent framework for collaborative action on tuberculosis and comorbidities^70^ across health systems will be important for addressing tuberculosis risk factors. The framework provides an evidence-based and patient-centred approach for coordinating with risk factor control initiatives throughout the continuum of tuberculosis care, including prevention, diagnosis and treatment, and care after the completion of tuberculosis treatment. A successful case study of the programme is Pakistan, which integrated smoking cessation support with routine tuberculosis care in 2017 and found that a quarter of participants stopped smoking.^71^ Mexico's innovative tuberculosis and diabetes partnership is another example that provides bidirectional services across the continuum of care and has been shown to improve tuberculosis treatment outcomes while screening 85% of all diagnosed tuberculosis cases for diabetes.^70^, ^72^

However, eliminating risk factors would still leave 1·00 million global tuberculosis deaths in 2020. This estimate underscores that other approaches, combined with addressing risk factors, will be important for reaching the upcoming End TB milestones. These approaches include considering the social determinants of tuberculosis, such as poverty, poor housing, overcrowding, undernutrition, and poor access to health care, which create environments conducive to widespread transmission. Tuberculosis subsequently augments existing social and economic vulnerabilities as it creates substantial losses in productivity^73^ and catastrophic financial costs^74^ for people living in poverty. The universal health coverage agenda will therefore be important to ending tuberculosis as it will ensure uninterrupted availability of and access to tuberculosis diagnostic tests and treatment for people at highest risk of tuberculosis.^73^

Finally, our secondary analysis of the impact of the COVID-19 pandemic on tuberculosis burden underscores the need for continued research and data. Although many models have predicted hundreds of thousands of additional tuberculosis deaths due to the COVID-19 pandemic,^75^, ^76^ our analyses of the available vital registration data have so far indicated variable impact in 2020 and 2021. Studies have shown substantial disruptions to tuberculosis health services, but a review of the impact of the COVID-19 pandemic on tuberculosis indicated that there are limited empirical data on the effects of the pandemic on tuberculosis outcomes.^77^ In the context of our analysis of 41 countries, we found many countries reporting lower than expected deaths, while a few countries had more deaths than expected in the absence of the COVID-19 pandemic, with Russia reporting an 8% increase in deaths in 2020 and a 15% increase in 2021, and Brazil reporting a 10% increase in 2021 (appendix 2 300–04). This analysis provides some indication that previously described disruptions in tuberculosis services and treatment in both Russia^78^, ^79^ and Brazil^80^ might have led to increases in tuberculosis mortality. However, this hypothesis does not explain why almost all the observed excess deaths occurred in individuals younger than 65 years. For example, this age group contributed all excess tuberculosis deaths in Russia and the USA in 2020 and 462 of the 480 excess deaths in Brazil in 2021 (appendix 2 300–04). If disruptions in tuberculosis services led to increases in tuberculosis mortality, then we would have seen increases in tuberculosis deaths across all age groups, including older age groups (≥65 years). Since this was not observed in our study, one potential explanation for the excess deaths in individuals younger than 65 years might be misclassification of COVID-19 deaths as tuberculosis deaths. A recent study in Brazil found marked levels of misclassified COVID-19 deaths in garbage codes and hypothesised that there are additional misclassified COVID-19 deaths in other respiratory diseases.^81^ Several other studies have also shown potential misclassified COVID-19 deaths in non-COVID-19 respiratory conditions,^82^, ^83^ which might be amplified in locations with scarce or inadequate SARS-CoV-2 diagnostic capabilities.^84^ Further investigations and scrutiny of cause of death data are urgently needed to understand the degree to which COVID-19 deaths were misassigned to tuberculosis and other respiratory conditions. Additionally, although several studies have shown disruptions to tuberculosis services, many found that tuberculosis treatment success rates did not change during the pandemic.^85^, ^86^ With additional evidence showing that the average duration until cure or death is 3 years for untreated patients with tuberculosis,^25^ this timeframe might have also provided opportunities for tuberculosis service recovery programmes^87^, ^88^, ^89^, ^90^ to mobilise and avert mortality.

Our results showed that many countries had negative excess (ie, lower than expected) deaths due to tuberculosis throughout the pandemic. For example, we found negative excess tuberculosis deaths in both 2020 and 2021 when aggregating across 41 of 204 countries, with notable reductions in the Philippines (a 14% reduction in 2020 and a 10% reduction in 2021), Peru (a 37% decrease in 2020), South Korea (a 10% decrease in 2020), and many other countries contributing negative excess deaths in both years (appendix 2 300–04). Other studies with empirical data have also reported similar reductions during the pandemic, with one study in South Africa reporting a decrease in undiagnosed tuberculosis deaths;^91^ another in Madurai, India, reporting a 50% decrease in deaths due to infectious diseases (which included deaths from tuberculosis);^92^ a study in Taiwan (province of China) reporting continued gradual decreases in tuberculosis mortality during the pandemic years;^93^ and a recent study in Indonesia showing a pre-pandemic to pandemic all-cause mortality rate ratio of 0·97 (95% CI 0·91–1·04) among people diagnosed with tuberculosis.^94^ These results suggest that public health measures for COVID-19 and mask wearing might have helped to prevent tuberculosis transmission, as observed with influenza^95^ and respiratory syncytial virus.^96^ Interestingly, our results showed that in most countries with reductions in tuberculosis mortality, the decreases were driven by the group aged 65 years and older. This observation provides some support for the hypothesis that individuals with tuberculosis and SARS-CoV-2 co-infection are more prone to dying from COVID-19 than individuals with SARS-CoV-2 infection only.^97^, ^98^ Considering that the cumulative percentage of the global population with SARS-CoV-2 infection at least once during the first 2 years of the pandemic was higher than 40%,^99^ that mortality due to COVID-19 is highest among older people,^100^ and the high fatality rates in individuals with tuberculosis and SARS-CoV-2 co-infection,^101^, ^102^ older individuals with co-infection, particularly those who were undiagnosed with tuberculosis at the beginning of the pandemic, might have died from COVID-19. Emerging evidence from India's national tuberculosis prevalence survey^103^ between 2019 and 2021 provides some support for potential reductions in transmission, as the study showed a marked reduction in tuberculosis prevalence coinciding with the peak in COVID-19 deaths during the second wave in the country. Although our analysis of vital registration data from 41 countries overall showed a variable impact of the COVID-19 pandemic on tuberculosis mortality, continued research is needed to accurately quantify this association, with a focus on potential misclassification of COVID-19 deaths as tuberculosis deaths and on the potential reduction in the numbers of patients with tuberculosis owing to patients with tuberculosis dying from COVID-19.

Insufficient empirical data on the impact of the COVID-19 pandemic on the tuberculosis burden is the primary reason we observed discrepancies in estimates between GBD 2021 and WHO's 2022 Global Tuberculosis Report^6^ for the years 2020 and 2021. Although both groups had similar estimates in 2019 (eg, both estimated approximately 1·4 million deaths), the tuberculosis results diverge in 2020–21, with GBD 2021 estimating 1·39 million (95% UI 1·28–1·54) deaths in 2020 and 1·35 million (1·23–1·52) deaths in 2021, whereas WHO estimated 1·49 million deaths in 2020 and 1·59 million deaths in 2021. Tuberculosis incidence estimates for 2021 were slightly lower in GBD 2021 (9·40 million [95% UI 8·36–10·5] cases) than in WHO's 2022 report (10·6 million [95% CI 9·9–11] cases) but with overlapping uncertainty intervals. WHO relied on mathematical models to generate tuberculosis incidence and death estimates for 26 countries in 2020–21. Due to the scarcity of empirical data, WHO made several assumptions, including assuming that any decreases in notification were solely attributed to delays in tuberculosis diagnosis and treatment. For GBD 2021, we did not make any changes to our modelling approach for estimation in 2020–21 as we could not validate potential assumptions with empirical data. For the years 2020–21, we used the same estimation framework as described in the methods to generate estimates for other years. The differing estimation approaches in 2020–21 subsequently also caused differences between GBD 2021 and WHO's 2022 report in estimates of the percentage changes towards the End TB milestones in 2021 (deaths: 5·9% for WHO *vs* 14·0% for GBD 2021; incidence: 10·0% for WHO *vs* 8·0% for GBD 2021; appendix 2 pp 150–213). As more empirical data on tuberculosis deaths and incidence during the pandemic become available, GBD and WHO estimates are expected to align more.

This study has multiple limitations. The primary limitation is data availability as there were gaps in data across countries, age groups, and years. In the absence of data, estimates were dependent on covariates with evidence of a biological relationship or strong relationship with tuberculosis, spatial relationships, and out-of-sample predictive validity assessments. The absence of data is reflected in larger uncertainty intervals. These data limitations also create challenges in our statistical triangulation approach, particularly in sub-Saharan Africa where data are more scarce, where our model attempts to triangulate between tuberculosis mortality and prevalence. In this study, we were unable to quantify tuberculosis mortality attributable to all important risk factors, with indoor air pollution and malnutrition not included. We plan to include malnutrition as a risk factor in future iterations of GBD as novel robust data establishing a causal link become available.^104^ We were also unable to quantify the burden attributable to the social determinants of tuberculosis (eg, poverty and educational attainment). Additionally, PAF estimations assume that there is a causal relationship between an exposure and an outcome without confounding, that exposure removal does not impact the distribution of unrelated risk factors, and that a feasible intervention to eliminate exposure exists.^105^ However, these assumptions might not always hold true in practice. Studies have also highlighted the potential for bias in Levin's formula for PAF calculation that we used in this analysis,^105^, ^106^ but the bias when using adjusted relative risks, as we have done here, is often small.^107^ Moreover, although we accounted for potential biases when quantifying the relationship between risk factors and tuberculosis through the inclusion of bias covariates, these covariates might not entirely identify and rectify bias if all or most input studies are inherently biased. Finally, we could not quantify the impact of the COVID-19 pandemic on the tuberculosis burden owing to insufficient empirical evidence of impact. Although the Institute for Health Metrics and Evaluation has estimated that there were 18 million excess all-cause deaths for the years 2020–21,^108^ there is a scarcity of data on what fraction of these, if any, are due to tuberculosis. Our assessment of the available vital registration data indicated that the COVID-19 pandemic did not significantly change tuberculosis mortality in 2020–21 in most countries. However, this analysis was restricted to 41 countries that had available data, which subsequently restricts the generalisability of our findings. The release of more data from other countries, particularly in settings with a high tuberculosis burden, and more years of data are urgently needed to understand the generalisability of our findings. In the next iteration of GBD, we plan to include new vital registration data, emerging prevalence surveys, and other empirical data sources to systematically quantify the impact of COVID-19 on tuberculosis incidence and mortality.

In conclusion, despite substantial progress in reducing the global burden of tuberculosis, our results show that the world did not achieve the 2020 interim End TB incidence and mortality milestones. Tuberculosis control programmes could consider closely evaluating those countries that achieved the 2020 milestones to better understand the drivers of their marked progress. We also observed that the pace of decline was unequal across age groups. Targeting the burden of disease in older adults (aged >50 years) will be crucial for achieving the upcoming End TB targets as this population represents a large share of the tuberculosis burden and has experienced minimal progress. Our analyses indicate that addressing risk factors, by collaborating with risk factor control initiatives, will also be important to reducing tuberculosis mortality among older adults and could be crucial for achieving the next End TB milestones. Finally, the impact of the COVID-19 pandemic on the global tuberculosis burden remains heterogenous and uncertain. Empirical data on the effects of the COVID-19 pandemic on tuberculosis are urgently needed.

## Data sharing

To download the data used in these analyses, please visit the Global Health Data Exchange website at https://ghdx.healthdata.org/record/ihme-data/gbd-2021-tuberculosis-incidence-mortality-1990-2021.

## Declaration of interests

J R Andrews reports grants or contracts from the US National Institutes of Health. Z Basharat reports other financial interests as an employee of Alpha Genomics, outside the submitted work. P J G Bettencourt reports other financial or non-financial interests as a project reviewer at Botnar Foundation, outside the submitted work. L Cegolon reports grants or contracts from ORCHESTRA project WP5 (grant agreement number 101016167); payment or honoraria for lectures, presentations, speakers bureaus, manuscript writing, or educational events from the University of Trieste (Italy) and University Pavia (Italy) academic lectures; and support for attending meetings or travel from the National Congress of the Italian Society of Occupational Medicine (Genoa: September, 2022; Turin: September, 2023) and the National Congress of the Italian Society of Public Health Medicine (Rome: May, 2023), all outside the submitted work. I Ilic reports support for this study from the Ministry of Education, Science and Technological development, Serbia (project number 175042, 2011–23). M Ilic reports support for this study from the Ministry of Education, Science and Technological development, Serbia (project number 451-03-47/2023-01/200111). N E Ismail reports unpaid leadership or fiduciary roles in board, society, committee, or advocacy groups, with the Malaysian Academy of Pharmacy as the Bursary and Council Member, outside the submitted work. K Krishan reports non-financial support from the UGC Centre of Advanced Study, CAS II, awarded to the Department of Anthropology, Panjab University (Chandigarh, India), outside the submitted work. M Lee reports support for this study from the Ministry of Education of South Korea and the National Research Foundation of Korea (NRF-2021R1I1A4A01057428) and Bio-convergence Technology Education Program through the Korea Institute for Advancement Technology (KIAT) funded by the Ministry of Trade, Industry and Energy (number P0017805). R J Maude reports support for this study from the Wellcome Trust, (grant number 220211) as it provides core funding for Mahidol Oxford Tropical Medicine Research Unit and contributes to their salary. L Monasta reports support for this study from the Italian Ministry of Health (Ricerca Corrente 34/2017), and payments made to the Institute for Maternal and Child Health IRCCS Burlo Garofolo. S Nomura reports support for this study from the Ministry of Education, Culture, Sports, Science and Technology of Japan (grant 21H03203) and from Precursory Research for Embryonic Science and Technology from the Japan Science and Technology Agency (grant JPMJPR22R8). V C F Pepito reports grants or contracts from the International Initiative for Impact Evaluation (3ie) and Sanofi Consumer Healthcare. L F Reyes reports grants or contracts, consulting fees, and payments or honoraria from MSD, GSK, and Pfizer, and support for attending meetings from GSK, outside the submitted work. O Rezahosseini reports grants or contracts from Rigshospitalet and A.P.Møllers fonden. J Ross reports grants or contracts from the US National Institutes of Health, the US Department of Veterans Affairs, and Merck. J P Silva reports salary support for this study from the Portuguese Foundation for Science and Technology. J A Singh reports consulting fees from AstraZeneca, Crealta/Horizon, Medisys, Fidia, PK Med, Two labs, Adept Field Solutions, Clinical Care Options, ClearView Healthcare Partners, Putnam Associates, Focus Forward, Guidehouse consulting (formerly Navigant), Spherix, MedIQ, Jupiter Life Science, UBM, Trio Health, Medscape, WebMD, Practice Point Communications, and the US National Institutes of Health and the American College of Rheumatology; payment of honoraria for lectures, presentations, speakers bureaus, manuscript writing, or education events as a member of the speaker's bureau of Simply Speaking; support for attending meetings as a past steering committee member of OMERACT (an international organisation that develops measures for clinical trials and receives funding from 12 pharmaceutical companies); participation on a data safety monitoring board or advisory board with the US Food and Drug Administration Arthritis Advisory Committee; leadership or fiduciary role in other board, society, committee, or advocacy groups, paid as a past steering committee member of OMERACT, unpaid as a Co-Chair of the Veterans Affairs Rheumatology Field Advisory Committee, and unpaid as an editor and Director of the UAB Cochrane Musculoskeletal Group Satellite Center on Network Meta-analysis; stock or stock options in atai Life Sciences, Kintara Therapeutics, Intelligent Biosolutions, Acumen Pharmaceuticals, TPT Global Tech, Vaxart Pharmaceuticals, Aytu BioPharma, Adaptimmune Therapeutics, GeoVax Labs, Pieris Pharmaceuticals, Enzolytics, Seres Therapeutics, Tonix Pharmaceuticals Holding, and Charlotte's Web Holdings; and previous stock options in Amarin, Viking, and Moderna Pharmaceuticals, all outside the submitted work. L K Stafford reports support for this study from the Bill & Melinda Gates Foundation and the Institute for Health Metrics and Evaluation (IHME). Z Wang reports grants or contracts from the Fred Hollows Foundation, Fonds de recherche du Québec - Santé, China Scholarship Council, and McGill University Global Health Scholars Program; consulting fees from the Fred Hollows Foundation; support for attending meetings or travel from McGill University (Graduate Mobility Award) and the Fred Hollows Foundation; and a leadership or fiduciary role in other board, society, committee, or advocacy groups, paid or unpaid as a member of the trainee advisory committee with the Consortium of Universities for Global Health, outside the submitted work. H Zhang reports grants or contracts from WHO research grants. M Zielińska reports other financial interests as an AstraZeneca employee, outside the submitted work.
