# Supplementary material for: Global, regional, and national age-specific progress towards the 2020 milestones of the WHO End TB Strategy: a systematic analysis for the Global Burden of Disease Study 2021
Source: Lancet Infect Dis. 2024 Jul;24(7):698–725. doi: 10.1016/S1473-3099(24)00007-0 (PMC11187709; doi:10.1016/S1473-3099(24)00007-0)
Supplement: Supplementary appendix 1 [file mmc1.pdf]

THE LANCET
Infectious Diseases

Supplementary appendix 1

This appendix formed part of the original submission and has been peer reviewed. We post it as supplied by the authors.

Supplement to: GBD 2021 Tuberculosis Collaborators. Global, regional, and national age-specific progress towards the 2020 milestones of the WHO End TB Strategy: a systematic analysis for the Global Burden of Disease Study 2021. *Lancet Infect Dis* 2024; published online March 19. https://doi.org/10.1016/S1473-3099(24)00007-0.

Appendix 1

Supplementary Methods Appendix to "Global, regional, and national age-specific progress towards the 2020 milestones of the END TB strategy: results from the Global Burden of Disease Study 2021"

This appendix provides further methodological detail and results for "Global, regional, and national age-specific progress towards the 2020 milestones of the END TB strategy: results from the Global Burden of Disease Study 2021."

All the material in the paper itself is novel although it builds off previous GBD tuberculosis burden analyses[1–4]. However, that parts of the supplemental methods appendix include sections adapted from the GBD Capstones published in The Lancet.[5,6] ICD System (International Classification of Diseases) and references are provided for reproduced sections.

Table of Contents

Methods **2**

Case Definition 2

Overview 4

Fatal Tuberculosis 6

Figure 1. Flowchart 6

Modelling fatal TB 6

Figure 2. Overall data quality by country 7

Estimating HIV-TB 14

Figure 3. Flowchart 14

Non-fatal Tuberculosis 16

Figure 4. Flowchart 16

Estimating TB incidence 20

Estimating TB prevalence 21

HIV-TB incidence and prevalence 26

Table 5. GATHER checklist of information that should be included in reports of global health estimates, with description of compliance and location of information 27

Comparative risk assessment **33**

High alcohol use 33

Smoking 44

Diabetes 56

Authors' Contributions 67

Methods

Case definition

Tuberculosis (TB) is an infectious disease caused by Mycobacterium tuberculosis. The case definition includes all forms of TB, including pulmonary TB and extrapulmonary TB, which are bacteriologically confirmed or clinically diagnosed. For TB, the ICD-10 codes are A10-A14, A15-A19.9, B90-B90.9, K67.3, K93.0, M49.0, N74.1, P37.0, U84.3, and ICD-9 codes are 010-019.9, 137-137.9, 138.0, 138.9, 320.4, 730.4-730.6. For HIV-TB, the ICD-10 code is B20.0

Overview

Our TB modelling strategy has not changed substantially from GBD 2019, but we made refinements to our modeling approach: we used the meta-regression with Bayesian priors, regularisation, and trimming (MR-BRT) model as the primary analytical engine to predict mortality-to-incidence (MI) ratios instead of a mixed- effects regression, and we used modelled excess mortality rate (EMR) as input in DisMod. First, we estimated risk-weighted prevalence of LTBI by location, year, age, and sex using data from population- based tuberculin surveys and cohort studies reporting the risk of developing active TB disease as a function of induration size. Next, we divided the inputs on prevalence (from surveys in low- and middle-income countries), incidence (notification data from countries with a four- or five-star rating, and estimated incidence for countries with a less than four-star rating), and cause-specific mortality rate (CSMR) by the risk-weighted LTBI prevalence to model TB among those at risk in each country. Next we ran MR-BRT (with GBD super-region fixed effects) using MI ratios (logit transformed) from locations with a 4- or 5- star rating on causes of death with HAQ index as a covariate anchoring the lower end of the HAQ index scale with a datapoint from the Bangalore study reporting that 49.2% of 126 untreated new pulmonary TB cases were dead at the end of the five-year follow up period, to predict age-sex-specific MI ratios for all locations and years. We then estimated age-sex-specific incidence using the predicted MI ratios and CSMR estimates. Finally, we modelled remission as a function of the HAQ index

and used estimated remission to convert MI ratios into excess mortality rates (EMR).

We used DisMod-MR 2.1, the GBD Bayesian meta-regression tool, to generate consistent trends in all parameters. We then multiplied the DisMod-MR 2.1 outputs by the risk-weighted prevalence of LTBI to get population-level estimates of all-form TB incidence and prevalence.

Fatal Tuberculosis

Figure 1. Tuberculosis (TB) mortality: input data, analytical processes, and outputs

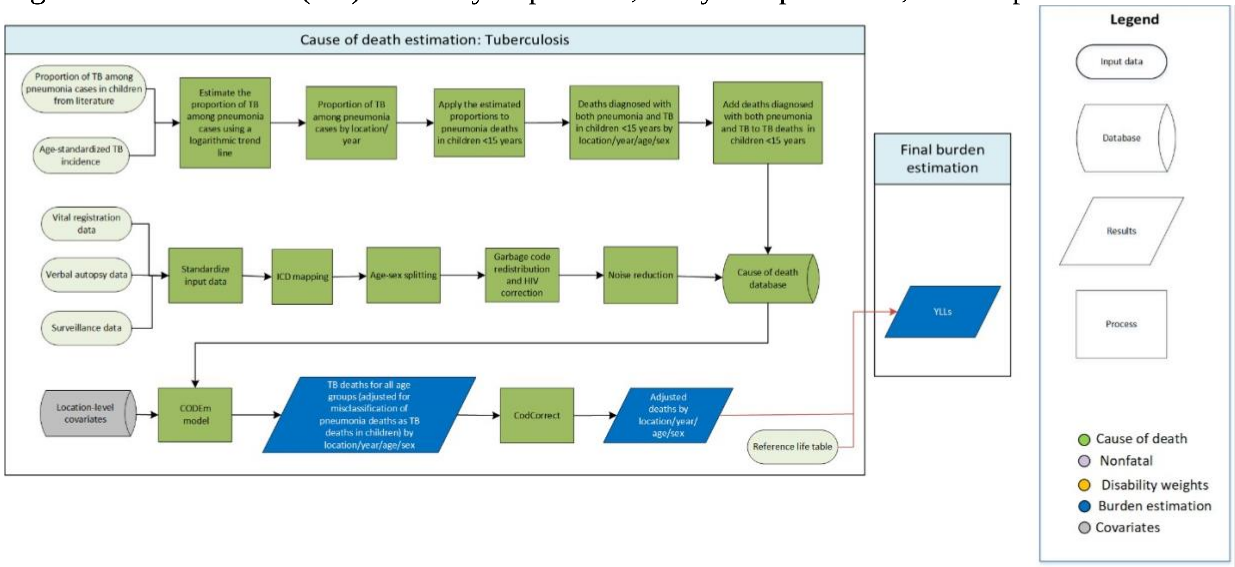

Modelling fatal TB

Input data for modelling tuberculosis (TB) mortality among HIV-negative individuals include vital registration, verbal autopsy, and surveillance data. Vital registration data were adjusted for garbage coding (including ill-defined codes and the use of intermediate causes) following GBD algorithms and misclassified HIV deaths (i.e. HIV deaths being assigned to other underlying causes of death such as tuberculosis or diarrhea because of stigma or misdiagnosis).

Verbal autopsy data in countries with age-standardised HIV prevalence greater than 5% were removed because of a high probability of misclassification, as verbal autopsy studies have poor validity in distinguishing HIV deaths from HIV-TB deaths.

Figure 2. Overall data quality by country

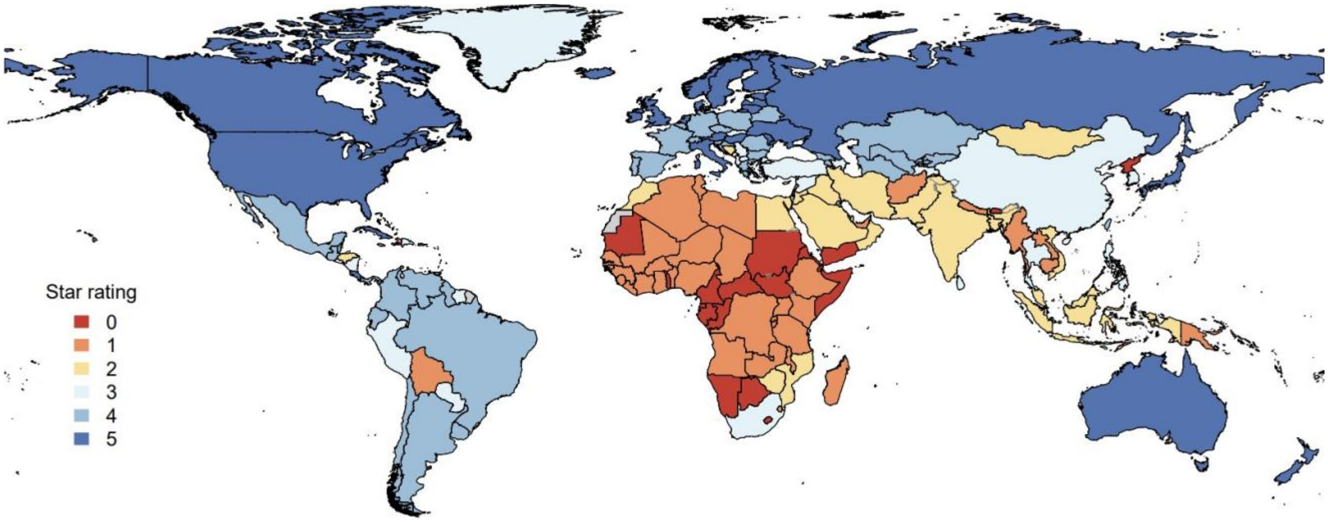

Redistribution[7]

A crucial aspect of enhancing the comparability of data for causes of death (CoD) is to deal with uninformative, so-called garbage codes. Garbage codes to which deaths were assigned should not be considered as the underlying CoD--for example: “heart failure”, “ill-defined cancer site”, “senility”, “ill-defined external causes of injuries”, and “septicaemia”. Redistribution is therefore the process of reallocating garbage-coded deaths to plausible underlying causes. For each group of diagnostically related garbage codes, we define a set of probable underlying causes of death and the proportion of garbage-coded deaths that are redistributed to each underlying cause, separately by GBD age group, sex, location, and year. The methods for redistributing these garbage-coded deaths have been previously described in detail.[7,8] While our underlying algorithm has not changed significantly since GBD 2013, several improvements were made in GBD 2019 and GBD 2021.

Redistribution of TB CoD data: Regress garbage codes versus non-garbage code[7]

For each redistribution package, we defined the “universe” of data as all deaths coded to either the package’s garbage codes or the package’s redistribution targets for each country, year, age, and sex. We then ran a regression based on the following equation separately for each target group and sex:

$$TG_{crt} = \alpha + \beta_1 Gar_{crt} + \beta_2 Age_{crt} Gar_{crt} + \theta_r Gar_{crt} + \gamma_r + \varepsilon_{ct}$$

Where:

TG_{crt} = percentage of deaths within the given garbage code's universe that were coded to a given target group, by country

Gar_{crt} = percentage of deaths within the given garbage code's universe that were coded to a given set of garbage codes

Age_{CRT} =age interaction term for the fixed effect on the interaction of garbage and age

α = constant

β_1 = slope coefficient describing the association between Gar_{crt} and G_{crt}

β_2 = slope coefficient describing the association between the interaction $Age_{crt}Gar_{crt}$ and G_{crt}

γ_r = region-specific random intercept (or super-region if the random effect on region is not significant)

θ_r = region-specific random slope (or super-region if the random effect on region is not significant)

ε_{ct} = standard error, normally distributed and calculated by bootstrapping

This regression was adjusted from GBD 2013 to include fixed effects on the interaction of garbage and age to ensure smooth age patterns. The random effects on location were included to help capture geographic differences in garbage coding for various causes. The regressions were first run with a random effect on the region, and in the case of failed convergence, they were attempted again with a random effect on the superregion. When models using a random effect on the superregion failed to converge, a fixed effect model considering only age was settled upon. We made this decision after investigating diagnostic visualisations that showed unlikely gaps between proportions assigned to different age groups.

Redistribution of TB CoD data: Computing redistribution uncertainty[1]

We assigned redistribution variance to each data point by calculating residual variance from a regression predicting the percentage of garbage coded deaths redistributed to a cause, given the proportion of garbage codes we observed for that location, year, age, sex, cause, and the age standardized relative

rate of major garbage codes across all causes. If there is a cause that has greater residual variance, we assume greater redistribution uncertainty.

To calculate variance, a dataset was generated containing percent garbage by location, year, age, sex, and cause, where percent garbage is determined by the equation:

$$pct_{garbage} = \frac{deaths_{redistributed} - deaths_{raw}}{deaths_{redistributed}}$$

A mixed-effect linear regression model was then fit to predict the logit percent of deaths from redistribution by age-standardized relative rate of major garbage codes.

$$logit\ (pct_{garbage_{ij}}) = \beta_0 + \beta_1 * \log(ASR_{majorgarbage_{ij}}) + \beta_2 * 15yearage_{ij} + \gamma_{1j} * \log(ASR_{majorgarbage_{ij}}) + u_j + e_{ij}, \quad \theta_{\{i\}} \sim N(0, \sigma^2)$$

Where:

i indexes dataset-location-year-age-sex-cause data points nested within *j* groups by GBD region

ASR major garbage: age standardized relative rate of major garbage

Residual variance, as estimated by the median absolute deviation (MAD), was calculated for each cause, sex, and age. The next step was to use the residual variance to calculate uncertainty around each data point in the CoD database. First, we calculated the percent garbage of each data point by treating all deaths that could not be directly mapped to a GBD cause as garbage. Percent garbage was calculated as:

$$pct_{garbage} = \frac{deaths_{redistributed} - deaths_{corrected}}{deaths_{corrected}}$$

Where:

death corrected: deaths post misdiagnosis correction

deaths redistributed: deaths post redistribution

Residual variance was matched to each data point and 100 draws were sampled from a normal distribution by using the cause, age, sex, specific residual variance, and mean of 0. The logit transformed percent

garbage was added to each value in the distribution. Each draw was then transformed out of logit space, and the post-redistribution deaths were calculated as

$$deaths = \frac{deaths_{corrected}}{1 - pct\_garbage}$$

Draws of deaths were processed through noise reduction before calculating the final redistribution variance passed to modeling in Cause of Death Ensemble modelling (CODEm) , which was added to the total data variance. The mean of the draws was not used as the final estimate because it was found that the logit transformation biases the distribution of cause fractions higher than if only point estimates are used.

HIV/AIDS misclassification correction[1]

In many location-years, certain causes of death known to be comorbid with HIV/AIDS (e.g., tuberculosis, other infectious diseases) are seen to have age patterns that diverge from those observed in location-years without widespread HIV epidemics and are in fact more reflective of HIV mortality trends. To identify these instances, a global relative age pattern is generated by using all VR deaths in countries with observed HIV prevalence less than 1% by using the following equation

$$RR_{asc} = \frac{R_{asc}}{\bar{x}(R_{65sc}, R_{70sc}, R_{75sc})}$$

Where:

RR_{asc} is the relative death rate for age group a, sex s, cause c

R_{asc} is the rate for that age group

$\bar{x}(R_{65sc}, R_{70sc}, R_{75sc})$ is the mean of the rates in ages 65–69, 70–74, and 75–79 for that sex and cause.

This is preferable to comparing mortality rates because we are able to isolate divergence in age pattern while accounting for varying levels of overall mortality by fixing death rates to age groups that are unlikely to be confounded by the presence of HIV. Expected deaths for an identified cause were then determined by the equation:

$$ED_{lyasc} = \bar{x}(R_{ly65sc}, R_{ly70sc}, R_{ly75sc}) \times p_{lasc} \times RR_{asc}$$

Where:

ED_{lasc} are deaths for location *l*, year *y*, age group *a*, sex *s*, and cause *c*;

$\bar{x}(R_{l65sc}, R_{l70sc}, R_{l75sc})$ is the mean of the rates for ages 65–69, 70–74, and 75–79 for that location-year-sex-cause

p_{lasc} is the population for that location-year-age-sex-cause

RR_{asc} is the global standard relative rate determined in the previous step for that age-sex-cause

The expected deaths remain attributed to that particular cause, while the difference between observed and expected are reallocated to HIV/AIDS.

Methods for correcting for a potential misclassification of tuberculosis deaths as pneumonia deaths in children

First, we estimated the proportion of tuberculosis among pneumonia cases as a function of age-standardized TB incidence using data from eight clinical studies[9–16] reporting the proportion of pneumonia cases that had tuberculosis (or the data to calculate them) and age-standardized TB incidence estimates. We used a logarithmic trend line to fit these data. Beginning in GBD 2019, we applied the estimated proportions to pneumonia deaths reported in data among children younger than 15 years to compute the number of deaths diagnosed with both pneumonia and TB, which were then added to child TB data. Following this correction in our input data, the CODEm model was ran to provide location-year-age-sex specific estimates.

The Cause of Death Ensemble model (CODEm)

TB mortality trends among HIV-negative individuals was modelled using the Cause of Death Ensemble modelling (CODEm) strategy, which is based on five general principles: identifying all available data, enhancing the comparability and quality of the dataset, developing a diverse set of possible models, assessing the predictive validity of all models, and selecting the models with the best performance in out-of- sample predictive validity tests. Possible models were identified using a covariate selection algorithm that yielded many plausible combinations of covariates which were then run through four classes of models. These model classes include modeling natural log rates and logit cause fractions using mixed effects linear models and spatiotemporal Gaussian Process Regression models. This generated a large variety of models that competed in predictive validity tests. An ensemble of CODEm models that performed best on out-of-sample predictive validity tests was then selected. In the following sections, we briefly provide overviews of CODEm components but additional details on how candidate models were developed, evaluated, and selection of best model are found elsewhere.[17]

Table 1: Candidate covariates and priors evaluated in CODEm for tuberculosis

	**Covariate**	**Direction**
Level 1	TB prevalence<br>Latent TB infection prevalence<br>TB summary exposure value (SEV) scalar<br>Liters of alcohol consumed per capita<br>Smoking prevalence<br>Cigarettes per capita<br>Fasting plasma glucose<br>TB strain prevalence-weighted transmission risk	+<br>+<br>+<br>+<br>+<br>+<br>+<br>+
Level 2	Healthcare access and quality (HAQ) Index<br>Adult underweight proportion<br>Indoor air pollution<br>Outdoor air pollution<br>Population density	-<br>+<br>+<br>+<br>+
Level 3	Log lag distributed income (LDI) per capita<br>Education (years per capita)<br>Socio-demographic Index (SDI)	-<br>-<br>-

I. Model pool development in CODEm

Because many factors may co-vary with TB deaths, a range of plausible statistical models are developed. In the CODEm framework, four families of statistical models are used: linear mixed effects regression (LMER) models of the natural log of the cause-specific death rate, LMER models of the logit of the cause fraction, spatiotemporal Gaussian process regression (ST-GPR) models of the natural logarithm of the cause-specific death rate, and ST-GPR models of the logit of the cause fraction (see the 2x2 table in Foreman et al). For each family of models, all plausible relationships between covariates and the response variable are identified. Covariates are categorized into three groups based on how strong the evidence of a causal relationship with TB: Covariates with strong proximal relations to TB, such as smoking, alcohol consumption, and diabetes, are ranked as level 1. TB covariates for which there is strong evidence for a relationship, but not a direct biological link, are placed in level 2. Covariates with weak evidence for a relationship, or which would be distal in the causal chain and thus may be mediated by factors in levels 1 or 2, are categorized as level 3. Based on the literature, we assign a prior on the direction of each covariate. Table 1 outlines the covariates for TB mortality and their expected directions. Because all possible combinations of selected covariates are considered for each family of models, multi-collinearity between covariates may produce implausible signs on coefficients or unstable coefficients. Each combination is therefore tested for statistical significance (covariate coefficients must have a coefficient with $p\text{-value} < 0{\cdot}05$) and plausibility (the coefficients must have the directions expected on the basis of the literature). Only covariate combinations meeting these criteria are retained. This selection process is run for both cause fractions and death rates, then ST-GPR and LMER-only models are created for each set of covariates. For a detailed explanation of differing covariate selection algorithms by covariate level, see Foreman et al.

II. Data variance estimation in CODEm

The families of models that go through ST-GPR incorporate information about data variance. The main inputs for a Gaussian process regression (GPR) are a mean function, a covariance function, and data variance for each data point. These inputs are described in detail in Foreman et al. Three components of data

variance are now used in CODEm: sampling variance, non-sampling variance, and garbage code redistribution variance. The computation of sampling variance and non-sampling variance has not changed since previous iterations of the GBD and is also described in Foreman et al. Garbage code redistribution variance is computed in the CoD database process. Since variance is additive, we calculate total data variance as the sum of sampling variance, non-sampling variance, and redistribution variance. Increased data variance in GPR results in the GPR draws not following the data point as closely.

III. Testing model pool on 15% sample

The performance of all models (individual and ensemble) is evaluated by means of out-of-sample predictive validity tests. Thirty percent of the data are randomly excluded from the initial model fits. These individual model fits are evaluated and ranked by using half of the excluded data (15% of the total), then used to construct the ensembles on the basis of their performance. Data are held out from the analysis on the basis of the cause-specific missingness patterns for ages and years across locations. Out-of-sample predictive validity testing is repeated 20 times for each model, which has been shown to produce stable results. These performance tests include the root mean square error (RMSE) for the log of TB death rate, the direction of the predicted versus actual trend in the data, and the coverage of the predicted 95% UI.

IV. Ensemble development and testing in CODEm

The component models are weighted on the basis of their predictive validity rank to determine their contribution to the ensemble estimate. The relative weights are determined both by the model ranks and by a parameter ψ, whose value determines how quickly the weights taper off as rank decreases. The distribution of ψ is described in more detail in Foreman et al. A set of ensemble models is then created by using the weights constructed from the combinations of ranks and ψ values. These ensembles are tested by using the predictive validity metrics described in the previous section on the remaining 15% of the data, and the ensemble with the best performance in out-of-sample trend and RMSE is chosen as the final model.

V. Final estimation in CODEm

Once a weighting scheme has been chosen, 1000 draws are created for the final ensemble, and the number of draws contributed by each model is proportional to its weight. The mean of the draws is used as the final estimate for the CODEm process, and a 95% UI is created from the 0·025 and 0·975 quantiles of the draws. The validity of the UI can be checked via its coverage of the out-of-sample data; ideally, the 95% UI would capture 95% of these data. Higher coverage suggests that the UIs are too large, and lower coverage suggests overfitting.

VI. Overview of CODEm parameters

Lambda: Controls smoothing as CODEm smooths residuals over age, time and space to add more information about patterns across countries, age, and time. The process works by computing residuals and weighting them by age and spatio-temporal proximity to data points. The weighted residuals are then added to the predictions. The weighting scheme is similar to standard LOESS weights and allows for points closer in space, time, and age to have higher weights while points further away to have lower weights. Across the GBD, a lambda as high as 2 is typically used for causes with sparse data to induce greater smoothing across time while a lambda as low as 0.5 is used for causes that are more data rich. For TB, a lambda value of 1.25 was used for smoothing over time.

Psi (ψ): CODEm assigns a rank for each of the component models based on how they perform in terms of root-mean-squared error and trend. Based on the ranks that are assigned to each component model, CODEm converts the ranks to weights that sum to 1. CODEm uses a monotonically decreasing function that determines what weight to apply to component models in their ranked order. The Psi parameter determines how quickly the weights decline and influences the relative weighting of models with Foreman et al. providing more details of Psi. For example, a Psi of 1.2 would provide the top performing model a weight of 32.2%, the second model 26.8%, the third 22.4%, and the last 18.6%. A Psi value of 1 provides equal weight across all models. CODEm selects the best Psi value by selecting the one that provides the lowest

metric in terms of RMSE and trend. For TB, CODEm selected a Psi value of 1.31.

Holdout ensemble proportion: Proportion of data in each of the knockouts to hold out to test the overall Ensemble performance. Currently set to 15 percent.

VII. Comparisons of TB mortality inputs to TB outputs after CODEm

The following table compares TB mortality vital registration data that was used as input into CODEm and final estimated TB mortality after modeling for select countries with data between 2015 and 2019. Complete time series of model fits by location, age, and sex will soon be available at the following website after the GBD 2021 iteration of the GBD is finalized: https://www.healthdata.org/data-tools-practices/interactive-visuals/causes-death-cod-visualization

Table 2. Comparison of TB deaths from vital registration data to final estimated TB deaths in GBD 2021

		TB deaths from vital registration (VR) data			Final estimated TB deaths in GBD 2021		
**Location**	**Age group**	**2015**	**2019**	**Percent change**	**2015**	**2019**	**Percent change**
Russia	All Ages	11100	6480	-41.6	10900 (10800-11000)	6500 (6410-6590)	-40.30 (-40.88, -39.80)
Russia	Under 5	57	22	-62.5	65 (60-68)	22 (20-23)	-66.46 (-68.69, -64.02)
Russia	5-14 years	9	7	-24.8	10 (9-10)	7 (6-7)	-32.55 (-34.26, -31.01)
Russia	15-49 years	4950	2520	-49.1	4820 (4770-4880)	2570 (2540-2610)	-46.65 (-47.34, -46.08)
Russia	50-69 years	5210	3190	-38.7	5120 (5040-5190)	3160 (3110-3220)	-38.14 (-38.83, -37.52)
Russia	70+ years	875	741	-15.3	877 (832-901)	735 (689-757)	-16.17 (-17.19, -15.30)
Mexico	All Ages	2420	2650	9.62	2440 (2370-2500)	2620 (2560-2700)	7.34 (5.18, 9.63)
Mexico	Under 5	26	32	23.1	27 (22-33)	29 (23-36)	4.17 (-7.93, 25.67)
Mexico	5-14 years	30	31	2.73	25 (23-28)	29 (26-31)	12.16 (5.14, 21.31)
Mexico	15-49 years	909	1000	10.5	890 (870-910)	987 (963-1020)	10.93 (8.49, 13.51)
Mexico	50-69 years	790	937	18.6	837 (814-860)	939 (913-974)	12.17 (10.09, 15.18)
Mexico	70+ years	663	647	-2.46	662 (630-686)	638 (602-663)	-3.65 (-5.53, -1.38)

Note: Final estimated TB deaths may not always match due to country-specific death envelopes and previous trends may impact estimates. The 2015 and 2019 columns represented TB deaths without HIV coinfection in those years for each respective data sources.

Estimating fatal HIV-TB

Figure 3. HIV-Tuberculosis mortality: input data, analytical processes, and outputs

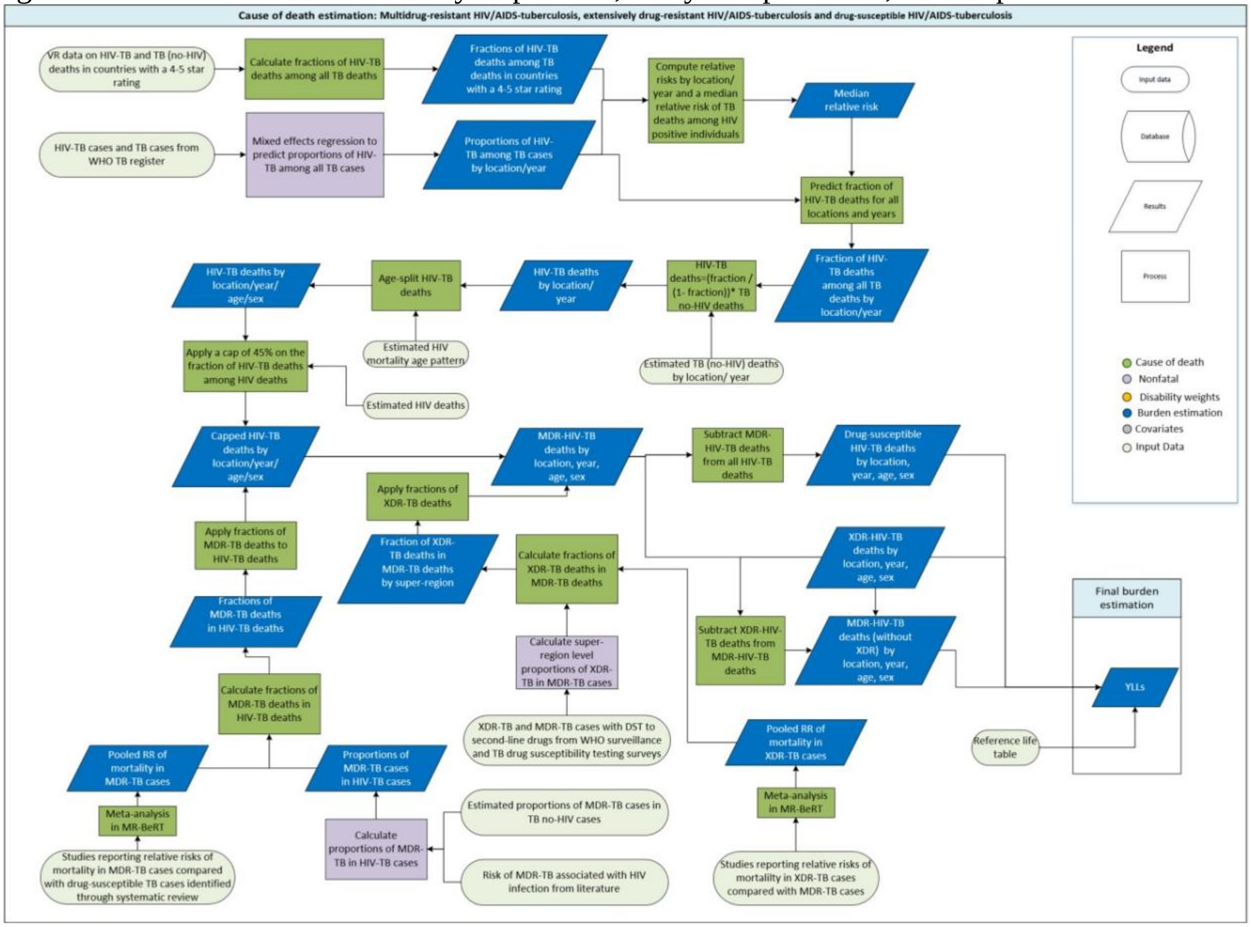

Input data for HIV/AIDS-tuberculosis (HIV-TB) mortality estimation include: (1) 1,277 site-years of vital registration data from countries with a four- or five-star rating where cause of death data for directly coded HIV-TB and tuberculosis (TB) were available, and (2) the number of TB cases (new and re-treatment) recorded as HIV-positive and the number of TB cases (new and re-treatment) with an HIV test result recorded in the TB register from the World Health Organization (WHO). We excluded data from countries with ten HIV-TB deaths or less. We also excluded data that were largely conflicting with the majority of data for other years from the same country.

To determine TB deaths in HIV-positive individuals, we first computed the fraction of HIV-TB deaths among all TB deaths using vital registration data from countries with a four- or five-star rating. We also calculated the proportion of TB cases that are HIV-positive (ie, number of TB cases recorded as HIV-

positive/number of TB cases with an HIV test result recorded in the WHO TB register). We used these proportions as input data for a mixed effects regression to predict the proportions of HIV-TB cases among all TB cases for all locations and years using an adult HIV death rate covariate. We estimated the fraction of HIV-TB deaths among all TB deaths in each location and year ($D_{c,y}$), defined by

$$D_{c,y} = \frac{P_{c,y}RR}{P_{c,y}RR + 1 - P_{c,y}}$$

where $P_{c,y}$ is the proportion of HIV-TB cases among all TB cases and RR is the relative risk of TB deaths in HIV positive individuals, defined by:

$$RR = \frac{D_{c,y}P_{c,y} - D_{c,y}}{D_{c,y}P_{c,y} - P_{c,y}}$$

We took the median relative risk (RR) from each calculation. We then applied the median RR and the predicted proportions of HIV-TB cases among all TB cases to get the fractions of HIV-TB deaths among all TB deaths for all locations and years. Location-year-specific HIV-TB deaths were then calculated using the following equation:

$$Deaths_{HIV-TB} = \frac{D_{c,y}}{1 - D_{c,y}} Deaths_{TB}$$

where $Deaths_{TB}$ is location-year specific deaths from the CODEm TB no-HIV model. Finally, we applied the age-sex pattern of the HIV mortality estimates to these HIV-TB deaths to generate location-year-age-sex-specific HIV-TB deaths. As the HIV-TB deaths were estimated based on the fraction of HIV-TB deaths among all TB deaths, the total number of HIV-TB deaths could exceed the total number of HIV deaths in some locations. To avoid this, we applied a cap of 45% on the fraction of HIV-TB deaths among HIV deaths, based on a review by Cox and colleagues[18] and a systematic review and meta-analysis by Ford and colleagues[19].

Modelling non-fatal TB

Input data include annual case notifications, data from prevalence surveys, and estimated cause specific mortality (CSMR) of TB among HIV-positive and HIV-negative individuals. We divided the inputs on prevalence, incidence, and CSMR by the estimated latent TB infection (LTBI) prevalence weighted by the risk of progression to active TB in order to model TB among those at risk in each country. From these inputs, we modeled remission and used estimated remission to compute excess mortality to give more guidance to the model. We used DisMod-MR 2.1, the GBD Bayesian meta-regression tool that adjusts for differences in methods between data sources and imposes consistency between data for different parameters. We then multiplied the DisMod-MR 2.1 outputs by the prevalence of LTBI to get population-level estimates of incidence and prevalence. We explain in more detail below the preparation of each of the input data sources and the modelling in DisMod-MR 2.1. Updated systematic reviewers were done in GBD 2021 for TB prevalence surveys and LTBI tuberculin surveys. The search terms, number of studies identified, and number of studies included are shown in the table below:

Figure 4. Tuberculosis (TB) morbidity: input data, analytical processes, and outputs

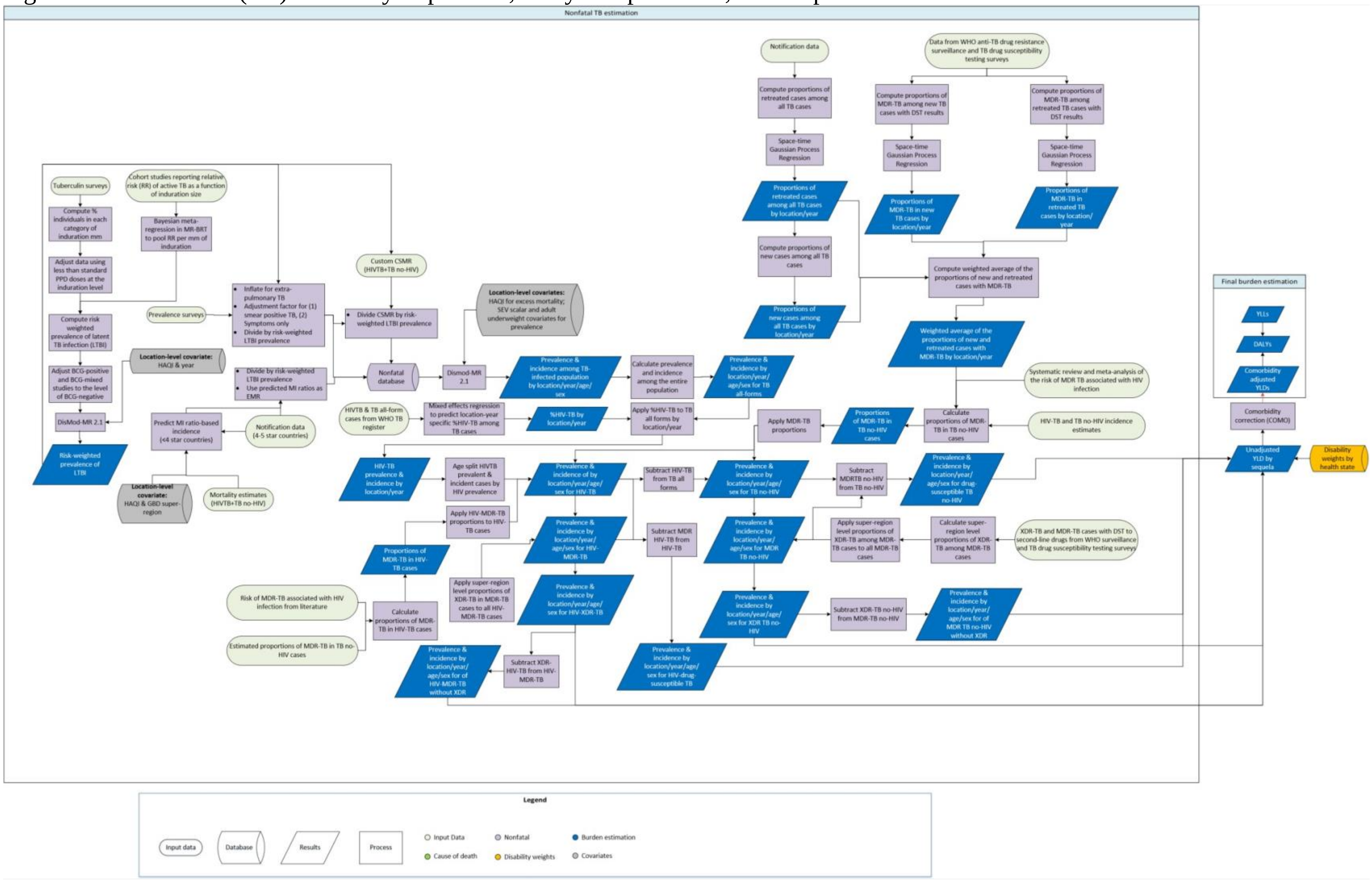

Note: Flowchart with code available at http://ghdx.healthdata.org/gbd-2019/code/nonfatal-13

Outcome	Search Terms	Total number of studies identified	Number of studies included
Tuberculosis	Pubmed: *("tuberculosis"[MeSH] OR tuberculosis[Title/Abstract] OR TB[Title/Abstract] OR Mycobacterium tuberculosis[Title/Abstract] AND prevalence[Title/Abstract] AND ("2019/02/01"[PDAT] : "2020/04/07"[PDAT]) NOT (animals\{SESH] NOT humans[MESH])*	479	3
LTBI (tuberculin surveys)	Pubmed: *("tuberculin survey"[tiab] OR (("risk"[MeSH Terms] OR "risk"[tiab] OR "risk of"[tiab]) AND ("tuberculosis"[MeSH Terms] OR "tuberculosis"[tiab] OR "tuberculous"[tiab]) AND ("infection"[MeSH Terms] OR "infection"[tiab])) OR (("risk"[MeSH Terms] OR "risk"[tiab] OR "risk of"[tiab]) AND TB[tiab] AND ("infection"[MeSH Terms] OR "infection"[tiab])) OR "latent tuberculosis infection"[tiab] OR "latent TB infection"[tiab] OR "latent tuberculosis"[MESH]) AND ("survey"[tiab] OR "surveys"[tiab]) NOT (animals[MESH] NOT humans[MESH]) ("2019/02/14"[PDAT] : "2020/03/30"[PDAT])*	31	1
LTBI (cohort studies)	Pubmed: ("tuberculin"[tiab] OR "Mantoux"[tiab] OR "induration"[tiab]) AND ("active"[tiab] AND ("tuberculosis"[MeSH] OR "tuberculosis"[tiab]) OR ("reactivation"[tiab] OR "reactivity"[tiab])) AND ("prospective"[tiab] OR "cohort"[tiab] OR "follow up"[tiab]) (“2019/02/13”[PDAT] : "2019/03/30"[PDAT])<br><br>Embase: ('tuberculin':ab,ti OR 'mantoux':ab,ti OR 'induration':ab,ti) AND ('active':ab,ti AND ('tuberculosis'/exp OR 'tuberculosis') OR 'reactivation':ab,ti OR 'reactivity':ab,ti) AND ('prospective':ab,ti OR 'cohort':ab,ti OR 'follow up':ab,ti) AND [1-2-2019]/sd NOT [2-4-2020]/sd	117	2

PRISMA Diagram of TB All Forms Prevalence in GBD2021

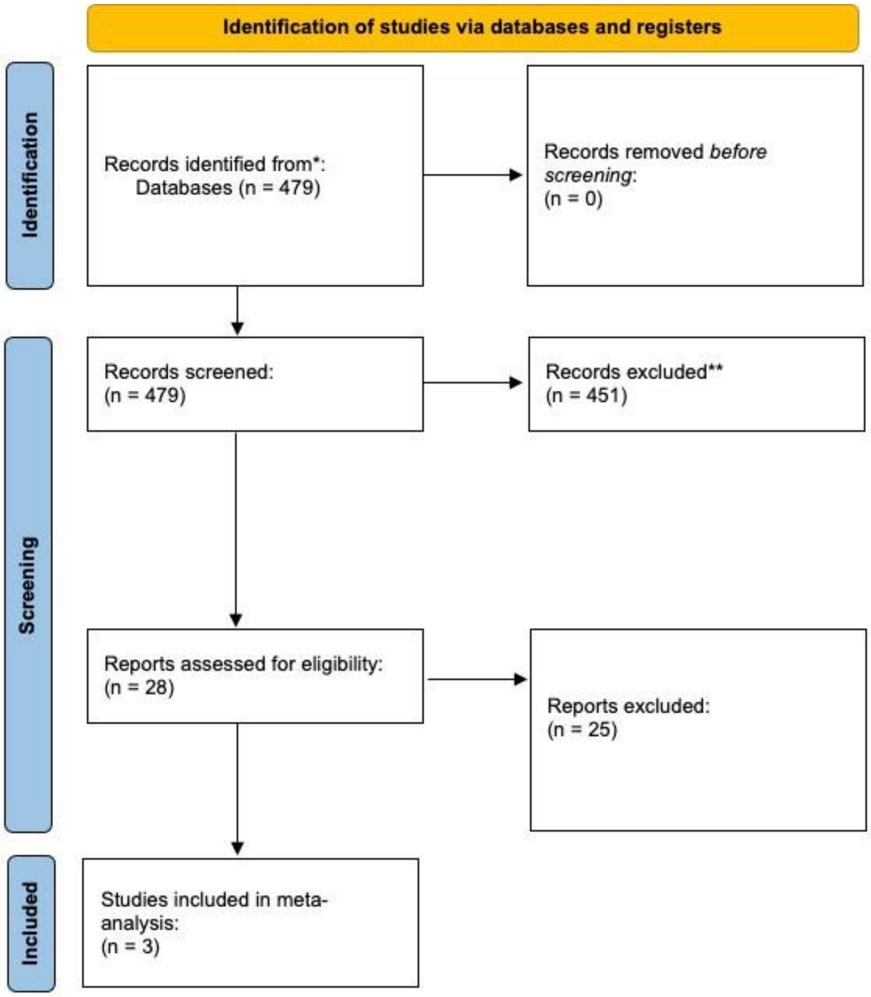

From: Page MJ, McKenzie JE, Bossuyt PM, Boutron I, Hoffmann TC, Mulrow CD, et al. The PRISMA 2020 statement: an updated guideline for reporting systematic reviews. BMJ 2021;372:n71. doi: 10.1136/bmj.n71

For more information, visit: http://www.prisma-statement.org/

PRISMA diagram of latent tuberculosis infections in GBD 2021

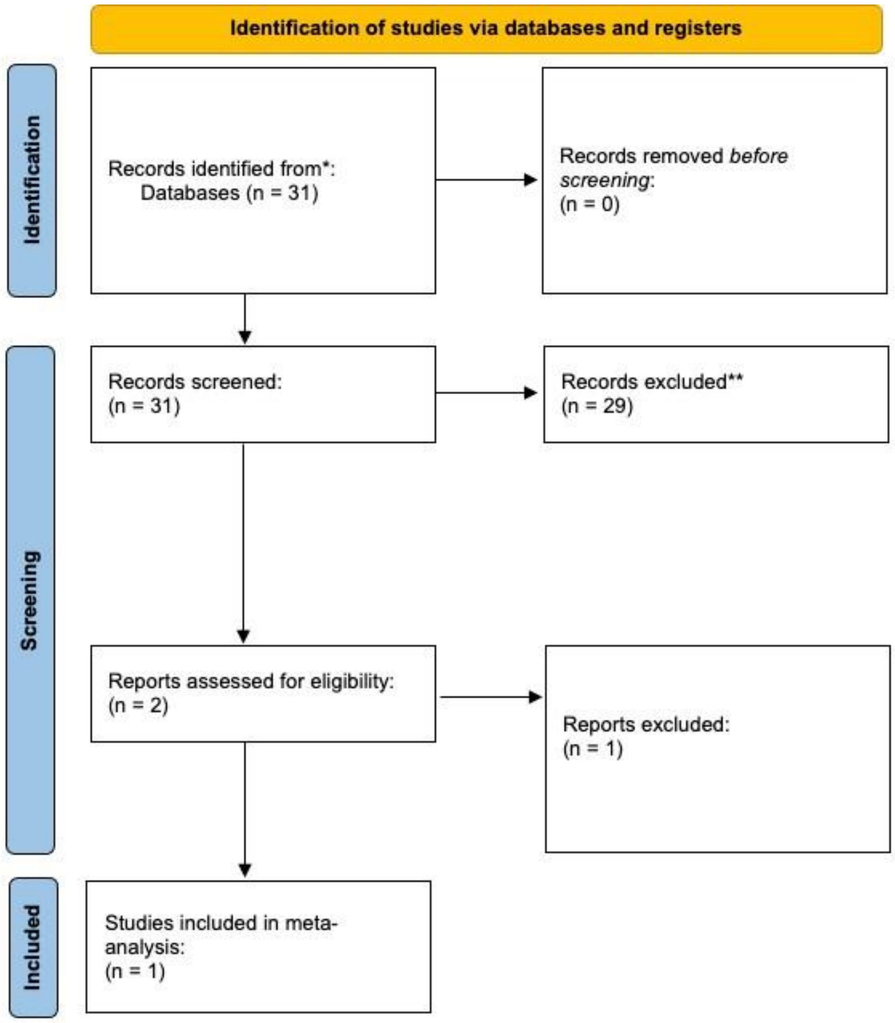

From: Page MJ, McKenzie JE, Bossuyt PM, Boutron I, Hoffmann TC, Mulrow CD, et al. The PRISMA 2020 statement: an updated guideline for reporting systematic reviews. BMJ 2021;372:n71. doi: 10.1136/bmj.n71

For more information, visit: http://www.prisma-statement.org/

Modelling TB incidence

Incidence inputs were from two different sources: (1) incidence from notification data for countries with a 4- or 5-star rating on their cause of death data as a proxy for the quality of health-related administrative data systems, and (2) estimated incidence for countries with a less than four-star rating. We used the age- and sex-specific notifications (all new and relapse cases combined) in our analysis. Prior to 2013, notification data were available by case type (new pulmonary smear-positive, new pulmonary smear-negative, and new extrapulmonary) and there were missing age data, especially for younger age groups in some countries. We imputed the missing age groups for the three forms of TB notifications. Smear-positive age-specific notifications were inflated with the proportion smear-unknown and relapsed cases only reported at the country-year level. Some countries reported only pulmonary smear-positive cases for selected years. Missing smear-negative and extrapulmonary cases were predicted from the adjusted smear-positive cases using a seemingly unrelated regression. All three types of notifications were added together to represent TB-all-form incidence for countries with a four- or five-star rating.

To generate incidence estimates for locations with a less than four-star rating, we implemented the MR-BRT model[20] with age and sex dummies and super-region fixed effects, using MI ratios (logit transformed) from locations with a 4- or 5-star rating on causes of death as input data with HAQ index[21,22] as a covariate anchoring the lower end of the HAQ index scale with a datapoint from a cohort study in the 1960s reporting that 49.2% of 126 untreated new pulmonary TB cases were dead at the end of the five-year follow-up period[23], in order to predict age-sex-specific MI ratios for all locations and years. We then used the MI ratios and cause-specific mortality estimates to compute the incidence input for DisMod-MR 2.1 for locations with a less than four-star rating. Finally, we computed the age-sex-specific incidence of TB among the latent TB-infected population, using TB incidence as the numerator and our estimated risk-weighted latent TB infection prevalence as the denominator.2.1 for locations with a less than four-star rating. Finally, we computed the age-sex-specific incidence of TB among the latent TB-infected

population, using TB incidence as the numerator and our estimated risk- weighted latent TB infection prevalence as the denominator.

In order to better align with case notification, we made an update to our approach in GBD 2021. We first determine the upper limit (99th percentile) of the fraction of all TB case notifications that are likely true TB cases (i.e. those that are bacteriologically confirmed) from countries with high quality information systems (countries with 4-5 star ratings as determined by our cause of death star rating system). We took the 99th percentile value and created a ratio with TB incidence estimates from DisMod MR 2.1. This resulting ratio was then applied to countries with low quality data to determine the likely true TB case notification rate. The primary objective for our update was to account for the fact that not all notified cases are bacteriologically confirmed and might lead to over estimation for TB incidence.

Modelling TB prevalence

Data from prevalence surveys reporting on pulmonary smear-positive TB and bacteriologically positive TB were included. Because incidence data are for all forms of TB, we adjusted prevalence surveys to account for extrapulmonary cases. We ran a spatiotemporal Gaussian process regression to predict location-year-age-sex-specific proportions of extrapulmonary TB among all TB cases using data on the three forms of TB from the incidence data above. We then computed the extrapulmonary inflation factor as *1+(proportion of extrapulmonary TB /(1- proportion of extrapulmonary TB))*, and applied it to data from prevalence surveys.

In GBD 2021, we used the MR-BRT model to derive adjustment factors for studies where the case definition was smear-positive TB rather than bacteriologically positive TB (reference category). For the adjustment, we identified all prevalence surveys that provided comparisons of smear-positive TB and bacteriologically positive TB from the same sample. Overall, 16 prevalence surveys from Cambodia, China, Ethiopia, Gambia, India, Myanmar, South Korea, the Philippines, Rwanda, and Vietnam were

included as inputs in the MR-BRT model. The model also contained covariates for sex and age to reflect gradients across demographics. In GBD 2021, we also computed an adjustment factor to adjust studies that used symptoms only as a screening method compared to studies using both symptoms and chest X-ray during screening (reference category). To derive the adjustment factor, we ran a MR-BRT model with data from six studies[24–29] comparing prevalence between using symptoms only as opposed to symptoms and chest X-ray in the same population as input. The adjustment factors are in the table below.

Finally, we computed the prevalence of TB among the TB-infected population, using TB prevalence as the numerator and our estimated risk-weighted LTBI prevalence as the denominator. We included two location-level covariates, namely, age-standardised adult underweight prevalence and log-transformed age-standardised summary exposure value (SEV) scalar for TB (a summary variable of the exposure levels of TB risk factors weighted by relative risk) to help inform variation of TB prevalence over year and geography.

Table 3. MR-BRT crosswalk relative odds ratio for tuberculosis prevalence

Reference or alternative case definition	Gamma	Beta coefficient, log (95% CI)	Relative rate ratio*
Bacteriologically positive	0.23	---	---
Smear positive		–0.46 (–0.70 to –0.22)	0.63 (0.50 to 0.80)
Symptoms and chest X-ray	0	---	---
Symptoms only		–0.37 (–0.50 to –0.25)	0.69 (0.61 to 0.78)

**MR-BRT crosswalk adjustments can be interpreted as the factor the alternative case definition is adjusted by to reflect what it would have been had it been measured using the reference case definition. If the log beta coefficient is negative, then the alternative is adjusted up to the reference. If the log beta coefficient is positive, then the alternative is adjusted down to the reference. The adjustment factor column is the exponentiated beta coefficient. For log beta coefficients, this is the relative rate between the two case definitions. The uncertainty in the adjustment was factor was incorporated during adjustments to non-reference prevalence data points by adding the variance of the adjustment factor to the variance in the data point.*

Modelling TB remission and excess mortality

In GBD 2021, we computed TB duration based on a systematic review of studies during the pre-chemotherapy era finding that duration from onset to cure or death is 3 years.[30] To anchor the lowest end of TB duration we assumed a duration of 6 months based on treatment regimens. We then linearly interpolated between 6 months and 3 years across the HAQ index to compute TB duration for every

country-year. We converted duration into remission by taking the inverse (ie, remission = 1/duration). Using HAQ index-based remission and estimated MI ratios, we computed excess mortality rate (EMR) with the following computation:

EMR = MI * Remission (formula derived from Prevalence = Incidence * Duration)

DisMod-MR 2.1

DisMod MR 2.1 description[31]

In GBD 2021, no substantial changes were made to DisMod-MR 2.1. The sequence of estimation occurs at five levels: global, super-region, region, country and, where applicable, subnational location. The super-region priors are generated at the global level with mixed-effects, nonlinear regression using all available data; the super-region fit, in turn, informs the region fit, and so on down the cascade. Subnational estimation was informed by the country fit and country covariates, plus an adjustment based on the average of the residuals between the subnational location’s available data and it’s prior. This mimicked the impact of a random effect on estimates between subnationals. At each level of the cascade, the DisMod-MR 2.1 enforces consistency between all parameters. Analysts have the choice to branch the cascade in terms of time and sex at different levels depending on data density. We used the default option to model TB, which is to branch by sex after the global fit but to retain all years of data until the lowest level in the cascade.

The coefficients for country covariates were re-estimated at each level of the cascade. For a given location, country coefficients were calculated using both data and prior information available for that location. In GBD 2021, we generated model fits for the years 1990, 1995, 2000, 2005, 2010, 2015, 2017, 2019, 2020 and 2021, and log-linearly interpolated estimates for the intervening years. The 95% uncertainty intervals were computed based on 1000 draws from the posterior distribution of the model using the 2.5th and 97.5th percentiles of the ordered 1000 values.

DisMod-MR 2.1 likelihood estimation

Analysts have the choice of using a Gaussian, log-Gaussian, Laplace or Log-Laplace likelihood function in DisMod-MR 2.1. We used the default log-Gaussian equation for the data likelihood, which is:

$$-log[p(y_j|\Phi)] = \log(\sqrt{2\pi}) + \log(\delta_j + s_j) + \frac{1}{2}(\frac{\log(a_j + \eta_j) - \log(m_j + \eta_j)}{\delta_j + s_j})^2$$

where, y_j is a 'measurement value' (i.e., data point); Φ denotes all model random variables; η_j is the offset value, eta, for a particular 'integrand' (prevalence, incidence, remission, excess mortality rate, cause-specific mortality rate) and a_j is the adjusted measurement for data point j, defined by:

$$a_j = e^{(-u_j - c_j)} y_j$$

where u_j is the total 'area effect' (i.e., the sum of the random effects at three levels of the cascade: super-region, region and country) and c_j is the total covariate effect (i.e., the mean combined fixed effects for sex, study level and country level covariates), defined by:

$$c_j = \sum_{k=0}^{K[I(j)]-1} \beta_{I(j),k} \hat{X}_{k,j}$$

with standard deviation

$$s_j = \sum_{l=0}^{L[I(j)]-1} \zeta_{I(j),l} Z_{k,j}$$

where k denotes the mean value of each data point in relation to a covariate (also called x-covariate); I(j) denotes a data point for a particular integrand, j; $\beta_{I(j),k}$ is the multiplier of the kth x-covariate for the ith integrand; $\hat{X}_{k,j}$ is the covariate value corresponding to the data point j for covariate k; l denotes the standard deviation of each data point in relation to a covariate (also called z-covariate); $\zeta_{I(j),k}$ is the multiplier of the lth z-covariate for the ith integrand; and δ_j is the standard deviation for adjusted measurement j, defined by:

$$\delta_j = log[y_j + e^{(-u_j - c_j)}\eta_j + c_j] - log[y_j + e^{(-u_j - c_j)}\eta_j]$$

Where m_j denotes the model for the jth measurement, not counting effects or measurement noise and defined by:

$$m_j = \frac{1}{B(j) - A(j)} \int_{A(j)}^{B(j)} I_j \text{(a) da}$$

where A(j) is the lower bound of the age range for a data point; B(j) is the upper bound of the age range for a data point; and I_j denotes the function of age corresponding to the integrand for data point j.

The source code for DisMod-MR 2.1 as well as the wrapper code is available at the following link:

https://github.com/ihmeuw/ihmemodelling/tree/master/gbd_2017/shared_code/central_comp/nonfatal/dismod.

Internally consistent modelling in DisMod-MR 2.1

For each location, we included the following as input in the DisMod model: case notifications for locations with a four- or five-star rating, predicted MI-ratio-based incidence for locations with a less than four-star rating, prevalence survey data where available, predicted excess mortality estimates, HAQ-based remission, and CSMR (TB and HIV-TB combined) by age and sex.

The output from the DisMod model was for all forms of TB in TB-infected populations, including both HIV-negative and HIV-positive individuals. We computed the incidence and prevalence of TB among the entire population by multiplying the prevalence of LTBI with the DisMod model estimates. Betas and exponentiated values from the DisMod model are shown in the eTable4.

Table 4. Beta coefficients and exponentiated values from the DisMod model

Covariate	Parameter	Prior on Beta	Beta (95% CI)	Exponentiated beta (95% CI)
Sex (male)	Prevalence	----	0.34 (0.31–0.38)	1.41 (1.36–1.46)
Sex (male)	Incidence	----	0.38 (0.38–0.39)	1.47 (1.46–1.47)
Age-standardised proportion adult underweight	Prevalence	0 to 3	2.39 (2.03–2.71)	10.88 (7.61–15.10)
Age-standardised SEV scalar (log-transformed)	Prevalence	0.75 to 1.25	0.75 (0.75–0.76)	2.12 (2.12–2.13)

HIV-TB incidence and prevalence

To distinguish HIV-TB from all forms of TB, we first estimated the proportions of HIV-TB cases among all TB cases using data on the number of TB cases recorded as HIV-positive and the number of TB cases with an HIV test result recorded in the WHO TB notifications register. We ran a mixed effects regression using the adult HIV death rate as a covariate to predict location-year-specific HIV-TB proportions, which were then applied to TB incident and prevalent cases from DisMod, to generate HIV-TB incident and prevalent cases by location and year. These cases were then age-sex split based on the age-sex pattern of estimated HIV prevalence by location-year to generate location-year-age-sex-specific HIV-TB incident and prevalent cases.

Details of tuberculosis duration

There are limited empirical population-based data on TB duration. To guide the TB burden estimation process in DisMod, we computed TB duration based on a systematic review of studies[30] during the pre-chemotherapy era finding that duration from onset to cure or death was 3 years. To anchor the lowest end of TB duration we assumed a duration of 6 months based on treatment regimens. We then linearly interpolated between 6 months and 3 years across the HAQ index to derive all-age and both-sex TB duration for every country-year. We then used these estimates of TB durations as priors in DisMod, and leverage DisMod's statistical triangulation approach using age-sex-specific TB mortality, prevalence, and incidence data to derive age-sex-specific TB duration estimates. The table below shows the subsequent implied all-form TB duration estimates in 2020 from this approach. Due to the scarcity of data, we have not estimated TB duration separately among HIV-positive and HIV-negative people. In the future rounds of the GBD, we can explore the possibility of using HIV-stratified notification and prevalence data to quantify TB duration by HIV status. However, there are concerns about using HIV-TB data from TB prevalence surveys because of high HIV testing refusal rates, reliance on self-reported measurements of HIV status, and incomplete HIV data from participants in national TB prevalence surveys.[32–35] We will

discuss this with GBD collaborators to see if they can help us identify additional data for estimating the duration of TB by HIV status.

Implied all-form TB duration by age group and by sex

Age-group	Sex	Implied all-form TB duration
All ages	Both sexes	1.75 (1.63-1.86)
Under 5 years	Both sexes	2.52 (2.23-2.74)
5 to 14 years	Both sexes	1.75 (1.45-2.16)
15 to 49 years	Both sexes	1.65 (1.50-1.83)
50 to 69 years	Both sexes	1.58 (1.41-1.78)
70 plus years	Both sexes	2.39 (2.12-2.69)
All ages	Female	1.78 (1.65-1.89)
All ages	Male	1.72 (1.61-1.84)

Note: TB duration was computed by taking the ratio of prevalence to incidence in the year 2020.

Overview of MR-BRT[20]

This section details the statistical models underlying the MR-BRT model, and fitting procedure to obtain estimates. This model was used in two instances in our TB modeling approach: (1) mortality-to-incidence (MI) ratio approach and (2) to derive adjustment factors for studies where the case definition was smear-positive TB rather than bacteriologically positive TB (reference category). Full details are in a recent publication by Zheng et al.[20]

The MR-BRT program is a set of wrappers customized for global health problems that use the open source mixed effects package LimeTr (https://github.com/zhengp0/limetr). We describe the basic functionality in the sections below.

I. Mixed-Effects Model

We consider the following nonlinear mixed effects model:

$$\begin{aligned} \boldsymbol{y}_i &= \mathbf{F}_i(\beta) + \mathbf{Z}_i\boldsymbol{u}_i + \boldsymbol{\epsilon}_i \\ \boldsymbol{u}_i &\sim N(\mathbf{0}, \boldsymbol{\Gamma}),\ \boldsymbol{\Gamma} = \mathrm{diag}(\gamma), \qquad \boldsymbol{\epsilon}_i \sim N(\mathbf{0}, \boldsymbol{\Lambda}), \end{aligned} \tag{1}$$

where $y_i \in \mathbb{R}^{n_i}$ is the vector of observations from the ith study, $\boldsymbol{\epsilon}_i \in \mathbb{R}^{n_i}$ are measurement errors with given covariance $\boldsymbol{\Lambda}$, $\boldsymbol{u}_i \in \mathbb{R}^{k_\gamma}$ are independent random effects, and $\mathbf{Z}_i \in \mathbb{R}^{n_i \times k_\gamma}$ is a linear map, and β are

regression coefficients. The models F_i may be nonlinear.

To fit (β, γ) we solve the marginal likelihood problem:

$$\min_{\beta,\gamma} f(\beta,\gamma) \coloneqq \sum_{i=1}^{m} \frac{1}{2}\left(y_i - \mathbf{F}_i(\beta)\right)^{\top} \left(\mathbf{Z}_i \boldsymbol{\Gamma} \mathbf{Z}_{\mathrm{i}}^{\top} + \boldsymbol{\Lambda}_{\boldsymbol{i}}\right)^{-1} \left(\boldsymbol{y}_i - \mathbf{F}_i(\beta)\right) + \frac{1}{2} ln\left|\mathbf{Z}_i \boldsymbol{\Gamma} \mathbf{Z}_{\mathrm{i}}^{\top} + \boldsymbol{\Lambda}_i\right| . \tag{2}$$

When the model is linear, we can write:

$$\mathbf{F}_i(\beta) = \boldsymbol{X}\beta. \tag{3}$$

II. Constraints and Priors

The ML estimate (2) can be extended to incorporate nonlinear inequality constraints

$$\mathbf{C}(\boldsymbol{\theta}) \leq c,$$

where $\boldsymbol{\theta} = (\beta, \gamma)$. Constraints play a key role for polynomial splines.

It is also essential to allow priors on parameters of interest. We assume that priors are given by a functional form:

$$\boldsymbol{\theta} \sim \exp\left(-\rho(\boldsymbol{\theta})\right)$$

The likelihood problem is then augmented by adding the term $\rho(\boldsymbol{\theta})$ to the ML objective. The function ρ may be nonlinear and nonconvex, but we assume it is smooth.

III. Trimming outliers

This Least trimmed squares (LTS) is a robust estimator for the standard regression problem[36]. Given the problem:

$$\min_{\beta} \sum_{i=1}^{n} \frac{1}{2} \left(y_i - \langle \boldsymbol{X}_i, \beta \rangle\right)^2, \tag{4}$$

The LTS estimator minimizes the sum of *smallest h* residuals rather than all residuals. These estimators were initially introduced to develop linear regression estimators that have a high breakdown point (in this case 50%) and good statistical efficiency (in this case $n^{-1/2}$). Breakdown refers to the percentage of outlying points which can be added to a dataset before the resulting M-estimator can change in an unbounded way. Here, outliers can affect both the outcomes and training data (features).

The set

$$\Delta_h \coloneqq \{\mathbf{W} : \mathbf{1}^\top \mathbf{W} = h, \quad \mathbf{0} \le \mathbf{W} \le \mathbf{1}\} \tag{6}$$

is known as the *capped simplex*, since it is the intersection of the h-simplex with the unit box. For a fixed β, the optimal solution of (5) with respect to $\mathbf{W}$ assigns weight 1 to each of the smallest h residuals, and 0 to the rest. Problem (5) is solved *jointly* in $(\beta, \mathbf{W})$, simultaneously finding the regression estimate and classifying the observations into inliers and outliers. This joint strategy makes LTS different from post-hoc analysis, where a model is fit first with all data, and then outliers are detected using that estimate. To explain how trimming enters the marginal likelihood problem, we focus on a single group term from the ML likelihood (2):

$$\left(\frac{1}{2}\left(\boldsymbol{y}_i - \mathbf{F}_{i(\beta)}\right)^\top \left(\mathbf{Z}_i \boldsymbol{\Gamma}^{-1} \mathbf{Z}_i^\top + \boldsymbol{\Lambda}_i\right)^{-1} \left(\boldsymbol{y}_i - \mathbf{F}_i(\beta)\right) + \frac{1}{2} \ln |\mathbf{Z}_i \boldsymbol{\Gamma}^{-1} \mathbf{Z}_i^\top + \boldsymbol{\Lambda}_i|\right)$$

We introduce auxiliary variables $\mathbf{W}_i \in \mathbb{R}^{n_i}$, and define

$$\boldsymbol{r}_i \coloneqq y_i - \mathbf{F}_i(\beta), \quad \mathbf{W}_i \coloneqq \operatorname{diag}(\mathbf{W}_i), \quad \sqrt{\mathbf{W}_i} \coloneqq \operatorname{diag}\left(\sqrt{\mathbf{W}_i}\right).$$

We now form the objective

$$\tfrac{1}{2} \boldsymbol{r}_I^\top \sqrt{\mathbf{W}_i} \left(\sqrt{\mathbf{W}_i} \mathbf{Z}_i \boldsymbol{\Gamma}^{-1} \mathbf{Z}_i^\top \sqrt{\mathbf{W}_i} + \boldsymbol{\Lambda}_i^{\odot \mathbf{W}_i}\right)^{-1} \sqrt{\mathbf{W}_i}\, \boldsymbol{r}_i + \tfrac{1}{2} \ln \left| \sqrt{\mathbf{W}_i}\, \mathbf{Z}_i \boldsymbol{\Gamma}^{-1} \mathbf{Z}_i^\top \sqrt{\mathbf{W}_i} + \boldsymbol{\Lambda}_i^{\odot \mathbf{W}_i} \right|, \tag{7}$$

where $\odot$ denotes the elementwise power operation:

$$\boldsymbol{\Lambda}_i^{\odot \mathbf{W}_i} \coloneqq \begin{bmatrix} (\lambda_{1j})^{w_{i1}} & 0 & \cdots & 0 \\ 0 & \ddots & \ddots & \vdots \\ 0 & \cdots & 0 & (\lambda_{in_i})^{w_{in_i}} \end{bmatrix} \tag{8}$$

When $w_{ij} = 1$, we recover the contribution of the ijth observation to the original likelihood. As $w_{ij} \downarrow 0$, the ijth contribution to the residual is correctly eliminated by $\sqrt{w_{ij}} \downarrow 0$. The jth row and column of $\sqrt{\mathbf{W}_i} \mathbf{Z}_i \boldsymbol{\Gamma}^{-1} \mathbf{Z}_i^\top \sqrt{\mathbf{W}_i}$ both go to 0, while the jth entry of $\boldsymbol{\Lambda}_i^{\odot \mathbf{W}_i}$ goes to 1, which effectively removes all impact of the jth point on the covariance matrix.

IV. Final Estimator

Putting together the trimmed ML with priors and constraints, we arrive at the following estimator.

$$\min_{\beta,\gamma,\mathbf{W}} f(\beta,\gamma,\mathbf{W}) := \sum_{i=1}^{m} \frac{1}{2} r_i^\top \sqrt{\mathbf{W}_i} \left(\sqrt{\mathbf{W}_i}\, \mathbf{Z}_i \mathbf{\Gamma}^{-1} \mathbf{Z}_i^\top \sqrt{\mathbf{W}_i} + \mathbf{\Lambda}_i^{\odot \mathbf{W}_i}\right)^{-1}$$

$$\sqrt{\mathbf{W}_i}\, r_i + \frac{1}{2} \ln |\sqrt{\mathbf{W}_i}\, \mathbf{Z}_i \mathbf{\Gamma}^{-1} \mathbf{Z}_i^\top \sqrt{\mathbf{W}_i} + \mathbf{\Lambda}_i^{\odot \mathbf{W}_i}| + \rho(\beta,\gamma,\mathbf{\Lambda}) \tag{9}$$

$$\text{s.t.} \quad \boldsymbol{r}_i = \boldsymbol{y}_i - \mathbf{F}_i(\beta), \quad \mathbf{1}^\top \mathbf{W} = h, \quad 0 \leq \mathbf{W} \leq 1, \quad \boldsymbol{C}\begin{pmatrix}\beta \\ \gamma\end{pmatrix} \leq c\,.$$

The fit is obtained using iterative optimization techniques. Problem (9) is nonlinear and non-smooth, and the optimization is implemented in the LimeTR package (https://github.com/zhengp0), and relies on the IPopt interior point method[37].

V. Applications of MR-BRT to TB estimation

As previously described, we used MR-BRT in the following instances: (1) mortality-to-incidence (MI) ratio approach, (2) to derive adjustment factors for studies where the case definition was smear-positive TB rather than bacteriologically positive TB (reference category), and (3) adjustment factors for studies with screening strategies of symptoms only as opposed to symptoms and chest X-ray. For the MI ratio regression, the only included covariate was the HAQ index. For (2), sex and age were included as covariates while no covariates were used in (3). In all of these models, we did not specify any splines nor priors. However, we did allow for trimming of 10% of the data during estimation. The random effects terms were on study.

Table 5. GATHER checklist of information that should be included in reports of global health estimates, with description of compliance and location of information for " Global, regional, and national age-specific progress towards the 2020 milestones of the END TB strategy: results from the Global Burden of Disease Study 2021".

#	GATHER checklist item	Description of compliance	Reference
**Objectives and funding**			
1	Define the indicators, populations, and time periods for which estimates were made.	Narrative provided in paper and methods appendix describing indicators, definitions, and populations	Main text (Introduction, pg. 5) and methods appendix (pg. 3–6)
2	List the funding sources for the work.	Funding sources listed in paper	Main text (Methods; pg. 8)
**Data Inputs**			
For all data inputs from multiple sources that are synthesized as part of the study:			
3	Describe how the data were identified and how the data were accessed.	Narrative description of data seeking methods provided	Main text (Methods; pg. 8, 10) and methods appendix (pg. 6, 16, 20)
4	Specify the inclusion and exclusion criteria. Identify all ad-hoc exclusions.	Narrative about inclusion and exclusion criteria by data type provided	Main text (Methods; pg. 5–7) and methods appendix (pg. 7, 18–20)
5	Provide information on all included data sources and their main characteristics. For each data source used, report reference information or contact name/institution, population represented, data collection method, year(s) of data collection, sex and age range, diagnostic criteria or measurement method, and sample size, as relevant.	An interactive, online data source tool that provides metadata for data sources by component, geography, cause, risk, or impairment has been developed	Information provided in the following online data file: https://ghdx.healthdata.org/record/ihme-data/gbd-2021-tuberculosis-incidence-mortality-1990-2021
6	Identify and describe any categories of input data that have potentially important biases (e.g., based on characteristics listed in item 5).	Summary of known biases included in methods appendix	Methods appendix (pg. 7, 22–24)
For data inputs that contribute to the analysis but were not synthesized as part of the study:			

7	Describe and give sources for any other data inputs.	Included in online data source tool, https://ghdx.healthdata.org/record/ihme-data/gbd-2021-tuberculosis-incidence-mortality-1990-2021	Information provided in the following online data file: https://ghdx.healthdata.org/record/ihme-data/gbd-2021-tuberculosis-incidence-mortality-1990-2021
For all data inputs:			
8	Provide all data inputs in a file format from which data can be efficiently extracted (e.g., a spreadsheet as opposed to a PDF), including all relevant meta-data listed in item 5. For any data inputs that cannot be shared due to ethical or legal, reasons, such as third-party ownership, provide a contact name or the name of the institution that retains the right to the data.	Downloads of input data available through online tools, including data visualization tools and data query tools, https://ghdx.healthdata.org/record/ihme-data/gbd-2021-tuberculosis-incidence-mortality-1990-2021; input data not available in tools will be made available upon request	The Global Health Data Exchange https://ghdx.healthdata.org/record/ihme-data/gbd-2021-tuberculosis-incidence-mortality-1990-2021

**Data analysis**			
9	Provide a conceptual overview of the data analysis method. A diagram may be helpful.	Flow diagrams of the overall methodological processes, as well as cause-specific modelling processes, have been provided	Main text (Methods; pg. 6-7)and methods appendix (eFigure 1, 3, 4)
10	Provide a detailed description of all steps of the analysis, including mathematical formulae. This description should cover, as relevant, data cleaning, data pre-processing, data adjustments and weighting of data sources, and mathematical or statistical model(s).	Flow diagrams and methodological write-ups	Main text (Methods; pg. 6–7)and methods appendix (eFigure 1, 3, 4)
11	Describe how candidate models were evaluated and how the final model(s) were selected.	Provided in the methodological write-up	Methods appendix (pg. 12–13)
12	Provide the results of an evaluation of model performance, if done, as well as the results of any relevant sensitivity analysis.	Provided in the methodological write-up	Methods appendix (pg. 12–13)
13	Describe methods for calculating uncertainty of the estimates. State which sources of uncertainty were, and were not, accounted for in the uncertainty analysis.	Provided in the methodological write-up	Main text (Methods, pg. 7–8) and methods appendix (8–27)
14	State how analytic or statistical source code used to generate estimates can be accessed.	Access statement provided	Code is provided in an online tool, https://ghdx.healthdata.org/record/ihme-data/gbd-2021-tuberculosis-incidence-mortality-1990-2021
**Results and Discussion**			
15	Provide published estimates in a file format from which data can be efficiently extracted.	Results are available through online data visualization tools, the Global Health Data Exchange, and the online data query tool https://ghdx.healthdata.org/record/ihme-data/gbd-2021-tuberculosis-incidence-mortality-1990-2021	Main text (table 1-3), methods appendix (etable 6-11), and online data tools (data visualization tools, data query tools, and the Global Health Data Exchange, https://ghdx.healthdata.org/record/ihme-data/gbd-2021-tuberculosis-

			incidence-mortality-1990-2021
16	Report a quantitative measure of the uncertainty of the estimates (e.g. uncertainty intervals).	Uncertainty intervals are provided with all results	Main text (Results, table 1–3), methods appendix (etables 6-11), and online data tools

References

1. Ledesma JR, Ma J, Vongpradith A, Maddison ER, Novotney A, Biehl MH, et al. Global, regional, and national sex differences in the global burden of tuberculosis by HIV status, 1990–2019: results from the Global Burden of Disease Study 2019. Lancet Infect Dis. 2022 Feb;22(2):222–41.
2. Kyu HH, Maddison ER, Henry NJ, Ledesma JR, Wiens KE, Reiner R, et al. Global, regional, and national burden of tuberculosis, 1990–2016: results from the Global Burden of Diseases, Injuries, and Risk Factors 2016 Study. Lancet Infect Dis [Internet]. 2018 Dec;18(12):1329–49. Available from: https://linkinghub.elsevier.com/retrieve/pii/S147330991830625X
3. Kyu HH, Maddison ER, Henry NJ, Mumford JE, Barber R, Shields C, et al. The global burden of tuberculosis: results from the Global Burden of Disease Study 2015. Lancet Infect Dis [Internet]. 2018 Mar;18(3):261–84. Available from: https://linkinghub.elsevier.com/retrieve/pii/S147330991730703X
4. Ma J, Vongpradith A, Ledesma JR, Novotney A, Yi S, Lim K, et al. Progress towards the 2020 milestones of the end TB strategy in Cambodia: estimates of age and sex specific TB incidence and mortality from the Global Burden of Disease Study 2019. BMC Infect Dis. 2022 Dec 3;22(1):904.
5. Vos T, Lim SS, Abbafati C, Abbas KM, Abbasi M, Abbasifard M, et al. Global burden of 369 diseases and injuries in 204 countries and territories, 1990–2019: a systematic analysis for the Global Burden of Disease Study 2019. The Lancet [Internet]. 2020 Oct;396(10258):1204–22. Available from: https://linkinghub.elsevier.com/retrieve/pii/S0140673620309259
6. Murray CJL, Aravkin AY, Zheng P, Abbafati C, Abbas KM, Abbasi-Kangevari M, et al. Global burden of 87 risk factors in 204 countries and territories, 1990–2019: a systematic analysis for the Global Burden of Disease Study 2019. The Lancet [Internet]. 2020 Oct;396(10258):1223–49. Available from: https://linkinghub.elsevier.com/retrieve/pii/S0140673620307522
7. Johnson SC, Cunningham M, Dippenaar IN, Sharara F, Wool EE, Agesa KM, et al. Public health utility of cause of death data: applying empirical algorithms to improve data quality. BMC Med Inform Decis Mak. 2021 Dec 2;21(1):175.
8. Naghavi M, Makela S, Foreman K, O'Brien J, Pourmalek F, Lozano R. Algorithms for enhancing public health utility of national causes-of-death data. Popul Health Metr [Internet]. 2010 Dec 10;8(1):9. Available from: https://pophealthmetrics.biomedcentral.com/articles/10.1186/1478-7954-8-9
9. Adegbola RA, Falade AG, Sam BE, Aidoo M, Baldeh I, Hazlett D, et al. The etiology of pneumonia in malnourished and well-nourished Gambian children. Pediatr Infect Dis J [Internet]. 1994 Nov;13(11):975–82. Available from: http://www.ncbi.nlm.nih.gov/pubmed/7845751
10. Chisti MJ, Graham SM, Duke T, Ahmed T, Ashraf H, Faruque ASG, et al. A prospective study of the prevalence of tuberculosis and bacteraemia in Bangladeshi children with severe malnutrition and pneumonia including an evaluation of Xpert MTB/RIF assay. PLoS One [Internet]. 2014;9(4):e93776. Available from: http://www.ncbi.nlm.nih.gov/pubmed/24695758
11. Madhi SA, Petersen K, Madhi A, Khoosal M, Klugman KP. Increased disease burden and antibiotic resistance of bacteria causing severe community-acquired lower respiratory tract infections in human immunodeficiency virus type 1-infected children. Clin Infect Dis [Internet]. 2000 Jul;31(1):170–6. Available from: http://www.ncbi.nlm.nih.gov/pubmed/10913417
12. McNally LM, Jeena PM, Gajee K, Thula SA, Sturm AW, Cassol S, et al. Effect of age, polymicrobial disease, and maternal HIV status on treatment response and cause of severe pneumonia in South African children: a prospective descriptive study. The Lancet [Internet]. 2007 Apr;369(9571):1440–51. Available from: https://linkinghub.elsevier.com/retrieve/pii/S0140673607606709
13. Moore DP, Klugman KP, Madhi SA. Role of streptococcus pneumoniae in hospitalization for acute community-acquired pneumonia associated with culture-confirmed mycobacterium tuberculosis in Children. Pediatr Infect Dis J [Internet]. 2010 Dec;29(12):1099–104. Available from: http://journals.lww.com/00006454-201012000-00009
14. Nantongo JM, Wobudeya E, Mupere E, Joloba M, Ssengooba W, Kisembo HN, et al. High incidence of pulmonary tuberculosis in children admitted with severe pneumonia in Uganda. BMC Pediatr [Internet]. 2013 Dec 31;13(1):16. Available from: http://bmcpediatr.biomedcentral.com/articles/10.1186/1471-2431-13-16
15. Zar HJ, Hanslo D, Tannenbaum E, Klein M, Argent A, Eley B, et al. Aetiology and outcome of pneumonia in human immunodeficiency virus-infected children hospitalized in South Africa. Acta Paediatr [Internet]. 2001 Feb;90(2):119–25. Available from: http://www.ncbi.nlm.nih.gov/pubmed/11236037

16. Moore DP, Higdon MM, Hammitt LL, Prosperi C, DeLuca AN, Da Silva P, et al. The incremental value of repeated induced sputum and gastric aspirate samples for the diagnosis of pulmonary tuberculosis in young children with acute community-acquired pneumonia. Clinical Infectious Diseases [Internet]. 2017 Jun 15;64(suppl_3):S309–16. Available from: http://academic.oup.com/cid/article/64/suppl_3/S309/3858220/The-Incremental-Value-of-Repeated-Induced-Sputum
17. Foreman KJ, Lozano R, Lopez AD, Murray CJ. Modeling causes of death: an integrated approach using CODEm. Popul Health Metr [Internet]. 2012 Dec 6;10(1):1. Available from: http://pophealthmetrics.biomedcentral.com/articles/10.1186/1478-7954-10-1
18. Cox JA, Lukande RL, Lucas S, Nelson AM, Van Marck E, Colebunders R. Autopsy causes of death in HIV-positive individuals in sub-Saharan Africa and correlation with clinical diagnoses. AIDS Rev [Internet]. 2010;12(4):183–94. Available from: http://www.ncbi.nlm.nih.gov/pubmed/21179183
19. Ford N, Matteelli A, Shubber Z, Hermans S, Meintjes G, Grinsztejn B, et al. TB as a cause of hospitalization and in-hospital mortality among people living with HIV worldwide: a systematic review and meta-analysis. J Int AIDS Soc [Internet]. 2016 Jan;19(1):20714. Available from: http://doi.wiley.com/10.7448/IAS.19.1.20714
20. Zheng P, Barber R, Sorensen RJD, Murray CJL, Aravkin AY. Trimmed constrained mixed effects models: formulations and algorithms. Journal of Computational and Graphical Statistics [Internet]. 2021 Jul 3;30(3):544–56. Available from: https://www.tandfonline.com/doi/full/10.1080/10618600.2020.1868303
21. Haakenstad A, Yearwood JA, Fullman N, Bintz C, Bienhoff K, Weaver MR, et al. Assessing performance of the Healthcare Access and Quality Index, overall and by select age groups, for 204 countries and territories, 1990–2019: a systematic analysis from the Global Burden of Disease Study 2019. Lancet Glob Health. 2022 Dec;10(12):e1715–43.
22. Fullman N, Yearwood J, Abay SM, Abbafati C, Abd-Allah F, Abdela J, et al. Measuring performance on the Healthcare Access and Quality Index for 195 countries and territories and selected subnational locations: a systematic analysis from the Global Burden of Disease Study 2016. The Lancet [Internet]. 2018 Jun;391(10136):2236–71. Available from: https://linkinghub.elsevier.com/retrieve/pii/S0140673618309942
23. National Tuberculosis Institute. Tuberculosis in a rural population of South India: a five-year epidemiological study. Bull World Health Organ [Internet]. 1974;51(5):473–88. Available from: http://www.ncbi.nlm.nih.gov/pubmed/4549498
24. Datta M, Radhamani MP, Sadacharam K, Selvaraj R, Rao DL, Rao RS, et al. Survey for tuberculosis in a tribal population in North Arcot District. The International Journal of Tuberculosis and Lung Disease [Internet]. 2001 Mar;5(3):240–9. Available from: http://www.ncbi.nlm.nih.gov/pubmed/11326823
25. Health and Family Welfare Department Government of Gujarat. Population based survey for assessing prevalence of pulmonary tuberculosis cases in the state of Gujarat, India (2011-2012). 2013.
26. Gothi G, Narayan R, Nair S, Chakraborty A, Srikantaramu N. Estimation of prevalence of bacillary tuberculosis on the basis of chest X-ray and/or symptomatic screening. Indian Journal of Medical Research. 1976;64(8):1150–9.
27. Chadha VK, Kumar P, Anjinappa SM, Singh S, Narasimhaiah S, Joshi M V., et al. Prevalence of Pulmonary Tuberculosis among Adults in a Rural Sub-District of South India. Pai M, editor. PLoS One [Internet]. 2012 Aug 15;7(8):e42625. Available from: https://dx.plos.org/10.1371/journal.pone.0042625
28. Pg G, Sadacharam K, Narayanan P. Yield of pulmonary tuberculosis cases by employing two screening methods in a community survey. Int J Tuberc Lung Dis. 2006 Apr 1;10:343–5.
29. Datta M, Pg G, Appegowda B, Rao K, Gopalan B. Tuberculosis in north Arcot district of Tamil Nadu – a sample survey. Indian Journal of Tuberculosis. 2000 Jan 1;47.
30. Tiemersma EW, van der Werf MJ, Borgdorff MW, Williams BG, Nagelkerke NJD. Natural History of Tuberculosis: Duration and Fatality of Untreated Pulmonary Tuberculosis in HIV Negative Patients: A Systematic Review. Pai M, editor. PLoS One [Internet]. 2011 Apr 4;6(4):e17601. Available from: https://dx.plos.org/10.1371/journal.pone.0017601
31. Flaxman AD, Vos T, Murray CJ. An integrative metaregression framework for descriptive epidemiology. First. Seattle, WA: University of Washington; 2015.
32. Law I, Floyd K. National tuberculosis prevalence surveys in Africa, 2008–2016: an overview of results and lessons learned. Tropical Medicine & International Health. 2020 Nov 12;25(11):1308–27.
33. Chanda-Kapata P, Kapata N, Klinkenberg E, Grobusch MP, Cobelens F. The prevalence of HIV among adults with pulmonary TB at a population level in Zambia. BMC Infect Dis. 2017 Dec 29;17(1):236.

34. Moyo S, Ismail F, Van der Walt M, Ismail N, Mkhondo N, Dlamini S, et al. Prevalence of bacteriologically confirmed pulmonary tuberculosis in South Africa, 2017–19: a multistage, cluster-based, cross-sectional survey. Lancet Infect Dis. 2022 Aug;22(8):1172–80.
35. Matji R, Maama L, Roscigno G, Lerotholi M, Agonafir M, Sekibira R, et al. Policy and programmatic directions for the Lesotho tuberculosis programme: Findings of the national tuberculosis prevalence survey, 2019. PLoS One. 2023 Mar 9;18(3):e0273245.
36. Aravkin A, Davis D. Trimmed Statistical Estimation via Variance Reduction. Mathematics of Operations Research. 2020 Feb;45(1):292–322.
37. Wächter A, Biegler LT. On the implementation of an interior-point filter line-search algorithm for large-scale nonlinear programming. Math Program. 2006 Mar 28;106(1):25–57.

High alcohol use

Flowchart

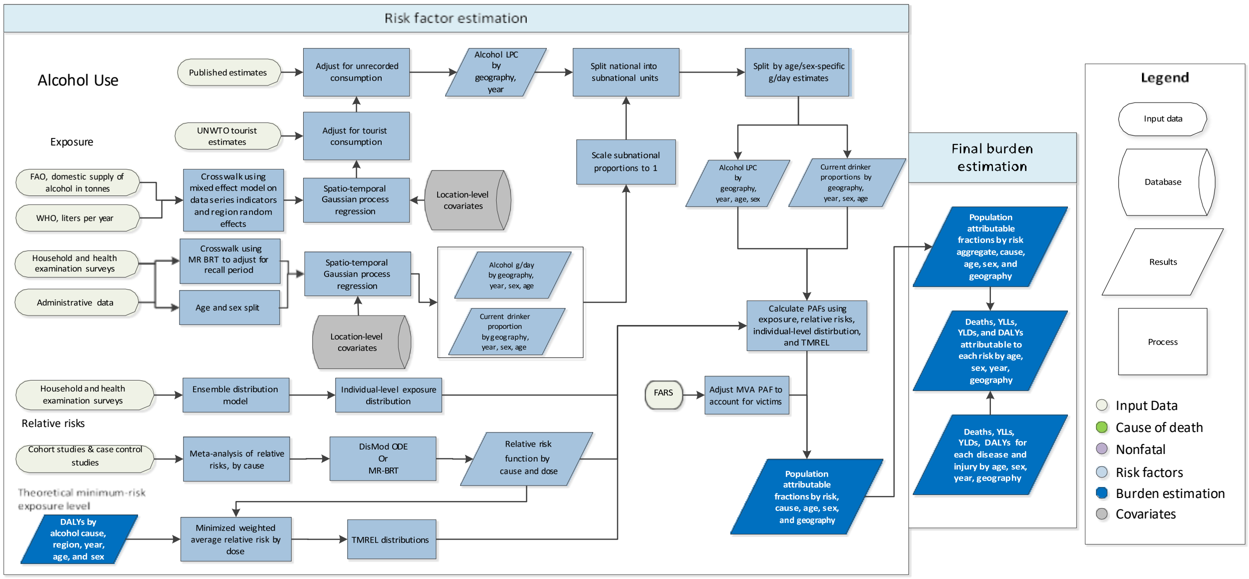

Input data and methodological summary

Definition

Exposure

High alcohol use is defined as alcohol consumption in excess of the theoretical minimum risk exposure level (TMREL), the level of alcohol consumption at which all-cause risk is minimised. Prior to GBD 2021, this risk factor was simply "Alcohol use" and quantified the burden of alcohol consumption over the entire exposure range. More details on the changes to the methodology can be found in the TMREL and "Population attributable fraction" sections of this appendix.

We defined exposure as the grams per day of pure alcohol consumed among current drinkers. We constructed this exposure using the indicators outlined below:

1. Current drinkers, defined as the proportion of individuals who have consumed at least one alcoholic beverage (or some approximation) in a 12-month period.
2. Alcohol consumption (in grams per day), defined as grams of alcohol consumed by current drinkers, per day, over a 12-month period.
3. Alcohol litres per capita (LPC) stock, defined in Liquid-based cytology (LBC) of pure alcohol, over a 12-month period.

We also used three additional indicators to adjust alcohol exposure estimates to account for different types of bias:

1. Number of tourists within a location, defined as the total amount of visitors to a location within a 12-month period.

2. Tourists' duration of stay, defined as the number of days resided in a hosting country.
3. Unrecorded alcohol stock, defined as a percentage of the total alcohol stock produced outside established markets.

Input data

Exposure

A systematic review of the literature was performed to extract data on our primary indicators. The Global Health Exchange (GHDx), IHME's online database of health-related data, was searched for population survey data containing participant-level information from which we could formulate the required alcohol use indicators on current drinkers and alcohol consumption. Data sources were included if they captured a sample representative of the geographical location under study. We documented relevant survey variables from each data source in a spreadsheet and extracted using STATA 13.1 and R 3.3. A total of 6926 potential data sources were available in the GHDx, of which 5764 have been screened and 1206 accepted.

Table 1: Data inputs for exposure for alcohol use.

	Countries with data	New sources	Total sources
Exposure	202	323	10,724

Relative risk

For relative risks, in GBD 2016 we performed a systematic literature review of all cohort and case-control studies reporting a relative risk, hazard ratio, or odds ratio for any risk-outcome pairs studied in GBD 2016. Studies were included if they reported a categorical or continuous dose for alcohol consumption, as well as uncertainty measures for their outcomes, and the population under study was representative.

In GBD 2021, we undertook an effort to update the relative risk curves, beginning with six risk-outcome pairs that were among those associated with the greatest burden: ischaemic heart disease, ischaemic stroke, intracerebral haemorrhage, diabetes mellitus type II, lower respiratory infection, and tuberculosis. We refined the search strings to capture a larger number of studies than was identified by previous search strings. Studies published between 01/01/1970 and 12/31/2019 were reviewed. Of those articles captured, cohort and case-control studies were included if they reported an association between alcohol use and a GBD outcome, a continuous dose for alcohol consumption, and effect size (relative risk, hazard ratio, or odds ratio) with uncertainty. Information on study type, confounders controlled for, sample representativeness, and measurement of exposure and outcomes was also extracted.

Table 2: Data inputs for relative risks for alcohol use

	Countries with data	New sources	Total sources
Relative risks	63	110	566

Data processing

Estimates of current drinking prevalence were split by age and sex where necessary. First, studies that reported prevalence for both sexes were split using a region-specific sex ratio estimated using meta-regression—Bayesian,

regularised, trimmed (MR-BRT). Second, where studies reported estimates across non-GBD age groups, these were split into standard five-year age groups using the global age pattern estimated by spatiotemporal Gaussian process regression (ST-GPR).

Table 3: MR-BRT sex splitting adjustment factors for current drinking

Data input	Gamma	Beta coefficient, log (95% CI)	Adjustment factor*
Female: Male	0	-0.16 (-0.17, -0.14)	0.85
Age <50	0	0.06 (0.06, 0.06)	1.07
East Asia	0.36	-1.02 (-1.74, -0.29)	0.36
Southeast Asia	0.64	-1.06 (-2.34, 0.22)	0.35
Central Asia	0.41	-0.35 (-1.16, 0.46)	0.70
Central Europe	0.18	-0.21 (-0.58, 0.14)	0.80
Eastern Europe	0.10	-0.07 (-0.28, 0.14)	0.93
High-income Asia Pacific	1.27	-1.11 (-4.90, 2.68)	0.33
Western Europe	0.08	0.03 (-0.14, 0.20)	1.03
Southern Latin America	1.26	-0.67 (-4.18, 2.84)	0.51
High-income North America	0.09	-0.07 (-0.26, 0.11)	0.93
Caribbean	0.25	-0.52 (-1.02, -0.03)	0.59
Andean Latin America	0.76	-0.16 (-1.66, 1.34)	0.85
Central Latin America	0.30	-0.52 (-1.12, 0.08)	0.59
Tropical Latin America	0.08	-0.61 (-0.79, -0.44)	0.54
North Africa and Middle East	1.21	-1.44 (-3.91, 1.03)	0.24
South Asia	0.71	-1.17 (-2.57, 0.23)	0.31
Eastern sub-Saharan Africa	0.28	-0.53 (-1.10, 0.03)	0.58
Southern sub-Saharan Africa	0.20	-0.16 (-0.56, 0.23)	0.85
Western sub-Saharan Africa	0.32	-0.19 (-0.83, 0.45)	0.83
Oceania	0.94	-0.54 (-2.42, 1.34)	0.58

**Adjustment factor is the transformed beta coefficient in normal space and can be interpreted as the factor by which the alternative case definition is adjusted to reflect the ratio by which both-sex datapoints were split.*

To allow for the inclusion of data that did not meet our reference definition for current drinking, two crosswalks were performed using MR-BRT. The first crosswalk converted estimates of one-month drinking prevalence to what

they would be if data represented estimates of 12-month drinking prevalence. This crosswalk incorporated two binary covariates: male and age ≥50. The second crosswalk converted estimates of one-week drinking prevalence to 12-month drinking prevalence. This crosswalk incorporated age <20 and male as covariates. The covariates utilised in both crosswalks were included as both x and z covariates. A uniform prior of 0 was set as the upper bound for the beta coefficients to enforce the logical constraint that one-month and one-week prevalence could not be greater than 12-month prevalence.

Table 4: MR-BRT crosswalk adjustment factors for alcohol use current drinking model

Data input	Reference or alternative case definition	Gamma	Beta coefficient, logit (95% UI)*	Adjustment factor**
12-month prevalence	Ref	---	---	---
1-month prevalence	Alt	0.22	-0.60 (-1.05, -0.16)	0.55 (0.35, 0.85)
Age ≥50		0.13	0.16 (-0.10, 0.43)	1.17 (0.9, 1.54)
Male		0.29	0.01 (-0.57, 0.59)	1.01 (0.57, 1.8)
1-week prevalence	Alt	0.46	-1.51 (-2.42, -0.59)	0.22 (0.09, 0.55)
Age <20		0.47	-0.29 (-1.34, 0.76)	0.75 (0.26, 2.14)
Male		0.00	0.38 (0.15, 0.60)	1.46 (1.16, 1.82)

**MR-BRT crosswalk adjustments can be interpreted as the factor the alternative case definition is adjusted by to reflect what it would have been had it been measured using the reference case definition. If the log/logit beta coefficient is negative, then the alternative is adjusted up to the reference. If the log/logit beta coefficient is positive, then the alternative is adjusted down to the reference.*
***The adjustment factor column is the exponentiated beta coefficient. For log beta coefficients, this is the relative rate between the two case definitions. For logit beta coefficients, this is the relative odds between the two case definitions.*

The raw data used in the supply-side model are domestic supply (WHO GISAH; FAO) and retail supply (Euromonitor) of litres of pure ethanol consumed. Domestic supply is calculated as the sum of production and imports, subtracting exports. The WHO and FAO sources were combined, so that FAO data were only used if there were no data available for that location-year from WHO. This was done because the WHO source takes into consideration FAO values when available. Since the WHO data are given in more granular alcohol types, the following adjustments were made:

$$LPC\ Pure\ Ethanol = 0.13 * \left(\frac{Wine}{0.973}\right)$$

$$LPC\ Pure\ Ethanol = 0.05 * \left(\frac{Beer}{0.989}\right)$$

$$LPC\ Pure\ Ethanol = 0.4 * \left(\frac{Spirits}{0.91}\right)$$

Three outliering strategies are used to omit implausible datapoints and data that created implausible model fluctuations. First, estimates from the current drinking model are used to calculate the grams of alcohol consumed per drinker per day. A point is outliered if the grams of pure ethanol per drinker per day for a given source-location-year is greater than 100 (approximately 10 drinks). These thresholds were chosen by using expert knowledge about reasonable consumption levels. In the second round of outliering, the mean LPC value over a 10-year window is calculated. If a point is over 70% of that mean value away from the mean value, it is outliered. The 70% limit was chosen using histograms of these distances. Additionally, some manual outliering is performed to account for edge cases. Finally, data smoothing is performed by taking a three-year rolling mean over each location-year.

Next, an imputation to fill in missing years is performed for all series to remove compositional bias from our final estimates. Since the data from our main sources cover different time periods, by imputing a complete time series for each data series, we reduce the probability that compositional bias of the sources is leading to biased final estimates. To impute the missing years for each series, we model the log ratio of each pair of sources as a function of an intercept and nested random effects on super-region, region, and location. The appropriate predicted ratio is multiplied by the source that we do have, which generates an estimated value for the missing source. For some locations where there was limited overlap between series, the predicted ratio did not make sense, and a regional ratio was used.

Finally, variance was calculated both across series (within a location-year) as well as across years (within a location-source). Additionally, if a location-year had one imputed point, the variance was multiplied by 2. If a location-year had two imputed points, the variance was multiplied by 4. The average estimates in each location-year were the input to an ST-GPR model. This uses a mixed-effects model modelled in log space with nested location random effects.

We obtained data on the number of tourists and their duration of stay from the UN World Tourism Organization.[3] We applied a crosswalk across different tourist categories, similar to the one used for the LPC data, to arrive at a consistent definition (ie, visitors to a country). We obtained estimates on unrecorded alcohol stock from data available in WHO GISAH database,[2] consisting of 189 locations. For locations with no data available, the national or regional average was used.

Modelling strategy

Exposure

While population-based surveys provide accurate estimates of the prevalence of current drinkers, they typically underestimate real alcohol consumption levels.[10-12] As a result, we considered the LPC input to be a better estimate of overall volume of consumption. Per capita consumption, however, does not provide age- and sex-specific consumption estimates needed to compute alcohol-attributable burden of disease. Therefore, we use the age-sex pattern of consumption among drinkers modelled from the population survey data and the overall volume of consumption from FAO, GISAH, and Euromonitor to determine the total amount of alcohol consumed within a location. In the paragraphs that follow, we outline how we estimated each primary input in the alcohol exposure model, as well as how we combined these inputs to arrive at our final estimate of grams per day of pure alcohol. We estimated all models below using 1000 draws.

For data obtained through surveys, we used spatiotemporal Gaussian process regression (ST-GPR) to construct

estimates for each location/year/age/sex. We chose to use ST-GPR due to its ability to leverage information across the nearby locations or time periods. We also modelled the alcohol LPC data, as well as the total number of tourists, using ST-GPR. To improve the LPC model fit in years beyond those in which data was available, we forecasted ST-GPR estimates using a damped holt function.

Given the heterogeneous nature of the estimates on unrecorded consumption, as well as the wide variation across countries and time periods, we took 1000 draws from the uniform distribution of the lowest and highest estimates available for a given country. We did this to incorporate the diffuse uncertainty within the unrecorded estimates reported. We used these 1000 draws in the equation below.

We adjusted the alcohol LPC for unrecorded consumption using the following equation:

$$Alcohol\ LPC = \frac{Alcohol\ LPC}{(1 - \%\ Unrecorded)}$$

We then adjusted the estimates for alcohol LPC for tourist consumption by adding in the per capita rate of consumption abroad and subtracting the per capita rate of tourist consumption domestically.

$$Alcohol\ LPC_d = Unadjusted\ Alcohol\ LPC_d + Alcohol\ LPC_{Domestic\ consumption\ abroad} - Alcohol\ LPC_{Tourist\ consumption\ domestically}$$

$$Alcohol\ LPC_i = \frac{\sum_l Tourist\ Population_l * Proportion\ of\ tourists_{i,l} * Unadjusted\ Alcohol\ LPC_l * \frac{Average\ length\ of\ stay_{i,l}}{365} *}{Population_d}$$

where:

l is the set of all locations, i is either Domestic consumption abroad *or* Tourist consumption domestically, *and d is a domestic location.*

After adjusting alcohol LPC by tourist consumption and unrecorded consumption for all location/years reported, sex-specific and age-specific estimates were generated by incorporating estimates modelled in ST-GPR for percentage of current drinkers within a location/year/sex/age, as well as consumption trends modelled in the ST-GPR grams per day model. We do this by first calculating the proportion of total consumption for a given location/year by age and sex, using the estimates of alcohol consumed per day, the population size, and the percentage of current drinkers. We then multiply this proportion of total stock for a given location/year/sex/age by the total stock for a given location/year to calculate the consumption in terms of LPC for a given location/year/sex/age. We then convert these estimates to be in terms of grams/per day. The following equations describe these calculations:

[illegible]

where:

$$l\ is\ a\ location, y\ is\ a\ year, s\ is\ a\ sex, and\ a\ is\ an\ age\ group$$

We then used the gamma distribution to estimate individual-level variation within location, year, sex, age drinking populations, following the recommendations of other published alcohol studies.[7,8] We chose parameters of the gamma distribution based on the mean and standard deviation of the 1000 draws of alcohol g/day exposure for a given population. Standard deviation was calculated using the following formula.[15] We tested several alternative models using our data and found this model performed best.

$$standard\ deviation = mean * (0.087 * female + 1.171)$$

Theoretical minimum-risk exposure level

The methods for calculating the TMREL were updated for GBD 2021. Previously, one global estimate of the TMREL was calculated. However, the contributions of each cause to overall health loss vary over geography, age, time, and sex, suggesting that the amount of alcohol that minimises health loss similarly varies over these domains. For this reason, in GBD 2021 we estimated an individual TMREL for each region, age, sex, and year.

For each region, age, sex, and year, we calculated TMREL by first calculating the overall risk attributable to alcohol. We did this by weighting each relative risk curve by the share of overall DALYs for a given cause. We then took the minimum of this overall-risk curve as the TMREL of alcohol use. More formally,

$$TMREL = argmin\ average\ overall\ risk_{\omega}(g/day)$$

$$Average\ overall\ risk_{\omega,l,y,a,s}(g/day) = \sum_{i}^{\omega} \log(RR_i(g/day)) * \frac{DALY_{i,l,y,a,s}}{\sum_i^{\omega} DALY_{i,l,y,a,s}}$$

Where:

ω *is the set of causes associated with alcohol,* i *is a given cause from that set,*
l *is a location,* y *is a year,* s *is a sex,* a *is an age group,* $DALY$ *is the DALY rate,*
and RR *is the dose response curve for a given cause and exposure level in grams per day.*

In other words, we chose TMREL as being the exposure that minimises the risk of suffering burden from any given cause related to alcohol. We weight the risk for a particular cause in our aggregation by the proportion of DALYs due to that cause (eg, since more observed people die from ischaemic heart disease, we weight the risk for ischaemic heart disease more in the above calculation of average risk compared to, say, diabetes, even if both have the same relative risk for a given level of consumption).

Figure 1: TMREL by region, age, and sex, 2020

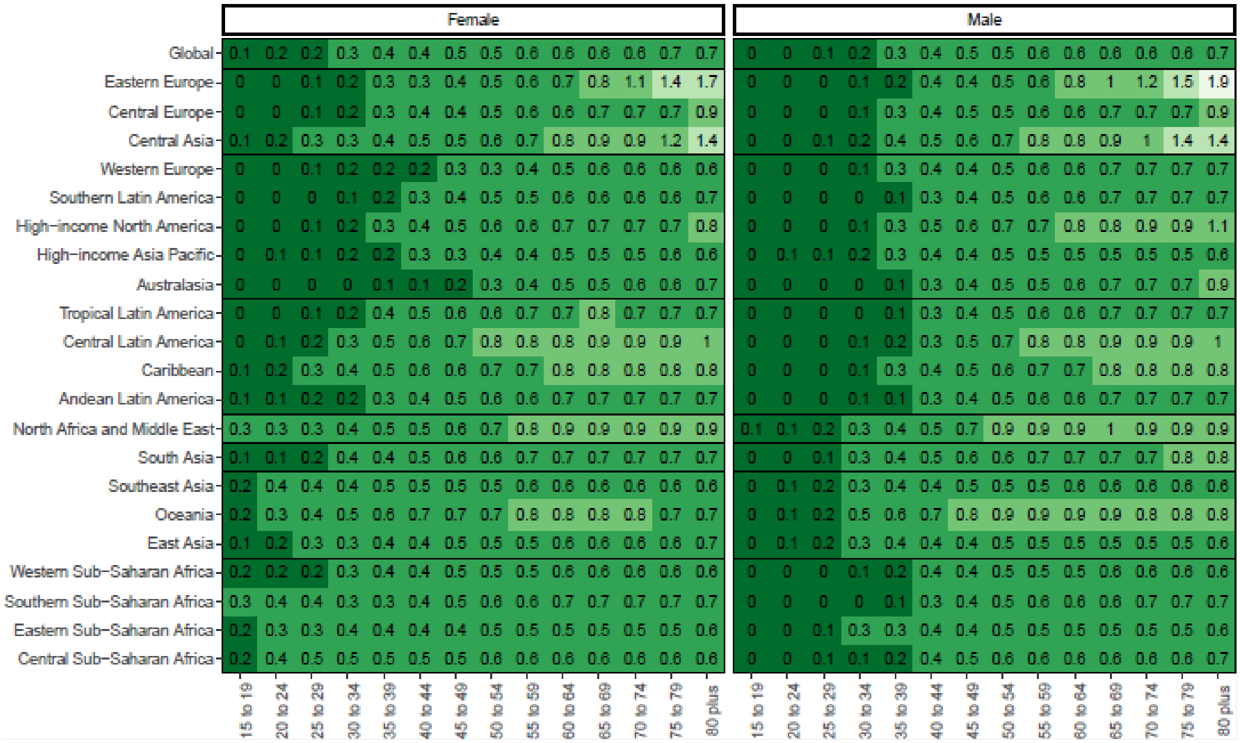

Relative risks

For GBD 2016 through 2019, we used the studies identified through a systematic review to calculate a dose–response, modelled using DisMod ODE. We chose DisMod ODE rather than a conventional mixed-effects meta-regression because of its ability to estimate non-parametric splines over doses (ie, for most alcohol causes, there is a non-linear relationship with different doses) and incorporate heterogeneous doses through dose-integration (ie, most studies report doses categorically in wide ranges. DisMod ODE estimates specific doses when categories overlap across studies, through an integration step.). We used the results of the meta-regression to estimate a non-parametric curve for all doses between zero and 100 g/day and their corresponding relative risks. For all causes, we assumed the relative risk was the same for all ages and sexes.

For GBD 2021, we used the studies identified through the updated systematic review to estimate new dose–response curves using MR-BRT for six outcomes among those associated with the greatest burden: ischaemic heart disease, ischaemic stroke, intracerebral haemorrhage, diabetes mellitus type II, lower respiratory infection, and tuberculosis. The relative risk curves for the remaining outcomes will be modelled using MR-BRT instead of DisMod ODE in the coming GBD rounds. Importantly, this new method takes into account the risk of biases in the relative risk estimation and incorporates unexplained between-study heterogeneity into the uncertainty of the relative risk estimates. The results of the meta-regression were used to estimate a non-parametric curve for all

doses between zero and 100 g/day and their corresponding relative risks.

We implemented the Fisher Scoring correction to the heterogeneity parameter, which corrects for data-sparse situations. In such cases, the between-study heterogeneity parameter estimate may be 0, simply from lack of data. The Fisher Scoring correction uses a quantile of gamma, which is sensitive to the number of studies, study design, and reported uncertainty.

We have also added methodology that can detect and flag publication bias. The approach is based on the classic Egger's Regression strategy, which is applied to the residuals in our model. In the current implementation, we do not correct for publication bias, but flag the risk-outcome pairs where the risk for publication bias is significant. In the table below, we list each risk-outcome pair that is updated in GBD 2021 along with several of the key modelling parameters and results. The formulation for MR-BRT is described in detail in the MR-BRT section of the appendix.

Table 5: MR-BRT splines and priors by type of risk

Risk-outcome	Type of risk	Spline degree, # interior knots	Priors and constraints
Ischaemic heart disease	J-shaped	Quadratic, 2 I knots	No monotonicity constraint
Ischaemic stroke	J-shaped	Quadratic, 3 I knots	No monotonicity constraint, right linear tail
Intracerebral haemorrhage	J-shaped	Cubic, 3 I knots	No monotonicity constraint, right linear tail
Type II diabetes mellitus	J-shaped	Cubic, 3 I knots	No monotonicity constraint, right linear tail
Tuberculosis	Harmful	Quadratic, 3 I knots	Monotonic increasing, right linear tail, Gaussian max derivative prior on the right tail (0, 0.001)
Lower respiratory infection	Harmful	Quadratic, 3 I knots	Monotonic increasing, right linear tail, Gaussian max derivative prior on the right tail (0, 0.001)

Table 6: MR-BRT parameters by risk-outcome pair

Risk-outcome	Type of risk	Selected covariates	Mean gamma solution	Publication bias result
Ischaemic heart disease	J-shaped	cv_incidence	0.158	No publication bias
Ischaemic stroke	J-shaped	cv_incidence	0.234	No publication bias
Intracerebral haemorrhage	J-shaped	cv_adjusted_2, cv_adjusted_1	0.09	No publication bias
Type II diabetes mellitus	J-shaped	None	0.117	No publication bias
Tuberculosis	Harmful	cv_sick_quitters, cv_incidence	19.488	No publication bias
Lower respiratory infection	Harmful	None	0	No publication bias

After evaluating all available evidence, we found insufficient evidence for a relationship between alcohol use and lower respiratory infection. Specifically, a simplified log-linear model was run, including only exposed and reference group dose data and study id as covariates, and a one-sided z-test was performed for the fixed-effects only model at alpha value set to 0.1. Based on this test, we removed alcohol use vs. lower respiratory infection as a risk-outcome pair for GBD 2021.

Population attributable fraction

We calculated population attributable fractions (PAFs) by setting the relative risk of alcohol consumption among abstainers and drinkers consuming alcohol below the TMREL to be 1. We then calculated PAFs for drinkers consuming alcohol in excess of the TMREL as we have previously. For each location, age, sex, year, and cause, we defined PAF as:

$$PAF(x) = \frac{P_A + \int_0^{TMREL} P(x)\ dx + \int_{TMREL}^{100} P(x) * RR_C(x)\ dx - RR_C(TMREL)}{P_A + \int_0^{TMREL} P(x)\ dx + \int_{TMREL}^{100} P(x) * RR_C(x)\ dx} \qquad P(x) = P_C * \Gamma(\boldsymbol{p})$$

where :

P_C *is the prevalence of current drinkers,* P_A *is the prevalence of abstainers, and*

$\boldsymbol{p}$ *are parameters determined by the mean and sd of exposure for that location, age, sex, and year;*

$RR_c(x)$ *is the global relative risk function for current drinkers for a given cause, and*

$TMREL$ *is the theoretical minimum risk exposure level for that location's region, age, sex, and year*

We performed the above equation for 1000 draws of the exposure and relative risk models. We then used the estimated PAF draws to calculate YLL, YLDs, and DALYs, as per the other risk factors.

For outcomes that are by definition caused by alcohol, such as liver cancer or cirrhosis due to alcohol use, PAFs are set to 1. PAFs for cirrhosis due to all causes that are in excess of the proportion of all cirrhosis burden due to alcohol are proportionally redistributed over cirrhosis due to hepatitis B, hepatitis C, and other causes. Similarly, PAFs for liver cancer due to all causes that are in excess of the proportion of all liver cancer burden due to alcohol are proportionally redistributed over liver cancer due to hepatitis B, hepatitis C, and other causes.

References

1. Food and Agriculture Organization of the United Nations (FAO). FAOSTAT Food Balance Sheets, October 2014. Rome, Italy: Food and Agriculture Organization of the United Nations (FAO).
2. World Health Organization (WHO). WHO Global Health Observatory - Recorded adult per capita alcohol consumption, Total per country. Geneva, Switzerland: World Health Organization (WHO).
3. UN World Tourism Organization (UNWTO). UN World Tourism Organization Compendium of Tourism Statistics 2015 [Electronic]. Madrid, Spain: UN World Tourism Organization (UNWTO), 2016.
4. Ramstedt, Mats. "How much alcohol do you buy? A comparison of self-reported alcohol purchases with actual sales." Addiction 105.4 (2010): 649-654.
5. Stockwell, Tim, et al. "Under-reporting of alcohol consumption in household surveys: a comparison of quantity–frequency, graduated–frequency and recent recall." Addiction 99.8 (2004): 1024-1033.
6. Kerr, William C., and Thomas K. Greenfield. "Distribution of alcohol consumption and expenditures and the impact of improved measurement on coverage of alcohol sales in the 2000 National Alcohol Survey." Alcoholism: Clinical and Experimental Research 31.10 (2007): 1714-1722.
7. Taylor, Bruce, et al. "The more you drink, the harder you fall: a systematic review and meta-analysis of how acute alcohol consumption and injury or collision risk increase together." Drug and alcohol dependence 110.1 (2010): 108-116.
8. Vinson, Daniel C., Guilherme Borges, and Cheryl J. Cherpitel. "The risk of intentional injury with acute and chronic alcohol exposures: a case-control and case-crossover study." Journal of studies on alcohol 64.3 (2003): 350-357.
9. Vinson, Daniel C., et al. "A population-based case-crossover and case-control study of alcohol and the risk of injury." Journal of studies on alcohol64.3 (2003): 358-366.
10. Fatal Accident Reporting System (FARS). National Highway Traffic Safety Administration, National Center for Statistics and Analysis Data Reporting and Information Division (NVS-424); 1985, 1990, 1995, 2000, 2005, 2010, 2015
11. Chen, Li-Hui, Susan P. Baker, and Guohua Li. "Drinking history and risk of fatal injury: comparison among specific injury causes." Accident Analysis & Prevention 37.2 (2005): 245-251.
12. Bell, Nicole S., et al. "Self-reported risk-taking behaviors and hospitalization for motor vehicle injury among active duty army personnel." American journal of preventive medicine 18.3 (2000): 85-95.
13. Margolis, Karen L., et al. "Risk factors for motor vehicle crashes in older women." The Journals of Gerontology Series A: Biological Sciences and Medical Sciences 57.3 (2002): M186-M191.
14. Sorock, Gary S., et al. "Alcohol-drinking history and fatal injury in older adults." Alcohol 40.3 (2006): 193-199.
15. Kehoe, Tara et al. "Determining the best population-level alcohol consumption model and its impact on estimates of alcohol-attributable harms." Population health metrics 10 6. (2012)

Smoking

Flowchart

Smoking

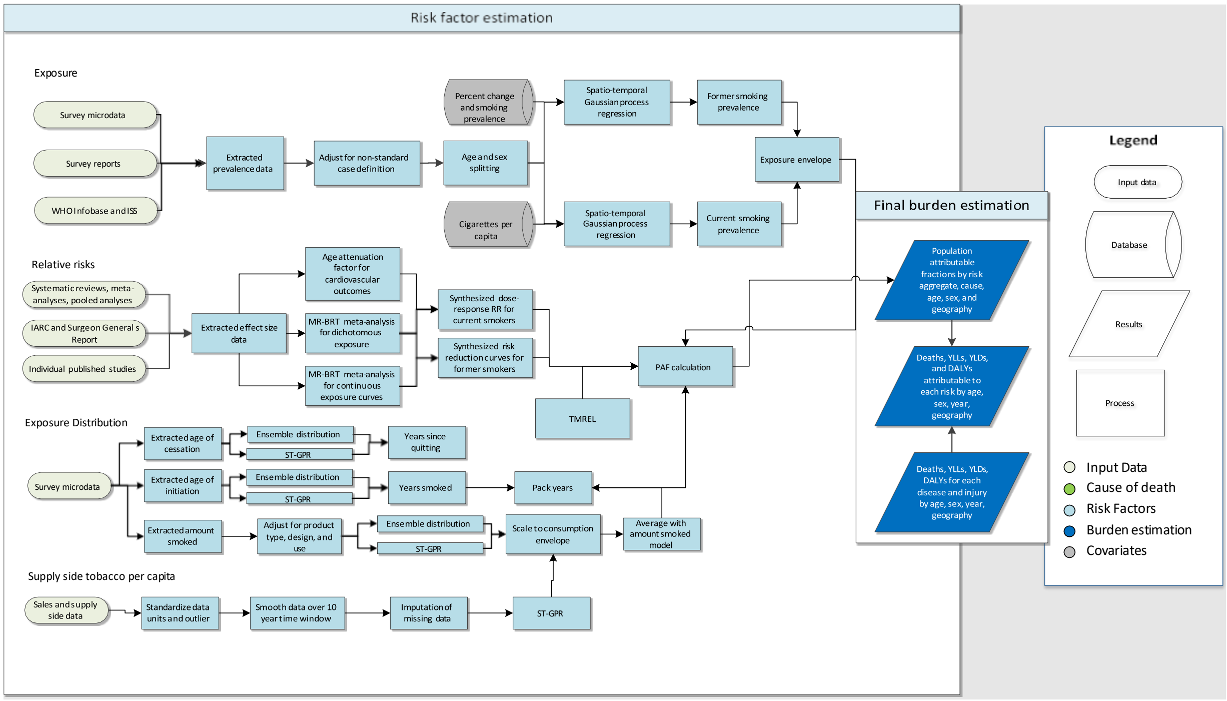

Input data and methodological summary

Definition

Exposure

As in previous GBD cycles, we estimated the prevalence of current smoking and the prevalence of former smoking using data from cross-sectional nationally representative household surveys. We defined current smokers as individuals who currently use any smoked tobacco product on a daily or occasional basis. We defined former smokers as individuals who quit using all smoked tobacco products for at least six months, where possible, or according to the definition used by the given survey.

Input data

Exposure

Our survey data extraction method for smoking exposure has not changed from previous GBD cycles. A systematic review of literature was performed to extract data on our primary exposure indicators. We searched the Global Health Data Exchange (GHDx), a comprehensive online catalog of health-related data created by IHME, for population survey data. We also included surveys that were recommended by our collaborators but were not in the GHDx. Regarding inclusion and exclusion criteria, we only included surveys that are nationally or subnationally representative (on state/province level). Surveys conducted among specific populations (eg, pregnant women, physicians) were excluded.

We extracted primary data from individual-level microdata and survey report tabulations. Specifically, we extracted data on current, former, and/or ever smoked tobacco use reported as any combination of frequency of use (daily, occasional, and unspecified, which includes both daily and occasional smokers) and type of smoked tobacco used (all smoked tobacco, cigarettes, hookah, and other smoked tobacco products such as cigars or pipes), resulting in 36 possible combinations. Other variants of tobacco products, for example hand-rolled cigarettes, were grouped into the four type categories listed above based on product similarities.

For microdata, we extracted relevant demographic information, including age, sex, location, and year, as well as survey metadata, including survey weights, primary sampling units, and strata. This information allowed us to tabulate individual-level data in the standard GBD five-year age-sex groups and produce accurate estimates of uncertainty. For survey report tabulations, we extracted data at the most granular age-sex group provided. After data were extracted, we carefully vetted the extracted data, fixed any extraction error and cautiously outliered problematic data due to quality concerns based on expert opinion. We documented relevant survey variables from each data source as well as outliered data in spreadsheets. We extracted data using STATA 13.1 and R 3.3.

Table 1: Data inputs for exposure for smoked tobacco

	**Countries with data**	**New sources**	**Total sources**
Exposure	201	164	3603

Relative risk

Since GBD 2016 we had performed systematic review and meta-analysis of all case-control and prospective cohort studies reporting a relative risk, hazard ratio, or odds ratio for any risk-outcome pair studied in GBD 2016. In GBD 2019, we had included 36 risk-outcome pairs for smoking. Studies were included if they reported a categorical or continuous dose for smoked tobacco consumption (pack-years or cigarettes per day) as well as uncertainty measures of the estimated risk, and the population under study was general population. Studies were excluded if they used cross-sectional or retrospective cohort design or if the study was conducted among specific populations (eg, people with diabetes or drug users, etc.).

In GBD 2021, we undertook an effort to improve our relative risk curves by refining our search strings to capture a larger number of studies than was identified by previous search strings. Studies published between 01/01/1970 and 05/31/2022 were reviewed. Of those articles captured, prospective cohort and case-control studies were included if they reported the effect sizes (relative risk, hazard ratio, or odds ratio) of an association between a continuous or categorical dose for smoked tobacco consumption and a GBD outcome with uncertainty. Information on study design, confounders controlled for, sample representativeness, and measurement of exposure and outcomes was also extracted.

Data processing

Crosswalk

Our GBD smoking case definitions were current smoking of any tobacco product and former smoking of any tobacco product. All other definitions were adjusted to be consistent with either of these definitions. Some sources contained information on more than one case definition, and these sources were used to develop the

adjustment coefficients to transform alternative case definitions to the GBD case definition. The adjustment coefficients were the beta values derived from linear regression models with one predictor and no intercept. We used the same crosswalk adjustment coefficients as in GBD 2019, and thus we have not included a methods explanation in this appendix, as it has been detailed previously.

Age and sex splitting

As in GBD 2019, we split data reported in broader age groups than the GBD five-year age groups or as both sexes combined by adapting the method reported in Ng et al1 to split using a sex-geography-time-specific reference age pattern. We separated the data into two sets: a training dataset, with data already falling into GBD sex-specific five-year age groups, and a split dataset, which reported data in aggregated age or sex groups. We then used spatiotemporal Gaussian process regression (ST-GPR) to estimate sex-geography-time-specific age patterns using data in the training dataset. The estimated age patterns were used to split each source in the split dataset.

The ST-GPR model used to estimate the age patterns for age-sex splitting used an age weight parameter value that minimises the effect of any age smoothing. This parameter choice allowed the estimated age pattern to be driven by data, rather than being enforced by any smoothing parameters of the model. These age-sex-split datapoints were to be incorporated in the final ST-GPR exposure model; thus, we did not want to doubly enforce a modelled age pattern for a given sex-location-year on a given aggregate datapoint.

Modelling strategy

Smoking prevalence modelling

We used ST-GPR to model current and former smoking prevalence. The model is identical to that in GBD 2019. Briefly, the mean function input to GPR is a complete time series of estimates generated from a mixed effects hierarchical linear model plus weighted residuals smoothed across time, space, and age. The linear model formula for current smoking, fit separately by sex using restricted maximum likelihood in R, is:

$$logit(p_{g,a,t}) = \beta_0 + \beta_1 CPC_{g,t} + \sum_{k=2}^{19} \beta_k I_{A[a]} + \alpha_s + \alpha_r + \alpha_g + \epsilon_{g,a,t}$$

Where $CPC_{g,t}$ is the tobacco consumption per capita covariate by geography g and time t, described above, $I_{A[a]}$ is a dummy variable indicating specific age group that the prevalence point $p_{g,a,t}$ captures, and α_s, α_r, and α_g are super-region, region, and geography random intercepts, respectively. $\epsilon_{g,a,t}$ represents the random error between the predicted log odds and the true log odds being modeled in the logistic regression Random effects were used in model fitting but not in prediction.

The linear model formula for former smoking is:

$$logit(p_{g,a,t}) = \beta_0 + \beta_1 PctChange_{A[a],g,t} + \beta_3 CSP_{A[a],g,t} + \sum_{k=3}^{20} \beta_k I_{A[a]} + \alpha_s + \alpha_r + \alpha_g + \epsilon_{g,a,t}$$

Where $PctChange_{A[a],g,t}$ is the percentage change in current smoking prevalence from the previous year, and $CSP_{A[a],g,t}$ is the current smoking prevalence by specific age group A, geography g, and time t that point $p_{g,a,t}$ captures, both derived from the current smoking ST-GPR model defined above.

Supply-side estimation

The methods for modelling supply-side-level data were consistent with those used in GBD 2019. The raw data were domestic supply (USDA Global Surveillance Database and UN FAO) and retail supply (Euromonitor) of tobacco. Domestic supply was calculated as production + imports – exports. The data went through three rounds of outliering based on reasonable consumption thresholds of number of cigarettes per smoker per day, distance from the ten-year rolling mean tobacco per capita, and manual outliering for edge cases. Finally, data smoothing was performed by taking a three-year rolling mean over each location-year.

Next, to impute the missing years for each series and remove compositional bias from our final estimates, we modelled the log ratio of each pair of sources as a function of an intercept and nested random effects on super-region, region, and location. The appropriate predicted ratio was multiplied by each source that we did have, and then the predictions were averaged to get the final imputed value. For some locations where there was limited overlap between series, the predicted ratio did not make sense, and a regional ratio was used.

Finally, variance was calculated both across series (within a location-year) as well as across years (within a location-source). Additionally, if a location-year had one imputed point, the variance was multiplied by 2. If a location-year had two imputed points, the variance was multiplied by 4. The average estimates in each location-year were the input to an ST-GPR model. For this, we used a simple mixed effects model, which was modelled in log space with nested location random effects. Subnational estimates were then further modelled by splitting the country-level estimates using current smoking prevalence.

Theoretical minimum-risk exposure level

The theoretical minimum-risk exposure level is 0.

Exposure among current and former smokers

Identical to GBD 2019, we estimated exposure among current smokers for two continuous indicators: cigarettes per smoker per day and pack-years. Pack-years incorporates aspects of both duration and amount. One pack-year represents the equivalent of smoking one pack of cigarettes (assuming a 20-cigarette pack) per day for one year. Since the pack-years indicator collapses duration and intensity into a single dimension, one pack-year of exposure can reflect smoking 40 cigarettes per day for six months or smoking 10 cigarettes per day for two years.

To produce these indicators, we simulated individual smoking histories based on distributions of age of initiation and amount smoked. We informed the simulation with cross-sectional survey data capturing these indicators, modelled at the mean level for all locations, years, ages, and sexes using ST-GPR. We rescaled estimates of cigarettes per smoker per day to an envelope of cigarette consumption based on supply-side data. We estimated pack-years of exposure by summing samples from age- and time-specific distributions of cigarettes per smoker for a birth cohort to capture both age trends and time trends and avoid the common assumption that the amount someone currently smokes is the amount they have smoked since they began smoking. All distributions were age-, sex-, and region-specific ensemble distributions, which were found to outperform any single distribution.

We estimated exposure among former smokers using years since cessation. We used ST-GPR to model mean age of cessation using cross-sectional survey data capturing age of cessation. Using these estimates, we generated ensemble distributions of years since cessation for every location, year, age group, and sex.

Relative risk

The same risk-outcome pairs from GBD 2019 were used for GBD 2021: tuberculosis, lower respiratory tract infections, oesophageal cancer, stomach cancer, bladder cancer, liver cancer, laryngeal cancer, lung cancer, breast cancer, cervical cancer, colorectal cancer, lip and oral cancer, nasopharyngeal cancer, other pharyngeal cancer, pancreatic cancer, kidney cancer, leukaemia, ischaemic heart disease, ischaemic stroke, haemorrhagic stroke, subarachnoid haemorrhage, atrial fibrillation and flutter, aortic aneurysm, peripheral arterial disease, chronic obstructive pulmonary disease, other chronic respiratory diseases, asthma, peptic ulcer disease, gallbladder and biliary tract diseases, Alzheimer's disease and other dementias, Parkinson's disease (protective), multiple sclerosis, type 2 diabetes, rheumatoid arthritis, low back pain, cataracts, macular degeneration, and fracture.

For GBD 2021, the risk of all risk-outcome pairs is evaluated by continuous smoking exposure level (ie, pack-year, cigarettes per smoker per day, and years since cessation), expect for fracture, whose risk is evaluated by binary smoking exposure (ie, smoker versus non-smoker/former smoker).

Dose-response risk curves

Since GBD 2016, we had used the studies identified through the systematic review to estimate dose–response risk of smoking on related health outcomes using DisMod ODE. We chose DisMod ODE rather than a conventional mixed effects meta-regression because of its ability to estimate nonparametric splines over doses (ie, there is usually a non-linear relationship between smoking exposure level and outcome risk) and incorporate heterogeneous doses through dose integration (ie, most studies report smoking exposure level categorically in wide ranges, and DisMod ODE can estimate risk of specific exposure level when categories overlap across studies, through an integration step).

For GBD 2021, we used the studies identified through the updated systematic review to estimate new dose–response curves using MR-BRT for all outcomes. Importantly, this new method takes into account the risk of biases in the RR estimation by selecting and including important covariates of the risk estimates in the model (eg, measurement of exposure and outcomes, representativeness, and adjustment level of the risk estimates) andincorporates unexplained between-study heterogeneity into the uncertainty of the RR estimates. The results of the meta-regression were used to estimate a non-parametric curve for all doses between zero and 100 pack-years or cigarettes per smoker per day and their corresponding relative risks. For all outcomes, we assumed the relative risk was the same for both sexes, expect for breast cancer, cervical cancer, and prostate cancer, which were assumed to apply only to female or to male.

For data-sparse risk–outcome pairs, we implemented the Fisher scoring correction to the heterogeneity parameter. When data are sparse, the between-study heterogeneity parameter estimate may be 0, simply due to lack of data. The Fisher scoring correction uses a quantile of gamma, which is sensitive to the number of studies, study design, and reported uncertainty.

We have also added methodology that can detect and flag publication bias. The approach is based on the classic Egger's regression strategy, which is applied to the residuals in our model. In the current implementation, we do not correct for publication bias, but flag the risk–outcome pairs where the risk for publication bias is significant.

For risk of former smokers, we estimated risk curves of former smokers compared to never smokers taking into account the rate of risk reduction among former smokers seen in the cohort and case-control studies, and the cumulative exposure among former smokers within each age, sex, location, and year group. For GBD 2021, we did

not include new data or change the method of estimating the risk curves of former smokers.

In the table below, we list each risk–outcome pair that is updated in GBD 2021 along with several of the key modelling parameters and results. The formulation for MR-BRT is described in detail in section XX of the appendix.

Table 5: MR-BRT model specifications by risk–outcome pair

Risk-outcome	Type of risk	Spline degree, # interior knots	Priors & constraints
Atrial fibrillation and flutter	Continuous, harmful	Quadratic, 3 I knots	Monotonic increasing, right linear tail, Gaussian max derivative prior on the right tail (0, 0.001)
Alzheimer's and other dementias	Continuous, harmful	Quadratic, 3 I knots	Monotonic increasing, right linear tail, Gaussian max derivative prior on the right tail (0, 0.001)
Aortic aneurism	Continuous, harmful	Quadratic, 3 I knots	Monotonic increasing, right linear tail, Gaussian max derivative prior on the right tail (0, 0.001)
Asthma	Continuous, harmful	Quadratic, 3 I knots	Monotonic increasing, right linear tail, Gaussian max derivative prior on the right tail (0, 0.001)
Bladder cancer	Continuous, harmful	Quadratic, 3 I knots	Monotonic increasing, right linear tail, Gaussian max derivative prior on the right tail (0, 0.001)
Breast cancer	Continuous, harmful	Quadratic, 3 I knots	Monotonic increasing, right linear tail, Gaussian max derivative prior on the right tail (0, 0.001)
Cataracts	Continuous, harmful	Quadratic, 3 I knots	Monotonic increasing, right linear tail, Gaussian max derivative prior on the right tail (0, 0.001)
Cervical cancer	Continuous, harmful	Quadratic, 3 I knots	Monotonic increasing, right linear tail, Gaussian max derivative prior on the right tail (0, 0.001)
Colon and rectum cancer	Continuous, harmful	Quadratic, 3 I knots	Monotonic increasing, right linear tail, Gaussian max derivative prior on the right tail (0, 0.001)
COPD	Continuous, harmful	Quadratic, 3 I knots	Monotonic increasing, right linear tail, Gaussian max derivative prior on the right tail (0, 0.001)

Diabetes	Continuous, harmful	Quadratic, 3 l knots	Monotonic increasing, right linear tail, Gaussian max derivative prior on the right tail (0, 0.001)
Oesophageal cancer	Continuous, harmful	Quadratic, 3 l knots	Monotonic increasing, right linear tail, Gaussian max derivative prior on the right tail (0, 0.001)
Gallbladder diseases	Continuous, harmful	Quadratic, 3 l knots	Monotonic increasing, right linear tail, Gaussian max derivative prior on the right tail (0, 0.001)
Fracture (hip and non-hip)	Dichotomous, harmful	N/A	N/A
Ischaemic heart disease	Continuous, harmful	Quadratic, 3 l knots	Monotonic increasing, right linear tail, Gaussian max derivative prior on the right tail (0, 0.001)
Kidney cancer	Continuous, harmful	Quadratic, 3 l knots	Monotonic increasing, right linear tail, Gaussian max derivative prior on the right tail (0, 0.001)
Laryngeal cancer	Continuous, harmful	Quadratic, 3 l knots	Monotonic increasing, right linear tail, Gaussian max derivative prior on the right tail (0, 0.001)
Lower back pain	Continuous, harmful	Quadratic, 3 l knots	Monotonic increasing, right linear tail, Gaussian max derivative prior on the right tail (0, 0.001)
Leukaemia	Continuous, harmful	Quadratic, 3 l knots	Monotonic increasing, right linear tail, Gaussian max derivative prior on the right tail (0, 0.001)
Lip and oral cavity cancer	Continuous, harmful	Quadratic, 3 l knots	Monotonic increasing, right linear tail, Gaussian max derivative prior on the right tail (0, 0.001)
Liver cancer	Continuous, harmful	Quadratic, 3 l knots	Monotonic increasing, right linear tail, Gaussian max derivative prior on the right tail (0, 0.001)
Lower respiratory infections	Continuous, harmful	Quadratic, 3 l knots	Monotonic increasing, right linear tail, Gaussian max derivative prior on the right tail (0, 0.001)
Lung cancer	Continuous, harmful	Quadratic, 3 l knots	Monotonic increasing, right linear tail, Gaussian max derivative prior on the right tail (0, 0.001)

Macular degeneration	Continuous, harmful	Quadratic, 3 I knots	Monotonic increasing, right linear tail, Gaussian max derivative prior on the right tail (0, 0.001)
Multiple sclerosis	Continuous, harmful	Quadratic, 3 I knots	Monotonic increasing, right linear tail, Gaussian max derivative prior on the right tail (0, 0.001)
Nasopharyngeal cancer	Continuous, harmful	Quadratic, 3 I knots	Monotonic increasing, right linear tail, Gaussian max derivative prior on the right tail (0, 0.001)
Other pharynx cancer	Continuous, harmful	Quadratic, 3 I knots	Monotonic increasing, right linear tail, Gaussian max derivative prior on the right tail (0, 0.001)
Pancreatic cancer	Continuous, Harmful	Quadratic, 3 I knots	Monotonic increasing, right linear tail, Gaussian max derivative prior on the right tail (0, 0.001)
Parkinson's disease	Continuous, protective	Quadratic, 3 I knots	Monotonic decreasing, right linear tail, Gaussian max derivative prior on the right tail (0, 0.001)
Peptic ulcer	Continuous, harmful	Quadratic, 3 I knots	Monotonic increasing, right linear tail, Gaussian max derivative prior on the right tail (0, 0.001)
Peripheral artery disease	Continuous, harmful	Quadratic, 3 I knots	Monotonic increasing, right linear tail, Gaussian max derivative prior on the right tail (0, 0.001)
Prostate cancer	Continuous, harmful	Quadratic, 3 I knots	Monotonic increasing, right linear tail, Gaussian max derivative prior on the right tail (0, 0.001)
Rheumatoid arthritis	Continuous, harmful	Quadratic, 3 I knots	Monotonic increasing, right linear tail, Gaussian max derivative prior on the right tail (0, 0.001)
Stomach cancer	Continuous, harmful	Quadratic, 3 I knots	Monotonic increasing, right linear tail, Gaussian max derivative prior on the right tail (0, 0.001)
Stroke (ischaemic stroke, haemorrhagic stroke, and subarachnoid haemorrhage)	Continuous, harmful	Quadratic, 3 I knots	Monotonic increasing, right linear tail, Gaussian max derivative prior on the right tail (0, 0.001)
Tuberculosis	Continuous, harmful	Quadratic, 3 I knots	Monotonic increasing, right linear tail, Gaussian max derivative prior on the right tail (0, 0.001)

Table 6: MR-BRT estimated parameters and bias covariates by risk–outcome pair

Risk–outcome	Unit of risk	Selected bias covariates	Mean gamma solution	publication bias
Atrial fibrillation and flutter	cigarettes per day	None	0.000	0
Alzheimer's and other dementias	cigarettes per day	None	0.000	1
Aortic aneurism	cigarettes per day	None	0.000	0
Asthma	cigarettes per day	None	1.651	0
Bladder cancer	pack-year	None	0.000	0
Breast cancer	pack-year	None	0.000	0
Cataracts	cigarettes per day	None	0.000	0
Cervical cancer	pack-year	None	0.000	0
Colon and rectum cancer	pack-year	None	0.000	0
COPD	pack-year	cv_subpopulation, cv_adj_L1	0.000	1
Diabetes (type 2)	cigarettes per day	cv_subpopulation	0.105	0
Oesophageal cancer	pack-year	cv_exposure_selfreport	0.000	0
Gallbladder diseases	cigarettes per day	cv_adj_L0	0.000	0
Fracture (hip and non-hip)	Binary smoking status	cv_adj_L1	0.099	1
Ischaemic heart disease	cigarettes per day	cv_adj_L2, cv_subpopulation, cv_adj_L1, cv_adj_L0, cv_older	0.206	1
Kidney cancer	pack-year	None	0.000	1
Laryngeal cancer	pack-year	cv_adj_L0	0.000	0
Lower back pain	cigarettes per day	None	0.000	0
Leukaemia	pack-year	None	0.000	0
Lip and oral cavity cancer	pack-year	cv_adj_L0	0.105	1
Liver cancer	pack-year	None	0.214	1
Lower respiratory infection	cigarettes per day	None	0.000	0
Lung cancer	pack-year	cv_adj_L1, cv_adj_L0	0.058	1

Macular degeneration	cigarettes per day	None	0.000	0
Multiple sclerosis	cigarettes per day	None	0.000	0
Nasopharyngeal cancer	pack-year	None	0.071	1
Other pharynx cancer	pack-year	cv_exposure_selfreport	0.000	0
Pancreatic cancer	pack-year	None	0.000	0
Parkinson’s disease	cigarettes per day	cv_adj_L2, cv_outcome_selfreport, cv_older	0.000	1
Peptic ulcer	cigarettes per day	cv_adj_L1, cv_subpopulation	0.000	0
Peripheral artery disease	cigarettes per day	cv_subpopulation	0.000	0
Prostate cancer	cigarettes per day	None	0.155	1
Rheumatoid arthritis	cigarettes per day	None	0.000	1
Stomach cancer	pack-year	None	0.000	0
Stroke (ischaemic stroke, haemorrhagic stroke, and subarachnoid haemorrhage)	cigarettes per day	None	0.104	0
Tuberculosis	cigarettes per day	None	0.099	0

† definitions of bias covariates:

cv_subpopulation: 0 for risk estimates are likely generalisable to the general population because the sample was based on the general population with reasonable exclusions for pre-existing disease states; 1 for risk estimates of sub-groups such as high-risk groups

cv_adj_L0, cv_adj_L1, cv_adj_L2: cascading dummy variables for adjustment level of the risk estimates (ie, how many confounders are adjusted for in the regression model for the risk estimate). There are four adjustment levels, namely, 1. no adjustment, 2. only adjusting for age and sex, 3. adjusting for age and sex and ≤3 other covariates, and 4. adjusting for age and sex and >3 other covariates. If the adjustment level is 1, cv_adj_L0=1, cv_adj_L1=1, cv_adj_L2=1; if the adjustment level is 2, cv_adj_L0=1, cv_adj_L1=1, cv_adj_L2=0; if the adjustment level is 3, then cv_adj_L0=1, cv_adj_L1=0, cv_adj_L2=0; if the adjustment level is 4, then cv_adj_L0=0, cv_adj_L1=0, cv_adj_L2=0.

cv_exposure_selfreport: 0 for measurement of exposure based on assays, tests, or physician observation and 1 for self-report exposure.

cv_outcome_selfreport: 0 for measurement of outcome based on assays, tests, or physician observation and 1 for self-report outcome.

cv_older: 0 if the population contains both young and old people; 1 if the population only contains old people.

Population attributable fraction (PAF)

As in GBD 2019, we estimated PAFs based on the following equation:

$$PAF = \frac{p(n) + p(f)\int \exp(x) * rr(x) + p(c)\int \exp(y) * rr(y) - 1}{p(n) + p(f)\int \exp(x) * rr(x) + p(c)\int \exp(y) * rr(y)}$$

where $p(n)$ is the prevalence of never smokers, $p(f)$ is the prevalence of former smokers, $p(c)$ is the prevalence of current smokers, $exp(x)$ is a distribution of years since quitting among former smokers, $rr(x)$ is the relative risk for years since quitting, $exp(y)$ is a distribution of cigarettes per smoker per day or pack-years, and $rr(y)$ is the relative risk for cigarettes per smoker per day or pack-years.

We used pack-years as the exposure definition for cancers and chronic respiratory diseases, and cigarettes per smoker per day for cardiovascular diseases and all other health outcomes.

References

1. Ng M, Freeman MK, Fleming TD, Robinson M, Dwyer-Lindgren L, Thomson B, et al. Smoking prevalence and cigarette consumption in 187 countries, 1980-2012. JAMA. 2014 Jan 8;311(2):183–92.
2. Zheng P, Aravkin A, Barber R, Sorensen R, Murray C. Trimmed Constrained Mixed Effects Models: Formulations and Algorithms. bioRxiv. 2020 Jan 29;2020.01.28.923599.

High fasting plasma glucose/Diabetes

Flowchart

***Figure 1**: Calculating high fasting plasma glucose attributable burden*

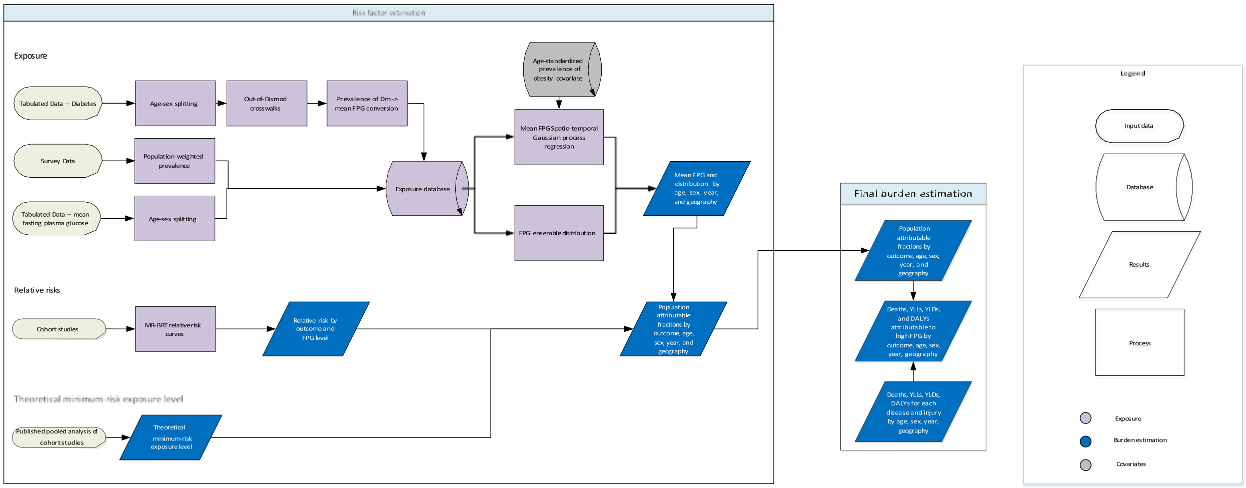

Input data and methodological summary

Definition

Exposure

High fasting plasma glucose (FPG) is measured as the mean FPG in a population, where FPG is a continuous exposure in units of mmol/L. Since FPG is along a continuum, we define high FPG as any level above the theoretical minimum-risk exposure level (TMREL), which is 4.9–5.3 mmol/L.

Data seeking

Exposure

Collaborator-provided sources that were either shared directly with us or were identified through searching the Global Health Data Exchange (GHDx) were reviewed for inclusion.

- 139 new sources were included in the FPG exposure model for GBD 2021.

No systematic review was conducted for the FPG exposure model for GBD 2021; the most recent systematic review was conducted for GBD 2019. In place of a systematic review, an "audit" of the current data in the FPG model was undertaken. The audit process involved returning to each data source to re-evaluate inclusion into the model, and to re-check data extractions for those sources that remain eligible for inclusion. Both GBD 2019 sources and the 139 new GBD 2021 sources were included in the audit.

Figure 2: *Diagram of data sources in the GBD 2021 FPG exposure model*

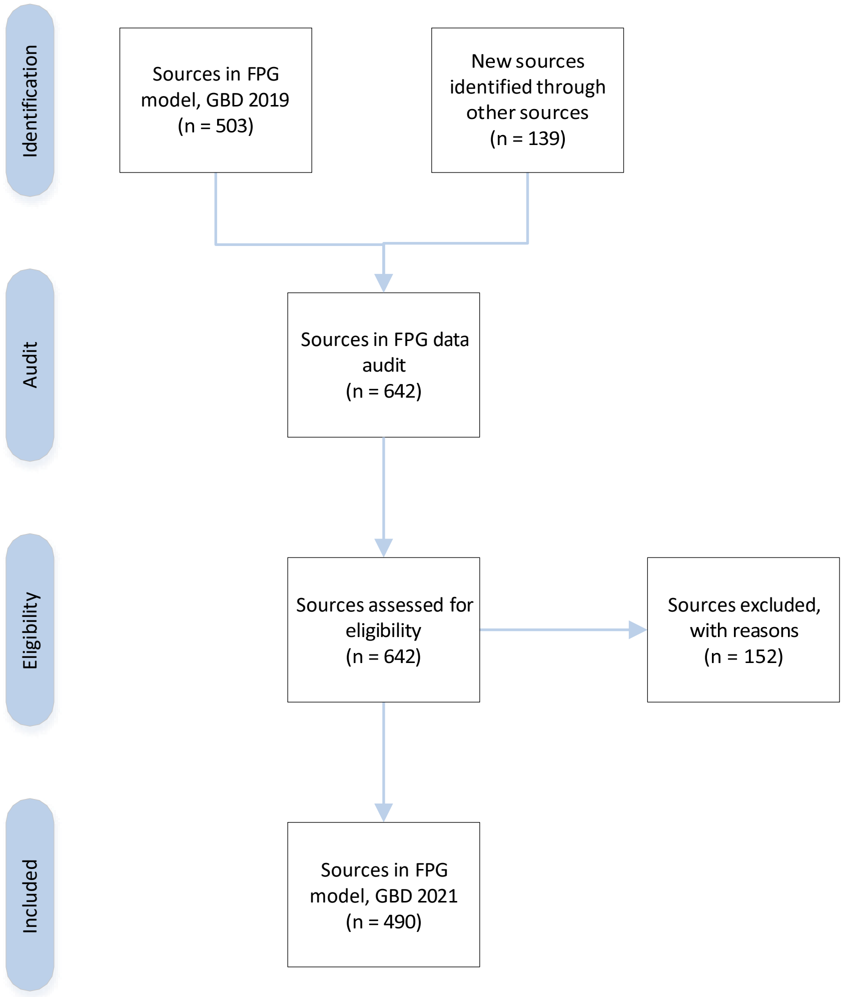

Common exclusion reasons include duplicative studies and not population representative.

Relative Risk

For each outcome, our goal was to extract the estimates from the original cohort study. To accomplish this, we re-reviewed the relative risk studies used in previous GBD rounds and looked for the original studies used in any meta-analysis. In the event that a study in the meta-analysis was a pooled analysis or we found a study in our PubMed search was a pooled study, we made an effort to document the cohorts used and attempted to identify different studies that reported estimates for each individual cohort. The search strings were grouped based on topics due to reports of multiple outcomes within the same study. Below are diagrams for the review of studies used in past rounds of GBD as well as the studies found in the review of sources.

Due to the number of relative risk studies we found and amount of time and personnel resources, we prioritised our search for additional studies. First, we looked for original articles used in the studies we accepted in previous GBD rounds. Second, we looked for original articles used in the additional meta-analysis studies we identified. Finally, we reviewed as many sources from the search string as we were able to within a 4-month time period. Below are the results for each effort.

Outcome: tuberculosis

- Previously accepted studies

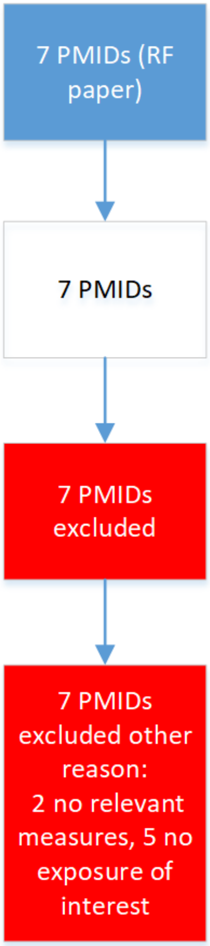

- Search string

((((tuberculosis[MeSH Terms]) OR tuberculosis[Title/Abstract])) AND (((((((Case-Control Studies[MeSH Terms] OR Cross-Over Studies[MeSH Terms] OR Cohort Studies[MeSH Terms] OR Systematic Review[Publication Type] OR Meta-Analysis[Publication Type] OR systematic review[Title/Abstract] OR meta-analysis[Title/Abstract] OR cohort[Title/Abstract] OR cross-over[Title/Abstract] OR crossover[Title/Abstract] OR case-control[Title/Abstract] OR prospective[Title/Abstract] OR retrospective[Title/Abstract] OR longitudinal[Title/Abstract] OR follow-up[Title/Abstract] OR Dose-Response Relationship, Drug[MeSH Terms] OR dose-response[Title/Abstract]) AND (Risk[MeSH Terms] OR Odds Ratio[MeSH Terms] OR risk[Title/Abstract] OR odds ratio[Title/Abstract] OR cross-product ratio[Title/Abstract] OR hazards ratio[Title/Abstract] OR hazard ratio[Title/Abstract]) AND (1970/01/01[PDat] : 2019/12/31[PDat]) NOT (animals[MeSH Terms] NOT Humans[MeSH Terms]))))))) AND ((((((diabetes[MeSH Terms]) OR diabetes[Title/Abstract]) OR hyperglycemia[MeSH Terms]) OR hyperglycemia[Title/Abstract]) OR blood glucose[MeSH Terms]) OR blood glucose[Title])

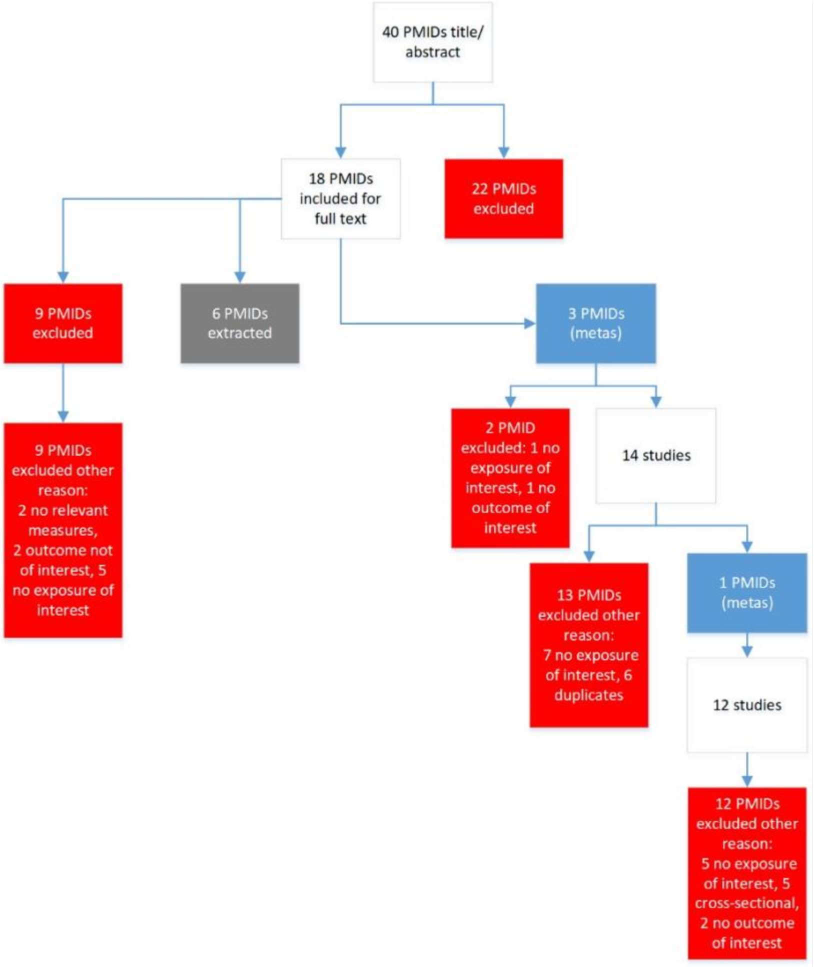

Data inputs

Exposure

We used all available sources on FPG and prevalence of diabetes in the FPG model. Data inputs came from three sources:

- Estimates of mean FPG in a representative population
- Individual-level data of FPG measured from surveys
- Estimates of diabetes prevalence in a representative population

Data sources that did not report mean FPG or prevalence of diabetes were excluded from analysis. When a study reported both mean FPG and prevalence of diabetes, we used the mean FPG for exposure estimates. Where possible, individual-level data supersede any data presented in a published study or report. Individual-level data were aggregated to produce estimates for each 5-year age group, sex, location, and year of a survey.

Table 1: *Data inputs for exposure for high FPG*

	**Countries with data**	**New sources**	**Total sources**
Exposure	151	133	493

Relative risk

***Table 2:** Data inputs for relative risks for high FPG*

	Countries with data	New sources	Total sources
Relative risks	37	236	240

Data processing

We performed several processing steps to the data in order to address sampling and measurement inconsistencies that ensure the data are comparable across data sources and between diabetes mellitus prevalence modelling efforts.

1. *Small sample size*

Data with a sample size of 10 or less were outliered prior to modelling.

2. *Diabetes prevalence processing*

We used an ensemble distribution to estimate mean FPG based on prevalence of diabetes for sources where data on mean FPG were not available, but there were data on diabetes prevalence. Essentially, we constructed a distribution based on unit-level data available in 31 countries. Before predicting mean FPG from prevalence of diabetes, we ensured that the prevalence of diabetes was based on the reference case definition: FPG greater than or equal to 126 mg/dL (7 mmol/L) or on treatment. For more details on how the case definition crosswalk is conducted, please see the diabetes mellitus appendix section. Then, we predicted out the mean FPG by age and sex.

3. *Age and sex splitting*: Reported estimates of mean FPG were split by age and sex where possible. First, if studies reported mean FPG for broad age groups by sex, and also by specific age groups but for both sexes combined, age-specific estimates were split by sex using the sex ratio from within the study. Second, input data reporting mean FPG for both sexes that could not be split using a within-study ratio were split using a sex ratio derived from a meta-analysis of existing sex-specific data using meta-regression—Bayesian, regularised, trimmed (MR-BRT).[1] Finally, where studies reported estimates across age groups spanning more than five years, these were split into five-year age groups using either the age midpoint of the estimate or the diabetes prevalence age pattern estimated by disease model—Bayesian meta-regression (DisMod-MR 2.1)[2] from a model that contained the subset of diabetes prevalence data with age range less than 25 years. Additional information on DisMod-MR 2.1 can be found in appendix 1, section 4.5 of the reference article.

Modelling strategy

Exposure

Exposure estimates were produced for every year between 1980 and 2021 for each national and subnational location, sex, and for each 5-year age group starting from 25 years. As in previous rounds of GBD, we used a spatiotemporal Gaussian process regression (ST-GPR)[3] framework to model the mean FPG at the location-, year-, age-, and sex-level. Additional information on ST-GPR can be found in appendix 1, section 3.3.3 of the reference article.

To inform our estimates in data-sparse countries, we systematically tested a range of covariates and selected age-specific prevalence of obesity as a covariate based on direction of the coefficient and significance level.

Mean FPG was estimated using a mixed-effects linear regression, run separately by sex:

$$\text{logit}(\text{FPG}_{c,a,t}) = \beta_0 + \beta_1 p_{\text{overweight}_{c,a,t}} + \sum_{k=2}^{16} \beta_k I_{A[a]} + \alpha_s + \alpha_r + \alpha_c + \epsilon_{c,a,t}$$

where $p_{\text{overweight}_{c,a,t}}$ is the prevalence of overweight, $I_{A[a]}$ is an indicator variable for a fixed effect on a given 5-year age group, and α_s α_r α_c are random effects at the super-region, region, and country level, respectively. The estimates were then propagated through the ST-GPR framework to obtain 1000 draws for each location, year, age, and sex.

FPG distributions were created using an ensemble distribution of FPG individual-level data.

Theoretical minimum-risk exposure level

The TMREL for FPG is 4.9–5.3 mmol/L. This was calculated by taking the person-year weighted average of the levels of FPG that were associated with the lowest risk of mortality in the pooled analyses of prospective cohort studies.[4]

Relative risks

In GBD 2021, we attributed burden of 25 level 4 diseases to high FPG. We made several updates in relative risk estimation and population attributable fraction (PAF) estimation detailed below.

First, we re-reviewed all the literature and opportunistically searched for new studies with information on association between blood glucose and each outcome. Please see the Data seeking section for relative risk above for more details.

Second, we used all the available data to create risk curves across the exposure domain for all outcomes. This resulted in transitioning all risk curves to continuous FPG exposure domains. In previous GBD rounds, some high FPG outcomes were modeled as categorical risk-outcome pairs due to the nature of the relative risk data.
We incorporated data in a meta-analytic tool to estimate the relative risk as well as evaluate the strength of evidence of the association between FPG and each outcome, for relationships that are not a PAF of 1, which include type 1 and type 2 diabetes. This means that 100% of type 1 and type 2 diabetes burden is attributable to high FPG. A description of the methods and approach can be found in the Evidence score documentation, section 6.1 (Continuous Risk-Outcome Pairs). Risk curves for each outcome can be found in the Burden of Proof tool.

To assess and estimate the relationship between the risk factors (e.g., fasting plasma glucose (FPG)) and TB, we utilize the Burden of Proof (BOP) meta-analytic framework (detailed methods of the Burden of Proof framework are published here: https://www.nature.com/articles/s41591-022-01973-2). In this framework, systematic biases due to various study attributes, including controlling for confounders are tested and adjusted for within the process and accounted for in the final risk ratios that we use for estimated relative risks.

In this approach, we extract data from relevant studies reporting on relative risk, hazard ratio, incidence rate ratio or odd ratios. In addition to extracting the risk ratio values, we also extract a wide range of study characteristics and study biases. This includes bias information about confounders that are or are not adjusted for per each reported risk ratio. The types of specific confounders found within each study are grouped into metabolic, behavioral, and demographic confounders. For example, if a study adjusted for age and sex, the demographic confounder bias covariate will be coded to 0, compared to a study that only reported on crude risk ratios, the demographic confounder will be coded to 1. Each study is, then labeled based on the level of adjustment by the types of confounders to create an incomplete confounder adjustment bias covariate.

This incomplete confounder adjustment bias covariate, amongst other study-level biases, is then tested for significance through an interaction term between the estimated risk ratio and each bias covariate in a linear meta-regression with the relative risks as the dependent variable. Bias covariates that are significant are retained in the model, and ultimately adjusted for in the final relative risk model along with its uncertainties around the risk on the continuum of the risk relationship between FPG and TB.

References

1. Zheng, P., Barber, R., Sorensen, R. J., Murray, C. J., & Aravkin, A. Y. (2021). Trimmed constrained mixed effects models: formulations and algorithms. Journal of Computational and Graphical Statistics, 1-13. https://www.tandfonline.com/doi/full/10.1080/10618600.2020.1868303
2. GBD 2019 Diseases and Injuries Collaborators. Global burden of 369 diseases and injuries in 204 countries and territories, 1990–2019: a systematic analysis for the Global Burden of Disease Study 2019. Lancet 2020; 396: 1204–22. doi: https://doi.org/10.1016/S0140-6736(20)30925-9
3. GBD 2019 Risk Factors Collaborators. Global burden of 87 risk factors in 204 countries and territories, 1990–2019: a systematic analysis for the Global Burden of Disease Study 2019. Lancet 2020; 396: 1223–49. doi: https://doi.org/10.1016/S0140-6736(20)30752-2
4. Singh GM, Danaei G, Farzadfar F, et al. The age-specific quantitative effects of metabolic risk factors on cardiovascular diseases and diabetes: a pooled analysis. PloS One 2013; 8: e65174.

Authors' Contributions

Managing the overall research enterprise

Amanda Novotney, Mohsen Naghavi, Simon I Hay, Christopher J L Murray, and Hmwe H Kyu.

Writing the first draft of the manuscript

Jorge R Ledesma, Jianing Ma, Meixin Zhang, Christopher J L Murray, and Hmwe H Kyu.

Primary responsibility for applying analytical methods to produce estimates

Jorge R Ledesma, Jianing Ma, Meixin Zhang, Ann Basting, Yvonne Xu, Xiaochen Dai, Sneha Ingle Nicholson, and Lauryn K Stafford.

Primary responsibility for seeking, cataloging, extracting, or cleaning data; designing or coding figures and tables

Jorge R Ledesma, Jianing Ma, Meixin Zhang, Ann V L Basting, Huong T Chu, and Avina Vongpradith.

Providing data or critical feedback on data sources

Hedayat Abbastabar, Meriem Abdoun, Richard Gyan Aboagye, Hassan Abolhassani, Hiwa Abubaker Ali, Abiola Victor Adepoju, Qorinah Estiningtyas Sakilah Adnani, Bright Opoku Ahinkorah, Danish Ahmad, Sajjad Ahmad, Ayman Ahmed, Haroon Ahmed, Mohammed Ahmed, Mohammed Albashtawy, Mohammad T. AlBataineh, Abid Ali, Liaqat Ali, Syed Shujait Shujait Ali, Awais Altaf, Nelson Alvis-Guzman, Nelson J. Alvis-Zakzuk, Ganiyu Adeniyi Amusa, Anton A Artamonov, Mulusew A Asemahagn, Sachin R Atre, Ahmed Y Azzam, Ashish D Badiye, Sara Bagherieh, Atif Amin Baig, Maciej Banach, Biswajit Banik, Mainak Bardhan, Hiba Jawdat Barqawi, Maryam Beiranvand, Melaku Ashagrie Belete, Uzma Iqbal Belgaumi, Akshaya Srikanth Bhagavathula, Gurjit Kaur Bhatti, Jasvinder Singh Bhatti, Boris Bikbov, Katrin Burkart, Vijay Kumar Chattu, Hitesh Chopra, Eunice Chung, Natália Cruz-Martins, Xiaochen Dai, Aso Mohammad Darwesh, Mahmood Dashti, Biniyam Demisse, Awoke Masrie Asrat Derese, Kebede Deribe, Hardik Dineshbhai Desai, Vinoth Gnana Chellaiyan Devanbu, Sameer Dhingra, Thao Huynh Phuong Do, Haneil Larson Dsouza, Diyan Ermawan Effendi, Aziz Eftekharimehrabad, Temitope Cyrus Ekundayo, Adeniyi Francis Fagbamigbe, Ayesha Fahim, Alireza Feizkhah, Kahsu Gebrekirstos Gebrekidan, Kazem Ghaffari, Amador Goodridge, Shi-Yang Guan, Sapna Gupta, Veer Bala Gupta, Vivek Kumar Gupta, Wase Benti Hailu, Harapan Harapan, Rumina Syeda Hasan, Johannes Haubold, Simon I Hay, Sung Hwi Hong, Mehdi Hosseinzadeh, Hong-Han Huynh, Segun Emmanuel

Ibitoye, Nahlah Elkudssiah Ismail, Mihajlo Jakovljevic, Mahsa Jalili, Jost B. Jonas, Charity Ehimwenma Joshua, Zubair Kabir, Himal Kandel, Kehinde Kazeem Kanmodi, Rami S. Kantar, Gebrehiwot G Kassa, Himanshu Khajuria, M Nuruzzaman Khan, Yusra H Khan, Mahammed Ziauddin Khan suheb, Khaled Khatab, Soewarta Kosen, Sindhura Lakshmi Koulmane Laxminarayana, Kewal Krishan, Mukhtar Kulimbet, Hmwe Hmwe Kyu, Dharmesh Kumar Lal, Kamaluddin Latief, Thao Thi Thu Le, Trang Diep Thanh Le, Caterina Ledda, Jorge R. Ledesma, Munjae Lee, Seung Won Lee, Kate E LeGrand, Stephen S Lim, Xuefeng Liu, Hong Luo, Kashish Malhotra, Tauqeer Hussain Mallhi, Aseer Manilal, Bernardo Alfonso Martinez-Guerra, Francisco Rogerlândio Martins-Melo, Roy Rillera Marzo, Hossein Masoumi-Asl, Walter Mendoza, Ritesh G Menezes, Arup Kumar Misra, Babak Moazen, Ali H Mokdad, Lorenzo Monasta, Francesk Mulita, Christopher J L Murray, Ahamarshan Jayaraman Nagarajan, Mohsen Naghavi, Ganesh R Naik, Sanjeev Nair, Zuhair S Natto, Biswa Prakash Nayak, Dang H Nguyen, Van Thanh Nguyen, Robina Khan Niazi, Sneha Ingle Nicholson, Chukwudi A Nnaji, Lawrence Achilles Nnyanzi, Shuhei Nomura, Bogdan Oancea, Kehinde O Obamiro, Ismail A. Odetokun, Chukwuma O Okereke, Osaretin Christabel Okonji, Edgar Ortiz-Brizuela, Uchechukwu Levi Osuagwu, Amel Ouyahia, Mahesh Padukudru P A, Romil R Parikh, Ashwaghosha Parthasarathi, Shankargouda Patil, Shrikant Pawar, Veincent Christian Filipino Pepito, Hoang Tran Pham, Maarten J Postma, Attur Ravindra Attur Prabhu, Elton Junio Sady Prates, Fakher Rahim, Masoud Rahmati, Shakthi Kumaran Ramasamy, Sowmya J Rao, Ahmed Mustafa Rashid, Zubair Ahmed Ratan, Nakul Ravikumar, Salman Rawaf, Luis Felipe Reyes, Mónica Rodrigues, Priyanka Roy, Basema Saddik, Umar Saeed, Sher Zaman Safi, Narjes Saheb Sharif-Askari, Afeez Abolarinwa Salami, Hossein Samadi Kafil, Sara Samadzadeh, Subramanian Senthilkumaran, Pritik A Shah, Masood Ali Shaikh, Sunder Sham, Medha Sharath, Aminu Shittu, Jasvinder A. Singh, Paramdeep Singh, Yonatan Solomon, Chandrashekhar T Sreeramareddy, Lauryn K Stafford, Chandan Kumar Swain, Takahiro Tabuchi, Mircea Tampa, Pugazhenthan Thangaraju, Chern Choong Chern Thum, Ruoyan Tobe-Gai, Temesgen Mohammed Toma, Tungki Pratama Umar, Job F M van Boven, Shoban Babu Varthya, Avina Vongpradith, Theo Vos, Ziyue Wang, Ronny Westerman, Tewodros Eshete Wonde, Siyan Yi, Naohiro Yonemoto, Chuanhua Yu, Francis Zeukeng, and Magdalena Zielińska.

Developing methods or computational machinery

Hiwa Abubaker Ali, Qorinah Estiningtyas Sakilah Adnani, Mohammed Ahmed, Tareq Mohammed Ali AL-Ahdal, Liaqat Ali, Aleksandr Y Aravkin, Mulusew A Asemahagn, Ahmed Y Azzam, Saeed Bahadorikhalili, Akshaya Srikanth Bhagavathula, Kaleb Coberly, Xiaochen Dai, Aso Mohammad Darwesh, Awoke Masrie Asrat Derese, Hardik Dineshbhai Desai, Aziz Eftekharimehrabad, Adeniyi Francis Fagbamigbe, Kazem Ghaffari, Simon I Hay, Mehdi Hosseinzadeh, Hong-Han Huynh, Kevin S Ikuta, Mahsa Jalili, Charity Ehimwenma Joshua, Hmwe Hmwe Kyu, Thao Thi Thu Le, Jorge R. Ledesma, Ali H Mokdad, Francesk Mulita, Christopher J L Murray, Mohsen Naghavi, Van Thanh Nguyen, Sneha Ingle Nicholson, Ashwaghosha Parthasarathi, Hoang Tran Pham, Mónica Rodrigues, Umar Saeed, Hesamaddin Shirzad-Aski, Lauryn K Stafford, Chandan Kumar Swain, Razieh Tavakoli Oliaee, Chern Choong Chern Thum, Shoban Babu Varthya, Theo Vos, Ronny Westerman, Tewodros Eshete Wonde, Francis Zeukeng, and Peng Zheng.

Providing critical feedback on methods or results

Hedayat Abbastabar, Meriem Abdoun, Deldar Morad Abdulah, Richard Gyan Aboagye, Hassan Abolhassani, Woldu Aberhe Abrha, Hiwa Abubaker Ali, Eman Abu-Gharbieh, Salahdein Aburuz, Isaac Yeboah Addo, Abiola Victor Adepoju, Kishor Adhikari, Qorinah Estiningtyas Sakilah Adnani, Saryia Adra, Abel Afework, Shahin Aghamiri, Williams Agyemang-Duah, Bright Opoku Ahinkorah, Danish Ahmad, Sajjad Ahmad, Amir Mahmoud Ahmadzade, Ayman Ahmed, Haroon Ahmed, Mohammed Ahmed, Karolina Akinosoglou, Tareq Mohammed Ali AL-Ahdal, Nazmul Alam, Mohammed Albashtawy, Mohammad T. AlBataineh, Adel Ali Saeed Al-Gheethi, Endale Alemayehu Ali, Liaqat Ali, Syed Shujait Shujait Ali, Zahid Ali, Kasim Allel, Awais Altaf, Jaffar A. Al-Tawfiq, Nelson Alvis-Guzman, Nelson J. Alvis-Zakzuk, Reza Amani, Ganiyu Adeniyi Amusa, Jimoh Amzat, Jason R Andrews, Razique Anwer, Damelash Areda, Anton A Artamonov, Raphael Taiwo Aruleba, Mulusew A Asemahagn, Sachin R Atre, Avinash Aujayeb, Davood Azadi, Sina Azadnajafabad, Ahmed Y Azzam, Muhammad Badar, Ashish D Badiye, Sara Bagherieh, Saeed Bahadorikhalili, Atif Amin Baig, Maciej Banach, Biswajit Banik, Mainak Bardhan, Hiba Jawdat Barqawi, Zarrin Basharat, Pritish Baskaran, Ann V L Basting, Saurav Basu, Melaku Ashagrie Belete, Makda Abate Belew, Uzma Iqbal Belgaumi, Apostolos Beloukas, Paulo J G Bettencourt, Akshaya Srikanth Bhagavathula, Nikha Bhardwaj, Pankaj Bhardwaj, Ashish Bhargava, Vivek Bhat, Gurjit Kaur Bhatti, Jasvinder Singh Bhatti, Boris Bikbov, Veera R Bitra, Vesna Bjegovic-Mikanovic, Danilo Buonsenso, Katrin Burkart, Yasser Bustanji, Zahid A Butt, Yu Cao, Austin Carter, Luca Cegolon, Muge Cevik, Yaacoub Chahine, Vijay Kumar Chattu, Hitesh Chopra, Huong Thi Chu, Natália Cruz-

Martins, Bashir Dabo, Omid Dadras, Xiaochen Dai, Isaac Darban, Jiregna Darega Gela, Aso Mohammad Darwesh, Mahmood Dashti, Berecha Hundessa Demessa, Biniyam Demisse, Solomon Demissie, Awoke Masrie Asrat Derese, Kebede Deribe, Hardik Dineshbhai Desai, Vinoth Gnana Chellaiyan Devanbu, Arkadeep Dhali, Kuldeep Dhama, Sameer Dhingra, Thao Huynh Phuong Do, Deepa Dongarwar, Haneil Larson Dsouza, John Dube, Arkadiusz Marian Dziedzic, Abdelaziz Ed-Dra, Ferry Efendi, Diyan Ermawan Effendi, Aziz Eftekharimehrabad, Nopryan Ekadinata, Temitope Cyrus Ekundayo, Muhammed Elhadi, Legesse Tesfaye Elilo, Theophilus I Emeto, Luchuo Engelbert Bain, Adeniyi Francis Fagbamigbe, Ayesha Fahim, Alireza Feizkhah, Getahun Fetensa, Florian Fischer, Abduzhappar Gaipov, Aravind P Gandhi, Rupesh K. Gautam, Miglas W Gebregergis, Mesfin Gebrehiwot, Kahsu Gebrekirstos Gebrekidan, Kazem Ghaffari, Ramy Mohamed Ghazy, Amador Goodridge, Shi-Yang Guan, Mesay Dechasa Gudeta, Rashid Abdi Guled, Sapna Gupta, Veer Bala Gupta, Vivek Kumar Gupta, Semira Goitom Hailu, Wase Benti Hailu, Samer Hamidi, Asif Hanif, Harapan Harapan, Rumina Syeda Hasan, Johannes Haubold, Simon I Hay, Kamal Hezam, Sung Hwi Hong, Nobuyuki Horita, Md. Belal Hossain, Mehdi Hosseinzadeh, Mihaela Hostiuc, Hong-Han Huynh, Segun Emmanuel Ibitoye, Kevin S Ikuta, Irena M. Ilic, Milena D. Ilic, Md. Rabiul Islam, Nahlah Elkudssiah Ismail, Mihajlo Jakovljevic, Mahsa Jalili, Jost B. Jonas, Nitin Joseph, Charity Ehimwenma Joshua, Zubair Kabir, Tanuj Kanchan, Himal Kandel, Kehinde Kazeem Kanmodi, Rami S. Kantar, Ibraheem M Karaye, Arman Karimi Behnagh, Gebrehiwot G Kassa, Navjot Kaur, Rimple Jeet Kaur, Himanshu Khajuria, Faham Khamesipour, M Nuruzzaman Khan, Yusra H Khan, Mahammed Ziauddin Khan suheb, Khaled Khatab, Fatemeh Khatami, Min Seo Kim, Parvaiz A Koul, Sindhura Lakshmi Koulmane Laxminarayana, Kewal Krishan, Burcu Kucuk Bicer, Md Abdul Kuddus, Mukhtar Kulimbet, Nithin Kumar, Hmwe Hmwe Kyu, Dharmesh Kumar Lal, Kamaluddin Latief, Thao Thi Thu Le, Trang Diep Thanh Le, Caterina Ledda, Jorge R. Ledesma, Seung Won Lee, Kate E LeGrand, Temesgen L. Lerango, Stephen S Lim, Chaojie Liu, Xuefeng Liu, Hong Luo, Hengliang Lv, Preetam Bhalchandra Mahajan, Amir Ali Mahboobipour, Azeem Majeed, Elaheh Malakan Rad, Kashish Malhotra, Muhammad Sajeel Ahmed Malik, Lesibana Anthony Malinga, Tauqeer Hussain Mallhi, Aseer Manilal, Bernardo Alfonso Martinez-Guerra, Francisco Rogerlândio Martins-Melo, Roy Rillera Marzo, Vasundhara Mathur, Richard James Maude, Ravi Mehrotra, Ziad A Memish, Walter Mendoza, Ritesh G Menezes, Tomislav Mestrovic, Laurette Mhlanga, Arup Kumar Misra, Sanjeev Misra, Prasanna Mithra, Babak Moazen, Ali H Mokdad, Catrin E Moore, Christopher J L Murray, Fungai Musaigwa, Raman Muthusamy, Ahamarshan Jayaraman Nagarajan, Mohsen Naghavi, Pirouz Naghavi, Ganesh R Naik, Gurudatta Naik, Sanjeev Nair, Tapas Sadasivan Nair, Zuhair S Natto, Biswa Prakash Nayak, Hadush Negash, Dang H Nguyen, Van Thanh Nguyen, Robina Khan Niazi, Sneha Ingle Nicholson, Chukwudi A Nnaji, Lawrence Achilles Nnyanzi, Efaq Ali Noman, Shuhei Nomura, Bogdan Oancea, Kehinde O Obamiro, Ismail A. Odetokun, Daniel Bogale Odo Odo, Oluwakemi Ololade Odukoya, Osaretin Christabel Okonji, Eyal Oren, Uchechukwu Levi Osuagwu, Amel Ouyahia, Mahesh Padukudru P A, Pragyan Paramita Parija, Romil R Parikh, Seoyeon Park, Ashwaghosha Parthasarathi, Shankargouda Patil, Shrikant Pawar, Minjin Peng, Veincent Christian Filipino Pepito, Prince Peprah, João Perdigão, Hoang Tran Pham, Maarten J Postma, Attur Ravindra Attur Prabhu, Manya Prasad, Akila Prashant, Elton Junio Sady Prates, Fakher Rahim, Mosiur Rahman, Muhammad Aziz Rahman, Sathish Rajaa, Shakthi Kumaran Ramasamy, Indu Ramachandra Rao, Sowmya J Rao, Ahmed Mustafa Rashid, Zubair Ahmed Ratan, Nakul Ravikumar, Salman Rawaf, Murali Mohan Rama Krishna Reddy, Elrashdy Moustafa Mohamed Redwan, Robert Reiner Jr., Luis Felipe Reyes, Nazila Rezaei, Mohsen Rezaeian, Omid Rezahosseini, Mónica Rodrigues, Jennifer M Ross, Siamak Sabour, Basema Saddik, Umar Saeed, Sher Zaman Safi, Fatemeh Saheb Sharif-Askari, Narjes Saheb Sharif-Askari, Biniyam Sahiledengle, Soumya Swaroop Sahoo, Afeez Abolarinwa Salami, Samreen Saleem, Mohamed A. Saleh, Hossein Samadi Kafil, Sara Samadzadeh, Yoseph Leonardo Samodra, Rama Krishna Sanjeev, Susan M Sawyer, Sabyasachi Senapati, Subramanian Senthilkumaran, Pritik A Shah, Samiah Shahid, Masood Ali Shaikh, Mohammad Ali Shamshirgaran, Mohd Shanawaz, Medha Sharath, Samendra P Sherchan, Hesamaddin Shirzad-Aski, Aminu Shittu, Emmanuel Edwar Siddig, Jasvinder A. Singh, Paramdeep Singh, Md Shahjahan Siraj, Siswanto Siswanto, Yonatan Solomon, Joan B Soriano, Chandrashekhar T Sreeramareddy, Vijay Kumar Srivastava, Lauryn K Stafford, Paschalis Steiropoulos, Chandan Kumar Swain, Mircea Tampa, Jacques JL Lukenze Tamuzi, Razieh Tavakoli Oliaee, Gebrehiwot Teklay, Edosa Geta Tesfaye, Belay Tessema, Pugazhenthan Thangaraju, Rekha Thapar, Chern Choong Chern Thum, Jansje Henny Vera Ticoalu, Ruoyan Tobe-Gai, Temesgen Mohammed Toma, Khai Hoan Tram, Aniefiok John Udoakang, Tungki Pratama Umar, Chukwuma David Umeokonkwo, Seyed Mohammad Vahabi, Job F M van Boven, Shoban Babu Varthya, Theo Vos, Ziyue Wang, Muktar S A Warsame, Ronny Westerman, Tewodros Eshete Wonde, Siyan Yi, Vahit Yiğit, Dong Keon Yon, Naohiro Yonemoto, Chuanhua Yu, Fathiah Zakham, Moein Zangiabadian, Francis Zeukeng, Haijun Zhang, Yang Zhao, and Magdalena Zielińska.

Drafting the work or revising it critically for important intellectual content

Hassan Abolhassani, Eman Abu-Gharbieh, Salahdein Aburuz, Isaac Yeboah Addo, Qorinah Estiningtyas Sakilah Adnani, Saryia Adra, Bright Opoku Ahinkorah, Danish Ahmad, Ayman Ahmed, Mohammed Ahmed, Karolina Akinosoglou, Mohammed Albashtawy, Mohammad T. AlBataineh, Abid Ali, Endale Alemayehu Ali, Syed Shujait Shujait Ali, Zahid Ali, Kasim Allel, Awais Altaf, Jaffar A. Al-Tawfiq, Nelson Alvis-Guzman, Nelson J. Alvis-Zakzuk, Reza Amani, Ganiyu Adeniyi Amusa, Jimoh Amzat, Jason R Andrews, Abhishek Anil, Raphael Taiwo Aruleba, Mulusew A Asemahagn, Avinash Aujayeb, Sina Azadnajafabad, Ahmed Y Azzam, Muhammad Badar, Ashish D Badiye, Sara Bagherieh, Atif Amin Baig, Maciej Banach, Mainak Bardhan, Hiba Jawdat Barqawi, Pritish Baskaran, Ann V L Basting, Maryam Beiranvand, Uzma Iqbal Belgaumi, Apostolos Beloukas, Akshaya Srikanth Bhagavathula, Ashish Bhargava, Vivek Bhat, Gurjit Kaur Bhatti, Jasvinder Singh Bhatti, Boris Bikbov, Veera R Bitra, Danilo Buonsenso, Yasser Bustanji, Paulo Camargos, Sinclair Carr, Felix Carvalho, Luca Cegolon, Muthia Cenderadewi, Muge Cevik, Yaacoub Chahine, Vijay Kumar Chattu, Patrick R Ching, Hitesh Chopra, Huong Thi Chu, Eunice Chung, Mareli M Claassens, Natália Cruz-Martins, Bashir Dabo, Sriharsha Dadana, Berecha Hundessa Demessa, Biniyam Demisse, Awoke Masrie Asrat Derese, Kebede Deribe, Hardik Dineshbhai Desai, Arkadeep Dhali, Sameer Dhingra, Deepa Dongarwar, Haneil Larson Dsouza, John Dube, Arkadiusz Marian Dziedzic, Muhammed Elhadi, Legesse Tesfaye Elilo, Theophilus I Emeto, Adeniyi Francis Fagbamigbe, Ayesha Fahim, Getahun Fetensa, Florian Fischer, Aravind P Gandhi, Rupesh K. Gautam, Miglas W Gebregergis, Mesfin Gebrehiwot, Kazem Ghaffari, Fariba Ghassemi, Amador Goodridge, Anmol Goyal, Mesay Dechasa Gudeta, Novianti Br Gultom, Sapna Gupta, Veer Bala Gupta, Vivek Kumar Gupta, Wase Benti Hailu, Harapan Harapan, Shoaib Hassan, Johannes Haubold, Simon I Hay, Kamal Hezam, Nobuyuki Horita, Md. Belal Hossain, Sorin Hostiuc, Hong-Han Huynh, Segun Emmanuel Ibitoye, Irena M. Ilic, Milena D. Ilic, Md. Rabiul Islam, Faisal Ismail, Nahlah Elkudssiah Ismail, Abdollah Jafarzadeh, Mihajlo Jakovljevic, Mahsa Jalili, Manthan Dilipkumar Janodia, Jost B. Jonas, Nitin Joseph, Charity Ehimwenma Joshua, Bhushan Dattatray Kamble, Himal Kandel, Rami S. Kantar, Navjot Kaur, Rimple Jeet Kaur, Himanshu Khajuria, M Nuruzzaman Khan, Yusra H Khan, Mahammed Ziauddin Khan suheb, Khaled Khatab, Min Seo Kim, Parvaiz A Koul, Sindhura Lakshmi Koulmane Laxminarayana, Kewal Krishan, Burcu Kucuk Bicer, Md Abdul Kuddus, Hmwe Hmwe Kyu, Iván Landires, Kamaluddin Latief, Caterina Ledda, Jorge R. Ledesma, Kate E LeGrand, Temesgen L. Lerango, Chaojie Liu, Xuefeng Liu, Platon D Lopukhov, Jianing Ma Ma, Preetam Bhalchandra Mahajan, Elaheh Malakan Rad, Tauqeer Hussain Mallhi, Aseer Manilal, Bernardo Alfonso Martinez-Guerra, Francisco Rogerlândio Martins-Melo, Roy Rillera Marzo, Hossein Masoumi-Asl, Vasundhara Mathur, Ziad A Memish, Walter Mendoza, Ritesh G Menezes, Muayad Aghali Merza, Tomislav Mestrovic, Arup Kumar Misra, Prasanna Mithra, Babak Moazen, Hussen Mohammed, Ali H Mokdad, Lorenzo Monasta, Catrin E Moore, Parsa Mousavi, Christopher J L Murray, Fungai Musaigwa, Raman Muthusamy, Ahamarshan Jayaraman Nagarajan, Mohsen Naghavi, Sanjeev Nair, Zuhair S Natto, Biswa Prakash Nayak, Hadush Negash, Dang H Nguyen, Van Thanh Nguyen, Robina Khan Niazi, Lawrence Achilles Nnyanzi, Bogdan Oancea, Kehinde O Obamiro, Ismail A. Odetokun, Daniel Bogale Odo Odo, Oluwakemi Ololade Odukoya, In-Hwan Oh, Osaretin Christabel Okonji, Eyal Oren, Edgar Ortiz-Brizuela, Uchechukwu Levi Osuagwu, Mahesh Padukudru P A, Pragyan Paramita Parija, Romil R Parikh, Seoyeon Park, Ashwaghosha Parthasarathi, Shankargouda Patil, Shrikant Pawar, Veincent Christian Filipino Pepito, João Perdigão, Norberto Perico, Hoang Tran Pham, Maarten J Postma, Manya Prasad, Akila Prashant, Elton Junio Sady Prates, Fakher Rahim, Masoud Rahmati, Sathish Rajaa, Sowmya J Rao, Deepthi Rapaka, Ahmed Mustafa Rashid, Zubair Ahmed Ratan, Nakul Ravikumar, Salman Rawaf, Elrashdy Moustafa Mohamed Redwan, Giuseppe Remuzzi, Luis Felipe Reyes, Nazila Rezaei, Omid Rezahosseini, Mónica Rodrigues, Jennifer M Ross, Priyanka Roy, Guilherme de Andrade Ruela, Siamak Sabour, Basema Saddik, Umar Saeed, Fatemeh Saheb Sharif-Askari, Narjes Saheb Sharif-Askari, Amirhossein Sahebkar, Biniyam Sahiledengle, Soumya Swaroop Sahoo, Nasir Salam, Afeez Abolarinwa Salami, Samreen Saleem, Joshua A Salomon, Sara Samadzadeh, Yoseph Leonardo Samodra, Aswini Saravanan, Susan M Sawyer, Siddharthan Selvaraj, Sabyasachi Senapati, Samiah Shahid, Mohammad Ali Shamshirgaran, Mohd Shanawaz, Medha Sharath, Samendra P Sherchan, Ranjitha S Shetty, Hesamaddin Shirzad-Aski, Aminu Shittu, Emmanuel Edwar Siddig, João Pedro Silva, Harpreet Singh, Jasvinder A. Singh, Paramdeep Singh, Surjit Singh, Ranjan Solanki, Yonatan Solomon, Joan B Soriano, Chandrashekhar T Sreeramareddy, Chandan Kumar Swain, Mircea Tampa, Jacques JL Lukenze Tamuzi, Nathan Y. Tat, Razieh Tavakoli Oliaee, Gebrehiwot Teklay, Pugazhenthan Thangaraju, Chern Choong Chern Thum, Imad M Tleyjeh, Temesgen Mohammed Toma, Khai Hoan Tram, Aniefiok John Udoakang, Chukwuma David Umeokonkwo, Asokan Govindaraj Vaithinathan, Job F M van Boven, Shoban Babu Varthya, Avina Vongpradith, Ziyue Wang, Muktar S A Warsame, Ronny Westerman, Tewodros Eshete Wonde, Sajad Yaghoubi, Vahit Yiğit, Dong Keon Yon, Naohiro

Yonemoto, Francis Zeukeng, Haijun Zhang, Meixin Zhang, Yang Zhao, and Magdalena Zielińska.

Managing the estimation or publications process

Mohammed Ahmed, Mohammed Albashtawy, Ahmed Y Azzam, Ann V L Basting, Yaacoub Chahine, Kazem Ghaffari, Mesay Dechasa Gudeta, Hailey Hagins, Simon I Hay, Hong-Han Huynh, Mahsa Jalili, M Nuruzzaman Khan, Hmwe Hmwe Kyu, Thao Thi Thu Le, Ali H Mokdad, Christopher J L Murray, Mohsen Naghavi, Van Thanh Nguyen, Amanda Novotney, Mahesh Padukudru P A, Hoang Tran Pham, Pugazhenthan Thangaraju, Chern Choong Chern Thum, Shoban Babu Varthya, Tewodros Eshete Wonde, and Yang Zhao.
