# Supplementary material for: Global, regional, and national age-specific progress towards the 2020 milestones of the WHO End TB Strategy: a systematic analysis for the Global Burden of Disease Study 2021
Source: Lancet Infect Dis. 2024 Jul;24(7):698–725. doi: 10.1016/S1473-3099(24)00007-0 (PMC11187709; doi:10.1016/S1473-3099(24)00007-0)
Supplement: Supplementary appendix 2 [file mmc2.pdf]

THE LANCET
Infectious Diseases

Supplementary appendix 2

This appendix formed part of the original submission and has been peer reviewed. We post it as supplied by the authors.

Supplement to: GBD 2021 Tuberculosis Collaborators. Global, regional, and national age-specific progress towards the 2020 milestones of the WHO End TB Strategy: a systematic analysis for the Global Burden of Disease Study 2021. *Lancet Infect Dis* 2024; published online March 19. https://doi.org/10.1016/S1473-3099(24)00007-0.

Supplementary Results Appendix to “Global, regional, and national age-specific progress towards the 2020 milestones of the END TB strategy: results from the Global Burden of Disease Study 2021”

Table of Contents

eFigure 1. Global age−sex distribution of all−form tuberculosis A) incidence and B) deaths in 2021

eFigure 2. Global age−sex distribution of all−form tuberculosis A) incidence rate and B) mortality rate in 2021

eFigure 3. All-form tuberculosis incidence rate (A) and mortality rate (B) per 100,000 population for children under 5 by geography in 2021

eFigure 4. All-form tuberculosis incidence rate (A) and mortality rate (B) per 100,000 population for children 5 to 14 years by geography in 2021

eFigure 5. All-form tuberculosis incidence rate (A) and mortality rate (B) per 100,000 population for adults 15 to 49 years by geography in 2021

eFigure 6. All-form tuberculosis incidence rate (A) and mortality rate (B) per 100,000 population for adults 50 to 69 years by geography in 2021

eFigure 7. All-form tuberculosis incidence rate (A) and mortality rate (B) per 100,000 population for adults 70 years and older by geography in 2021

eFigure 8. Temporal trends for all−form tuberculosis incidence rate per 100,000 population from 1990 to 2021 for the top 20 high tuberculosis burden countries in the GBD

eFigure 9. Temporal trends for deaths due to all-form tuberculosis deaths from 1990 to 2021 for the top 20 high tuberculosis burden countries in the GBD

eFigure 10. Global age−sex−specific progress towards WHO interim milestones for (A) all−form tuberculosis incidence rate and (B) deaths due to all−form tuberculosis, 1990−2021

eFigure 11. Diagnostic plot for the quasi-Poisson regression model: Pearson residuals against fitted values

eTable 1. All-form tuberculosis incident cases and deaths, age-standardised rates of incidence and mortality per 100,000 population, and corresponding annualized rates of change by age groups for 204 countries and territories (2021)

eTable 2. Progress towards the END-TB 2020 milestones in all-form tuberculosis incidence rate per 100,000 population and in deaths due to all-form tuberculosis by age for 204 countries and territories (2020), percent change from 2015 to 2020

eTable 3. Percent change from 2015 to 2021 in age-standardised all-form tuberculosis incidence rate per 100,000 population and in deaths due to all-form tuberculosis by age for 204 countries and territories

eTable 4. Risk deleted deaths due to all-form tuberculosis for alcohol use, smoking, and diabetes, and all three risk factors combined by age groups for 204 countries and territories (2020) with percent change between 2015 observed deaths and 2020 all-risk deleted deaths

eTable 5. Risk deleted deaths due to all-form tuberculosis for alcohol use, smoking, and diabetes, and all three risk factors combined by age groups for 204 countries and territories in (2021) with percent change between 2015 observed deaths and 2021 all-risk deleted deaths

eTable 6. Observed and expected deaths due to tuberculosis without HIV coinfection in 2020 and 2021 by age for countries with available cause-specific vital registration data

eFigure 1. Global age−sex distribution of all−form tuberculosis A) incidence and B) deaths in 2021

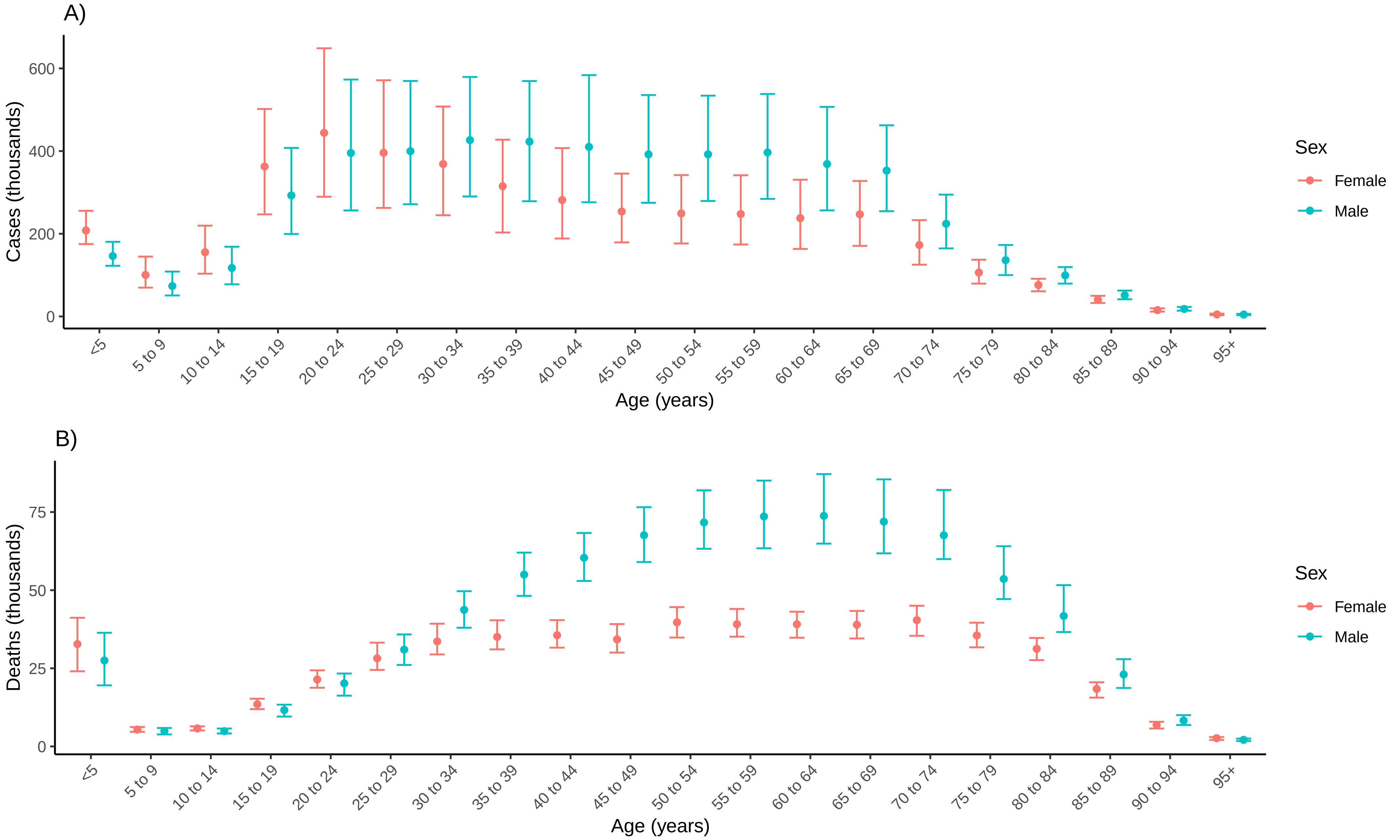

eFigure 2. Global age−sex distribution of all−form tuberculosis A) incidence rate and B) mortality rate in 2021

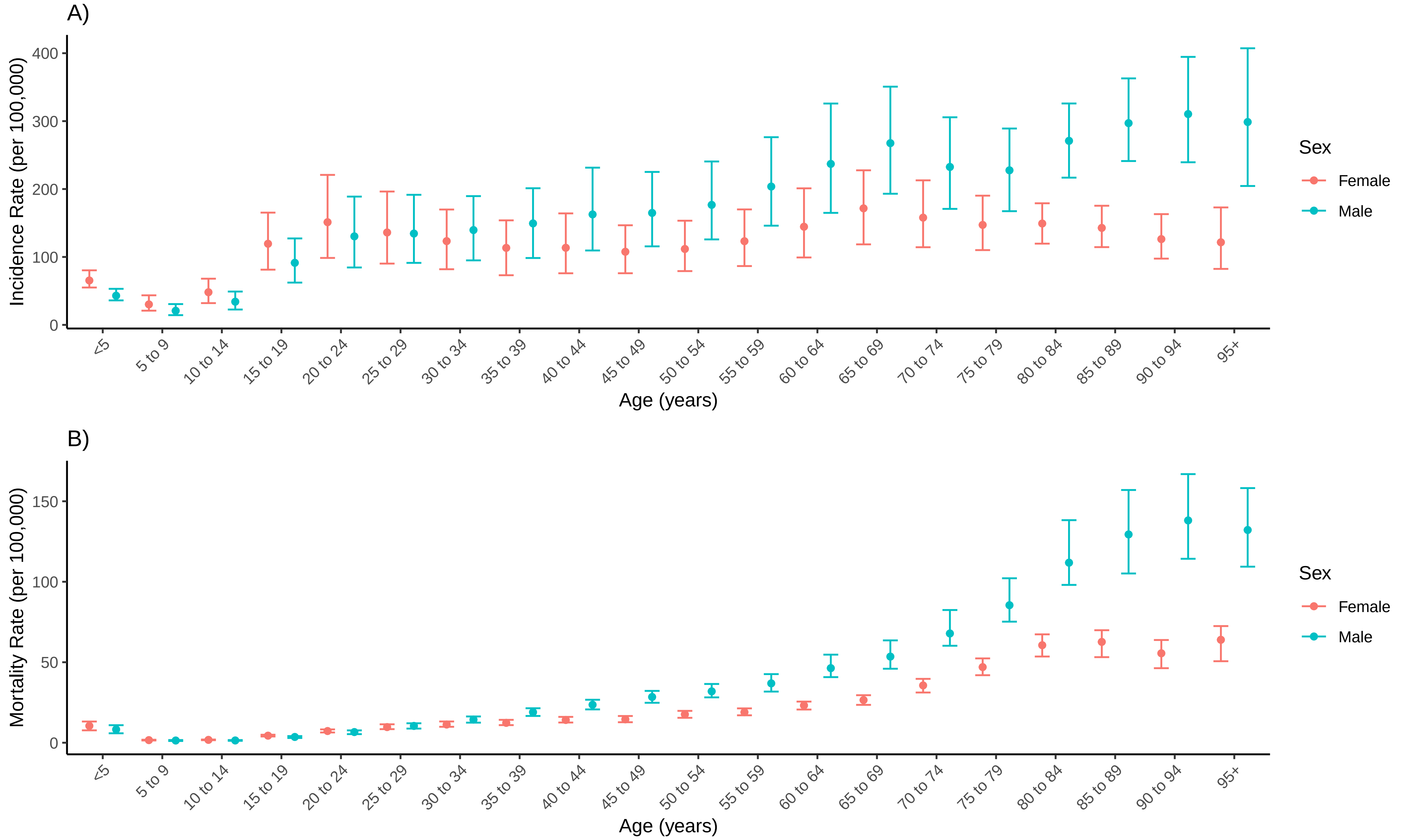

eFigure 3a. All−form TB incidence rates, <5 years, both sex, 2021

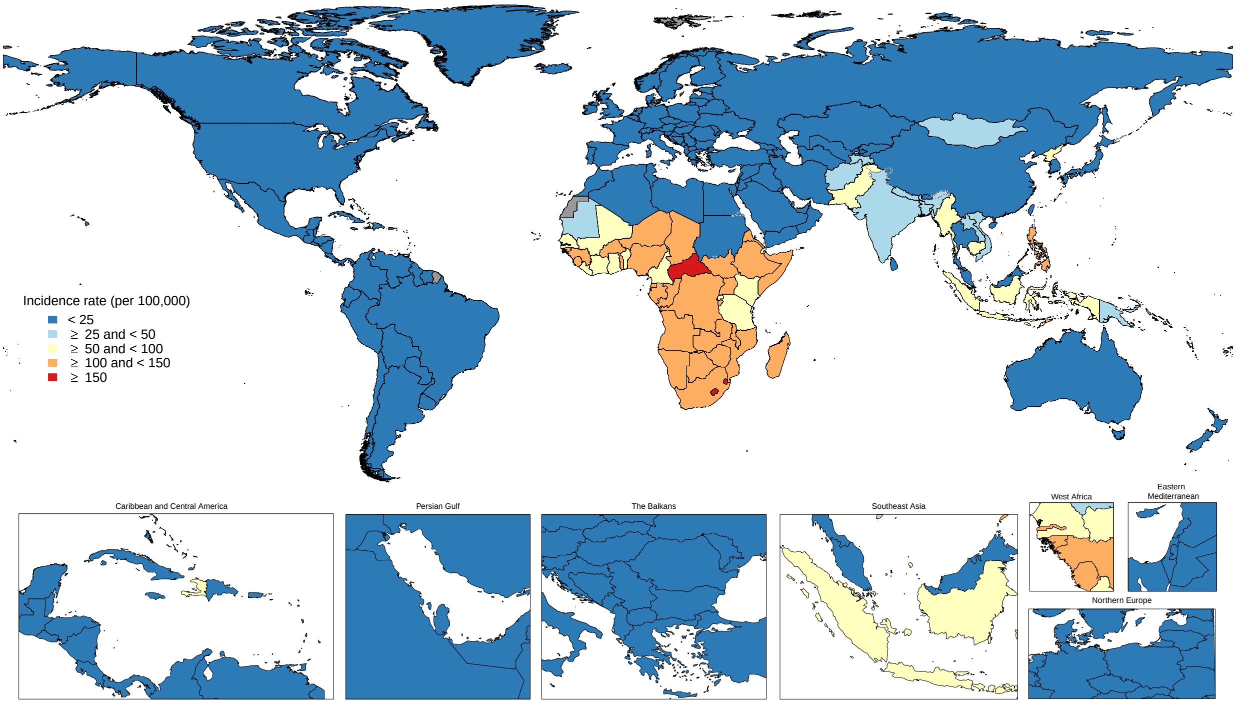

eFigure 3b. All−form TB mortality rates, <5 years, both sex, 2021

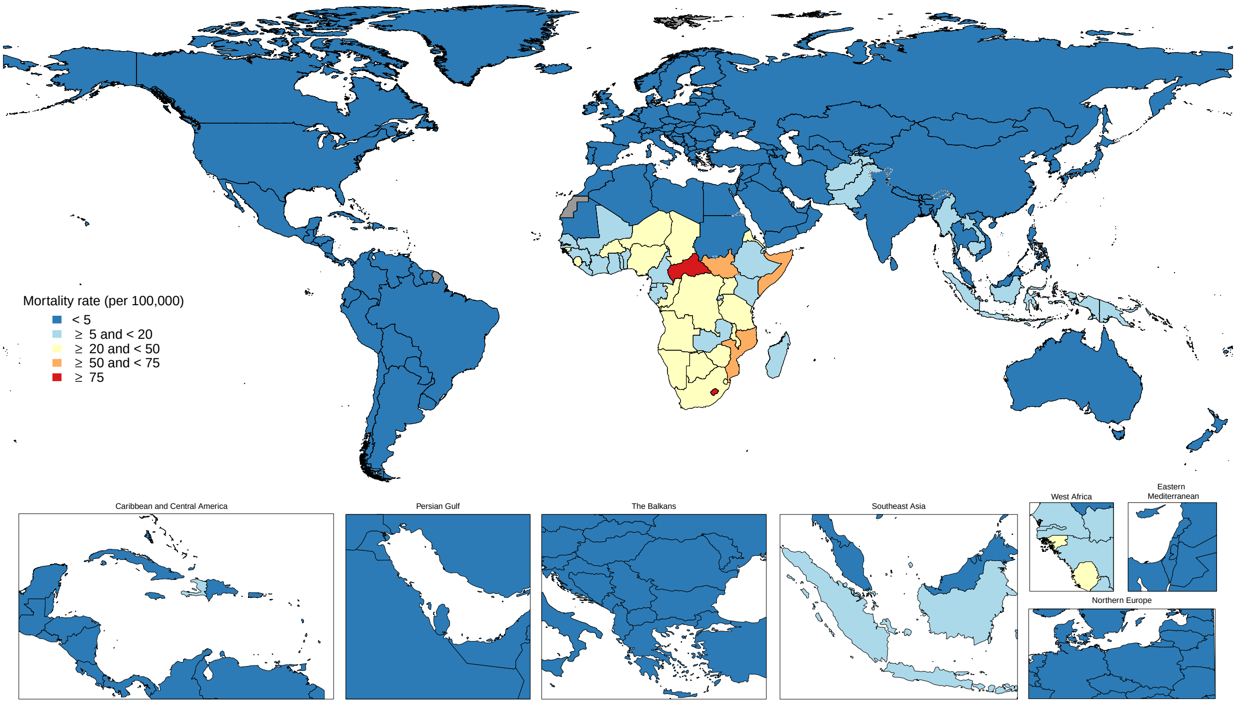

eFigure 4a. All−form TB incidence rates, 5−14 years, both sex, 2021

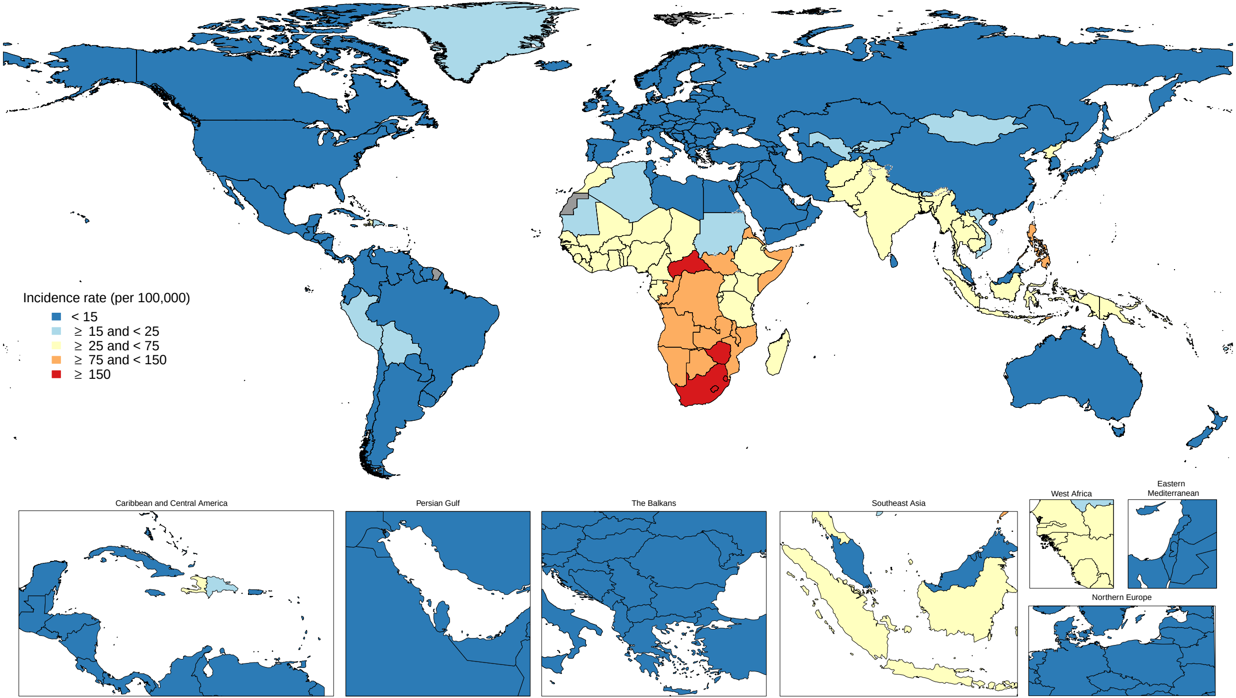

eFigure 4b. All−form TB mortality rates, 5−14 years, both sex, 2021

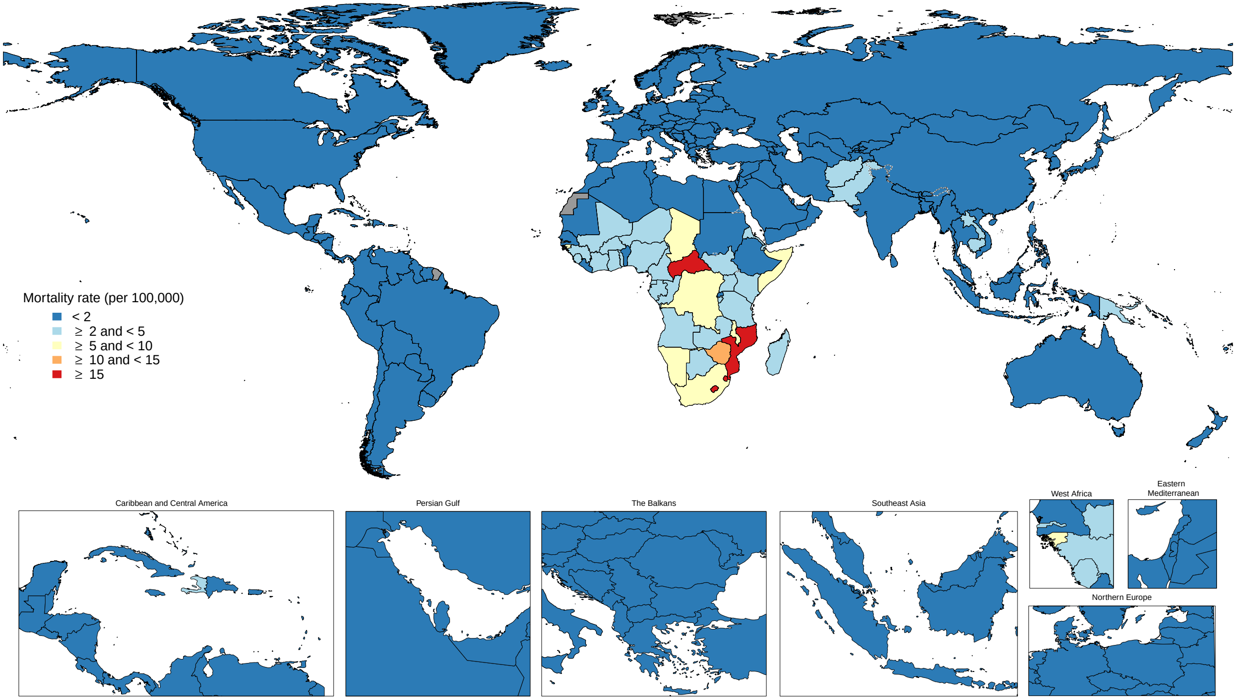

eFigure 5a. All−form TB incidence rates, 15−49 years, both sex, 2021

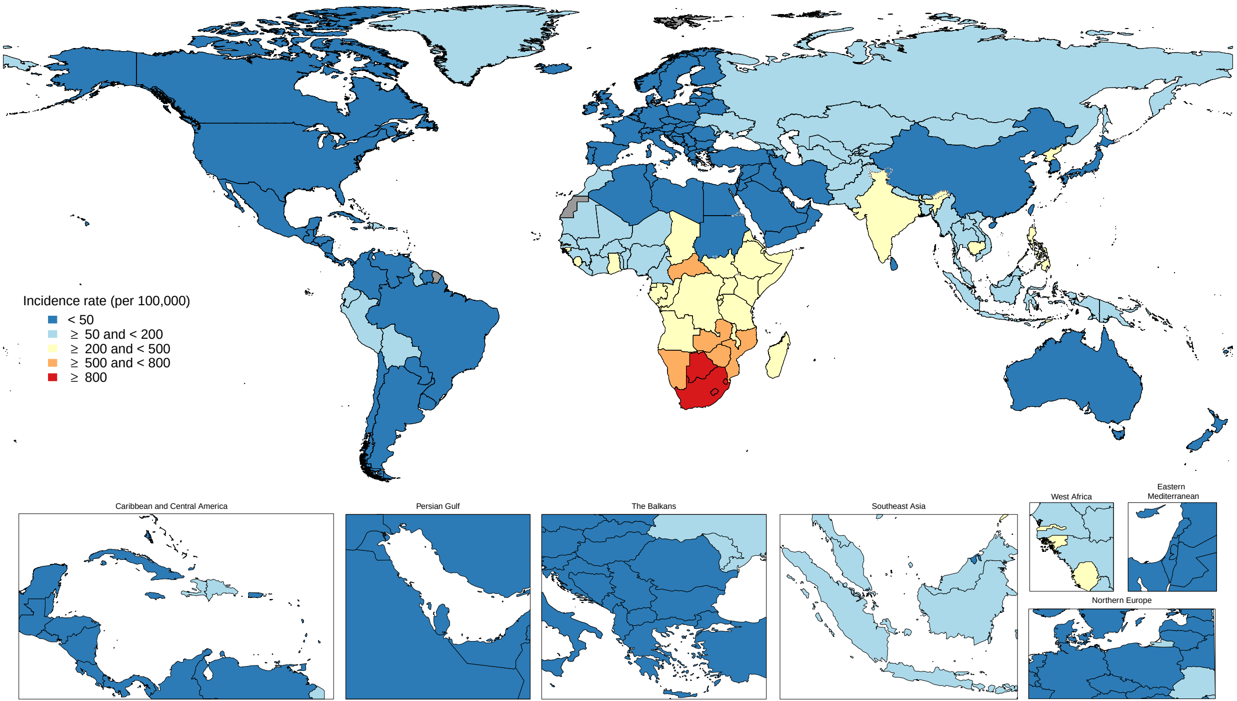

eFigure 5b. All−form TB mortality rates, 15−49 years, both sex, 2021

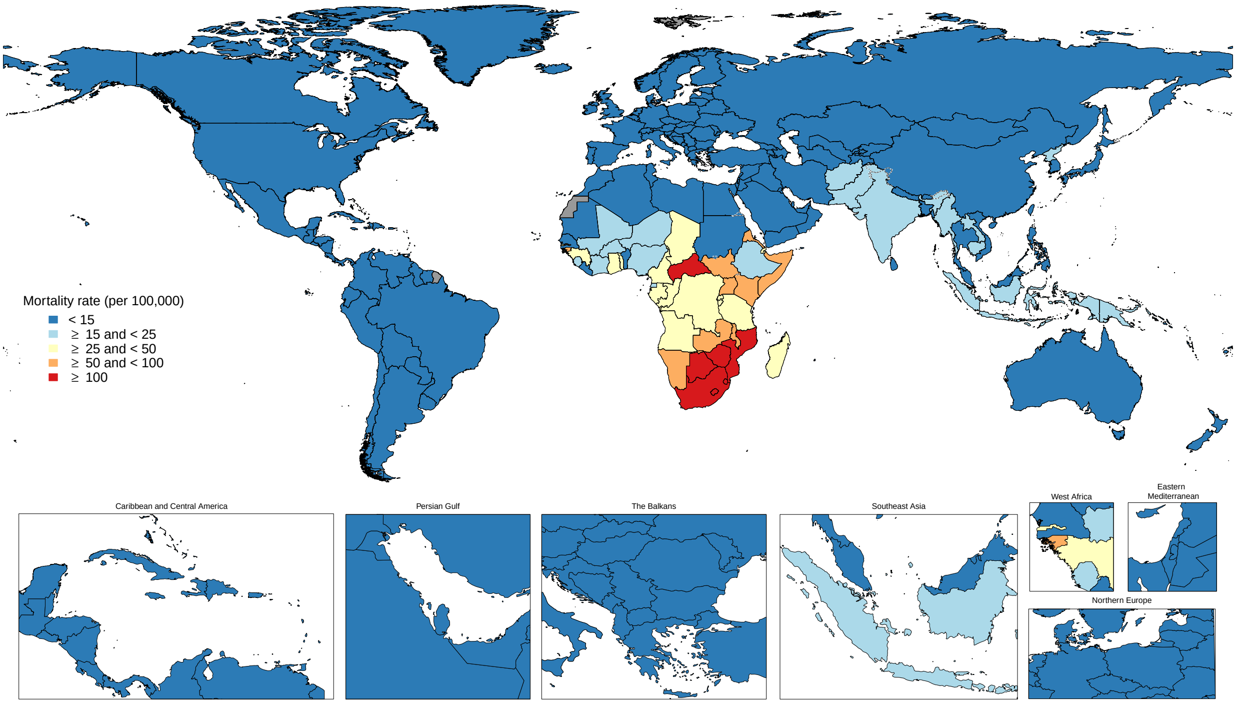

eFigure 6a. All−form TB incidence rates, 50−69 years, both sex, 2021

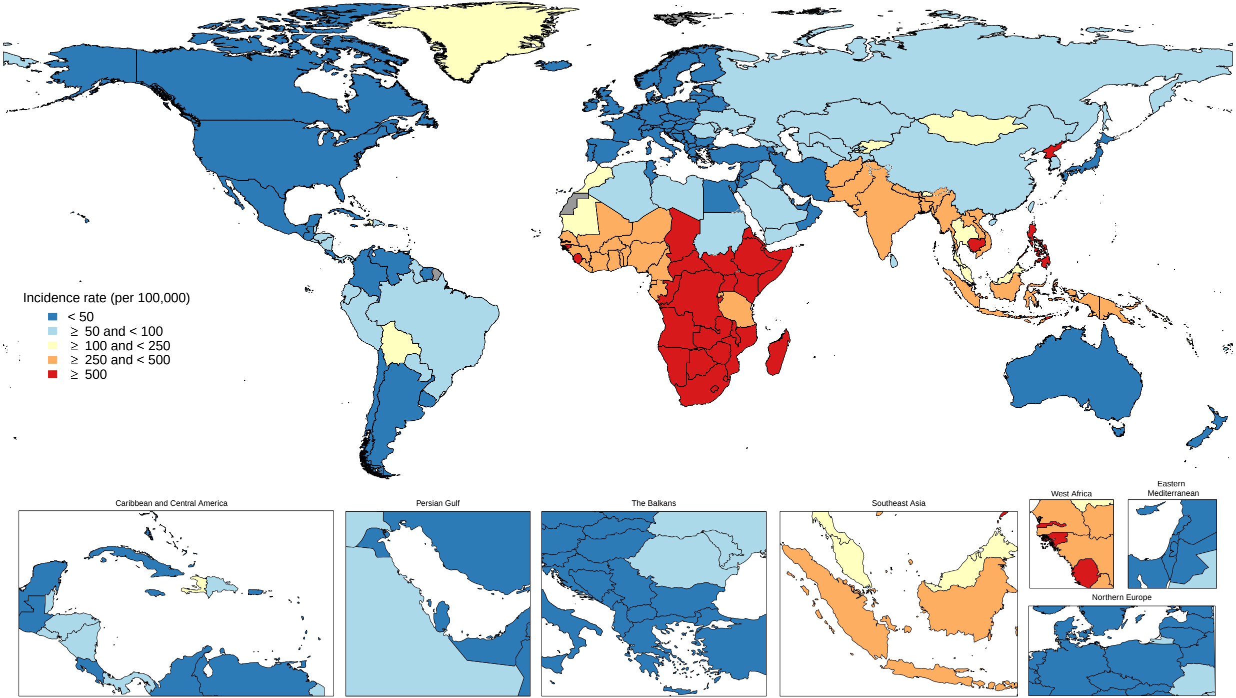

eFigure 6b. All−form TB mortality rates, 50−69 years, both sex, 2021

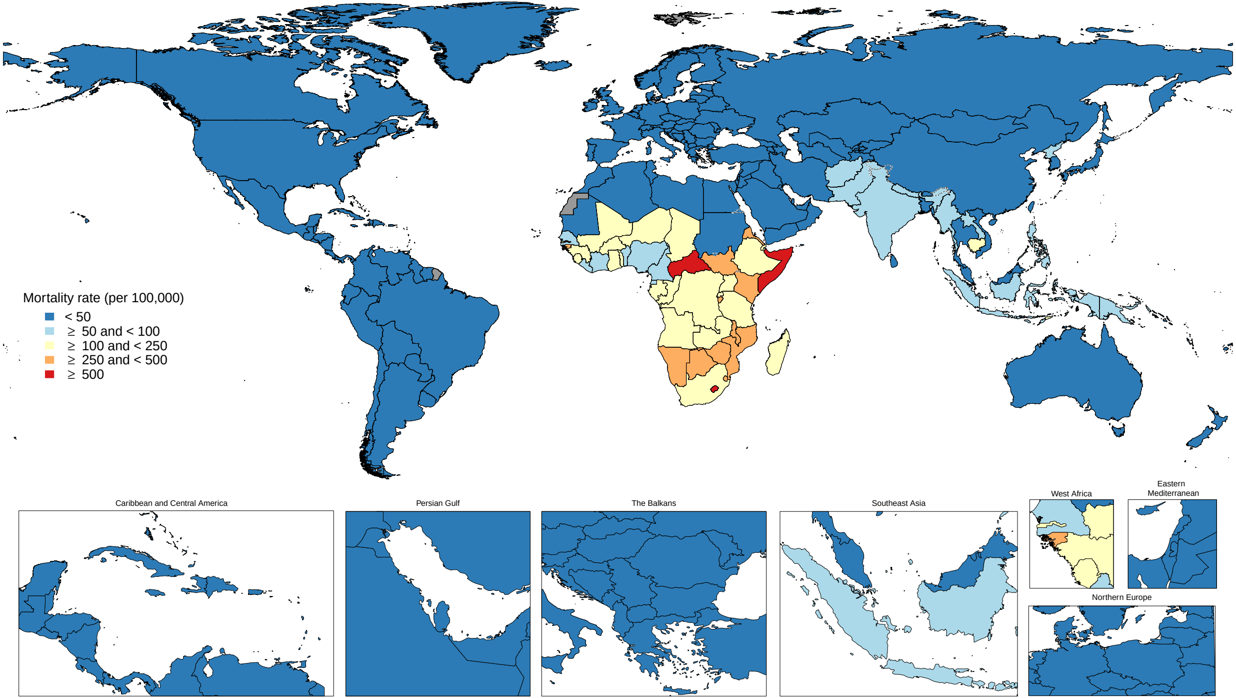

eFigure 7a. All−form TB incidence rates, 70+ years, both sex, 2021

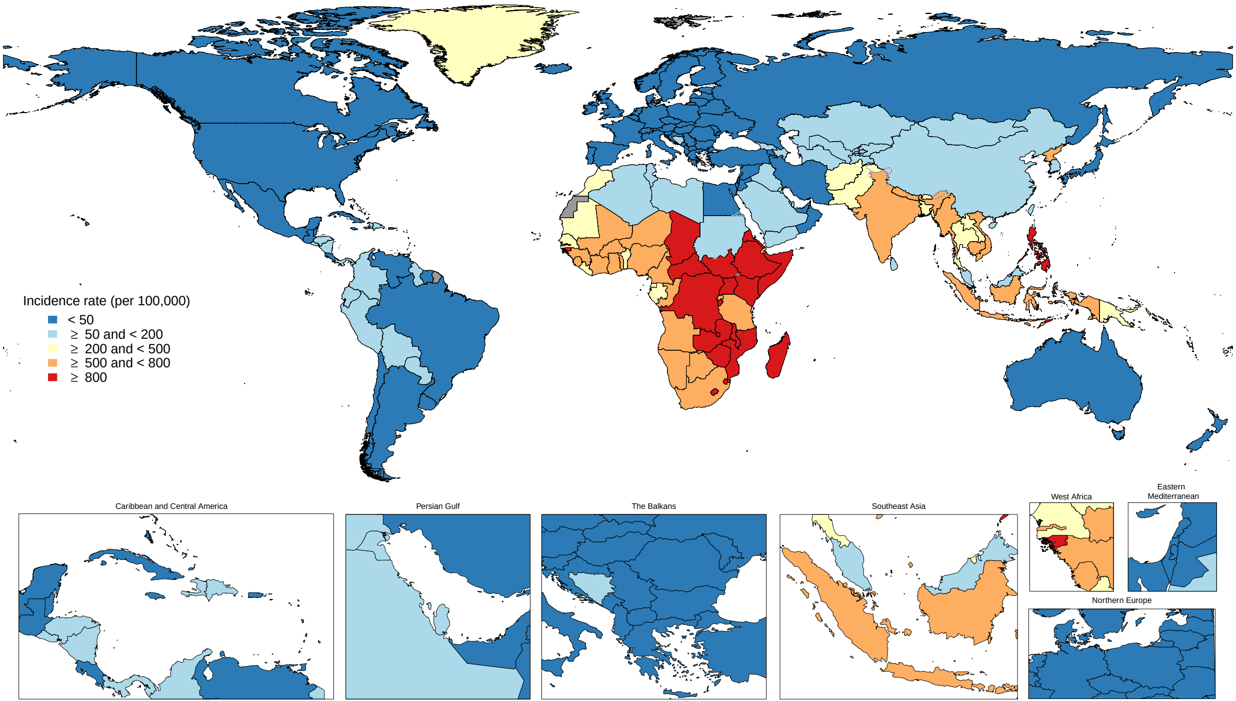

eFigure 7b. All−form TB mortality rates, 70+ years, both sex, 2021

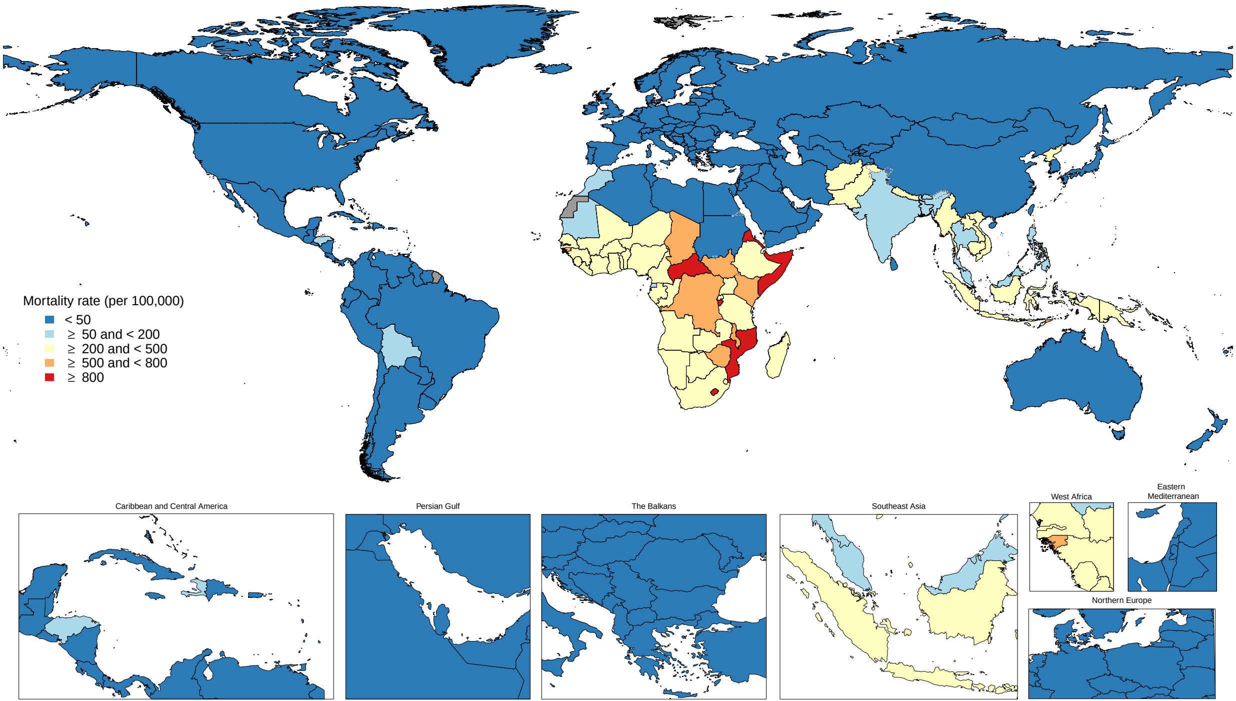

eFigure 8. Temporal trends for all−form tuberculosis incidence rate per 100,000 population from 1990 to 2021 for the top 20 high tuberculosis burden countries in the GBD

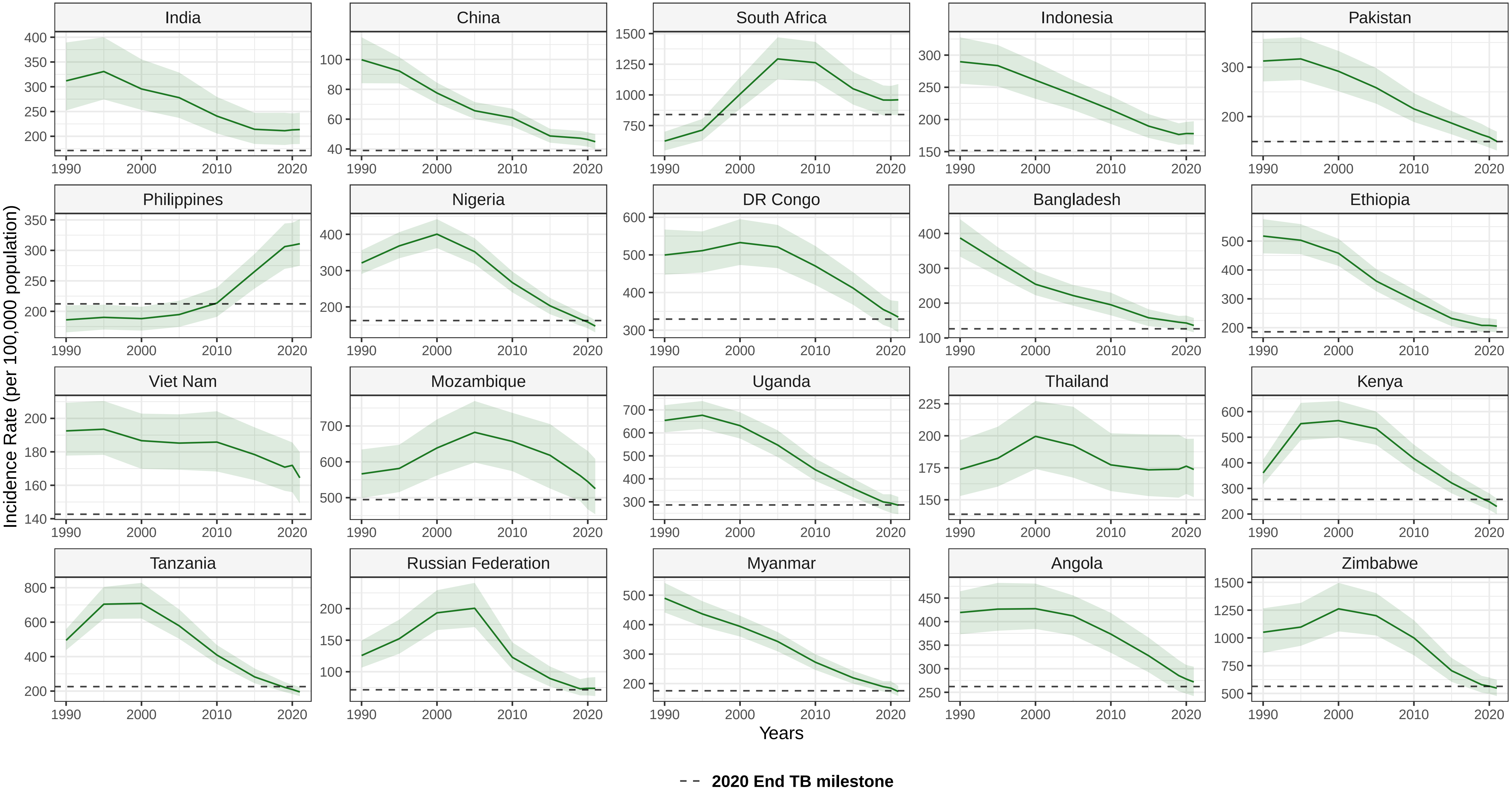

eFigure 9. Temporal trends for deaths due to all−form tuberculosis deaths from 1990 to 2021 for the top 20 high tuberculosis burden countries in the GBD

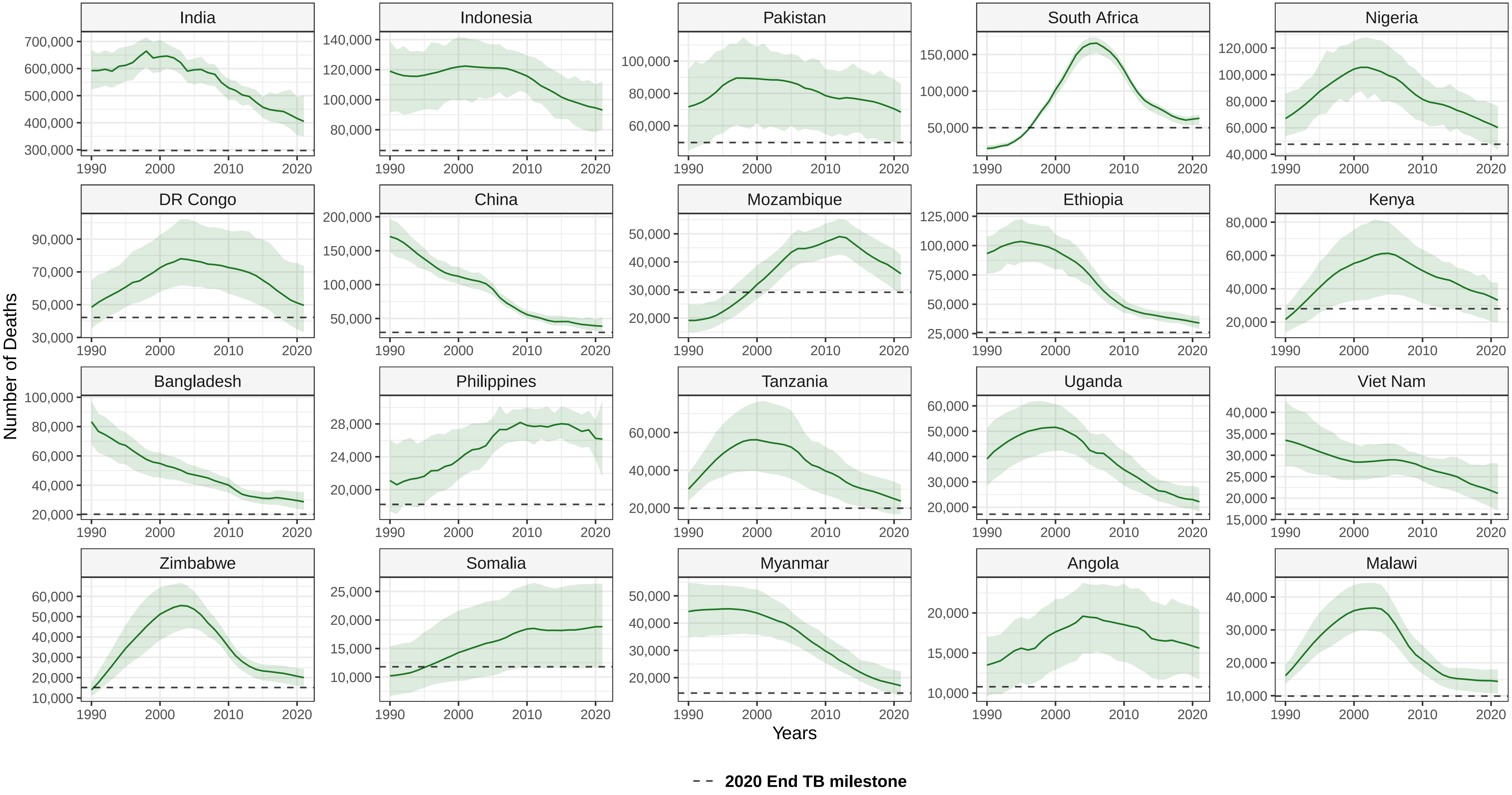

eFigure 10. Global age−sex−specific progress towards WHO interim milestones for (A) all−form tuberculosis incidence rate and (B) deaths due to all−form tuberculosis, 1990−2021.

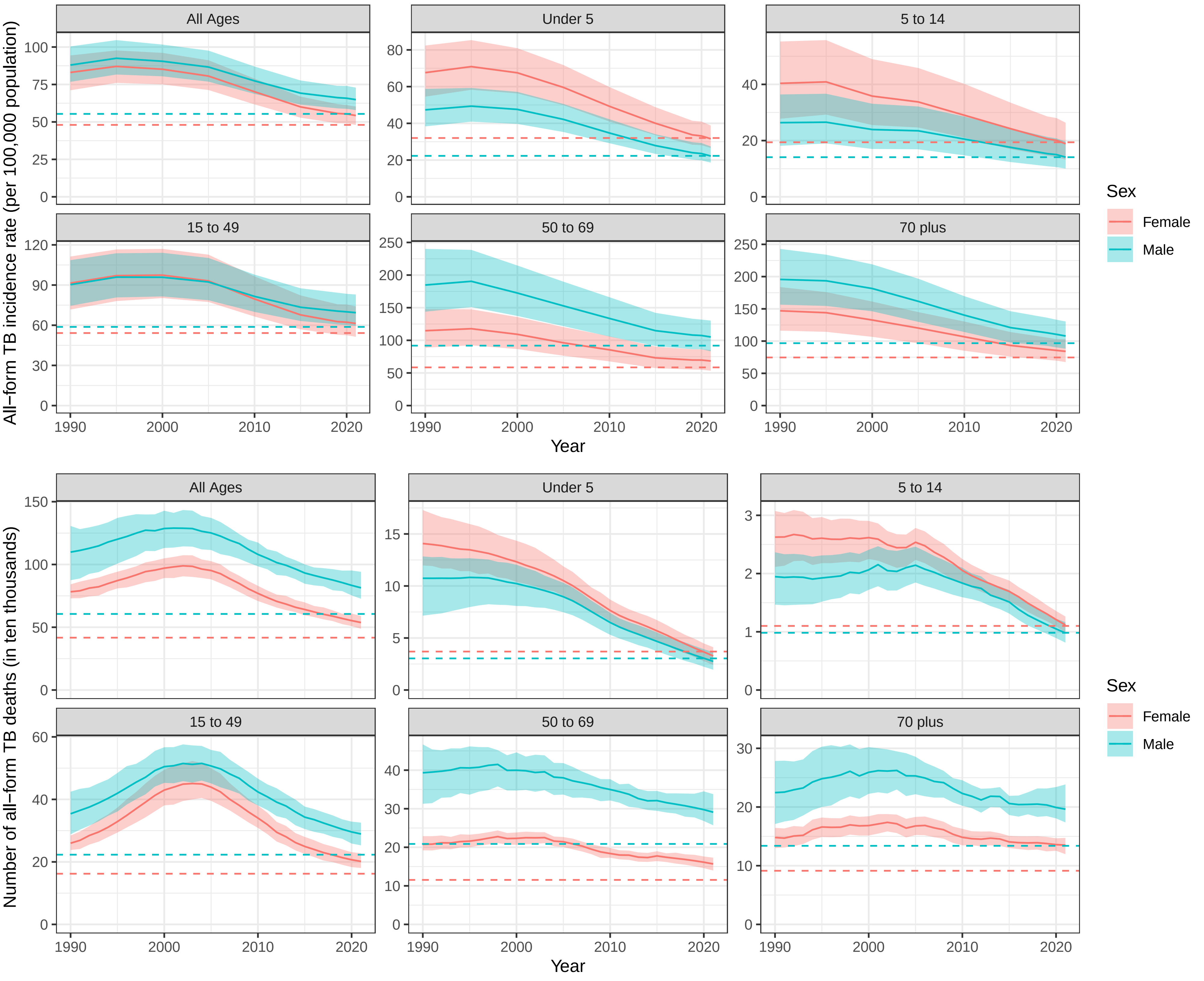

eFigure 11. Diagnostic plot for the quasi-Poisson regression model: Pearson residuals against fitted values

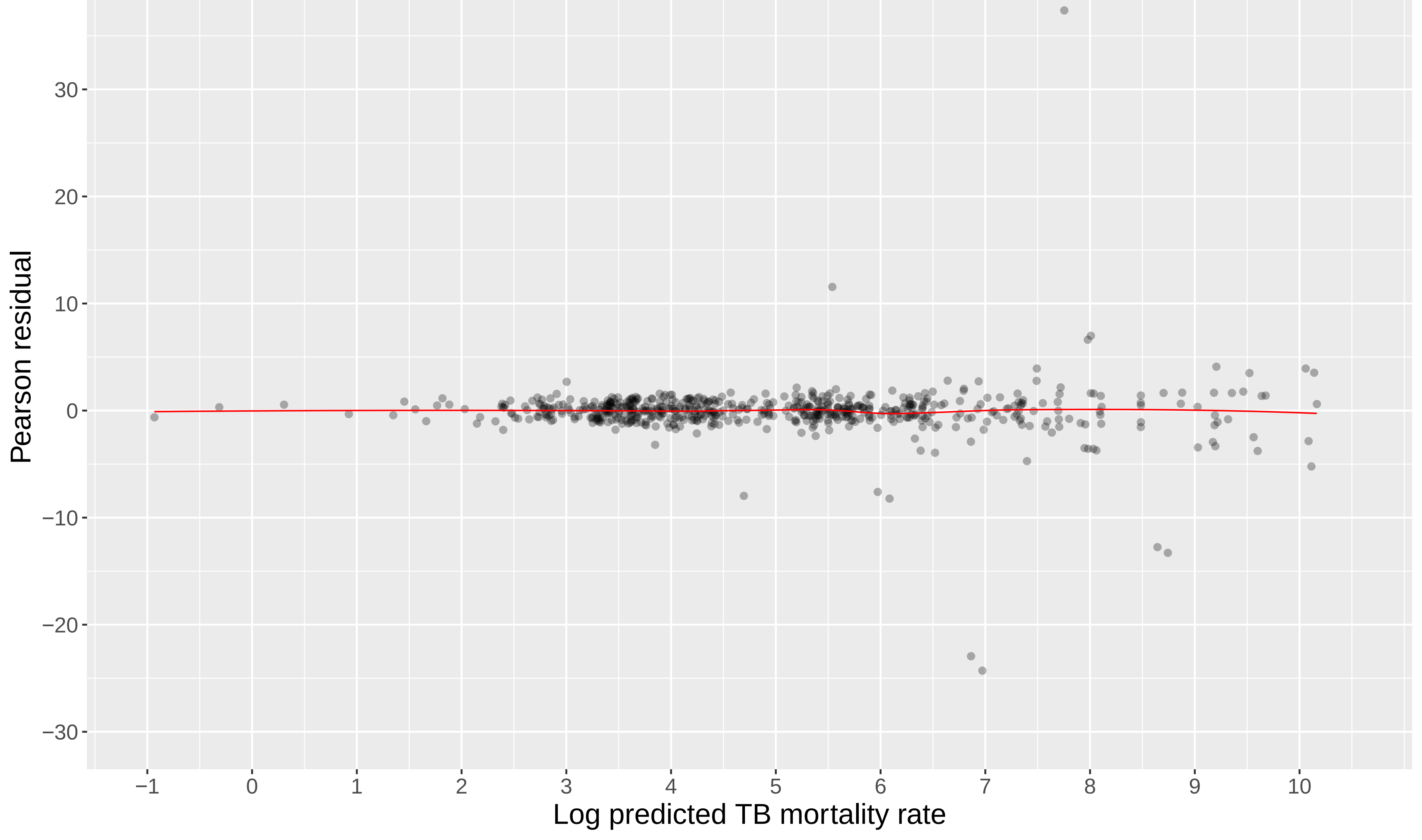

eTable 1. All-form tuberculosis incident cases and deaths, age-standardised rates of incidence and mortality per 100,000 population, and corresponding annualized rates of change by age groups for 204 countries and territories (2021).

Location	Age group	Number of cases	Rate per 100,000 cases	Annualized rate of change (1990-2010)	Annualized rate of change (2010-2021)	Number of deaths	Rate per 100,000 deaths	Annualized deaths (1990-2010)	Annualized deaths (2010-2021)
**Global**	**All Ages**	**9400000 (8360000 to 10500000)**	**115 (102 to 128)**	**-0·734 (-0·914 to -0·523)**	**-1·94 (-2·09 to -1·78)**	**1350000 (1230000 to 1520000)**	**16·2 (14·8 to 18·2)**	**-1·40 (-1·82 to -0·928)**	**-4·01 (-4·84 to -2·96)**
	**Under 5**	**354000 (298000 to 436000)**	**53·8 (45·3 to 66·3)**	**-1·56 (-1·91 to -1·29)**	**-4·05 (-4·32 to -3·77)**	**60300 (43500 to 76700)**	**9·16 (6·61 to 11·7)**	**-3·09 (-3·68 to -2·52)**	**-7·85 (-9·63 to -5·84)**
	**5-14 years**	**446000 (315000 to 623000)**	**33·0 (23·3 to 46·0)**	**-1·48 (-1·79 to -1·10)**	**-3·71 (-4·03 to -3·41)**	**20900 (18300 to 24000)**	**1·55 (1·36 to 1·78)**	**-1·22 (-1·80 to -0·547)**	**-6·60 (-7·71 to -5·57)**
	**15-49 years**	**5160000 (4380000 to 6180000)**	**131 (111 to 157)**	**-0·596 (-0·842 to -0·336)**	**-1·90 (-2·09 to -1·74)**	**491000 (439000 to 546000)**	**12·4 (11·1 to 13·8)**	**-0·400 (-0·873 to 0·0442)**	**-4·72 (-5·50 to -3·87)**
	**50-69 years**	**2490000 (1960000 to 3100000)**	**173 (137 to 216)**	**-1·56 (-1·81 to -1·28)**	**-2·12 (-2·33 to -1·96)**	**448000 (404000 to 510000)**	**31·2 (28·1 to 35·5)**	**-2·79 (-3·23 to -2·22)**	**-4·36 (-5·23 to -2·99)**
	**70+ years**	**949000 (770000 to 1150000)**	**192 (156 to 234)**	**-1·63 (-1·92 to -1·37)**	**-2·29 (-2·52 to -2·06)**	**331000 (302000 to 379000)**	**67·0 (61·1 to 76·6)**	**-2·88 (-3·41 to -2·25)**	**-3·96 (-4·80 to -2·63)**
Central Europe, Eastern Europe, and Central Asia	All Ages	219000 (188000 to 262000)	46·9 (40·3 to 56·2)	-0·0580 (-0·374 to 0·249)	-4·28 (-4·79 to -3·80)	15900 (14600 to 17300)	3·01 (2·77 to 3·29)	1·60 (1·40 to 1·78)	-8·65 (-9·32 to -8·08)
	Under 5	2260 (1860 to 2900)	8·78 (7·25 to 11·3)	-2·07 (-2·44 to -1·68)	-4·94 (-5·50 to -4·43)	334 (267 to 441)	1·30 (1·04 to 1·72)	-3·07 (-3·77 to -2·42)	-6·90 (-8·85 to -4·68)
	5-14 years	6020 (4140 to 8540)	10·9 (7·52 to 15·5)	-1·13 (-1·58 to -0·659)	-4·43 (-5·09 to -3·52)	66·2 (59·4 to 75·1)	0·120 (0·108 to 0·136)	-0·0316 (-0·505 to 0·385)	-7·86 (-8·68 to -6·90)
	15-49 years	140000 (113000 to 175000)	70·9 (57·3 to 88·6)	-0·0144 (-0·432 to 0·363)	-4·12 (-4·80 to -3·46)	6900 (6260 to 7800)	3·49 (3·17 to 3·94)	3·40 (3·19 to 3·67)	-9·40 (-10·0 to -8·89)
	50-69 years	55900 (42100 to 72900)	56·0 (42·2 to 73·1)	-0·807 (-1·27 to -0·285)	-3·77 (-4·39 to -3·10)	6410 (5850 to 7030)	6·42 (5·86 to 7·05)	0·238 (0·0892 to 0·388)	-8·83 (-9·67 to -7·94)
	70+ years	14300 (11200 to 18000)	36·0 (28·2 to 45·5)	-1·76 (-2·14 to -1·40)	-3·61 (-4·19 to -3·08)	2150 (2040 to 2270)	5·42 (5·16 to 5·73)	-2·68 (-2·81 to -2·53)	-5·32 (-5·74 to -4·90)
Central Asia	All Ages	51800 (45600 to 60100)	54·2 (47·8 to 61·9)	-0·216 (-0·557 to 0·117)	-4·28 (-4·75 to -3·78)	4430 (3960 to 4950)	4·69 (4·19 to 5·25)	-0·238 (-0·653 to 0·163)	-7·26 (-8·25 to -6·12)
	Under 5	1440 (1200 to 1840)	14·4 (12·0 to 18·4)	-2·43 (-2·80 to -2·02)	-4·82 (-5·54 to -4·01)	288 (222 to 390)	2·89 (2·22 to 3·90)	-3·79 (-4·68 to -2·95)	-7·83 (-10·2 to -5·40)
	5-14 years	2660 (1870 to 3650)	15·0 (10·6 to 20·7)	-0·629 (-1·23 to -0·0965)	-4·81 (-5·55 to -3·85)	48·8 (43·7 to 56·1)	0·276 (0·247 to 0·317)	-0·928 (-1·66 to -0·246)	-7·35 (-8·55 to -6·04)
	15-49 years	31900 (26600 to 39500)	65·5 (54·6 to 80·9)	-0·600 (-1·02 to -0·166)	-4·62 (-5·15 to -4·03)	2120 (1870 to 2400)	4·36 (3·84 to 4·92)	0·759 (0·375 to 1·16)	-8·03 (-9·05 to -7·05)
	50-69 years	12900 (9740 to 15900)	80·9 (60·9 to 99·6)	-0·329 (-0·823 to 0·108)	-3·30 (-3·92 to -2·42)	1570 (1370 to 1770)	9·81 (8·59 to 11·1)	-1·21 (-1·74 to -0·714)	-7·32 (-8·60 to -6·01)
	70+ years	2870 (2280 to 3600)	84·5 (67·0 to 106)	-0·774 (-1·23 to -0·278)	-1·60 (-2·33 to -1·01)	400 (358 to 449)	11·8 (10·5 to 13·2)	-0·735 (-1·42 to -0·103)	-4·74 (-5·81 to -3·68)
Armenia	All Ages	778 (675 to 915)	23·2 (20·0 to 27·3)	0·420 (-0·00532 to 0·926)	-4·99 (-5·73 to -4·22)	40·1 (35·5 to 45·3)	1·08 (0·955 to 1·21)	1·47 (0·871 to 2·15)	-10·7 (-11·8 to -9·54)
	Under 5	9·29 (7·25 to 12·2)	4·99 (3·90 to 6·54)	-2·49 (-3·12 to -1·72)	-5·98 (-7·61 to -4·48)	0·656 (0·519 to 0·846)	0·352 (0·279 to 0·454)	-3·99 (-4·93 to -3·03)	-10·1 (-11·8 to -7·40)

eTable 1. All-form tuberculosis incident cases and deaths, age-standardised rates of incidence and mortality per 100,000 population, and corresponding annualized rates of change by age groups for 204 countries and territories (2021).

Location	Age group	Number of cases	Rate per 100,000 cases	Annualized rate of change (1990-2010)	Annualized rate of change (2010-2021)	Number of deaths	Rate per 100,000 deaths	Annualized deaths (1990-2010)	Annualized deaths (2010-2021)
	5-14 years	18·9 (12·6 to 27·2)	4·66 (3·10 to 6·69)	-1·35 (-2·33 to -0·608)	-6·08 (-7·56 to -4·53)	0·128 (0·109 to 0·148)	0·0315 (0·0268 to 0·0363)	-1·81 (-2·90 to -0·649)	-10·9 (-12·6 to -9·40)
	15-49 years	432 (360 to 546)	29·7 (24·8 to 37·5)	0·0762 (-0·425 to 0·627)	-5·37 (-6·30 to -4·47)	16·5 (14·8 to 18·3)	1·13 (1·02 to 1·25)	1·06 (0·464 to 1·72)	-10·0 (-11·1 to -8·84)
	50-69 years	249 (182 to 324)	35·4 (26·0 to 46·1)	0·397 (-0·308 to 1·07)	-4·42 (-5·66 to -3·13)	14·4 (12·6 to 16·4)	2·04 (1·79 to 2·33)	0·109 (-0·497 to 0·757)	-12·8 (-14·1 to -11·6)
	70+ years	68·2 (52·3 to 86·0)	27·9 (21·4 to 35·2)	-2·42 (-3·19 to -1·56)	-3·71 (-5·22 to -2·67)	8·51 (7·41 to 9·57)	3·48 (3·03 to 3·91)	1·81 (1·11 to 2·72)	-9·33 (-10·7 to -8·05)
Azerbaijan	All Ages	5800 (4930 to 7090)	51·2 (43·8 to 61·8)	-0·922 (-1·40 to -0·442)	-2·38 (-3·18 to -1·48)	416 (307 to 612)	3·79 (2·83 to 5·50)	-1·48 (-2·61 to -0·0746)	-7·68 (-10·3 to -4·96)
	Under 5	94·3 (72·7 to 123)	13·1 (10·1 to 17·1)	-3·26 (-4·00 to -2·39)	-4·61 (-5·84 to -2·94)	19·4 (12·9 to 28·5)	2·69 (1·78 to 3·95)	-4·99 (-6·90 to -3·33)	-8·22 (-11·3 to -5·01)
	5-14 years	216 (151 to 296)	13·1 (9·23 to 18·0)	-1·65 (-2·43 to -0·776)	-3·45 (-4·93 to -1·97)	5·47 (4·15 to 6·96)	0·333 (0·253 to 0·424)	-1·24 (-3·87 to 1·04)	-6·84 (-9·33 to -4·05)
	15-49 years	3890 (3100 to 4870)	69·8 (55·6 to 87·5)	-1·24 (-1·86 to -0·648)	-2·16 (-3·00 to -1·03)	228 (159 to 337)	4·09 (2·85 to 6·04)	-0·772 (-2·17 to 0·292)	-7·12 (-9·71 to -4·95)
	50-69 years	1390 (1040 to 1770)	63·6 (47·4 to 80·9)	-1·33 (-2·03 to -0·705)	-2·99 (-4·24 to -1·69)	121 (79·5 to 190)	5·53 (3·64 to 8·69)	-2·41 (-4·25 to -0·566)	-10·8 (-14·3 to -5·91)
	70+ years	210 (165 to 263)	54·9 (43·1 to 68·6)	-1·31 (-1·95 to -0·628)	-2·35 (-3·30 to -1·04)	43·1 (31·1 to 62·0)	11·2 (8·11 to 16·2)	-0·945 (-2·61 to 0·760)	-5·57 (-8·42 to -2·59)
Georgia	All Ages	2060 (1810 to 2410)	52·5 (45·3 to 62·0)	1·69 (1·33 to 2·06)	-3·34 (-4·01 to -2·58)	114 (99·9 to 131)	2·34 (2·07 to 2·66)	-0·239 (-0·853 to 0·350)	-7·68 (-9·06 to -6·31)
	Under 5	21·0 (16·7 to 28·0)	8·64 (6·87 to 11·5)	-1·71 (-2·42 to -0·828)	-5·35 (-6·69 to -4·19)	0·955 (0·766 to 1·21)	0·392 (0·315 to 0·497)	-4·81 (-5·84 to -3·68)	-11·0 (-13·0 to -8·81)
	5-14 years	61·9 (42·9 to 90·3)	12·6 (8·71 to 18·3)	2·55 (1·67 to 3·44)	-6·95 (-8·05 to -5·65)	0·216 (0·189 to 0·243)	0·0439 (0·0384 to 0·0494)	-1·93 (-3·20 to -0·512)	-13·2 (-14·9 to -11·1)
	15-49 years	1170 (938 to 1520)	73·4 (58·6 to 94·8)	1·96 (1·50 to 2·47)	-3·55 (-4·55 to -2·47)	36·7 (32·8 to 40·9)	2·30 (2·05 to 2·56)	-0·332 (-0·883 to 0·0549)	-10·2 (-11·3 to -8·96)
	50-69 years	610 (470 to 774)	67·9 (52·4 to 86·1)	0·811 (0·305 to 1·36)	-1·93 (-3·08 to -0·865)	48·6 (40·9 to 57·7)	5·41 (4·55 to 6·42)	-1·31 (-2·55 to -0·154)	-6·96 (-8·80 to -5·28)
	70+ years	195 (156 to 239)	52·3 (41·9 to 64·1)	-0·480 (-1·10 to 0·365)	-1·63 (-2·65 to -0·370)	27·8 (23·6 to 31·8)	7·44 (6·34 to 8·54)	0·744 (-0·379 to 1·91)	-4·32 (-5·92 to -2·81)
Kazakhstan	All Ages	10300 (8910 to 12100)	53·6 (46·3 to 62·3)	0·0564 (-0·430 to 0·474)	-7·00 (-7·82 to -6·14)	571 (501 to 635)	2·94 (2·60 to 3·27)	0·344 (-0·192 to 0·911)	-12·4 (-13·7 to -11·1)
	Under 5	170 (134 to 229)	8·75 (6·88 to 11·8)	-2·30 (-3·01 to -1·67)	-9·08 (-10·5 to -7·96)	16·2 (12·8 to 19·7)	0·830 (0·658 to 1·01)	-3·57 (-4·85 to -2·19)	-14·1 (-16·2 to -12·2)
	5-14 years	313 (210 to 440)	9·00 (6·04 to 12·7)	-0·896 (-1·86 to -0·00202)	-10·6 (-11·9 to -9·10)	3·13 (2·79 to 3·65)	0·0899 (0·0801 to 0·105)	-1·55 (-2·96 to -0·296)	-13·3 (-14·9 to -11·4)
	15-49 years	6680 (5440 to 8440)	71·4 (58·1 to 90·2)	0·0530 (-0·580 to 0·690)	-7·03 (-7·95 to -6·10)	275 (243 to 302)	2·94 (2·59 to 3·23)	1·58 (0·752 to 2·41)	-13·8 (-15·2 to -12·5)
	50-69 years	2520 (1900 to 3240)	75·1 (56·5 to 96·3)	-0·883 (-1·56 to -0·222)	-5·00 (-5·94 to -3·76)	200 (169 to 230)	5·95 (5·03 to 6·84)	-1·62 (-2·03 to -1·09)	-11·4 (-13·1 to -9·90)
	70+ years	599 (460 to 754)	73·6 (56·6 to 92·7)	-2·04 (-2·73 to -1·25)	-2·32 (-3·55 to -1·08)	76·9 (68·4 to 85·7)	9·45 (8·42 to 10·5)	-2·58 (-3·26 to -1·80)	-3·53 (-4·79 to -2·13)

eTable 1. All-form tuberculosis incident cases and deaths, age-standardised rates of incidence and mortality per 100,000 population, and corresponding annualized rates of change by age groups for 204 countries and territories (2021).

Location	Age group	Number of cases	Rate per 100,000 cases	Annualized rate of change (1990-2010)	Annualized rate of change (2010-2021)	Number of deaths	Rate per 100,000 deaths	Annualized deaths (1990-2010)	Annualized deaths (2010-2021)
Kyrgyzstan	All Ages	5330 (4630 to 6140)	83·5 (73·0 to 96·8)	0·619 (0·214 to 1·01)	-2·93 (-3·69 to -2·24)	347 (297 to 412)	5·68 (4·86 to 6·76)	0·929 (0·377 to 1·36)	-7·34 (-9·02 to -5·70)
	Under 5	109 (88·2 to 142)	13·8 (11·1 to 17·9)	-1·76 (-2·54 to -0·941)	-5·45 (-6·88 to -4·07)	11·3 (9·67 to 13·2)	1·42 (1·22 to 1·66)	-4·43 (-5·29 to -3·37)	-9·71 (-11·4 to -7·82)
	5-14 years	286 (198 to 398)	19·3 (13·4 to 26·9)	1·50 (0·734 to 2·28)	-3·24 (-4·61 to -2·04)	2·74 (2·35 to 3·17)	0·185 (0·159 to 0·214)	-3·26 (-4·34 to -1·84)	-6·63 (-8·42 to -5·13)
	15-49 years	3410 (2770 to 4240)	99·1 (80·4 to 123)	0·132 (-0·362 to 0·587)	-3·21 (-3·99 to -2·37)	183 (158 to 216)	5·31 (4·59 to 6·29)	1·98 (1·16 to 2·73)	-7·83 (-9·39 to -6·17)
	50-69 years	1250 (927 to 1620)	131 (96·7 to 168)	0·765 (0·105 to 1·44)	-1·44 (-2·53 to -0·190)	127 (107 to 154)	13·3 (11·1 to 16·1)	0·501 (-0·0913 to 1·10)	-6·93 (-8·88 to -5·03)
	70+ years	276 (214 to 344)	144 (112 to 179)	0·337 (-0·339 to 0·987)	-0·0469 (-1·19 to 0·964)	22·7 (19·0 to 27·6)	11·8 (9·91 to 14·4)	0·646 (0·126 to 1·11)	-6·23 (-8·20 to -4·34)
Mongolia	All Ages	3270 (2850 to 3840)	100 (87·9 to 116)	-0·546 (-0·966 to -0·0212)	-4·69 (-5·31 to -4·02)	379 (264 to 559)	12·2 (8·65 to 17·4)	-1·50 (-3·51 to -0·00601)	-7·26 (-10·4 to -4·47)
	Under 5	100 (82·4 to 124)	25·7 (21·1 to 31·7)	-4·51 (-5·26 to -3·81)	-5·52 (-6·73 to -4·29)	13·9 (9·58 to 20·6)	3·57 (2·45 to 5·28)	-7·76 (-9·56 to -5·84)	-11·5 (-15·1 to -8·11)
	5-14 years	130 (89·5 to 182)	18·7 (12·9 to 26·1)	-2·82 (-3·99 to -1·91)	-6·22 (-7·65 to -4·73)	4·23 (2·98 to 5·81)	0·607 (0·428 to 0·836)	-2·40 (-4·33 to -0·145)	-7·93 (-10·9 to -4·54)
	15-49 years	2220 (1840 to 2740)	132 (109 to 162)	-0·790 (-1·28 to -0·220)	-4·19 (-5·18 to -3·38)	215 (149 to 344)	12·8 (8·85 to 20·4)	-0·00512 (-2·60 to 2·07)	-6·72 (-9·60 to -4·06)
	50-69 years	727 (541 to 927)	152 (113 to 194)	-1·10 (-1·86 to -0·532)	-4·27 (-5·45 to -2·87)	123 (80·4 to 172)	25·7 (16·8 to 36·0)	-2·75 (-5·12 to -0·730)	-8·32 (-11·8 to -5·24)
	70+ years	90·4 (71·2 to 113)	107 (84·6 to 134)	-2·15 (-2·95 to -1·28)	-4·39 (-5·36 to -3·44)	22·1 (16·2 to 29·0)	26·3 (19·2 to 34·5)	-2·72 (-5·19 to -0·696)	-5·73 (-8·88 to -2·60)
Tajikistan	All Ages	5200 (4390 to 6150)	55·8 (47·8 to 65·3)	-1·48 (-2·00 to -1·06)	-3·96 (-4·72 to -3·23)	756 (563 to 970)	9·03 (6·62 to 11·5)	-1·92 (-3·15 to -0·680)	-4·67 (-6·99 to -2·38)
	Under 5	337 (268 to 438)	25·2 (20·0 to 32·7)	-2·59 (-3·25 to -1·85)	-3·50 (-4·77 to -2·05)	124 (78·1 to 190)	9·29 (5·84 to 14·2)	-3·99 (-6·08 to -1·75)	-4·70 (-8·02 to -0·0662)
	5-14 years	294 (197 to 417)	13·1 (8·76 to 18·6)	-3·06 (-3·82 to -2·26)	-4·23 (-5·58 to -2·62)	12·1 (8·44 to 16·8)	0·538 (0·376 to 0·750)	-2·76 (-4·38 to -1·09)	-4·79 (-7·68 to -2·53)
	15-49 years	3390 (2780 to 4250)	66·0 (54·1 to 82·7)	-1·85 (-2·41 to -1·36)	-4·25 (-5·16 to -3·39)	327 (231 to 437)	6·35 (4·49 to 8·49)	-1·25 (-2·97 to 0·397)	-5·32 (-8·11 to -2·87)
	50-69 years	997 (748 to 1290)	80·7 (60·5 to 104)	-0·899 (-1·43 to -0·332)	-3·94 (-4·87 to -2·68)	241 (171 to 329)	19·5 (13·8 to 26·6)	-1·34 (-2·93 to 0·461)	-5·22 (-7·76 to -2·70)
	70+ years	175 (139 to 222)	87·8 (70·0 to 111)	-0·259 (-0·876 to 0·345)	-2·88 (-4·00 to -1·83)	51·6 (33·9 to 71·5)	25·9 (17·0 to 35·9)	1·10 (-0·385 to 2·75)	-6·37 (-9·89 to -3·88)
Turkmenistan	All Ages	2510 (2110 to 3000)	48·7 (41·3 to 57·8)	-0·201 (-0·591 to 0·236)	-2·99 (-3·91 to -2·06)	388 (300 to 492)	7·71 (5·98 to 9·77)	0·618 (0·351 to 0·943)	-4·89 (-6·83 to -2·72)
	Under 5	73·9 (60·7 to 93·3)	13·7 (11·2 to 17·3)	-3·03 (-3·76 to -2·36)	-2·88 (-4·41 to -1·31)	19·1 (15·5 to 23·6)	3·53 (2·87 to 4·37)	-4·05 (-4·95 to -3·06)	-7·06 (-8·76 to -5·23)
	5-14 years	110 (72·9 to 163)	11·2 (7·40 to 16·6)	-2·18 (-2·79 to -1·48)	0·733 (-0·875 to 2·47)	3·02 (2·58 to 3·51)	0·307 (0·262 to 0·357)	-1·74 (-3·06 to -0·660)	-6·75 (-8·33 to -4·89)
	15-49 years	1720 (1350 to 2140)	64·3 (50·4 to 79·8)	-0·293 (-0·731 to 0·218)	-3·36 (-4·53 to -2·27)	232 (179 to 296)	8·65 (6·70 to 11·1)	2·13 (1·54 to 2·60)	-5·11 (-7·03 to -2·74)

eTable 1. All-form tuberculosis incident cases and deaths, age-standardised rates of incidence and mortality per 100,000 population, and corresponding annualized rates of change by age groups for 204 countries and territories (2021).

Location	Age group	Number of cases	Rate per 100,000 cases	Annualized rate of change (1990-2010)	Annualized rate of change (2010-2021)	Number of deaths	Rate per 100,000 deaths	Annualized deaths (1990-2010)	Annualized deaths (2010-2021)
	50-69 years	512 (373 to 663)	63·9 (46·6 to 82·8)	-1·14 (-1·76 to -0·530)	-3·58 (-4·82 to -2·41)	115 (85·8 to 149)	14·3 (10·7 to 18·6)	-0·992 (-1·34 to -0·618)	-5·64 (-8·10 to -3·29)
	70+ years	89·3 (68·9 to 115)	57·0 (44·0 to 73·2)	-1·86 (-2·57 to -1·25)	-2·87 (-3·84 to -1·78)	19·6 (16·0 to 23·8)	12·5 (10·2 to 15·2)	-0·408 (-0·805 to 0·0234)	-5·77 (-7·79 to -3·92)
Uzbekistan	All Ages	16600 (14400 to 18900)	51·0 (44·3 to 57·4)	-0·298 (-0·817 to 0·115)	-3·70 (-4·52 to -2·90)	1420 (1230 to 1670)	4·45 (3·85 to 5·26)	0·0970 (-0·972 to 0·727)	-6·26 (-7·82 to -3·99)
	Under 5	527 (432 to 691)	13·7 (11·3 to 18·0)	-2·23 (-2·95 to -1·42)	-4·18 (-5·34 to -2·83)	82·7 (65·5 to 105)	2·16 (1·71 to 2·74)	-2·78 (-4·56 to -1·66)	-9·41 (-11·9 to -6·55)
	5-14 years	1230 (888 to 1650)	19·7 (14·2 to 26·3)	0·286 (-0·706 to 1·16)	-3·71 (-5·01 to -2·22)	17·7 (15·0 to 20·7)	0·284 (0·240 to 0·330)	0·621 (-0·645 to 1·68)	-7·46 (-9·36 to -5·32)
	15-49 years	9030 (7490 to 11400)	50·6 (42·0 to 63·8)	-1·28 (-1·97 to -0·772)	-4·52 (-5·41 to -3·56)	613 (538 to 724)	3·44 (3·02 to 4·06)	1·06 (0·0628 to 1·72)	-7·43 (-9·05 to -5·07)
	50-69 years	4670 (3460 to 5750)	87·2 (64·6 to 107)	0·0671 (-0·697 to 0·755)	-2·90 (-3·93 to -1·77)	577 (485 to 689)	10·8 (9·05 to 12·9)	-1·24 (-2·42 to -0·483)	-6·24 (-8·10 to -3·41)
	70+ years	1170 (902 to 1500)	123 (94·6 to 158)	0·829 (0·161 to 1·60)	-1·32 (-2·64 to -0·0565)	128 (109 to 149)	13·5 (11·5 to 15·6)	-0·232 (-1·42 to 0·680)	-4·45 (-6·38 to -1·93)
Central Europe	All Ages	20400 (17500 to 23500)	14·0 (12·0 to 16·2)	-1·97 (-2·31 to -1·63)	-3·49 (-3·80 to -3·05)	1860 (1700 to 2020)	1·01 (0·915 to 1·10)	-2·97 (-3·20 to -2·75)	-5·51 (-6·29 to -4·80)
	Under 5	195 (158 to 249)	3·50 (2·82 to 4·47)	-4·38 (-4·99 to -3·75)	-3·08 (-3·79 to -2·27)	13·1 (11·1 to 15·1)	0·235 (0·198 to 0·270)	-7·76 (-8·09 to -7·36)	-4·36 (-5·75 to -2·64)
	5-14 years	489 (325 to 703)	4·03 (2·68 to 5·80)	-2·83 (-3·36 to -2·34)	-3·91 (-4·74 to -3·21)	4·11 (3·13 to 5·25)	0·0339 (0·0258 to 0·0434)	-3·40 (-3·88 to -2·97)	-7·92 (-8·77 to -6·82)
	15-49 years	8540 (6920 to 10600)	16·2 (13·1 to 20·1)	-2·11 (-2·55 to -1·55)	-3·93 (-4·45 to -3·40)	409 (364 to 452)	0·776 (0·692 to 0·858)	-2·83 (-3·17 to -2·45)	-6·67 (-7·57 to -5·88)
	50-69 years	7550 (5550 to 9640)	25·1 (18·5 to 32·1)	-2·55 (-3·11 to -1·94)	-3·07 (-3·58 to -2·47)	766 (694 to 836)	2·55 (2·31 to 2·78)	-3·87 (-4·20 to -3·57)	-6·48 (-7·38 to -5·59)
	70+ years	3580 (2880 to 4430)	24·1 (19·4 to 29·9)	-3·53 (-3·90 to -3·15)	-4·20 (-4·66 to -3·65)	672 (618 to 726)	4·53 (4·16 to 4·89)	-5·34 (-5·61 to -5·07)	-5·34 (-5·94 to -4·75)
Albania	All Ages	343 (299 to 390)	10·5 (9·22 to 12·0)	-1·87 (-2·29 to -1·36)	-1·52 (-2·05 to -1·02)	33·0 (22·7 to 50·5)	0·859 (0·594 to 1·29)	-4·11 (-5·93 to -1·71)	-1·49 (-5·29 to 2·40)
	Under 5	5·32 (4·15 to 7·04)	3·73 (2·91 to 4·93)	-4·48 (-5·38 to -3·59)	-2·92 (-4·48 to -1·35)	0·505 (0·389 to 0·730)	0·354 (0·273 to 0·512)	-9·20 (-11·0 to -7·12)	-3·95 (-6·99 to -0·157)
	5-14 years	10·1 (6·86 to 14·1)	3·36 (2·28 to 4·67)	-2·34 (-3·18 to -1·28)	-3·03 (-4·44 to -1·56)	0·0988 (0·0751 to 0·134)	0·0328 (0·0249 to 0·0446)	-4·20 (-5·57 to -2·98)	-9·39 (-11·8 to -6·05)
	15-49 years	131 (107 to 166)	10·4 (8·51 to 13·2)	-2·44 (-3·04 to -1·64)	-2·77 (-3·32 to -2·15)	5·16 (3·39 to 7·99)	0·410 (0·270 to 0·635)	-4·05 (-5·69 to -1·68)	-4·78 (-8·34 to -1·10)
	50-69 years	123 (92·6 to 159)	17·7 (13·3 to 22·9)	-3·19 (-3·87 to -2·47)	-2·21 (-3·17 to -1·21)	8·56 (5·26 to 14·2)	1·23 (0·758 to 2·04)	-7·12 (-9·19 to -4·37)	-5·78 (-9·77 to -1·32)
	70+ years	73·4 (57·9 to 92·9)	27·0 (21·4 to 34·3)	-3·84 (-4·48 to -3·15)	-1·85 (-2·83 to -0·650)	18·7 (12·4 to 28·9)	6·89 (4·57 to 10·7)	-6·68 (-8·46 to -3·99)	-1·56 (-5·70 to 2·42)
Bosnia and Herzegovina	All Ages	841 (734 to 970)	18·5 (16·2 to 21·1)	-1·98 (-2·47 to -1·59)	-1·83 (-2·36 to -1·26)	111 (88·7 to 134)	1·91 (1·53 to 2·30)	-2·75 (-3·88 to -1·67)	-3·90 (-6·28 to -1·29)
	Under 5	5·79 (4·58 to 7·39)	3·83 (3·03 to 4·90)	-4·15 (-4·98 to -3·21)	-2·59 (-3·86 to -1·46)	0·279 (0·212 to 0·373)	0·185 (0·140 to 0·247)	-6·06 (-7·94 to -4·05)	-4·04 (-7·43 to -0·258)

eTable 1. All-form tuberculosis incident cases and deaths, age-standardised rates of incidence and mortality per 100,000 population, and corresponding annualized rates of change by age groups for 204 countries and territories (2021).

Location	Age group	Number of cases	Rate per 100,000 cases	Annualized rate of change (1990-2010)	Annualized rate of change (2010-2021)	Number of deaths	Rate per 100,000 deaths	Annualized deaths (1990-2010)	Annualized deaths (2010-2021)
	5-14 years	14·4 (9·52 to 20·4)	4·24 (2·80 to 6·00)	-3·09 (-3·94 to -2·02)	-2·35 (-3·85 to -0·803)	0·262 (0·206 to 0·330)	0·0772 (0·0608 to 0·0972)	-4·55 (-6·35 to -3·08)	-2·52 (-5·23 to -0·0758)
	15-49 years	271 (225 to 341)	18·2 (15·1 to 22·9)	-2·85 (-3·45 to -2·22)	-2·48 (-3·12 to -1·77)	11·7 (8·37 to 15·2)	0·783 (0·562 to 1·02)	-4·33 (-5·65 to -2·90)	-6·45 (-9·72 to -3·28)
	50-69 years	335 (259 to 422)	35·8 (27·7 to 45·1)	-2·88 (-3·49 to -2·12)	-2·36 (-3·19 to -1·37)	34·9 (27·1 to 45·7)	3·73 (2·90 to 4·89)	-5·05 (-6·36 to -3·76)	-6·25 (-9·29 to -3·13)
	70+ years	215 (174 to 262)	55·2 (44·7 to 67·4)	-3·79 (-4·38 to -3·13)	-2·47 (-3·43 to -1·60)	63·5 (51·8 to 75·5)	16·3 (13·3 to 19·4)	-5·74 (-6·92 to -4·59)	-4·00 (-6·58 to -1·11)
Bulgaria	All Ages	1090 (942 to 1260)	13·3 (11·6 to 15·5)	-0·354 (-0·709 to 0·0556)	-4·20 (-4·87 to -3·57)	83·6 (74·7 to 93·8)	0·686 (0·613 to 0·770)	-1·67 (-2·19 to -1·08)	-6·14 (-7·31 to -5·07)
	Under 5	13·8 (10·3 to 18·1)	4·56 (3·39 to 5·96)	-0·953 (-1·70 to -0·180)	-3·50 (-5·30 to -1·77)	0·228 (0·185 to 0·270)	0·0754 (0·0612 to 0·0891)	-5·58 (-6·04 to -5·16)	-11·0 (-13·0 to -9·25)
	5-14 years	45·9 (30·5 to 65·4)	6·81 (4·54 to 9·71)	1·27 (0·271 to 2·43)	-3·79 (-4·94 to -2·55)	0·0921 (0·0750 to 0·120)	0·0137 (0·0111 to 0·0178)	-3·95 (-4·78 to -3·00)	-10·2 (-11·5 to -9·25)
	15-49 years	457 (375 to 588)	15·6 (12·8 to 20·0)	0·254 (-0·154 to 0·932)	-4·63 (-5·46 to -3·72)	13·9 (11·9 to 15·7)	0·473 (0·404 to 0·535)	-2·68 (-3·30 to -2·00)	-5·98 (-7·73 to -4·30)
	50-69 years	378 (281 to 474)	20·5 (15·2 to 25·7)	-1·41 (-2·07 to -0·733)	-3·52 (-4·37 to -2·59)	34·4 (29·7 to 40·2)	1·87 (1·61 to 2·18)	-2·23 (-3·02 to -1·45)	-7·20 (-8·75 to -5·83)
	70+ years	194 (156 to 238)	18·8 (15·1 to 23·1)	-2·73 (-3·44 to -2·03)	-4·71 (-5·78 to -3·59)	35·0 (31·7 to 38·6)	3·40 (3·08 to 3·75)	-3·18 (-3·91 to -2·43)	-6·54 (-7·71 to -5·41)
Croatia	All Ages	416 (362 to 464)	7·05 (6·21 to 8·04)	-3·66 (-4·07 to -3·28)	-4·71 (-5·45 to -4·09)	53·5 (46·9 to 59·9)	0·645 (0·565 to 0·716)	-5·29 (-5·70 to -4·87)	-6·33 (-7·56 to -5·24)
	Under 5	3·96 (2·97 to 5·14)	2·17 (1·63 to 2·82)	-4·35 (-5·26 to -3·45)	-3·59 (-4·92 to -2·32)	0·138 (0·110 to 0·169)	0·0758 (0·0603 to 0·0925)	-6·63 (-7·32 to -5·86)	-7·14 (-8·75 to -5·38)
	5-14 years	8·40 (5·27 to 12·0)	2·02 (1·27 to 2·88)	-4·16 (-5·07 to -3·30)	-5·00 (-6·52 to -3·39)	0·109 (0·0741 to 0·160)	0·0262 (0·0179 to 0·0386)	-4·21 (-5·76 to -2·78)	-7·81 (-8·99 to -5·95)
	15-49 years	128 (104 to 159)	7·01 (5·69 to 8·67)	-4·26 (-4·74 to -3·73)	-5·81 (-6·48 to -5·15)	5·54 (4·76 to 6·32)	0·303 (0·260 to 0·345)	-6·17 (-6·58 to -5·71)	-9·50 (-10·6 to -8·43)
	50-69 years	155 (119 to 198)	13·3 (10·2 to 17·0)	-4·37 (-4·88 to -3·83)	-4·67 (-5·52 to -3·86)	15·8 (13·6 to 18·1)	1·36 (1·17 to 1·55)	-7·71 (-8·33 to -7·06)	-7·82 (-9·31 to -6·45)
	70+ years	120 (95·6 to 148)	19·5 (15·6 to 24·0)	-4·40 (-4·97 to -3·77)	-4·85 (-5·88 to -3·76)	31·9 (27·5 to 36·5)	5·18 (4·47 to 5·94)	-6·63 (-7·03 to -6·16)	-6·22 (-7·51 to -5·19)
Czechia	All Ages	507 (432 to 591)	3·86 (3·33 to 4·53)	-4·07 (-4·49 to -3·67)	-3·50 (-4·10 to -2·97)	32·8 (29·4 to 36·5)	0·173 (0·154 to 0·194)	-5·41 (-5·88 to -4·89)	-6·53 (-7·42 to -5·53)
	Under 5	7·83 (5·94 to 10·7)	1·39 (1·06 to 1·90)	-4·27 (-4·93 to -3·42)	-1·96 (-3·24 to -0·938)	0·0883 (0·0676 to 0·115)	0·0157 (0·0120 to 0·0204)	-7·19 (-8·15 to -6·39)	-11·1 (-12·4 to -9·17)
	5-14 years	13·1 (8·47 to 19·4)	1·13 (0·733 to 1·68)	-5·27 (-6·06 to -4·49)	-0·789 (-1·76 to 0·229)	0·124 (0·0784 to 0·188)	0·0108 (0·00679 to 0·0163)	-3·36 (-5·15 to -1·54)	-8·44 (-9·78 to -5·83)
	15-49 years	217 (175 to 269)	4·59 (3·71 to 5·71)	-4·31 (-4·86 to -3·75)	-2·02 (-2·76 to -1·36)	5·15 (4·40 to 5·99)	0·109 (0·0933 to 0·127)	-5·22 (-5·75 to -4·61)	-7·86 (-9·01 to -6·80)
	50-69 years	163 (122 to 208)	6·13 (4·61 to 7·84)	-4·67 (-5·35 to -3·92)	-4·28 (-5·13 to -3·42)	9·40 (8·04 to 10·9)	0·354 (0·303 to 0·410)	-6·30 (-6·99 to -5·47)	-9·49 (-10·8 to -7·83)

eTable 1. All-form tuberculosis incident cases and deaths, age-standardised rates of incidence and mortality per 100,000 population, and corresponding annualized rates of change by age groups for 204 countries and territories (2021).

Location	Age group	Number of cases	Rate per 100,000 cases	Annualized rate of change (1990-2010)	Annualized rate of change (2010-2021)	Number of deaths	Rate per 100,000 deaths	Annualized deaths (1990-2010)	Annualized deaths (2010-2021)
	70+ years	107 (83·4 to 132)	6·92 (5·40 to 8·57)	-5·33 (-5·99 to -4·67)	-6·22 (-7·16 to -5·06)	18·0 (16·1 to 19·7)	1·16 (1·04 to 1·27)	-7·24 (-7·80 to -6·74)	-6·11 (-6·97 to -5·20)
Hungary	All Ages	634 (550 to 742)	4·96 (4·30 to 5·80)	-3·55 (-4·00 to -3·13)	-5·68 (-6·15 to -5·08)	62·4 (55·0 to 69·1)	0·374 (0·331 to 0·414)	-7·06 (-7·53 to -6·57)	-8·31 (-9·42 to -7·49)
	Under 5	7·77 (5·90 to 10·0)	1·71 (1·30 to 2·20)	-5·34 (-6·09 to -4·65)	-2·92 (-4·51 to -1·38)	0·264 (0·209 to 0·336)	0·0582 (0·0459 to 0·0740)	-7·61 (-8·18 to -7·16)	-10·1 (-11·6 to -8·63)
	5-14 years	11·7 (7·54 to 17·0)	1·25 (0·807 to 1·83)	-6·99 (-7·84 to -6·09)	-2·18 (-3·51 to -0·979)	0·240 (0·155 to 0·346)	0·0257 (0·0166 to 0·0371)	-6·44 (-7·00 to -5·77)	-10·8 (-11·6 to -9·77)
	15-49 years	233 (188 to 296)	5·37 (4·32 to 6·83)	-3·61 (-4·11 to -2·97)	-5·89 (-6·67 to -5·12)	10·0 (8·55 to 11·6)	0·231 (0·197 to 0·268)	-6·92 (-7·42 to -6·31)	-9·94 (-11·3 to -8·73)
	50-69 years	249 (188 to 314)	9·89 (7·45 to 12·5)	-3·35 (-4·06 to -2·39)	-5·84 (-6·64 to -5·04)	25·7 (22·3 to 29·3)	1·02 (0·883 to 1·16)	-7·63 (-8·42 to -6·83)	-8·26 (-9·76 to -6·89)
	70+ years	132 (104 to 164)	9·80 (7·69 to 12·2)	-5·84 (-6·56 to -5·09)	-6·36 (-7·50 to -5·39)	26·2 (22·6 to 29·6)	1·94 (1·68 to 2·20)	-8·93 (-9·40 to -8·51)	-9·21 (-10·2 to -8·06)
Montenegro	All Ages	66·5 (56·5 to 77·7)	8·69 (7·45 to 10·2)	-0·846 (-1·27 to -0·463)	-2·75 (-3·24 to -2·09)	6·49 (5·02 to 8·51)	0·730 (0·569 to 0·954)	-0·184 (-1·74 to 1·19)	-4·00 (-6·40 to -1·48)
	Under 5	0·820 (0·605 to 1·08)	2·27 (1·67 to 2·98)	-2·62 (-3·29 to -1·89)	-3·62 (-5·11 to -2·53)	0·0202 (0·0143 to 0·0274)	0·0558 (0·0396 to 0·0756)	-4·60 (-6·27 to -2·96)	-11·4 (-14·6 to -8·44)
	5-14 years	1·32 (0·848 to 1·85)	1·75 (1·13 to 2·46)	-2·55 (-3·28 to -1·84)	-3·06 (-4·00 to -1·97)	0·0164 (0·0107 to 0·0258)	0·0218 (0·0142 to 0·0343)	-1·41 (-3·04 to -0·0155)	-7·33 (-9·79 to -4·64)
	15-49 years	26·7 (21·8 to 34·4)	9·15 (7·46 to 11·8)	-1·61 (-2·10 to -1·16)	-3·02 (-3·68 to -2·24)	1·05 (0·781 to 1·44)	0·360 (0·267 to 0·491)	-1·78 (-3·54 to -0·00121)	-6·06 (-9·13 to -3·42)
	50-69 years	26·4 (19·4 to 33·7)	16·9 (12·4 to 21·5)	-1·00 (-1·57 to -0·386)	-3·02 (-3·81 to -2·08)	2·52 (1·95 to 3·48)	1·61 (1·25 to 2·22)	-1·21 (-3·11 to 0·338)	-5·93 (-8·78 to -3·12)
	70+ years	11·2 (8·90 to 14·1)	19·3 (15·3 to 24·2)	-1·45 (-1·96 to -0·907)	-3·11 (-4·02 to -2·08)	2·88 (2·23 to 3·60)	4·95 (3·83 to 6·19)	-1·28 (-2·84 to 0·134)	-2·17 (-4·98 to 0·335)
North Macedonia	All Ages	362 (315 to 419)	12·8 (11·3 to 14·7)	-2·22 (-2·69 to -1·77)	-1·91 (-2·49 to -1·32)	33·0 (24·6 to 44·1)	1·11 (0·843 to 1·47)	-3·45 (-4·77 to -2·36)	-6·63 (-9·30 to -3·99)
	Under 5	2·60 (2·03 to 3·31)	2·59 (2·02 to 3·29)	-6·29 (-6·93 to -5·56)	-3·86 (-4·97 to -2·70)	0·0739 (0·0569 to 0·0963)	0·0735 (0·0566 to 0·0958)	-14·5 (-15·7 to -13·4)	-11·0 (-13·6 to -8·34)
	5-14 years	5·23 (3·50 to 7·76)	2·30 (1·54 to 3·42)	-4·25 (-5·09 to -3·46)	-2·94 (-4·14 to -2·04)	0·0677 (0·0494 to 0·0906)	0·0298 (0·0217 to 0·0399)	-6·44 (-7·79 to -5·10)	-5·08 (-8·27 to -2·09)
	15-49 years	135 (110 to 170)	12·3 (9·98 to 15·5)	-2·98 (-3·55 to -2·41)	-2·60 (-3·36 to -1·77)	4·70 (3·50 to 6·72)	0·427 (0·318 to 0·611)	-4·61 (-5·69 to -3·52)	-9·28 (-12·4 to -5·83)
	50-69 years	151 (112 to 187)	27·1 (20·0 to 33·6)	-2·53 (-3·16 to -1·75)	-2·34 (-3·08 to -1·48)	12·1 (8·81 to 17·4)	2·17 (1·58 to 3·12)	-4·69 (-6·34 to -3·42)	-8·96 (-12·4 to -5·20)
	70+ years	67·9 (52·5 to 85·6)	35·7 (27·6 to 45·1)	-3·40 (-4·11 to -2·77)	-2·53 (-3·45 to -1·51)	16·1 (12·3 to 20·2)	8·46 (6·49 to 10·6)	-4·39 (-5·79 to -2·91)	-6·07 (-8·28 to -3·55)
Poland	All Ages	5730 (4830 to 6680)	11·1 (9·39 to 13·1)	-3·18 (-3·60 to -2·84)	-2·43 (-2·87 to -1·91)	441 (405 to 476)	0·707 (0·648 to 0·764)	-5·09 (-5·28 to -4·88)	-4·54 (-5·32 to -3·82)
	Under 5	41·6 (31·2 to 55·0)	2·21 (1·66 to 2·92)	-4·40 (-4·95 to -3·74)	-0·0444 (-0·741 to 0·607)	0·313 (0·234 to 0·437)	0·0166 (0·0124 to 0·0232)	-5·97 (-7·31 to -4·40)	-8·75 (-9·45 to -8·11)

eTable 1. All-form tuberculosis incident cases and deaths, age-standardised rates of incidence and mortality per 100,000 population, and corresponding annualized rates of change by age groups for 204 countries and territories (2021).

Location	Age group	Number of cases	Rate per 100,000 cases	Annualized rate of change (1990-2010)	Annualized rate of change (2010-2021)	Number of deaths	Rate per 100,000 deaths	Annualized deaths (1990-2010)	Annualized deaths (2010-2021)
	5-14 years	78·3 (48·2 to 121)	1·96 (1·20 to 3·01)	-4·63 (-5·22 to -4·09)	1·02 (0·170 to 1·68)	0·179 (0·131 to 0·245)	0·00448 (0·00328 to 0·00612)	-4·53 (-5·67 to -3·16)	-6·90 (-7·57 to -6·22)
	15-49 years	2180 (1720 to 2810)	12·2 (9·62 to 15·7)	-3·83 (-4·39 to -3·17)	-2·38 (-2·95 to -1·82)	94·0 (86·1 to 104)	0·524 (0·480 to 0·578)	-3·92 (-4·21 to -3·49)	-4·49 (-5·26 to -3·82)
	50-69 years	2350 (1710 to 3100)	24·1 (17·6 to 31·7)	-3·75 (-4·36 to -3·14)	-2·38 (-3·05 to -1·54)	194 (175 to 212)	1·98 (1·79 to 2·17)	-6·45 (-6·74 to -6·22)	-5·34 (-6·30 to -4·39)
	70+ years	1070 (843 to 1380)	22·9 (18·0 to 29·5)	-5·11 (-5·52 to -4·77)	-4·21 (-4·72 to -3·63)	153 (139 to 163)	3·28 (2·97 to 3·49)	-8·03 (-8·19 to -7·85)	-5·36 (-5·97 to -4·82)
Romania	All Ages	8520 (7320 to 9900)	38·1 (32·8 to 44·5)	-0·601 (-1·04 to -0·141)	-3·75 (-4·31 to -3·07)	816 (697 to 939)	2·86 (2·43 to 3·32)	-0·00241 (-0·257 to 0·227)	-5·52 (-6·81 to -4·53)
	Under 5	89·6 (69·4 to 112)	9·56 (7·40 to 12·0)	-4·13 (-5·17 to -3·15)	-3·59 (-4·90 to -2·48)	10·5 (8·75 to 12·4)	1·12 (0·933 to 1·32)	-6·86 (-7·29 to -6·42)	-2·95 (-4·66 to -0·837)
	5-14 years	256 (170 to 365)	12·4 (8·19 to 17·6)	-2·47 (-3·22 to -1·83)	-4·74 (-5·92 to -3·69)	2·09 (1·48 to 2·82)	0·101 (0·0714 to 0·136)	-2·57 (-3·10 to -2·05)	-7·84 (-8·95 to -6·36)
	15-49 years	4050 (3250 to 5110)	48·6 (39·0 to 61·4)	-0·682 (-1·19 to -0·0572)	-4·11 (-4·94 to -3·29)	223 (191 to 259)	2·68 (2·29 to 3·11)	-1·00 (-1·51 to -0·427)	-6·69 (-8·02 to -5·58)
	50-69 years	2940 (2140 to 3770)	57·9 (42·3 to 74·5)	-0·723 (-1·49 to -0·0606)	-3·33 (-4·34 to -2·36)	359 (305 to 418)	7·08 (6·03 to 8·26)	0·121 (-0·508 to 0·718)	-6·76 (-8·24 to -5·42)
	70+ years	1190 (934 to 1460)	47·1 (36·9 to 57·8)	-1·30 (-1·98 to -0·282)	-3·49 (-4·54 to -2·35)	221 (193 to 246)	8·75 (7·63 to 9·73)	-0·717 (-1·15 to -0·297)	-3·80 (-5·19 to -2·84)
Serbia	All Ages	1100 (964 to 1280)	9·51 (8·41 to 11·2)	-1·94 (-2·34 to -1·57)	-2·54 (-3·15 to -1·95)	125 (95·9 to 157)	0·864 (0·678 to 1·07)	-2·95 (-4·60 to -1·06)	-4·80 (-7·38 to -2·26)
	Under 5	8·44 (6·60 to 11·0)	2·29 (1·79 to 2·99)	-4·30 (-4·85 to -3·49)	-3·86 (-5·14 to -2·69)	0·402 (0·320 to 0·494)	0·109 (0·0867 to 0·134)	-10·4 (-11·9 to -8·74)	-4·47 (-6·89 to -2·08)
	5-14 years	30·2 (20·3 to 43·9)	3·15 (2·12 to 4·58)	-2·10 (-2·77 to -1·18)	-3·00 (-4·20 to -1·74)	0·718 (0·514 to 0·957)	0·0749 (0·0536 to 0·0998)	-2·45 (-4·11 to -0·559)	-4·77 (-7·97 to -1·34)
	15-49 years	416 (340 to 516)	9·81 (8·02 to 12·2)	-2·49 (-3·01 to -1·99)	-2·61 (-3·40 to -1·85)	22·0 (18·3 to 27·2)	0·520 (0·431 to 0·643)	-3·98 (-5·59 to -2·19)	-5·33 (-7·46 to -3·42)
	50-69 years	404 (306 to 505)	17·8 (13·5 to 22·2)	-2·41 (-3·00 to -1·69)	-2·91 (-3·80 to -2·04)	45·0 (34·6 to 55·5)	1·98 (1·52 to 2·45)	-4·18 (-6·02 to -2·13)	-5·69 (-7·94 to -3·13)
	70+ years	245 (194 to 306)	22·7 (17·9 to 28·3)	-2·67 (-3·33 to -2·15)	-3·20 (-4·31 to -2·34)	56·8 (41·9 to 74·3)	5·25 (3·88 to 6·88)	-4·41 (-6·21 to -2·72)	-5·30 (-8·41 to -2·06)
Slovakia	All Ages	320 (275 to 376)	4·45 (3·86 to 5·19)	-2·62 (-3·01 to -2·28)	-2·48 (-3·07 to -1·77)	31·7 (25·8 to 38·8)	0·364 (0·295 to 0·442)	-4·90 (-5·96 to -4·07)	-3·88 (-5·64 to -2·20)
	Under 5	3·50 (2·73 to 4·40)	1·22 (0·955 to 1·54)	-3·54 (-4·26 to -2·75)	-3·16 (-4·51 to -1·79)	0·136 (0·103 to 0·174)	0·0474 (0·0360 to 0·0608)	-6·49 (-7·83 to -5·35)	-5·59 (-8·23 to -3·47)
	5-14 years	3·46 (2·25 to 5·21)	0·606 (0·395 to 0·913)	-4·44 (-5·27 to -3·64)	-3·48 (-4·95 to -2·01)	0·0321 (0·0236 to 0·0419)	0·00563 (0·00414 to 0·00734)	-5·56 (-7·03 to -4·06)	-7·75 (-10·2 to -5·43)
	15-49 years	126 (103 to 157)	4·87 (3·99 to 6·08)	-3·20 (-3·60 to -2·82)	-2·61 (-3·45 to -1·84)	5·39 (4·12 to 7·07)	0·208 (0·159 to 0·274)	-6·13 (-7·03 to -4·99)	-5·09 (-7·56 to -2·53)
	50-69 years	124 (94·2 to 158)	8·93 (6·80 to 11·4)	-3·17 (-3·84 to -2·59)	-2·86 (-3·70 to -1·73)	12·2 (9·11 to 16·2)	0·884 (0·658 to 1·17)	-6·03 (-7·38 to -4·88)	-5·64 (-8·41 to -2·91)

eTable 1. All-form tuberculosis incident cases and deaths, age-standardised rates of incidence and mortality per 100,000 population, and corresponding annualized rates of change by age groups for 204 countries and territories (2021).

Location	Age group	Number of cases	Rate per 100,000 cases	Annualized rate of change (1990-2010)	Annualized rate of change (2010-2021)	Number of deaths	Rate per 100,000 deaths	Annualized deaths (1990-2010)	Annualized deaths (2010-2021)
	70+ years	63·1 (51·3 to 78·1)	10·5 (8·53 to 13·0)	-3·95 (-4·56 to -3·46)	-3·09 (-4·12 to -2·16)	13·9 (11·2 to 17·7)	2·31 (1·87 to 2·95)	-5·90 (-7·19 to -4·82)	-4·06 (-6·10 to -2·19)
Slovenia	All Ages	123 (106 to 142)	4·41 (3·88 to 5·16)	-4·04 (-4·38 to -3·65)	-3·83 (-4·40 to -3·08)	7·54 (6·50 to 8·39)	0·177 (0·153 to 0·197)	-4·03 (-4·36 to -3·72)	-9·15 (-10·3 to -8·02)
	Under 5	1·56 (1·18 to 2·07)	1·60 (1·21 to 2·12)	-3·71 (-4·52 to -2·75)	-2·90 (-4·09 to -1·62)	0·0106 (0·00873 to 0·0127)	0·0109 (0·00892 to 0·0130)	-8·37 (-9·21 to -7·79)	-14·0 (-15·2 to -12·7)
	5-14 years	3·14 (2·07 to 4·74)	1·46 (0·967 to 2·21)	-3·68 (-4·41 to -2·97)	-3·14 (-4·75 to -2·16)	0·0143 (0·00983 to 0·0205)	0·00667 (0·00459 to 0·00954)	-5·67 (-6·19 to -4·63)	-9·33 (-10·1 to -8·44)
	15-49 years	40·2 (32·6 to 49·4)	4·58 (3·71 to 5·64)	-4·98 (-5·46 to -4·33)	-4·35 (-5·41 to -3·59)	0·693 (0·602 to 0·793)	0·0790 (0·0686 to 0·0904)	-5·79 (-6·19 to -5·39)	-12·8 (-14·1 to -11·2)
	50-69 years	43·1 (32·8 to 54·7)	7·44 (5·65 to 9·43)	-4·54 (-5·10 to -4·02)	-4·08 (-5·08 to -3·23)	2·14 (1·83 to 2·42)	0·368 (0·316 to 0·417)	-5·71 (-6·20 to -5·16)	-12·1 (-13·7 to -10·7)
	70+ years	35·3 (27·0 to 43·4)	11·8 (9·00 to 14·4)	-4·49 (-5·29 to -3·80)	-4·29 (-5·46 to -3·01)	4·69 (3·98 to 5·19)	1·56 (1·32 to 1·73)	-5·06 (-5·54 to -4·62)	-8·76 (-9·91 to -7·65)
Eastern Europe	All Ages	147000 (123000 to 181000)	62·1 (52·2 to 76·2)	0·298 (-0·0802 to 0·639)	-4·43 (-5·12 to -3·81)	9560 (8510 to 10700)	3·44 (3·04 to 3·86)	3·29 (3·12 to 3·50)	-9·76 (-10·6 to -8·84)
	Under 5	632 (508 to 820)	6·25 (5·02 to 8·10)	-1·61 (-2·15 to -1·01)	-7·34 (-8·30 to -6·66)	31·9 (27·3 to 37·8)	0·316 (0·269 to 0·373)	-1·43 (-1·82 to -1·04)	-12·5 (-13·0 to -11·8)
	5-14 years	2870 (1870 to 4260)	11·3 (7·39 to 16·8)	-1·48 (-2·05 to -0·924)	-4·87 (-6·07 to -3·70)	13·3 (10·9 to 16·4)	0·0526 (0·0431 to 0·0648)	0·562 (0·250 to 0·862)	-10·9 (-11·3 to -10·5)
	15-49 years	99800 (78600 to 125000)	104 (81·6 to 130)	0·296 (-0·162 to 0·732)	-3·92 (-4·71 to -3·09)	4370 (3760 to 5030)	4·54 (3·90 to 5·23)	5·19 (4·98 to 5·44)	-10·2 (-11·0 to -9·52)
	50-69 years	35400 (26100 to 47700)	65·9 (48·7 to 88·7)	-0·388 (-0·885 to 0·157)	-4·49 (-5·27 to -3·59)	4070 (3600 to 4680)	7·58 (6·70 to 8·71)	1·61 (1·46 to 1·77)	-10·1 (-11·2 to -8·78)
	70+ years	7840 (6000 to 10100)	36·7 (28·1 to 47·3)	-1·13 (-1·56 to -0·676)	-3·74 (-4·47 to -2·99)	1070 (986 to 1160)	5·03 (4·61 to 5·45)	-1·39 (-1·52 to -1·25)	-5·39 (-6·06 to -4·74)
Belarus	All Ages	2490 (2090 to 2950)	22·3 (18·9 to 26·4)	-0·298 (-0·810 to 0·236)	-5·73 (-6·51 to -4·97)	245 (205 to 295)	1·86 (1·54 to 2·24)	3·59 (3·09 to 4·03)	-10·3 (-12·1 to -8·56)
	Under 5	15·7 (11·9 to 20·5)	3·37 (2·56 to 4·38)	-3·33 (-4·25 to -2·58)	-5·83 (-7·74 to -4·60)	0·834 (0·656 to 1·12)	0·178 (0·140 to 0·239)	-0·826 (-1·86 to 0·325)	-12·5 (-14·4 to -9·85)
	5-14 years	29·9 (19·0 to 43·5)	2·69 (1·71 to 3·91)	-5·49 (-6·45 to -4·56)	-5·70 (-7·11 to -4·46)	0·326 (0·262 to 0·420)	0·0293 (0·0236 to 0·0379)	0·973 (0·256 to 1·69)	-10·1 (-11·4 to -8·87)
	15-49 years	1460 (1140 to 1840)	34·2 (26·9 to 43·2)	-0·772 (-1·44 to -0·105)	-5·65 (-6·61 to -4·58)	88·3 (70·5 to 104)	2·08 (1·66 to 2·46)	4·94 (4·51 to 5·42)	-11·7 (-13·3 to -9·98)
	50-69 years	787 (588 to 995)	31·0 (23·2 to 39·2)	0·508 (-0·168 to 1·37)	-5·48 (-6·43 to -4·34)	127 (102 to 155)	5·01 (4·03 to 6·12)	2·69 (1·99 to 3·39)	-10·1 (-12·3 to -8·06)
	70+ years	205 (160 to 257)	21·4 (16·8 to 27·0)	-1·22 (-1·94 to -0·524)	-4·06 (-5·26 to -2·77)	28·6 (24·3 to 33·3)	3·00 (2·54 to 3·49)	-1·20 (-1·72 to -0·788)	-7·59 (-9·15 to -6·20)
Estonia	All Ages	202 (175 to 235)	13·3 (11·5 to 15·6)	-2·77 (-3·25 to -2·20)	-5·41 (-5·98 to -4·85)	25·3 (21·9 to 29·1)	1·39 (1·18 to 1·65)	0·482 (0·0836 to 0·854)	-9·10 (-10·4 to -7·95)
	Under 5	2·24 (1·77 to 2·85)	3·24 (2·55 to 4·12)	-3·39 (-4·09 to -2·62)	-4·59 (-6·07 to -2·68)	0·0958 (0·0752 to 0·129)	0·139 (0·109 to 0·187)	-3·32 (-4·11 to -2·54)	-12·3 (-14·1 to -9·50)

eTable 1. All-form tuberculosis incident cases and deaths, age-standardised rates of incidence and mortality per 100,000 population, and corresponding annualized rates of change by age groups for 204 countries and territories (2021).

Location	Age group	Number of cases	Rate per 100,000 cases	Annualized rate of change (1990-2010)	Annualized rate of change (2010-2021)	Number of deaths	Rate per 100,000 deaths	Annualized deaths (1990-2010)	Annualized deaths (2010-2021)
	5-14 years	5·72 (3·80 to 8·74)	3·89 (2·58 to 5·95)	-3·74 (-4·59 to -2·68)	-2·92 (-4·45 to -1·51)	0·0468 (0·0313 to 0·0684)	0·0319 (0·0213 to 0·0465)	-1·20 (-2·27 to -0·0587)	-9·93 (-12·0 to -6·29)
	15-49 years	107 (86·0 to 136)	18·6 (15·0 to 23·6)	-3·40 (-3·86 to -2·73)	-5·75 (-6·47 to -5·05)	9·58 (7·32 to 12·8)	1·67 (1·28 to 2·23)	0·488 (-0·0948 to 1·02)	-9·09 (-10·8 to -6·64)
	50-69 years	59·9 (43·9 to 77·9)	18·2 (13·3 to 23·6)	-2·02 (-2·68 to -1·18)	-4·93 (-5·72 to -3·99)	10·3 (9·05 to 11·9)	3·14 (2·75 to 3·62)	-0·518 (-1·03 to 0·0479)	-9·59 (-11·0 to -8·17)
	70+ years	27·0 (21·6 to 33·5)	14·1 (11·3 to 17·5)	-2·07 (-2·83 to -1·38)	-3·84 (-5·18 to -2·60)	5·24 (4·61 to 6·03)	2·74 (2·41 to 3·15)	0·0570 (-0·571 to 0·632)	-8·40 (-9·53 to -6·83)
Latvia	All Ages	515 (438 to 599)	24·7 (21·0 to 28·9)	-1·77 (-2·20 to -1·36)	-4·69 (-5·49 to -4·14)	55·2 (48·6 to 62·7)	2·08 (1·79 to 2·44)	0·282 (-0·209 to 0·838)	-6·74 (-7·57 to -5·64)
	Under 5	4·48 (3·53 to 5·75)	4·78 (3·77 to 6·14)	-1·90 (-2·59 to -1·18)	-6·50 (-7·83 to -5·47)	0·179 (0·140 to 0·231)	0·191 (0·150 to 0·247)	-2·17 (-2·71 to -1·50)	-12·0 (-13·1 to -10·6)
	5-14 years	11·9 (7·96 to 17·4)	5·85 (3·91 to 8·56)	-1·79 (-2·74 to -0·718)	-6·42 (-8·00 to -4·87)	0·101 (0·0659 to 0·152)	0·0497 (0·0324 to 0·0748)	-0·881 (-1·53 to -0·142)	-8·24 (-10·1 to -6·73)
	15-49 years	294 (237 to 367)	37·3 (30·1 to 46·5)	-1·87 (-2·39 to -1·44)	-4·49 (-5·23 to -3·70)	20·7 (16·5 to 26·1)	2·62 (2·10 to 3·31)	0·721 (0·0787 to 1·39)	-6·50 (-7·58 to -5·40)
	50-69 years	151 (111 to 191)	30·1 (22·1 to 37·9)	-2·00 (-2·60 to -1·32)	-3·94 (-4·97 to -3·03)	23·8 (21·4 to 27·2)	4·73 (4·25 to 5·41)	-0·582 (-1·13 to 0·0144)	-7·72 (-8·76 to -6·40)
	70+ years	53·8 (42·4 to 68·5)	19·1 (15·1 to 24·3)	-3·13 (-3·71 to -2·34)	-3·11 (-4·29 to -1·91)	10·5 (9·32 to 11·6)	3·72 (3·31 to 4·13)	-1·74 (-2·40 to -1·17)	-5·22 (-6·39 to -4·15)
Lithuania	All Ages	1040 (901 to 1200)	32·8 (28·3 to 37·8)	-0·989 (-1·36 to -0·504)	-4·12 (-4·80 to -3·34)	98·3 (87·9 to 111)	2·42 (2·16 to 2·76)	1·41 (1·07 to 1·84)	-8·50 (-9·60 to -7·46)
	Under 5	7·89 (6·14 to 10·5)	6·00 (4·66 to 8·00)	-1·61 (-2·61 to -0·864)	-5·96 (-7·40 to -4·84)	0·221 (0·187 to 0·269)	0·168 (0·142 to 0·205)	-1·61 (-2·33 to -1·02)	-15·8 (-17·3 to -14·3)
	5-14 years	24·7 (16·8 to 36·7)	8·95 (6·08 to 13·3)	-0·196 (-1·01 to 0·780)	-4·87 (-6·03 to -3·42)	0·116 (0·0876 to 0·159)	0·0421 (0·0317 to 0·0577)	-1·04 (-1·93 to -0·145)	-6·83 (-8·08 to -5·09)
	15-49 years	534 (440 to 657)	46·2 (38·1 to 56·9)	-1·52 (-2·05 to -0·877)	-4·08 (-4·81 to -3·16)	31·9 (28·0 to 36·6)	2·76 (2·42 to 3·17)	1·30 (0·717 to 1·80)	-9·20 (-10·3 to -8·04)
	50-69 years	356 (272 to 445)	46·6 (35·7 to 58·3)	-0·429 (-1·07 to 0·465)	-3·88 (-4·73 to -2·89)	44·3 (39·5 to 50·8)	5·81 (5·17 to 6·65)	0·818 (0·398 to 1·32)	-9·96 (-11·2 to -8·68)
	70+ years	115 (91·3 to 144)	28·6 (22·7 to 35·8)	-1·72 (-2·31 to -1·07)	-4·10 (-5·20 to -3·09)	21·7 (19·0 to 24·2)	5·40 (4·73 to 6·02)	-0·604 (-1·08 to -0·107)	-6·28 (-7·38 to -5·36)
Republic of Moldova	All Ages	2350 (1980 to 2730)	54·8 (46·6 to 63·9)	1·33 (0·817 to 1·81)	-3·98 (-4·84 to -2·89)	138 (124 to 154)	2·72 (2·43 to 3·05)	4·83 (4·43 to 5·21)	-11·1 (-12·1 to -10·1)
	Under 5	14·1 (11·0 to 18·2)	9·17 (7·13 to 11·8)	-1·62 (-2·38 to -0·852)	-4·34 (-5·67 to -3·02)	0·800 (0·615 to 1·02)	0·519 (0·398 to 0·662)	-4·09 (-5·10 to -3·14)	-7·23 (-8·83 to -5·20)
	5-14 years	50·9 (35·8 to 74·6)	13·8 (9·72 to 20·3)	-0·620 (-1·58 to 0·427)	-2·44 (-3·74 to -0·818)	0·207 (0·163 to 0·266)	0·0563 (0·0444 to 0·0723)	0·813 (0·154 to 1·70)	-10·1 (-11·4 to -8·67)
	15-49 years	1500 (1190 to 1850)	83·9 (66·6 to 103)	0·821 (0·333 to 1·36)	-4·08 (-5·19 to -3·03)	53·2 (46·9 to 60·7)	2·97 (2·62 to 3·39)	5·14 (4·77 to 5·56)	-12·6 (-13·6 to -11·6)
	50-69 years	653 (508 to 839)	70·0 (54·5 to 90·0)	1·41 (0·732 to 2·12)	-3·74 (-4·98 to -2·51)	67·0 (59·2 to 75·9)	7·18 (6·34 to 8·14)	3·75 (3·22 to 4·29)	-11·4 (-12·7 to -10·3)
	70+ years	131 (101 to 162)	37·5 (29·1 to 46·4)	-0·256 (-0·942 to 0·512)	-2·84 (-4·08 to -1·46)	17·0 (15·3 to 18·7)	4·89 (4·39 to 5·36)	0·941 (0·243 to 1·51)	-7·10 (-8·23 to -6·08)

eTable 1. All-form tuberculosis incident cases and deaths, age-standardised rates of incidence and mortality per 100,000 population, and corresponding annualized rates of change by age groups for 204 countries and territories (2021).

Location	Age group	Number of cases	Rate per 100,000 cases	Annualized rate of change (1990-2010)	Annualized rate of change (2010-2021)	Number of deaths	Rate per 100,000 deaths	Annualized deaths (1990-2010)	Annualized deaths (2010-2021)
Russian Federation	All Ages	107000 (88900 to 133000)	65·2 (54·6 to 81·6)	-0·118 (-0·535 to 0·285)	-4·66 (-5·50 to -3·93)	6140 (5610 to 6840)	3·20 (2·91 to 3·59)	3·32 (3·17 to 3·47)	-11·1 (-11·7 to -10·4)
	Under 5	485 (385 to 628)	6·37 (5·06 to 8·25)	-1·83 (-2·39 to -1·17)	-8·34 (-9·43 to -7·47)	23·2 (20·0 to 27·8)	0·305 (0·263 to 0·365)	-1·98 (-2·41 to -1·59)	-13·6 (-14·3 to -12·8)
	5-14 years	2280 (1470 to 3360)	12·4 (7·97 to 18·2)	-1·83 (-2·45 to -1·26)	-5·39 (-6·78 to -4·23)	9·67 (7·85 to 12·0)	0·0523 (0·0425 to 0·0651)	0·714 (0·422 to 0·988)	-12·3 (-12·7 to -11·9)
	15-49 years	73700 (57100 to 93100)	109 (84·7 to 138)	-0·0921 (-0·593 to 0·386)	-4·08 (-5·05 to -3·18)	2930 (2590 to 3440)	4·34 (3·84 to 5·10)	5·16 (4·96 to 5·33)	-11·4 (-12·0 to -10·7)
	50-69 years	24900 (18200 to 33800)	67·1 (49·1 to 91·4)	-0·896 (-1·41 to -0·212)	-4·62 (-5·55 to -3·46)	2410 (2220 to 2670)	6·52 (5·99 to 7·20)	1·51 (1·40 to 1·62)	-11·8 (-12·6 to -11·0)
	70+ years	5350 (4020 to 6900)	37·4 (28·1 to 48·2)	-1·44 (-1·90 to -0·975)	-3·96 (-4·79 to -3·06)	766 (718 to 818)	5·35 (5·02 to 5·72)	-1·21 (-1·30 to -1·10)	-5·20 (-5·67 to -4·62)
Ukraine	All Ages	33300 (28100 to 39900)	65·6 (55·1 to 79·1)	1·87 (1·40 to 2·34)	-3·69 (-4·57 to -2·87)	2860 (2090 to 3810)	4·79 (3·51 to 6·31)	3·21 (2·77 to 3·81)	-6·02 (-8·45 to -3·60)
	Under 5	103 (80·1 to 139)	6·47 (5·03 to 8·72)	-0·334 (-0·995 to 0·346)	-3·52 (-4·55 to -2·09)	6·61 (5·43 to 8·23)	0·415 (0·341 to 0·517)	2·01 (1·31 to 2·78)	-8·43 (-9·72 to -7·10)
	5-14 years	468 (292 to 700)	9·85 (6·15 to 14·7)	0·737 (-0·0156 to 1·48)	-3·01 (-4·06 to -1·81)	2·87 (2·33 to 3·51)	0·0603 (0·0491 to 0·0739)	-0·421 (-1·07 to 0·335)	-5·43 (-6·72 to -4·16)
	15-49 years	22200 (17900 to 28200)	110 (88·4 to 140)	1·95 (1·28 to 2·56)	-3·33 (-4·47 to -2·20)	1240 (897 to 1590)	6·12 (4·43 to 7·88)	5·72 (5·17 to 6·21)	-6·54 (-8·65 to -4·81)
	50-69 years	8550 (6040 to 11500)	73·5 (52·0 to 99·1)	1·01 (0·223 to 1·58)	-4·05 (-5·13 to -2·93)	1390 (972 to 1970)	11·9 (8·35 to 17·0)	1·71 (1·24 to 2·23)	-6·00 (-9·01 to -3·03)
	70+ years	1960 (1450 to 2520)	40·2 (29·7 to 51·6)	-0·174 (-0·923 to 0·525)	-3·15 (-4·47 to -1·79)	226 (171 to 286)	4·62 (3·50 to 5·86)	-2·10 (-2·51 to -1·67)	-5·57 (-8·00 to -3·14)
High-income	All Ages	99300 (87900 to 114000)	7·29 (6·36 to 8·56)	-2·94 (-3·18 to -2·69)	-1·93 (-2·16 to -1·69)	12700 (11200 to 13900)	0·576 (0·519 to 0·630)	-4·45 (-4·86 to -4·22)	-2·11 (-2·42 to -1·76)
	Under 5	937 (727 to 1190)	1·73 (1·34 to 2·20)	-2·33 (-2·74 to -1·94)	-3·42 (-3·70 to -2·94)	24·2 (20·0 to 28·4)	0·0445 (0·0368 to 0·0524)	-8·40 (-8·93 to -7·92)	-6·05 (-6·79 to -5·35)
	5-14 years	2370 (1590 to 3440)	1·94 (1·30 to 2·81)	-2·92 (-3·37 to -2·49)	-2·41 (-2·79 to -1·98)	19·2 (16·3 to 22·1)	0·0157 (0·0133 to 0·0181)	-9·11 (-9·80 to -8·34)	-4·29 (-4·85 to -3·71)
	15-49 years	40900 (33500 to 50900)	8·45 (6·91 to 10·5)	-3·04 (-3·44 to -2·69)	-2·52 (-2·74 to -2·30)	1470 (1170 to 1780)	0·303 (0·241 to 0·367)	-6·50 (-6·80 to -6·24)	-3·86 (-4·23 to -3·47)
	50-69 years	27600 (21000 to 34300)	9·94 (7·58 to 12·4)	-3·84 (-4·37 to -3·32)	-2·05 (-2·45 to -1·72)	2330 (2090 to 2520)	0·839 (0·754 to 0·908)	-7·28 (-7·68 to -6·91)	-3·83 (-4·38 to -3·44)
	70+ years	27500 (22600 to 33900)	17·9 (14·8 to 22·1)	-3·20 (-3·57 to -2·91)	-2·13 (-2·59 to -1·66)	8820 (7290 to 9740)	5·76 (4·76 to 6·36)	-4·14 (-4·63 to -3·86)	-3·24 (-3·65 to -2·79)
Australasia	All Ages	1740 (1510 to 2030)	5·24 (4·50 to 6·22)	-2·87 (-3·29 to -2·50)	0·118 (-0·269 to 0·489)	83·2 (74·8 to 90·9)	0·151 (0·136 to 0·165)	-4·23 (-4·66 to -3·91)	-2·40 (-3·20 to -1·73)
	Under 5	38·5 (29·4 to 50·4)	2·12 (1·62 to 2·78)	-2·47 (-3·08 to -1·74)	-2·14 (-3·21 to -1·08)	0·165 (0·141 to 0·189)	0·00909 (0·00778 to 0·0104)	-6·77 (-7·14 to -6·38)	-6·95 (-8·49 to -5·61)
	5-14 years	51·8 (34·4 to 76·1)	1·32 (0·880 to 1·94)	-3·83 (-4·70 to -2·90)	-0·614 (-1·45 to 0·183)	0·111 (0·0928 to 0·138)	0·00285 (0·00237 to 0·00352)	-6·04 (-6·52 to -5·54)	-5·86 (-7·19 to -4·91)

eTable 1. All-form tuberculosis incident cases and deaths, age-standardised rates of incidence and mortality per 100,000 population, and corresponding annualized rates of change by age groups for 204 countries and territories (2021).

Location	Age group	Number of cases	Rate per 100,000 cases	Annualized rate of change (1990-2010)	Annualized rate of change (2010-2021)	Number of deaths	Rate per 100,000 deaths	Annualized deaths (1990-2010)	Annualized deaths (2010-2021)
	15-49 years	1070 (866 to 1300)	7·38 (6·00 to 8·99)	-2·34 (-2·95 to -1·76)	0·619 (0·102 to 1·21)	6·16 (5·09 to 7·55)	0·0426 (0·0353 to 0·0523)	-7·14 (-7·79 to -6·58)	-4·70 (-5·32 to -4·14)
	50-69 years	341 (256 to 433)	4·78 (3·58 to 6·06)	-4·38 (-4·93 to -3·76)	0·0389 (-0·691 to 0·761)	18·1 (16·3 to 19·9)	0·253 (0·228 to 0·278)	-6·63 (-7·04 to -6·33)	-3·44 (-4·15 to -2·71)
	70+ years	240 (193 to 298)	6·59 (5·29 to 8·17)	-3·78 (-4·47 to -3·08)	-1·86 (-2·83 to -1·13)	58·7 (50·6 to 64·2)	1·61 (1·39 to 1·76)	-4·24 (-4·58 to -3·87)	-3·80 (-4·71 to -3·05)
Australia	All Ages	1380 (1210 to 1600)	4·94 (4·26 to 5·81)	-2·53 (-2·95 to -2·10)	-0·270 (-0·710 to 0·160)	77·5 (69·4 to 84·8)	0·166 (0·150 to 0·182)	-3·82 (-4·30 to -3·46)	-1·17 (-1·94 to -0·501)
	Under 5	29·0 (21·9 to 38·1)	1·93 (1·45 to 2·54)	-2·78 (-3·55 to -1·87)	-1·91 (-3·12 to -0·526)	0·139 (0·117 to 0·161)	0·00923 (0·00777 to 0·0107)	-6·19 (-6·59 to -5·70)	-5·97 (-7·58 to -4·55)
	5-14 years	33·1 (21·7 to 48·4)	1·02 (0·669 to 1·49)	-3·40 (-4·48 to -2·35)	-1·92 (-2·98 to -1·03)	0·101 (0·0841 to 0·125)	0·00312 (0·00259 to 0·00386)	-5·22 (-5·76 to -4·67)	-5·16 (-6·65 to -4·09)
	15-49 years	827 (680 to 1000)	6·89 (5·67 to 8·34)	-1·99 (-2·64 to -1·36)	-0·0299 (-0·690 to 0·683)	5·58 (4·63 to 6·85)	0·0464 (0·0386 to 0·0570)	-7·06 (-7·74 to -6·43)	-3·01 (-3·67 to -2·39)
	50-69 years	282 (213 to 354)	4·74 (3·57 to 5·95)	-3·93 (-4·56 to -3·29)	-0·00160 (-0·874 to 0·911)	16·4 (14·7 to 18·1)	0·275 (0·247 to 0·305)	-6·34 (-6·86 to -5·89)	-1·89 (-2·67 to -1·10)
	70+ years	210 (169 to 260)	6·82 (5·50 to 8·45)	-3·55 (-4·33 to -2·76)	-1·63 (-2·68 to -0·815)	55·2 (47·5 to 60·4)	1·79 (1·54 to 1·96)	-3·87 (-4·27 to -3·41)	-2·77 (-3·69 to -1·99)
New Zealand	All Ages	356 (302 to 433)	6·72 (5·57 to 8·20)	-4·26 (-4·63 to -3·86)	1·80 (0·928 to 2·62)	5·71 (5·22 to 6·19)	0·0694 (0·0644 to 0·0752)	-5·69 (-6·07 to -5·33)	-11·5 (-12·2 to -10·8)
	Under 5	9·49 (7·19 to 12·6)	3·04 (2·30 to 4·02)	-1·46 (-2·11 to -0·804)	-2·76 (-4·00 to -1·33)	0·0263 (0·0223 to 0·0304)	0·00843 (0·00713 to 0·00973)	-8·09 (-8·85 to -7·22)	-10·8 (-12·6 to -8·87)
	5-14 years	18·7 (12·3 to 28·2)	2·79 (1·84 to 4·22)	-4·94 (-5·66 to -4·12)	2·41 (1·25 to 3·72)	0·0102 (0·00865 to 0·0123)	0·00152 (0·00129 to 0·00183)	-8·94 (-9·63 to -8·28)	-10·9 (-12·0 to -9·75)
	15-49 years	239 (183 to 304)	9·82 (7·53 to 12·5)	-3·79 (-4·32 to -3·10)	3·29 (2·19 to 4·47)	0·578 (0·470 to 0·724)	0·0237 (0·0193 to 0·0298)	-7·42 (-7·97 to -7·00)	-13·5 (-14·3 to -12·9)
	50-69 years	59·2 (43·0 to 80·1)	4·95 (3·60 to 6·70)	-6·17 (-6·70 to -5·46)	0·219 (-0·903 to 1·27)	1·68 (1·54 to 1·79)	0·140 (0·129 to 0·150)	-7·50 (-7·89 to -7·02)	-11·9 (-12·8 to -11·1)
	70+ years	29·6 (22·4 to 38·4)	5·29 (4·00 to 6·86)	-4·83 (-5·53 to -4·15)	-3·39 (-4·77 to -1·98)	3·42 (2·95 to 3·77)	0·611 (0·527 to 0·674)	-5·68 (-6·08 to -5·33)	-12·9 (-13·8 to -12·2)
High-income Asia Pacific	All Ages	47900 (41400 to 53800)	15·7 (13·8 to 18·4)	-2·39 (-2·84 to -1·96)	-1·85 (-2·24 to -1·45)	6460 (5280 to 7290)	1·08 (0·915 to 1·20)	-3·52 (-4·30 to -3·06)	-1·00 (-1·62 to -0·358)
	Under 5	57·7 (43·8 to 73·9)	0·894 (0·679 to 1·15)	-6·52 (-7·44 to -5·72)	-3·82 (-5·02 to -2·80)	4·18 (3·66 to 4·57)	0·0648 (0·0568 to 0·0708)	-8·39 (-9·79 to -7·17)	-7·08 (-7·92 to -6·36)
	5-14 years	138 (89·9 to 210)	0·862 (0·563 to 1·32)	-5·53 (-6·67 to -4·43)	-6·18 (-7·42 to -4·93)	2·54 (2·20 to 2·80)	0·0159 (0·0138 to 0·0175)	-13·1 (-14·1 to -11·8)	-6·35 (-7·52 to -5·30)
	15-49 years	11900 (9900 to 15000)	15·3 (12·7 to 19·2)	-2·86 (-3·63 to -2·17)	-4·33 (-4·77 to -3·95)	170 (154 to 188)	0·218 (0·197 to 0·241)	-8·20 (-8·97 to -7·40)	-7·19 (-8·42 to -6·13)
	50-69 years	16200 (12500 to 20400)	32·4 (25·1 to 41·0)	-3·47 (-4·28 to -2·61)	-1·74 (-2·36 to -1·19)	660 (590 to 742)	1·32 (1·18 to 1·49)	-8·46 (-9·10 to -7·75)	-5·71 (-6·75 to -4·77)
	70+ years	19600 (16000 to 24000)	56·0 (45·7 to 68·8)	-4·34 (-4·86 to -3·90)	-2·40 (-2·99 to -1·69)	5620 (4510 to 6380)	16·1 (12·9 to 18·3)	-4·84 (-5·68 to -4·36)	-2·89 (-3·52 to -2·21)

eTable 1. All-form tuberculosis incident cases and deaths, age-standardised rates of incidence and mortality per 100,000 population, and corresponding annualized rates of change by age groups for 204 countries and territories (2021).

Location	Age group	Number of cases	Rate per 100,000 cases	Annualized rate of change (1990-2010)	Annualized rate of change (2010-2021)	Number of deaths	Rate per 100,000 deaths	Annualized deaths (1990-2010)	Annualized deaths (2010-2021)
Brunei Darussalam	All Ages	249 (217 to 288)	60·0 (52·1 to 67·7)	-1·30 (-1·86 to -0·748)	-0·723 (-1·51 to -0·0213)	17·6 (14·6 to 21·0)	6·87 (5·64 to 8·25)	-4·77 (-6·35 to -3·50)	-0·833 (-2·92 to 1·24)
	Under 5	1·15 (0·884 to 1·46)	3·71 (2·86 to 4·74)	-3·88 (-4·79 to -3·16)	-2·48 (-3·85 to -1·08)	0·127 (0·0967 to 0·171)	0·410 (0·313 to 0·553)	-4·61 (-6·29 to -2·91)	-3·06 (-5·69 to -0·734)
	5-14 years	2·87 (1·82 to 4·14)	4·51 (2·86 to 6·51)	-3·26 (-4·04 to -2·30)	-1·60 (-2·71 to -0·307)	0·0639 (0·0527 to 0·0795)	0·100 (0·0828 to 0·125)	-6·35 (-7·75 to -4·91)	-5·94 (-8·27 to -3·76)
	15-49 years	128 (106 to 158)	47·1 (38·9 to 58·1)	-1·91 (-2·71 to -0·925)	-1·94 (-2·63 to -1·08)	3·36 (2·70 to 4·07)	1·24 (0·996 to 1·50)	-6·12 (-7·23 to -4·88)	-1·21 (-3·56 to 1·48)
	50-69 years	78·7 (60·2 to 95·9)	110 (84·4 to 134)	-1·89 (-2·74 to -1·06)	-1·48 (-2·63 to -0·498)	5·00 (4·01 to 6·29)	7·01 (5·62 to 8·81)	-7·13 (-8·56 to -5·76)	-2·55 (-4·80 to -0·174)
	70+ years	38·2 (28·8 to 48·6)	280 (211 to 356)	-3·09 (-4·13 to -1·94)	-3·12 (-4·00 to -2·03)	9·09 (7·19 to 11·1)	66·6 (52·7 to 81·7)	-5·30 (-7·75 to -3·82)	-4·49 (-6·91 to -2·31)
Japan	All Ages	15700 (13700 to 18000)	6·36 (5·65 to 7·41)	-2·63 (-3·11 to -2·09)	-2·33 (-2·73 to -1·79)	3880 (3140 to 4340)	0·698 (0·596 to 0·766)	-1·51 (-2·02 to -1·22)	-0·204 (-0·825 to 0·187)
	Under 5	27·1 (20·2 to 35·4)	0·592 (0·440 to 0·771)	-4·61 (-5·30 to -3·65)	-1·10 (-2·06 to -0·162)	2·59 (2·07 to 2·87)	0·0564 (0·0452 to 0·0626)	-1·59 (-2·68 to -0·897)	-2·82 (-3·33 to -2·36)
	5-14 years	46·1 (27·9 to 73·2)	0·424 (0·257 to 0·674)	-6·41 (-7·67 to -4·97)	-0·317 (-1·17 to 0·514)	0·863 (0·678 to 0·986)	0·00795 (0·00624 to 0·00908)	-2·38 (-3·46 to -1·62)	-2·07 (-2·48 to -1·73)
	15-49 years	2960 (2410 to 3800)	5·84 (4·75 to 7·49)	-2·59 (-3·25 to -1·84)	-3·22 (-3·82 to -2·57)	43·2 (38·7 to 46·6)	0·0853 (0·0763 to 0·0919)	-5·38 (-5·85 to -5·07)	-4·43 (-4·76 to -4·15)
	50-69 years	3650 (2740 to 4740)	11·2 (8·40 to 14·5)	-4·61 (-5·22 to -3·64)	-4·52 (-5·05 to -4·07)	211 (196 to 224)	0·647 (0·600 to 0·686)	-7·92 (-8·22 to -7·67)	-5·96 (-6·31 to -5·58)
	70+ years	9020 (7360 to 11300)	31·2 (25·5 to 39·1)	-4·84 (-5·42 to -4·19)	-3·17 (-3·80 to -2·49)	3630 (2880 to 4070)	12·5 (9·95 to 14·1)	-3·57 (-4·09 to -3·29)	-2·47 (-3·04 to -2·10)
Republic of Korea	All Ages	29800 (25500 to 33600)	38·4 (33·6 to 43·9)	-2·47 (-2·98 to -2·04)	-2·03 (-2·61 to -1·42)	2500 (2080 to 2940)	2·79 (2·32 to 3·27)	-5·48 (-6·49 to -4·66)	-2·18 (-3·37 to -0·833)
	Under 5	25·1 (18·4 to 31·9)	1·62 (1·19 to 2·06)	-6·96 (-8·09 to -5·85)	-5·24 (-6·92 to -4·05)	1·30 (1·13 to 1·48)	0·0836 (0·0729 to 0·0954)	-9·86 (-11·3 to -8·47)	-11·2 (-12·8 to -9·39)
	5-14 years	80·6 (55·0 to 116)	1·78 (1·22 to 2·57)	-5·36 (-6·74 to -3·95)	-7·82 (-9·05 to -6·32)	1·48 (1·21 to 1·72)	0·0326 (0·0267 to 0·0381)	-14·4 (-15·7 to -13·0)	-7·34 (-9·23 to -5·75)
	15-49 years	7980 (6580 to 10000)	32·8 (27·1 to 41·2)	-3·57 (-4·50 to -2·74)	-5·05 (-5·62 to -4·49)	119 (104 to 135)	0·490 (0·429 to 0·556)	-9·34 (-10·3 to -8·38)	-8·13 (-9·70 to -6·65)
	50-69 years	11700 (9060 to 15000)	74·8 (57·8 to 95·8)	-3·94 (-5·13 to -2·99)	-3·10 (-4·00 to -2·30)	430 (360 to 510)	2·75 (2·30 to 3·25)	-10·2 (-11·2 to -9·05)	-7·79 (-9·48 to -6·29)
	70+ years	10000 (7980 to 12200)	181 (144 to 221)	-4·64 (-5·43 to -3·79)	-2·48 (-3·61 to -1·43)	1950 (1590 to 2330)	35·2 (28·6 to 42·0)	-7·48 (-8·61 to -6·42)	-4·17 (-5·63 to -2·56)
Singapore	All Ages	2120 (1840 to 2440)	28·2 (24·7 to 32·1)	-2·65 (-3·15 to -2·18)	-0·461 (-1·14 to 0·226)	56·2 (50·9 to 62·3)	0·692 (0·627 to 0·771)	-5·30 (-5·69 to -4·82)	-5·00 (-5·75 to -4·24)
	Under 5	4·31 (3·01 to 5·65)	1·51 (1·05 to 1·98)	-4·81 (-5·68 to -3·98)	-3·90 (-5·72 to -2·64)	0·175 (0·142 to 0·231)	0·0612 (0·0496 to 0·0807)	-7·68 (-8·55 to -6·55)	-9·86 (-11·3 to -8·46)
	5-14 years	8·17 (5·62 to 11·8)	1·55 (1·07 to 2·25)	-3·85 (-4·65 to -2·94)	-4·28 (-5·41 to -2·80)	0·136 (0·116 to 0·156)	0·0258 (0·0220 to 0·0297)	-5·77 (-6·66 to -4·98)	-8·09 (-9·29 to -6·71)

eTable 1. All-form tuberculosis incident cases and deaths, age-standardised rates of incidence and mortality per 100,000 population, and corresponding annualized rates of change by age groups for 204 countries and territories (2021).

Location	Age group	Number of cases	Rate per 100,000 cases	Annualized rate of change (1990-2010)	Annualized rate of change (2010-2021)	Number of deaths	Rate per 100,000 deaths	Annualized deaths (1990-2010)	Annualized deaths (2010-2021)
	15-49 years	879 (715 to 1120)	29·5 (24·0 to 37·6)	-2·48 (-3·09 to -1·76)	-0·725 (-1·74 to 0·144)	4·79 (4·19 to 5·49)	0·161 (0·140 to 0·184)	-6·53 (-7·16 to -5·82)	-7·08 (-7·88 to -6·25)
	50-69 years	734 (563 to 925)	50·7 (38·9 to 63·8)	-4·32 (-5·23 to -3·55)	-1·99 (-3·02 to -0·685)	13·1 (11·7 to 14·7)	0·907 (0·808 to 1·02)	-7·75 (-8·39 to -6·92)	-10·0 (-10·9 to -9·06)
	70+ years	490 (380 to 612)	101 (78·7 to 127)	-5·03 (-5·67 to -4·43)	-2·79 (-3·84 to -1·83)	37·9 (32·5 to 42·8)	7·85 (6·72 to 8·86)	-6·32 (-6·76 to -5·83)	-8·23 (-9·16 to -7·40)
High-income North America	All Ages	10800 (9480 to 12600)	2·48 (2·17 to 2·96)	-3·16 (-3·36 to -2·95)	-0·340 (-0·632 to 0·00860)	1080 (1000 to 1180)	0·183 (0·168 to 0·201)	-7·39 (-7·84 to -7·09)	-0·466 (-0·786 to -0·179)
	Under 5	218 (165 to 283)	1·06 (0·805 to 1·38)	2·22 (1·91 to 2·54)	-3·11 (-3·62 to -2·70)	3·28 (2·92 to 3·81)	0·0160 (0·0143 to 0·0186)	-10·0 (-10·6 to -9·57)	-2·12 (-2·76 to -1·51)
	5-14 years	302 (202 to 446)	0·669 (0·448 to 0·990)	-0·761 (-1·19 to -0·206)	0·210 (-0·499 to 0·896)	1·71 (1·53 to 2·02)	0·00379 (0·00339 to 0·00448)	-9·55 (-9·98 to -9·17)	-1·45 (-1·77 to -1·06)
	15-49 years	4830 (3890 to 6090)	2·86 (2·31 to 3·61)	-2·96 (-3·33 to -2·66)	-0·673 (-1·29 to -0·141)	173 (148 to 210)	0·103 (0·0876 to 0·125)	-10·4 (-10·9 to -9·89)	-2·21 (-2·89 to -1·61)
	50-69 years	3170 (2390 to 4030)	3·43 (2·58 to 4·36)	-4·55 (-4·89 to -4·19)	-0·856 (-1·38 to -0·373)	369 (335 to 415)	0·399 (0·361 to 0·448)	-7·08 (-7·26 to -6·86)	-1·04 (-1·33 to -0·774)
	70+ years	2300 (1820 to 2960)	5·31 (4·19 to 6·83)	-3·90 (-4·16 to -3·63)	-0·352 (-0·736 to 0·0235)	531 (468 to 565)	1·23 (1·08 to 1·30)	-6·50 (-6·75 to -6·33)	-2·03 (-2·28 to -1·71)
Canada	All Ages	2060 (1810 to 2370)	4·65 (4·03 to 5·45)	-2·24 (-2·54 to -1·97)	0·367 (-0·0206 to 0·824)	127 (116 to 144)	0·195 (0·174 to 0·225)	-6·32 (-6·95 to -5·91)	-0·968 (-1·70 to -0·391)
	Under 5	32·2 (24·1 to 42·3)	1·70 (1·27 to 2·23)	-0·237 (-0·862 to 0·420)	0·0686 (-0·758 to 1·27)	0·273 (0·219 to 0·337)	0·0144 (0·0115 to 0·0178)	-9·13 (-9·64 to -8·65)	-3·13 (-4·31 to -2·09)
	5-14 years	53·8 (35·8 to 78·5)	1·26 (0·838 to 1·84)	-0·489 (-1·07 to 0·190)	0·959 (-0·0899 to 2·07)	0·173 (0·142 to 0·222)	0·00404 (0·00333 to 0·00520)	-9·79 (-10·2 to -9·32)	-1·39 (-2·18 to -0·645)
	15-49 years	948 (763 to 1170)	5·70 (4·59 to 7·05)	-1·86 (-2·14 to -1·55)	0·654 (0·137 to 1·09)	20·3 (16·0 to 26·3)	0·122 (0·0963 to 0·158)	-9·01 (-9·64 to -8·54)	-2·28 (-3·14 to -1·47)
	50-69 years	552 (421 to 691)	5·61 (4·28 to 7·02)	-3·98 (-4·44 to -3·47)	-0·166 (-0·808 to 0·642)	33·5 (28·5 to 39·6)	0·340 (0·290 to 0·402)	-7·52 (-8·15 to -6·88)	-1·76 (-2·61 to -0·944)
	70+ years	474 (379 to 594)	9·83 (7·87 to 12·3)	-3·45 (-4·09 to -3·01)	-0·912 (-1·77 to 0·192)	72·9 (62·9 to 80·1)	1·51 (1·31 to 1·66)	-6·01 (-6·44 to -5·65)	-2·45 (-2·99 to -1·87)
Greenland	All Ages	55·5 (47·4 to 67·9)	85·9 (74·1 to 101)	1·14 (0·802 to 1·53)	-1·68 (-2·28 to -0·867)	2·47 (2·13 to 2·92)	4·14 (3·54 to 5·03)	-3·34 (-4·21 to -2·45)	-1·75 (-3·40 to -0·161)
	Under 5	0·446 (0·333 to 0·570)	11·0 (8·23 to 14·1)	-1·74 (-2·55 to -1·09)	-3·82 (-4·95 to -2·59)	0·0161 (0·0121 to 0·0202)	0·398 (0·300 to 0·500)	-7·21 (-8·28 to -5·87)	-7·22 (-9·07 to -5·14)
	5-14 years	1·24 (0·806 to 1·91)	16·1 (10·5 to 24·7)	1·78 (0·978 to 2·67)	-2·91 (-4·34 to -1·60)	0·00209 (0·00156 to 0·00290)	0·0271 (0·0202 to 0·0376)	-7·78 (-9·86 to -5·59)	-6·55 (-9·75 to -3·98)
	15-49 years	22·7 (18·0 to 28·6)	85·7 (67·8 to 108)	1·27 (0·800 to 1·68)	-3·56 (-4·31 to -2·72)	0·314 (0·260 to 0·367)	1·19 (0·983 to 1·39)	-3·91 (-5·10 to -2·81)	-5·15 (-6·62 to -3·55)
	50-69 years	24·3 (17·9 to 31·6)	163 (120 to 212)	-0·250 (-0·738 to 0·242)	-1·28 (-2·39 to -0·192)	1·18 (0·965 to 1·38)	7·92 (6·49 to 9·29)	-4·98 (-5·99 to -3·73)	-3·97 (-5·51 to -2·34)
	70+ years	6·86 (5·45 to 8·78)	228 (181 to 292)	-1·40 (-2·18 to -0·769)	-0·503 (-1·60 to 0·524)	0·964 (0·770 to 1·28)	32·1 (25·6 to 42·7)	-5·83 (-6·79 to -4·77)	-3·30 (-5·63 to -0·920)

eTable 1. All-form tuberculosis incident cases and deaths, age-standardised rates of incidence and mortality per 100,000 population, and corresponding annualized rates of change by age groups for 204 countries and territories (2021).

Location	Age group	Number of cases	Rate per 100,000 cases	Annualized rate of change (1990-2010)	Annualized rate of change (2010-2021)	Number of deaths	Rate per 100,000 deaths	Annualized deaths (1990-2010)	Annualized deaths (2010-2021)
United States of America	All Ages	8710 (7640 to 10200)	2·22 (1·94 to 2·67)	-3·35 (-3·58 to -3·12)	-0·517 (-0·878 to -0·104)	949 (881 to 1030)	0·181 (0·167 to 0·198)	-7·53 (-7·96 to -7·21)	-0·400 (-0·700 to -0·0960)
	Under 5	185 (139 to 242)	0·997 (0·748 to 1·30)	2·61 (2·26 to 2·98)	-3·57 (-4·19 to -3·08)	2·99 (2·67 to 3·44)	0·0161 (0·0144 to 0·0185)	-10·1 (-10·7 to -9·62)	-1·99 (-2·62 to -1·35)
	5-14 years	247 (165 to 367)	0·604 (0·403 to 0·898)	-0·773 (-1·26 to -0·144)	-0·0131 (-1·04 to 0·848)	1·54 (1·38 to 1·79)	0·00376 (0·00338 to 0·00438)	-9·52 (-9·99 to -9·11)	-1·46 (-1·79 to -1·05)
	15-49 years	3860 (3050 to 4920)	2·54 (2·01 to 3·23)	-3·17 (-3·58 to -2·84)	-0·952 (-1·69 to -0·312)	152 (131 to 183)	0·100 (0·0861 to 0·120)	-10·5 (-11·1 to -10·0)	-2·20 (-2·85 to -1·58)
	50-69 years	2590 (1930 to 3350)	3·14 (2·34 to 4·05)	-4·69 (-5·06 to -4·30)	-1·01 (-1·61 to -0·393)	335 (305 to 375)	0·404 (0·369 to 0·453)	-7·04 (-7·21 to -6·83)	-0·953 (-1·23 to -0·694)
	70+ years	1820 (1430 to 2360)	4·73 (3·72 to 6·14)	-4·14 (-4·40 to -3·82)	-0·240 (-0·713 to 0·139)	457 (402 to 488)	1·19 (1·05 to 1·27)	-6·60 (-6·86 to -6·45)	-1·97 (-2·24 to -1·62)
Southern Latin America	All Ages	12700 (11200 to 14800)	17·4 (15·2 to 20·3)	-2·24 (-2·55 to -1·91)	-0·776 (-1·16 to -0·364)	1930 (1630 to 2140)	2·42 (2·02 to 2·69)	-2·94 (-3·53 to -2·45)	-1·89 (-2·46 to -1·31)
	Under 5	167 (122 to 215)	3·89 (2·86 to 5·02)	-3·44 (-4·06 to -2·66)	-2·74 (-3·94 to -1·63)	10·7 (8·84 to 12·7)	0·250 (0·207 to 0·297)	-7·88 (-8·56 to -7·38)	-6·09 (-7·19 to -4·94)
	5-14 years	584 (400 to 828)	5·72 (3·91 to 8·10)	-2·82 (-3·63 to -1·87)	-1·03 (-2·09 to -0·103)	10·9 (9·33 to 12·6)	0·107 (0·0913 to 0·123)	-5·01 (-5·81 to -4·26)	-3·50 (-4·30 to -2·64)
	15-49 years	7780 (6440 to 9510)	22·4 (18·6 to 27·4)	-2·22 (-2·64 to -1·77)	-0·603 (-1·13 to -0·0961)	780 (591 to 934)	2·25 (1·70 to 2·69)	-1·95 (-3·17 to -1·15)	-2·41 (-2·99 to -1·73)
	50-69 years	2950 (2230 to 3740)	22·7 (17·1 to 28·7)	-2·95 (-3·44 to -2·39)	-1·57 (-2·09 to -1·00)	645 (549 to 728)	4·95 (4·22 to 5·60)	-4·67 (-5·13 to -4·21)	-1·71 (-2·44 to -1·01)
	70+ years	1220 (996 to 1490)	22·3 (18·1 to 27·2)	-3·11 (-3·58 to -2·67)	-2·33 (-3·05 to -1·80)	488 (442 to 519)	8·88 (8·05 to 9·43)	-4·05 (-4·39 to -3·79)	-3·53 (-4·11 to -3·02)
Argentina	All Ages	8820 (7680 to 10200)	18·3 (15·9 to 21·2)	-1·94 (-2·33 to -1·55)	-0·730 (-1·22 to -0·250)	1270 (1040 to 1440)	2·45 (1·99 to 2·79)	-2·78 (-3·56 to -2·07)	-1·44 (-2·02 to -0·718)
	Under 5	130 (93·6 to 171)	4·32 (3·12 to 5·72)	-2·80 (-3·62 to -1·86)	-2·63 (-4·11 to -1·31)	8·08 (6·42 to 9·78)	0·270 (0·214 to 0·327)	-7·87 (-8·72 to -7·27)	-5·49 (-6·60 to -4·21)
	5-14 years	477 (331 to 677)	6·63 (4·60 to 9·41)	-1·98 (-2·97 to -0·813)	-0·994 (-2·20 to 0·0349)	8·29 (6·96 to 9·71)	0·115 (0·0969 to 0·135)	-4·42 (-5·54 to -3·49)	-3·41 (-4·37 to -2·42)
	15-49 years	5670 (4620 to 6910)	24·1 (19·6 to 29·3)	-1·88 (-2·38 to -1·35)	-0·688 (-1·36 to 0·00765)	557 (413 to 681)	2·37 (1·75 to 2·89)	-1·52 (-2·86 to -0·477)	-2·45 (-3·11 to -1·64)
	50-69 years	1850 (1360 to 2370)	22·6 (16·6 to 28·9)	-2·60 (-3·18 to -1·94)	-1·41 (-2·18 to -0·523)	426 (356 to 494)	5·19 (4·34 to 6·02)	-4·06 (-4·74 to -3·48)	-0·945 (-1·72 to -0·0462)
	70+ years	689 (549 to 855)	19·3 (15·4 to 24·0)	-3·17 (-3·79 to -2·59)	-2·03 (-2·90 to -1·18)	267 (244 to 291)	7·50 (6·84 to 8·16)	-4·54 (-5·01 to -4·20)	-2·04 (-2·72 to -1·31)
Chile	All Ages	2900 (2530 to 3380)	13·4 (11·7 to 15·7)	-3·26 (-3·63 to -2·93)	-1·12 (-1·52 to -0·611)	591 (515 to 649)	2·50 (2·17 to 2·75)	-3·19 (-3·62 to -2·91)	-3·05 (-3·73 to -2·25)
	Under 5	28·0 (21·6 to 35·5)	2·58 (1·98 to 3·27)	-5·59 (-6·41 to -4·72)	-3·14 (-4·32 to -1·82)	2·35 (1·98 to 2·67)	0·216 (0·182 to 0·246)	-8·01 (-8·60 to -7·54)	-7·86 (-9·31 to -6·55)
	5-14 years	72·7 (49·3 to 100)	2·84 (1·92 to 3·91)	-6·67 (-7·58 to -5·83)	-0·969 (-2·32 to 0·522)	2·41 (2·14 to 2·72)	0·0941 (0·0833 to 0·106)	-6·56 (-7·22 to -5·78)	-3·83 (-5·50 to -2·58)

eTable 1. All-form tuberculosis incident cases and deaths, age-standardised rates of incidence and mortality per 100,000 population, and corresponding annualized rates of change by age groups for 204 countries and territories (2021).

Location	Age group	Number of cases	Rate per 100,000 cases	Annualized rate of change (1990-2010)	Annualized rate of change (2010-2021)	Number of deaths	Rate per 100,000 deaths	Annualized deaths (1990-2010)	Annualized deaths (2010-2021)
	15-49 years	1510 (1240 to 1860)	15·8 (13·0 to 19·6)	-3·63 (-4·16 to -3·19)	-0·813 (-1·36 to -0·0697)	198 (156 to 224)	2·08 (1·64 to 2·36)	-2·95 (-3·84 to -2·40)	-2·56 (-3·58 to -1·29)
	50-69 years	858 (663 to 1090)	21·0 (16·2 to 26·7)	-4·13 (-4·63 to -3·51)	-2·12 (-2·96 to -1·22)	193 (169 to 214)	4·72 (4·14 to 5·23)	-6·19 (-6·71 to -5·80)	-3·61 (-4·65 to -2·44)
	70+ years	433 (359 to 527)	27·8 (23·0 to 33·8)	-3·35 (-3·99 to -2·82)	-3·42 (-4·39 to -2·56)	196 (176 to 216)	12·6 (11·3 to 13·9)	-3·95 (-4·29 to -3·62)	-6·31 (-6·95 to -5·53)
Uruguay	All Ages	995 (851 to 1180)	26·9 (22·8 to 32·1)	-1·17 (-1·52 to -0·781)	0·148 (-0·450 to 0·644)	76·3 (67·1 to 86·7)	1·68 (1·45 to 1·96)	-3·72 (-4·28 to -2·90)	0·111 (-0·615 to 0·834)
	Under 5	8·99 (6·79 to 12·0)	4·64 (3·50 to 6·17)	-3·34 (-4·34 to -2·48)	-2·28 (-3·71 to -0·824)	0·273 (0·214 to 0·338)	0·141 (0·110 to 0·174)	-7·14 (-7·93 to -6·15)	-6·49 (-8·90 to -4·15)
	5-14 years	34·6 (23·6 to 50·1)	7·42 (5·07 to 10·8)	-1·67 (-2·60 to -0·743)	-0·514 (-1·58 to 0·943)	0·242 (0·202 to 0·292)	0·0520 (0·0434 to 0·0628)	-5·14 (-5·77 to -4·42)	-3·79 (-5·10 to -2·70)
	15-49 years	606 (482 to 748)	37·0 (29·4 to 45·6)	-0·607 (-1·11 to -0·159)	0·366 (-0·444 to 1·02)	25·1 (19·5 to 32·3)	1·53 (1·19 to 1·97)	-2·17 (-3·05 to -0·863)	-1·22 (-1·91 to -0·414)
	50-69 years	244 (181 to 299)	33·5 (24·8 to 41·1)	-2·03 (-2·53 to -1·40)	-0·506 (-1·17 to 0·191)	26·2 (22·8 to 30·0)	3·59 (3·13 to 4·11)	-5·15 (-5·88 to -4·43)	0·317 (-0·699 to 1·34)
	70+ years	101 (82·3 to 126)	27·0 (21·9 to 33·6)	-3·03 (-3·60 to -2·44)	-1·28 (-2·35 to -0·222)	24·6 (21·1 to 27·5)	6·54 (5·62 to 7·31)	-4·98 (-5·80 to -4·12)	-0·230 (-1·13 to 0·842)
Western Europe	All Ages	26200 (22900 to 30400)	5·95 (4·95 to 7·01)	-3·47 (-3·68 to -3·31)	-2·66 (-2·82 to -2·45)	3100 (2760 to 3380)	0·335 (0·299 to 0·379)	-5·02 (-5·27 to -4·80)	-4·07 (-4·40 to -3·72)
	Under 5	456 (350 to 596)	2·15 (1·65 to 2·81)	-2·02 (-2·61 to -1·57)	-3·95 (-4·51 to -3·25)	5·83 (4·32 to 7·97)	0·0275 (0·0204 to 0·0376)	-7·44 (-7·77 to -7·00)	-5·75 (-6·41 to -5·13)
	5-14 years	1290 (852 to 1930)	2·75 (1·82 to 4·12)	-2·13 (-2·69 to -1·71)	-3·30 (-3·93 to -2·75)	3·92 (2·97 to 5·14)	0·00835 (0·00633 to 0·0110)	-7·44 (-7·74 to -7·05)	-5·58 (-6·01 to -5·07)
	15-49 years	15300 (12300 to 19000)	8·12 (6·51 to 10·1)	-3·16 (-3·42 to -2·96)	-2·11 (-2·39 to -1·82)	341 (264 to 449)	0·181 (0·140 to 0·238)	-7·28 (-7·54 to -6·93)	-6·45 (-6·80 to -5·99)
	50-69 years	4960 (3710 to 6200)	4·31 (3·23 to 5·39)	-4·57 (-4·84 to -4·28)	-2·56 (-2·84 to -2·32)	636 (559 to 732)	0·553 (0·487 to 0·637)	-6·73 (-7·05 to -6·26)	-4·74 (-5·13 to -4·35)
	70+ years	4170 (3420 to 5140)	6·33 (5·20 to 7·81)	-4·51 (-4·77 to -4·23)	-3·60 (-3·98 to -3·23)	2120 (1820 to 2280)	3·22 (2·77 to 3·46)	-5·01 (-5·32 to -4·77)	-4·82 (-5·15 to -4·48)
Andorra	All Ages	4·93 (4·21 to 5·96)	4·78 (4·09 to 5·65)	-3·23 (-3·74 to -2·82)	-2·64 (-3·34 to -1·97)	0·107 (0·0805 to 0·140)	0·0677 (0·0513 to 0·0894)	-2·27 (-4·27 to -0·391)	-1·97 (-4·13 to 0·420)
	Under 5	0·0939 (0·0695 to 0·122)	3·72 (2·75 to 4·84)	-3·72 (-4·58 to -3·02)	-2·98 (-4·07 to -1·89)	0·0000664 (0·0000451 to 0·0000889)	0·00263 (0·00179 to 0·00352)	-8·59 (-10·3 to -6·66)	-6·25 (-9·25 to -3·05)
	5-14 years	0·0786 (0·0443 to 0·133)	1·03 (0·579 to 1·73)	-3·25 (-4·42 to -1·76)	-3·25 (-5·52 to -1·51)	0·0000435 (0·0000335 to 0·0000540)	0·000569 (0·000438 to 0·000707)	-6·17 (-7·88 to -4·58)	-4·87 (-7·20 to -2·64)
	15-49 years	2·13 (1·65 to 2·66)	5·16 (3·99 to 6·46)	-3·59 (-4·26 to -2·91)	-3·47 (-4·52 to -2·35)	0·0110 (0·00826 to 0·0142)	0·0267 (0·0200 to 0·0345)	-3·30 (-4·87 to -1·42)	-4·30 (-6·76 to -1·93)
	50-69 years	1·80 (1·29 to 2·36)	7·24 (5·18 to 9·48)	-3·39 (-3·86 to -2·89)	-2·63 (-3·45 to -1·99)	0·0256 (0·0178 to 0·0349)	0·103 (0·0715 to 0·141)	-4·53 (-7·02 to -2·05)	-4·44 (-7·86 to -0·888)
	70+ years	0·833 (0·669 to 1·06)	8·93 (7·17 to 11·4)	-3·22 (-3·84 to -2·53)	-2·71 (-3·45 to -1·82)	0·0700 (0·0522 to 0·0944)	0·750 (0·559 to 1·01)	-3·38 (-5·31 to -1·32)	-3·09 (-5·30 to -0·652)

eTable 1. All-form tuberculosis incident cases and deaths, age-standardised rates of incidence and mortality per 100,000 population, and corresponding annualized rates of change by age groups for 204 countries and territories (2021).

Location	Age group	Number of cases	Rate per 100,000 cases	Annualized rate of change (1990-2010)	Annualized rate of change (2010-2021)	Number of deaths	Rate per 100,000 deaths	Annualized deaths (1990-2010)	Annualized deaths (2010-2021)
Austria	All Ages	484 (427 to 554)	5·23 (4·45 to 6·14)	-3·84 (-4·19 to -3·50)	-2·45 (-2·86 to -1·94)	55·2 (49·3 to 59·8)	0·303 (0·270 to 0·336)	-5·90 (-6·34 to -5·57)	-2·83 (-3·41 to -2·18)
	Under 5	10·3 (7·67 to 14·3)	2·39 (1·77 to 3·31)	-2·27 (-3·19 to -1·38)	-1·99 (-3·33 to 0·375)	0·0640 (0·0492 to 0·0851)	0·0148 (0·0114 to 0·0197)	-8·01 (-8·51 to -7·37)	-6·86 (-8·03 to -5·94)
	5-14 years	20·0 (13·1 to 30·2)	2·31 (1·51 to 3·49)	-2·99 (-3·76 to -2·16)	-2·26 (-3·40 to -0·977)	0·0424 (0·0321 to 0·0573)	0·00490 (0·00372 to 0·00662)	-7·01 (-7·65 to -6·35)	-6·11 (-7·17 to -5·08)
	15-49 years	277 (225 to 341)	6·88 (5·57 to 8·44)	-3·93 (-4·49 to -3·44)	-1·58 (-2·28 to -0·797)	5·32 (4·03 to 7·13)	0·132 (0·0998 to 0·177)	-7·64 (-8·15 to -7·04)	-5·54 (-6·50 to -4·72)
	50-69 years	103 (76·7 to 126)	4·26 (3·19 to 5·24)	-3·94 (-4·38 to -3·58)	-3·93 (-4·66 to -3·20)	12·2 (11·1 to 13·8)	0·508 (0·462 to 0·571)	-7·10 (-7·69 to -6·63)	-5·18 (-6·05 to -4·41)
	70+ years	73·6 (60·7 to 92·2)	5·92 (4·88 to 7·41)	-4·36 (-4·97 to -3·85)	-3·21 (-4·02 to -2·46)	37·5 (32·3 to 41·3)	3·02 (2·60 to 3·32)	-6·21 (-6·67 to -5·84)	-2·58 (-3·15 to -1·86)
Belgium	All Ages	716 (616 to 824)	6·32 (5·24 to 7·35)	-2·33 (-2·64 to -2·00)	-2·23 (-2·79 to -1·69)	62·6 (55·0 to 69·6)	0·275 (0·239 to 0·316)	-3·71 (-4·22 to -3·22)	-5·20 (-5·91 to -4·57)
	Under 5	15·5 (11·2 to 20·8)	2·61 (1·89 to 3·51)	-1·36 (-2·14 to -0·474)	-2·29 (-3·41 to -0·625)	0·103 (0·0745 to 0·145)	0·0175 (0·0126 to 0·0245)	-7·04 (-7·44 to -6·47)	-6·55 (-7·47 to -5·71)
	5-14 years	38·1 (25·3 to 56·5)	2·89 (1·92 to 4·28)	-1·40 (-2·22 to -0·511)	-1·74 (-2·99 to -0·512)	0·0762 (0·0586 to 0·101)	0·00578 (0·00444 to 0·00765)	-6·42 (-7·00 to -5·77)	-5·57 (-6·36 to -4·73)
	15-49 years	441 (349 to 545)	8·82 (6·99 to 10·9)	-1·83 (-2·26 to -1·31)	-1·46 (-2·26 to -0·910)	6·60 (4·95 to 8·94)	0·132 (0·0992 to 0·179)	-5·58 (-6·06 to -5·12)	-6·66 (-7·33 to -6·04)
	50-69 years	134 (101 to 168)	4·54 (3·42 to 5·67)	-3·44 (-3·95 to -2·83)	-2·64 (-3·37 to -1·97)	15·3 (13·4 to 17·6)	0·518 (0·454 to 0·596)	-5·83 (-6·36 to -5·36)	-4·22 (-5·18 to -3·25)
	70+ years	87·7 (71·7 to 109)	5·46 (4·46 to 6·78)	-3·58 (-4·25 to -2·99)	-4·30 (-5·21 to -3·63)	40·5 (34·8 to 44·9)	2·52 (2·16 to 2·79)	-3·73 (-4·26 to -3·24)	-6·15 (-6·87 to -5·51)
Cyprus	All Ages	58·5 (51·4 to 68·5)	3·71 (3·20 to 4·37)	-3·27 (-3·59 to -3·04)	-2·62 (-3·10 to -2·19)	6·59 (5·50 to 7·83)	0·346 (0·293 to 0·404)	-5·71 (-7·91 to -3·50)	-2·92 (-4·96 to -0·476)
	Under 5	1·27 (0·960 to 1·68)	1·70 (1·28 to 2·24)	-3·47 (-4·79 to -2·36)	-2·82 (-4·05 to -1·42)	0·00977 (0·00713 to 0·0139)	0·0130 (0·00950 to 0·0185)	-7·85 (-9·71 to -6·21)	-5·54 (-7·46 to -3·72)
	5-14 years	1·68 (1·07 to 2·45)	1·17 (0·745 to 1·71)	-3·08 (-4·00 to -2·30)	-1·82 (-3·03 to -0·266)	0·00864 (0·00635 to 0·0120)	0·00601 (0·00442 to 0·00838)	-4·94 (-6·99 to -2·90)	-3·13 (-5·08 to -0·947)
	15-49 years	29·3 (23·3 to 37·6)	4·15 (3·31 to 5·32)	-3·12 (-3·48 to -2·87)	-3·37 (-4·02 to -2·80)	0·802 (0·606 to 1·08)	0·114 (0·0858 to 0·152)	-4·02 (-6·04 to -1·93)	-4·38 (-6·03 to -2·68)
	50-69 years	12·9 (9·66 to 16·3)	4·35 (3·25 to 5·47)	-4·20 (-4·59 to -3·83)	-2·48 (-3·12 to -1·90)	1·39 (1·12 to 1·74)	0·466 (0·376 to 0·586)	-7·21 (-8·89 to -4·38)	-3·99 (-6·29 to -0·912)
	70+ years	13·3 (10·7 to 16·5)	9·80 (7·93 to 12·2)	-4·89 (-5·45 to -4·40)	-2·51 (-3·34 to -1·61)	4·38 (3·58 to 5·36)	3·24 (2·64 to 3·96)	-6·69 (-9·07 to -4·63)	-4·75 (-6·94 to -2·56)
Denmark	All Ages	236 (205 to 281)	4·01 (3·39 to 4·82)	-2·93 (-3·31 to -2·58)	-2·64 (-3·16 to -2·09)	20·7 (18·2 to 22·3)	0·178 (0·158 to 0·193)	-4·40 (-4·87 to -3·99)	-4·75 (-5·54 to -3·96)
	Under 5	6·58 (4·77 to 8·76)	2·12 (1·54 to 2·82)	-2·78 (-3·75 to -1·90)	-1·89 (-3·10 to 0·624)	0·0183 (0·0139 to 0·0245)	0·00590 (0·00447 to 0·00787)	-9·68 (-10·0 to -9·38)	-6·01 (-7·17 to -4·90)
	5-14 years	12·5 (8·16 to 18·6)	1·94 (1·27 to 2·88)	-3·12 (-4·15 to -2·30)	-1·70 (-3·08 to -0·608)	0·0149 (0·0128 to 0·0179)	0·00231 (0·00200 to 0·00278)	-8·22 (-8·74 to -7·78)	-5·37 (-6·49 to -4·32)

eTable 1. All-form tuberculosis incident cases and deaths, age-standardised rates of incidence and mortality per 100,000 population, and corresponding annualized rates of change by age groups for 204 countries and territories (2021).

Location	Age group	Number of cases	Rate per 100,000 cases	Annualized rate of change (1990-2010)	Annualized rate of change (2010-2021)	Number of deaths	Rate per 100,000 deaths	Annualized deaths (1990-2010)	Annualized deaths (2010-2021)
	15-49 years	141 (112 to 177)	5·46 (4·35 to 6·88)	-2·34 (-2·82 to -1·87)	-2·47 (-3·14 to -1·74)	1·59 (1·29 to 2·00)	0·0619 (0·0502 to 0·0776)	-5·80 (-6·16 to -5·46)	-7·97 (-8·60 to -7·34)
	50-69 years	48·3 (35·4 to 61·6)	3·29 (2·41 to 4·20)	-3·33 (-3·96 to -2·87)	-2·29 (-3·11 to -1·54)	5·03 (4·50 to 5·62)	0·343 (0·307 to 0·384)	-5·55 (-6·18 to -4·91)	-5·50 (-6·58 to -4·49)
	70+ years	28·6 (23·0 to 36·4)	3·33 (2·67 to 4·24)	-3·96 (-4·44 to -3·44)	-4·67 (-5·67 to -3·76)	14·0 (12·0 to 15·3)	1·63 (1·40 to 1·78)	-3·95 (-4·44 to -3·44)	-6·55 (-7·44 to -5·82)
Finland	All Ages	232 (206 to 266)	3·68 (3·11 to 4·34)	-3·83 (-4·18 to -3·54)	-2·47 (-2·85 to -2·00)	48·8 (41·5 to 53·4)	0·375 (0·331 to 0·405)	-4·42 (-4·84 to -4·04)	-4·10 (-4·88 to -3·37)
	Under 5	4·47 (3·27 to 6·09)	1·84 (1·35 to 2·51)	-2·77 (-3·66 to -1·92)	-1·99 (-3·84 to -0·183)	0·0299 (0·0249 to 0·0341)	0·0123 (0·0103 to 0·0140)	-6·14 (-6·71 to -5·59)	-7·14 (-8·95 to -5·72)
	5-14 years	11·1 (7·30 to 16·6)	1·83 (1·21 to 2·75)	-2·55 (-3·12 to -1·96)	-1·71 (-2·94 to -0·453)	0·0371 (0·0316 to 0·0423)	0·00614 (0·00522 to 0·00699)	-5·67 (-6·38 to -4·85)	-6·02 (-7·49 to -4·85)
	15-49 years	102 (81·5 to 125)	4·36 (3·48 to 5·34)	-3·64 (-4·06 to -3·23)	-1·66 (-2·26 to -0·962)	3·65 (3·18 to 4·01)	0·156 (0·136 to 0·171)	-5·91 (-6·48 to -5·29)	-5·27 (-6·06 to -4·40)
	50-69 years	47·2 (35·2 to 58·8)	3·34 (2·49 to 4·16)	-5·34 (-5·71 to -4·88)	-3·20 (-3·97 to -2·35)	8·22 (7·57 to 8·89)	0·582 (0·536 to 0·629)	-7·24 (-7·75 to -6·77)	-5·47 (-6·33 to -4·66)
	70+ years	67·5 (55·6 to 82·4)	7·20 (5·93 to 8·80)	-4·41 (-5·05 to -3·73)	-4·98 (-5·66 to -4·15)	36·9 (29·7 to 40·7)	3·94 (3·17 to 4·35)	-5·03 (-5·53 to -4·55)	-6·29 (-7·12 to -5·40)
France	All Ages	4220 (3720 to 4880)	5·87 (4·99 to 6·89)	-4·41 (-4·75 to -4·15)	-2·26 (-2·67 to -1·81)	794 (689 to 881)	0·540 (0·468 to 0·627)	-5·41 (-5·85 to -4·98)	-5·14 (-5·78 to -4·48)
	Under 5	68·8 (51·6 to 93·4)	1·95 (1·46 to 2·64)	-4·36 (-5·25 to -3·58)	-1·79 (-2·80 to -0·597)	2·09 (1·52 to 2·88)	0·0590 (0·0429 to 0·0814)	-6·85 (-7·40 to -6·15)	-5·48 (-6·33 to -4·26)
	5-14 years	182 (117 to 267)	2·26 (1·44 to 3·31)	-4·95 (-5·83 to -4·02)	-0·678 (-1·93 to 0·765)	1·25 (0·936 to 1·64)	0·0155 (0·0116 to 0·0203)	-6·45 (-7·07 to -5·55)	-6·19 (-7·08 to -5·12)
	15-49 years	2230 (1790 to 2790)	7·87 (6·34 to 9·86)	-4·36 (-4·85 to -3·96)	-1·18 (-1·83 to -0·461)	83·2 (62·9 to 110)	0·294 (0·222 to 0·387)	-9·20 (-9·83 to -8·71)	-6·71 (-7·45 to -6·00)
	50-69 years	793 (579 to 984)	4·79 (3·49 to 5·94)	-4·80 (-5·17 to -4·27)	-2·90 (-3·61 to -2·13)	134 (111 to 164)	0·809 (0·667 to 0·989)	-6·48 (-7·11 to -5·66)	-5·80 (-6·65 to -5·00)
	70+ years	952 (784 to 1170)	9·58 (7·89 to 11·8)	-5·06 (-5·49 to -4·66)	-5·01 (-5·73 to -4·27)	573 (475 to 630)	5·77 (4·78 to 6·34)	-5·40 (-5·84 to -4·92)	-6·39 (-7·17 to -5·63)
Germany	All Ages	4560 (3970 to 5300)	5·47 (4·57 to 6·57)	-4·59 (-4·91 to -4·26)	-0·219 (-0·803 to 0·245)	494 (426 to 545)	0·258 (0·231 to 0·288)	-7·19 (-7·69 to -6·74)	-1·54 (-2·37 to -0·892)
	Under 5	88·9 (65·4 to 117)	2·20 (1·62 to 2·90)	-2·84 (-3·68 to -1·96)	-0·843 (-2·09 to 0·115)	0·637 (0·468 to 0·865)	0·0158 (0·0116 to 0·0214)	-9·38 (-9·83 to -8·95)	-4·28 (-5·59 to -3·45)
	5-14 years	146 (98·3 to 213)	1·85 (1·24 to 2·69)	-3·77 (-4·67 to -2·70)	-0·165 (-1·15 to 1·15)	0·384 (0·287 to 0·498)	0·00485 (0·00363 to 0·00629)	-9·49 (-10·0 to -8·94)	-4·45 (-5·36 to -3·67)
	15-49 years	2760 (2220 to 3560)	7·76 (6·25 to 10·0)	-4·41 (-4·87 to -3·95)	1·54 (0·604 to 2·30)	42·7 (34·7 to 54·0)	0·120 (0·0975 to 0·152)	-9·54 (-10·1 to -9·04)	-4·66 (-5·30 to -3·96)
	50-69 years	855 (641 to 1050)	3·52 (2·64 to 4·31)	-5·15 (-5·63 to -4·66)	-2·54 (-3·36 to -1·80)	112 (97·1 to 129)	0·461 (0·400 to 0·531)	-8·87 (-9·33 to -8·42)	-2·73 (-3·52 to -1·86)
	70+ years	708 (567 to 859)	5·22 (4·19 to 6·34)	-5·61 (-6·02 to -5·08)	-2·08 (-2·85 to -1·08)	338 (290 to 373)	2·49 (2·14 to 2·76)	-7·62 (-8·35 to -6·99)	-1·24 (-2·18 to -0·536)

eTable 1. All-form tuberculosis incident cases and deaths, age-standardised rates of incidence and mortality per 100,000 population, and corresponding annualized rates of change by age groups for 204 countries and territories (2021).

Location	Age group	Number of cases	Rate per 100,000 cases	Annualized rate of change (1990-2010)	Annualized rate of change (2010-2021)	Number of deaths	Rate per 100,000 deaths	Annualized deaths (1990-2010)	Annualized deaths (2010-2021)
Greece	All Ages	413 (370 to 469)	3·47 (3·00 to 4·03)	-2·33 (-2·69 to -1·93)	-2·95 (-3·47 to -2·43)	111 (101 to 119)	0·469 (0·437 to 0·494)	-1·04 (-1·40 to -0·643)	-5·34 (-6·11 to -4·66)
	Under 5	6·29 (4·65 to 8·68)	1·49 (1·10 to 2·05)	-1·86 (-2·72 to -0·912)	-2·17 (-3·40 to -1·23)	0·104 (0·0908 to 0·119)	0·0247 (0·0215 to 0·0282)	-4·88 (-5·57 to -4·26)	-3·70 (-5·41 to -2·12)
	5-14 years	13·5 (8·72 to 19·4)	1·39 (0·897 to 1·99)	-2·55 (-3·45 to -1·56)	-2·14 (-3·42 to -0·930)	0·0656 (0·0566 to 0·0775)	0·00675 (0·00582 to 0·00797)	-5·00 (-5·78 to -4·08)	-5·70 (-7·29 to -4·09)
	15-49 years	176 (143 to 219)	4·06 (3·30 to 5·05)	-3·25 (-3·73 to -2·72)	-1·76 (-2·45 to -1·09)	11·1 (10·1 to 12·1)	0·257 (0·234 to 0·280)	-2·57 (-3·07 to -2·08)	-3·70 (-4·85 to -2·60)
	50-69 years	99·9 (74·8 to 122)	3·67 (2·75 to 4·50)	-3·12 (-3·82 to -2·64)	-1·72 (-2·51 to -0·993)	22·2 (20·8 to 24·8)	0·816 (0·763 to 0·910)	-4·00 (-4·48 to -3·47)	-3·89 (-4·96 to -2·82)
	70+ years	117 (94·8 to 142)	6·77 (5·48 to 8·21)	-2·08 (-2·58 to -1·55)	-6·41 (-7·30 to -5·66)	77·6 (67·6 to 85·3)	4·48 (3·90 to 4·92)	-2·12 (-2·60 to -1·65)	-7·72 (-8·61 to -6·95)
Iceland	All Ages	13·5 (11·6 to 16·0)	3·72 (3·10 to 4·45)	-2·92 (-3·26 to -2·57)	-2·59 (-3·08 to -2·16)	1·63 (1·43 to 1·83)	0·279 (0·248 to 0·316)	-4·09 (-4·49 to -3·64)	-3·05 (-3·88 to -2·24)
	Under 5	0·376 (0·269 to 0·509)	1·71 (1·22 to 2·32)	-2·36 (-3·33 to -1·25)	-1·56 (-2·52 to -0·0879)	0·00312 (0·00246 to 0·00394)	0·0142 (0·0112 to 0·0179)	-6·92 (-7·73 to -6·07)	-4·75 (-6·05 to -3·35)
	5-14 years	0·989 (0·636 to 1·54)	2·17 (1·40 to 3·37)	-1·93 (-2·57 to -1·26)	-1·34 (-2·47 to -0·577)	0·00275 (0·00234 to 0·00328)	0·00604 (0·00514 to 0·00720)	-7·92 (-8·61 to -7·05)	-3·01 (-4·30 to -1·53)
	15-49 years	7·88 (6·05 to 10·4)	4·78 (3·67 to 6·29)	-2·66 (-3·07 to -2·17)	-2·64 (-3·27 to -1·95)	0·203 (0·155 to 0·266)	0·123 (0·0944 to 0·161)	-5·69 (-6·06 to -5·27)	-2·09 (-2·76 to -1·27)
	50-69 years	2·22 (1·65 to 2·82)	2·77 (2·06 to 3·51)	-4·35 (-4·78 to -3·95)	-2·41 (-3·11 to -1·57)	0·297 (0·266 to 0·332)	0·370 (0·331 to 0·413)	-6·66 (-7·15 to -6·17)	-3·88 (-4·92 to -2·79)
	70+ years	2·03 (1·66 to 2·49)	5·37 (4·40 to 6·57)	-3·94 (-4·60 to -3·42)	-3·73 (-4·65 to -2·90)	1·12 (0·936 to 1·27)	2·97 (2·47 to 3·37)	-4·14 (-4·63 to -3·56)	-5·25 (-6·15 to -4·33)
Ireland	All Ages	243 (208 to 284)	4·82 (4·05 to 5·69)	-2·99 (-3·42 to -2·63)	-3·45 (-3·97 to -3·02)	24·4 (21·6 to 26·7)	0·325 (0·291 to 0·357)	-5·00 (-5·47 to -4·60)	-4·96 (-5·72 to -4·13)
	Under 5	6·00 (4·32 to 8·13)	2·01 (1·45 to 2·72)	-2·53 (-3·62 to -1·44)	-2·41 (-3·74 to -0·933)	0·0585 (0·0483 to 0·0675)	0·0196 (0·0162 to 0·0226)	-5·99 (-6·71 to -5·25)	-5·88 (-7·58 to -4·33)
	5-14 years	14·5 (9·02 to 21·7)	2·07 (1·29 to 3·11)	-3·25 (-4·22 to -2·29)	-2·68 (-4·27 to -1·54)	0·0411 (0·0324 to 0·0489)	0·00588 (0·00464 to 0·00699)	-5·55 (-6·40 to -4·61)	-6·57 (-8·31 to -4·86)
	15-49 years	145 (115 to 182)	6·27 (4·99 to 7·88)	-2·79 (-3·33 to -2·25)	-3·33 (-3·92 to -2·87)	4·20 (3·44 to 4·79)	0·182 (0·149 to 0·208)	-3·44 (-4·09 to -2·91)	-5·38 (-6·59 to -4·14)
	50-69 years	45·9 (33·7 to 56·9)	4·11 (3·02 to 5·10)	-3·92 (-4·46 to -3·43)	-3·34 (-4·11 to -2·52)	5·07 (4·54 to 5·52)	0·455 (0·407 to 0·495)	-7·01 (-7·50 to -6·57)	-6·97 (-7·94 to -6·10)
	70+ years	32·0 (25·4 to 39·8)	6·15 (4·88 to 7·65)	-4·34 (-4·95 to -3·67)	-4·70 (-5·43 to -3·89)	15·0 (12·7 to 17·0)	2·88 (2·44 to 3·26)	-5·27 (-5·84 to -4·69)	-7·09 (-7·90 to -5·93)
Israel	All Ages	270 (232 to 316)	2·79 (2·37 to 3·30)	-3·31 (-3·72 to -2·88)	-3·12 (-3·49 to -2·63)	29·3 (26·3 to 32·3)	0·232 (0·209 to 0·255)	-4·84 (-5·25 to -4·41)	-4·14 (-4·99 to -3·38)
	Under 5	13·1 (9·58 to 17·3)	1·43 (1·04 to 1·88)	-2·67 (-3·52 to -1·82)	-2·26 (-3·56 to -0·207)	0·116 (0·0848 to 0·160)	0·0126 (0·00924 to 0·0174)	-8·64 (-9·00 to -8·23)	-7·14 (-8·26 to -6·12)
	5-14 years	19·2 (12·6 to 27·6)	1·13 (0·735 to 1·62)	-3·77 (-4·53 to -2·97)	-2·72 (-3·87 to -1·48)	0·0663 (0·0532 to 0·0851)	0·00388 (0·00311 to 0·00498)	-7·09 (-7·53 to -6·59)	-7·04 (-7·97 to -6·02)

eTable 1. All-form tuberculosis incident cases and deaths, age-standardised rates of incidence and mortality per 100,000 population, and corresponding annualized rates of change by age groups for 204 countries and territories (2021).

Location	Age group	Number of cases	Rate per 100,000 cases	Annualized rate of change (1990-2010)	Annualized rate of change (2010-2021)	Number of deaths	Rate per 100,000 deaths	Annualized deaths (1990-2010)	Annualized deaths (2010-2021)
	15-49 years	160 (127 to 197)	3·55 (2·83 to 4·39)	-3·08 (-3·59 to -2·47)	-3·07 (-3·64 to -2·51)	3·43 (2·73 to 4·34)	0·0764 (0·0607 to 0·0965)	-5·70 (-6·09 to -5·35)	-5·86 (-6·58 to -5·23)
	50-69 years	40·6 (30·4 to 50·8)	2·46 (1·84 to 3·08)	-3·95 (-4·40 to -3·53)	-3·31 (-4·03 to -2·46)	6·06 (5·47 to 6·77)	0·368 (0·332 to 0·411)	-6·48 (-6·92 to -6·10)	-4·73 (-5·68 to -3·72)
	70+ years	37·1 (30·2 to 46·1)	4·49 (3·66 to 5·58)	-4·45 (-5·01 to -3·85)	-4·34 (-5·13 to -3·44)	19·7 (16·5 to 21·8)	2·38 (2·00 to 2·64)	-4·92 (-5·48 to -4·36)	-5·37 (-6·29 to -4·59)
Italy	All Ages	3410 (2920 to 4040)	6·12 (4·95 to 7·46)	-3·49 (-3·76 to -3·23)	-1·88 (-2·29 to -1·54)	289 (256 to 312)	0·191 (0·176 to 0·205)	-4·27 (-4·69 to -4·00)	-3·69 (-4·22 to -3·40)
	Under 5	54·4 (41·2 to 73·1)	2·51 (1·90 to 3·37)	-1·74 (-2·48 to -1·16)	-1·63 (-2·29 to -0·879)	0·220 (0·176 to 0·252)	0·0102 (0·00810 to 0·0116)	-7·31 (-7·62 to -6·92)	-6·96 (-8·48 to -5·75)
	5-14 years	139 (89·0 to 217)	2·56 (1·64 to 4·00)	-2·28 (-2·80 to -1·87)	-2·11 (-2·81 to -1·54)	0·235 (0·210 to 0·265)	0·00432 (0·00388 to 0·00488)	-6·14 (-6·36 to -5·97)	-5·97 (-6·52 to -5·27)
	15-49 years	2070 (1600 to 2640)	8·42 (6·49 to 10·7)	-2·83 (-3·10 to -2·58)	-0·953 (-1·46 to -0·536)	22·2 (18·7 to 26·8)	0·0902 (0·0760 to 0·109)	-6·19 (-6·61 to -5·83)	-6·12 (-6·56 to -5·75)
	50-69 years	656 (491 to 834)	3·84 (2·87 to 4·88)	-5·26 (-5·63 to -4·96)	-2·17 (-2·54 to -1·79)	52·7 (49·2 to 56·6)	0·308 (0·288 to 0·332)	-7·39 (-7·75 to -7·05)	-5·24 (-5·59 to -4·86)
	70+ years	490 (396 to 630)	4·66 (3·76 to 5·99)	-4·74 (-5·10 to -4·37)	-3·42 (-3·96 to -2·88)	214 (177 to 233)	2·03 (1·69 to 2·22)	-4·56 (-5·02 to -4·29)	-4·65 (-5·11 to -4·33)
Luxembourg	All Ages	49·2 (41·5 to 59·1)	7·69 (6·27 to 9·11)	-3·34 (-3·83 to -2·90)	-1·56 (-2·03 to -0·968)	1·42 (1·23 to 1·60)	0·129 (0·115 to 0·150)	-5·77 (-6·20 to -5·35)	-5·13 (-6·31 to -4·15)
	Under 5	1·26 (0·880 to 1·71)	3·80 (2·66 to 5·18)	-2·50 (-3·66 to -1·60)	-2·22 (-3·38 to -0·449)	0·00201 (0·00162 to 0·00251)	0·00607 (0·00490 to 0·00758)	-9·01 (-9·52 to -8·43)	-6·46 (-7·89 to -5·01)
	5-14 years	3·04 (2·02 to 4·65)	4·45 (2·96 to 6·82)	-1·94 (-2·92 to -1·26)	-1·25 (-2·65 to 0·0340)	0·000771 (0·000647 to 0·000941)	0·00113 (0·000949 to 0·00138)	-11·7 (-12·2 to -11·2)	-6·57 (-7·82 to -5·08)
	15-49 years	33·6 (26·4 to 43·6)	10·6 (8·31 to 13·7)	-3·13 (-3·82 to -2·66)	-0·940 (-1·52 to -0·150)	0·157 (0·119 to 0·219)	0·0494 (0·0375 to 0·0690)	-7·67 (-8·03 to -7·32)	-7·20 (-8·13 to -6·31)
	50-69 years	7·47 (5·55 to 9·56)	4·69 (3·48 to 6·01)	-3·86 (-4·40 to -3·33)	-2·68 (-3·51 to -1·76)	0·300 (0·263 to 0·345)	0·189 (0·165 to 0·216)	-6·66 (-7·10 to -6·25)	-6·83 (-7·77 to -5·57)
	70+ years	3·80 (3·12 to 4·84)	5·72 (4·69 to 7·28)	-4·61 (-5·14 to -3·98)	-3·78 (-4·72 to -2·91)	0·956 (0·791 to 1·09)	1·44 (1·19 to 1·63)	-5·36 (-5·92 to -4·86)	-4·66 (-5·98 to -3·58)
Malta	All Ages	94·7 (77·6 to 114)	24·4 (19·4 to 29·7)	-2·12 (-2·62 to -1·71)	-0·841 (-1·48 to -0·162)	0·819 (0·750 to 0·915)	0·0902 (0·0821 to 0·103)	-4·05 (-4·43 to -3·65)	-4·03 (-4·87 to -3·17)
	Under 5	2·04 (1·46 to 2·76)	9·26 (6·65 to 12·6)	-2·50 (-3·58 to -1·38)	-2·20 (-3·77 to -0·295)	0·00139 (0·00106 to 0·00189)	0·00631 (0·00480 to 0·00857)	-5·85 (-6·55 to -5·19)	-5·84 (-6·93 to -4·58)
	5-14 years	4·97 (3·29 to 7·57)	11·8 (7·83 to 18·0)	-1·54 (-2·45 to -0·942)	-1·38 (-2·51 to 0·149)	0·00144 (0·00119 to 0·00168)	0·00343 (0·00284 to 0·00400)	-6·37 (-6·92 to -5·86)	-0·834 (-2·88 to 1·25)
	15-49 years	71·3 (54·3 to 89·7)	36·6 (27·9 to 46·1)	-1·49 (-2·06 to -0·982)	0·440 (-0·275 to 1·27)	0·0968 (0·0777 to 0·125)	0·0497 (0·0399 to 0·0640)	-4·64 (-4·99 to -4·36)	-4·72 (-5·35 to -3·92)
	50-69 years	9·84 (7·28 to 12·9)	8·80 (6·51 to 11·5)	-3·81 (-4·28 to -3·36)	-2·88 (-3·59 to -2·14)	0·164 (0·150 to 0·192)	0·147 (0·134 to 0·172)	-6·67 (-7·22 to -6·05)	-5·77 (-6·71 to -4·67)
	70+ years	6·62 (5·23 to 8·45)	9·22 (7·28 to 11·8)	-4·35 (-4·99 to -3·79)	-4·79 (-5·84 to -3·93)	0·555 (0·481 to 0·620)	0·773 (0·670 to 0·864)	-5·70 (-6·22 to -5·21)	-6·87 (-7·76 to -5·97)

eTable 1. All-form tuberculosis incident cases and deaths, age-standardised rates of incidence and mortality per 100,000 population, and corresponding annualized rates of change by age groups for 204 countries and territories (2021).

Location	Age group	Number of cases	Rate per 100,000 cases	Annualized rate of change (1990-2010)	Annualized rate of change (2010-2021)	Number of deaths	Rate per 100,000 deaths	Annualized deaths (1990-2010)	Annualized deaths (2010-2021)
Monaco	All Ages	2·36 (2·09 to 2·68)	4·72 (4·08 to 5·56)	-3·17 (-3·43 to -2·87)	-1·54 (-2·10 to -1·09)	0·584 (0·472 to 0·720)	0·608 (0·493 to 0·748)	-4·42 (-5·80 to -2·83)	-1·92 (-4·09 to 0·239)
	Under 5	0·0233 (0·0178 to 0·0299)	1·44 (1·10 to 1·85)	-2·99 (-3·95 to -2·19)	-2·75 (-3·99 to -1·15)	0·000278 (0·000203 to 0·000355)	0·0172 (0·0125 to 0·0219)	-9·09 (-10·4 to -7·61)	-5·80 (-8·55 to -3·23)
	5-14 years	0·0321 (0·0210 to 0·0484)	0·954 (0·625 to 1·44)	-3·10 (-3·78 to -2·45)	-4·23 (-5·57 to -2·92)	0·000148 (0·000116 to 0·000190)	0·00441 (0·00344 to 0·00565)	-5·19 (-6·63 to -3·71)	-5·43 (-7·96 to -3·09)
	15-49 years	0·790 (0·629 to 1·02)	5·62 (4·48 to 7·26)	-3·08 (-3·54 to -2·71)	-1·87 (-2·54 to -1·17)	0·0385 (0·0276 to 0·0511)	0·274 (0·197 to 0·363)	-4·03 (-5·39 to -2·33)	-3·64 (-6·22 to -1·36)
	50-69 years	0·699 (0·514 to 0·863)	5·97 (4·38 to 7·37)	-3·15 (-3·63 to -2·64)	-1·32 (-2·19 to -0·626)	0·120 (0·0930 to 0·153)	1·02 (0·794 to 1·30)	-5·29 (-6·93 to -3·27)	-2·55 (-5·16 to -0·314)
	70+ years	0·819 (0·681 to 1·02)	11·5 (9·57 to 14·4)	-3·15 (-3·59 to -2·73)	-1·83 (-2·57 to -0·915)	0·426 (0·337 to 0·536)	5·98 (4·74 to 7·53)	-4·38 (-6·05 to -2·68)	-2·70 (-4·82 to -0·413)
Netherlands	All Ages	742 (636 to 850)	4·25 (3·56 to 4·94)	-3·47 (-3·81 to -3·13)	-2·25 (-2·72 to -1·84)	89·9 (80·0 to 101)	0·271 (0·240 to 0·318)	-4·72 (-5·24 to -4·29)	-3·25 (-3·87 to -2·68)
	Under 5	13·9 (10·3 to 18·4)	1·61 (1·20 to 2·14)	-3·99 (-4·80 to -3·07)	-1·91 (-3·21 to -0·571)	0·170 (0·120 to 0·240)	0·0197 (0·0139 to 0·0279)	-6·88 (-7·35 to -6·14)	-5·27 (-6·04 to -4·43)
	5-14 years	29·3 (19·1 to 44·2)	1·61 (1·05 to 2·43)	-5·81 (-6·88 to -5·09)	-1·48 (-2·54 to -0·465)	0·135 (0·101 to 0·179)	0·00742 (0·00555 to 0·00981)	-6·28 (-6·91 to -5·60)	-4·48 (-5·22 to -3·60)
	15-49 years	446 (358 to 552)	6·02 (4·84 to 7·45)	-3·17 (-3·60 to -2·75)	-1·58 (-2·23 to -1·01)	9·51 (7·01 to 13·3)	0·128 (0·0947 to 0·179)	-7·60 (-8·09 to -7·18)	-5·37 (-5·94 to -4·74)
	50-69 years	138 (102 to 172)	2·95 (2·18 to 3·68)	-3·01 (-3·53 to -2·48)	-3·31 (-3·93 to -2·72)	19·7 (16·8 to 23·6)	0·423 (0·361 to 0·506)	-5·84 (-6·37 to -5·27)	-4·95 (-5·67 to -4·12)
	70+ years	115 (93·8 to 143)	4·67 (3·82 to 5·80)	-3·56 (-3·94 to -3·06)	-3·93 (-4·60 to -3·16)	60·3 (51·0 to 65·6)	2·46 (2·08 to 2·67)	-4·47 (-4·95 to -4·08)	-4·99 (-5·55 to -4·40)
Norway	All Ages	248 (212 to 286)	4·20 (3·41 to 4·91)	-2·20 (-2·72 to -1·83)	-3·44 (-3·75 to -3·08)	46·4 (40·3 to 50·5)	0·421 (0·368 to 0·459)	-3·27 (-3·61 to -3·08)	-2·00 (-2·48 to -1·55)
	Under 5	3·87 (2·84 to 5·25)	1·38 (1·01 to 1·87)	-3·76 (-4·41 to -3·06)	-0·983 (-1·71 to -0·218)	0·0423 (0·0313 to 0·0533)	0·0151 (0·0111 to 0·0189)	-6·99 (-7·40 to -6·63)	-1·82 (-2·55 to -1·09)
	5-14 years	12·1 (7·30 to 19·0)	1·88 (1·14 to 2·95)	-1·59 (-2·50 to -0·812)	-1·83 (-2·44 to -1·24)	0·0370 (0·0250 to 0·0513)	0·00576 (0·00388 to 0·00799)	-4·79 (-5·14 to -4·31)	-2·38 (-2·88 to -1·78)
	15-49 years	137 (104 to 176)	5·48 (4·16 to 7·06)	0·0657 (-0·323 to 0·337)	-4·41 (-4·74 to -4·12)	2·30 (1·71 to 3·01)	0·0922 (0·0686 to 0·120)	-4·42 (-4·71 to -4·03)	-3·56 (-4·05 to -3·04)
	50-69 years	35·7 (26·5 to 45·3)	2·73 (2·03 to 3·47)	-6·32 (-6·81 to -5·75)	-2·79 (-3·20 to -2·40)	6·09 (5·58 to 6·66)	0·467 (0·427 to 0·511)	-5·46 (-5·66 to -5·23)	-4·28 (-4·78 to -3·74)
	70+ years	59·9 (49·4 to 74·9)	8·65 (7·14 to 10·8)	-3·82 (-4·42 to -3·17)	-2·63 (-3·07 to -2·06)	37·9 (32·1 to 41·5)	5·48 (4·64 to 6·00)	-2·43 (-2·82 to -2·24)	-3·23 (-3·71 to -2·75)
Portugal	All Ages	1410 (1220 to 1640)	11·5 (9·82 to 13·5)	-3·62 (-4·02 to -3·22)	-3·33 (-3·97 to -2·84)	270 (241 to 306)	1·28 (1·09 to 1·51)	-2·79 (-3·37 to -2·07)	-3·23 (-3·98 to -2·51)
	Under 5	10·8 (7·90 to 14·3)	2·55 (1·86 to 3·36)	-5·97 (-6·92 to -4·89)	-2·65 (-3·84 to -0·288)	0·403 (0·282 to 0·570)	0·0947 (0·0664 to 0·134)	-10·9 (-11·7 to -9·48)	-2·54 (-3·42 to -1·56)
	5-14 years	34·2 (22·0 to 50·7)	3·65 (2·35 to 5·41)	-5·65 (-6·59 to -4·74)	-2·97 (-4·49 to -2·06)	0·292 (0·204 to 0·403)	0·0312 (0·0218 to 0·0431)	-10·1 (-10·8 to -8·74)	-3·16 (-4·01 to -2·16)

eTable 1. All-form tuberculosis incident cases and deaths, age-standardised rates of incidence and mortality per 100,000 population, and corresponding annualized rates of change by age groups for 204 countries and territories (2021).

Location	Age group	Number of cases	Rate per 100,000 cases	Annualized rate of change (1990-2010)	Annualized rate of change (2010-2021)	Number of deaths	Rate per 100,000 deaths	Annualized deaths (1990-2010)	Annualized deaths (2010-2021)
	15-49 years	708 (566 to 902)	15·5 (12·4 to 19·7)	-3·83 (-4·36 to -3·22)	-4·15 (-4·91 to -3·36)	50·6 (35·5 to 70·9)	1·11 (0·774 to 1·55)	-2·24 (-3·18 to -0·550)	-6·65 (-7·44 to -5·86)
	50-69 years	379 (289 to 474)	13·1 (9·98 to 16·4)	-3·18 (-3·60 to -2·78)	-2·50 (-3·28 to -1·63)	68·6 (58·1 to 81·4)	2·37 (2·01 to 2·81)	-5·06 (-5·68 to -4·21)	-3·66 (-4·53 to -2·75)
	70+ years	274 (227 to 340)	15·5 (12·9 to 19·2)	-3·71 (-4·27 to -3·21)	-2·45 (-3·28 to -1·49)	150 (131 to 167)	8·48 (7·41 to 9·41)	-4·06 (-4·61 to -3·56)	-3·55 (-4·32 to -2·76)
San Marino	All Ages	0·844 (0·749 to 0·971)	2·05 (1·78 to 2·45)	-2·82 (-3·21 to -2·48)	-1·19 (-1·71 to -0·721)	0·125 (0·0798 to 0·171)	0·144 (0·0966 to 0·195)	-3·60 (-4·94 to -2·18)	-1·19 (-4·10 to 1·92)
	Under 5	0·0108 (0·00781 to 0·0142)	0·892 (0·646 to 1·18)	-3·03 (-3·81 to -2·16)	-2·73 (-4·18 to -1·64)	0·000106 (0·0000778 to 0·000146)	0·00879 (0·00643 to 0·0121)	-10·0 (-11·7 to -8·18)	-4·15 (-7·24 to -1·26)
	5-14 years	0·0144 (0·00949 to 0·0216)	0·452 (0·298 to 0·678)	-3·73 (-4·67 to -2·87)	-3·44 (-5·08 to -2·18)	0·0000534 (0·0000397 to 0·0000744)	0·00167 (0·00125 to 0·00233)	-7·36 (-8·84 to -5·84)	-4·05 (-6·38 to -1·14)
	15-49 years	0·335 (0·275 to 0·422)	2·44 (2·00 to 3·07)	-2·92 (-3·59 to -2·44)	-1·97 (-2·65 to -1·31)	0·00655 (0·00465 to 0·00901)	0·0477 (0·0338 to 0·0656)	-4·85 (-5·93 to -3·75)	-4·17 (-6·48 to -1·54)
	50-69 years	0·232 (0·172 to 0·291)	2·47 (1·83 to 3·10)	-2·96 (-3·41 to -2·46)	-0·942 (-1·84 to -0·109)	0·0188 (0·0123 to 0·0274)	0·200 (0·131 to 0·292)	-5·23 (-6·55 to -4·01)	-3·53 (-7·17 to -0·220)
	70+ years	0·251 (0·206 to 0·314)	4·83 (3·96 to 6·02)	-3·25 (-3·73 to -2·76)	-1·13 (-1·85 to -0·373)	0·0996 (0·0608 to 0·137)	1·91 (1·17 to 2·64)	-4·67 (-6·03 to -3·03)	-2·22 (-5·20 to 0·882)
Spain	All Ages	2850 (2500 to 3340)	5·73 (4·86 to 6·77)	-4·30 (-4·65 to -3·87)	-4·01 (-4·57 to -3·48)	350 (312 to 389)	0·357 (0·309 to 0·404)	-5·69 (-6·18 to -5·22)	-4·32 (-5·03 to -3·56)
	Under 5	26·7 (16·8 to 38·0)	1·45 (0·911 to 2·07)	-4·89 (-6·11 to -3·65)	-4·52 (-5·90 to -3·28)	0·597 (0·419 to 0·856)	0·0324 (0·0227 to 0·0465)	-8·71 (-9·17 to -8·25)	-5·31 (-6·19 to -4·34)
	5-14 years	116 (70·8 to 171)	2·49 (1·53 to 3·68)	-3·88 (-5·17 to -2·38)	-3·80 (-5·02 to -2·76)	0·464 (0·338 to 0·635)	0·0100 (0·00727 to 0·0137)	-8·98 (-9·36 to -8·62)	-5·28 (-6·08 to -4·34)
	15-49 years	1510 (1220 to 1910)	7·54 (6·09 to 9·57)	-4·66 (-5·19 to -4·08)	-4·29 (-4·89 to -3·68)	44·4 (32·1 to 61·5)	0·222 (0·161 to 0·308)	-8·27 (-8·70 to -7·83)	-8·13 (-8·99 to -7·23)
	50-69 years	711 (505 to 905)	5·78 (4·10 to 7·35)	-4·09 (-4·67 to -3·44)	-3·06 (-3·94 to -2·16)	77·8 (65·5 to 90·3)	0·632 (0·532 to 0·733)	-7·28 (-7·87 to -6·69)	-4·91 (-5·86 to -3·96)
	70+ years	491 (386 to 620)	7·25 (5·71 to 9·16)	-4·34 (-5·05 to -3·84)	-3·49 (-4·37 to -2·46)	227 (191 to 249)	3·35 (2·82 to 3·68)	-5·15 (-5·63 to -4·71)	-4·97 (-5·76 to -4·26)
Sweden	All Ages	662 (545 to 811)	6·59 (5·18 to 8·29)	-1·35 (-1·80 to -0·891)	-0·913 (-1·44 to -0·268)	56·5 (48·0 to 63·3)	0·224 (0·194 to 0·254)	-4·60 (-4·97 to -4·28)	-5·08 (-5·97 to -4·33)
	Under 5	16·9 (12·2 to 22·9)	2·90 (2·09 to 3·92)	-0·376 (-1·29 to 0·389)	0·141 (-1·12 to 1·04)	0·0661 (0·0445 to 0·0936)	0·0113 (0·00762 to 0·0160)	-5·90 (-6·88 to -5·03)	-7·37 (-8·20 to -6·47)
	5-14 years	44·6 (28·2 to 69·9)	3·60 (2·28 to 5·65)	1·48 (0·589 to 2·13)	0·461 (-0·634 to 1·55)	0·0339 (0·0229 to 0·0470)	0·00274 (0·00185 to 0·00380)	-5·10 (-5·96 to -3·87)	-7·61 (-8·54 to -6·18)
	15-49 years	414 (301 to 536)	9·14 (6·64 to 11·8)	0·782 (0·344 to 1·21)	-0·135 (-0·964 to 0·678)	2·35 (1·50 to 3·39)	0·0518 (0·0332 to 0·0749)	-6·74 (-7·47 to -5·93)	-4·92 (-5·65 to -3·98)
	50-69 years	84·9 (62·9 to 111)	3·49 (2·59 to 4·58)	-4·33 (-4·82 to -3·84)	-0·776 (-1·55 to 0·0628)	5·66 (4·82 to 6·65)	0·233 (0·198 to 0·274)	-6·61 (-7·05 to -6·05)	-5·83 (-6·79 to -4·89)
	70+ years	102 (84·4 to 126)	6·39 (5·29 to 7·91)	-4·14 (-4·55 to -3·65)	-4·37 (-5·29 to -3·43)	48·4 (40·1 to 54·2)	3·03 (2·51 to 3·40)	-4·30 (-4·68 to -3·95)	-6·58 (-7·54 to -5·82)

eTable 1. All-form tuberculosis incident cases and deaths, age-standardised rates of incidence and mortality per 100,000 population, and corresponding annualized rates of change by age groups for 204 countries and territories (2021).

Location	Age group	Number of cases	Rate per 100,000 cases	Annualized rate of change (1990-2010)	Annualized rate of change (2010-2021)	Number of deaths	Rate per 100,000 deaths	Annualized deaths (1990-2010)	Annualized deaths (2010-2021)
Switzerland	All Ages	442 (385 to 520)	5·12 (4·25 to 6·05)	-3·53 (-3·94 to -3·24)	-1·71 (-2·13 to -1·33)	33·4 (29·0 to 36·8)	0·174 (0·154 to 0·197)	-5·15 (-5·58 to -4·79)	-5·24 (-5·98 to -4·62)
	Under 5	9·43 (6·71 to 12·6)	2·13 (1·52 to 2·84)	-2·37 (-3·35 to -1·29)	-1·08 (-2·56 to 0·419)	0·0476 (0·0369 to 0·0648)	0·0108 (0·00834 to 0·0147)	-9·57 (-9·94 to -9·21)	-6·78 (-7·57 to -5·72)
	5-14 years	19·5 (12·8 to 29·5)	2·19 (1·44 to 3·31)	-3·31 (-4·26 to -2·32)	0·123 (-1·11 to 0·940)	0·0374 (0·0293 to 0·0480)	0·00420 (0·00329 to 0·00539)	-7·98 (-8·39 to -7·58)	-6·18 (-7·18 to -5·15)
	15-49 years	287 (236 to 357)	7·16 (5·90 to 8·92)	-3·07 (-3·61 to -2·67)	-0·913 (-1·56 to -0·371)	3·03 (2·24 to 4·14)	0·0756 (0·0559 to 0·103)	-6·93 (-7·24 to -6·61)	-7·78 (-8·47 to -7·00)
	50-69 years	70·6 (51·9 to 88·2)	3·02 (2·22 to 3·77)	-4·39 (-4·82 to -3·94)	-2·92 (-3·64 to -2·13)	6·25 (5·48 to 7·47)	0·267 (0·235 to 0·320)	-6·81 (-7·34 to -6·22)	-6·76 (-7·71 to -5·81)
	70+ years	56·2 (46·0 to 70·8)	4·51 (3·69 to 5·68)	-4·70 (-5·16 to -4·12)	-3·28 (-4·06 to -2·52)	24·0 (19·4 to 26·4)	1·92 (1·56 to 2·12)	-5·07 (-5·65 to -4·67)	-5·92 (-6·80 to -5·11)
United Kingdom	All Ages	4800 (3940 to 5620)	7·49 (6·00 to 8·84)	-1·53 (-1·85 to -1·27)	-4·55 (-4·87 to -4·26)	315 (290 to 339)	0·259 (0·237 to 0·287)	-3·46 (-3·63 to -3·34)	-4·08 (-4·32 to -3·89)
	Under 5	95·0 (70·8 to 127)	2·60 (1·94 to 3·47)	1·54 (0·772 to 2·05)	-9·34 (-9·94 to -8·30)	1·04 (0·780 to 1·41)	0·0286 (0·0213 to 0·0386)	-5·02 (-5·33 to -4·50)	-6·56 (-6·97 to -6·10)
	5-14 years	428 (265 to 647)	5·27 (3·26 to 7·96)	1·65 (1·00 to 2·13)	-6·45 (-7·36 to -5·55)	0·686 (0·510 to 0·894)	0·00843 (0·00627 to 0·0110)	-4·71 (-4·94 to -4·24)	-5·35 (-6·02 to -4·95)
	15-49 years	3150 (2360 to 3980)	10·4 (7·79 to 13·1)	-1·06 (-1·37 to -0·812)	-4·45 (-4·86 to -4·05)	42·8 (34·2 to 54·6)	0·141 (0·113 to 0·180)	-3·67 (-3·83 to -3·43)	-5·52 (-5·76 to -5·32)
	50-69 years	677 (492 to 868)	4·09 (2·97 to 5·24)	-4·84 (-5·15 to -4·52)	-1·70 (-1·99 to -1·46)	76·0 (70·1 to 83·4)	0·458 (0·423 to 0·503)	-5·10 (-5·23 to -4·92)	-4·95 (-5·21 to -4·69)
	70+ years	443 (362 to 560)	4·85 (3·96 to 6·12)	-3·73 (-4·11 to -3·36)	-2·69 (-3·05 to -2·37)	194 (175 to 206)	2·12 (1·91 to 2·26)	-3·10 (-3·34 to -2·96)	-4·75 (-4·96 to -4·53)
Latin America and Caribbean	All Ages	207000 (181000 to 239000)	33·1 (29·0 to 38·0)	-2·25 (-2·51 to -1·94)	-0·870 (-1·17 to -0·506)	24200 (20600 to 29100)	3·87 (3·30 to 4·65)	-4·17 (-4·59 to -3·70)	-2·28 (-2·96 to -1·45)
	Under 5	4040 (3320 to 5020)	8·54 (7·01 to 10·6)	-4·95 (-5·26 to -4·56)	-2·46 (-2·90 to -2·04)	552 (442 to 709)	1·17 (0·934 to 1·50)	-8·14 (-8·96 to -7·42)	-5·60 (-7·12 to -3·88)
	5-14 years	7520 (5280 to 10600)	7·84 (5·50 to 11·0)	-4·30 (-4·74 to -3·71)	-1·37 (-1·99 to -0·826)	266 (219 to 352)	0·277 (0·228 to 0·367)	-6·62 (-7·35 to -5·91)	-4·35 (-5·41 to -3·14)
	15-49 years	125000 (104000 to 153000)	40·0 (33·5 to 49·0)	-2·47 (-2·79 to -2·12)	-1·01 (-1·47 to -0·566)	10100 (8190 to 12500)	3·23 (2·62 to 4·01)	-4·17 (-4·77 to -3·48)	-2·89 (-3·46 to -2·17)
	50-69 years	51800 (39400 to 66000)	49·6 (37·7 to 63·2)	-2·56 (-2·88 to -2·23)	-1·70 (-2·25 to -1·16)	8040 (6850 to 9630)	7·69 (6·56 to 9·22)	-5·25 (-5·60 to -4·84)	-3·10 (-3·85 to -2·23)
	70+ years	18600 (14900 to 23400)	54·0 (43·1 to 67·7)	-3·62 (-3·90 to -3·34)	-2·82 (-3·15 to -2·39)	5230 (4650 to 6090)	15·2 (13·5 to 17·7)	-5·67 (-5·94 to -5·35)	-4·09 (-4·99 to -3·05)
Andean Latin America	All Ages	43800 (37700 to 51400)	65·9 (57·4 to 76·7)	-4·31 (-4·74 to -3·95)	-1·36 (-1·79 to -0·938)	4710 (3820 to 6040)	7·56 (6·14 to 9·72)	-5·95 (-6·58 to -5·24)	-4·29 (-6·29 to -2·41)
	Under 5	972 (773 to 1180)	15·8 (12·6 to 19·2)	-6·03 (-6·57 to -5·31)	-4·28 (-5·30 to -3·51)	98·6 (75·4 to 126)	1·60 (1·22 to 2·05)	-10·3 (-11·5 to -9·35)	-9·62 (-11·8 to -7·36)
	5-14 years	2220 (1620 to 3120)	18·6 (13·6 to 26·1)	-5·99 (-6·60 to -5·12)	-2·96 (-3·98 to -2·04)	61·6 (47·6 to 78·0)	0·516 (0·398 to 0·653)	-8·94 (-9·77 to -7·84)	-7·32 (-9·58 to -5·12)

eTable 1. All-form tuberculosis incident cases and deaths, age-standardised rates of incidence and mortality per 100,000 population, and corresponding annualized rates of change by age groups for 204 countries and territories (2021).

Location	Age group	Number of cases	Rate per 100,000 cases	Annualized rate of change (1990-2010)	Annualized rate of change (2010-2021)	Number of deaths	Rate per 100,000 deaths	Annualized deaths (1990-2010)	Annualized deaths (2010-2021)
	15-49 years	27100 (22400 to 34600)	77·6 (64·1 to 99·0)	-4·60 (-5·08 to -4·14)	-1·47 (-1·99 to -0·858)	1790 (1440 to 2260)	5·12 (4·11 to 6·47)	-6·13 (-6·90 to -5·19)	-5·08 (-6·99 to -3·38)
	50-69 years	9150 (6870 to 11400)	93·6 (70·3 to 117)	-4·33 (-4·74 to -3·78)	-1·72 (-2·22 to -1·18)	1420 (1120 to 1810)	14·5 (11·4 to 18·5)	-7·00 (-7·77 to -6·21)	-4·71 (-6·90 to -2·45)
	70+ years	4270 (3470 to 5300)	130 (106 to 161)	-5·09 (-5·48 to -4·68)	-1·98 (-2·59 to -1·47)	1340 (1080 to 1740)	40·8 (32·8 to 53·1)	-6·47 (-7·21 to -5·70)	-4·76 (-6·74 to -2·61)
Bolivia (Plurinational State of)	All Ages	8220 (7150 to 9220)	74·1 (65·1 to 83·8)	-3·65 (-4·15 to -3·17)	-2·91 (-3·55 to -2·34)	1500 (1030 to 2080)	15·7 (10·8 to 21·6)	-5·95 (-7·21 to -4·88)	-3·94 (-5·88 to -1·60)
	Under 5	257 (204 to 321)	21·6 (17·1 to 26·9)	-5·52 (-6·16 to -4·80)	-4·87 (-6·25 to -3·41)	42·8 (28·9 to 57·6)	3·58 (2·42 to 4·83)	-9·66 (-11·3 to -7·95)	-9·35 (-12·0 to -6·92)
	5-14 years	469 (327 to 669)	20·5 (14·3 to 29·2)	-5·09 (-5·93 to -4·31)	-4·40 (-5·82 to -3·06)	20·0 (13·0 to 29·0)	0·872 (0·568 to 1·27)	-8·59 (-10·5 to -7·12)	-7·61 (-9·71 to -4·93)
	15-49 years	4660 (3850 to 5780)	74·5 (61·5 to 92·5)	-4·13 (-4·66 to -3·51)	-3·23 (-4·07 to -2·47)	473 (324 to 684)	7·56 (5·17 to 10·9)	-6·67 (-8·13 to -5·39)	-4·63 (-7·30 to -1·75)
	50-69 years	1960 (1470 to 2440)	123 (92·3 to 153)	-3·22 (-3·83 to -2·62)	-3·18 (-4·10 to -2·35)	503 (346 to 680)	31·6 (21·7 to 42·7)	-6·47 (-7·74 to -5·18)	-4·69 (-7·06 to -2·16)
	70+ years	871 (689 to 1120)	188 (149 to 242)	-4·09 (-4·68 to -3·52)	-3·51 (-4·50 to -2·62)	464 (317 to 657)	100 (68·5 to 142)	-5·57 (-6·93 to -4·42)	-4·28 (-6·13 to -1·93)
Ecuador	All Ages	8790 (7600 to 10300)	48·6 (42·4 to 56·8)	-3·77 (-4·17 to -3·34)	-0·625 (-1·26 to 0·0375)	621 (514 to 752)	3·64 (3·02 to 4·38)	-4·73 (-5·39 to -4·21)	-7·96 (-9·45 to -6·44)
	Under 5	165 (131 to 207)	9·92 (7·91 to 12·5)	-5·39 (-6·13 to -4·66)	-4·19 (-5·48 to -2·93)	10·1 (7·51 to 13·4)	0·609 (0·452 to 0·806)	-8·26 (-9·10 to -7·31)	-10·9 (-13·3 to -8·32)
	5-14 years	364 (252 to 499)	10·7 (7·40 to 14·6)	-6·49 (-7·22 to -5·50)	-2·17 (-3·33 to -0·830)	7·45 (6·07 to 8·87)	0·219 (0·178 to 0·260)	-7·67 (-8·49 to -6·91)	-11·2 (-12·6 to -10·1)
	15-49 years	5660 (4560 to 7100)	60·3 (48·5 to 75·6)	-3·75 (-4·37 to -3·17)	-0·680 (-1·50 to 0·172)	290 (230 to 369)	3·09 (2·45 to 3·93)	-4·15 (-5·23 to -3·43)	-8·73 (-10·1 to -7·42)
	50-69 years	1770 (1330 to 2250)	66·4 (49·8 to 84·5)	-4·02 (-4·65 to -3·44)	-1·24 (-2·15 to -0·322)	154 (125 to 191)	5·78 (4·67 to 7·14)	-6·73 (-7·40 to -6·00)	-8·80 (-10·8 to -6·77)
	70+ years	827 (679 to 1040)	88·4 (72·6 to 112)	-5·01 (-5·46 to -4·40)	-2·37 (-3·50 to -1·57)	159 (130 to 195)	17·0 (14·0 to 20·8)	-6·61 (-7·10 to -6·13)	-8·80 (-10·9 to -6·80)
Peru	All Ages	26700 (22700 to 31600)	72·3 (61·9 to 84·6)	-4·64 (-5·16 to -4·18)	-1·08 (-1·66 to -0·419)	2580 (1920 to 3520)	7·31 (5·43 to 10·0)	-6·47 (-7·50 to -5·42)	-3·28 (-6·52 to -0·647)
	Under 5	550 (432 to 669)	16·7 (13·1 to 20·3)	-6·33 (-7·22 to -5·40)	-4·11 (-5·60 to -2·88)	45·7 (33·6 to 65·8)	1·39 (1·02 to 1·99)	-11·1 (-12·7 to -9·91)	-9·49 (-12·3 to -6·16)
	5-14 years	1390 (973 to 1990)	22·3 (15·6 to 32·0)	-6·06 (-6·78 to -4·98)	-2·64 (-4·17 to -1·11)	34·2 (23·5 to 48·0)	0·548 (0·377 to 0·770)	-9·63 (-10·9 to -8·33)	-5·91 (-9·08 to -2·74)
	15-49 years	16800 (13600 to 21500)	87·0 (70·5 to 111)	-4·95 (-5·63 to -4·46)	-1·18 (-1·95 to -0·351)	1030 (765 to 1360)	5·32 (3·96 to 7·06)	-6·73 (-7·79 to -5·41)	-3·94 (-6·66 to -1·34)
	50-69 years	5420 (4120 to 6770)	98·1 (74·5 to 123)	-4·80 (-5·31 to -4·11)	-1·24 (-1·97 to -0·484)	762 (515 to 1050)	13·8 (9·33 to 19·0)	-7·54 (-8·96 to -6·24)	-3·47 (-7·62 to -0·278)
	70+ years	2570 (2050 to 3150)	137 (109 to 168)	-5·52 (-5·99 to -5·02)	-1·09 (-2·06 to -0·330)	714 (513 to 1010)	37·9 (27·2 to 53·7)	-7·02 (-8·12 to -5·63)	-3·62 (-6·87 to -0·903)

eTable 1. All-form tuberculosis incident cases and deaths, age-standardised rates of incidence and mortality per 100,000 population, and corresponding annualized rates of change by age groups for 204 countries and territories (2021).

Location	Age group	Number of cases	Rate per 100,000 cases	Annualized rate of change (1990-2010)	Annualized rate of change (2010-2021)	Number of deaths	Rate per 100,000 deaths	Annualized deaths (1990-2010)	Annualized deaths (2010-2021)
Caribbean	All Ages	19400 (17100 to 22000)	39·9 (35·0 to 45·0)	-1·26 (-1·56 to -0·974)	-1·22 (-1·60 to -0·820)	3380 (2230 to 7970)	6·75 (4·46 to 15·8)	-2·97 (-4·19 to -1·97)	-2·50 (-4·61 to -0·849)
	Under 5	1070 (862 to 1350)	27·8 (22·3 to 35·0)	-2·83 (-3·36 to -2·40)	-2·11 (-2·97 to -1·04)	249 (173 to 395)	6·43 (4·48 to 10·2)	-5·36 (-6·52 to -3·88)	-3·91 (-6·60 to -1·33)
	5-14 years	1450 (995 to 2020)	19·0 (13·0 to 26·4)	-2·18 (-2·73 to -1·55)	-1·75 (-2·89 to -0·904)	85·3 (56·4 to 175)	1·12 (0·739 to 2·30)	-2·45 (-4·12 to -1·05)	-2·28 (-4·69 to -0·236)
	15-49 years	11700 (9630 to 14100)	48·8 (40·2 to 58·9)	-0·953 (-1·31 to -0·626)	-1·12 (-1·60 to -0·603)	1530 (996 to 3680)	6·41 (4·16 to 15·3)	-2·57 (-3·80 to -1·38)	-3·30 (-5·31 to -1·80)
	50-69 years	3840 (3060 to 4720)	43·6 (34·8 to 53·6)	-1·43 (-1·73 to -1·14)	-1·18 (-1·79 to -0·653)	960 (616 to 2530)	10·9 (6·99 to 28·7)	-3·44 (-4·71 to -2·26)	-2·37 (-4·14 to -0·699)
	70+ years	1340 (1080 to 1670)	41·9 (33·8 to 52·1)	-1·89 (-2·33 to -1·46)	-2·10 (-2·68 to -1·58)	550 (367 to 1270)	17·2 (11·5 to 39·8)	-3·06 (-4·23 to -1·88)	-2·24 (-4·10 to 0·261)
Antigua and Barbuda	All Ages	13·6 (11·7 to 16·2)	14·2 (12·3 to 16·7)	-2·16 (-2·50 to -1·80)	-2·23 (-2·81 to -1·74)	0·382 (0·337 to 0·440)	0·372 (0·329 to 0·428)	-5·84 (-6·18 to -5·53)	-3·88 (-4·48 to -3·26)
	Under 5	0·475 (0·354 to 0·606)	9·01 (6·72 to 11·5)	-2·80 (-3·55 to -2·00)	-2·02 (-3·24 to -0·898)	0·00237 (0·00198 to 0·00290)	0·0450 (0·0375 to 0·0550)	-5·02 (-6·04 to -3·66)	-7·54 (-9·45 to -5·98)
	5-14 years	0·820 (0·544 to 1·19)	7·05 (4·68 to 10·3)	-2·92 (-3·70 to -2·21)	-2·28 (-3·59 to -1·10)	0·00178 (0·00150 to 0·00220)	0·0153 (0·0129 to 0·0189)	-5·09 (-5·86 to -4·41)	-7·42 (-8·73 to -6·35)
	15-49 years	7·82 (6·25 to 9·89)	16·5 (13·2 to 20·9)	-2·15 (-2·63 to -1·68)	-2·60 (-3·17 to -2·02)	0·129 (0·105 to 0·166)	0·272 (0·221 to 0·350)	-6·01 (-6·31 to -5·69)	-5·34 (-6·12 to -4·55)
	50-69 years	3·51 (2·59 to 4·77)	18·0 (13·3 to 24·5)	-2·16 (-2·76 to -1·53)	-2·64 (-3·48 to -1·47)	0·138 (0·119 to 0·156)	0·708 (0·612 to 0·803)	-6·75 (-7·18 to -6·20)	-5·00 (-5·85 to -3·98)
	70+ years	0·968 (0·711 to 1·25)	17·2 (12·7 to 22·3)	-3·22 (-3·90 to -2·46)	-2·97 (-4·10 to -1·90)	0·110 (0·101 to 0·124)	1·97 (1·79 to 2·21)	-5·24 (-5·71 to -4·77)	-6·02 (-6·99 to -4·86)
Bahamas	All Ages	53·8 (46·6 to 62·7)	12·9 (11·3 to 14·9)	-2·72 (-3·16 to -2·25)	-1·34 (-1·94 to -0·656)	9·58 (7·59 to 12·6)	2·27 (1·81 to 2·96)	-3·49 (-3·92 to -3·00)	-2·94 (-4·07 to -1·40)
	Under 5	1·16 (0·932 to 1·47)	5·49 (4·43 to 6·99)	-3·54 (-4·19 to -2·90)	-2·37 (-3·95 to -0·803)	0·0487 (0·0369 to 0·0658)	0·231 (0·175 to 0·313)	-5·93 (-6·68 to -5·10)	-4·67 (-6·43 to -2·81)
	5-14 years	2·99 (2·03 to 4·25)	4·98 (3·37 to 7·08)	-3·24 (-4·15 to -2·50)	-2·54 (-3·86 to -1·07)	0·0546 (0·0435 to 0·0716)	0·0908 (0·0723 to 0·119)	-4·82 (-5·55 to -4·17)	-3·91 (-4·99 to -2·65)
	15-49 years	30·6 (25·3 to 38·9)	14·6 (12·1 to 18·6)	-2·87 (-3·45 to -2·27)	-1·94 (-2·65 to -1·19)	5·16 (3·91 to 6·97)	2·46 (1·87 to 3·32)	-3·53 (-4·04 to -3·08)	-3·83 (-4·89 to -2·46)
	50-69 years	15·4 (11·2 to 20·2)	19·9 (14·4 to 26·0)	-3·04 (-3·55 to -2·46)	-1·13 (-1·96 to 0·0630)	2·96 (2·35 to 3·87)	3·81 (3·03 to 4·98)	-5·26 (-5·89 to -4·66)	-3·90 (-5·33 to -2·11)
	70+ years	3·67 (2·87 to 4·65)	18·9 (14·8 to 24·0)	-3·52 (-4·11 to -2·93)	-2·86 (-4·09 to -1·70)	1·36 (1·16 to 1·63)	6·99 (5·98 to 8·42)	-4·24 (-4·85 to -3·53)	-3·79 (-5·17 to -2·01)
Barbados	All Ages	17·1 (14·7 to 19·9)	5·02 (4·36 to 5·92)	-3·05 (-3·57 to -2·67)	-1·41 (-1·98 to -0·825)	1·85 (1·46 to 2·30)	0·435 (0·340 to 0·550)	-4·88 (-5·18 to -4·48)	-2·55 (-4·25 to -0·614)
	Under 5	0·412 (0·304 to 0·530)	3·03 (2·23 to 3·89)	-2·95 (-3·85 to -2·11)	-2·04 (-3·17 to -0·882)	0·00658 (0·00474 to 0·00892)	0·0484 (0·0348 to 0·0655)	-6·66 (-7·59 to -5·79)	-4·05 (-5·67 to -2·00)
	5-14 years	0·719 (0·451 to 1·09)	2·15 (1·35 to 3·24)	-3·32 (-4·10 to -2·53)	-1·67 (-3·13 to -0·506)	0·00563 (0·00355 to 0·00749)	0·0168 (0·0106 to 0·0224)	-6·95 (-7·65 to -6·24)	-6·53 (-9·70 to -3·42)

eTable 1. All-form tuberculosis incident cases and deaths, age-standardised rates of incidence and mortality per 100,000 population, and corresponding annualized rates of change by age groups for 204 countries and territories (2021).

Location	Age group	Number of cases	Rate per 100,000 cases	Annualized rate of change (1990-2010)	Annualized rate of change (2010-2021)	Number of deaths	Rate per 100,000 deaths	Annualized deaths (1990-2010)	Annualized deaths (2010-2021)
	15-49 years	7·88 (6·32 to 9·96)	5·64 (4·53 to 7·13)	-3·28 (-3·83 to -2·81)	-1·85 (-2·44 to -1·17)	0·551 (0·402 to 0·743)	0·395 (0·288 to 0·532)	-5·27 (-5·61 to -4·80)	-4·04 (-5·41 to -2·63)
	50-69 years	5·50 (4·08 to 7·25)	6·87 (5·09 to 9·04)	-2·95 (-3·52 to -2·41)	-1·67 (-2·63 to -0·280)	0·622 (0·469 to 0·796)	0·776 (0·585 to 0·993)	-5·78 (-6·20 to -5·33)	-3·37 (-5·33 to -1·52)
	70+ years	2·58 (1·98 to 3·26)	8·04 (6·19 to 10·2)	-3·79 (-4·41 to -3·21)	-1·93 (-2·99 to -0·700)	0·664 (0·532 to 0·833)	2·07 (1·66 to 2·60)	-5·03 (-5·48 to -4·47)	-3·41 (-5·27 to -1·25)
Belize	All Ages	107 (92·2 to 124)	26·7 (23·4 to 30·8)	-2·39 (-2·88 to -1·93)	-1·78 (-2·33 to -1·22)	17·2 (14·5 to 21·0)	4·71 (4·01 to 5·64)	-2·66 (-3·04 to -2·22)	-4·40 (-5·20 to -3·55)
	Under 5	2·85 (2·24 to 3·59)	7·48 (5·88 to 9·42)	-5·09 (-5·86 to -4·55)	-4·45 (-5·92 to -3·37)	0·258 (0·205 to 0·341)	0·676 (0·538 to 0·895)	-9·33 (-10·1 to -8·74)	-7·58 (-8·94 to -5·80)
	5-14 years	7·84 (5·21 to 11·0)	9·23 (6·13 to 13·0)	-4·56 (-5·33 to -3·75)	-2·63 (-4·13 to -1·28)	0·173 (0·135 to 0·219)	0·204 (0·158 to 0·258)	-6·27 (-6·85 to -5·73)	-7·73 (-9·29 to -6·50)
	15-49 years	62·3 (51·6 to 79·2)	26·3 (21·8 to 33·4)	-2·40 (-2·93 to -1·75)	-2·53 (-3·22 to -1·76)	9·48 (7·48 to 12·3)	4·00 (3·15 to 5·18)	-1·64 (-2·07 to -1·12)	-5·67 (-6·39 to -4·90)
	50-69 years	27·5 (20·1 to 35·0)	50·2 (36·6 to 63·8)	-1·05 (-1·71 to -0·362)	-1·99 (-3·12 to -0·785)	4·70 (3·95 to 5·60)	8·56 (7·19 to 10·2)	-3·36 (-3·84 to -2·97)	-5·08 (-6·14 to -4·13)
	70+ years	6·23 (4·96 to 7·99)	44·6 (35·5 to 57·2)	-2·93 (-3·58 to -2·29)	-3·64 (-4·91 to -2·56)	2·57 (2·24 to 2·94)	18·4 (16·0 to 21·1)	-4·00 (-4·46 to -3·42)	-4·98 (-6·23 to -3·72)
Bermuda	All Ages	10·3 (8·83 to 12·5)	14·3 (12·1 to 17·3)	-2·57 (-3·22 to -2·06)	-2·01 (-2·56 to -1·33)	0·0475 (0·0405 to 0·0556)	0·0428 (0·0349 to 0·0522)	-6·86 (-7·27 to -6·34)	-4·22 (-5·24 to -3·04)
	Under 5	0·186 (0·141 to 0·241)	7·28 (5·52 to 9·44)	-3·87 (-4·62 to -3·13)	-2·35 (-3·91 to -1·15)	0·000140 (0·000105 to 0·000179)	0·00551 (0·00413 to 0·00703)	-8·12 (-9·09 to -7·37)	-9·73 (-11·7 to -7·60)
	5-14 years	0·333 (0·224 to 0·492)	5·65 (3·81 to 8·36)	-3·99 (-4·91 to -3·16)	-2·31 (-3·74 to -1·03)	0·000124 (0·0000923 to 0·000170)	0·00211 (0·00157 to 0·00289)	-6·63 (-7·45 to -5·72)	-7·05 (-8·74 to -5·45)
	15-49 years	4·85 (3·87 to 6·37)	18·1 (14·4 to 23·7)	-2·78 (-3·40 to -2·28)	-2·11 (-2·76 to -1·61)	0·0103 (0·00747 to 0·0141)	0·0385 (0·0278 to 0·0524)	-7·86 (-8·30 to -7·37)	-5·73 (-6·59 to -4·86)
	50-69 years	3·84 (2·87 to 5·19)	20·0 (14·9 to 27·0)	-1·45 (-2·15 to -0·874)	-2·02 (-2·88 to -1·06)	0·0144 (0·0113 to 0·0177)	0·0751 (0·0586 to 0·0922)	-8·36 (-8·88 to -7·84)	-6·23 (-7·31 to -5·02)
	70+ years	1·13 (0·866 to 1·46)	12·6 (9·61 to 16·2)	-3·68 (-4·38 to -2·93)	-2·58 (-3·52 to -1·49)	0·0224 (0·0194 to 0·0265)	0·249 (0·216 to 0·295)	-7·57 (-8·09 to -6·95)	-5·98 (-7·29 to -4·63)
Cuba	All Ages	624 (543 to 726)	4·71 (4·10 to 5·49)	-2·57 (-3·01 to -2·00)	-2·12 (-2·67 to -1·59)	36·0 (31·2 to 39·8)	0·210 (0·180 to 0·236)	-3·80 (-4·18 to -3·45)	-3·42 (-4·70 to -2·22)
	Under 5	12·5 (9·81 to 15·8)	2·29 (1·80 to 2·90)	-2·94 (-3·73 to -2·10)	-2·98 (-4·21 to -1·79)	0·0880 (0·0695 to 0·107)	0·0162 (0·0128 to 0·0196)	-7·64 (-8·24 to -7·10)	-7·34 (-9·44 to -5·33)
	5-14 years	11·9 (7·77 to 17·4)	0·964 (0·630 to 1·41)	-3·18 (-4·12 to -2·39)	-4·64 (-6·12 to -3·21)	0·0749 (0·0607 to 0·0855)	0·00607 (0·00493 to 0·00694)	-7·17 (-7·70 to -6·63)	-8·94 (-10·5 to -7·61)
	15-49 years	299 (242 to 362)	5·89 (4·75 to 7·12)	-2·53 (-3·19 to -1·76)	-1·84 (-2·57 to -1·18)	8·06 (6·61 to 9·87)	0·159 (0·130 to 0·194)	-4·82 (-5·31 to -4·38)	-3·89 (-5·06 to -2·89)
	50-69 years	221 (167 to 285)	6·99 (5·27 to 9·00)	-2·88 (-3·60 to -2·29)	-2·86 (-3·74 to -2·07)	15·4 (12·9 to 17·7)	0·487 (0·408 to 0·559)	-4·40 (-5·01 to -3·90)	-3·93 (-5·38 to -2·31)
	70+ years	78·9 (63·7 to 97·5)	6·35 (5·13 to 7·85)	-5·10 (-5·68 to -4·46)	-3·71 (-4·88 to -2·68)	12·4 (10·6 to 13·9)	0·996 (0·854 to 1·12)	-5·25 (-5·67 to -4·72)	-6·85 (-8·33 to -5·66)

eTable 1. All-form tuberculosis incident cases and deaths, age-standardised rates of incidence and mortality per 100,000 population, and corresponding annualized rates of change by age groups for 204 countries and territories (2021).

Location	Age group	Number of cases	Rate per 100,000 cases	Annualized rate of change (1990-2010)	Annualized rate of change (2010-2021)	Number of deaths	Rate per 100,000 deaths	Annualized deaths (1990-2010)	Annualized deaths (2010-2021)
Dominica	All Ages	13·4 (11·7 to 15·3)	18·2 (15·9 to 20·7)	-2·18 (-2·59 to -1·68)	-1·20 (-1·83 to -0·614)	2·90 (2·21 to 3·71)	3·76 (2·85 to 4·77)	-3·92 (-4·68 to -3·00)	-0·531 (-2·55 to 1·40)
	Under 5	0·258 (0·204 to 0·324)	7·43 (5·88 to 9·32)	-3·00 (-3·70 to -2·31)	-1·26 (-2·63 to 0·0767)	0·0358 (0·0246 to 0·0489)	1·03 (0·708 to 1·41)	-3·54 (-4·96 to -2·42)	1·21 (-1·74 to 4·10)
	5-14 years	0·630 (0·445 to 0·892)	6·17 (4·36 to 8·74)	-2·72 (-3·46 to -1·93)	-1·55 (-2·86 to -0·334)	0·0258 (0·0186 to 0·0334)	0·253 (0·182 to 0·327)	-3·39 (-4·47 to -2·30)	-1·14 (-3·57 to 1·20)
	15-49 years	6·69 (5·55 to 8·19)	19·8 (16·4 to 24·2)	-2·41 (-3·02 to -1·85)	-2·02 (-2·84 to -1·31)	0·948 (0·672 to 1·30)	2·81 (1·99 to 3·83)	-3·49 (-4·32 to -2·42)	-2·03 (-4·01 to -0·275)
	50-69 years	4·23 (3·20 to 5·39)	28·0 (21·2 to 35·7)	-2·24 (-2·83 to -1·63)	-1·73 (-2·52 to -0·788)	1·12 (0·808 to 1·50)	7·41 (5·36 to 9·93)	-4·86 (-5·85 to -3·89)	-1·68 (-4·28 to 0·622)
	70+ years	1·54 (1·22 to 1·93)	34·2 (27·0 to 42·8)	-4·04 (-4·56 to -3·39)	-1·40 (-2·39 to -0·512)	0·776 (0·569 to 1·00)	17·2 (12·6 to 22·2)	-6·02 (-6·94 to -5·12)	-1·33 (-3·68 to 0·658)
Dominican Republic	All Ages	5650 (4960 to 6610)	51·0 (44·9 to 59·0)	-2·26 (-2·74 to -1·77)	-1·46 (-1·96 to -0·985)	852 (587 to 1340)	8·08 (5·59 to 12·8)	-2·62 (-4·24 to -0·992)	-2·60 (-5·07 to 0·355)
	Under 5	150 (122 to 192)	14·5 (11·8 to 18·6)	-5·25 (-6·15 to -4·57)	-3·50 (-4·69 to -2·28)	13·3 (9·18 to 18·3)	1·29 (0·888 to 1·78)	-10·0 (-11·5 to -8·72)	-7·46 (-10·6 to -4·01)
	5-14 years	317 (223 to 443)	16·6 (11·7 to 23·2)	-3·97 (-4·66 to -3·30)	-3·01 (-4·15 to -1·83)	11·3 (7·74 to 17·2)	0·595 (0·406 to 0·900)	-5·24 (-6·97 to -3·46)	-3·89 (-6·66 to -1·01)
	15-49 years	3410 (2790 to 4320)	58·2 (47·6 to 73·7)	-2·43 (-2·99 to -1·88)	-1·85 (-2·49 to -1·31)	353 (236 to 548)	6·03 (4·03 to 9·34)	-2·30 (-4·20 to -0·630)	-4·98 (-7·23 to -2·84)
	50-69 years	1190 (904 to 1500)	71·9 (54·6 to 90·6)	-1·73 (-2·23 to -1·11)	-1·42 (-2·24 to -0·616)	245 (156 to 403)	14·8 (9·43 to 24·3)	-2·83 (-4·90 to -1·03)	-2·29 (-6·18 to 1·28)
	70+ years	576 (463 to 713)	104 (83·4 to 129)	-2·04 (-2·64 to -1·40)	-1·27 (-2·04 to -0·213)	229 (156 to 393)	41·2 (28·1 to 70·8)	-3·41 (-5·27 to -1·83)	-0·923 (-3·79 to 2·23)
Grenada	All Ages	9·34 (8·00 to 10·8)	8·68 (7·56 to 10·0)	-2·40 (-2·86 to -1·98)	-1·85 (-2·42 to -1·22)	0·433 (0·376 to 0·493)	0·397 (0·348 to 0·450)	-5·31 (-5·80 to -4·75)	-5·81 (-6·87 to -4·80)
	Under 5	0·408 (0·314 to 0·537)	5·94 (4·57 to 7·83)	-2·86 (-3·49 to -2·21)	-2·00 (-3·48 to -0·739)	0·00312 (0·00240 to 0·00401)	0·0454 (0·0349 to 0·0583)	-8·08 (-9·32 to -6·70)	-9·40 (-11·1 to -7·11)
	5-14 years	0·638 (0·403 to 0·967)	4·27 (2·70 to 6·46)	-3·28 (-4·18 to -2·51)	-1·88 (-3·65 to -0·393)	0·00337 (0·00282 to 0·00396)	0·0225 (0·0188 to 0·0265)	-5·88 (-6·63 to -5·04)	-6·88 (-8·48 to -5·70)
	15-49 years	5·34 (4·29 to 6·76)	10·0 (8·04 to 12·7)	-2·60 (-3·13 to -1·93)	-1·91 (-2·56 to -1·13)	0·149 (0·122 to 0·179)	0·280 (0·229 to 0·336)	-5·91 (-6·40 to -5·46)	-7·78 (-8·75 to -6·80)
	50-69 years	2·34 (1·72 to 3·16)	10·9 (7·99 to 14·7)	-2·44 (-3·02 to -1·71)	-2·47 (-3·46 to -1·46)	0·160 (0·136 to 0·188)	0·745 (0·630 to 0·874)	-6·66 (-7·31 to -5·99)	-6·54 (-7·88 to -5·07)
	70+ years	0·605 (0·452 to 0·806)	10·2 (7·65 to 13·6)	-3·73 (-4·39 to -2·93)	-3·78 (-4·90 to -2·47)	0·116 (0·103 to 0·129)	1·97 (1·74 to 2·18)	-4·20 (-4·74 to -3·59)	-7·37 (-8·39 to -6·12)
Guyana	All Ages	371 (319 to 435)	49·0 (42·5 to 56·8)	0·479 (0·0281 to 1·03)	-3·19 (-3·88 to -2·47)	72·9 (56·2 to 91·4)	10·2 (7·91 to 12·7)	-1·07 (-1·66 to -0·211)	-5·72 (-7·37 to -4·01)
	Under 5	8·94 (7·06 to 11·4)	12·0 (9·47 to 15·3)	-3·80 (-4·52 to -3·17)	-4·47 (-5·56 to -3·03)	0·926 (0·668 to 1·22)	1·24 (0·896 to 1·64)	-5·76 (-6·47 to -4·78)	-8·27 (-10·1 to -6·69)
	5-14 years	17·7 (12·4 to 25·4)	12·8 (8·90 to 18·3)	-3·70 (-4·56 to -2·70)	-3·55 (-4·86 to -2·11)	0·662 (0·518 to 0·832)	0·477 (0·373 to 0·599)	-3·81 (-4·62 to -3·04)	-9·08 (-10·4 to -7·22)

eTable 1. All-form tuberculosis incident cases and deaths, age-standardised rates of incidence and mortality per 100,000 population, and corresponding annualized rates of change by age groups for 204 countries and territories (2021).

Location	Age group	Number of cases	Rate per 100,000 cases	Annualized rate of change (1990-2010)	Annualized rate of change (2010-2021)	Number of deaths	Rate per 100,000 deaths	Annualized deaths (1990-2010)	Annualized deaths (2010-2021)
	15-49 years	230 (193 to 282)	57·4 (48·2 to 70·5)	1·63 (1·09 to 2·27)	-4·04 (-4·87 to -3·08)	39·3 (30·0 to 51·0)	9·82 (7·49 to 12·8)	-0·233 (-0·862 to 0·602)	-6·78 (-8·15 to -5·27)
	50-69 years	96·9 (74·6 to 121)	79·1 (60·8 to 99·0)	-0·572 (-1·11 to -0·0111)	-2·38 (-3·59 to -1·33)	23·7 (18·0 to 29·8)	19·4 (14·7 to 24·3)	-2·91 (-3·61 to -2·19)	-6·19 (-8·07 to -4·22)
	70+ years	17·5 (13·8 to 22·2)	60·5 (47·9 to 76·6)	-3·42 (-4·10 to -2·65)	-4·23 (-5·29 to -3·34)	8·31 (6·64 to 10·3)	28·7 (22·9 to 35·8)	-4·82 (-5·71 to -4·06)	-5·61 (-8·08 to -3·65)
Haiti	All Ages	11300 (9850 to 12800)	97·8 (86·5 to 109)	-1·88 (-2·32 to -1·45)	-2·32 (-2·94 to -1·65)	2170 (1270 to 6600)	22·5 (12·9 to 72·0)	-4·45 (-6·00 to -3·23)	-3·69 (-6·14 to -1·58)
	Under 5	848 (673 to 1060)	54·0 (42·9 to 67·6)	-3·70 (-4·38 to -3·13)	-3·04 (-4·03 to -1·65)	225 (154 to 364)	14·3 (9·82 to 23·2)	-6·29 (-7·52 to -4·70)	-4·86 (-7·70 to -2·12)
	5-14 years	1020 (683 to 1430)	36·5 (24·5 to 51·2)	-2·62 (-3·48 to -1·57)	-2·80 (-4·39 to -1·45)	69·5 (43·9 to 157)	2·50 (1·58 to 5·65)	-2·86 (-4·77 to -1·37)	-3·46 (-6·37 to -1·07)
	15-49 years	6940 (5630 to 8230)	101 (82·1 to 120)	-1·86 (-2·42 to -1·37)	-2·65 (-3·44 to -1·76)	1020 (590 to 3100)	14·9 (8·61 to 45·2)	-4·56 (-6·06 to -2·95)	-4·51 (-7·16 to -2·45)
	50-69 years	1970 (1560 to 2400)	144 (114 to 175)	-1·20 (-1·62 to -0·787)	-1·53 (-2·57 to -0·667)	601 (329 to 2140)	43·8 (24·0 to 156)	-3·74 (-5·48 to -2·14)	-2·90 (-5·26 to -0·581)
	70+ years	532 (412 to 656)	187 (145 to 231)	-1·49 (-2·57 to -0·732)	-2·15 (-3·05 to -1·12)	253 (127 to 928)	89·0 (44·7 to 327)	-2·93 (-5·01 to -1·39)	-2·38 (-5·33 to 0·445)
Jamaica	All Ages	127 (110 to 146)	4·26 (3·72 to 4·90)	-2·34 (-2·70 to -2·03)	-2·51 (-3·01 to -1·94)	24·4 (18·9 to 31·3)	0·790 (0·613 to 1·02)	-3·29 (-3·73 to -2·81)	-1·95 (-3·24 to -0·371)
	Under 5	4·03 (3·15 to 5·26)	2·36 (1·84 to 3·07)	-3·18 (-3·85 to -2·42)	-1·58 (-2·90 to -0·519)	0·145 (0·110 to 0·198)	0·0849 (0·0643 to 0·116)	-7·78 (-8·69 to -6·66)	-5·01 (-6·05 to -3·68)
	5-14 years	6·39 (4·16 to 9·16)	1·55 (1·01 to 2·22)	-2·89 (-3·72 to -1·98)	-2·79 (-4·45 to -1·50)	0·150 (0·116 to 0·192)	0·0362 (0·0280 to 0·0464)	-5·96 (-6·66 to -5·12)	-4·49 (-5·51 to -3·32)
	15-49 years	68·9 (56·8 to 87·2)	4·50 (3·71 to 5·70)	-2·59 (-3·11 to -2·15)	-3·29 (-4·09 to -2·58)	10·9 (7·88 to 15·6)	0·710 (0·515 to 1·02)	-3·06 (-3·47 to -2·57)	-2·75 (-3·68 to -1·64)
	50-69 years	32·8 (24·8 to 42·4)	6·51 (4·93 to 8·43)	-2·62 (-3·33 to -2·12)	-2·98 (-4·11 to -1·90)	7·61 (5·73 to 9·64)	1·51 (1·14 to 1·91)	-4·49 (-5·08 to -3·74)	-3·14 (-4·51 to -0·918)
	70+ years	14·5 (11·8 to 18·5)	7·98 (6·50 to 10·2)	-3·45 (-4·03 to -2·76)	-2·71 (-3·56 to -1·80)	5·60 (4·53 to 6·79)	3·08 (2·49 to 3·74)	-4·69 (-5·17 to -4·26)	-2·82 (-4·85 to -1·04)
Puerto Rico	All Ages	114 (98·4 to 131)	2·76 (2·42 to 3·25)	-4·91 (-5·42 to -4·51)	-2·69 (-3·27 to -2·07)	21·4 (17·7 to 25·7)	0·414 (0·330 to 0·516)	-6·67 (-7·19 to -6·24)	-6·75 (-7·86 to -5·52)
	Under 5	1·57 (1·23 to 2·12)	1·50 (1·17 to 2·02)	-4·14 (-4·91 to -3·18)	-2·96 (-4·18 to -1·67)	0·0378 (0·0292 to 0·0508)	0·0359 (0·0277 to 0·0483)	-7·30 (-7·80 to -6·87)	-9·74 (-10·8 to -8·48)
	5-14 years	3·64 (2·23 to 5·49)	1·07 (0·657 to 1·62)	-4·13 (-5·30 to -3·20)	-3·72 (-5·94 to -1·98)	0·0377 (0·0267 to 0·0531)	0·0111 (0·00788 to 0·0156)	-7·20 (-7·56 to -6·74)	-11·2 (-12·7 to -9·93)
	15-49 years	43·1 (35·3 to 54·8)	2·94 (2·41 to 3·74)	-6·16 (-6·61 to -5·45)	-2·99 (-3·69 to -2·17)	5·98 (4·62 to 7·85)	0·408 (0·316 to 0·536)	-8·71 (-9·29 to -8·23)	-8·86 (-9·97 to -7·98)
	50-69 years	36·9 (27·2 to 47·4)	4·29 (3·16 to 5·52)	-4·88 (-5·52 to -4·35)	-3·45 (-4·25 to -2·51)	7·29 (5·76 to 9·16)	0·850 (0·671 to 1·07)	-6·14 (-6·64 to -5·61)	-7·70 (-9·00 to -6·67)
	70+ years	28·6 (23·0 to 35·8)	5·44 (4·37 to 6·80)	-5·19 (-5·84 to -4·64)	-4·90 (-6·23 to -3·50)	8·09 (6·96 to 9·37)	1·54 (1·32 to 1·78)	-6·80 (-7·19 to -6·35)	-8·55 (-9·70 to -7·34)

eTable 1. All-form tuberculosis incident cases and deaths, age-standardised rates of incidence and mortality per 100,000 population, and corresponding annualized rates of change by age groups for 204 countries and territories (2021).

Location	Age group	Number of cases	Rate per 100,000 cases	Annualized rate of change (1990-2010)	Annualized rate of change (2010-2021)	Number of deaths	Rate per 100,000 deaths	Annualized deaths (1990-2010)	Annualized deaths (2010-2021)
Saint Kitts and Nevis	All Ages	6·64 (5·74 to 7·69)	9·90 (8·69 to 11·3)	-3·67 (-4·13 to -3·17)	-1·11 (-1·77 to -0·451)	1·23 (0·983 to 1·46)	1·79 (1·45 to 2·11)	-4·87 (-5·61 to -3·90)	-2·20 (-3·34 to -0·892)
	Under 5	0·139 (0·111 to 0·174)	4·55 (3·65 to 5·70)	-4·57 (-5·36 to -3·86)	-1·57 (-2·93 to -0·535)	0·0124 (0·00864 to 0·0173)	0·406 (0·283 to 0·566)	-5·74 (-6·75 to -4·22)	-2·36 (-3·63 to -0·768)
	5-14 years	0·182 (0·126 to 0·248)	2·67 (1·86 to 3·64)	-3·75 (-4·60 to -2·87)	-3·65 (-5·00 to -2·30)	0·00592 (0·00487 to 0·00735)	0·0872 (0·0717 to 0·108)	-6·08 (-6·78 to -5·20)	-3·15 (-4·92 to -1·56)
	15-49 years	3·50 (2·89 to 4·36)	11·1 (9·14 to 13·8)	-3·99 (-4·56 to -3·37)	-2·04 (-2·88 to -1·27)	0·441 (0·323 to 0·587)	1·40 (1·02 to 1·86)	-5·10 (-6·25 to -3·37)	-2·96 (-3·95 to -1·46)
	50-69 years	2·32 (1·73 to 2·93)	16·1 (12·0 to 20·3)	-4·01 (-4·86 to -3·21)	-0·871 (-1·79 to 0·120)	0·539 (0·433 to 0·623)	3·73 (3·00 to 4·31)	-5·04 (-5·80 to -4·23)	-3·73 (-5·16 to -2·39)
	70+ years	0·492 (0·386 to 0·616)	17·7 (13·9 to 22·2)	-4·38 (-5·01 to -3·63)	-2·99 (-3·96 to -1·92)	0·229 (0·196 to 0·261)	8·24 (7·05 to 9·39)	-4·99 (-5·43 to -4·54)	-4·70 (-5·83 to -3·52)
Saint Lucia	All Ages	21·8 (18·8 to 25·5)	10·7 (9·26 to 12·3)	-3·37 (-3·78 to -2·92)	-0·820 (-1·40 to -0·0277)	4·51 (3·71 to 5·38)	2·02 (1·66 to 2·41)	-4·85 (-5·25 to -4·45)	-1·34 (-2·95 to 0·191)
	Under 5	0·385 (0·310 to 0·494)	4·36 (3·50 to 5·60)	-4·46 (-5·08 to -3·60)	-2·35 (-3·80 to -1·05)	0·0172 (0·0130 to 0·0220)	0·195 (0·147 to 0·249)	-8·46 (-9·53 to -7·52)	-5·40 (-7·23 to -3·31)
	5-14 years	0·794 (0·519 to 1·11)	3·81 (2·49 to 5·34)	-4·22 (-5·03 to -3·24)	-2·22 (-3·49 to -0·967)	0·0225 (0·0184 to 0·0269)	0·108 (0·0881 to 0·129)	-4·71 (-5·49 to -4·00)	-4·86 (-6·42 to -3·33)
	15-49 years	10·8 (8·90 to 13·9)	11·7 (9·62 to 15·0)	-3·96 (-4·61 to -3·39)	-1·38 (-2·05 to -0·381)	1·65 (1·35 to 2·00)	1·78 (1·46 to 2·16)	-5·11 (-5·52 to -4·66)	-3·37 (-4·58 to -2·10)
	50-69 years	7·00 (5·34 to 8·99)	16·8 (12·8 to 21·5)	-3·91 (-4·57 to -3·34)	-1·83 (-2·72 to -0·936)	1·51 (1·19 to 1·82)	3·63 (2·85 to 4·36)	-7·18 (-7·67 to -6·65)	-2·71 (-4·69 to -0·903)
	70+ years	2·86 (2·30 to 3·65)	21·1 (17·0 to 26·9)	-5·09 (-5·62 to -4·49)	-2·36 (-3·30 to -1·37)	1·30 (1·06 to 1·54)	9·58 (7·78 to 11·3)	-7·00 (-7·47 to -6·50)	-2·26 (-3·95 to -0·721)
Saint Vincent and the Grenadines	All Ages	17·1 (14·6 to 19·6)	13·9 (12·0 to 15·9)	-2·50 (-2·96 to -1·89)	-2·15 (-2·83 to -1·46)	2·70 (2·27 to 3·27)	2·07 (1·72 to 2·51)	-3·81 (-4·23 to -3·35)	-3·67 (-4·56 to -2·64)
	Under 5	0·473 (0·365 to 0·620)	6·58 (5·07 to 8·61)	-4·08 (-4·74 to -3·41)	-1·89 (-3·35 to -0·558)	0·0145 (0·0105 to 0·0190)	0·201 (0·146 to 0·264)	-8·73 (-9·77 to -7·87)	-7·69 (-9·13 to -5·83)
	5-14 years	1·05 (0·701 to 1·54)	5·93 (3·95 to 8·68)	-3·42 (-4·09 to -2·72)	-2·74 (-4·34 to -1·65)	0·0213 (0·0171 to 0·0264)	0·120 (0·0963 to 0·149)	-5·75 (-6·55 to -5·02)	-4·93 (-6·09 to -3·76)
	15-49 years	8·75 (7·08 to 11·1)	15·5 (12·5 to 19·6)	-2·86 (-3·46 to -2·10)	-2·56 (-3·39 to -1·79)	1·12 (0·843 to 1·48)	1·99 (1·49 to 2·62)	-3·99 (-4·33 to -3·52)	-5·22 (-5·92 to -4·35)
	50-69 years	4·99 (3·70 to 6·47)	20·2 (15·0 to 26·2)	-2·58 (-3·29 to -1·89)	-2·83 (-3·57 to -1·90)	0·927 (0·787 to 1·13)	3·76 (3·19 to 4·57)	-5·40 (-6·11 to -4·76)	-4·17 (-5·46 to -2·74)
	70+ years	1·78 (1·37 to 2·26)	22·4 (17·3 to 28·4)	-3·87 (-4·48 to -3·10)	-3·09 (-4·54 to -1·88)	0·621 (0·559 to 0·694)	7·80 (7·03 to 8·72)	-5·89 (-6·44 to -5·31)	-4·30 (-5·36 to -3·14)
Suriname	All Ages	113 (99·6 to 130)	18·8 (16·6 to 21·7)	-2·08 (-2·57 to -1·59)	-2·01 (-2·60 to -1·38)	16·7 (12·1 to 22·7)	2·71 (1·97 to 3·67)	-3·34 (-4·36 to -2·12)	-2·08 (-4·16 to -0·253)
	Under 5	3·19 (2·60 to 3·93)	7·17 (5·84 to 8·83)	-3·74 (-4·52 to -3·01)	-3·26 (-4·34 to -2·08)	0·278 (0·193 to 0·383)	0·625 (0·433 to 0·860)	-6·75 (-7·81 to -5·41)	-3·81 (-6·24 to -2·10)
	5-14 years	5·92 (3·99 to 8·10)	6·00 (4·05 to 8·20)	-3·68 (-4·32 to -2·71)	-2·34 (-3·65 to -1·02)	0·188 (0·144 to 0·245)	0·190 (0·146 to 0·248)	-5·46 (-6·74 to -3·47)	-2·71 (-4·51 to -0·673)

eTable 1. All-form tuberculosis incident cases and deaths, age-standardised rates of incidence and mortality per 100,000 population, and corresponding annualized rates of change by age groups for 204 countries and territories (2021).

Location	Age group	Number of cases	Rate per 100,000 cases	Annualized rate of change (1990-2010)	Annualized rate of change (2010-2021)	Number of deaths	Rate per 100,000 deaths	Annualized deaths (1990-2010)	Annualized deaths (2010-2021)
	15-49 years	66·9 (56·0 to 83·2)	23·3 (19·5 to 29·0)	-2·05 (-2·65 to -1·34)	-2·25 (-2·85 to -1·53)	8·25 (5·90 to 11·5)	2·87 (2·05 to 4·00)	-3·13 (-4·12 to -1·78)	-2·85 (-4·91 to -0·889)
	50-69 years	28·0 (20·9 to 34·5)	24·3 (18·1 to 29·9)	-2·25 (-2·90 to -1·75)	-2·05 (-3·03 to -1·20)	5·55 (4·14 to 7·71)	4·80 (3·58 to 6·67)	-4·43 (-5·47 to -3·34)	-2·49 (-4·61 to -0·177)
	70+ years	8·81 (7·07 to 10·7)	26·5 (21·3 to 32·2)	-2·84 (-3·36 to -2·19)	-2·81 (-3·51 to -2·06)	2·43 (1·73 to 3·27)	7·29 (5·21 to 9·81)	-4·69 (-5·99 to -3·60)	-3·60 (-6·40 to -0·645)
Trinidad and Tobago	All Ages	174 (150 to 206)	11·1 (9·51 to 13·1)	-1·43 (-1·92 to -0·951)	-1·79 (-2·40 to -1·26)	26·2 (20·6 to 34·0)	1·53 (1·20 to 1·98)	-3·37 (-3·66 to -3·03)	-2·36 (-3·81 to -0·766)
	Under 5	3·45 (2·80 to 4·42)	4·28 (3·48 to 5·49)	-3·29 (-4·20 to -2·40)	-2·91 (-4·26 to -1·52)	0·156 (0·118 to 0·206)	0·194 (0·146 to 0·256)	-5·35 (-6·04 to -4·68)	-4·70 (-6·04 to -2·96)
	5-14 years	5·80 (3·87 to 8·10)	3·02 (2·02 to 4·22)	-4·00 (-4·90 to -3·20)	-2·73 (-3·78 to -1·53)	0·134 (0·107 to 0·169)	0·0697 (0·0557 to 0·0882)	-7·65 (-8·48 to -7·04)	-3·74 (-4·91 to -2·34)
	15-49 years	92·9 (75·2 to 117)	13·5 (10·9 to 16·9)	-1·67 (-2·24 to -1·08)	-1·65 (-2·45 to -0·775)	11·7 (8·59 to 15·7)	1·70 (1·24 to 2·28)	-3·58 (-3·88 to -3·23)	-2·78 (-3·94 to -1·54)
	50-69 years	55·7 (41·7 to 68·3)	17·5 (13·1 to 21·4)	-1·89 (-2·48 to -1·33)	-2·40 (-3·25 to -1·30)	9·59 (7·54 to 12·1)	3·01 (2·36 to 3·78)	-5·10 (-5·51 to -4·57)	-2·79 (-4·62 to -1·11)
	70+ years	16·6 (13·2 to 21·4)	14·9 (11·8 to 19·1)	-4·01 (-4·69 to -3·31)	-2·82 (-3·81 to -1·80)	4·63 (3·65 to 5·74)	4·13 (3·26 to 5·13)	-6·23 (-6·72 to -5·71)	-4·34 (-6·53 to -2·35)
United States Virgin Islands	All Ages	5·05 (4·33 to 5·83)	4·64 (4·04 to 5·31)	-2·38 (-2·82 to -1·88)	-1·17 (-1·71 to -0·628)	0·866 (0·601 to 1·19)	0·756 (0·531 to 1·05)	-3·67 (-5·06 to -2·50)	0·387 (-2·14 to 2·30)
	Under 5	0·0738 (0·0598 to 0·0926)	1·88 (1·53 to 2·36)	-3·56 (-4·36 to -2·80)	-3·51 (-4·66 to -2·42)	0·00192 (0·00137 to 0·00267)	0·0490 (0·0351 to 0·0680)	-6·63 (-8·64 to -4·45)	-5·79 (-8·24 to -3·15)
	5-14 years	0·101 (0·0675 to 0·144)	1·06 (0·712 to 1·52)	-4·51 (-5·29 to -3·65)	-4·00 (-5·57 to -2·77)	0·00160 (0·00114 to 0·00218)	0·0169 (0·0120 to 0·0231)	-6·91 (-8·59 to -5·35)	-7·25 (-9·55 to -4·95)
	15-49 years	1·77 (1·50 to 2·24)	5·24 (4·43 to 6·62)	-3·02 (-3·50 to -2·58)	-2·64 (-3·29 to -2·02)	0·309 (0·215 to 0·444)	0·915 (0·637 to 1·31)	-3·79 (-5·09 to -2·81)	-0·520 (-2·97 to 1·21)
	50-69 years	1·98 (1·53 to 2·55)	7·68 (5·93 to 9·89)	-2·26 (-2·82 to -1·79)	-1·17 (-2·05 to -0·384)	0·264 (0·172 to 0·379)	1·02 (0·667 to 1·47)	-5·68 (-7·49 to -3·93)	-1·11 (-3·88 to 1·26)
	70+ years	1·12 (0·864 to 1·42)	8·70 (6·70 to 11·0)	-3·25 (-3·88 to -2·68)	-2·00 (-2·98 to -1·13)	0·289 (0·200 to 0·404)	2·24 (1·55 to 3·13)	-6·03 (-7·70 to -4·70)	-2·19 (-4·82 to 0·326)
Central Latin America	All Ages	56300 (49800 to 63900)	21·6 (19·2 to 24·4)	-2·29 (-2·52 to -1·99)	-0·0873 (-0·404 to 0·274)	7720 (6690 to 9040)	3·01 (2·62 to 3·52)	-4·92 (-5·28 to -4·47)	-1·17 (-1·95 to -0·303)
	Under 5	895 (742 to 1100)	4·46 (3·69 to 5·48)	-5·21 (-5·63 to -4·72)	-2·95 (-3·43 to -2·29)	92·6 (73·6 to 114)	0·461 (0·366 to 0·566)	-9·50 (-10·2 to -8·95)	-4·31 (-5·46 to -2·55)
	5-14 years	1840 (1240 to 2620)	4·24 (2·85 to 6·03)	-4·14 (-4·68 to -3·47)	-1·86 (-2·59 to -1·24)	69·5 (58·9 to 80·4)	0·160 (0·136 to 0·185)	-8·50 (-9·08 to -7·99)	-3·31 (-4·09 to -2·36)
	15-49 years	30800 (25800 to 38000)	23·1 (19·4 to 28·5)	-2·52 (-2·89 to -2·14)	-0·315 (-0·643 to 0·120)	3060 (2500 to 3700)	2·30 (1·88 to 2·78)	-4·77 (-5·34 to -3·98)	-1·81 (-2·47 to -1·13)
	50-69 years	16500 (12500 to 20600)	38·6 (29·3 to 48·2)	-2·99 (-3·44 to -2·61)	-1·15 (-1·64 to -0·603)	2600 (2250 to 3020)	6·09 (5·26 to 7·07)	-6·56 (-6·87 to -6·20)	-2·44 (-3·32 to -1·40)
	70+ years	6280 (5070 to 7820)	46·0 (37·1 to 57·3)	-4·07 (-4·43 to -3·63)	-2·77 (-3·13 to -2·36)	1900 (1690 to 2170)	13·9 (12·4 to 15·9)	-7·00 (-7·23 to -6·73)	-3·75 (-4·66 to -2·79)

eTable 1. All-form tuberculosis incident cases and deaths, age-standardised rates of incidence and mortality per 100,000 population, and corresponding annualized rates of change by age groups for 204 countries and territories (2021).

Location	Age group	Number of cases	Rate per 100,000 cases	Annualized rate of change (1990-2010)	Annualized rate of change (2010-2021)	Number of deaths	Rate per 100,000 deaths	Annualized deaths (1990-2010)	Annualized deaths (2010-2021)
Colombia	All Ages	11100 (9810 to 13000)	20·6 (18·3 to 24·1)	-2·08 (-2·51 to -1·62)	-0·809 (-1·27 to -0·301)	1280 (1080 to 1510)	2·36 (1·99 to 2·79)	-3·61 (-3·94 to -3·09)	-0·312 (-1·25 to 0·794)
	Under 5	152 (119 to 195)	4·41 (3·47 to 5·66)	-4·63 (-5·33 to -3·86)	-3·93 (-4·99 to -2·41)	11·6 (8·38 to 15·4)	0·338 (0·244 to 0·447)	-7·94 (-9·19 to -6·81)	-4·92 (-7·24 to -2·47)
	5-14 years	207 (146 to 286)	2·89 (2·04 to 3·99)	-4·28 (-5·56 to -3·14)	-4·89 (-6·20 to -3·76)	7·59 (6·28 to 9·04)	0·106 (0·0876 to 0·126)	-7·12 (-7·85 to -6·35)	-4·38 (-5·72 to -2·96)
	15-49 years	5750 (4690 to 7120)	22·0 (18·0 to 27·3)	-2·65 (-3·24 to -2·01)	-1·17 (-1·88 to -0·522)	403 (310 to 544)	1·54 (1·19 to 2·08)	-3·97 (-4·56 to -3·03)	-2·03 (-2·89 to -1·21)
	50-69 years	3310 (2490 to 4380)	36·2 (27·3 to 47·9)	-2·79 (-3·56 to -1·87)	-1·88 (-2·82 to -0·870)	441 (359 to 521)	4·82 (3·93 to 5·70)	-5·43 (-5·89 to -4·83)	-1·36 (-2·70 to -0·109)
	70+ years	1670 (1330 to 2050)	52·4 (41·6 to 64·5)	-2·61 (-3·47 to -1·90)	-2·79 (-3·75 to -1·90)	418 (359 to 481)	13·1 (11·3 to 15·1)	-5·45 (-5·84 to -5·00)	-1·99 (-3·36 to -0·556)
Costa Rica	All Ages	434 (377 to 500)	8·27 (7·19 to 9·47)	-2·72 (-3·06 to -2·27)	-1·93 (-2·53 to -1·26)	65·9 (58·9 to 72·9)	1·21 (1·09 to 1·34)	-4·94 (-5·26 to -4·64)	-0·789 (-1·79 to -0·0217)
	Under 5	6·21 (4·84 to 7·84)	2·01 (1·57 to 2·54)	-4·47 (-5·32 to -3·60)	-3·96 (-5·43 to -2·78)	0·285 (0·228 to 0·353)	0·0924 (0·0740 to 0·114)	-8·85 (-9·36 to -8·20)	-5·95 (-7·04 to -4·66)
	5-14 years	9·81 (6·53 to 13·9)	1·38 (0·921 to 1·96)	-3·21 (-4·15 to -2·41)	-4·80 (-6·17 to -3·17)	0·306 (0·255 to 0·370)	0·0432 (0·0359 to 0·0521)	-7·49 (-8·00 to -7·01)	-4·35 (-5·25 to -3·41)
	15-49 years	213 (179 to 268)	8·52 (7·15 to 10·7)	-3·15 (-3·56 to -2·50)	-2·76 (-3·56 to -2·05)	20·7 (17·3 to 24·8)	0·828 (0·690 to 0·990)	-5·06 (-5·53 to -4·64)	-1·12 (-2·03 to -0·385)
	50-69 years	139 (106 to 175)	15·2 (11·6 to 19·1)	-3·99 (-4·50 to -3·33)	-2·31 (-3·15 to -1·45)	24·5 (21·1 to 27·3)	2·68 (2·31 to 2·99)	-6·91 (-7·38 to -6·50)	-1·69 (-2·92 to -0·610)
	70+ years	65·9 (52·6 to 81·0)	21·0 (16·8 to 25·8)	-4·66 (-5·29 to -4·11)	-3·31 (-4·39 to -2·27)	20·1 (17·4 to 23·1)	6·41 (5·56 to 7·36)	-7·05 (-7·47 to -6·56)	-4·57 (-5·71 to -3·51)
El Salvador	All Ages	2510 (2190 to 2860)	39·1 (34·3 to 44·8)	-2·88 (-3·31 to -2·44)	-2·09 (-2·73 to -1·52)	253 (199 to 334)	4·01 (3·15 to 5·32)	-5·69 (-6·50 to -4·91)	-1·57 (-3·63 to 0·758)
	Under 5	49·5 (39·6 to 63·7)	8·24 (6·59 to 10·6)	-6·69 (-7·78 to -5·86)	-4·29 (-6·08 to -2·54)	2·60 (2·08 to 3·42)	0·433 (0·347 to 0·569)	-12·2 (-13·4 to -11·2)	-7·20 (-9·29 to -4·46)
	5-14 years	96·8 (64·3 to 135)	7·95 (5·28 to 11·1)	-4·82 (-5·86 to -3·70)	-3·93 (-5·43 to -2·64)	2·67 (2·13 to 3·47)	0·219 (0·175 to 0·285)	-7·63 (-8·76 to -6·59)	-5·04 (-6·96 to -2·73)
	15-49 years	1400 (1160 to 1710)	42·4 (34·9 to 51·7)	-2·68 (-3·21 to -2·17)	-2·72 (-3·49 to -1·91)	94·8 (73·0 to 128)	2·86 (2·20 to 3·87)	-5·14 (-6·11 to -3·99)	-2·53 (-4·37 to -0·251)
	50-69 years	625 (471 to 787)	66·7 (50·2 to 83·9)	-3·77 (-4·38 to -3·27)	-2·21 (-3·09 to -1·34)	74·9 (54·1 to 105)	7·98 (5·77 to 11·2)	-7·18 (-8·25 to -6·32)	-2·20 (-4·30 to 0·693)
	70+ years	330 (272 to 409)	86·1 (71·1 to 107)	-4·58 (-5·28 to -3·88)	-2·65 (-3·38 to -1·69)	77·6 (58·9 to 99·3)	20·3 (15·4 to 25·9)	-7·40 (-8·39 to -6·31)	-2·69 (-5·04 to -0·383)
Guatemala	All Ages	3000 (2710 to 3390)	21·4 (19·2 to 24·3)	-3·31 (-3·71 to -2·91)	-1·68 (-2·22 to -1·16)	581 (497 to 680)	4·61 (3·95 to 5·35)	-7·28 (-8·00 to -6·67)	-2·44 (-3·45 to -1·38)
	Under 5	94·7 (76·3 to 115)	6·07 (4·89 to 7·37)	-5·49 (-6·16 to -4·68)	-3·96 (-5·38 to -2·77)	13·7 (11·0 to 16·1)	0·878 (0·704 to 1·04)	-9·85 (-10·8 to -8·97)	-8·11 (-9·69 to -6·27)
	5-14 years	160 (116 to 217)	4·73 (3·43 to 6·42)	-4·89 (-5·94 to -3·92)	-3·33 (-4·69 to -1·98)	8·92 (7·33 to 10·4)	0·264 (0·217 to 0·308)	-10·6 (-11·6 to -9·78)	-5·45 (-6·31 to -4·45)
	15-49 years	1740 (1470 to 2110)	20·7 (17·5 to 25·1)	-3·25 (-3·78 to -2·64)	-2·57 (-3·16 to -1·85)	274 (226 to 328)	3·25 (2·68 to 3·90)	-6·75 (-7·70 to -5·85)	-4·26 (-5·13 to -3·40)

eTable 1. All-form tuberculosis incident cases and deaths, age-standardised rates of incidence and mortality per 100,000 population, and corresponding annualized rates of change by age groups for 204 countries and territories (2021).

Location	Age group	Number of cases	Rate per 100,000 cases	Annualized rate of change (1990-2010)	Annualized rate of change (2010-2021)	Number of deaths	Rate per 100,000 deaths	Annualized deaths (1990-2010)	Annualized deaths (2010-2021)
	50-69 years	747 (587 to 931)	40·8 (32·1 to 50·8)	-3·52 (-4·14 to -2·96)	-2·05 (-3·02 to -1·06)	172 (147 to 201)	9·42 (8·02 to 11·0)	-8·45 (-9·02 to -7·84)	-2·76 (-4·06 to -1·45)
	70+ years	260 (207 to 319)	44·1 (35·1 to 54·2)	-6·06 (-6·99 to -5·15)	-3·35 (-4·20 to -2·17)	113 (98·4 to 128)	19·1 (16·7 to 21·7)	-9·53 (-10·0 to -9·15)	-2·39 (-3·59 to -1·10)
Honduras	All Ages	3470 (3080 to 3910)	41·6 (37·2 to 46·8)	-2·00 (-2·43 to -1·55)	-2·10 (-2·64 to -1·53)	744 (579 to 982)	10·8 (8·19 to 14·1)	-3·37 (-4·80 to -2·15)	-2·42 (-4·80 to -0·0557)
	Under 5	97·2 (78·4 to 116)	8·87 (7·16 to 10·6)	-4·64 (-5·30 to -3·88)	-4·08 (-5·26 to -2·96)	13·0 (9·91 to 17·5)	1·19 (0·904 to 1·59)	-7·63 (-8·87 to -6·50)	-5·61 (-7·46 to -3·91)
	5-14 years	169 (113 to 236)	7·73 (5·18 to 10·8)	-4·42 (-5·42 to -3·34)	-4·84 (-6·33 to -3·40)	8·54 (5·76 to 11·3)	0·391 (0·264 to 0·517)	-7·78 (-9·13 to -6·33)	-6·46 (-9·26 to -3·79)
	15-49 years	1850 (1550 to 2190)	34·1 (28·6 to 40·5)	-2·50 (-2·99 to -2·03)	-3·04 (-3·76 to -2·31)	236 (160 to 327)	4·36 (2·95 to 6·04)	-5·22 (-6·87 to -3·79)	-3·60 (-6·64 to -0·299)
	50-69 years	978 (778 to 1200)	88·6 (70·5 to 109)	-1·18 (-1·60 to -0·747)	-1·71 (-2·70 to -0·511)	277 (202 to 381)	25·0 (18·3 to 34·5)	-3·01 (-4·71 to -1·20)	-2·79 (-5·75 to 0·496)
	70+ years	382 (304 to 484)	121 (96·6 to 154)	-1·90 (-2·57 to -0·995)	-2·39 (-3·41 to -1·47)	209 (146 to 273)	66·5 (46·4 to 86·5)	-2·38 (-4·52 to -0·972)	-3·55 (-6·37 to -0·988)
Mexico	All Ages	25000 (21700 to 29300)	18·6 (16·2 to 21·6)	-2·25 (-2·54 to -1·97)	0·888 (0·419 to 1·37)	3190 (2780 to 3670)	2·42 (2·12 to 2·77)	-5·43 (-5·84 to -4·95)	-2·10 (-2·95 to -1·29)
	Under 5	284 (232 to 360)	2·88 (2·35 to 3·64)	-5·46 (-5·99 to -4·84)	-2·46 (-3·02 to -1·71)	31·2 (24·7 to 38·4)	0·316 (0·250 to 0·389)	-10·3 (-11·5 to -9·53)	-2·91 (-4·43 to -0·772)
	5-14 years	840 (520 to 1230)	3·79 (2·34 to 5·54)	-3·68 (-4·22 to -3·10)	0·413 (-0·436 to 1·27)	28·2 (25·0 to 32·3)	0·127 (0·113 to 0·145)	-9·51 (-10·2 to -8·80)	-1·73 (-2·36 to -0·847)
	15-49 years	13800 (11300 to 17200)	20·2 (16·5 to 25·1)	-2·48 (-2·81 to -2·14)	0·744 (0·256 to 1·22)	1430 (1210 to 1710)	2·09 (1·76 to 2·49)	-5·03 (-5·67 to -4·26)	-2·18 (-2·82 to -1·54)
	50-69 years	7660 (5810 to 10100)	34·8 (26·4 to 45·8)	-3·06 (-3·40 to -2·76)	-0·435 (-1·17 to 0·288)	1060 (917 to 1210)	4·82 (4·17 to 5·49)	-7·26 (-7·62 to -6·95)	-3·66 (-4·77 to -2·70)
	70+ years	2440 (1900 to 3110)	36·1 (28·2 to 46·1)	-4·92 (-5·39 to -4·45)	-2·60 (-3·06 to -2·17)	642 (577 to 709)	9·50 (8·54 to 10·5)	-8·18 (-8·38 to -7·99)	-5·63 (-6·58 to -4·73)
Nicaragua	All Ages	1970 (1720 to 2250)	32·0 (28·2 to 36·4)	-2·68 (-3·14 to -2·21)	-2·10 (-2·67 to -1·54)	238 (186 to 302)	4·52 (3·49 to 5·78)	-4·84 (-5·49 to -3·61)	-3·22 (-5·43 to -1·16)
	Under 5	47·9 (38·8 to 59·6)	7·37 (5·97 to 9·16)	-6·11 (-7·15 to -5·10)	-4·79 (-6·20 to -3·36)	4·66 (3·42 to 6·06)	0·717 (0·525 to 0·931)	-10·9 (-12·1 to -9·47)	-8·27 (-10·4 to -5·28)
	5-14 years	80·7 (55·1 to 112)	6·07 (4·14 to 8·44)	-5·59 (-6·72 to -4·54)	-4·46 (-5·86 to -3·29)	1·89 (1·50 to 2·30)	0·142 (0·112 to 0·173)	-8·71 (-9·96 to -7·42)	-9·55 (-11·2 to -7·58)
	15-49 years	1150 (929 to 1430)	31·9 (25·9 to 39·8)	-2·98 (-3·55 to -2·55)	-2·35 (-3·20 to -1·61)	84·2 (64·9 to 104)	2·35 (1·81 to 2·90)	-5·31 (-5·95 to -4·24)	-3·49 (-5·61 to -1·57)
	50-69 years	502 (382 to 628)	59·2 (45·1 to 74·1)	-2·84 (-3·38 to -2·23)	-2·99 (-4·00 to -1·98)	79·2 (59·4 to 110)	9·36 (7·02 to 13·0)	-5·56 (-6·48 to -3·89)	-4·49 (-7·02 to -1·99)
	70+ years	195 (157 to 239)	78·2 (62·8 to 95·6)	-3·19 (-3·88 to -2·60)	-3·71 (-4·68 to -2·64)	68·4 (52·3 to 87·4)	27·4 (20·9 to 35·0)	-4·85 (-5·83 to -3·72)	-5·29 (-7·86 to -3·11)
Panama	All Ages	1540 (1350 to 1810)	35·1 (30·9 to 41·3)	-1·37 (-1·79 to -0·916)	-1·91 (-2·52 to -1·32)	209 (168 to 246)	4·76 (3·81 to 5·60)	-3·32 (-3·81 to -2·66)	-3·21 (-4·74 to -1·89)
	Under 5	34·9 (28·0 to 44·0)	9·41 (7·55 to 11·9)	-4·11 (-4·97 to -3·26)	-3·69 (-5·03 to -2·13)	4·04 (3·12 to 5·03)	1·09 (0·840 to 1·35)	-5·36 (-6·45 to -4·22)	-6·40 (-7·79 to -4·88)

eTable 1. All-form tuberculosis incident cases and deaths, age-standardised rates of incidence and mortality per 100,000 population, and corresponding annualized rates of change by age groups for 204 countries and territories (2021).

Location	Age group	Number of cases	Rate per 100,000 cases	Annualized rate of change (1990-2010)	Annualized rate of change (2010-2021)	Number of deaths	Rate per 100,000 deaths	Annualized deaths (1990-2010)	Annualized deaths (2010-2021)
	5-14 years	62·6 (43·4 to 84·8)	8·01 (5·55 to 10·8)	-5·28 (-6·07 to -4·29)	-2·61 (-4·44 to -1·12)	2·12 (1·72 to 2·58)	0·271 (0·220 to 0·330)	-5·83 (-6·73 to -4·75)	-5·59 (-6·83 to -4·21)
	15-49 years	883 (728 to 1120)	40·8 (33·6 to 51·8)	-0·883 (-1·35 to -0·360)	-2·08 (-2·86 to -1·45)	83·5 (65·7 to 110)	3·86 (3·04 to 5·06)	-3·02 (-3·72 to -2·08)	-3·58 (-4·63 to -2·58)
	50-69 years	398 (293 to 499)	55·7 (41·0 to 69·9)	-1·59 (-2·18 to -0·955)	-2·10 (-2·93 to -1·06)	65·3 (51·9 to 79·3)	9·15 (7·28 to 11·1)	-4·31 (-5·02 to -3·64)	-3·91 (-5·89 to -2·19)
	70+ years	158 (125 to 194)	60·6 (47·7 to 74·5)	-3·25 (-3·87 to -2·70)	-3·58 (-4·56 to -2·43)	54·2 (44·0 to 62·8)	20·8 (16·9 to 24·0)	-5·52 (-5·92 to -5·12)	-4·75 (-6·75 to -3·12)
Venezuela (Bolivarian Republic of)	All Ages	7250 (6450 to 8290)	25·8 (22·8 to 29·2)	-2·25 (-2·69 to -1·77)	1·48 (1·03 to 2·00)	1150 (912 to 1470)	3·98 (3·15 to 5·07)	-3·92 (-4·25 to -3·54)	3·13 (1·12 to 5·12)
	Under 5	129 (104 to 163)	5·90 (4·74 to 7·48)	-4·81 (-5·58 to -3·91)	-1·47 (-2·82 to -0·0316)	11·5 (8·84 to 14·7)	0·527 (0·404 to 0·674)	-10·0 (-10·5 to -9·46)	1·34 (-0·699 to 3·68)
	5-14 years	216 (149 to 293)	4·87 (3·37 to 6·61)	-4·20 (-5·41 to -2·97)	-0·278 (-1·40 to 1·04)	9·27 (7·55 to 11·7)	0·209 (0·170 to 0·263)	-6·75 (-7·32 to -5·74)	1·64 (0·198 to 3·24)
	15-49 years	4000 (3310 to 4910)	30·4 (25·2 to 37·4)	-2·50 (-3·19 to -1·69)	1·94 (1·21 to 2·76)	430 (318 to 568)	3·27 (2·42 to 4·32)	-3·28 (-3·79 to -2·49)	2·88 (1·31 to 4·56)
	50-69 years	2120 (1600 to 2620)	40·4 (30·4 to 50·0)	-3·11 (-3·82 to -2·30)	-0·481 (-1·19 to 0·269)	409 (303 to 534)	7·79 (5·77 to 10·2)	-5·93 (-6·34 to -5·48)	1·24 (-1·15 to 3·56)
	70+ years	788 (626 to 997)	49·1 (39·0 to 62·1)	-3·73 (-4·42 to -3·15)	-2·43 (-3·14 to -1·69)	294 (234 to 377)	18·3 (14·6 to 23·5)	-6·24 (-6·69 to -5·83)	-1·07 (-3·27 to 1·29)
Tropical Latin America	All Ages	87300 (75700 to 103000)	35·2 (30·6 to 41·3)	-1·21 (-1·55 to -0·814)	-1·25 (-1·71 to -0·613)	8360 (7270 to 9580)	3·26 (2·84 to 3·74)	-2·44 (-2·80 to -1·92)	-2·22 (-2·58 to -1·89)
	Under 5	1100 (908 to 1370)	6·38 (5·28 to 7·97)	-5·28 (-5·66 to -4·82)	-1·93 (-2·62 to -1·16)	113 (92·2 to 138)	0·654 (0·536 to 0·800)	-7·74 (-8·77 to -6·74)	-7·62 (-9·09 to -6·00)
	5-14 years	2010 (1320 to 2880)	6·08 (3·99 to 8·72)	-3·88 (-4·47 to -3·13)	0·207 (-1·13 to 1·41)	49·7 (39·8 to 61·3)	0·151 (0·121 to 0·186)	-4·14 (-4·99 to -3·32)	-5·94 (-6·68 to -5·20)
	15-49 years	55100 (45200 to 68600)	46·0 (37·8 to 57·2)	-1·58 (-2·07 to -1·06)	-1·32 (-2·05 to -0·643)	3700 (3030 to 4500)	3·08 (2·52 to 3·76)	-2·93 (-3·40 to -2·26)	-2·61 (-2·93 to -2·28)
	50-69 years	22300 (16700 to 29600)	51·8 (38·6 to 68·5)	-1·58 (-2·06 to -1·14)	-2·15 (-3·07 to -1·24)	3050 (2710 to 3450)	7·08 (6·29 to 7·99)	-3·06 (-3·39 to -2·70)	-3·05 (-3·49 to -2·54)
	70+ years	6730 (5240 to 8570)	46·9 (36·5 to 59·7)	-2·73 (-3·22 to -2·25)	-3·45 (-4·05 to -2·83)	1450 (1320 to 1540)	10·1 (9·20 to 10·7)	-3·68 (-3·95 to -3·47)	-4·26 (-4·65 to -3·88)
Brazil	All Ages	85000 (73600 to 100000)	35·3 (30·7 to 41·5)	-1·22 (-1·58 to -0·835)	-1·24 (-1·72 to -0·589)	8030 (7010 to 9230)	3·22 (2·81 to 3·70)	-2·49 (-2·86 to -1·96)	-2·20 (-2·57 to -1·90)
	Under 5	1050 (868 to 1310)	6·33 (5·24 to 7·94)	-5·41 (-5·80 to -4·93)	-1·84 (-2·51 to -1·06)	104 (85·1 to 128)	0·626 (0·514 to 0·773)	-7·94 (-8·98 to -6·93)	-7·72 (-9·23 to -6·18)
	5-14 years	1920 (1260 to 2760)	6·07 (3·98 to 8·73)	-3·97 (-4·57 to -3·21)	0·338 (-1·07 to 1·60)	46·1 (36·9 to 57·6)	0·146 (0·117 to 0·182)	-4·20 (-5·08 to -3·33)	-6·02 (-6·80 to -5·29)
	15-49 years	53700 (44000 to 66700)	46·3 (37·9 to 57·6)	-1·59 (-2·09 to -1·07)	-1·30 (-2·04 to -0·608)	3560 (2920 to 4350)	3·07 (2·52 to 3·75)	-3·00 (-3·46 to -2·31)	-2·59 (-2·94 to -2·27)
	50-69 years	21800 (16200 to 28900)	51·7 (38·5 to 68·6)	-1·60 (-2·08 to -1·16)	-2·15 (-3·12 to -1·23)	2940 (2620 to 3330)	6·98 (6·23 to 7·91)	-3·11 (-3·45 to -2·74)	-3·04 (-3·48 to -2·55)

eTable 1. All-form tuberculosis incident cases and deaths, age-standardised rates of incidence and mortality per 100,000 population, and corresponding annualized rates of change by age groups for 204 countries and territories (2021).

Location	Age group	Number of cases	Rate per 100,000 cases	Annualized rate of change (1990-2010)	Annualized rate of change (2010-2021)	Number of deaths	Rate per 100,000 deaths	Annualized deaths (1990-2010)	Annualized deaths (2010-2021)
	70+ years	6560 (5090 to 8360)	46·6 (36·2 to 59·4)	-2·77 (-3·26 to -2·29)	-3·47 (-4·09 to -2·84)	1380 (1260 to 1470)	9·81 (8·98 to 10·4)	-3·74 (-4·02 to -3·55)	-4·27 (-4·66 to -3·88)
Paraguay	All Ages	2280 (1980 to 2660)	32·5 (28·4 to 37·7)	-0·288 (-0·694 to 0·167)	-1·47 (-2·06 to -0·900)	325 (254 to 408)	5·08 (3·99 to 6·39)	-0·747 (-1·47 to 0·246)	-2·75 (-4·60 to -0·779)
	Under 5	48·7 (39·7 to 61·1)	7·50 (6·11 to 9·40)	-2·03 (-2·62 to -1·48)	-3·58 (-4·60 to -2·38)	8·90 (6·80 to 11·2)	1·37 (1·05 to 1·72)	-3·69 (-4·90 to -2·25)	-6·11 (-8·25 to -3·24)
	5-14 years	86·5 (57·6 to 122)	6·37 (4·25 to 8·98)	-1·91 (-2·76 to -1·13)	-2·46 (-3·77 to -1·24)	3·55 (2·82 to 4·36)	0·261 (0·207 to 0·321)	-3·39 (-4·49 to -2·22)	-4·99 (-6·90 to -3·04)
	15-49 years	1440 (1180 to 1780)	37·3 (30·6 to 46·1)	-0·662 (-1·10 to -0·139)	-1·85 (-2·73 to -1·17)	136 (103 to 163)	3·52 (2·68 to 4·22)	-0·524 (-1·39 to 0·388)	-3·41 (-5·09 to -1·78)
	50-69 years	531 (388 to 660)	53·1 (38·8 to 66·0)	-0·459 (-0·980 to 0·171)	-2·14 (-3·18 to -0·909)	111 (85·1 to 149)	11·1 (8·51 to 14·9)	-1·39 (-2·42 to 0·193)	-3·47 (-5·89 to -0·908)
	70+ years	178 (144 to 221)	59·1 (47·6 to 73·2)	-1·22 (-1·70 to -0·694)	-2·59 (-3·63 to -1·67)	66·1 (49·6 to 84·2)	21·9 (16·4 to 27·9)	-2·04 (-2·90 to -1·08)	-3·83 (-6·27 to -1·16)
North Africa and Middle East	All Ages	169000 (149000 to 195000)	29·7 (26·4 to 33·8)	-2·36 (-2·59 to -2·05)	-2·87 (-3·31 to -2·49)	19600 (15600 to 26900)	4·13 (3·27 to 5·76)	-3·94 (-4·58 to -3·09)	-3·81 (-5·23 to -2·18)
	Under 5	7310 (5740 to 9170)	12·0 (9·39 to 15·0)	-3·20 (-3·70 to -2·74)	-5·47 (-6·35 to -4·72)	1030 (767 to 1310)	1·68 (1·26 to 2·15)	-5·51 (-6·26 to -4·74)	-6·94 (-8·62 to -4·38)
	5-14 years	14800 (10500 to 20500)	12·1 (8·62 to 16·8)	-2·21 (-2·54 to -1·75)	-6·22 (-7·21 to -5·34)	424 (337 to 582)	0·347 (0·276 to 0·477)	-3·66 (-4·41 to -2·85)	-6·80 (-8·29 to -4·95)
	15-49 years	88700 (72700 to 108000)	26·5 (21·7 to 32·3)	-2·59 (-2·93 to -2·13)	-2·58 (-2·96 to -2·18)	6740 (5370 to 8920)	2·02 (1·61 to 2·67)	-3·56 (-4·39 to -2·74)	-3·77 (-5·18 to -1·91)
	50-69 years	39800 (30300 to 49600)	46·8 (35·6 to 58·4)	-2·80 (-3·33 to -2·29)	-2·95 (-3·46 to -2·42)	5790 (4500 to 8260)	6·81 (5·29 to 9·72)	-5·20 (-6·04 to -4·14)	-4·92 (-6·60 to -3·24)
	70+ years	18100 (14500 to 22500)	88·8 (71·3 to 111)	-3·24 (-3·57 to -2·89)	-3·09 (-3·49 to -2·73)	5660 (4280 to 8480)	27·9 (21·1 to 41·7)	-4·70 (-5·79 to -3·65)	-4·55 (-6·00 to -2·82)
Afghanistan	All Ages	33400 (28400 to 38500)	161 (143 to 183)	-1·78 (-2·25 to -1·43)	-5·07 (-6·19 to -4·14)	6590 (4970 to 9890)	47·5 (33·3 to 81·9)	-4·52 (-5·69 to -3·48)	-5·63 (-7·46 to -3·71)
	Under 5	2610 (2040 to 3330)	47·6 (37·2 to 60·7)	-2·60 (-3·40 to -1·69)	-8·16 (-9·76 to -6·73)	644 (462 to 875)	11·7 (8·42 to 16·0)	-5·49 (-6·86 to -3·86)	-7·28 (-10·4 to -4·62)
	5-14 years	4790 (3280 to 6750)	55·0 (37·6 to 77·4)	-0·250 (-1·14 to 0·909)	-9·15 (-11·1 to -7·70)	246 (172 to 377)	2·82 (1·98 to 4·32)	-3·37 (-4·98 to -1·83)	-6·71 (-9·61 to -4·21)
	15-49 years	19100 (15200 to 23600)	129 (103 to 159)	-1·61 (-2·27 to -0·941)	-3·70 (-4·95 to -2·61)	2930 (2170 to 4320)	19·8 (14·7 to 29·2)	-2·98 (-4·35 to -1·63)	-5·19 (-7·55 to -2·62)
	50-69 years	5020 (3690 to 6190)	273 (201 to 337)	0·136 (-0·490 to 0·880)	-4·64 (-5·98 to -3·13)	1670 (1160 to 2610)	91·0 (63·2 to 142)	-1·73 (-3·31 to 0·0172)	-6·61 (-9·34 to -3·46)
	70+ years	1910 (1500 to 2480)	472 (370 to 613)	-1·24 (-1·91 to -0·518)	-2·56 (-3·79 to -1·28)	1100 (715 to 2090)	271 (177 to 515)	-1·82 (-3·55 to -0·268)	-4·09 (-6·83 to -1·45)
Algeria	All Ages	18200 (16200 to 21400)	45·2 (40·1 to 51·9)	-2·96 (-3·55 to -2·54)	-2·47 (-3·15 to -1·94)	1610 (1260 to 2120)	5·19 (4·02 to 6·88)	-4·15 (-5·16 to -2·85)	-2·76 (-5·12 to 0·0477)
	Under 5	723 (535 to 930)	15·4 (11·4 to 19·8)	-4·24 (-5·16 to -3·32)	-5·09 (-6·79 to -3·43)	41·7 (28·7 to 59·8)	0·887 (0·610 to 1·27)	-8·01 (-9·57 to -5·98)	-9·19 (-12·7 to -5·05)

eTable 1. All-form tuberculosis incident cases and deaths, age-standardised rates of incidence and mortality per 100,000 population, and corresponding annualized rates of change by age groups for 204 countries and territories (2021).

Location	Age group	Number of cases	Rate per 100,000 cases	Annualized rate of change (1990-2010)	Annualized rate of change (2010-2021)	Number of deaths	Rate per 100,000 deaths	Annualized deaths (1990-2010)	Annualized deaths (2010-2021)
	5-14 years	1390 (944 to 1950)	16·1 (11·0 to 22·7)	-4·50 (-5·93 to -3·31)	-4·54 (-5·90 to -3·14)	19·9 (14·5 to 27·9)	0·231 (0·168 to 0·324)	-7·76 (-8·92 to -6·11)	-6·66 (-9·33 to -3·70)
	15-49 years	8390 (6800 to 10500)	37·1 (30·0 to 46·2)	-3·40 (-3·91 to -2·90)	-2·59 (-3·42 to -1·77)	386 (298 to 527)	1·70 (1·32 to 2·33)	-4·97 (-5·96 to -3·76)	-3·08 (-5·35 to 0·0139)
	50-69 years	5000 (3870 to 6300)	76·6 (59·3 to 96·7)	-3·14 (-3·69 to -2·71)	-2·53 (-3·27 to -1·67)	437 (315 to 615)	6·70 (4·83 to 9·44)	-4·91 (-6·34 to -3·10)	-4·16 (-6·74 to -0·565)
	70+ years	2730 (2150 to 3440)	158 (124 to 199)	-3·01 (-3·60 to -2·47)	-2·37 (-3·41 to -1·40)	723 (536 to 962)	41·8 (31·0 to 55·6)	-4·24 (-5·58 to -2·95)	-3·21 (-5·91 to -0·138)
Bahrain	All Ages	213 (178 to 256)	15·4 (13·5 to 17·9)	-2·79 (-3·24 to -2·10)	-0·587 (-1·26 to -0·0273)	11·7 (9·47 to 16·2)	1·55 (1·26 to 2·06)	-5·55 (-6·66 to -4·16)	-2·85 (-4·62 to -0·327)
	Under 5	4·31 (3·21 to 5·53)	4·60 (3·43 to 5·91)	-4·34 (-5·28 to -3·40)	-1·46 (-2·88 to 0·255)	0·144 (0·107 to 0·186)	0·153 (0·115 to 0·198)	-7·11 (-8·27 to -5·83)	-4·06 (-6·36 to -1·34)
	5-14 years	10·8 (7·47 to 15·1)	5·31 (3·68 to 7·43)	-3·64 (-4·57 to -2·72)	-0·361 (-1·91 to 1·12)	0·0929 (0·0690 to 0·128)	0·0458 (0·0340 to 0·0631)	-5·68 (-7·09 to -3·69)	-5·18 (-7·22 to -2·00)
	15-49 years	135 (107 to 165)	13·7 (10·9 to 16·8)	-2·96 (-3·48 to -2·25)	-0·686 (-1·58 to 0·123)	4·56 (3·51 to 6·70)	0·466 (0·359 to 0·684)	-5·48 (-6·63 to -3·99)	-3·02 (-4·87 to -0·0596)
	50-69 years	50·3 (38·8 to 63·2)	22·0 (17·0 to 27·7)	-4·33 (-5·16 to -3·53)	-1·99 (-2·94 to -1·02)	3·88 (3·07 to 5·19)	1·70 (1·34 to 2·27)	-7·72 (-8·89 to -6·20)	-5·53 (-7·88 to -3·21)
	70+ years	12·7 (10·0 to 16·1)	50·4 (39·7 to 64·0)	-3·09 (-3·65 to -2·48)	-3·21 (-4·35 to -2·27)	3·03 (2·29 to 4·13)	12·0 (9·09 to 16·4)	-5·64 (-7·03 to -4·11)	-6·07 (-8·37 to -3·79)
Egypt	All Ages	9070 (8030 to 10100)	10·1 (9·02 to 11·1)	-3·55 (-3·95 to -3·15)	-3·39 (-4·08 to -2·77)	850 (679 to 1050)	1·31 (1·04 to 1·59)	-5·06 (-5·85 to -4·12)	-4·50 (-7·00 to -1·84)
	Under 5	494 (393 to 631)	3·79 (3·01 to 4·84)	-5·23 (-6·22 to -4·26)	-5·30 (-6·70 to -4·05)	27·5 (19·7 to 34·5)	0·211 (0·151 to 0·265)	-6·75 (-8·47 to -5·18)	-9·42 (-12·8 to -5·49)
	5-14 years	787 (566 to 1100)	3·31 (2·37 to 4·61)	-5·09 (-6·30 to -3·74)	-5·51 (-6·92 to -4·03)	14·1 (11·0 to 18·5)	0·0594 (0·0462 to 0·0776)	-7·50 (-8·87 to -6·11)	-9·44 (-12·6 to -5·67)
	15-49 years	4940 (4080 to 5910)	9·25 (7·63 to 11·1)	-3·63 (-4·15 to -3·13)	-3·08 (-3·85 to -2·19)	283 (222 to 365)	0·529 (0·415 to 0·683)	-6·26 (-7·29 to -5·16)	-4·22 (-7·05 to -1·55)
	50-69 years	2220 (1840 to 2770)	17·0 (14·1 to 21·2)	-2·79 (-3·50 to -2·26)	-3·14 (-4·53 to -2·08)	340 (259 to 432)	2·60 (1·98 to 3·31)	-4·90 (-5·83 to -3·84)	-5·08 (-7·91 to -2·34)
	70+ years	622 (498 to 782)	27·4 (21·9 to 34·4)	-2·70 (-3·56 to -1·89)	-3·72 (-5·01 to -2·34)	186 (144 to 236)	8·20 (6·36 to 10·4)	-3·55 (-4·61 to -2·31)	-4·69 (-7·44 to -2·30)
Iran (Islamic Republic of)	All Ages	13300 (12000 to 15000)	15·7 (14·1 to 17·4)	-0·676 (-1·04 to -0·313)	-0·419 (-0·901 to 0·0906)	963 (830 to 1150)	1·23 (1·04 to 1·48)	-2·33 (-3·26 to -0·925)	-1·53 (-2·60 to 0·399)
	Under 5	274 (223 to 352)	4·46 (3·63 to 5·73)	-3·38 (-3·80 to -3·08)	-5·92 (-6·60 to -5·21)	12·2 (10·4 to 13·7)	0·198 (0·169 to 0·222)	-6·60 (-7·82 to -5·12)	-12·4 (-13·8 to -11·0)
	5-14 years	870 (608 to 1230)	6·21 (4·33 to 8·77)	-1·90 (-2·52 to -1·32)	-3·32 (-3·89 to -2·58)	14·3 (12·6 to 16·0)	0·102 (0·0898 to 0·114)	-3·04 (-4·25 to -1·47)	-6·93 (-8·07 to -5·55)
	15-49 years	7050 (5880 to 8490)	14·9 (12·4 to 17·9)	-0·626 (-0·979 to -0·306)	-0·142 (-0·944 to 0·713)	332 (279 to 391)	0·701 (0·589 to 0·824)	-1·59 (-2·57 to 0·0139)	-1·03 (-2·24 to 0·631)
	50-69 years	3370 (2670 to 4180)	23·9 (18·9 to 29·6)	-1·95 (-2·23 to -1·65)	-0·865 (-1·21 to -0·505)	262 (224 to 318)	1·85 (1·59 to 2·25)	-5·00 (-6·03 to -3·41)	-2·69 (-3·83 to -0·544)

eTable 1. All-form tuberculosis incident cases and deaths, age-standardised rates of incidence and mortality per 100,000 population, and corresponding annualized rates of change by age groups for 204 countries and territories (2021).

Location	Age group	Number of cases	Rate per 100,000 cases	Annualized rate of change (1990-2010)	Annualized rate of change (2010-2021)	Number of deaths	Rate per 100,000 deaths	Annualized deaths (1990-2010)	Annualized deaths (2010-2021)
	70+ years	1770 (1460 to 2160)	48·6 (40·0 to 59·2)	-1·23 (-1·57 to -0·858)	-0·874 (-1·23 to -0·462)	342 (282 to 422)	9·36 (7·74 to 11·5)	-3·82 (-5·06 to -2·55)	-2·66 (-4·01 to -0·497)
Iraq	All Ages	9130 (7840 to 10600)	27·3 (24·1 to 31·5)	-2·67 (-3·17 to -2·24)	-4·00 (-4·58 to -3·38)	859 (630 to 1140)	3·66 (2·72 to 4·85)	-4·28 (-5·81 to -2·88)	-4·82 (-7·60 to -1·84)
	Under 5	332 (252 to 418)	7·73 (5·86 to 9·74)	-4·04 (-4·91 to -3·03)	-6·58 (-8·20 to -4·93)	15·9 (11·2 to 23·4)	0·371 (0·260 to 0·545)	-6·83 (-8·56 to -4·96)	-10·8 (-14·5 to -6·92)
	5-14 years	764 (538 to 1070)	8·33 (5·86 to 11·7)	-3·48 (-4·66 to -2·60)	-7·93 (-9·54 to -6·34)	11·3 (8·77 to 15·6)	0·124 (0·0956 to 0·171)	-6·00 (-7·96 to -4·43)	-13·1 (-16·0 to -10·2)
	15-49 years	4730 (3830 to 5920)	21·4 (17·3 to 26·7)	-2·67 (-3·19 to -2·15)	-4·66 (-5·35 to -3·96)	244 (174 to 362)	1·10 (0·785 to 1·64)	-4·03 (-5·70 to -2·38)	-7·43 (-10·6 to -3·69)
	50-69 years	2390 (1810 to 3060)	51·8 (39·3 to 66·3)	-2·55 (-3·26 to -1·95)	-3·31 (-4·06 to -2·41)	310 (214 to 439)	6·72 (4·63 to 9·52)	-4·81 (-6·93 to -2·87)	-5·45 (-8·66 to -1·68)
	70+ years	908 (721 to 1170)	91·5 (72·7 to 117)	-2·80 (-3·29 to -2·22)	-2·75 (-3·72 to -1·76)	278 (206 to 390)	28·0 (20·8 to 39·3)	-4·18 (-5·85 to -2·48)	-4·06 (-7·20 to -0·446)
Jordan	All Ages	651 (560 to 750)	6·26 (5·55 to 7·16)	-3·10 (-3·46 to -2·67)	-1·55 (-2·08 to -0·951)	48·3 (36·2 to 61·1)	0·684 (0·513 to 0·861)	-4·86 (-6·22 to -3·15)	-1·01 (-3·22 to 1·37)
	Under 5	18·9 (13·7 to 26·2)	1·72 (1·25 to 2·39)	-4·55 (-5·50 to -3·65)	-3·61 (-5·21 to -2·22)	0·838 (0·643 to 1·12)	0·0764 (0·0586 to 0·102)	-6·87 (-8·14 to -5·30)	-4·66 (-7·44 to -1·56)
	5-14 years	60·9 (39·9 to 92·4)	2·40 (1·58 to 3·64)	-3·72 (-4·80 to -2·80)	-2·55 (-3·97 to -1·41)	0·737 (0·559 to 0·926)	0·0291 (0·0221 to 0·0365)	-7·91 (-9·43 to -6·40)	-1·94 (-5·14 to 1·13)
	15-49 years	341 (270 to 426)	4·99 (3·94 to 6·22)	-3·42 (-3·87 to -2·92)	-2·28 (-2·95 to -1·56)	13·5 (10·3 to 17·6)	0·197 (0·150 to 0·257)	-5·36 (-6·84 to -3·68)	-2·25 (-4·30 to -0·0782)
	50-69 years	164 (124 to 203)	10·6 (8·05 to 13·2)	-3·06 (-3·58 to -2·55)	-3·16 (-3·81 to -2·60)	16·3 (12·2 to 21·9)	1·06 (0·787 to 1·42)	-5·63 (-7·24 to -3·33)	-4·17 (-6·94 to -1·24)
	70+ years	65·6 (49·8 to 83·1)	21·4 (16·2 to 27·1)	-3·25 (-3·82 to -2·69)	-2·58 (-3·42 to -1·80)	16·9 (12·3 to 21·2)	5·51 (3·99 to 6·90)	-5·61 (-7·06 to -3·88)	-2·77 (-5·35 to -0·572)
Kuwait	All Ages	835 (708 to 1000)	17·8 (15·6 to 20·5)	-1·48 (-1·94 to -1·06)	-2·37 (-3·12 to -1·76)	41·2 (34·5 to 48·8)	1·45 (1·20 to 1·72)	-2·54 (-2·96 to -2·15)	-1·58 (-3·21 to 0·00291)
	Under 5	10·3 (7·77 to 13·0)	3·90 (2·93 to 4·91)	-4·35 (-5·47 to -3·43)	-4·42 (-6·09 to -2·81)	0·291 (0·245 to 0·354)	0·110 (0·0924 to 0·134)	-4·11 (-4·81 to -3·11)	-8·40 (-10·1 to -6·45)
	5-14 years	27·9 (18·3 to 40·5)	4·81 (3·15 to 6·97)	-5·04 (-6·21 to -3·87)	-3·95 (-5·24 to -2·59)	0·333 (0·283 to 0·385)	0·0573 (0·0487 to 0·0664)	-4·68 (-5·52 to -4·04)	-5·47 (-7·03 to -3·80)
	15-49 years	551 (442 to 705)	18·1 (14·5 to 23·1)	-1·24 (-1·76 to -0·754)	-3·14 (-4·04 to -2·36)	12·3 (10·4 to 14·8)	0·404 (0·341 to 0·485)	-4·09 (-4·58 to -3·55)	-2·91 (-4·37 to -1·33)
	50-69 years	189 (144 to 239)	28·6 (21·8 to 36·2)	-2·67 (-3·21 to -2·05)	-2·33 (-3·32 to -1·39)	12·1 (9·69 to 15·0)	1·83 (1·47 to 2·27)	-4·72 (-5·31 to -4·13)	-3·75 (-5·98 to -1·70)
	70+ years	57·5 (47·1 to 71·1)	58·3 (47·7 to 72·1)	-2·73 (-3·42 to -2·13)	-2·11 (-3·22 to -1·23)	16·2 (13·6 to 19·3)	16·4 (13·8 to 19·5)	-2·60 (-3·09 to -2·07)	-1·45 (-2·90 to 0·0378)
Lebanon	All Ages	727 (644 to 845)	12·4 (11·1 to 14·2)	-2·81 (-3·23 to -2·35)	-2·83 (-3·37 to -2·28)	60·6 (49·2 to 73·1)	0·962 (0·785 to 1·16)	-4·21 (-5·87 to -2·42)	-3·50 (-5·61 to -0·790)
	Under 5	14·6 (10·9 to 18·7)	3·60 (2·70 to 4·60)	-4·48 (-5·51 to -3·41)	-4·65 (-6·50 to -2·93)	0·376 (0·270 to 0·531)	0·0927 (0·0666 to 0·131)	-7·54 (-9·81 to -4·54)	-6·69 (-9·15 to -3·76)
	5-14 years	28·4 (19·6 to 42·4)	3·26 (2·25 to 4·87)	-4·44 (-5·56 to -3·22)	-4·86 (-6·44 to -3·62)	0·282 (0·223 to 0·350)	0·0323 (0·0256 to 0·0402)	-6·64 (-8·67 to -3·88)	-6·14 (-8·94 to -3·11)

eTable 1. All-form tuberculosis incident cases and deaths, age-standardised rates of incidence and mortality per 100,000 population, and corresponding annualized rates of change by age groups for 204 countries and territories (2021).

Location	Age group	Number of cases	Rate per 100,000 cases	Annualized rate of change (1990-2010)	Annualized rate of change (2010-2021)	Number of deaths	Rate per 100,000 deaths	Annualized deaths (1990-2010)	Annualized deaths (2010-2021)
	15-49 years	310 (248 to 384)	10·3 (8·24 to 12·7)	-3·89 (-4·28 to -3·43)	-3·23 (-3·98 to -2·40)	11·4 (9·19 to 14·0)	0·380 (0·305 to 0·463)	-6·49 (-8·25 to -4·55)	-4·11 (-6·45 to -1·29)
	50-69 years	195 (152 to 243)	22·6 (17·6 to 28·1)	-2·74 (-3·36 to -1·91)	-3·41 (-4·27 to -2·63)	14·8 (12·4 to 17·7)	1·72 (1·44 to 2·05)	-5·81 (-7·65 to -4·10)	-4·72 (-6·93 to -2·32)
	70+ years	179 (144 to 225)	46·6 (37·3 to 58·5)	-3·07 (-3·64 to -2·48)	-1·95 (-3·06 to -0·923)	33·6 (26·1 to 40·8)	8·75 (6·79 to 10·6)	-5·61 (-7·18 to -3·83)	-3·33 (-5·67 to -0·300)
Libya	All Ages	1990 (1730 to 2350)	31·0 (27·0 to 35·1)	-2·04 (-2·51 to -1·59)	-0·0236 (-0·640 to 0·586)	169 (106 to 247)	3·13 (2·02 to 4·56)	-2·99 (-4·84 to -0·854)	1·38 (-1·11 to 4·10)
	Under 5	51·3 (40·6 to 65·1)	12·1 (9·61 to 15·4)	-3·49 (-4·46 to -2·64)	-0·863 (-2·26 to 0·423)	3·82 (1·98 to 5·83)	0·902 (0·469 to 1·38)	-4·71 (-7·04 to -2·34)	-0·0620 (-3·52 to 3·23)
	5-14 years	117 (82·0 to 162)	10·9 (7·68 to 15·2)	-3·23 (-4·07 to -2·35)	-0·606 (-2·06 to 0·785)	1·64 (1·04 to 2·34)	0·153 (0·0977 to 0·219)	-4·33 (-6·28 to -2·12)	-1·23 (-4·40 to 1·43)
	15-49 years	1080 (880 to 1330)	26·2 (21·3 to 32·3)	-2·34 (-2·75 to -1·84)	-0·947 (-1·68 to -0·102)	57·3 (33·8 to 87·1)	1·39 (0·820 to 2·11)	-2·65 (-4·79 to -0·342)	-0·0656 (-2·62 to 2·59)
	50-69 years	522 (404 to 665)	50·3 (38·9 to 64·1)	-2·52 (-3·05 to -1·72)	-1·40 (-2·39 to -0·787)	53·1 (31·9 to 83·7)	5·12 (3·08 to 8·07)	-4·50 (-6·75 to -1·95)	-0·609 (-3·06 to 2·26)
	70+ years	225 (176 to 277)	103 (80·1 to 126)	-2·78 (-3·23 to -2·16)	-0·633 (-1·46 to 0·281)	52·9 (34·2 to 76·2)	24·1 (15·6 to 34·8)	-4·23 (-6·38 to -2·08)	-0·146 (-3·05 to 2·88)
Morocco	All Ages	26000 (22500 to 30200)	71·5 (62·6 to 82·6)	-2·07 (-2·48 to -1·61)	-2·99 (-3·57 to -2·32)	3480 (2160 to 7680)	10·9 (6·75 to 24·2)	-2·83 (-4·58 to -1·53)	-3·75 (-6·28 to -1·17)
	Under 5	712 (538 to 896)	21·9 (16·6 to 27·6)	-4·18 (-4·98 to -3·36)	-5·86 (-7·34 to -4·15)	38·2 (25·2 to 59·9)	1·17 (0·775 to 1·84)	-7·24 (-9·06 to -5·17)	-12·9 (-16·0 to -9·57)
	5-14 years	1710 (1210 to 2430)	26·1 (18·5 to 37·1)	-3·37 (-4·17 to -2·44)	-5·27 (-6·42 to -3·54)	27·1 (20·9 to 38·7)	0·414 (0·319 to 0·591)	-4·95 (-6·99 to -2·82)	-9·89 (-12·7 to -7·43)
	15-49 years	11800 (9420 to 14500)	60·8 (48·5 to 74·5)	-2·28 (-2·83 to -1·65)	-3·92 (-4·62 to -3·16)	741 (524 to 1360)	3·81 (2·70 to 7·02)	-2·98 (-4·89 to -1·57)	-6·73 (-9·61 to -3·52)
	50-69 years	7870 (5920 to 9750)	125 (93·9 to 155)	-2·12 (-3·01 to -1·46)	-2·73 (-3·77 to -1·61)	1240 (724 to 2800)	19·6 (11·5 to 44·4)	-4·02 (-6·46 to -2·59)	-4·09 (-7·24 to -1·35)
	70+ years	3860 (3030 to 4790)	233 (183 to 290)	-2·17 (-2·69 to -1·56)	-2·95 (-3·63 to -1·97)	1440 (831 to 3450)	86·9 (50·3 to 209)	-3·18 (-5·55 to -1·49)	-4·08 (-6·56 to -1·26)
Oman	All Ages	342 (297 to 408)	10·3 (9·07 to 11·8)	-3·15 (-3·63 to -2·65)	-3·97 (-4·59 to -3·23)	17·8 (14·0 to 26·9)	0·919 (0·697 to 1·39)	-5·26 (-7·21 to -3·19)	-7·50 (-9·78 to -4·11)
	Under 5	13·2 (10·0 to 16·7)	3·11 (2·36 to 3·94)	-5·00 (-6·02 to -3·94)	-4·00 (-5·46 to -2·20)	0·338 (0·255 to 0·456)	0·0798 (0·0601 to 0·107)	-10·8 (-13·6 to -8·73)	-7·77 (-10·3 to -4·40)
	5-14 years	25·7 (17·4 to 37·4)	3·22 (2·18 to 4·68)	-4·60 (-5·70 to -3·56)	-2·88 (-4·66 to -1·21)	0·172 (0·129 to 0·235)	0·0215 (0·0161 to 0·0294)	-8·21 (-10·2 to -6·53)	-9·03 (-11·7 to -5·79)
	15-49 years	203 (166 to 254)	6·77 (5·55 to 8·49)	-3·48 (-3·93 to -2·94)	-4·60 (-5·56 to -3·52)	6·57 (4·88 to 10·6)	0·219 (0·163 to 0·355)	-5·78 (-7·92 to -3·64)	-7·10 (-10·2 to -3·24)
	50-69 years	68·4 (51·4 to 87·3)	16·4 (12·3 to 20·9)	-3·55 (-4·13 to -2·77)	-4·33 (-5·02 to -3·60)	4·60 (3·36 to 6·84)	1·10 (0·805 to 1·64)	-5·89 (-8·37 to -3·41)	-10·9 (-14·3 to -6·26)
	70+ years	32·3 (25·0 to 40·2)	46·8 (36·2 to 58·2)	-3·08 (-3·65 to -2·52)	-1·88 (-3·05 to -0·947)	6·11 (4·26 to 9·31)	8·84 (6·17 to 13·5)	-5·06 (-7·09 to -2·93)	-5·43 (-8·16 to -2·17)
Palestine	All Ages	395 (339 to 457)	10·2 (9·03 to 11·8)	-2·90 (-3·31 to -2·40)	-3·00 (-3·49 to -2·37)	27·8 (19·5 to 38·8)	1·17 (0·858 to 1·63)	-4·84 (-5·95 to -3·27)	-4·88 (-6·65 to -2·32)

eTable 1. All-form tuberculosis incident cases and deaths, age-standardised rates of incidence and mortality per 100,000 population, and corresponding annualized rates of change by age groups for 204 countries and territories (2021).

Location	Age group	Number of cases	Rate per 100,000 cases	Annualized rate of change (1990-2010)	Annualized rate of change (2010-2021)	Number of deaths	Rate per 100,000 deaths	Annualized deaths (1990-2010)	Annualized deaths (2010-2021)
	Under 5	22·0 (17·3 to 28·0)	3·60 (2·82 to 4·56)	-3·62 (-4·64 to -2·71)	-4·92 (-6·41 to -3·47)	0·928 (0·646 to 1·51)	0·151 (0·105 to 0·246)	-6·72 (-8·54 to -5·21)	-10·3 (-13·3 to -6·33)
	5-14 years	42·2 (28·0 to 62·4)	3·37 (2·23 to 4·97)	-3·34 (-4·31 to -2·47)	-4·14 (-5·44 to -2·41)	0·521 (0·375 to 0·772)	0·0416 (0·0299 to 0·0615)	-5·81 (-7·29 to -3·84)	-6·29 (-9·60 to -3·48)
	15-49 years	198 (154 to 249)	7·45 (5·81 to 9·39)	-2·88 (-3·39 to -2·42)	-3·27 (-3·84 to -2·53)	6·48 (4·46 to 10·4)	0·244 (0·168 to 0·393)	-4·68 (-6·25 to -2·74)	-4·25 (-6·26 to -1·55)
	50-69 years	92·2 (70·2 to 119)	18·1 (13·8 to 23·3)	-2·65 (-3·25 to -2·08)	-3·91 (-4·59 to -3·30)	9·08 (5·66 to 13·7)	1·78 (1·11 to 2·69)	-4·63 (-6·37 to -2·51)	-6·50 (-9·09 to -3·73)
	70+ years	41·3 (32·5 to 52·2)	39·8 (31·3 to 50·3)	-2·48 (-3·04 to -1·88)	-3·93 (-4·74 to -3·02)	10·8 (8·01 to 14·8)	10·4 (7·72 to 14·3)	-3·67 (-4·84 to -1·93)	-6·53 (-8·35 to -3·61)
Qatar	All Ages	715 (614 to 871)	32·2 (28·8 to 36·4)	-3·20 (-3·70 to -2·71)	-4·51 (-5·39 to -3·69)	20·9 (14·5 to 28·4)	1·75 (1·24 to 2·34)	-5·89 (-7·17 to -4·30)	-8·48 (-11·7 to -5·02)
	Under 5	15·3 (11·6 to 20·2)	8·30 (6·30 to 11·0)	-4·89 (-5·71 to -3·87)	-4·89 (-6·56 to -3·19)	0·268 (0·190 to 0·367)	0·145 (0·103 to 0·199)	-7·95 (-10·1 to -6·08)	-9·56 (-12·2 to -7·41)
	5-14 years	29·2 (19·9 to 43·1)	9·44 (6·42 to 13·9)	-4·53 (-5·34 to -3·67)	-4·36 (-6·09 to -2·80)	0·157 (0·126 to 0·203)	0·0508 (0·0406 to 0·0657)	-7·16 (-9·04 to -5·14)	-9·42 (-12·0 to -6·82)
	15-49 years	526 (417 to 678)	24·0 (19·0 to 30·9)	-3·44 (-3·98 to -3·01)	-4·56 (-5·54 to -3·54)	12·7 (8·84 to 17·6)	0·579 (0·403 to 0·803)	-5·65 (-7·38 to -3·83)	-7·70 (-11·4 to -3·79)
	50-69 years	119 (88·0 to 148)	43·7 (32·3 to 54·3)	-4·23 (-4·80 to -3·41)	-4·98 (-5·75 to -3·99)	4·59 (2·94 to 6·48)	1·69 (1·08 to 2·38)	-7·80 (-9·54 to -5·74)	-11·5 (-15·2 to -7·92)
	70+ years	25·1 (19·3 to 31·4)	143 (110 to 179)	-2·83 (-3·38 to -2·33)	-4·39 (-5·52 to -3·37)	3·23 (2·42 to 4·48)	18·4 (13·8 to 25·5)	-5·75 (-7·08 to -4·28)	-11·1 (-14·2 to -8·26)
Saudi Arabia	All Ages	14400 (12200 to 17500)	42·0 (37·2 to 48·5)	-2·67 (-3·30 to -1·89)	-2·81 (-3·50 to -2·22)	1140 (841 to 1660)	5·39 (4·05 to 7·59)	-4·23 (-5·78 to -2·30)	-4·86 (-7·30 to -1·74)
	Under 5	166 (124 to 212)	6·80 (5·09 to 8·70)	-6·51 (-7·58 to -5·51)	-6·46 (-8·02 to -4·57)	3·65 (2·55 to 5·32)	0·150 (0·105 to 0·219)	-12·0 (-14·1 to -9·99)	-12·8 (-15·8 to -9·04)
	5-14 years	500 (333 to 731)	9·73 (6·49 to 14·3)	-5·88 (-7·09 to -4·86)	-5·80 (-7·51 to -3·61)	3·97 (2·86 to 5·33)	0·0774 (0·0557 to 0·104)	-8·99 (-10·6 to -6·96)	-10·2 (-13·1 to -6·92)
	15-49 years	9970 (7970 to 12500)	39·4 (31·5 to 49·4)	-2·55 (-3·20 to -1·61)	-3·05 (-3·84 to -2·38)	602 (423 to 902)	2·38 (1·67 to 3·56)	-3·47 (-5·43 to -1·15)	-4·85 (-7·65 to -1·22)
	50-69 years	3060 (2280 to 3830)	70·8 (52·8 to 88·6)	-2·44 (-3·45 to -1·41)	-4·13 (-5·00 to -3·10)	324 (238 to 460)	7·51 (5·51 to 10·7)	-4·71 (-6·49 to -2·53)	-6·92 (-9·32 to -4·28)
	70+ years	677 (529 to 857)	130 (102 to 165)	-3·29 (-4·02 to -2·41)	-5·29 (-6·37 to -4·31)	208 (151 to 296)	40·0 (29·1 to 57·1)	-5·01 (-6·64 to -2·87)	-7·27 (-9·66 to -4·61)
Sudan	All Ages	17000 (14800 to 19400)	51·2 (45·6 to 58·4)	-2·71 (-3·15 to -2·27)	-3·64 (-4·21 to -3·02)	1540 (969 to 2270)	6·59 (4·10 to 9·66)	-4·95 (-6·12 to -3·51)	-5·74 (-7·70 to -3·38)
	Under 5	1070 (832 to 1340)	18·9 (14·7 to 23·8)	-3·70 (-4·60 to -2·62)	-5·31 (-6·67 to -4·03)	142 (84·3 to 204)	2·51 (1·50 to 3·62)	-7·10 (-8·60 to -5·56)	-10·6 (-13·2 to -8·04)
	5-14 years	2100 (1480 to 2970)	19·2 (13·5 to 27·1)	-3·33 (-4·12 to -2·36)	-4·70 (-6·06 to -3·53)	46·4 (28·4 to 67·0)	0·423 (0·259 to 0·612)	-5·18 (-7·20 to -2·85)	-7·31 (-9·93 to -4·75)
	15-49 years	9270 (7400 to 11200)	41·5 (33·1 to 50·2)	-2·48 (-3·07 to -1·80)	-3·94 (-4·59 to -3·27)	561 (352 to 849)	2·51 (1·57 to 3·80)	-3·53 (-5·11 to -1·65)	-5·69 (-8·05 to -3·31)
	50-69 years	3080 (2340 to 3850)	85·3 (64·7 to 106)	-2·19 (-2·93 to -1·48)	-3·34 (-4·22 to -2·54)	388 (241 to 604)	10·7 (6·67 to 16·7)	-4·56 (-6·12 to -2·73)	-5·07 (-7·14 to -2·73)

eTable 1. All-form tuberculosis incident cases and deaths, age-standardised rates of incidence and mortality per 100,000 population, and corresponding annualized rates of change by age groups for 204 countries and territories (2021).

Location	Age group	Number of cases	Rate per 100,000 cases	Annualized rate of change (1990-2010)	Annualized rate of change (2010-2021)	Number of deaths	Rate per 100,000 deaths	Annualized deaths (1990-2010)	Annualized deaths (2010-2021)
	70+ years	1470 (1180 to 1800)	172 (137 to 210)	-2·09 (-2·77 to -1·35)	-3·14 (-4·24 to -2·20)	402 (245 to 594)	46·9 (28·6 to 69·4)	-3·36 (-4·69 to -1·66)	-4·20 (-6·62 to -1·27)
Syrian Arab Republic	All Ages	1400 (1210 to 1620)	10·3 (9·00 to 12·0)	-3·87 (-4·38 to -3·44)	0·889 (0·123 to 1·71)	101 (73·6 to 138)	0·906 (0·666 to 1·22)	-6·86 (-8·62 to -5·00)	2·78 (0·139 to 5·83)
	Under 5	30·9 (23·7 to 40·3)	3·07 (2·36 to 4·01)	-6·13 (-7·07 to -5·14)	-2·38 (-3·82 to -0·504)	0·917 (0·597 to 1·38)	0·0912 (0·0594 to 0·137)	-12·8 (-15·0 to -11·0)	-4·17 (-7·69 to -0·760)
	5-14 years	125 (84·3 to 180)	4·68 (3·17 to 6·76)	-5·16 (-5·99 to -4·22)	-0·334 (-2·02 to 1·36)	1·42 (1·06 to 2·16)	0·0534 (0·0399 to 0·0813)	-8·27 (-9·90 to -6·55)	-4·72 (-7·03 to -1·11)
	15-49 years	665 (535 to 823)	9·36 (7·53 to 11·6)	-3·71 (-4·17 to -3·29)	-0·325 (-1·20 to 0·462)	21·2 (14·6 to 30·1)	0·298 (0·206 to 0·424)	-7·48 (-9·30 to -5·39)	-0·891 (-3·90 to 2·30)
	50-69 years	415 (309 to 528)	15·5 (11·6 to 19·7)	-3·70 (-4·42 to -3·03)	-1·48 (-2·20 to -0·799)	38·8 (26·4 to 56·5)	1·45 (0·985 to 2·11)	-6·49 (-8·66 to -4·14)	-2·13 (-5·36 to 1·64)
	70+ years	160 (127 to 203)	27·0 (21·5 to 34·4)	-4·01 (-4·65 to -3·44)	-2·07 (-3·07 to -1·06)	39·1 (30·2 to 52·5)	6·62 (5·11 to 8·89)	-5·88 (-7·87 to -4·02)	-2·27 (-4·83 to 0·236)
Tunisia	All Ages	2900 (2560 to 3410)	23·1 (20·3 to 27·0)	-2·94 (-3·37 to -2·48)	-3·20 (-3·70 to -2·64)	232 (158 to 403)	1·88 (1·29 to 3·23)	-3·19 (-5·20 to -1·29)	-2·60 (-5·51 to 0·781)
	Under 5	78·6 (61·3 to 102)	8·81 (6·87 to 11·4)	-4·83 (-5·67 to -4·13)	-4·92 (-6·62 to -3·74)	1·69 (1·08 to 2·76)	0·190 (0·121 to 0·310)	-10·4 (-12·8 to -8·37)	-10·5 (-13·5 to -7·19)
	5-14 years	142 (96·8 to 203)	7·57 (5·17 to 10·8)	-4·92 (-5·83 to -4·07)	-5·13 (-6·51 to -3·65)	1·23 (0·859 to 2·04)	0·0656 (0·0459 to 0·109)	-7·66 (-9·65 to -5·53)	-8·08 (-11·1 to -5·14)
	15-49 years	1220 (988 to 1560)	20·3 (16·3 to 25·7)	-3·20 (-3·64 to -2·72)	-3·58 (-4·25 to -2·76)	46·0 (31·8 to 77·2)	0·761 (0·527 to 1·28)	-3·39 (-5·75 to -0·920)	-2·83 (-5·15 to 0·365)
	50-69 years	903 (676 to 1180)	38·6 (28·9 to 50·5)	-3·86 (-4·35 to -3·24)	-3·74 (-4·49 to -3·04)	70·5 (43·7 to 129)	3·02 (1·87 to 5·51)	-5·21 (-7·90 to -2·67)	-3·73 (-7·46 to 0·312)
	70+ years	552 (451 to 698)	79·3 (64·7 to 100)	-3·55 (-4·09 to -2·96)	-4·06 (-5·00 to -3·18)	113 (77·3 to 190)	16·2 (11·1 to 27·2)	-4·04 (-6·21 to -1·94)	-4·58 (-7·58 to -0·873)
Türkiye	All Ages	10800 (9390 to 12900)	12·0 (10·5 to 14·2)	-4·49 (-5·07 to -3·89)	-2·67 (-3·53 to -1·92)	883 (708 to 1110)	1·00 (0·805 to 1·25)	-8·12 (-10·1 to -6·09)	-3·23 (-5·47 to -0·708)
	Under 5	204 (152 to 261)	3·67 (2·73 to 4·69)	-6·87 (-7·71 to -5·84)	-6·24 (-7·99 to -4·85)	7·46 (5·30 to 10·0)	0·134 (0·0954 to 0·181)	-15·0 (-17·8 to -12·8)	-10·9 (-13·4 to -8·00)
	5-14 years	510 (353 to 727)	3·93 (2·72 to 5·61)	-6·89 (-7·72 to -5·72)	-4·54 (-6·09 to -3·18)	5·53 (4·62 to 6·78)	0·0427 (0·0357 to 0·0523)	-11·4 (-13·3 to -9·57)	-7·28 (-9·58 to -4·48)
	15-49 years	4360 (3500 to 5550)	9·92 (7·96 to 12·6)	-5·05 (-5·89 to -4·29)	-3·58 (-4·32 to -2·84)	147 (119 to 188)	0·334 (0·270 to 0·428)	-9·73 (-11·4 to -7·43)	-5·30 (-7·16 to -2·51)
	50-69 years	3570 (2530 to 4650)	22·2 (15·7 to 28·9)	-4·52 (-5·55 to -3·58)	-3·19 (-4·15 to -1·98)	293 (229 to 381)	1·82 (1·42 to 2·37)	-9·29 (-11·2 to -6·66)	-4·98 (-7·57 to -1·83)
	70+ years	2140 (1650 to 2740)	42·2 (32·4 to 53·9)	-4·68 (-5·47 to -3·84)	-2·82 (-3·80 to -1·85)	430 (332 to 544)	8·46 (6·53 to 10·7)	-8·58 (-10·9 to -6·44)	-4·14 (-6·84 to -1·83)
United Arab Emirates	All Ages	477 (397 to 577)	9·36 (8·24 to 10·6)	-3·07 (-3·69 to -2·43)	-3·97 (-5·23 to -2·44)	34·3 (15·0 to 52·7)	3·13 (1·47 to 4·74)	-6·27 (-8·67 to -4·37)	-2·00 (-4·87 to 1·41)
	Under 5	11·0 (8·56 to 13·9)	2·55 (1·98 to 3·21)	-4·10 (-4·99 to -3·27)	-5·10 (-6·78 to -3·70)	0·196 (0·0903 to 0·451)	0·0452 (0·0209 to 0·104)	-8·78 (-10·7 to -6·59)	-5·90 (-8·51 to -2·76)

eTable 1. All-form tuberculosis incident cases and deaths, age-standardised rates of incidence and mortality per 100,000 population, and corresponding annualized rates of change by age groups for 204 countries and territories (2021).

Location	Age group	Number of cases	Rate per 100,000 cases	Annualized rate of change (1990-2010)	Annualized rate of change (2010-2021)	Number of deaths	Rate per 100,000 deaths	Annualized deaths (1990-2010)	Annualized deaths (2010-2021)
	5-14 years	12·9 (8·16 to 18·7)	1·42 (0·900 to 2·06)	-4·62 (-5·93 to -3·53)	-7·03 (-8·50 to -5·98)	0·135 (0·0570 to 0·235)	0·0149 (0·00630 to 0·0260)	-7·77 (-9·91 to -5·83)	-9·19 (-11·7 to -5·71)
	15-49 years	299 (241 to 383)	4·36 (3·51 to 5·58)	-3·27 (-3·95 to -2·52)	-5·18 (-6·78 to -3·73)	14·1 (5·71 to 22·9)	0·206 (0·0832 to 0·334)	-5·38 (-8·77 to -2·91)	-5·08 (-8·09 to -1·74)
	50-69 years	133 (98·5 to 170)	9·62 (7·14 to 12·3)	-3·04 (-3·86 to -2·34)	-5·02 (-6·26 to -3·74)	9·17 (3·83 to 17·3)	0·665 (0·277 to 1·26)	-6·97 (-10·1 to -4·66)	-10·8 (-14·2 to -7·32)
	70+ years	21·0 (16·6 to 26·1)	43·4 (34·3 to 54·0)	-3·00 (-3·97 to -1·96)	-1·69 (-2·75 to -0·454)	10·7 (4·84 to 14·7)	22·1 (10·0 to 30·4)	-5·04 (-6·86 to -3·16)	0·684 (-2·56 to 3·56)
Yemen	All Ages	6550 (5720 to 7490)	27·5 (24·0 to 31·2)	-3·18 (-3·68 to -2·71)	-1·60 (-2·10 to -0·987)	947 (597 to 1400)	5·88 (3·58 to 8·80)	-4·70 (-6·03 to -3·67)	-1·70 (-3·83 to 0·336)
	Under 5	452 (364 to 567)	9·62 (7·75 to 12·1)	-4·56 (-5·34 to -3·64)	-3·24 (-4·29 to -2·11)	87·2 (58·3 to 129)	1·86 (1·24 to 2·76)	-6·73 (-8·58 to -4·91)	-5·97 (-8·80 to -3·17)
	5-14 years	707 (502 to 1050)	7·78 (5·52 to 11·5)	-4·14 (-4·92 to -3·27)	-3·21 (-4·50 to -2·22)	28·6 (15·7 to 40·3)	0·315 (0·173 to 0·443)	-5·02 (-7·31 to -2·54)	-3·71 (-7·32 to -0·774)
	15-49 years	3500 (2830 to 4200)	20·9 (16·9 to 25·1)	-3·17 (-3·75 to -2·50)	-1·82 (-2·56 to -1·08)	300 (192 to 499)	1·79 (1·15 to 2·98)	-4·04 (-5·49 to -2·52)	-1·34 (-3·57 to 1·28)
	50-69 years	1320 (994 to 1670)	51·9 (39·2 to 65·9)	-2·65 (-3·39 to -2·01)	-1·31 (-1·98 to -0·500)	279 (155 to 431)	11·0 (6·10 to 17·0)	-4·64 (-6·36 to -2·67)	-1·49 (-4·48 to 1·03)
	70+ years	570 (437 to 706)	95·8 (73·4 to 119)	-2·87 (-3·56 to -2·18)	-1·22 (-2·09 to -0·328)	253 (163 to 369)	42·5 (27·4 to 62·0)	-3·59 (-5·12 to -2·37)	-0·980 (-3·31 to 1·20)
South Asia	All Ages	3640000 (3140000 to 4190000)	208 (180 to 239)	-1·58 (-1·98 to -1·18)	-1·50 (-1·76 to -1·21)	509000 (458000 to 591000)	33·4 (30·0 to 38·8)	-2·61 (-3·04 to -2·06)	-3·70 (-4·88 to -2·17)
	Under 5	62600 (50000 to 77800)	39·5 (31·5 to 49·0)	-3·20 (-3·78 to -2·65)	-4·26 (-4·86 to -3·71)	9970 (7950 to 12300)	6·29 (5·01 to 7·76)	-5·78 (-6·76 to -4·74)	-7·12 (-8·90 to -4·81)
	5-14 years	134000 (90800 to 189000)	38·3 (26·1 to 54·1)	-3·17 (-3·90 to -2·45)	-3·62 (-4·28 to -3·04)	5740 (4900 to 6740)	1·65 (1·41 to 1·93)	-4·53 (-5·23 to -3·60)	-5·88 (-7·19 to -4·56)
	15-49 years	2020000 (1700000 to 2540000)	200 (169 to 252)	-1·79 (-2·31 to -1·32)	-2·09 (-2·42 to -1·73)	166000 (149000 to 192000)	16·5 (14·8 to 19·1)	-2·63 (-3·13 to -2·08)	-5·17 (-6·38 to -3·70)
	50-69 years	1040000 (786000 to 1320000)	400 (303 to 507)	-2·05 (-2·55 to -1·49)	-2·33 (-2·66 to -2·03)	181000 (159000 to 216000)	69·8 (61·2 to 83·3)	-3·62 (-4·09 to -2·93)	-5·03 (-6·31 to -3·17)
	70+ years	389000 (304000 to 481000)	532 (415 to 657)	-2·33 (-2·86 to -1·89)	-2·09 (-2·51 to -1·68)	147000 (129000 to 171000)	200 (176 to 233)	-3·32 (-3·91 to -2·50)	-4·16 (-5·48 to -2·55)
Bangladesh	All Ages	224000 (191000 to 259000)	142 (121 to 163)	-3·42 (-3·92 to -2·91)	-3·29 (-4·14 to -2·53)	28700 (23100 to 35400)	21·2 (16·9 to 26·3)	-5·13 (-6·18 to -3·92)	-4·11 (-6·56 to -1·87)
	Under 5	4640 (3620 to 5880)	32·3 (25·2 to 41·0)	-5·43 (-6·28 to -4·58)	-5·76 (-7·57 to -4·37)	494 (323 to 676)	3·44 (2·25 to 4·71)	-9·21 (-10·9 to -7·76)	-12·0 (-15·4 to -8·62)
	5-14 years	14500 (10400 to 20800)	46·3 (33·1 to 66·4)	-3·51 (-5·15 to -2·13)	-5·22 (-7·54 to -2·67)	625 (465 to 804)	1·99 (1·48 to 2·56)	-5·93 (-7·32 to -4·14)	-8·70 (-11·7 to -5·78)
	15-49 years	116000 (93200 to 147000)	131 (106 to 168)	-3·98 (-4·51 to -3·35)	-4·19 (-5·21 to -3·27)	7410 (5780 to 9160)	8·42 (6·57 to 10·4)	-6·16 (-7·23 to -4·75)	-5·25 (-7·59 to -2·40)
	50-69 years	61500 (46400 to 80700)	261 (197 to 343)	-4·12 (-4·70 to -3·62)	-4·45 (-5·38 to -3·43)	9370 (7120 to 12700)	39·8 (30·2 to 54·0)	-5·63 (-6·95 to -3·57)	-7·04 (-10·4 to -4·22)

eTable 1. All-form tuberculosis incident cases and deaths, age-standardised rates of incidence and mortality per 100,000 population, and corresponding annualized rates of change by age groups for 204 countries and territories (2021).

Location	Age group	Number of cases	Rate per 100,000 cases	Annualized rate of change (1990-2010)	Annualized rate of change (2010-2021)	Number of deaths	Rate per 100,000 deaths	Annualized deaths (1990-2010)	Annualized deaths (2010-2021)
	70+ years	27600 (22000 to 34700)	374 (298 to 471)	-4·06 (-4·80 to -3·20)	-4·15 (-5·55 to -2·99)	10800 (8140 to 13600)	147 (110 to 184)	-4·65 (-6·62 to -2·53)	-5·84 (-8·22 to -3·56)
Bhutan	All Ages	826 (710 to 991)	114 (98·4 to 134)	-2·13 (-2·79 to -1·62)	-1·29 (-1·96 to -0·453)	108 (59·1 to 193)	17·4 (9·56 to 31·0)	-3·72 (-5·19 to -2·16)	-2·82 (-4·88 to -0·614)
	Under 5	11·8 (8·85 to 15·1)	19·3 (14·5 to 24·7)	-3·54 (-4·78 to -2·55)	-4·30 (-5·91 to -2·64)	1·60 (0·859 to 2·76)	2·62 (1·41 to 4·52)	-7·49 (-10·2 to -3·66)	-8·70 (-12·1 to -5·74)
	5-14 years	26·5 (17·1 to 39·4)	21·0 (13·5 to 31·2)	-2·79 (-4·12 to -1·40)	-3·30 (-5·74 to -0·954)	0·675 (0·376 to 1·17)	0·535 (0·298 to 0·924)	-5·24 (-8·43 to 0·295)	-6·22 (-9·70 to -2·76)
	15-49 years	455 (375 to 564)	104 (85·6 to 129)	-3·01 (-3·68 to -2·46)	-2·02 (-2·97 to -1·11)	27·4 (14·6 to 49·9)	6·25 (3·34 to 11·4)	-5·55 (-7·31 to -3·33)	-4·22 (-6·75 to -1·73)
	50-69 years	229 (173 to 303)	229 (173 to 303)	-3·31 (-3·95 to -2·71)	-1·97 (-2·95 to -0·987)	33·8 (18·8 to 67·9)	33·8 (18·8 to 67·9)	-5·88 (-7·52 to -3·71)	-4·64 (-7·23 to -1·98)
	70+ years	105 (81·3 to 131)	324 (252 to 406)	-2·94 (-3·60 to -2·15)	-2·25 (-3·37 to -1·12)	44·3 (23·4 to 73·8)	137 (72·4 to 229)	-4·23 (-5·82 to -2·52)	-3·65 (-5·98 to -1·43)
India	All Ages	3020000 (2610000 to 3500000)	219 (190 to 253)	-1·28 (-1·80 to -0·710)	-1·09 (-1·39 to -0·770)	405000 (351000 to 500000)	33·2 (28·8 to 40·9)	-2·41 (-2·91 to -1·85)	-3·70 (-4·99 to -1·86)
	Under 5	37200 (29100 to 47100)	33·4 (26·2 to 42·3)	-2·99 (-3·86 to -2·18)	-4·76 (-5·30 to -4·09)	4770 (3590 to 6590)	4·28 (3·23 to 5·92)	-5·54 (-6·80 to -4·16)	-9·14 (-11·7 to -5·99)
	5-14 years	85400 (58200 to 120000)	33·5 (22·8 to 47·1)	-3·44 (-4·31 to -2·49)	-3·49 (-4·23 to -2·84)	3140 (2460 to 3890)	1·23 (0·963 to 1·53)	-4·85 (-5·61 to -3·78)	-6·46 (-8·55 to -4·51)
	15-49 years	1680000 (1420000 to 2120000)	216 (182 to 271)	-1·47 (-2·14 to -0·862)	-1·70 (-2·08 to -1·32)	131000 (115000 to 161000)	16·8 (14·8 to 20·7)	-2·47 (-3·03 to -1·90)	-5·41 (-6·68 to -3·73)
	50-69 years	886000 (674000 to 1120000)	424 (323 to 538)	-1·79 (-2·41 to -1·03)	-2·02 (-2·38 to -1·71)	148000 (124000 to 186000)	70·7 (59·4 to 89·2)	-3·60 (-4·21 to -2·86)	-5·01 (-6·33 to -2·86)
	70+ years	331000 (257000 to 410000)	555 (432 to 688)	-2·09 (-2·81 to -1·48)	-1·71 (-2·11 to -1·21)	118000 (103000 to 144000)	198 (173 to 241)	-3·27 (-4·00 to -2·53)	-3·96 (-5·26 to -2·39)
Nepal	All Ages	43600 (37600 to 51100)	159 (138 to 184)	-2·91 (-3·44 to -2·46)	-2·79 (-3·46 to -2·12)	7010 (4730 to 10000)	30·3 (20·3 to 42·9)	-4·73 (-6·17 to -3·01)	-3·22 (-5·87 to -0·300)
	Under 5	958 (758 to 1190)	30·8 (24·4 to 38·3)	-4·36 (-5·60 to -3·37)	-5·85 (-6·82 to -4·75)	65·4 (44·2 to 92·9)	2·10 (1·42 to 2·99)	-9·77 (-11·9 to -8·05)	-11·8 (-14·3 to -8·39)
	5-14 years	1640 (1110 to 2320)	26·8 (18·1 to 37·8)	-3·69 (-5·06 to -2·37)	-4·59 (-5·77 to -3·13)	50·3 (36·1 to 66·7)	0·822 (0·590 to 1·09)	-7·47 (-8·95 to -5·74)	-7·24 (-9·89 to -4·65)
	15-49 years	20700 (17400 to 26200)	124 (105 to 157)	-3·55 (-4·15 to -2·93)	-3·93 (-4·85 to -3·15)	1660 (1170 to 2480)	9·97 (7·02 to 14·9)	-5·50 (-7·02 to -3·96)	-5·38 (-7·77 to -2·65)
	50-69 years	14200 (11300 to 18500)	348 (275 to 452)	-3·29 (-4·21 to -2·60)	-3·24 (-4·18 to -2·22)	2700 (1740 to 4060)	65·9 (42·6 to 99·2)	-5·62 (-7·51 to -3·58)	-4·42 (-7·55 to -0·702)
	70+ years	6070 (4690 to 7640)	521 (403 to 656)	-3·40 (-4·30 to -2·61)	-3·33 (-4·19 to -2·41)	2540 (1710 to 3610)	218 (147 to 310)	-4·69 (-6·91 to -2·53)	-4·10 (-7·77 to -0·725)
Pakistan	All Ages	353000 (309000 to 399000)	184 (162 to 208)	-1·86 (-2·10 to -1·47)	-3·31 (-3·79 to -2·83)	68400 (48800 to 85900)	47·8 (33·4 to 60·0)	-1·88 (-3·12 to -0·836)	-3·79 (-6·05 to -1·66)
	Under 5	19800 (16000 to 24500)	66·5 (53·8 to 82·4)	-2·44 (-2·87 to -1·94)	-3·56 (-4·37 to -2·83)	4640 (3270 to 5970)	15·6 (11·0 to 20·1)	-4·01 (-5·96 to -2·25)	-4·54 (-7·36 to -2·58)

eTable 1. All-form tuberculosis incident cases and deaths, age-standardised rates of incidence and mortality per 100,000 population, and corresponding annualized rates of change by age groups for 204 countries and territories (2021).

Location	Age group	Number of cases	Rate per 100,000 cases	Annualized rate of change (1990-2010)	Annualized rate of change (2010-2021)	Number of deaths	Rate per 100,000 deaths	Annualized deaths (1990-2010)	Annualized deaths (2010-2021)
	5-14 years	32000 (21800 to 46100)	57·5 (39·2 to 82·7)	-1·65 (-2·04 to -1·04)	-3·79 (-4·64 to -2·93)	1920 (1540 to 2320)	3·45 (2·77 to 4·17)	-1·07 (-2·56 to 0·790)	-4·51 (-6·73 to -1·87)
	15-49 years	199000 (166000 to 250000)	163 (136 to 205)	-1·86 (-2·21 to -1·43)	-3·61 (-4·12 to -3·05)	25500 (17900 to 32800)	20·9 (14·7 to 26·9)	-0·975 (-2·27 to 0·192)	-4·07 (-6·38 to -2·06)
	50-69 years	76600 (58000 to 97700)	332 (252 to 424)	-2·34 (-2·65 to -1·90)	-3·48 (-4·31 to -2·89)	21400 (14300 to 27900)	92·7 (62·2 to 121)	-1·78 (-3·07 to -0·336)	-4·22 (-6·93 to -1·57)
	70+ years	24900 (19800 to 31300)	491 (392 to 619)	-2·08 (-2·50 to -1·55)	-3·60 (-4·65 to -2·47)	15000 (10200 to 19200)	297 (201 to 380)	-1·80 (-2·86 to -0·607)	-4·02 (-6·13 to -1·62)
Southeast Asia, East Asia, and Oceania	All Ages	2060000 (1870000 to 2290000)	84·5 (76·8 to 92·2)	-1·44 (-1·68 to -1·14)	-1·23 (-1·42 to -1·04)	237000 (215000 to 281000)	9·09 (8·26 to 10·7)	-2·75 (-3·33 to -2·00)	-2·98 (-4·01 to -1·59)
	Under 5	46700 (39100 to 58000)	33·8 (28·3 to 41·9)	-3·52 (-3·88 to -3·14)	-2·04 (-2·29 to -1·77)	3520 (2790 to 4370)	2·55 (2·01 to 3·16)	-6·66 (-7·38 to -6·07)	-8·28 (-9·90 to -6·17)
	5-14 years	81100 (57400 to 110000)	26·4 (18·7 to 36·0)	-3·12 (-3·47 to -2·71)	-2·33 (-2·73 to -1·93)	1790 (1570 to 2040)	0·582 (0·512 to 0·664)	-4·04 (-4·56 to -3·48)	-8·29 (-9·26 to -7·21)
	15-49 years	875000 (739000 to 1020000)	82·1 (69·3 to 95·3)	-1·72 (-2·09 to -1·33)	-1·30 (-1·53 to -1·09)	59700 (53700 to 68700)	5·59 (5·04 to 6·44)	-2·45 (-3·01 to -1·75)	-3·47 (-4·59 to -2·36)
	50-69 years	740000 (607000 to 903000)	142 (117 to 174)	-2·38 (-2·69 to -2·08)	-2·17 (-2·44 to -1·94)	90400 (81200 to 108000)	17·4 (15·6 to 20·9)	-4·69 (-5·37 to -3·79)	-4·34 (-5·63 to -2·78)
	70+ years	318000 (267000 to 380000)	207 (173 to 247)	-2·23 (-2·53 to -1·87)	-3·13 (-3·48 to -2·88)	81500 (73200 to 97300)	52·9 (47·6 to 63·2)	-4·37 (-5·08 to -3·52)	-5·40 (-6·34 to -3·90)
East Asia	All Ages	730000 (653000 to 815000)	41·6 (37·2 to 45·5)	-2·18 (-2·62 to -1·68)	-2·64 (-2·96 to -2·32)	50400 (42700 to 62700)	2·50 (2·13 to 3·10)	-5·50 (-6·27 to -4·67)	-3·56 (-5·06 to -1·64)
	Under 5	11400 (9680 to 14100)	14·3 (12·1 to 17·7)	-5·01 (-5·53 to -4·42)	-5·56 (-6·18 to -5·04)	334 (274 to 404)	0·417 (0·342 to 0·505)	-11·3 (-12·2 to -10·5)	-12·5 (-14·3 to -10·7)
	5-14 years	16400 (11800 to 23000)	8·76 (6·29 to 12·3)	-4·55 (-5·07 to -3·99)	-6·52 (-7·17 to -5·89)	135 (115 to 165)	0·0721 (0·0617 to 0·0880)	-7·36 (-8·10 to -6·49)	-12·4 (-13·8 to -11·0)
	15-49 years	277000 (235000 to 328000)	40·3 (34·1 to 47·6)	-2·61 (-3·22 to -1·99)	-2·60 (-3·12 to -2·05)	9710 (8230 to 12200)	1·41 (1·20 to 1·77)	-5·01 (-5·87 to -4·24)	-3·95 (-5·44 to -2·19)
	50-69 years	280000 (227000 to 343000)	71·3 (57·6 to 87·2)	-3·11 (-3·69 to -2·43)	-3·70 (-4·31 to -3·34)	19400 (16300 to 24400)	4·94 (4·14 to 6·20)	-7·71 (-8·68 to -6·77)	-5·78 (-7·45 to -3·75)
	70+ years	145000 (116000 to 177000)	117 (94·1 to 143)	-2·70 (-3·22 to -1·98)	-4·30 (-4·79 to -3·90)	20700 (17300 to 26500)	16·8 (14·0 to 21·4)	-7·23 (-7·96 to -6·32)	-5·93 (-7·49 to -4·05)
China	All Ages	639000 (570000 to 711000)	37·5 (33·6 to 41·2)	-2·45 (-2·91 to -1·92)	-2·79 (-3·13 to -2·45)	38700 (31300 to 52100)	1·98 (1·62 to 2·66)	-6·23 (-7·19 to -5·24)	-3·96 (-5·97 to -1·46)
	Under 5	9800 (8240 to 12200)	12·6 (10·6 to 15·7)	-5·52 (-6·10 to -4·80)	-5·58 (-6·26 to -5·06)	264 (216 to 330)	0·340 (0·278 to 0·425)	-12·1 (-13·3 to -11·2)	-13·0 (-14·8 to -10·7)
	5-14 years	14300 (10300 to 20000)	7·88 (5·67 to 11·0)	-5·02 (-5·55 to -4·40)	-6·55 (-7·29 to -5·91)	103 (87·3 to 128)	0·0564 (0·0480 to 0·0705)	-8·15 (-8·88 to -7·15)	-13·0 (-14·5 to -11·4)
	15-49 years	240000 (204000 to 285000)	36·2 (30·7 to 43·0)	-2·86 (-3·50 to -2·20)	-2·87 (-3·39 to -2·25)	7150 (5760 to 9690)	1·08 (0·869 to 1·46)	-5·66 (-6·64 to -4·61)	-4·75 (-6·67 to -2·42)

eTable 1. All-form tuberculosis incident cases and deaths, age-standardised rates of incidence and mortality per 100,000 population, and corresponding annualized rates of change by age groups for 204 countries and territories (2021).

Location	Age group	Number of cases	Rate per 100,000 cases	Annualized rate of change (1990-2010)	Annualized rate of change (2010-2021)	Number of deaths	Rate per 100,000 deaths	Annualized deaths (1990-2010)	Annualized deaths (2010-2021)
	50-69 years	246000 (198000 to 299000)	64·6 (52·0 to 78·7)	-3·35 (-3·95 to -2·65)	-3·86 (-4·46 to -3·46)	14400 (11100 to 19500)	3·79 (2·93 to 5·11)	-8·56 (-9·71 to -7·55)	-6·32 (-8·63 to -3·64)
	70+ years	129000 (103000 to 158000)	108 (86·4 to 132)	-2·99 (-3·53 to -2·25)	-4·20 (-4·68 to -3·85)	16700 (13600 to 22600)	14·0 (11·4 to 19·0)	-7·87 (-8·78 to -6·84)	-6·06 (-7·81 to -3·57)
Democratic People's Republic of Korea	All Ages	80800 (72500 to 89900)	268 (240 to 294)	0·437 (0·177 to 0·673)	-1·17 (-1·79 to -0·486)	11000 (7710 to 15200)	34·4 (24·2 to 47·2)	-0·133 (-1·73 to 1·06)	-1·99 (-4·22 to 0·0435)
	Under 5	1490 (1240 to 1890)	98·7 (82·2 to 125)	-0·586 (-1·26 to -0·00352)	-3·99 (-5·19 to -2·24)	68·3 (43·9 to 98·9)	4·51 (2·90 to 6·54)	-3·57 (-5·59 to -1·51)	-8·98 (-12·5 to -5·69)
	5-14 years	1860 (1230 to 2650)	57·2 (37·7 to 81·2)	-0·398 (-1·29 to 0·338)	-4·49 (-6·12 to -2·64)	31·7 (22·0 to 45·1)	0·973 (0·676 to 1·38)	-2·39 (-4·24 to -0·416)	-7·22 (-9·58 to -4·35)
	15-49 years	33500 (27300 to 38900)	242 (198 to 281)	-0·0101 (-0·426 to 0·379)	-1·80 (-2·68 to -0·939)	2500 (1770 to 3730)	18·1 (12·8 to 26·9)	-0·728 (-2·94 to 0·870)	-2·56 (-5·19 to 0·668)
	50-69 years	31100 (26000 to 37400)	516 (431 to 620)	0·347 (-0·147 to 0·849)	-2·01 (-3·06 to -1·05)	4890 (3290 to 6720)	80·9 (54·6 to 111)	-0·691 (-2·33 to 0·770)	-3·79 (-5·98 to -1·53)
	70+ years	12800 (10200 to 15700)	730 (583 to 897)	-0·540 (-1·04 to -0·0647)	-2·15 (-3·64 to -0·721)	3500 (2460 to 4630)	200 (141 to 265)	-1·64 (-2·94 to -0·181)	-3·60 (-6·62 to -1·26)
Taiwan (Province of China)	All Ages	10500 (9140 to 11800)	33·7 (29·6 to 37·8)	1·38 (0·757 to 1·91)	-2·80 (-3·59 to -1·92)	688 (618 to 748)	1·65 (1·49 to 1·78)	-5·74 (-6·10 to -5·38)	-2·93 (-3·72 to -2·23)
	Under 5	133 (106 to 167)	14·9 (11·9 to 18·7)	-3·64 (-4·42 to -2·76)	4·14 (2·74 to 5·69)	1·45 (1·22 to 1·66)	0·162 (0·136 to 0·186)	-9·78 (-10·5 to -9·24)	-5·97 (-7·78 to -4·19)
	5-14 years	201 (132 to 297)	9·77 (6·43 to 14·4)	-5·85 (-7·00 to -4·55)	8·05 (5·47 to 10·5)	0·677 (0·590 to 0·782)	0·0330 (0·0287 to 0·0381)	-9·55 (-10·2 to -8·89)	-6·43 (-7·87 to -5·13)
	15-49 years	3430 (2740 to 4160)	30·2 (24·1 to 36·6)	-0·0243 (-0·881 to 0·805)	-0·786 (-2·21 to 0·622)	54·2 (48·1 to 61·0)	0·477 (0·423 to 0·536)	-7·21 (-7·72 to -6·70)	-5·21 (-6·00 to -4·47)
	50-69 years	3610 (2800 to 4590)	53·1 (41·2 to 67·5)	-1·19 (-2·11 to -0·157)	-3·54 (-5·07 to -1·97)	139 (125 to 151)	2·04 (1·84 to 2·22)	-11·0 (-11·6 to -10·6)	-4·30 (-5·25 to -3·34)
	70+ years	3140 (2400 to 3880)	124 (95·1 to 154)	0·969 (0·104 to 1·68)	-8·65 (-9·85 to -7·19)	493 (431 to 543)	19·5 (17·1 to 21·5)	-7·74 (-8·18 to -7·14)	-5·78 (-6·66 to -4·97)
Oceania	All Ages	15000 (13400 to 16700)	132 (121 to 143)	0·0291 (-0·180 to 0·263)	-0·885 (-1·24 to -0·511)	3390 (2840 to 4000)	39·5 (32·9 to 46·6)	-0·612 (-1·99 to 0·562)	-1·89 (-3·48 to -0·573)
	Under 5	836 (666 to 1020)	43·2 (34·4 to 52·9)	0·273 (-0·319 to 0·801)	-2·20 (-3·05 to -1·41)	218 (115 to 324)	11·3 (5·95 to 16·7)	0·378 (-1·31 to 1·71)	-5·36 (-7·80 to -3·17)
	5-14 years	1430 (1000 to 1990)	45·3 (31·8 to 63·2)	0·0962 (-0·478 to 0·680)	-1·79 (-2·76 to -0·786)	93·2 (71·8 to 128)	2·96 (2·28 to 4·06)	-0·485 (-2·06 to 1·15)	-4·19 (-6·25 to -1·84)
	15-49 years	8450 (7110 to 9660)	119 (101 to 137)	0·0644 (-0·235 to 0·415)	-0·968 (-1·48 to -0·539)	1280 (1010 to 1560)	18·1 (14·3 to 22·1)	-0·213 (-1·73 to 1·27)	-2·30 (-4·20 to -0·445)
	50-69 years	3240 (2710 to 3860)	217 (181 to 258)	-0·805 (-1·14 to -0·408)	-0·785 (-1·27 to -0·291)	1140 (914 to 1460)	76·1 (61·1 to 97·7)	-1·87 (-3·52 to -0·493)	-1·89 (-4·00 to 0·0147)
	70+ years	1050 (887 to 1160)	379 (321 to 421)	-0·724 (-1·17 to -0·205)	-1·20 (-1·85 to -0·479)	661 (531 to 839)	239 (192 to 304)	-1·03 (-2·29 to 0·189)	-2·14 (-4·34 to -0·152)
American Samoa	All Ages	7·46 (6·55 to 8·26)	15·2 (13·5 to 16·8)	-1·09 (-1·35 to -0·819)	0·140 (-0·488 to 0·638)	1·12 (0·966 to 1·39)	2·42 (2·09 to 2·98)	-1·74 (-2·57 to -0·456)	0·965 (-0·441 to 2·48)

eTable 1. All-form tuberculosis incident cases and deaths, age-standardised rates of incidence and mortality per 100,000 population, and corresponding annualized rates of change by age groups for 204 countries and territories (2021).

Location	Age group	Number of cases	Rate per 100,000 cases	Annualized rate of change (1990-2010)	Annualized rate of change (2010-2021)	Number of deaths	Rate per 100,000 deaths	Annualized deaths (1990-2010)	Annualized deaths (2010-2021)
	Under 5	0·160 (0·124 to 0·209)	4·30 (3·35 to 5·64)	-0·918 (-1·63 to -0·407)	-2·58 (-4·10 to -1·49)	0·00591 (0·00463 to 0·00769)	0·159 (0·125 to 0·207)	-4·14 (-5·52 to -2·69)	-4·11 (-6·80 to -1·69)
	5-14 years	0·335 (0·228 to 0·495)	3·20 (2·17 to 4·73)	-1·67 (-2·45 to -1·09)	-6·06 (-7·62 to -4·40)	0·0111 (0·00816 to 0·0142)	0·106 (0·0779 to 0·136)	-4·01 (-5·63 to -2·14)	-2·16 (-4·72 to 0·105)
	15-49 years	3·77 (3·11 to 4·38)	15·7 (13·0 to 18·3)	-1·22 (-1·63 to -0·854)	0·0609 (-0·581 to 0·706)	0·299 (0·226 to 0·392)	1·25 (0·941 to 1·63)	-1·91 (-3·30 to -0·0795)	-0·696 (-3·25 to 1·88)
	50-69 years	2·36 (1·90 to 2·92)	25·1 (20·3 to 31·2)	-2·26 (-2·70 to -1·81)	-1·26 (-2·01 to -0·697)	0·464 (0·391 to 0·579)	4·95 (4·16 to 6·17)	-3·58 (-4·59 to -2·06)	-1·45 (-3·07 to 0·0466)
	70+ years	0·843 (0·665 to 1·03)	38·1 (30·0 to 46·5)	-2·22 (-2·75 to -1·76)	-1·44 (-2·12 to -0·752)	0·344 (0·278 to 0·430)	15·5 (12·6 to 19·4)	-3·37 (-4·64 to -2·10)	-1·92 (-3·76 to -0·151)
Cook Islands	All Ages	2·60 (2·28 to 2·94)	12·6 (11·1 to 14·5)	-1·45 (-1·79 to -1·12)	-0·618 (-1·19 to -0·150)	0·277 (0·233 to 0·351)	1·14 (0·951 to 1·43)	-2·84 (-4·21 to -1·31)	-1·56 (-3·57 to 0·315)
	Under 5	0·0407 (0·0311 to 0·0526)	3·62 (2·77 to 4·68)	-2·51 (-3·32 to -1·62)	-2·18 (-3·07 to -1·26)	0·00103 (0·000686 to 0·00147)	0·0912 (0·0610 to 0·130)	-6·91 (-8·34 to -5·11)	-3·30 (-6·30 to -0·410)
	5-14 years	0·0833 (0·0549 to 0·120)	3·13 (2·06 to 4·50)	-3·35 (-4·24 to -2·57)	-3·42 (-4·53 to -2·10)	0·000824 (0·000610 to 0·00109)	0·0309 (0·0229 to 0·0408)	-8·52 (-10·3 to -6·82)	-5·22 (-7·89 to -2·69)
	15-49 years	1·02 (0·830 to 1·26)	12·7 (10·3 to 15·7)	-1·82 (-2·28 to -1·36)	-1·24 (-1·82 to -0·669)	0·0429 (0·0330 to 0·0564)	0·532 (0·409 to 0·700)	-3·90 (-5·60 to -1·99)	-2·72 (-5·37 to -0·167)
	50-69 years	0·944 (0·699 to 1·18)	21·8 (16·1 to 27·2)	-2·64 (-3·13 to -2·19)	-1·99 (-2·66 to -1·16)	0·104 (0·0833 to 0·139)	2·41 (1·92 to 3·20)	-4·95 (-6·48 to -3·18)	-4·32 (-6·57 to -1·92)
	70+ years	0·515 (0·408 to 0·644)	33·1 (26·2 to 41·4)	-2·64 (-3·05 to -2·29)	-1·75 (-2·60 to -0·928)	0·127 (0·105 to 0·156)	8·18 (6·72 to 9·99)	-5·08 (-6·42 to -3·50)	-4·22 (-6·28 to -2·14)
Fiji	All Ages	313 (279 to 344)	35·7 (32·1 to 39·2)	-1·65 (-1·93 to -1·34)	-1·21 (-1·61 to -0·743)	47·3 (37·0 to 61·4)	6·15 (4·87 to 7·90)	-0·907 (-1·84 to 0·141)	-1·66 (-3·79 to 0·695)
	Under 5	11·0 (8·66 to 14·1)	12·1 (9·52 to 15·5)	-1·22 (-1·93 to -0·545)	-2·44 (-3·55 to -1·46)	0·860 (0·627 to 1·14)	0·944 (0·688 to 1·26)	0·157 (-1·28 to 2·06)	-4·65 (-7·38 to -2·21)
	5-14 years	32·6 (22·7 to 44·6)	17·9 (12·5 to 24·6)	-2·10 (-2·75 to -1·26)	-1·08 (-2·45 to 0·169)	1·08 (0·833 to 1·42)	0·595 (0·459 to 0·785)	-2·45 (-4·31 to -0·626)	-1·50 (-4·67 to 2·23)
	15-49 years	145 (119 to 164)	31·1 (25·3 to 35·1)	-2·18 (-2·46 to -1·79)	-1·52 (-2·06 to -0·927)	11·9 (9·23 to 15·8)	2·55 (1·97 to 3·37)	-2·32 (-3·41 to -1·36)	-2·54 (-4·83 to -0·0569)
	50-69 years	93·1 (76·8 to 111)	61·3 (50·6 to 73·0)	-2·42 (-2·77 to -1·82)	-1·58 (-2·37 to -1·03)	20·0 (14·8 to 28·2)	13·2 (9·75 to 18·6)	-2·24 (-3·52 to -0·805)	-2·92 (-5·84 to 0·102)
	70+ years	30·4 (23·8 to 36·1)	94·6 (74·1 to 112)	-2·47 (-2·97 to -1·95)	-1·85 (-2·75 to -0·938)	13·4 (10·5 to 17·6)	41·7 (32·6 to 54·7)	-2·14 (-3·56 to -0·889)	-2·67 (-5·25 to -0·0297)
Guam	All Ages	67·0 (59·0 to 75·6)	37·8 (33·6 to 42·5)	-0·394 (-0·720 to -0·0676)	1·07 (0·602 to 1·60)	9·20 (7·71 to 10·7)	4·76 (3·98 to 5·49)	-0·600 (-1·33 to 0·111)	1·56 (0·281 to 2·85)
	Under 5	1·31 (1·05 to 1·66)	10·2 (8·23 to 13·0)	-0·702 (-1·34 to -0·135)	-1·31 (-2·33 to -0·0418)	0·0679 (0·0534 to 0·0859)	0·531 (0·418 to 0·672)	-1·22 (-2·15 to -0·273)	-3·12 (-4·61 to -1·52)
	5-14 years	3·63 (2·40 to 5·18)	15·3 (10·1 to 21·8)	-1·52 (-2·33 to -0·746)	-0·287 (-1·56 to 1·02)	0·105 (0·0831 to 0·129)	0·441 (0·349 to 0·541)	-1·91 (-3·25 to -0·787)	0·106 (-1·97 to 2·27)
	15-49 years	28·5 (23·3 to 34·3)	38·2 (31·2 to 46·0)	-0·423 (-0·799 to -0·00234)	0·166 (-0·446 to 0·900)	2·49 (2·07 to 2·92)	3·33 (2·77 to 3·91)	0·0243 (-1·12 to 0·737)	0·743 (-0·708 to 2·10)

eTable 1. All-form tuberculosis incident cases and deaths, age-standardised rates of incidence and mortality per 100,000 population, and corresponding annualized rates of change by age groups for 204 countries and territories (2021).

Location	Age group	Number of cases	Rate per 100,000 cases	Annualized rate of change (1990-2010)	Annualized rate of change (2010-2021)	Number of deaths	Rate per 100,000 deaths	Annualized deaths (1990-2010)	Annualized deaths (2010-2021)
	50-69 years	22·9 (17·9 to 28·6)	64·1 (50·1 to 80·1)	-1·63 (-2·09 to -1·18)	0·475 (-0·211 to 1·36)	3·82 (3·13 to 4·72)	10·7 (8·78 to 13·2)	-3·09 (-4·20 to -1·91)	-0·0146 (-1·73 to 1·88)
	70+ years	10·6 (8·69 to 13·0)	86·9 (71·0 to 107)	-1·85 (-2·46 to -1·14)	-0·0603 (-0·911 to 0·816)	2·71 (2·22 to 3·27)	22·2 (18·1 to 26·7)	-4·18 (-5·43 to -3·19)	-2·32 (-4·20 to -0·475)
Kiribati	All Ages	355 (320 to 393)	345 (315 to 375)	-0·358 (-0·579 to -0·150)	-0·707 (-1·10 to -0·351)	85·9 (63·1 to 121)	108 (80·9 to 147)	-1·34 (-2·72 to 0·379)	-1·55 (-3·43 to 0·364)
	Under 5	14·1 (11·1 to 18·1)	98·5 (77·4 to 126)	-1·57 (-2·17 to -0·956)	-2·23 (-3·44 to -1·13)	2·20 (1·46 to 3·44)	15·4 (10·2 to 24·0)	-3·59 (-5·41 to -2·20)	-6·24 (-9·20 to -2·40)
	5-14 years	40·6 (27·1 to 56·5)	147 (97·9 to 204)	-0·625 (-1·12 to -0·0801)	-1·60 (-2·51 to -0·635)	2·09 (1·57 to 2·74)	7·52 (5·67 to 9·89)	-2·91 (-4·49 to -1·35)	-3·27 (-6·09 to -0·725)
	15-49 years	192 (160 to 219)	309 (258 to 353)	-0·190 (-0·552 to 0·156)	-0·733 (-1·27 to -0·238)	31·1 (21·1 to 44·6)	50·2 (34·0 to 71·8)	-0·995 (-2·63 to 0·878)	-2·10 (-4·43 to 0·132)
	50-69 years	84·8 (70·9 to 96·9)	581 (486 to 665)	-0·823 (-1·11 to -0·478)	-0·893 (-1·52 to -0·231)	33·7 (24·3 to 49·0)	231 (167 to 336)	-1·77 (-3·45 to 0·00580)	-1·98 (-4·15 to 0·118)
	70+ years	24·2 (21·1 to 26·8)	973 (850 to 1080)	-0·785 (-1·16 to -0·388)	-1·50 (-2·31 to -0·750)	16·8 (13·4 to 21·3)	675 (540 to 855)	-1·06 (-2·41 to 0·469)	-2·04 (-4·15 to -0·198)
Marshall Islands	All Ages	192 (168 to 216)	390 (347 to 430)	0·315 (0·0455 to 0·618)	-0·834 (-1·25 to -0·341)	33·4 (20·2 to 45·0)	86·2 (53·5 to 114)	-0·330 (-1·26 to 0·768)	-1·08 (-2·76 to 0·615)
	Under 5	5·90 (4·57 to 7·55)	104 (80·5 to 133)	0·122 (-0·526 to 0·787)	-2·64 (-3·97 to -1·47)	0·481 (0·313 to 0·668)	8·48 (5·51 to 11·8)	-0·00293 (-1·98 to 1·65)	-5·25 (-7·63 to -2·66)
	5-14 years	17·0 (11·5 to 23·5)	144 (97·3 to 200)	0·0932 (-0·678 to 0·768)	-2·23 (-3·43 to -1·05)	0·547 (0·341 to 0·717)	4·64 (2·90 to 6·08)	-0·106 (-2·01 to 1·61)	-3·86 (-6·47 to -1·12)
	15-49 years	109 (89·9 to 127)	358 (295 to 417)	-0·150 (-0·469 to 0·191)	-1·50 (-1·97 to -0·923)	13·1 (8·13 to 18·2)	42·8 (26·7 to 59·8)	-0·429 (-1·38 to 0·688)	-2·55 (-4·32 to -0·821)
	50-69 years	48·7 (40·2 to 57·6)	679 (560 to 803)	-0·964 (-1·36 to -0·638)	-1·27 (-1·93 to -0·620)	13·4 (7·51 to 18·8)	186 (105 to 263)	-2·23 (-3·65 to -1·03)	-2·68 (-4·40 to -0·415)
	70+ years	11·6 (9·62 to 13·4)	1010 (838 to 1170)	-0·727 (-1·19 to -0·241)	-2·06 (-2·93 to -1·13)	5·91 (3·62 to 8·29)	515 (316 to 722)	-1·61 (-2·73 to -0·196)	-3·07 (-5·53 to -1·47)
Micronesia (Federated States of)	All Ages	80·1 (71·7 to 88·2)	85·0 (77·1 to 93·6)	-1·40 (-1·70 to -1·08)	-0·268 (-0·774 to 0·224)	12·8 (9·10 to 17·4)	16·5 (12·0 to 22·3)	-2·68 (-4·02 to -1·21)	-0·385 (-2·00 to 1·55)
	Under 5	2·16 (1·71 to 2·79)	22·8 (18·0 to 29·5)	-2·06 (-2·85 to -1·33)	-1·90 (-3·06 to -0·530)	0·160 (0·111 to 0·213)	1·69 (1·17 to 2·25)	-4·50 (-6·20 to -3·32)	-3·21 (-5·98 to -0·605)
	5-14 years	6·26 (4·17 to 8·43)	29·6 (19·7 to 39·9)	-2·04 (-2·85 to -1·30)	-2·08 (-3·38 to -0·576)	0·165 (0·125 to 0·220)	0·781 (0·593 to 1·04)	-4·29 (-5·75 to -2·72)	-3·57 (-5·70 to -1·23)
	15-49 years	42·5 (34·8 to 48·7)	79·0 (64·7 to 90·5)	-1·54 (-1·87 to -1·15)	-0·904 (-1·46 to -0·294)	4·33 (3·07 to 5·82)	8·04 (5·70 to 10·8)	-2·52 (-4·09 to -1·11)	-1·71 (-3·69 to 0·542)
	50-69 years	23·5 (19·5 to 28·9)	151 (125 to 185)	-2·52 (-2·97 to -2·10)	-0·558 (-1·26 to 0·0792)	5·58 (3·68 to 8·01)	35·8 (23·6 to 51·3)	-4·28 (-5·82 to -2·21)	-1·26 (-3·30 to 1·29)
	70+ years	5·68 (4·67 to 6·70)	225 (185 to 265)	-1·99 (-2·44 to -1·55)	-1·63 (-2·20 to -1·06)	2·53 (1·88 to 3·41)	100 (74·3 to 135)	-3·16 (-4·86 to -1·50)	-2·66 (-5·26 to -0·0745)
Nauru	All Ages	15·5 (13·7 to 17·3)	169 (152 to 186)	0·390 (0·142 to 0·646)	-2·43 (-2·88 to -1·87)	2·61 (1·83 to 3·59)	38·6 (27·0 to 55·4)	0·662 (-0·660 to 1·96)	-5·46 (-7·38 to -3·38)

eTable 1. All-form tuberculosis incident cases and deaths, age-standardised rates of incidence and mortality per 100,000 population, and corresponding annualized rates of change by age groups for 204 countries and territories (2021).

Location	Age group	Number of cases	Rate per 100,000 cases	Annualized rate of change (1990-2010)	Annualized rate of change (2010-2021)	Number of deaths	Rate per 100,000 deaths	Annualized deaths (1990-2010)	Annualized deaths (2010-2021)
	Under 5	0·667 (0·522 to 0·842)	47·7 (37·3 to 60·2)	0·780 (-0·0116 to 1·43)	-3·61 (-4·86 to -2·41)	0·0670 (0·0429 to 0·0964)	4·79 (3·07 to 6·90)	1·96 (0·136 to 3·83)	-8·73 (-11·2 to -5·74)
	5-14 years	1·62 (1·13 to 2·27)	62·6 (43·8 to 88·1)	0·662 (0·0636 to 1·16)	-3·27 (-4·57 to -1·89)	0·0563 (0·0391 to 0·0736)	2·18 (1·51 to 2·85)	0·962 (-0·871 to 2·38)	-7·19 (-9·57 to -4·22)
	15-49 years	9·07 (7·39 to 10·4)	158 (129 to 181)	0·224 (-0·167 to 0·528)	-2·34 (-3·04 to -1·61)	1·05 (0·747 to 1·54)	18·2 (13·0 to 26·8)	0·510 (-0·735 to 1·95)	-5·70 (-7·87 to -3·37)
	50-69 years	3·19 (2·60 to 3·84)	296 (241 to 357)	-0·224 (-0·621 to 0·362)	-2·65 (-3·40 to -1·66)	0·916 (0·552 to 1·26)	85·2 (51·3 to 117)	0·0172 (-2·12 to 1·65)	-5·99 (-8·64 to -3·44)
	70+ years	0·927 (0·765 to 1·10)	410 (338 to 484)	-0·207 (-0·736 to 0·461)	-3·63 (-4·62 to -2·53)	0·525 (0·357 to 0·935)	232 (158 to 413)	0·00792 (-1·69 to 1·84)	-5·62 (-8·50 to -2·03)
Niue	All Ages	0·594 (0·528 to 0·653)	31·8 (28·6 to 35·1)	-0·796 (-1·12 to -0·546)	-1·16 (-1·54 to -0·747)	0·108 (0·0806 to 0·138)	5·27 (3·94 to 6·76)	-1·81 (-3·20 to -0·702)	-2·29 (-3·69 to -0·258)
	Under 5	0·0141 (0·0110 to 0·0181)	12·0 (9·37 to 15·4)	-0·450 (-1·08 to 0·231)	-1·40 (-2·44 to -0·181)	0·00132 (0·000865 to 0·00178)	1·12 (0·737 to 1·51)	-1·09 (-2·59 to 0·418)	-2·01 (-4·77 to 0·942)
	5-14 years	0·0365 (0·0247 to 0·0497)	13·5 (9·17 to 18·4)	-0·970 (-1·65 to -0·0543)	-1·75 (-2·97 to -0·316)	0·00108 (0·000780 to 0·00144)	0·400 (0·289 to 0·533)	-2·47 (-4·45 to -0·579)	-3·01 (-5·34 to -0·523)
	15-49 years	0·220 (0·180 to 0·261)	28·2 (23·1 to 33·4)	-1·37 (-1·70 to -1·08)	-1·31 (-1·81 to -0·796)	0·0175 (0·0118 to 0·0243)	2·24 (1·50 to 3·10)	-2·45 (-4·68 to -0·616)	-2·29 (-4·62 to 0·670)
	50-69 years	0·220 (0·179 to 0·270)	57·4 (46·6 to 70·5)	-1·63 (-1·98 to -1·23)	-1·20 (-2·03 to -0·502)	0·0438 (0·0300 to 0·0602)	11·4 (7·83 to 15·7)	-3·36 (-5·40 to -1·86)	-1·85 (-4·43 to 0·596)
	70+ years	0·102 (0·0816 to 0·122)	86·1 (68·7 to 103)	-1·66 (-2·11 to -1·21)	-1·48 (-2·26 to -0·532)	0·0440 (0·0341 to 0·0548)	37·1 (28·8 to 46·2)	-2·91 (-4·13 to -1·79)	-2·47 (-4·55 to -0·248)
Northern Mariana Islands	All Ages	46·3 (39·8 to 51·3)	89·7 (79·3 to 99·4)	-1·18 (-1·55 to -0·824)	0·272 (-0·302 to 0·787)	4·64 (3·55 to 5·88)	9·37 (7·25 to 11·8)	-2·12 (-3·31 to -0·485)	0·788 (-1·24 to 2·84)
	Under 5	0·821 (0·639 to 1·07)	25·6 (19·9 to 33·4)	-0·796 (-1·49 to -0·280)	-1·16 (-2·34 to 0·143)	0·0191 (0·0143 to 0·0253)	0·595 (0·445 to 0·788)	-3·19 (-4·72 to -1·61)	-3·47 (-5·46 to -1·55)
	5-14 years	2·23 (1·54 to 3·09)	27·7 (19·2 to 38·4)	-1·56 (-2·22 to -0·924)	-1·21 (-2·41 to -0·219)	0·0244 (0·0156 to 0·0332)	0·303 (0·194 to 0·412)	-3·40 (-4·99 to -1·94)	-3·56 (-6·91 to -0·250)
	15-49 years	19·6 (16·5 to 23·3)	82·6 (69·7 to 98·3)	-1·64 (-2·25 to -1·15)	-0·961 (-1·66 to -0·202)	0·888 (0·688 to 1·20)	3·74 (2·90 to 5·06)	-3·28 (-5·01 to -1·31)	-3·23 (-5·44 to -0·334)
	50-69 years	18·6 (13·9 to 23·1)	163 (122 to 202)	-2·21 (-2·66 to -1·83)	-0·403 (-1·38 to 0·484)	2·28 (1·67 to 2·94)	20·0 (14·7 to 25·8)	-4·55 (-6·09 to -2·70)	-1·30 (-3·61 to 1·23)
	70+ years	5·05 (3·91 to 6·38)	238 (185 to 301)	-1·87 (-2·35 to -1·30)	-1·52 (-2·28 to -0·650)	1·43 (0·990 to 2·24)	67·3 (46·7 to 106)	-3·87 (-5·44 to -2·32)	-2·70 (-5·13 to 1·16)
Palau	All Ages	8·35 (7·41 to 9·50)	42·0 (37·2 to 47·7)	-0·199 (-0·555 to 0·0535)	0·261 (-0·356 to 0·863)	1·54 (1·21 to 1·98)	7·50 (6·10 to 9·30)	-0·951 (-2·27 to 0·780)	0·184 (-1·85 to 2·58)
	Under 5	0·109 (0·0872 to 0·140)	11·5 (9·21 to 14·8)	-0·837 (-1·53 to -0·0859)	-1·37 (-2·28 to -0·263)	0·00942 (0·00737 to 0·0120)	0·995 (0·778 to 1·27)	-2·87 (-4·60 to -1·18)	-2·35 (-4·86 to 0·517)
	5-14 years	0·272 (0·184 to 0·397)	11·7 (7·97 to 17·2)	-1·33 (-1·95 to -0·671)	-1·75 (-2·96 to -0·434)	0·00635 (0·00486 to 0·00792)	0·274 (0·210 to 0·342)	-3·93 (-5·35 to -1·90)	-2·13 (-4·77 to 0·373)
	15-49 years	3·97 (3·30 to 4·79)	43·8 (36·4 to 52·9)	-0·273 (-0·711 to 0·0798)	-0·121 (-1·01 to 0·544)	0·445 (0·359 to 0·568)	4·91 (3·96 to 6·26)	-0·536 (-2·45 to 1·65)	-0·849 (-3·42 to 1·70)

eTable 1. All-form tuberculosis incident cases and deaths, age-standardised rates of incidence and mortality per 100,000 population, and corresponding annualized rates of change by age groups for 204 countries and territories (2021).

Location	Age group	Number of cases	Rate per 100,000 cases	Annualized rate of change (1990-2010)	Annualized rate of change (2010-2021)	Number of deaths	Rate per 100,000 deaths	Annualized deaths (1990-2010)	Annualized deaths (2010-2021)
	50-69 years	3·11 (2·50 to 3·88)	64·1 (51·4 to 79·8)	-1·71 (-2·13 to -1·29)	-0·862 (-1·56 to -0·0291)	0·675 (0·489 to 0·990)	13·9 (10·1 to 20·4)	-3·08 (-4·65 to -0·902)	-2·05 (-4·34 to 0·822)
	70+ years	0·888 (0·716 to 1·10)	97·4 (78·5 to 121)	-1·01 (-1·59 to -0·515)	-1·69 (-2·48 to -0·769)	0·401 (0·311 to 0·525)	44·0 (34·1 to 57·6)	-2·20 (-3·94 to -0·653)	-2·77 (-4·98 to -0·284)
Papua New Guinea	All Ages	12500 (11100 to 14000)	155 (142 to 168)	-0·0752 (-0·332 to 0·198)	-1·17 (-1·58 to -0·744)	2900 (2380 to 3470)	50·2 (40·9 to 60·3)	-0·890 (-2·53 to 0·364)	-2·30 (-3·98 to -0·898)
	Under 5	724 (572 to 891)	47·6 (37·6 to 58·6)	0·188 (-0·489 to 0·786)	-2·55 (-3·48 to -1·68)	201 (102 to 300)	13·2 (6·71 to 19·7)	0·127 (-1·67 to 1·65)	-5·86 (-8·38 to -3·59)
	5-14 years	1180 (833 to 1660)	49·3 (34·8 to 69·2)	0·129 (-0·563 to 0·792)	-2·06 (-3·19 to -0·932)	81·9 (62·0 to 115)	3·42 (2·59 to 4·78)	-0·744 (-2·60 to 1·10)	-4·60 (-6·80 to -2·09)
	15-49 years	7120 (5990 to 8160)	133 (112 to 153)	-0·0555 (-0·395 to 0·337)	-1·27 (-1·87 to -0·781)	1100 (847 to 1370)	20·7 (15·9 to 25·7)	-0·494 (-2·14 to 1·30)	-2·73 (-4·81 to -0·752)
	50-69 years	2620 (2200 to 3120)	254 (214 to 303)	-0·662 (-1·06 to -0·199)	-1·12 (-1·68 to -0·527)	958 (749 to 1270)	92·9 (72·7 to 123)	-1·79 (-3·73 to -0·178)	-2·35 (-4·72 to -0·233)
	70+ years	847 (715 to 940)	486 (411 to 540)	-0·535 (-1·05 to 0·0904)	-1·35 (-2·08 to -0·451)	554 (430 to 722)	318 (247 to 415)	-0·846 (-2·36 to 0·639)	-2·32 (-4·84 to -0·188)
Samoa	All Ages	87·5 (77·7 to 97·3)	48·2 (43·2 to 53·2)	-0·831 (-1·08 to -0·559)	-0·664 (-1·11 to -0·168)	14·2 (10·7 to 18·8)	9·44 (7·15 to 12·5)	-1·96 (-3·19 to -0·805)	-0·866 (-3·11 to 0·954)
	Under 5	4·00 (3·18 to 4·97)	13·7 (10·9 to 17·0)	-1·27 (-1·85 to -0·642)	-1·32 (-2·50 to -0·331)	0·300 (0·198 to 0·449)	1·03 (0·678 to 1·53)	-3·94 (-6·10 to -1·97)	-2·70 (-6·29 to 0·641)
	5-14 years	7·69 (5·59 to 11·0)	15·2 (11·0 to 21·7)	-1·64 (-2·30 to -0·970)	-2·03 (-3·33 to -0·760)	0·215 (0·158 to 0·292)	0·424 (0·312 to 0·576)	-4·32 (-6·26 to -2·66)	-2·35 (-4·89 to 0·114)
	15-49 years	42·5 (34·8 to 50·1)	42·4 (34·7 to 50·0)	-0·940 (-1·34 to -0·571)	-0·638 (-1·22 to -0·0307)	3·82 (2·65 to 4·92)	3·80 (2·64 to 4·91)	-2·01 (-3·79 to -0·214)	-0·317 (-3·28 to 2·39)
	50-69 years	24·0 (19·9 to 29·0)	90·3 (74·8 to 109)	-1·32 (-1·73 to -0·969)	-0·757 (-1·53 to -0·158)	5·39 (3·90 to 7·64)	20·3 (14·7 to 28·8)	-3·15 (-4·55 to -1·51)	-1·38 (-4·49 to 1·51)
	70+ years	9·27 (7·54 to 11·1)	136 (110 to 163)	-1·39 (-1·95 to -0·786)	-1·18 (-2·13 to -0·304)	4·45 (3·23 to 6·10)	65·1 (47·2 to 89·2)	-2·80 (-4·57 to -1·47)	-1·95 (-4·44 to 0·659)
Solomon Islands	All Ages	433 (387 to 483)	80·9 (73·3 to 89·2)	-0·578 (-0·833 to -0·296)	-0·471 (-0·941 to -0·0469)	84·9 (66·5 to 109)	21·4 (17·1 to 27·4)	-1·03 (-2·27 to 0·582)	-1·15 (-2·84 to 1·06)
	Under 5	23·6 (18·3 to 29·2)	24·7 (19·2 to 30·7)	-0·616 (-1·22 to -0·120)	-1·53 (-2·85 to -0·494)	2·41 (1·74 to 3·25)	2·53 (1·83 to 3·41)	-2·50 (-4·20 to -1·09)	-4·05 (-6·65 to -1·23)
	5-14 years	44·8 (29·8 to 62·8)	27·2 (18·1 to 38·1)	-1·25 (-1·89 to -0·525)	-0·625 (-1·86 to 0·444)	1·97 (1·37 to 3·01)	1·20 (0·834 to 1·83)	-1·70 (-3·37 to 0·780)	-2·90 (-5·74 to 0·692)
	15-49 years	241 (199 to 277)	70·1 (57·8 to 80·4)	-0·785 (-1·12 to -0·477)	-0·607 (-1·31 to -0·0194)	35·7 (26·2 to 44·4)	10·4 (7·61 to 12·9)	-0·627 (-2·57 to 1·94)	-1·33 (-3·44 to 0·795)
	50-69 years	91·0 (72·9 to 109)	138 (111 to 166)	-0·781 (-1·13 to -0·458)	-1·23 (-1·90 to -0·592)	27·0 (20·5 to 37·6)	41·0 (31·1 to 57·1)	-2·26 (-4·18 to -0·663)	-1·54 (-3·98 to 1·07)
	70+ years	32·2 (27·1 to 36·2)	243 (204 to 273)	-0·920 (-1·36 to -0·489)	-0·889 (-1·72 to -0·0162)	17·8 (14·1 to 22·0)	134 (107 to 166)	-1·69 (-3·26 to -0·373)	-2·04 (-3·98 to 0·146)
Tokelau	All Ages	0·488 (0·442 to 0·540)	34·6 (31·2 to 38·3)	-1·17 (-1·53 to -0·914)	-2·25 (-2·65 to -1·81)	0·0852 (0·0653 to 0·117)	5·81 (4·45 to 8·01)	-2·69 (-3·75 to -1·24)	-4·23 (-6·93 to -1·62)

eTable 1. All-form tuberculosis incident cases and deaths, age-standardised rates of incidence and mortality per 100,000 population, and corresponding annualized rates of change by age groups for 204 countries and territories (2021).

Location	Age group	Number of cases	Rate per 100,000 cases	Annualized rate of change (1990-2010)	Annualized rate of change (2010-2021)	Number of deaths	Rate per 100,000 deaths	Annualized deaths (1990-2010)	Annualized deaths (2010-2021)
	Under 5	0·00902 (0·00707 to 0·0116)	9·11 (7·15 to 11·7)	-1·75 (-2·50 to -0·819)	-2·91 (-4·35 to -1·56)	0·000592 (0·000430 to 0·000903)	0·598 (0·434 to 0·912)	-4·95 (-6·80 to -2·75)	-2·57 (-5·46 to 0·0991)
	5-14 years	0·0312 (0·0212 to 0·0440)	10·7 (7·28 to 15·1)	-2·03 (-2·91 to -1·10)	-3·25 (-4·60 to -1·70)	0·000739 (0·000533 to 0·000979)	0·254 (0·183 to 0·336)	-5·17 (-7·12 to -3·21)	-4·86 (-7·83 to -2·26)
	15-49 years	0·210 (0·174 to 0·248)	31·9 (26·4 to 37·7)	-1·55 (-1·95 to -1·20)	-1·99 (-2·66 to -1·32)	0·0172 (0·0129 to 0·0241)	2·62 (1·96 to 3·67)	-3·41 (-5·04 to -1·72)	-3·18 (-5·48 to -0·615)
	50-69 years	0·148 (0·120 to 0·182)	63·8 (51·7 to 78·3)	-2·01 (-2·63 to -1·32)	-2·25 (-2·92 to -1·30)	0·0290 (0·0203 to 0·0415)	12·5 (8·76 to 17·9)	-4·52 (-6·47 to -2·93)	-4·04 (-7·13 to -0·949)
	70+ years	0·0906 (0·0742 to 0·110)	98·9 (81·0 to 120)	-2·01 (-2·56 to -1·46)	-2·35 (-3·20 to -1·45)	0·0376 (0·0275 to 0·0498)	41·0 (30·1 to 54·3)	-3·75 (-5·00 to -1·78)	-4·17 (-7·18 to -1·36)
Tonga	All Ages	32·7 (28·7 to 35·9)	34·9 (30·9 to 38·2)	-0·496 (-0·790 to -0·215)	-1·03 (-1·47 to -0·578)	5·01 (3·81 to 6·44)	6·09 (4·67 to 7·80)	-0·979 (-2·37 to 0·435)	-1·98 (-3·85 to 0·394)
	Under 5	1·41 (1·12 to 1·86)	9·78 (7·76 to 12·9)	-0·588 (-1·19 to 0·0386)	-2·59 (-3·60 to -1·43)	0·0836 (0·0609 to 0·111)	0·580 (0·422 to 0·768)	-2·35 (-4·10 to -0·566)	-6·54 (-9·53 to -3·79)
	5-14 years	2·80 (1·93 to 3·94)	11·4 (7·84 to 16·0)	-0·881 (-1·54 to -0·186)	-2·14 (-3·51 to -0·896)	0·0678 (0·0463 to 0·0902)	0·275 (0·188 to 0·366)	-1·75 (-3·54 to 0·144)	-3·74 (-6·48 to -0·978)
	15-49 years	15·1 (12·0 to 17·8)	30·6 (24·3 to 36·1)	-0·661 (-0·997 to -0·346)	-1·11 (-1·60 to -0·588)	1·08 (0·743 to 1·44)	2·19 (1·51 to 2·93)	-1·07 (-2·89 to 0·868)	-2·39 (-4·86 to 0·390)
	50-69 years	8·93 (7·28 to 10·8)	65·2 (53·1 to 79·2)	-1·04 (-1·41 to -0·644)	-1·51 (-2·14 to -0·758)	1·85 (1·34 to 2·49)	13·5 (9·75 to 18·2)	-2·05 (-3·49 to -0·149)	-2·94 (-5·40 to -0·187)
	70+ years	4·50 (3·63 to 5·39)	103 (82·9 to 123)	-0·966 (-1·41 to -0·470)	-1·19 (-2·00 to -0·203)	1·93 (1·54 to 2·54)	44·1 (35·0 to 57·9)	-1·85 (-3·25 to -0·274)	-2·48 (-4·74 to 0·192)
Tuvalu	All Ages	26·1 (23·5 to 28·7)	223 (201 to 245)	-1·60 (-1·91 to -1·38)	-1·47 (-1·86 to -1·06)	4·57 (3·18 to 5·60)	43·1 (30·2 to 52·4)	-3·98 (-4·90 to -2·95)	-2·53 (-4·48 to -0·677)
	Under 5	0·805 (0·629 to 1·01)	62·7 (49·0 to 78·8)	-3·05 (-3·97 to -2·26)	-2·67 (-3·72 to -1·43)	0·0634 (0·0457 to 0·0849)	4·94 (3·56 to 6·62)	-8·59 (-9·95 to -6·97)	-5·73 (-8·40 to -2·91)
	5-14 years	2·04 (1·43 to 2·94)	83·6 (58·5 to 120)	-2·29 (-3·13 to -1·60)	-2·55 (-3·78 to -1·43)	0·0610 (0·0460 to 0·0788)	2·49 (1·88 to 3·22)	-6·66 (-8·23 to -4·97)	-4·79 (-7·08 to -2·62)
	15-49 years	12·6 (10·2 to 14·5)	201 (163 to 233)	-1·57 (-1·93 to -1·18)	-1·56 (-2·15 to -0·927)	1·20 (0·828 to 1·58)	19·2 (13·2 to 25·2)	-4·18 (-6·04 to -2·75)	-2·81 (-5·17 to -0·451)
	50-69 years	7·68 (6·38 to 9·18)	408 (340 to 489)	-2·05 (-2·45 to -1·64)	-1·40 (-1·95 to -0·824)	1·81 (1·21 to 2·39)	96·5 (64·4 to 127)	-4·78 (-5·82 to -3·37)	-2·53 (-5·49 to -0·312)
	70+ years	3·06 (2·49 to 3·55)	600 (489 to 697)	-2·00 (-2·69 to -1·38)	-2·10 (-2·91 to -1·37)	1·42 (0·991 to 1·78)	280 (195 to 350)	-3·81 (-5·35 to -2·49)	-3·23 (-5·29 to -0·889)
Vanuatu	All Ages	165 (148 to 183)	64·2 (58·1 to 70·2)	-0·380 (-0·652 to -0·133)	-0·244 (-0·632 to 0·147)	35·0 (21·5 to 49·0)	17·8 (11·0 to 24·6)	-0·883 (-2·26 to 0·261)	-0·731 (-2·32 to 0·905)
	Under 5	8·39 (6·65 to 10·5)	19·9 (15·8 to 24·9)	-0·0116 (-0·555 to 0·609)	-0·646 (-1·52 to 0·268)	0·974 (0·563 to 1·46)	2·31 (1·34 to 3·47)	-0·523 (-2·50 to 1·14)	-4·12 (-7·27 to -1·32)
	5-14 years	17·9 (12·4 to 24·9)	24·0 (16·7 to 33·4)	-0·291 (-0·996 to 0·526)	0·679 (-0·516 to 1·71)	0·779 (0·520 to 1·05)	1·05 (0·699 to 1·42)	-0·737 (-2·82 to 1·28)	-3·37 (-6·09 to -1·02)
	15-49 years	88·8 (74·5 to 101)	56·9 (47·7 to 64·9)	-0·738 (-1·06 to -0·384)	-0·629 (-1·22 to -0·100)	13·0 (7·63 to 18·8)	8·31 (4·89 to 12·0)	-0·977 (-2·68 to 0·485)	-1·04 (-3·28 to 1·04)

eTable 1. All-form tuberculosis incident cases and deaths, age-standardised rates of incidence and mortality per 100,000 population, and corresponding annualized rates of change by age groups for 204 countries and territories (2021).

Location	Age group	Number of cases	Rate per 100,000 cases	Annualized rate of change (1990-2010)	Annualized rate of change (2010-2021)	Number of deaths	Rate per 100,000 deaths	Annualized deaths (1990-2010)	Annualized deaths (2010-2021)
	50-69 years	38·7 (31·8 to 46·6)	115 (94·1 to 138)	-0·976 (-1·36 to -0·569)	-0·967 (-1·60 to -0·271)	12·8 (6·84 to 19·4)	37·9 (20·3 to 57·4)	-2·02 (-3·75 to -0·687)	-1·51 (-3·50 to 0·563)
	70+ years	11·5 (9·46 to 12·9)	173 (142 to 194)	-1·05 (-1·61 to -0·497)	-1·65 (-2·52 to -0·804)	7·49 (5·18 to 10·9)	113 (77·8 to 164)	-1·65 (-3·03 to -0·218)	-1·86 (-3·85 to -0·111)
Southeast Asia	All Ages	1320000 (1200000 to 1460000)	188 (172 to 206)	-1·14 (-1·29 to -0·944)	-0·594 (-0·760 to -0·372)	183000 (165000 to 217000)	28·7 (25·9 to 34·0)	-1·90 (-2·49 to -1·05)	-3·18 (-4·37 to -1·72)
	Under 5	34400 (28600 to 43000)	61·2 (50·9 to 76·4)	-2·94 (-3·26 to -2·65)	-0·271 (-0·608 to 0·144)	2970 (2310 to 3660)	5·28 (4·10 to 6·51)	-6·00 (-7·01 to -5·22)	-7·57 (-9·49 to -5·22)
	5-14 years	63300 (44800 to 87100)	54·4 (38·5 to 74·9)	-2·99 (-3·40 to -2·54)	-0·135 (-0·623 to 0·346)	1560 (1360 to 1770)	1·34 (1·17 to 1·52)	-4·41 (-5·05 to -3·79)	-7·11 (-8·26 to -5·92)
	15-49 years	590000 (494000 to 683000)	159 (133 to 184)	-1·51 (-1·75 to -1·28)	-1·69 (-1·85 to -1·52)	48700 (43800 to 56000)	13·1 (11·8 to 15·1)	-2·25 (-2·82 to -1·55)	-4·79 (-6·07 to -3·69)
	50-69 years	456000 (374000 to 554000)	366 (300 to 444)	-1·79 (-2·00 to -1·58)	-1·26 (-1·52 to -1·01)	69800 (61600 to 84500)	56·0 (49·4 to 67·7)	-3·15 (-3·91 to -2·06)	-4·14 (-5·64 to -2·56)
	70+ years	172000 (146000 to 202000)	573 (487 to 671)	-1·59 (-1·82 to -1·35)	-1·48 (-1·78 to -1·12)	60100 (53200 to 71700)	200 (177 to 238)	-2·65 (-3·38 to -1·57)	-4·56 (-5·63 to -2·91)
Cambodia	All Ages	42900 (38400 to 47900)	282 (255 to 312)	-1·70 (-2·05 to -1·35)	-2·84 (-3·52 to -2·23)	6900 (4650 to 9440)	54·2 (37·9 to 74·5)	-2·46 (-3·45 to -1·48)	-3·54 (-5·40 to -1·36)
	Under 5	1300 (1040 to 1600)	74·3 (59·6 to 91·7)	-3·52 (-4·54 to -2·52)	-4·49 (-6·04 to -2·73)	132 (76·5 to 215)	7·57 (4·37 to 12·3)	-6·71 (-8·23 to -5·06)	-9·46 (-12·1 to -6·20)
	5-14 years	2150 (1430 to 3040)	63·8 (42·4 to 90·4)	-3·01 (-4·38 to -1·53)	-4·98 (-6·65 to -3·68)	83·0 (55·6 to 121)	2·47 (1·65 to 3·58)	-5·19 (-6·49 to -3·77)	-8·23 (-10·4 to -5·86)
	15-49 years	21100 (17500 to 24900)	234 (195 to 276)	-2·10 (-2·61 to -1·58)	-3·69 (-4·44 to -2·94)	1920 (1320 to 2720)	21·3 (14·7 to 30·2)	-2·62 (-3·80 to -1·45)	-5·88 (-7·75 to -2·72)
	50-69 years	14000 (11200 to 17000)	594 (472 to 719)	-2·60 (-3·29 to -2·13)	-3·08 (-4·24 to -2·22)	2840 (1830 to 3870)	120 (77·2 to 164)	-3·56 (-4·90 to -2·08)	-4·10 (-6·41 to -1·67)
	70+ years	4380 (3560 to 5160)	800 (652 to 943)	-2·34 (-3·27 to -1·62)	-3·49 (-4·61 to -2·33)	1920 (1330 to 2720)	352 (243 to 497)	-2·57 (-3·82 to -1·37)	-4·07 (-6·08 to -1·86)
Indonesia	All Ages	497000 (448000 to 550000)	190 (172 to 209)	-1·48 (-1·76 to -1·21)	-1·72 (-1·97 to -1·44)	93100 (80700 to 112000)	42·0 (36·4 to 51·1)	-1·45 (-2·30 to -0·0490)	-3·30 (-5·35 to -1·37)
	Under 5	13000 (10700 to 15800)	59·2 (49·0 to 72·3)	-3·01 (-3·37 to -2·59)	-3·64 (-4·18 to -3·08)	1520 (1130 to 1960)	6·92 (5·15 to 8·96)	-6·00 (-7·36 to -4·70)	-6·97 (-9·73 to -4·32)
	5-14 years	20000 (14000 to 28200)	44·1 (30·9 to 62·2)	-3·25 (-3·82 to -2·67)	-3·96 (-4·46 to -3·41)	688 (582 to 832)	1·52 (1·28 to 1·83)	-4·57 (-5·59 to -3·34)	-7·38 (-9·15 to -5·70)
	15-49 years	197000 (163000 to 226000)	129 (107 to 148)	-2·46 (-2·76 to -2·13)	-3·25 (-3·50 to -2·94)	24400 (20800 to 29900)	15·9 (13·5 to 19·5)	-2·41 (-3·20 to -1·14)	-4·94 (-7·06 to -2·86)
	50-69 years	194000 (158000 to 235000)	400 (325 to 483)	-1·07 (-1·35 to -0·752)	-2·09 (-2·47 to -1·77)	37300 (31100 to 45400)	76·7 (64·0 to 93·4)	-2·22 (-3·19 to -0·584)	-4·47 (-6·83 to -2·33)
	70+ years	71900 (60000 to 85200)	745 (623 to 884)	-0·467 (-0·817 to -0·139)	-1·79 (-2·29 to -1·29)	29300 (25300 to 35400)	303 (262 to 367)	-1·45 (-2·67 to 0·414)	-4·37 (-6·37 to -2·15)

eTable 1. All-form tuberculosis incident cases and deaths, age-standardised rates of incidence and mortality per 100,000 population, and corresponding annualized rates of change by age groups for 204 countries and territories (2021).

Location	Age group	Number of cases	Rate per 100,000 cases	Annualized rate of change (1990-2010)	Annualized rate of change (2010-2021)	Number of deaths	Rate per 100,000 deaths	Annualized deaths (1990-2010)	Annualized deaths (2010-2021)
Lao People's Democratic Republic	All Ages	7940 (6790 to 9200)	131 (113 to 148)	-3·94 (-4·25 to -3·63)	-3·60 (-4·20 to -3·06)	1880 (1240 to 2600)	37·4 (25·3 to 50·9)	-4·22 (-5·30 to -2·98)	-4·39 (-6·38 to -1·99)
	Under 5	317 (250 to 395)	38·2 (30·1 to 47·6)	-4·18 (-4·93 to -3·39)	-5·12 (-6·63 to -3·40)	76·0 (53·2 to 115)	9·16 (6·41 to 13·9)	-6·70 (-8·32 to -4·79)	-8·89 (-12·5 to -4·88)
	5-14 years	449 (317 to 646)	30·6 (21·6 to 44·0)	-4·49 (-5·31 to -3·86)	-5·21 (-6·51 to -3·95)	31·0 (20·2 to 43·6)	2·11 (1·37 to 2·97)	-5·58 (-7·04 to -4·00)	-7·93 (-10·5 to -4·97)
	15-49 years	3840 (3130 to 4830)	95·8 (78·2 to 121)	-4·46 (-4·83 to -4·05)	-4·52 (-5·25 to -3·91)	618 (391 to 906)	15·4 (9·76 to 22·6)	-3·89 (-5·11 to -2·50)	-5·80 (-8·39 to -3·30)
	50-69 years	2520 (1930 to 3180)	284 (218 to 358)	-4·08 (-4·53 to -3·61)	-4·02 (-4·84 to -3·25)	677 (416 to 952)	76·4 (46·9 to 107)	-4·63 (-6·01 to -2·91)	-5·24 (-7·84 to -2·34)
	70+ years	824 (656 to 1050)	428 (341 to 544)	-3·25 (-3·71 to -2·79)	-3·94 (-4·84 to -3·02)	473 (300 to 664)	246 (156 to 345)	-3·28 (-4·83 to -1·55)	-4·23 (-7·17 to -1·24)
Malaysia	All Ages	20600 (18200 to 23100)	64·9 (57·8 to 72·1)	-1·52 (-1·88 to -1·18)	-0·418 (-0·945 to 0·0690)	2350 (2020 to 2820)	8·23 (7·05 to 9·79)	-3·00 (-3·91 to -1·93)	-1·08 (-2·39 to 1·00)
	Under 5	284 (232 to 362)	11·6 (9·42 to 14·7)	-3·12 (-3·80 to -2·31)	-2·29 (-3·40 to -1·31)	8·77 (6·68 to 11·0)	0·357 (0·272 to 0·448)	-8·02 (-9·45 to -6·51)	-3·13 (-4·93 to -1·35)
	5-14 years	612 (420 to 874)	11·9 (8·15 to 16·9)	-3·27 (-3·90 to -2·48)	-2·44 (-3·62 to -1·12)	13·0 (10·3 to 16·1)	0·253 (0·199 to 0·313)	-5·62 (-7·12 to -3·86)	-3·67 (-5·25 to -1·78)
	15-49 years	10600 (8750 to 13000)	59·7 (49·3 to 73·0)	-1·75 (-2·20 to -1·34)	-0·975 (-1·72 to -0·263)	696 (568 to 884)	3·92 (3·20 to 4·98)	-2·62 (-3·84 to -1·42)	-2·40 (-3·80 to -0·476)
	50-69 years	6480 (4920 to 8020)	129 (97·6 to 159)	-2·46 (-2·95 to -1·95)	-1·22 (-1·94 to -0·557)	826 (675 to 990)	16·4 (13·4 to 19·7)	-4·60 (-5·71 to -3·10)	-2·44 (-4·47 to 0·195)
	70+ years	2640 (2130 to 3320)	187 (150 to 235)	-2·93 (-3·41 to -2·37)	-2·25 (-3·08 to -1·46)	803 (673 to 1000)	56·8 (47·6 to 71·1)	-4·43 (-5·56 to -2·79)	-3·70 (-5·50 to -1·24)
Maldives	All Ages	255 (224 to 291)	55·7 (49·5 to 62·4)	-3·99 (-4·45 to -3·52)	-1·74 (-2·29 to -1·15)	17·6 (13·8 to 23·5)	4·76 (3·77 to 6·18)	-9·37 (-10·8 to -7·56)	-4·07 (-6·19 to -1·38)
	Under 5	4·53 (3·61 to 5·65)	14·3 (11·4 to 17·8)	-4·44 (-5·16 to -3·69)	-3·11 (-4·05 to -2·04)	0·205 (0·143 to 0·275)	0·646 (0·452 to 0·865)	-13·1 (-15·0 to -11·0)	-8·61 (-11·8 to -5·51)
	5-14 years	8·78 (6·06 to 12·1)	12·8 (8·86 to 17·7)	-5·37 (-6·14 to -4·50)	-4·82 (-6·30 to -3·66)	0·161 (0·129 to 0·228)	0·236 (0·188 to 0·333)	-13·4 (-15·1 to -11·4)	-7·66 (-10·5 to -4·16)
	15-49 years	141 (117 to 173)	41·8 (34·4 to 51·1)	-5·32 (-5·94 to -4·67)	-2·35 (-3·26 to -1·54)	6·76 (4·93 to 8·94)	2·00 (1·46 to 2·64)	-11·4 (-12·5 to -9·76)	-3·11 (-6·32 to -0·0686)
	50-69 years	70·1 (54·7 to 86·6)	108 (84·3 to 133)	-4·58 (-5·21 to -3·87)	-2·78 (-3·68 to -2·05)	4·90 (3·44 to 6·62)	7·55 (5·30 to 10·2)	-11·1 (-13·3 to -8·63)	-6·10 (-9·32 to -2·86)
	70+ years	30·1 (24·7 to 36·7)	221 (182 to 270)	-3·25 (-4·03 to -2·38)	-1·48 (-2·56 to -0·547)	5·60 (4·43 to 7·25)	41·1 (32·6 to 53·3)	-8·12 (-11·4 to -4·99)	-4·67 (-6·66 to -2·37)
Mauritius	All Ages	204 (178 to 237)	13·6 (12·0 to 15·7)	-1·29 (-1·68 to -0·971)	0·162 (-0·249 to 0·676)	16·3 (14·8 to 17·7)	0·965 (0·869 to 1·05)	-3·39 (-3·76 to -3·02)	-0·701 (-1·32 to -0·120)
	Under 5	3·69 (2·85 to 4·94)	5·73 (4·44 to 7·68)	-1·33 (-1·97 to -0·682)	-0·299 (-1·17 to 0·732)	0·0490 (0·0418 to 0·0574)	0·0762 (0·0650 to 0·0893)	-6·03 (-6·92 to -5·30)	-4·32 (-6·00 to -3·06)
	5-14 years	5·31 (3·34 to 7·83)	3·71 (2·33 to 5·47)	-2·13 (-2·72 to -1·42)	-1·12 (-2·14 to 0·0582)	0·0445 (0·0353 to 0·0561)	0·0311 (0·0247 to 0·0392)	-5·46 (-6·31 to -4·62)	-4·64 (-6·41 to -3·33)

eTable 1. All-form tuberculosis incident cases and deaths, age-standardised rates of incidence and mortality per 100,000 population, and corresponding annualized rates of change by age groups for 204 countries and territories (2021).

Location	Age group	Number of cases	Rate per 100,000 cases	Annualized rate of change (1990-2010)	Annualized rate of change (2010-2021)	Number of deaths	Rate per 100,000 deaths	Annualized deaths (1990-2010)	Annualized deaths (2010-2021)
	15-49 years	94·2 (77·4 to 116)	14·7 (12·1 to 18·2)	-1·52 (-1·92 to -1·05)	-0·172 (-0·730 to 0·430)	4·97 (4·24 to 5·81)	0·777 (0·663 to 0·909)	-3·17 (-3·75 to -2·67)	-1·18 (-2·00 to -0·402)
	50-69 years	72·3 (55·6 to 90·2)	22·3 (17·1 to 27·8)	-3·08 (-3·64 to -2·52)	-1·15 (-2·08 to -0·198)	7·01 (6·31 to 7·82)	2·16 (1·95 to 2·41)	-6·01 (-6·54 to -5·49)	-2·70 (-3·79 to -1·85)
	70+ years	28·7 (22·4 to 36·0)	28·6 (22·4 to 35·9)	-2·38 (-2·96 to -1·64)	-1·73 (-2·78 to -0·962)	4·19 (3·81 to 4·58)	4·17 (3·79 to 4·57)	-5·67 (-6·13 to -5·13)	-3·99 (-4·75 to -2·91)
Myanmar	All Ages	97600 (88900 to 108000)	180 (164 to 199)	-2·93 (-3·18 to -2·67)	-4·12 (-4·55 to -3·57)	17000 (13300 to 22300)	35·2 (27·2 to 45·7)	-3·03 (-4·32 to -1·73)	-6·21 (-8·26 to -4·03)
	Under 5	2800 (2230 to 3510)	53·6 (42·8 to 67·2)	-3·40 (-4·00 to -2·65)	-4·78 (-5·99 to -3·51)	533 (334 to 763)	10·2 (6·39 to 14·6)	-6·17 (-7·99 to -4·38)	-10·5 (-13·3 to -7·98)
	5-14 years	4900 (3260 to 6920)	47·2 (31·4 to 66·6)	-3·91 (-4·63 to -3·05)	-5·96 (-7·31 to -4·55)	203 (145 to 278)	1·95 (1·40 to 2·67)	-5·13 (-6·59 to -3·34)	-10·4 (-13·2 to -8·11)
	15-49 years	47700 (40100 to 55300)	162 (137 to 188)	-3·04 (-3·42 to -2·63)	-4·65 (-5·25 to -4·01)	4990 (3900 to 6700)	17·0 (13·3 to 22·8)	-2·53 (-4·09 to -1·15)	-8·70 (-10·8 to -6·42)
	50-69 years	31000 (25400 to 37400)	338 (277 to 407)	-3·95 (-4·35 to -3·59)	-4·38 (-5·05 to -3·72)	6200 (4530 to 8410)	67·6 (49·4 to 91·7)	-4·41 (-5·89 to -3·01)	-6·41 (-8·80 to -3·62)
	70+ years	11300 (8950 to 13000)	504 (400 to 583)	-3·44 (-3·95 to -3·04)	-4·81 (-5·82 to -3·77)	5120 (3700 to 7000)	229 (165 to 313)	-3·44 (-5·24 to -1·86)	-5·29 (-7·98 to -2·72)
Philippines	All Ages	352000 (311000 to 398000)	341 (304 to 385)	0·690 (0·539 to 0·838)	3·42 (3·11 to 3·86)	26200 (21500 to 30800)	30·4 (25·3 to 35·7)	-0·707 (-1·77 to 0·324)	-2·09 (-3·88 to -0·389)
	Under 5	13000 (10500 to 16700)	116 (93·6 to 149)	-3·07 (-3·29 to -2·84)	12·8 (12·3 to 13·3)	509 (394 to 656)	4·54 (3·51 to 5·85)	-4·73 (-6·03 to -3·00)	-6·06 (-8·41 to -3·25)
	5-14 years	27900 (20000 to 38800)	123 (87·8 to 170)	-1·09 (-1·40 to -0·708)	12·6 (11·8 to 13·4)	420 (368 to 478)	1·84 (1·61 to 2·10)	-3·28 (-4·51 to -1·89)	-4·65 (-6·27 to -3·17)
	15-49 years	178000 (145000 to 207000)	297 (241 to 345)	0·905 (0·704 to 1·13)	1·24 (0·814 to 1·69)	7410 (6000 to 8780)	12·3 (9·99 to 14·6)	-1·84 (-2·92 to -0·857)	-3·02 (-4·86 to -1·28)
	50-69 years	101000 (80400 to 129000)	650 (515 to 828)	-0·589 (-0·848 to -0·350)	2·35 (1·85 to 2·96)	10700 (8580 to 13100)	68·8 (55·0 to 84·1)	-0·583 (-1·83 to 0·647)	-3·34 (-5·46 to -1·36)
	70+ years	31500 (26000 to 36300)	872 (720 to 1000)	-1·51 (-1·79 to -1·22)	5·28 (4·78 to 5·91)	7080 (6010 to 8140)	196 (166 to 225)	-0·877 (-2·24 to 0·324)	-3·16 (-4·71 to -1·68)
Seychelles	All Ages	37·1 (32·4 to 42·2)	32·1 (28·2 to 35·9)	-0·886 (-1·16 to -0·578)	-0·840 (-1·30 to -0·307)	3·69 (3·16 to 4·44)	3·24 (2·79 to 3·88)	-2·48 (-3·08 to -1·74)	-2·70 (-4·38 to -1·35)
	Under 5	0·634 (0·511 to 0·794)	8·05 (6·49 to 10·1)	-1·07 (-1·97 to -0·406)	-1·22 (-2·34 to -0·210)	0·0247 (0·0196 to 0·0312)	0·314 (0·248 to 0·396)	-1·77 (-3·00 to -0·595)	-1·90 (-3·76 to 0·188)
	5-14 years	0·832 (0·542 to 1·19)	5·36 (3·49 to 7·65)	-1·62 (-2·17 to -0·987)	-2·63 (-3·67 to -1·26)	0·0109 (0·00868 to 0·0139)	0·0702 (0·0559 to 0·0897)	-3·09 (-4·40 to -1·74)	-6·68 (-8·97 to -4·42)
	15-49 years	17·6 (14·5 to 21·0)	32·5 (26·7 to 38·8)	-1·13 (-1·66 to -0·689)	-1·14 (-1·87 to -0·477)	1·08 (0·869 to 1·31)	2·00 (1·60 to 2·42)	-2·33 (-3·28 to -1·45)	-3·41 (-5·05 to -1·55)
	50-69 years	13·7 (10·4 to 16·9)	61·0 (46·5 to 75·5)	-1·96 (-2·57 to -1·55)	-1·72 (-2·51 to -1·07)	1·40 (1·11 to 1·76)	6·28 (4·96 to 7·85)	-4·20 (-5·19 to -3·10)	-4·45 (-6·76 to -2·57)
	70+ years	4·44 (3·37 to 5·50)	80·5 (61·1 to 99·6)	-1·45 (-1·96 to -0·938)	-1·96 (-2·79 to -1·27)	1·17 (0·991 to 1·41)	21·2 (17·9 to 25·5)	-2·68 (-3·48 to -1·74)	-3·26 (-4·85 to -1·61)

eTable 1. All-form tuberculosis incident cases and deaths, age-standardised rates of incidence and mortality per 100,000 population, and corresponding annualized rates of change by age groups for 204 countries and territories (2021).

Location	Age group	Number of cases	Rate per 100,000 cases	Annualized rate of change (1990-2010)	Annualized rate of change (2010-2021)	Number of deaths	Rate per 100,000 deaths	Annualized deaths (1990-2010)	Annualized deaths (2010-2021)
Sri Lanka	All Ages	11400 (10200 to 13000)	45·7 (41·0 to 51·7)	-0·0797 (-0·471 to 0·301)	-0·750 (-1·20 to -0·287)	909 (620 to 1300)	3·55 (2·46 to 5·08)	-3·58 (-4·61 to -2·24)	-3·92 (-7·13 to -0·967)
	Under 5	163 (126 to 205)	10·4 (8·05 to 13·1)	-0·433 (-1·29 to 0·351)	-2·41 (-3·55 to -1·15)	3·05 (2·22 to 4·08)	0·195 (0·142 to 0·260)	-5·77 (-6·69 to -4·49)	-9·69 (-12·4 to -6·93)
	5-14 years	294 (198 to 424)	8·30 (5·58 to 12·0)	-1·50 (-2·26 to -0·640)	-4·25 (-5·73 to -2·67)	3·64 (2·54 to 5·09)	0·103 (0·0718 to 0·144)	-7·91 (-9·13 to -6·60)	-8·62 (-11·8 to -5·90)
	15-49 years	4620 (3810 to 5670)	41·9 (34·6 to 51·4)	-0·953 (-1·39 to -0·601)	-0·996 (-1·65 to -0·470)	169 (117 to 237)	1·53 (1·06 to 2·15)	-5·65 (-6·65 to -4·42)	-4·78 (-7·61 to -1·67)
	50-69 years	4430 (3480 to 5470)	95·9 (75·2 to 118)	-0·788 (-1·28 to -0·308)	-1·79 (-2·55 to -0·946)	354 (222 to 536)	7·66 (4·81 to 11·6)	-4·89 (-6·22 to -3·11)	-5·97 (-9·58 to -2·49)
	70+ years	1910 (1440 to 2400)	125 (94·6 to 158)	-0·843 (-1·32 to -0·294)	-2·19 (-2·89 to -1·51)	380 (267 to 513)	24·9 (17·6 to 33·7)	-4·72 (-5·85 to -3·31)	-6·30 (-9·19 to -3·85)
Thailand	All Ages	116000 (101000 to 132000)	137 (120 to 155)	0·102 (-0·232 to 0·411)	-0·187 (-0·657 to 0·410)	12700 (10600 to 15000)	13·1 (10·8 to 15·5)	-1·77 (-2·90 to -0·231)	-0·265 (-1·73 to 1·44)
	Under 5	665 (508 to 842)	23·5 (18·0 to 29·8)	-1·98 (-2·68 to -1·16)	-2·62 (-3·86 to -1·18)	25·6 (21·1 to 31·2)	0·904 (0·747 to 1·10)	-6·65 (-8·21 to -5·36)	-5·54 (-7·61 to -3·86)
	5-14 years	2720 (1860 to 3800)	39·1 (26·7 to 54·8)	-0·841 (-1·56 to -0·0382)	-1·41 (-2·53 to -0·0864)	27·5 (21·9 to 35·7)	0·396 (0·316 to 0·514)	-4·44 (-6·33 to -2·41)	-6·26 (-8·73 to -4·14)
	15-49 years	45700 (36400 to 55800)	143 (114 to 175)	0·0346 (-0·573 to 0·551)	-1·10 (-1·77 to -0·430)	3180 (2210 to 4180)	9·98 (6·91 to 13·1)	-0·268 (-1·69 to 1·23)	-1·91 (-3·05 to -0·407)
	50-69 years	43800 (33700 to 53700)	233 (179 to 286)	-2·04 (-2·49 to -1·49)	-1·56 (-2·29 to -0·811)	3880 (2970 to 5020)	20·7 (15·8 to 26·8)	-5·47 (-6·79 to -3·79)	-1·82 (-3·88 to 0·193)
	70+ years	22900 (18700 to 28800)	368 (300 to 462)	-2·30 (-2·77 to -1·75)	-1·96 (-2·97 to -1·18)	5610 (4310 to 6960)	90·1 (69·1 to 112)	-5·42 (-6·72 to -3·96)	-3·77 (-5·97 to -1·38)
Timor-Leste	All Ages	3690 (3370 to 4050)	342 (312 to 374)	-1·44 (-1·76 to -1·17)	-0·0255 (-0·393 to 0·294)	649 (446 to 976)	72·7 (50·1 to 112)	-3·18 (-4·20 to -1·98)	0·0668 (-1·99 to 2·07)
	Under 5	203 (166 to 256)	110 (89·7 to 138)	-2·79 (-3·60 to -2·11)	-0·221 (-1·56 to 1·04)	34·4 (24·6 to 47·9)	18·6 (13·3 to 25·9)	-8·52 (-10·2 to -7·08)	-2·63 (-6·09 to 0·0364)
	5-14 years	301 (201 to 429)	89·8 (59·8 to 128)	-2·98 (-3·70 to -2·23)	0·394 (-0·644 to 1·54)	12·1 (9·32 to 16·5)	3·61 (2·77 to 4·91)	-6·38 (-7·96 to -5·02)	-2·32 (-4·61 to 0·261)
	15-49 years	1500 (1240 to 1730)	218 (179 to 251)	-2·65 (-3·09 to -2·24)	-0·358 (-0·841 to 0·162)	144 (98·6 to 197)	20·9 (14·3 to 28·6)	-4·74 (-6·00 to -3·21)	0·266 (-2·20 to 2·49)
	50-69 years	1130 (924 to 1370)	795 (650 to 963)	-0·782 (-1·22 to -0·280)	-0·769 (-1·47 to -0·104)	227 (151 to 342)	160 (106 to 240)	-2·69 (-4·12 to -0·862)	-0·979 (-3·48 to 1·81)
	70+ years	555 (456 to 630)	1230 (1010 to 1390)	-0·986 (-1·58 to -0·498)	-0·533 (-1·23 to 0·159)	232 (157 to 407)	512 (347 to 901)	-2·61 (-3·97 to -0·525)	-0·658 (-3·26 to 1·59)
Viet Nam	All Ages	165000 (150000 to 180000)	160 (147 to 174)	-0·179 (-0·437 to 0·147)	-1·11 (-1·54 to -0·590)	21100 (17100 to 28100)	23·1 (18·5 to 30·1)	-2·34 (-3·58 to -1·01)	-3·44 (-5·19 to -1·34)
	Under 5	2690 (2130 to 3330)	33·0 (26·2 to 41·0)	-1·92 (-2·48 to -1·31)	-3·70 (-5·01 to -2·56)	128 (86·7 to 176)	1·57 (1·06 to 2·17)	-7·71 (-9·54 to -5·75)	-9·30 (-12·5 to -6·00)
	5-14 years	3800 (2640 to 5310)	22·9 (15·9 to 32·0)	-2·34 (-3·26 to -1·28)	-5·99 (-7·65 to -4·00)	75·4 (55·6 to 100)	0·454 (0·334 to 0·604)	-4·65 (-6·41 to -3·15)	-7·43 (-10·6 to -4·45)

eTable 1. All-form tuberculosis incident cases and deaths, age-standardised rates of incidence and mortality per 100,000 population, and corresponding annualized rates of change by age groups for 204 countries and territories (2021).

Location	Age group	Number of cases	Rate per 100,000 cases	Annualized rate of change (1990-2010)	Annualized rate of change (2010-2021)	Number of deaths	Rate per 100,000 deaths	Annualized deaths (1990-2010)	Annualized deaths (2010-2021)
	15-49 years	78000 (66100 to 88900)	150 (127 to 171)	-0·290 (-0·689 to 0·259)	-1·37 (-2·01 to -0·736)	5090 (4130 to 6340)	9·78 (7·93 to 12·2)	-1·74 (-3·24 to 0·221)	-4·68 (-6·72 to -2·59)
	50-69 years	56300 (46000 to 66800)	297 (243 to 353)	-1·61 (-2·11 to -1·17)	-1·33 (-2·03 to -0·673)	6710 (4760 to 9610)	35·4 (25·1 to 50·7)	-4·83 (-6·21 to -3·27)	-3·88 (-6·07 to -1·36)
	70+ years	24100 (21200 to 26700)	539 (476 to 599)	-1·67 (-2·11 to -1·14)	-2·03 (-2·72 to -1·38)	9110 (6900 to 11700)	204 (155 to 263)	-3·64 (-4·94 to -2·08)	-4·24 (-6·11 to -2·45)
Sub-Saharan Africa	All Ages	3010000 (2670000 to 3370000)	351 (315 to 391)	-0·418 (-0·564 to -0·242)	-4·06 (-4·23 to -3·82)	533000 (456000 to 609000)	83·2 (72·3 to 94·5)	-0·814 (-1·39 to -0·363)	-6·10 (-7·24 to -5·08)
	Under 5	230000 (191000 to 282000)	133 (111 to 163)	-2·05 (-2·34 to -1·73)	-5·46 (-5·83 to -5·02)	44800 (31200 to 57800)	25·9 (18·1 to 33·5)	-3·36 (-3·98 to -2·68)	-9·23 (-11·2 to -7·03)
	5-14 years	201000 (139000 to 281000)	66·4 (45·9 to 92·6)	-0·203 (-0·487 to 0·195)	-5·18 (-5·51 to -4·80)	12600 (10300 to 15100)	4·17 (3·40 to 4·97)	1·39 (0·555 to 2·28)	-7·88 (-9·22 to -6·83)
	15-49 years	1870000 (1580000 to 2220000)	342 (289 to 406)	-0·0102 (-0·185 to 0·188)	-4·28 (-4·50 to -4·01)	240000 (204000 to 280000)	43·9 (37·3 to 51·2)	0·784 (0·0659 to 1·40)	-7·15 (-8·18 to -6·20)
	50-69 years	538000 (424000 to 671000)	595 (468 to 741)	-0·697 (-0·929 to -0·491)	-3·60 (-3·92 to -3·21)	154000 (130000 to 178000)	170 (144 to 197)	-1·03 (-1·71 to -0·413)	-4·90 (-6·00 to -3·64)
	70+ years	163000 (135000 to 194000)	831 (689 to 992)	-0·744 (-0·970 to -0·503)	-3·20 (-3·51 to -2·88)	81400 (70200 to 91700)	416 (358 to 468)	-1·55 (-2·16 to -0·981)	-3·60 (-4·61 to -2·07)
Central Sub-Saharan Africa	All Ages	445000 (396000 to 497000)	435 (389 to 485)	-0·379 (-0·586 to -0·172)	-3·01 (-3·43 to -2·59)	78600 (57000 to 105000)	107 (77·9 to 144)	-1·02 (-1·83 to -0·212)	-5·74 (-7·60 to -3·95)
	Under 5	50800 (41200 to 62800)	241 (195 to 298)	-0·973 (-1·49 to -0·526)	-4·14 (-5·02 to -3·35)	7170 (4190 to 10300)	34·0 (19·9 to 49·0)	-3·00 (-3·96 to -1·96)	-11·2 (-13·3 to -8·60)
	5-14 years	37900 (25900 to 51900)	101 (68·8 to 138)	-0·734 (-1·23 to -0·258)	-4·10 (-4·85 to -3·03)	2250 (1740 to 3140)	5·98 (4·63 to 8·34)	0·256 (-0·758 to 1·39)	-7·61 (-9·49 to -5·72)
	15-49 years	245000 (203000 to 287000)	376 (311 to 441)	-0·154 (-0·493 to 0·172)	-3·13 (-3·70 to -2·59)	31700 (22700 to 42300)	48·7 (34·9 to 64·9)	0·286 (-0·418 to 1·18)	-5·93 (-7·69 to -4·02)
	50-69 years	93300 (75500 to 117000)	836 (677 to 1050)	-0·151 (-0·455 to 0·184)	-2·22 (-2·95 to -1·72)	27300 (18300 to 37400)	245 (164 to 335)	-0·606 (-2·08 to 0·636)	-4·25 (-6·28 to -2·19)
	70+ years	18300 (15000 to 21700)	968 (795 to 1150)	0·124 (-0·306 to 0·663)	-3·00 (-4·06 to -2·18)	10100 (7130 to 13900)	535 (378 to 737)	-0·711 (-1·82 to 0·648)	-3·75 (-5·89 to -1·78)
Angola	All Ages	88900 (79100 to 99500)	373 (338 to 410)	-0·579 (-0·898 to -0·271)	-2·89 (-3·50 to -2·37)	15600 (11700 to 20400)	90·4 (68·4 to 116)	-2·16 (-3·56 to -1·13)	-5·26 (-7·25 to -2·98)
	Under 5	11900 (9440 to 14700)	211 (168 to 261)	-0·889 (-1·51 to -0·262)	-4·36 (-5·42 to -3·44)	1990 (1300 to 2720)	35·4 (23·1 to 48·3)	-4·20 (-5·79 to -2·68)	-10·1 (-12·9 to -7·52)
	5-14 years	7760 (5430 to 10600)	80·8 (56·5 to 110)	-1·48 (-2·18 to -0·793)	-4·12 (-5·31 to -2·87)	446 (307 to 596)	4·64 (3·20 to 6·20)	-1·85 (-3·73 to -0·636)	-7·22 (-9·61 to -5·30)
	15-49 years	49800 (41400 to 59300)	340 (282 to 404)	-0·0250 (-0·379 to 0·354)	-2·54 (-3·31 to -1·83)	6890 (4920 to 9520)	47·0 (33·6 to 64·9)	0·000238 (-1·73 to 1·28)	-4·29 (-6·23 to -2·14)
	50-69 years	16300 (13000 to 20100)	679 (544 to 840)	-0·842 (-1·24 to -0·449)	-2·07 (-2·95 to -1·09)	4570 (3300 to 5980)	191 (138 to 250)	-2·12 (-3·94 to -0·648)	-3·85 (-6·60 to -1·19)

eTable 1. All-form tuberculosis incident cases and deaths, age-standardised rates of incidence and mortality per 100,000 population, and corresponding annualized rates of change by age groups for 204 countries and territories (2021).

Location	Age group	Number of cases	Rate per 100,000 cases	Annualized rate of change (1990-2010)	Annualized rate of change (2010-2021)	Number of deaths	Rate per 100,000 deaths	Annualized deaths (1990-2010)	Annualized deaths (2010-2021)
	70+ years	3160 (2520 to 3750)	787 (629 to 935)	-1·01 (-1·40 to -0·400)	-2·13 (-3·28 to -1·21)	1700 (1190 to 2270)	424 (295 to 565)	-2·00 (-3·47 to -0·410)	-3·48 (-5·77 to -0·750)
Central African Republic	All Ages	33300 (30100 to 37200)	787 (715 to 866)	0·0395 (-0·307 to 0·294)	-1·56 (-2·00 to -1·16)	9500 (6730 to 12700)	298 (215 to 386)	0·281 (-0·707 to 1·43)	-3·39 (-5·32 to -1·78)
	Under 5	3640 (2920 to 4590)	434 (348 to 547)	-0·629 (-1·20 to -0·104)	-2·54 (-3·54 to -1·53)	1060 (744 to 1500)	127 (88·7 to 178)	-1·14 (-2·70 to 0·223)	-6·50 (-9·57 to -3·82)
	5-14 years	2990 (2030 to 4320)	207 (140 to 299)	-0·405 (-1·09 to 0·376)	-1·23 (-2·66 to -0·0258)	264 (189 to 338)	18·2 (13·1 to 23·4)	2·00 (0·590 to 3·65)	-5·21 (-7·28 to -3·46)
	15-49 years	19100 (16200 to 21800)	715 (607 to 817)	0·447 (0·0528 to 0·803)	-1·77 (-2·32 to -1·28)	4180 (3110 to 5660)	157 (117 to 212)	1·72 (0·717 to 2·97)	-4·06 (-6·04 to -1·94)
	50-69 years	6570 (5570 to 7810)	1400 (1180 to 1660)	0·0674 (-0·363 to 0·482)	-1·72 (-2·34 to -0·988)	3210 (2100 to 4280)	683 (447 to 909)	0·159 (-1·14 to 1·70)	-2·42 (-4·44 to -0·576)
	70+ years	1070 (888 to 1200)	1670 (1390 to 1880)	-0·174 (-0·752 to 0·547)	-1·21 (-2·32 to -0·435)	781 (513 to 1020)	1220 (804 to 1590)	-0·201 (-1·22 to 1·14)	-1·57 (-2·93 to 0·00965)
Congo	All Ages	15100 (13000 to 17200)	331 (289 to 367)	-0·779 (-1·06 to -0·472)	-2·62 (-3·18 to -2·02)	2800 (2000 to 4180)	77·4 (55·9 to 111)	-1·87 (-2·93 to -0·461)	-4·10 (-5·95 to -2·03)
	Under 5	1000 (802 to 1270)	159 (127 to 200)	-1·62 (-2·38 to -1·11)	-4·66 (-5·85 to -3·59)	129 (86·7 to 182)	20·4 (13·7 to 28·7)	-3·57 (-5·09 to -2·17)	-9·98 (-12·4 to -7·02)
	5-14 years	1020 (724 to 1420)	78·4 (55·9 to 110)	-0·281 (-0·969 to 0·412)	-4·28 (-5·29 to -2·93)	57·2 (37·9 to 82·2)	4·41 (2·92 to 6·34)	2·15 (0·236 to 4·11)	-7·73 (-9·76 to -4·90)
	15-49 years	9270 (7680 to 11300)	329 (273 to 400)	-0·789 (-1·08 to -0·428)	-2·79 (-3·50 to -2·00)	1380 (961 to 2040)	48·9 (34·1 to 72·5)	-1·11 (-2·50 to 0·288)	-4·45 (-6·39 to -2·06)
	50-69 years	3240 (2560 to 3990)	591 (468 to 728)	-1·15 (-1·48 to -0·735)	-2·45 (-3·23 to -1·81)	914 (630 to 1400)	167 (115 to 255)	-2·61 (-4·11 to -0·758)	-3·87 (-5·96 to -1·66)
	70+ years	594 (469 to 718)	617 (488 to 747)	-1·20 (-1·75 to -0·685)	-2·49 (-3·46 to -1·43)	318 (230 to 425)	330 (239 to 442)	-2·28 (-3·53 to -0·757)	-3·68 (-5·66 to -1·21)
Democratic Republic of the Congo	All Ages	301000 (265000 to 339000)	449 (400 to 507)	-0·301 (-0·582 to -0·0353)	-3·10 (-3·66 to -2·54)	49600 (33100 to 73700)	106 (70·1 to 158)	-0·781 (-1·81 to 0·142)	-6·23 (-8·18 to -4·18)
	Under 5	33800 (27400 to 42000)	249 (202 to 310)	-0·950 (-1·64 to -0·262)	-4·14 (-5·22 to -3·01)	3930 (2190 to 6570)	29·0 (16·1 to 48·4)	-2·82 (-4·30 to -1·67)	-12·7 (-16·2 to -9·05)
	5-14 years	25600 (17300 to 35400)	105 (71·0 to 145)	-0·569 (-1·13 to -0·0117)	-4·25 (-5·47 to -2·87)	1460 (1090 to 2270)	5·96 (4·48 to 9·27)	0·581 (-0·821 to 1·87)	-7·92 (-10·1 to -5·87)
	15-49 years	163000 (133000 to 192000)	376 (307 to 444)	-0·180 (-0·598 to 0·214)	-3·36 (-4·05 to -2·56)	18800 (12900 to 28000)	43·4 (29·9 to 64·7)	0·283 (-0·664 to 1·20)	-6·74 (-9·14 to -4·46)
	50-69 years	65800 (52500 to 83800)	885 (706 to 1130)	0·106 (-0·302 to 0·512)	-2·21 (-3·13 to -1·52)	18300 (11500 to 27800)	246 (154 to 373)	-0·0982 (-1·94 to 1·20)	-4·58 (-6·65 to -2·09)
	70+ years	13200 (10800 to 15800)	1040 (856 to 1250)	0·492 (-0·0296 to 1·13)	-3·19 (-4·28 to -2·25)	7170 (4690 to 10500)	567 (371 to 829)	-0·299 (-1·75 to 1·39)	-3·90 (-6·52 to -1·61)
Equatorial Guinea	All Ages	2640 (2220 to 3030)	221 (195 to 250)	-3·44 (-3·87 to -2·91)	-3·11 (-3·73 to -2·58)	332 (205 to 511)	39·7 (26·1 to 57·3)	-7·18 (-8·93 to -5·12)	-4·87 (-7·11 to -2·19)
	Under 5	236 (197 to 292)	126 (105 to 156)	-3·35 (-3·94 to -2·48)	-4·51 (-5·55 to -3·51)	28·2 (15·5 to 46·2)	15·1 (8·25 to 24·7)	-7·46 (-9·63 to -4·97)	-9·35 (-11·8 to -6·40)

eTable 1. All-form tuberculosis incident cases and deaths, age-standardised rates of incidence and mortality per 100,000 population, and corresponding annualized rates of change by age groups for 204 countries and territories (2021).

Location	Age group	Number of cases	Rate per 100,000 cases	Annualized rate of change (1990-2010)	Annualized rate of change (2010-2021)	Number of deaths	Rate per 100,000 deaths	Annualized deaths (1990-2010)	Annualized deaths (2010-2021)
	5-14 years	243 (165 to 333)	61·2 (41·5 to 83·8)	-4·14 (-4·89 to -3·48)	-2·36 (-3·84 to -1·32)	13·6 (7·89 to 24·8)	3·41 (1·98 to 6·23)	-5·60 (-7·75 to -3·01)	-4·19 (-6·85 to 0·348)
	15-49 years	1710 (1380 to 2110)	211 (170 to 261)	-2·64 (-3·14 to -2·09)	-3·60 (-4·43 to -2·81)	182 (106 to 283)	22·4 (13·1 to 34·9)	-4·70 (-6·95 to -2·34)	-5·05 (-7·49 to -2·12)
	50-69 years	366 (278 to 462)	378 (287 to 476)	-4·13 (-4·81 to -3·62)	-3·03 (-3·71 to -2·31)	74·9 (49·4 to 111)	77·3 (51·0 to 115)	-8·18 (-9·96 to -5·72)	-3·76 (-6·53 to -0·469)
	70+ years	77·0 (61·6 to 98·0)	402 (322 to 512)	-4·88 (-5·63 to -4·05)	-3·10 (-4·07 to -2·18)	34·0 (22·6 to 49·2)	177 (118 to 257)	-7·59 (-9·22 to -5·25)	-3·24 (-5·65 to 0·333)
Gabon	All Ages	3990 (3500 to 4650)	252 (223 to 292)	1·46 (1·18 to 1·84)	-3·94 (-4·43 to -3·30)	714 (466 to 1050)	55·5 (36·0 to 80·7)	0·139 (-1·58 to 1·83)	-5·37 (-7·27 to -2·80)
	Under 5	247 (192 to 307)	116 (90·0 to 144)	1·27 (0·646 to 1·84)	-7·16 (-8·10 to -6·06)	22·0 (13·7 to 33·2)	10·3 (6·41 to 15·5)	1·25 (-0·379 to 2·80)	-15·5 (-19·1 to -12·5)
	5-14 years	281 (197 to 387)	66·0 (46·3 to 91·0)	2·05 (1·47 to 2·76)	-4·35 (-5·57 to -3·01)	13·6 (8·48 to 19·3)	3·20 (1·99 to 4·55)	2·44 (0·666 to 4·06)	-7·01 (-9·32 to -4·51)
	15-49 years	2340 (1960 to 2910)	252 (211 to 314)	1·85 (1·43 to 2·25)	-4·25 (-4·92 to -3·46)	323 (208 to 480)	34·9 (22·4 to 51·7)	2·15 (0·451 to 3·90)	-6·31 (-8·51 to -3·50)
	50-69 years	945 (708 to 1170)	452 (339 to 558)	0·829 (0·325 to 1·28)	-3·45 (-4·23 to -2·59)	260 (157 to 374)	124 (75·1 to 179)	-1·25 (-3·68 to 0·817)	-3·84 (-5·93 to -0·709)
	70+ years	180 (133 to 226)	444 (328 to 557)	-0·199 (-0·799 to 0·278)	-3·68 (-4·71 to -2·41)	95·4 (61·8 to 135)	235 (152 to 333)	-1·90 (-3·41 to -0·245)	-3·90 (-6·38 to -0·885)
Eastern Sub-Saharan Africa	All Ages	1150000 (1010000 to 1300000)	372 (333 to 416)	-1·14 (-1·34 to -0·954)	-3·76 (-3·98 to -3·49)	226000 (188000 to 264000)	100 (81·4 to 116)	-2·15 (-2·80 to -1·71)	-5·50 (-6·57 to -4·08)
	Under 5	78900 (65600 to 97600)	124 (103 to 153)	-2·28 (-2·64 to -1·85)	-5·18 (-5·54 to -4·67)	16300 (11900 to 21600)	25·6 (18·6 to 33·9)	-4·39 (-5·25 to -3·67)	-9·52 (-11·4 to -7·26)
	5-14 years	76300 (51700 to 108000)	66·5 (45·1 to 94·6)	0·151 (-0·280 to 0·612)	-4·73 (-5·07 to -4·37)	5120 (4190 to 6190)	4·46 (3·66 to 5·40)	1·22 (0·271 to 2·25)	-7·50 (-8·81 to -6·27)
	15-49 years	718000 (597000 to 883000)	343 (285 to 422)	-0·962 (-1·20 to -0·703)	-4·25 (-4·51 to -3·99)	105000 (83600 to 124000)	50·0 (39·9 to 59·3)	-0·974 (-1·77 to -0·361)	-6·58 (-7·72 to -5·38)
	50-69 years	206000 (163000 to 257000)	656 (521 to 818)	-1·60 (-1·92 to -1·28)	-3·65 (-3·94 to -3·24)	63200 (50400 to 75000)	201 (161 to 239)	-2·36 (-3·08 to -1·81)	-4·74 (-5·86 to -3·19)
	70+ years	70400 (58600 to 83100)	1020 (850 to 1210)	-1·29 (-1·56 to -0·974)	-2·86 (-3·15 to -2·53)	36900 (30100 to 43500)	536 (437 to 631)	-2·32 (-3·20 to -1·57)	-3·33 (-4·46 to -1·77)
Burundi	All Ages	36500 (32000 to 42000)	411 (365 to 473)	-1·61 (-1·95 to -1·23)	-4·58 (-5·25 to -3·99)	7760 (5740 to 9920)	130 (90·7 to 169)	-3·01 (-4·21 to -1·98)	-5·13 (-6·96 to -3·36)
	Under 5	2990 (2430 to 3730)	138 (112 to 172)	-1·93 (-2·48 to -1·29)	-5·88 (-6·86 to -5·00)	530 (295 to 837)	24·5 (13·6 to 38·8)	-3·97 (-5·39 to -2·48)	-11·6 (-15·9 to -7·75)
	5-14 years	2680 (1830 to 3830)	72·5 (49·5 to 104)	-0·662 (-1·24 to 0·183)	-5·38 (-6·45 to -4·25)	165 (125 to 215)	4·46 (3·39 to 5·83)	0·497 (-0·866 to 2·02)	-8·49 (-10·9 to -5·98)
	15-49 years	21200 (17500 to 26600)	339 (281 to 426)	-1·67 (-2·16 to -1·08)	-4·99 (-5·87 to -4·29)	3020 (2270 to 3900)	48·3 (36·4 to 62·4)	-2·42 (-4·18 to -1·21)	-6·15 (-8·38 to -3·93)
	50-69 years	7400 (5810 to 9410)	786 (617 to 998)	-1·60 (-2·26 to -1·07)	-3·38 (-4·13 to -2·56)	2570 (1760 to 3470)	272 (187 to 368)	-2·92 (-3·99 to -1·46)	-3·18 (-5·74 to -0·867)

eTable 1. All-form tuberculosis incident cases and deaths, age-standardised rates of incidence and mortality per 100,000 population, and corresponding annualized rates of change by age groups for 204 countries and territories (2021).

Location	Age group	Number of cases	Rate per 100,000 cases	Annualized rate of change (1990-2010)	Annualized rate of change (2010-2021)	Number of deaths	Rate per 100,000 deaths	Annualized deaths (1990-2010)	Annualized deaths (2010-2021)
	70+ years	2280 (1800 to 2800)	1270 (1000 to 1550)	-1·26 (-2·00 to -0·505)	-2·55 (-3·69 to -1·20)	1480 (975 to 2020)	820 (542 to 1120)	-2·22 (-3·23 to -1·11)	-1·90 (-4·51 to 0·152)
Comoros	All Ages	1600 (1400 to 1900)	258 (224 to 302)	-1·89 (-2·34 to -1·38)	-1·25 (-1·92 to -0·482)	309 (222 to 427)	62·3 (44·4 to 84·7)	-3·23 (-4·66 to -1·82)	0·123 (-2·00 to 2·11)
	Under 5	64·1 (48·5 to 79·8)	78·8 (59·6 to 98·2)	-3·17 (-3·98 to -2·32)	-2·97 (-4·24 to -1·48)	11·6 (6·36 to 17·7)	14·3 (7·83 to 21·7)	-5·19 (-7·46 to -3·09)	-5·83 (-9·18 to -3·04)
	5-14 years	43·8 (31·5 to 65·8)	27·6 (19·8 to 41·4)	-3·54 (-4·23 to -2·56)	-3·41 (-4·69 to -2·12)	2·37 (1·60 to 3·46)	1·49 (1·01 to 2·18)	-5·37 (-7·64 to -2·97)	-0·444 (-3·18 to 3·72)
	15-49 years	827 (681 to 1070)	211 (174 to 273)	-2·56 (-3·19 to -1·89)	-2·27 (-3·12 to -1·34)	85·6 (58·7 to 128)	21·9 (15·0 to 32·6)	-4·83 (-7·12 to -2·79)	1·26 (-1·47 to 4·58)
	50-69 years	473 (361 to 609)	527 (402 to 678)	-2·06 (-2·78 to -1·36)	-1·69 (-2·48 to -0·678)	107 (75·2 to 154)	119 (83·8 to 172)	-3·92 (-5·59 to -2·44)	-1·79 (-4·85 to 0·517)
	70+ years	191 (143 to 238)	843 (629 to 1050)	-1·59 (-2·34 to -0·751)	-1·36 (-2·31 to -0·0986)	102 (71·3 to 141)	450 (314 to 623)	-2·64 (-4·34 to -1·17)	-1·36 (-3·38 to 0·954)
Djibouti	All Ages	2770 (2370 to 3240)	274 (240 to 312)	-0·504 (-1·03 to -0·0219)	-3·68 (-4·38 to -3·02)	507 (277 to 737)	68·6 (37·6 to 98·2)	0·813 (-0·822 to 2·61)	-4·56 (-7·19 to -2·05)
	Under 5	120 (98·2 to 151)	82·5 (67·4 to 103)	-2·42 (-3·38 to -1·41)	-5·62 (-6·79 to -4·42)	26·0 (16·5 to 38·8)	17·9 (11·3 to 26·6)	-1·02 (-3·13 to 0·680)	-9·62 (-12·5 to -6·94)
	5-14 years	109 (74·6 to 155)	40·7 (27·9 to 57·9)	-1·13 (-2·46 to 0·429)	-4·06 (-5·30 to -2·64)	5·61 (3·25 to 8·78)	2·10 (1·22 to 3·28)	0·994 (-1·52 to 2·93)	-6·30 (-8·73 to -4·04)
	15-49 years	1660 (1370 to 2100)	241 (199 to 305)	-0·784 (-1·40 to -0·125)	-4·35 (-5·24 to -3·46)	208 (115 to 310)	30·1 (16·7 to 44·9)	1·72 (-0·169 to 3·81)	-5·75 (-8·01 to -3·08)
	50-69 years	715 (538 to 918)	538 (404 to 690)	-1·01 (-1·80 to -0·350)	-3·47 (-4·64 to -2·46)	178 (94·6 to 263)	134 (71·1 to 197)	-0·842 (-2·86 to 1·26)	-4·58 (-8·01 to -1·56)
	70+ years	167 (128 to 213)	763 (584 to 974)	-0·809 (-1·63 to -0·131)	-3·25 (-4·62 to -1·90)	89·0 (49·5 to 121)	406 (226 to 554)	-0·982 (-2·83 to 0·774)	-3·84 (-7·04 to -1·29)
Eritrea	All Ages	27400 (23600 to 31400)	561 (493 to 633)	-1·39 (-1·74 to -0·987)	-2·24 (-2·89 to -1·37)	4830 (3010 to 7520)	144 (89·7 to 210)	-1·67 (-3·37 to -0·457)	-3·35 (-5·37 to -1·26)
	Under 5	2080 (1650 to 2550)	227 (180 to 277)	-2·27 (-2·87 to -1·56)	-3·25 (-4·31 to -1·94)	301 (187 to 468)	32·7 (20·4 to 51·0)	-3·76 (-5·86 to -2·17)	-5·75 (-8·45 to -2·11)
	5-14 years	1220 (816 to 1750)	76·1 (50·8 to 109)	-1·75 (-2·66 to -0·892)	-3·50 (-4·97 to -2·09)	58·9 (35·4 to 104)	3·67 (2·20 to 6·47)	-0·532 (-2·90 to 0·995)	-6·51 (-8·50 to -3·95)
	15-49 years	16600 (13400 to 20700)	486 (391 to 606)	-1·59 (-2·04 to -1·08)	-2·70 (-3·56 to -1·74)	2020 (1190 to 3430)	59·1 (34·7 to 100)	-1·46 (-3·74 to -0·0668)	-4·41 (-6·78 to -1·75)
	50-69 years	6040 (4710 to 7620)	1080 (844 to 1370)	-1·58 (-2·27 to -0·939)	-2·31 (-3·34 to -1·25)	1700 (1080 to 2480)	304 (193 to 444)	-2·22 (-4·15 to -0·756)	-3·78 (-5·74 to -1·81)
	70+ years	1370 (1060 to 1710)	1500 (1160 to 1860)	-0·986 (-1·95 to -0·289)	-2·10 (-3·32 to -0·795)	755 (460 to 1120)	822 (501 to 1220)	-1·35 (-2·72 to 0·100)	-2·49 (-4·44 to -0·492)
Ethiopia	All Ages	224000 (200000 to 249000)	298 (269 to 328)	-2·80 (-3·04 to -2·46)	-3·31 (-3·66 to -3·01)	34000 (28900 to 40300)	66·2 (57·1 to 78·5)	-5·92 (-6·76 to -5·02)	-5·38 (-6·87 to -3·38)
	Under 5	16100 (12800 to 20100)	101 (80·3 to 126)	-3·96 (-4·43 to -3·41)	-3·96 (-4·63 to -3·16)	2420 (1780 to 3110)	15·1 (11·1 to 19·5)	-8·32 (-9·24 to -7·06)	-8·22 (-10·5 to -5·46)

eTable 1. All-form tuberculosis incident cases and deaths, age-standardised rates of incidence and mortality per 100,000 population, and corresponding annualized rates of change by age groups for 204 countries and territories (2021).

Location	Age group	Number of cases	Rate per 100,000 cases	Annualized rate of change (1990-2010)	Annualized rate of change (2010-2021)	Number of deaths	Rate per 100,000 deaths	Annualized deaths (1990-2010)	Annualized deaths (2010-2021)
	5-14 years	13600 (9150 to 19400)	48·1 (32·2 to 68·4)	-2·06 (-2·61 to -1·46)	-4·26 (-4·88 to -3·62)	566 (432 to 724)	1·99 (1·52 to 2·55)	-3·97 (-5·28 to -2·52)	-7·89 (-9·64 to -6·01)
	15-49 years	131000 (107000 to 155000)	238 (194 to 282)	-2·23 (-2·57 to -1·89)	-4·19 (-4·57 to -3·84)	12100 (9890 to 14400)	21·9 (18·0 to 26·1)	-5·05 (-6·01 to -4·07)	-6·96 (-8·64 to -4·83)
	50-69 years	43600 (35600 to 53500)	576 (470 to 706)	-3·75 (-4·06 to -3·44)	-3·35 (-3·92 to -2·88)	9590 (7940 to 11800)	127 (105 to 156)	-6·25 (-7·10 to -5·20)	-5·72 (-7·58 to -3·05)
	70+ years	19600 (16300 to 22800)	993 (825 to 1160)	-3·07 (-3·43 to -2·70)	-2·15 (-2·72 to -1·59)	9340 (7950 to 11200)	474 (403 to 569)	-4·40 (-5·57 to -2·95)	-3·96 (-5·53 to -1·75)
Kenya	All Ages	115000 (99700 to 130000)	305 (270 to 339)	0·721 (0·550 to 0·861)	-5·42 (-5·64 to -5·17)	33100 (20000 to 43700)	119 (71·1 to 159)	1·49 (0·577 to 2·29)	-5·79 (-7·14 to -4·01)
	Under 5	4040 (3310 to 5020)	67·9 (55·7 to 84·4)	-2·25 (-2·55 to -1·92)	-8·45 (-8·77 to -8·14)	1180 (717 to 1550)	19·8 (12·0 to 26·1)	-2·92 (-3·88 to -2·26)	-9·87 (-11·4 to -8·16)
	5-14 years	6630 (4430 to 9360)	52·1 (34·8 to 73·6)	4·57 (4·12 to 5·09)	-7·33 (-7·68 to -6·91)	542 (304 to 679)	4·26 (2·39 to 5·34)	6·49 (4·83 to 7·95)	-11·0 (-12·3 to -9·59)
	15-49 years	72600 (59800 to 89100)	278 (229 to 341)	0·462 (0·225 to 0·674)	-6·28 (-6·58 to -5·93)	14400 (8760 to 19000)	55·1 (33·5 to 72·7)	2·48 (1·48 to 3·45)	-8·17 (-9·57 to -6·53)
	50-69 years	22300 (17700 to 27700)	515 (409 to 640)	0·804 (0·535 to 1·05)	-5·58 (-5·90 to -5·21)	10800 (6580 to 13500)	250 (152 to 313)	1·87 (0·659 to 2·83)	-5·37 (-6·88 to -3·28)
	70+ years	9270 (7490 to 11200)	993 (803 to 1200)	-0·144 (-0·376 to 0·128)	-3·53 (-3·88 to -3·17)	6180 (3750 to 8730)	662 (402 to 935)	-0·436 (-1·37 to 0·349)	-3·19 (-4·76 to -0·930)
Madagascar	All Ages	60200 (51900 to 68900)	299 (263 to 342)	-2·39 (-2·71 to -1·95)	-2·68 (-3·20 to -1·96)	10600 (7660 to 14600)	78·5 (58·1 to 108)	-2·98 (-4·39 to -2·03)	-2·98 (-4·72 to -1·00)
	Under 5	4380 (3490 to 5470)	107 (85·4 to 134)	-2·90 (-3·50 to -2·10)	-4·15 (-5·18 to -2·99)	785 (457 to 1240)	19·2 (11·2 to 30·5)	-4·48 (-6·21 to -2·90)	-7·53 (-11·3 to -2·82)
	5-14 years	3010 (2020 to 4310)	39·4 (26·4 to 56·4)	-3·29 (-4·04 to -2·54)	-3·84 (-5·10 to -2·34)	164 (113 to 238)	2·14 (1·47 to 3·12)	-4·78 (-6·52 to -3·35)	-4·75 (-6·95 to -1·43)
	15-49 years	36200 (29900 to 47000)	256 (212 to 333)	-2·31 (-2·73 to -1·67)	-3·17 (-3·82 to -2·23)	4220 (3060 to 5880)	29·9 (21·7 to 41·7)	-2·66 (-4·17 to -1·43)	-3·17 (-5·00 to -0·951)
	50-69 years	13600 (10500 to 17100)	580 (449 to 731)	-1·76 (-2·35 to -1·15)	-2·61 (-3·60 to -1·75)	3720 (2600 to 5350)	159 (111 to 229)	-1·79 (-3·81 to -0·174)	-2·82 (-5·29 to -0·278)
	70+ years	3020 (2290 to 3800)	829 (628 to 1040)	-1·51 (-2·31 to -0·737)	-2·68 (-3·80 to -1·69)	1740 (1280 to 2430)	478 (352 to 669)	-1·44 (-2·98 to -0·0959)	-2·77 (-4·57 to -0·273)
Malawi	All Ages	68400 (59200 to 79600)	466 (408 to 540)	0·328 (0·0172 to 0·667)	-4·90 (-5·49 to -4·38)	14300 (10600 to 18000)	134 (96·3 to 170)	-0·635 (-1·36 to -0·0620)	-6·13 (-7·70 to -4·63)
	Under 5	4670 (3750 to 6020)	171 (138 to 221)	-0·0819 (-0·860 to 0·651)	-7·12 (-8·42 to -5·71)	654 (455 to 969)	24·0 (16·7 to 35·6)	-3·19 (-4·23 to -2·33)	-15·3 (-18·1 to -12·5)
	5-14 years	6470 (4540 to 8950)	120 (84·0 to 166)	1·62 (0·694 to 2·53)	-4·63 (-5·82 to -3·57)	506 (354 to 726)	9·37 (6·56 to 13·4)	5·14 (3·64 to 6·75)	-8·05 (-11·2 to -5·41)
	15-49 years	43400 (35600 to 54300)	448 (367 to 561)	0·389 (-0·0100 to 0·908)	-5·41 (-6·18 to -4·75)	7690 (5810 to 9720)	79·4 (60·0 to 100)	0·736 (-0·339 to 1·59)	-6·83 (-8·64 to -5·01)
	50-69 years	10600 (8090 to 13600)	793 (607 to 1020)	0·287 (-0·206 to 0·836)	-4·67 (-5·59 to -3·93)	3840 (2770 to 5180)	288 (208 to 389)	-0·468 (-1·88 to 0·715)	-3·42 (-5·66 to -1·25)

eTable 1. All-form tuberculosis incident cases and deaths, age-standardised rates of incidence and mortality per 100,000 population, and corresponding annualized rates of change by age groups for 204 countries and territories (2021).

Location	Age group	Number of cases	Rate per 100,000 cases	Annualized rate of change (1990-2010)	Annualized rate of change (2010-2021)	Number of deaths	Rate per 100,000 deaths	Annualized deaths (1990-2010)	Annualized deaths (2010-2021)
	70+ years	3340 (2600 to 4260)	1070 (836 to 1370)	0·204 (-0·363 to 0·784)	-4·17 (-5·06 to -3·26)	1660 (1180 to 2310)	534 (380 to 744)	-1·57 (-3·06 to 0·0411)	-3·39 (-5·71 to -1·15)
Mozambiqu e	All Ages	163000 (141000 to 189000)	735 (653 to 850)	0·738 (0·386 to 1·09)	-2·04 (-2·74 to -1·24)	35800 (28400 to 42500)	204 (159 to 245)	1·83 (0·713 to 2·95)	-5·29 (-7·02 to -4·04)
	Under 5	10700 (8750 to 13000)	207 (169 to 251)	-1·25 (-1·91 to -0·498)	-4·61 (-5·48 to -2·83)	2780 (2020 to 3630)	53·7 (39·0 to 70·1)	-2·23 (-3·59 to -0·904)	-10·5 (-12·8 to -7·90)
	5-14 years	12400 (8300 to 17700)	137 (91·3 to 195)	0·815 (-0·0540 to 1·81)	-1·55 (-2·76 to -0·528)	1460 (1150 to 1790)	16·1 (12·7 to 19·7)	3·72 (1·82 to 5·61)	-0·962 (-2·73 to 0·519)
	15-49 years	108000 (89000 to 136000)	758 (624 to 956)	1·63 (1·07 to 2·07)	-1·99 (-2·86 to -1·20)	20700 (16500 to 24100)	145 (115 to 169)	5·93 (4·67 to 7·25)	-4·85 (-6·50 to -3·64)
	50-69 years	24700 (19200 to 30400)	1170 (910 to 1440)	0·202 (-0·421 to 0·902)	-1·86 (-2·69 to -1·03)	7300 (5410 to 9420)	345 (256 to 445)	1·73 (0·235 to 3·13)	-5·09 (-7·38 to -3·07)
	70+ years	7130 (5740 to 8660)	1660 (1340 to 2010)	0·00886 (-0·722 to 0·741)	-1·80 (-2·72 to -0·549)	3540 (2440 to 4350)	823 (568 to 1010)	-0·455 (-1·81 to 0·715)	-3·35 (-6·41 to -1·23)
Rwanda	All Ages	25700 (22000 to 30400)	250 (222 to 285)	-2·33 (-2·79 to -1·85)	-3·45 (-4·13 to -2·77)	5130 (3850 to 6920)	68·2 (50·6 to 91·5)	-4·05 (-5·02 to -2·80)	-4·13 (-5·99 to -1·96)
	Under 5	1170 (917 to 1460)	66·6 (52·4 to 83·3)	-2·98 (-3·75 to -2·12)	-6·33 (-7·70 to -5·05)	203 (143 to 287)	11·6 (8·15 to 16·4)	-5·60 (-6·96 to -3·81)	-11·5 (-14·1 to -8·70)
	5-14 years	1200 (803 to 1750)	37·1 (24·9 to 54·2)	-1·47 (-2·38 to -0·750)	-7·08 (-8·47 to -5·83)	55·4 (38·2 to 75·8)	1·72 (1·19 to 2·35)	-0·132 (-2·00 to 1·76)	-14·5 (-17·2 to -12·1)
	15-49 years	16100 (13000 to 20800)	235 (189 to 303)	-2·39 (-2·96 to -1·65)	-4·01 (-4·92 to -3·27)	2220 (1690 to 3020)	32·4 (24·7 to 44·1)	-3·28 (-4·41 to -1·95)	-5·37 (-7·08 to -3·57)
	50-69 years	5560 (4200 to 6910)	463 (349 to 575)	-3·21 (-3·93 to -2·52)	-3·06 (-4·04 to -2·03)	1720 (1210 to 2480)	143 (101 to 206)	-5·12 (-6·45 to -3·77)	-3·27 (-5·55 to -0·0435)
	70+ years	1670 (1320 to 2090)	676 (536 to 845)	-2·93 (-3·96 to -1·96)	-2·81 (-3·92 to -1·81)	938 (670 to 1310)	380 (271 to 531)	-4·21 (-5·70 to -2·78)	-3·20 (-6·60 to -0·202)
Somalia	All Ages	79500 (68700 to 91700)	634 (552 to 740)	-0·380 (-0·681 to -0·0807)	-2·27 (-2·88 to -1·54)	18800 (11700 to 26400)	228 (135 to 331)	-0·182 (-1·19 to 0·987)	-3·25 (-4·80 to -1·53)
	Under 5	7340 (5610 to 9370)	178 (136 to 227)	-0·822 (-1·58 to -0·189)	-3·55 (-5·00 to -2·15)	2330 (1410 to 3640)	56·4 (34·3 to 88·1)	-1·21 (-2·77 to 0·464)	-6·59 (-9·93 to -3·81)
	5-14 years	5050 (3410 to 7130)	81·5 (54·9 to 115)	-0·724 (-1·47 to 0·0695)	-2·43 (-3·82 to -0·872)	332 (175 to 557)	5·36 (2·83 to 8·98)	2·16 (0·191 to 4·05)	-4·85 (-6·95 to -2·08)
	15-49 years	48500 (39700 to 60600)	487 (399 to 608)	-0·538 (-0·945 to -0·104)	-2·29 (-3·12 to -1·39)	8050 (4610 to 11600)	80·8 (46·3 to 117)	-0·0349 (-1·45 to 1·36)	-2·15 (-3·99 to 0·313)
	50-69 years	15300 (11600 to 19800)	1360 (1030 to 1760)	0·152 (-0·280 to 0·668)	-0·855 (-1·71 to 0·130)	5720 (3630 to 8280)	509 (323 to 736)	-0·203 (-1·47 to 1·30)	-1·82 (-3·74 to 0·293)
	70+ years	3350 (2560 to 4400)	1760 (1340 to 2310)	-0·109 (-0·834 to 0·419)	-1·50 (-2·58 to -0·402)	2390 (1340 to 3800)	1260 (702 to 2000)	-0·218 (-1·33 to 1·40)	-1·62 (-3·48 to 0·372)
South Sudan	All Ages	32800 (28300 to 37200)	482 (419 to 545)	-1·17 (-1·55 to -0·753)	-0·247 (-0·734 to 0·271)	6400 (4360 to 8980)	122 (84·3 to 169)	-1·71 (-3·09 to -0·560)	-0·218 (-2·33 to 2·25)
	Under 5	2880 (2280 to 3570)	184 (146 to 228)	-1·76 (-2·37 to -0·956)	-0·513 (-1·62 to 0·628)	997 (691 to 1500)	63·8 (44·2 to 96·1)	-3·37 (-5·34 to -1·45)	-0·908 (-4·18 to 2·18)

eTable 1. All-form tuberculosis incident cases and deaths, age-standardised rates of incidence and mortality per 100,000 population, and corresponding annualized rates of change by age groups for 204 countries and territories (2021).

Location	Age group	Number of cases	Rate per 100,000 cases	Annualized rate of change (1990-2010)	Annualized rate of change (2010-2021)	Number of deaths	Rate per 100,000 deaths	Annualized deaths (1990-2010)	Annualized deaths (2010-2021)
	5-14 years	2250 (1460 to 3190)	82·3 (53·6 to 117)	-1·24 (-1·88 to -0·529)	0·463 (-0·625 to 1·62)	122 (79·2 to 176)	4·48 (2·90 to 6·43)	0·837 (-1·29 to 2·74)	-0·132 (-3·02 to 3·27)
	15-49 years	19100 (15700 to 23700)	429 (353 to 533)	-0·711 (-1·25 to -0·0530)	-0·762 (-1·40 to -0·129)	2360 (1600 to 3380)	53·0 (36·0 to 76·0)	0·950 (-0·817 to 2·74)	-1·16 (-3·95 to 1·33)
	50-69 years	6940 (5260 to 8650)	879 (666 to 1100)	-1·12 (-1·83 to -0·473)	-0·662 (-1·38 to 0·0628)	1980 (1280 to 2840)	251 (162 to 360)	-1·82 (-3·84 to 0·146)	-0·119 (-2·52 to 2·39)
	70+ years	1680 (1350 to 2070)	1200 (969 to 1490)	-0·483 (-1·25 to 0·282)	-1·25 (-2·27 to 0·171)	942 (655 to 1420)	677 (471 to 1020)	-1·07 (-2·76 to 0·332)	-1·24 (-3·41 to 2·20)
Uganda	All Ages	123000 (106000 to 139000)	400 (359 to 446)	-1·99 (-2·46 to -1·61)	-3·94 (-4·76 to -3·08)	22100 (18200 to 27700)	100 (80·0 to 127)	-3·72 (-4·72 to -2·68)	-6·70 (-8·51 to -5·00)
	Under 5	9720 (7820 to 12100)	133 (107 to 166)	-2·94 (-3·61 to -2·16)	-5·63 (-6·66 to -4·17)	1790 (1170 to 2730)	24·4 (15·9 to 37·3)	-5·14 (-6·29 to -4·23)	-10·7 (-13·8 to -8·11)
	5-14 years	8410 (5720 to 11800)	67·2 (45·7 to 94·2)	0·162 (-0·996 to 1·07)	-7·04 (-8·70 to -5·13)	429 (303 to 587)	3·43 (2·42 to 4·69)	0·840 (-1·45 to 3·24)	-12·3 (-14·9 to -9·22)
	15-49 years	81600 (64500 to 96900)	406 (321 to 482)	-1·97 (-2·52 to -1·51)	-4·14 (-5·34 to -3·13)	11700 (9330 to 15300)	58·2 (46·4 to 75·9)	-3·61 (-4·69 to -2·32)	-7·73 (-9·74 to -5·75)
	50-69 years	17600 (14500 to 21300)	633 (522 to 768)	-1·76 (-2·28 to -1·26)	-3·62 (-4·68 to -2·72)	5400 (3820 to 7150)	195 (138 to 257)	-2·62 (-4·12 to -1·20)	-5·23 (-7·39 to -2·95)
	70+ years	6150 (5360 to 6860)	1020 (891 to 1140)	-1·06 (-1·40 to -0·652)	-2·17 (-3·45 to -1·32)	2830 (2130 to 3700)	470 (354 to 615)	-1·72 (-3·56 to -0·353)	-3·55 (-5·87 to -1·39)
United Republic of Tanzania	All Ages	114000 (99900 to 131000)	263 (233 to 296)	-0·948 (-1·44 to -0·490)	-6·74 (-7·34 to -6·09)	23700 (16700 to 32300)	70·2 (49·6 to 94·9)	-1·38 (-3·20 to -0·0556)	-7·14 (-9·07 to -5·12)
	Under 5	8550 (6890 to 10800)	96·7 (77·9 to 123)	-2·04 (-3·33 to -0·819)	-6·93 (-8·16 to -5·47)	1860 (1250 to 2520)	21·1 (14·1 to 28·5)	-3·88 (-5·86 to -2·51)	-10·5 (-13·3 to -8·07)
	5-14 years	7360 (4960 to 10800)	47·3 (31·9 to 69·7)	1·74 (0·345 to 3·48)	-7·72 (-8·85 to -6·61)	461 (300 to 673)	2·96 (1·93 to 4·33)	3·13 (0·644 to 5·09)	-10·6 (-12·7 to -8·57)
	15-49 years	68600 (56900 to 85700)	243 (202 to 304)	-1·13 (-1·69 to -0·581)	-7·52 (-8·38 to -6·75)	10800 (7210 to 15800)	38·4 (25·6 to 55·9)	-0·331 (-2·54 to 1·41)	-8·83 (-10·8 to -6·64)
	50-69 years	21000 (16100 to 26600)	444 (340 to 562)	-1·14 (-1·75 to -0·403)	-6·10 (-6·89 to -5·47)	6490 (4450 to 9230)	137 (94·0 to 195)	-1·54 (-3·45 to -0·0808)	-5·50 (-7·89 to -3·01)
	70+ years	8450 (6800 to 10500)	761 (612 to 942)	-0·513 (-1·10 to 0·143)	-4·86 (-5·86 to -4·05)	4070 (2830 to 5380)	367 (255 to 484)	-2·07 (-3·86 to -0·588)	-3·39 (-5·88 to -1·03)
Zambia	All Ages	74600 (63600 to 87100)	496 (436 to 571)	0·0218 (-0·429 to 0·378)	-3·59 (-4·15 to -3·00)	8680 (5580 to 12900)	78·8 (51·0 to 110)	-1·11 (-2·03 to -0·172)	-10·0 (-12·9 to -6·98)
	Under 5	4060 (3240 to 5240)	139 (111 to 179)	-1·08 (-1·73 to -0·298)	-7·43 (-9·03 to -5·84)	471 (280 to 712)	16·1 (9·57 to 24·3)	-3·49 (-4·87 to -2·38)	-17·1 (-20·8 to -13·2)
	5-14 years	5690 (3880 to 8170)	106 (72·6 to 153)	2·32 (1·57 to 3·36)	-5·18 (-6·48 to -3·79)	240 (142 to 376)	4·49 (2·65 to 7·04)	5·94 (3·85 to 8·02)	-13·4 (-17·5 to -8·76)
	15-49 years	52100 (42300 to 66200)	540 (437 to 686)	0·0525 (-0·546 to 0·591)	-3·57 (-4·31 to -2·94)	5130 (3060 to 7930)	53·1 (31·7 to 82·1)	0·0442 (-1·31 to 1·29)	-10·5 (-13·9 to -7·17)
	50-69 years	9980 (7630 to 12800)	760 (581 to 978)	-0·340 (-1·00 to 0·327)	-3·65 (-4·55 to -2·55)	2000 (1210 to 2900)	152 (92·5 to 221)	-1·63 (-3·00 to -0·256)	-7·88 (-11·1 to -4·95)

eTable 1. All-form tuberculosis incident cases and deaths, age-standardised rates of incidence and mortality per 100,000 population, and corresponding annualized rates of change by age groups for 204 countries and territories (2021).

Location	Age group	Number of cases	Rate per 100,000 cases	Annualized rate of change (1990-2010)	Annualized rate of change (2010-2021)	Number of deaths	Rate per 100,000 deaths	Annualized deaths (1990-2010)	Annualized deaths (2010-2021)
	70+ years	2720 (2090 to 3410)	1010 (776 to 1270)	-0·476 (-1·27 to 0·242)	-3·54 (-4·59 to -2·41)	837 (523 to 1200)	311 (195 to 444)	-2·45 (-3·92 to -1·01)	-7·34 (-9·75 to -4·60)
Southern Sub-Saharan Africa	All Ages	688000 (604000 to 776000)	847 (755 to 950)	2·61 (2·43 to 2·79)	-3·05 (-3·34 to -2·75)	95600 (84500 to 105000)	130 (116 to 143)	6·09 (5·27 to 6·79)	-7·06 (-7·79 to -6·25)
	Under 5	18000 (14900 to 22600)	225 (185 to 281)	-0·985 (-1·27 to -0·714)	-6·61 (-7·09 to -6·21)	2730 (2270 to 3420)	34·0 (28·2 to 42·6)	1·31 (0·263 to 2·05)	-11·0 (-12·5 to -8·93)
	5-14 years	29100 (20300 to 39500)	182 (127 to 246)	2·35 (1·92 to 2·81)	-6·50 (-7·17 to -5·87)	1460 (1240 to 1690)	9·13 (7·73 to 10·5)	7·48 (6·45 to 8·37)	-10·3 (-11·6 to -9·07)
	15-49 years	528000 (446000 to 611000)	1220 (1030 to 1410)	2·66 (2·44 to 2·91)	-2·78 (-3·12 to -2·48)	57000 (48700 to 62400)	132 (113 to 144)	7·03 (6·05 to 7·81)	-7·86 (-8·70 to -7·14)
	50-69 years	92600 (76300 to 109000)	891 (733 to 1050)	2·10 (1·88 to 2·34)	-3·27 (-3·68 to -2·86)	26900 (24300 to 30300)	258 (233 to 292)	4·94 (3·97 to 5·67)	-6·16 (-6·83 to -5·27)
	70+ years	20800 (17800 to 23600)	778 (667 to 885)	1·93 (1·74 to 2·15)	-2·97 (-3·35 to -2·63)	7530 (6750 to 8540)	282 (253 to 320)	2·30 (1·01 to 3·19)	-5·11 (-5·90 to -3·78)
Botswana	All Ages	14600 (12400 to 17100)	606 (524 to 702)	3·60 (3·09 to 3·98)	-4·58 (-5·55 to -3·75)	2660 (1850 to 3360)	127 (89·0 to 159)	3·52 (1·99 to 4·92)	-6·07 (-7·86 to -4·26)
	Under 5	427 (341 to 538)	181 (145 to 228)	1·82 (1·25 to 2·34)	-5·19 (-6·36 to -3·95)	52·0 (29·2 to 78·1)	22·1 (12·4 to 33·2)	-1·12 (-2·77 to 0·140)	-8·51 (-12·1 to -4·67)
	5-14 years	514 (361 to 723)	111 (78·0 to 156)	4·52 (3·64 to 5·10)	-8·45 (-9·78 to -7·02)	17·5 (12·5 to 23·9)	3·78 (2·69 to 5·16)	5·69 (3·74 to 8·18)	-17·5 (-19·6 to -14·9)
	15-49 years	11000 (9020 to 13600)	810 (663 to 997)	3·01 (2·43 to 3·49)	-4·99 (-6·11 to -3·99)	1590 (1080 to 2030)	117 (79·4 to 149)	4·19 (2·36 to 5·64)	-7·54 (-9·51 to -5·63)
	50-69 years	2290 (1710 to 2900)	826 (619 to 1050)	2·68 (2·07 to 3·41)	-3·79 (-4·69 to -2·97)	833 (565 to 1110)	301 (204 to 401)	1·25 (-0·723 to 3·22)	-4·59 (-6·70 to -2·37)
	70+ years	358 (269 to 498)	612 (460 to 851)	0·468 (-0·272 to 1·13)	-3·37 (-4·55 to -1·79)	172 (125 to 217)	293 (213 to 370)	-0·914 (-2·68 to 0·896)	-4·53 (-6·27 to -2·35)
Eswatini	All Ages	9310 (7950 to 11100)	852 (748 to 1010)	3·50 (3·11 to 3·92)	-4·25 (-4·99 to -3·43)	1960 (1610 to 2330)	218 (178 to 262)	9·39 (7·34 to 11·3)	-7·72 (-9·64 to -6·02)
	Under 5	360 (285 to 454)	256 (203 to 323)	2·14 (1·52 to 2·88)	-6·85 (-7·96 to -5·53)	60·2 (46·4 to 82·5)	42·9 (33·1 to 58·8)	8·08 (6·42 to 10·2)	-16·4 (-18·4 to -14·2)
	5-14 years	679 (459 to 959)	250 (169 to 352)	3·12 (2·34 to 3·70)	-3·70 (-4·94 to -2·61)	47·6 (33·5 to 61·0)	17·5 (12·3 to 22·4)	8·21 (5·92 to 10·2)	-8·69 (-11·7 to -6·01)
	15-49 years	6990 (5750 to 8870)	1130 (933 to 1440)	3·31 (2·75 to 3·83)	-4·63 (-5·47 to -3·74)	1310 (1080 to 1570)	212 (176 to 254)	11·7 (9·61 to 13·8)	-8·05 (-10·3 to -5·89)
	50-69 years	1090 (822 to 1390)	1050 (795 to 1340)	2·25 (1·70 to 2·71)	-4·41 (-5·40 to -3·17)	452 (310 to 633)	437 (300 to 611)	5·58 (3·52 to 7·44)	-7·00 (-9·55 to -3·96)
	70+ years	181 (130 to 265)	806 (576 to 1180)	1·99 (0·914 to 2·79)	-4·14 (-5·75 to -2·25)	93·1 (66·7 to 123)	414 (297 to 547)	2·80 (0·983 to 4·72)	-6·04 (-8·57 to -3·74)
Lesotho	All Ages	19700 (17400 to 22700)	1150 (1020 to 1290)	3·02 (2·70 to 3·27)	-2·41 (-3·01 to -1·84)	6010 (5140 to 6880)	411 (345 to 465)	7·20 (5·55 to 8·75)	-2·37 (-3·63 to -1·25)
	Under 5	978 (808 to 1200)	480 (397 to 589)	1·04 (0·365 to 1·69)	-1·09 (-1·98 to -0·156)	355 (269 to 443)	174 (132 to 217)	5·38 (3·95 to 6·86)	-1·35 (-2·84 to 0·746)

eTable 1. All-form tuberculosis incident cases and deaths, age-standardised rates of incidence and mortality per 100,000 population, and corresponding annualized rates of change by age groups for 204 countries and territories (2021).

Location	Age group	Number of cases	Rate per 100,000 cases	Annualized rate of change (1990-2010)	Annualized rate of change (2010-2021)	Number of deaths	Rate per 100,000 deaths	Annualized deaths (1990-2010)	Annualized deaths (2010-2021)
	5-14 years	1440 (977 to 2020)	338 (229 to 474)	2·47 (1·65 to 3·21)	-1·40 (-2·56 to -0·330)	129 (99·6 to 158)	30·3 (23·3 to 37·1)	7·68 (6·04 to 9·34)	-3·75 (-5·77 to -2·15)
	15-49 years	13300 (11000 to 16700)	1330 (1100 to 1660)	2·74 (2·40 to 3·13)	-3·20 (-3·89 to -2·48)	3370 (2870 to 3970)	335 (286 to 395)	8·91 (7·49 to 10·5)	-3·19 (-4·59 to -1·63)
	50-69 years	3280 (2540 to 4090)	1710 (1330 to 2130)	3·05 (2·48 to 3·55)	-2·10 (-2·93 to -1·12)	1760 (1330 to 2290)	917 (694 to 1200)	5·50 (3·44 to 7·12)	-1·56 (-3·31 to 0·297)
	70+ years	694 (502 to 975)	1440 (1040 to 2030)	2·56 (1·79 to 3·19)	-1·53 (-2·97 to 0·555)	401 (311 to 502)	834 (646 to 1050)	3·04 (1·16 to 4·38)	-2·17 (-4·01 to -0·465)
Namibia	All Ages	13300 (11500 to 15400)	582 (511 to 664)	0·780 (0·378 to 1·32)	-4·07 (-4·81 to -3·49)	2080 (1560 to 2660)	112 (85·3 to 141)	3·20 (1·80 to 4·59)	-5·16 (-6·93 to -3·27)
	Under 5	539 (446 to 689)	194 (160 to 247)	-0·618 (-1·40 to 0·416)	-5·70 (-6·77 to -4·22)	56·9 (41·8 to 78·8)	20·4 (15·0 to 28·3)	2·89 (1·54 to 4·28)	-13·9 (-16·4 to -10·8)
	5-14 years	811 (579 to 1130)	148 (106 to 207)	1·18 (0·366 to 2·13)	-5·12 (-6·61 to -3·59)	37·3 (27·7 to 52·2)	6·82 (5·06 to 9·55)	5·00 (3·34 to 6·73)	-10·5 (-12·9 to -7·83)
	15-49 years	9550 (8130 to 11900)	740 (630 to 924)	0·697 (0·137 to 1·31)	-4·41 (-5·34 to -3·68)	1120 (827 to 1440)	86·5 (64·1 to 111)	5·06 (3·63 to 6·50)	-6·08 (-8·03 to -4·07)
	50-69 years	2060 (1540 to 2580)	808 (603 to 1010)	-0·0680 (-0·607 to 0·430)	-3·76 (-4·76 to -2·76)	681 (511 to 881)	267 (200 to 345)	0·672 (-0·734 to 2·26)	-3·75 (-5·58 to -1·66)
	70+ years	376 (295 to 476)	628 (493 to 795)	-0·421 (-1·00 to 0·243)	-3·48 (-4·57 to -2·47)	185 (136 to 255)	310 (228 to 426)	-0·503 (-1·81 to 1·22)	-4·24 (-6·35 to -1·54)
South Africa	All Ages	546000 (475000 to 619000)	912 (800 to 1030)	3·53 (3·29 to 3·74)	-2·49 (-2·82 to -2·15)	62800 (53300 to 68200)	111 (95·3 to 120)	7·36 (6·47 to 8·13)	-7·56 (-8·38 to -6·80)
	Under 5	11000 (8820 to 13700)	222 (178 to 277)	-1·27 (-1·56 to -0·978)	-7·16 (-7·64 to -6·62)	1250 (1020 to 1550)	25·1 (20·6 to 31·2)	1·81 (0·279 to 3·12)	-11·1 (-12·6 to -9·39)
	5-14 years	18400 (12800 to 24300)	180 (125 to 237)	2·27 (1·77 to 2·76)	-7·01 (-7·74 to -6·25)	697 (609 to 785)	6·81 (5·94 to 7·66)	6·67 (5·39 to 7·72)	-11·7 (-12·8 to -10·5)
	15-49 years	430000 (359000 to 497000)	1380 (1150 to 1600)	3·96 (3·76 to 4·19)	-2·05 (-2·49 to -1·69)	38800 (31500 to 42600)	125 (101 to 137)	8·34 (7·50 to 9·08)	-8·32 (-9·31 to -7·61)
	50-69 years	70600 (59000 to 83000)	852 (712 to 1000)	2·76 (2·49 to 3·00)	-3·11 (-3·53 to -2·53)	17200 (15400 to 18700)	207 (186 to 226)	6·02 (5·04 to 6·90)	-6·90 (-7·56 to -6·00)
	70+ years	16200 (14200 to 17900)	734 (642 to 811)	2·35 (2·13 to 2·59)	-2·82 (-3·22 to -2·39)	4880 (4480 to 5530)	221 (202 to 250)	2·88 (1·49 to 3·91)	-5·53 (-6·30 to -4·30)
Zimbabwe	All Ages	85400 (74300 to 97100)	661 (583 to 752)	-0·246 (-0·654 to 0·170)	-5·47 (-6·07 to -4·69)	20000 (15500 to 24400)	202 (148 to 248)	3·47 (2·35 to 4·56)	-6·73 (-8·40 to -5·35)
	Under 5	4710 (3860 to 5980)	213 (175 to 271)	-0·723 (-1·24 to -0·195)	-6·20 (-7·68 to -5·16)	956 (686 to 1320)	43·3 (31·0 to 59·8)	-0·0749 (-0·900 to 0·570)	-12·6 (-15·3 to -9·83)
	5-14 years	7260 (5020 to 9920)	178 (123 to 243)	2·54 (1·80 to 3·41)	-6·09 (-7·27 to -4·85)	535 (386 to 679)	13·1 (9·45 to 16·6)	10·2 (8·92 to 11·8)	-9·34 (-11·4 to -7·30)
	15-49 years	57200 (47900 to 69900)	739 (619 to 902)	-1·03 (-1·58 to -0·539)	-6·00 (-6·86 to -5·20)	10800 (8730 to 13000)	139 (113 to 168)	3·82 (2·51 to 5·30)	-7·51 (-9·51 to -5·95)
	50-69 years	13300 (9550 to 17300)	1030 (740 to 1340)	0·467 (-0·145 to 0·975)	-3·73 (-4·65 to -2·61)	5960 (4060 to 7750)	462 (314 to 600)	3·88 (2·12 to 5·45)	-4·82 (-6·55 to -2·79)

eTable 1. All-form tuberculosis incident cases and deaths, age-standardised rates of incidence and mortality per 100,000 population, and corresponding annualized rates of change by age groups for 204 countries and territories (2021).

Location	Age group	Number of cases	Rate per 100,000 cases	Annualized rate of change (1990-2010)	Annualized rate of change (2010-2021)	Number of deaths	Rate per 100,000 deaths	Annualized deaths (1990-2010)	Annualized deaths (2010-2021)
	70+ years	2900 (2220 to 3750)	1090 (836 to 1410)	1·36 (0·673 to 2·02)	-3·55 (-4·70 to -2·16)	1800 (1200 to 2270)	676 (451 to 856)	2·42 (0·204 to 3·69)	-4·04 (-6·10 to -1·92)
Western Sub-Saharan Africa	All Ages	722000 (640000 to 803000)	211 (188 to 239)	-0·985 (-1·23 to -0·634)	-4·90 (-5·29 to -4·55)	132000 (102000 to 163000)	52·4 (42·1 to 63·8)	-1·72 (-2·51 to -0·827)	-5·46 (-6·87 to -4·03)
	Under 5	82400 (67100 to 102000)	103 (84·0 to 128)	-2·37 (-2·81 to -1·97)	-5·89 (-6·49 to -5·13)	18600 (11700 to 27300)	23·3 (14·6 to 34·1)	-2·96 (-3·68 to -2·03)	-7·37 (-10·0 to -5·11)
	5-14 years	57800 (40700 to 81800)	42·9 (30·2 to 60·7)	-1·16 (-1·57 to -0·657)	-4·71 (-5·33 to -4·15)	3810 (2810 to 4850)	2·82 (2·08 to 3·60)	0·0509 (-1·05 to 1·38)	-6·48 (-8·18 to -5·01)
	15-49 years	382000 (320000 to 474000)	167 (139 to 207)	-0·300 (-0·658 to 0·124)	-5·13 (-5·52 to -4·70)	46800 (35400 to 61000)	20·4 (15·5 to 26·6)	-0·0892 (-1·20 to 1·10)	-6·19 (-7·42 to -4·80)
	50-69 years	147000 (111000 to 183000)	390 (296 to 488)	-0·897 (-1·25 to -0·621)	-4·35 (-4·96 to -3·57)	36400 (28100 to 45100)	96·7 (74·7 to 120)	-1·78 (-2·74 to -0·500)	-4·31 (-5·73 to -2·51)
	70+ years	53400 (43000 to 66500)	655 (528 to 815)	-1·10 (-1·46 to -0·688)	-3·86 (-4·39 to -3·39)	26900 (22100 to 32300)	330 (271 to 397)	-1·77 (-2·57 to -0·853)	-3·60 (-4·80 to -1·90)
Benin	All Ages	12700 (11100 to 14100)	137 (122 to 154)	-1·30 (-1·70 to -0·860)	-4·15 (-4·88 to -3·56)	2200 (1560 to 3100)	32·8 (24·2 to 44·8)	-2·65 (-3·82 to -1·61)	-4·55 (-6·55 to -2·64)
	Under 5	1660 (1340 to 2100)	70·7 (57·4 to 89·8)	-2·87 (-3·57 to -1·97)	-5·23 (-6·71 to -3·83)	267 (134 to 594)	11·4 (5·74 to 25·4)	-3·62 (-5·18 to -1·76)	-8·73 (-12·8 to -5·35)
	5-14 years	1060 (693 to 1480)	28·4 (18·5 to 39·7)	-1·13 (-2·41 to 0·596)	-4·43 (-5·90 to -2·99)	62·2 (38·6 to 100)	1·66 (1·03 to 2·68)	-0·506 (-2·31 to 1·33)	-6·97 (-9·23 to -5·10)
	15-49 years	6590 (5440 to 8210)	105 (87·0 to 131)	-0·714 (-1·31 to -0·191)	-4·40 (-5·10 to -3·67)	809 (578 to 1120)	12·9 (9·24 to 17·9)	-0·694 (-2·39 to 0·704)	-4·01 (-6·00 to -2·19)
	50-69 years	2490 (1850 to 3200)	264 (196 to 339)	-1·11 (-1·66 to -0·612)	-3·57 (-4·79 to -2·48)	593 (414 to 877)	62·9 (43·9 to 93·0)	-2·98 (-4·68 to -1·87)	-3·46 (-5·87 to -0·107)
	70+ years	935 (741 to 1150)	418 (331 to 514)	-1·36 (-2·04 to -0·669)	-3·61 (-4·64 to -2·74)	467 (356 to 621)	209 (159 to 278)	-2·85 (-3·93 to -1·48)	-3·75 (-6·02 to -1·56)
Burkina Faso	All Ages	30900 (27200 to 34600)	198 (174 to 224)	-2·17 (-2·47 to -1·74)	-4·86 (-5·44 to -4·30)	6510 (4980 to 7970)	55·6 (43·9 to 67·7)	-3·34 (-4·41 to -2·19)	-4·31 (-6·43 to -2·77)
	Under 5	4600 (3670 to 5680)	112 (89·3 to 138)	-3·14 (-3·66 to -2·41)	-5·13 (-6·26 to -3·90)	973 (612 to 1570)	23·7 (14·9 to 38·3)	-4·42 (-5·73 to -2·95)	-5·68 (-8·48 to -2·33)
	5-14 years	2140 (1460 to 3040)	34·1 (23·3 to 48·5)	-1·56 (-2·22 to -0·704)	-5·65 (-6·82 to -4·20)	171 (127 to 214)	2·73 (2·03 to 3·41)	0·329 (-1·08 to 2·23)	-6·54 (-8·97 to -4·08)
	15-49 years	15100 (12500 to 19000)	147 (122 to 185)	-2·39 (-2·88 to -1·75)	-5·57 (-6·32 to -4·88)	2090 (1520 to 2650)	20·3 (14·7 to 25·7)	-4·24 (-5·50 to -2·87)	-5·72 (-7·93 to -3·91)
	50-69 years	6430 (4970 to 8030)	387 (300 to 484)	-1·16 (-1·76 to -0·619)	-3·82 (-4·66 to -2·75)	1850 (1420 to 2330)	112 (85·4 to 140)	-2·08 (-3·47 to -0·472)	-2·95 (-5·07 to -0·943)
	70+ years	2670 (2110 to 3280)	634 (502 to 779)	-1·17 (-1·89 to -0·355)	-3·35 (-4·36 to -2·36)	1420 (1090 to 1800)	337 (260 to 427)	-1·37 (-2·57 to -0·0701)	-2·71 (-5·07 to -0·727)
Cabo Verde	All Ages	472 (415 to 542)	90·4 (79·3 to 102)	-2·53 (-3·00 to -2·13)	-2·98 (-3·60 to -2·40)	64·5 (44·0 to 114)	13·9 (9·54 to 24·4)	-3·43 (-4·69 to -2·10)	-2·18 (-4·15 to 0·382)
	Under 5	18·0 (14·3 to 22·9)	40·8 (32·4 to 52·0)	-4·64 (-5·40 to -3·73)	-5·53 (-6·80 to -4·38)	0·522 (0·342 to 0·935)	1·18 (0·777 to 2·12)	-8·28 (-10·2 to -5·78)	-10·9 (-14·0 to -8·10)

eTable 1. All-form tuberculosis incident cases and deaths, age-standardised rates of incidence and mortality per 100,000 population, and corresponding annualized rates of change by age groups for 204 countries and territories (2021).

Location	Age group	Number of cases	Rate per 100,000 cases	Annualized rate of change (1990-2010)	Annualized rate of change (2010-2021)	Number of deaths	Rate per 100,000 deaths	Annualized deaths (1990-2010)	Annualized deaths (2010-2021)
	5-14 years	18·3 (12·4 to 26·2)	18·4 (12·5 to 26·4)	-2·68 (-3·34 to -1·80)	-4·57 (-5·68 to -3·28)	0·429 (0·300 to 0·657)	0·432 (0·303 to 0·663)	-1·55 (-3·66 to 0·795)	-7·99 (-10·1 to -6·10)
	15-49 years	224 (183 to 277)	71·4 (58·3 to 88·3)	-2·70 (-3·41 to -2·12)	-4·72 (-5·36 to -3·99)	16·4 (10·5 to 28·7)	5·22 (3·36 to 9·15)	-2·55 (-3·74 to -0·770)	-6·08 (-8·37 to -3·81)
	50-69 years	152 (115 to 197)	186 (140 to 241)	-2·29 (-2·87 to -1·65)	-2·22 (-3·22 to -1·14)	22·4 (14·5 to 42·6)	27·4 (17·7 to 52·2)	-3·66 (-5·37 to -2·05)	-1·69 (-4·61 to 1·91)
	70+ years	60·2 (49·6 to 74·1)	294 (242 to 362)	-2·53 (-3·06 to -2·05)	-2·09 (-3·03 to -0·876)	24·8 (17·5 to 41·5)	121 (85·4 to 203)	-3·92 (-5·39 to -2·74)	-0·268 (-2·34 to 2·40)
Cameroon	All Ages	45800 (40300 to 52100)	198 (175 to 224)	2·02 (1·57 to 2·49)	-6·59 (-7·32 to -5·86)	8420 (5060 to 12900)	48·2 (28·3 to 72·9)	1·52 (-0·396 to 3·10)	-8·07 (-10·4 to -5·51)
	Under 5	4780 (3840 to 6000)	98·3 (79·0 to 123)	0·571 (-0·111 to 1·28)	-7·21 (-8·59 to -5·60)	676 (439 to 1160)	13·9 (9·04 to 23·8)	1·32 (-0·394 to 2·87)	-12·5 (-15·0 to -9·59)
	5-14 years	3910 (2720 to 5450)	45·5 (31·7 to 63·4)	1·92 (1·27 to 2·73)	-5·65 (-7·23 to -4·21)	246 (160 to 371)	2·86 (1·86 to 4·32)	3·97 (1·92 to 6·33)	-8·60 (-10·2 to -6·39)
	15-49 years	25900 (21600 to 31100)	168 (140 to 202)	2·60 (1·99 to 3·21)	-7·16 (-7·86 to -6·42)	4070 (2440 to 6610)	26·4 (15·8 to 42·9)	3·75 (1·70 to 5·64)	-8·97 (-11·1 to -6·45)
	50-69 years	8460 (6330 to 11000)	354 (265 to 461)	1·97 (1·42 to 2·47)	-5·76 (-7·01 to -4·48)	2260 (1280 to 3590)	94·7 (53·7 to 151)	0·322 (-1·78 to 2·07)	-5·98 (-8·07 to -3·05)
	70+ years	2690 (2110 to 3360)	538 (422 to 671)	1·06 (0·205 to 1·89)	-5·33 (-6·39 to -4·33)	1170 (750 to 1730)	234 (150 to 346)	-1·77 (-3·47 to 0·0121)	-4·92 (-7·69 to -2·70)
Chad	All Ages	33200 (29300 to 37100)	286 (256 to 323)	-1·27 (-1·66 to -0·939)	-3·36 (-3·95 to -2·56)	7440 (5620 to 9670)	90·3 (66·4 to 117)	-0·876 (-2·01 to 0·357)	-4·29 (-6·55 to -2·62)
	Under 5	5580 (4300 to 6950)	154 (118 to 191)	-2·52 (-3·21 to -1·89)	-3·85 (-5·17 to -2·39)	1390 (930 to 1930)	38·1 (25·6 to 53·1)	-0·974 (-2·53 to 0·600)	-5·99 (-9·15 to -2·99)
	5-14 years	3780 (2580 to 5100)	70·3 (47·9 to 94·8)	-1·04 (-1·90 to -0·197)	-2·55 (-3·72 to -1·15)	291 (192 to 383)	5·41 (3·57 to 7·12)	1·62 (-0·186 to 3·40)	-4·83 (-7·80 to -2·61)
	15-49 years	15900 (13200 to 20100)	214 (179 to 270)	-0·365 (-0·951 to 0·0667)	-3·43 (-4·24 to -2·51)	2560 (1880 to 3540)	34·5 (25·4 to 47·7)	0·914 (-0·541 to 2·22)	-3·93 (-5·91 to -1·82)
	50-69 years	5860 (4580 to 7250)	543 (424 to 672)	-0·618 (-1·14 to -0·0335)	-2·76 (-3·73 to -1·60)	1960 (1400 to 2600)	181 (129 to 241)	-0·369 (-1·59 to 1·10)	-2·86 (-5·22 to -0·840)
	70+ years	2040 (1640 to 2470)	855 (690 to 1040)	-0·801 (-1·54 to -0·0920)	-2·87 (-3·89 to -1·21)	1240 (856 to 1590)	522 (359 to 668)	-0·912 (-2·38 to 0·532)	-2·72 (-5·44 to -0·678)
Côte d'Ivoire	All Ages	40500 (35500 to 45900)	197 (174 to 225)	-1·62 (-2·00 to -1·05)	-5·19 (-5·78 to -4·61)	6680 (4490 to 9510)	45·2 (31·6 to 64·6)	-1·35 (-2·48 to -0·195)	-7·31 (-9·37 to -5·12)
	Under 5	4290 (3510 to 5410)	98·6 (80·5 to 124)	-3·32 (-3·95 to -2·55)	-5·73 (-6·95 to -4·46)	544 (347 to 920)	12·5 (7·97 to 21·1)	-3·30 (-4·41 to -1·97)	-10·8 (-14·0 to -7·78)
	5-14 years	3610 (2510 to 5150)	50·0 (34·7 to 71·3)	-0·826 (-1·60 to -0·0307)	-4·23 (-5·39 to -3·07)	212 (147 to 312)	2·93 (2·03 to 4·33)	2·34 (0·403 to 4·31)	-7·13 (-8·85 to -5·19)
	15-49 years	22500 (18800 to 27400)	165 (137 to 200)	-1·73 (-2·25 to -1·08)	-5·71 (-6·40 to -5·00)	2910 (1950 to 4160)	21·2 (14·2 to 30·3)	-1·21 (-2·25 to 0·189)	-8·73 (-11·1 to -6·49)
	50-69 years	7830 (5960 to 10000)	359 (273 to 460)	-1·38 (-2·07 to -0·928)	-4·59 (-5·58 to -3·44)	1970 (1260 to 2930)	90·3 (57·8 to 134)	-1·57 (-3·17 to -0·00266)	-5·75 (-7·98 to -3·58)
	70+ years	2250 (1800 to 2820)	540 (431 to 676)	-1·35 (-2·09 to -0·570)	-4·16 (-5·07 to -3·15)	1050 (765 to 1520)	252 (183 to 364)	-1·96 (-3·37 to -0·428)	-4·65 (-7·27 to -2·31)

eTable 1. All-form tuberculosis incident cases and deaths, age-standardised rates of incidence and mortality per 100,000 population, and corresponding annualized rates of change by age groups for 204 countries and territories (2021).

Location	Age group	Number of cases	Rate per 100,000 cases	Annualized rate of change (1990-2010)	Annualized rate of change (2010-2021)	Number of deaths	Rate per 100,000 deaths	Annualized deaths (1990-2010)	Annualized deaths (2010-2021)
Gambia	All Ages	4590 (3980 to 5260)	267 (235 to 305)	-1·28 (-1·78 to -0·879)	-3·30 (-4·12 to -2·45)	753 (526 to 1140)	61·2 (42·7 to 94·4)	-0·0323 (-1·45 to 1·20)	-3·02 (-4·64 to -1·25)
	Under 5	533 (413 to 678)	149 (116 to 190)	-2·99 (-3·77 to -2·18)	-4·09 (-5·89 to -2·12)	38·9 (25·4 to 61·9)	10·9 (7·10 to 17·3)	-2·48 (-4·03 to -0·755)	-7·11 (-9·67 to -4·07)
	5-14 years	337 (226 to 490)	53·1 (35·6 to 77·1)	-2·15 (-3·79 to -0·810)	-2·21 (-4·54 to 0·542)	15·3 (10·2 to 22·8)	2·41 (1·60 to 3·59)	-0·163 (-1·65 to 1·49)	-3·98 (-6·74 to -1·84)
	15-49 years	2490 (2020 to 3100)	210 (170 to 262)	-0·940 (-1·45 to -0·430)	-3·51 (-4·47 to -2·72)	333 (219 to 508)	28·1 (18·4 to 42·8)	1·47 (-0·152 to 3·06)	-3·26 (-5·03 to -1·27)
	50-69 years	882 (653 to 1130)	519 (385 to 667)	-1·00 (-1·64 to -0·386)	-3·11 (-4·03 to -2·26)	206 (136 to 329)	121 (80·1 to 194)	-1·15 (-2·80 to 0·418)	-2·39 (-4·51 to -0·499)
	70+ years	339 (269 to 427)	757 (600 to 952)	-1·38 (-1·94 to -0·866)	-2·85 (-3·93 to -1·78)	160 (106 to 246)	358 (237 to 549)	-1·83 (-3·04 to -0·284)	-1·66 (-3·99 to 0·460)
Ghana	All Ages	63700 (55400 to 72800)	244 (214 to 279)	-0·471 (-0·960 to 0·0372)	-3·94 (-4·56 to -3·22)	12100 (9210 to 16100)	61·1 (46·8 to 81·4)	0·123 (-1·36 to 1·44)	-4·84 (-6·64 to -2·88)
	Under 5	3870 (3110 to 4810)	83·4 (67·0 to 104)	-2·95 (-3·72 to -1·89)	-5·94 (-7·69 to -4·38)	341 (220 to 686)	7·35 (4·75 to 14·8)	-1·60 (-3·31 to -0·171)	-13·1 (-16·1 to -9·46)
	5-14 years	4120 (2820 to 5730)	50·0 (34·2 to 69·6)	-1·04 (-2·33 to 0·234)	-4·01 (-6·02 to -1·92)	204 (151 to 288)	2·47 (1·83 to 3·49)	2·12 (0·0986 to 4·18)	-8·06 (-10·3 to -6·33)
	15-49 years	36200 (30200 to 45200)	206 (172 to 258)	-0·272 (-0·919 to 0·336)	-4·44 (-5·25 to -3·53)	5320 (4030 to 7250)	30·3 (23·0 to 41·3)	1·16 (-0·357 to 2·74)	-5·72 (-7·49 to -3·99)
	50-69 years	14600 (11300 to 18300)	465 (359 to 581)	-0·491 (-1·07 to -0·0171)	-3·27 (-4·59 to -2·14)	3760 (2780 to 5310)	120 (88·4 to 169)	-0·954 (-3·05 to 0·831)	-4·05 (-6·83 to -1·54)
	70+ years	4960 (4000 to 6160)	720 (581 to 895)	-0·758 (-1·44 to -0·0425)	-2·86 (-4·08 to -1·67)	2450 (1740 to 3350)	356 (253 to 486)	-1·89 (-3·32 to -0·159)	-2·85 (-5·38 to -0·163)
Guinea	All Ages	19400 (17200 to 21700)	207 (182 to 236)	0·234 (-0·199 to 0·684)	-4·32 (-4·93 to -3·66)	4260 (3130 to 5740)	60·0 (44·4 to 79·8)	-0·670 (-1·73 to 0·531)	-5·19 (-7·03 to -2·88)
	Under 5	2270 (1770 to 2830)	101 (78·9 to 126)	-1·79 (-2·49 to -1·11)	-5·58 (-6·70 to -4·22)	390 (230 to 707)	17·3 (10·2 to 31·4)	-2·89 (-4·25 to -0·963)	-9·55 (-12·3 to -6·06)
	5-14 years	1440 (1020 to 2080)	38·1 (26·9 to 54·9)	-0·847 (-1·58 to -0·0958)	-4·07 (-5·33 to -2·85)	119 (82·2 to 167)	3·14 (2·17 to 4·40)	0·711 (-0·951 to 2·46)	-6·22 (-8·60 to -4·02)
	15-49 years	10100 (8400 to 12600)	164 (137 to 206)	1·35 (0·837 to 1·90)	-4·16 (-5·01 to -3·41)	1650 (1230 to 2120)	27·0 (20·1 to 34·7)	1·89 (0·751 to 3·44)	-4·26 (-6·13 to -2·10)
	50-69 years	3940 (3050 to 4870)	397 (307 to 491)	0·726 (0·256 to 1·21)	-3·13 (-4·04 to -2·14)	1190 (846 to 1600)	120 (85·2 to 162)	-0·407 (-1·82 to 1·11)	-3·16 (-5·09 to -0·773)
	70+ years	1660 (1340 to 1980)	639 (516 to 763)	0·541 (-0·298 to 1·29)	-3·43 (-4·44 to -2·23)	909 (650 to 1270)	350 (250 to 491)	-0·713 (-2·25 to 1·17)	-3·51 (-5·80 to -0·536)
Guinea-Bissau	All Ages	4860 (4300 to 5560)	332 (294 to 379)	-0·874 (-1·29 to -0·339)	-2·25 (-2·87 to -1·57)	1390 (1020 to 1810)	127 (99·7 to 158)	-0·837 (-1·93 to 0·713)	-2·59 (-4·57 to -0·648)
	Under 5	514 (418 to 632)	155 (126 to 190)	-2·53 (-3·27 to -1·79)	-4·10 (-5·35 to -3·02)	132 (64·5 to 194)	39·6 (19·4 to 58·4)	-1·59 (-3·22 to 0·255)	-6·27 (-11·2 to -2·81)
	5-14 years	396 (276 to 562)	69·9 (48·9 to 99·4)	-1·59 (-2·36 to -0·725)	-2·50 (-3·65 to -1·26)	34·9 (22·4 to 47·9)	6·17 (3·95 to 8·47)	1·07 (-0·975 to 3·37)	-5·69 (-8·49 to -2·17)
	15-49 years	2910 (2420 to 3590)	291 (242 to 359)	-0·132 (-0·743 to 0·486)	-1·70 (-2·57 to -0·746)	728 (480 to 1040)	72·8 (48·0 to 103)	0·582 (-1·04 to 2·75)	-1·36 (-3·56 to 0·611)

eTable 1. All-form tuberculosis incident cases and deaths, age-standardised rates of incidence and mortality per 100,000 population, and corresponding annualized rates of change by age groups for 204 countries and territories (2021).

Location	Age group	Number of cases	Rate per 100,000 cases	Annualized rate of change (1990-2010)	Annualized rate of change (2010-2021)	Number of deaths	Rate per 100,000 deaths	Annualized deaths (1990-2010)	Annualized deaths (2010-2021)
	50-69 years	826 (640 to 1030)	589 (456 to 733)	-0·850 (-1·42 to -0·368)	-2·64 (-3·62 to -1·24)	353 (256 to 437)	252 (183 to 311)	-1·47 (-2·95 to 0·167)	-2·66 (-4·92 to -0·580)
	70+ years	209 (166 to 257)	823 (655 to 1010)	-1·38 (-2·12 to -0·599)	-3·01 (-4·09 to -1·52)	142 (111 to 171)	558 (439 to 674)	-1·83 (-3·36 to -0·476)	-3·08 (-5·41 to -0·678)
Liberia	All Ages	6280 (5510 to 7070)	162 (145 to 182)	-3·55 (-4·05 to -3·04)	-3·47 (-3·99 to -2·93)	907 (575 to 1580)	34·1 (21·6 to 59·6)	-4·09 (-6·00 to -2·37)	-4·54 (-6·69 to -2·29)
	Under 5	599 (480 to 755)	78·2 (62·6 to 98·5)	-5·55 (-6·23 to -4·79)	-4·48 (-5·64 to -3·56)	63·8 (34·0 to 125)	8·33 (4·43 to 16·3)	-8·36 (-10·3 to -5·66)	-8·39 (-12·8 to -3·87)
	5-14 years	449 (309 to 622)	31·6 (21·8 to 43·8)	-4·77 (-5·62 to -3·94)	-3·39 (-4·53 to -1·89)	23·6 (15·9 to 43·4)	1·66 (1·12 to 3·05)	-3·76 (-6·30 to -1·06)	-4·59 (-6·90 to -1·25)
	15-49 years	3600 (2950 to 4380)	128 (105 to 156)	-2·91 (-3·50 to -2·34)	-3·70 (-4·38 to -3·00)	384 (232 to 667)	13·7 (8·29 to 23·8)	-1·23 (-3·39 to 0·992)	-4·12 (-6·44 to -1·40)
	50-69 years	1230 (951 to 1560)	316 (244 to 401)	-2·60 (-3·23 to -2·01)	-3·12 (-4·12 to -2·08)	252 (157 to 432)	64·7 (40·1 to 111)	-2·87 (-4·79 to -0·865)	-3·95 (-6·26 to -1·07)
	70+ years	395 (308 to 493)	495 (386 to 618)	-2·46 (-3·20 to -1·64)	-3·43 (-4·70 to -2·41)	183 (115 to 340)	230 (145 to 427)	-2·48 (-4·61 to -0·479)	-3·87 (-6·33 to -1·24)
Mali	All Ages	30600 (26600 to 34400)	201 (178 to 229)	-2·18 (-2·53 to -1·80)	-3·81 (-4·50 to -3·17)	5750 (4280 to 8090)	56·5 (42·5 to 80·0)	-2·90 (-3·83 to -1·93)	-4·53 (-6·37 to -2·91)
	Under 5	4370 (3570 to 5520)	95·4 (77·8 to 120)	-3·64 (-4·37 to -2·95)	-4·15 (-5·30 to -2·79)	687 (443 to 1120)	15·0 (9·68 to 24·5)	-4·20 (-5·45 to -2·50)	-6·60 (-9·37 to -2·98)
	5-14 years	2460 (1740 to 3540)	35·2 (24·9 to 50·7)	-2·43 (-3·21 to -1·60)	-3·77 (-5·24 to -2·67)	142 (99·4 to 201)	2·02 (1·42 to 2·87)	-0·689 (-2·43 to 1·07)	-6·19 (-8·14 to -4·04)
	15-49 years	14500 (11900 to 18000)	138 (113 to 171)	-1·42 (-1·80 to -0·870)	-4·15 (-4·89 to -3·42)	1760 (1340 to 2480)	16·8 (12·8 to 23·6)	-1·08 (-2·34 to 0·163)	-5·01 (-6·92 to -3·38)
	50-69 years	6640 (4960 to 8250)	401 (299 to 499)	-1·70 (-2·23 to -1·13)	-2·75 (-3·71 to -1·81)	1670 (1220 to 2380)	101 (73·5 to 144)	-2·88 (-4·25 to -1·48)	-3·20 (-5·59 to -0·956)
	70+ years	2610 (2070 to 3220)	706 (562 to 872)	-1·79 (-2·57 to -0·940)	-2·69 (-4·00 to -1·47)	1490 (1020 to 2100)	402 (278 to 569)	-2·71 (-4·01 to -1·54)	-2·66 (-4·59 to -0·259)
Mauritania	All Ages	2600 (2310 to 2950)	84·1 (73·6 to 95·3)	-3·46 (-3·88 to -3·04)	-3·58 (-4·35 to -3·09)	438 (293 to 689)	19·6 (13·1 to 31·0)	-5·00 (-6·11 to -3·85)	-4·86 (-7·14 to -1·87)
	Under 5	288 (224 to 361)	43·9 (34·1 to 54·9)	-4·14 (-4·90 to -3·43)	-5·14 (-6·42 to -3·88)	25·5 (16·3 to 45·7)	3·88 (2·49 to 6·96)	-4·96 (-6·63 to -3·33)	-9·45 (-12·7 to -5·87)
	5-14 years	198 (137 to 278)	16·6 (11·4 to 23·3)	-3·57 (-4·41 to -2·83)	-4·47 (-6·01 to -3·42)	8·43 (5·90 to 11·8)	0·705 (0·494 to 0·990)	-4·38 (-5·72 to -2·61)	-6·82 (-9·64 to -3·28)
	15-49 years	1090 (874 to 1360)	53·2 (42·5 to 66·2)	-3·47 (-4·05 to -2·82)	-3·82 (-4·75 to -3·17)	102 (65·8 to 163)	4·97 (3·20 to 7·94)	-5·06 (-6·31 to -3·63)	-5·16 (-7·78 to -2·02)
	50-69 years	704 (548 to 896)	183 (142 to 233)	-3·07 (-3·71 to -2·41)	-2·69 (-3·81 to -1·59)	141 (87·5 to 226)	36·7 (22·7 to 58·6)	-5·53 (-7·21 to -3·86)	-4·47 (-7·12 to -1·27)
	70+ years	316 (251 to 389)	310 (247 to 382)	-2·87 (-3·59 to -1·96)	-3·27 (-4·18 to -2·26)	160 (105 to 257)	157 (103 to 253)	-4·16 (-5·56 to -2·85)	-4·18 (-6·69 to -1·39)
Niger	All Ages	37300 (32700 to 41500)	239 (209 to 268)	-2·24 (-2·67 to -1·78)	-3·26 (-3·82 to -2·56)	6890 (4750 to 10000)	64·4 (42·2 to 90·3)	-3·97 (-4·95 to -2·94)	-3·42 (-5·25 to -1·10)
	Under 5	6220 (4990 to 7770)	122 (97·9 to 152)	-4·04 (-4·80 to -3·29)	-3·60 (-4·72 to -2·52)	1480 (899 to 2750)	29·0 (17·6 to 53·9)	-6·86 (-8·71 to -5·08)	-4·35 (-7·16 to -1·20)

eTable 1. All-form tuberculosis incident cases and deaths, age-standardised rates of incidence and mortality per 100,000 population, and corresponding annualized rates of change by age groups for 204 countries and territories (2021).

Location	Age group	Number of cases	Rate per 100,000 cases	Annualized rate of change (1990-2010)	Annualized rate of change (2010-2021)	Number of deaths	Rate per 100,000 deaths	Annualized deaths (1990-2010)	Annualized deaths (2010-2021)
	5-14 years	3820 (2580 to 5600)	49·8 (33·7 to 73·1)	-2·92 (-3·68 to -2·13)	-3·36 (-4·66 to -2·13)	231 (158 to 362)	3·01 (2·06 to 4·73)	-3·80 (-5·71 to -1·96)	-3·91 (-6·43 to -0·770)
	15-49 years	17100 (13800 to 21300)	165 (133 to 206)	-1·57 (-2·00 to -0·862)	-3·66 (-4·47 to -2·93)	1850 (1250 to 2640)	17·8 (12·0 to 25·4)	-1·84 (-3·04 to -0·625)	-4·59 (-6·49 to -1·94)
	50-69 years	7530 (5690 to 9430)	482 (364 to 604)	-1·38 (-2·03 to -0·811)	-2·26 (-3·21 to -1·24)	1930 (1220 to 2700)	124 (78·1 to 173)	-2·98 (-4·29 to -1·65)	-2·14 (-4·69 to 0·315)
	70+ years	2580 (2060 to 3300)	758 (605 to 968)	-1·44 (-2·24 to -0·779)	-2·50 (-3·74 to -1·34)	1400 (911 to 1960)	412 (267 to 574)	-2·52 (-4·23 to -0·882)	-2·39 (-4·50 to 0·423)
Nigeria	All Ages	340000 (300000 to 378000)	213 (190 to 241)	-0·924 (-1·15 to -0·608)	-5·42 (-5·78 to -5·09)	60100 (42800 to 76400)	50·3 (37·5 to 62·7)	-2·12 (-3·20 to -0·872)	-5·73 (-7·94 to -3·58)
	Under 5	37900 (31100 to 47400)	102 (83·8 to 128)	-2·00 (-2·45 to -1·64)	-6·77 (-7·36 to -6·19)	10900 (6700 to 15900)	29·4 (18·0 to 42·7)	-2·94 (-3·88 to -1·81)	-6·95 (-10·5 to -4·07)
	5-14 years	26700 (19000 to 37800)	41·4 (29·4 to 58·7)	-1·07 (-1·51 to -0·510)	-5·39 (-5·90 to -4·80)	1860 (1260 to 2520)	2·88 (1·96 to 3·91)	-0·563 (-1·66 to 0·929)	-6·59 (-9·28 to -4·53)
	15-49 years	182000 (151000 to 225000)	169 (140 to 209)	0·0670 (-0·226 to 0·476)	-5·39 (-5·78 to -4·98)	19100 (13200 to 25800)	17·7 (12·3 to 23·9)	-0·191 (-1·69 to 1·44)	-6·26 (-8·39 to -4·03)
	50-69 years	67900 (51000 to 85800)	378 (284 to 478)	-1·05 (-1·42 to -0·765)	-5·18 (-5·70 to -4·51)	15600 (11000 to 19900)	86·9 (61·0 to 111)	-2·15 (-3·63 to -0·309)	-4·78 (-7·39 to -1·71)
	70+ years	25700 (20600 to 32200)	687 (550 to 860)	-1·14 (-1·45 to -0·796)	-4·34 (-4·83 to -3·77)	12600 (9890 to 15100)	337 (264 to 404)	-1·60 (-2·66 to -0·535)	-3·97 (-5·57 to -1·88)
Sao Tome and Principe	All Ages	133 (117 to 152)	83·5 (72·9 to 95·6)	-2·45 (-2·84 to -2·05)	-3·40 (-4·09 to -2·77)	18·7 (13·2 to 30·8)	16·4 (11·3 to 27·6)	-3·02 (-4·34 to -1·51)	-4·51 (-6·45 to -2·02)
	Under 5	8·60 (6·85 to 10·9)	34·5 (27·5 to 43·6)	-4·46 (-5·41 to -3·77)	-5·17 (-6·37 to -3·72)	0·180 (0·101 to 0·447)	0·720 (0·405 to 1·79)	-8·33 (-10·1 to -5·58)	-14·4 (-18·5 to -10·0)
	5-14 years	5·89 (4·04 to 8·31)	11·1 (7·64 to 15·7)	-4·78 (-5·60 to -4·06)	-4·02 (-5·39 to -2·43)	0·138 (0·0930 to 0·233)	0·261 (0·176 to 0·440)	-5·98 (-7·73 to -3·96)	-10·5 (-13·6 to -5·84)
	15-49 years	66·2 (54·7 to 80·2)	58·6 (48·5 to 71·0)	-2·01 (-2·59 to -1·45)	-4·07 (-4·96 to -3·01)	5·21 (3·18 to 8·95)	4·61 (2·82 to 7·92)	-1·33 (-3·28 to 0·460)	-5·45 (-8·35 to -2·27)
	50-69 years	38·7 (30·3 to 50·8)	182 (142 to 238)	-1·52 (-2·03 to -0·855)	-3·47 (-4·40 to -2·48)	7·22 (5·06 to 12·3)	33·9 (23·8 to 57·6)	-2·59 (-4·36 to -0·860)	-4·55 (-6·66 to -2·09)
	70+ years	13·4 (10·7 to 16·6)	292 (234 to 362)	-1·53 (-2·30 to -0·853)	-4·08 (-4·96 to -2·95)	5·92 (3·97 to 9·82)	129 (86·6 to 214)	-2·09 (-3·64 to -0·424)	-4·59 (-6·53 to -2·74)
Senegal	All Ages	17100 (14900 to 19500)	149 (131 to 168)	-1·66 (-1·99 to -1·25)	-3·37 (-3·99 to -2·77)	3140 (2310 to 4040)	37·3 (27·0 to 48·2)	-2·53 (-3·46 to -1·46)	-3·42 (-5·55 to -1·54)
	Under 5	1450 (1150 to 1830)	63·8 (50·8 to 80·8)	-3·34 (-4·16 to -2·64)	-5·21 (-6·83 to -4·01)	134 (89·7 to 244)	5·89 (3·95 to 10·7)	-4·46 (-5·94 to -3·02)	-10·6 (-13·0 to -7·39)
	5-14 years	1110 (762 to 1560)	27·2 (18·6 to 38·2)	-2·29 (-3·05 to -1·44)	-3·76 (-4·96 to -2·60)	59·5 (41·0 to 81·7)	1·45 (1·00 to 2·00)	-1·88 (-3·52 to -0·200)	-5·12 (-7·46 to -3·00)
	15-49 years	8630 (7170 to 10900)	112 (92·7 to 141)	-1·55 (-2·05 to -1·04)	-4·01 (-4·93 to -3·20)	1020 (758 to 1450)	13·2 (9·79 to 18·8)	-2·01 (-3·35 to -0·613)	-4·32 (-6·23 to -2·35)
	50-69 years	4180 (3210 to 5310)	296 (228 to 377)	-1·52 (-2·03 to -0·919)	-2·41 (-3·62 to -1·34)	989 (714 to 1310)	70·2 (50·7 to 93·3)	-3·14 (-4·57 to -1·71)	-2·65 (-5·03 to -0·577)

eTable 1. All-form tuberculosis incident cases and deaths, age-standardised rates of incidence and mortality per 100,000 population, and corresponding annualized rates of change by age groups for 204 countries and territories (2021).

Location	Age group	Number of cases	Rate per 100,000 cases	Annualized rate of change (1990-2010)	Annualized rate of change (2010-2021)	Number of deaths	Rate per 100,000 deaths	Annualized deaths (1990-2010)	Annualized deaths (2010-2021)
	70+ years	1730 (1370 to 2080)	490 (386 to 588)	-1·45 (-2·10 to -0·706)	-2·46 (-3·77 to -1·34)	940 (654 to 1300)	265 (185 to 366)	-2·67 (-3·93 to -1·30)	-2·27 (-4·38 to -0·417)
Sierra Leone	All Ages	17700 (15800 to 19900)	272 (242 to 309)	-0·612 (-1·02 to -0·271)	-4·60 (-5·20 to -3·99)	2850 (2130 to 3690)	58·1 (43·1 to 76·3)	-0·917 (-1·79 to 0·114)	-5·93 (-7·63 to -3·95)
	Under 5	2040 (1660 to 2600)	152 (123 to 193)	-2·24 (-2·87 to -1·50)	-5·51 (-6·83 to -4·22)	397 (271 to 609)	29·6 (20·2 to 45·4)	-2·57 (-3·80 to -1·12)	-8·54 (-11·9 to -5·11)
	5-14 years	1190 (819 to 1690)	53·2 (36·7 to 75·5)	-1·39 (-2·09 to -0·560)	-4·75 (-6·24 to -3·21)	68·0 (49·9 to 87·7)	3·04 (2·23 to 3·93)	0·276 (-1·06 to 1·97)	-6·82 (-9·27 to -4·11)
	15-49 years	9600 (7850 to 12100)	216 (177 to 272)	0·352 (-0·147 to 0·978)	-5·00 (-5·86 to -4·14)	1040 (779 to 1420)	23·5 (17·5 to 31·9)	2·13 (0·827 to 3·49)	-6·12 (-7·82 to -3·93)
	50-69 years	3500 (2670 to 4410)	520 (397 to 655)	-0·189 (-0·712 to 0·326)	-4·05 (-5·04 to -2·90)	746 (526 to 1020)	111 (78·2 to 152)	-0·414 (-1·89 to 1·15)	-4·98 (-7·29 to -2·33)
	70+ years	1320 (1020 to 1670)	771 (596 to 975)	-0·490 (-1·24 to 0·239)	-4·37 (-5·18 to -3·34)	590 (441 to 775)	344 (257 to 452)	-0·993 (-2·04 to 0·444)	-4·81 (-6·93 to -2·48)
Togo	All Ages	14300 (12500 to 16400)	226 (199 to 258)	-0·506 (-0·943 to -0·0545)	-4·35 (-5·12 to -3·81)	2560 (1880 to 3850)	55·3 (40·6 to 82·9)	1·12 (-0·305 to 2·51)	-6·02 (-7·92 to -3·78)
	Under 5	1240 (1000 to 1560)	106 (85·6 to 133)	-2·64 (-3·30 to -1·81)	-5·38 (-6·84 to -4·09)	130 (72·6 to 237)	11·0 (6·19 to 20·2)	-1·09 (-2·59 to 0·528)	-11·7 (-15·4 to -7·31)
	5-14 years	1070 (749 to 1490)	50·0 (35·1 to 69·6)	-1·09 (-1·98 to -0·457)	-4·07 (-5·55 to -2·89)	61·7 (44·9 to 87·7)	2·89 (2·10 to 4·11)	1·75 (0·0129 to 3·86)	-6·90 (-9·70 to -4·51)
	15-49 years	7810 (6500 to 9650)	188 (156 to 232)	-0·252 (-0·771 to 0·257)	-5·18 (-5·95 to -4·42)	1080 (759 to 1600)	26·0 (18·3 to 38·4)	2·57 (0·994 to 4·30)	-7·62 (-9·73 to -5·44)
	50-69 years	3310 (2460 to 4300)	436 (323 to 565)	-0·500 (-1·02 to 0·0110)	-3·88 (-4·79 to -2·80)	854 (566 to 1380)	112 (74·4 to 181)	0·103 (-1·65 to 2·04)	-5·37 (-7·93 to -2·70)
	70+ years	867 (666 to 1090)	600 (461 to 756)	-1·16 (-1·86 to -0·463)	-3·73 (-4·70 to -2·79)	439 (325 to 641)	304 (225 to 443)	-1·30 (-2·34 to -0·119)	-4·37 (-6·37 to -1·61)

eTable 2. Progress towards the END-TB 2020 milestones in all-form tuberculosis incidence rate per 100,000 population and in deaths due to all-form tuberculosis by age for 204 countries and territories (2020), percent change from 2015 to 2020.

Location	Age group	2015 Rate	2020 Rate	Incidence Percent Change	Incidence Milestone	2015 Deaths	2020 Deaths	Mortality Percent Change	Mortality Milestone
**Global**	**All Ages**	**129 (114 to 144)**	**121 (108 to 135)**	**-6·26 (-7·25 to -5·27)**	**103 (91·5 to 115)**	**1570000 (1450000 to 1700000)**	**1390000 (1280000 to 1540000)**	**-11·9 (-17·0 to -5·77)**	**1020000 (943000 to 1100000)**
	**Under 5**	**67·9 (57·0 to 82·9)**	**56·7 (48·0 to 70·2)**	**-16·5 (-18·4 to -14·8)**	**54·3 (45·6 to 66·3)**	**104000 (84000 to 119000)**	**67200 (50200 to 83000)**	**-35·3 (-41·7 to -26·7)**	**67300 (54600 to 77600)**
	**5-14 years**	**41·9 (29·4 to 58·0)**	**35·1 (24·7 to 48·8)**	**-16·2 (-17·9 to -14·2)**	**33·5 (23·5 to 46·4)**	**32000 (28400 to 35500)**	**22600 (20000 to 25600)**	**-29·5 (-34·1 to -25·5)**	**20800 (18500 to 23100)**
	**15-49 years**	**141 (120 to 169)**	**132 (113 to 159)**	**-6·29 (-7·70 to -5·05)**	**113 (96·1 to 135)**	**593000 (534000 to 650000)**	**503000 (455000 to 549000)**	**-15·2 (-20·2 to -10·0)**	**385000 (347000 to 422000)**
	**50-69 years**	**188 (149 to 232)**	**177 (141 to 218)**	**-5·72 (-7·39 to -4·02)**	**150 (119 to 186)**	**499000 (467000 to 528000)**	**459000 (421000 to 515000)**	**-7·97 (-14·1 to -0·472)**	**324000 (304000 to 343000)**
	**70+ years**	**214 (172 to 260)**	**196 (160 to 238)**	**-8·48 (-10·4 to -6·74)**	**171 (138 to 208)**	**347000 (322000 to 367000)**	**335000 (307000 to 378000)**	**-3·29 (-9·07 to 5·56)**	**225000 (209000 to 239000)**
Central Europe, Eastern Europe, and Central Asia	All Ages	64·2 (55·9 to 76·0)	53·7 (46·5 to 63·9)	-16·5 (-19·4 to -13·9)	51·4 (44·7 to 60·8)	26900 (25900 to 28600)	16400 (15600 to 17600)	-38·8 (-40·6 to -36·5)	17500 (16800 to 18600)
	Under 5	11·7 (9·76 to 15·1)	9·43 (7·82 to 12·2)	-19·6 (-24·4 to -16·8)	9·39 (7·81 to 12·1)	602 (507 to 705)	351 (287 to 454)	-41·6 (-50·0 to -33·7)	391 (330 to 458)
	5-14 years	13·9 (9·46 to 19·2)	11·7 (8·00 to 16·4)	-15·7 (-20·1 to -11·3)	11·1 (7·57 to 15·4)	103 (93·6 to 113)	70·1 (63·0 to 78·8)	-31·9 (-35·5 to -27·7)	66·9 (60·9 to 73·5)
	15-49 years	86·1 (69·6 to 106)	72·8 (58·9 to 92·3)	-15·5 (-19·5 to -12·3)	68·8 (55·7 to 85·1)	12400 (11600 to 13900)	7160 (6570 to 8070)	-42·4 (-43·9 to -40·3)	8090 (7560 to 9060)
	50-69 years	66·7 (50·0 to 84·6)	56·9 (42·7 to 73·3)	-14·7 (-18·3 to -10·9)	53·4 (40·0 to 67·7)	11000 (10800 to 11300)	6610 (6340 to 7040)	-40·0 (-42·5 to -37·2)	7170 (7010 to 7350)
	70+ years	43·2 (33·5 to 54·6)	36·6 (28·9 to 46·3)	-15·3 (-19·6 to -11·3)	34·6 (26·8 to 43·7)	2680 (2550 to 2760)	2240 (2100 to 2330)	-16·5 (-18·6 to -14·4)	1740 (1660 to 1790)
Central Asia	All Ages	67·4 (59·0 to 78·0)	56·6 (49·7 to 65·5)	-16·0 (-18·4 to -12·3)	53·9 (47·2 to 62·4)	6430 (6060 to 6820)	4520 (4150 to 4980)	-29·7 (-34·3 to -24·1)	4180 (3940 to 4430)
	Under 5	18·3 (15·5 to 22·9)	15·0 (12·5 to 19·1)	-18·1 (-24·2 to -12·1)	14·7 (12·4 to 18·3)	477 (385 to 569)	303 (239 to 404)	-36·3 (-46·5 to -26·9)	310 (250 to 370)
	5-14 years	19·5 (13·7 to 27·1)	16·0 (11·0 to 21·8)	-17·9 (-23·5 to -12·3)	15·6 (11·0 to 21·7)	72·7 (64·3 to 79·2)	50·6 (45·4 to 57·8)	-30·4 (-35·4 to -24·4)	47·3 (41·8 to 51·5)
	15-49 years	81·9 (69·9 to 101)	68·4 (57·5 to 84·3)	-16·5 (-19·6 to -12·5)	65·5 (55·9 to 80·7)	3260 (3060 to 3470)	2160 (1980 to 2370)	-33·6 (-37·9 to -29·0)	2120 (1990 to 2250)
	50-69 years	96·2 (72·7 to 119)	84·5 (64·6 to 106)	-12·1 (-16·9 to -6·31)	76·9 (58·1 to 95·0)	2100 (1980 to 2230)	1590 (1440 to 1750)	-24·4 (-30·9 to -16·3)	1370 (1290 to 1450)
	70+ years	99·3 (79·2 to 122)	87·6 (69·5 to 112)	-11·7 (-17·1 to -6·49)	79·5 (63·3 to 97·3)	522 (479 to 560)	411 (374 to 453)	-21·1 (-26·2 to -16·1)	339 (312 to 364)
Armenia	All Ages	32·9 (28·5 to 37·7)	27·3 (23·9 to 31·5)	-17·2 (-23·1 to -10·6)	26·3 (22·8 to 30·2)	76·5 (69·5 to 83·3)	44·0 (40·6 to 48·3)	-42·4 (-47·8 to -36·6)	49·7 (45·2 to 54·1)
	Under 5	6·45 (5·10 to 8·17)	4·96 (3·90 to 6·30)	-22·9 (-32·2 to -13·0)	5·16 (4·08 to 6·53)	1·27 (1·01 to 1·51)	0·754 (0·594 to 0·943)	-40·7 (-48·7 to -30·6)	0·827 (0·656 to 0·983)
	5-14 years	5·71 (3·87 to 8·35)	4·44 (2·97 to 6·30)	-22·3 (-30·5 to -13·7)	4·57 (3·10 to 6·68)	0·283 (0·242 to 0·323)	0·143 (0·124 to 0·165)	-49·3 (-56·1 to -40·5)	0·184 (0·157 to 0·210)

eTable 2. Progress towards the END-TB 2020 milestones in all-form tuberculosis incidence rate per 100,000 population and in deaths due to all-form tuberculosis by age for 204 countries and territories (2020), percent change from 2015 to 2020.

Location	Age group	2015 Rate	2020 Rate	Incidence Percent Change	Incidence Milestone	2015 Deaths	2020 Deaths	Mortality Percent Change	Mortality Milestone
	15-49 years	38·0 (30·9 to 46·3)	31·3 (26·2 to 39·0)	-17·4 (-24·8 to -10·7)	30·4 (24·7 to 37·0)	26·0 (24·2 to 28·0)	18·2 (16·8 to 20·1)	-29·9 (-36·1 to -22·2)	16·9 (15·7 to 18·2)
	50-69 years	44·8 (33·9 to 57·3)	37·4 (27·9 to 48·5)	-16·3 (-24·9 to -6·24)	35·9 (27·1 to 45·8)	34·6 (31·5 to 37·7)	15·4 (13·9 to 17·1)	-55·4 (-59·6 to -50·9)	22·5 (20·5 to 24·5)
	70+ years	33·0 (25·7 to 41·1)	28·8 (22·3 to 35·4)	-12·5 (-21·9 to -0·705)	26·4 (20·6 to 32·9)	14·3 (12·4 to 16·1)	9·45 (8·20 to 10·7)	-33·9 (-41·2 to -26·9)	9·31 (8·04 to 10·5)
Azerbaijan	All Ages	61·4 (52·7 to 73·1)	55·6 (47·8 to 66·1)	-9·39 (-16·2 to -2·26)	49·1 (42·1 to 58·5)	678 (539 to 934)	444 (347 to 655)	-34·5 (-44·9 to -16·5)	441 (350 to 607)
	Under 5	16·6 (13·2 to 21·4)	13·7 (10·8 to 17·4)	-17·1 (-26·1 to -5·50)	13·3 (10·6 to 17·2)	35·2 (25·8 to 49·0)	20·9 (14·2 to 30·1)	-40·3 (-53·4 to -26·5)	22·9 (16·8 to 31·8)
	5-14 years	15·0 (10·0 to 21·0)	13·2 (9·16 to 18·3)	-11·6 (-21·8 to -0·436)	12·0 (8·00 to 16·8)	6·25 (4·55 to 8·16)	5·61 (4·26 to 7·12)	-9·68 (-25·3 to 14·3)	4·06 (2·95 to 5·31)
	15-49 years	77·4 (63·9 to 96·2)	70·4 (57·6 to 87·6)	-8·87 (-17·3 to -0·191)	61·9 (51·1 to 76·9)	357 (277 to 486)	242 (180 to 366)	-32·2 (-43·4 to -18·8)	232 (180 to 316)
	50-69 years	70·8 (52·5 to 90·5)	63·7 (48·4 to 79·8)	-9·73 (-18·6 to 0·717)	56·7 (42·0 to 72·4)	224 (167 to 329)	126 (87·5 to 210)	-43·1 (-56·4 to -10·3)	146 (108 to 214)
	70+ years	60·2 (47·9 to 74·3)	55·2 (42·3 to 70·2)	-8·02 (-20·3 to 2·91)	48·1 (38·3 to 59·5)	55·5 (41·6 to 75·5)	48·2 (36·2 to 69·2)	-12·8 (-29·2 to 4·88)	36·1 (27·0 to 49·1)
Georgia	All Ages	71·5 (62·3 to 82·1)	60·7 (52·5 to 70·8)	-15·2 (-20·3 to -9·52)	57·2 (49·9 to 65·7)	199 (180 to 219)	124 (110 to 137)	-37·9 (-43·7 to -33·0)	130 (117 to 143)
	Under 5	11·4 (8·82 to 14·2)	8·86 (7·13 to 12·0)	-22·3 (-32·0 to -12·6)	9·12 (7·06 to 11·3)	2·01 (1·65 to 2·50)	1·05 (0·851 to 1·30)	-47·8 (-54·9 to -39·0)	1·31 (1·07 to 1·63)
	5-14 years	18·8 (13·3 to 26·7)	14·3 (9·92 to 21·0)	-23·8 (-32·4 to -11·7)	15·1 (10·7 to 21·4)	0·603 (0·510 to 0·700)	0·296 (0·259 to 0·335)	-50·8 (-56·8 to -43·6)	0·392 (0·332 to 0·455)
	15-49 years	93·1 (76·5 to 114)	78·3 (65·5 to 98·3)	-15·8 (-21·1 to -9·57)	74·5 (61·2 to 91·6)	71·0 (66·5 to 76·2)	39·5 (36·4 to 43·1)	-44·4 (-48·5 to -40·0)	46·1 (43·2 to 49·5)
	50-69 years	78·8 (59·8 to 101)	71·2 (53·9 to 89·8)	-9·55 (-17·2 to 0·00963)	63·0 (47·8 to 80·6)	87·2 (74·5 to 99·3)	52·7 (44·9 to 60·4)	-39·5 (-47·2 to -31·9)	56·7 (48·4 to 64·6)
	70+ years	59·2 (47·1 to 73·4)	53·4 (42·9 to 66·5)	-9·52 (-19·4 to -0·853)	47·3 (37·7 to 58·7)	38·5 (33·2 to 44·2)	30·1 (26·0 to 34·2)	-21·6 (-31·2 to -14·4)	25·0 (21·6 to 28·7)
Kazakhstan	All Ages	74·9 (64·5 to 87·9)	58·0 (50·5 to 69·4)	-22·5 (-26·4 to -16·7)	59·9 (51·6 to 70·3)	826 (774 to 889)	571 (515 to 620)	-30·8 (-36·1 to -25·8)	537 (503 to 578)
	Under 5	11·3 (8·91 to 14·9)	8·61 (6·92 to 11·2)	-23·9 (-35·1 to -12·5)	9·08 (7·12 to 11·9)	26·4 (22·0 to 31·4)	17·2 (13·9 to 20·7)	-35·0 (-43·4 to -23·3)	17·2 (14·3 to 20·4)
	5-14 years	12·3 (8·38 to 18·0)	9·06 (5·85 to 12·3)	-26·3 (-37·0 to -14·9)	9·85 (6·71 to 14·4)	5·93 (5·05 to 7·03)	3·21 (2·81 to 3·73)	-45·6 (-53·7 to -37·2)	3·86 (3·28 to 4·57)
	15-49 years	99·0 (82·7 to 124)	76·6 (62·1 to 96·2)	-22·5 (-28·6 to -14·3)	79·2 (66·2 to 98·9)	451 (416 to 491)	273 (245 to 296)	-39·4 (-44·2 to -34·7)	293 (270 to 319)
	50-69 years	95·5 (70·4 to 121)	79·4 (61·2 to 101)	-16·8 (-24·6 to -7·74)	76·4 (56·3 to 97·1)	277 (255 to 309)	200 (174 to 224)	-27·8 (-34·6 to -20·2)	180 (166 to 201)
	70+ years	86·2 (66·8 to 108)	77·7 (60·2 to 101)	-9·77 (-19·1 to 0·181)	68·9 (53·4 to 86·7)	65·6 (61·5 to 73·2)	78·1 (70·7 to 85·7)	19·2 (8·24 to 28·4)	42·6 (40·0 to 47·6)
Kyrgyzstan	All Ages	97·3 (84·6 to 114)	83·5 (72·5 to 98·3)	-14·2 (-18·7 to -9·12)	77·9 (67·7 to 91·1)	470 (421 to 513)	344 (291 to 405)	-26·7 (-36·7 to -14·2)	305 (274 to 333)

eTable 2. Progress towards the END-TB 2020 milestones in all-form tuberculosis incidence rate per 100,000 population and in deaths due to all-form tuberculosis by age for 204 countries and territories (2020), percent change from 2015 to 2020.

Location	Age group	2015 Rate	2020 Rate	Incidence Percent Change	Incidence Milestone	2015 Deaths	2020 Deaths	Mortality Percent Change	Mortality Milestone
	Under 5	17·8 (14·4 to 22·4)	14·2 (11·6 to 17·9)	-19·6 (-30·0 to -8·11)	14·2 (11·5 to 18·0)	18·5 (16·3 to 21·2)	11·9 (10·3 to 13·8)	-35·7 (-45·0 to -25·1)	12·1 (10·6 to 13·8)
	5-14 years	27·1 (18·9 to 37·5)	21·8 (15·0 to 30·3)	-19·4 (-27·2 to -7·94)	21·7 (15·1 to 30·0)	3·26 (2·74 to 3·72)	2·83 (2·40 to 3·25)	-13·1 (-26·1 to 1·55)	2·12 (1·78 to 2·42)
	15-49 years	125 (106 to 155)	107 (88·2 to 134)	-14·6 (-20·5 to -6·61)	100 (85·0 to 124)	262 (233 to 289)	179 (152 to 210)	-31·6 (-40·6 to -21·0)	170 (152 to 188)
	50-69 years	150 (114 to 187)	138 (103 to 172)	-8·34 (-17·1 to 0·997)	120 (91·5 to 149)	155 (138 to 170)	127 (104 to 151)	-17·4 (-31·3 to -0·00266)	100 (89·6 to 110)
	70+ years	171 (135 to 212)	154 (122 to 192)	-9·54 (-19·4 to 4·22)	137 (108 to 170)	31·4 (28·2 to 35·1)	23·0 (19·1 to 27·6)	-26·9 (-36·3 to -13·8)	20·4 (18·3 to 22·8)
Mongolia	All Ages	132 (116 to 154)	104 (89·7 to 122)	-21·7 (-26·8 to -16·6)	106 (93·1 to 123)	549 (387 to 753)	400 (296 to 578)	-26·9 (-41·0 to -9·56)	357 (252 to 489)
	Under 5	35·7 (29·2 to 44·9)	27·3 (22·0 to 34·4)	-23·5 (-31·7 to -12·6)	28·6 (23·4 to 35·9)	23·0 (15·8 to 32·1)	14·0 (10·1 to 20·6)	-38·3 (-54·1 to -18·6)	14·9 (10·3 to 20·9)
	5-14 years	28·3 (19·5 to 40·4)	20·0 (13·9 to 28·4)	-29·4 (-38·5 to -19·4)	22·6 (15·6 to 32·3)	4·90 (3·58 to 6·21)	5·14 (3·59 to 6·63)	5·39 (-17·6 to 30·8)	3·18 (2·33 to 4·04)
	15-49 years	171 (144 to 209)	139 (115 to 174)	-19·1 (-25·7 to -11·9)	137 (116 to 167)	322 (226 to 453)	232 (169 to 360)	-27·7 (-40·6 to -10·7)	209 (147 to 295)
	50-69 years	199 (158 to 245)	161 (126 to 203)	-19·0 (-26·3 to -10·6)	159 (126 to 196)	171 (116 to 232)	127 (90·1 to 175)	-25·0 (-43·7 to -3·24)	111 (75·5 to 151)
	70+ years	141 (112 to 179)	111 (87·7 to 141)	-20·6 (-30·3 to -10·8)	113 (89·4 to 143)	28·8 (20·9 to 38·2)	21·7 (16·4 to 28·7)	-23·9 (-39·6 to -4·12)	18·7 (13·6 to 24·9)
Tajikistan	All Ages	63·4 (55·5 to 73·7)	52·3 (44·6 to 62·3)	-17·6 (-22·0 to -12·1)	50·8 (44·4 to 59·0)	881 (683 to 1120)	769 (571 to 960)	-12·4 (-27·5 to 5·75)	573 (444 to 726)
	Under 5	32·1 (25·5 to 40·2)	26·9 (21·4 to 34·9)	-16·2 (-26·4 to -5·28)	25·7 (20·4 to 32·1)	162 (106 to 220)	131 (82·4 to 194)	-18·2 (-40·1 to 9·31)	105 (68·8 to 143)
	5-14 years	16·1 (10·8 to 22·7)	13·0 (8·88 to 18·5)	-18·7 (-30·4 to -8·09)	12·8 (8·67 to 18·2)	11·8 (8·79 to 15·5)	12·3 (8·62 to 16·8)	4·72 (-12·9 to 29·8)	7·68 (5·71 to 10·1)
	15-49 years	81·8 (67·6 to 98·7)	67·2 (54·2 to 83·5)	-17·9 (-24·0 to -11·5)	65·4 (54·1 to 79·0)	401 (286 to 526)	325 (235 to 426)	-18·3 (-35·1 to 1·60)	260 (186 to 342)
	50-69 years	99·7 (78·2 to 129)	82·6 (64·4 to 105)	-17·1 (-25·0 to -8·43)	79·7 (62·6 to 103)	238 (181 to 304)	244 (171 to 320)	2·94 (-18·3 to 27·9)	155 (118 to 198)
	70+ years	108 (85·1 to 136)	91·0 (70·3 to 112)	-15·5 (-23·5 to -5·85)	86·3 (68·1 to 109)	68·6 (49·0 to 91·8)	55·8 (36·8 to 75·7)	-18·3 (-35·2 to -0·952)	44·6 (31·9 to 59·6)
Turkmenistan	All Ages	59·5 (51·5 to 70·1)	50·5 (42·9 to 61·7)	-15·1 (-21·0 to -7·31)	47·6 (41·2 to 56·1)	486 (456 to 520)	397 (320 to 487)	-18·3 (-32·7 to -1·82)	316 (296 to 338)
	Under 5	16·0 (12·6 to 19·4)	14·6 (11·8 to 18·1)	-8·71 (-20·0 to 3·58)	12·8 (10·1 to 15·5)	30·2 (26·0 to 34·9)	20·3 (16·6 to 24·9)	-32·7 (-41·7 to -22·2)	19·6 (16·9 to 22·7)
	5-14 years	9·66 (6·58 to 14·1)	11·9 (8·06 to 18·0)	23·3 (3·92 to 48·8)	7·73 (5·27 to 11·3)	4·09 (3·54 to 4·68)	3·10 (2·65 to 3·60)	-24·0 (-34·3 to -13·7)	2·66 (2·30 to 3·04)
	15-49 years	79·9 (64·6 to 99·3)	66·8 (54·7 to 85·2)	-16·4 (-23·7 to -6·92)	63·9 (51·7 to 79·5)	297 (276 to 327)	237 (188 to 293)	-20·1 (-34·9 to -3·25)	193 (179 to 213)
	50-69 years	83·4 (62·3 to 110)	66·5 (48·1 to 87·3)	-20·3 (-27·8 to -10·6)	66·7 (49·8 to 88·4)	131 (122 to 140)	117 (92·1 to 148)	-10·7 (-29·9 to 10·1)	85·2 (79·3 to 91·2)

eTable 2. Progress towards the END-TB 2020 milestones in all-form tuberculosis incidence rate per 100,000 population and in deaths due to all-form tuberculosis by age for 204 countries and territories (2020), percent change from 2015 to 2020.

Location	Age group	2015 Rate	2020 Rate	Incidence Percent Change	Incidence Milestone	2015 Deaths	2020 Deaths	Mortality Percent Change	Mortality Milestone
	70+ years	69·6 (52·4 to 86·1)	58·8 (46·2 to 72·8)	-15·3 (-25·0 to -1·54)	55·7 (42·0 to 68·8)	23·8 (22·0 to 25·7)	19·7 (16·4 to 23·4)	-17·1 (-30·1 to -2·32)	15·5 (14·3 to 16·7)
Uzbekistan	All Ages	58·0 (50·9 to 66·5)	50·5 (43·7 to 58·0)	-12·8 (-18·8 to -6·61)	46·4 (40·7 to 53·2)	2270 (2100 to 2460)	1420 (1280 to 1600)	-37·1 (-43·8 to -29·4)	1470 (1370 to 1600)
	Under 5	17·3 (14·3 to 22·0)	14·2 (11·5 to 18·5)	-18·1 (-26·9 to -7·66)	13·9 (11·5 to 17·6)	179 (142 to 224)	86·4 (69·4 to 110)	-51·5 (-58·4 to -42·2)	116 (92·2 to 145)
	5-14 years	25·8 (18·2 to 36·0)	21·3 (15·1 to 28·9)	-17·0 (-28·2 to -5·97)	20·6 (14·5 to 28·8)	35·6 (30·3 to 41·8)	17·9 (15·1 to 21·2)	-49·6 (-55·1 to -44·8)	23·1 (19·7 to 27·1)
	15-49 years	60·5 (51·9 to 73·7)	52·2 (44·0 to 63·9)	-13·7 (-21·0 to -5·84)	48·4 (41·5 to 59·0)	1070 (1000 to 1160)	618 (561 to 698)	-42·3 (-48·4 to -35·8)	697 (653 to 753)
	50-69 years	101 (74·3 to 128)	91·7 (70·4 to 112)	-9·42 (-18·0 to -0·515)	81·1 (59·4 to 102)	783 (715 to 856)	577 (504 to 657)	-26·2 (-36·9 to -15·6)	509 (465 to 557)
	70+ years	147 (119 to 177)	128 (101 to 164)	-12·5 (-22·2 to -2·07)	117 (94·9 to 142)	195 (177 to 212)	125 (111 to 141)	-35·8 (-43·3 to -27·4)	127 (115 to 138)
Central Europe	All Ages	21·4 (18·7 to 24·6)	18·5 (16·0 to 21·6)	-13·6 (-16·4 to -11·0)	17·1 (14·9 to 19·7)	2730 (2640 to 2850)	1970 (1850 to 2080)	-28·0 (-30·9 to -25·4)	1780 (1720 to 1850)
	Under 5	4·28 (3·42 to 5·49)	3·61 (2·89 to 4·60)	-15·6 (-22·1 to -9·98)	3·42 (2·73 to 4·39)	19·1 (17·6 to 20·6)	14·0 (12·2 to 15·9)	-26·7 (-35·1 to -17·2)	12·4 (11·4 to 13·4)
	5-14 years	5·10 (3·59 to 7·12)	4·34 (2·98 to 6·11)	-14·9 (-20·5 to -6·74)	4·08 (2·87 to 5·69)	7·49 (6·01 to 9·40)	4·51 (3·41 to 5·77)	-39·9 (-44·2 to -34·0)	4·87 (3·91 to 6·11)
	15-49 years	20·5 (16·8 to 25·1)	17·2 (14·0 to 21·2)	-16·2 (-21·0 to -12·3)	16·4 (13·4 to 20·1)	667 (630 to 713)	430 (394 to 460)	-35·5 (-38·5 to -32·2)	433 (409 to 463)
	50-69 years	29·5 (22·0 to 37·8)	26·2 (19·6 to 33·2)	-11·1 (-14·8 to -6·05)	23·6 (17·6 to 30·3)	1200 (1150 to 1270)	803 (751 to 851)	-33·0 (-36·1 to -29·6)	779 (750 to 823)
	70+ years	29·6 (23·5 to 36·8)	25·1 (20·3 to 31·0)	-15·2 (-18·7 to -11·5)	23·7 (18·8 to 29·4)	839 (791 to 880)	715 (667 to 757)	-14·8 (-18·2 to -11·5)	545 (514 to 572)
Albania	All Ages	13·3 (11·8 to 15·2)	12·9 (11·2 to 14·6)	-3·03 (-8·22 to 1·67)	10·7 (9·44 to 12·1)	42·6 (31·4 to 59·0)	35·0 (24·5 to 53·0)	-17·5 (-40·1 to 4·38)	27·7 (20·4 to 38·4)
	Under 5	4·39 (3·40 to 5·76)	3·90 (3·04 to 4·99)	-10·9 (-22·7 to 0·323)	3·52 (2·72 to 4·61)	0·799 (0·572 to 1·09)	0·557 (0·416 to 0·797)	-29·4 (-46·9 to -1·09)	0·520 (0·372 to 0·706)
	5-14 years	3·84 (2·62 to 5·36)	3·49 (2·40 to 4·88)	-8·83 (-20·4 to 1·35)	3·07 (2·10 to 4·28)	0·227 (0·184 to 0·284)	0·102 (0·0781 to 0·131)	-54·5 (-66·1 to -41·0)	0·148 (0·120 to 0·184)
	15-49 years	11·3 (9·50 to 14·1)	10·5 (8·46 to 13·0)	-6·93 (-13·4 to 1·47)	9·00 (7·60 to 11·3)	8·61 (6·56 to 12·1)	5·50 (3·74 to 8·21)	-35·8 (-53·1 to -17·9)	5·60 (4·26 to 7·88)
	50-69 years	19·1 (14·3 to 25·5)	17·9 (13·7 to 23·0)	-6·14 (-15·2 to 1·85)	15·3 (11·4 to 20·4)	14·3 (9·94 to 20·7)	8·70 (5·44 to 14·3)	-38·6 (-57·5 to -17·2)	9·27 (6·46 to 13·5)
	70+ years	29·7 (22·9 to 37·0)	27·6 (22·0 to 34·2)	-6·90 (-15·9 to 3·78)	23·8 (18·3 to 29·6)	18·7 (13·7 to 26·3)	20·1 (13·8 to 31·2)	7·88 (-21·1 to 33·8)	12·2 (8·92 to 17·1)
Bosnia and Herzegovina	All Ages	28·2 (24·2 to 32·4)	26·5 (22·8 to 30·3)	-6·16 (-11·2 to -1·27)	22·6 (19·3 to 26·0)	167 (147 to 197)	124 (104 to 147)	-25·5 (-34·6 to -11·0)	108 (95·2 to 128)
	Under 5	4·45 (3·54 to 5·73)	3·76 (2·91 to 4·75)	-15·4 (-25·9 to -1·86)	3·56 (2·83 to 4·59)	0·426 (0·340 to 0·505)	0·326 (0·242 to 0·422)	-23·0 (-44·7 to 0·0219)	0·277 (0·221 to 0·328)
	5-14 years	4·87 (3·27 to 7·12)	4·34 (2·88 to 6·26)	-10·9 (-22·0 to 1·96)	3·90 (2·61 to 5·70)	0·438 (0·365 to 0·526)	0·308 (0·243 to 0·378)	-29·6 (-40·8 to -14·0)	0·285 (0·237 to 0·342)

eTable 2. Progress towards the END-TB 2020 milestones in all-form tuberculosis incidence rate per 100,000 population and in deaths due to all-form tuberculosis by age for 204 countries and territories (2020), percent change from 2015 to 2020.

Location	Age group	2015 Rate	2020 Rate	Incidence Percent Change	Incidence Milestone	2015 Deaths	2020 Deaths	Mortality Percent Change	Mortality Milestone
	15-49 years	20·3 (16·4 to 25·1)	18·8 (15·4 to 23·2)	-7·41 (-14·7 to -1·28)	16·2 (13·1 to 20·1)	23·9 (20·0 to 29·2)	13·3 (11·1 to 16·3)	-44·1 (-53·7 to -33·8)	15·5 (13·0 to 19·0)
	50-69 years	41·0 (30·5 to 52·9)	37·4 (28·6 to 46·9)	-8·47 (-16·6 to 0·163)	32·8 (24·4 to 42·3)	57·1 (48·3 to 69·2)	39·1 (30·9 to 49·2)	-31·3 (-42·2 to -14·1)	37·1 (31·4 to 45·0)
	70+ years	65·5 (50·7 to 80·8)	58·8 (47·5 to 71·2)	-9·88 (-19·6 to 0·365)	52·4 (40·5 to 64·6)	85·0 (74·2 to 101)	71·1 (60·0 to 84·8)	-16·2 (-29·3 to -1·01)	55·2 (48·2 to 65·5)
Bulgaria	All Ages	20·0 (17·6 to 23·2)	16·9 (14·7 to 19·5)	-15·4 (-19·4 to -11·5)	16·0 (14·1 to 18·6)	123 (114 to 132)	88·7 (83·0 to 95·6)	-27·7 (-34·0 to -21·7)	79·9 (74·0 to 85·7)
	Under 5	5·92 (4·51 to 7·97)	4·98 (3·77 to 6·71)	-15·7 (-28·3 to -1·74)	4·74 (3·61 to 6·37)	0·421 (0·385 to 0·456)	0·225 (0·197 to 0·254)	-46·5 (-54·8 to -39·1)	0·274 (0·250 to 0·296)
	5-14 years	9·44 (6·38 to 13·3)	7·65 (5·17 to 10·7)	-18·9 (-28·4 to -9·04)	7·55 (5·10 to 10·6)	0·160 (0·129 to 0·201)	0·101 (0·0814 to 0·133)	-36·9 (-44·0 to -31·2)	0·104 (0·0839 to 0·131)
	15-49 years	19·9 (16·5 to 24·4)	16·7 (13·5 to 21·2)	-15·7 (-21·1 to -9·94)	15·9 (13·2 to 19·5)	21·7 (19·8 to 23·8)	14·7 (13·1 to 16·1)	-32·1 (-40·6 to -24·6)	14·1 (12·9 to 15·5)
	50-69 years	24·6 (18·3 to 30·3)	21·1 (15·6 to 26·1)	-14·0 (-21·5 to -7·34)	19·7 (14·6 to 24·2)	58·0 (52·8 to 63·9)	35·8 (32·9 to 40·4)	-38·1 (-44·9 to -31·9)	37·7 (34·3 to 41·6)
	70+ years	23·6 (18·9 to 29·8)	19·6 (15·9 to 24·4)	-16·5 (-23·3 to -8·49)	18·8 (15·1 to 23·8)	42·6 (38·9 to 46·0)	37·8 (34·8 to 40·8)	-11·1 (-18·6 to -2·50)	27·7 (25·3 to 29·9)
Croatia	All Ages	11·8 (10·4 to 13·4)	10·1 (8·63 to 11·6)	-14·7 (-19·1 to -9·55)	9·46 (8·36 to 10·7)	77·6 (71·2 to 84·7)	57·4 (51·4 to 63·2)	-26·1 (-31·4 to -20·2)	50·5 (46·3 to 55·0)
	Under 5	2·61 (2·02 to 3·40)	2·15 (1·58 to 2·72)	-17·6 (-26·4 to -5·91)	2·09 (1·62 to 2·72)	0·241 (0·208 to 0·286)	0·156 (0·129 to 0·186)	-35·2 (-44·6 to -22·5)	0·157 (0·135 to 0·186)
	5-14 years	2·51 (1·68 to 3·67)	2·01 (1·35 to 2·85)	-19·8 (-28·8 to -11·8)	2·01 (1·34 to 2·93)	0·206 (0·147 to 0·285)	0·114 (0·0785 to 0·163)	-44·5 (-49·2 to -40·2)	0·134 (0·0955 to 0·185)
	15-49 years	8·64 (7·16 to 11·0)	7·12 (5·80 to 8·75)	-17·5 (-23·5 to -11·7)	6·91 (5·73 to 8·83)	11·0 (10·3 to 11·9)	5·66 (5·04 to 6·38)	-48·4 (-52·1 to -44·4)	7·13 (6·68 to 7·73)
	50-69 years	16·3 (12·3 to 20·3)	13·7 (10·4 to 17·3)	-16·1 (-23·8 to -8·30)	13·0 (9·85 to 16·3)	27·1 (24·7 to 29·5)	16·9 (15·1 to 19·0)	-37·6 (-42·8 to -32·1)	17·6 (16·1 to 19·2)
	70+ years	23·8 (19·2 to 30·1)	20·2 (16·4 to 24·3)	-15·1 (-22·7 to -6·44)	19·1 (15·4 to 24·1)	39·1 (35·1 to 43·0)	34·6 (29·9 to 38·9)	-11·6 (-19·1 to -4·00)	25·4 (22·8 to 28·0)
Czechia	All Ages	5·39 (4·61 to 6·12)	4·75 (4·09 to 5·41)	-11·8 (-17·2 to -6·83)	4·31 (3·69 to 4·90)	61·6 (57·0 to 66·1)	34·4 (31·3 to 37·4)	-44·2 (-47·8 to -40·2)	40·1 (37·0 to 43·0)
	Under 5	1·50 (1·14 to 1·90)	1·33 (0·971 to 1·72)	-11·2 (-23·6 to 0·855)	1·20 (0·911 to 1·52)	0·221 (0·178 to 0·259)	0·0954 (0·0760 to 0·125)	-56·8 (-60·6 to -51·5)	0·143 (0·116 to 0·168)
	5-14 years	1·13 (0·735 to 1·65)	1·05 (0·666 to 1·53)	-6·93 (-17·3 to 7·06)	0·903 (0·588 to 1·32)	0·251 (0·176 to 0·343)	0·139 (0·0902 to 0·206)	-45·0 (-50·8 to -32·2)	0·163 (0·114 to 0·223)
	15-49 years	4·92 (4·04 to 6·18)	4·45 (3·64 to 5·57)	-9·53 (-16·3 to -1·18)	3·94 (3·23 to 4·94)	10·5 (9·46 to 11·5)	5·35 (4·81 to 6·24)	-48·9 (-52·6 to -44·9)	6·80 (6·15 to 7·45)
	50-69 years	7·21 (5·59 to 8·98)	6·26 (4·72 to 7·73)	-13·1 (-20·7 to -5·98)	5·77 (4·47 to 7·18)	23·3 (21·0 to 26·0)	9·39 (8·22 to 10·6)	-59·7 (-63·6 to -55·7)	15·2 (13·6 to 16·9)
	70+ years	8·62 (7·00 to 10·7)	7·17 (5·73 to 8·87)	-16·7 (-25·6 to -6·77)	6·89 (5·60 to 8·56)	27·4 (24·6 to 29·7)	19·4 (17·5 to 21·3)	-29·1 (-33·4 to -24·5)	17·8 (16·0 to 19·3)
Hungary	All Ages	8·75 (7·63 to 10·0)	6·90 (5·99 to 8·06)	-21·1 (-24·8 to -15·9)	7·00 (6·10 to 8·03)	117 (110 to 124)	65·8 (60·3 to 70·2)	-43·7 (-47·5 to -39·5)	76·0 (71·2 to 80·8)

eTable 2. Progress towards the END-TB 2020 milestones in all-form tuberculosis incidence rate per 100,000 population and in deaths due to all-form tuberculosis by age for 204 countries and territories (2020), percent change from 2015 to 2020.

Location	Age group	2015 Rate	2020 Rate	Incidence Percent Change	Incidence Milestone	2015 Deaths	2020 Deaths	Mortality Percent Change	Mortality Milestone
	Under 5	1·96 (1·55 to 2·49)	1·64 (1·24 to 2·13)	-16·2 (-28·1 to -5·41)	1·57 (1·24 to 1·99)	0·623 (0·527 to 0·753)	0·297 (0·244 to 0·358)	-52·2 (-58·7 to -45·6)	0·405 (0·343 to 0·490)
	5-14 years	1·35 (0·899 to 1·90)	1·14 (0·767 to 1·65)	-15·0 (-26·7 to -4·16)	1·08 (0·719 to 1·52)	0·637 (0·420 to 0·910)	0·300 (0·192 to 0·434)	-53·1 (-56·5 to -50·0)	0·414 (0·273 to 0·591)
	15-49 years	7·07 (5·84 to 8·68)	5·53 (4·48 to 6·79)	-21·7 (-28·9 to -14·3)	5·66 (4·67 to 6·95)	21·8 (19·9 to 23·8)	10·3 (9·19 to 11·4)	-52·7 (-56·3 to -48·4)	14·2 (12·9 to 15·4)
	50-69 years	13·6 (10·0 to 17·5)	10·6 (8·09 to 13·2)	-21·4 (-27·8 to -13·5)	10·8 (8·00 to 14·0)	49·9 (45·6 to 54·8)	26·5 (23·9 to 29·3)	-46·7 (-51·7 to -41·7)	32·4 (29·7 to 35·6)
	70+ years	13·4 (10·7 to 16·6)	10·2 (8·13 to 12·7)	-24·1 (-31·2 to -15·2)	10·7 (8·57 to 13·3)	44·0 (40·2 to 47·5)	28·4 (25·3 to 31·6)	-35·5 (-40·0 to -30·5)	28·6 (26·1 to 30·9)
Montenegro	All Ages	12·2 (10·6 to 14·3)	10·9 (9·57 to 12·7)	-10·4 (-15·7 to -5·22)	9·72 (8·45 to 11·4)	8·51 (6·91 to 10·9)	7·28 (5·65 to 9·53)	-14·4 (-27·7 to -1·58)	5·53 (4·49 to 7·12)
	Under 5	2·70 (2·02 to 3·46)	2·21 (1·67 to 2·82)	-17·9 (-26·0 to -6·36)	2·16 (1·62 to 2·76)	0·0383 (0·0287 to 0·0491)	0·0240 (0·0169 to 0·0318)	-37·1 (-50·9 to -20·0)	0·0249 (0·0186 to 0·0319)
	5-14 years	1·99 (1·32 to 2·84)	1·74 (1·13 to 2·56)	-12·1 (-21·4 to -1·59)	1·59 (1·05 to 2·28)	0·0253 (0·0183 to 0·0358)	0·0171 (0·0107 to 0·0264)	-33·1 (-44·2 to -18·8)	0·0165 (0·0119 to 0·0233)
	15-49 years	10·2 (8·49 to 13·0)	9·11 (7·47 to 11·3)	-10·8 (-17·1 to -3·12)	8·17 (6·79 to 10·4)	1·54 (1·17 to 1·96)	1·12 (0·840 to 1·51)	-27·1 (-41·0 to -12·4)	0·998 (0·758 to 1·28)
	50-69 years	19·5 (14·7 to 25·2)	17·3 (13·0 to 21·9)	-11·2 (-19·7 to -2·90)	15·6 (11·7 to 20·2)	3·74 (2·95 to 4·94)	2·92 (2·17 to 3·96)	-21·7 (-35·4 to -6·93)	2·43 (1·92 to 3·21)
	70+ years	23·1 (18·0 to 28·8)	19·8 (15·7 to 24·8)	-13·8 (-21·2 to -4·10)	18·4 (14·4 to 23·0)	3·17 (2·60 to 3·94)	3·20 (2·42 to 4·00)	0·995 (-14·8 to 16·8)	2·06 (1·69 to 2·56)
North Macedonia	All Ages	17·6 (15·3 to 20·4)	17·0 (14·7 to 19·7)	-3·42 (-8·09 to 2·46)	14·1 (12·2 to 16·3)	58·7 (49·4 to 70·9)	34·3 (26·9 to 45·1)	-41·4 (-51·7 to -20·8)	38·1 (32·1 to 46·1)
	Under 5	3·16 (2·49 to 4·20)	2·63 (2·06 to 3·37)	-16·4 (-26·2 to -6·26)	2·53 (1·99 to 3·36)	0·229 (0·185 to 0·275)	0·0830 (0·0647 to 0·102)	-63·6 (-72·3 to -51·5)	0·149 (0·120 to 0·179)
	5-14 years	2·77 (1·82 to 4·05)	2·35 (1·59 to 3·46)	-15·0 (-24·2 to -5·55)	2·22 (1·46 to 3·24)	0·146 (0·116 to 0·176)	0·0966 (0·0717 to 0·130)	-33·6 (-46·3 to -17·7)	0·0948 (0·0752 to 0·115)
	15-49 years	13·1 (10·6 to 16·4)	12·4 (9·92 to 15·6)	-5·29 (-12·9 to 3·30)	10·5 (8·50 to 13·1)	10·6 (8·80 to 12·3)	4·77 (3·62 to 6·75)	-54·8 (-64·2 to -31·3)	6·86 (5·72 to 8·02)
	50-69 years	29·9 (22·3 to 37·5)	28·1 (21·1 to 35·7)	-5·81 (-13·0 to 5·20)	23·9 (17·8 to 30·0)	24·4 (19·7 to 31·4)	12·3 (9·13 to 17·9)	-49·1 (-60·3 to -24·1)	15·9 (12·8 to 20·4)
	70+ years	40·5 (31·5 to 52·2)	37·0 (28·5 to 46·7)	-8·32 (-16·6 to 2·10)	32·4 (25·2 to 41·8)	23·3 (19·6 to 28·0)	17·0 (13·9 to 21·2)	-26·9 (-38·9 to -12·9)	15·2 (12·8 to 18·2)
Poland	All Ages	16·9 (14·3 to 20·1)	15·2 (13·0 to 18·0)	-9·68 (-12·6 to -6·78)	13·5 (11·4 to 16·1)	632 (612 to 652)	462 (438 to 487)	-26·9 (-30·8 to -23·7)	411 (398 to 424)
	Under 5	2·22 (1·70 to 2·97)	2·03 (1·55 to 2·69)	-8·59 (-14·3 to -3·56)	1·78 (1·36 to 2·38)	0·612 (0·446 to 0·847)	0·346 (0·261 to 0·476)	-43·4 (-46·0 to -40·5)	0·398 (0·290 to 0·550)
	5-14 years	1·98 (1·21 to 2·98)	1·92 (1·19 to 2·96)	-3·04 (-8·75 to 1·44)	1·58 (0·969 to 2·38)	0·286 (0·220 to 0·387)	0·197 (0·146 to 0·262)	-31·2 (-34·1 to -28·5)	0·186 (0·143 to 0·252)
	15-49 years	13·2 (10·5 to 17·0)	12·2 (9·75 to 15·6)	-8·05 (-11·8 to -4·59)	10·6 (8·42 to 13·6)	140 (132 to 152)	97·1 (91·2 to 105)	-30·6 (-34·0 to -27·3)	90·9 (86·0 to 98·5)
	50-69 years	28·0 (20·3 to 37·4)	25·0 (18·2 to 32·9)	-10·7 (-15·5 to -5·79)	22·4 (16·2 to 29·9)	303 (293 to 313)	202 (190 to 214)	-33·3 (-37·7 to -29·5)	197 (190 to 203)

eTable 2. Progress towards the END-TB 2020 milestones in all-form tuberculosis incidence rate per 100,000 population and in deaths due to all-form tuberculosis by age for 204 countries and territories (2020), percent change from 2015 to 2020.

Location	Age group	2015 Rate	2020 Rate	Incidence Percent Change	Incidence Milestone	2015 Deaths	2020 Deaths	Mortality Percent Change	Mortality Milestone
	70+ years	27·6 (21·4 to 35·4)	23·7 (18·8 to 29·9)	-14·1 (-19·2 to -9·91)	22·1 (17·1 to 28·3)	188 (177 to 197)	162 (150 to 172)	-13·7 (-17·8 to -10·1)	122 (115 to 128)
Romania	All Ages	57·4 (50·2 to 65·6)	48·5 (41·9 to 56·8)	-15·5 (-20·2 to -10·3)	45·9 (40·2 to 52·5)	1180 (1130 to 1280)	859 (769 to 943)	-27·5 (-33·7 to -23·2)	770 (732 to 834)
	Under 5	12·6 (9·47 to 16·6)	10·5 (8·38 to 13·5)	-16·6 (-25·5 to -6·35)	10·1 (7·57 to 13·2)	14·4 (13·2 to 15·6)	11·1 (9·63 to 12·7)	-23·0 (-33·0 to -11·9)	9·37 (8·60 to 10·2)
	5-14 years	16·1 (11·7 to 22·6)	13·8 (9·66 to 19·7)	-14·4 (-22·8 to 0·0428)	12·9 (9·34 to 18·1)	3·67 (2·78 to 4·82)	2·23 (1·56 to 3·05)	-39·3 (-44·3 to -33·3)	2·39 (1·81 to 3·14)
	15-49 years	65·2 (52·8 to 79·8)	53·6 (43·8 to 66·3)	-17·8 (-25·5 to -10·7)	52·1 (42·2 to 63·8)	367 (339 to 407)	237 (210 to 263)	-35·3 (-40·9 to -29·3)	238 (220 to 265)
	50-69 years	70·1 (53·0 to 89·3)	61·2 (45·9 to 79·3)	-12·7 (-21·0 to -3·48)	56·1 (42·4 to 71·5)	531 (493 to 580)	374 (335 to 411)	-29·5 (-36·5 to -24·4)	345 (320 to 377)
	70+ years	56·9 (45·6 to 68·7)	49·3 (39·9 to 60·7)	-13·3 (-21·1 to -5·28)	45·5 (36·5 to 55·0)	269 (249 to 289)	234 (208 to 256)	-12·8 (-19·9 to -7·49)	175 (162 to 188)
Serbia	All Ages	13·9 (12·0 to 16·1)	12·6 (11·0 to 14·8)	-8·74 (-14·6 to -3·34)	11·1 (9·62 to 12·9)	162 (139 to 185)	130 (105 to 160)	-19·6 (-29·4 to -3·54)	105 (90·1 to 120)
	Under 5	2·72 (2·08 to 3·57)	2·26 (1·76 to 2·90)	-16·7 (-29·2 to -4·38)	2·18 (1·66 to 2·86)	0·601 (0·483 to 0·733)	0·453 (0·375 to 0·542)	-24·0 (-40·1 to -2·49)	0·391 (0·314 to 0·477)
	5-14 years	3·76 (2·49 to 5·62)	3·42 (2·30 to 4·96)	-8·92 (-19·7 to 5·34)	3·01 (1·99 to 4·49)	1·25 (0·972 to 1·60)	0·784 (0·534 to 1·05)	-36·5 (-59·0 to -9·51)	0·815 (0·632 to 1·04)
	15-49 years	11·0 (9·01 to 13·6)	9·97 (8·05 to 12·4)	-8·90 (-15·7 to -0·560)	8·76 (7·21 to 10·9)	30·7 (26·8 to 34·8)	22·8 (19·2 to 28·2)	-25·5 (-36·2 to -8·84)	19·9 (17·4 to 22·6)
	50-69 years	20·4 (15·4 to 26·0)	18·2 (13·7 to 23·8)	-10·8 (-18·0 to -1·65)	16·4 (12·3 to 20·8)	65·2 (56·0 to 74·3)	49·2 (39·7 to 59·0)	-24·4 (-33·3 to -8·30)	42·4 (36·4 to 48·3)
	70+ years	26·5 (20·7 to 33·1)	23·4 (19·1 to 29·0)	-11·3 (-20·8 to -1·91)	21·2 (16·6 to 26·5)	64·5 (52·9 to 75·9)	57·1 (43·8 to 72·7)	-11·4 (-25·0 to 8·17)	41·9 (34·4 to 49·3)
Slovakia	All Ages	6·50 (5·60 to 7·58)	5·90 (5·13 to 6·92)	-9·19 (-14·8 to -4·29)	5·20 (4·48 to 6·06)	40·2 (34·3 to 47·9)	32·3 (25·9 to 38·8)	-19·5 (-28·5 to -9·71)	26·1 (22·3 to 31·1)
	Under 5	1·41 (1·10 to 1·72)	1·20 (0·916 to 1·56)	-15·1 (-26·0 to -6·24)	1·13 (0·880 to 1·38)	0·188 (0·160 to 0·226)	0·150 (0·116 to 0·188)	-20·2 (-34·6 to -5·61)	0·122 (0·104 to 0·147)
	5-14 years	0·697 (0·461 to 1·01)	0·576 (0·365 to 0·807)	-17·1 (-28·5 to -3·36)	0·557 (0·369 to 0·807)	0·0474 (0·0366 to 0·0613)	0·0372 (0·0274 to 0·0469)	-21·5 (-31·4 to -3·33)	0·0308 (0·0238 to 0·0398)
	15-49 years	5·29 (4·27 to 6·59)	4·81 (3·85 to 6·10)	-9·03 (-16·6 to -1·04)	4·23 (3·42 to 5·27)	7·92 (6·41 to 9·53)	5·47 (4·17 to 6·94)	-31·0 (-39·8 to -19·0)	5·15 (4·16 to 6·20)
	50-69 years	10·2 (7·54 to 13·4)	9·09 (6·88 to 11·9)	-10·5 (-17·7 to -2·25)	8·14 (6·03 to 10·8)	17·8 (14·2 to 22·3)	12·3 (9·35 to 16·7)	-30·6 (-45·3 to -11·2)	11·6 (9·25 to 14·5)
	70+ years	12·2 (9·83 to 15·2)	10·6 (8·72 to 13·0)	-12·5 (-20·1 to -5·36)	9·73 (7·86 to 12·1)	14·3 (11·7 to 17·1)	14·3 (11·4 to 17·4)	0·859 (-13·7 to 14·6)	9·27 (7·60 to 11·1)
Slovenia	All Ages	6·82 (5·94 to 7·71)	6·05 (5·28 to 6·86)	-11·3 (-15·8 to -5·84)	5·46 (4·75 to 6·16)	15·2 (14·0 to 16·3)	8·10 (7·16 to 8·78)	-46·7 (-51·0 to -41·0)	9·87 (9·11 to 10·6)
	Under 5	1·78 (1·30 to 2·36)	1·58 (1·14 to 2·11)	-11·1 (-22·4 to 1·14)	1·42 (1·04 to 1·89)	0·0286 (0·0238 to 0·0337)	0·0117 (0·00973 to 0·0139)	-59·0 (-63·6 to -54·1)	0·0186 (0·0155 to 0·0219)
	5-14 years	1·59 (1·04 to 2·35)	1·46 (0·995 to 2·23)	-8·19 (-16·9 to 2·72)	1·27 (0·831 to 1·88)	0·0269 (0·0202 to 0·0366)	0·0167 (0·0112 to 0·0246)	-38·4 (-44·4 to -32·9)	0·0175 (0·0131 to 0·0238)

eTable 2. Progress towards the END-TB 2020 milestones in all-form tuberculosis incidence rate per 100,000 population and in deaths due to all-form tuberculosis by age for 204 countries and territories (2020), percent change from 2015 to 2020.

Location	Age group	2015 Rate	2020 Rate	Incidence Percent Change	Incidence Milestone	2015 Deaths	2020 Deaths	Mortality Percent Change	Mortality Milestone
	15-49 years	5·34 (4·31 to 6·79)	4·49 (3·58 to 5·70)	-16·0 (-21·8 to -8·99)	4·27 (3·45 to 5·44)	2·10 (1·94 to 2·30)	0·717 (0·639 to 0·808)	-65·9 (-69·3 to -62·5)	1·37 (1·26 to 1·50)
	50-69 years	8·67 (6·62 to 11·0)	7·75 (5·72 to 9·85)	-10·5 (-19·1 to -1·81)	6·93 (5·30 to 8·77)	5·51 (5·14 to 5·96)	2·19 (1·94 to 2·38)	-60·2 (-64·5 to -55·7)	3·58 (3·34 to 3·87)
	70+ years	14·1 (11·0 to 17·0)	12·3 (9·73 to 15·0)	-12·7 (-21·5 to -2·52)	11·3 (8·78 to 13·6)	7·53 (6·70 to 8·16)	5·16 (4·53 to 5·68)	-31·4 (-36·6 to -24·6)	4·89 (4·36 to 5·31)
Eastern Europe	All Ages	86·8 (73·9 to 105)	71·9 (61·5 to 87·9)	-17·2 (-20·8 to -13·8)	69·4 (59·1 to 84·1)	17700 (16800 to 19100)	9950 (9270 to 11000)	-43·8 (-45·9 to -40·7)	11500 (10900 to 12400)
	Under 5	10·4 (8·18 to 13·6)	7·46 (5·90 to 9·88)	-28·0 (-33·1 to -24·1)	8·29 (6·54 to 10·9)	106 (95·2 to 119)	34·0 (28·9 to 40·7)	-67·9 (-69·9 to -65·7)	68·7 (61·9 to 77·4)
	5-14 years	14·9 (9·79 to 21·2)	12·3 (8·11 to 17·8)	-17·4 (-23·6 to -11·7)	11·9 (7·84 to 17·0)	22·6 (19·0 to 27·4)	15·0 (12·3 to 18·3)	-33·9 (-36·2 to -31·8)	14·7 (12·3 to 17·8)
	15-49 years	124 (98·3 to 156)	105 (83·6 to 135)	-15·2 (-19·9 to -11·4)	99·4 (78·7 to 124)	8520 (7680 to 9710)	4570 (4040 to 5300)	-46·4 (-48·0 to -44·0)	5540 (4990 to 6310)
	50-69 years	80·3 (60·0 to 103)	66·0 (49·7 to 87·4)	-17·8 (-22·4 to -13·0)	64·2 (48·0 to 82·2)	7730 (7510 to 7950)	4220 (3960 to 4580)	-45·4 (-48·5 to -41·7)	5020 (4880 to 5170)
	70+ years	43·2 (32·0 to 55·1)	36·6 (28·2 to 46·5)	-15·2 (-21·1 to -9·23)	34·6 (25·6 to 44·1)	1320 (1260 to 1360)	1110 (1040 to 1160)	-15·8 (-18·5 to -12·7)	856 (819 to 882)
Belarus	All Ages	35·2 (30·3 to 41·0)	28·6 (23·8 to 34·3)	-18·6 (-24·2 to -13·3)	28·1 (24·2 to 32·8)	391 (362 to 428)	252 (222 to 284)	-35·4 (-43·6 to -28·1)	254 (235 to 278)
	Under 5	4·09 (3·26 to 5·51)	3·29 (2·65 to 4·14)	-19·4 (-28·8 to -6·45)	3·27 (2·61 to 4·41)	2·24 (1·86 to 2·71)	0·927 (0·743 to 1·21)	-58·5 (-64·1 to -51·0)	1·46 (1·21 to 1·76)
	5-14 years	2·65 (1·76 to 3·87)	2·47 (1·61 to 3·60)	-6·65 (-18·5 to 5·29)	2·12 (1·41 to 3·10)	0·463 (0·384 to 0·577)	0·349 (0·284 to 0·436)	-24·6 (-29·9 to -18·9)	0·301 (0·250 to 0·375)
	15-49 years	43·8 (36·3 to 54·7)	36·3 (28·4 to 46·2)	-17·2 (-24·9 to -10·2)	35·1 (29·1 to 43·8)	162 (147 to 181)	88·6 (76·1 to 102)	-45·2 (-51·7 to -39·6)	105 (95·6 to 118)
	50-69 years	41·9 (31·4 to 53·1)	33·7 (25·2 to 43·3)	-19·6 (-28·2 to -13·1)	33·5 (25·1 to 42·5)	188 (173 to 208)	133 (115 to 150)	-28·9 (-39·5 to -19·5)	122 (112 to 135)
	70+ years	27·7 (22·3 to 35·3)	23·6 (18·6 to 29·1)	-14·7 (-23·7 to -4·97)	22·2 (17·8 to 28·3)	39·0 (36·3 to 41·7)	29·3 (26·1 to 33·3)	-24·8 (-31·5 to -16·8)	25·4 (23·6 to 27·1)
Estonia	All Ages	18·7 (16·0 to 22·0)	15·7 (13·4 to 18·4)	-16·1 (-20·7 to -11·3)	14·9 (12·8 to 17·6)	45·3 (41·4 to 48·9)	26·5 (23·5 to 30·2)	-41·5 (-45·9 to -36·4)	29·5 (26·9 to 31·8)
	Under 5	4·08 (3·20 to 5·27)	3·22 (2·50 to 4·10)	-21·0 (-30·1 to -8·54)	3·27 (2·56 to 4·22)	0·224 (0·183 to 0·259)	0·105 (0·0839 to 0·139)	-53·0 (-58·9 to -43·9)	0·146 (0·119 to 0·168)
	5-14 years	4·36 (2·94 to 6·63)	3·82 (2·55 to 5·70)	-12·2 (-23·6 to 2·35)	3·49 (2·36 to 5·30)	0·0961 (0·0715 to 0·118)	0·0608 (0·0417 to 0·0856)	-37·0 (-42·3 to -26·0)	0·0625 (0·0465 to 0·0764)
	15-49 years	22·0 (18·1 to 27·7)	18·5 (14·9 to 24·0)	-15·6 (-21·4 to -10·2)	17·6 (14·5 to 22·2)	18·9 (15·4 to 22·0)	9·98 (7·69 to 13·0)	-47·3 (-51·8 to -40·8)	12·3 (10·0 to 14·3)
	50-69 years	22·7 (17·3 to 28·1)	19·1 (14·0 to 24·1)	-16·1 (-23·4 to -9·58)	18·2 (13·9 to 22·5)	18·1 (16·7 to 19·4)	11·2 (10·3 to 12·3)	-38·0 (-43·8 to -31·7)	11·8 (10·9 to 12·6)
	70+ years	17·1 (13·2 to 21·4)	14·8 (11·3 to 18·5)	-13·2 (-21·2 to -3·52)	13·7 (10·6 to 17·2)	8·00 (7·21 to 8·65)	5·17 (4·56 to 5·73)	-35·4 (-40·7 to -30·6)	5·20 (4·68 to 5·62)
Latvia	All Ages	34·6 (30·2 to 40·3)	28·6 (24·6 to 34·1)	-17·4 (-21·7 to -11·5)	27·7 (24·2 to 32·2)	95·1 (85·4 to 106)	55·2 (48·9 to 62·7)	-41·9 (-45·7 to -37·2)	61·8 (55·5 to 68·7)

eTable 2. Progress towards the END-TB 2020 milestones in all-form tuberculosis incidence rate per 100,000 population and in deaths due to all-form tuberculosis by age for 204 countries and territories (2020), percent change from 2015 to 2020.

Location	Age group	2015 Rate	2020 Rate	Incidence Percent Change	Incidence Milestone	2015 Deaths	2020 Deaths	Mortality Percent Change	Mortality Milestone
	Under 5	6·76 (5·24 to 8·90)	4·91 (3·79 to 6·39)	-27·3 (-36·0 to -18·8)	5·41 (4·19 to 7·12)	0·465 (0·373 to 0·580)	0·196 (0·157 to 0·250)	-57·7 (-61·4 to -53·2)	0·302 (0·242 to 0·377)
	5-14 years	8·12 (5·69 to 11·9)	6·43 (4·28 to 9·06)	-20·6 (-30·7 to -7·30)	6·49 (4·55 to 9·50)	0·166 (0·120 to 0·227)	0·114 (0·0765 to 0·168)	-31·4 (-36·9 to -25·1)	0·108 (0·0780 to 0·148)
	15-49 years	46·1 (37·6 to 57·0)	38·5 (30·7 to 47·8)	-16·5 (-22·2 to -9·06)	36·9 (30·1 to 45·6)	36·0 (30·4 to 43·9)	20·6 (16·7 to 26·1)	-42·9 (-47·0 to -38·8)	23·4 (19·7 to 28·5)
	50-69 years	37·7 (28·0 to 48·4)	31·6 (23·9 to 41·3)	-15·9 (-23·3 to -7·10)	30·1 (22·4 to 38·7)	43·3 (39·5 to 47·5)	23·5 (21·3 to 26·0)	-45·7 (-49·7 to -40·4)	28·1 (25·6 to 30·9)
	70+ years	21·5 (16·9 to 27·2)	19·1 (14·8 to 22·9)	-10·7 (-23·8 to 1·13)	17·2 (13·5 to 21·8)	15·2 (14·0 to 16·3)	10·9 (9·91 to 11·7)	-28·6 (-34·5 to -22·5)	9·91 (9·11 to 10·6)
Lithuania	All Ages	47·3 (41·2 to 55·5)	39·7 (34·6 to 46·5)	-16·0 (-20·0 to -10·8)	37·9 (32·9 to 44·4)	210 (198 to 224)	105 (97·1 to 115)	-49·9 (-53·9 to -45·9)	136 (129 to 146)
	Under 5	8·61 (6·68 to 11·4)	6·10 (4·65 to 7·83)	-29·0 (-38·1 to -17·9)	6·89 (5·35 to 9·09)	0·843 (0·725 to 0·977)	0·246 (0·209 to 0·295)	-70·8 (-74·8 to -66·6)	0·548 (0·471 to 0·635)
	5-14 years	12·5 (8·93 to 17·2)	9·41 (6·49 to 13·8)	-24·8 (-34·0 to -15·6)	10·0 (7·15 to 13·7)	0·218 (0·178 to 0·263)	0·163 (0·124 to 0·221)	-25·5 (-34·0 to -13·1)	0·141 (0·115 to 0·171)
	15-49 years	55·4 (45·7 to 69·3)	47·5 (39·4 to 59·6)	-14·1 (-19·3 to -7·52)	44·3 (36·5 to 55·4)	69·5 (64·8 to 75·1)	34·7 (31·5 to 39·3)	-50·0 (-54·0 to -45·5)	45·2 (42·1 to 48·8)
	50-69 years	59·4 (44·4 to 74·0)	49·5 (37·8 to 63·8)	-16·6 (-23·2 to -8·40)	47·5 (35·5 to 59·2)	102 (94·1 to 109)	46·5 (42·8 to 51·7)	-54·3 (-59·0 to -50·5)	66·2 (61·2 to 70·9)
	70+ years	36·2 (29·0 to 45·1)	30·4 (24·0 to 37·6)	-15·8 (-26·3 to -9·41)	29·0 (23·2 to 36·1)	37·2 (34·0 to 40·4)	23·2 (20·7 to 25·6)	-37·6 (-42·4 to -32·4)	24·2 (22·1 to 26·3)
Republic of Moldova	All Ages	86·3 (74·8 to 102)	69·8 (58·6 to 83·6)	-19·1 (-24·9 to -12·1)	69·1 (59·8 to 81·3)	276 (256 to 293)	144 (132 to 154)	-48·0 (-51·2 to -45·3)	180 (166 to 190)
	Under 5	13·1 (10·0 to 17·7)	9·96 (7·82 to 13·5)	-23·7 (-35·6 to -8·95)	10·5 (8·01 to 14·1)	1·80 (1·42 to 2·19)	0·900 (0·701 to 1·12)	-49·9 (-57·3 to -42·4)	1·17 (0·921 to 1·43)
	5-14 years	17·9 (12·2 to 24·6)	14·4 (10·1 to 20·0)	-19·7 (-29·3 to -6·59)	14·3 (9·73 to 19·7)	0·409 (0·335 to 0·501)	0·223 (0·179 to 0·291)	-45·6 (-50·5 to -40·8)	0·266 (0·218 to 0·326)
	15-49 years	108 (91·4 to 136)	88·8 (70·5 to 110)	-17·9 (-25·5 to -8·54)	86·6 (73·1 to 109)	114 (104 to 124)	54·6 (49·1 to 61·5)	-52·3 (-55·4 to -48·9)	74·4 (67·6 to 80·7)
	50-69 years	96·5 (72·4 to 122)	75·8 (59·7 to 94·2)	-21·3 (-29·9 to -11·7)	77·2 (57·9 to 97·8)	138 (127 to 149)	70·5 (64·1 to 76·7)	-48·9 (-52·8 to -44·6)	89·8 (82·5 to 96·6)
	70+ years	48·1 (38·5 to 61·5)	40·2 (30·8 to 50·5)	-16·1 (-26·9 to -4·37)	38·4 (30·8 to 49·2)	21·6 (19·8 to 23·3)	17·4 (16·2 to 18·9)	-19·1 (-25·9 to -13·6)	14·0 (12·9 to 15·2)
Russian Federation	All Ages	89·2 (76·5 to 108)	73·7 (62·4 to 90·6)	-17·4 (-22·1 to -13·5)	71·4 (61·2 to 86·6)	12600 (11900 to 13400)	6460 (5990 to 6990)	-48·6 (-50·5 to -47·2)	8160 (7740 to 8730)
	Under 5	11·3 (8·88 to 14·9)	7·94 (6·20 to 10·5)	-29·7 (-34·5 to -25·6)	9·03 (7·11 to 11·9)	83·9 (75·5 to 95·1)	24·3 (20·7 to 29·3)	-71·1 (-73·0 to -68·8)	54·5 (49·1 to 61·8)
	5-14 years	16·8 (11·0 to 23·8)	13·7 (8·95 to 19·8)	-18·5 (-24·8 to -12·5)	13·4 (8·79 to 19·0)	17·1 (14·1 to 21·0)	11·0 (8·95 to 13·5)	-35·8 (-37·3 to -34·1)	11·1 (9·18 to 13·6)
	15-49 years	130 (102 to 162)	110 (86·6 to 140)	-15·3 (-20·6 to -11·0)	104 (82·0 to 129)	6320 (5760 to 7130)	3100 (2720 to 3510)	-50·9 (-52·5 to -49·6)	4110 (3740 to 4630)
	50-69 years	79·5 (59·2 to 103)	65·4 (49·1 to 86·2)	-17·7 (-22·9 to -12·9)	63·6 (47·4 to 82·1)	5250 (5170 to 5370)	2520 (2400 to 2650)	-52·0 (-54·1 to -50·0)	3420 (3360 to 3490)

eTable 2. Progress towards the END-TB 2020 milestones in all-form tuberculosis incidence rate per 100,000 population and in deaths due to all-form tuberculosis by age for 204 countries and territories (2020), percent change from 2015 to 2020.

Location	Age group	2015 Rate	2020 Rate	Incidence Percent Change	Incidence Milestone	2015 Deaths	2020 Deaths	Mortality Percent Change	Mortality Milestone
	70+ years	43·1 (31·6 to 54·2)	36·4 (27·9 to 46·4)	-15·4 (-22·1 to -8·43)	34·5 (25·3 to 43·4)	879 (834 to 903)	797 (737 to 835)	-9·29 (-12·6 to -6·64)	571 (542 to 587)
Ukraine	All Ages	96·7 (81·2 to 119)	81·0 (66·7 to 97·5)	-16·1 (-23·0 to -9·26)	77·3 (65·0 to 95·0)	4120 (3780 to 4600)	2910 (2470 to 3500)	-29·4 (-38·4 to -17·5)	2680 (2460 to 2990)
	Under 5	8·42 (6·51 to 11·2)	6·61 (5·29 to 9·02)	-21·2 (-31·7 to -12·5)	6·73 (5·21 to 8·96)	16·2 (14·5 to 18·2)	7·33 (6·17 to 9·02)	-54·7 (-59·8 to -49·0)	10·5 (9·45 to 11·9)
	5-14 years	11·4 (7·36 to 16·9)	9·93 (6·60 to 15·1)	-13·1 (-26·5 to -1·38)	9·15 (5·88 to 13·6)	4·20 (3·62 to 4·91)	3·09 (2·58 to 3·79)	-26·5 (-34·1 to -16·0)	2·73 (2·35 to 3·19)
	15-49 years	135 (108 to 174)	115 (90·9 to 145)	-14·9 (-24·1 to -4·98)	108 (86·8 to 140)	1800 (1540 to 2150)	1260 (1050 to 1550)	-30·0 (-37·8 to -22·0)	1170 (1000 to 1400)
	50-69 years	94·5 (65·9 to 125)	78·0 (56·2 to 104)	-17·3 (-28·4 to -6·87)	75·6 (52·7 to 99·8)	1990 (1810 to 2160)	1410 (1130 to 1690)	-28·8 (-40·4 to -12·7)	1290 (1180 to 1400)
	70+ years	49·2 (36·2 to 65·5)	41·9 (31·4 to 53·8)	-14·7 (-23·1 to -3·69)	39·4 (29·0 to 52·4)	318 (295 to 340)	226 (191 to 260)	-28·8 (-38·8 to -18·6)	206 (192 to 221)
High-income	All Ages	10·3 (9·20 to 11·7)	9·40 (8·37 to 10·8)	-8·37 (-10·2 to -6·76)	8·21 (7·36 to 9·37)	13200 (11800 to 14300)	12500 (11000 to 13600)	-5·23 (-7·26 to -3·09)	8590 (7690 to 9300)
	Under 5	2·19 (1·73 to 2·81)	1·81 (1·40 to 2·33)	-17·6 (-20·4 to -14·6)	1·76 (1·38 to 2·25)	39·6 (33·6 to 45·4)	26·0 (21·6 to 30·7)	-34·4 (-37·6 to -30·7)	25·7 (21·9 to 29·5)
	5-14 years	2·23 (1·52 to 3·24)	2·15 (1·46 to 3·15)	-3·77 (-6·41 to -1·03)	1·78 (1·22 to 2·59)	24·6 (20·7 to 28·1)	19·7 (16·8 to 22·7)	-19·7 (-23·5 to -15·7)	16·0 (13·5 to 18·3)
	15-49 years	9·96 (8·10 to 12·4)	8·86 (7·21 to 11·0)	-11·0 (-12·7 to -8·89)	7·96 (6·48 to 9·88)	1800 (1440 to 2120)	1500 (1200 to 1810)	-16·3 (-18·8 to -13·8)	1170 (935 to 1380)
	50-69 years	11·3 (8·67 to 13·9)	10·3 (7·81 to 12·7)	-9·40 (-13·6 to -6·49)	9·07 (6·93 to 11·2)	2700 (2490 to 2920)	2350 (2120 to 2540)	-13·1 (-15·6 to -11·0)	1750 (1620 to 1900)
	70+ years	20·4 (17·0 to 24·7)	18·3 (15·1 to 22·3)	-10·3 (-15·2 to -6·76)	16·3 (13·6 to 19·8)	8660 (7320 to 9370)	8630 (7120 to 9510)	-0·334 (-3·03 to 2·71)	5630 (4760 to 6090)
Australasia	All Ages	5·62 (4·86 to 6·53)	5·55 (4·77 to 6·48)	-1·17 (-4·72 to 2·82)	4·50 (3·89 to 5·22)	93·8 (84·7 to 101)	81·2 (72·7 to 88·7)	-13·4 (-17·0 to -9·46)	61·0 (55·1 to 65·7)
	Under 5	2·23 (1·73 to 2·92)	2·03 (1·58 to 2·66)	-8·96 (-18·0 to -0·615)	1·79 (1·39 to 2·34)	0·278 (0·254 to 0·306)	0·187 (0·165 to 0·212)	-32·5 (-38·7 to -25·9)	0·181 (0·165 to 0·199)
	5-14 years	1·20 (0·812 to 1·72)	1·25 (0·825 to 1·80)	4·32 (-2·31 to 11·2)	0·957 (0·650 to 1·37)	0·166 (0·145 to 0·189)	0·106 (0·0876 to 0·131)	-36·3 (-41·8 to -30·5)	0·108 (0·0945 to 0·123)
	15-49 years	7·36 (6·01 to 9·21)	7·29 (5·91 to 9·19)	-0·869 (-5·35 to 5·13)	5·89 (4·81 to 7·37)	7·82 (6·73 to 9·43)	6·17 (5·16 to 7·58)	-21·2 (-25·1 to -18·1)	5·08 (4·37 to 6·13)
	50-69 years	4·77 (3·67 to 6·03)	4·77 (3·65 to 5·96)	0·0117 (-7·91 to 7·75)	3·82 (2·94 to 4·82)	21·8 (20·3 to 23·9)	18·1 (16·4 to 20·1)	-17·0 (-21·3 to -12·4)	14·2 (13·2 to 15·5)
	70+ years	7·08 (5·69 to 8·72)	6·62 (5·31 to 8·19)	-6·39 (-13·0 to 0·341)	5·66 (4·55 to 6·98)	63·7 (55·4 to 69·1)	56·6 (48·3 to 61·7)	-11·1 (-15·6 to -7·38)	41·4 (36·0 to 44·9)
Australia	All Ages	5·60 (4·84 to 6·45)	5·34 (4·59 to 6·20)	-4·68 (-8·65 to 0·0311)	4·48 (3·87 to 5·16)	77·2 (69·5 to 83·9)	75·7 (67·6 to 82·8)	-2·00 (-6·09 to 2·62)	50·2 (45·2 to 54·5)
	Under 5	2·04 (1·56 to 2·72)	1·86 (1·40 to 2·43)	-8·82 (-19·6 to 0·891)	1·63 (1·25 to 2·17)	0·219 (0·197 to 0·244)	0·163 (0·143 to 0·185)	-25·7 (-33·2 to -18·1)	0·142 (0·128 to 0·159)
	5-14 years	0·996 (0·674 to 1·42)	0·956 (0·631 to 1·38)	-4·00 (-12·0 to 4·11)	0·797 (0·539 to 1·14)	0·138 (0·121 to 0·159)	0·0948 (0·0784 to 0·118)	-31·6 (-37·6 to -24·7)	0·0899 (0·0784 to 0·103)

eTable 2. Progress towards the END-TB 2020 milestones in all-form tuberculosis incidence rate per 100,000 population and in deaths due to all-form tuberculosis by age for 204 countries and territories (2020), percent change from 2015 to 2020.

Location	Age group	2015 Rate	2020 Rate	Incidence Percent Change	Incidence Milestone	2015 Deaths	2020 Deaths	Mortality Percent Change	Mortality Milestone
	15-49 years	7·34 (5·97 to 9·12)	6·89 (5·62 to 8·56)	-6·11 (-11·0 to -0·393)	5·87 (4·77 to 7·30)	6·12 (5·25 to 7·36)	5·59 (4·69 to 6·88)	-8·70 (-12·9 to -4·60)	3·98 (3·41 to 4·78)
	50-69 years	4·82 (3·65 to 6·09)	4·76 (3·69 to 6·03)	-1·12 (-9·67 to 7·78)	3·85 (2·92 to 4·88)	17·1 (15·7 to 18·8)	16·4 (14·8 to 18·2)	-4·03 (-8·89 to 1·73)	11·1 (10·2 to 12·2)
	70+ years	7·13 (5·68 to 8·82)	6·76 (5·42 to 8·46)	-5·21 (-13·7 to 1·90)	5·71 (4·54 to 7·06)	53·7 (46·7 to 58·7)	53·5 (45·5 to 58·3)	-0·407 (-5·49 to 3·90)	34·9 (30·3 to 38·2)
New Zealand	All Ages	5·72 (4·96 to 6·73)	6·63 (5·67 to 8·00)	15·9 (9·30 to 22·5)	4·57 (3·97 to 5·38)	16·6 (15·3 to 17·6)	5·49 (4·97 to 5·89)	-66·9 (-68·8 to -65·2)	10·8 (9·93 to 11·5)
	Under 5	3·21 (2·53 to 4·15)	2·87 (2·19 to 3·85)	-10·5 (-17·8 to -2·41)	2·57 (2·02 to 3·32)	0·0588 (0·0514 to 0·0656)	0·0249 (0·0213 to 0·0284)	-57·5 (-63·6 to -51·3)	0·0382 (0·0334 to 0·0427)
	5-14 years	2·15 (1·48 to 3·09)	2·66 (1·78 to 4·00)	23·7 (9·41 to 36·0)	1·72 (1·18 to 2·47)	0·0272 (0·0242 to 0·0310)	0·0108 (0·00918 to 0·0125)	-60·5 (-64·3 to -56·5)	0·0177 (0·0157 to 0·0201)
	15-49 years	7·43 (6·06 to 9·33)	9·27 (7·30 to 11·9)	24·7 (15·6 to 36·8)	5·95 (4·85 to 7·46)	1·70 (1·47 to 2·03)	0·579 (0·475 to 0·720)	-66·0 (-68·8 to -63·9)	1·10 (0·953 to 1·32)
	50-69 years	4·53 (3·38 to 5·84)	4·81 (3·49 to 6·32)	6·03 (-3·35 to 16·4)	3·63 (2·71 to 4·67)	4·75 (4·49 to 5·02)	1·73 (1·59 to 1·84)	-63·6 (-65·8 to -60·9)	3·09 (2·92 to 3·26)
	70+ years	6·79 (5·29 to 8·39)	5·90 (4·40 to 7·55)	-13·2 (-24·0 to -3·96)	5·43 (4·23 to 6·71)	10·0 (8·94 to 10·9)	3·14 (2·68 to 3·46)	-68·6 (-70·8 to -66·5)	6·51 (5·81 to 7·07)
High-income Asia Pacific	All Ages	29·1 (25·3 to 33·1)	26·6 (23·3 to 29·9)	-8·53 (-12·1 to -5·50)	23·3 (20·3 to 26·5)	6210 (5260 to 6870)	6200 (5080 to 6930)	-0·164 (-4·45 to 3·78)	4040 (3420 to 4460)
	Under 5	1·06 (0·827 to 1·34)	0·876 (0·674 to 1·13)	-16·9 (-23·9 to -10·8)	0·844 (0·662 to 1·08)	7·34 (6·38 to 8·13)	4·55 (3·91 to 5·14)	-38·0 (-42·0 to -33·5)	4·77 (4·15 to 5·28)
	5-14 years	1·07 (0·730 to 1·56)	0·897 (0·602 to 1·32)	-16·1 (-22·7 to -8·96)	0·855 (0·584 to 1·25)	3·26 (2·92 to 3·67)	2·71 (2·39 to 3·00)	-16·8 (-24·3 to -7·63)	2·12 (1·90 to 2·38)
	15-49 years	19·9 (16·5 to 24·3)	16·4 (13·4 to 20·4)	-17·8 (-21·2 to -13·8)	15·9 (13·2 to 19·4)	252 (229 to 281)	177 (159 to 196)	-29·5 (-34·6 to -23·2)	164 (149 to 183)
	50-69 years	37·6 (29·2 to 47·0)	34·0 (26·1 to 42·7)	-9·50 (-16·6 to -3·97)	30·1 (23·4 to 37·6)	827 (766 to 919)	657 (593 to 734)	-20·6 (-26·7 to -14·5)	538 (498 to 598)
	70+ years	65·2 (54·0 to 80·1)	57·0 (46·6 to 69·5)	-12·4 (-18·6 to -7·50)	52·1 (43·2 to 64·1)	5120 (4220 to 5700)	5360 (4290 to 6040)	4·66 (0·0628 to 9·19)	3330 (2740 to 3700)
Brunei Darussalam	All Ages	60·3 (54·0 to 69·4)	56·3 (49·8 to 65·0)	-6·50 (-11·6 to -1·93)	48·2 (43·2 to 55·5)	18·0 (15·7 to 21·5)	17·9 (15·0 to 21·4)	-0·602 (-15·8 to 18·4)	11·7 (10·2 to 14·0)
	Under 5	4·45 (3·55 to 5·82)	3·77 (2·88 to 4·78)	-15·1 (-26·5 to -4·06)	3·56 (2·84 to 4·66)	0·168 (0·137 to 0·218)	0·135 (0·104 to 0·179)	-19·5 (-34·1 to 0·793)	0·109 (0·0894 to 0·142)
	5-14 years	5·23 (3·39 to 7·77)	4·65 (3·08 to 6·87)	-11·0 (-22·4 to 1·72)	4·19 (2·71 to 6·22)	0·103 (0·0853 to 0·125)	0·0704 (0·0573 to 0·0867)	-31·6 (-43·4 to -17·8)	0·0672 (0·0554 to 0·0816)
	15-49 years	56·1 (46·7 to 68·6)	47·9 (39·4 to 59·3)	-14·6 (-19·5 to -8·28)	44·9 (37·3 to 54·9)	3·61 (3·09 to 4·33)	3·64 (2·97 to 4·35)	0·913 (-11·9 to 20·6)	2·35 (2·01 to 2·81)
	50-69 years	125 (99·7 to 155)	117 (89·2 to 141)	-6·98 (-15·4 to 2·05)	100 (79·7 to 124)	4·69 (3·85 to 5·64)	5·07 (4·07 to 6·28)	8·40 (-8·83 to 28·1)	3·05 (2·51 to 3·66)
	70+ years	342 (269 to 436)	295 (225 to 378)	-13·5 (-24·1 to -3·78)	274 (215 to 349)	9·45 (7·98 to 11·4)	8·97 (7·14 to 11·3)	-4·88 (-21·5 to 11·8)	6·14 (5·19 to 7·40)
Japan	All Ages	14·4 (12·4 to 16·4)	12·4 (10·8 to 14·1)	-14·3 (-19·1 to -10·4)	11·5 (9·96 to 13·1)	3450 (2880 to 3800)	3700 (2980 to 4140)	6·95 (3·07 to 9·37)	2250 (1870 to 2470)

eTable 2. Progress towards the END-TB 2020 milestones in all-form tuberculosis incidence rate per 100,000 population and in deaths due to all-form tuberculosis by age for 204 countries and territories (2020), percent change from 2015 to 2020.

Location	Age group	2015 Rate	2020 Rate	Incidence Percent Change	Incidence Milestone	2015 Deaths	2020 Deaths	Mortality Percent Change	Mortality Milestone
	Under 5	0·573 (0·436 to 0·747)	0·569 (0·425 to 0·742)	-0·718 (-7·72 to 4·78)	0·458 (0·349 to 0·597)	3·08 (2·35 to 3·70)	2·70 (2·14 to 3·06)	-12·1 (-19·1 to -5·83)	2·00 (1·53 to 2·41)
	5-14 years	0·382 (0·234 to 0·573)	0·404 (0·243 to 0·641)	5·57 (-3·09 to 15·6)	0·306 (0·187 to 0·458)	0·913 (0·698 to 1·11)	0·893 (0·708 to 1·02)	-1·88 (-8·02 to 2·45)	0·593 (0·454 to 0·723)
	15-49 years	7·11 (5·85 to 8·91)	5·95 (4·80 to 7·69)	-16·3 (-22·2 to -9·99)	5·69 (4·68 to 7·13)	55·2 (49·6 to 60·9)	45·5 (40·6 to 49·3)	-17·6 (-19·5 to -16·1)	35·9 (32·2 to 39·6)
	50-69 years	16·2 (12·6 to 20·7)	11·7 (8·82 to 15·1)	-27·7 (-33·0 to -22·6)	13·0 (10·1 to 16·6)	279 (260 to 301)	220 (202 to 235)	-21·4 (-23·3 to -19·4)	182 (169 to 195)
	70+ years	37·7 (31·3 to 45·6)	31·4 (25·5 to 38·7)	-16·7 (-21·0 to -12·0)	30·2 (25·0 to 36·5)	3120 (2540 to 3450)	3430 (2710 to 3860)	9·96 (6·33 to 12·4)	2020 (1650 to 2240)
Republic of Korea	All Ages	64·2 (56·9 to 73·3)	60·2 (52·5 to 67·6)	-6·20 (-11·6 to -1·96)	51·4 (45·5 to 58·6)	2670 (2290 to 3030)	2430 (2030 to 2830)	-8·87 (-17·4 to -0·998)	1730 (1490 to 1970)
	Under 5	2·01 (1·55 to 2·59)	1·58 (1·18 to 2·02)	-21·2 (-32·4 to -10·3)	1·61 (1·24 to 2·07)	3·83 (3·33 to 4·29)	1·54 (1·32 to 1·76)	-59·7 (-66·2 to -52·4)	2·49 (2·17 to 2·79)
	5-14 years	2·55 (1·78 to 3·60)	1·94 (1·34 to 2·76)	-23·9 (-32·5 to -16·2)	2·04 (1·42 to 2·88)	2·07 (1·75 to 2·42)	1·61 (1·38 to 1·87)	-21·9 (-33·0 to -8·70)	1·35 (1·14 to 1·57)
	15-49 years	43·5 (36·0 to 52·8)	35·7 (29·1 to 44·6)	-18·0 (-22·5 to -12·7)	34·8 (28·8 to 42·3)	185 (163 to 214)	123 (108 to 141)	-33·6 (-39·6 to -25·0)	121 (106 to 139)
	50-69 years	90·0 (69·4 to 113)	79·4 (60·7 to 100)	-11·7 (-20·8 to -4·17)	72·0 (55·5 to 90·0)	522 (461 to 618)	418 (349 to 499)	-19·8 (-29·3 to -9·46)	339 (300 to 402)
	70+ years	211 (172 to 264)	187 (150 to 227)	-11·2 (-20·7 to -2·78)	168 (138 to 212)	1960 (1630 to 2280)	1890 (1540 to 2250)	-3·40 (-13·8 to 7·20)	1270 (1060 to 1480)
Singapore	All Ages	41·8 (36·6 to 49·3)	39·5 (34·8 to 46·0)	-5·35 (-10·4 to 0·773)	33·4 (29·3 to 39·5)	70·2 (64·3 to 76·3)	56·2 (51·6 to 61·6)	-19·9 (-23·8 to -16·4)	45·6 (41·8 to 49·6)
	Under 5	1·83 (1·39 to 2·35)	1·48 (1·12 to 1·95)	-19·0 (-29·6 to -9·31)	1·47 (1·11 to 1·88)	0·262 (0·218 to 0·333)	0·174 (0·132 to 0·237)	-33·8 (-41·7 to -25·2)	0·171 (0·142 to 0·217)
	5-14 years	1·89 (1·24 to 2·81)	1·64 (1·08 to 2·46)	-13·5 (-25·5 to -3·32)	1·52 (0·993 to 2·25)	0·173 (0·153 to 0·200)	0·133 (0·114 to 0·156)	-22·9 (-31·9 to -14·8)	0·113 (0·0997 to 0·130)
	15-49 years	35·0 (28·5 to 44·0)	32·1 (26·3 to 39·9)	-8·21 (-15·6 to -0·712)	28·0 (22·8 to 35·2)	7·31 (6·44 to 8·23)	5·10 (4·54 to 5·80)	-30·2 (-34·3 to -26·1)	4·75 (4·19 to 5·35)
	50-69 years	63·2 (48·6 to 78·0)	54·7 (41·3 to 67·9)	-13·3 (-20·5 to -4·87)	50·5 (38·9 to 62·4)	21·0 (18·4 to 23·8)	13·7 (12·2 to 15·3)	-34·7 (-39·6 to -28·7)	13·6 (12·0 to 15·5)
	70+ years	122 (96·4 to 148)	108 (86·1 to 135)	-11·3 (-20·3 to -2·30)	97·7 (77·1 to 118)	41·5 (36·3 to 46·0)	37·1 (32·1 to 41·6)	-10·6 (-16·0 to -5·77)	26·9 (23·6 to 29·9)
High-income North America	All Ages	2·74 (2·41 to 3·19)	2·88 (2·54 to 3·36)	5·21 (1·23 to 8·47)	2·19 (1·93 to 2·55)	950 (869 to 1050)	1050 (965 to 1150)	10·7 (8·84 to 12·9)	617 (565 to 680)
	Under 5	1·27 (0·985 to 1·62)	1·02 (0·775 to 1·33)	-19·5 (-24·2 to -16·2)	1·02 (0·788 to 1·30)	3·73 (3·41 to 4·17)	3·42 (3·02 to 3·93)	-8·26 (-13·2 to -3·92)	2·42 (2·22 to 2·71)
	5-14 years	0·590 (0·397 to 0·858)	0·670 (0·450 to 0·995)	13·5 (4·92 to 21·6)	0·472 (0·317 to 0·687)	1·52 (1·35 to 1·78)	1·80 (1·62 to 2·09)	18·3 (15·4 to 21·9)	0·987 (0·875 to 1·16)
	15-49 years	2·76 (2·28 to 3·47)	2·84 (2·29 to 3·56)	2·53 (-3·15 to 7·74)	2·21 (1·82 to 2·78)	156 (130 to 194)	174 (150 to 210)	11·6 (7·25 to 16·7)	102 (84·7 to 126)
	50-69 years	3·28 (2·48 to 4·07)	3·37 (2·55 to 4·26)	2·77 (-2·00 to 8·54)	2·62 (1·98 to 3·26)	333 (298 to 377)	363 (329 to 409)	9·12 (6·71 to 11·5)	216 (193 to 245)

eTable 2. Progress towards the END-TB 2020 milestones in all-form tuberculosis incidence rate per 100,000 population and in deaths due to all-form tuberculosis by age for 204 countries and territories (2020), percent change from 2015 to 2020.

Location	Age group	2015 Rate	2020 Rate	Incidence Percent Change	Incidence Milestone	2015 Deaths	2020 Deaths	Mortality Percent Change	Mortality Milestone
	70+ years	4·94 (3·93 to 6·17)	5·27 (4·21 to 6·69)	6·65 (2·80 to 10·6)	3·95 (3·15 to 4·94)	456 (396 to 488)	509 (442 to 546)	11·8 (10·0 to 13·8)	296 (258 to 317)
Canada	All Ages	5·01 (4·53 to 5·79)	5·24 (4·67 to 6·03)	4·77 (0·550 to 9·25)	4·00 (3·62 to 4·63)	126 (112 to 142)	124 (113 to 140)	-0·971 (-5·30 to 3·15)	81·7 (73·0 to 92·6)
	Under 5	1·74 (1·31 to 2·21)	1·73 (1·30 to 2·30)	-0·665 (-11·9 to 8·84)	1·39 (1·05 to 1·77)	0·324 (0·277 to 0·405)	0·291 (0·237 to 0·358)	-10·1 (-18·4 to -2·59)	0·210 (0·180 to 0·263)
	5-14 years	1·19 (0·783 to 1·71)	1·28 (0·837 to 1·83)	7·38 (-6·64 to 19·4)	0·952 (0·626 to 1·37)	0·167 (0·137 to 0·217)	0·171 (0·140 to 0·220)	2·41 (-4·45 to 9·76)	0·109 (0·0893 to 0·141)
	15-49 years	5·28 (4·39 to 6·49)	5·47 (4·45 to 6·73)	3·57 (-1·91 to 8·58)	4·23 (3·51 to 5·19)	19·2 (15·0 to 25·6)	20·0 (16·0 to 25·9)	4·39 (-1·33 to 9·94)	12·5 (9·74 to 16·7)
	50-69 years	5·08 (3·78 to 6·33)	5·27 (4·05 to 6·73)	3·85 (-3·25 to 11·9)	4·06 (3·02 to 5·07)	35·6 (29·9 to 42·2)	32·8 (27·9 to 38·5)	-7·68 (-13·2 to -1·95)	23·1 (19·4 to 27·4)
	70+ years	9·26 (7·39 to 11·2)	9·44 (7·52 to 11·3)	2·06 (-8·33 to 12·1)	7·41 (5·91 to 8·95)	70·4 (62·2 to 76·7)	71·1 (62·0 to 77·1)	1·02 (-3·11 to 5·53)	45·7 (40·4 to 49·9)
Greenland	All Ages	130 (112 to 154)	111 (96·4 to 133)	-14·6 (-19·1 to -8·25)	104 (89·2 to 123)	2·71 (2·23 to 3·15)	2·64 (2·23 to 3·13)	-2·61 (-17·5 to 9·96)	1·76 (1·45 to 2·05)
	Under 5	13·9 (10·7 to 17·3)	11·2 (8·44 to 14·7)	-18·8 (-31·2 to -4·07)	11·1 (8·57 to 13·8)	0·0244 (0·0184 to 0·0311)	0·0181 (0·0135 to 0·0225)	-25·6 (-37·4 to -12·1)	0·0159 (0·0120 to 0·0202)
	5-14 years	25·2 (16·4 to 38·0)	19·2 (12·5 to 29·0)	-23·6 (-31·7 to -13·7)	20·1 (13·1 to 30·4)	0·00269 (0·00199 to 0·00376)	0·00216 (0·00162 to 0·00302)	-19·0 (-37·6 to 1·72)	0·00175 (0·00130 to 0·00244)
	15-49 years	132 (106 to 164)	98·3 (79·5 to 122)	-25·5 (-31·2 to -19·2)	106 (84·7 to 132)	0·418 (0·338 to 0·491)	0·329 (0·266 to 0·390)	-21·3 (-31·6 to -8·94)	0·272 (0·220 to 0·319)
	50-69 years	201 (148 to 257)	183 (140 to 230)	-8·96 (-15·9 to -0·249)	161 (118 to 206)	1·25 (1·02 to 1·46)	1·27 (1·06 to 1·48)	1·61 (-13·3 to 13·7)	0·815 (0·663 to 0·947)
	70+ years	245 (190 to 312)	245 (183 to 313)	0·212 (-10·4 to 12·3)	196 (152 to 250)	1·01 (0·775 to 1·27)	1·02 (0·799 to 1·34)	1·01 (-24·3 to 28·3)	0·659 (0·504 to 0·822)
United States of America	All Ages	2·46 (2·16 to 2·89)	2·59 (2·29 to 3·04)	5·30 (0·929 to 8·83)	1·97 (1·73 to 2·31)	822 (752 to 901)	925 (849 to 1010)	12·6 (10·7 to 14·8)	534 (489 to 586)
	Under 5	1·22 (0·945 to 1·57)	0·951 (0·717 to 1·24)	-22·3 (-27·1 to -19·0)	0·978 (0·756 to 1·25)	3·38 (3·12 to 3·73)	3·11 (2·77 to 3·56)	-7·94 (-13·4 to -3·84)	2·20 (2·03 to 2·42)
	5-14 years	0·528 (0·349 to 0·773)	0·604 (0·402 to 0·905)	14·2 (3·22 to 24·3)	0·422 (0·279 to 0·618)	1·35 (1·20 to 1·56)	1·62 (1·47 to 1·87)	20·4 (17·0 to 24·7)	0·877 (0·783 to 1·01)
	15-49 years	2·47 (2·04 to 3·12)	2·53 (2·01 to 3·22)	2·48 (-4·64 to 8·51)	1·98 (1·63 to 2·49)	137 (115 to 168)	154 (133 to 183)	12·8 (8·40 to 17·9)	88·9 (75·0 to 109)
	50-69 years	3·03 (2·29 to 3·79)	3·11 (2·31 to 4·01)	2·61 (-3·31 to 8·67)	2·42 (1·83 to 3·03)	296 (265 to 333)	329 (299 to 368)	11·2 (8·80 to 13·6)	192 (172 to 217)
	70+ years	4·38 (3·48 to 5·51)	4·73 (3·74 to 6·06)	7·86 (3·06 to 12·7)	3·51 (2·78 to 4·41)	384 (332 to 412)	437 (377 to 470)	13·8 (11·9 to 15·9)	250 (216 to 268)
Southern Latin America	All Ages	20·0 (17·3 to 23·3)	19·0 (16·7 to 22·1)	-4·59 (-7·92 to -1·61)	16·0 (13·9 to 18·6)	2230 (1880 to 2490)	2000 (1700 to 2230)	-10·3 (-13·7 to -6·49)	1450 (1220 to 1620)
	Under 5	4·76 (3·64 to 6·08)	4·12 (3·21 to 5·37)	-13·5 (-21·7 to -5·16)	3·81 (2·92 to 4·86)	19·7 (17·0 to 22·0)	11·7 (9·78 to 13·5)	-40·4 (-45·8 to -34·5)	12·8 (11·0 to 14·3)
	5-14 years	5·99 (4·29 to 8·26)	5·81 (4·10 to 8·07)	-3·05 (-11·6 to 5·87)	4·79 (3·43 to 6·61)	14·4 (12·1 to 16·2)	11·2 (9·56 to 13·1)	-22·2 (-27·6 to -15·2)	9·35 (7·86 to 10·5)

eTable 2. Progress towards the END-TB 2020 milestones in all-form tuberculosis incidence rate per 100,000 population and in deaths due to all-form tuberculosis by age for 204 countries and territories (2020), percent change from 2015 to 2020.

Location	Age group	2015 Rate	2020 Rate	Incidence Percent Change	Incidence Milestone	2015 Deaths	2020 Deaths	Mortality Percent Change	Mortality Milestone
	15-49 years	24·1 (19·7 to 29·5)	22·8 (18·5 to 27·7)	-5·09 (-8·72 to -1·04)	19·2 (15·7 to 23·6)	929 (705 to 1090)	802 (615 to 959)	-13·7 (-18·2 to -8·64)	604 (458 to 709)
	50-69 years	24·8 (19·0 to 30·9)	23·1 (17·7 to 28·7)	-6·67 (-11·6 to -1·20)	19·8 (15·2 to 24·7)	711 (620 to 791)	666 (575 to 743)	-6·40 (-10·8 to -2·11)	462 (403 to 514)
	70+ years	25·1 (20·3 to 30·8)	22·7 (18·6 to 27·9)	-9·33 (-14·4 to -3·99)	20·1 (16·2 to 24·6)	554 (503 to 588)	509 (462 to 539)	-8·16 (-11·7 to -5·10)	360 (327 to 382)
Argentina	All Ages	20·7 (17·9 to 24·0)	19·8 (17·2 to 22·7)	-4·15 (-8·31 to 0·162)	16·5 (14·3 to 19·2)	1460 (1210 to 1660)	1330 (1100 to 1530)	-8·68 (-12·6 to -3·53)	949 (790 to 1080)
	Under 5	5·33 (3·98 to 6·78)	4·63 (3·56 to 6·08)	-13·1 (-23·9 to -3·71)	4·27 (3·19 to 5·42)	14·5 (12·2 to 16·3)	8·93 (7·19 to 10·5)	-38·2 (-43·5 to -30·9)	9·40 (7·92 to 10·6)
	5-14 years	7·05 (5·02 to 9·69)	6·86 (4·78 to 9·62)	-2·72 (-13·3 to 7·75)	5·64 (4·02 to 7·75)	10·7 (8·71 to 12·2)	8·75 (7·34 to 10·4)	-17·9 (-24·2 to -9·70)	6·94 (5·66 to 7·96)
	15-49 years	26·1 (21·2 to 32·2)	24·8 (20·2 to 29·8)	-5·03 (-10·1 to -0·0343)	20·9 (17·0 to 25·8)	666 (499 to 797)	578 (435 to 711)	-13·2 (-17·7 to -7·63)	433 (324 to 518)
	50-69 years	24·4 (18·8 to 30·5)	22·9 (17·4 to 28·4)	-6·15 (-12·8 to 2·39)	19·5 (15·1 to 24·4)	472 (408 to 528)	449 (383 to 511)	-4·84 (-9·41 to 0·414)	307 (266 to 343)
	70+ years	21·4 (17·2 to 26·8)	19·7 (15·8 to 24·4)	-7·83 (-16·1 to -0·250)	17·1 (13·7 to 21·5)	296 (273 to 316)	287 (264 to 311)	-3·04 (-7·24 to 2·08)	193 (177 to 205)
Chile	All Ages	15·8 (14·0 to 18·6)	15·1 (13·2 to 17·8)	-4·43 (-8·50 to 0·105)	12·7 (11·2 to 14·8)	682 (610 to 733)	592 (517 to 637)	-13·3 (-17·7 to -8·00)	444 (397 to 476)
	Under 5	2·89 (2·24 to 3·75)	2·53 (1·94 to 3·25)	-12·4 (-23·7 to -1·01)	2·31 (1·79 to 3·00)	4·72 (4·22 to 5·35)	2·49 (2·18 to 2·83)	-47·1 (-54·0 to -40·3)	3·07 (2·74 to 3·47)
	5-14 years	2·41 (1·61 to 3·36)	2·49 (1·69 to 3·58)	3·46 (-7·37 to 12·7)	1·93 (1·29 to 2·69)	3·39 (3·02 to 3·88)	2·25 (2·01 to 2·53)	-33·5 (-41·7 to -26·2)	2·20 (1·96 to 2·52)
	15-49 years	16·0 (13·2 to 19·7)	15·3 (12·6 to 19·0)	-4·51 (-9·50 to 1·67)	12·8 (10·6 to 15·8)	234 (185 to 262)	198 (157 to 226)	-15·4 (-22·3 to -7·92)	152 (121 to 171)
	50-69 years	22·9 (17·6 to 28·5)	21·3 (16·2 to 26·8)	-6·95 (-13·5 to -0·426)	18·3 (14·0 to 22·8)	209 (186 to 226)	191 (167 to 208)	-8·51 (-14·9 to -2·13)	136 (121 to 147)
	70+ years	32·8 (26·6 to 39·9)	28·5 (23·1 to 35·6)	-12·8 (-21·3 to -4·41)	26·2 (21·3 to 31·9)	231 (205 to 249)	198 (176 to 217)	-14·4 (-18·4 to -9·45)	150 (133 to 162)
Uruguay	All Ages	32·5 (28·0 to 37·5)	30·5 (26·2 to 35·5)	-6·10 (-11·6 to -2·03)	26·0 (22·4 to 30·0)	86·9 (76·5 to 101)	75·3 (66·1 to 86·5)	-13·4 (-19·0 to -8·31)	56·5 (49·7 to 65·7)
	Under 5	5·70 (4·22 to 7·41)	4·76 (3·41 to 6·37)	-16·3 (-29·6 to -1·72)	4·56 (3·38 to 5·93)	0·509 (0·442 to 0·598)	0·306 (0·241 to 0·369)	-39·9 (-48·4 to -30·4)	0·331 (0·287 to 0·388)
	5-14 years	8·21 (5·60 to 11·7)	7·76 (5·33 to 11·2)	-5·49 (-16·4 to 6·12)	6·56 (4·48 to 9·36)	0·329 (0·274 to 0·396)	0·190 (0·151 to 0·233)	-42·3 (-49·6 to -36·3)	0·214 (0·178 to 0·257)
	15-49 years	41·7 (33·7 to 50·3)	38·8 (31·5 to 48·5)	-6·81 (-13·9 to -0·818)	33·3 (27·0 to 40·2)	29·1 (22·3 to 38·5)	25·5 (19·8 to 33·3)	-12·2 (-17·6 to -6·68)	18·9 (14·5 to 25·0)
	50-69 years	37·8 (28·2 to 46·9)	35·2 (26·9 to 43·6)	-6·86 (-15·2 to 1·76)	30·3 (22·5 to 37·5)	30·3 (26·6 to 34·8)	25·5 (22·3 to 29·8)	-15·8 (-23·0 to -7·75)	19·7 (17·3 to 22·6)
	70+ years	30·4 (24·6 to 37·2)	27·4 (21·7 to 34·1)	-9·71 (-17·4 to -0·0112)	24·3 (19·7 to 29·8)	26·7 (23·2 to 29·4)	23·7 (20·3 to 26·3)	-10·9 (-17·8 to -3·11)	17·3 (15·1 to 19·1)
Western Europe	All Ages	7·18 (6·22 to 8·29)	6·37 (5·54 to 7·38)	-11·3 (-12·9 to -9·76)	5·74 (4·98 to 6·63)	3730 (3350 to 4100)	3190 (2830 to 3460)	-14·5 (-16·8 to -12·5)	2420 (2180 to 2660)

eTable 2. Progress towards the END-TB 2020 milestones in all-form tuberculosis incidence rate per 100,000 population and in deaths due to all-form tuberculosis by age for 204 countries and territories (2020), percent change from 2015 to 2020.

Location	Age group	2015 Rate	2020 Rate	Incidence Percent Change	Incidence Milestone	2015 Deaths	2020 Deaths	Mortality Percent Change	Mortality Milestone
	Under 5	2·89 (2·20 to 3·79)	2·36 (1·79 to 3·07)	-18·4 (-21·8 to -14·8)	2·31 (1·76 to 3·03)	8·52 (6·35 to 11·2)	6·07 (4·49 to 8·26)	-28·8 (-31·9 to -26·1)	5·54 (4·13 to 7·29)
	5-14 years	3·56 (2·38 to 5·32)	3·29 (2·20 to 4·90)	-7·67 (-10·5 to -4·78)	2·84 (1·91 to 4·26)	5·24 (4·04 to 6·88)	3·92 (2·98 to 5·12)	-25·1 (-27·4 to -23·0)	3·41 (2·63 to 4·47)
	15-49 years	9·55 (7·70 to 11·9)	8·65 (6·87 to 10·7)	-9·50 (-11·6 to -7·53)	7·64 (6·16 to 9·53)	450 (357 to 585)	344 (267 to 451)	-23·7 (-26·3 to -21·4)	293 (232 to 380)
	50-69 years	5·12 (3·82 to 6·34)	4·50 (3·37 to 5·58)	-12·2 (-14·5 to -9·85)	4·10 (3·06 to 5·07)	805 (720 to 933)	642 (565 to 742)	-20·3 (-22·4 to -18·0)	523 (468 to 607)
	70+ years	7·61 (6·22 to 9·40)	6·57 (5·37 to 8·01)	-13·6 (-16·2 to -11·1)	6·08 (4·98 to 7·52)	2460 (2130 to 2620)	2190 (1880 to 2360)	-10·9 (-13·7 to -8·76)	1600 (1390 to 1700)
Andorra	All Ages	6·04 (5·15 to 7·07)	5·19 (4·31 to 6·17)	-14·0 (-20·5 to -8·56)	4·83 (4·12 to 5·66)	0·109 (0·0882 to 0·135)	0·105 (0·0790 to 0·141)	-3·15 (-20·7 to 15·0)	0·0708 (0·0573 to 0·0881)
	Under 5	4·00 (2·90 to 5·23)	3·22 (2·45 to 4·41)	-19·2 (-33·2 to -5·84)	3·20 (2·32 to 4·18)	0·000127 (0·000100 to 0·000166)	0·0000925 (0·0000680 to 0·000120)	-26·8 (-38·5 to -9·83)	0·0000825 (0·0000650 to 0·000108)
	5-14 years	1·10 (0·629 to 1·71)	0·859 (0·477 to 1·45)	-21·9 (-39·2 to -5·38)	0·880 (0·503 to 1·37)	0·0000662 (0·0000534 to 0·0000825)	0·0000471 (0·0000371 to 0·0000597)	-28·6 (-41·1 to -15·3)	0·0000430 (0·0000347 to 0·0000536)
	15-49 years	5·65 (4·30 to 7·25)	4·60 (3·43 to 5·93)	-18·5 (-27·3 to -10·2)	4·52 (3·44 to 5·80)	0·0140 (0·0108 to 0·0172)	0·0113 (0·00859 to 0·0145)	-19·5 (-37·6 to -5·89)	0·00910 (0·00704 to 0·0112)
	50-69 years	7·77 (5·59 to 10·4)	6·58 (4·85 to 8·80)	-15·3 (-23·4 to -8·90)	6·21 (4·47 to 8·36)	0·0260 (0·0201 to 0·0330)	0·0250 (0·0174 to 0·0344)	-3·91 (-27·8 to 22·4)	0·0169 (0·0131 to 0·0214)
	70+ years	10·0 (7·91 to 12·5)	8·57 (6·82 to 10·6)	-14·6 (-22·6 to -6·64)	8·04 (6·33 to 10·0)	0·0687 (0·0518 to 0·0861)	0·0691 (0·0506 to 0·0946)	0·685 (-16·5 to 19·6)	0·0447 (0·0337 to 0·0559)
Austria	All Ages	6·17 (5·34 to 7·17)	5·58 (4·83 to 6·39)	-9·52 (-13·5 to -5·56)	4·93 (4·27 to 5·73)	68·3 (61·6 to 74·2)	59·0 (52·4 to 63·8)	-13·6 (-17·9 to -10·1)	44·4 (40·1 to 48·3)
	Under 5	2·58 (1·83 to 3·39)	2·28 (1·70 to 2·98)	-11·4 (-20·2 to 5·36)	2·06 (1·46 to 2·71)	0·0842 (0·0664 to 0·109)	0·0680 (0·0540 to 0·0869)	-19·0 (-25·8 to -11·7)	0·0547 (0·0432 to 0·0711)
	5-14 years	2·28 (1·52 to 3·33)	2·16 (1·42 to 3·24)	-5·20 (-16·5 to 4·54)	1·83 (1·22 to 2·66)	0·0655 (0·0518 to 0·0830)	0·0460 (0·0358 to 0·0598)	-29·9 (-34·7 to -24·6)	0·0426 (0·0337 to 0·0539)
	15-49 years	7·50 (5·99 to 9·37)	7·07 (5·69 to 8·67)	-5·82 (-11·1 to -0·340)	6·00 (4·79 to 7·50)	7·18 (5·69 to 9·31)	5·50 (4·14 to 7·24)	-23·6 (-28·2 to -18·0)	4·67 (3·70 to 6·05)
	50-69 years	5·39 (3·90 to 6·50)	4·56 (3·45 to 5·73)	-15·3 (-22·7 to -7·48)	4·31 (3·12 to 5·20)	17·6 (16·0 to 19·5)	12·6 (11·4 to 14·1)	-28·2 (-32·4 to -23·4)	11·4 (10·4 to 12·7)
	70+ years	7·04 (5·75 to 8·69)	6·21 (5·05 to 7·77)	-11·6 (-18·9 to -2·98)	5·63 (4·60 to 6·96)	43·4 (38·2 to 46·9)	40·8 (35·1 to 44·6)	-6·01 (-10·3 to -0·979)	28·2 (24·8 to 30·5)
Belgium	All Ages	7·53 (6·45 to 8·80)	6·58 (5·68 to 7·62)	-12·7 (-17·1 to -8·55)	6·03 (5·16 to 7·04)	83·1 (74·0 to 91·7)	64·2 (56·3 to 71·1)	-22·7 (-26·3 to -18·8)	54·0 (48·1 to 59·6)
	Under 5	3·15 (2·28 to 4·17)	2·64 (1·93 to 3·53)	-16·0 (-26·2 to -4·62)	2·52 (1·82 to 3·34)	0·139 (0·102 to 0·187)	0·106 (0·0753 to 0·147)	-23·7 (-27·8 to -18·4)	0·0901 (0·0660 to 0·122)
	5-14 years	3·14 (2·00 to 4·50)	2·93 (1·89 to 4·38)	-6·76 (-15·1 to 2·89)	2·51 (1·60 to 3·60)	0·0941 (0·0717 to 0·122)	0·0757 (0·0574 to 0·0987)	-19·5 (-24·3 to -13·7)	0·0612 (0·0466 to 0·0793)
	15-49 years	10·4 (8·33 to 12·8)	9·27 (7·44 to 11·5)	-10·6 (-15·7 to -4·77)	8·30 (6·66 to 10·3)	8·97 (6·95 to 11·8)	6·38 (4·75 to 8·71)	-28·9 (-32·6 to -25·1)	5·83 (4·52 to 7·67)
	50-69 years	5·65 (4·23 to 7·02)	4·86 (3·56 to 6·04)	-14·0 (-20·7 to -7·10)	4·52 (3·38 to 5·62)	17·7 (15·7 to 20·5)	14·6 (12·9 to 16·9)	-17·5 (-23·0 to -11·4)	11·5 (10·2 to 13·3)

eTable 2. Progress towards the END-TB 2020 milestones in all-form tuberculosis incidence rate per 100,000 population and in deaths due to all-form tuberculosis by age for 204 countries and territories (2020), percent change from 2015 to 2020.

Location	Age group	2015 Rate	2020 Rate	Incidence Percent Change	Incidence Milestone	2015 Deaths	2020 Deaths	Mortality Percent Change	Mortality Milestone
	70+ years	7·06 (5·70 to 8·76)	5·79 (4·74 to 7·24)	-17·8 (-26·5 to -10·7)	5·65 (4·56 to 7·01)	56·2 (49·2 to 62·1)	43·0 (36·8 to 47·6)	-23·4 (-27·8 to -20·1)	36·5 (32·0 to 40·4)
Cyprus	All Ages	4·78 (4·22 to 5·59)	4·34 (3·85 to 5·05)	-9·16 (-12·9 to -3·29)	3·82 (3·38 to 4·47)	6·83 (5·74 to 8·26)	6·53 (5·54 to 7·73)	-4·21 (-19·2 to 10·9)	4·44 (3·73 to 5·37)
	Under 5	1·87 (1·38 to 2·42)	1·56 (1·17 to 1·98)	-16·6 (-26·7 to -7·20)	1·50 (1·10 to 1·94)	0·0108 (0·00807 to 0·0146)	0·00989 (0·00722 to 0·0139)	-8·73 (-19·9 to 5·94)	0·00705 (0·00524 to 0·00949)
	5-14 years	1·17 (0·742 to 1·69)	1·07 (0·702 to 1·60)	-8·19 (-16·8 to 3·22)	0·934 (0·594 to 1·35)	0·00789 (0·00612 to 0·0103)	0·00885 (0·00645 to 0·0124)	12·3 (-5·72 to 35·1)	0·00513 (0·00398 to 0·00672)
	15-49 years	4·78 (3·80 to 6·07)	4·09 (3·22 to 5·19)	-14·3 (-20·9 to -8·46)	3·83 (3·04 to 4·85)	0·854 (0·666 to 1·14)	0·791 (0·602 to 1·05)	-7·29 (-18·8 to 6·42)	0·555 (0·433 to 0·742)
	50-69 years	4·89 (3·64 to 6·10)	4·57 (3·46 to 5·64)	-6·35 (-12·9 to 2·03)	3·91 (2·91 to 4·88)	1·57 (1·27 to 1·95)	1·44 (1·18 to 1·80)	-7·82 (-23·6 to 14·1)	1·02 (0·824 to 1·27)
	70+ years	10·9 (8·46 to 13·9)	10·3 (8·01 to 13·0)	-5·76 (-16·2 to 4·20)	8·72 (6·77 to 11·1)	4·39 (3·56 to 5·38)	4·27 (3·56 to 5·19)	-2·31 (-18·5 to 12·6)	2·85 (2·31 to 3·50)
Denmark	All Ages	4·73 (4·05 to 5·60)	4·23 (3·64 to 5·01)	-10·6 (-15·0 to -5·52)	3·79 (3·24 to 4·48)	24·6 (22·2 to 27·0)	20·5 (18·2 to 22·1)	-16·8 (-21·3 to -12·1)	16·0 (14·4 to 17·5)
	Under 5	2·24 (1·61 to 2·96)	2·06 (1·51 to 2·74)	-7·81 (-20·7 to 5·52)	1·79 (1·29 to 2·37)	0·0228 (0·0177 to 0·0297)	0·0184 (0·0140 to 0·0243)	-19·3 (-26·3 to -12·9)	0·0148 (0·0115 to 0·0193)
	5-14 years	1·90 (1·26 to 2·78)	1·90 (1·27 to 2·84)	-0·227 (-9·22 to 10·1)	1·52 (1·01 to 2·22)	0·0186 (0·0152 to 0·0233)	0·0170 (0·0143 to 0·0206)	-8·48 (-15·4 to -1·19)	0·0121 (0·00987 to 0·0151)
	15-49 years	6·31 (5·10 to 7·89)	5·77 (4·61 to 7·05)	-8·47 (-15·6 to -0·147)	5·05 (4·08 to 6·31)	2·13 (1·75 to 2·60)	1·66 (1·38 to 2·06)	-21·7 (-25·3 to -17·4)	1·38 (1·14 to 1·69)
	50-69 years	4·10 (2·90 to 4·98)	3·50 (2·64 to 4·37)	-14·5 (-20·7 to -7·82)	3·28 (2·32 to 3·98)	6·42 (5·83 to 7·11)	5·13 (4·63 to 5·72)	-20·1 (-25·9 to -14·3)	4·17 (3·79 to 4·62)
	70+ years	4·04 (3·26 to 5·09)	3·38 (2·71 to 4·16)	-16·2 (-22·5 to -5·54)	3·23 (2·61 to 4·07)	16·0 (13·8 to 17·5)	13·7 (11·6 to 14·9)	-14·8 (-20·0 to -9·55)	10·4 (8·98 to 11·4)
Finland	All Ages	4·70 (4·21 to 5·33)	4·26 (3·81 to 4·83)	-9·29 (-12·8 to -4·81)	3·76 (3·37 to 4·27)	55·6 (47·1 to 60·2)	48·5 (41·3 to 52·8)	-12·7 (-17·1 to -7·68)	36·1 (30·6 to 39·1)
	Under 5	1·93 (1·44 to 2·60)	1·79 (1·32 to 2·40)	-7·48 (-18·6 to 5·29)	1·55 (1·15 to 2·08)	0·0424 (0·0363 to 0·0470)	0·0304 (0·0259 to 0·0344)	-28·2 (-36·3 to -20·3)	0·0276 (0·0236 to 0·0306)
	5-14 years	1·75 (1·14 to 2·60)	1·79 (1·20 to 2·65)	2·37 (-7·15 to 13·9)	1·40 (0·913 to 2·08)	0·0488 (0·0431 to 0·0549)	0·0305 (0·0259 to 0·0353)	-37·4 (-45·7 to -27·5)	0·0317 (0·0280 to 0·0357)
	15-49 years	4·74 (3·85 to 5·91)	4·39 (3·44 to 5·44)	-7·36 (-13·3 to -2·54)	3·79 (3·08 to 4·73)	4·12 (3·61 to 4·51)	3·91 (3·40 to 4·27)	-5·22 (-11·6 to 1·14)	2·68 (2·35 to 2·93)
	50-69 years	4·02 (3·00 to 5·08)	3·41 (2·52 to 4·28)	-15·1 (-22·1 to -8·82)	3·22 (2·40 to 4·06)	10·6 (9·87 to 11·5)	8·42 (7·75 to 9·19)	-20·7 (-25·6 to -14·9)	6·90 (6·42 to 7·50)
	70+ years	9·39 (7·82 to 11·5)	7·59 (6·17 to 9·46)	-19·1 (-26·4 to -10·4)	7·51 (6·25 to 9·20)	40·7 (33·0 to 45·2)	36·1 (29·1 to 39·8)	-11·3 (-17·0 to -5·65)	26·5 (21·5 to 29·4)
France	All Ages	7·20 (6·34 to 8·20)	6·55 (5·72 to 7·48)	-8·98 (-14·1 to -4·67)	5·76 (5·07 to 6·56)	942 (822 to 1050)	821 (709 to 913)	-12·8 (-17·2 to -8·08)	612 (534 to 685)
	Under 5	2·22 (1·57 to 2·88)	1·91 (1·41 to 2·51)	-13·8 (-25·9 to -0·639)	1·77 (1·25 to 2·31)	2·86 (2·05 to 3·87)	2·13 (1·56 to 2·88)	-25·5 (-30·3 to -20·2)	1·86 (1·34 to 2·52)
	5-14 years	2·22 (1·50 to 3·25)	2·19 (1·46 to 3·18)	-1·36 (-11·4 to 10·2)	1·78 (1·20 to 2·60)	1·75 (1·31 to 2·40)	1·28 (0·965 to 1·71)	-27·1 (-31·7 to -23·2)	1·14 (0·853 to 1·56)

eTable 2. Progress towards the END-TB 2020 milestones in all-form tuberculosis incidence rate per 100,000 population and in deaths due to all-form tuberculosis by age for 204 countries and territories (2020), percent change from 2015 to 2020.

Location	Age group	2015 Rate	2020 Rate	Incidence Percent Change	Incidence Milestone	2015 Deaths	2020 Deaths	Mortality Percent Change	Mortality Milestone
	15-49 years	8·62 (6·94 to 10·6)	8·02 (6·38 to 9·85)	-6·91 (-14·0 to -0·186)	6·89 (5·55 to 8·49)	99·2 (77·6 to 130)	84·4 (64·3 to 112)	-15·0 (-19·5 to -11·4)	64·5 (50·4 to 84·3)
	50-69 years	5·71 (4·26 to 7·14)	5·06 (3·78 to 6·32)	-11·3 (-17·4 to -3·72)	4·57 (3·41 to 5·71)	174 (147 to 215)	139 (115 to 172)	-20·2 (-25·2 to -15·5)	113 (95·7 to 140)
	70+ years	12·3 (10·0 to 15·2)	10·2 (8·42 to 12·5)	-17·1 (-23·4 to -9·63)	9·81 (8·02 to 12·2)	665 (560 to 731)	595 (489 to 654)	-10·5 (-15·5 to -5·05)	432 (364 to 475)
Germany	All Ages	5·60 (4·84 to 6·43)	5·62 (4·82 to 6·57)	0·280 (-4·50 to 5·24)	4·48 (3·87 to 5·14)	504 (453 to 549)	505 (439 to 557)	0·0531 (-3·75 to 4·49)	328 (294 to 357)
	Under 5	2·34 (1·72 to 3·06)	2·14 (1·59 to 2·78)	-8·45 (-20·0 to 1·49)	1·87 (1·37 to 2·45)	0·759 (0·593 to 0·985)	0·654 (0·480 to 0·879)	-14·0 (-20·7 to -9·55)	0·493 (0·385 to 0·640)
	5-14 years	1·81 (1·23 to 2·77)	1·80 (1·18 to 2·64)	-0·501 (-9·76 to 9·21)	1·45 (0·984 to 2·21)	0·409 (0·331 to 0·513)	0·371 (0·280 to 0·483)	-9·51 (-16·5 to -3·25)	0·266 (0·215 to 0·333)
	15-49 years	7·64 (6·21 to 9·52)	8·22 (6·67 to 10·3)	7·57 (0·0831 to 16·7)	6·11 (4·97 to 7·61)	52·1 (43·1 to 65·1)	43·4 (35·2 to 54·8)	-16·7 (-21·1 to -12·3)	33·9 (28·0 to 42·3)
	50-69 years	4·11 (3·01 to 5·13)	3·73 (2·80 to 4·68)	-9·14 (-16·4 to -1·84)	3·29 (2·41 to 4·11)	121 (108 to 139)	115 (99·3 to 133)	-5·32 (-10·3 to 0·492)	78·7 (70·3 to 90·3)
	70+ years	5·60 (4·43 to 6·99)	5·38 (4·37 to 6·62)	-3·90 (-10·5 to 2·16)	4·48 (3·54 to 5·59)	330 (284 to 364)	346 (297 to 381)	4·70 (-0·472 to 10·1)	215 (185 to 236)
Greece	All Ages	4·50 (3·96 to 5·17)	4·10 (3·67 to 4·65)	-8·94 (-12·7 to -5·13)	3·60 (3·17 to 4·14)	118 (105 to 126)	112 (102 to 121)	-5·01 (-10·3 to 0·175)	76·8 (68·3 to 81·9)
	Under 5	1·67 (1·23 to 2·19)	1·42 (1·07 to 1·87)	-14·4 (-24·4 to -2·68)	1·33 (0·982 to 1·75)	0·149 (0·131 to 0·165)	0·110 (0·0961 to 0·122)	-26·4 (-36·6 to -17·3)	0·0970 (0·0851 to 0·107)
	5-14 years	1·44 (0·947 to 2·10)	1·36 (0·893 to 1·97)	-5·17 (-15·5 to 8·35)	1·15 (0·757 to 1·68)	0·104 (0·0914 to 0·117)	0·0670 (0·0576 to 0·0788)	-35·3 (-46·7 to -17·9)	0·0676 (0·0594 to 0·0759)
	15-49 years	4·44 (3·54 to 5·55)	4·09 (3·29 to 5·15)	-7·93 (-13·6 to -2·31)	3·55 (2·83 to 4·44)	15·1 (13·5 to 16·5)	11·6 (10·6 to 12·8)	-22·6 (-30·7 to -13·6)	9·80 (8·76 to 10·7)
	50-69 years	4·14 (3·03 to 5·17)	3·76 (2·85 to 4·57)	-9·04 (-16·6 to -0·312)	3·31 (2·43 to 4·14)	22·7 (21·0 to 24·5)	22·7 (20·9 to 25·3)	-0·0335 (-8·47 to 7·50)	14·8 (13·6 to 15·9)
	70+ years	8·03 (6·59 to 9·81)	6·91 (5·71 to 8·43)	-13·8 (-20·1 to -5·76)	6·43 (5·27 to 7·85)	80·1 (67·9 to 87·6)	77·6 (67·3 to 84·9)	-2·98 (-8·05 to 3·93)	52·0 (44·1 to 56·9)
Iceland	All Ages	4·48 (3·81 to 5·34)	3·84 (3·28 to 4·54)	-14·2 (-18·7 to -10·6)	3·58 (3·05 to 4·27)	1·64 (1·41 to 1·84)	1·53 (1·32 to 1·73)	-6·81 (-12·8 to -0·204)	1·07 (0·913 to 1·20)
	Under 5	1·84 (1·30 to 2·56)	1·65 (1·21 to 2·26)	-10·4 (-20·8 to 6·00)	1·48 (1·04 to 2·05)	0·00367 (0·00299 to 0·00470)	0·00316 (0·00249 to 0·00396)	-13·8 (-21·9 to -3·32)	0·00238 (0·00194 to 0·00305)
	5-14 years	2·34 (1·56 to 3·71)	2·19 (1·45 to 3·40)	-6·66 (-18·2 to 5·40)	1·88 (1·25 to 2·97)	0·00222 (0·00183 to 0·00280)	0·00246 (0·00172 to 0·00314)	11·0 (-17·7 to 36·0)	0·00145 (0·00119 to 0·00182)
	15-49 years	5·71 (4·43 to 7·34)	4·77 (3·68 to 6·07)	-16·4 (-21·3 to -11·2)	4·57 (3·55 to 5·87)	0·183 (0·145 to 0·248)	0·207 (0·160 to 0·278)	13·5 (6·97 to 20·4)	0·119 (0·0944 to 0·161)
	50-69 years	3·16 (2·37 to 4·04)	2·78 (2·06 to 3·52)	-11·7 (-20·2 to -2·41)	2·52 (1·90 to 3·23)	0·314 (0·281 to 0·344)	0·295 (0·271 to 0·328)	-6·16 (-14·1 to 0·836)	0·204 (0·183 to 0·223)
	70+ years	6·39 (5·15 to 8·01)	5·36 (4·38 to 6·67)	-16·0 (-23·3 to -7·90)	5·11 (4·12 to 6·41)	1·14 (0·933 to 1·29)	1·02 (0·833 to 1·17)	-10·3 (-16·4 to -2·82)	0·740 (0·607 to 0·837)
Ireland	All Ages	5·90 (5·11 to 6·87)	5·06 (4·36 to 5·90)	-14·2 (-18·3 to -9·50)	4·72 (4·09 to 5·50)	29·7 (26·7 to 31·8)	25·5 (22·5 to 27·8)	-14·2 (-19·2 to -7·36)	19·3 (17·4 to 20·7)

eTable 2. Progress towards the END-TB 2020 milestones in all-form tuberculosis incidence rate per 100,000 population and in deaths due to all-form tuberculosis by age for 204 countries and territories (2020), percent change from 2015 to 2020.

Location	Age group	2015 Rate	2020 Rate	Incidence Percent Change	Incidence Milestone	2015 Deaths	2020 Deaths	Mortality Percent Change	Mortality Milestone
	Under 5	2·15 (1·58 to 2·85)	1·90 (1·39 to 2·58)	-11·3 (-21·7 to -0·202)	1·72 (1·26 to 2·28)	0·0841 (0·0695 to 0·0964)	0·0613 (0·0504 to 0·0707)	-27·0 (-34·9 to -16·6)	0·0547 (0·0452 to 0·0627)
	5-14 years	2·12 (1·40 to 3·09)	1·99 (1·28 to 2·93)	-5·82 (-14·2 to 7·00)	1·69 (1·12 to 2·47)	0·0523 (0·0441 to 0·0616)	0·0440 (0·0351 to 0·0523)	-15·7 (-27·7 to 0·728)	0·0340 (0·0287 to 0·0401)
	15-49 years	7·49 (5·98 to 9·23)	6·39 (5·02 to 7·80)	-14·6 (-20·5 to -9·04)	5·99 (4·78 to 7·38)	5·36 (4·43 to 6·01)	4·33 (3·56 to 4·87)	-19·1 (-25·6 to -11·7)	3·49 (2·88 to 3·91)
	50-69 years	5·19 (3·93 to 6·33)	4·46 (3·33 to 5·63)	-13·9 (-20·7 to -7·12)	4·15 (3·14 to 5·06)	6·57 (6·03 to 7·08)	5·33 (4·86 to 5·76)	-18·7 (-24·0 to -12·6)	4·27 (3·92 to 4·60)
	70+ years	7·97 (6·55 to 9·76)	6·47 (5·25 to 8·11)	-18·9 (-24·8 to -13·2)	6·38 (5·24 to 7·81)	17·6 (14·8 to 19·1)	15·7 (13·3 to 17·7)	-10·9 (-17·4 to -2·50)	11·5 (9·59 to 12·4)
Israel	All Ages	3·25 (2·79 to 3·87)	2·86 (2·46 to 3·38)	-11·9 (-17·4 to -7·22)	2·60 (2·23 to 3·10)	30·3 (27·1 to 33·3)	29·6 (26·6 to 32·4)	-2·01 (-7·98 to 3·28)	19·7 (17·6 to 21·6)
	Under 5	1·52 (1·12 to 1·97)	1·37 (1·03 to 1·84)	-9·43 (-20·4 to 10·8)	1·21 (0·898 to 1·58)	0·157 (0·123 to 0·207)	0·127 (0·0937 to 0·174)	-19·0 (-26·8 to -10·9)	0·102 (0·0799 to 0·135)
	5-14 years	1·03 (0·689 to 1·53)	1·03 (0·647 to 1·54)	0·420 (-13·3 to 10·7)	0·822 (0·551 to 1·22)	0·0875 (0·0705 to 0·109)	0·0703 (0·0577 to 0·0875)	-19·5 (-25·5 to -10·6)	0·0569 (0·0458 to 0·0712)
	15-49 years	4·20 (3·34 to 5·31)	3·63 (2·88 to 4·56)	-13·5 (-19·7 to -7·94)	3·36 (2·67 to 4·25)	3·89 (3·07 to 4·92)	3·51 (2·81 to 4·44)	-9·72 (-15·3 to -3·35)	2·53 (2·00 to 3·20)
	50-69 years	2·99 (2·24 to 3·71)	2·61 (1·97 to 3·28)	-12·7 (-20·2 to -4·72)	2·39 (1·79 to 2·97)	7·24 (6·62 to 8·01)	6·25 (5·68 to 6·89)	-13·7 (-19·4 to -7·94)	4·71 (4·30 to 5·21)
	70+ years	5·55 (4·49 to 6·98)	4·68 (3·85 to 5·99)	-15·7 (-23·5 to -8·18)	4·44 (3·59 to 5·58)	18·9 (15·9 to 20·8)	19·7 (16·5 to 21·7)	4·27 (-2·77 to 10·1)	12·3 (10·4 to 13·5)
Italy	All Ages	6·40 (5·44 to 7·56)	5·63 (4·79 to 6·70)	-12·0 (-14·1 to -10·3)	5·12 (4·35 to 6·05)	388 (351 to 416)	305 (272 to 327)	-21·5 (-24·6 to -18·9)	252 (228 to 270)
	Under 5	2·73 (2·05 to 3·59)	2·35 (1·76 to 3·15)	-14·0 (-17·3 to -10·1)	2·18 (1·64 to 2·87)	0·431 (0·378 to 0·499)	0·236 (0·198 to 0·267)	-45·3 (-50·8 to -38·2)	0·280 (0·246 to 0·324)
	5-14 years	2·78 (1·80 to 4·31)	2·45 (1·55 to 3·80)	-11·8 (-16·4 to -7·56)	2·22 (1·44 to 3·45)	0·329 (0·288 to 0·385)	0·246 (0·220 to 0·281)	-25·0 (-30·0 to -17·8)	0·214 (0·187 to 0·250)
	15-49 years	8·98 (6·96 to 11·3)	8·07 (6·23 to 10·3)	-10·2 (-13·1 to -7·43)	7·19 (5·57 to 9·02)	38·8 (33·9 to 45·7)	22·2 (18·7 to 27·1)	-42·7 (-45·5 to -40·4)	25·2 (22·1 to 29·7)
	50-69 years	4·41 (3·27 to 5·60)	3·97 (2·94 to 5·01)	-10·0 (-13·2 to -6·76)	3·53 (2·62 to 4·48)	72·4 (66·3 to 80·2)	52·5 (49·0 to 57·0)	-27·5 (-30·3 to -25·1)	47·1 (43·1 to 52·1)
	70+ years	5·63 (4·54 to 7·12)	4·87 (4·00 to 6·31)	-13·4 (-16·7 to -10·2)	4·50 (3·63 to 5·69)	276 (237 to 301)	230 (193 to 249)	-16·8 (-20·0 to -14·0)	180 (154 to 195)
Luxembourg	All Ages	8·67 (7·38 to 10·3)	7·66 (6·49 to 9·11)	-11·6 (-15·8 to -5·62)	6·93 (5·90 to 8·27)	1·62 (1·44 to 1·81)	1·47 (1·31 to 1·64)	-9·31 (-15·6 to -2·33)	1·05 (0·936 to 1·18)
	Under 5	4·27 (3·21 to 5·74)	3·78 (2·78 to 5·16)	-11·4 (-23·2 to -0·444)	3·41 (2·57 to 4·59)	0·00231 (0·00183 to 0·00307)	0·00214 (0·00171 to 0·00257)	-6·89 (-18·7 to 4·94)	0·00150 (0·00119 to 0·00200)
	5-14 years	4·80 (3·18 to 7·28)	4·47 (2·96 to 6·78)	-6·50 (-15·1 to 4·39)	3·84 (2·54 to 5·83)	0·00116 (0·000919 to 0·00149)	0·000735 (0·000603 to 0·000918)	-36·4 (-45·3 to -22·9)	0·000754 (0·000597 to 0·000972)
	15-49 years	11·7 (9·25 to 14·6)	10·6 (8·39 to 13·3)	-9·45 (-14·9 to -1·56)	9·33 (7·40 to 11·7)	0·194 (0·151 to 0·265)	0·154 (0·116 to 0·215)	-20·9 (-26·8 to -14·9)	0·126 (0·0979 to 0·172)
	50-69 years	5·82 (4·26 to 7·38)	4·81 (3·50 to 6·10)	-17·2 (-24·1 to -8·83)	4·65 (3·41 to 5·90)	0·354 (0·311 to 0·394)	0·292 (0·259 to 0·330)	-17·3 (-24·0 to -9·18)	0·230 (0·202 to 0·256)

eTable 2. Progress towards the END-TB 2020 milestones in all-form tuberculosis incidence rate per 100,000 population and in deaths due to all-form tuberculosis by age for 204 countries and territories (2020), percent change from 2015 to 2020.

Location	Age group	2015 Rate	2020 Rate	Incidence Percent Change	Incidence Milestone	2015 Deaths	2020 Deaths	Mortality Percent Change	Mortality Milestone
	70+ years	6·93 (5·60 to 8·96)	5·78 (4·67 to 7·27)	-16·5 (-23·2 to -8·86)	5·54 (4·48 to 7·16)	1·07 (0·924 to 1·19)	1·02 (0·874 to 1·14)	-4·55 (-12·4 to 2·83)	0·696 (0·601 to 0·775)
Malta	All Ages	25·8 (21·3 to 31·4)	21·8 (18·4 to 25·9)	-15·5 (-20·3 to -10·5)	20·6 (17·1 to 25·1)	0·926 (0·846 to 1·01)	0·767 (0·696 to 0·860)	-17·1 (-21·4 to -12·0)	0·602 (0·550 to 0·655)
	Under 5	10·4 (7·62 to 13·9)	9·10 (6·59 to 11·9)	-12·7 (-21·8 to 1·48)	8·35 (6·10 to 11·1)	0·00176 (0·00139 to 0·00235)	0·00142 (0·00109 to 0·00192)	-19·6 (-26·0 to -12·7)	0·00115 (0·000903 to 0·00153)
	5-14 years	12·9 (8·37 to 20·0)	11·8 (7·80 to 18·2)	-8·81 (-18·5 to 1·30)	10·3 (6·70 to 16·0)	0·000958 (0·000794 to 0·00116)	0·00161 (0·00130 to 0·00193)	68·8 (34·4 to 110)	0·000623 (0·000516 to 0·000756)
	15-49 years	43·1 (34·1 to 55·1)	37·2 (29·7 to 46·3)	-13·7 (-19·4 to -7·65)	34·5 (27·3 to 44·1)	0·116 (0·0926 to 0·151)	0·100 (0·0815 to 0·129)	-13·4 (-18·2 to -9·41)	0·0751 (0·0602 to 0·0978)
	50-69 years	10·2 (7·73 to 13·1)	8·73 (6·61 to 11·0)	-14·7 (-21·2 to -8·32)	8·20 (6·19 to 10·5)	0·230 (0·207 to 0·256)	0·155 (0·140 to 0·180)	-32·6 (-37·2 to -27·8)	0·149 (0·134 to 0·166)
	70+ years	11·4 (9·19 to 14·5)	9·49 (7·58 to 11·8)	-16·8 (-25·1 to -9·35)	9·14 (7·35 to 11·6)	0·578 (0·512 to 0·630)	0·510 (0·435 to 0·573)	-11·8 (-16·9 to -5·53)	0·376 (0·333 to 0·410)
Monaco	All Ages	6·84 (5·99 to 7·81)	6·48 (5·71 to 7·42)	-5·32 (-9·51 to -1·97)	5·48 (4·79 to 6·25)	0·623 (0·523 to 0·761)	0·587 (0·477 to 0·727)	-5·72 (-20·3 to 8·49)	0·405 (0·340 to 0·494)
	Under 5	1·61 (1·24 to 2·09)	1·32 (1·01 to 1·75)	-17·7 (-25·7 to -8·27)	1·29 (0·991 to 1·67)	0·000394 (0·000298 to 0·000505)	0·000287 (0·000210 to 0·000357)	-27·0 (-38·9 to -14·9)	0·000256 (0·000194 to 0·000328)
	5-14 years	1·10 (0·690 to 1·66)	0·863 (0·552 to 1·29)	-21·1 (-31·5 to -13·6)	0·878 (0·552 to 1·33)	0·000211 (0·000163 to 0·000267)	0·000155 (0·000124 to 0·000194)	-26·2 (-39·1 to -10·4)	0·000137 (0·000106 to 0·000173)
	15-49 years	6·09 (4·93 to 7·60)	5·59 (4·55 to 7·03)	-8·16 (-14·6 to -1·34)	4·87 (3·94 to 6·08)	0·0484 (0·0365 to 0·0619)	0·0396 (0·0284 to 0·0511)	-18·0 (-34·0 to -2·16)	0·0314 (0·0237 to 0·0402)
	50-69 years	6·67 (4·98 to 8·51)	6·37 (4·76 to 8·03)	-4·33 (-10·7 to 3·97)	5·34 (3·98 to 6·81)	0·129 (0·0995 to 0·165)	0·119 (0·0923 to 0·153)	-7·64 (-22·4 to 8·30)	0·0838 (0·0647 to 0·108)
	70+ years	13·2 (10·9 to 16·5)	12·3 (10·1 to 15·1)	-6·79 (-13·6 to -0·853)	10·5 (8·70 to 13·2)	0·445 (0·368 to 0·567)	0·428 (0·340 to 0·541)	-3·69 (-19·6 to 13·4)	0·289 (0·239 to 0·369)
Netherlands	All Ages	4·98 (4·36 to 5·76)	4·48 (3·88 to 5·14)	-9·88 (-14·5 to -5·72)	3·98 (3·49 to 4·61)	108 (94·2 to 121)	91·1 (80·7 to 103)	-15·4 (-19·2 to -11·8)	70·0 (61·2 to 78·4)
	Under 5	1·73 (1·26 to 2·28)	1·56 (1·14 to 2·08)	-9·81 (-22·5 to 3·86)	1·38 (1·01 to 1·83)	0·230 (0·165 to 0·322)	0·172 (0·123 to 0·243)	-24·8 (-29·4 to -19·9)	0·149 (0·107 to 0·209)
	5-14 years	1·60 (1·05 to 2·33)	1·59 (1·02 to 2·40)	-0·167 (-9·16 to 13·3)	1·28 (0·837 to 1·87)	0·177 (0·129 to 0·244)	0·131 (0·101 to 0·172)	-25·5 (-31·3 to -19·6)	0·115 (0·0842 to 0·159)
	15-49 years	6·83 (5·56 to 8·47)	6·26 (5·08 to 7·70)	-8·33 (-14·2 to -2·15)	5·46 (4·45 to 6·78)	12·2 (8·97 to 16·9)	9·81 (7·23 to 13·7)	-19·8 (-24·1 to -16·3)	7·96 (5·83 to 11·0)
	50-69 years	3·59 (2·71 to 4·45)	3·13 (2·32 to 3·89)	-12·9 (-19·3 to -7·99)	2·87 (2·17 to 3·56)	24·8 (21·4 to 30·0)	19·7 (16·8 to 23·5)	-20·6 (-24·5 to -15·6)	16·1 (13·9 to 19·5)
	70+ years	5·77 (4·74 to 7·11)	4·85 (4·00 to 6·04)	-15·9 (-22·8 to -9·62)	4·62 (3·79 to 5·69)	70·2 (59·3 to 76·8)	61·2 (51·4 to 66·4)	-12·7 (-17·1 to -8·27)	45·6 (38·6 to 49·9)
Norway	All Ages	5·53 (4·61 to 6·52)	4·78 (4·04 to 5·51)	-13·6 (-15·7 to -11·4)	4·43 (3·69 to 5·21)	44·3 (38·7 to 48·1)	45·2 (39·7 to 49·2)	1·87 (-1·64 to 4·72)	28·8 (25·2 to 31·3)
	Under 5	1·35 (0·978 to 1·85)	1·25 (0·909 to 1·72)	-7·70 (-12·7 to -1·47)	1·08 (0·782 to 1·48)	0·0499 (0·0375 to 0·0624)	0·0444 (0·0329 to 0·0552)	-11·0 (-16·0 to -6·68)	0·0324 (0·0244 to 0·0405)

eTable 2. Progress towards the END-TB 2020 milestones in all-form tuberculosis incidence rate per 100,000 population and in deaths due to all-form tuberculosis by age for 204 countries and territories (2020), percent change from 2015 to 2020.

Location	Age group	2015 Rate	2020 Rate	Incidence Percent Change	Incidence Milestone	2015 Deaths	2020 Deaths	Mortality Percent Change	Mortality Milestone
	5-14 years	1·95 (1·18 to 3·04)	1·91 (1·14 to 2·99)	-2·22 (-6·07 to 2·03)	1·56 (0·946 to 2·43)	0·0410 (0·0271 to 0·0552)	0·0390 (0·0269 to 0·0532)	-4·83 (-8·73 to 0·433)	0·0266 (0·0176 to 0·0358)
	15-49 years	7·20 (5·43 to 9·26)	5·98 (4·52 to 7·61)	-17·0 (-19·3 to -14·8)	5·76 (4·34 to 7·41)	2·37 (1·73 to 3·14)	2·36 (1·78 to 3·06)	-0·0269 (-3·12 to 3·54)	1·54 (1·13 to 2·04)
	50-69 years	2·98 (2·21 to 3·79)	2·66 (1·97 to 3·36)	-10·9 (-14·4 to -8·05)	2·39 (1·77 to 3·03)	6·47 (6·04 to 7·07)	6·12 (5·56 to 6·70)	-5·44 (-8·71 to -1·72)	4·21 (3·92 to 4·59)
	70+ years	10·0 (8·20 to 12·5)	8·61 (7·04 to 10·8)	-13·9 (-17·6 to -8·48)	8·02 (6·56 to 9·98)	35·4 (29·9 to 38·6)	36·6 (31·0 to 40·1)	3·37 (-0·367 to 6·50)	23·0 (19·4 to 25·1)
Portugal	All Ages	16·3 (14·1 to 19·0)	14·2 (12·4 to 16·4)	-12·8 (-17·7 to -9·41)	13·0 (11·3 to 15·2)	309 (272 to 353)	271 (240 to 310)	-12·2 (-16·2 to -7·94)	201 (177 to 229)
	Under 5	2·84 (2·14 to 3·73)	2·39 (1·74 to 3·23)	-15·6 (-25·4 to -5·21)	2·27 (1·71 to 2·98)	0·458 (0·327 to 0·638)	0·423 (0·299 to 0·594)	-7·75 (-12·3 to -1·72)	0·298 (0·213 to 0·415)
	5-14 years	3·63 (2·40 to 5·29)	3·42 (2·21 to 5·10)	-5·86 (-15·6 to 8·42)	2·91 (1·92 to 4·23)	0·362 (0·253 to 0·513)	0·309 (0·218 to 0·410)	-14·3 (-21·6 to -7·37)	0·235 (0·164 to 0·333)
	15-49 years	19·2 (15·2 to 23·9)	16·4 (13·3 to 20·0)	-14·6 (-19·7 to -8·76)	15·3 (12·2 to 19·1)	64·4 (47·5 to 88·8)	50·7 (35·9 to 69·9)	-21·2 (-25·8 to -17·5)	41·8 (30·9 to 57·7)
	50-69 years	16·9 (12·5 to 21·1)	14·6 (10·8 to 18·5)	-13·4 (-21·2 to -6·89)	13·5 (9·99 to 16·9)	83·2 (71·7 to 102)	68·6 (57·8 to 82·7)	-17·5 (-22·1 to -11·8)	54·0 (46·6 to 66·0)
	70+ years	18·4 (15·3 to 22·7)	16·3 (13·5 to 20·3)	-11·2 (-18·0 to -4·37)	14·7 (12·2 to 18·2)	161 (143 to 177)	151 (132 to 169)	-5·84 (-11·2 to -0·527)	104 (92·9 to 115)
San Marino	All Ages	2·61 (2·30 to 3·00)	2·54 (2·25 to 2·87)	-2·61 (-6·91 to 2·37)	2·09 (1·84 to 2·40)	0·129 (0·102 to 0·167)	0·128 (0·0898 to 0·169)	-0·946 (-20·3 to 21·9)	0·0842 (0·0662 to 0·109)
	Under 5	0·987 (0·726 to 1·33)	0·827 (0·624 to 1·07)	-16·0 (-25·9 to -2·32)	0·790 (0·581 to 1·07)	0·000165 (0·000126 to 0·000221)	0·000131 (0·0000941 to 0·000181)	-19·9 (-33·7 to 1·13)	0·000107 (0·0000822 to 0·000143)
	5-14 years	0·491 (0·311 to 0·745)	0·401 (0·258 to 0·602)	-18·1 (-29·8 to -4·78)	0·393 (0·249 to 0·596)	0·0000733 (0·0000597 to 0·0000887)	0·0000573 (0·0000431 to 0·0000780)	-21·8 (-35·4 to -5·76)	0·0000477 (0·0000388 to 0·0000577)
	15-49 years	2·53 (2·08 to 3·22)	2·33 (1·92 to 2·94)	-8·17 (-13·6 to -1·28)	2·03 (1·67 to 2·57)	0·00849 (0·00668 to 0·0105)	0·00696 (0·00505 to 0·00897)	-18·0 (-35·7 to -1·55)	0·00552 (0·00434 to 0·00680)
	50-69 years	2·58 (1·91 to 3·18)	2·51 (1·87 to 3·14)	-2·39 (-9·39 to 6·38)	2·06 (1·53 to 2·54)	0·0210 (0·0162 to 0·0278)	0·0194 (0·0131 to 0·0269)	-7·61 (-32·8 to 18·0)	0·0137 (0·0105 to 0·0181)
	70+ years	4·99 (4·12 to 6·12)	4·93 (4·03 to 6·10)	-1·06 (-7·18 to 6·26)	3·99 (3·29 to 4·90)	0·0997 (0·0759 to 0·131)	0·102 (0·0680 to 0·134)	2·05 (-18·2 to 26·7)	0·0648 (0·0493 to 0·0852)
Spain	All Ages	8·05 (6·97 to 9·44)	6·52 (5·67 to 7·65)	-19·0 (-22·9 to -15·2)	6·44 (5·58 to 7·55)	435 (395 to 493)	361 (322 to 401)	-17·1 (-21·5 to -12·3)	283 (257 to 321)
	Under 5	1·99 (1·32 to 2·80)	1·46 (0·997 to 2·09)	-26·6 (-33·9 to -17·7)	1·59 (1·06 to 2·24)	0·895 (0·659 to 1·23)	0·658 (0·457 to 0·939)	-26·7 (-32·2 to -20·9)	0·581 (0·428 to 0·801)
	5-14 years	3·03 (1·93 to 4·35)	2·51 (1·49 to 3·67)	-17·3 (-26·6 to -9·00)	2·43 (1·54 to 3·48)	0·620 (0·461 to 0·833)	0·455 (0·326 to 0·630)	-26·7 (-31·2 to -20·8)	0·403 (0·300 to 0·541)
	15-49 years	9·69 (7·76 to 12·4)	7·78 (6·09 to 9·90)	-19·7 (-24·7 to -14·1)	7·75 (6·21 to 9·91)	56·4 (42·3 to 76·1)	43·8 (31·7 to 60·5)	-22·4 (-27·3 to -17·1)	36·6 (27·5 to 49·5)
	50-69 years	7·48 (5·49 to 9·40)	6·16 (4·49 to 7·75)	-17·7 (-22·8 to -12·4)	5·99 (4·39 to 7·52)	103 (89·9 to 123)	75·9 (63·7 to 88·5)	-26·3 (-31·4 to -21·3)	67·0 (58·4 to 80·1)
	70+ years	9·31 (7·46 to 11·6)	7·65 (6·03 to 9·71)	-17·7 (-24·4 to -12·0)	7·45 (5·97 to 9·26)	274 (237 to 301)	240 (202 to 264)	-12·5 (-17·1 to -7·05)	178 (154 to 196)

eTable 2. Progress towards the END-TB 2020 milestones in all-form tuberculosis incidence rate per 100,000 population and in deaths due to all-form tuberculosis by age for 204 countries and territories (2020), percent change from 2015 to 2020.

Location	Age group	2015 Rate	2020 Rate	Incidence Percent Change	Incidence Milestone	2015 Deaths	2020 Deaths	Mortality Percent Change	Mortality Milestone
Sweden	All Ages	6·84 (5·81 to 8·12)	6·39 (5·27 to 7·48)	-6·70 (-11·0 to -2·17)	5·47 (4·64 to 6·50)	72·9 (64·4 to 80·3)	60·2 (53·3 to 65·3)	-17·4 (-21·2 to -13·3)	47·4 (41·9 to 52·2)
	Under 5	2·66 (1·96 to 3·54)	2·67 (1·93 to 3·54)	0·399 (-9·73 to 8·98)	2·13 (1·57 to 2·83)	0·106 (0·0732 to 0·147)	0·0681 (0·0470 to 0·0946)	-36·0 (-39·4 to -31·8)	0·0692 (0·0476 to 0·0952)
	5-14 years	3·78 (2·44 to 5·62)	3·59 (2·28 to 5·59)	-5·21 (-18·6 to 7·10)	3·03 (1·95 to 4·50)	0·0508 (0·0352 to 0·0712)	0·0347 (0·0245 to 0·0463)	-31·5 (-36·2 to -26·9)	0·0330 (0·0229 to 0·0463)
	15-49 years	9·79 (7·71 to 12·5)	9·16 (6·89 to 11·7)	-6·60 (-13·0 to 0·271)	7·83 (6·17 to 10·0)	3·12 (2·09 to 4·42)	2·41 (1·57 to 3·43)	-22·8 (-27·4 to -18·8)	2·03 (1·36 to 2·87)
	50-69 years	3·42 (2·56 to 4·40)	3·43 (2·57 to 4·46)	0·116 (-6·89 to 8·42)	2·74 (2·05 to 3·52)	8·18 (7·27 to 9·24)	5·91 (5·23 to 6·79)	-27·7 (-31·6 to -24·1)	5·32 (4·73 to 6·01)
	70+ years	7·58 (6·12 to 9·28)	6·56 (5·39 to 8·23)	-13·4 (-18·4 to -7·44)	6·07 (4·90 to 7·42)	61·4 (52·4 to 67·4)	51·7 (44·6 to 56·2)	-15·7 (-19·8 to -11·0)	39·9 (34·1 to 43·8)
Switzerland	All Ages	5·78 (5·06 to 6·76)	5·22 (4·39 to 6·03)	-9·79 (-13·3 to -5·61)	4·63 (4·05 to 5·41)	39·8 (35·3 to 43·3)	34·5 (30·2 to 38·1)	-13·3 (-17·8 to -8·56)	25·9 (23·0 to 28·1)
	Under 5	2·33 (1·67 to 3·05)	2·14 (1·59 to 2·90)	-8·27 (-19·1 to 8·53)	1·87 (1·34 to 2·44)	0·0679 (0·0510 to 0·0921)	0·0495 (0·0397 to 0·0669)	-26·9 (-32·2 to -18·8)	0·0441 (0·0331 to 0·0599)
	5-14 years	2·10 (1·38 to 3·13)	2·15 (1·41 to 3·23)	2·28 (-7·10 to 12·8)	1·68 (1·11 to 2·50)	0·0470 (0·0373 to 0·0603)	0·0375 (0·0296 to 0·0478)	-20·2 (-26·1 to -13·0)	0·0306 (0·0243 to 0·0392)
	15-49 years	8·14 (6·71 to 9·99)	7·53 (6·02 to 9·20)	-7·46 (-12·2 to -1·97)	6·51 (5·37 to 7·99)	4·14 (3·16 to 5·43)	3·03 (2·24 to 4·15)	-27·1 (-31·2 to -22·0)	2·69 (2·06 to 3·53)
	50-69 years	3·71 (2·73 to 4·69)	3·23 (2·37 to 4·08)	-12·9 (-19·5 to -6·75)	2·97 (2·18 to 3·75)	8·70 (7·83 to 10·1)	5·83 (5·05 to 7·04)	-33·0 (-37·4 to -27·5)	5·66 (5·09 to 6·56)
	70+ years	5·34 (4·36 to 6·62)	4·71 (3·85 to 5·81)	-11·7 (-18·1 to -4·11)	4·27 (3·49 to 5·30)	26·9 (22·8 to 29·2)	25·6 (21·1 to 28·1)	-4·72 (-9·90 to 0·580)	17·5 (14·8 to 19·0)
United Kingdom	All Ages	10·2 (8·36 to 11·9)	8·51 (6·98 to 9·98)	-16·2 (-17·4 to -15·0)	8·12 (6·69 to 9·51)	462 (424 to 496)	321 (294 to 344)	-30·5 (-31·2 to -29·7)	300 (275 to 322)
	Under 5	5·72 (4·32 to 7·66)	4·11 (3·07 to 5·49)	-28·2 (-30·5 to -24·0)	4·58 (3·46 to 6·13)	1·96 (1·47 to 2·58)	1·09 (0·813 to 1·48)	-44·1 (-46·0 to -42·1)	1·27 (0·954 to 1·68)
	5-14 years	9·81 (6·36 to 14·7)	8·61 (5·49 to 13·0)	-12·3 (-15·5 to -9·40)	7·85 (5·09 to 11·7)	0·963 (0·766 to 1·22)	0·655 (0·480 to 0·845)	-32·1 (-37·4 to -29·5)	0·626 (0·498 to 0·793)
	15-49 years	14·9 (11·3 to 18·7)	12·7 (9·49 to 15·9)	-15·3 (-17·0 to -13·6)	12·0 (9·07 to 15·0)	69·3 (57·0 to 86·7)	43·2 (34·5 to 55·2)	-37·7 (-39·3 to -36·3)	45·0 (37·0 to 56·4)
	50-69 years	4·34 (3·14 to 5·58)	3·85 (2·79 to 4·95)	-11·4 (-13·4 to -9·33)	3·47 (2·51 to 4·47)	112 (104 to 122)	76·2 (71·2 to 83·5)	-31·6 (-32·5 to -30·6)	72·5 (67·6 to 79·2)
	70+ years	5·76 (4·72 to 7·41)	4·73 (3·89 to 5·98)	-17·9 (-19·9 to -15·7)	4·61 (3·77 to 5·93)	278 (249 to 294)	200 (179 to 212)	-28·1 (-29·0 to -27·2)	181 (162 to 191)
Latin America and Caribbean	All Ages	36·0 (31·7 to 41·0)	34·7 (30·4 to 39·8)	-3·42 (-5·77 to -0·669)	28·8 (25·3 to 32·8)	25800 (22600 to 31200)	24700 (21200 to 29200)	-4·32 (-9·16 to 1·35)	16800 (14700 to 20300)
	Under 5	9·50 (7·94 to 11·7)	8·65 (7·29 to 10·8)	-8·93 (-12·4 to -5·44)	7·60 (6·35 to 9·33)	785 (640 to 986)	590 (460 to 750)	-25·0 (-32·9 to -15·0)	511 (416 to 641)
	5-14 years	7·91 (5·54 to 10·8)	7·87 (5·64 to 11·0)	-0·467 (-5·06 to 5·26)	6·32 (4·43 to 8·65)	347 (289 to 468)	285 (236 to 371)	-17·7 (-23·4 to -9·85)	225 (188 to 304)
	15-49 years	41·3 (34·8 to 50·1)	39·9 (33·5 to 48·5)	-3·55 (-7·06 to -0·0735)	33·1 (27·8 to 40·1)	11400 (9440 to 14200)	10300 (8370 to 12800)	-10·0 (-14·0 to -5·73)	7420 (6140 to 9250)

eTable 2. Progress towards the END-TB 2020 milestones in all-form tuberculosis incidence rate per 100,000 population and in deaths due to all-form tuberculosis by age for 204 countries and territories (2020), percent change from 2015 to 2020.

Location	Age group	2015 Rate	2020 Rate	Incidence Percent Change	Incidence Milestone	2015 Deaths	2020 Deaths	Mortality Percent Change	Mortality Milestone
	50-69 years	54·3 (42·5 to 67·5)	49·9 (38·3 to 63·1)	-8·13 (-12·0 to -3·12)	43·4 (34·0 to 54·0)	7960 (7130 to 9610)	8140 (7040 to 9660)	2·29 (-4·33 to 9·20)	5170 (4630 to 6250)
	70+ years	64·0 (52·0 to 79·1)	55·0 (44·6 to 68·5)	-14·0 (-16·7 to -11·5)	51·2 (41·6 to 63·3)	5290 (4810 to 5960)	5390 (4830 to 6100)	1·98 (-4·94 to 10·3)	3440 (3130 to 3870)
Andean Latin America	All Ages	65·2 (56·7 to 75·5)	64·6 (56·4 to 74·5)	-0·862 (-4·96 to 2·64)	52·2 (45·3 to 60·4)	5200 (4510 to 6100)	4880 (4030 to 6040)	-5·96 (-19·5 to 10·0)	3380 (2930 to 3960)
	Under 5	18·4 (15·3 to 23·4)	16·1 (13·2 to 20·4)	-12·3 (-20·7 to -3·67)	14·7 (12·2 to 18·7)	168 (131 to 203)	110 (86·5 to 139)	-34·5 (-45·7 to -21·5)	109 (84·9 to 132)
	5-14 years	19·7 (13·8 to 27·1)	18·9 (13·7 to 26·8)	-3·88 (-13·9 to 7·83)	15·7 (11·0 to 21·7)	87·8 (71·2 to 102)	69·4 (55·1 to 91·9)	-20·8 (-34·5 to -4·71)	57·0 (46·3 to 66·6)
	15-49 years	76·6 (63·1 to 96·4)	75·4 (61·9 to 94·4)	-1·47 (-6·57 to 3·16)	61·3 (50·4 to 77·1)	2050 (1700 to 2430)	1850 (1500 to 2320)	-9·47 (-22·9 to 4·49)	1330 (1100 to 1580)
	50-69 years	94·5 (71·8 to 118)	91·5 (69·6 to 115)	-3·23 (-8·74 to 2·58)	75·6 (57·4 to 94·0)	1450 (1240 to 1800)	1440 (1150 to 1810)	-0·435 (-17·6 to 19·7)	942 (805 to 1170)
	70+ years	135 (109 to 164)	130 (105 to 160)	-3·79 (-9·68 to 2·90)	108 (87·5 to 131)	1440 (1260 to 1730)	1410 (1160 to 1810)	-2·17 (-17·8 to 17·2)	939 (819 to 1130)
Bolivia (Plurinational State of)	All Ages	82·4 (71·6 to 93·6)	71·1 (61·4 to 82·2)	-13·7 (-18·0 to -8·25)	65·9 (57·3 to 74·9)	1780 (1160 to 2620)	1550 (1070 to 2120)	-12·7 (-26·2 to 4·08)	1160 (752 to 1700)
	Under 5	28·5 (23·1 to 36·2)	22·7 (18·4 to 28·6)	-20·1 (-29·0 to -7·79)	22·8 (18·5 to 28·9)	75·6 (49·7 to 99·9)	46·9 (33·0 to 62·9)	-37·7 (-49·8 to -24·4)	49·1 (32·3 to 64·9)
	5-14 years	26·0 (18·7 to 36·4)	21·1 (15·0 to 28·9)	-18·5 (-27·9 to -3·87)	20·8 (15·0 to 29·1)	30·9 (19·8 to 43·7)	21·2 (13·8 to 29·8)	-31·0 (-42·8 to -17·0)	20·1 (12·9 to 28·4)
	15-49 years	88·8 (73·2 to 113)	74·7 (60·5 to 93·7)	-15·8 (-21·3 to -7·77)	71·0 (58·5 to 90·2)	570 (354 to 823)	483 (324 to 685)	-14·6 (-31·8 to 3·64)	371 (230 to 535)
	50-69 years	149 (117 to 184)	128 (95·6 to 162)	-13·9 (-21·0 to -5·73)	119 (93·5 to 147)	575 (384 to 854)	512 (352 to 679)	-10·4 (-27·6 to 8·31)	374 (250 to 555)
	70+ years	236 (187 to 298)	199 (156 to 252)	-15·4 (-24·4 to -6·17)	189 (149 to 238)	532 (366 to 752)	485 (342 to 684)	-8·23 (-21·6 to 6·91)	346 (238 to 489)
Ecuador	All Ages	47·5 (41·5 to 55·5)	32·8 (29·0 to 39·0)	-30·8 (-34·3 to -28·1)	38·0 (33·2 to 44·4)	1110 (947 to 1290)	652 (545 to 797)	-41·0 (-48·7 to -33·3)	719 (616 to 839)
	Under 5	12·1 (9·67 to 15·2)	6·82 (5·29 to 8·56)	-43·4 (-51·5 to -35·8)	9·65 (7·74 to 12·1)	22·4 (17·5 to 26·8)	11·0 (8·54 to 14·0)	-50·4 (-60·4 to -40·0)	14·5 (11·4 to 17·4)
	5-14 years	10·7 (7·28 to 14·5)	7·03 (4·84 to 9·79)	-34·4 (-42·3 to -24·6)	8·60 (5·83 to 11·6)	18·8 (16·6 to 21·2)	8·27 (6·71 to 9·82)	-56·0 (-62·1 to -51·2)	12·2 (10·8 to 13·8)
	15-49 years	59·1 (48·0 to 75·1)	40·5 (33·3 to 51·6)	-31·4 (-35·7 to -26·8)	47·2 (38·4 to 60·1)	531 (423 to 653)	303 (241 to 386)	-43·0 (-49·4 to -35·3)	345 (275 to 424)
	50-69 years	67·4 (52·4 to 83·8)	45·5 (35·6 to 56·8)	-32·5 (-37·8 to -25·6)	53·9 (41·9 to 67·1)	267 (232 to 310)	161 (133 to 197)	-39·9 (-50·6 to -30·0)	174 (151 to 201)
	70+ years	95·3 (77·9 to 116)	62·5 (49·8 to 76·4)	-34·3 (-39·4 to -27·4)	76·2 (62·3 to 92·4)	266 (233 to 290)	170 (142 to 197)	-36·2 (-46·3 to -25·0)	173 (151 to 188)
Peru	All Ages	68·4 (59·2 to 79·7)	78·4 (67·9 to 90·3)	14·7 (8·54 to 21·3)	54·8 (47·4 to 63·7)	2310 (1940 to 2660)	2680 (2010 to 3560)	16·6 (-9·71 to 46·4)	1500 (1260 to 1730)
	Under 5	17·9 (14·7 to 22·9)	18·5 (14·8 to 23·4)	3·54 (-9·99 to 18·6)	14·3 (11·7 to 18·3)	69·6 (53·2 to 90·0)	51·6 (40·5 to 66·0)	-25·4 (-42·0 to -3·53)	45·3 (34·6 to 58·5)

eTable 2. Progress towards the END-TB 2020 milestones in all-form tuberculosis incidence rate per 100,000 population and in deaths due to all-form tuberculosis by age for 204 countries and territories (2020), percent change from 2015 to 2020.

Location	Age group	2015 Rate	2020 Rate	Incidence Percent Change	Incidence Milestone	2015 Deaths	2020 Deaths	Mortality Percent Change	Mortality Milestone
	5-14 years	22·3 (15·7 to 30·7)	24·6 (17·4 to 35·4)	10·7 (-3·94 to 29·9)	17·8 (12·5 to 24·5)	38·0 (30·8 to 48·1)	39·9 (29·3 to 56·3)	5·22 (-21·4 to 32·0)	24·7 (20·0 to 31·3)
	15-49 years	81·1 (65·5 to 104)	92·6 (75·2 to 115)	14·4 (6·30 to 21·9)	64·8 (52·4 to 83·4)	945 (782 to 1130)	1060 (800 to 1340)	12·9 (-11·9 to 37·7)	614 (508 to 732)
	50-69 years	91·7 (67·6 to 116)	103 (77·3 to 130)	12·6 (1·70 to 23·0)	73·4 (54·1 to 92·5)	607 (494 to 708)	770 (549 to 1030)	27·3 (-9·03 to 70·8)	395 (321 to 460)
	70+ years	128 (103 to 159)	145 (118 to 179)	13·3 (3·58 to 23·8)	102 (82·6 to 127)	646 (543 to 786)	758 (578 to 1010)	17·6 (-9·74 to 55·0)	420 (353 to 511)
Caribbean	All Ages	45·0 (39·7 to 50·4)	40·8 (36·4 to 45·6)	-9·21 (-12·6 to -5·30)	36·0 (31·7 to 40·3)	4060 (2780 to 8610)	3490 (2340 to 8060)	-14·7 (-24·2 to 0·0361)	2640 (1810 to 5590)
	Under 5	31·3 (25·5 to 38·8)	28·5 (23·9 to 35·7)	-8·72 (-16·7 to 1·05)	25·0 (20·4 to 31·0)	322 (230 to 497)	259 (170 to 418)	-19·6 (-36·1 to -3·28)	209 (150 to 323)
	5-14 years	21·7 (15·3 to 30·7)	19·2 (13·5 to 27·2)	-11·1 (-21·8 to -2·02)	17·4 (12·2 to 24·6)	104 (67·7 to 210)	87·9 (58·0 to 177)	-14·6 (-26·7 to 0·870)	67·4 (44·0 to 137)
	15-49 years	53·5 (44·8 to 63·4)	48·5 (39·9 to 57·3)	-9·24 (-13·9 to -4·51)	42·8 (35·9 to 50·7)	2030 (1340 to 4280)	1590 (1040 to 3780)	-22·4 (-30·8 to -11·1)	1320 (873 to 2780)
	50-69 years	47·2 (37·8 to 58·5)	43·6 (35·3 to 53·5)	-7·67 (-12·4 to -2·62)	37·8 (30·3 to 46·8)	1030 (675 to 2690)	989 (644 to 2560)	-5·21 (-18·2 to 12·1)	672 (439 to 1750)
	70+ years	49·2 (39·6 to 61·5)	41·9 (33·3 to 51·5)	-14·9 (-19·8 to -9·76)	39·4 (31·7 to 49·2)	569 (401 to 1250)	564 (388 to 1240)	-0·968 (-15·2 to 18·4)	370 (260 to 812)
Antigua and Barbuda	All Ages	17·2 (14·8 to 20·1)	14·7 (12·5 to 17·1)	-14·5 (-19·5 to -9·36)	13·8 (11·9 to 16·1)	0·475 (0·424 to 0·554)	0·375 (0·318 to 0·433)	-21·1 (-26·5 to -16·7)	0·309 (0·275 to 0·360)
	Under 5	9·98 (7·62 to 13·1)	8·70 (6·76 to 11·1)	-12·6 (-26·3 to 0·119)	7·99 (6·09 to 10·4)	0·00434 (0·00347 to 0·00527)	0·00228 (0·00188 to 0·00281)	-47·4 (-55·1 to -39·0)	0·00282 (0·00225 to 0·00343)
	5-14 years	8·02 (5·19 to 11·7)	7·04 (4·56 to 10·2)	-11·9 (-24·1 to 0·101)	6·41 (4·15 to 9·38)	0·00332 (0·00269 to 0·00397)	0·00202 (0·00159 to 0·00250)	-39·2 (-47·0 to -28·2)	0·00216 (0·00175 to 0·00258)
	15-49 years	19·0 (15·2 to 24·0)	15·8 (12·9 to 19·6)	-16·5 (-22·0 to -10·5)	15·2 (12·1 to 19·2)	0·186 (0·154 to 0·230)	0·136 (0·108 to 0·174)	-27·0 (-31·6 to -22·2)	0·121 (0·100 to 0·149)
	50-69 years	20·9 (15·1 to 28·3)	17·5 (12·6 to 23·2)	-16·1 (-24·7 to -5·84)	16·7 (12·1 to 22·6)	0·157 (0·138 to 0·185)	0·130 (0·109 to 0·151)	-17·1 (-23·4 to -10·0)	0·102 (0·0895 to 0·120)
	70+ years	20·5 (15·6 to 25·7)	17·5 (13·1 to 22·6)	-14·8 (-25·1 to -2·87)	16·4 (12·5 to 20·5)	0·125 (0·115 to 0·136)	0·105 (0·0939 to 0·117)	-16·0 (-24·1 to -6·70)	0·0813 (0·0750 to 0·0886)
Bahamas	All Ages	15·0 (13·1 to 17·3)	14·0 (12·2 to 16·0)	-6·63 (-12·3 to -1·46)	12·0 (10·5 to 13·9)	9·70 (7·95 to 12·1)	9·66 (7·71 to 12·6)	-0·534 (-10·1 to 9·68)	6·31 (5·17 to 7·89)
	Under 5	6·40 (4·95 to 8·35)	5·59 (4·38 to 7·31)	-12·5 (-19·8 to -2·66)	5·12 (3·96 to 6·68)	0·0683 (0·0541 to 0·0899)	0·0505 (0·0390 to 0·0680)	-26·1 (-34·0 to -13·7)	0·0444 (0·0352 to 0·0585)
	5-14 years	6·11 (3·97 to 8·77)	5·54 (3·74 to 7·88)	-8·99 (-20·1 to 2·41)	4·89 (3·18 to 7·02)	0·0652 (0·0495 to 0·0886)	0·0592 (0·0477 to 0·0767)	-8·80 (-18·0 to 0·319)	0·0424 (0·0322 to 0·0576)
	15-49 years	16·3 (13·6 to 20·4)	14·8 (12·0 to 18·4)	-9·34 (-17·7 to -2·11)	13·0 (10·9 to 16·3)	5·48 (4·26 to 7·27)	5·23 (3·99 to 7·07)	-4·51 (-13·4 to 4·80)	3·56 (2·77 to 4·72)
	50-69 years	21·6 (16·0 to 27·5)	19·7 (14·2 to 25·1)	-8·53 (-17·4 to 0·597)	17·3 (12·8 to 22·0)	2·73 (2·25 to 3·29)	2·94 (2·37 to 3·81)	7·72 (-2·73 to 20·3)	1·77 (1·46 to 2·14)
	70+ years	21·8 (17·3 to 28·1)	19·1 (15·2 to 24·2)	-12·3 (-20·9 to -2·20)	17·5 (13·9 to 22·4)	1·37 (1·18 to 1·49)	1·37 (1·19 to 1·64)	0·616 (-10·9 to 12·2)	0·888 (0·768 to 0·969)

eTable 2. Progress towards the END-TB 2020 milestones in all-form tuberculosis incidence rate per 100,000 population and in deaths due to all-form tuberculosis by age for 204 countries and territories (2020), percent change from 2015 to 2020.

Location	Age group	2015 Rate	2020 Rate	Incidence Percent Change	Incidence Milestone	2015 Deaths	2020 Deaths	Mortality Percent Change	Mortality Milestone
Barbados	All Ages	6·17 (5·37 to 7·23)	5·52 (4·82 to 6·47)	-10·4 (-15·5 to -4·71)	4·93 (4·29 to 5·79)	1·92 (1·62 to 2·23)	1·92 (1·55 to 2·36)	-0·160 (-13·3 to 16·1)	1·25 (1·05 to 1·45)
	Under 5	3·35 (2·48 to 4·46)	2·96 (2·29 to 3·89)	-11·3 (-23·4 to -3·06)	2·68 (1·98 to 3·57)	0·00824 (0·00613 to 0·0106)	0·00671 (0·00489 to 0·00901)	-18·6 (-28·1 to -5·90)	0·00536 (0·00398 to 0·00689)
	5-14 years	2·39 (1·54 to 3·50)	2·12 (1·36 to 3·18)	-11·0 (-22·1 to 3·38)	1·91 (1·23 to 2·80)	0·0101 (0·00843 to 0·0121)	0·00562 (0·00364 to 0·00764)	-44·2 (-59·2 to -27·1)	0·00654 (0·00548 to 0·00789)
	15-49 years	6·31 (5·07 to 7·96)	5·43 (4·43 to 6·85)	-13·9 (-19·5 to -5·73)	5·05 (4·06 to 6·37)	0·637 (0·498 to 0·803)	0·579 (0·434 to 0·772)	-9·22 (-18·8 to 1·69)	0·414 (0·324 to 0·522)
	50-69 years	7·51 (5·52 to 10·1)	6·71 (4·97 to 8·87)	-10·6 (-16·9 to -4·04)	6·01 (4·42 to 8·04)	0·651 (0·554 to 0·754)	0·669 (0·522 to 0·832)	2·78 (-11·2 to 20·7)	0·423 (0·360 to 0·490)
	70+ years	8·77 (6·76 to 11·3)	7·80 (6·13 to 9·63)	-10·8 (-20·5 to -1·71)	7·02 (5·41 to 9·05)	0·619 (0·546 to 0·674)	0·662 (0·529 to 0·823)	7·00 (-10·0 to 29·8)	0·402 (0·355 to 0·438)
Belize	All Ages	28·1 (24·3 to 32·4)	24·9 (21·6 to 28·5)	-11·4 (-17·0 to -6·04)	22·5 (19·5 to 25·9)	19·0 (15·9 to 23·7)	17·2 (14·6 to 20·9)	-9·63 (-14·5 to -3·55)	12·3 (10·4 to 15·4)
	Under 5	9·41 (7·38 to 11·7)	7·44 (5·98 to 9·47)	-20·6 (-32·2 to -4·55)	7·53 (5·91 to 9·39)	0·363 (0·288 to 0·448)	0·272 (0·219 to 0·359)	-25·1 (-32·8 to -17·5)	0·236 (0·187 to 0·291)
	5-14 years	10·3 (6·95 to 14·6)	9·07 (6·23 to 12·7)	-11·3 (-25·3 to 4·68)	8·20 (5·56 to 11·7)	0·257 (0·212 to 0·308)	0·193 (0·152 to 0·245)	-24·8 (-32·3 to -17·9)	0·167 (0·138 to 0·200)
	15-49 years	30·7 (25·2 to 37·9)	26·1 (21·6 to 32·7)	-14·8 (-21·0 to -8·31)	24·5 (20·2 to 30·3)	11·2 (8·79 to 14·6)	9·51 (7·51 to 12·3)	-15·0 (-19·1 to -10·4)	7·27 (5·72 to 9·49)
	50-69 years	62·3 (47·7 to 77·9)	52·5 (39·3 to 66·8)	-15·7 (-23·0 to -6·80)	49·9 (38·2 to 62·3)	4·73 (4·15 to 5·63)	4·63 (4·03 to 5·45)	-2·13 (-10·6 to 6·31)	3·08 (2·69 to 3·66)
	70+ years	57·0 (43·6 to 74·1)	46·7 (35·4 to 58·8)	-17·8 (-27·6 to -8·37)	45·6 (34·9 to 59·3)	2·45 (2·26 to 2·61)	2·55 (2·27 to 2·87)	4·06 (-4·43 to 15·8)	1·59 (1·47 to 1·69)
Bermuda	All Ages	18·1 (15·4 to 22·2)	15·5 (12·9 to 18·6)	-14·6 (-18·8 to -8·52)	14·5 (12·3 to 17·7)	0·0635 (0·0544 to 0·0740)	0·0453 (0·0384 to 0·0540)	-28·6 (-34·4 to -22·0)	0·0412 (0·0353 to 0·0481)
	Under 5	8·16 (6·12 to 10·5)	7·23 (5·09 to 9·55)	-11·1 (-23·0 to 4·74)	6·53 (4·90 to 8·41)	0·000309 (0·000243 to 0·000389)	0·000160 (0·000121 to 0·000206)	-48·1 (-55·2 to -40·5)	0·000201 (0·000158 to 0·000253)
	5-14 years	6·42 (4·31 to 9·31)	5·64 (3·81 to 8·50)	-11·8 (-21·4 to 2·69)	5·14 (3·45 to 7·45)	0·000215 (0·000174 to 0·000277)	0·000136 (0·000103 to 0·000187)	-36·9 (-44·8 to -29·3)	0·000140 (0·000113 to 0·000180)
	15-49 years	20·4 (16·5 to 27·0)	17·2 (13·6 to 22·3)	-15·6 (-21·4 to -7·29)	16·3 (13·2 to 21·6)	0·0165 (0·0126 to 0·0221)	0·00980 (0·00696 to 0·0135)	-40·7 (-45·3 to -36·0)	0·0107 (0·00819 to 0·0143)
	50-69 years	22·0 (16·1 to 29·8)	18·8 (13·5 to 25·7)	-14·5 (-21·5 to -6·23)	17·6 (12·9 to 23·9)	0·0196 (0·0162 to 0·0241)	0·0140 (0·0110 to 0·0175)	-28·3 (-33·8 to -22·5)	0·0127 (0·0105 to 0·0156)
	70+ years	14·1 (11·0 to 18·2)	12·0 (9·36 to 15·5)	-15·1 (-25·6 to -0·270)	11·3 (8·77 to 14·5)	0·0268 (0·0227 to 0·0306)	0·0212 (0·0179 to 0·0243)	-21·1 (-28·0 to -12·7)	0·0174 (0·0148 to 0·0199)
Cuba	All Ages	6·42 (5·68 to 7·40)	5·61 (4·86 to 6·57)	-12·6 (-17·8 to -7·78)	5·14 (4·54 to 5·92)	47·6 (44·3 to 52·1)	37·4 (34·0 to 41·0)	-21·4 (-26·4 to -16·7)	30·9 (28·8 to 33·9)
	Under 5	2·51 (1·98 to 3·19)	2·22 (1·69 to 2·79)	-11·4 (-23·7 to 1·57)	2·01 (1·59 to 2·55)	0·169 (0·152 to 0·190)	0·108 (0·0920 to 0·125)	-35·9 (-44·5 to -25·7)	0·110 (0·0989 to 0·123)
	5-14 years	0·910 (0·603 to 1·26)	0·838 (0·539 to 1·18)	-7·61 (-19·8 to 9·93)	0·728 (0·482 to 1·01)	0·155 (0·140 to 0·175)	0·0860 (0·0762 to 0·0957)	-44·5 (-49·1 to -39·6)	0·101 (0·0911 to 0·114)

eTable 2. Progress towards the END-TB 2020 milestones in all-form tuberculosis incidence rate per 100,000 population and in deaths due to all-form tuberculosis by age for 204 countries and territories (2020), percent change from 2015 to 2020.

Location	Age group	2015 Rate	2020 Rate	Incidence Percent Change	Incidence Milestone	2015 Deaths	2020 Deaths	Mortality Percent Change	Mortality Milestone
	15-49 years	6·81 (5·68 to 8·54)	5·95 (4·96 to 7·34)	-12·5 (-19·6 to -4·10)	5·45 (4·54 to 6·83)	12·9 (10·9 to 15·5)	8·50 (7·04 to 10·3)	-33·9 (-37·9 to -30·5)	8·36 (7·08 to 10·1)
	50-69 years	8·39 (6·31 to 10·9)	7·19 (5·43 to 9·21)	-14·2 (-20·8 to -6·95)	6·71 (5·05 to 8·68)	17·7 (16·0 to 19·5)	15·9 (14·0 to 17·8)	-10·5 (-17·3 to -2·77)	11·5 (10·4 to 12·7)
	70+ years	8·11 (6·52 to 10·0)	6·53 (5·25 to 8·08)	-19·3 (-27·5 to -8·67)	6·49 (5·21 to 8·03)	16·6 (15·3 to 18·0)	12·8 (11·5 to 14·0)	-23·1 (-29·4 to -17·5)	10·8 (9·97 to 11·7)
Dominica	All Ages	21·3 (18·7 to 24·4)	20·2 (17·8 to 23·2)	-5·02 (-10·4 to 0·942)	17·0 (15·0 to 19·5)	3·06 (2·46 to 3·90)	2·94 (2·25 to 3·73)	-4·06 (-14·9 to 10·1)	1·99 (1·60 to 2·53)
	Under 5	8·18 (6·53 to 10·2)	7·51 (5·99 to 9·32)	-7·92 (-17·7 to 3·60)	6·54 (5·22 to 8·20)	0·0494 (0·0383 to 0·0651)	0·0373 (0·0258 to 0·0505)	-24·7 (-37·2 to -11·7)	0·0321 (0·0249 to 0·0423)
	5-14 years	6·85 (4·85 to 9·64)	6·35 (4·28 to 8·99)	-7·01 (-17·8 to 6·48)	5·48 (3·88 to 7·72)	0·0345 (0·0274 to 0·0447)	0·0275 (0·0198 to 0·0350)	-20·2 (-31·4 to -8·45)	0·0224 (0·0178 to 0·0291)
	15-49 years	22·2 (18·2 to 27·6)	20·3 (17·0 to 25·3)	-8·60 (-14·3 to -2·34)	17·8 (14·6 to 22·1)	1·12 (0·838 to 1·53)	0·974 (0·711 to 1·35)	-13·1 (-23·0 to -0·973)	0·729 (0·545 to 0·997)
	50-69 years	31·6 (24·4 to 39·3)	28·7 (21·5 to 35·4)	-9·15 (-17·2 to -0·414)	25·3 (19·6 to 31·5)	1·08 (0·809 to 1·41)	1·12 (0·823 to 1·48)	3·36 (-12·1 to 24·9)	0·704 (0·526 to 0·917)
	70+ years	36·9 (29·5 to 46·3)	34·8 (28·0 to 43·4)	-5·79 (-14·6 to 1·38)	29·5 (23·6 to 37·0)	0·771 (0·631 to 0·926)	0·779 (0·563 to 0·982)	0·906 (-13·6 to 15·5)	0·501 (0·410 to 0·602)
Dominican Republic	All Ages	60·9 (53·4 to 70·1)	49·2 (43·1 to 56·4)	-19·1 (-23·6 to -13·6)	48·7 (42·7 to 56·0)	1160 (857 to 1530)	900 (635 to 1390)	-22·7 (-33·9 to -10·0)	755 (557 to 994)
	Under 5	18·4 (14·9 to 22·8)	13·3 (10·7 to 16·8)	-27·5 (-36·9 to -15·7)	14·7 (11·9 to 18·3)	17·5 (12·1 to 24·2)	13·0 (8·17 to 18·4)	-25·4 (-49·1 to 0·0602)	11·4 (7·89 to 15·7)
	5-14 years	21·9 (14·7 to 30·4)	15·9 (10·9 to 21·7)	-26·8 (-37·5 to -10·8)	17·5 (11·7 to 24·3)	13·8 (10·4 to 17·4)	12·9 (8·74 to 18·3)	-6·15 (-23·4 to 11·3)	8·95 (6·78 to 11·3)
	15-49 years	70·6 (57·6 to 88·8)	56·4 (46·4 to 70·3)	-20·2 (-25·7 to -13·3)	56·5 (46·1 to 71·0)	605 (419 to 820)	372 (250 to 551)	-38·6 (-46·0 to -29·4)	394 (272 to 533)
	50-69 years	84·0 (64·3 to 106)	68·5 (51·8 to 85·6)	-18·3 (-24·8 to -11·2)	67·2 (51·4 to 84·8)	294 (210 to 414)	263 (176 to 420)	-10·9 (-29·6 to 12·5)	191 (136 to 269)
	70+ years	123 (99·3 to 155)	100 (81·7 to 125)	-18·7 (-26·8 to -8·89)	98·6 (79·5 to 124)	231 (176 to 314)	239 (167 to 377)	3·06 (-16·3 to 31·4)	150 (114 to 204)
Grenada	All Ages	10·0 (8·45 to 11·8)	8·82 (7·43 to 10·5)	-11·9 (-16·9 to -6·79)	8·02 (6·76 to 9·45)	0·691 (0·613 to 0·785)	0·442 (0·390 to 0·497)	-36·0 (-41·1 to -30·6)	0·449 (0·398 to 0·510)
	Under 5	6·64 (4·89 to 8·43)	5·81 (4·38 to 7·58)	-12·3 (-26·4 to 1·89)	5·31 (3·91 to 6·75)	0·00741 (0·00575 to 0·00974)	0·00341 (0·00263 to 0·00448)	-53·9 (-59·0 to -47·0)	0·00482 (0·00374 to 0·00633)
	5-14 years	4·76 (3·07 to 6·88)	4·24 (2·76 to 6·14)	-10·5 (-26·5 to 1·44)	3·80 (2·46 to 5·50)	0·00649 (0·00566 to 0·00746)	0·00376 (0·00314 to 0·00440)	-42·1 (-47·7 to -35·3)	0·00422 (0·00368 to 0·00485)
	15-49 years	11·0 (8·90 to 13·8)	9·59 (7·65 to 11·9)	-12·5 (-18·0 to -7·33)	8·76 (7·12 to 11·0)	0·274 (0·227 to 0·328)	0·154 (0·128 to 0·183)	-43·6 (-48·1 to -39·4)	0·178 (0·148 to 0·213)
	50-69 years	12·5 (9·34 to 16·4)	10·8 (7·82 to 14·2)	-13·8 (-20·5 to -5·69)	10·0 (7·47 to 13·1)	0·251 (0·219 to 0·287)	0·163 (0·140 to 0·187)	-35·0 (-42·3 to -28·6)	0·163 (0·143 to 0·186)
	70+ years	12·8 (9·73 to 16·4)	10·2 (7·58 to 13·2)	-19·6 (-29·3 to -8·58)	10·2 (7·78 to 13·1)	0·153 (0·142 to 0·168)	0·118 (0·107 to 0·132)	-22·6 (-29·6 to -16·3)	0·0993 (0·0924 to 0·109)
Guyana	All Ages	63·4 (55·2 to 72·4)	52·7 (46·1 to 60·8)	-16·9 (-22·2 to -12·1)	50·7 (44·2 to 57·9)	107 (85·1 to 131)	79·7 (63·5 to 98·4)	-25·7 (-34·9 to -16·3)	69·8 (55·3 to 84·9)

eTable 2. Progress towards the END-TB 2020 milestones in all-form tuberculosis incidence rate per 100,000 population and in deaths due to all-form tuberculosis by age for 204 countries and territories (2020), percent change from 2015 to 2020.

Location	Age group	2015 Rate	2020 Rate	Incidence Percent Change	Incidence Milestone	2015 Deaths	2020 Deaths	Mortality Percent Change	Mortality Milestone
	Under 5	15·9 (13·2 to 20·3)	12·4 (10·2 to 14·8)	-22·1 (-31·3 to -13·7)	12·7 (10·6 to 16·2)	1·65 (1·25 to 2·11)	1·05 (0·778 to 1·37)	-36·2 (-45·8 to -28·1)	1·07 (0·815 to 1·37)
	5-14 years	15·6 (11·2 to 21·5)	13·4 (9·63 to 18·6)	-14·2 (-25·7 to -0·144)	12·5 (8·95 to 17·2)	1·20 (0·993 to 1·55)	0·757 (0·607 to 0·956)	-36·8 (-43·6 to -29·5)	0·779 (0·645 to 1·01)
	15-49 years	78·0 (65·2 to 94·2)	63·1 (52·7 to 76·4)	-19·0 (-24·8 to -13·2)	62·4 (52·1 to 75·4)	60·6 (47·0 to 77·3)	43·1 (33·1 to 55·4)	-28·8 (-36·7 to -20·6)	39·4 (30·5 to 50·2)
	50-69 years	101 (82·4 to 122)	85·1 (66·2 to 107)	-15·8 (-23·6 to -8·28)	80·9 (65·9 to 97·3)	33·0 (25·9 to 39·5)	25·8 (20·2 to 32·1)	-21·6 (-34·2 to -8·03)	21·4 (16·8 to 25·7)
	70+ years	82·7 (65·6 to 103)	66·2 (51·2 to 82·5)	-19·8 (-28·8 to -10·5)	66·2 (52·5 to 82·3)	11·0 (9·40 to 12·8)	9·01 (7·18 to 11·0)	-17·9 (-32·3 to -3·93)	7·14 (6·11 to 8·31)
Haiti	All Ages	101 (88·3 to 115)	90·3 (79·4 to 103)	-10·2 (-15·7 to -5·45)	80·4 (70·7 to 92·1)	2450 (1460 to 7000)	2220 (1290 to 6650)	-10·2 (-23·0 to 3·07)	1590 (948 to 4550)
	Under 5	64·8 (51·4 to 81·7)	57·3 (48·1 to 72·8)	-11·2 (-20·4 to 2·26)	51·8 (41·1 to 65·4)	290 (201 to 456)	235 (153 to 383)	-19·1 (-36·7 to -1·63)	189 (131 to 297)
	5-14 years	43·5 (30·7 to 62·2)	38·1 (26·1 to 54·9)	-11·9 (-25·0 to 0·384)	34·8 (24·5 to 49·7)	83·9 (50·3 to 187)	70·3 (44·1 to 156)	-15·5 (-29·5 to 1·31)	54·6 (32·7 to 122)
	15-49 years	118 (97·9 to 139)	103 (84·2 to 124)	-12·3 (-18·6 to -5·53)	94·3 (78·3 to 111)	1210 (690 to 3440)	1050 (610 to 3220)	-13·5 (-26·2 to 0·437)	788 (448 to 2240)
	50-69 years	156 (127 to 191)	147 (118 to 177)	-5·08 (-12·7 to 4·21)	124 (102 to 152)	606 (324 to 2270)	607 (333 to 2160)	-0·751 (-15·3 to 15·5)	394 (211 to 1480)
	70+ years	216 (172 to 276)	195 (150 to 246)	-9·47 (-16·4 to -0·143)	173 (138 to 220)	258 (135 to 933)	255 (131 to 905)	-1·60 (-20·8 to 21·4)	168 (87·9 to 607)
Jamaica	All Ages	5·04 (4·34 to 5·87)	4·53 (3·92 to 5·30)	-9·96 (-14·5 to -5·02)	4·03 (3·47 to 4·70)	27·2 (21·6 to 34·6)	24·4 (18·3 to 31·4)	-10·2 (-22·3 to 3·78)	17·7 (14·1 to 22·5)
	Under 5	2·67 (2·05 to 3·54)	2·34 (1·73 to 3·02)	-12·0 (-20·7 to -1·01)	2·13 (1·64 to 2·83)	0·213 (0·160 to 0·276)	0·155 (0·118 to 0·210)	-27·2 (-33·7 to -19·8)	0·138 (0·104 to 0·180)
	5-14 years	1·78 (1·17 to 2·45)	1·58 (1·07 to 2·30)	-11·1 (-20·5 to -0·444)	1·42 (0·932 to 1·96)	0·198 (0·156 to 0·258)	0·156 (0·112 to 0·201)	-21·3 (-37·0 to -11·9)	0·129 (0·101 to 0·168)
	15-49 years	5·16 (4·30 to 6·41)	4·51 (3·71 to 5·64)	-12·6 (-17·7 to -6·30)	4·13 (3·44 to 5·13)	12·5 (8·84 to 17·6)	11·0 (7·83 to 15·7)	-12·1 (-20·4 to -1·70)	8·13 (5·75 to 11·4)
	50-69 years	7·46 (5·68 to 9·60)	6·62 (4·89 to 8·58)	-11·2 (-18·6 to -3·08)	5·97 (4·54 to 7·68)	8·06 (6·46 to 10·1)	7·59 (5·52 to 9·95)	-5·69 (-22·0 to 12·0)	5·24 (4·20 to 6·59)
	70+ years	9·35 (7·62 to 11·6)	8·10 (6·46 to 9·86)	-13·2 (-22·4 to -1·85)	7·48 (6·09 to 9·27)	6·22 (5·52 to 6·91)	5·53 (4·42 to 6·67)	-11·0 (-28·8 to 7·76)	4·04 (3·59 to 4·49)
Puerto Rico	All Ages	3·59 (3·18 to 4·04)	3·33 (2·87 to 3·80)	-7·25 (-12·9 to -2·06)	2·87 (2·55 to 3·23)	34·8 (30·0 to 41·5)	21·7 (18·4 to 26·1)	-37·6 (-41·5 to -34·2)	22·6 (19·5 to 26·9)
	Under 5	1·70 (1·28 to 2·18)	1·49 (1·12 to 1·98)	-11·9 (-20·0 to 1·83)	1·36 (1·02 to 1·74)	0·134 (0·104 to 0·174)	0·0425 (0·0337 to 0·0547)	-68·2 (-70·9 to -65·3)	0·0870 (0·0673 to 0·113)
	5-14 years	1·21 (0·787 to 1·81)	1·09 (0·681 to 1·66)	-10·4 (-22·6 to 5·01)	0·972 (0·630 to 1·45)	0·101 (0·0783 to 0·133)	0·0419 (0·0306 to 0·0590)	-58·5 (-62·7 to -54·8)	0·0654 (0·0509 to 0·0867)
	15-49 years	3·02 (2·48 to 3·79)	2·71 (2·20 to 3·44)	-10·0 (-17·4 to -3·40)	2·42 (1·99 to 3·03)	10·5 (8·42 to 13·6)	6·24 (4·96 to 8·17)	-40·8 (-44·2 to -37·5)	6·85 (5·48 to 8·87)
	50-69 years	4·72 (3·58 to 5·94)	4·21 (3·18 to 5·33)	-10·7 (-18·3 to -4·14)	3·77 (2·86 to 4·75)	12·4 (10·3 to 15·4)	7·46 (6·13 to 9·36)	-39·8 (-43·6 to -35·6)	8·05 (6·72 to 10·0)

eTable 2. Progress towards the END-TB 2020 milestones in all-form tuberculosis incidence rate per 100,000 population and in deaths due to all-form tuberculosis by age for 204 countries and territories (2020), percent change from 2015 to 2020.

Location	Age group	2015 Rate	2020 Rate	Incidence Percent Change	Incidence Milestone	2015 Deaths	2020 Deaths	Mortality Percent Change	Mortality Milestone
	70+ years	6·68 (5·35 to 8·28)	5·60 (4·48 to 7·02)	-16·0 (-24·4 to -5·94)	5·34 (4·28 to 6·62)	11·6 (10·4 to 12·6)	7·92 (7·07 to 8·78)	-31·8 (-37·1 to -27·8)	7·55 (6·76 to 8·22)
Saint Kitts and Nevis	All Ages	12·0 (10·5 to 14·0)	11·3 (9·86 to 13·0)	-5·54 (-10·3 to -1·06)	9·58 (8·36 to 11·2)	1·24 (1·03 to 1·50)	1·23 (0·986 to 1·48)	-0·561 (-8·35 to 7·31)	0·805 (0·666 to 0·977)
	Under 5	4·97 (3·94 to 6·17)	4·58 (3·71 to 5·79)	-7·74 (-17·3 to 6·99)	3·98 (3·15 to 4·94)	0·0139 (0·0101 to 0·0186)	0·0129 (0·00908 to 0·0178)	-7·28 (-15·8 to 1·19)	0·00901 (0·00657 to 0·0121)
	5-14 years	3·51 (2·45 to 4·87)	2·82 (1·96 to 3·94)	-19·3 (-30·2 to -8·75)	2·80 (1·96 to 3·90)	0·00805 (0·00683 to 0·00957)	0·00623 (0·00505 to 0·00756)	-22·5 (-33·8 to -9·50)	0·00523 (0·00444 to 0·00622)
	15-49 years	12·6 (10·4 to 15·7)	11·3 (9·46 to 14·0)	-10·5 (-16·4 to -3·32)	10·1 (8·36 to 12·6)	0·488 (0·362 to 0·662)	0·454 (0·336 to 0·617)	-6·95 (-13·4 to 0·856)	0·317 (0·235 to 0·430)
	50-69 years	16·4 (12·5 to 20·5)	15·9 (11·9 to 20·0)	-3·33 (-13·2 to 8·54)	13·1 (9·97 to 16·4)	0·493 (0·405 to 0·571)	0·532 (0·433 to 0·611)	7·94 (-2·20 to 19·0)	0·320 (0·263 to 0·371)
	70+ years	20·5 (16·2 to 25·1)	17·8 (14·1 to 22·2)	-13·3 (-21·8 to -1·18)	16·4 (13·0 to 20·0)	0·235 (0·214 to 0·253)	0·226 (0·198 to 0·253)	-3·88 (-14·5 to 6·57)	0·153 (0·139 to 0·165)
Saint Lucia	All Ages	12·9 (11·2 to 15·0)	12·3 (10·6 to 14·4)	-4·47 (-10·1 to 2·05)	10·3 (8·97 to 12·0)	4·28 (3·81 to 4·83)	4·57 (3·88 to 5·35)	6·91 (-3·10 to 20·6)	2·78 (2·48 to 3·14)
	Under 5	4·96 (3·92 to 6·24)	4·31 (3·43 to 5·72)	-12·9 (-23·6 to -3·37)	3·97 (3·14 to 4·99)	0·0265 (0·0207 to 0·0329)	0·0183 (0·0138 to 0·0230)	-31·0 (-40·4 to -17·7)	0·0172 (0·0135 to 0·0214)
	5-14 years	4·42 (2·79 to 6·48)	3·95 (2·64 to 5·66)	-10·4 (-22·7 to 0·282)	3·53 (2·23 to 5·18)	0·0278 (0·0236 to 0·0338)	0·0242 (0·0202 to 0·0288)	-12·7 (-24·4 to -1·09)	0·0181 (0·0153 to 0·0220)
	15-49 years	12·6 (10·3 to 15·8)	11·6 (9·27 to 15·0)	-7·87 (-16·6 to 0·472)	10·1 (8·24 to 12·6)	1·77 (1·50 to 2·13)	1·69 (1·40 to 2·02)	-4·09 (-11·9 to 5·96)	1·15 (0·977 to 1·38)
	50-69 years	18·7 (13·7 to 23·6)	17·1 (12·7 to 22·6)	-8·30 (-20·0 to 0·321)	14·9 (11·0 to 18·9)	1·41 (1·25 to 1·58)	1·52 (1·26 to 1·78)	7·26 (-3·90 to 22·9)	0·918 (0·813 to 1·03)
	70+ years	23·4 (18·9 to 29·1)	21·4 (16·9 to 26·5)	-8·54 (-17·5 to 0·320)	18·7 (15·2 to 23·3)	1·04 (0·953 to 1·14)	1·32 (1·15 to 1·52)	26·6 (11·9 to 41·4)	0·678 (0·620 to 0·742)
Saint Vincent and the Grenadines	All Ages	17·3 (15·1 to 20·0)	15·3 (13·2 to 17·6)	-11·7 (-16·7 to -6·33)	13·8 (12·0 to 16·0)	3·41 (2·87 to 4·10)	2·75 (2·33 to 3·35)	-19·5 (-25·1 to -11·3)	2·22 (1·86 to 2·66)
	Under 5	7·31 (5·90 to 9·45)	6·30 (4·94 to 8·43)	-13·6 (-26·1 to -2·47)	5·85 (4·72 to 7·56)	0·0281 (0·0216 to 0·0369)	0·0155 (0·0115 to 0·0204)	-44·7 (-51·7 to -36·5)	0·0183 (0·0141 to 0·0240)
	5-14 years	7·11 (4·61 to 10·2)	6·06 (4·00 to 8·88)	-14·4 (-27·6 to 0·394)	5·69 (3·69 to 8·15)	0·0314 (0·0255 to 0·0384)	0·0227 (0·0182 to 0·0282)	-27·7 (-33·8 to -21·3)	0·0204 (0·0166 to 0·0250)
	15-49 years	18·4 (15·1 to 22·6)	16·0 (13·1 to 20·4)	-13·2 (-19·3 to -4·71)	14·7 (12·1 to 18·1)	1·60 (1·25 to 2·07)	1·16 (0·879 to 1·52)	-27·9 (-33·0 to -22·2)	1·04 (0·810 to 1·34)
	50-69 years	24·3 (18·0 to 31·7)	20·7 (15·6 to 27·0)	-14·5 (-23·8 to -7·72)	19·4 (14·4 to 25·3)	1·03 (0·871 to 1·24)	0·925 (0·779 to 1·12)	-10·2 (-18·3 to 1·53)	0·671 (0·566 to 0·806)
	70+ years	27·5 (21·4 to 35·4)	23·3 (18·0 to 28·9)	-14·9 (-23·7 to -4·55)	22·0 (17·1 to 28·3)	0·716 (0·651 to 0·787)	0·624 (0·563 to 0·686)	-12·7 (-20·5 to -3·11)	0·465 (0·423 to 0·512)
Suriname	All Ages	21·4 (18·6 to 24·6)	19·8 (17·5 to 22·7)	-7·44 (-12·5 to -2·19)	17·1 (14·9 to 19·7)	18·0 (13·7 to 23·0)	17·6 (13·5 to 22·6)	-2·21 (-13·4 to 12·6)	11·7 (8·93 to 14·9)
	Under 5	8·28 (6·68 to 10·2)	7·15 (5·79 to 9·13)	-13·5 (-25·1 to -1·70)	6·63 (5·35 to 8·16)	0·341 (0·248 to 0·469)	0·290 (0·203 to 0·380)	-14·7 (-28·5 to -0·888)	0·222 (0·161 to 0·305)
	5-14 years	6·70 (4·58 to 9·49)	6·09 (4·11 to 8·62)	-8·68 (-19·4 to 4·35)	5·36 (3·66 to 7·59)	0·208 (0·163 to 0·268)	0·195 (0·151 to 0·259)	-5·83 (-20·9 to 8·69)	0·135 (0·106 to 0·174)

eTable 2. Progress towards the END-TB 2020 milestones in all-form tuberculosis incidence rate per 100,000 population and in deaths due to all-form tuberculosis by age for 204 countries and territories (2020), percent change from 2015 to 2020.

Location	Age group	2015 Rate	2020 Rate	Incidence Percent Change	Incidence Milestone	2015 Deaths	2020 Deaths	Mortality Percent Change	Mortality Milestone
	15-49 years	26·1 (21·6 to 31·9)	23·9 (19·9 to 29·3)	-8·43 (-15·4 to -1·89)	20·9 (17·3 to 25·5)	9·41 (6·75 to 12·8)	8·61 (6·21 to 11·9)	-8·32 (-20·2 to 5·29)	6·12 (4·39 to 8·33)
	50-69 years	26·3 (20·4 to 33·3)	24·4 (18·1 to 30·4)	-7·38 (-15·4 to 0·258)	21·1 (16·3 to 26·6)	5·35 (4·19 to 6·74)	5·79 (4·36 to 7·56)	8·24 (-4·49 to 25·2)	3·48 (2·72 to 4·38)
	70+ years	30·1 (24·1 to 38·0)	26·8 (21·4 to 33·2)	-10·8 (-18·8 to -1·39)	24·0 (19·3 to 30·4)	2·66 (2·14 to 3·20)	2·66 (2·09 to 3·22)	0·258 (-14·5 to 19·2)	1·73 (1·39 to 2·08)
Trinidad and Tobago	All Ages	14·5 (12·6 to 16·8)	12·9 (11·1 to 14·9)	-11·2 (-16·2 to -5·31)	11·6 (10·1 to 13·5)	27·0 (22·1 to 33·9)	26·7 (21·2 to 32·9)	-0·898 (-12·7 to 12·6)	17·5 (14·4 to 22·0)
	Under 5	4·86 (3·82 to 6·24)	4·15 (3·14 to 5·36)	-14·6 (-23·7 to -5·23)	3·89 (3·06 to 4·99)	0·220 (0·166 to 0·289)	0·165 (0·124 to 0·220)	-25·0 (-32·7 to -14·9)	0·143 (0·108 to 0·188)
	5-14 years	3·17 (2·06 to 4·47)	2·86 (1·87 to 4·02)	-9·58 (-24·0 to 5·89)	2·54 (1·65 to 3·58)	0·151 (0·117 to 0·200)	0·144 (0·114 to 0·179)	-4·07 (-11·3 to 5·55)	0·0979 (0·0761 to 0·130)
	15-49 years	15·8 (12·8 to 19·3)	13·9 (11·2 to 17·6)	-11·4 (-18·2 to -2·00)	12·6 (10·2 to 15·5)	12·5 (9·46 to 17·1)	12·0 (8·87 to 16·0)	-4·18 (-12·7 to 6·44)	8·15 (6·15 to 11·1)
	50-69 years	20·9 (15·9 to 26·0)	18·2 (13·2 to 22·7)	-13·1 (-21·6 to -3·51)	16·8 (12·7 to 20·8)	9·99 (8·45 to 12·2)	9·79 (7·85 to 12·0)	-2·03 (-16·5 to 14·3)	6·50 (5·49 to 7·96)
	70+ years	17·3 (13·2 to 21·4)	15·1 (11·9 to 19·8)	-12·3 (-22·5 to -2·27)	13·8 (10·6 to 17·1)	4·10 (3·75 to 4·59)	4·64 (3·89 to 5·61)	13·4 (-3·00 to 33·3)	2·66 (2·43 to 2·99)
United States Virgin Islands	All Ages	6·17 (5·41 to 7·13)	5·84 (5·06 to 6·71)	-5·24 (-9·37 to -1·29)	4·94 (4·33 to 5·71)	0·959 (0·724 to 1·22)	0·875 (0·613 to 1·19)	-8·88 (-24·7 to 4·62)	0·624 (0·470 to 0·791)
	Under 5	2·18 (1·68 to 2·82)	1·78 (1·39 to 2·27)	-18·2 (-27·9 to -9·60)	1·74 (1·34 to 2·26)	0·00362 (0·00254 to 0·00520)	0·00207 (0·00149 to 0·00290)	-42·1 (-58·9 to -23·4)	0·00235 (0·00165 to 0·00338)
	5-14 years	1·23 (0·807 to 1·74)	1·01 (0·690 to 1·47)	-17·2 (-27·6 to -6·29)	0·982 (0·645 to 1·40)	0·00287 (0·00218 to 0·00383)	0·00169 (0·00126 to 0·00232)	-41·0 (-50·2 to -29·4)	0·00187 (0·00142 to 0·00249)
	15-49 years	5·88 (4·79 to 7·24)	5·23 (4·33 to 6·64)	-11·1 (-16·9 to -5·64)	4·70 (3·83 to 5·80)	0·393 (0·287 to 0·529)	0·320 (0·225 to 0·457)	-18·7 (-31·5 to -7·38)	0·256 (0·187 to 0·344)
	50-69 years	8·36 (6·26 to 10·6)	7·76 (5·89 to 9·90)	-7·16 (-13·3 to -1·27)	6·69 (5·01 to 8·50)	0·305 (0·219 to 0·409)	0·268 (0·178 to 0·383)	-12·1 (-30·4 to 8·73)	0·198 (0·142 to 0·266)
	70+ years	9·56 (7·34 to 12·3)	8·71 (6·75 to 11·3)	-8·77 (-15·7 to 0·876)	7·65 (5·87 to 9·81)	0·255 (0·196 to 0·314)	0·283 (0·199 to 0·386)	11·1 (-5·81 to 30·3)	0·165 (0·128 to 0·204)
Central Latin America	All Ages	22·2 (19·9 to 24·9)	22·2 (19·7 to 25·0)	-0·0911 (-2·21 to 2·23)	17·8 (15·9 to 19·9)	7530 (6830 to 8320)	7730 (6860 to 8590)	2·70 (-2·63 to 8·86)	4890 (4440 to 5410)
	Under 5	5·32 (4·42 to 6·56)	4·52 (3·80 to 5·57)	-15·0 (-18·3 to -11·2)	4·26 (3·54 to 5·25)	128 (106 to 147)	99·1 (78·9 to 117)	-22·4 (-28·7 to -12·1)	83·0 (69·2 to 95·8)
	5-14 years	4·45 (3·14 to 6·11)	4·25 (2·89 to 5·99)	-4·54 (-10·4 to 1·00)	3·56 (2·51 to 4·89)	86·5 (76·2 to 98·7)	73·6 (63·3 to 84·1)	-14·9 (-20·1 to -8·88)	56·2 (49·6 to 64·1)
	15-49 years	23·4 (19·8 to 28·0)	23·1 (19·3 to 28·0)	-1·42 (-4·23 to 1·70)	18·7 (15·8 to 22·4)	3140 (2650 to 3780)	3070 (2540 to 3690)	-2·26 (-6·73 to 2·51)	2040 (1720 to 2450)
	50-69 years	41·0 (32·1 to 50·4)	38·8 (29·7 to 47·8)	-5·39 (-9·11 to -1·15)	32·8 (25·7 to 40·3)	2360 (2190 to 2530)	2590 (2330 to 2870)	9·60 (2·79 to 17·3)	1530 (1430 to 1640)
	70+ years	55·2 (45·3 to 67·8)	47·9 (39·6 to 59·4)	-13·3 (-15·9 to -10·5)	44·2 (36·2 to 54·2)	1820 (1680 to 1920)	1910 (1760 to 2100)	4·92 (-0·721 to 11·1)	1180 (1090 to 1250)
Colombia	All Ages	24·8 (21·8 to 28·2)	23·1 (20·2 to 26·5)	-6·91 (-11·8 to -0·684)	19·8 (17·5 to 22·5)	1180 (1050 to 1330)	1310 (1140 to 1520)	11·4 (4·50 to 18·4)	764 (680 to 866)

eTable 2. Progress towards the END-TB 2020 milestones in all-form tuberculosis incidence rate per 100,000 population and in deaths due to all-form tuberculosis by age for 204 countries and territories (2020), percent change from 2015 to 2020.

Location	Age group	2015 Rate	2020 Rate	Incidence Percent Change	Incidence Milestone	2015 Deaths	2020 Deaths	Mortality Percent Change	Mortality Milestone
	Under 5	5·78 (4·73 to 6·97)	4·64 (3·77 to 5·84)	-19·5 (-29·2 to -8·62)	4·62 (3·78 to 5·57)	15·0 (11·7 to 19·6)	12·7 (9·33 to 16·2)	-15·0 (-30·9 to -0·0770)	9·77 (7·60 to 12·7)
	5-14 years	3·54 (2·38 to 4·63)	2·96 (2·11 to 3·99)	-16·2 (-25·8 to -2·64)	2·83 (1·90 to 3·70)	9·21 (7·96 to 11·2)	8·37 (7·13 to 10·0)	-8·92 (-19·9 to -0·277)	5·98 (5·18 to 7·28)
	15-49 years	24·8 (21·2 to 30·8)	22·3 (18·7 to 27·2)	-9·94 (-15·8 to -2·87)	19·9 (16·9 to 24·6)	419 (340 to 542)	420 (328 to 556)	0·279 (-5·27 to 5·68)	272 (221 to 352)
	50-69 years	41·0 (32·0 to 50·9)	37·0 (27·7 to 46·7)	-9·71 (-16·4 to -3·79)	32·8 (25·6 to 40·7)	373 (334 to 413)	447 (394 to 509)	19·8 (11·3 to 29·9)	243 (217 to 269)
	70+ years	65·0 (52·3 to 79·3)	56·3 (45·2 to 68·9)	-13·2 (-21·2 to -4·19)	52·0 (41·8 to 63·4)	359 (327 to 387)	420 (376 to 456)	17·1 (7·24 to 25·7)	233 (213 to 252)
Costa Rica	All Ages	9·58 (8·39 to 11·0)	8·94 (7·93 to 10·3)	-6·59 (-11·5 to -0·291)	7·66 (6·71 to 8·79)	58·3 (53·7 to 63·6)	64·8 (58·7 to 71·5)	11·1 (5·70 to 17·6)	37·9 (34·9 to 41·3)
	Under 5	2·46 (1·91 to 3·17)	2·01 (1·53 to 2·61)	-18·0 (-28·6 to -4·68)	1·96 (1·53 to 2·54)	0·415 (0·351 to 0·496)	0·312 (0·253 to 0·392)	-24·9 (-32·9 to -16·9)	0·270 (0·228 to 0·323)
	5-14 years	1·58 (1·09 to 2·13)	1·31 (0·889 to 1·86)	-17·0 (-29·4 to -3·05)	1·26 (0·869 to 1·70)	0·358 (0·300 to 0·425)	0·315 (0·267 to 0·373)	-12·0 (-18·2 to -5·33)	0·232 (0·195 to 0·276)
	15-49 years	9·41 (7·80 to 11·6)	8·42 (7·14 to 10·4)	-10·4 (-16·7 to -3·01)	7·52 (6·24 to 9·30)	19·0 (16·0 to 22·8)	20·0 (16·8 to 23·8)	5·03 (0·697 to 10·6)	12·4 (10·4 to 14·8)
	50-69 years	16·1 (12·4 to 20·0)	14·9 (11·3 to 18·8)	-7·63 (-14·0 to 0·190)	12·9 (9·93 to 16·0)	20·1 (18·4 to 22·0)	23·8 (21·3 to 26·4)	18·1 (10·6 to 27·2)	13·1 (11·9 to 14·3)
	70+ years	24·2 (19·5 to 29·6)	21·1 (17·0 to 25·0)	-12·7 (-20·0 to -2·94)	19·3 (15·6 to 23·7)	18·4 (16·5 to 19·9)	20·4 (18·1 to 22·7)	11·0 (4·00 to 19·2)	12·0 (10·7 to 12·9)
El Salvador	All Ages	44·4 (39·3 to 50·5)	40·2 (35·7 to 45·9)	-9·30 (-14·3 to -4·86)	35·5 (31·5 to 40·4)	304 (261 to 354)	259 (214 to 311)	-14·8 (-24·0 to -1·86)	197 (170 to 230)
	Under 5	10·6 (8·49 to 13·4)	8·46 (6·78 to 10·4)	-20·3 (-31·6 to -9·27)	8·52 (6·79 to 10·7)	4·65 (3·81 to 5·62)	2·85 (2·26 to 3·60)	-38·6 (-49·1 to -25·2)	3·02 (2·48 to 3·66)
	5-14 years	10·3 (7·21 to 14·5)	8·41 (5·50 to 12·0)	-18·6 (-29·5 to -6·39)	8·28 (5·77 to 11·6)	3·99 (3·28 to 4·83)	2·72 (2·24 to 3·48)	-31·7 (-41·8 to -20·3)	2·59 (2·13 to 3·14)
	15-49 years	50·0 (41·3 to 61·5)	44·7 (37·0 to 54·8)	-10·5 (-17·0 to -5·38)	40·0 (33·0 to 49·2)	128 (105 to 157)	95·9 (77·0 to 124)	-25·1 (-33·0 to -12·3)	83·2 (68·0 to 102)
	50-69 years	75·7 (58·6 to 92·8)	67·4 (50·5 to 84·6)	-11·0 (-17·3 to -2·05)	60·6 (46·9 to 74·2)	79·6 (66·9 to 93·7)	77·4 (61·4 to 99·9)	-2·84 (-16·0 to 18·3)	51·7 (43·5 to 60·9)
	70+ years	101 (83·2 to 124)	88·4 (71·8 to 109)	-12·6 (-20·8 to -5·30)	81·0 (66·5 to 98·9)	87·6 (72·5 to 101)	80·1 (64·9 to 101)	-8·62 (-20·5 to 5·20)	56·9 (47·1 to 65·9)
Guatemala	All Ages	21·1 (18·7 to 23·4)	19·5 (17·4 to 21·9)	-7·55 (-13·3 to -2·59)	16·8 (15·0 to 18·7)	605 (533 to 687)	585 (519 to 659)	-3·17 (-8·56 to 2·30)	393 (347 to 446)
	Under 5	8·22 (6·67 to 10·2)	6·61 (5·44 to 8·10)	-19·4 (-27·3 to -9·95)	6·57 (5·33 to 8·13)	24·6 (20·7 to 28·4)	14·7 (11·8 to 17·5)	-39·9 (-47·6 to -31·3)	16·0 (13·4 to 18·4)
	5-14 years	5·80 (4·11 to 7·66)	4·92 (3·57 to 6·75)	-15·0 (-23·9 to -2·25)	4·64 (3·29 to 6·13)	12·3 (10·5 to 14·5)	9·72 (8·17 to 11·3)	-20·8 (-27·5 to -14·4)	7·98 (6·80 to 9·42)
	15-49 years	23·9 (20·4 to 28·4)	21·3 (18·1 to 25·6)	-11·2 (-17·4 to -5·40)	19·2 (16·3 to 22·7)	294 (248 to 354)	274 (229 to 322)	-6·82 (-12·3 to -2·10)	191 (161 to 230)
	50-69 years	46·4 (38·6 to 56·5)	41·8 (33·6 to 52·0)	-10·0 (-17·7 to -1·72)	37·1 (30·9 to 45·2)	172 (153 to 195)	173 (152 to 194)	0·343 (-8·36 to 8·49)	112 (99·4 to 127)

eTable 2. Progress towards the END-TB 2020 milestones in all-form tuberculosis incidence rate per 100,000 population and in deaths due to all-form tuberculosis by age for 204 countries and territories (2020), percent change from 2015 to 2020.

Location	Age group	2015 Rate	2020 Rate	Incidence Percent Change	Incidence Milestone	2015 Deaths	2020 Deaths	Mortality Percent Change	Mortality Milestone
	70+ years	52·6 (42·0 to 64·3)	45·6 (37·1 to 56·1)	-13·3 (-20·0 to -3·73)	42·0 (33·6 to 51·4)	101 (94·7 to 108)	114 (101 to 126)	12·6 (3·20 to 21·6)	65·8 (61·6 to 70·1)
Honduras	All Ages	38·6 (34·8 to 42·5)	35·1 (31·2 to 38·9)	-9·24 (-13·7 to -4·54)	30·9 (27·8 to 34·0)	780 (549 to 1020)	753 (575 to 990)	-3·14 (-18·1 to 14·8)	507 (357 to 662)
	Under 5	11·2 (8·94 to 13·7)	9·15 (7·48 to 10·9)	-17·8 (-26·9 to -5·53)	8·93 (7·15 to 10·9)	17·4 (13·2 to 22·9)	13·6 (10·2 to 16·8)	-21·3 (-36·3 to -1·44)	11·3 (8·61 to 14·9)
	5-14 years	9·95 (6·79 to 13·5)	8·02 (5·73 to 11·2)	-19·0 (-29·4 to -4·43)	7·96 (5·43 to 10·8)	11·6 (7·75 to 15·6)	8·94 (5·82 to 12·2)	-22·2 (-36·9 to -4·28)	7·51 (5·04 to 10·1)
	15-49 years	39·8 (32·9 to 47·2)	34·5 (28·7 to 41·3)	-13·2 (-19·7 to -6·40)	31·8 (26·4 to 37·7)	234 (160 to 318)	235 (154 to 319)	1·33 (-20·4 to 35·3)	152 (104 to 207)
	50-69 years	100 (82·2 to 123)	92·1 (74·9 to 111)	-8·27 (-14·7 to 0·0772)	80·4 (65·7 to 98·6)	287 (214 to 365)	281 (206 to 379)	-1·95 (-18·9 to 19·2)	186 (139 to 237)
	70+ years	146 (117 to 183)	128 (102 to 165)	-12·0 (-18·9 to -3·94)	117 (93·8 to 146)	230 (155 to 311)	215 (149 to 280)	-6·09 (-20·8 to 7·44)	150 (101 to 202)
Mexico	All Ages	17·4 (15·4 to 19·9)	19·0 (16·6 to 22·1)	9·07 (5·11 to 13·5)	13·9 (12·3 to 15·9)	3170 (2910 to 3540)	3190 (2880 to 3570)	0·743 (-6·53 to 6·37)	2060 (1890 to 2300)
	Under 5	3·21 (2·63 to 4·09)	2·76 (2·26 to 3·49)	-14·0 (-19·4 to -7·99)	2·57 (2·10 to 3·27)	39·4 (32·5 to 45·9)	32·9 (26·7 to 39·9)	-16·3 (-26·1 to -2·39)	25·6 (21·1 to 29·9)
	5-14 years	3·37 (2·25 to 4·80)	3·71 (2·33 to 5·43)	9·90 (1·40 to 19·4)	2·69 (1·80 to 3·84)	33·3 (29·4 to 38·1)	29·6 (26·3 to 33·5)	-10·9 (-17·1 to -4·08)	21·6 (19·1 to 24·8)
	15-49 years	17·8 (15·0 to 21·8)	19·8 (16·3 to 24·6)	11·3 (5·61 to 16·9)	14·2 (12·0 to 17·4)	1440 (1250 to 1730)	1430 (1220 to 1700)	-0·935 (-6·90 to 3·97)	938 (810 to 1120)
	50-69 years	35·2 (26·9 to 44·3)	34·7 (26·1 to 45·1)	-1·68 (-7·18 to 3·29)	28·2 (21·5 to 35·4)	986 (927 to 1070)	1050 (946 to 1160)	6·91 (-3·52 to 14·3)	641 (603 to 693)
	70+ years	43·1 (34·3 to 54·3)	37·0 (29·2 to 46·7)	-14·3 (-17·1 to -10·8)	34·5 (27·5 to 43·4)	671 (640 to 696)	650 (584 to 700)	-3·09 (-10·8 to 3·34)	436 (416 to 452)
Nicaragua	All Ages	32·8 (29·0 to 37·2)	29·9 (26·4 to 34·1)	-8·76 (-12·8 to -3·10)	26·3 (23·2 to 29·7)	260 (237 to 297)	240 (201 to 284)	-7·67 (-17·6 to 5·43)	169 (154 to 193)
	Under 5	9·51 (7·41 to 11·8)	7·69 (6·21 to 9·67)	-18·9 (-30·6 to -7·80)	7·61 (5·93 to 9·43)	7·72 (5·80 to 9·52)	5·12 (3·78 to 6·76)	-33·6 (-43·9 to -16·8)	5·02 (3·77 to 6·19)
	5-14 years	7·59 (5·24 to 10·3)	6·26 (4·20 to 8·74)	-17·3 (-27·8 to -7·16)	6·07 (4·19 to 8·28)	3·68 (3·09 to 4·71)	2·19 (1·79 to 2·67)	-40·5 (-48·7 to -31·2)	2·39 (2·01 to 3·06)
	15-49 years	36·2 (29·6 to 44·1)	32·4 (27·1 to 39·8)	-10·4 (-16·0 to -2·95)	29·0 (23·7 to 35·3)	96·8 (83·9 to 110)	84·7 (71·1 to 101)	-12·5 (-24·1 to 0·703)	62·9 (54·6 to 71·6)
	50-69 years	68·5 (53·0 to 84·0)	60·2 (46·5 to 74·9)	-12·2 (-21·6 to -4·22)	54·8 (42·4 to 67·2)	80·1 (69·1 to 93·3)	78·6 (63·3 to 99·1)	-1·70 (-15·9 to 15·3)	52·0 (44·9 to 60·7)
	70+ years	95·0 (76·8 to 117)	80·6 (65·3 to 97·9)	-15·0 (-22·1 to -7·96)	76·0 (61·4 to 93·4)	71·4 (61·5 to 85·9)	69·1 (56·6 to 84·1)	-3·12 (-16·3 to 13·8)	46·4 (40·0 to 55·9)
Panama	All Ages	41·5 (37·1 to 47·4)	36·9 (32·4 to 42·3)	-11·1 (-15·6 to -6·38)	33·2 (29·7 to 37·9)	237 (207 to 271)	216 (192 to 254)	-8·69 (-16·0 to -2·75)	154 (135 to 176)
	Under 5	13·6 (11·1 to 17·1)	10·4 (8·34 to 13·0)	-22·9 (-35·4 to -10·7)	10·9 (8·85 to 13·7)	6·71 (5·34 to 7·77)	4·39 (3·40 to 5·36)	-34·5 (-42·1 to -24·7)	4·36 (3·47 to 5·05)
	5-14 years	9·96 (7·15 to 13·6)	8·52 (5·92 to 11·7)	-14·4 (-27·8 to -1·70)	7·97 (5·72 to 10·9)	2·94 (2·46 to 3·46)	2·28 (1·94 to 2·77)	-22·6 (-28·4 to -15·3)	1·91 (1·60 to 2·25)

eTable 2. Progress towards the END-TB 2020 milestones in all-form tuberculosis incidence rate per 100,000 population and in deaths due to all-form tuberculosis by age for 204 countries and territories (2020), percent change from 2015 to 2020.

Location	Age group	2015 Rate	2020 Rate	Incidence Percent Change	Incidence Milestone	2015 Deaths	2020 Deaths	Mortality Percent Change	Mortality Milestone
	15-49 years	48·7 (39·8 to 59·0)	42·8 (35·5 to 53·4)	-12·0 (-17·5 to -5·02)	39·0 (31·8 to 47·2)	98·5 (79·2 to 123)	86·0 (70·4 to 112)	-12·7 (-18·7 to -7·40)	64·0 (51·5 to 79·9)
	50-69 years	62·4 (47·7 to 76·3)	55·5 (41·8 to 67·8)	-11·1 (-17·8 to -3·18)	49·9 (38·2 to 61·1)	69·3 (62·0 to 77·0)	67·4 (59·8 to 76·5)	-2·61 (-12·8 to 7·26)	45·0 (40·3 to 50·1)
	70+ years	73·6 (59·1 to 91·1)	62·9 (51·8 to 78·6)	-14·5 (-21·1 to -6·51)	58·9 (47·3 to 72·8)	59·3 (53·3 to 65·2)	56·0 (48·8 to 61·1)	-5·52 (-14·9 to 2·64)	38·5 (34·6 to 42·4)
Venezuela (Bolivarian Republic of)	All Ages	26·3 (23·3 to 30·1)	26·7 (23·6 to 30·2)	1·40 (-4·43 to 6·63)	21·1 (18·6 to 24·0)	939 (846 to 1090)	1110 (878 to 1420)	18·4 (-1·27 to 46·7)	610 (550 to 706)
	Under 5	6·50 (5·27 to 8·24)	5·95 (4·80 to 7·33)	-8·36 (-18·4 to 3·34)	5·20 (4·22 to 6·59)	11·8 (10·3 to 13·9)	12·4 (9·78 to 16·6)	5·17 (-16·7 to 30·7)	7·68 (6·69 to 9·07)
	5-14 years	4·88 (3·45 to 6·61)	4·73 (3·27 to 6·64)	-2·90 (-14·3 to 11·1)	3·90 (2·76 to 5·29)	9·21 (8·01 to 10·8)	9·43 (7·87 to 11·9)	2·28 (-12·1 to 18·1)	5·99 (5·21 to 7·03)
	15-49 years	30·2 (25·3 to 36·8)	29·7 (24·5 to 36·4)	-1·63 (-8·81 to 4·48)	24·1 (20·2 to 29·4)	404 (336 to 515)	422 (329 to 566)	4·12 (-11·0 to 23·4)	263 (219 to 335)
	50-69 years	40·9 (31·6 to 49·4)	40·2 (30·0 to 50·6)	-1·63 (-9·72 to 6·02)	32·7 (25·3 to 39·6)	293 (268 to 329)	386 (298 to 494)	31·7 (6·24 to 69·8)	191 (174 to 214)
	70+ years	54·9 (44·7 to 66·1)	50·4 (39·7 to 62·7)	-8·11 (-16·9 to 0·0166)	43·9 (35·8 to 52·9)	220 (200 to 238)	283 (228 to 368)	28·4 (3·91 to 64·9)	143 (130 to 155)
Tropical Latin America	All Ages	41·5 (36·4 to 48·3)	38·8 (33·8 to 45·5)	-6·50 (-10·4 to -1·31)	33·2 (29·1 to 38·6)	9010 (7850 to 10400)	8560 (7470 to 9830)	-4·96 (-7·62 to -2·03)	5850 (5100 to 6770)
	Under 5	6·98 (5·81 to 8·49)	6·45 (5·44 to 7·97)	-7·56 (-12·7 to -1·32)	5·58 (4·65 to 6·79)	168 (139 to 204)	122 (101 to 147)	-27·3 (-36·3 to -18·7)	109 (90·7 to 132)
	5-14 years	5·43 (3·71 to 7·65)	6·10 (4·01 to 8·60)	12·1 (1·00 to 23·8)	4·34 (2·97 to 6·12)	68·9 (56·7 to 81·9)	54·1 (43·8 to 65·3)	-21·4 (-25·9 to -16·0)	44·8 (36·9 to 53·2)
	15-49 years	49·2 (41·6 to 60·3)	46·5 (38·0 to 57·4)	-5·39 (-11·7 to 0·486)	39·3 (33·3 to 48·2)	4200 (3390 to 5220)	3750 (3100 to 4570)	-10·6 (-13·0 to -7·65)	2730 (2210 to 3390)
	50-69 years	59·8 (46·0 to 74·8)	52·9 (39·3 to 69·8)	-11·7 (-17·7 to -3·76)	47·8 (36·8 to 59·8)	3120 (2820 to 3460)	3120 (2770 to 3530)	0·232 (-2·97 to 3·69)	2020 (1830 to 2250)
	70+ years	58·9 (46·4 to 73·2)	47·3 (37·2 to 60·1)	-19·7 (-23·9 to -15·3)	47·2 (37·1 to 58·6)	1460 (1310 to 1530)	1510 (1370 to 1600)	3·49 (0·797 to 6·63)	946 (851 to 997)
Brazil	All Ages	41·7 (36·5 to 48·5)	39·0 (33·9 to 45·8)	-6·47 (-10·4 to -1·22)	33·4 (29·2 to 38·8)	8640 (7520 to 10000)	8220 (7190 to 9490)	-4·82 (-7·52 to -2·14)	5620 (4890 to 6530)
	Under 5	6·87 (5·72 to 8·38)	6·38 (5·36 to 7·88)	-7·19 (-12·7 to -0·780)	5·50 (4·57 to 6·71)	154 (128 to 188)	113 (93·5 to 136)	-27·0 (-36·8 to -17·4)	100 (83·1 to 122)
	5-14 years	5·33 (3·64 to 7·52)	6·06 (3·96 to 8·54)	13·5 (1·51 to 25·4)	4·27 (2·91 to 6·01)	64·5 (52·6 to 77·4)	48·6 (38·8 to 60·0)	-24·7 (-29·7 to -18·6)	41·9 (34·2 to 50·3)
	15-49 years	49·4 (41·8 to 60·6)	46·8 (38·2 to 57·7)	-5·27 (-11·8 to 0·777)	39·5 (33·4 to 48·4)	4040 (3260 to 5040)	3610 (2990 to 4420)	-10·4 (-13·3 to -7·59)	2630 (2120 to 3280)
	50-69 years	59·7 (45·9 to 74·7)	52·8 (39·2 to 69·9)	-11·7 (-18·0 to -3·68)	47·8 (36·7 to 59·8)	3000 (2710 to 3350)	3010 (2660 to 3420)	0·359 (-3·14 to 3·71)	1950 (1760 to 2180)
	70+ years	58·7 (46·1 to 72·8)	47·0 (36·9 to 59·7)	-19·9 (-24·1 to -15·3)	46·9 (36·9 to 58·2)	1390 (1250 to 1470)	1440 (1310 to 1530)	3·83 (1·06 to 6·89)	901 (815 to 954)
Paraguay	All Ages	35·9 (31·2 to 41·4)	33·3 (29·0 to 38·8)	-7·28 (-11·6 to -2·38)	28·7 (24·9 to 33·1)	363 (311 to 425)	333 (264 to 409)	-8·11 (-21·3 to 5·33)	236 (202 to 276)

eTable 2. Progress towards the END-TB 2020 milestones in all-form tuberculosis incidence rate per 100,000 population and in deaths due to all-form tuberculosis by age for 204 countries and territories (2020), percent change from 2015 to 2020.

Location	Age group	2015 Rate	2020 Rate	Incidence Percent Change	Incidence Milestone	2015 Deaths	2020 Deaths	Mortality Percent Change	Mortality Milestone
	Under 5	9·52 (7·73 to 11·5)	8·23 (6·63 to 10·2)	-13·4 (-22·8 to 3·08)	7·62 (6·18 to 9·16)	13·7 (11·1 to 17·1)	9·42 (7·26 to 11·9)	-30·9 (-42·8 to -11·7)	8·90 (7·22 to 11·1)
	5-14 years	7·66 (5·44 to 10·6)	6·93 (4·75 to 9·89)	-9·11 (-20·8 to 4·64)	6·12 (4·35 to 8·50)	4·36 (3·66 to 5·21)	5·54 (4·37 to 7·01)	27·2 (4·53 to 53·5)	2·84 (2·38 to 3·38)
	15-49 years	42·8 (35·5 to 52·2)	38·9 (32·5 to 49·1)	-8·88 (-14·7 to -2·55)	34·2 (28·4 to 41·8)	159 (130 to 185)	138 (107 to 163)	-13·1 (-23·7 to -2·01)	103 (84·8 to 120)
	50-69 years	62·8 (47·6 to 77·7)	56·1 (41·8 to 68·9)	-10·6 (-18·4 to -1·63)	50·2 (38·1 to 62·2)	116 (97·9 to 144)	112 (87·0 to 148)	-2·76 (-20·1 to 16·3)	75·3 (63·7 to 93·8)
	70+ years	71·6 (57·9 to 87·7)	62·3 (49·8 to 77·3)	-12·9 (-20·8 to -5·67)	57·3 (46·3 to 70·2)	69·8 (58·6 to 81·6)	67·5 (51·1 to 81·9)	-3·03 (-20·2 to 17·4)	45·4 (38·1 to 53·1)
North Africa and Middle East	All Ages	31·5 (27·7 to 35·8)	28·1 (24·9 to 32·5)	-10·7 (-13·2 to -8·66)	25·2 (22·2 to 28·6)	22600 (18700 to 31700)	20200 (16300 to 28300)	-10·5 (-17·6 to -3·06)	14700 (12200 to 20600)
	Under 5	15·1 (12·5 to 18·5)	12·6 (9·82 to 15·4)	-16·8 (-21·8 to -11·8)	12·1 (9·97 to 14·8)	1660 (1260 to 2080)	1140 (849 to 1470)	-30·9 (-38·8 to -19·3)	1080 (821 to 1350)
	5-14 years	15·6 (11·4 to 21·9)	12·7 (9·02 to 17·6)	-18·5 (-24·5 to -13·0)	12·4 (9·11 to 17·5)	612 (481 to 829)	454 (369 to 623)	-25·6 (-33·9 to -15·9)	398 (313 to 539)
	15-49 years	31·3 (25·8 to 37·7)	27·7 (22·7 to 33·6)	-11·5 (-14·0 to -9·18)	25·1 (20·7 to 30·2)	7960 (6400 to 10200)	6930 (5530 to 9100)	-12·9 (-20·8 to -4·60)	5180 (4160 to 6630)
	50-69 years	54·5 (42·0 to 68·1)	48·5 (36·8 to 59·5)	-10·9 (-13·4 to -7·58)	43·6 (33·6 to 54·5)	5900 (4870 to 8700)	5790 (4590 to 8450)	-1·93 (-10·3 to 9·05)	3840 (3170 to 5650)
	70+ years	109 (87·6 to 134)	92·8 (75·2 to 115)	-14·6 (-18·1 to -12·1)	86·9 (70·1 to 108)	6410 (5020 to 10800)	5870 (4500 to 8980)	-8·28 (-17·0 to 3·08)	4170 (3260 to 7010)
Afghanistan	All Ages	130 (112 to 150)	115 (97·1 to 130)	-11·8 (-17·5 to -5·27)	104 (89·7 to 120)	7500 (5620 to 11600)	6790 (5120 to 10300)	-9·05 (-19·2 to 3·07)	4870 (3650 to 7520)
	Under 5	62·4 (50·1 to 77·6)	51·4 (39·1 to 64·2)	-17·6 (-28·4 to -5·13)	50·0 (40·1 to 62·1)	874 (621 to 1210)	704 (507 to 974)	-18·8 (-35·7 to 1·46)	568 (404 to 784)
	5-14 years	66·8 (47·8 to 96·0)	58·1 (40·8 to 83·7)	-12·6 (-25·7 to -1·90)	53·4 (38·2 to 76·8)	337 (243 to 543)	264 (195 to 404)	-21·2 (-36·3 to -3·90)	219 (158 to 353)
	15-49 years	157 (124 to 194)	138 (109 to 166)	-11·8 (-18·1 to -4·26)	126 (98·8 to 155)	3280 (2410 to 4720)	3030 (2200 to 4440)	-7·58 (-24·6 to 9·08)	2130 (1570 to 3070)
	50-69 years	389 (302 to 490)	304 (227 to 382)	-21·7 (-29·6 to -12·3)	311 (242 to 392)	1570 (1100 to 2810)	1630 (1140 to 2680)	4·68 (-10·3 to 25·7)	1020 (716 to 1830)
	70+ years	540 (418 to 675)	494 (390 to 620)	-8·37 (-17·4 to 1·30)	432 (334 to 540)	1430 (889 to 2940)	1170 (765 to 2230)	-17·4 (-29·7 to -1·70)	930 (578 to 1910)
Algeria	All Ages	47·2 (40·8 to 54·6)	41·8 (36·9 to 48·6)	-11·3 (-15·2 to -7·12)	37·8 (32·6 to 43·7)	1740 (1320 to 2260)	1620 (1260 to 2090)	-6·60 (-21·5 to 13·9)	1130 (856 to 1470)
	Under 5	20·9 (15·9 to 26·0)	16·3 (12·3 to 20·8)	-22·1 (-30·2 to -14·4)	16·7 (12·7 to 20·8)	89·7 (64·2 to 121)	48·3 (33·1 to 68·6)	-46·0 (-58·5 to -33·0)	58·3 (41·7 to 79·0)
	5-14 years	20·5 (14·4 to 29·9)	16·7 (11·4 to 23·6)	-18·3 (-28·7 to -3·92)	16·4 (11·5 to 23·9)	24·7 (18·4 to 33·3)	20·1 (14·8 to 27·6)	-18·6 (-30·2 to 0·0612)	16·1 (11·9 to 21·6)
	15-49 years	42·9 (34·4 to 53·6)	37·8 (30·6 to 46·9)	-11·8 (-19·1 to -5·26)	34·3 (27·5 to 42·9)	450 (344 to 667)	388 (307 to 524)	-13·4 (-28·1 to 4·19)	292 (224 to 433)
	50-69 years	87·3 (64·6 to 113)	77·2 (57·3 to 96·8)	-11·4 (-18·0 to -5·21)	69·8 (51·7 to 90·3)	435 (322 to 596)	437 (317 to 587)	0·761 (-18·1 to 32·2)	283 (209 to 388)

eTable 2. Progress towards the END-TB 2020 milestones in all-form tuberculosis incidence rate per 100,000 population and in deaths due to all-form tuberculosis by age for 204 countries and territories (2020), percent change from 2015 to 2020.

Location	Age group	2015 Rate	2020 Rate	Incidence Percent Change	Incidence Milestone	2015 Deaths	2020 Deaths	Mortality Percent Change	Mortality Milestone
	70+ years	182 (144 to 221)	160 (127 to 203)	-11·9 (-19·6 to -2·70)	146 (116 to 177)	742 (543 to 1000)	729 (538 to 991)	-1·35 (-19·2 to 21·8)	482 (353 to 650)
Bahrain	All Ages	14·1 (12·0 to 17·4)	14·3 (12·4 to 17·2)	2·01 (-4·47 to 8·53)	11·2 (9·57 to 13·9)	11·8 (10·1 to 14·2)	11·4 (9·26 to 15·3)	-3·69 (-15·7 to 10·6)	7·67 (6·56 to 9·22)
	Under 5	5·09 (4·11 to 6·50)	4·76 (3·66 to 5·88)	-6·42 (-19·2 to 4·31)	4·07 (3·28 to 5·20)	0·250 (0·209 to 0·299)	0·157 (0·123 to 0·202)	-37·3 (-46·9 to -24·5)	0·163 (0·136 to 0·195)
	5-14 years	5·68 (3·80 to 8·05)	5·41 (3·73 to 7·65)	-4·46 (-19·5 to 10·4)	4·54 (3·04 to 6·44)	0·132 (0·106 to 0·173)	0·0985 (0·0754 to 0·136)	-25·5 (-35·7 to -8·99)	0·0859 (0·0688 to 0·112)
	15-49 years	14·2 (11·5 to 18·0)	14·2 (11·5 to 17·7)	0·160 (-7·79 to 7·99)	11·4 (9·23 to 14·4)	5·46 (4·55 to 6·87)	4·59 (3·61 to 6·57)	-16·2 (-27·3 to -2·46)	3·55 (2·96 to 4·46)
	50-69 years	23·8 (17·6 to 30·3)	23·1 (17·2 to 28·0)	-2·84 (-11·5 to 6·05)	19·0 (14·1 to 24·2)	3·32 (2·76 to 4·23)	3·63 (2·95 to 4·75)	9·26 (-6·13 to 28·8)	2·16 (1·79 to 2·75)
	70+ years	60·4 (47·7 to 75·0)	55·2 (43·0 to 69·8)	-8·55 (-17·8 to 4·26)	48·3 (38·1 to 60·0)	2·63 (2·21 to 3·17)	2·91 (2·26 to 3·84)	10·7 (-7·45 to 31·2)	1·71 (1·43 to 2·06)
Egypt	All Ages	11·2 (10·1 to 12·4)	9·18 (8·08 to 10·3)	-18·3 (-23·1 to -14·4)	9·00 (8·09 to 9·90)	1150 (1030 to 1280)	883 (749 to 1030)	-23·1 (-34·3 to -11·0)	748 (669 to 832)
	Under 5	5·76 (4·58 to 7·08)	4·12 (3·23 to 5·24)	-28·2 (-38·1 to -16·9)	4·61 (3·67 to 5·66)	62·9 (46·8 to 79·5)	31·7 (22·3 to 40·0)	-49·0 (-63·1 to -31·4)	40·9 (30·4 to 51·7)
	5-14 years	4·90 (3·38 to 6·99)	3·43 (2·46 to 4·83)	-29·7 (-38·0 to -17·7)	3·92 (2·71 to 5·59)	20·9 (16·5 to 26·3)	15·1 (12·1 to 19·2)	-26·8 (-42·9 to -8·24)	13·6 (10·7 to 17·1)
	15-49 years	12·0 (9·96 to 14·0)	9·87 (8·14 to 11·9)	-18·0 (-24·0 to -12·3)	9·63 (7·97 to 11·2)	377 (320 to 420)	294 (242 to 358)	-21·7 (-33·3 to -8·03)	245 (208 to 273)
	50-69 years	21·9 (18·0 to 26·2)	18·7 (15·6 to 22·4)	-14·5 (-22·1 to -6·77)	17·5 (14·4 to 20·9)	465 (415 to 533)	350 (290 to 418)	-24·5 (-36·6 to -9·11)	302 (269 to 346)
	70+ years	36·2 (29·3 to 43·3)	29·1 (23·1 to 36·5)	-19·5 (-27·4 to -9·59)	28·9 (23·4 to 34·7)	226 (196 to 275)	192 (159 to 235)	-14·5 (-28·9 to -1·72)	147 (127 to 179)
Iran (Islamic Republic of)	All Ages	16·3 (14·5 to 18·3)	15·7 (14·2 to 17·6)	-3·39 (-6·06 to -0·0540)	13·0 (11·6 to 14·6)	1220 (1080 to 1310)	986 (866 to 1170)	-19·3 (-25·4 to -7·70)	793 (702 to 852)
	Under 5	6·55 (5·37 to 8·45)	4·78 (3·83 to 6·13)	-27·1 (-31·1 to -23·2)	5·24 (4·29 to 6·76)	43·0 (37·7 to 47·2)	15·6 (13·5 to 17·7)	-63·8 (-67·6 to -60·6)	28·0 (24·5 to 30·7)
	5-14 years	8·06 (5·70 to 11·3)	6·55 (4·62 to 9·01)	-18·7 (-22·7 to -14·6)	6·45 (4·56 to 9·01)	21·3 (19·4 to 22·9)	15·1 (13·5 to 16·8)	-28·8 (-33·8 to -21·6)	13·8 (12·6 to 14·9)
	15-49 years	15·5 (12·9 to 18·5)	15·0 (12·6 to 17·8)	-3·31 (-7·30 to 1·49)	12·4 (10·3 to 14·8)	431 (384 to 468)	338 (290 to 397)	-21·5 (-27·2 to -10·6)	280 (249 to 304)
	50-69 years	24·9 (19·5 to 30·8)	24·1 (19·1 to 29·2)	-3·22 (-6·23 to -0·480)	19·9 (15·6 to 24·7)	303 (273 to 330)	263 (230 to 314)	-13·5 (-20·0 to -0·0955)	197 (177 to 214)
	70+ years	52·2 (42·7 to 64·4)	49·8 (40·9 to 60·6)	-4·56 (-8·41 to -0·710)	41·7 (34·2 to 51·5)	422 (363 to 463)	354 (298 to 428)	-16·1 (-23·9 to -2·99)	274 (236 to 301)
Iraq	All Ages	27·5 (23·6 to 32·3)	23·4 (20·3 to 27·4)	-14·9 (-20·2 to -10·2)	22·0 (18·9 to 25·8)	969 (764 to 1210)	894 (664 to 1180)	-7·60 (-21·8 to 13·7)	630 (497 to 785)
	Under 5	11·2 (8·57 to 14·0)	8·27 (6·44 to 10·3)	-26·0 (-34·8 to -16·3)	8·96 (6·86 to 11·2)	33·1 (24·7 to 43·7)	18·5 (12·2 to 26·0)	-43·9 (-57·5 to -25·0)	21·5 (16·1 to 28·4)
	5-14 years	13·4 (9·29 to 19·4)	9·31 (6·52 to 13·1)	-30·2 (-40·7 to -21·2)	10·7 (7·44 to 15·5)	29·5 (24·8 to 37·1)	12·4 (9·88 to 17·0)	-58·0 (-66·3 to -49·8)	19·2 (16·1 to 24·1)

eTable 2. Progress towards the END-TB 2020 milestones in all-form tuberculosis incidence rate per 100,000 population and in deaths due to all-form tuberculosis by age for 204 countries and territories (2020), percent change from 2015 to 2020.

Location	Age group	2015 Rate	2020 Rate	Incidence Percent Change	Incidence Milestone	2015 Deaths	2020 Deaths	Mortality Percent Change	Mortality Milestone
	15-49 years	28·0 (22·6 to 35·0)	22·8 (18·8 to 28·3)	-18·6 (-24·7 to -12·1)	22·4 (18·1 to 28·0)	340 (258 to 451)	256 (184 to 355)	-24·6 (-38·1 to -5·67)	221 (168 to 293)
	50-69 years	63·0 (47·3 to 77·9)	54·4 (41·8 to 69·4)	-13·7 (-19·5 to -6·76)	50·4 (37·8 to 62·3)	309 (237 to 400)	314 (217 to 427)	1·76 (-17·9 to 27·5)	201 (154 to 260)
	70+ years	107 (85·9 to 134)	94·9 (76·1 to 117)	-11·1 (-19·1 to -2·25)	85·6 (68·7 to 107)	258 (206 to 325)	294 (221 to 406)	14·0 (-7·52 to 46·1)	168 (134 to 211)
Jordan	All Ages	5·51 (4·75 to 6·41)	5·46 (4·66 to 6·36)	-0·872 (-6·68 to 5·94)	4·41 (3·80 to 5·13)	37·0 (31·4 to 44·6)	47·0 (36·0 to 57·9)	27·0 (3·09 to 50·4)	24·1 (20·4 to 29·0)
	Under 5	2·05 (1·43 to 2·75)	1·77 (1·21 to 2·48)	-13·4 (-25·4 to 0·0581)	1·64 (1·14 to 2·20)	1·15 (0·905 to 1·50)	0·895 (0·680 to 1·19)	-22·2 (-35·1 to -6·93)	0·748 (0·588 to 0·978)
	5-14 years	2·60 (1·72 to 3·81)	2·49 (1·64 to 3·63)	-4·15 (-16·8 to 11·3)	2·08 (1·38 to 3·05)	0·747 (0·598 to 0·918)	0·777 (0·591 to 0·983)	4·52 (-15·4 to 23·4)	0·485 (0·389 to 0·597)
	15-49 years	5·55 (4·48 to 6·90)	5·21 (4·20 to 6·49)	-6·05 (-12·9 to 2·92)	4·44 (3·58 to 5·52)	11·0 (9·42 to 13·0)	13·2 (10·4 to 16·7)	20·3 (-0·998 to 43·5)	7·16 (6·12 to 8·48)
	50-69 years	12·2 (9·11 to 15·4)	11·2 (8·19 to 14·2)	-8·03 (-14·1 to -1·50)	9·77 (7·29 to 12·3)	11·1 (9·21 to 14·1)	15·4 (11·9 to 19·8)	39·2 (11·9 to 70·8)	7·22 (5·99 to 9·18)
	70+ years	24·0 (18·8 to 30·5)	22·5 (17·4 to 27·9)	-5·83 (-13·7 to 4·66)	19·2 (15·0 to 24·4)	13·0 (10·6 to 16·2)	16·7 (12·5 to 20·9)	28·2 (3·33 to 50·3)	8·46 (6·86 to 10·5)
Kuwait	All Ages	20·7 (17·5 to 24·0)	18·4 (15·4 to 22·4)	-11·3 (-17·4 to -5·08)	16·6 (14·0 to 19·2)	35·6 (33·4 to 38·8)	39·2 (35·2 to 42·9)	10·2 (-3·83 to 21·5)	23·1 (21·7 to 25·2)
	Under 5	4·70 (3·58 to 5·70)	3·87 (2·94 to 5·06)	-17·6 (-29·3 to -7·07)	3·76 (2·86 to 4·56)	0·643 (0·575 to 0·713)	0·280 (0·248 to 0·318)	-56·3 (-62·0 to -49·3)	0·418 (0·374 to 0·464)
	5-14 years	5·03 (3·47 to 7·29)	4·68 (3·06 to 6·81)	-6·96 (-19·0 to 6·44)	4·03 (2·77 to 5·83)	0·454 (0·408 to 0·515)	0·339 (0·304 to 0·373)	-25·1 (-35·9 to -16·3)	0·295 (0·265 to 0·334)
	15-49 years	22·5 (17·9 to 27·7)	18·9 (15·4 to 23·5)	-16·2 (-22·9 to -8·58)	18·0 (14·4 to 22·2)	12·5 (11·8 to 13·6)	11·9 (10·8 to 13·0)	-5·25 (-17·3 to 5·53)	8·15 (7·65 to 8·85)
	50-69 years	33·1 (25·2 to 41·6)	28·9 (21·6 to 37·3)	-12·5 (-21·7 to -5·84)	26·5 (20·2 to 33·3)	9·28 (8·45 to 10·1)	11·6 (10·3 to 13·1)	25·2 (7·66 to 40·3)	6·03 (5·49 to 6·59)
	70+ years	60·8 (49·5 to 73·1)	58·3 (47·0 to 71·2)	-3·99 (-13·0 to 5·61)	48·6 (39·6 to 58·5)	12·7 (11·1 to 14·1)	15·1 (13·0 to 16·8)	19·4 (3·55 to 32·5)	8·24 (7·23 to 9·19)
Lebanon	All Ages	14·4 (12·7 to 16·7)	13·4 (11·8 to 15·3)	-7·15 (-12·7 to -2·43)	11·5 (10·1 to 13·3)	64·9 (56·6 to 76·5)	60·5 (50·8 to 72·6)	-6·59 (-18·9 to 8·24)	42·2 (36·8 to 49·7)
	Under 5	4·44 (3·52 to 5·50)	3·57 (2·71 to 4·52)	-19·5 (-29·9 to -8·89)	3·55 (2·82 to 4·40)	0·567 (0·400 to 0·764)	0·408 (0·296 to 0·584)	-27·9 (-40·6 to -13·2)	0·369 (0·260 to 0·497)
	5-14 years	3·97 (2·78 to 5·56)	3·18 (2·14 to 4·62)	-19·7 (-28·4 to -9·74)	3·18 (2·22 to 4·45)	0·363 (0·275 to 0·455)	0·304 (0·245 to 0·388)	-15·7 (-31·2 to 1·93)	0·236 (0·179 to 0·296)
	15-49 years	12·0 (9·59 to 15·0)	10·4 (8·40 to 13·0)	-13·3 (-20·3 to -6·28)	9·58 (7·67 to 12·0)	13·0 (10·7 to 15·4)	11·6 (9·44 to 13·8)	-10·1 (-24·0 to 4·44)	8·44 (6·99 to 10·0)
	50-69 years	26·4 (19·6 to 33·0)	23·8 (18·2 to 29·7)	-9·97 (-16·4 to -1·82)	21·1 (15·6 to 26·4)	16·6 (13·5 to 19·7)	14·7 (12·6 to 17·6)	-11·2 (-24·0 to 7·10)	10·8 (8·80 to 12·8)
	70+ years	51·1 (41·5 to 63·7)	48·4 (39·0 to 59·2)	-5·13 (-13·8 to 2·68)	40·9 (33·2 to 51·0)	34·4 (28·7 to 41·9)	33·5 (26·7 to 42·0)	-2·47 (-17·2 to 14·2)	22·3 (18·7 to 27·2)
Libya	All Ages	29·1 (25·4 to 34·0)	28·4 (24·6 to 34·1)	-2·42 (-7·10 to 3·54)	23·3 (20·3 to 27·2)	146 (103 to 197)	164 (104 to 234)	11·5 (-8·47 to 32·6)	95·0 (66·8 to 128)

eTable 2. Progress towards the END-TB 2020 milestones in all-form tuberculosis incidence rate per 100,000 population and in deaths due to all-form tuberculosis by age for 204 countries and territories (2020), percent change from 2015 to 2020.

Location	Age group	2015 Rate	2020 Rate	Incidence Percent Change	Incidence Milestone	2015 Deaths	2020 Deaths	Mortality Percent Change	Mortality Milestone
	Under 5	13·0 (10·2 to 16·4)	12·2 (9·17 to 15·4)	-6·46 (-16·7 to 6·58)	10·4 (8·17 to 13·1)	4·91 (2·81 to 7·35)	3·99 (2·19 to 5·98)	-19·0 (-34·3 to -0·836)	3·19 (1·82 to 4·77)
	5-14 years	11·7 (8·18 to 16·4)	10·6 (7·31 to 15·3)	-8·59 (-19·5 to 3·87)	9·35 (6·55 to 13·1)	1·91 (1·30 to 2·54)	1·69 (1·08 to 2·49)	-11·5 (-28·4 to 5·80)	1·24 (0·843 to 1·65)
	15-49 years	28·1 (22·6 to 34·3)	25·9 (20·9 to 32·8)	-7·85 (-15·7 to 0·640)	22·5 (18·1 to 27·4)	54·2 (36·4 to 78·4)	56·4 (34·1 to 85·0)	3·45 (-16·3 to 26·1)	35·3 (23·7 to 51·0)
	50-69 years	53·7 (41·6 to 66·8)	49·7 (37·5 to 62·5)	-7·36 (-13·0 to 1·79)	42·9 (33·3 to 53·4)	38·7 (25·4 to 53·9)	49·9 (30·4 to 76·9)	28·1 (3·68 to 55·4)	25·2 (16·5 to 35·1)
	70+ years	106 (85·6 to 132)	102 (80·8 to 128)	-4·24 (-12·3 to 6·98)	85·1 (68·4 to 105)	46·4 (33·0 to 63·7)	51·6 (34·6 to 73·2)	11·4 (-11·4 to 37·0)	30·1 (21·5 to 41·4)
Morocco	All Ages	83·4 (72·7 to 95·1)	73·1 (64·8 to 83·9)	-12·3 (-16·8 to -7·36)	66·7 (58·2 to 76·1)	4170 (2710 to 8680)	3620 (2280 to 7990)	-13·5 (-26·7 to 3·55)	2710 (1760 to 5640)
	Under 5	31·0 (24·2 to 38·6)	23·5 (18·4 to 29·8)	-23·9 (-33·8 to -11·0)	24·8 (19·4 to 30·8)	101 (73·4 to 142)	45·8 (30·9 to 72·5)	-54·8 (-64·7 to -43·4)	65·7 (47·7 to 92·6)
	5-14 years	35·9 (25·0 to 51·9)	28·1 (19·9 to 40·5)	-21·6 (-32·6 to -7·73)	28·7 (20·0 to 41·5)	53·1 (40·6 to 76·4)	30·0 (22·9 to 42·0)	-43·1 (-53·7 to -30·4)	34·5 (26·4 to 49·6)
	15-49 years	77·2 (63·2 to 92·3)	64·5 (51·9 to 81·6)	-16·4 (-22·4 to -11·5)	61·8 (50·5 to 73·9)	1080 (740 to 1810)	780 (554 to 1430)	-28·0 (-39·5 to -12·0)	704 (481 to 1180)
	50-69 years	144 (110 to 189)	129 (97·2 to 162)	-10·4 (-18·6 to -0·110)	116 (87·7 to 151)	1290 (768 to 2840)	1270 (747 to 2890)	-1·48 (-20·9 to 22·5)	839 (499 to 1850)
	70+ years	284 (222 to 354)	247 (195 to 305)	-12·8 (-21·8 to -4·68)	227 (177 to 284)	1640 (996 to 4040)	1490 (857 to 3580)	-8·83 (-23·1 to 13·3)	1060 (647 to 2630)
Oman	All Ages	8·59 (7·52 to 10·1)	7·25 (6·34 to 8·62)	-15·5 (-20·0 to -9·45)	6·87 (6·02 to 8·06)	22·4 (16·9 to 31·2)	17·8 (14·3 to 26·4)	-20·2 (-32·4 to -4·70)	14·5 (11·0 to 20·3)
	Under 5	3·87 (3·08 to 4·90)	3·09 (2·31 to 3·92)	-19·9 (-29·6 to -6·34)	3·09 (2·46 to 3·92)	0·617 (0·459 to 0·857)	0·395 (0·301 to 0·539)	-35·8 (-45·0 to -26·8)	0·401 (0·298 to 0·557)
	5-14 years	3·71 (2·57 to 5·33)	3·13 (2·13 to 4·57)	-15·5 (-26·3 to -4·84)	2·97 (2·05 to 4·26)	0·283 (0·219 to 0·385)	0·185 (0·137 to 0·242)	-34·5 (-45·6 to -15·3)	0·184 (0·143 to 0·250)
	15-49 years	8·18 (6·42 to 10·0)	6·70 (5·53 to 8·24)	-17·9 (-23·8 to -8·23)	6·54 (5·14 to 8·02)	8·23 (6·20 to 12·2)	6·39 (4·89 to 10·1)	-22·3 (-36·8 to -3·62)	5·35 (4·03 to 7·91)
	50-69 years	19·9 (15·0 to 25·5)	16·8 (12·4 to 21·3)	-15·2 (-21·9 to -9·82)	15·9 (12·0 to 20·4)	6·32 (4·48 to 8·53)	4·29 (3·22 to 6·12)	-31·5 (-45·7 to 0·315)	4·11 (2·91 to 5·55)
	70+ years	52·9 (41·8 to 66·5)	48·7 (38·3 to 61·8)	-7·85 (-16·6 to 0·905)	42·3 (33·4 to 53·2)	6·93 (4·61 to 9·95)	6·57 (4·27 to 9·72)	-5·10 (-21·5 to 16·7)	4·50 (3·00 to 6·47)
Palestine	All Ages	8·88 (7·65 to 10·3)	7·74 (6·67 to 8·90)	-12·8 (-17·4 to -8·44)	7·11 (6·12 to 8·21)	32·4 (24·7 to 42·7)	28·0 (19·6 to 37·8)	-13·8 (-24·8 to 1·50)	21·1 (16·1 to 27·8)
	Under 5	4·75 (3·67 to 5·98)	3·73 (2·81 to 4·71)	-21·3 (-30·4 to -8·97)	3·80 (2·93 to 4·79)	1·76 (1·21 to 2·53)	1·04 (0·712 to 1·64)	-41·1 (-53·9 to -24·3)	1·14 (0·784 to 1·64)
	5-14 years	4·20 (2·91 to 6·05)	3·45 (2·40 to 4·86)	-17·7 (-28·8 to -5·52)	3·36 (2·33 to 4·84)	1·06 (0·788 to 1·63)	0·656 (0·466 to 0·953)	-37·8 (-49·2 to -23·8)	0·689 (0·512 to 1·06)
	15-49 years	8·72 (7·05 to 10·8)	7·40 (5·92 to 9·18)	-15·1 (-21·5 to -9·88)	6·98 (5·64 to 8·65)	7·48 (5·57 to 11·2)	6·46 (4·49 to 10·5)	-13·6 (-27·3 to 0·522)	4·86 (3·62 to 7·28)
	50-69 years	21·9 (17·1 to 28·0)	18·6 (14·0 to 23·6)	-15·2 (-22·9 to -8·55)	17·5 (13·7 to 22·4)	9·73 (6·82 to 13·6)	8·89 (5·64 to 13·3)	-8·64 (-25·4 to 11·6)	6·32 (4·43 to 8·87)

eTable 2. Progress towards the END-TB 2020 milestones in all-form tuberculosis incidence rate per 100,000 population and in deaths due to all-form tuberculosis by age for 204 countries and territories (2020), percent change from 2015 to 2020.

Location	Age group	2015 Rate	2020 Rate	Incidence Percent Change	Incidence Milestone	2015 Deaths	2020 Deaths	Mortality Percent Change	Mortality Milestone
	70+ years	50·1 (40·0 to 63·9)	41·6 (33·0 to 52·0)	-16·7 (-23·9 to -9·64)	40·0 (32·0 to 51·1)	12·4 (9·46 to 16·2)	10·9 (8·16 to 14·5)	-11·6 (-24·5 to 12·0)	8·06 (6·15 to 10·5)
Qatar	All Ages	29·9 (25·6 to 36·5)	24·8 (21·1 to 30·2)	-16·9 (-22·3 to -10·4)	23·9 (20·5 to 29·2)	28·7 (22·7 to 36·0)	20·7 (14·5 to 27·7)	-28·0 (-42·2 to -14·9)	18·7 (14·7 to 23·4)
	Under 5	10·5 (8·10 to 13·2)	8·21 (6·29 to 10·5)	-22·0 (-32·0 to -11·4)	8·44 (6·48 to 10·5)	0·418 (0·322 to 0·535)	0·312 (0·216 to 0·422)	-25·3 (-37·4 to -10·1)	0·272 (0·209 to 0·348)
	5-14 years	10·9 (7·40 to 15·9)	9·35 (6·28 to 13·8)	-14·1 (-25·2 to -0·317)	8·74 (5·92 to 12·7)	0·236 (0·192 to 0·300)	0·152 (0·121 to 0·196)	-35·5 (-47·4 to -20·7)	0·153 (0·125 to 0·195)
	15-49 years	29·7 (24·3 to 37·7)	24·8 (19·9 to 30·8)	-16·5 (-23·1 to -8·77)	23·7 (19·4 to 30·2)	16·7 (12·7 to 21·0)	12·8 (8·96 to 16·9)	-23·2 (-39·3 to -8·18)	10·9 (8·26 to 13·7)
	50-69 years	55·7 (40·7 to 69·5)	46·0 (33·7 to 57·7)	-17·4 (-24·8 to -9·50)	44·6 (32·6 to 55·6)	7·41 (5·74 to 9·43)	4·34 (2·80 to 6·20)	-41·4 (-54·5 to -26·3)	4·82 (3·73 to 6·13)
	70+ years	185 (140 to 235)	154 (122 to 189)	-16·6 (-26·8 to -7·18)	148 (112 to 188)	3·94 (3·18 to 5·10)	3·04 (2·30 to 4·20)	-22·7 (-38·7 to -4·90)	2·56 (2·07 to 3·31)
Saudi Arabia	All Ages	45·6 (38·4 to 54·1)	40·5 (34·2 to 49·5)	-11·2 (-17·8 to -4·76)	36·5 (30·7 to 43·3)	1220 (993 to 1550)	1150 (857 to 1650)	-6·07 (-22·1 to 12·9)	793 (646 to 1010)
	Under 5	9·39 (7·27 to 11·7)	6·74 (5·33 to 8·52)	-28·0 (-38·1 to -16·9)	7·51 (5·81 to 9·34)	7·95 (5·88 to 10·1)	4·07 (2·93 to 5·90)	-48·9 (-58·0 to -35·6)	5·17 (3·82 to 6·57)
	5-14 years	13·0 (9·28 to 18·6)	9·68 (6·55 to 13·7)	-25·1 (-36·0 to -10·7)	10·4 (7·42 to 14·9)	6·44 (5·00 to 8·62)	4·19 (3·07 to 5·46)	-34·9 (-45·9 to -21·5)	4·18 (3·25 to 5·60)
	15-49 years	48·6 (37·9 to 60·9)	42·4 (34·1 to 52·9)	-12·6 (-20·5 to -5·24)	38·9 (30·3 to 48·7)	644 (503 to 861)	609 (427 to 911)	-5·71 (-25·1 to 16·2)	418 (327 to 560)
	50-69 years	90·6 (66·9 to 113)	75·5 (56·7 to 95·7)	-16·7 (-23·0 to -8·68)	72·5 (53·5 to 90·3)	327 (264 to 423)	319 (236 to 449)	-2·80 (-19·4 to 20·6)	213 (171 to 275)
	70+ years	177 (141 to 221)	141 (112 to 174)	-20·3 (-28·4 to -9·74)	142 (112 to 177)	235 (187 to 304)	213 (159 to 301)	-9·22 (-22·6 to 11·8)	153 (122 to 197)
Sudan	All Ages	48·1 (41·2 to 54·5)	40·7 (35·1 to 46·4)	-15·5 (-19·7 to -10·8)	38·5 (32·9 to 43·6)	1900 (1230 to 2690)	1600 (1010 to 2310)	-15·5 (-25·0 to -1·64)	1240 (799 to 1750)
	Under 5	26·1 (20·2 to 32·8)	20·3 (15·7 to 25·3)	-21·8 (-33·2 to -11·3)	20·8 (16·1 to 26·3)	290 (196 to 408)	162 (95·6 to 226)	-44·2 (-53·3 to -33·7)	189 (127 to 265)
	5-14 years	25·0 (17·7 to 35·6)	20·3 (14·1 to 28·1)	-18·8 (-29·2 to -9·39)	20·0 (14·1 to 28·5)	69·7 (43·4 to 97·1)	49·8 (29·6 to 72·2)	-27·8 (-41·4 to -11·0)	45·3 (28·2 to 63·1)
	15-49 years	52·3 (42·0 to 63·0)	43·3 (35·1 to 53·5)	-17·2 (-22·9 to -11·5)	41·9 (33·6 to 50·4)	668 (393 to 1010)	579 (365 to 859)	-12·9 (-27·0 to 1·65)	434 (256 to 658)
	50-69 years	104 (80·2 to 130)	87·9 (66·8 to 110)	-15·3 (-22·4 to -7·91)	83·1 (64·1 to 104)	413 (242 to 603)	393 (241 to 600)	-4·39 (-18·0 to 11·4)	269 (158 to 392)
	70+ years	206 (167 to 256)	178 (142 to 218)	-13·7 (-21·9 to -3·88)	165 (133 to 205)	459 (267 to 700)	418 (257 to 612)	-8·28 (-21·8 to 12·9)	299 (173 to 455)
Syrian Arab Republic	All Ages	10·1 (8·79 to 12·0)	10·1 (8·85 to 11·6)	-0·126 (-6·65 to 6·05)	8·07 (7·03 to 9·63)	118 (87·6 to 149)	104 (76·3 to 137)	-11·4 (-30·7 to 9·13)	76·4 (57·0 to 96·9)
	Under 5	3·70 (2·96 to 4·78)	3·13 (2·43 to 4·01)	-15·2 (-26·4 to -4·84)	2·96 (2·37 to 3·83)	2·97 (2·06 to 4·31)	1·06 (0·707 to 1·55)	-64·1 (-71·8 to -53·3)	1·93 (1·34 to 2·80)
	5-14 years	5·17 (3·66 to 7·60)	4·90 (3·27 to 6·97)	-4·98 (-16·1 to 10·1)	4·14 (2·93 to 6·08)	3·47 (2·74 to 4·31)	1·67 (1·29 to 2·39)	-51·9 (-60·1 to -39·8)	2·26 (1·78 to 2·80)

eTable 2. Progress towards the END-TB 2020 milestones in all-form tuberculosis incidence rate per 100,000 population and in deaths due to all-form tuberculosis by age for 204 countries and territories (2020), percent change from 2015 to 2020.

Location	Age group	2015 Rate	2020 Rate	Incidence Percent Change	Incidence Milestone	2015 Deaths	2020 Deaths	Mortality Percent Change	Mortality Milestone
	15-49 years	10·5 (8·57 to 12·9)	9·69 (7·66 to 11·9)	-7·25 (-15·6 to 0·820)	8·36 (6·85 to 10·4)	30·8 (22·9 to 38·8)	22·0 (15·9 to 31·0)	-28·0 (-45·6 to -5·09)	20·0 (14·9 to 25·2)
	50-69 years	17·3 (13·3 to 21·5)	15·6 (12·0 to 19·8)	-10·1 (-16·4 to -4·80)	13·9 (10·6 to 17·2)	38·9 (27·0 to 51·8)	39·1 (27·3 to 55·9)	1·33 (-23·8 to 31·2)	25·3 (17·6 to 33·7)
	70+ years	31·9 (25·0 to 40·3)	27·5 (21·9 to 33·7)	-13·8 (-22·2 to -1·43)	25·5 (20·0 to 32·2)	41·4 (32·4 to 51·2)	39·7 (31·1 to 52·4)	-3·56 (-21·5 to 19·5)	26·9 (21·1 to 33·3)
Tunisia	All Ages	28·2 (25·0 to 32·8)	24·2 (21·5 to 27·9)	-14·1 (-18·9 to -10·5)	22·5 (20·0 to 26·2)	260 (180 to 377)	236 (167 to 395)	-9·21 (-25·9 to 11·6)	169 (117 to 245)
	Under 5	11·6 (8·89 to 14·7)	8·93 (6·68 to 11·3)	-22·4 (-36·7 to -10·4)	9·24 (7·12 to 11·7)	3·57 (2·38 to 5·59)	1·95 (1·25 to 3·19)	-45·5 (-59·2 to -29·6)	2·32 (1·55 to 3·63)
	5-14 years	9·55 (6·43 to 13·8)	7·35 (4·99 to 10·5)	-22·9 (-32·6 to -9·76)	7·64 (5·14 to 11·0)	1·94 (1·36 to 2·99)	1·37 (0·956 to 2·35)	-29·0 (-43·3 to -15·9)	1·26 (0·881 to 1·95)
	15-49 years	24·2 (19·6 to 29·7)	20·0 (16·2 to 24·6)	-17·0 (-22·4 to -12·2)	19·3 (15·6 to 23·7)	56·5 (36·2 to 86·9)	47·4 (33·4 to 79·6)	-16·0 (-29·9 to 3·52)	36·7 (23·5 to 56·5)
	50-69 years	45·0 (34·3 to 57·7)	38·2 (29·4 to 48·2)	-15·0 (-20·6 to -8·78)	36·0 (27·5 to 46·2)	73·2 (47·3 to 112)	70·6 (44·6 to 122)	-3·19 (-25·3 to 21·6)	47·6 (30·7 to 72·5)
	70+ years	97·2 (79·7 to 119)	80·6 (63·3 to 101)	-16·9 (-26·1 to -10·2)	77·7 (63·7 to 94·9)	125 (84·4 to 174)	115 (77·9 to 188)	-8·01 (-26·1 to 12·5)	81·2 (54·9 to 113)
Türkiye	All Ages	14·9 (13·0 to 17·5)	13·1 (11·6 to 15·3)	-12·0 (-16·6 to -7·66)	11·9 (10·4 to 14·0)	1030 (913 to 1190)	907 (725 to 1120)	-11·7 (-29·0 to 4·43)	669 (593 to 771)
	Under 5	5·16 (4·12 to 6·48)	3·79 (3·01 to 4·77)	-26·3 (-38·3 to -14·5)	4·13 (3·30 to 5·19)	17·7 (13·5 to 22·2)	8·67 (6·26 to 11·5)	-51·0 (-59·7 to -40·0)	11·5 (8·80 to 14·4)
	5-14 years	5·00 (3·42 to 6·95)	3·93 (2·69 to 5·49)	-21·2 (-31·1 to -9·03)	4·00 (2·74 to 5·56)	8·66 (7·12 to 10·4)	6·23 (5·09 to 7·55)	-28·0 (-39·1 to -14·8)	5·63 (4·63 to 6·76)
	15-49 years	12·0 (9·71 to 14·9)	10·1 (8·05 to 12·7)	-16·3 (-22·2 to -10·4)	9·61 (7·76 to 12·0)	192 (167 to 222)	150 (123 to 189)	-21·7 (-35·1 to -7·67)	125 (108 to 144)
	50-69 years	26·3 (18·1 to 34·5)	23·0 (17·1 to 29·5)	-12·6 (-21·2 to -4·73)	21·1 (14·4 to 27·6)	328 (272 to 394)	297 (236 to 382)	-9·02 (-27·3 to 17·8)	213 (177 to 256)
	70+ years	51·3 (40·1 to 63·8)	43·9 (34·5 to 56·7)	-14·4 (-21·8 to -4·99)	41·0 (32·0 to 51·0)	482 (401 to 573)	445 (345 to 545)	-7·57 (-26·4 to 9·53)	313 (261 to 372)
United Arab Emirates	All Ages	5·56 (4·66 to 6·67)	4·64 (3·95 to 5·65)	-16·2 (-24·6 to -5·77)	4·45 (3·73 to 5·34)	39·3 (17·7 to 60·2)	34·5 (14·9 to 52·5)	-10·5 (-29·6 to 14·7)	25·5 (11·5 to 39·1)
	Under 5	3·28 (2·60 to 4·07)	2·49 (1·94 to 3·18)	-23·9 (-32·8 to -11·9)	2·62 (2·08 to 3·26)	0·374 (0·169 to 0·815)	0·239 (0·108 to 0·546)	-36·2 (-49·3 to -19·7)	0·243 (0·110 to 0·530)
	5-14 years	1·92 (1·23 to 2·74)	1·29 (0·843 to 1·97)	-32·4 (-41·6 to -21·7)	1·53 (0·981 to 2·20)	0·202 (0·0933 to 0·384)	0·137 (0·0574 to 0·235)	-31·0 (-45·1 to -4·56)	0·131 (0·0607 to 0·250)
	15-49 years	5·35 (4·20 to 6·65)	4·10 (3·38 to 5·31)	-23·1 (-33·3 to -12·3)	4·28 (3·36 to 5·32)	22·2 (8·53 to 36·7)	16·1 (6·58 to 25·8)	-26·4 (-44·6 to 0·200)	14·4 (5·54 to 23·8)
	50-69 years	12·0 (8·88 to 15·3)	9·52 (7·30 to 12·0)	-20·6 (-27·5 to -12·5)	9·59 (7·10 to 12·3)	8·18 (3·32 to 15·2)	8·03 (3·29 to 15·8)	0·0970 (-24·6 to 30·2)	5·32 (2·16 to 9·89)
	70+ years	51·3 (38·6 to 64·3)	45·8 (35·9 to 57·3)	-10·5 (-18·8 to 0·170)	41·1 (30·9 to 51·4)	8·30 (3·82 to 11·4)	10·1 (4·41 to 14·0)	22·7 (-3·79 to 53·1)	5·40 (2·48 to 7·41)
Yemen	All Ages	21·0 (18·3 to 23·6)	20·0 (17·5 to 22·6)	-4·94 (-9·72 to -0·0625)	16·8 (14·7 to 18·9)	841 (520 to 1240)	949 (582 to 1420)	13·0 (-1·36 to 30·0)	547 (338 to 806)

eTable 2. Progress towards the END-TB 2020 milestones in all-form tuberculosis incidence rate per 100,000 population and in deaths due to all-form tuberculosis by age for 204 countries and territories (2020), percent change from 2015 to 2020.

Location	Age group	2015 Rate	2020 Rate	Incidence Percent Change	Incidence Milestone	2015 Deaths	2020 Deaths	Mortality Percent Change	Mortality Milestone
	Under 5	11·6 (9·04 to 14·5)	10·2 (7·85 to 12·6)	-12·3 (-21·6 to -2·58)	9·29 (7·23 to 11·6)	122 (77·5 to 173)	94·6 (62·1 to 137)	-21·9 (-37·3 to -2·18)	79·2 (50·4 to 112)
	5-14 years	9·08 (6·41 to 12·9)	7·92 (5·60 to 11·0)	-12·6 (-22·9 to -0·681)	7·26 (5·13 to 10·3)	28·9 (16·8 to 44·0)	29·4 (16·1 to 44·2)	2·07 (-24·0 to 27·7)	18·8 (10·9 to 28·6)
	15-49 years	23·2 (19·1 to 28·0)	21·6 (17·6 to 26·4)	-6·93 (-12·7 to -0·342)	18·6 (15·2 to 22·4)	252 (157 to 396)	298 (186 to 491)	18·2 (1·72 to 42·1)	164 (102 to 257)
	50-69 years	55·9 (43·0 to 69·4)	53·6 (41·7 to 66·9)	-3·91 (-11·5 to 4·67)	44·7 (34·4 to 55·5)	229 (129 to 364)	276 (150 to 428)	20·8 (0·178 to 42·2)	149 (83·8 to 237)
	70+ years	103 (82·3 to 130)	98·6 (78·3 to 123)	-4·22 (-14·7 to 4·88)	82·5 (65·8 to 104)	209 (126 to 331)	252 (160 to 373)	21·0 (4·25 to 41·2)	136 (81·9 to 215)
South Asia	All Ages	205 (177 to 235)	199 (172 to 228)	-3·06 (-5·10 to -1·25)	164 (142 to 188)	573000 (536000 to 611000)	523000 (460000 to 598000)	-8·71 (-16·4 to 1·24)	373000 (348000 to 397000)
	Under 5	48·2 (39·0 to 59·7)	43·1 (34·0 to 53·7)	-10·7 (-14·6 to -7·34)	38·6 (31·2 to 47·8)	17800 (15300 to 20200)	11200 (9140 to 13800)	-36·9 (-46·9 to -26·0)	11600 (9920 to 13100)
	5-14 years	47·7 (33·0 to 67·5)	40·6 (27·9 to 56·7)	-14·9 (-19·4 to -11·2)	38·2 (26·4 to 54·0)	8230 (7250 to 9190)	6090 (5150 to 7050)	-26·1 (-32·7 to -17·4)	5350 (4710 to 5970)
	15-49 years	216 (181 to 273)	201 (171 to 254)	-6·59 (-9·20 to -4·10)	173 (145 to 218)	186000 (172000 to 202000)	169000 (148000 to 188000)	-9·39 (-19·3 to -0·412)	121000 (112000 to 131000)
	50-69 years	440 (331 to 561)	410 (315 to 516)	-6·61 (-9·71 to -4·01)	352 (265 to 449)	213000 (198000 to 229000)	189000 (164000 to 224000)	-11·0 (-19·3 to 0·197)	138000 (129000 to 149000)
	70+ years	560 (436 to 707)	534 (418 to 660)	-4·52 (-7·94 to -1·36)	448 (349 to 566)	148000 (137000 to 160000)	148000 (130000 to 170000)	-0·103 (-9·08 to 13·1)	96100 (89100 to 104000)
Bangladesh	All Ages	158 (134 to 182)	143 (122 to 164)	-9·45 (-15·7 to -2·83)	126 (107 to 146)	31100 (27100 to 35500)	29600 (24000 to 35500)	-5·02 (-18·3 to 12·0)	20200 (17600 to 23100)
	Under 5	47·0 (36·3 to 60·4)	35·7 (28·0 to 47·0)	-23·8 (-32·5 to -14·6)	37·6 (29·1 to 48·3)	1250 (875 to 1680)	584 (401 to 777)	-52·8 (-64·0 to -40·1)	812 (569 to 1090)
	5-14 years	64·2 (43·3 to 92·4)	52·5 (36·2 to 73·0)	-18·0 (-26·2 to -7·22)	51·4 (34·7 to 73·9)	1010 (801 to 1240)	693 (517 to 893)	-31·3 (-46·2 to -18·1)	657 (521 to 809)
	15-49 years	159 (128 to 194)	139 (112 to 170)	-12·4 (-21·0 to -5·14)	127 (102 to 156)	8740 (7270 to 10200)	7700 (6160 to 9580)	-11·8 (-24·4 to 6·58)	5680 (4730 to 6600)
	50-69 years	326 (238 to 419)	275 (210 to 353)	-15·4 (-21·8 to -5·72)	261 (191 to 335)	10400 (8620 to 12700)	9580 (7290 to 12700)	-7·49 (-24·4 to 13·0)	6740 (5600 to 8270)
	70+ years	454 (359 to 575)	392 (316 to 500)	-13·5 (-22·4 to -6·17)	363 (287 to 460)	9780 (7830 to 11600)	11000 (8540 to 13600)	12·9 (-4·49 to 35·4)	6360 (5090 to 7510)
Bhutan	All Ages	120 (101 to 142)	112 (92·7 to 133)	-6·61 (-13·4 to -0·213)	96·1 (80·6 to 114)	125 (69·4 to 204)	110 (61·2 to 199)	-12·2 (-22·3 to 0·685)	81·2 (45·1 to 133)
	Under 5	24·8 (18·3 to 31·9)	20·1 (15·3 to 25·8)	-18·7 (-29·8 to -9·04)	19·8 (14·6 to 25·5)	2·94 (1·46 to 4·77)	1·77 (0·981 to 2·95)	-39·3 (-52·2 to -20·1)	1·91 (0·947 to 3·10)
	5-14 years	26·3 (18·3 to 40·0)	21·8 (14·4 to 32·8)	-16·9 (-30·3 to -2·07)	21·1 (14·6 to 32·0)	1·06 (0·601 to 1·83)	0·725 (0·404 to 1·24)	-31·1 (-45·9 to -11·2)	0·691 (0·390 to 1·19)
	15-49 years	119 (94·7 to 148)	107 (84·9 to 134)	-9·96 (-18·6 to 1·01)	94·9 (75·7 to 118)	33·2 (18·1 to 58·4)	28·1 (15·2 to 51·8)	-15·0 (-31·1 to 5·96)	21·6 (11·8 to 38·0)

eTable 2. Progress towards the END-TB 2020 milestones in all-form tuberculosis incidence rate per 100,000 population and in deaths due to all-form tuberculosis by age for 204 countries and territories (2020), percent change from 2015 to 2020.

Location	Age group	2015 Rate	2020 Rate	Incidence Percent Change	Incidence Milestone	2015 Deaths	2020 Deaths	Mortality Percent Change	Mortality Milestone
	50-69 years	267 (197 to 357)	239 (179 to 313)	-10·1 (-18·2 to -0·596)	214 (158 to 286)	38·8 (21·0 to 72·5)	34·5 (18·6 to 70·0)	-11·4 (-24·6 to 7·93)	25·2 (13·7 to 47·1)
	70+ years	388 (294 to 502)	342 (270 to 436)	-11·7 (-20·8 to 0·0787)	311 (235 to 402)	48·9 (25·7 to 78·4)	44·9 (23·6 to 73·4)	-8·49 (-20·6 to 11·4)	31·8 (16·7 to 50·9)
India	All Ages	214 (185 to 247)	213 (184 to 246)	-0·512 (-2·98 to 1·71)	171 (148 to 198)	458000 (419000 to 496000)	416000 (354000 to 495000)	-9·17 (-18·4 to 2·37)	297000 (272000 to 322000)
	Under 5	39·2 (31·9 to 48·4)	36·2 (27·9 to 45·2)	-7·60 (-14·2 to -1·98)	31·3 (25·5 to 38·7)	9590 (7730 to 11800)	5450 (4100 to 7160)	-43·0 (-56·1 to -26·0)	6230 (5030 to 7690)
	5-14 years	41·0 (28·2 to 57·9)	34·9 (23·8 to 49·2)	-15·0 (-21·0 to -9·24)	32·8 (22·6 to 46·3)	4580 (3910 to 5380)	3280 (2560 to 4070)	-28·5 (-39·0 to -17·5)	2980 (2540 to 3490)
	15-49 years	225 (188 to 284)	215 (181 to 271)	-4·47 (-7·74 to -1·88)	180 (150 to 227)	147000 (135000 to 160000)	133000 (114000 to 158000)	-9·53 (-20·4 to 2·33)	95700 (87800 to 104000)
	50-69 years	456 (343 to 583)	434 (335 to 547)	-4·76 (-8·24 to -1·50)	365 (275 to 466)	177000 (161000 to 194000)	155000 (132000 to 188000)	-12·4 (-22·0 to 0·170)	115000 (105000 to 126000)
	70+ years	565 (436 to 717)	553 (432 to 681)	-2·08 (-5·94 to 1·99)	452 (349 to 574)	119000 (108000 to 130000)	118000 (101000 to 141000)	-0·424 (-10·1 to 13·3)	77300 (70100 to 84800)
Nepal	All Ages	164 (143 to 187)	142 (122 to 164)	-13·7 (-18·5 to -8·99)	131 (115 to 150)	8060 (5600 to 10300)	7270 (4930 to 10100)	-9·69 (-25·7 to 5·96)	5240 (3640 to 6720)
	Under 5	41·6 (31·8 to 52·2)	31·1 (24·5 to 38·9)	-25·3 (-33·2 to -14·5)	33·3 (25·4 to 41·8)	125 (87·2 to 175)	72·0 (47·8 to 102)	-42·2 (-54·5 to -27·1)	81·4 (56·7 to 114)
	5-14 years	34·7 (25·1 to 47·7)	27·1 (19·2 to 38·8)	-21·6 (-30·2 to -11·5)	27·8 (20·0 to 38·2)	83·0 (57·2 to 109)	55·1 (40·9 to 72·1)	-33·3 (-43·9 to -18·6)	53·9 (37·2 to 70·8)
	15-49 years	153 (126 to 196)	125 (104 to 160)	-18·2 (-24·8 to -11·5)	122 (101 to 157)	2080 (1530 to 2790)	1740 (1230 to 2620)	-16·2 (-29·0 to -1·87)	1350 (995 to 1810)
	50-69 years	425 (326 to 546)	357 (283 to 460)	-15·9 (-23·6 to -9·36)	340 (261 to 437)	3080 (2030 to 4210)	2790 (1790 to 4090)	-9·42 (-30·0 to 11·8)	2010 (1320 to 2730)
	70+ years	639 (513 to 792)	536 (416 to 673)	-16·0 (-25·3 to -6·42)	511 (410 to 634)	2690 (1810 to 3590)	2620 (1800 to 3610)	-2·34 (-21·5 to 23·6)	1750 (1180 to 2340)
Pakistan	All Ages	187 (164 to 211)	158 (137 to 177)	-15·2 (-19·3 to -11·6)	149 (131 to 169)	76300 (56000 to 95200)	70500 (50200 to 88800)	-7·00 (-26·3 to 16·5)	49600 (36400 to 61900)
	Under 5	88·9 (70·9 to 111)	74·3 (59·6 to 93·7)	-16·4 (-22·2 to -11·1)	71·1 (56·7 to 88·9)	6820 (4760 to 8680)	5120 (4040 to 6700)	-24·3 (-41·6 to -6·76)	4430 (3100 to 5640)
	5-14 years	74·6 (51·3 to 108)	62·2 (42·4 to 88·9)	-16·6 (-23·5 to -9·34)	59·7 (41·0 to 86·2)	2550 (2040 to 3110)	2060 (1640 to 2500)	-18·8 (-35·4 to -1·02)	1660 (1320 to 2020)
	15-49 years	208 (174 to 262)	173 (146 to 215)	-16·9 (-21·6 to -12·5)	166 (139 to 210)	28200 (21000 to 35800)	26000 (18100 to 33400)	-6·98 (-28·5 to 22·2)	18300 (13700 to 23300)
	50-69 years	412 (314 to 529)	348 (263 to 442)	-15·4 (-21·4 to -9·20)	329 (251 to 423)	22200 (16000 to 29100)	21600 (14700 to 29600)	-1·64 (-27·2 to 32·8)	14400 (10400 to 18900)
	70+ years	614 (481 to 764)	517 (408 to 637)	-15·8 (-21·9 to -7·74)	491 (385 to 611)	16500 (11300 to 21400)	15600 (11000 to 19900)	-4·53 (-22·4 to 17·7)	10700 (7340 to 13900)
Southeast Asia, East Asia, and Oceania	All Ages	97·5 (88·6 to 106)	95·9 (87·5 to 105)	-1·72 (-3·32 to -0·442)	78·0 (70·9 to 85·1)	265000 (248000 to 286000)	240000 (219000 to 273000)	-9·45 (-16·9 to -0·0543)	173000 (161000 to 186000)

eTable 2. Progress towards the END-TB 2020 milestones in all-form tuberculosis incidence rate per 100,000 population and in deaths due to all-form tuberculosis by age for 204 countries and territories (2020), percent change from 2015 to 2020.

Location	Age group	2015 Rate	2020 Rate	Incidence Percent Change	Incidence Milestone	2015 Deaths	2020 Deaths	Mortality Percent Change	Mortality Milestone
	Under 5	39·1 (32·6 to 48·3)	34·6 (28·9 to 42·7)	-11·4 (-13·6 to -9·52)	31·3 (26·1 to 38·6)	6020 (5020 to 6970)	3860 (3090 to 4750)	-35·9 (-43·1 to -28·5)	3910 (3260 to 4530)
	5-14 years	30·4 (21·7 to 41·4)	27·8 (19·9 to 37·4)	-8·39 (-11·0 to -6·19)	24·3 (17·3 to 33·1)	2810 (2540 to 3070)	1950 (1720 to 2210)	-30·7 (-36·6 to -25·2)	1830 (1650 to 2000)
	15-49 years	84·6 (72·5 to 97·1)	83·6 (71·3 to 97·2)	-1·21 (-3·31 to 1·25)	67·7 (58·0 to 77·7)	77800 (72200 to 84400)	62000 (56500 to 70500)	-20·2 (-29·0 to -10·5)	50500 (46900 to 54900)
	50-69 years	154 (127 to 184)	145 (119 to 175)	-5·56 (-8·08 to -3·40)	123 (102 to 148)	92300 (85400 to 99900)	90700 (80000 to 104000)	-1·65 (-10·9 to 10·4)	60000 (55500 to 64900)
	70+ years	244 (205 to 285)	215 (181 to 256)	-12·0 (-14·5 to -9·23)	195 (164 to 228)	86500 (78700 to 95000)	81700 (74000 to 93800)	-5·59 (-12·2 to 4·66)	56300 (51100 to 61800)
East Asia	All Ages	53·9 (48·9 to 59·0)	51·1 (45·9 to 56·4)	-5·20 (-8·50 to -2·56)	43·1 (39·1 to 47·2)	58500 (52700 to 65600)	51000 (43700 to 59900)	-12·6 (-24·6 to 1·07)	38000 (34200 to 42700)
	Under 5	17·7 (14·7 to 21·7)	16·2 (13·6 to 19·9)	-8·04 (-13·3 to -3·30)	14·1 (11·8 to 17·4)	806 (723 to 905)	403 (332 to 482)	-50·0 (-56·2 to -42·5)	524 (470 to 588)
	5-14 years	11·3 (8·09 to 15·3)	9·71 (7·09 to 13·6)	-13·9 (-19·6 to -7·58)	9·02 (6·47 to 12·2)	215 (192 to 240)	145 (123 to 172)	-32·5 (-38·5 to -24·2)	140 (125 to 156)
	15-49 years	43·7 (38·1 to 50·9)	41·5 (35·4 to 48·4)	-4·99 (-9·09 to 0·984)	34·9 (30·5 to 40·7)	13900 (12300 to 15500)	10200 (8410 to 12200)	-26·5 (-36·3 to -15·8)	9010 (7980 to 10100)
	50-69 years	81·8 (66·9 to 97·5)	74·3 (60·6 to 89·8)	-9·17 (-14·7 to -5·52)	65·4 (53·5 to 78·0)	20600 (18200 to 22900)	19500 (16200 to 22700)	-5·40 (-19·7 to 11·3)	13400 (11900 to 14900)
	70+ years	145 (118 to 172)	122 (98·2 to 149)	-15·7 (-19·8 to -11·2)	116 (94·4 to 137)	23000 (20800 to 26400)	20800 (18100 to 24700)	-9·30 (-20·8 to 3·99)	14900 (13500 to 17200)
China	All Ages	48·8 (44·2 to 53·6)	46·4 (41·6 to 51·2)	-4·87 (-8·37 to -1·97)	39·0 (35·4 to 42·9)	45500 (39900 to 54300)	39300 (32300 to 48500)	-13·5 (-27·8 to 4·39)	29600 (26000 to 35300)
	Under 5	15·4 (12·8 to 19·0)	14·7 (12·3 to 18·1)	-4·37 (-10·1 to 0·965)	12·3 (10·2 to 15·2)	672 (595 to 773)	326 (270 to 403)	-51·5 (-57·6 to -43·8)	437 (387 to 502)
	5-14 years	9·88 (7·05 to 13·3)	8·76 (6·45 to 12·1)	-11·3 (-17·5 to -3·46)	7·90 (5·64 to 10·6)	159 (142 to 184)	110 (93·8 to 133)	-31·2 (-38·0 to -22·0)	104 (92·3 to 120)
	15-49 years	39·5 (34·6 to 46·6)	37·4 (31·9 to 43·8)	-5·40 (-9·77 to 0·889)	31·6 (27·7 to 37·3)	10800 (9390 to 12600)	7560 (6060 to 9380)	-30·0 (-41·8 to -16·0)	7030 (6100 to 8210)
	50-69 years	74·5 (60·8 to 88·4)	67·5 (54·9 to 81·7)	-9·37 (-15·0 to -5·63)	59·6 (48·7 to 70·7)	15600 (13300 to 18600)	14500 (11600 to 17700)	-6·46 (-24·0 to 16·1)	10100 (8620 to 12100)
	70+ years	130 (105 to 156)	112 (90·2 to 137)	-13·6 (-17·9 to -8·88)	104 (84·0 to 125)	18300 (16100 to 22000)	16800 (14000 to 20800)	-8·20 (-22·3 to 8·79)	11900 (10500 to 14300)
Democratic People's Republic of Korea	All Ages	328 (298 to 360)	312 (275 to 343)	-4·81 (-9·45 to -0·180)	262 (238 to 288)	12200 (8870 to 16400)	11100 (8030 to 15100)	-8·92 (-19·9 to 3·92)	7910 (5770 to 10700)
	Under 5	127 (104 to 157)	102 (84·6 to 128)	-19·8 (-28·4 to -7·31)	102 (83·1 to 126)	132 (85·8 to 184)	76·0 (48·9 to 109)	-42·1 (-55·0 to -28·9)	85·5 (55·8 to 120)
	5-14 years	75·8 (50·7 to 111)	59·6 (41·1 to 87·1)	-21·0 (-33·8 to -6·54)	60·6 (40·5 to 88·9)	54·5 (40·8 to 83·1)	34·6 (24·7 to 48·8)	-36·1 (-46·4 to -22·2)	35·4 (26·5 to 54·0)
	15-49 years	273 (222 to 312)	250 (211 to 287)	-8·38 (-14·1 to -1·87)	218 (178 to 249)	2970 (2060 to 4480)	2570 (1780 to 3890)	-12·7 (-28·3 to 7·49)	1930 (1340 to 2910)
	50-69 years	576 (475 to 687)	531 (441 to 631)	-7·75 (-14·4 to -0·327)	461 (380 to 550)	4910 (3470 to 6760)	4830 (3280 to 6580)	-1·47 (-16·2 to 12·1)	3190 (2260 to 4390)

eTable 2. Progress towards the END-TB 2020 milestones in all-form tuberculosis incidence rate per 100,000 population and in deaths due to all-form tuberculosis by age for 204 countries and territories (2020), percent change from 2015 to 2020.

Location	Age group	2015 Rate	2020 Rate	Incidence Percent Change	Incidence Milestone	2015 Deaths	2020 Deaths	Mortality Percent Change	Mortality Milestone
	70+ years	826 (668 to 971)	741 (582 to 904)	-10·2 (-18·7 to 0·769)	660 (534 to 777)	4110 (2790 to 5490)	3570 (2570 to 4760)	-13·1 (-28·0 to -0·929)	2670 (1820 to 3570)
Taiwan (Province of China)	All Ages	53·7 (48·8 to 59·1)	44·4 (39·5 to 49·4)	-17·4 (-23·6 to -11·3)	43·0 (39·1 to 47·3)	803 (744 to 850)	682 (629 to 729)	-15·0 (-18·9 to -10·7)	522 (484 to 552)
	Under 5	10·2 (8·26 to 12·6)	15·0 (12·0 to 19·3)	46·8 (30·2 to 69·9)	8·20 (6·61 to 10·1)	2·60 (2·28 to 2·90)	1·51 (1·34 to 1·66)	-42·0 (-50·6 to -32·9)	1·69 (1·48 to 1·89)
	5-14 years	4·08 (2·81 to 5·86)	9·98 (6·75 to 14·8)	145 (99·4 to 198)	3·26 (2·25 to 4·69)	0·923 (0·831 to 1·03)	0·685 (0·599 to 0·766)	-25·7 (-32·3 to -19·2)	0·600 (0·540 to 0·672)
	15-49 years	29·0 (25·3 to 33·2)	30·0 (23·8 to 36·0)	3·38 (-8·68 to 17·7)	23·2 (20·3 to 26·5)	75·9 (67·9 to 84·2)	55·7 (49·8 to 62·4)	-26·6 (-29·9 to -22·0)	49·4 (44·2 to 54·7)
	50-69 years	65·4 (53·9 to 77·1)	53·0 (41·2 to 67·6)	-19·0 (-31·3 to -7·41)	52·3 (43·1 to 61·7)	155 (144 to 167)	144 (132 to 156)	-6·91 (-13·0 to 0·179)	101 (93·7 to 109)
	70+ years	247 (204 to 297)	131 (101 to 164)	-46·9 (-53·7 to -40·6)	197 (164 to 238)	569 (511 to 610)	480 (427 to 519)	-15·5 (-20·0 to -10·5)	370 (332 to 396)
Oceania	All Ages	114 (102 to 125)	112 (99·9 to 122)	-1·67 (-4·85 to 2·18)	91·3 (81·9 to 100)	3280 (2760 to 3950)	3410 (2820 to 4010)	3·98 (-6·45 to 17·3)	2130 (1790 to 2570)
	Under 5	51·0 (41·0 to 63·7)	45·8 (37·0 to 56·7)	-10·0 (-17·7 to -1·12)	40·8 (32·8 to 51·0)	264 (162 to 399)	233 (131 to 324)	-11·6 (-27·6 to 5·79)	172 (105 to 259)
	5-14 years	51·9 (36·2 to 71·4)	47·5 (33·9 to 65·5)	-8·38 (-17·4 to 2·16)	41·6 (29·0 to 57·1)	102 (74·3 to 132)	95·6 (71·3 to 127)	-5·90 (-20·3 to 13·6)	66·3 (48·3 to 85·6)
	15-49 years	126 (107 to 145)	125 (105 to 144)	-1·48 (-6·00 to 4·00)	101 (85·6 to 116)	1290 (1090 to 1570)	1290 (1020 to 1570)	0·0520 (-11·6 to 17·9)	838 (705 to 1020)
	50-69 years	225 (188 to 267)	225 (189 to 261)	0·0180 (-5·29 to 6·57)	180 (150 to 213)	1010 (817 to 1210)	1130 (904 to 1450)	11·2 (-2·44 to 26·8)	659 (531 to 785)
	70+ years	410 (347 to 455)	394 (335 to 445)	-3·77 (-9·11 to 3·68)	328 (278 to 364)	613 (506 to 750)	666 (536 to 827)	8·96 (-6·16 to 25·6)	398 (329 to 488)
American Samoa	All Ages	14·1 (12·3 to 15·5)	14·6 (12·8 to 16·1)	3·88 (-1·11 to 8·94)	11·3 (9·80 to 12·4)	1·07 (0·962 to 1·22)	1·17 (1·01 to 1·44)	9·39 (-6·33 to 30·5)	0·699 (0·625 to 0·791)
	Under 5	4·71 (3·65 to 6·24)	4·23 (3·35 to 5·42)	-9·81 (-19·2 to 2·13)	3·76 (2·92 to 4·99)	0·00986 (0·00808 to 0·0127)	0·00653 (0·00515 to 0·00852)	-33·7 (-45·3 to -18·5)	0·00641 (0·00525 to 0·00823)
	5-14 years	3·47 (2·39 to 5·17)	3·18 (2·18 to 4·43)	-8·00 (-18·5 to 5·17)	2·78 (1·91 to 4·14)	0·0148 (0·0116 to 0·0183)	0·00823 (0·00515 to 0·0118)	-44·4 (-61·7 to -26·1)	0·00963 (0·00755 to 0·0119)
	15-49 years	15·6 (12·3 to 18·4)	15·4 (12·5 to 18·1)	-0·873 (-6·70 to 5·36)	12·5 (9·80 to 14·7)	0·323 (0·260 to 0·387)	0·323 (0·262 to 0·410)	0·559 (-22·3 to 28·2)	0·210 (0·169 to 0·252)
	50-69 years	25·5 (20·9 to 30·7)	24·8 (19·8 to 30·0)	-2·73 (-8·95 to 3·83)	20·4 (16·7 to 24·6)	0·412 (0·350 to 0·478)	0·487 (0·415 to 0·596)	18·3 (2·85 to 40·4)	0·268 (0·228 to 0·310)
	70+ years	39·4 (30·8 to 48·4)	38·2 (29·7 to 46·7)	-3·02 (-13·2 to 6·31)	31·5 (24·6 to 38·7)	0·315 (0·267 to 0·371)	0·349 (0·279 to 0·444)	11·0 (-6·32 to 31·7)	0·205 (0·174 to 0·241)
Cook Islands	All Ages	14·9 (13·3 to 16·6)	14·4 (12·6 to 16·6)	-3·76 (-8·79 to 1·79)	11·9 (10·6 to 13·3)	0·304 (0·266 to 0·355)	0·282 (0·237 to 0·351)	-7·27 (-19·6 to 6·05)	0·198 (0·173 to 0·231)
	Under 5	4·00 (3·14 to 5·21)	3·42 (2·61 to 4·37)	-14·4 (-25·2 to -5·41)	3·20 (2·51 to 4·17)	0·00102 (0·000742 to 0·00141)	0·000976 (0·000664 to 0·00142)	-4·83 (-22·6 to 14·4)	0·000666 (0·000483 to 0·000919)

eTable 2. Progress towards the END-TB 2020 milestones in all-form tuberculosis incidence rate per 100,000 population and in deaths due to all-form tuberculosis by age for 204 countries and territories (2020), percent change from 2015 to 2020.

Location	Age group	2015 Rate	2020 Rate	Incidence Percent Change	Incidence Milestone	2015 Deaths	2020 Deaths	Mortality Percent Change	Mortality Milestone
	5-14 years	3·51 (2·38 to 4·99)	2·81 (1·93 to 4·10)	-19·7 (-30·7 to -5·99)	2·81 (1·91 to 4·00)	0·00112 (0·000862 to 0·00147)	0·000863 (0·000638 to 0·00113)	-22·7 (-34·8 to -8·87)	0·000727 (0·000560 to 0·000956)
	15-49 years	13·4 (10·8 to 16·3)	12·2 (9·73 to 15·1)	-8·77 (-14·5 to -1·62)	10·7 (8·67 to 13·1)	0·0550 (0·0438 to 0·0661)	0·0449 (0·0346 to 0·0578)	-18·5 (-31·5 to -4·24)	0·0358 (0·0285 to 0·0430)
	50-69 years	23·3 (17·7 to 29·1)	21·9 (16·4 to 27·1)	-6·10 (-13·6 to 2·09)	18·7 (14·2 to 23·2)	0·110 (0·0951 to 0·131)	0·108 (0·0869 to 0·143)	-1·69 (-16·2 to 17·5)	0·0714 (0·0618 to 0·0853)
	70+ years	36·7 (28·8 to 47·1)	34·1 (26·9 to 43·2)	-6·98 (-17·0 to 3·03)	29·3 (23·0 to 37·7)	0·137 (0·116 to 0·166)	0·127 (0·106 to 0·152)	-7·02 (-21·8 to 7·29)	0·0891 (0·0756 to 0·108)
Fiji	All Ages	36·4 (32·5 to 39·9)	34·5 (30·8 to 37·9)	-5·11 (-9·46 to 0·195)	29·1 (26·0 to 31·9)	52·6 (42·4 to 66·7)	48·2 (37·7 to 63·6)	-8·28 (-22·4 to 9·35)	34·2 (27·6 to 43·4)
	Under 5	14·1 (11·1 to 18·2)	12·3 (9·93 to 16·0)	-12·4 (-24·3 to 1·14)	11·3 (8·86 to 14·6)	1·19 (0·926 to 1·57)	0·896 (0·663 to 1·17)	-24·2 (-36·8 to -9·37)	0·770 (0·602 to 1·02)
	5-14 years	19·6 (13·0 to 28·4)	18·5 (12·6 to 26·7)	-5·13 (-18·4 to 11·6)	15·7 (10·4 to 22·7)	1·14 (0·885 to 1·48)	1·16 (0·885 to 1·53)	2·21 (-18·1 to 24·9)	0·740 (0·576 to 0·961)
	15-49 years	34·0 (27·8 to 39·8)	31·9 (26·0 to 36·6)	-6·28 (-12·1 to 0·533)	27·2 (22·2 to 31·8)	14·3 (11·7 to 18·7)	12·2 (9·48 to 16·0)	-14·3 (-28·0 to 3·98)	9·27 (7·60 to 12·2)
	50-69 years	66·4 (54·6 to 79·9)	62·3 (51·9 to 74·6)	-6·20 (-12·9 to -0·404)	53·2 (43·7 to 63·9)	22·1 (17·7 to 27·8)	20·3 (15·3 to 28·1)	-7·81 (-23·5 to 13·1)	14·4 (11·5 to 18·0)
	70+ years	106 (86·4 to 126)	97·8 (80·1 to 115)	-8·08 (-16·1 to 0·534)	85·2 (69·1 to 100)	13·9 (11·2 to 17·9)	13·6 (10·4 to 17·9)	-1·89 (-17·6 to 20·0)	9·04 (7·28 to 11·6)
Guam	All Ages	40·2 (35·3 to 45·4)	42·5 (37·6 to 47·4)	5·81 (-0·0783 to 11·9)	32·2 (28·2 to 36·3)	8·90 (7·92 to 10·1)	9·22 (7·82 to 10·6)	3·59 (-7·81 to 15·7)	5·79 (5·15 to 6·57)
	Under 5	11·2 (8·91 to 14·2)	10·1 (8·07 to 13·0)	-9·66 (-18·8 to 3·73)	8·95 (7·13 to 11·4)	0·0948 (0·0790 to 0·114)	0·0712 (0·0573 to 0·0865)	-24·9 (-34·3 to -14·9)	0·0617 (0·0514 to 0·0739)
	5-14 years	15·5 (10·7 to 21·4)	15·1 (10·1 to 20·9)	-1·89 (-14·0 to 14·8)	12·4 (8·59 to 17·1)	0·0846 (0·0704 to 0·100)	0·123 (0·102 to 0·147)	46·1 (23·4 to 71·7)	0·0550 (0·0458 to 0·0651)
	15-49 years	38·7 (32·6 to 47·0)	39·3 (31·6 to 47·1)	1·54 (-5·81 to 9·33)	31·0 (26·1 to 37·6)	2·42 (2·13 to 2·80)	2·50 (2·11 to 2·89)	3·38 (-8·34 to 14·4)	1·58 (1·38 to 1·82)
	50-69 years	63·4 (48·4 to 79·1)	64·6 (48·9 to 79·4)	2·02 (-6·89 to 12·0)	50·7 (38·7 to 63·2)	3·70 (3·17 to 4·36)	3·87 (3·20 to 4·65)	4·71 (-10·5 to 21·4)	2·41 (2·06 to 2·84)
	70+ years	89·6 (72·4 to 109)	89·4 (72·8 to 109)	-0·0382 (-7·21 to 8·54)	71·7 (58·0 to 87·3)	2·60 (2·18 to 2·99)	2·65 (2·15 to 3·18)	2·14 (-12·5 to 15·3)	1·69 (1·42 to 1·94)
Kiribati	All Ages	309 (279 to 341)	303 (276 to 334)	-2·12 (-6·18 to 2·39)	248 (223 to 273)	89·7 (67·5 to 115)	86·1 (64·8 to 118)	-4·12 (-16·9 to 7·92)	58·3 (43·8 to 75·0)
	Under 5	114 (87·2 to 145)	101 (79·1 to 128)	-11·2 (-21·6 to 1·61)	91·5 (69·7 to 116)	3·05 (2·09 to 4·02)	2·32 (1·51 to 3·25)	-23·8 (-42·6 to 0·906)	1·99 (1·36 to 2·61)
	5-14 years	164 (115 to 232)	150 (103 to 202)	-8·68 (-20·1 to 1·86)	131 (91·9 to 186)	2·30 (1·63 to 2·89)	2·13 (1·60 to 2·73)	-6·77 (-28·3 to 16·8)	1·49 (1·06 to 1·88)
	15-49 years	328 (282 to 374)	321 (273 to 368)	-1·99 (-8·54 to 4·35)	262 (226 to 299)	33·8 (23·8 to 44·0)	31·3 (21·6 to 43·6)	-7·49 (-21·1 to 8·99)	22·0 (15·4 to 28·6)
	50-69 years	601 (506 to 702)	600 (504 to 697)	-0·120 (-6·04 to 5·84)	481 (405 to 562)	32·9 (24·5 to 43·5)	33·6 (24·7 to 47·0)	2·23 (-11·1 to 18·1)	21·4 (15·9 to 28·3)
	70+ years	1080 (949 to 1200)	1000 (892 to 1100)	-7·25 (-13·4 to -1·35)	866 (759 to 958)	17·7 (13·2 to 21·9)	16·7 (13·2 to 21·1)	-5·21 (-16·4 to 8·31)	11·5 (8·56 to 14·3)

eTable 2. Progress towards the END-TB 2020 milestones in all-form tuberculosis incidence rate per 100,000 population and in deaths due to all-form tuberculosis by age for 204 countries and territories (2020), percent change from 2015 to 2020.

Location	Age group	2015 Rate	2020 Rate	Incidence Percent Change	Incidence Milestone	2015 Deaths	2020 Deaths	Mortality Percent Change	Mortality Milestone
Marshall Islands	All Ages	357 (316 to 402)	349 (310 to 384)	-2·34 (-6·99 to 1·82)	286 (253 to 321)	35·0 (21·9 to 46·5)	33·7 (20·7 to 45·8)	-4·07 (-13·5 to 8·09)	22·8 (14·2 to 30·2)
	Under 5	120 (93·2 to 150)	105 (82·6 to 132)	-11·8 (-22·5 to 1·19)	95·8 (74·6 to 120)	0·664 (0·410 to 0·925)	0·507 (0·312 to 0·702)	-23·1 (-39·8 to -3·39)	0·431 (0·266 to 0·601)
	5-14 years	166 (113 to 228)	149 (102 to 207)	-9·84 (-21·4 to 2·47)	132 (90·6 to 182)	0·728 (0·483 to 0·958)	0·580 (0·366 to 0·781)	-19·9 (-35·7 to -1·19)	0·473 (0·314 to 0·623)
	15-49 years	391 (324 to 452)	371 (310 to 421)	-5·09 (-11·3 to 0·251)	313 (259 to 362)	14·7 (9·57 to 19·4)	13·3 (8·34 to 18·4)	-9·65 (-20·1 to 2·04)	9·59 (6·22 to 12·6)
	50-69 years	729 (595 to 866)	688 (556 to 823)	-5·60 (-11·2 to -0·279)	583 (476 to 693)	14·0 (8·43 to 18·6)	13·6 (7·76 to 19·2)	-3·02 (-15·9 to 12·5)	9·08 (5·48 to 12·1)
	70+ years	1120 (926 to 1270)	1030 (867 to 1170)	-8·27 (-16·3 to -0·453)	895 (741 to 1010)	4·94 (3·09 to 6·59)	5·69 (3·55 to 8·00)	15·5 (-3·53 to 34·5)	3·21 (2·01 to 4·29)
Micronesia (Federated States of)	All Ages	80·4 (72·1 to 88·1)	77·8 (68·9 to 84·9)	-3·16 (-8·33 to 1·24)	64·3 (57·7 to 70·4)	12·9 (9·27 to 16·6)	12·8 (9·02 to 17·1)	-0·550 (-11·4 to 13·7)	8·36 (6·02 to 10·8)
	Under 5	25·4 (19·6 to 31·9)	22·4 (17·6 to 28·3)	-11·4 (-22·8 to -0·280)	20·3 (15·7 to 25·5)	0·208 (0·145 to 0·278)	0·164 (0·117 to 0·223)	-20·9 (-36·7 to -4·19)	0·135 (0·0940 to 0·181)
	5-14 years	34·2 (23·3 to 49·0)	29·6 (19·8 to 41·9)	-13·4 (-22·0 to -4·09)	27·4 (18·6 to 39·2)	0·233 (0·168 to 0·304)	0·174 (0·133 to 0·233)	-24·7 (-39·7 to -7·08)	0·152 (0·109 to 0·198)
	15-49 years	85·0 (69·5 to 98·3)	79·9 (65·6 to 91·7)	-5·98 (-10·8 to 1·05)	68·0 (55·6 to 78·6)	4·69 (3·24 to 6·16)	4·40 (3·09 to 5·87)	-6·08 (-20·8 to 11·3)	3·05 (2·11 to 4·01)
	50-69 years	157 (126 to 191)	150 (127 to 180)	-4·62 (-12·1 to 3·67)	126 (101 to 153)	5·28 (3·65 to 7·37)	5·61 (3·68 to 7·91)	6·47 (-8·99 to 26·6)	3·43 (2·37 to 4·79)
	70+ years	251 (211 to 286)	225 (188 to 260)	-10·1 (-18·2 to -1·31)	201 (169 to 228)	2·44 (1·84 to 3·14)	2·43 (1·82 to 3·20)	-0·163 (-17·2 to 17·8)	1·59 (1·20 to 2·04)
Nauru	All Ages	159 (140 to 179)	145 (127 to 161)	-8·41 (-13·0 to -1·46)	127 (112 to 143)	3·38 (2·14 to 4·86)	2·69 (1·91 to 3·65)	-20·0 (-30·3 to -6·59)	2·20 (1·39 to 3·16)
	Under 5	58·3 (44·8 to 72·0)	49·1 (38·0 to 63·2)	-15·6 (-28·2 to -3·40)	46·7 (35·9 to 57·6)	0·104 (0·0632 to 0·142)	0·0707 (0·0458 to 0·101)	-31·7 (-42·8 to -15·3)	0·0677 (0·0411 to 0·0920)
	5-14 years	75·9 (53·6 to 106)	65·2 (45·4 to 93·2)	-13·9 (-25·8 to -0·419)	60·7 (42·9 to 84·8)	0·0808 (0·0542 to 0·106)	0·0594 (0·0420 to 0·0776)	-25·8 (-37·6 to -0·448)	0·0525 (0·0352 to 0·0690)
	15-49 years	179 (150 to 207)	165 (136 to 191)	-8·16 (-14·0 to -0·799)	143 (120 to 166)	1·37 (0·882 to 1·88)	1·08 (0·772 to 1·58)	-20·6 (-31·3 to -5·82)	0·889 (0·573 to 1·22)
	50-69 years	337 (276 to 408)	305 (256 to 366)	-9·29 (-15·6 to -0·912)	270 (221 to 326)	1·21 (0·688 to 1·72)	0·947 (0·571 to 1·30)	-20·7 (-35·1 to -8·07)	0·784 (0·447 to 1·12)
	70+ years	485 (416 to 555)	423 (355 to 499)	-12·6 (-20·2 to -3·98)	388 (333 to 444)	0·625 (0·370 to 1·20)	0·531 (0·356 to 0·962)	-13·9 (-29·5 to 5·68)	0·407 (0·240 to 0·779)
Niue	All Ages	37·0 (32·9 to 41·2)	35·9 (32·4 to 39·8)	-3·01 (-7·35 to 1·73)	29·6 (26·4 to 33·0)	0·119 (0·0923 to 0·149)	0·109 (0·0827 to 0·140)	-8·32 (-18·2 to 4·95)	0·0777 (0·0600 to 0·0968)
	Under 5	13·0 (10·1 to 16·5)	12·3 (9·82 to 15·8)	-5·24 (-18·4 to 6·21)	10·4 (8·08 to 13·2)	0·00150 (0·00105 to 0·00201)	0·00134 (0·000889 to 0·00176)	-10·6 (-26·9 to 10·1)	0·000974 (0·000685 to 0·00131)
	5-14 years	14·9 (10·3 to 20·3)	13·9 (9·45 to 19·4)	-6·27 (-18·0 to 9·91)	11·9 (8·25 to 16·2)	0·00134 (0·000957 to 0·00182)	0·00112 (0·000803 to 0·00156)	-15·8 (-29·4 to 1·01)	0·000870 (0·000622 to 0·00118)

eTable 2. Progress towards the END-TB 2020 milestones in all-form tuberculosis incidence rate per 100,000 population and in deaths due to all-form tuberculosis by age for 204 countries and territories (2020), percent change from 2015 to 2020.

Location	Age group	2015 Rate	2020 Rate	Incidence Percent Change	Incidence Milestone	2015 Deaths	2020 Deaths	Mortality Percent Change	Mortality Milestone
	15-49 years	30·0 (25·1 to 35·3)	28·5 (23·4 to 32·9)	-5·04 (-9·90 to 0·487)	24·0 (20·0 to 28·2)	0·0199 (0·0133 to 0·0280)	0·0178 (0·0120 to 0·0248)	-10·1 (-24·5 to 6·99)	0·0129 (0·00864 to 0·0182)
	50-69 years	59·2 (47·5 to 72·1)	57·8 (46·5 to 71·1)	-2·28 (-8·16 to 5·30)	47·4 (38·0 to 57·7)	0·0435 (0·0308 to 0·0592)	0·0441 (0·0305 to 0·0610)	1·77 (-15·4 to 16·8)	0·0282 (0·0200 to 0·0385)
	70+ years	93·2 (74·4 to 112)	88·6 (72·1 to 107)	-4·80 (-14·3 to 3·39)	74·6 (59·5 to 90·0)	0·0533 (0·0409 to 0·0646)	0·0451 (0·0353 to 0·0558)	-15·2 (-26·4 to -1·50)	0·0346 (0·0266 to 0·0420)
Northern Mariana Islands	All Ages	94·5 (82·1 to 106)	94·6 (80·9 to 108)	0·109 (-4·21 to 5·91)	75·6 (65·6 to 84·4)	4·57 (3·61 to 6·02)	4·40 (3·16 to 5·70)	-3·37 (-19·7 to 16·7)	2·97 (2·35 to 3·91)
	Under 5	28·2 (22·1 to 36·7)	25·4 (20·3 to 32·9)	-9·79 (-18·6 to 2·77)	22·6 (17·7 to 29·4)	0·0311 (0·0235 to 0·0411)	0·0209 (0·0156 to 0·0274)	-32·8 (-42·4 to -20·4)	0·0202 (0·0153 to 0·0267)
	5-14 years	30·7 (21·2 to 43·1)	27·3 (18·5 to 38·8)	-10·7 (-21·8 to 1·86)	24·5 (17·0 to 34·5)	0·0404 (0·0308 to 0·0496)	0·0228 (0·00686 to 0·0354)	-43·3 (-83·1 to -11·9)	0·0262 (0·0200 to 0·0322)
	15-49 years	87·6 (73·2 to 105)	82·7 (68·3 to 101)	-5·50 (-10·7 to 0·448)	70·1 (58·6 to 84·1)	1·16 (0·860 to 1·59)	0·899 (0·625 to 1·24)	-22·5 (-37·0 to -3·42)	0·756 (0·559 to 1·04)
	50-69 years	167 (126 to 205)	162 (122 to 203)	-2·46 (-9·19 to 4·20)	133 (101 to 164)	2·25 (1·74 to 2·96)	2·25 (1·60 to 2·90)	0·119 (-17·4 to 24·3)	1·47 (1·13 to 1·93)
	70+ years	265 (207 to 327)	242 (194 to 303)	-8·37 (-16·0 to 1·84)	212 (166 to 262)	1·08 (0·868 to 1·39)	1·21 (0·890 to 1·62)	12·6 (-9·23 to 44·6)	0·700 (0·564 to 0·906)
Palau	All Ages	45·0 (39·5 to 50·3)	47·3 (42·0 to 52·9)	5·03 (-0·955 to 10·6)	36·0 (31·6 to 40·2)	1·51 (1·23 to 1·82)	1·52 (1·20 to 1·94)	0·375 (-12·4 to 19·5)	0·983 (0·802 to 1·18)
	Under 5	12·4 (9·89 to 16·0)	11·7 (9·39 to 15·0)	-4·90 (-14·7 to 7·78)	9·89 (7·91 to 12·8)	0·0128 (0·00980 to 0·0172)	0·00956 (0·00739 to 0·0122)	-24·9 (-36·6 to -8·62)	0·00832 (0·00637 to 0·0112)
	5-14 years	12·6 (8·63 to 18·2)	11·7 (8·14 to 16·5)	-6·70 (-18·0 to 8·51)	10·1 (6·91 to 14·6)	0·00748 (0·00591 to 0·00902)	0·00645 (0·00499 to 0·00802)	-13·4 (-29·1 to 3·96)	0·00486 (0·00384 to 0·00587)
	15-49 years	44·5 (37·2 to 52·2)	45·8 (38·3 to 54·8)	3·01 (-3·93 to 10·9)	35·6 (29·7 to 41·8)	0·518 (0·411 to 0·645)	0·454 (0·364 to 0·587)	-12·1 (-26·5 to 7·43)	0·337 (0·267 to 0·419)
	50-69 years	66·1 (52·5 to 82·6)	65·3 (52·3 to 79·4)	-1·07 (-8·68 to 6·84)	52·9 (42·0 to 66·1)	0·626 (0·472 to 0·788)	0·655 (0·483 to 0·939)	4·76 (-15·0 to 27·5)	0·407 (0·307 to 0·512)
	70+ years	108 (86·8 to 134)	101 (79·4 to 127)	-6·19 (-16·7 to 3·12)	86·3 (69·4 to 107)	0·348 (0·284 to 0·437)	0·391 (0·309 to 0·500)	12·9 (-2·51 to 34·9)	0·226 (0·185 to 0·284)
Papua New Guinea	All Ages	129 (115 to 142)	125 (111 to 136)	-2·74 (-6·26 to 1·85)	103 (92·0 to 113)	2770 (2300 to 3380)	2910 (2360 to 3470)	5·19 (-6·63 to 19·8)	1800 (1500 to 2200)
	Under 5	57·5 (46·1 to 71·6)	50·8 (41·0 to 63·0)	-11·5 (-19·8 to -1·83)	46·0 (36·8 to 57·3)	241 (146 to 368)	214 (118 to 302)	-10·9 (-27·5 to 7·44)	157 (94·8 to 239)
	5-14 years	57·7 (40·1 to 79·4)	52·0 (37·2 to 72·5)	-9·49 (-19·6 to 2·10)	46·1 (32·1 to 63·5)	88·8 (62·4 to 116)	83·8 (61·3 to 113)	-5·22 (-20·4 to 16·4)	57·7 (40·6 to 75·4)
	15-49 years	143 (120 to 164)	139 (118 to 161)	-2·44 (-7·59 to 3·85)	114 (96·3 to 131)	1100 (911 to 1360)	1110 (864 to 1380)	1·00 (-11·5 to 20·3)	714 (592 to 884)
	50-69 years	270 (226 to 321)	266 (224 to 307)	-1·33 (-7·68 to 6·12)	216 (181 to 257)	835 (655 to 1010)	944 (739 to 1250)	13·3 (-2·48 to 30·5)	543 (426 to 658)
	70+ years	533 (451 to 594)	508 (430 to 576)	-4·65 (-10·9 to 4·21)	426 (361 to 475)	505 (402 to 629)	558 (433 to 715)	11·0 (-5·81 to 29·8)	328 (261 to 409)

eTable 2. Progress towards the END-TB 2020 milestones in all-form tuberculosis incidence rate per 100,000 population and in deaths due to all-form tuberculosis by age for 204 countries and territories (2020), percent change from 2015 to 2020.

Location	Age group	2015 Rate	2020 Rate	Incidence Percent Change	Incidence Milestone	2015 Deaths	2020 Deaths	Mortality Percent Change	Mortality Milestone
Samoa	All Ages	42·8 (38·3 to 47·1)	41·6 (37·1 to 45·8)	-2·97 (-7·89 to 3·73)	34·3 (30·7 to 37·7)	15·0 (11·6 to 19·5)	14·2 (10·8 to 18·6)	-4·69 (-18·3 to 8·98)	9·75 (7·52 to 12·7)
	Under 5	14·9 (11·7 to 19·4)	13·7 (10·9 to 17·5)	-8·27 (-22·2 to 5·85)	12·0 (9·39 to 15·5)	0·330 (0·226 to 0·467)	0·305 (0·202 to 0·445)	-6·36 (-30·5 to 26·8)	0·214 (0·147 to 0·303)
	5-14 years	17·4 (12·0 to 24·4)	15·4 (10·9 to 21·3)	-11·1 (-23·5 to 3·52)	13·9 (9·59 to 19·5)	0·245 (0·178 to 0·351)	0·221 (0·168 to 0·290)	-8·81 (-27·9 to 16·3)	0·159 (0·116 to 0·228)
	15-49 years	44·4 (36·1 to 52·3)	43·1 (35·4 to 49·8)	-2·88 (-8·80 to 2·65)	35·6 (28·9 to 41·9)	4·10 (2·92 to 5·65)	3·85 (2·69 to 4·92)	-5·34 (-21·6 to 14·5)	2·67 (1·90 to 3·67)
	50-69 years	93·5 (76·9 to 115)	91·6 (75·8 to 111)	-1·89 (-8·75 to 6·23)	74·8 (61·5 to 91·8)	5·47 (3·99 to 7·19)	5·41 (3·97 to 7·46)	-0·640 (-17·9 to 17·6)	3·56 (2·59 to 4·67)
	70+ years	146 (119 to 174)	140 (114 to 167)	-4·24 (-11·8 to 5·51)	117 (95·1 to 139)	4·85 (3·70 to 6·72)	4·45 (3·22 to 6·21)	-7·98 (-20·0 to 10·9)	3·15 (2·41 to 4·37)
Solomon Islands	All Ages	64·6 (58·9 to 71·8)	64·6 (58·2 to 71·1)	-0·112 (-4·97 to 3·88)	51·7 (47·1 to 57·4)	83·6 (62·2 to 110)	85·5 (66·2 to 111)	2·61 (-10·5 to 21·2)	54·3 (40·4 to 71·4)
	Under 5	27·0 (21·6 to 34·1)	25·2 (19·9 to 31·7)	-6·65 (-20·2 to 5·59)	21·6 (17·3 to 27·3)	2·97 (2·15 to 3·95)	2·42 (1·67 to 3·32)	-17·6 (-37·0 to 4·94)	1·93 (1·40 to 2·57)
	5-14 years	28·3 (18·7 to 39·9)	27·2 (19·3 to 38·2)	-3·28 (-16·6 to 14·4)	22·6 (14·9 to 31·9)	2·32 (1·60 to 3·31)	1·99 (1·31 to 3·06)	-13·5 (-32·5 to 10·9)	1·51 (1·04 to 2·15)
	15-49 years	72·6 (62·2 to 84·6)	72·0 (60·5 to 82·9)	-0·728 (-7·18 to 5·14)	58·0 (49·7 to 67·7)	35·3 (25·0 to 47·4)	36·1 (26·7 to 45·1)	2·56 (-10·7 to 21·8)	22·9 (16·2 to 30·8)
	50-69 years	146 (119 to 175)	142 (116 to 168)	-3·13 (-10·2 to 3·61)	117 (95·3 to 140)	25·0 (17·5 to 34·2)	27·1 (20·2 to 37·3)	9·13 (-8·99 to 34·9)	16·2 (11·4 to 22·2)
	70+ years	255 (215 to 289)	250 (215 to 280)	-1·66 (-9·14 to 5·82)	204 (172 to 231)	18·0 (13·4 to 22·3)	17·9 (14·2 to 22·3)	-0·178 (-13·9 to 16·9)	11·7 (8·74 to 14·5)
Tokelau	All Ages	39·2 (34·9 to 43·1)	36·4 (32·8 to 40·3)	-6·91 (-10·7 to -2·45)	31·3 (27·9 to 34·5)	0·102 (0·0726 to 0·143)	0·0865 (0·0655 to 0·115)	-14·7 (-28·3 to 2·20)	0·0663 (0·0472 to 0·0928)
	Under 5	10·3 (8·27 to 13·5)	9·01 (6·99 to 11·4)	-12·1 (-23·3 to 0·513)	8·23 (6·61 to 10·8)	0·000688 (0·000476 to 0·000987)	0·000590 (0·000426 to 0·000862)	-13·5 (-28·2 to 8·26)	0·000447 (0·000309 to 0·000642)
	5-14 years	12·5 (8·53 to 18·1)	10·6 (7·54 to 15·1)	-14·9 (-24·9 to -2·56)	10·0 (6·83 to 14·5)	0·000896 (0·000613 to 0·00118)	0·000756 (0·000560 to 0·000998)	-14·9 (-31·1 to 5·47)	0·000583 (0·000399 to 0·000766)
	15-49 years	35·0 (29·2 to 40·3)	32·7 (26·7 to 39·0)	-6·64 (-13·9 to 1·15)	28·0 (23·3 to 32·2)	0·0194 (0·0134 to 0·0293)	0·0175 (0·0131 to 0·0241)	-9·02 (-22·6 to 7·58)	0·0126 (0·00869 to 0·0190)
	50-69 years	69·9 (55·1 to 85·0)	66·2 (54·7 to 80·3)	-5·08 (-11·8 to 2·08)	55·9 (44·1 to 68·0)	0·0337 (0·0233 to 0·0484)	0·0305 (0·0222 to 0·0428)	-8·70 (-27·6 to 12·2)	0·0219 (0·0152 to 0·0315)
	70+ years	112 (90·8 to 135)	104 (84·7 to 128)	-7·02 (-15·5 to 1·41)	89·3 (72·7 to 108)	0·0475 (0·0336 to 0·0671)	0·0372 (0·0279 to 0·0489)	-21·1 (-35·4 to -6·53)	0·0309 (0·0218 to 0·0436)
Tonga	All Ages	32·6 (28·8 to 35·8)	31·2 (27·8 to 34·6)	-4·17 (-8·39 to 0·599)	26·1 (23·1 to 28·6)	5·79 (4·52 to 7·52)	5·12 (3·95 to 6·56)	-11·3 (-23·6 to 1·10)	3·76 (2·94 to 4·89)
	Under 5	11·5 (9·12 to 14·7)	10·0 (7·87 to 12·8)	-12·4 (-23·7 to 0·268)	9·17 (7·29 to 11·7)	0·124 (0·0940 to 0·154)	0·0883 (0·0636 to 0·112)	-28·2 (-41·7 to -10·3)	0·0803 (0·0611 to 0·100)
	5-14 years	13·0 (9·01 to 18·0)	11·6 (7·94 to 16·5)	-10·5 (-22·5 to 3·38)	10·4 (7·21 to 14·4)	0·0867 (0·0649 to 0·114)	0·0708 (0·0477 to 0·0951)	-18·2 (-33·8 to 0·839)	0·0564 (0·0422 to 0·0742)
	15-49 years	32·7 (26·6 to 38·6)	31·0 (24·8 to 36·1)	-5·12 (-11·7 to 2·41)	26·1 (21·3 to 30·9)	1·31 (0·951 to 1·74)	1·11 (0·769 to 1·48)	-14·7 (-30·0 to 1·52)	0·851 (0·618 to 1·13)

eTable 2. Progress towards the END-TB 2020 milestones in all-form tuberculosis incidence rate per 100,000 population and in deaths due to all-form tuberculosis by age for 204 countries and territories (2020), percent change from 2015 to 2020.

Location	Age group	2015 Rate	2020 Rate	Incidence Percent Change	Incidence Milestone	2015 Deaths	2020 Deaths	Mortality Percent Change	Mortality Milestone
	50-69 years	70·4 (56·9 to 84·6)	66·4 (53·4 to 79·4)	-5·59 (-10·9 to 0·457)	56·3 (45·5 to 67·7)	2·02 (1·46 to 2·93)	1·87 (1·34 to 2·56)	-6·92 (-23·3 to 12·0)	1·31 (0·947 to 1·90)
	70+ years	111 (89·4 to 135)	106 (87·5 to 126)	-3·95 (-11·2 to 3·55)	88·8 (71·5 to 108)	2·25 (1·80 to 3·05)	1·98 (1·56 to 2·62)	-11·7 (-24·3 to 3·33)	1·46 (1·17 to 1·98)
Tuvalu	All Ages	229 (205 to 252)	217 (195 to 239)	-5·43 (-9·66 to -1·35)	183 (164 to 202)	5·08 (3·31 to 6·25)	4·64 (3·21 to 5·76)	-8·48 (-22·6 to 5·20)	3·30 (2·15 to 4·06)
	Under 5	73·5 (57·6 to 94·1)	64·1 (50·6 to 82·5)	-12·4 (-22·9 to -0·0957)	58·8 (46·1 to 75·3)	0·0857 (0·0605 to 0·115)	0·0656 (0·0479 to 0·0863)	-23·2 (-37·5 to -4·88)	0·0557 (0·0393 to 0·0746)
	5-14 years	99·6 (65·5 to 143)	86·8 (56·8 to 123)	-12·6 (-24·6 to -1·63)	79·7 (52·4 to 115)	0·0840 (0·0620 to 0·113)	0·0641 (0·0457 to 0·0832)	-23·3 (-35·3 to -8·13)	0·0546 (0·0403 to 0·0732)
	15-49 years	220 (179 to 254)	207 (169 to 234)	-5·92 (-12·1 to 0·451)	176 (143 to 203)	1·34 (0·849 to 1·73)	1·22 (0·825 to 1·60)	-8·36 (-25·9 to 9·15)	0·871 (0·552 to 1·12)
	50-69 years	436 (361 to 537)	417 (349 to 503)	-4·22 (-9·22 to 2·37)	349 (288 to 429)	2·04 (1·35 to 2·61)	1·87 (1·25 to 2·46)	-8·02 (-23·1 to 5·42)	1·32 (0·876 to 1·70)
	70+ years	671 (566 to 777)	617 (510 to 730)	-8·06 (-16·3 to 0·575)	537 (453 to 622)	1·54 (1·02 to 1·98)	1·42 (0·964 to 1·79)	-7·06 (-23·6 to 9·77)	0·998 (0·665 to 1·29)
Vanuatu	All Ages	53·5 (48·3 to 59·9)	53·1 (47·5 to 59·6)	-0·637 (-5·77 to 3·95)	42·8 (38·6 to 47·9)	32·5 (18·9 to 47·0)	34·8 (21·2 to 48·8)	7·49 (-5·00 to 19·3)	21·1 (12·3 to 30·5)
	Under 5	21·1 (17·0 to 27·2)	19·8 (15·9 to 25·0)	-5·82 (-17·2 to 3·91)	16·9 (13·6 to 21·8)	1·05 (0·628 to 1·58)	1·05 (0·615 to 1·54)	1·24 (-16·8 to 19·5)	0·683 (0·408 to 1·03)
	5-14 years	24·5 (16·2 to 34·7)	23·9 (16·5 to 33·0)	-1·96 (-14·0 to 10·2)	19·6 (13·0 to 27·7)	0·825 (0·519 to 1·13)	0·863 (0·590 to 1·21)	5·81 (-15·0 to 29·9)	0·536 (0·337 to 0·737)
	15-49 years	59·0 (49·6 to 68·5)	57·9 (48·3 to 65·8)	-1·82 (-9·44 to 4·52)	47·2 (39·6 to 54·8)	12·1 (6·93 to 17·5)	12·8 (7·54 to 18·5)	6·45 (-8·89 to 23·0)	7·88 (4·51 to 11·4)
	50-69 years	119 (97·5 to 141)	115 (94·4 to 136)	-3·17 (-8·58 to 2·33)	95·4 (78·0 to 113)	11·6 (6·01 to 17·6)	12·6 (6·65 to 19·3)	9·36 (-8·98 to 28·8)	7·52 (3·90 to 11·4)
	70+ years	187 (158 to 212)	174 (149 to 197)	-6·68 (-13·2 to 2·83)	149 (127 to 169)	6·95 (4·46 to 9·54)	7·48 (5·08 to 10·4)	8·01 (-5·12 to 28·2)	4·52 (2·90 to 6·20)
Southeast Asia	All Ages	192 (174 to 211)	191 (174 to 209)	-0·889 (-2·77 to 0·554)	154 (139 to 169)	204000 (186000 to 221000)	186000 (167000 to 212000)	-8·67 (-18·6 to 4·27)	132000 (121000 to 144000)
	Under 5	68·2 (56·7 to 84·6)	62·4 (52·1 to 77·0)	-8·49 (-11·3 to -6·04)	54·5 (45·3 to 67·7)	4950 (4040 to 5810)	3230 (2560 to 3970)	-34·8 (-43·1 to -25·6)	3220 (2630 to 3780)
	5-14 years	56·4 (39·8 to 77·3)	55·5 (39·6 to 76·2)	-1·53 (-4·84 to 1·90)	45·1 (31·8 to 61·8)	2490 (2220 to 2740)	1710 (1510 to 1920)	-31·5 (-38·2 to -25·2)	1620 (1440 to 1780)
	15-49 years	172 (146 to 197)	163 (137 to 190)	-5·57 (-7·46 to -3·98)	138 (116 to 158)	62600 (57700 to 68900)	50600 (45300 to 58300)	-19·1 (-29·2 to -7·25)	40700 (37500 to 44800)
	50-69 years	384 (314 to 464)	369 (304 to 444)	-3·78 (-6·17 to -1·59)	307 (251 to 371)	70700 (64100 to 77300)	70100 (61500 to 81000)	-0·624 (-12·1 to 16·0)	45900 (41700 to 50300)
	70+ years	625 (536 to 723)	591 (507 to 689)	-5·55 (-8·13 to -2·75)	500 (429 to 578)	62900 (55000 to 69400)	60200 (53700 to 69300)	-4·28 (-12·1 to 9·57)	40900 (35800 to 45100)
Cambodia	All Ages	298 (269 to 329)	263 (234 to 291)	-11·7 (-15·7 to -7·41)	239 (215 to 263)	7570 (5300 to 9670)	6990 (4810 to 9600)	-7·60 (-17·9 to 7·50)	4920 (3450 to 6290)
	Under 5	99·3 (81·3 to 122)	79·7 (64·7 to 97·7)	-19·6 (-30·5 to -9·23)	79·4 (65·1 to 98·0)	229 (128 to 347)	146 (85·2 to 229)	-35·7 (-49·0 to -20·8)	149 (83·3 to 225)

eTable 2. Progress towards the END-TB 2020 milestones in all-form tuberculosis incidence rate per 100,000 population and in deaths due to all-form tuberculosis by age for 204 countries and territories (2020), percent change from 2015 to 2020.

Location	Age group	2015 Rate	2020 Rate	Incidence Percent Change	Incidence Milestone	2015 Deaths	2020 Deaths	Mortality Percent Change	Mortality Milestone
	5-14 years	91·0 (62·8 to 125)	71·2 (48·9 to 97·4)	-21·5 (-32·4 to -11·5)	72·8 (50·3 to 100)	141 (94·2 to 194)	92·1 (62·2 to 133)	-34·6 (-43·9 to -19·1)	91·4 (61·2 to 126)
	15-49 years	289 (241 to 331)	247 (206 to 289)	-14·4 (-20·7 to -8·58)	231 (193 to 265)	2390 (1750 to 3180)	1990 (1420 to 2800)	-16·6 (-29·2 to 1·28)	1550 (1130 to 2070)
	50-69 years	701 (565 to 847)	613 (497 to 745)	-12·4 (-19·0 to -6·13)	561 (452 to 677)	2890 (1980 to 3990)	2840 (1850 to 3870)	-1·27 (-14·5 to 13·1)	1880 (1290 to 2590)
	70+ years	993 (794 to 1200)	839 (667 to 1040)	-15·5 (-23·4 to -8·30)	794 (635 to 957)	1930 (1220 to 2510)	1920 (1330 to 2610)	-0·329 (-15·0 to 19·1)	1260 (790 to 1630)
Indonesia	All Ages	190 (171 to 208)	178 (162 to 196)	-6·07 (-7·73 to -4·22)	152 (137 to 167)	102000 (87000 to 117000)	94500 (78300 to 110000)	-6·72 (-23·9 to 18·9)	66200 (56600 to 75800)
	Under 5	73·7 (60·7 to 90·7)	61·6 (51·2 to 75·8)	-16·3 (-19·6 to -13·0)	58·9 (48·6 to 72·6)	2470 (1950 to 3060)	1630 (1260 to 2190)	-33·7 (-47·4 to -19·0)	1600 (1270 to 1990)
	5-14 years	56·3 (38·7 to 78·8)	45·0 (31·6 to 63·2)	-20·1 (-23·1 to -16·7)	45·1 (30·9 to 63·0)	1120 (935 to 1310)	762 (639 to 937)	-31·9 (-43·2 to -19·3)	730 (608 to 849)
	15-49 years	151 (125 to 172)	131 (109 to 152)	-12·9 (-15·4 to -10·8)	121 (100 to 137)	30400 (26600 to 35800)	25400 (21000 to 30800)	-16·1 (-33·9 to 8·21)	19800 (17300 to 23300)
	50-69 years	438 (358 to 532)	400 (328 to 483)	-8·50 (-12·0 to -5·43)	350 (287 to 425)	37200 (31200 to 43400)	37400 (30100 to 44200)	1·06 (-18·6 to 32·0)	24200 (20300 to 28200)
	70+ years	806 (668 to 941)	749 (622 to 875)	-7·06 (-10·2 to -3·75)	644 (534 to 753)	30600 (25600 to 34800)	29400 (24500 to 35300)	-3·50 (-18·3 to 23·8)	19900 (16600 to 22600)
Lao People's Democratic Republic	All Ages	128 (113 to 148)	112 (98·0 to 130)	-12·5 (-17·9 to -7·85)	102 (90·6 to 119)	2220 (1440 to 2970)	1930 (1280 to 2630)	-13·1 (-24·7 to -0·0307)	1440 (935 to 1930)
	Under 5	51·4 (40·9 to 63·1)	41·4 (33·1 to 51·3)	-19·3 (-29·2 to -0·567)	41·1 (32·7 to 50·5)	122 (80·5 to 167)	82·8 (56·7 to 122)	-31·5 (-49·6 to -8·58)	79·2 (52·3 to 109)
	5-14 years	41·3 (27·7 to 59·9)	33·3 (23·2 to 46·5)	-19·0 (-30·2 to -7·15)	33·0 (22·1 to 48·0)	47·9 (32·4 to 69·4)	32·8 (21·7 to 46·5)	-31·2 (-46·3 to -15·6)	31·1 (21·1 to 45·1)
	15-49 years	119 (95·8 to 147)	99·7 (81·7 to 126)	-16·0 (-22·3 to -9·58)	95·0 (76·6 to 118)	801 (537 to 1090)	647 (414 to 937)	-19·0 (-31·5 to -5·73)	520 (349 to 707)
	50-69 years	344 (256 to 440)	295 (221 to 371)	-14·0 (-20·3 to -7·52)	275 (205 to 352)	737 (470 to 1020)	687 (423 to 960)	-6·55 (-25·0 to 13·2)	479 (305 to 664)
	70+ years	529 (422 to 662)	450 (364 to 564)	-14·8 (-20·8 to -6·07)	423 (338 to 530)	514 (305 to 690)	478 (300 to 657)	-6·66 (-24·3 to 9·84)	334 (198 to 448)
Malaysia	All Ages	65·4 (57·5 to 74·6)	65·2 (57·6 to 74·6)	-0·223 (-5·31 to 4·65)	52·3 (46·0 to 59·7)	2260 (2000 to 2570)	2150 (1850 to 2570)	-5·13 (-13·6 to 5·14)	1470 (1300 to 1670)
	Under 5	13·1 (10·5 to 17·0)	11·5 (9·01 to 13·9)	-11·9 (-22·4 to -1·03)	10·5 (8·44 to 13·6)	13·5 (10·3 to 16·2)	9·07 (7·39 to 11·1)	-32·7 (-40·4 to -22·9)	8·78 (6·71 to 10·5)
	5-14 years	13·7 (9·18 to 19·6)	12·4 (8·27 to 17·5)	-9·92 (-21·9 to 3·02)	11·0 (7·34 to 15·7)	18·2 (14·1 to 22·8)	10·5 (7·56 to 13·3)	-42·3 (-56·8 to -29·4)	11·9 (9·19 to 14·9)
	15-49 years	62·1 (51·2 to 75·8)	60·4 (48·5 to 74·8)	-2·78 (-8·61 to 4·78)	49·7 (40·9 to 60·6)	705 (586 to 859)	660 (538 to 822)	-6·39 (-14·5 to 6·54)	458 (381 to 558)
	50-69 years	136 (104 to 168)	130 (102 to 161)	-3·79 (-11·9 to 5·57)	109 (83·2 to 134)	838 (728 to 962)	790 (657 to 942)	-5·63 (-16·9 to 8·94)	545 (473 to 625)
	70+ years	207 (166 to 256)	191 (154 to 238)	-7·55 (-14·9 to 2·86)	166 (133 to 205)	687 (583 to 793)	675 (563 to 834)	-1·64 (-13·4 to 9·26)	446 (379 to 515)

eTable 2. Progress towards the END-TB 2020 milestones in all-form tuberculosis incidence rate per 100,000 population and in deaths due to all-form tuberculosis by age for 204 countries and territories (2020), percent change from 2015 to 2020.

Location	Age group	2015 Rate	2020 Rate	Incidence Percent Change	Incidence Milestone	2015 Deaths	2020 Deaths	Mortality Percent Change	Mortality Milestone
Maldives	All Ages	51·7 (45·2 to 59·5)	48·8 (43·5 to 55·5)	-5·48 (-10·4 to -1·15)	41·4 (36·2 to 47·6)	18·5 (16·5 to 20·8)	16·7 (14·1 to 20·8)	-9·63 (-20·9 to 6·38)	12·0 (10·7 to 13·5)
	Under 5	17·1 (13·5 to 21·0)	14·7 (11·9 to 18·3)	-14·0 (-24·7 to -4·37)	13·7 (10·8 to 16·8)	0·410 (0·323 to 0·536)	0·214 (0·153 to 0·283)	-47·6 (-59·0 to -34·9)	0·266 (0·210 to 0·348)
	5-14 years	16·8 (11·7 to 24·4)	13·9 (9·72 to 19·7)	-17·2 (-26·8 to -7·47)	13·5 (9·39 to 19·5)	0·267 (0·204 to 0·333)	0·171 (0·135 to 0·236)	-35·1 (-48·5 to -0·677)	0·174 (0·132 to 0·216)
	15-49 years	45·6 (37·2 to 57·0)	41·0 (33·7 to 50·6)	-9·86 (-16·4 to -1·81)	36·5 (29·8 to 45·6)	6·35 (5·52 to 7·39)	6·55 (5·20 to 8·35)	3·51 (-19·3 to 26·7)	4·12 (3·59 to 4·80)
	50-69 years	117 (84·9 to 142)	108 (80·6 to 132)	-7·11 (-15·3 to 1·72)	93·5 (67·9 to 114)	4·58 (3·73 to 5·74)	4·48 (3·44 to 5·85)	-1·96 (-20·6 to 25·2)	2·98 (2·42 to 3·73)
	70+ years	236 (188 to 302)	231 (191 to 284)	-1·98 (-10·4 to 8·06)	189 (151 to 241)	6·86 (5·95 to 7·84)	5·26 (4·35 to 6·57)	-23·3 (-34·5 to -11·7)	4·46 (3·87 to 5·10)
Mauritius	All Ages	14·6 (12·9 to 16·8)	15·2 (13·4 to 17·4)	4·08 (-0·954 to 10·0)	11·7 (10·3 to 13·4)	16·9 (15·6 to 18·2)	16·5 (15·0 to 17·9)	-2·18 (-7·52 to 2·00)	11·0 (10·1 to 11·8)
	Under 5	5·85 (4·39 to 7·48)	5·59 (4·33 to 7·11)	-4·32 (-14·2 to 7·69)	4·68 (3·51 to 5·99)	0·0685 (0·0588 to 0·0771)	0·0570 (0·0476 to 0·0702)	-16·6 (-27·1 to -4·15)	0·0445 (0·0382 to 0·0501)
	5-14 years	3·85 (2·37 to 5·70)	3·53 (2·27 to 5·31)	-8·03 (-20·0 to 4·49)	3·08 (1·89 to 4·56)	0·0604 (0·0511 to 0·0718)	0·0602 (0·0494 to 0·0718)	-0·261 (-9·19 to 9·07)	0·0393 (0·0332 to 0·0466)
	15-49 years	14·0 (11·6 to 17·1)	14·0 (11·5 to 17·0)	0·118 (-4·95 to 6·01)	11·2 (9·30 to 13·7)	5·45 (4·81 to 6·25)	5·36 (4·51 to 6·22)	-1·60 (-7·96 to 4·46)	3·54 (3·13 to 4·06)
	50-69 years	20·8 (15·7 to 25·6)	21·1 (15·9 to 26·7)	1·70 (-6·46 to 12·6)	16·6 (12·6 to 20·5)	7·19 (6·56 to 7·86)	7·02 (6·30 to 7·76)	-2·37 (-9·44 to 3·61)	4·67 (4·26 to 5·11)
	70+ years	28·3 (22·4 to 35·9)	27·6 (21·5 to 34·7)	-2·54 (-14·0 to 9·57)	22·7 (17·9 to 28·7)	4·09 (3·74 to 4·43)	3·99 (3·60 to 4·31)	-2·31 (-8·31 to 4·02)	2·66 (2·43 to 2·88)
Myanmar	All Ages	219 (200 to 243)	185 (169 to 208)	-15·8 (-19·5 to -11·5)	176 (160 to 194)	22100 (18800 to 26500)	17700 (14000 to 22900)	-20·0 (-29·7 to -5·17)	14300 (12200 to 17200)
	Under 5	71·9 (57·3 to 90·1)	58·6 (46·9 to 72·9)	-18·2 (-27·8 to -6·87)	57·5 (45·9 to 72·1)	922 (570 to 1230)	598 (369 to 834)	-34·8 (-47·9 to -20·4)	599 (370 to 803)
	5-14 years	69·2 (46·2 to 98·3)	53·1 (35·9 to 74·9)	-23·0 (-33·8 to -14·1)	55·3 (37·0 to 78·6)	379 (268 to 492)	227 (166 to 310)	-39·9 (-52·3 to -25·4)	246 (174 to 320)
	15-49 years	215 (183 to 248)	176 (146 to 203)	-18·3 (-23·1 to -12·6)	172 (146 to 199)	7900 (6440 to 9500)	5380 (4290 to 7190)	-31·9 (-40·6 to -19·1)	5130 (4190 to 6180)
	50-69 years	424 (342 to 509)	354 (296 to 420)	-16·5 (-21·0 to -11·3)	339 (274 to 407)	7070 (5560 to 8900)	6310 (4710 to 8450)	-10·9 (-25·6 to 10·7)	4600 (3610 to 5780)
	70+ years	665 (560 to 750)	540 (452 to 621)	-18·7 (-26·4 to -8·96)	532 (448 to 600)	5790 (4640 to 7280)	5180 (3820 to 6930)	-10·8 (-23·9 to 9·22)	3770 (3020 to 4730)
Philippines	All Ages	265 (237 to 294)	308 (272 to 345)	16·2 (13·9 to 19·2)	212 (190 to 235)	28000 (26200 to 30100)	26200 (23700 to 28500)	-6·24 (-17·1 to 4·34)	18200 (17100 to 19600)
	Under 5	103 (85·8 to 131)	113 (91·9 to 144)	9·03 (3·99 to 13·0)	82·8 (68·6 to 105)	851 (681 to 1030)	547 (429 to 696)	-35·3 (-50·3 to -13·1)	553 (442 to 671)
	5-14 years	85·6 (62·3 to 118)	121 (86·8 to 168)	41·5 (35·3 to 47·8)	68·5 (49·8 to 94·2)	610 (540 to 696)	451 (382 to 512)	-25·9 (-36·1 to -14·0)	397 (351 to 452)
	15-49 years	272 (226 to 317)	296 (243 to 347)	8·89 (5·86 to 13·2)	217 (181 to 254)	8360 (7770 to 9000)	7280 (6580 to 7890)	-12·8 (-22·6 to -2·71)	5440 (5050 to 5850)

eTable 2. Progress towards the END-TB 2020 milestones in all-form tuberculosis incidence rate per 100,000 population and in deaths due to all-form tuberculosis by age for 204 countries and territories (2020), percent change from 2015 to 2020.

Location	Age group	2015 Rate	2020 Rate	Incidence Percent Change	Incidence Milestone	2015 Deaths	2020 Deaths	Mortality Percent Change	Mortality Milestone
	50-69 years	583 (472 to 722)	651 (515 to 814)	11·7 (7·46 to 15·1)	466 (378 to 578)	11000 (10200 to 11900)	10700 (9520 to 11800)	-2·31 (-15·1 to 9·75)	7160 (6620 to 7740)
	70+ years	747 (620 to 849)	877 (735 to 1030)	17·3 (13·6 to 22·1)	598 (496 to 679)	7180 (6690 to 7680)	7220 (6520 to 7850)	0·629 (-10·3 to 11·5)	4660 (4350 to 4990)
Seychelles	All Ages	36·0 (31·8 to 41·1)	35·2 (31·0 to 40·1)	-2·09 (-6·33 to 2·89)	28·8 (25·4 to 32·9)	4·37 (3·99 to 4·89)	3·84 (3·36 to 4·64)	-12·2 (-21·4 to -2·42)	2·84 (2·59 to 3·18)
	Under 5	8·54 (6·69 to 10·3)	7·91 (6·39 to 9·80)	-7·15 (-18·2 to 7·57)	6·83 (5·35 to 8·26)	0·0292 (0·0226 to 0·0348)	0·0256 (0·0205 to 0·0311)	-12·1 (-23·8 to 3·06)	0·0190 (0·0147 to 0·0226)
	5-14 years	5·76 (3·97 to 8·27)	5·38 (3·81 to 7·86)	-6·20 (-17·1 to 7·52)	4·61 (3·18 to 6·61)	0·0262 (0·0191 to 0·0329)	0·0114 (0·00871 to 0·0150)	-55·9 (-67·2 to -40·7)	0·0170 (0·0124 to 0·0214)
	15-49 years	34·5 (29·0 to 41·4)	33·0 (27·5 to 40·1)	-4·33 (-10·2 to 1·57)	27·6 (23·2 to 33·1)	1·47 (1·25 to 1·72)	1·28 (1·06 to 1·49)	-13·1 (-23·1 to -2·02)	0·958 (0·814 to 1·12)
	50-69 years	63·6 (50·3 to 79·6)	60·6 (47·8 to 74·9)	-4·58 (-11·9 to 4·89)	50·9 (40·3 to 63·7)	1·58 (1·33 to 1·89)	1·55 (1·26 to 1·96)	-2·17 (-16·1 to 14·5)	1·03 (0·863 to 1·23)
	70+ years	86·9 (68·9 to 109)	81·2 (63·3 to 101)	-6·29 (-17·3 to 2·92)	69·5 (55·1 to 87·1)	1·26 (1·13 to 1·45)	0·975 (0·845 to 1·17)	-22·6 (-31·7 to -12·9)	0·819 (0·733 to 0·943)
Sri Lanka	All Ages	50·8 (44·5 to 58·8)	49·9 (43·4 to 57·3)	-1·76 (-6·18 to 3·51)	40·7 (35·6 to 47·1)	1070 (907 to 1280)	866 (512 to 1390)	-19·1 (-47·3 to 13·1)	694 (590 to 830)
	Under 5	11·3 (8·91 to 14·0)	9·85 (8·02 to 12·3)	-12·8 (-23·3 to -0·734)	9·05 (7·13 to 11·2)	5·88 (4·80 to 6·93)	3·53 (2·66 to 4·83)	-39·9 (-52·7 to -23·5)	3·82 (3·12 to 4·50)
	5-14 years	9·23 (6·47 to 13·1)	8·03 (5·30 to 11·7)	-12·8 (-22·1 to -3·74)	7·38 (5·17 to 10·5)	5·43 (4·25 to 6·71)	3·30 (0·355 to 5·48)	-38·9 (-94·0 to 1·24)	3·53 (2·76 to 4·36)
	15-49 years	42·2 (35·1 to 51·4)	40·4 (33·2 to 50·2)	-4·13 (-11·6 to 2·66)	33·8 (28·0 to 41·1)	191 (163 to 232)	164 (107 to 250)	-14·1 (-39·0 to 17·4)	124 (106 to 151)
	50-69 years	102 (75·6 to 126)	95·2 (71·6 to 116)	-6·51 (-12·7 to 0·729)	81·6 (60·5 to 101)	457 (366 to 548)	349 (200 to 573)	-23·8 (-54·7 to 6·60)	297 (238 to 356)
	70+ years	139 (110 to 178)	127 (101 to 161)	-8·76 (-17·9 to 1·48)	111 (88·3 to 143)	409 (334 to 505)	346 (205 to 523)	-15·4 (-48·6 to 17·7)	266 (217 to 329)
Thailand	All Ages	173 (153 to 201)	176 (155 to 198)	1·72 (-4·59 to 6·51)	139 (122 to 161)	12700 (10800 to 14900)	12700 (10400 to 15000)	0·294 (-12·4 to 21·6)	8250 (7030 to 9680)
	Under 5	27·1 (21·3 to 35·1)	23·7 (19·1 to 30·7)	-12·1 (-22·0 to -1·72)	21·6 (17·0 to 28·1)	43·4 (36·3 to 49·4)	27·4 (23·2 to 32·7)	-36·8 (-44·9 to -27·6)	28·2 (23·6 to 32·1)
	5-14 years	44·6 (30·4 to 61·7)	41·5 (28·8 to 57·5)	-6·75 (-18·7 to 4·46)	35·7 (24·3 to 49·3)	39·7 (34·1 to 46·5)	30·2 (24·8 to 37·5)	-23·8 (-38·0 to -9·24)	25·8 (22·2 to 30·2)
	15-49 years	151 (123 to 182)	148 (121 to 183)	-1·99 (-9·77 to 3·59)	121 (98·7 to 146)	4160 (3030 to 5590)	3340 (2360 to 4380)	-19·4 (-27·7 to -9·87)	2700 (1970 to 3640)
	50-69 years	247 (184 to 306)	234 (174 to 293)	-5·03 (-13·7 to 4·74)	197 (148 to 245)	3480 (2880 to 4130)	3850 (3040 to 4780)	10·9 (-7·95 to 35·4)	2260 (1870 to 2680)
	70+ years	410 (332 to 513)	387 (318 to 478)	-5·41 (-14·3 to 5·49)	328 (266 to 410)	4960 (4060 to 5800)	5440 (4300 to 6650)	10·1 (-9·13 to 42·4)	3220 (2640 to 3770)
Timor-Leste	All Ages	262 (237 to 288)	264 (241 to 291)	0·563 (-3·32 to 4·54)	210 (189 to 230)	608 (414 to 954)	647 (451 to 960)	6·71 (-6·09 to 19·0)	395 (269 to 620)
	Under 5	113 (88·7 to 141)	113 (91·9 to 140)	0·283 (-14·3 to 13·9)	90·6 (70·9 to 113)	37·4 (24·8 to 50·8)	34·5 (23·8 to 45·5)	-7·09 (-24·0 to 12·4)	24·3 (16·1 to 33·0)

eTable 2. Progress towards the END-TB 2020 milestones in all-form tuberculosis incidence rate per 100,000 population and in deaths due to all-form tuberculosis by age for 204 countries and territories (2020), percent change from 2015 to 2020.

Location	Age group	2015 Rate	2020 Rate	Incidence Percent Change	Incidence Milestone	2015 Deaths	2020 Deaths	Mortality Percent Change	Mortality Milestone
	5-14 years	90·8 (62·1 to 129)	92·9 (63·0 to 126)	2·60 (-6·36 to 16·1)	72·6 (49·7 to 103)	13·8 (10·2 to 18·4)	12·5 (9·25 to 16·3)	-8·93 (-26·4 to 13·9)	8·97 (6·60 to 12·0)
	15-49 years	216 (178 to 250)	213 (174 to 247)	-1·29 (-6·59 to 6·21)	173 (142 to 200)	135 (91·9 to 170)	143 (96·4 to 193)	6·17 (-9·80 to 25·0)	87·7 (59·7 to 111)
	50-69 years	831 (683 to 1010)	802 (664 to 947)	-3·43 (-9·71 to 3·09)	665 (546 to 810)	226 (153 to 364)	227 (150 to 329)	1·12 (-12·3 to 21·7)	147 (99·6 to 237)
	70+ years	1250 (1020 to 1440)	1250 (1050 to 1420)	-0·547 (-9·80 to 7·94)	1000 (818 to 1150)	196 (129 to 348)	230 (155 to 395)	17·7 (-1·43 to 35·8)	127 (83·9 to 227)
Viet Nam	All Ages	178 (163 to 195)	172 (156 to 186)	-3·57 (-8·26 to 0·510)	143 (130 to 156)	25000 (21400 to 29500)	21800 (17900 to 28100)	-13·1 (-22·1 to -1·62)	16300 (13900 to 19200)
	Under 5	40·9 (33·1 to 51·5)	34·5 (26·4 to 42·5)	-15·5 (-24·6 to -5·78)	32·8 (26·5 to 41·2)	254 (199 to 328)	142 (103 to 194)	-43·8 (-57·8 to -27·4)	165 (129 to 213)
	5-14 years	31·3 (21·8 to 44·4)	24·2 (17·2 to 33·7)	-22·6 (-31·0 to -14·1)	25·1 (17·5 to 35·5)	111 (89·6 to 139)	80·8 (60·4 to 104)	-26·8 (-40·5 to -10·2)	71·9 (58·2 to 90·4)
	15-49 years	168 (146 to 189)	158 (137 to 176)	-6·03 (-12·2 to -0·0313)	135 (117 to 151)	7480 (5950 to 8990)	5520 (4500 to 6800)	-26·1 (-36·1 to -13·0)	4860 (3870 to 5840)
	50-69 years	315 (261 to 365)	308 (261 to 361)	-1·97 (-9·02 to 5·86)	252 (209 to 292)	6580 (5370 to 8280)	6790 (4940 to 9770)	2·83 (-12·1 to 21·8)	4280 (3490 to 5380)
	70+ years	628 (552 to 698)	584 (523 to 654)	-6·88 (-13·4 to 1·26)	502 (442 to 558)	10600 (8270 to 13000)	9230 (7080 to 11900)	-12·8 (-23·7 to -1·34)	6880 (5370 to 8430)
Sub-Saharan Africa	All Ages	333 (297 to 370)	276 (246 to 306)	-17·1 (-18·2 to -15·6)	266 (238 to 296)	646000 (557000 to 727000)	549000 (476000 to 624000)	-15·1 (-20·1 to -8·55)	420000 (362000 to 473000)
	Under 5	187 (156 to 227)	143 (121 to 177)	-23·6 (-26·2 to -21·2)	149 (125 to 182)	76700 (59000 to 90300)	50000 (35400 to 62600)	-35·0 (-42·7 to -26·7)	49800 (38300 to 58700)
	5-14 years	93·0 (64·9 to 128)	71·6 (49·3 to 99·6)	-23·0 (-25·6 to -21·0)	74·4 (51·9 to 102)	19900 (16700 to 22800)	13700 (11400 to 16100)	-31·1 (-36·7 to -26·3)	12900 (10900 to 14800)
	15-49 years	435 (366 to 515)	356 (303 to 427)	-18·3 (-20·0 to -16·7)	348 (293 to 412)	295000 (255000 to 341000)	246000 (210000 to 288000)	-16·6 (-21·7 to -11·0)	192000 (166000 to 221000)
	50-69 years	724 (581 to 897)	617 (491 to 760)	-14·8 (-16·8 to -12·5)	579 (465 to 717)	166000 (144000 to 187000)	156000 (133000 to 179000)	-6·04 (-12·2 to 2·10)	108000 (93300 to 122000)
	70+ years	1010 (836 to 1200)	866 (722 to 1030)	-14·3 (-15·9 to -12·6)	808 (669 to 961)	89000 (77800 to 97200)	83500 (72400 to 92600)	-6·15 (-11·4 to 2·64)	57900 (50600 to 63200)
Central Sub-Saharan Africa	All Ages	396 (355 to 438)	335 (300 to 366)	-15·4 (-19·5 to -11·7)	317 (284 to 350)	96900 (75600 to 123000)	80800 (59600 to 106000)	-16·6 (-25·4 to -6·56)	63000 (49100 to 79700)
	Under 5	325 (264 to 394)	252 (206 to 314)	-22·5 (-29·4 to -16·7)	260 (211 to 315)	15500 (10500 to 22100)	8210 (4960 to 11100)	-47·0 (-54·7 to -37·2)	10100 (6810 to 14300)
	5-14 years	136 (95·9 to 187)	105 (69·9 to 144)	-22·7 (-29·7 to -15·5)	109 (76·7 to 149)	3260 (2500 to 4250)	2450 (1930 to 3270)	-24·6 (-35·2 to -14·5)	2120 (1630 to 2760)
	15-49 years	462 (383 to 531)	388 (321 to 452)	-16·2 (-21·3 to -10·5)	370 (307 to 424)	38800 (30100 to 49700)	32400 (23700 to 42600)	-16·5 (-25·0 to -6·24)	25200 (19600 to 32300)
	50-69 years	951 (777 to 1150)	859 (698 to 1040)	-9·68 (-14·8 to -4·16)	761 (622 to 922)	28300 (20300 to 36500)	27500 (18700 to 37300)	-2·78 (-15·9 to 11·3)	18400 (13200 to 23700)
	70+ years	1180 (976 to 1360)	1010 (838 to 1180)	-14·2 (-20·9 to -7·07)	944 (781 to 1090)	11100 (7590 to 14600)	10200 (7300 to 13900)	-7·39 (-20·3 to 6·21)	7190 (4930 to 9470)

eTable 2. Progress towards the END-TB 2020 milestones in all-form tuberculosis incidence rate per 100,000 population and in deaths due to all-form tuberculosis by age for 204 countries and territories (2020), percent change from 2015 to 2020.

Location	Age group	2015 Rate	2020 Rate	Incidence Percent Change	Incidence Milestone	2015 Deaths	2020 Deaths	Mortality Percent Change	Mortality Milestone
Angola	All Ages	328 (293 to 366)	278 (247 to 308)	-15·1 (-19·6 to -10·2)	262 (234 to 293)	16600 (11700 to 21300)	15900 (12100 to 20800)	-4·12 (-16·5 to 11·0)	10800 (7590 to 13800)
	Under 5	287 (234 to 356)	222 (182 to 269)	-22·6 (-31·4 to -11·2)	230 (187 to 285)	3420 (2510 to 4400)	2200 (1540 to 3040)	-35·7 (-47·7 to -22·4)	2230 (1630 to 2860)
	5-14 years	107 (77·9 to 149)	84·4 (58·6 to 117)	-20·8 (-31·4 to -9·59)	85·6 (62·3 to 119)	571 (424 to 740)	458 (326 to 610)	-19·5 (-34·2 to -2·40)	371 (276 to 481)
	15-49 years	405 (337 to 461)	346 (283 to 407)	-14·4 (-20·4 to -7·62)	324 (269 to 369)	6700 (4660 to 9320)	6930 (4910 to 9810)	3·66 (-9·46 to 20·7)	4360 (3030 to 6060)
	50-69 years	751 (606 to 923)	689 (563 to 850)	-8·20 (-13·7 to -0·0731)	600 (485 to 739)	4250 (2970 to 5800)	4580 (3360 to 6010)	8·78 (-10·9 to 31·4)	2760 (1930 to 3770)
	70+ years	887 (731 to 1030)	802 (642 to 952)	-9·60 (-18·6 to -0·754)	710 (585 to 826)	1650 (1060 to 2290)	1700 (1160 to 2270)	3·49 (-12·4 to 21·9)	1070 (690 to 1490)
Central African Republic	All Ages	678 (605 to 747)	628 (563 to 700)	-7·41 (-11·4 to -2·24)	543 (484 to 598)	11100 (8570 to 13500)	9870 (7260 to 12900)	-10·7 (-21·1 to 1·55)	7180 (5570 to 8750)
	Under 5	538 (434 to 652)	456 (352 to 563)	-15·2 (-25·0 to -6·37)	430 (347 to 521)	1630 (1190 to 2090)	1160 (833 to 1610)	-28·2 (-43·4 to -11·5)	1060 (774 to 1360)
	5-14 years	223 (162 to 314)	215 (143 to 299)	-3·52 (-14·4 to 9·02)	178 (129 to 251)	335 (258 to 411)	271 (196 to 350)	-18·8 (-30·7 to -4·71)	217 (168 to 267)
	15-49 years	812 (683 to 925)	740 (614 to 863)	-8·89 (-14·8 to -2·14)	650 (546 to 740)	5040 (3880 to 6310)	4360 (3300 to 5770)	-13·4 (-22·4 to -2·90)	3270 (2520 to 4100)
	50-69 years	1530 (1290 to 1790)	1430 (1220 to 1690)	-6·31 (-13·4 to 1·14)	1230 (1030 to 1430)	3220 (2390 to 4270)	3260 (2160 to 4250)	1·05 (-11·9 to 13·5)	2100 (1550 to 2770)
	70+ years	1840 (1630 to 2060)	1710 (1450 to 1930)	-6·85 (-16·8 to 0·714)	1470 (1300 to 1650)	831 (553 to 1100)	815 (538 to 1070)	-1·84 (-12·5 to 10·5)	540 (359 to 716)
Congo	All Ages	327 (293 to 366)	287 (251 to 323)	-12·2 (-16·8 to -7·93)	262 (234 to 293)	3180 (2250 to 4480)	2880 (2070 to 4280)	-9·56 (-20·4 to 4·09)	2070 (1460 to 2920)
	Under 5	215 (172 to 262)	161 (129 to 202)	-24·9 (-32·8 to -14·1)	172 (138 to 209)	269 (178 to 362)	152 (97·8 to 218)	-43·7 (-53·9 to -31·6)	175 (115 to 235)
	5-14 years	103 (72·5 to 146)	81·5 (58·0 to 114)	-20·5 (-30·6 to -8·99)	82·1 (58·0 to 117)	81·9 (54·8 to 115)	59·1 (38·0 to 84·8)	-27·7 (-37·5 to -12·4)	53·3 (35·6 to 74·9)
	15-49 years	395 (329 to 463)	340 (283 to 410)	-13·7 (-19·4 to -7·09)	316 (264 to 371)	1620 (1070 to 2430)	1430 (985 to 2130)	-12·2 (-23·1 to 0·709)	1050 (698 to 1580)
	50-69 years	670 (545 to 812)	605 (478 to 723)	-9·76 (-16·6 to -3·26)	536 (436 to 649)	856 (584 to 1230)	915 (639 to 1380)	7·01 (-8·13 to 25·8)	557 (379 to 801)
	70+ years	721 (572 to 849)	633 (506 to 782)	-12·2 (-20·3 to -2·52)	577 (458 to 679)	348 (256 to 469)	324 (233 to 428)	-6·77 (-19·2 to 8·63)	226 (166 to 305)
Democratic Republic of the Congo	All Ages	412 (368 to 454)	345 (307 to 379)	-16·1 (-21·4 to -10·9)	329 (294 to 363)	64900 (48800 to 89800)	51100 (35000 to 75400)	-21·4 (-30·8 to -8·99)	42200 (31700 to 58400)
	Under 5	337 (269 to 409)	259 (209 to 328)	-22·9 (-32·2 to -15·4)	269 (215 to 327)	10000 (6420 to 16000)	4630 (2650 to 7070)	-54·1 (-63·9 to -41·1)	6530 (4170 to 10400)
	5-14 years	145 (100 to 200)	109 (72·1 to 151)	-24·6 (-33·1 to -15·2)	116 (80·2 to 160)	2230 (1690 to 3170)	1630 (1200 to 2390)	-26·7 (-42·3 to -13·3)	1450 (1100 to 2060)
	15-49 years	470 (386 to 544)	389 (318 to 459)	-17·2 (-23·4 to -9·95)	376 (309 to 435)	24900 (19000 to 34500)	19200 (13300 to 27900)	-23·0 (-33·9 to -9·78)	16200 (12400 to 22400)

eTable 2. Progress towards the END-TB 2020 milestones in all-form tuberculosis incidence rate per 100,000 population and in deaths due to all-form tuberculosis by age for 204 countries and territories (2020), percent change from 2015 to 2020.

Location	Age group	2015 Rate	2020 Rate	Incidence Percent Change	Incidence Milestone	2015 Deaths	2020 Deaths	Mortality Percent Change	Mortality Milestone
	50-69 years	1010 (818 to 1220)	913 (735 to 1100)	-10·0 (-16·9 to -3·41)	812 (654 to 979)	19700 (13500 to 27000)	18400 (11800 to 27600)	-6·48 (-22·4 to 8·37)	12800 (8790 to 17500)
	70+ years	1300 (1070 to 1500)	1100 (891 to 1300)	-15·6 (-24·4 to -6·04)	1040 (858 to 1200)	8080 (5320 to 11300)	7240 (4830 to 10400)	-10·0 (-25·7 to 5·91)	5250 (3460 to 7330)
Equatorial Guinea	All Ages	191 (165 to 219)	181 (158 to 208)	-5·15 (-11·1 to 0·399)	153 (132 to 175)	324 (203 to 527)	339 (211 to 527)	4·92 (-11·1 to 22·6)	210 (132 to 343)
	Under 5	154 (127 to 192)	131 (103 to 163)	-14·7 (-23·3 to -1·40)	123 (102 to 154)	39·5 (22·7 to 62·7)	32·0 (18·7 to 50·5)	-19·0 (-30·9 to -4·94)	25·7 (14·7 to 40·7)
	5-14 years	66·2 (45·6 to 91·2)	64·0 (44·1 to 89·0)	-3·07 (-15·2 to 10·6)	53·0 (36·5 to 72·9)	14·2 (8·32 to 21·6)	13·1 (7·98 to 23·0)	-8·31 (-24·7 to 18·2)	9·24 (5·41 to 14·0)
	15-49 years	241 (198 to 291)	222 (184 to 273)	-7·89 (-13·5 to -0·529)	193 (159 to 233)	171 (100 to 290)	184 (106 to 291)	8·10 (-9·49 to 28·2)	111 (65·2 to 188)
	50-69 years	401 (312 to 505)	392 (302 to 482)	-2·08 (-10·8 to 7·59)	321 (250 to 404)	63·1 (42·0 to 102)	75·4 (49·0 to 112)	19·8 (-0·858 to 48·2)	41·0 (27·3 to 66·2)
	70+ years	441 (346 to 555)	418 (332 to 525)	-5·21 (-12·8 to 4·14)	353 (277 to 444)	35·8 (25·3 to 52·9)	34·4 (22·7 to 49·0)	-3·64 (-19·1 to 15·1)	23·3 (16·4 to 34·4)
Gabon	All Ages	275 (243 to 311)	232 (202 to 264)	-15·7 (-21·2 to -9·98)	220 (194 to 249)	848 (543 to 1160)	755 (487 to 1100)	-11·1 (-21·9 to 2·85)	551 (353 to 756)
	Under 5	176 (142 to 216)	123 (101 to 153)	-29·8 (-36·0 to -22·1)	140 (113 to 173)	58·9 (39·6 to 81·4)	30·0 (20·0 to 43·5)	-49·1 (-59·2 to -38·4)	38·3 (25·7 to 52·9)
	5-14 years	91·1 (61·5 to 124)	71·8 (49·3 to 101)	-20·9 (-31·6 to -7·64)	72·9 (49·2 to 98·8)	21·0 (13·0 to 30·9)	15·7 (10·1 to 22·9)	-24·9 (-35·3 to -12·1)	13·6 (8·44 to 20·1)
	15-49 years	325 (266 to 390)	268 (225 to 330)	-17·4 (-24·8 to -9·38)	260 (213 to 312)	403 (247 to 580)	340 (214 to 500)	-15·6 (-25·8 to 0·301)	262 (161 to 377)
	50-69 years	537 (425 to 673)	472 (369 to 589)	-12·0 (-19·2 to -5·03)	430 (340 to 538)	253 (152 to 362)	273 (165 to 400)	8·17 (-8·87 to 27·8)	164 (98·9 to 236)
	70+ years	542 (431 to 654)	463 (363 to 574)	-14·4 (-24·0 to -6·45)	434 (345 to 523)	113 (70·1 to 150)	96·1 (63·1 to 136)	-14·2 (-27·0 to 4·56)	73·2 (45·6 to 97·4)
Eastern Sub-Saharan Africa	All Ages	330 (294 to 373)	281 (248 to 315)	-15·1 (-16·7 to -13·5)	264 (235 to 298)	269000 (224000 to 305000)	234000 (190000 to 270000)	-12·9 (-18·2 to -6·08)	175000 (145000 to 198000)
	Under 5	169 (141 to 208)	132 (109 to 163)	-21·9 (-24·7 to -19·1)	135 (113 to 166)	28200 (22100 to 33900)	18400 (13900 to 23800)	-35·0 (-42·1 to -24·1)	18400 (14400 to 22000)
	5-14 years	90·9 (64·5 to 128)	71·4 (48·7 to 102)	-21·5 (-25·4 to -17·9)	72·7 (51·6 to 102)	8030 (6600 to 9360)	5630 (4660 to 6690)	-29·8 (-35·9 to -23·5)	5220 (4290 to 6080)
	15-49 years	432 (360 to 529)	358 (301 to 438)	-17·3 (-19·1 to -15·6)	346 (288 to 423)	126000 (104000 to 147000)	108000 (87800 to 128000)	-14·1 (-20·0 to -7·86)	81800 (67300 to 95700)
	50-69 years	795 (632 to 988)	681 (540 to 838)	-14·4 (-17·2 to -11·8)	636 (506 to 790)	67800 (55900 to 77200)	64200 (50800 to 75000)	-5·16 (-11·9 to 3·02)	44000 (36400 to 50200)
	70+ years	1200 (993 to 1420)	1060 (881 to 1250)	-11·8 (-14·0 to -9·33)	959 (794 to 1140)	38900 (32100 to 44000)	37800 (31200 to 44300)	-2·95 (-10·3 to 6·62)	25300 (20800 to 28600)
Burundi	All Ages	370 (322 to 419)	291 (255 to 336)	-21·3 (-24·4 to -18·3)	296 (258 to 335)	8640 (7200 to 10700)	7750 (5870 to 9750)	-10·3 (-22·0 to 2·42)	5620 (4680 to 6950)
	Under 5	205 (170 to 256)	151 (122 to 194)	-26·1 (-34·1 to -19·0)	164 (136 to 205)	1110 (766 to 1470)	584 (323 to 897)	-47·6 (-62·0 to -31·2)	720 (498 to 959)

eTable 2. Progress towards the END-TB 2020 milestones in all-form tuberculosis incidence rate per 100,000 population and in deaths due to all-form tuberculosis by age for 204 countries and territories (2020), percent change from 2015 to 2020.

Location	Age group	2015 Rate	2020 Rate	Incidence Percent Change	Incidence Milestone	2015 Deaths	2020 Deaths	Mortality Percent Change	Mortality Milestone
	5-14 years	103 (71·9 to 144)	77·5 (52·5 to 109)	-24·8 (-34·3 to -13·9)	82·7 (57·5 to 115)	234 (185 to 285)	180 (139 to 235)	-22·9 (-37·1 to -9·91)	152 (120 to 185)
	15-49 years	469 (382 to 592)	358 (299 to 451)	-23·6 (-28·2 to -18·9)	375 (306 to 474)	3450 (2870 to 4400)	3010 (2330 to 3780)	-12·8 (-25·1 to 1·33)	2240 (1870 to 2860)
	50-69 years	975 (758 to 1220)	820 (643 to 1010)	-15·8 (-22·3 to -7·47)	780 (606 to 978)	2480 (1930 to 3050)	2530 (1790 to 3410)	1·97 (-11·9 to 18·2)	1610 (1250 to 1980)
	70+ years	1520 (1230 to 1800)	1310 (1070 to 1580)	-13·5 (-25·0 to -3·58)	1210 (980 to 1440)	1370 (1050 to 1720)	1450 (986 to 1930)	6·07 (-7·84 to 22·1)	888 (684 to 1120)
Comoros	All Ages	237 (210 to 273)	217 (189 to 260)	-8·45 (-13·8 to -1·96)	190 (168 to 218)	327 (238 to 420)	311 (226 to 431)	-4·98 (-16·2 to 9·55)	213 (155 to 273)
	Under 5	95·7 (74·4 to 122)	81·8 (65·4 to 105)	-14·3 (-24·4 to 1·41)	76·5 (59·5 to 97·4)	19·2 (12·3 to 27·8)	12·5 (7·68 to 18·8)	-34·1 (-48·4 to -15·7)	12·5 (7·97 to 18·1)
	5-14 years	33·4 (23·0 to 49·5)	28·9 (19·6 to 41·3)	-13·5 (-23·4 to -1·18)	26·7 (18·4 to 39·6)	3·32 (2·23 to 4·84)	2·47 (1·66 to 3·64)	-24·7 (-42·6 to -1·06)	2·16 (1·45 to 3·15)
	15-49 years	249 (208 to 307)	214 (177 to 282)	-14·0 (-20·0 to -7·30)	199 (166 to 246)	96·9 (68·7 to 132)	86·8 (59·6 to 129)	-10·3 (-23·8 to 3·69)	63·0 (44·6 to 86·1)
	50-69 years	598 (472 to 739)	538 (410 to 684)	-10·0 (-18·2 to 0·346)	478 (377 to 591)	106 (74·4 to 143)	106 (74·7 to 154)	0·934 (-15·1 to 22·4)	68·7 (48·3 to 92·7)
	70+ years	955 (762 to 1190)	862 (666 to 1070)	-9·63 (-17·8 to 3·08)	764 (609 to 952)	102 (71·7 to 130)	103 (72·1 to 140)	0·611 (-13·1 to 20·2)	66·5 (46·6 to 84·4)
Djibouti	All Ages	276 (238 to 320)	227 (198 to 269)	-17·8 (-22·5 to -11·9)	221 (191 to 256)	567 (307 to 778)	517 (284 to 751)	-9·05 (-23·5 to 3·43)	369 (199 to 506)
	Under 5	115 (89·3 to 145)	86·1 (68·7 to 107)	-24·7 (-34·1 to -14·5)	91·7 (71·4 to 116)	43·1 (27·4 to 61·6)	29·4 (18·6 to 42·2)	-31·2 (-46·8 to -8·15)	28·0 (17·8 to 40·0)
	5-14 years	52·4 (35·0 to 74·9)	41·3 (28·3 to 59·7)	-20·9 (-31·9 to -10·5)	41·9 (28·0 to 59·9)	6·52 (3·88 to 9·29)	5·76 (3·48 to 8·86)	-11·6 (-25·1 to 3·83)	4·24 (2·52 to 6·04)
	15-49 years	316 (257 to 396)	249 (205 to 317)	-20·9 (-27·8 to -12·9)	252 (205 to 317)	258 (134 to 376)	214 (118 to 316)	-17·1 (-27·8 to -5·53)	167 (87·2 to 244)
	50-69 years	675 (517 to 855)	558 (413 to 694)	-17·3 (-24·3 to -9·24)	540 (414 to 684)	179 (88·3 to 265)	177 (92·4 to 261)	-0·654 (-20·1 to 18·9)	117 (57·4 to 172)
	70+ years	949 (742 to 1170)	789 (617 to 993)	-16·7 (-25·5 to -8·25)	759 (594 to 940)	80·9 (41·9 to 111)	90·0 (49·9 to 126)	11·5 (-10·6 to 29·0)	52·6 (27·3 to 72·1)
Eritrea	All Ages	472 (413 to 530)	412 (360 to 471)	-12·9 (-16·8 to -7·78)	378 (330 to 424)	5160 (3370 to 7310)	4860 (3080 to 7460)	-5·64 (-15·4 to 6·93)	3350 (2190 to 4750)
	Under 5	271 (217 to 333)	225 (175 to 286)	-17·0 (-26·0 to -7·21)	217 (174 to 266)	397 (245 to 626)	320 (188 to 515)	-19·0 (-33·8 to 4·29)	258 (159 to 407)
	5-14 years	93·1 (62·7 to 133)	75·4 (49·8 to 108)	-18·9 (-30·0 to -7·28)	74·5 (50·2 to 106)	78·5 (42·9 to 119)	60·9 (34·0 to 105)	-22·6 (-32·3 to -8·83)	51·0 (27·9 to 77·1)
	15-49 years	569 (466 to 685)	483 (396 to 599)	-15·2 (-20·5 to -8·69)	455 (373 to 548)	2240 (1390 to 3350)	2040 (1210 to 3360)	-8·74 (-21·9 to 6·00)	1460 (907 to 2180)
	50-69 years	1230 (975 to 1510)	1090 (863 to 1360)	-11·7 (-17·7 to -5·80)	988 (780 to 1210)	1670 (1100 to 2370)	1680 (1100 to 2460)	0·785 (-11·8 to 16·0)	1090 (713 to 1540)
	70+ years	1700 (1330 to 2060)	1500 (1140 to 1850)	-12·0 (-20·9 to -3·17)	1360 (1060 to 1650)	768 (474 to 1130)	755 (465 to 1130)	-1·36 (-14·6 to 14·6)	499 (308 to 737)

eTable 2. Progress towards the END-TB 2020 milestones in all-form tuberculosis incidence rate per 100,000 population and in deaths due to all-form tuberculosis by age for 204 countries and territories (2020), percent change from 2015 to 2020.

Location	Age group	2015 Rate	2020 Rate	Incidence Percent Change	Incidence Milestone	2015 Deaths	2020 Deaths	Mortality Percent Change	Mortality Milestone
Ethiopia	All Ages	232 (205 to 258)	208 (187 to 233)	-10·5 (-13·1 to -7·54)	186 (164 to 206)	40000 (35800 to 44000)	35000 (30700 to 40000)	-12·4 (-21·9 to -1·62)	26000 (23300 to 28600)
	Under 5	126 (101 to 158)	104 (83·7 to 128)	-17·3 (-22·7 to -12·2)	101 (80·4 to 126)	3780 (2920 to 4740)	2650 (2070 to 3410)	-29·5 (-42·4 to -14·5)	2460 (1900 to 3080)
	5-14 years	60·2 (40·9 to 85·0)	48·9 (33·4 to 68·2)	-18·7 (-23·8 to -13·3)	48·2 (32·7 to 68·0)	908 (706 to 1100)	613 (491 to 806)	-32·3 (-42·2 to -19·3)	590 (459 to 718)
	15-49 years	279 (228 to 328)	241 (199 to 289)	-13·7 (-16·7 to -10·4)	223 (183 to 262)	14600 (12900 to 16600)	12400 (10500 to 14700)	-14·8 (-26·1 to -5·31)	9480 (8380 to 10800)
	50-69 years	645 (522 to 804)	583 (481 to 713)	-9·58 (-14·0 to -5·54)	516 (418 to 643)	10500 (9080 to 11800)	9730 (8290 to 11500)	-6·78 (-19·1 to 5·99)	6800 (5900 to 7660)
	70+ years	1090 (899 to 1270)	1020 (852 to 1160)	-6·34 (-11·0 to -2·31)	868 (719 to 1010)	10300 (9070 to 11600)	9630 (7960 to 11300)	-6·54 (-17·1 to 7·09)	6700 (5900 to 7550)
Kenya	All Ages	321 (281 to 365)	248 (216 to 281)	-22·9 (-24·1 to -21·1)	257 (225 to 292)	43100 (27500 to 52700)	35100 (20400 to 43800)	-18·6 (-30·3 to -6·70)	28000 (17900 to 34200)
	Under 5	115 (93·5 to 142)	76·2 (61·9 to 94·8)	-33·5 (-34·8 to -32·0)	91·6 (74·8 to 113)	2380 (1440 to 3080)	1390 (868 to 1830)	-41·3 (-47·4 to -34·1)	1550 (939 to 2000)
	5-14 years	87·6 (59·4 to 123)	58·5 (39·4 to 81·7)	-33·2 (-34·8 to -30·7)	70·1 (47·5 to 98·5)	1050 (658 to 1340)	600 (344 to 774)	-43·1 (-48·8 to -36·0)	685 (428 to 870)
	15-49 years	413 (344 to 501)	303 (251 to 375)	-26·6 (-27·9 to -24·7)	330 (275 to 401)	21000 (13300 to 25800)	15600 (9090 to 19300)	-26·0 (-36·1 to -14·0)	13700 (8620 to 16700)
	50-69 years	735 (582 to 915)	559 (447 to 697)	-23·9 (-25·8 to -21·6)	588 (466 to 732)	12400 (7810 to 15100)	11200 (6620 to 13900)	-9·35 (-24·0 to 7·72)	8030 (5080 to 9790)
	70+ years	1270 (1030 to 1520)	1050 (852 to 1260)	-17·2 (-19·1 to -14·7)	1020 (827 to 1210)	6270 (3780 to 8590)	6320 (3610 to 8830)	0·996 (-17·4 to 21·2)	4080 (2460 to 5580)
Madagascar	All Ages	255 (225 to 290)	223 (194 to 259)	-12·4 (-16·7 to -7·17)	204 (180 to 232)	11300 (8470 to 14800)	10800 (7690 to 14600)	-4·19 (-18·2 to 11·2)	7330 (5510 to 9600)
	Under 5	143 (114 to 176)	118 (93·9 to 147)	-17·4 (-27·8 to -6·30)	114 (90·9 to 141)	1370 (887 to 1930)	882 (516 to 1290)	-35·6 (-49·4 to -17·9)	892 (576 to 1250)
	5-14 years	51·3 (33·9 to 72·5)	42·0 (27·3 to 59·6)	-18·1 (-27·5 to -6·32)	41·1 (27·1 to 58·0)	212 (159 to 292)	174 (123 to 242)	-17·7 (-34·7 to -0·830)	138 (103 to 190)
	15-49 years	321 (269 to 407)	272 (223 to 348)	-15·5 (-20·0 to -9·92)	257 (215 to 325)	4350 (3230 to 5700)	4260 (3020 to 5810)	-1·90 (-16·3 to 15·4)	2830 (2100 to 3700)
	50-69 years	698 (546 to 873)	618 (479 to 780)	-11·4 (-19·1 to -3·55)	559 (436 to 699)	3590 (2550 to 4950)	3720 (2550 to 5250)	3·83 (-17·9 to 23·4)	2330 (1660 to 3220)
	70+ years	1010 (793 to 1210)	878 (690 to 1080)	-12·9 (-21·1 to -3·66)	807 (634 to 968)	1750 (1300 to 2420)	1770 (1290 to 2490)	0·743 (-15·6 to 22·8)	1140 (845 to 1580)
Malawi	All Ages	411 (355 to 466)	370 (323 to 426)	-10·1 (-14·9 to -5·09)	329 (284 to 373)	15200 (11700 to 18400)	14600 (10800 to 18000)	-4·26 (-13·8 to 5·71)	9880 (7590 to 12000)
	Under 5	252 (210 to 308)	189 (147 to 239)	-25·1 (-34·3 to -15·4)	202 (168 to 246)	1870 (1440 to 2420)	794 (561 to 1150)	-57·6 (-66·1 to -47·0)	1210 (934 to 1570)
	5-14 years	154 (112 to 211)	134 (93·3 to 192)	-12·6 (-21·6 to -1·18)	123 (89·7 to 169)	796 (643 to 1010)	588 (446 to 807)	-26·2 (-38·9 to -13·2)	517 (418 to 659)
	15-49 years	536 (432 to 658)	471 (382 to 581)	-12·0 (-18·8 to -4·61)	429 (346 to 527)	7590 (5980 to 9590)	7670 (5730 to 9530)	1·23 (-9·90 to 12·7)	4930 (3890 to 6230)

eTable 2. Progress towards the END-TB 2020 milestones in all-form tuberculosis incidence rate per 100,000 population and in deaths due to all-form tuberculosis by age for 204 countries and territories (2020), percent change from 2015 to 2020.

Location	Age group	2015 Rate	2020 Rate	Incidence Percent Change	Incidence Milestone	2015 Deaths	2020 Deaths	Mortality Percent Change	Mortality Milestone
	50-69 years	894 (687 to 1110)	816 (617 to 1030)	-8·71 (-15·5 to 0·0846)	715 (550 to 884)	3340 (2370 to 4370)	3800 (2690 to 5040)	13·8 (-0·830 to 30·4)	2170 (1540 to 2840)
	70+ years	1210 (962 to 1500)	1110 (898 to 1430)	-7·82 (-17·4 to 2·58)	964 (770 to 1200)	1610 (1100 to 2120)	1710 (1200 to 2440)	6·42 (-8·77 to 22·3)	1050 (715 to 1380)
Mozambiqu e	All Ages	618 (526 to 705)	545 (467 to 630)	-11·9 (-16·2 to -6·24)	495 (420 to 564)	44900 (38700 to 51400)	37500 (30000 to 44500)	-16·6 (-26·4 to -7·33)	29200 (25200 to 33400)
	Under 5	285 (227 to 366)	226 (186 to 285)	-20·6 (-28·4 to -10·0)	228 (182 to 292)	4940 (4050 to 6010)	3350 (2480 to 4470)	-32·0 (-44·7 to -16·8)	3210 (2630 to 3900)
	5-14 years	165 (115 to 238)	146 (97·5 to 213)	-11·7 (-23·8 to 0·680)	132 (92·4 to 191)	1650 (1390 to 1920)	1540 (1230 to 1890)	-6·31 (-19·8 to 8·78)	1070 (901 to 1250)
	15-49 years	899 (749 to 1100)	786 (650 to 979)	-12·6 (-18·5 to -6·44)	719 (599 to 876)	24500 (21600 to 27600)	21200 (16900 to 24500)	-13·4 (-23·7 to -4·63)	15900 (14000 to 18000)
	50-69 years	1350 (1070 to 1650)	1210 (964 to 1460)	-10·9 (-17·6 to -4·52)	1080 (855 to 1320)	9690 (7530 to 12000)	7680 (5700 to 9710)	-20·8 (-32·5 to -7·84)	6300 (4900 to 7780)
	70+ years	1930 (1520 to 2270)	1700 (1350 to 2040)	-11·8 (-20·9 to -1·38)	1550 (1220 to 1810)	4130 (3140 to 5100)	3670 (2550 to 4620)	-10·9 (-26·2 to 4·17)	2690 (2040 to 3320)
Rwanda	All Ages	228 (196 to 265)	198 (172 to 230)	-13·1 (-18·2 to -7·37)	183 (157 to 212)	5510 (4290 to 6920)	5180 (3940 to 6900)	-6·20 (-16·4 to 7·00)	3580 (2790 to 4500)
	Under 5	93·4 (72·5 to 115)	70·1 (56·8 to 87·2)	-24·9 (-31·2 to -17·7)	74·7 (58·0 to 91·8)	368 (242 to 510)	234 (155 to 335)	-36·1 (-50·0 to -22·4)	239 (157 to 332)
	5-14 years	60·0 (42·2 to 83·4)	42·0 (29·8 to 60·9)	-29·9 (-38·6 to -22·2)	48·0 (33·7 to 66·8)	158 (118 to 203)	65·1 (46·9 to 87·4)	-58·8 (-66·9 to -49·7)	103 (76·9 to 132)
	15-49 years	286 (236 to 370)	241 (201 to 304)	-15·7 (-22·2 to -8·75)	229 (189 to 296)	2500 (1970 to 3180)	2250 (1720 to 3060)	-10·0 (-18·9 to -0·248)	1620 (1280 to 2070)
	50-69 years	526 (405 to 667)	470 (368 to 591)	-10·5 (-18·4 to -1·99)	421 (324 to 534)	1620 (1220 to 2130)	1710 (1230 to 2460)	5·28 (-13·0 to 28·4)	1060 (790 to 1380)
	70+ years	780 (624 to 959)	691 (538 to 867)	-11·2 (-19·0 to -3·09)	624 (499 to 767)	868 (646 to 1110)	923 (679 to 1290)	6·53 (-14·3 to 31·8)	564 (420 to 722)
Somalia	All Ages	436 (382 to 500)	375 (326 to 430)	-13·8 (-19·5 to -9·66)	348 (305 to 400)	18200 (11600 to 25800)	18800 (11700 to 26400)	3·46 (-6·41 to 15·0)	11800 (7560 to 16700)
	Under 5	230 (179 to 281)	185 (146 to 232)	-19·5 (-31·7 to -8·56)	184 (143 to 225)	2920 (1880 to 4540)	2480 (1520 to 3770)	-15·0 (-32·0 to 2·89)	1900 (1220 to 2950)
	5-14 years	101 (70·8 to 147)	83·0 (55·4 to 121)	-18·0 (-28·5 to -6·44)	81·2 (56·7 to 117)	380 (209 to 629)	350 (185 to 571)	-7·80 (-20·7 to 14·5)	247 (136 to 409)
	15-49 years	572 (459 to 725)	491 (408 to 618)	-14·0 (-20·5 to -9·07)	457 (367 to 580)	6880 (4090 to 10000)	7840 (4520 to 11500)	14·0 (-1·46 to 33·7)	4470 (2660 to 6530)
	50-69 years	1490 (1160 to 1930)	1400 (1060 to 1800)	-6·21 (-14·1 to 1·39)	1200 (925 to 1540)	5940 (3700 to 8550)	5750 (3530 to 8280)	-3·08 (-13·6 to 10·7)	3860 (2400 to 5560)
	70+ years	1960 (1490 to 2490)	1800 (1390 to 2370)	-8·13 (-17·6 to 2·45)	1570 (1190 to 2000)	2040 (1120 to 3110)	2390 (1310 to 3760)	17·5 (4·03 to 31·7)	1330 (728 to 2020)
South Sudan	All Ages	349 (306 to 399)	341 (298 to 390)	-2·02 (-7·80 to 5·93)	279 (245 to 319)	6840 (5040 to 9270)	6470 (4470 to 8940)	-5·35 (-15·7 to 6·68)	4440 (3280 to 6020)
	Under 5	197 (160 to 243)	191 (155 to 231)	-3·21 (-15·7 to 7·10)	158 (128 to 194)	1360 (944 to 1870)	1040 (749 to 1600)	-23·5 (-39·9 to -3·24)	887 (614 to 1210)

eTable 2. Progress towards the END-TB 2020 milestones in all-form tuberculosis incidence rate per 100,000 population and in deaths due to all-form tuberculosis by age for 204 countries and territories (2020), percent change from 2015 to 2020.

Location	Age group	2015 Rate	2020 Rate	Incidence Percent Change	Incidence Milestone	2015 Deaths	2020 Deaths	Mortality Percent Change	Mortality Milestone
	5-14 years	84·1 (56·5 to 117)	82·3 (57·4 to 115)	-1·94 (-13·6 to 13·6)	67·3 (45·2 to 93·3)	147 (100 to 202)	131 (86·3 to 184)	-10·7 (-26·0 to 12·1)	95·7 (65·3 to 131)
	15-49 years	462 (384 to 570)	432 (362 to 549)	-6·43 (-13·8 to -0·494)	370 (307 to 456)	2700 (1870 to 3750)	2390 (1640 to 3380)	-11·0 (-24·1 to 1·61)	1750 (1220 to 2440)
	50-69 years	922 (720 to 1170)	887 (685 to 1130)	-3·75 (-12·3 to 4·60)	738 (576 to 935)	1640 (1120 to 2330)	1930 (1270 to 2730)	18·1 (0·714 to 37·4)	1070 (731 to 1520)
	70+ years	1320 (1050 to 1600)	1220 (986 to 1510)	-7·81 (-17·1 to 5·42)	1060 (844 to 1280)	987 (718 to 1410)	973 (689 to 1420)	-1·42 (-16·3 to 19·4)	642 (466 to 917)
Uganda	All Ages	358 (321 to 401)	294 (252 to 333)	-17·8 (-24·0 to -12·0)	286 (256 to 321)	26500 (22300 to 30900)	23000 (19200 to 28400)	-13·2 (-23·6 to -2·28)	17200 (14500 to 20100)
	Under 5	182 (144 to 228)	140 (113 to 174)	-22·9 (-32·3 to -14·1)	145 (115 to 182)	2640 (1750 to 3660)	1840 (1180 to 2680)	-30·1 (-42·2 to -11·2)	1720 (1130 to 2380)
	5-14 years	107 (80·3 to 143)	73·0 (49·2 to 103)	-32·0 (-44·5 to -20·0)	85·7 (64·2 to 114)	1030 (778 to 1290)	515 (363 to 691)	-50·2 (-58·1 to -39·1)	671 (506 to 836)
	15-49 years	520 (444 to 599)	420 (342 to 501)	-19·3 (-27·9 to -12·1)	416 (355 to 479)	14100 (11600 to 17000)	12200 (10000 to 15800)	-13·1 (-25·7 to -1·29)	9160 (7530 to 11000)
	50-69 years	766 (650 to 895)	650 (534 to 775)	-15·1 (-22·0 to -7·58)	613 (520 to 716)	5600 (4210 to 6810)	5510 (3990 to 7110)	-1·74 (-16·2 to 14·1)	3640 (2740 to 4430)
	70+ years	1170 (1050 to 1280)	1040 (913 to 1170)	-10·7 (-20·6 to -1·27)	935 (839 to 1020)	3130 (2520 to 3940)	2900 (2190 to 3700)	-7·14 (-18·8 to 11·3)	2030 (1640 to 2560)
United Republic of Tanzania	All Ages	282 (245 to 332)	209 (183 to 237)	-25·7 (-29·4 to -21·6)	226 (196 to 265)	30600 (20900 to 39300)	24900 (16700 to 34000)	-18·6 (-29·2 to -8·88)	19900 (13600 to 25600)
	Under 5	147 (118 to 184)	106 (84·0 to 134)	-27·8 (-36·6 to -16·3)	118 (94·8 to 148)	3580 (2490 to 4600)	2170 (1450 to 3050)	-39·5 (-49·5 to -26·9)	2330 (1620 to 2990)
	5-14 years	78·1 (55·1 to 115)	53·2 (36·7 to 75·3)	-31·7 (-38·6 to -22·5)	62·4 (44·0 to 91·6)	891 (547 to 1290)	518 (335 to 754)	-41·7 (-49·1 to -34·1)	579 (355 to 840)
	15-49 years	369 (301 to 477)	263 (220 to 324)	-28·6 (-33·4 to -24·0)	295 (241 to 382)	14700 (9320 to 20000)	11400 (7330 to 16400)	-22·4 (-32·8 to -12·0)	9560 (6060 to 13000)
	50-69 years	606 (460 to 761)	470 (350 to 585)	-22·4 (-29·2 to -14·0)	485 (368 to 609)	6950 (4600 to 9200)	6640 (4350 to 9330)	-4·38 (-20·0 to 11·8)	4520 (2990 to 5980)
	70+ years	1000 (790 to 1250)	806 (647 to 998)	-19·5 (-27·1 to -10·9)	802 (632 to 999)	4480 (3170 to 5760)	4180 (2970 to 5570)	-6·52 (-23·2 to 13·2)	2910 (2060 to 3750)
Zambia	All Ages	465 (394 to 536)	410 (352 to 469)	-11·8 (-16·2 to -5·93)	372 (315 to 429)	11800 (7990 to 16000)	9130 (5910 to 13300)	-23·4 (-33·5 to -10·8)	7690 (5190 to 10400)
	Under 5	214 (170 to 265)	153 (122 to 195)	-28·5 (-37·5 to -16·5)	171 (136 to 212)	1430 (1010 to 1980)	558 (336 to 846)	-61·2 (-69·9 to -51·4)	929 (654 to 1280)
	5-14 years	153 (103 to 217)	121 (83·2 to 170)	-20·4 (-30·1 to -9·92)	122 (82·5 to 173)	471 (299 to 623)	282 (163 to 437)	-40·8 (-50·5 to -20·0)	306 (194 to 405)
	15-49 years	659 (541 to 837)	583 (486 to 725)	-11·5 (-17·6 to -4·54)	527 (433 to 670)	6840 (4460 to 9500)	5380 (3170 to 8200)	-22·0 (-32·5 to -7·30)	4450 (2900 to 6170)
	50-69 years	909 (700 to 1140)	786 (613 to 992)	-13·5 (-19·0 to -5·01)	727 (560 to 913)	2070 (1370 to 2890)	2030 (1290 to 2910)	-2·35 (-16·6 to 16·2)	1350 (893 to 1880)
	70+ years	1230 (957 to 1510)	1050 (826 to 1290)	-14·1 (-20·4 to -7·10)	981 (766 to 1210)	1020 (660 to 1370)	879 (542 to 1230)	-13·9 (-27·7 to 0·716)	663 (429 to 892)

eTable 2. Progress towards the END-TB 2020 milestones in all-form tuberculosis incidence rate per 100,000 population and in deaths due to all-form tuberculosis by age for 204 countries and territories (2020), percent change from 2015 to 2020.

Location	Age group	2015 Rate	2020 Rate	Incidence Percent Change	Incidence Milestone	2015 Deaths	2020 Deaths	Mortality Percent Change	Mortality Milestone
Southern Sub-Saharan Africa	All Ages	969 (853 to 1100)	863 (759 to 961)	-11·0 (-13·4 to -8·63)	775 (682 to 877)	117000 (107000 to 125000)	95700 (85600 to 104000)	-18·3 (-22·2 to -14·1)	76100 (69400 to 81500)
	Under 5	301 (248 to 378)	237 (196 to 292)	-21·4 (-25·6 to -17·7)	241 (198 to 302)	4240 (3670 to 4880)	2790 (2320 to 3540)	-34·3 (-40·6 to -24·7)	2760 (2390 to 3170)
	5-14 years	269 (192 to 362)	205 (144 to 272)	-23·8 (-27·5 to -20·5)	215 (153 to 289)	3420 (3070 to 3690)	1620 (1380 to 1860)	-52·7 (-57·7 to -48·0)	2230 (1990 to 2400)
	15-49 years	1360 (1150 to 1590)	1220 (1030 to 1400)	-10·3 (-13·6 to -7·77)	1090 (919 to 1270)	71100 (63400 to 77800)	56600 (49000 to 62000)	-20·4 (-24·4 to -16·8)	46200 (41200 to 50600)
	50-69 years	1020 (837 to 1210)	899 (755 to 1060)	-11·8 (-14·0 to -8·85)	816 (670 to 967)	29900 (27200 to 32400)	27000 (24300 to 30000)	-9·73 (-14·0 to -4·91)	19400 (17700 to 21100)
	70+ years	886 (747 to 1000)	791 (676 to 891)	-10·8 (-13·5 to -7·03)	709 (598 to 802)	8410 (7590 to 8960)	7680 (6910 to 8550)	-8·66 (-13·5 to 0·473)	5460 (4930 to 5820)
Botswana	All Ages	758 (642 to 908)	645 (544 to 769)	-14·8 (-20·4 to -8·46)	606 (513 to 727)	3220 (2340 to 3870)	2740 (1870 to 3470)	-15·3 (-26·0 to -2·99)	2090 (1520 to 2510)
	Under 5	226 (183 to 285)	192 (151 to 239)	-14·8 (-24·7 to -0·883)	181 (146 to 228)	71·7 (46·2 to 104)	53·8 (29·5 to 79·6)	-24·8 (-42·8 to -3·50)	46·6 (30·1 to 67·5)
	5-14 years	181 (127 to 259)	125 (88·5 to 174)	-30·7 (-39·1 to -20·4)	145 (102 to 208)	46·2 (33·6 to 56·2)	19·5 (14·2 to 26·9)	-57·9 (-65·3 to -50·6)	30·0 (21·8 to 36·5)
	15-49 years	1030 (842 to 1320)	862 (713 to 1060)	-16·5 (-23·6 to -9·59)	827 (674 to 1050)	2110 (1550 to 2650)	1670 (1110 to 2110)	-21·2 (-31·4 to -8·85)	1370 (1010 to 1720)
	50-69 years	967 (712 to 1260)	856 (636 to 1070)	-11·4 (-19·4 to -0·134)	774 (569 to 1010)	811 (573 to 1060)	826 (559 to 1090)	1·67 (-14·8 to 18·2)	527 (372 to 691)
	70+ years	711 (525 to 922)	631 (491 to 822)	-11·0 (-20·1 to 0·338)	569 (420 to 738)	181 (134 to 231)	171 (127 to 214)	-5·22 (-18·8 to 10·8)	118 (86·9 to 150)
Eswatini	All Ages	1040 (879 to 1230)	859 (735 to 1050)	-17·3 (-22·4 to -11·3)	831 (703 to 983)	2960 (2540 to 3490)	2060 (1700 to 2440)	-30·2 (-40·0 to -20·8)	1920 (1650 to 2270)
	Under 5	373 (299 to 471)	279 (219 to 349)	-25·0 (-30·6 to -17·9)	298 (239 to 377)	162 (123 to 200)	66·7 (51·2 to 87·8)	-58·7 (-66·7 to -49·6)	105 (80·2 to 130)
	5-14 years	342 (235 to 467)	276 (190 to 394)	-19·2 (-28·3 to -7·91)	274 (188 to 373)	109 (89·0 to 136)	58·0 (39·8 to 77·6)	-46·1 (-67·4 to -22·5)	70·8 (57·9 to 88·6)
	15-49 years	1500 (1230 to 1900)	1210 (1010 to 1580)	-18·8 (-25·0 to -12·5)	1200 (983 to 1520)	1980 (1700 to 2480)	1370 (1140 to 1650)	-30·4 (-43·4 to -18·9)	1290 (1100 to 1610)
	50-69 years	1330 (999 to 1670)	1100 (831 to 1370)	-17·1 (-26·5 to -8·69)	1060 (799 to 1330)	592 (459 to 756)	464 (323 to 634)	-21·7 (-34·1 to -7·15)	385 (298 to 492)
	70+ years	1000 (692 to 1490)	835 (592 to 1150)	-16·2 (-29·6 to -5·56)	804 (554 to 1190)	112 (83·7 to 142)	97·8 (70·9 to 130)	-12·7 (-26·3 to 0·878)	73·0 (54·4 to 92·0)
Lesotho	All Ages	1220 (1060 to 1400)	1090 (954 to 1260)	-10·6 (-14·8 to -5·86)	976 (852 to 1120)	7980 (6910 to 9250)	6300 (5410 to 7170)	-20·9 (-28·2 to -13·6)	5190 (4490 to 6010)
	Under 5	499 (403 to 622)	495 (401 to 613)	-0·661 (-10·2 to 10·1)	400 (322 to 497)	288 (212 to 355)	360 (277 to 442)	25·6 (9·54 to 50·1)	187 (138 to 231)
	5-14 years	392 (272 to 545)	351 (236 to 477)	-10·3 (-22·5 to 0·292)	314 (218 to 436)	208 (179 to 247)	137 (109 to 165)	-33·6 (-55·2 to -21·4)	135 (116 to 161)
	15-49 years	1600 (1310 to 1970)	1380 (1180 to 1720)	-13·3 (-19·2 to -7·83)	1280 (1050 to 1580)	4630 (3880 to 5630)	3530 (3040 to 4190)	-23·4 (-31·3 to -13·4)	3010 (2520 to 3660)

eTable 2. Progress towards the END-TB 2020 milestones in all-form tuberculosis incidence rate per 100,000 population and in deaths due to all-form tuberculosis by age for 204 countries and territories (2020), percent change from 2015 to 2020.

Location	Age group	2015 Rate	2020 Rate	Incidence Percent Change	Incidence Milestone	2015 Deaths	2020 Deaths	Mortality Percent Change	Mortality Milestone
	50-69 years	2000 (1590 to 2500)	1770 (1400 to 2160)	-11·5 (-18·7 to -4·73)	1600 (1270 to 2000)	2390 (1890 to 2800)	1850 (1400 to 2380)	-22·7 (-32·2 to -11·3)	1560 (1230 to 1820)
	70+ years	1630 (1160 to 2160)	1480 (1100 to 2090)	-9·14 (-22·3 to 3·16)	1300 (925 to 1730)	466 (345 to 580)	421 (330 to 533)	-9·43 (-21·7 to 1·56)	303 (224 to 377)
Namibia	All Ages	673 (568 to 784)	566 (486 to 657)	-15·8 (-20·3 to -12·0)	538 (454 to 627)	2730 (2060 to 3440)	2180 (1640 to 2800)	-19·8 (-29·9 to -8·92)	1770 (1340 to 2240)
	Under 5	264 (204 to 330)	203 (164 to 256)	-23·0 (-31·6 to -12·8)	212 (163 to 264)	108 (81·3 to 148)	60·5 (44·7 to 81·4)	-44·0 (-54·7 to -30·1)	70·4 (52·8 to 96·2)
	5-14 years	213 (148 to 300)	161 (111 to 231)	-24·1 (-34·3 to -12·5)	170 (118 to 240)	86·8 (66·6 to 108)	43·0 (31·7 to 58·7)	-50·4 (-59·9 to -35·6)	56·4 (43·3 to 70·3)
	15-49 years	922 (745 to 1150)	764 (631 to 938)	-17·1 (-22·7 to -12·1)	738 (596 to 921)	1590 (1130 to 2110)	1190 (878 to 1530)	-24·7 (-34·9 to -14·4)	1030 (734 to 1370)
	50-69 years	948 (719 to 1170)	828 (626 to 1040)	-12·6 (-20·6 to -4·51)	759 (575 to 934)	734 (557 to 921)	696 (515 to 884)	-5·19 (-18·5 to 8·90)	477 (362 to 599)
	70+ years	743 (597 to 921)	647 (502 to 814)	-12·8 (-23·8 to -1·80)	595 (478 to 737)	208 (165 to 283)	191 (143 to 260)	-8·35 (-21·8 to 5·25)	135 (107 to 184)
South Africa	All Ages	1050 (921 to 1190)	958 (835 to 1070)	-8·76 (-11·6 to -5·92)	840 (737 to 950)	76900 (67800 to 81700)	61700 (54000 to 66100)	-19·8 (-22·6 to -15·4)	50000 (44000 to 53100)
	Under 5	296 (241 to 364)	231 (185 to 287)	-21·9 (-25·1 to -18·3)	237 (193 to 291)	2090 (1840 to 2410)	1300 (1110 to 1530)	-37·8 (-43·7 to -29·3)	1360 (1200 to 1570)
	5-14 years	274 (197 to 370)	207 (145 to 271)	-24·7 (-29·0 to -21·0)	219 (158 to 296)	1890 (1720 to 2040)	780 (675 to 874)	-58·6 (-62·8 to -54·3)	1230 (1120 to 1320)
	15-49 years	1480 (1240 to 1720)	1370 (1140 to 1570)	-7·44 (-11·0 to -4·07)	1180 (992 to 1370)	48100 (40700 to 51600)	37600 (31500 to 40800)	-21·9 (-25·2 to -18·2)	31200 (26500 to 33600)
	50-69 years	965 (800 to 1140)	856 (727 to 1000)	-11·2 (-13·7 to -8·10)	772 (640 to 914)	19400 (18000 to 20600)	17100 (15900 to 18400)	-11·6 (-15·4 to -6·27)	12600 (11700 to 13400)
	70+ years	828 (708 to 914)	744 (648 to 814)	-10·1 (-13·6 to -6·41)	662 (566 to 732)	5510 (5130 to 5880)	4950 (4580 to 5530)	-10·2 (-15·3 to -1·98)	3580 (3330 to 3820)
Zimbabwe	All Ages	705 (604 to 819)	566 (496 to 644)	-19·7 (-24·9 to -15·3)	564 (483 to 656)	23300 (18700 to 26500)	20700 (16000 to 24800)	-11·3 (-19·1 to -3·26)	15100 (12100 to 17200)
	Under 5	300 (247 to 389)	230 (188 to 283)	-23·2 (-34·8 to -13·6)	240 (198 to 311)	1520 (1150 to 1990)	946 (679 to 1340)	-37·7 (-48·6 to -24·5)	987 (747 to 1290)
	5-14 years	253 (178 to 363)	195 (136 to 281)	-22·8 (-33·4 to -11·3)	202 (143 to 290)	1090 (949 to 1280)	583 (417 to 748)	-46·3 (-60·6 to -32·3)	708 (617 to 833)
	15-49 years	976 (791 to 1240)	760 (636 to 931)	-22·1 (-27·6 to -16·4)	781 (633 to 989)	12700 (10800 to 14800)	11300 (9180 to 13200)	-11·6 (-20·4 to -2·81)	8280 (7010 to 9590)
	50-69 years	1200 (887 to 1560)	1050 (772 to 1360)	-12·2 (-20·0 to -2·40)	957 (709 to 1250)	6010 (4340 to 7670)	6050 (4180 to 7800)	0·467 (-9·51 to 9·82)	3910 (2820 to 4980)
	70+ years	1280 (975 to 1590)	1110 (858 to 1420)	-12·7 (-22·4 to -2·27)	1020 (780 to 1270)	1930 (1320 to 2370)	1840 (1230 to 2310)	-4·36 (-16·2 to 7·43)	1250 (856 to 1540)
Western Sub-Saharan Africa	All Ages	201 (179 to 223)	158 (140 to 174)	-21·6 (-23·3 to -19·7)	161 (143 to 179)	163000 (135000 to 195000)	138000 (113000 to 171000)	-15·4 (-23·8 to -7·37)	106000 (87400 to 127000)
	Under 5	150 (123 to 181)	113 (91·8 to 139)	-25·0 (-28·1 to -21·5)	120 (98·6 to 145)	28700 (20800 to 38000)	20600 (13000 to 29900)	-28·7 (-38·5 to -17·8)	18700 (13500 to 24700)

eTable 2. Progress towards the END-TB 2020 milestones in all-form tuberculosis incidence rate per 100,000 population and in deaths due to all-form tuberculosis by age for 204 countries and territories (2020), percent change from 2015 to 2020.

Location	Age group	2015 Rate	2020 Rate	Incidence Percent Change	Incidence Milestone	2015 Deaths	2020 Deaths	Mortality Percent Change	Mortality Milestone
	5-14 years	59·3 (40·4 to 82·8)	46·4 (32·6 to 65·3)	-21·8 (-25·5 to -18·0)	47·5 (32·3 to 66·2)	5210 (4040 to 6390)	4040 (3110 to 5040)	-22·6 (-30·5 to -14·1)	3390 (2630 to 4150)
	15-49 years	232 (193 to 284)	178 (150 to 222)	-23·2 (-25·2 to -21·1)	186 (155 to 228)	59000 (46100 to 76200)	48800 (37100 to 63500)	-17·3 (-24·8 to -9·27)	38400 (30000 to 49600)
	50-69 years	509 (397 to 635)	412 (317 to 512)	-19·0 (-21·5 to -14·4)	407 (318 to 508)	39800 (32800 to 48000)	37100 (29200 to 45500)	-7·01 (-17·3 to 4·20)	25900 (21300 to 31200)
	70+ years	854 (691 to 1050)	695 (564 to 859)	-18·7 (-21·4 to -15·8)	684 (553 to 843)	30600 (26000 to 34600)	27900 (22900 to 32900)	-9·04 (-18·1 to -0·104)	19900 (16900 to 22500)
Benin	All Ages	130 (114 to 144)	101 (88·3 to 113)	-22·4 (-26·9 to -17·0)	104 (90·9 to 115)	2660 (1990 to 3560)	2300 (1640 to 3140)	-13·8 (-26·6 to -0·981)	1730 (1290 to 2310)
	Under 5	104 (84·1 to 127)	75·8 (61·1 to 92·5)	-27·2 (-36·9 to -17·0)	83·5 (67·3 to 102)	484 (297 to 794)	297 (151 to 578)	-39·7 (-52·4 to -22·7)	314 (193 to 516)
	5-14 years	41·6 (28·9 to 59·0)	30·8 (21·3 to 43·3)	-25·8 (-34·4 to -16·1)	33·3 (23·1 to 47·2)	86·2 (57·2 to 126)	68·0 (42·4 to 107)	-21·3 (-35·2 to -8·93)	56·1 (37·2 to 81·9)
	15-49 years	147 (120 to 179)	113 (93·2 to 141)	-23·0 (-28·2 to -16·2)	117 (96·2 to 143)	946 (701 to 1260)	844 (606 to 1170)	-10·8 (-24·2 to 1·94)	615 (456 to 821)
	50-69 years	346 (272 to 433)	278 (211 to 350)	-19·4 (-26·5 to -13·1)	277 (217 to 346)	607 (447 to 772)	606 (424 to 882)	-0·235 (-18·4 to 22·5)	395 (291 to 502)
	70+ years	561 (442 to 686)	444 (354 to 550)	-20·8 (-27·7 to -13·6)	449 (354 to 549)	537 (413 to 678)	481 (364 to 630)	-10·2 (-24·2 to 6·99)	349 (269 to 441)
Burkina Faso	All Ages	194 (172 to 215)	145 (129 to 162)	-25·1 (-29·4 to -20·9)	155 (138 to 172)	7820 (6290 to 9030)	6790 (5280 to 8190)	-13·2 (-23·7 to -3·64)	5080 (4090 to 5870)
	Under 5	162 (131 to 199)	120 (96·6 to 145)	-26·0 (-32·3 to -17·6)	130 (105 to 159)	1380 (939 to 2000)	1040 (650 to 1630)	-24·7 (-35·8 to -6·15)	898 (610 to 1300)
	5-14 years	50·2 (34·0 to 71·0)	36·2 (25·2 to 49·9)	-27·7 (-36·3 to -19·5)	40·2 (27·2 to 56·8)	236 (183 to 289)	184 (134 to 234)	-22·1 (-33·2 to -9·90)	153 (119 to 188)
	15-49 years	219 (179 to 267)	158 (130 to 197)	-27·8 (-33·4 to -22·4)	175 (143 to 214)	2670 (2140 to 3220)	2180 (1660 to 2680)	-18·0 (-31·9 to -6·42)	1730 (1390 to 2090)
	50-69 years	518 (412 to 636)	407 (312 to 501)	-21·3 (-29·1 to -13·0)	415 (330 to 509)	2010 (1600 to 2420)	1900 (1440 to 2380)	-5·74 (-19·5 to 7·41)	1310 (1040 to 1570)
	70+ years	839 (667 to 1060)	664 (530 to 814)	-20·7 (-28·6 to -13·0)	671 (533 to 846)	1520 (1320 to 1790)	1490 (1160 to 1880)	-2·23 (-16·7 to 11·7)	988 (855 to 1160)
Cabo Verde	All Ages	96·2 (85·2 to 110)	85·6 (75·3 to 99·6)	-11·0 (-16·1 to -4·92)	77·0 (68·2 to 87·7)	65·4 (48·6 to 101)	67·0 (46·3 to 115)	2·04 (-13·6 to 18·7)	42·5 (31·6 to 65·8)
	Under 5	51·4 (40·8 to 64·1)	40·2 (32·0 to 51·2)	-21·6 (-29·2 to -9·80)	41·1 (32·7 to 51·3)	0·948 (0·628 to 2·01)	0·578 (0·386 to 1·12)	-38·0 (-52·7 to -21·2)	0·616 (0·408 to 1·30)
	5-14 years	23·6 (16·3 to 33·7)	19·1 (13·5 to 25·6)	-18·9 (-27·5 to -7·35)	18·9 (13·1 to 27·0)	0·638 (0·438 to 1·02)	0·490 (0·338 to 0·770)	-22·9 (-34·9 to -13·6)	0·415 (0·285 to 0·664)
	15-49 years	91·3 (76·4 to 113)	74·4 (61·8 to 91·3)	-18·4 (-22·7 to -12·5)	73·1 (61·1 to 90·6)	22·1 (14·4 to 36·5)	17·0 (11·0 to 29·5)	-22·7 (-36·5 to -9·59)	14·4 (9·33 to 23·7)
	50-69 years	197 (148 to 259)	184 (140 to 234)	-6·17 (-14·2 to 3·61)	157 (118 to 207)	16·4 (11·4 to 27·3)	22·7 (15·0 to 42·4)	38·2 (8·22 to 69·1)	10·6 (7·44 to 17·8)
	70+ years	329 (261 to 399)	303 (244 to 363)	-7·73 (-17·6 to 2·50)	263 (209 to 319)	25·4 (19·0 to 37·0)	26·2 (18·7 to 42·6)	2·86 (-13·8 to 23·5)	16·5 (12·3 to 24·1)

eTable 2. Progress towards the END-TB 2020 milestones in all-form tuberculosis incidence rate per 100,000 population and in deaths due to all-form tuberculosis by age for 204 countries and territories (2020), percent change from 2015 to 2020.

Location	Age group	2015 Rate	2020 Rate	Incidence Percent Change	Incidence Milestone	2015 Deaths	2020 Deaths	Mortality Percent Change	Mortality Milestone
Cameroon	All Ages	225 (198 to 255)	157 (138 to 176)	-30·1 (-33·9 to -25·7)	180 (158 to 204)	13000 (8100 to 19300)	9050 (5480 to 14000)	-30·5 (-39·2 to -17·8)	8470 (5260 to 12500)
	Under 5	156 (125 to 195)	106 (86·3 to 131)	-32·0 (-39·1 to -24·0)	125 (99·7 to 156)	1370 (895 to 2030)	836 (535 to 1370)	-39·4 (-47·5 to -27·2)	892 (582 to 1320)
	5-14 years	68·0 (47·5 to 93·9)	49·3 (34·2 to 70·0)	-27·3 (-36·4 to -17·6)	54·4 (38·0 to 75·1)	365 (217 to 541)	258 (163 to 378)	-29·1 (-37·5 to -16·6)	237 (141 to 351)
	15-49 years	275 (231 to 339)	186 (153 to 228)	-32·2 (-37·1 to -26·8)	220 (185 to 271)	6770 (4000 to 10600)	4370 (2620 to 7060)	-35·3 (-43·5 to -24·4)	4400 (2600 to 6910)
	50-69 years	528 (402 to 664)	381 (287 to 484)	-27·7 (-34·0 to -19·4)	423 (321 to 531)	3080 (1800 to 4700)	2360 (1340 to 3720)	-23·3 (-34·6 to -9·09)	2000 (1170 to 3060)
	70+ years	793 (639 to 1000)	577 (452 to 726)	-27·1 (-33·6 to -20·5)	634 (512 to 802)	1440 (902 to 2060)	1230 (778 to 1860)	-14·9 (-28·5 to -2·25)	936 (586 to 1340)
Chad	All Ages	230 (203 to 254)	194 (172 to 221)	-15·8 (-21·0 to -10·6)	184 (162 to 203)	7860 (5950 to 9530)	7660 (5710 to 9850)	-2·57 (-15·7 to 7·84)	5110 (3870 to 6200)
	Under 5	194 (153 to 239)	161 (133 to 201)	-17·1 (-27·0 to -7·51)	155 (123 to 191)	1650 (1170 to 2080)	1440 (1000 to 1900)	-12·5 (-31·6 to 8·71)	1070 (761 to 1350)
	5-14 years	86·7 (58·9 to 123)	73·5 (51·8 to 102)	-15·1 (-24·2 to -4·96)	69·4 (47·2 to 98·4)	318 (228 to 413)	299 (215 to 395)	-5·45 (-22·9 to 14·0)	207 (148 to 268)
	15-49 years	265 (220 to 322)	222 (181 to 280)	-16·4 (-22·1 to -9·84)	212 (176 to 258)	2680 (2030 to 3480)	2660 (1960 to 3620)	-0·666 (-13·6 to 12·3)	1750 (1320 to 2260)
	50-69 years	634 (504 to 807)	556 (428 to 693)	-12·3 (-19·4 to -6·45)	507 (403 to 645)	1890 (1310 to 2340)	1990 (1360 to 2640)	5·45 (-9·63 to 19·8)	1230 (852 to 1520)
	70+ years	1030 (808 to 1270)	881 (708 to 1080)	-14·2 (-23·9 to -4·23)	823 (646 to 1020)	1320 (916 to 1710)	1260 (860 to 1610)	-3·89 (-16·7 to 9·61)	858 (596 to 1110)
Côte d'Ivoire	All Ages	206 (179 to 234)	155 (136 to 176)	-24·3 (-28·5 to -19·1)	164 (143 to 187)	10100 (7250 to 13700)	7100 (4780 to 9830)	-29·7 (-37·8 to -20·7)	6570 (4710 to 8890)
	Under 5	144 (113 to 175)	106 (86·3 to 131)	-26·7 (-36·8 to -16·7)	115 (90·6 to 140)	1230 (873 to 1910)	635 (399 to 1020)	-48·6 (-57·5 to -35·4)	798 (567 to 1240)
	5-14 years	67·1 (46·9 to 92·3)	54·2 (37·1 to 76·7)	-19·0 (-29·7 to -9·20)	53·7 (37·6 to 73·8)	342 (243 to 501)	220 (152 to 329)	-35·6 (-43·8 to -23·7)	223 (158 to 326)
	15-49 years	242 (203 to 300)	178 (146 to 222)	-26·5 (-32·5 to -21·3)	194 (162 to 240)	4730 (3270 to 6730)	3120 (2090 to 4490)	-34·1 (-43·2 to -24·9)	3080 (2120 to 4380)
	50-69 years	483 (367 to 604)	375 (288 to 472)	-22·2 (-29·2 to -13·5)	387 (294 to 483)	2520 (1720 to 3480)	2030 (1280 to 2970)	-19·8 (-30·4 to -7·21)	1640 (1110 to 2260)
	70+ years	722 (574 to 905)	561 (448 to 699)	-22·1 (-30·4 to -13·4)	577 (460 to 724)	1270 (918 to 1610)	1100 (808 to 1560)	-13·6 (-26·5 to 1·43)	828 (597 to 1050)
Gambia	All Ages	232 (203 to 272)	196 (172 to 219)	-15·2 (-22·7 to -6·79)	185 (163 to 218)	751 (581 to 1110)	778 (549 to 1160)	3·40 (-9·74 to 19·8)	488 (378 to 723)
	Under 5	184 (144 to 227)	151 (122 to 195)	-17·1 (-36·0 to 1·45)	147 (115 to 182)	62·9 (45·4 to 98·6)	47·8 (32·3 to 78·5)	-24·0 (-36·5 to -8·14)	40·9 (29·5 to 64·1)
	5-14 years	58·1 (44·7 to 73·1)	54·7 (37·6 to 74·7)	-5·67 (-28·7 to 25·6)	46·5 (35·7 to 58·5)	17·8 (12·6 to 24·8)	16·0 (10·7 to 24·0)	-9·57 (-26·2 to 9·46)	11·5 (8·18 to 16·1)
	15-49 years	262 (207 to 327)	218 (180 to 271)	-16·6 (-24·6 to -8·38)	209 (166 to 262)	319 (226 to 481)	338 (226 to 507)	5·87 (-8·60 to 23·2)	208 (147 to 313)

eTable 2. Progress towards the END-TB 2020 milestones in all-form tuberculosis incidence rate per 100,000 population and in deaths due to all-form tuberculosis by age for 204 countries and territories (2020), percent change from 2015 to 2020.

Location	Age group	2015 Rate	2020 Rate	Incidence Percent Change	Incidence Milestone	2015 Deaths	2020 Deaths	Mortality Percent Change	Mortality Milestone
	50-69 years	626 (473 to 771)	523 (390 to 650)	-16·4 (-24·2 to -8·71)	501 (378 to 617)	183 (132 to 274)	207 (139 to 323)	13·0 (-5·97 to 31·0)	119 (85·7 to 178)
	70+ years	897 (695 to 1100)	763 (586 to 968)	-14·8 (-23·5 to -3·86)	718 (556 to 884)	168 (115 to 239)	169 (112 to 255)	0·415 (-18·0 to 17·7)	110 (75·0 to 155)
Ghana	All Ages	246 (219 to 277)	198 (177 to 229)	-19·2 (-23·9 to -12·8)	196 (175 to 221)	14700 (11800 to 18500)	12700 (9760 to 16900)	-13·7 (-23·5 to -1·35)	9580 (7680 to 12000)
	Under 5	122 (99·1 to 146)	87·7 (70·9 to 107)	-28·2 (-37·8 to -16·1)	97·8 (79·3 to 117)	911 (640 to 1530)	426 (281 to 836)	-53·8 (-61·7 to -40·8)	592 (416 to 992)
	5-14 years	70·0 (49·2 to 100)	53·4 (36·9 to 73·5)	-23·5 (-32·8 to -10·4)	56·0 (39·3 to 80·3)	336 (254 to 439)	229 (171 to 323)	-31·8 (-40·7 to -21·6)	219 (165 to 285)
	15-49 years	281 (231 to 346)	222 (186 to 276)	-20·7 (-26·6 to -14·5)	224 (185 to 277)	6730 (5150 to 8520)	5650 (4330 to 7630)	-16·1 (-25·1 to -6·38)	4370 (3350 to 5540)
	50-69 years	584 (463 to 718)	489 (380 to 598)	-16·4 (-22·8 to -9·50)	467 (371 to 574)	4120 (3100 to 5450)	3880 (2880 to 5430)	-5·65 (-20·5 to 11·9)	2680 (2010 to 3540)
	70+ years	895 (709 to 1120)	758 (599 to 931)	-15·2 (-24·6 to -6·38)	716 (567 to 897)	2640 (2080 to 3310)	2530 (1820 to 3330)	-4·09 (-20·2 to 12·8)	1720 (1350 to 2150)
Guinea	All Ages	200 (177 to 223)	156 (140 to 177)	-21·9 (-26·2 to -18·2)	160 (142 to 178)	5550 (4280 to 6720)	4550 (3320 to 6060)	-18·2 (-28·4 to -6·68)	3610 (2780 to 4370)
	Under 5	148 (117 to 180)	110 (88·2 to 132)	-25·9 (-34·6 to -15·1)	119 (93·7 to 144)	732 (492 to 1080)	461 (270 to 810)	-37·6 (-49·9 to -21·8)	476 (320 to 705)
	5-14 years	53·4 (37·5 to 76·0)	40·7 (27·8 to 56·9)	-23·6 (-33·8 to -12·9)	42·8 (30·0 to 60·8)	152 (118 to 191)	126 (90·0 to 169)	-17·3 (-30·8 to -2·35)	99·0 (76·8 to 124)
	15-49 years	227 (186 to 281)	178 (147 to 224)	-21·6 (-26·7 to -15·6)	181 (149 to 225)	2060 (1540 to 2650)	1740 (1330 to 2240)	-15·1 (-26·5 to -1·98)	1340 (1000 to 1720)
	50-69 years	509 (415 to 620)	423 (332 to 538)	-16·9 (-23·8 to -9·38)	407 (332 to 496)	1400 (1040 to 1720)	1240 (882 to 1650)	-11·4 (-25·4 to 1·70)	909 (674 to 1120)
	70+ years	854 (683 to 1040)	687 (552 to 858)	-19·5 (-26·8 to -10·2)	683 (547 to 830)	1210 (918 to 1560)	974 (714 to 1320)	-19·3 (-31·6 to -1·86)	784 (597 to 1010)
Guinea-Bissau	All Ages	277 (241 to 314)	243 (211 to 275)	-12·3 (-17·6 to -5·76)	222 (193 to 251)	1410 (1060 to 1830)	1420 (1050 to 1870)	1·16 (-9·51 to 14·2)	914 (687 to 1190)
	Under 5	194 (156 to 232)	161 (127 to 192)	-16·9 (-26·8 to -6·00)	155 (125 to 185)	143 (93·6 to 215)	132 (71·1 to 194)	-7·86 (-34·9 to 12·5)	92·8 (60·8 to 139)
	5-14 years	86·6 (60·5 to 118)	73·4 (50·8 to 102)	-15·0 (-24·6 to -3·83)	69·3 (48·4 to 94·1)	47·7 (32·5 to 62·7)	35·9 (24·5 to 48·5)	-23·9 (-40·1 to 19·6)	31·0 (21·1 to 40·8)
	15-49 years	336 (274 to 410)	300 (247 to 369)	-10·8 (-17·6 to -3·10)	269 (219 to 328)	697 (496 to 983)	744 (500 to 1080)	7·09 (-8·33 to 24·8)	453 (323 to 639)
	50-69 years	701 (567 to 854)	609 (471 to 757)	-13·2 (-22·3 to -3·79)	561 (454 to 683)	359 (261 to 445)	361 (266 to 444)	0·820 (-11·8 to 16·2)	233 (170 to 289)
	70+ years	1010 (815 to 1230)	850 (676 to 1050)	-15·8 (-24·6 to -5·58)	809 (652 to 986)	159 (122 to 191)	147 (113 to 176)	-7·33 (-20·7 to 5·36)	103 (79·1 to 124)
Liberia	All Ages	146 (130 to 167)	122 (108 to 139)	-16·3 (-21·0 to -10·2)	117 (104 to 134)	1070 (716 to 1660)	925 (595 to 1610)	-14·6 (-24·2 to -1·24)	698 (465 to 1080)
	Under 5	105 (85·6 to 130)	83·1 (66·2 to 105)	-20·7 (-30·7 to -8·59)	83·9 (68·5 to 104)	128 (82·4 to 212)	72·6 (41·0 to 143)	-43·7 (-59·2 to -20·7)	82·9 (53·5 to 138)

eTable 2. Progress towards the END-TB 2020 milestones in all-form tuberculosis incidence rate per 100,000 population and in deaths due to all-form tuberculosis by age for 204 countries and territories (2020), percent change from 2015 to 2020.

Location	Age group	2015 Rate	2020 Rate	Incidence Percent Change	Incidence Milestone	2015 Deaths	2020 Deaths	Mortality Percent Change	Mortality Milestone
	5-14 years	40·3 (27·7 to 57·5)	33·6 (23·2 to 47·2)	-16·5 (-25·9 to -4·33)	32·3 (22·2 to 46·0)	28·3 (19·9 to 43·9)	24·6 (17·2 to 44·4)	-13·2 (-28·7 to 8·71)	18·4 (13·0 to 28·6)
	15-49 years	168 (138 to 209)	137 (115 to 172)	-17·9 (-24·4 to -10·4)	134 (111 to 168)	433 (282 to 692)	388 (235 to 677)	-11·0 (-23·1 to 4·95)	281 (183 to 450)
	50-69 years	393 (300 to 495)	337 (256 to 417)	-14·1 (-20·4 to -4·62)	314 (240 to 396)	260 (174 to 389)	252 (155 to 429)	-3·67 (-18·2 to 15·7)	169 (113 to 253)
	70+ years	637 (509 to 795)	526 (420 to 665)	-17·3 (-25·9 to -9·54)	510 (407 to 636)	225 (153 to 361)	187 (119 to 338)	-17·2 (-30·5 to -0·170)	146 (99·4 to 234)
Mali	All Ages	162 (143 to 180)	132 (117 to 148)	-18·1 (-21·8 to -13·2)	129 (114 to 144)	6580 (5130 to 8690)	6000 (4470 to 8370)	-8·97 (-19·6 to 1·75)	4280 (3330 to 5650)
	Under 5	121 (95·0 to 142)	97·5 (77·2 to 119)	-19·1 (-28·4 to -11·8)	96·6 (76·0 to 113)	862 (621 to 1330)	752 (474 to 1220)	-13·1 (-30·3 to 5·19)	560 (404 to 866)
	5-14 years	45·1 (30·3 to 63·9)	36·2 (25·0 to 50·6)	-19·5 (-28·6 to -12·5)	36·1 (24·3 to 51·1)	168 (123 to 238)	153 (110 to 212)	-8·69 (-22·0 to 2·07)	109 (79·9 to 155)
	15-49 years	180 (149 to 223)	144 (120 to 179)	-19·7 (-24·9 to -14·2)	144 (119 to 178)	2140 (1620 to 2910)	1830 (1400 to 2520)	-14·4 (-24·3 to -2·85)	1390 (1050 to 1890)
	50-69 years	483 (378 to 604)	418 (321 to 512)	-13·4 (-19·5 to -5·86)	386 (302 to 483)	1820 (1340 to 2500)	1720 (1250 to 2390)	-5·50 (-18·7 to 14·6)	1180 (870 to 1620)
	70+ years	864 (663 to 1050)	739 (590 to 901)	-14·2 (-22·1 to -5·51)	691 (531 to 843)	1600 (1150 to 2090)	1550 (1060 to 2080)	-3·02 (-15·9 to 12·4)	1040 (746 to 1360)
Mauritania	All Ages	75·4 (66·6 to 84·8)	62·8 (55·0 to 71·3)	-16·7 (-20·8 to -12·2)	60·3 (53·3 to 67·8)	492 (348 to 759)	441 (299 to 683)	-10·7 (-24·1 to 8·21)	320 (226 to 493)
	Under 5	60·2 (48·1 to 74·3)	46·7 (36·9 to 59·4)	-22·3 (-31·8 to -12·0)	48·2 (38·5 to 59·4)	47·1 (31·0 to 75·1)	27·4 (17·5 to 51·1)	-42·2 (-54·4 to -26·7)	30·6 (20·2 to 48·8)
	5-14 years	22·5 (15·6 to 31·8)	18·3 (12·5 to 26·0)	-18·7 (-28·4 to -6·86)	18·0 (12·5 to 25·4)	12·0 (8·60 to 16·2)	8·81 (6·00 to 12·4)	-26·3 (-41·7 to -8·04)	7·80 (5·59 to 10·5)
	15-49 years	69·5 (56·7 to 85·8)	56·0 (44·6 to 68·4)	-19·4 (-24·4 to -14·2)	55·6 (45·4 to 68·7)	113 (74·5 to 164)	102 (65·8 to 158)	-10·2 (-26·5 to 5·97)	73·4 (48·4 to 107)
	50-69 years	222 (170 to 278)	195 (147 to 246)	-12·1 (-19·2 to -3·29)	177 (136 to 223)	144 (96·3 to 230)	141 (88·8 to 226)	-2·84 (-21·5 to 18·5)	93·9 (62·6 to 150)
	70+ years	393 (309 to 491)	333 (262 to 411)	-15·2 (-22·9 to -7·17)	315 (247 to 392)	175 (128 to 280)	162 (111 to 259)	-7·65 (-21·8 to 11·8)	114 (83·1 to 182)
Niger	All Ages	182 (159 to 205)	155 (137 to 175)	-14·7 (-19·7 to -9·24)	146 (127 to 164)	7420 (5360 to 10600)	7090 (4910 to 10100)	-4·55 (-16·2 to 10·4)	4820 (3490 to 6910)
	Under 5	151 (120 to 185)	128 (101 to 165)	-15·0 (-26·2 to -1·98)	120 (96·0 to 148)	1940 (1170 to 3390)	1600 (992 to 2900)	-17·6 (-33·8 to -3·53)	1260 (762 to 2200)
	5-14 years	62·0 (42·1 to 87·1)	52·9 (36·6 to 72·5)	-14·4 (-27·3 to -3·40)	49·6 (33·7 to 69·7)	286 (210 to 395)	247 (173 to 391)	-13·5 (-30·8 to 5·78)	186 (137 to 257)
	15-49 years	207 (169 to 252)	173 (142 to 222)	-16·7 (-24·2 to -10·1)	166 (135 to 202)	2000 (1460 to 2780)	1870 (1280 to 2640)	-6·50 (-18·4 to 12·3)	1300 (952 to 1800)
	50-69 years	557 (439 to 693)	494 (384 to 603)	-11·3 (-18·1 to -3·39)	445 (351 to 554)	1900 (1320 to 2720)	1930 (1230 to 2670)	2·04 (-14·0 to 24·5)	1230 (855 to 1770)
	70+ years	897 (695 to 1110)	780 (618 to 964)	-12·9 (-20·9 to -5·22)	717 (556 to 892)	1300 (882 to 1920)	1440 (931 to 2000)	10·9 (-6·89 to 27·3)	843 (573 to 1250)

eTable 2. Progress towards the END-TB 2020 milestones in all-form tuberculosis incidence rate per 100,000 population and in deaths due to all-form tuberculosis by age for 204 countries and territories (2020), percent change from 2015 to 2020.

Location	Age group	2015 Rate	2020 Rate	Incidence Percent Change	Incidence Milestone	2015 Deaths	2020 Deaths	Mortality Percent Change	Mortality Milestone
Nigeria	All Ages	203 (180 to 225)	158 (141 to 175)	-22·0 (-24·1 to -20·1)	162 (144 to 180)	73200 (59500 to 88300)	62500 (47500 to 78400)	-14·5 (-29·2 to 0·902)	47600 (38700 to 57400)
	Under 5	158 (130 to 193)	116 (94·9 to 145)	-26·6 (-29·8 to -22·7)	127 (104 to 154)	16600 (11700 to 21500)	12100 (7230 to 17300)	-27·5 (-41·6 to -11·1)	10800 (7640 to 14000)
	5-14 years	59·0 (40·3 to 82·7)	45·6 (32·3 to 64·7)	-22·7 (-27·0 to -18·0)	47·2 (32·2 to 66·1)	2550 (1800 to 3250)	1960 (1310 to 2550)	-22·9 (-36·0 to -8·72)	1660 (1170 to 2110)
	15-49 years	234 (195 to 285)	180 (151 to 222)	-23·1 (-25·1 to -21·1)	187 (156 to 228)	22500 (17200 to 28400)	19600 (13600 to 26300)	-12·6 (-28·0 to 5·64)	14600 (11200 to 18500)
	50-69 years	505 (389 to 630)	405 (310 to 506)	-20·0 (-22·7 to -16·2)	404 (312 to 504)	16700 (12800 to 20700)	15800 (11500 to 19700)	-4·88 (-25·9 to 21·9)	10800 (8330 to 13500)
	70+ years	911 (726 to 1120)	737 (593 to 915)	-19·0 (-21·8 to -16·1)	729 (581 to 894)	14900 (11600 to 17500)	13100 (10400 to 15900)	-11·9 (-24·7 to 1·14)	9680 (7570 to 11400)
Sao Tome and Principe	All Ages	74·7 (66·3 to 83·5)	61·7 (53·9 to 71·8)	-17·4 (-21·9 to -11·7)	59·7 (53·0 to 66·8)	22·1 (16·7 to 32·8)	19·1 (13·7 to 31·6)	-13·7 (-25·9 to -0·0687)	14·4 (10·8 to 21·3)
	Under 5	45·9 (36·0 to 57·9)	33·5 (25·9 to 42·2)	-26·9 (-35·5 to -16·3)	36·8 (28·8 to 46·3)	0·476 (0·285 to 0·989)	0·218 (0·129 to 0·530)	-54·4 (-65·0 to -39·3)	0·309 (0·185 to 0·643)
	5-14 years	14·6 (9·83 to 20·5)	11·3 (7·86 to 16·3)	-22·8 (-31·9 to -9·53)	11·7 (7·87 to 16·4)	0·261 (0·187 to 0·392)	0·157 (0·102 to 0·266)	-39·8 (-53·3 to -12·9)	0·170 (0·122 to 0·255)
	15-49 years	75·4 (62·2 to 93·8)	59·5 (47·7 to 74·5)	-21·0 (-27·1 to -14·4)	60·4 (49·8 to 75·1)	6·21 (4·51 to 9·32)	5·30 (3·23 to 8·85)	-15·3 (-32·9 to 2·43)	4·04 (2·93 to 6·06)
	50-69 years	225 (171 to 279)	186 (138 to 228)	-17·3 (-23·6 to -7·87)	180 (137 to 223)	7·27 (5·22 to 11·0)	7·24 (5·06 to 12·1)	-0·335 (-14·7 to 21·8)	4·72 (3·39 to 7·18)
	70+ years	382 (294 to 472)	304 (244 to 384)	-20·3 (-28·6 to -11·3)	306 (236 to 378)	7·86 (5·49 to 11·6)	6·19 (4·15 to 9·75)	-21·3 (-31·8 to -7·67)	5·11 (3·57 to 7·52)
Senegal	All Ages	140 (122 to 159)	114 (99·1 to 127)	-18·8 (-23·9 to -14·0)	112 (97·3 to 128)	3760 (3040 to 4680)	3300 (2520 to 4240)	-12·5 (-21·9 to -0·198)	2450 (1980 to 3040)
	Under 5	89·9 (70·6 to 113)	66·6 (54·4 to 84·1)	-25·8 (-34·5 to -17·1)	71·9 (56·5 to 90·7)	303 (219 to 469)	166 (114 to 298)	-45·2 (-57·3 to -29·0)	197 (142 to 305)
	5-14 years	36·0 (24·3 to 50·0)	28·7 (20·2 to 40·6)	-20·2 (-30·9 to -9·59)	28·8 (19·5 to 40·0)	81·1 (61·0 to 108)	61·9 (43·3 to 83·8)	-23·5 (-34·1 to -8·83)	52·7 (39·6 to 69·9)
	15-49 years	152 (126 to 190)	119 (99·6 to 146)	-21·6 (-28·5 to -14·4)	121 (101 to 152)	1300 (1000 to 1690)	1080 (815 to 1520)	-17·4 (-27·1 to -2·17)	847 (653 to 1100)
	50-69 years	366 (274 to 447)	309 (234 to 394)	-15·5 (-22·7 to -6·85)	293 (219 to 357)	1080 (819 to 1420)	1020 (742 to 1370)	-5·67 (-18·8 to 8·37)	700 (532 to 925)
	70+ years	614 (496 to 769)	511 (412 to 641)	-16·7 (-26·4 to -8·64)	492 (397 to 615)	998 (781 to 1260)	977 (714 to 1340)	-2·25 (-18·2 to 13·9)	649 (508 to 820)
Sierra Leone	All Ages	268 (236 to 305)	214 (188 to 239)	-20·2 (-24·4 to -15·6)	215 (189 to 244)	3530 (2810 to 4320)	2950 (2190 to 3800)	-16·6 (-26·0 to -3·36)	2300 (1830 to 2810)
	Under 5	215 (173 to 264)	166 (128 to 206)	-22·5 (-31·5 to -11·5)	172 (138 to 211)	632 (473 to 883)	437 (299 to 654)	-31·1 (-44·1 to -11·1)	411 (308 to 574)
	5-14 years	74·2 (50·0 to 105)	57·3 (39·3 to 82·5)	-22·6 (-31·7 to -12·9)	59·4 (40·0 to 83·8)	95·2 (73·8 to 122)	73·3 (53·3 to 94·1)	-23·0 (-35·8 to -7·26)	61·9 (48·0 to 79·4)
	15-49 years	300 (253 to 374)	233 (192 to 293)	-22·3 (-28·8 to -16·6)	240 (202 to 299)	1310 (1010 to 1700)	1080 (813 to 1490)	-17·7 (-26·9 to -7·23)	852 (656 to 1110)

eTable 2. Progress towards the END-TB 2020 milestones in all-form tuberculosis incidence rate per 100,000 population and in deaths due to all-form tuberculosis by age for 204 countries and territories (2020), percent change from 2015 to 2020.

Location	Age group	2015 Rate	2020 Rate	Incidence Percent Change	Incidence Milestone	2015 Deaths	2020 Deaths	Mortality Percent Change	Mortality Milestone
	50-69 years	675 (525 to 832)	551 (427 to 704)	-18·3 (-25·1 to -10·5)	540 (420 to 665)	816 (580 to 1040)	757 (539 to 1020)	-7·37 (-23·0 to 11·6)	531 (377 to 678)
	70+ years	1010 (800 to 1270)	819 (649 to 1000)	-19·1 (-26·5 to -9·03)	811 (640 to 1010)	681 (531 to 862)	608 (463 to 790)	-10·6 (-22·6 to 7·39)	443 (345 to 560)
Togo	All Ages	228 (202 to 260)	181 (160 to 207)	-20·7 (-24·9 to -16·1)	183 (162 to 208)	3390 (2640 to 4610)	2700 (2010 to 3970)	-20·5 (-31·7 to -10·3)	2200 (1710 to 2990)
	Under 5	145 (114 to 174)	109 (86·1 to 135)	-24·7 (-35·6 to -12·3)	116 (91·6 to 140)	271 (179 to 417)	153 (88·6 to 275)	-44·2 (-56·1 to -25·5)	176 (117 to 271)
	5-14 years	66·6 (45·4 to 94·6)	52·6 (36·0 to 74·0)	-20·8 (-33·8 to -8·51)	53·3 (36·3 to 75·7)	91·4 (66·1 to 131)	68·5 (50·2 to 100)	-25·0 (-38·9 to -11·4)	59·4 (43·0 to 85·3)
	15-49 years	268 (225 to 330)	203 (168 to 253)	-24·1 (-29·8 to -19·4)	215 (180 to 264)	1630 (1180 to 2310)	1160 (824 to 1680)	-28·6 (-40·2 to -18·7)	1060 (766 to 1500)
	50-69 years	563 (436 to 701)	455 (347 to 574)	-19·1 (-26·3 to -11·3)	450 (349 to 561)	947 (714 to 1370)	874 (591 to 1390)	-7·98 (-22·4 to 8·91)	616 (464 to 892)
	70+ years	765 (598 to 958)	623 (493 to 785)	-18·5 (-26·5 to -10·3)	612 (478 to 767)	455 (359 to 589)	445 (335 to 638)	-2·28 (-18·8 to 18·3)	296 (234 to 383)

eTable 3. Percent change from 2015 to 2021 in age-standardised all-form tuberculosis incidence rate per 100,000 population and in deaths due to all-form tuberculosis by age for 204 countries and territories.

Location	Age group	2015 Rate	2021 Rate	Incidence Percent Change	Incidence Milestone	2015 Deaths	2021 Deaths	Mortality Percent Change	Mortality Milestone
**Global**	**All Ages**	**129 (114 to 144)**	**119 (106 to 133)**	**-7·79 (-9·05 to -6·70)**	**103 (91·5 to 115)**	**1570000 (1450000 to 1700000)**	**1350000 (1230000 to 1520000)**	**-14·1 (-19·5 to -6·50)**	**1020000 (943000 to 1100000)**
	**Under 5**	**67·9 (57·0 to 82·9)**	**53·8 (45·3 to 66·3)**	**-20·8 (-23·0 to -18·7)**	**54·3 (45·6 to 66·3)**	**104000 (84000 to 119000)**	**60300 (43500 to 76700)**	**-42·0 (-48·9 to -32·4)**	**67300 (54600 to 77600)**
	**5-14 years**	**41·9 (29·4 to 58·0)**	**33·0 (23·3 to 46·0)**	**-21·3 (-23·1 to -19·6)**	**33·5 (23·5 to 46·4)**	**32000 (28400 to 35500)**	**20900 (18300 to 24000)**	**-34·7 (-39·7 to -29·7)**	**20800 (18500 to 23100)**
	**15-49 years**	**141 (120 to 169)**	**131 (111 to 157)**	**-7·41 (-8·46 to -6·24)**	**113 (96·1 to 135)**	**593000 (534000 to 650000)**	**491000 (439000 to 546000)**	**-17·1 (-22·3 to -10·4)**	**385000 (347000 to 422000)**
	**50-69 years**	**188 (149 to 232)**	**173 (137 to 216)**	**-7·70 (-9·06 to -6·33)**	**150 (119 to 186)**	**499000 (467000 to 528000)**	**448000 (404000 to 510000)**	**-10·2 (-16·8 to -1·69)**	**324000 (304000 to 343000)**
	**70+ years**	**214 (172 to 260)**	**192 (156 to 234)**	**-10·4 (-12·1 to -9·01)**	**171 (138 to 208)**	**347000 (322000 to 367000)**	**331000 (302000 to 379000)**	**-4·37 (-10·8 to 5·97)**	**225000 (209000 to 239000)**
Central Europe, Eastern Europe, and Central Asia	All Ages	64·2 (55·9 to 76·0)	52·3 (45·1 to 62·7)	-18·5 (-22·2 to -14·9)	51·4 (44·7 to 60·8)	26900 (25900 to 28600)	15900 (14600 to 17300)	-41·0 (-44·7 to -36·8)	17500 (16800 to 18600)
	Under 5	11·7 (9·76 to 15·1)	8·78 (7·25 to 11·3)	-25·2 (-28·6 to -21·9)	9·39 (7·81 to 12·1)	602 (507 to 705)	334 (267 to 441)	-44·5 (-53·8 to -35·0)	391 (330 to 458)
	5-14 years	13·9 (9·46 to 19·2)	10·9 (7·52 to 15·5)	-21·4 (-27·3 to -16·3)	11·1 (7·57 to 15·4)	103 (93·6 to 113)	66·2 (59·4 to 75·1)	-35·6 (-39·5 to -30·8)	66·9 (60·9 to 73·5)
	15-49 years	86·1 (69·6 to 106)	70·9 (57·3 to 88·6)	-17·6 (-21·9 to -12·5)	68·8 (55·7 to 85·1)	12400 (11600 to 13900)	6900 (6260 to 7800)	-44·5 (-47·7 to -41·6)	8090 (7560 to 9060)
	50-69 years	66·7 (50·0 to 84·6)	56·0 (42·2 to 73·1)	-16·0 (-20·5 to -11·2)	53·4 (40·0 to 67·7)	11000 (10800 to 11300)	6410 (5850 to 7030)	-41·9 (-46·8 to -35·8)	7170 (7010 to 7350)
	70+ years	43·2 (33·5 to 54·6)	36·0 (28·2 to 45·5)	-16·6 (-21·8 to -11·8)	34·6 (26·8 to 43·7)	2680 (2550 to 2760)	2150 (2040 to 2270)	-19·8 (-23·5 to -16·3)	1740 (1660 to 1790)
Central Asia	All Ages	67·4 (59·0 to 78·0)	54·1 (47·6 to 62·8)	-19·7 (-22·5 to -16·4)	53·9 (47·2 to 62·4)	6430 (6060 to 6820)	4430 (3960 to 4950)	-31·1 (-37·4 to -23·8)	4180 (3940 to 4430)
	Under 5	18·3 (15·5 to 22·9)	14·4 (12·0 to 18·4)	-21·1 (-26·3 to -15·8)	14·7 (12·4 to 18·3)	477 (385 to 569)	288 (222 to 390)	-39·4 (-50·3 to -28·1)	310 (250 to 370)
	5-14 years	19·5 (13·7 to 27·1)	15·0 (10·6 to 20·7)	-22·7 (-28·0 to -16·1)	15·6 (11·0 to 21·7)	72·7 (64·3 to 79·2)	48·8 (43·7 to 56·1)	-32·9 (-38·7 to -25·9)	47·3 (41·8 to 51·5)
	15-49 years	81·9 (69·9 to 101)	65·5 (54·6 to 80·9)	-20·1 (-23·8 to -16·4)	65·5 (55·9 to 80·7)	3260 (3060 to 3470)	2120 (1870 to 2400)	-34·8 (-40·6 to -27·8)	2120 (1990 to 2250)
	50-69 years	96·2 (72·7 to 119)	80·9 (60·9 to 99·6)	-15·9 (-20·7 to -10·1)	76·9 (58·1 to 95·0)	2100 (1980 to 2230)	1570 (1370 to 1770)	-25·4 (-33·2 to -16·1)	1370 (1290 to 1450)
	70+ years	99·3 (79·2 to 122)	84·5 (67·0 to 106)	-14·9 (-20·1 to -8·83)	79·5 (63·3 to 97·3)	522 (479 to 560)	400 (358 to 449)	-23·2 (-29·5 to -15·3)	339 (312 to 364)
Armenia	All Ages	32·9 (28·5 to 37·7)	26·0 (22·5 to 30·5)	-21·1 (-27·4 to -15·3)	26·3 (22·8 to 30·2)	76·5 (69·5 to 83·3)	40·1 (35·5 to 45·3)	-47·5 (-53·4 to -40·2)	49·7 (45·2 to 54·1)
	Under 5	6·45 (5·10 to 8·17)	4·99 (3·90 to 6·54)	-22·5 (-33·2 to -11·3)	5·16 (4·08 to 6·53)	1·27 (1·01 to 1·51)	0·656 (0·519 to 0·846)	-48·4 (-56·5 to -37·2)	0·827 (0·656 to 0·983)
	5-14 years	5·71 (3·87 to 8·35)	4·66 (3·10 to 6·69)	-18·4 (-29·3 to -7·35)	4·57 (3·10 to 6·68)	0·283 (0·242 to 0·323)	0·128 (0·109 to 0·148)	-54·6 (-61·6 to -46·7)	0·184 (0·157 to 0·210)

eTable 3. Percent change from 2015 to 2021 in age-standardised all-form tuberculosis incidence rate per 100,000 population and in deaths due to all-form tuberculosis by age for 204 countries and territories.

Location	Age group	2015 Rate	2021 Rate	Incidence Percent Change	Incidence Milestone	2015 Deaths	2021 Deaths	Mortality Percent Change	Mortality Milestone
	15-49 years	38·0 (30·9 to 46·3)	29·7 (24·8 to 37·5)	-21·7 (-30·2 to -13·3)	30·4 (24·7 to 37·0)	26·0 (24·2 to 28·0)	16·5 (14·8 to 18·3)	-36·6 (-43·2 to -27·4)	16·9 (15·7 to 18·2)
	50-69 years	44·8 (33·9 to 57·3)	35·4 (26·0 to 46·1)	-20·8 (-29·6 to -10·1)	35·9 (27·1 to 45·8)	34·6 (31·5 to 37·7)	14·4 (12·6 to 16·4)	-58·4 (-63·8 to -52·0)	22·5 (20·5 to 24·5)
	70+ years	33·0 (25·7 to 41·1)	27·9 (21·4 to 35·2)	-15·1 (-24·8 to -5·64)	26·4 (20·6 to 32·9)	14·3 (12·4 to 16·1)	8·51 (7·41 to 9·57)	-40·5 (-47·8 to -32·2)	9·31 (8·04 to 10·5)
Azerbaijan	All Ages	61·4 (52·7 to 73·1)	55·2 (46·9 to 67·5)	-10·0 (-15·8 to -1·98)	49·1 (42·1 to 58·5)	678 (539 to 934)	416 (307 to 612)	-38·4 (-50·8 to -18·5)	441 (350 to 607)
	Under 5	16·6 (13·2 to 21·4)	13·1 (10·1 to 17·1)	-21·0 (-30·2 to -4·03)	13·3 (10·6 to 17·2)	35·2 (25·8 to 49·0)	19·4 (12·9 to 28·5)	-44·6 (-57·8 to -30·1)	22·9 (16·8 to 31·8)
	5-14 years	15·0 (10·0 to 21·0)	13·1 (9·23 to 18·0)	-12·0 (-21·6 to -0·797)	12·0 (8·00 to 16·8)	6·25 (4·55 to 8·16)	5·47 (4·15 to 6·96)	-11·9 (-28·6 to 10·8)	4·06 (2·95 to 5·31)
	15-49 years	77·4 (63·9 to 96·2)	69·8 (55·6 to 87·5)	-9·74 (-18·5 to 0·313)	61·9 (51·1 to 76·9)	357 (277 to 486)	228 (159 to 337)	-36·2 (-50·2 to -21·4)	232 (180 to 316)
	50-69 years	70·8 (52·5 to 90·5)	63·6 (47·4 to 80·9)	-9·99 (-18·5 to 0·443)	56·7 (42·0 to 72·4)	224 (167 to 329)	121 (79·5 to 190)	-45·6 (-59·2 to -11·9)	146 (108 to 214)
	70+ years	60·2 (47·9 to 74·3)	54·9 (43·1 to 68·6)	-8·61 (-19·7 to 1·68)	48·1 (38·3 to 59·5)	55·5 (41·6 to 75·5)	43·1 (31·1 to 62·0)	-22·0 (-38·9 to -3·99)	36·1 (27·0 to 49·1)
Georgia	All Ages	71·5 (62·3 to 82·1)	57·2 (50·1 to 66·9)	-20·1 (-24·5 to -14·4)	57·2 (49·9 to 65·7)	199 (180 to 219)	114 (99·9 to 131)	-42·6 (-49·1 to -35·8)	130 (117 to 143)
	Under 5	11·4 (8·82 to 14·2)	8·64 (6·87 to 11·5)	-24·1 (-34·2 to -14·0)	9·12 (7·06 to 11·3)	2·01 (1·65 to 2·50)	0·955 (0·766 to 1·21)	-52·4 (-60·2 to -42·9)	1·31 (1·07 to 1·63)
	5-14 years	18·8 (13·3 to 26·7)	12·6 (8·71 to 18·3)	-33·3 (-41·2 to -24·8)	15·1 (10·7 to 21·4)	0·603 (0·510 to 0·700)	0·216 (0·189 to 0·243)	-64·0 (-69·1 to -58·2)	0·392 (0·332 to 0·455)
	15-49 years	93·1 (76·5 to 114)	73·4 (58·6 to 94·8)	-21·2 (-26·4 to -14·5)	74·5 (61·2 to 91·6)	71·0 (66·5 to 76·2)	36·7 (32·8 to 40·9)	-48·2 (-53·3 to -43·3)	46·1 (43·2 to 49·5)
	50-69 years	78·8 (59·8 to 101)	67·9 (52·4 to 86·1)	-13·7 (-22·4 to -4·70)	63·0 (47·8 to 80·6)	87·2 (74·5 to 99·3)	48·6 (40·9 to 57·7)	-44·2 (-52·7 to -35·2)	56·7 (48·4 to 64·6)
	70+ years	59·2 (47·1 to 73·4)	52·3 (41·9 to 64·1)	-11·3 (-19·7 to -0·897)	47·3 (37·7 to 58·7)	38·5 (33·2 to 44·2)	27·8 (23·6 to 31·8)	-27·8 (-38·8 to -18·8)	25·0 (21·6 to 28·7)
Kazakhstan	All Ages	74·9 (64·5 to 87·9)	54·3 (47·0 to 63·8)	-27·5 (-31·6 to -22·8)	59·9 (51·6 to 70·3)	826 (774 to 889)	571 (501 to 635)	-30·9 (-38·9 to -23·8)	537 (503 to 578)
	Under 5	11·3 (8·91 to 14·9)	8·75 (6·88 to 11·8)	-22·7 (-33·2 to -11·3)	9·08 (7·12 to 11·9)	26·4 (22·0 to 31·4)	16·2 (12·8 to 19·7)	-38·7 (-47·7 to -26·6)	17·2 (14·3 to 20·4)
	5-14 years	12·3 (8·38 to 18·0)	9·00 (6·04 to 12·7)	-26·8 (-38·2 to -14·0)	9·85 (6·71 to 14·4)	5·93 (5·05 to 7·03)	3·13 (2·79 to 3·65)	-47·0 (-55·6 to -37·3)	3·86 (3·28 to 4·57)
	15-49 years	99·0 (82·7 to 124)	71·4 (58·1 to 90·2)	-27·8 (-32·7 to -23·0)	79·2 (66·2 to 98·9)	451 (416 to 491)	275 (243 to 302)	-39·1 (-46·5 to -32·3)	293 (270 to 319)
	50-69 years	95·5 (70·4 to 121)	75·1 (56·5 to 96·3)	-21·3 (-28·3 to -11·3)	76·4 (56·3 to 97·1)	277 (255 to 309)	200 (169 to 230)	-27·7 (-36·5 to -17·0)	180 (166 to 201)
	70+ years	86·2 (66·8 to 108)	73·6 (56·6 to 92·7)	-14·5 (-24·5 to -2·15)	68·9 (53·4 to 86·7)	65·6 (61·5 to 73·2)	76·9 (68·4 to 85·7)	17·4 (4·66 to 31·3)	42·6 (40·0 to 47·6)
Kyrgyzstan	All Ages	97·3 (84·6 to 114)	77·7 (67·4 to 89·5)	-20·1 (-25·3 to -14·2)	77·9 (67·7 to 91·1)	470 (421 to 513)	347 (297 to 412)	-26·1 (-38·1 to -13·0)	305 (274 to 333)

eTable 3. Percent change from 2015 to 2021 in age-standardised all-form tuberculosis incidence rate per 100,000 population and in deaths due to all-form tuberculosis by age for 204 countries and territories.

Location	Age group	2015 Rate	2021 Rate	Incidence Percent Change	Incidence Milestone	2015 Deaths	2021 Deaths	Mortality Percent Change	Mortality Milestone
	Under 5	17·8 (14·4 to 22·4)	13·8 (11·1 to 17·9)	-22·3 (-33·9 to -8·88)	14·2 (11·5 to 18·0)	18·5 (16·3 to 21·2)	11·3 (9·67 to 13·2)	-39·0 (-48·7 to -27·6)	12·1 (10·6 to 13·8)
	5-14 years	27·1 (18·9 to 37·5)	19·3 (13·4 to 26·9)	-28·3 (-37·5 to -16·6)	21·7 (15·1 to 30·0)	3·26 (2·74 to 3·72)	2·74 (2·35 to 3·17)	-15·5 (-27·8 to -1·05)	2·12 (1·78 to 2·42)
	15-49 years	125 (106 to 155)	99·1 (80·4 to 123)	-21·0 (-27·0 to -14·5)	100 (85·0 to 124)	262 (233 to 289)	183 (158 to 216)	-30·2 (-40·6 to -18·7)	170 (152 to 188)
	50-69 years	150 (114 to 187)	131 (96·7 to 168)	-13·0 (-22·2 to -4·54)	120 (91·5 to 149)	155 (138 to 170)	127 (107 to 154)	-17·5 (-33·3 to 0·168)	100 (89·6 to 110)
	70+ years	171 (135 to 212)	144 (112 to 179)	-15·4 (-25·5 to -0·790)	137 (108 to 170)	31·4 (28·2 to 35·1)	22·7 (19·0 to 27·6)	-27·8 (-39·5 to -14·0)	20·4 (18·3 to 22·8)
Mongolia	All Ages	132 (116 to 154)	98·0 (85·4 to 115)	-26·0 (-31·1 to -21·5)	106 (93·1 to 123)	549 (387 to 753)	379 (264 to 559)	-30·8 (-46·9 to -9·84)	357 (252 to 489)
	Under 5	35·7 (29·2 to 44·9)	25·7 (21·1 to 31·7)	-28·0 (-36·1 to -18·8)	28·6 (23·4 to 35·9)	23·0 (15·8 to 32·1)	13·9 (9·58 to 20·6)	-38·2 (-58·0 to -11·3)	14·9 (10·3 to 20·9)
	5-14 years	28·3 (19·5 to 40·4)	18·7 (12·9 to 26·1)	-33·6 (-43·1 to -24·8)	22·6 (15·6 to 32·3)	4·90 (3·58 to 6·21)	4·23 (2·98 to 5·81)	-13·2 (-31·5 to 14·6)	3·18 (2·33 to 4·04)
	15-49 years	171 (144 to 209)	132 (109 to 162)	-23·1 (-30·1 to -16·7)	137 (116 to 167)	322 (226 to 453)	215 (149 to 344)	-33·0 (-47·4 to -15·2)	209 (147 to 295)
	50-69 years	199 (158 to 245)	152 (113 to 194)	-23·8 (-30·8 to -14·5)	159 (126 to 196)	171 (116 to 232)	123 (80·4 to 172)	-27·3 (-48·1 to 0·570)	111 (75·5 to 151)
	70+ years	141 (112 to 179)	107 (84·6 to 134)	-23·5 (-33·3 to -14·4)	113 (89·4 to 143)	28·8 (20·9 to 38·2)	22·1 (16·2 to 29·0)	-22·5 (-38·8 to 0·441)	18·7 (13·6 to 24·9)
Tajikistan	All Ages	63·4 (55·5 to 73·7)	51·2 (43·2 to 60·5)	-19·4 (-24·2 to -14·7)	50·8 (44·4 to 59·0)	881 (683 to 1120)	756 (563 to 970)	-13·9 (-30·6 to 5·01)	573 (444 to 726)
	Under 5	32·1 (25·5 to 40·2)	25·2 (20·0 to 32·7)	-21·3 (-33·1 to -10·0)	25·7 (20·4 to 32·1)	162 (106 to 220)	124 (78·1 to 190)	-22·3 (-46·6 to 4·76)	105 (68·8 to 143)
	5-14 years	16·1 (10·8 to 22·7)	13·1 (8·76 to 18·6)	-18·3 (-28·6 to -8·10)	12·8 (8·67 to 18·2)	11·8 (8·79 to 15·5)	12·1 (8·44 to 16·8)	2·69 (-15·3 to 28·9)	7·68 (5·71 to 10·1)
	15-49 years	81·8 (67·6 to 98·7)	66·0 (54·1 to 82·7)	-19·3 (-24·9 to -13·2)	65·4 (54·1 to 79·0)	401 (286 to 526)	327 (231 to 437)	-17·9 (-37·0 to 2·30)	260 (186 to 342)
	50-69 years	99·7 (78·2 to 129)	80·7 (60·5 to 104)	-19·0 (-27·1 to -9·40)	79·7 (62·6 to 103)	238 (181 to 304)	241 (171 to 329)	1·64 (-19·9 to 30·2)	155 (118 to 198)
	70+ years	108 (85·1 to 136)	87·8 (70·0 to 111)	-18·5 (-26·7 to -8·53)	86·3 (68·1 to 109)	68·6 (49·0 to 91·8)	51·6 (33·9 to 71·5)	-24·3 (-41·7 to -5·41)	44·6 (31·9 to 59·6)
Turkmenistan	All Ages	59·5 (51·5 to 70·1)	48·6 (40·9 to 58·2)	-18·3 (-25·5 to -10·5)	47·6 (41·2 to 56·1)	486 (456 to 520)	388 (300 to 492)	-20·1 (-36·2 to -0·608)	316 (296 to 338)
	Under 5	16·0 (12·6 to 19·4)	13·7 (11·2 to 17·3)	-14·3 (-25·5 to 0·381)	12·8 (10·1 to 15·5)	30·2 (26·0 to 34·9)	19·1 (15·5 to 23·6)	-36·7 (-46·1 to -25·3)	19·6 (16·9 to 22·7)
	5-14 years	9·66 (6·58 to 14·1)	11·2 (7·40 to 16·6)	16·2 (-1·15 to 41·6)	7·73 (5·27 to 11·3)	4·09 (3·54 to 4·68)	3·02 (2·58 to 3·51)	-26·0 (-37·2 to -14·5)	2·66 (2·30 to 3·04)
	15-49 years	79·9 (64·6 to 99·3)	64·3 (50·4 to 79·8)	-19·5 (-28·1 to -9·90)	63·9 (51·7 to 79·5)	297 (276 to 327)	232 (179 to 296)	-22·0 (-37·2 to -2·32)	193 (179 to 213)
	50-69 years	83·4 (62·3 to 110)	63·9 (46·6 to 82·8)	-23·4 (-30·8 to -15·3)	66·7 (49·8 to 88·4)	131 (122 to 140)	115 (85·8 to 149)	-12·3 (-33·3 to 12·0)	85·2 (79·3 to 91·2)

eTable 3. Percent change from 2015 to 2021 in age-standardised all-form tuberculosis incidence rate per 100,000 population and in deaths due to all-form tuberculosis by age for 204 countries and territories.

Location	Age group	2015 Rate	2021 Rate	Incidence Percent Change	Incidence Milestone	2015 Deaths	2021 Deaths	Mortality Percent Change	Mortality Milestone
	70+ years	69·6 (52·4 to 86·1)	57·0 (44·0 to 73·2)	-18·0 (-26·8 to -7·03)	55·7 (42·0 to 68·8)	23·8 (22·0 to 25·7)	19·6 (16·0 to 23·8)	-17·4 (-32·2 to -0·919)	15·5 (14·3 to 16·7)
Uzbekistan	All Ages	58·0 (50·9 to 66·5)	48·5 (41·9 to 55·2)	-16·2 (-21·5 to -10·4)	46·4 (40·7 to 53·2)	2270 (2100 to 2460)	1420 (1230 to 1670)	-37·3 (-45·1 to -26·8)	1470 (1370 to 1600)
	Under 5	17·3 (14·3 to 22·0)	13·7 (11·3 to 18·0)	-20·5 (-30·7 to -11·5)	13·9 (11·5 to 17·6)	179 (142 to 224)	82·7 (65·5 to 105)	-53·5 (-61·3 to -43·1)	116 (92·2 to 145)
	5-14 years	25·8 (18·2 to 36·0)	19·7 (14·2 to 26·3)	-23·6 (-32·7 to -12·8)	20·6 (14·5 to 28·8)	35·6 (30·3 to 41·8)	17·7 (15·0 to 20·7)	-50·1 (-56·3 to -44·6)	23·1 (19·7 to 27·1)
	15-49 years	60·5 (51·9 to 73·7)	50·6 (42·0 to 63·8)	-16·4 (-23·6 to -9·37)	48·4 (41·5 to 59·0)	1070 (1000 to 1160)	613 (538 to 724)	-42·8 (-50·6 to -34·3)	697 (653 to 753)
	50-69 years	101 (74·3 to 128)	87·2 (64·6 to 107)	-14·0 (-21·5 to -6·70)	81·1 (59·4 to 102)	783 (715 to 856)	577 (485 to 689)	-26·2 (-38·1 to -11·9)	509 (465 to 557)
	70+ years	147 (119 to 177)	123 (94·6 to 158)	-16·3 (-26·7 to -5·36)	117 (94·9 to 142)	195 (177 to 212)	128 (109 to 149)	-34·1 (-42·9 to -24·1)	127 (115 to 138)
Central Europe	All Ages	21·4 (18·7 to 24·6)	17·7 (15·2 to 20·4)	-17·6 (-20·6 to -14·9)	17·1 (14·9 to 19·7)	2730 (2640 to 2850)	1860 (1700 to 2020)	-31·7 (-36·7 to -27·6)	1780 (1720 to 1850)
	Under 5	4·28 (3·42 to 5·49)	3·50 (2·82 to 4·47)	-18·1 (-24·2 to -11·5)	3·42 (2·73 to 4·39)	19·1 (17·6 to 20·6)	13·1 (11·1 to 15·1)	-31·4 (-41·6 to -19·7)	12·4 (11·4 to 13·4)
	5-14 years	5·10 (3·59 to 7·12)	4·03 (2·68 to 5·80)	-20·9 (-29·1 to -13·8)	4·08 (2·87 to 5·69)	7·49 (6·01 to 9·40)	4·11 (3·13 to 5·25)	-45·2 (-49·0 to -40·8)	4·87 (3·91 to 6·11)
	15-49 years	20·5 (16·8 to 25·1)	16·2 (13·1 to 20·1)	-20·9 (-24·7 to -16·9)	16·4 (13·4 to 20·1)	667 (630 to 713)	409 (364 to 452)	-38·7 (-43·7 to -34·5)	433 (409 to 463)
	50-69 years	29·5 (22·0 to 37·8)	25·1 (18·5 to 32·1)	-14·8 (-18·4 to -10·7)	23·6 (17·6 to 30·3)	1200 (1150 to 1270)	766 (694 to 836)	-36·1 (-41·5 to -31·4)	779 (750 to 823)
	70+ years	29·6 (23·5 to 36·8)	24·1 (19·4 to 29·9)	-18·6 (-22·8 to -15·4)	23·7 (18·8 to 29·4)	839 (791 to 880)	672 (618 to 726)	-19·9 (-24·1 to -15·4)	545 (514 to 572)
Albania	All Ages	13·3 (11·8 to 15·2)	12·8 (11·2 to 14·6)	-3·67 (-8·81 to 1·50)	10·7 (9·44 to 12·1)	42·6 (31·4 to 59·0)	33·0 (22·7 to 50·5)	-22·1 (-46·1 to 1·57)	27·7 (20·4 to 38·4)
	Under 5	4·39 (3·40 to 5·76)	3·73 (2·91 to 4·93)	-14·8 (-25·3 to -4·19)	3·52 (2·72 to 4·61)	0·799 (0·572 to 1·09)	0·505 (0·389 to 0·730)	-35·8 (-52·2 to -8·20)	0·520 (0·372 to 0·706)
	5-14 years	3·84 (2·62 to 5·36)	3·36 (2·28 to 4·67)	-12·2 (-22·0 to 0·363)	3·07 (2·10 to 4·28)	0·227 (0·184 to 0·284)	0·0988 (0·0751 to 0·134)	-56·2 (-65·9 to -43·0)	0·148 (0·120 to 0·184)
	15-49 years	11·3 (9·50 to 14·1)	10·4 (8·51 to 13·2)	-7·57 (-12·8 to -0·758)	9·00 (7·60 to 11·3)	8·61 (6·56 to 12·1)	5·16 (3·39 to 7·99)	-39·7 (-58·4 to -20·3)	5·60 (4·26 to 7·88)
	50-69 years	19·1 (14·3 to 25·5)	17·7 (13·3 to 22·9)	-7·03 (-16·6 to 1·45)	15·3 (11·4 to 20·4)	14·3 (9·94 to 20·7)	8·56 (5·26 to 14·2)	-39·5 (-60·0 to -11·8)	9·27 (6·46 to 13·5)
	70+ years	29·7 (22·9 to 37·0)	27·0 (21·4 to 34·3)	-8·79 (-18·0 to -0·124)	23·8 (18·3 to 29·6)	18·7 (13·7 to 26·3)	18·7 (12·4 to 28·9)	0·313 (-30·8 to 28·9)	12·2 (8·92 to 17·1)
Bosnia and Herzegovina	All Ages	28·2 (24·2 to 32·4)	25·5 (22·2 to 29·4)	-9·83 (-15·0 to -4·49)	22·6 (19·3 to 26·0)	167 (147 to 197)	111 (88·7 to 134)	-33·5 (-47·8 to -16·7)	108 (95·2 to 128)
	Under 5	4·45 (3·54 to 5·73)	3·83 (3·03 to 4·90)	-13·7 (-25·7 to -0·303)	3·56 (2·83 to 4·59)	0·426 (0·340 to 0·505)	0·279 (0·212 to 0·373)	-33·9 (-54·5 to -8·72)	0·277 (0·221 to 0·328)
	5-14 years	4·87 (3·27 to 7·12)	4·24 (2·80 to 6·00)	-12·9 (-23·1 to 0·373)	3·90 (2·61 to 5·70)	0·438 (0·365 to 0·526)	0·262 (0·206 to 0·330)	-40·0 (-49·2 to -25·2)	0·285 (0·237 to 0·342)

eTable 3. Percent change from 2015 to 2021 in age-standardised all-form tuberculosis incidence rate per 100,000 population and in deaths due to all-form tuberculosis by age for 204 countries and territories.

Location	Age group	2015 Rate	2021 Rate	Incidence Percent Change	Incidence Milestone	2015 Deaths	2021 Deaths	Mortality Percent Change	Mortality Milestone
	15-49 years	20·3 (16·4 to 25·1)	18·2 (15·1 to 22·9)	-10·2 (-15·6 to -2·57)	16·2 (13·1 to 20·1)	23·9 (20·0 to 29·2)	11·7 (8·37 to 15·2)	-50·9 (-64·5 to -36·6)	15·5 (13·0 to 19·0)
	50-69 years	41·0 (30·5 to 52·9)	35·8 (27·7 to 45·1)	-12·4 (-20·3 to -3·38)	32·8 (24·4 to 42·3)	57·1 (48·3 to 69·2)	34·9 (27·1 to 45·7)	-38·6 (-54·0 to -21·8)	37·1 (31·4 to 45·0)
	70+ years	65·5 (50·7 to 80·8)	55·2 (44·7 to 67·4)	-15·4 (-24·2 to -6·09)	52·4 (40·5 to 64·6)	85·0 (74·2 to 101)	63·5 (51·8 to 75·5)	-25·1 (-39·9 to -8·39)	55·2 (48·2 to 65·5)
Bulgaria	All Ages	20·0 (17·6 to 23·2)	16·0 (13·9 to 18·5)	-19·9 (-23·9 to -15·3)	16·0 (14·1 to 18·6)	123 (114 to 132)	83·6 (74·7 to 93·8)	-31·9 (-40·2 to -24·0)	79·9 (74·0 to 85·7)
	Under 5	5·92 (4·51 to 7·97)	4·56 (3·39 to 5·96)	-22·9 (-33·5 to -10·3)	4·74 (3·61 to 6·37)	0·421 (0·385 to 0·456)	0·228 (0·185 to 0·270)	-45·8 (-54·6 to -34·7)	0·274 (0·250 to 0·296)
	5-14 years	9·44 (6·38 to 13·3)	6·81 (4·54 to 9·71)	-27·8 (-37·2 to -17·1)	7·55 (5·10 to 10·6)	0·160 (0·129 to 0·201)	0·0921 (0·0750 to 0·120)	-42·5 (-49·1 to -36·3)	0·104 (0·0839 to 0·131)
	15-49 years	19·9 (16·5 to 24·4)	15·6 (12·8 to 20·0)	-21·5 (-26·8 to -13·6)	15·9 (13·2 to 19·5)	21·7 (19·8 to 23·8)	13·9 (11·9 to 15·7)	-35·9 (-45·8 to -26·1)	14·1 (12·9 to 15·5)
	50-69 years	24·6 (18·3 to 30·3)	20·5 (15·2 to 25·7)	-16·4 (-23·2 to -6·31)	19·7 (14·6 to 24·2)	58·0 (52·8 to 63·9)	34·4 (29·7 to 40·2)	-40·5 (-49·6 to -31·8)	37·7 (34·3 to 41·6)
	70+ years	23·6 (18·9 to 29·8)	18·8 (15·1 to 23·1)	-20·0 (-27·9 to -10·6)	18·8 (15·1 to 23·8)	42·6 (38·9 to 46·0)	35·0 (31·7 to 38·6)	-17·9 (-26·3 to -9·64)	27·7 (25·3 to 29·9)
Croatia	All Ages	11·8 (10·4 to 13·4)	9·87 (8·59 to 11·0)	-16·5 (-20·5 to -11·7)	9·46 (8·36 to 10·7)	77·6 (71·2 to 84·7)	53·5 (46·9 to 59·9)	-31·1 (-37·6 to -23·7)	50·5 (46·3 to 55·0)
	Under 5	2·61 (2·02 to 3·40)	2·17 (1·63 to 2·82)	-16·5 (-27·6 to -5·21)	2·09 (1·62 to 2·72)	0·241 (0·208 to 0·286)	0·138 (0·110 to 0·169)	-42·5 (-52·6 to -29·0)	0·157 (0·135 to 0·186)
	5-14 years	2·51 (1·68 to 3·67)	2·02 (1·27 to 2·88)	-19·4 (-30·5 to -7·29)	2·01 (1·34 to 2·93)	0·206 (0·147 to 0·285)	0·109 (0·0741 to 0·160)	-47·4 (-51·8 to -42·8)	0·134 (0·0955 to 0·185)
	15-49 years	8·64 (7·16 to 11·0)	7·01 (5·69 to 8·67)	-18·8 (-24·5 to -13·3)	6·91 (5·73 to 8·83)	11·0 (10·3 to 11·9)	5·54 (4·76 to 6·32)	-49·5 (-55·1 to -43·7)	7·13 (6·68 to 7·73)
	50-69 years	16·3 (12·3 to 20·3)	13·3 (10·2 to 17·0)	-18·3 (-24·7 to -10·0)	13·0 (9·85 to 16·3)	27·1 (24·7 to 29·5)	15·8 (13·6 to 18·1)	-41·6 (-48·8 to -34·0)	17·6 (16·1 to 19·2)
	70+ years	23·8 (19·2 to 30·1)	19·5 (15·6 to 24·0)	-18·1 (-25·7 to -9·88)	19·1 (15·4 to 24·1)	39·1 (35·1 to 43·0)	31·9 (27·5 to 36·5)	-18·6 (-26·0 to -11·2)	25·4 (22·8 to 28·0)
Czechia	All Ages	5·39 (4·61 to 6·12)	4·77 (4·06 to 5·56)	-11·5 (-17·8 to -6·84)	4·31 (3·69 to 4·90)	61·6 (57·0 to 66·1)	32·8 (29·4 to 36·5)	-46·8 (-51·7 to -41·3)	40·1 (37·0 to 43·0)
	Under 5	1·50 (1·14 to 1·90)	1·39 (1·06 to 1·90)	-6·78 (-19·3 to 7·30)	1·20 (0·911 to 1·52)	0·221 (0·178 to 0·259)	0·0883 (0·0676 to 0·115)	-60·0 (-64·3 to -53·4)	0·143 (0·116 to 0·168)
	5-14 years	1·13 (0·735 to 1·65)	1·13 (0·733 to 1·68)	0·466 (-9·75 to 16·1)	0·903 (0·588 to 1·32)	0·251 (0·176 to 0·343)	0·124 (0·0784 to 0·188)	-50·7 (-55·8 to -37·7)	0·163 (0·114 to 0·223)
	15-49 years	4·92 (4·04 to 6·18)	4·59 (3·71 to 5·71)	-6·56 (-14·7 to 0·705)	3·94 (3·23 to 4·94)	10·5 (9·46 to 11·5)	5·15 (4·40 to 5·99)	-50·8 (-56·0 to -45·2)	6·80 (6·15 to 7·45)
	50-69 years	7·21 (5·59 to 8·98)	6·13 (4·61 to 7·84)	-14·9 (-23·1 to -7·02)	5·77 (4·47 to 7·18)	23·3 (21·0 to 26·0)	9·40 (8·04 to 10·9)	-59·6 (-64·8 to -53·9)	15·2 (13·6 to 16·9)
	70+ years	8·62 (7·00 to 10·7)	6·92 (5·40 to 8·57)	-19·7 (-27·4 to -8·88)	6·89 (5·60 to 8·56)	27·4 (24·6 to 29·7)	18·0 (16·1 to 19·7)	-34·2 (-39·7 to -27·7)	17·8 (16·0 to 19·3)
Hungary	All Ages	8·75 (7·63 to 10·0)	6·60 (5·73 to 7·73)	-24·5 (-29·8 to -19·6)	7·00 (6·10 to 8·03)	117 (110 to 124)	62·4 (55·0 to 69·1)	-46·6 (-52·5 to -41·4)	76·0 (71·2 to 80·8)

eTable 3. Percent change from 2015 to 2021 in age-standardised all-form tuberculosis incidence rate per 100,000 population and in deaths due to all-form tuberculosis by age for 204 countries and territories.

Location	Age group	2015 Rate	2021 Rate	Incidence Percent Change	Incidence Milestone	2015 Deaths	2021 Deaths	Mortality Percent Change	Mortality Milestone
	Under 5	1·96 (1·55 to 2·49)	1·71 (1·30 to 2·20)	-12·7 (-24·3 to 0·791)	1·57 (1·24 to 1·99)	0·623 (0·527 to 0·753)	0·264 (0·209 to 0·336)	-57·5 (-64·2 to -49·6)	0·405 (0·343 to 0·490)
	5-14 years	1·35 (0·899 to 1·90)	1·25 (0·807 to 1·83)	-7·24 (-18·9 to 5·82)	1·08 (0·719 to 1·52)	0·637 (0·420 to 0·910)	0·240 (0·155 to 0·346)	-62·4 (-65·1 to -60·1)	0·414 (0·273 to 0·591)
	15-49 years	7·07 (5·84 to 8·68)	5·37 (4·32 to 6·83)	-24·1 (-30·2 to -17·2)	5·66 (4·67 to 6·95)	21·8 (19·9 to 23·8)	10·0 (8·55 to 11·6)	-54·0 (-60·3 to -47·9)	14·2 (12·9 to 15·4)
	50-69 years	13·6 (10·0 to 17·5)	9·89 (7·45 to 12·5)	-26·9 (-35·1 to -20·1)	10·8 (8·00 to 14·0)	49·9 (45·6 to 54·8)	25·7 (22·3 to 29·3)	-48·3 (-55·1 to -41·3)	32·4 (29·7 to 35·6)
	70+ years	13·4 (10·7 to 16·6)	9·80 (7·69 to 12·2)	-26·9 (-35·4 to -19·7)	10·7 (8·57 to 13·3)	44·0 (40·2 to 47·5)	26·2 (22·6 to 29·6)	-40·6 (-46·5 to -34·8)	28·6 (26·1 to 30·9)
Montenegro	All Ages	12·2 (10·6 to 14·3)	10·8 (9·14 to 12·6)	-11·4 (-15·9 to -6·66)	9·72 (8·45 to 11·4)	8·51 (6·91 to 10·9)	6·49 (5·02 to 8·51)	-23·6 (-36·7 to -8·40)	5·53 (4·49 to 7·12)
	Under 5	2·70 (2·02 to 3·46)	2·27 (1·67 to 2·98)	-15·9 (-25·3 to -3·80)	2·16 (1·62 to 2·76)	0·0383 (0·0287 to 0·0491)	0·0202 (0·0143 to 0·0274)	-47·0 (-58·9 to -33·3)	0·0249 (0·0186 to 0·0319)
	5-14 years	1·99 (1·32 to 2·84)	1·75 (1·13 to 2·46)	-11·9 (-23·9 to -0·315)	1·59 (1·05 to 2·28)	0·0253 (0·0183 to 0·0358)	0·0164 (0·0107 to 0·0258)	-35·4 (-46·6 to -23·1)	0·0165 (0·0119 to 0·0233)
	15-49 years	10·2 (8·49 to 13·0)	9·15 (7·46 to 11·8)	-10·3 (-16·8 to -3·69)	8·17 (6·79 to 10·4)	1·54 (1·17 to 1·96)	1·05 (0·781 to 1·44)	-31·2 (-46·9 to -17·3)	0·998 (0·758 to 1·28)
	50-69 years	19·5 (14·7 to 25·2)	16·9 (12·4 to 21·5)	-13·3 (-22·1 to -4·85)	15·6 (11·7 to 20·2)	3·74 (2·95 to 4·94)	2·52 (1·95 to 3·48)	-32·3 (-45·4 to -16·6)	2·43 (1·92 to 3·21)
	70+ years	23·1 (18·0 to 28·8)	19·3 (15·3 to 24·2)	-16·0 (-24·9 to -7·64)	18·4 (14·4 to 23·0)	3·17 (2·60 to 3·94)	2·88 (2·23 to 3·60)	-9·14 (-23·2 to 6·13)	2·06 (1·69 to 2·56)
North Macedonia	All Ages	17·6 (15·3 to 20·4)	16·6 (14·5 to 19·3)	-5·61 (-11·0 to 0·459)	14·1 (12·2 to 16·3)	58·7 (49·4 to 70·9)	33·0 (24·6 to 44·1)	-43·5 (-56·3 to -23·9)	38·1 (32·1 to 46·1)
	Under 5	3·16 (2·49 to 4·20)	2·59 (2·02 to 3·29)	-17·8 (-26·9 to -8·17)	2·53 (1·99 to 3·36)	0·229 (0·185 to 0·275)	0·0739 (0·0569 to 0·0963)	-67·6 (-75·0 to -56·1)	0·149 (0·120 to 0·179)
	5-14 years	2·77 (1·82 to 4·05)	2·30 (1·54 to 3·42)	-16·9 (-25·4 to -7·28)	2·22 (1·46 to 3·24)	0·146 (0·116 to 0·176)	0·0677 (0·0494 to 0·0906)	-53·4 (-64·1 to -39·8)	0·0948 (0·0752 to 0·115)
	15-49 years	13·1 (10·6 to 16·4)	12·3 (9·98 to 15·5)	-6·05 (-14·3 to 2·28)	10·5 (8·50 to 13·1)	10·6 (8·80 to 12·3)	4·70 (3·50 to 6·72)	-55·4 (-66·9 to -35·4)	6·86 (5·72 to 8·02)
	50-69 years	29·9 (22·3 to 37·5)	27·1 (20·0 to 33·6)	-9·21 (-16·8 to -0·214)	23·9 (17·8 to 30·0)	24·4 (19·7 to 31·4)	12·1 (8·81 to 17·4)	-50·1 (-64·1 to -25·0)	15·9 (12·8 to 20·4)
	70+ years	40·5 (31·5 to 52·2)	35·7 (27·6 to 45·1)	-11·6 (-20·3 to -1·50)	32·4 (25·2 to 41·8)	23·3 (19·6 to 28·0)	16·1 (12·3 to 20·2)	-30·8 (-44·5 to -14·0)	15·2 (12·8 to 18·2)
Poland	All Ages	16·9 (14·3 to 20·1)	15·0 (12·6 to 17·5)	-11·3 (-14·2 to -8·37)	13·5 (11·4 to 16·1)	632 (612 to 652)	441 (405 to 476)	-30·2 (-35·9 to -24·7)	411 (398 to 424)
	Under 5	2·22 (1·70 to 2·97)	2·21 (1·66 to 2·92)	-0·463 (-6·68 to 4·30)	1·78 (1·36 to 2·38)	0·612 (0·446 to 0·847)	0·313 (0·234 to 0·437)	-48·8 (-52·2 to -45·1)	0·398 (0·290 to 0·550)
	5-14 years	1·98 (1·21 to 2·98)	1·96 (1·20 to 3·01)	-0·909 (-5·51 to 4·86)	1·58 (0·969 to 2·38)	0·286 (0·220 to 0·387)	0·179 (0·131 to 0·245)	-37·5 (-40·5 to -34·0)	0·186 (0·143 to 0·252)
	15-49 years	13·2 (10·5 to 17·0)	12·2 (9·62 to 15·7)	-8·16 (-11·4 to -4·91)	10·6 (8·42 to 13·6)	140 (132 to 152)	94·0 (86·1 to 104)	-32·7 (-38·3 to -27·9)	90·9 (86·0 to 98·5)
	50-69 years	28·0 (20·3 to 37·4)	24·1 (17·6 to 31·7)	-13·7 (-17·6 to -9·58)	22·4 (16·2 to 29·9)	303 (293 to 313)	194 (175 to 212)	-36·1 (-42·3 to -29·7)	197 (190 to 203)

eTable 3. Percent change from 2015 to 2021 in age-standardised all-form tuberculosis incidence rate per 100,000 population and in deaths due to all-form tuberculosis by age for 204 countries and territories.

Location	Age group	2015 Rate	2021 Rate	Incidence Percent Change	Incidence Milestone	2015 Deaths	2021 Deaths	Mortality Percent Change	Mortality Milestone
	70+ years	27·6 (21·4 to 35·4)	22·9 (18·0 to 29·5)	-16·8 (-21·2 to -12·9)	22·1 (17·1 to 28·3)	188 (177 to 197)	153 (139 to 163)	-18·5 (-24·0 to -13·7)	122 (115 to 128)
Romania	All Ages	57·4 (50·2 to 65·6)	45·0 (38·6 to 52·3)	-21·6 (-26·6 to -16·2)	45·9 (40·2 to 52·5)	1180 (1130 to 1280)	816 (697 to 939)	-31·1 (-39·7 to -23·8)	770 (732 to 834)
	Under 5	12·6 (9·47 to 16·6)	9·56 (7·40 to 12·0)	-24·1 (-33·3 to -13·6)	10·1 (7·57 to 13·2)	14·4 (13·2 to 15·6)	10·5 (8·75 to 12·4)	-27·4 (-40·3 to -14·0)	9·37 (8·60 to 10·2)
	5-14 years	16·1 (11·7 to 22·6)	12·4 (8·19 to 17·6)	-23·0 (-34·8 to -13·2)	12·9 (9·34 to 18·1)	3·67 (2·78 to 4·82)	2·09 (1·48 to 2·82)	-43·0 (-47·3 to -38·8)	2·39 (1·81 to 3·14)
	15-49 years	65·2 (52·8 to 79·8)	48·6 (39·0 to 61·4)	-25·3 (-30·8 to -19·2)	52·1 (42·2 to 63·8)	367 (339 to 407)	223 (191 to 259)	-39·0 (-46·2 to -32·2)	238 (220 to 265)
	50-69 years	70·1 (53·0 to 89·3)	57·9 (42·3 to 74·5)	-17·4 (-25·8 to -6·26)	56·1 (42·4 to 71·5)	531 (493 to 580)	359 (305 to 418)	-32·4 (-41·6 to -24·1)	345 (320 to 377)
	70+ years	56·9 (45·6 to 68·7)	47·1 (36·9 to 57·8)	-17·3 (-26·5 to -8·91)	45·5 (36·5 to 55·0)	269 (249 to 289)	221 (193 to 246)	-17·6 (-26·2 to -9·16)	175 (162 to 188)
Serbia	All Ages	13·9 (12·0 to 16·1)	12·4 (10·8 to 14·3)	-10·7 (-16·8 to -4·50)	11·1 (9·62 to 12·9)	162 (139 to 185)	125 (95·9 to 157)	-22·9 (-35·1 to -6·29)	105 (90·1 to 120)
	Under 5	2·72 (2·08 to 3·57)	2·29 (1·79 to 2·99)	-15·5 (-29·5 to -3·14)	2·18 (1·66 to 2·86)	0·601 (0·483 to 0·733)	0·402 (0·320 to 0·494)	-32·7 (-47·7 to -16·7)	0·391 (0·314 to 0·477)
	5-14 years	3·76 (2·49 to 5·62)	3·15 (2·12 to 4·58)	-16·1 (-27·1 to -5·59)	3·01 (1·99 to 4·49)	1·25 (0·972 to 1·60)	0·718 (0·514 to 0·957)	-42·1 (-60·1 to -22·0)	0·815 (0·632 to 1·04)
	15-49 years	11·0 (9·01 to 13·6)	9·81 (8·02 to 12·2)	-10·4 (-17·8 to -3·10)	8·76 (7·21 to 10·9)	30·7 (26·8 to 34·8)	22·0 (18·3 to 27·2)	-28·1 (-39·5 to -12·8)	19·9 (17·4 to 22·6)
	50-69 years	20·4 (15·4 to 26·0)	17·8 (13·5 to 22·2)	-12·9 (-21·7 to -1·44)	16·4 (12·3 to 20·8)	65·2 (56·0 to 74·3)	45·0 (34·6 to 55·5)	-30·9 (-43·3 to -17·3)	42·4 (36·4 to 48·3)
	70+ years	26·5 (20·7 to 33·1)	22·7 (17·9 to 28·3)	-14·1 (-22·9 to -2·90)	21·2 (16·6 to 26·5)	64·5 (52·9 to 75·9)	56·8 (41·9 to 74·3)	-11·8 (-28·4 to 9·00)	41·9 (34·4 to 49·3)
Slovakia	All Ages	6·50 (5·60 to 7·58)	5·89 (5·06 to 6·93)	-9·34 (-14·9 to -2·29)	5·20 (4·48 to 6·06)	40·2 (34·3 to 47·9)	31·7 (25·8 to 38·8)	-21·0 (-33·5 to -7·22)	26·1 (22·3 to 31·1)
	Under 5	1·41 (1·10 to 1·72)	1·22 (0·955 to 1·54)	-13·1 (-24·3 to -3·58)	1·13 (0·880 to 1·38)	0·188 (0·160 to 0·226)	0·136 (0·103 to 0·174)	-27·7 (-43·4 to -9·85)	0·122 (0·104 to 0·147)
	5-14 years	0·697 (0·461 to 1·01)	0·606 (0·395 to 0·913)	-12·8 (-23·7 to 1·43)	0·557 (0·369 to 0·807)	0·0474 (0·0366 to 0·0613)	0·0321 (0·0236 to 0·0419)	-32·1 (-42·8 to -15·6)	0·0308 (0·0238 to 0·0398)
	15-49 years	5·29 (4·27 to 6·59)	4·87 (3·99 to 6·08)	-7·96 (-16·2 to -0·608)	4·23 (3·42 to 5·27)	7·92 (6·41 to 9·53)	5·39 (4·12 to 7·07)	-31·9 (-42·7 to -15·6)	5·15 (4·16 to 6·20)
	50-69 years	10·2 (7·54 to 13·4)	8·93 (6·80 to 11·4)	-12·1 (-19·4 to -2·93)	8·14 (6·03 to 10·8)	17·8 (14·2 to 22·3)	12·2 (9·11 to 16·2)	-30·9 (-46·2 to -13·3)	11·6 (9·25 to 14·5)
	70+ years	12·2 (9·83 to 15·2)	10·5 (8·53 to 13·0)	-13·5 (-22·1 to -6·96)	9·73 (7·86 to 12·1)	14·3 (11·7 to 17·1)	13·9 (11·2 to 17·7)	-2·12 (-16·6 to 17·9)	9·27 (7·60 to 11·1)
Slovenia	All Ages	6·82 (5·94 to 7·71)	5·96 (5·12 to 6·84)	-12·6 (-17·0 to -6·67)	5·46 (4·75 to 6·16)	15·2 (14·0 to 16·3)	7·54 (6·50 to 8·39)	-50·3 (-56·0 to -43·7)	9·87 (9·11 to 10·6)
	Under 5	1·78 (1·30 to 2·36)	1·60 (1·21 to 2·12)	-9·87 (-23·4 to 4·41)	1·42 (1·04 to 1·89)	0·0286 (0·0238 to 0·0337)	0·0106 (0·00873 to 0·0127)	-62·7 (-67·3 to -57·1)	0·0186 (0·0155 to 0·0219)
	5-14 years	1·59 (1·04 to 2·35)	1·46 (0·967 to 2·21)	-7·88 (-18·8 to 1·91)	1·27 (0·831 to 1·88)	0·0269 (0·0202 to 0·0366)	0·0143 (0·00983 to 0·0205)	-47·1 (-51·6 to -42·4)	0·0175 (0·0131 to 0·0238)

eTable 3. Percent change from 2015 to 2021 in age-standardised all-form tuberculosis incidence rate per 100,000 population and in deaths due to all-form tuberculosis by age for 204 countries and territories.

Location	Age group	2015 Rate	2021 Rate	Incidence Percent Change	Incidence Milestone	2015 Deaths	2021 Deaths	Mortality Percent Change	Mortality Milestone
	15-49 years	5·34 (4·31 to 6·79)	4·58 (3·71 to 5·64)	-14·2 (-20·1 to -6·69)	4·27 (3·45 to 5·44)	2·10 (1·94 to 2·30)	0·693 (0·602 to 0·793)	-67·0 (-71·6 to -61·8)	1·37 (1·26 to 1·50)
	50-69 years	8·67 (6·62 to 11·0)	7·44 (5·65 to 9·43)	-14·1 (-22·6 to -5·59)	6·93 (5·30 to 8·77)	5·51 (5·14 to 5·96)	2·14 (1·83 to 2·42)	-61·1 (-67·4 to -55·7)	3·58 (3·34 to 3·87)
	70+ years	14·1 (11·0 to 17·0)	11·8 (9·00 to 14·4)	-16·3 (-24·5 to -4·32)	11·3 (8·78 to 13·6)	7·53 (6·70 to 8·16)	4·69 (3·98 to 5·19)	-37·7 (-44·0 to -29·3)	4·89 (4·36 to 5·31)
Eastern Europe	All Ages	86·8 (73·9 to 105)	70·9 (59·7 to 87·3)	-18·4 (-23·2 to -14·0)	69·4 (59·1 to 84·1)	17700 (16800 to 19100)	9560 (8510 to 10700)	-46·0 (-50·7 to -40·4)	11500 (10900 to 12400)
	Under 5	10·4 (8·18 to 13·6)	6·25 (5·02 to 8·10)	-39·6 (-43·9 to -35·7)	8·29 (6·54 to 10·9)	106 (95·2 to 119)	31·9 (27·3 to 37·8)	-69·8 (-72·0 to -67·6)	68·7 (61·9 to 77·4)
	5-14 years	14·9 (9·79 to 21·2)	11·3 (7·39 to 16·8)	-24·2 (-31·6 to -17·5)	11·9 (7·84 to 17·0)	22·6 (19·0 to 27·4)	13·3 (10·9 to 16·4)	-41·2 (-44·0 to -38·5)	14·7 (12·3 to 17·8)
	15-49 years	124 (98·3 to 156)	104 (81·6 to 130)	-16·6 (-22·0 to -10·7)	99·4 (78·7 to 124)	8520 (7680 to 9710)	4370 (3760 to 5030)	-48·7 (-52·4 to -44·8)	5540 (4990 to 6310)
	50-69 years	80·3 (60·0 to 103)	65·9 (48·7 to 88·7)	-18·0 (-24·3 to -11·4)	64·2 (48·0 to 82·2)	7730 (7510 to 7950)	4070 (3600 to 4680)	-47·3 (-53·5 to -39·6)	5020 (4880 to 5170)
	70+ years	43·2 (32·0 to 55·1)	36·7 (28·1 to 47·3)	-15·0 (-21·8 to -7·31)	34·6 (25·6 to 44·1)	1320 (1260 to 1360)	1070 (986 to 1160)	-18·5 (-23·7 to -12·7)	856 (819 to 882)
Belarus	All Ages	35·2 (30·3 to 41·0)	26·7 (22·4 to 31·7)	-23·9 (-30·3 to -17·9)	28·1 (24·2 to 32·8)	391 (362 to 428)	245 (205 to 295)	-37·3 (-49·1 to -26·3)	254 (235 to 278)
	Under 5	4·09 (3·26 to 5·51)	3·37 (2·56 to 4·38)	-17·5 (-29·3 to -5·44)	3·27 (2·61 to 4·41)	2·24 (1·86 to 2·71)	0·834 (0·656 to 1·12)	-62·7 (-69·0 to -54·3)	1·46 (1·21 to 1·76)
	5-14 years	2·65 (1·76 to 3·87)	2·69 (1·71 to 3·91)	1·84 (-10·4 to 19·2)	2·12 (1·41 to 3·10)	0·463 (0·384 to 0·577)	0·326 (0·262 to 0·420)	-29·7 (-36·4 to -22·5)	0·301 (0·250 to 0·375)
	15-49 years	43·8 (36·3 to 54·7)	34·2 (26·9 to 43·2)	-21·8 (-29·7 to -13·9)	35·1 (29·1 to 43·8)	162 (147 to 181)	88·3 (70·5 to 104)	-45·4 (-55·2 to -37·1)	105 (95·6 to 118)
	50-69 years	41·9 (31·4 to 53·1)	31·0 (23·2 to 39·2)	-25·9 (-34·6 to -18·9)	33·5 (25·1 to 42·5)	188 (173 to 208)	127 (102 to 155)	-32·2 (-47·1 to -17·8)	122 (112 to 135)
	70+ years	27·7 (22·3 to 35·3)	21·4 (16·8 to 27·0)	-22·5 (-32·0 to -12·7)	22·2 (17·8 to 28·3)	39·0 (36·3 to 41·7)	28·6 (24·3 to 33·3)	-26·5 (-37·1 to -13·4)	25·4 (23·6 to 27·1)
Estonia	All Ages	18·7 (16·0 to 22·0)	15·4 (13·3 to 18·0)	-17·7 (-22·0 to -12·7)	14·9 (12·8 to 17·6)	45·3 (41·4 to 48·9)	25·3 (21·9 to 29·1)	-44·2 (-49·7 to -37·4)	29·5 (26·9 to 31·8)
	Under 5	4·08 (3·20 to 5·27)	3·24 (2·55 to 4·12)	-20·7 (-31·3 to -7·58)	3·27 (2·56 to 4·22)	0·224 (0·183 to 0·259)	0·0958 (0·0752 to 0·129)	-57·3 (-63·0 to -48·1)	0·146 (0·119 to 0·168)
	5-14 years	4·36 (2·94 to 6·63)	3·89 (2·58 to 5·95)	-10·7 (-21·3 to 4·21)	3·49 (2·36 to 5·30)	0·0961 (0·0715 to 0·118)	0·0468 (0·0313 to 0·0684)	-51·6 (-56·3 to -40·7)	0·0625 (0·0465 to 0·0764)
	15-49 years	22·0 (18·1 to 27·7)	18·6 (15·0 to 23·6)	-15·4 (-22·6 to -8·20)	17·6 (14·5 to 22·2)	18·9 (15·4 to 22·0)	9·58 (7·32 to 12·8)	-49·5 (-54·4 to -41·8)	12·3 (10·0 to 14·3)
	50-69 years	22·7 (17·3 to 28·1)	18·2 (13·3 to 23·6)	-20·1 (-26·5 to -12·6)	18·2 (13·9 to 22·5)	18·1 (16·7 to 19·4)	10·3 (9·05 to 11·9)	-42·8 (-49·5 to -34·7)	11·8 (10·9 to 12·6)
	70+ years	17·1 (13·2 to 21·4)	14·1 (11·3 to 17·5)	-17·3 (-26·7 to -7·09)	13·7 (10·6 to 17·2)	8·00 (7·21 to 8·65)	5·24 (4·61 to 6·03)	-34·5 (-42·1 to -26·3)	5·20 (4·68 to 5·62)
Latvia	All Ages	34·6 (30·2 to 40·3)	27·5 (23·4 to 32·0)	-20·5 (-25·9 to -15·0)	27·7 (24·2 to 32·2)	95·1 (85·4 to 106)	55·2 (48·6 to 62·7)	-41·9 (-46·9 to -35·6)	61·8 (55·5 to 68·7)

eTable 3. Percent change from 2015 to 2021 in age-standardised all-form tuberculosis incidence rate per 100,000 population and in deaths due to all-form tuberculosis by age for 204 countries and territories.

Location	Age group	2015 Rate	2021 Rate	Incidence Percent Change	Incidence Milestone	2015 Deaths	2021 Deaths	Mortality Percent Change	Mortality Milestone
	Under 5	6·76 (5·24 to 8·90)	4·78 (3·77 to 6·14)	-29·0 (-41·1 to -17·0)	5·41 (4·19 to 7·12)	0·465 (0·373 to 0·580)	0·179 (0·140 to 0·231)	-61·5 (-64·9 to -56·7)	0·302 (0·242 to 0·377)
	5-14 years	8·12 (5·69 to 11·9)	5·85 (3·91 to 8·56)	-27·8 (-37·7 to -16·4)	6·49 (4·55 to 9·50)	0·166 (0·120 to 0·227)	0·101 (0·0659 to 0·152)	-39·4 (-46·5 to -32·3)	0·108 (0·0780 to 0·148)
	15-49 years	46·1 (37·6 to 57·0)	37·3 (30·1 to 46·5)	-19·2 (-25·0 to -12·1)	36·9 (30·1 to 45·6)	36·0 (30·4 to 43·9)	20·7 (16·5 to 26·1)	-42·6 (-47·3 to -36·9)	23·4 (19·7 to 28·5)
	50-69 years	37·7 (28·0 to 48·4)	30·1 (22·1 to 37·9)	-20·1 (-28·3 to -10·6)	30·1 (22·4 to 38·7)	43·3 (39·5 to 47·5)	23·8 (21·4 to 27·2)	-44·9 (-50·9 to -37·0)	28·1 (25·6 to 30·9)
	70+ years	21·5 (16·9 to 27·2)	19·1 (15·1 to 24·3)	-10·7 (-21·9 to 2·61)	17·2 (13·5 to 21·8)	15·2 (14·0 to 16·3)	10·5 (9·32 to 11·6)	-31·3 (-39·4 to -23·9)	9·91 (9·11 to 10·6)
Lithuania	All Ages	47·3 (41·2 to 55·5)	38·0 (33·0 to 44·0)	-19·6 (-24·3 to -13·9)	37·9 (32·9 to 44·4)	210 (198 to 224)	98·3 (87·9 to 111)	-53·1 (-58·2 to -47·8)	136 (129 to 146)
	Under 5	8·61 (6·68 to 11·4)	6·00 (4·66 to 8·00)	-30·3 (-39·7 to -17·9)	6·89 (5·35 to 9·09)	0·843 (0·725 to 0·977)	0·221 (0·187 to 0·269)	-73·7 (-77·6 to -69·4)	0·548 (0·471 to 0·635)
	5-14 years	12·5 (8·93 to 17·2)	8·95 (6·08 to 13·3)	-28·5 (-37·7 to -18·7)	10·0 (7·15 to 13·7)	0·218 (0·178 to 0·263)	0·116 (0·0876 to 0·159)	-46·7 (-52·5 to -38·3)	0·141 (0·115 to 0·171)
	15-49 years	55·4 (45·7 to 69·3)	46·2 (38·1 to 56·9)	-16·6 (-22·1 to -10·8)	44·3 (36·5 to 55·4)	69·5 (64·8 to 75·1)	31·9 (28·0 to 36·6)	-54·1 (-58·8 to -49·3)	45·2 (42·1 to 48·8)
	50-69 years	59·4 (44·4 to 74·0)	46·6 (35·7 to 58·3)	-21·4 (-29·3 to -13·7)	47·5 (35·5 to 59·2)	102 (94·1 to 109)	44·3 (39·5 to 50·8)	-56·4 (-62·3 to -50·8)	66·2 (61·2 to 70·9)
	70+ years	36·2 (29·0 to 45·1)	28·6 (22·7 to 35·8)	-20·8 (-30·2 to -10·4)	29·0 (23·2 to 36·1)	37·2 (34·0 to 40·4)	21·7 (19·0 to 24·2)	-41·7 (-49·0 to -34·9)	24·2 (22·1 to 26·3)
Republic of Moldova	All Ages	86·3 (74·8 to 102)	65·4 (55·2 to 76·1)	-24·2 (-30·2 to -17·5)	69·1 (59·8 to 81·3)	276 (256 to 293)	138 (124 to 154)	-50·0 (-54·9 to -45·0)	180 (166 to 190)
	Under 5	13·1 (10·0 to 17·7)	9·17 (7·13 to 11·8)	-29·8 (-40·5 to -16·8)	10·5 (8·01 to 14·1)	1·80 (1·42 to 2·19)	0·800 (0·615 to 1·02)	-55·5 (-63·3 to -47·7)	1·17 (0·921 to 1·43)
	5-14 years	17·9 (12·2 to 24·6)	13·8 (9·72 to 20·3)	-22·8 (-33·0 to -7·95)	14·3 (9·73 to 19·7)	0·409 (0·335 to 0·501)	0·207 (0·163 to 0·266)	-49·4 (-54·2 to -44·6)	0·266 (0·218 to 0·326)
	15-49 years	108 (91·4 to 136)	83·9 (66·6 to 103)	-22·5 (-29·9 to -13·9)	86·6 (73·1 to 109)	114 (104 to 124)	53·2 (46·9 to 60·7)	-53·5 (-58·0 to -48·8)	74·4 (67·6 to 80·7)
	50-69 years	96·5 (72·4 to 122)	70·0 (54·5 to 90·0)	-27·3 (-37·3 to -19·0)	77·2 (57·9 to 97·8)	138 (127 to 149)	67·0 (59·2 to 75·9)	-51·5 (-56·6 to -45·1)	89·8 (82·5 to 96·6)
	70+ years	48·1 (38·5 to 61·5)	37·5 (29·1 to 46·4)	-21·8 (-31·5 to -11·2)	38·4 (30·8 to 49·2)	21·6 (19·8 to 23·3)	17·0 (15·3 to 18·7)	-20·9 (-29·7 to -13·1)	14·0 (12·9 to 15·2)
Russian Federation	All Ages	89·2 (76·5 to 108)	73·6 (61·4 to 91·5)	-17·5 (-22·5 to -12·0)	71·4 (61·2 to 86·6)	12600 (11900 to 13400)	6140 (5610 to 6840)	-51·1 (-54·2 to -48·0)	8160 (7740 to 8730)
	Under 5	11·3 (8·88 to 14·9)	6·37 (5·06 to 8·25)	-43·6 (-48·6 to -39·0)	9·03 (7·11 to 11·9)	83·9 (75·5 to 95·1)	23·2 (20·0 to 27·8)	-72·4 (-74·4 to -70·0)	54·5 (49·1 to 61·8)
	5-14 years	16·8 (11·0 to 23·8)	12·4 (7·97 to 18·2)	-26·6 (-34·7 to -18·3)	13·4 (8·79 to 19·0)	17·1 (14·1 to 21·0)	9·67 (7·85 to 12·0)	-43·4 (-45·7 to -41·6)	11·1 (9·18 to 13·6)
	15-49 years	130 (102 to 162)	109 (84·7 to 138)	-15·8 (-22·0 to -8·92)	104 (82·0 to 129)	6320 (5760 to 7130)	2930 (2590 to 3440)	-53·7 (-56·1 to -51·1)	4110 (3740 to 4630)
	50-69 years	79·5 (59·2 to 103)	67·1 (49·1 to 91·4)	-15·7 (-23·5 to -8·12)	63·6 (47·4 to 82·1)	5250 (5170 to 5370)	2410 (2220 to 2670)	-54·1 (-58·0 to -50·1)	3420 (3360 to 3490)

eTable 3. Percent change from 2015 to 2021 in age-standardised all-form tuberculosis incidence rate per 100,000 population and in deaths due to all-form tuberculosis by age for 204 countries and territories.

Location	Age group	2015 Rate	2021 Rate	Incidence Percent Change	Incidence Milestone	2015 Deaths	2021 Deaths	Mortality Percent Change	Mortality Milestone
	70+ years	43·1 (31·6 to 54·2)	37·4 (28·1 to 48·2)	-13·1 (-21·7 to -3·86)	34·5 (25·3 to 43·4)	879 (834 to 903)	766 (718 to 818)	-12·9 (-17·2 to -6·88)	571 (542 to 587)
Ukraine	All Ages	96·7 (81·2 to 119)	77·2 (65·2 to 92·7)	-20·1 (-25·5 to -14·1)	77·3 (65·0 to 95·0)	4120 (3780 to 4600)	2860 (2090 to 3810)	-30·6 (-47·8 to -11·4)	2680 (2460 to 2990)
	Under 5	8·42 (6·51 to 11·2)	6·47 (5·03 to 8·72)	-23·0 (-32·0 to -13·2)	6·73 (5·21 to 8·96)	16·2 (14·5 to 18·2)	6·61 (5·43 to 8·23)	-59·2 (-64·5 to -53·3)	10·5 (9·45 to 11·9)
	5-14 years	11·4 (7·36 to 16·9)	9·85 (6·15 to 14·7)	-14·0 (-24·5 to -1·38)	9·15 (5·88 to 13·6)	4·20 (3·62 to 4·91)	2·87 (2·33 to 3·51)	-31·9 (-40·8 to -21·6)	2·73 (2·35 to 3·19)
	15-49 years	135 (108 to 174)	110 (88·4 to 140)	-18·9 (-26·2 to -9·24)	108 (86·8 to 140)	1800 (1540 to 2150)	1240 (897 to 1590)	-31·2 (-44·4 to -17·3)	1170 (1000 to 1400)
	50-69 years	94·5 (65·9 to 125)	73·5 (52·0 to 99·1)	-22·1 (-29·7 to -11·7)	75·6 (52·7 to 99·8)	1990 (1810 to 2160)	1390 (972 to 1970)	-30·1 (-50·4 to -5·08)	1290 (1180 to 1400)
	70+ years	49·2 (36·2 to 65·5)	40·2 (29·7 to 51·6)	-18·1 (-26·4 to -7·33)	39·4 (29·0 to 52·4)	318 (295 to 340)	226 (171 to 286)	-28·9 (-45·4 to -10·2)	206 (192 to 221)
High-income	All Ages	10·3 (9·20 to 11·7)	9·10 (8·05 to 10·4)	-11·4 (-13·5 to -9·32)	8·21 (7·36 to 9·37)	13200 (11800 to 14300)	12700 (11200 to 13900)	-4·20 (-6·77 to -1·81)	8590 (7690 to 9300)
	Under 5	2·19 (1·73 to 2·81)	1·73 (1·34 to 2·20)	-21·3 (-24·4 to -18·5)	1·76 (1·38 to 2·25)	39·6 (33·6 to 45·4)	24·2 (20·0 to 28·4)	-38·9 (-43·2 to -34·8)	25·7 (21·9 to 29·5)
	5-14 years	2·23 (1·52 to 3·24)	1·94 (1·30 to 2·81)	-13·1 (-16·8 to -9·23)	1·78 (1·22 to 2·59)	24·6 (20·7 to 28·1)	19·2 (16·3 to 22·1)	-21·7 (-26·1 to -17·2)	16·0 (13·5 to 18·3)
	15-49 years	9·96 (8·10 to 12·4)	8·45 (6·91 to 10·5)	-15·2 (-17·9 to -12·5)	7·96 (6·48 to 9·88)	1800 (1440 to 2120)	1470 (1170 to 1780)	-18·1 (-20·8 to -15·1)	1170 (935 to 1380)
	50-69 years	11·3 (8·67 to 13·9)	9·94 (7·58 to 12·4)	-12·4 (-15·4 to -9·45)	9·07 (6·93 to 11·2)	2700 (2490 to 2920)	2330 (2090 to 2520)	-13·8 (-16·6 to -11·2)	1750 (1620 to 1900)
	70+ years	20·4 (17·0 to 24·7)	17·9 (14·8 to 22·1)	-11·9 (-16·7 to -8·31)	16·3 (13·6 to 19·8)	8660 (7320 to 9370)	8820 (7290 to 9740)	1·87 (-0·860 to 5·05)	5630 (4760 to 6090)
Australasia	All Ages	5·62 (4·86 to 6·53)	5·61 (4·89 to 6·56)	-0·131 (-4·50 to 4·75)	4·50 (3·89 to 5·22)	93·8 (84·7 to 101)	83·2 (74·8 to 90·9)	-11·3 (-15·8 to -6·89)	61·0 (55·1 to 65·7)
	Under 5	2·23 (1·73 to 2·92)	2·12 (1·62 to 2·78)	-4·92 (-14·7 to 5·18)	1·79 (1·39 to 2·34)	0·278 (0·254 to 0·306)	0·165 (0·141 to 0·189)	-40·5 (-48·0 to -31·7)	0·181 (0·165 to 0·199)
	5-14 years	1·20 (0·812 to 1·72)	1·32 (0·880 to 1·94)	10·3 (1·36 to 19·6)	0·957 (0·650 to 1·37)	0·166 (0·145 to 0·189)	0·111 (0·0928 to 0·138)	-32·7 (-40·0 to -26·2)	0·108 (0·0945 to 0·123)
	15-49 years	7·36 (6·01 to 9·21)	7·38 (6·00 to 8·99)	0·343 (-5·86 to 7·20)	5·89 (4·81 to 7·37)	7·82 (6·73 to 9·43)	6·16 (5·09 to 7·55)	-21·3 (-25·8 to -17·8)	5·08 (4·37 to 6·13)
	50-69 years	4·77 (3·67 to 6·03)	4·78 (3·58 to 6·06)	0·139 (-6·09 to 8·61)	3·82 (2·94 to 4·82)	21·8 (20·3 to 23·9)	18·1 (16·3 to 19·9)	-17·3 (-22·1 to -11·9)	14·2 (13·2 to 15·5)
	70+ years	7·08 (5·69 to 8·72)	6·59 (5·29 to 8·17)	-6·86 (-15·0 to 4·42)	5·66 (4·55 to 6·98)	63·7 (55·4 to 69·1)	58·7 (50·6 to 64·2)	-7·89 (-12·9 to -2·85)	41·4 (36·0 to 44·9)
Australia	All Ages	5·60 (4·84 to 6·45)	5·36 (4·69 to 6·22)	-4·31 (-9·22 to 0·471)	4·48 (3·87 to 5·16)	77·2 (69·5 to 83·9)	77·5 (69·4 to 84·8)	0·273 (-4·77 to 5·37)	50·2 (45·2 to 54·5)
	Under 5	2·04 (1·56 to 2·72)	1·93 (1·45 to 2·54)	-5·20 (-16·2 to 7·47)	1·63 (1·25 to 2·17)	0·219 (0·197 to 0·244)	0·139 (0·117 to 0·161)	-36·6 (-45·1 to -26·2)	0·142 (0·128 to 0·159)
	5-14 years	0·996 (0·674 to 1·42)	1·02 (0·669 to 1·49)	2·18 (-7·44 to 14·1)	0·797 (0·539 to 1·14)	0·138 (0·121 to 0·159)	0·101 (0·0841 to 0·125)	-26·8 (-35·7 to -19·4)	0·0899 (0·0784 to 0·103)

eTable 3. Percent change from 2015 to 2021 in age-standardised all-form tuberculosis incidence rate per 100,000 population and in deaths due to all-form tuberculosis by age for 204 countries and territories.

Location	Age group	2015 Rate	2021 Rate	Incidence Percent Change	Incidence Milestone	2015 Deaths	2021 Deaths	Mortality Percent Change	Mortality Milestone
	15-49 years	7·34 (5·97 to 9·12)	6·89 (5·67 to 8·34)	-6·16 (-13·3 to 0·429)	5·87 (4·77 to 7·30)	6·12 (5·25 to 7·36)	5·58 (4·63 to 6·85)	-8·88 (-13·7 to -4·88)	3·98 (3·41 to 4·78)
	50-69 years	4·82 (3·65 to 6·09)	4·74 (3·57 to 5·95)	-1·55 (-8·49 to 7·46)	3·85 (2·92 to 4·88)	17·1 (15·7 to 18·8)	16·4 (14·7 to 18·1)	-4·08 (-9·81 to 2·27)	11·1 (10·2 to 12·2)
	70+ years	7·13 (5·68 to 8·82)	6·82 (5·50 to 8·45)	-4·22 (-13·5 to 9·31)	5·71 (4·54 to 7·06)	53·7 (46·7 to 58·7)	55·2 (47·5 to 60·4)	2·93 (-3·05 to 8·97)	34·9 (30·3 to 38·2)
New Zealand	All Ages	5·72 (4·96 to 6·73)	6·88 (5·84 to 8·39)	20·3 (10·4 to 30·2)	4·57 (3·97 to 5·38)	16·6 (15·3 to 17·6)	5·71 (5·22 to 6·19)	-65·5 (-67·7 to -63·7)	10·8 (9·93 to 11·5)
	Under 5	3·21 (2·53 to 4·15)	3·04 (2·30 to 4·02)	-5·38 (-13·6 to 4·84)	2·57 (2·02 to 3·32)	0·0588 (0·0514 to 0·0656)	0·0263 (0·0223 to 0·0304)	-55·1 (-62·2 to -46·7)	0·0382 (0·0334 to 0·0427)
	5-14 years	2·15 (1·48 to 3·09)	2·79 (1·84 to 4·22)	29·7 (16·8 to 42·5)	1·72 (1·18 to 2·47)	0·0272 (0·0242 to 0·0310)	0·0102 (0·00865 to 0·0123)	-62·5 (-67·2 to -58·0)	0·0177 (0·0157 to 0·0201)
	15-49 years	7·43 (6·06 to 9·33)	9·82 (7·53 to 12·5)	32·0 (18·9 to 47·7)	5·95 (4·85 to 7·46)	1·70 (1·47 to 2·03)	0·578 (0·470 to 0·724)	-66·1 (-69·1 to -63·8)	1·10 (0·953 to 1·32)
	50-69 years	4·53 (3·38 to 5·84)	4·95 (3·60 to 6·70)	9·12 (-2·23 to 20·1)	3·63 (2·71 to 4·67)	4·75 (4·49 to 5·02)	1·68 (1·54 to 1·79)	-64·7 (-67·0 to -62·0)	3·09 (2·92 to 3·26)
	70+ years	6·79 (5·29 to 8·39)	5·29 (4·00 to 6·86)	-22·1 (-32·7 to -10·8)	5·43 (4·23 to 6·71)	10·0 (8·94 to 10·9)	3·42 (2·95 to 3·77)	-65·8 (-68·3 to -63·4)	6·51 (5·81 to 7·07)
High-income Asia Pacific	All Ages	29·1 (25·3 to 33·1)	25·8 (22·3 to 29·0)	-11·3 (-14·7 to -7·98)	23·3 (20·3 to 26·5)	6210 (5260 to 6870)	6460 (5280 to 7290)	3·99 (-1·02 to 8·97)	4040 (3420 to 4460)
	Under 5	1·06 (0·827 to 1·34)	0·894 (0·679 to 1·15)	-15·3 (-21·9 to -9·17)	0·844 (0·662 to 1·08)	7·34 (6·38 to 8·13)	4·18 (3·66 to 4·57)	-42·9 (-46·8 to -38·0)	4·77 (4·15 to 5·28)
	5-14 years	1·07 (0·730 to 1·56)	0·862 (0·563 to 1·32)	-19·5 (-26·7 to -11·1)	0·855 (0·584 to 1·25)	3·26 (2·92 to 3·67)	2·54 (2·20 to 2·80)	-22·0 (-30·1 to -13·2)	2·12 (1·90 to 2·38)
	15-49 years	19·9 (16·5 to 24·3)	15·3 (12·7 to 19·2)	-23·3 (-27·4 to -19·3)	15·9 (13·2 to 19·4)	252 (229 to 281)	170 (154 to 188)	-32·2 (-38·1 to -26·8)	164 (149 to 183)
	50-69 years	37·6 (29·2 to 47·0)	32·4 (25·1 to 41·0)	-13·7 (-18·7 to -8·92)	30·1 (23·4 to 37·6)	827 (766 to 919)	660 (590 to 742)	-20·2 (-27·7 to -13·3)	538 (498 to 598)
	70+ years	65·2 (54·0 to 80·1)	56·0 (45·7 to 68·8)	-14·0 (-20·6 to -9·44)	52·1 (43·2 to 64·1)	5120 (4220 to 5700)	5620 (4510 to 6380)	9·80 (4·82 to 15·1)	3330 (2740 to 3700)
Brunei Darussalam	All Ages	60·3 (54·0 to 69·4)	55·1 (48·0 to 63·9)	-8·51 (-13·8 to -2·16)	48·2 (43·2 to 55·5)	18·0 (15·7 to 21·5)	17·6 (14·6 to 21·0)	-1·93 (-15·9 to 18·5)	11·7 (10·2 to 14·0)
	Under 5	4·45 (3·55 to 5·82)	3·71 (2·86 to 4·74)	-16·6 (-29·9 to -5·85)	3·56 (2·84 to 4·66)	0·168 (0·137 to 0·218)	0·127 (0·0967 to 0·171)	-24·2 (-39·7 to -1·87)	0·109 (0·0894 to 0·142)
	5-14 years	5·23 (3·39 to 7·77)	4·51 (2·86 to 6·51)	-13·8 (-26·7 to -1·36)	4·19 (2·71 to 6·22)	0·103 (0·0853 to 0·125)	0·0639 (0·0527 to 0·0795)	-37·9 (-50·6 to -24·2)	0·0672 (0·0554 to 0·0816)
	15-49 years	56·1 (46·7 to 68·6)	47·1 (38·9 to 58·1)	-16·1 (-21·3 to -8·49)	44·9 (37·3 to 54·9)	3·61 (3·09 to 4·33)	3·36 (2·70 to 4·07)	-6·89 (-20·0 to 16·8)	2·35 (2·01 to 2·81)
	50-69 years	125 (99·7 to 155)	110 (84·4 to 134)	-12·2 (-20·6 to -5·29)	100 (79·7 to 124)	4·69 (3·85 to 5·64)	5·00 (4·01 to 6·29)	7·05 (-10·1 to 27·8)	3·05 (2·51 to 3·66)
	70+ years	342 (269 to 436)	280 (211 to 356)	-18·1 (-27·3 to -6·70)	274 (215 to 349)	9·45 (7·98 to 11·4)	9·09 (7·19 to 11·1)	-3·54 (-20·6 to 16·4)	6·14 (5·19 to 7·40)
Japan	All Ages	14·4 (12·4 to 16·4)	12·3 (10·7 to 14·1)	-14·8 (-18·8 to -10·5)	11·5 (9·96 to 13·1)	3450 (2880 to 3800)	3880 (3140 to 4340)	12·4 (8·86 to 14·9)	2250 (1870 to 2470)

eTable 3. Percent change from 2015 to 2021 in age-standardised all-form tuberculosis incidence rate per 100,000 population and in deaths due to all-form tuberculosis by age for 204 countries and territories.

Location	Age group	2015 Rate	2021 Rate	Incidence Percent Change	Incidence Milestone	2015 Deaths	2021 Deaths	Mortality Percent Change	Mortality Milestone
	Under 5	0·573 (0·436 to 0·747)	0·592 (0·440 to 0·771)	3·27 (-5·34 to 8·91)	0·458 (0·349 to 0·597)	3·08 (2·35 to 3·70)	2·59 (2·07 to 2·87)	-15·7 (-22·8 to -7·68)	2·00 (1·53 to 2·41)
	5-14 years	0·382 (0·234 to 0·573)	0·424 (0·257 to 0·674)	10·8 (1·41 to 21·5)	0·306 (0·187 to 0·458)	0·913 (0·698 to 1·11)	0·863 (0·678 to 0·986)	-5·15 (-11·6 to -0·673)	0·593 (0·454 to 0·723)
	15-49 years	7·11 (5·85 to 8·91)	5·84 (4·75 to 7·49)	-17·9 (-24·0 to -11·4)	5·69 (4·68 to 7·13)	55·2 (49·6 to 60·9)	43·2 (38·7 to 46·6)	-21·7 (-23·6 to -20·0)	35·9 (32·2 to 39·6)
	50-69 years	16·2 (12·6 to 20·7)	11·2 (8·40 to 14·5)	-31·1 (-35·5 to -27·0)	13·0 (10·1 to 16·6)	279 (260 to 301)	211 (196 to 224)	-24·4 (-26·0 to -22·3)	182 (169 to 195)
	70+ years	37·7 (31·3 to 45·6)	31·2 (25·5 to 39·1)	-17·2 (-21·9 to -12·1)	30·2 (25·0 to 36·5)	3120 (2540 to 3450)	3630 (2880 to 4070)	16·3 (13·3 to 18·9)	2020 (1650 to 2240)
Republic of Korea	All Ages	64·2 (56·9 to 73·3)	57·8 (49·5 to 65·1)	-9·98 (-14·6 to -5·17)	51·4 (45·5 to 58·6)	2670 (2290 to 3030)	2500 (2080 to 2940)	-6·19 (-16·9 to 3·74)	1730 (1490 to 1970)
	Under 5	2·01 (1·55 to 2·59)	1·62 (1·19 to 2·06)	-19·5 (-29·6 to -8·89)	1·61 (1·24 to 2·07)	3·83 (3·33 to 4·29)	1·30 (1·13 to 1·48)	-66·0 (-71·6 to -59·6)	2·49 (2·17 to 2·79)
	5-14 years	2·55 (1·78 to 3·60)	1·78 (1·22 to 2·57)	-30·2 (-39·3 to -21·2)	2·04 (1·42 to 2·88)	2·07 (1·75 to 2·42)	1·48 (1·21 to 1·72)	-28·4 (-41·2 to -14·8)	1·35 (1·14 to 1·57)
	15-49 years	43·5 (36·0 to 52·8)	32·8 (27·1 to 41·2)	-24·5 (-30·3 to -19·5)	34·8 (28·8 to 42·3)	185 (163 to 214)	119 (104 to 135)	-35·7 (-43·1 to -28·1)	121 (106 to 139)
	50-69 years	90·0 (69·4 to 113)	74·8 (57·8 to 95·8)	-16·9 (-24·3 to -10·4)	72·0 (55·5 to 90·0)	522 (461 to 618)	430 (360 to 510)	-17·6 (-29·3 to -6·06)	339 (300 to 402)
	70+ years	211 (172 to 264)	181 (144 to 221)	-14·1 (-22·6 to -5·67)	168 (138 to 212)	1960 (1630 to 2280)	1950 (1590 to 2330)	-0·108 (-13·1 to 13·3)	1270 (1060 to 1480)
Singapore	All Ages	41·8 (36·6 to 49·3)	36·9 (32·1 to 42·6)	-11·5 (-17·0 to -4·29)	33·4 (29·3 to 39·5)	70·2 (64·3 to 76·3)	56·2 (50·9 to 62·3)	-19·9 (-24·6 to -15·2)	45·6 (41·8 to 49·6)
	Under 5	1·83 (1·39 to 2·35)	1·51 (1·05 to 1·98)	-17·7 (-27·9 to -6·47)	1·47 (1·11 to 1·88)	0·262 (0·218 to 0·333)	0·175 (0·142 to 0·231)	-33·2 (-42·8 to -23·5)	0·171 (0·142 to 0·217)
	5-14 years	1·89 (1·24 to 2·81)	1·55 (1·07 to 2·25)	-17·9 (-30·8 to -8·08)	1·52 (0·993 to 2·25)	0·173 (0·153 to 0·200)	0·136 (0·116 to 0·156)	-21·6 (-29·7 to -12·0)	0·113 (0·0997 to 0·130)
	15-49 years	35·0 (28·5 to 44·0)	29·5 (24·0 to 37·6)	-15·8 (-21·5 to -10·2)	28·0 (22·8 to 35·2)	7·31 (6·44 to 8·23)	4·79 (4·19 to 5·49)	-34·5 (-38·8 to -30·6)	4·75 (4·19 to 5·35)
	50-69 years	63·2 (48·6 to 78·0)	50·7 (38·9 to 63·8)	-19·7 (-26·4 to -11·3)	50·5 (38·9 to 62·4)	21·0 (18·4 to 23·8)	13·1 (11·7 to 14·7)	-37·3 (-42·9 to -31·1)	13·6 (12·0 to 15·5)
	70+ years	122 (96·4 to 148)	101 (78·7 to 127)	-17·0 (-24·7 to -5·76)	97·7 (77·1 to 118)	41·5 (36·3 to 46·0)	37·9 (32·5 to 42·8)	-8·46 (-14·4 to -2·46)	26·9 (23·6 to 29·9)
High-income North America	All Ages	2·74 (2·41 to 3·19)	2·92 (2·56 to 3·41)	6·87 (3·51 to 10·4)	2·19 (1·93 to 2·55)	950 (869 to 1050)	1080 (1000 to 1180)	13·6 (10·6 to 16·5)	617 (565 to 680)
	Under 5	1·27 (0·985 to 1·62)	1·06 (0·805 to 1·38)	-16·4 (-20·6 to -12·6)	1·02 (0·788 to 1·30)	3·73 (3·41 to 4·17)	3·28 (2·92 to 3·81)	-12·2 (-19·1 to -6·47)	2·42 (2·22 to 2·71)
	5-14 years	0·590 (0·397 to 0·858)	0·669 (0·448 to 0·990)	13·2 (3·85 to 20·7)	0·472 (0·317 to 0·687)	1·52 (1·35 to 1·78)	1·71 (1·53 to 2·02)	12·7 (9·45 to 17·8)	0·987 (0·875 to 1·16)
	15-49 years	2·76 (2·28 to 3·47)	2·86 (2·31 to 3·61)	3·49 (-2·68 to 9·20)	2·21 (1·82 to 2·78)	156 (130 to 194)	173 (148 to 210)	10·8 (7·03 to 15·5)	102 (84·7 to 126)
	50-69 years	3·28 (2·48 to 4·07)	3·43 (2·58 to 4·36)	4·51 (-0·919 to 9·47)	2·62 (1·98 to 3·26)	333 (298 to 377)	369 (335 to 415)	11·0 (8·42 to 14·6)	216 (193 to 245)

eTable 3. Percent change from 2015 to 2021 in age-standardised all-form tuberculosis incidence rate per 100,000 population and in deaths due to all-form tuberculosis by age for 204 countries and territories.

Location	Age group	2015 Rate	2021 Rate	Incidence Percent Change	Incidence Milestone	2015 Deaths	2021 Deaths	Mortality Percent Change	Mortality Milestone
	70+ years	4·94 (3·93 to 6·17)	5·31 (4·19 to 6·83)	7·57 (3·48 to 11·8)	3·95 (3·15 to 4·94)	456 (396 to 488)	531 (468 to 565)	16·6 (13·6 to 20·5)	296 (258 to 317)
Canada	All Ages	5·01 (4·53 to 5·79)	5·50 (4·84 to 6·33)	9·84 (5·16 to 15·5)	4·00 (3·62 to 4·63)	126 (112 to 142)	127 (116 to 144)	1·17 (-4·14 to 6·34)	81·7 (73·0 to 92·6)
	Under 5	1·74 (1·31 to 2·21)	1·70 (1·27 to 2·23)	-2·57 (-14·2 to 7·48)	1·39 (1·05 to 1·77)	0·324 (0·277 to 0·405)	0·273 (0·219 to 0·337)	-15·6 (-25·0 to -6·21)	0·210 (0·180 to 0·263)
	5-14 years	1·19 (0·783 to 1·71)	1·26 (0·838 to 1·84)	5·96 (-6·87 to 18·3)	0·952 (0·626 to 1·37)	0·167 (0·137 to 0·217)	0·173 (0·142 to 0·222)	3·42 (-3·55 to 11·6)	0·109 (0·0893 to 0·141)
	15-49 years	5·28 (4·39 to 6·49)	5·70 (4·59 to 7·05)	7·79 (1·22 to 13·9)	4·23 (3·51 to 5·19)	19·2 (15·0 to 25·6)	20·3 (16·0 to 26·3)	5·67 (-0·664 to 11·7)	12·5 (9·74 to 16·7)
	50-69 years	5·08 (3·78 to 6·33)	5·61 (4·28 to 7·02)	10·7 (2·93 to 20·6)	4·06 (3·02 to 5·07)	35·6 (29·9 to 42·2)	33·5 (28·5 to 39·6)	-5·87 (-12·2 to -0·00920)	23·1 (19·4 to 27·4)
	70+ years	9·26 (7·39 to 11·2)	9·83 (7·87 to 12·3)	6·23 (-3·82 to 18·9)	7·41 (5·91 to 8·95)	70·4 (62·2 to 76·7)	72·9 (62·9 to 80·1)	3·59 (-1·52 to 9·21)	45·7 (40·4 to 49·9)
Greenland	All Ages	130 (112 to 154)	98·9 (84·5 to 121)	-23·9 (-29·6 to -18·9)	104 (89·2 to 123)	2·71 (2·23 to 3·15)	2·47 (2·13 to 2·92)	-8·61 (-22·0 to 5·88)	1·76 (1·45 to 2·05)
	Under 5	13·9 (10·7 to 17·3)	11·0 (8·23 to 14·1)	-20·4 (-31·2 to -8·73)	11·1 (8·57 to 13·8)	0·0244 (0·0184 to 0·0311)	0·0161 (0·0121 to 0·0202)	-33·7 (-45·1 to -22·2)	0·0159 (0·0120 to 0·0202)
	5-14 years	25·2 (16·4 to 38·0)	16·1 (10·5 to 24·7)	-36·0 (-45·7 to -29·0)	20·1 (13·1 to 30·4)	0·00269 (0·00199 to 0·00376)	0·00209 (0·00156 to 0·00290)	-21·6 (-43·5 to -3·39)	0·00175 (0·00130 to 0·00244)
	15-49 years	132 (106 to 164)	85·7 (67·8 to 108)	-35·1 (-41·8 to -29·5)	106 (84·7 to 132)	0·418 (0·338 to 0·491)	0·314 (0·260 to 0·367)	-24·9 (-34·3 to -13·9)	0·272 (0·220 to 0·319)
	50-69 years	201 (148 to 257)	163 (120 to 212)	-18·7 (-27·7 to -8·56)	161 (118 to 206)	1·25 (1·02 to 1·46)	1·18 (0·965 to 1·38)	-5·83 (-19·8 to 8·78)	0·815 (0·663 to 0·947)
	70+ years	245 (190 to 312)	228 (181 to 292)	-6·58 (-17·6 to 3·80)	196 (152 to 250)	1·01 (0·775 to 1·27)	0·964 (0·770 to 1·28)	-4·13 (-27·4 to 22·9)	0·659 (0·504 to 0·822)
United States of America	All Ages	2·46 (2·16 to 2·89)	2·62 (2·30 to 3·07)	6·22 (2·28 to 10·5)	1·97 (1·73 to 2·31)	822 (752 to 901)	949 (881 to 1030)	15·5 (12·3 to 18·8)	534 (489 to 586)
	Under 5	1·22 (0·945 to 1·57)	0·997 (0·748 to 1·30)	-18·5 (-22·8 to -15·0)	0·978 (0·756 to 1·25)	3·38 (3·12 to 3·73)	2·99 (2·67 to 3·44)	-11·7 (-18·9 to -5·15)	2·20 (2·03 to 2·42)
	5-14 years	0·528 (0·349 to 0·773)	0·604 (0·403 to 0·898)	14·1 (3·36 to 23·0)	0·422 (0·279 to 0·618)	1·35 (1·20 to 1·56)	1·54 (1·38 to 1·79)	14·0 (10·4 to 19·3)	0·877 (0·783 to 1·01)
	15-49 years	2·47 (2·04 to 3·12)	2·54 (2·01 to 3·23)	2·73 (-4·77 to 9·74)	1·98 (1·63 to 2·49)	137 (115 to 168)	152 (131 to 183)	11·6 (7·37 to 16·7)	88·9 (75·0 to 109)
	50-69 years	3·03 (2·29 to 3·79)	3·14 (2·34 to 4·05)	3·50 (-2·65 to 8·47)	2·42 (1·83 to 3·03)	296 (265 to 333)	335 (305 to 375)	13·2 (10·1 to 16·7)	192 (172 to 217)
	70+ years	4·38 (3·48 to 5·51)	4·73 (3·72 to 6·14)	7·87 (3·82 to 12·5)	3·51 (2·78 to 4·41)	384 (332 to 412)	457 (402 to 488)	19·1 (15·8 to 23·3)	250 (216 to 268)
Southern Latin America	All Ages	20·0 (17·3 to 23·3)	18·8 (16·5 to 21·9)	-5·95 (-9·43 to -2·52)	16·0 (13·9 to 18·6)	2230 (1880 to 2490)	1930 (1630 to 2140)	-13·2 (-17·1 to -8·26)	1450 (1220 to 1620)
	Under 5	4·76 (3·64 to 6·08)	3·89 (2·86 to 5·02)	-18·2 (-27·4 to -10·6)	3·81 (2·92 to 4·86)	19·7 (17·0 to 22·0)	10·7 (8·84 to 12·7)	-45·6 (-51·6 to -39·7)	12·8 (11·0 to 14·3)
	5-14 years	5·99 (4·29 to 8·26)	5·72 (3·91 to 8·10)	-4·55 (-14·0 to 5·18)	4·79 (3·43 to 6·61)	14·4 (12·1 to 16·2)	10·9 (9·33 to 12·6)	-23·8 (-31·2 to -16·5)	9·35 (7·86 to 10·5)

eTable 3. Percent change from 2015 to 2021 in age-standardised all-form tuberculosis incidence rate per 100,000 population and in deaths due to all-form tuberculosis by age for 204 countries and territories.

Location	Age group	2015 Rate	2021 Rate	Incidence Percent Change	Incidence Milestone	2015 Deaths	2021 Deaths	Mortality Percent Change	Mortality Milestone
	15-49 years	24·1 (19·7 to 29·5)	22·4 (18·6 to 27·4)	-6·67 (-11·8 to -1·74)	19·2 (15·7 to 23·6)	929 (705 to 1090)	780 (591 to 934)	-16·0 (-20·3 to -10·5)	604 (458 to 709)
	50-69 years	24·8 (19·0 to 30·9)	22·7 (17·1 to 28·7)	-8·37 (-12·5 to -3·64)	19·8 (15·2 to 24·7)	711 (620 to 791)	645 (549 to 728)	-9·41 (-15·0 to -3·89)	462 (403 to 514)
	70+ years	25·1 (20·3 to 30·8)	22·3 (18·1 to 27·2)	-11·3 (-16·9 to -4·50)	20·1 (16·2 to 24·6)	554 (503 to 588)	488 (442 to 519)	-11·9 (-15·9 to -8·47)	360 (327 to 382)
Argentina	All Ages	20·7 (17·9 to 24·0)	19·4 (16·9 to 22·5)	-6·13 (-11·0 to -1·46)	16·5 (14·3 to 19·2)	1460 (1210 to 1660)	1270 (1040 to 1440)	-13·2 (-17·2 to -7·44)	949 (790 to 1080)
	Under 5	5·33 (3·98 to 6·78)	4·32 (3·12 to 5·72)	-18·9 (-30·3 to -7·90)	4·27 (3·19 to 5·42)	14·5 (12·2 to 16·3)	8·08 (6·42 to 9·78)	-44·1 (-50·2 to -36·8)	9·40 (7·92 to 10·6)
	5-14 years	7·05 (5·02 to 9·69)	6·63 (4·60 to 9·41)	-5·92 (-16·2 to 5·34)	5·64 (4·02 to 7·75)	10·7 (8·71 to 12·2)	8·29 (6·96 to 9·71)	-22·2 (-30·2 to -14·1)	6·94 (5·66 to 7·96)
	15-49 years	26·1 (21·2 to 32·2)	24·1 (19·6 to 29·3)	-7·55 (-13·8 to -0·992)	20·9 (17·0 to 25·8)	666 (499 to 797)	557 (413 to 681)	-16·3 (-21·4 to -10·2)	433 (324 to 518)
	50-69 years	24·4 (18·8 to 30·5)	22·6 (16·6 to 28·9)	-7·57 (-13·8 to -0·948)	19·5 (15·1 to 24·4)	472 (408 to 528)	426 (356 to 494)	-9·85 (-16·1 to -3·79)	307 (266 to 343)
	70+ years	21·4 (17·2 to 26·8)	19·3 (15·4 to 24·0)	-9·63 (-18·5 to -1·17)	17·1 (13·7 to 21·5)	296 (273 to 316)	267 (244 to 291)	-9·81 (-14·6 to -4·94)	193 (177 to 205)
Chile	All Ages	15·8 (14·0 to 18·6)	15·4 (13·5 to 18·0)	-2·71 (-7·18 to 1·69)	12·7 (11·2 to 14·8)	682 (610 to 733)	591 (515 to 649)	-13·4 (-18·2 to -7·05)	444 (397 to 476)
	Under 5	2·89 (2·24 to 3·75)	2·58 (1·98 to 3·27)	-10·7 (-24·7 to 0·287)	2·31 (1·79 to 3·00)	4·72 (4·22 to 5·35)	2·35 (1·98 to 2·67)	-50·0 (-57·9 to -43·4)	3·07 (2·74 to 3·47)
	5-14 years	2·41 (1·61 to 3·36)	2·84 (1·92 to 3·91)	17·8 (5·67 to 30·9)	1·93 (1·29 to 2·69)	3·39 (3·02 to 3·88)	2·41 (2·14 to 2·72)	-28·7 (-36·1 to -21·1)	2·20 (1·96 to 2·52)
	15-49 years	16·0 (13·2 to 19·7)	15·8 (13·0 to 19·6)	-0·966 (-7·48 to 5·74)	12·8 (10·6 to 15·8)	234 (185 to 262)	198 (156 to 224)	-15·6 (-21·3 to -8·01)	152 (121 to 171)
	50-69 years	22·9 (17·6 to 28·5)	21·0 (16·2 to 26·7)	-8·32 (-16·1 to -1·87)	18·3 (14·0 to 22·8)	209 (186 to 226)	193 (169 to 214)	-7·77 (-15·0 to -0·712)	136 (121 to 147)
	70+ years	32·8 (26·6 to 39·9)	27·8 (23·0 to 33·8)	-15·2 (-22·7 to -7·27)	26·2 (21·3 to 31·9)	231 (205 to 249)	196 (176 to 216)	-15·1 (-19·9 to -9·27)	150 (133 to 162)
Uruguay	All Ages	32·5 (28·0 to 37·5)	29·2 (25·0 to 34·7)	-10·1 (-16·0 to -4·94)	26·0 (22·4 to 30·0)	86·9 (76·5 to 101)	76·3 (67·1 to 86·7)	-12·2 (-18·4 to -6·50)	56·5 (49·7 to 65·7)
	Under 5	5·70 (4·22 to 7·41)	4·64 (3·50 to 6·17)	-18·6 (-29·7 to -6·17)	4·56 (3·38 to 5·93)	0·509 (0·442 to 0·598)	0·273 (0·214 to 0·338)	-46·3 (-55·6 to -35·0)	0·331 (0·287 to 0·388)
	5-14 years	8·21 (5·60 to 11·7)	7·42 (5·07 to 10·8)	-9·38 (-21·6 to 2·81)	6·56 (4·48 to 9·36)	0·329 (0·274 to 0·396)	0·242 (0·202 to 0·292)	-26·4 (-34·0 to -18·6)	0·214 (0·178 to 0·257)
	15-49 years	41·7 (33·7 to 50·3)	37·0 (29·4 to 45·6)	-11·4 (-19·2 to -5·27)	33·3 (27·0 to 40·2)	29·1 (22·3 to 38·5)	25·1 (19·5 to 32·3)	-13·9 (-19·6 to -8·22)	18·9 (14·5 to 25·0)
	50-69 years	37·8 (28·2 to 46·9)	33·5 (24·8 to 41·1)	-11·5 (-21·0 to -3·11)	30·3 (22·5 to 37·5)	30·3 (26·6 to 34·8)	26·2 (22·8 to 30·0)	-13·7 (-21·4 to -5·51)	19·7 (17·3 to 22·6)
	70+ years	30·4 (24·6 to 37·2)	27·0 (21·9 to 33·6)	-11·1 (-18·9 to -1·16)	24·3 (19·7 to 29·8)	26·7 (23·2 to 29·4)	24·6 (21·1 to 27·5)	-7·66 (-15·7 to 1·46)	17·3 (15·1 to 19·1)
Western Europe	All Ages	7·18 (6·22 to 8·29)	5·99 (5·23 to 6·94)	-16·6 (-18·1 to -14·9)	5·74 (4·98 to 6·63)	3730 (3350 to 4100)	3100 (2760 to 3380)	-16·8 (-18·7 to -14·7)	2420 (2180 to 2660)

eTable 3. Percent change from 2015 to 2021 in age-standardised all-form tuberculosis incidence rate per 100,000 population and in deaths due to all-form tuberculosis by age for 204 countries and territories.

Location	Age group	2015 Rate	2021 Rate	Incidence Percent Change	Incidence Milestone	2015 Deaths	2021 Deaths	Mortality Percent Change	Mortality Milestone
	Under 5	2·89 (2·20 to 3·79)	2·15 (1·65 to 2·81)	-25·5 (-28·8 to -21·5)	2·31 (1·76 to 3·03)	8·52 (6·35 to 11·2)	5·83 (4·32 to 7·97)	-31·6 (-35·3 to -28·4)	5·54 (4·13 to 7·29)
	5-14 years	3·56 (2·38 to 5·32)	2·75 (1·82 to 4·12)	-22·6 (-26·2 to -18·6)	2·84 (1·91 to 4·26)	5·24 (4·04 to 6·88)	3·92 (2·97 to 5·14)	-25·3 (-27·6 to -23·0)	3·41 (2·63 to 4·47)
	15-49 years	9·55 (7·70 to 11·9)	8·12 (6·51 to 10·1)	-15·0 (-17·1 to -12·6)	7·64 (6·16 to 9·53)	450 (357 to 585)	341 (264 to 449)	-24·5 (-27·4 to -22·1)	293 (232 to 380)
	50-69 years	5·12 (3·82 to 6·34)	4·31 (3·23 to 5·39)	-15·7 (-18·5 to -13·7)	4·10 (3·06 to 5·07)	805 (720 to 933)	636 (559 to 732)	-21·0 (-23·6 to -18·5)	523 (468 to 607)
	70+ years	7·61 (6·22 to 9·40)	6·33 (5·20 to 7·81)	-16·7 (-19·6 to -14·6)	6·08 (4·98 to 7·52)	2460 (2130 to 2620)	2120 (1820 to 2280)	-14·0 (-16·2 to -11·6)	1600 (1390 to 1700)
Andorra	All Ages	6·04 (5·15 to 7·07)	5·76 (4·92 to 6·97)	-4·53 (-10·2 to 1·33)	4·83 (4·12 to 5·66)	0·109 (0·0882 to 0·135)	0·107 (0·0805 to 0·140)	-1·87 (-20·8 to 17·7)	0·0708 (0·0573 to 0·0881)
	Under 5	4·00 (2·90 to 5·23)	3·72 (2·75 to 4·84)	-6·62 (-16·8 to 6·88)	3·20 (2·32 to 4·18)	0·000127 (0·000100 to 0·000166)	0·0000664 (0·0000451 to 0·0000889)	-47·6 (-60·4 to -35·9)	0·0000825 (0·0000650 to 0·000108)
	5-14 years	1·10 (0·629 to 1·71)	1·03 (0·579 to 1·73)	-6·31 (-24·6 to 8·98)	0·880 (0·503 to 1·37)	0·0000662 (0·0000534 to 0·0000825)	0·0000435 (0·0000335 to 0·0000540)	-34·0 (-46·2 to -19·0)	0·0000430 (0·0000347 to 0·0000536)
	15-49 years	5·65 (4·30 to 7·25)	5·16 (3·99 to 6·46)	-8·64 (-15·5 to -0·493)	4·52 (3·44 to 5·80)	0·0140 (0·0108 to 0·0172)	0·0110 (0·00826 to 0·0142)	-21·3 (-38·7 to -6·61)	0·00910 (0·00704 to 0·0112)
	50-69 years	7·77 (5·59 to 10·4)	7·24 (5·18 to 9·48)	-6·76 (-15·1 to 0·181)	6·21 (4·47 to 8·36)	0·0260 (0·0201 to 0·0330)	0·0256 (0·0178 to 0·0349)	-1·19 (-25·6 to 26·1)	0·0169 (0·0131 to 0·0214)
	70+ years	10·0 (7·91 to 12·5)	8·93 (7·17 to 11·4)	-11·1 (-19·4 to -3·17)	8·04 (6·33 to 10·0)	0·0687 (0·0518 to 0·0861)	0·0700 (0·0522 to 0·0944)	2·14 (-17·0 to 22·4)	0·0447 (0·0337 to 0·0559)
Austria	All Ages	6·17 (5·34 to 7·17)	5·39 (4·75 to 6·17)	-12·6 (-17·2 to -7·99)	4·93 (4·27 to 5·73)	68·3 (61·6 to 74·2)	55·2 (49·3 to 59·8)	-19·2 (-23·3 to -15·8)	44·4 (40·1 to 48·3)
	Under 5	2·58 (1·83 to 3·39)	2·39 (1·77 to 3·31)	-6·92 (-18·8 to 5·79)	2·06 (1·46 to 2·71)	0·0842 (0·0664 to 0·109)	0·0640 (0·0492 to 0·0851)	-23·9 (-30·7 to -16·5)	0·0547 (0·0432 to 0·0711)
	5-14 years	2·28 (1·52 to 3·33)	2·31 (1·51 to 3·49)	1·29 (-12·2 to 10·2)	1·83 (1·22 to 2·66)	0·0655 (0·0518 to 0·0830)	0·0424 (0·0321 to 0·0573)	-35·5 (-41·3 to -28·7)	0·0426 (0·0337 to 0·0539)
	15-49 years	7·50 (5·99 to 9·37)	6·88 (5·57 to 8·44)	-8·26 (-14·7 to -1·74)	6·00 (4·79 to 7·50)	7·18 (5·69 to 9·31)	5·32 (4·03 to 7·13)	-26·1 (-31·3 to -20·2)	4·67 (3·70 to 6·05)
	50-69 years	5·39 (3·90 to 6·50)	4·26 (3·19 to 5·24)	-20·8 (-26·9 to -14·1)	4·31 (3·12 to 5·20)	17·6 (16·0 to 19·5)	12·2 (11·1 to 13·8)	-30·4 (-35·0 to -24·9)	11·4 (10·4 to 12·7)
	70+ years	7·04 (5·75 to 8·69)	5·92 (4·88 to 7·41)	-15·9 (-22·2 to -9·46)	5·63 (4·60 to 6·96)	43·4 (38·2 to 46·9)	37·5 (32·3 to 41·3)	-13·6 (-17·5 to -8·25)	28·2 (24·8 to 30·5)
Belgium	All Ages	7·53 (6·45 to 8·80)	6·24 (5·37 to 7·18)	-17·1 (-22·2 to -12·8)	6·03 (5·16 to 7·04)	83·1 (74·0 to 91·7)	62·6 (55·0 to 69·6)	-24·6 (-28·8 to -20·5)	54·0 (48·1 to 59·6)
	Under 5	3·15 (2·28 to 4·17)	2·61 (1·89 to 3·51)	-16·8 (-24·4 to -4·63)	2·52 (1·82 to 3·34)	0·139 (0·102 to 0·187)	0·103 (0·0745 to 0·145)	-25·3 (-31·4 to -18·6)	0·0901 (0·0660 to 0·122)
	5-14 years	3·14 (2·00 to 4·50)	2·89 (1·92 to 4·28)	-7·96 (-18·7 to 4·26)	2·51 (1·60 to 3·60)	0·0941 (0·0717 to 0·122)	0·0762 (0·0586 to 0·101)	-19·0 (-24·4 to -13·3)	0·0612 (0·0466 to 0·0793)
	15-49 years	10·4 (8·33 to 12·8)	8·82 (6·99 to 10·9)	-14·9 (-22·8 to -7·98)	8·30 (6·66 to 10·3)	8·97 (6·95 to 11·8)	6·60 (4·95 to 8·94)	-26·5 (-30·2 to -22·2)	5·83 (4·52 to 7·67)
	50-69 years	5·65 (4·23 to 7·02)	4·54 (3·42 to 5·67)	-19·6 (-25·6 to -13·7)	4·52 (3·38 to 5·62)	17·7 (15·7 to 20·5)	15·3 (13·4 to 17·6)	-13·6 (-20·7 to -7·03)	11·5 (10·2 to 13·3)

eTable 3. Percent change from 2015 to 2021 in age-standardised all-form tuberculosis incidence rate per 100,000 population and in deaths due to all-form tuberculosis by age for 204 countries and territories.

Location	Age group	2015 Rate	2021 Rate	Incidence Percent Change	Incidence Milestone	2015 Deaths	2021 Deaths	Mortality Percent Change	Mortality Milestone
	70+ years	7·06 (5·70 to 8·76)	5·46 (4·46 to 6·78)	-22·6 (-30·2 to -14·2)	5·65 (4·56 to 7·01)	56·2 (49·2 to 62·1)	40·5 (34·8 to 44·9)	-27·8 (-32·1 to -24·1)	36·5 (32·0 to 40·4)
Cyprus	All Ages	4·78 (4·22 to 5·59)	4·31 (3·78 to 5·04)	-9·84 (-14·0 to -5·71)	3·82 (3·38 to 4·47)	6·83 (5·74 to 8·26)	6·59 (5·50 to 7·83)	-3·22 (-20·1 to 13·3)	4·44 (3·73 to 5·37)
	Under 5	1·87 (1·38 to 2·42)	1·70 (1·28 to 2·24)	-9·17 (-18·9 to 0·606)	1·50 (1·10 to 1·94)	0·0108 (0·00807 to 0·0146)	0·00977 (0·00713 to 0·0139)	-9·80 (-21·8 to 5·00)	0·00705 (0·00524 to 0·00949)
	5-14 years	1·17 (0·742 to 1·69)	1·17 (0·745 to 1·71)	0·559 (-7·85 to 11·6)	0·934 (0·594 to 1·35)	0·00789 (0·00612 to 0·0103)	0·00864 (0·00635 to 0·0120)	9·63 (-8·00 to 27·2)	0·00513 (0·00398 to 0·00672)
	15-49 years	4·78 (3·80 to 6·07)	4·15 (3·31 to 5·32)	-13·3 (-19·6 to -6·56)	3·83 (3·04 to 4·85)	0·854 (0·666 to 1·14)	0·802 (0·606 to 1·08)	-5·96 (-17·7 to 12·1)	0·555 (0·433 to 0·742)
	50-69 years	4·89 (3·64 to 6·10)	4·35 (3·25 to 5·47)	-10·8 (-18·5 to -3·80)	3·91 (2·91 to 4·88)	1·57 (1·27 to 1·95)	1·39 (1·12 to 1·74)	-11·4 (-28·0 to 9·82)	1·02 (0·824 to 1·27)
	70+ years	10·9 (8·46 to 13·9)	9·80 (7·93 to 12·2)	-9·92 (-18·8 to -0·0890)	8·72 (6·77 to 11·1)	4·39 (3·56 to 5·38)	4·38 (3·58 to 5·36)	0·275 (-19·4 to 17·9)	2·85 (2·31 to 3·50)
Denmark	All Ages	4·73 (4·05 to 5·60)	4·04 (3·50 to 4·81)	-14·6 (-20·0 to -9·85)	3·79 (3·24 to 4·48)	24·6 (22·2 to 27·0)	20·7 (18·2 to 22·3)	-16·1 (-20·8 to -10·1)	16·0 (14·4 to 17·5)
	Under 5	2·24 (1·61 to 2·96)	2·12 (1·54 to 2·82)	-5·34 (-18·4 to 4·97)	1·79 (1·29 to 2·37)	0·0228 (0·0177 to 0·0297)	0·0183 (0·0139 to 0·0245)	-19·6 (-28·0 to -11·0)	0·0148 (0·0115 to 0·0193)
	5-14 years	1·90 (1·26 to 2·78)	1·94 (1·27 to 2·88)	2·08 (-7·89 to 14·8)	1·52 (1·01 to 2·22)	0·0186 (0·0152 to 0·0233)	0·0149 (0·0128 to 0·0179)	-19·9 (-26·4 to -13·3)	0·0121 (0·00987 to 0·0151)
	15-49 years	6·31 (5·10 to 7·89)	5·46 (4·35 to 6·88)	-13·4 (-20·9 to -6·23)	5·05 (4·08 to 6·31)	2·13 (1·75 to 2·60)	1·59 (1·29 to 2·00)	-25·2 (-29·1 to -20·7)	1·38 (1·14 to 1·69)
	50-69 years	4·10 (2·90 to 4·98)	3·29 (2·41 to 4·20)	-19·6 (-26·9 to -13·1)	3·28 (2·32 to 3·98)	6·42 (5·83 to 7·11)	5·03 (4·50 to 5·62)	-21·6 (-27·8 to -14·5)	4·17 (3·79 to 4·62)
	70+ years	4·04 (3·26 to 5·09)	3·33 (2·67 to 4·24)	-17·5 (-25·7 to -6·92)	3·23 (2·61 to 4·07)	16·0 (13·8 to 17·5)	14·0 (12·0 to 15·3)	-12·6 (-17·5 to -6·26)	10·4 (8·98 to 11·4)
Finland	All Ages	4·70 (4·21 to 5·33)	4·19 (3·72 to 4·80)	-10·8 (-15·1 to -7·09)	3·76 (3·37 to 4·27)	55·6 (47·1 to 60·2)	48·8 (41·5 to 53·4)	-12·1 (-17·7 to -6·08)	36·1 (30·6 to 39·1)
	Under 5	1·93 (1·44 to 2·60)	1·84 (1·35 to 2·51)	-4·59 (-18·1 to 9·37)	1·55 (1·15 to 2·08)	0·0424 (0·0363 to 0·0470)	0·0299 (0·0249 to 0·0341)	-29·4 (-38·3 to -18·7)	0·0276 (0·0236 to 0·0306)
	5-14 years	1·75 (1·14 to 2·60)	1·83 (1·21 to 2·75)	4·70 (-6·81 to 16·5)	1·40 (0·913 to 2·08)	0·0488 (0·0431 to 0·0549)	0·0371 (0·0316 to 0·0423)	-23·9 (-32·5 to -12·2)	0·0317 (0·0280 to 0·0357)
	15-49 years	4·74 (3·85 to 5·91)	4·36 (3·48 to 5·34)	-8·09 (-14·6 to -1·20)	3·79 (3·08 to 4·73)	4·12 (3·61 to 4·51)	3·65 (3·18 to 4·01)	-11·4 (-17·5 to -5·67)	2·68 (2·35 to 2·93)
	50-69 years	4·02 (3·00 to 5·08)	3·34 (2·49 to 4·16)	-16·7 (-23·1 to -10·3)	3·22 (2·40 to 4·06)	10·6 (9·87 to 11·5)	8·22 (7·57 to 8·89)	-22·5 (-28·1 to -16·0)	6·90 (6·42 to 7·50)
	70+ years	9·39 (7·82 to 11·5)	7·20 (5·93 to 8·80)	-23·2 (-29·0 to -16·8)	7·51 (6·25 to 9·20)	40·7 (33·0 to 45·2)	36·9 (29·7 to 40·7)	-9·39 (-16·0 to -3·40)	26·5 (21·5 to 29·4)
France	All Ages	7·20 (6·34 to 8·20)	6·36 (5·61 to 7·35)	-11·6 (-16·4 to -7·18)	5·76 (5·07 to 6·56)	942 (822 to 1050)	794 (689 to 881)	-15·7 (-20·3 to -11·1)	612 (534 to 685)
	Under 5	2·22 (1·57 to 2·88)	1·95 (1·46 to 2·64)	-12·0 (-20·6 to 0·409)	1·77 (1·25 to 2·31)	2·86 (2·05 to 3·87)	2·09 (1·52 to 2·88)	-27·0 (-32·7 to -20·6)	1·86 (1·34 to 2·52)
	5-14 years	2·22 (1·50 to 3·25)	2·26 (1·44 to 3·31)	1·55 (-8·76 to 14·1)	1·78 (1·20 to 2·60)	1·75 (1·31 to 2·40)	1·25 (0·936 to 1·64)	-28·6 (-33·5 to -24·2)	1·14 (0·853 to 1·56)

eTable 3. Percent change from 2015 to 2021 in age-standardised all-form tuberculosis incidence rate per 100,000 population and in deaths due to all-form tuberculosis by age for 204 countries and territories.

Location	Age group	2015 Rate	2021 Rate	Incidence Percent Change	Incidence Milestone	2015 Deaths	2021 Deaths	Mortality Percent Change	Mortality Milestone
	15-49 years	8·62 (6·94 to 10·6)	7·87 (6·34 to 9·86)	-8·62 (-16·3 to -0·896)	6·89 (5·55 to 8·49)	99·2 (77·6 to 130)	83·2 (62·9 to 110)	-16·1 (-21·4 to -11·7)	64·5 (50·4 to 84·3)
	50-69 years	5·71 (4·26 to 7·14)	4·79 (3·49 to 5·94)	-16·2 (-21·3 to -8·20)	4·57 (3·41 to 5·71)	174 (147 to 215)	134 (111 to 164)	-22·7 (-28·1 to -17·1)	113 (95·7 to 140)
	70+ years	12·3 (10·0 to 15·2)	9·58 (7·89 to 11·8)	-21·8 (-28·4 to -16·5)	9·81 (8·02 to 12·2)	665 (560 to 731)	573 (475 to 630)	-13·8 (-18·8 to -8·74)	432 (364 to 475)
Germany	All Ages	5·60 (4·84 to 6·43)	5·34 (4·65 to 6·21)	-4·64 (-10·3 to 1·28)	4·48 (3·87 to 5·14)	504 (453 to 549)	494 (426 to 545)	-2·09 (-6·56 to 2·74)	328 (294 to 357)
	Under 5	2·34 (1·72 to 3·06)	2·20 (1·62 to 2·90)	-6·03 (-18·4 to 4·88)	1·87 (1·37 to 2·45)	0·759 (0·593 to 0·985)	0·637 (0·468 to 0·865)	-16·1 (-24·4 to -9·59)	0·493 (0·385 to 0·640)
	5-14 years	1·81 (1·23 to 2·77)	1·85 (1·24 to 2·69)	2·08 (-10·3 to 16·6)	1·45 (0·984 to 2·21)	0·409 (0·331 to 0·513)	0·384 (0·287 to 0·498)	-6·29 (-13·7 to 0·234)	0·266 (0·215 to 0·333)
	15-49 years	7·64 (6·21 to 9·52)	7·76 (6·25 to 10·0)	1·62 (-5·91 to 10·5)	6·11 (4·97 to 7·61)	52·1 (43·1 to 65·1)	42·7 (34·7 to 54·0)	-18·0 (-23·2 to -12·8)	33·9 (28·0 to 42·3)
	50-69 years	4·11 (3·01 to 5·13)	3·52 (2·64 to 4·31)	-14·1 (-22·6 to -7·47)	3·29 (2·41 to 4·11)	121 (108 to 139)	112 (97·1 to 129)	-7·47 (-13·1 to -0·513)	78·7 (70·3 to 90·3)
	70+ years	5·60 (4·43 to 6·99)	5·22 (4·19 to 6·34)	-6·70 (-15·2 to 0·321)	4·48 (3·54 to 5·59)	330 (284 to 364)	338 (290 to 373)	2·43 (-2·91 to 8·76)	215 (185 to 236)
Greece	All Ages	4·50 (3·96 to 5·17)	4·06 (3·63 to 4·61)	-9·94 (-13·7 to -5·84)	3·60 (3·17 to 4·14)	118 (105 to 126)	111 (101 to 119)	-5·91 (-11·1 to 0·0175)	76·8 (68·3 to 81·9)
	Under 5	1·67 (1·23 to 2·19)	1·49 (1·10 to 2·05)	-10·4 (-21·9 to 3·58)	1·33 (0·982 to 1·75)	0·149 (0·131 to 0·165)	0·104 (0·0908 to 0·119)	-30·0 (-40·9 to -18·1)	0·0970 (0·0851 to 0·107)
	5-14 years	1·44 (0·947 to 2·10)	1·39 (0·897 to 1·99)	-3·22 (-14·7 to 9·54)	1·15 (0·757 to 1·68)	0·104 (0·0914 to 0·117)	0·0656 (0·0566 to 0·0775)	-36·6 (-47·7 to -23·0)	0·0676 (0·0594 to 0·0759)
	15-49 years	4·44 (3·54 to 5·55)	4·06 (3·30 to 5·05)	-8·49 (-14·0 to -1·45)	3·55 (2·83 to 4·44)	15·1 (13·5 to 16·5)	11·1 (10·1 to 12·1)	-26·1 (-33·8 to -19·2)	9·80 (8·76 to 10·7)
	50-69 years	4·14 (3·03 to 5·17)	3·67 (2·75 to 4·50)	-11·2 (-17·5 to -3·99)	3·31 (2·43 to 4·14)	22·7 (21·0 to 24·5)	22·2 (20·8 to 24·8)	-2·30 (-10·0 to 5·73)	14·8 (13·6 to 15·9)
	70+ years	8·03 (6·59 to 9·81)	6·77 (5·48 to 8·21)	-15·5 (-23·6 to -8·19)	6·43 (5·27 to 7·85)	80·1 (67·9 to 87·6)	77·6 (67·6 to 85·3)	-2·99 (-9·01 to 4·11)	52·0 (44·1 to 56·9)
Iceland	All Ages	4·48 (3·81 to 5·34)	3·85 (3·32 to 4·56)	-14·0 (-18·0 to -9·69)	3·58 (3·05 to 4·27)	1·64 (1·41 to 1·84)	1·63 (1·43 to 1·83)	-0·681 (-8·35 to 6·80)	1·07 (0·913 to 1·20)
	Under 5	1·84 (1·30 to 2·56)	1·71 (1·22 to 2·32)	-7·03 (-17·1 to 7·48)	1·48 (1·04 to 2·05)	0·00367 (0·00299 to 0·00470)	0·00312 (0·00246 to 0·00394)	-14·8 (-25·1 to -1·89)	0·00238 (0·00194 to 0·00305)
	5-14 years	2·34 (1·56 to 3·71)	2·17 (1·40 to 3·37)	-7·35 (-17·8 to 1·53)	1·88 (1·25 to 2·97)	0·00222 (0·00183 to 0·00280)	0·00275 (0·00234 to 0·00328)	24·3 (7·95 to 45·7)	0·00145 (0·00119 to 0·00182)
	15-49 years	5·71 (4·43 to 7·34)	4·78 (3·67 to 6·29)	-16·2 (-21·2 to -11·0)	4·57 (3·55 to 5·87)	0·183 (0·145 to 0·248)	0·203 (0·155 to 0·266)	11·2 (3·81 to 18·5)	0·119 (0·0944 to 0·161)
	50-69 years	3·16 (2·37 to 4·04)	2·77 (2·06 to 3·51)	-12·1 (-19·4 to -4·45)	2·52 (1·90 to 3·23)	0·314 (0·281 to 0·344)	0·297 (0·266 to 0·332)	-5·39 (-15·4 to 3·70)	0·204 (0·183 to 0·223)
	70+ years	6·39 (5·15 to 8·01)	5·37 (4·40 to 6·57)	-15·9 (-22·5 to -8·08)	5·11 (4·12 to 6·41)	1·14 (0·933 to 1·29)	1·12 (0·936 to 1·27)	-1·26 (-9·71 to 6·83)	0·740 (0·607 to 0·837)
Ireland	All Ages	5·90 (5·11 to 6·87)	4·92 (4·22 to 5·74)	-16·6 (-20·6 to -12·0)	4·72 (4·09 to 5·50)	29·7 (26·7 to 31·8)	24·4 (21·6 to 26·7)	-17·9 (-24·0 to -10·8)	19·3 (17·4 to 20·7)

eTable 3. Percent change from 2015 to 2021 in age-standardised all-form tuberculosis incidence rate per 100,000 population and in deaths due to all-form tuberculosis by age for 204 countries and territories.

Location	Age group	2015 Rate	2021 Rate	Incidence Percent Change	Incidence Milestone	2015 Deaths	2021 Deaths	Mortality Percent Change	Mortality Milestone
	Under 5	2·15 (1·58 to 2·85)	2·01 (1·45 to 2·72)	-6·13 (-18·6 to 10·5)	1·72 (1·26 to 2·28)	0·0841 (0·0695 to 0·0964)	0·0585 (0·0483 to 0·0675)	-30·3 (-40·6 to -18·2)	0·0547 (0·0452 to 0·0627)
	5-14 years	2·12 (1·40 to 3·09)	2·07 (1·29 to 3·11)	-2·22 (-14·9 to 12·4)	1·69 (1·12 to 2·47)	0·0523 (0·0441 to 0·0616)	0·0411 (0·0324 to 0·0489)	-21·2 (-33·7 to -5·27)	0·0340 (0·0287 to 0·0401)
	15-49 years	7·49 (5·98 to 9·23)	6·27 (4·99 to 7·88)	-16·2 (-21·4 to -11·4)	5·99 (4·78 to 7·38)	5·36 (4·43 to 6·01)	4·20 (3·44 to 4·79)	-21·6 (-29·8 to -11·8)	3·49 (2·88 to 3·91)
	50-69 years	5·19 (3·93 to 6·33)	4·11 (3·02 to 5·10)	-20·7 (-26·3 to -13·1)	4·15 (3·14 to 5·06)	6·57 (6·03 to 7·08)	5·07 (4·54 to 5·52)	-22·7 (-28·5 to -15·8)	4·27 (3·92 to 4·60)
	70+ years	7·97 (6·55 to 9·76)	6·15 (4·88 to 7·65)	-22·9 (-28·4 to -17·0)	6·38 (5·24 to 7·81)	17·6 (14·8 to 19·1)	15·0 (12·7 to 17·0)	-14·9 (-22·0 to -6·35)	11·5 (9·59 to 12·4)
Israel	All Ages	3·25 (2·79 to 3·87)	2·81 (2·42 to 3·30)	-13·5 (-18·0 to -8·88)	2·60 (2·23 to 3·10)	30·3 (27·1 to 33·3)	29·3 (26·3 to 32·3)	-3·01 (-10·4 to 3·23)	19·7 (17·6 to 21·6)
	Under 5	1·52 (1·12 to 1·97)	1·43 (1·04 to 1·88)	-5·82 (-16·4 to 12·2)	1·21 (0·898 to 1·58)	0·157 (0·123 to 0·207)	0·116 (0·0848 to 0·160)	-26·2 (-33·6 to -17·6)	0·102 (0·0799 to 0·135)
	5-14 years	1·03 (0·689 to 1·53)	1·13 (0·735 to 1·62)	9·59 (-3·64 to 24·1)	0·822 (0·551 to 1·22)	0·0875 (0·0705 to 0·109)	0·0663 (0·0532 to 0·0851)	-24·2 (-30·9 to -16·4)	0·0569 (0·0458 to 0·0712)
	15-49 years	4·20 (3·34 to 5·31)	3·55 (2·83 to 4·39)	-15·4 (-20·2 to -9·94)	3·36 (2·67 to 4·25)	3·89 (3·07 to 4·92)	3·43 (2·73 to 4·34)	-11·8 (-17·8 to -5·83)	2·53 (2·00 to 3·20)
	50-69 years	2·99 (2·24 to 3·71)	2·46 (1·84 to 3·08)	-17·5 (-23·6 to -10·9)	2·39 (1·79 to 2·97)	7·24 (6·62 to 8·01)	6·06 (5·47 to 6·77)	-16·3 (-22·2 to -8·51)	4·71 (4·30 to 5·21)
	70+ years	5·55 (4·49 to 6·98)	4·49 (3·66 to 5·58)	-19·0 (-25·6 to -11·9)	4·44 (3·59 to 5·58)	18·9 (15·9 to 20·8)	19·7 (16·5 to 21·8)	4·17 (-3·87 to 11·5)	12·3 (10·4 to 13·5)
Italy	All Ages	6·40 (5·44 to 7·56)	5·71 (4·87 to 6·75)	-10·9 (-13·2 to -8·86)	5·12 (4·35 to 6·05)	388 (351 to 416)	289 (256 to 312)	-25·6 (-28·7 to -23·3)	252 (228 to 270)
	Under 5	2·73 (2·05 to 3·59)	2·51 (1·90 to 3·37)	-8·09 (-12·6 to -2·79)	2·18 (1·64 to 2·87)	0·431 (0·378 to 0·499)	0·220 (0·176 to 0·252)	-48·8 (-56·1 to -41·1)	0·280 (0·246 to 0·324)
	5-14 years	2·78 (1·80 to 4·31)	2·56 (1·64 to 4·00)	-7·76 (-11·5 to -2·81)	2·22 (1·44 to 3·45)	0·329 (0·288 to 0·385)	0·235 (0·210 to 0·265)	-28·6 (-32·5 to -22·0)	0·214 (0·187 to 0·250)
	15-49 years	8·98 (6·96 to 11·3)	8·42 (6·49 to 10·7)	-6·31 (-9·65 to -3·46)	7·19 (5·57 to 9·02)	38·8 (33·9 to 45·7)	22·2 (18·7 to 26·8)	-42·8 (-45·5 to -40·5)	25·2 (22·1 to 29·7)
	50-69 years	4·41 (3·27 to 5·60)	3·84 (2·87 to 4·88)	-12·9 (-15·9 to -10·3)	3·53 (2·62 to 4·48)	72·4 (66·3 to 80·2)	52·7 (49·2 to 56·6)	-27·2 (-30·2 to -24·6)	47·1 (43·1 to 52·1)
	70+ years	5·63 (4·54 to 7·12)	4·66 (3·76 to 5·99)	-17·1 (-20·3 to -13·8)	4·50 (3·63 to 5·69)	276 (237 to 301)	214 (177 to 233)	-22·7 (-26·1 to -19·9)	180 (154 to 195)
Luxembourg	All Ages	8·67 (7·38 to 10·3)	7·63 (6·45 to 9·18)	-11·9 (-16·1 to -7·10)	6·93 (5·90 to 8·27)	1·62 (1·44 to 1·81)	1·42 (1·23 to 1·60)	-12·7 (-19·7 to -4·77)	1·05 (0·936 to 1·18)
	Under 5	4·27 (3·21 to 5·74)	3·80 (2·66 to 5·18)	-10·9 (-21·2 to 2·16)	3·41 (2·57 to 4·59)	0·00231 (0·00183 to 0·00307)	0·00201 (0·00162 to 0·00251)	-12·9 (-23·0 to -2·71)	0·00150 (0·00119 to 0·00200)
	5-14 years	4·80 (3·18 to 7·28)	4·45 (2·96 to 6·82)	-7·04 (-17·1 to 3·11)	3·84 (2·54 to 5·83)	0·00116 (0·000919 to 0·00149)	0·000771 (0·000647 to 0·000941)	-33·3 (-40·0 to -24·4)	0·000754 (0·000597 to 0·000972)
	15-49 years	11·7 (9·25 to 14·6)	10·6 (8·31 to 13·7)	-9·18 (-16·2 to -1·55)	9·33 (7·40 to 11·7)	0·194 (0·151 to 0·265)	0·157 (0·119 to 0·219)	-19·6 (-26·6 to -13·0)	0·126 (0·0979 to 0·172)
	50-69 years	5·82 (4·26 to 7·38)	4·69 (3·48 to 6·01)	-19·3 (-26·5 to -10·5)	4·65 (3·41 to 5·90)	0·354 (0·311 to 0·394)	0·300 (0·263 to 0·345)	-15·1 (-23·3 to -3·90)	0·230 (0·202 to 0·256)

eTable 3. Percent change from 2015 to 2021 in age-standardised all-form tuberculosis incidence rate per 100,000 population and in deaths due to all-form tuberculosis by age for 204 countries and territories.

Location	Age group	2015 Rate	2021 Rate	Incidence Percent Change	Incidence Milestone	2015 Deaths	2021 Deaths	Mortality Percent Change	Mortality Milestone
	70+ years	6·93 (5·60 to 8·96)	5·72 (4·69 to 7·28)	-17·4 (-23·3 to -11·2)	5·54 (4·48 to 7·16)	1·07 (0·924 to 1·19)	0·956 (0·791 to 1·09)	-10·7 (-18·3 to -2·62)	0·696 (0·601 to 0·775)
Malta	All Ages	25·8 (21·3 to 31·4)	21·4 (17·5 to 25·9)	-16·9 (-21·3 to -11·3)	20·6 (17·1 to 25·1)	0·926 (0·846 to 1·01)	0·819 (0·750 to 0·915)	-11·5 (-17·4 to -6·34)	0·602 (0·550 to 0·655)
	Under 5	10·4 (7·62 to 13·9)	9·26 (6·65 to 12·6)	-11·2 (-20·7 to -1·21)	8·35 (6·10 to 11·1)	0·00176 (0·00139 to 0·00235)	0·00139 (0·00106 to 0·00189)	-21·4 (-28·4 to -12·5)	0·00115 (0·000903 to 0·00153)
	5-14 years	12·9 (8·37 to 20·0)	11·8 (7·83 to 18·0)	-8·28 (-18·9 to 0·643)	10·3 (6·70 to 16·0)	0·000958 (0·000794 to 0·00116)	0·00144 (0·00119 to 0·00168)	51·0 (28·0 to 80·1)	0·000623 (0·000516 to 0·000756)
	15-49 years	43·1 (34·1 to 55·1)	36·6 (27·9 to 46·1)	-15·0 (-20·8 to -8·13)	34·5 (27·3 to 44·1)	0·116 (0·0926 to 0·151)	0·0968 (0·0777 to 0·125)	-16·3 (-21·4 to -11·7)	0·0751 (0·0602 to 0·0978)
	50-69 years	10·2 (7·73 to 13·1)	8·80 (6·51 to 11·5)	-14·1 (-20·9 to -6·12)	8·20 (6·19 to 10·5)	0·230 (0·207 to 0·256)	0·164 (0·150 to 0·192)	-28·4 (-34·1 to -23·0)	0·149 (0·134 to 0·166)
	70+ years	11·4 (9·19 to 14·5)	9·22 (7·28 to 11·8)	-19·3 (-25·3 to -12·7)	9·14 (7·35 to 11·6)	0·578 (0·512 to 0·630)	0·555 (0·481 to 0·620)	-3·89 (-9·88 to 2·58)	0·376 (0·333 to 0·410)
Monaco	All Ages	6·84 (5·99 to 7·81)	6·24 (5·52 to 7·08)	-8·78 (-13·6 to -4·79)	5·48 (4·79 to 6·25)	0·623 (0·523 to 0·761)	0·584 (0·472 to 0·720)	-6·06 (-22·2 to 11·8)	0·405 (0·340 to 0·494)
	Under 5	1·61 (1·24 to 2·09)	1·44 (1·10 to 1·85)	-10·7 (-19·7 to -0·868)	1·29 (0·991 to 1·67)	0·000394 (0·000298 to 0·000505)	0·000278 (0·000203 to 0·000355)	-29·1 (-43·5 to -14·2)	0·000256 (0·000194 to 0·000328)
	5-14 years	1·10 (0·690 to 1·66)	0·954 (0·625 to 1·44)	-12·9 (-23·0 to -0·632)	0·878 (0·552 to 1·33)	0·000211 (0·000163 to 0·000267)	0·000148 (0·000116 to 0·000190)	-29·5 (-44·3 to -14·8)	0·000137 (0·000106 to 0·000173)
	15-49 years	6·09 (4·93 to 7·60)	5·62 (4·48 to 7·26)	-7·69 (-14·5 to -1·45)	4·87 (3·94 to 6·08)	0·0484 (0·0365 to 0·0619)	0·0385 (0·0276 to 0·0511)	-20·2 (-36·6 to -2·15)	0·0314 (0·0237 to 0·0402)
	50-69 years	6·67 (4·98 to 8·51)	5·97 (4·38 to 7·37)	-10·4 (-17·8 to -2·49)	5·34 (3·98 to 6·81)	0·129 (0·0995 to 0·165)	0·120 (0·0930 to 0·153)	-6·92 (-23·2 to 13·1)	0·0838 (0·0647 to 0·108)
	70+ years	13·2 (10·9 to 16·5)	11·5 (9·57 to 14·4)	-12·6 (-20·6 to -5·39)	10·5 (8·70 to 13·2)	0·445 (0·368 to 0·567)	0·426 (0·337 to 0·536)	-4·11 (-20·3 to 16·0)	0·289 (0·239 to 0·369)
Netherlands	All Ages	4·98 (4·36 to 5·76)	4·31 (3·70 to 4·94)	-13·4 (-16·9 to -10·0)	3·98 (3·49 to 4·61)	108 (94·2 to 121)	89·9 (80·0 to 101)	-16·5 (-20·6 to -12·3)	70·0 (61·2 to 78·4)
	Under 5	1·73 (1·26 to 2·28)	1·61 (1·20 to 2·14)	-6·70 (-17·6 to 5·98)	1·38 (1·01 to 1·83)	0·230 (0·165 to 0·322)	0·170 (0·120 to 0·240)	-25·8 (-31·5 to -20·8)	0·149 (0·107 to 0·209)
	5-14 years	1·60 (1·05 to 2·33)	1·61 (1·05 to 2·43)	0·918 (-11·1 to 16·8)	1·28 (0·837 to 1·87)	0·177 (0·129 to 0·244)	0·135 (0·101 to 0·179)	-23·3 (-28·3 to -17·6)	0·115 (0·0842 to 0·159)
	15-49 years	6·83 (5·56 to 8·47)	6·02 (4·84 to 7·45)	-11·8 (-17·6 to -6·14)	5·46 (4·45 to 6·78)	12·2 (8·97 to 16·9)	9·51 (7·01 to 13·3)	-22·3 (-26·2 to -18·1)	7·96 (5·83 to 11·0)
	50-69 years	3·59 (2·71 to 4·45)	2·95 (2·18 to 3·68)	-17·9 (-24·2 to -12·6)	2·87 (2·17 to 3·56)	24·8 (21·4 to 30·0)	19·7 (16·8 to 23·6)	-20·4 (-24·7 to -14·7)	16·1 (13·9 to 19·5)
	70+ years	5·77 (4·74 to 7·11)	4·67 (3·82 to 5·80)	-19·0 (-25·2 to -12·2)	4·62 (3·79 to 5·69)	70·2 (59·3 to 76·8)	60·3 (51·0 to 65·6)	-14·0 (-18·8 to -9·14)	45·6 (38·6 to 49·9)
Norway	All Ages	5·53 (4·61 to 6·52)	4·59 (3·91 to 5·28)	-17·1 (-19·3 to -14·3)	4·43 (3·69 to 5·21)	44·3 (38·7 to 48·1)	46·4 (40·3 to 50·5)	4·61 (1·16 to 9·20)	28·8 (25·2 to 31·3)
	Under 5	1·35 (0·978 to 1·85)	1·38 (1·01 to 1·87)	1·75 (-3·56 to 7·75)	1·08 (0·782 to 1·48)	0·0499 (0·0375 to 0·0624)	0·0423 (0·0313 to 0·0533)	-15·0 (-19·2 to -10·4)	0·0324 (0·0244 to 0·0405)

eTable 3. Percent change from 2015 to 2021 in age-standardised all-form tuberculosis incidence rate per 100,000 population and in deaths due to all-form tuberculosis by age for 204 countries and territories.

Location	Age group	2015 Rate	2021 Rate	Incidence Percent Change	Incidence Milestone	2015 Deaths	2021 Deaths	Mortality Percent Change	Mortality Milestone
	5-14 years	1·95 (1·18 to 3·04)	1·88 (1·14 to 2·95)	-3·48 (-7·83 to 1·56)	1·56 (0·946 to 2·43)	0·0410 (0·0271 to 0·0552)	0·0370 (0·0250 to 0·0513)	-9·87 (-14·0 to -4·32)	0·0266 (0·0176 to 0·0358)
	15-49 years	7·20 (5·43 to 9·26)	5·48 (4·16 to 7·06)	-23·9 (-26·6 to -21·1)	5·76 (4·34 to 7·41)	2·37 (1·73 to 3·14)	2·30 (1·71 to 3·01)	-2·71 (-6·24 to 2·20)	1·54 (1·13 to 2·04)
	50-69 years	2·98 (2·21 to 3·79)	2·73 (2·03 to 3·47)	-8·38 (-11·4 to -5·27)	2·39 (1·77 to 3·03)	6·47 (6·04 to 7·07)	6·09 (5·58 to 6·66)	-5·85 (-9·50 to -1·48)	4·21 (3·92 to 4·59)
	70+ years	10·0 (8·20 to 12·5)	8·65 (7·14 to 10·8)	-13·6 (-16·7 to -7·84)	8·02 (6·56 to 9·98)	35·4 (29·9 to 38·6)	37·9 (32·1 to 41·5)	7·07 (3·46 to 11·7)	23·0 (19·4 to 25·1)
Portugal	All Ages	16·3 (14·1 to 19·0)	13·3 (11·5 to 15·4)	-18·4 (-22·9 to -14·0)	13·0 (11·3 to 15·2)	309 (272 to 353)	270 (241 to 306)	-12·6 (-17·2 to -8·28)	201 (177 to 229)
	Under 5	2·84 (2·14 to 3·73)	2·55 (1·86 to 3·36)	-9·93 (-18·2 to 1·96)	2·27 (1·71 to 2·98)	0·458 (0·327 to 0·638)	0·403 (0·282 to 0·570)	-12·1 (-17·4 to -6·23)	0·298 (0·213 to 0·415)
	5-14 years	3·63 (2·40 to 5·29)	3·65 (2·35 to 5·41)	0·626 (-15·4 to 19·8)	2·91 (1·92 to 4·23)	0·362 (0·253 to 0·513)	0·292 (0·204 to 0·403)	-19·2 (-24·4 to -13·3)	0·235 (0·164 to 0·333)
	15-49 years	19·2 (15·2 to 23·9)	15·5 (12·4 to 19·7)	-19·3 (-25·0 to -13·5)	15·3 (12·2 to 19·1)	64·4 (47·5 to 88·8)	50·6 (35·5 to 70·9)	-21·5 (-26·7 to -16·9)	41·8 (30·9 to 57·7)
	50-69 years	16·9 (12·5 to 21·1)	13·1 (9·98 to 16·4)	-22·4 (-29·5 to -15·4)	13·5 (9·99 to 16·9)	83·2 (71·7 to 102)	68·6 (58·1 to 81·4)	-17·5 (-22·9 to -10·8)	54·0 (46·6 to 66·0)
	70+ years	18·4 (15·3 to 22·7)	15·5 (12·9 to 19·2)	-15·7 (-23·1 to -7·54)	14·7 (12·2 to 18·2)	161 (143 to 177)	150 (131 to 167)	-6·56 (-12·6 to -1·31)	104 (92·9 to 115)
San Marino	All Ages	2·61 (2·30 to 3·00)	2·58 (2·29 to 2·96)	-1·09 (-4·92 to 3·33)	2·09 (1·84 to 2·40)	0·129 (0·102 to 0·167)	0·125 (0·0798 to 0·171)	-3·31 (-26·5 to 23·0)	0·0842 (0·0662 to 0·109)
	Under 5	0·987 (0·726 to 1·33)	0·892 (0·646 to 1·18)	-9·51 (-21·5 to 4·71)	0·790 (0·581 to 1·07)	0·000165 (0·000126 to 0·000221)	0·000106 (0·0000778 to 0·000146)	-35·2 (-50·7 to -15·2)	0·000107 (0·0000822 to 0·000143)
	5-14 years	0·491 (0·311 to 0·745)	0·452 (0·298 to 0·678)	-7·94 (-18·8 to 2·42)	0·393 (0·249 to 0·596)	0·0000733 (0·0000597 to 0·0000887)	0·0000534 (0·0000397 to 0·0000744)	-27·1 (-40·7 to -8·90)	0·0000477 (0·0000388 to 0·0000577)
	15-49 years	2·53 (2·08 to 3·22)	2·44 (2·00 to 3·07)	-3·59 (-10·4 to 2·19)	2·03 (1·67 to 2·57)	0·00849 (0·00668 to 0·0105)	0·00655 (0·00465 to 0·00901)	-22·8 (-40·6 to -5·40)	0·00552 (0·00434 to 0·00680)
	50-69 years	2·58 (1·91 to 3·18)	2·47 (1·83 to 3·10)	-4·13 (-10·3 to 3·65)	2·06 (1·53 to 2·54)	0·0210 (0·0162 to 0·0278)	0·0188 (0·0123 to 0·0274)	-10·4 (-39·5 to 22·4)	0·0137 (0·0105 to 0·0181)
	70+ years	4·99 (4·12 to 6·12)	4·83 (3·96 to 6·02)	-3·24 (-10·4 to 4·23)	3·99 (3·29 to 4·90)	0·0997 (0·0759 to 0·131)	0·0996 (0·0608 to 0·137)	0·0122 (-25·1 to 28·7)	0·0648 (0·0493 to 0·0852)
Spain	All Ages	8·05 (6·97 to 9·44)	6·26 (5·49 to 7·33)	-22·2 (-26·6 to -18·2)	6·44 (5·58 to 7·55)	435 (395 to 493)	350 (312 to 389)	-19·5 (-24·2 to -14·8)	283 (257 to 321)
	Under 5	1·99 (1·32 to 2·80)	1·45 (0·911 to 2·07)	-27·1 (-36·5 to -18·6)	1·59 (1·06 to 2·24)	0·895 (0·659 to 1·23)	0·597 (0·419 to 0·856)	-33·4 (-38·6 to -28·1)	0·581 (0·428 to 0·801)
	5-14 years	3·03 (1·93 to 4·35)	2·49 (1·53 to 3·68)	-18·0 (-27·6 to -8·56)	2·43 (1·54 to 3·48)	0·620 (0·461 to 0·833)	0·464 (0·338 to 0·635)	-25·1 (-30·4 to -19·5)	0·403 (0·300 to 0·541)
	15-49 years	9·69 (7·76 to 12·4)	7·54 (6·09 to 9·57)	-22·1 (-28·2 to -17·6)	7·75 (6·21 to 9·91)	56·4 (42·3 to 76·1)	44·4 (32·1 to 61·5)	-21·4 (-26·5 to -15·9)	36·6 (27·5 to 49·5)
	50-69 years	7·48 (5·49 to 9·40)	5·78 (4·10 to 7·35)	-22·8 (-30·0 to -15·5)	5·99 (4·39 to 7·52)	103 (89·9 to 123)	77·8 (65·5 to 90·3)	-24·5 (-29·8 to -18·9)	67·0 (58·4 to 80·1)
	70+ years	9·31 (7·46 to 11·6)	7·25 (5·71 to 9·16)	-22·0 (-29·0 to -14·8)	7·45 (5·97 to 9·26)	274 (237 to 301)	227 (191 to 249)	-17·3 (-22·2 to -12·3)	178 (154 to 196)

eTable 3. Percent change from 2015 to 2021 in age-standardised all-form tuberculosis incidence rate per 100,000 population and in deaths due to all-form tuberculosis by age for 204 countries and territories.

Location	Age group	2015 Rate	2021 Rate	Incidence Percent Change	Incidence Milestone	2015 Deaths	2021 Deaths	Mortality Percent Change	Mortality Milestone
Sweden	All Ages	6·84 (5·81 to 8·12)	6·38 (5·25 to 7·82)	-6·80 (-11·6 to -1·67)	5·47 (4·64 to 6·50)	72·9 (64·4 to 80·3)	56·5 (48·0 to 63·3)	-22·5 (-29·8 to -17·6)	47·4 (41·9 to 52·2)
	Under 5	2·66 (1·96 to 3·54)	2·90 (2·09 to 3·92)	8·71 (-1·14 to 19·9)	2·13 (1·57 to 2·83)	0·106 (0·0732 to 0·147)	0·0661 (0·0445 to 0·0936)	-38·0 (-41·5 to -33·4)	0·0692 (0·0476 to 0·0952)
	5-14 years	3·78 (2·44 to 5·62)	3·60 (2·28 to 5·65)	-4·94 (-16·9 to 5·95)	3·03 (1·95 to 4·50)	0·0508 (0·0352 to 0·0712)	0·0339 (0·0229 to 0·0470)	-33·3 (-38·0 to -28·4)	0·0330 (0·0229 to 0·0463)
	15-49 years	9·79 (7·71 to 12·5)	9·14 (6·64 to 11·8)	-6·79 (-14·3 to 0·176)	7·83 (6·17 to 10·0)	3·12 (2·09 to 4·42)	2·35 (1·50 to 3·39)	-25·1 (-30·1 to -20·7)	2·03 (1·36 to 2·87)
	50-69 years	3·42 (2·56 to 4·40)	3·49 (2·59 to 4·58)	1·91 (-6·03 to 9·02)	2·74 (2·05 to 3·52)	8·18 (7·27 to 9·24)	5·66 (4·82 to 6·65)	-30·8 (-38·3 to -25·7)	5·32 (4·73 to 6·01)
	70+ years	7·58 (6·12 to 9·28)	6·39 (5·29 to 7·91)	-15·7 (-21·6 to -9·67)	6·07 (4·90 to 7·42)	61·4 (52·4 to 67·4)	48·4 (40·1 to 54·2)	-21·2 (-28·5 to -15·9)	39·9 (34·1 to 43·8)
Switzerland	All Ages	5·78 (5·06 to 6·76)	4·96 (4·31 to 5·83)	-14·3 (-17·9 to -9·74)	4·63 (4·05 to 5·41)	39·8 (35·3 to 43·3)	33·4 (29·0 to 36·8)	-16·3 (-21·3 to -11·0)	25·9 (23·0 to 28·1)
	Under 5	2·33 (1·67 to 3·05)	2·13 (1·52 to 2·84)	-8·30 (-20·8 to 7·30)	1·87 (1·34 to 2·44)	0·0679 (0·0510 to 0·0921)	0·0476 (0·0369 to 0·0648)	-29·7 (-35·7 to -21·0)	0·0441 (0·0331 to 0·0599)
	5-14 years	2·10 (1·38 to 3·13)	2·19 (1·44 to 3·31)	3·97 (-6·70 to 20·7)	1·68 (1·11 to 2·50)	0·0470 (0·0373 to 0·0603)	0·0374 (0·0293 to 0·0480)	-20·4 (-26·3 to -11·3)	0·0306 (0·0243 to 0·0392)
	15-49 years	8·14 (6·71 to 9·99)	7·16 (5·90 to 8·92)	-12·1 (-18·0 to -5·18)	6·51 (5·37 to 7·99)	4·14 (3·16 to 5·43)	3·03 (2·24 to 4·14)	-27·0 (-31·7 to -21·1)	2·69 (2·06 to 3·53)
	50-69 years	3·71 (2·73 to 4·69)	3·02 (2·22 to 3·77)	-18·5 (-24·5 to -12·4)	2·97 (2·18 to 3·75)	8·70 (7·83 to 10·1)	6·25 (5·48 to 7·47)	-28·3 (-33·5 to -22·0)	5·66 (5·09 to 6·56)
	70+ years	5·34 (4·36 to 6·62)	4·51 (3·69 to 5·68)	-15·6 (-22·2 to -8·24)	4·27 (3·49 to 5·30)	26·9 (22·8 to 29·2)	24·0 (19·4 to 26·4)	-10·7 (-17·0 to -5·39)	17·5 (14·8 to 19·0)
United Kingdom	All Ages	10·2 (8·36 to 11·9)	7·07 (5·81 to 8·29)	-30·4 (-32·4 to -28·6)	8·12 (6·69 to 9·51)	462 (424 to 496)	315 (290 to 339)	-31·8 (-33·3 to -30·6)	300 (275 to 322)
	Under 5	5·72 (4·32 to 7·66)	2·60 (1·94 to 3·47)	-54·6 (-57·1 to -51·5)	4·58 (3·46 to 6·13)	1·96 (1·47 to 2·58)	1·04 (0·780 to 1·41)	-46·7 (-49·1 to -44·0)	1·27 (0·954 to 1·68)
	5-14 years	9·81 (6·36 to 14·7)	5·27 (3·26 to 7·96)	-46·4 (-49·9 to -42·3)	7·85 (5·09 to 11·7)	0·963 (0·766 to 1·22)	0·686 (0·510 to 0·894)	-28·9 (-33·4 to -26·3)	0·626 (0·498 to 0·793)
	15-49 years	14·9 (11·3 to 18·7)	10·4 (7·79 to 13·1)	-30·5 (-32·8 to -28·5)	12·0 (9·07 to 15·0)	69·3 (57·0 to 86·7)	42·8 (34·2 to 54·6)	-38·3 (-40·0 to -36·8)	45·0 (37·0 to 56·4)
	50-69 years	4·34 (3·14 to 5·58)	4·09 (2·97 to 5·24)	-5·87 (-8·21 to -3·41)	3·47 (2·51 to 4·47)	112 (104 to 122)	76·0 (70·1 to 83·4)	-31·9 (-33·4 to -30·2)	72·5 (67·6 to 79·2)
	70+ years	5·76 (4·72 to 7·41)	4·85 (3·96 to 6·12)	-15·9 (-18·2 to -12·8)	4·61 (3·77 to 5·93)	278 (249 to 294)	194 (175 to 206)	-30·1 (-31·6 to -28·5)	181 (162 to 191)
Latin America and Caribbean	All Ages	36·0 (31·7 to 41·0)	34·8 (30·4 to 40·2)	-3·28 (-6·08 to -0·0935)	28·8 (25·3 to 32·8)	25800 (22600 to 31200)	24200 (20600 to 29100)	-6·28 (-12·3 to 1·53)	16800 (14700 to 20300)
	Under 5	9·50 (7·94 to 11·7)	8·54 (7·01 to 10·6)	-10·1 (-14·3 to -6·55)	7·60 (6·35 to 9·33)	785 (640 to 986)	552 (442 to 709)	-29·7 (-38·3 to -20·1)	511 (416 to 641)
	5-14 years	7·91 (5·54 to 10·8)	7·84 (5·50 to 11·0)	-0·933 (-6·99 to 5·60)	6·32 (4·43 to 8·65)	347 (289 to 468)	266 (219 to 352)	-23·2 (-29·9 to -16·1)	225 (188 to 304)
	15-49 years	41·3 (34·8 to 50·1)	40·0 (33·5 to 49·0)	-3·28 (-6·92 to 0·451)	33·1 (27·8 to 40·1)	11400 (9440 to 14200)	10100 (8190 to 12500)	-11·6 (-16·6 to -5·60)	7420 (6140 to 9250)

eTable 3. Percent change from 2015 to 2021 in age-standardised all-form tuberculosis incidence rate per 100,000 population and in deaths due to all-form tuberculosis by age for 204 countries and territories.

Location	Age group	2015 Rate	2021 Rate	Incidence Percent Change	Incidence Milestone	2015 Deaths	2021 Deaths	Mortality Percent Change	Mortality Milestone
	50-69 years	54·3 (42·5 to 67·5)	49·6 (37·7 to 63·2)	-8·80 (-13·0 to -4·29)	43·4 (34·0 to 54·0)	7960 (7130 to 9610)	8040 (6850 to 9630)	0·956 (-6·63 to 9·82)	5170 (4630 to 6250)
	70+ years	64·0 (52·0 to 79·1)	54·0 (43·1 to 67·7)	-15·7 (-18·2 to -12·5)	51·2 (41·6 to 63·3)	5290 (4810 to 5960)	5230 (4650 to 6090)	-1·09 (-9·98 to 9·02)	3440 (3130 to 3870)
Andean Latin America	All Ages	65·2 (56·7 to 75·5)	66·2 (57·0 to 77·8)	1·44 (-1·98 to 5·60)	52·2 (45·3 to 60·4)	5200 (4510 to 6100)	4710 (3820 to 6040)	-9·36 (-27·0 to 9·09)	3380 (2930 to 3960)
	Under 5	18·4 (15·3 to 23·4)	15·8 (12·6 to 19·2)	-14·1 (-20·6 to -6·97)	14·7 (12·2 to 18·7)	168 (131 to 203)	98·6 (75·4 to 126)	-41·0 (-53·5 to -28·2)	109 (84·9 to 132)
	5-14 years	19·7 (13·8 to 27·1)	18·6 (13·6 to 26·1)	-5·33 (-14·0 to 4·50)	15·7 (11·0 to 21·7)	87·8 (71·2 to 102)	61·6 (47·6 to 78·0)	-29·7 (-42·9 to -16·7)	57·0 (46·3 to 66·6)
	15-49 years	76·6 (63·1 to 96·4)	77·6 (64·1 to 99·0)	1·37 (-3·30 to 6·78)	61·3 (50·4 to 77·1)	2050 (1700 to 2430)	1790 (1440 to 2260)	-12·3 (-27·6 to 4·19)	1330 (1100 to 1580)
	50-69 years	94·5 (71·8 to 118)	93·6 (70·3 to 117)	-1·05 (-6·30 to 4·56)	75·6 (57·4 to 94·0)	1450 (1240 to 1800)	1420 (1120 to 1810)	-2·06 (-23·0 to 21·5)	942 (805 to 1170)
	70+ years	135 (109 to 164)	130 (106 to 161)	-3·30 (-9·88 to 3·54)	108 (87·5 to 131)	1440 (1260 to 1730)	1340 (1080 to 1740)	-7·47 (-25·1 to 13·2)	939 (819 to 1130)
Bolivia (Plurinational State of)	All Ages	82·4 (71·6 to 93·6)	69·7 (60·6 to 78·2)	-15·4 (-20·4 to -11·0)	65·9 (57·3 to 74·9)	1780 (1160 to 2620)	1500 (1030 to 2080)	-15·3 (-29·9 to 2·93)	1160 (752 to 1700)
	Under 5	28·5 (23·1 to 36·2)	21·6 (17·1 to 26·9)	-24·2 (-32·5 to -14·5)	22·8 (18·5 to 28·9)	75·6 (49·7 to 99·9)	42·8 (28·9 to 57·6)	-43·1 (-56·3 to -32·4)	49·1 (32·3 to 64·9)
	5-14 years	26·0 (18·7 to 36·4)	20·5 (14·3 to 29·2)	-21·1 (-31·1 to -8·74)	20·8 (15·0 to 29·1)	30·9 (19·8 to 43·7)	20·0 (13·0 to 29·0)	-35·0 (-45·4 to -19·8)	20·1 (12·9 to 28·4)
	15-49 years	88·8 (73·2 to 113)	74·5 (61·5 to 92·5)	-16·0 (-22·6 to -10·9)	71·0 (58·5 to 90·2)	570 (354 to 823)	473 (324 to 684)	-16·3 (-32·3 to 4·66)	371 (230 to 535)
	50-69 years	149 (117 to 184)	123 (92·3 to 153)	-17·3 (-23·4 to -9·27)	119 (93·5 to 147)	575 (384 to 854)	503 (346 to 680)	-11·9 (-30·8 to 9·86)	374 (250 to 555)
	70+ years	236 (187 to 298)	188 (149 to 242)	-20·2 (-29·4 to -11·0)	189 (149 to 238)	532 (366 to 752)	464 (317 to 657)	-12·3 (-25·9 to 5·36)	346 (238 to 489)
Ecuador	All Ages	47·5 (41·5 to 55·5)	48·6 (42·1 to 57·2)	2·51 (-3·58 to 7·91)	38·0 (33·2 to 44·4)	1110 (947 to 1290)	621 (514 to 752)	-43·8 (-52·5 to -33·3)	719 (616 to 839)
	Under 5	12·1 (9·67 to 15·2)	9·92 (7·91 to 12·5)	-17·7 (-27·1 to -9·71)	9·65 (7·74 to 12·1)	22·4 (17·5 to 26·8)	10·1 (7·51 to 13·4)	-54·6 (-65·4 to -41·6)	14·5 (11·4 to 17·4)
	5-14 years	10·7 (7·28 to 14·5)	10·7 (7·40 to 14·6)	-0·736 (-10·8 to 9·35)	8·60 (5·83 to 11·6)	18·8 (16·6 to 21·2)	7·45 (6·07 to 8·87)	-60·4 (-66·3 to -55·9)	12·2 (10·8 to 13·8)
	15-49 years	59·1 (48·0 to 75·1)	60·3 (48·5 to 75·6)	2·15 (-5·62 to 9·19)	47·2 (38·4 to 60·1)	531 (423 to 653)	290 (230 to 369)	-45·4 (-52·4 to -37·1)	345 (275 to 424)
	50-69 years	67·4 (52·4 to 83·8)	66·4 (49·8 to 84·5)	-1·52 (-10·7 to 6·29)	53·9 (41·9 to 67·1)	267 (232 to 310)	154 (125 to 191)	-42·3 (-53·3 to -27·4)	174 (151 to 201)
	70+ years	95·3 (77·9 to 116)	88·4 (72·6 to 112)	-7·09 (-16·3 to 2·44)	76·2 (62·3 to 92·4)	266 (233 to 290)	159 (130 to 195)	-40·0 (-51·1 to -25·3)	173 (151 to 188)
Peru	All Ages	68·4 (59·2 to 79·7)	73·7 (62·6 to 87·1)	7·78 (1·37 to 13·8)	54·8 (47·4 to 63·7)	2310 (1940 to 2660)	2580 (1920 to 3520)	12·2 (-17·1 to 45·3)	1500 (1260 to 1730)
	Under 5	17·9 (14·7 to 22·9)	16·7 (13·1 to 20·3)	-6·63 (-17·3 to 7·67)	14·3 (11·7 to 18·3)	69·6 (53·2 to 90·0)	45·7 (33·6 to 65·8)	-33·9 (-50·4 to -10·3)	45·3 (34·6 to 58·5)

eTable 3. Percent change from 2015 to 2021 in age-standardised all-form tuberculosis incidence rate per 100,000 population and in deaths due to all-form tuberculosis by age for 204 countries and territories.

Location	Age group	2015 Rate	2021 Rate	Incidence Percent Change	Incidence Milestone	2015 Deaths	2021 Deaths	Mortality Percent Change	Mortality Milestone
	5-14 years	22·3 (15·7 to 30·7)	22·3 (15·6 to 32·0)	0·225 (-12·7 to 16·0)	17·8 (12·5 to 24·5)	38·0 (30·8 to 48·1)	34·2 (23·5 to 48·0)	-9·85 (-37·7 to 14·7)	24·7 (20·0 to 31·3)
	15-49 years	81·1 (65·5 to 104)	87·0 (70·5 to 111)	7·40 (-0·593 to 15·4)	64·8 (52·4 to 83·4)	945 (782 to 1130)	1030 (765 to 1360)	9·06 (-17·2 to 38·0)	614 (508 to 732)
	50-69 years	91·7 (67·6 to 116)	98·1 (74·5 to 123)	7·11 (-0·650 to 16·9)	73·4 (54·1 to 92·5)	607 (494 to 708)	762 (515 to 1050)	25·9 (-13·6 to 72·4)	395 (321 to 460)
	70+ years	128 (103 to 159)	137 (109 to 168)	6·96 (-2·85 to 20·7)	102 (82·6 to 127)	646 (543 to 786)	714 (513 to 1010)	10·6 (-20·1 to 51·5)	420 (353 to 511)
Caribbean	All Ages	45·0 (39·7 to 50·4)	40·9 (36·1 to 46·4)	-9·07 (-12·7 to -5·56)	36·0 (31·7 to 40·3)	4060 (2780 to 8610)	3380 (2230 to 7970)	-17·5 (-27·8 to -2·90)	2640 (1810 to 5590)
	Under 5	31·3 (25·5 to 38·8)	27·8 (22·3 to 35·0)	-11·0 (-19·8 to -1·64)	25·0 (20·4 to 31·0)	322 (230 to 497)	249 (173 to 395)	-22·6 (-37·7 to -5·36)	209 (150 to 323)
	5-14 years	21·7 (15·3 to 30·7)	19·0 (13·0 to 26·4)	-12·4 (-19·9 to -0·765)	17·4 (12·2 to 24·6)	104 (67·7 to 210)	85·3 (56·4 to 175)	-17·0 (-29·5 to -0·870)	67·4 (44·0 to 137)
	15-49 years	53·5 (44·8 to 63·4)	48·8 (40·2 to 58·9)	-8·69 (-12·8 to -4·40)	42·8 (35·9 to 50·7)	2030 (1340 to 4280)	1530 (996 to 3680)	-25·2 (-35·3 to -12·4)	1320 (873 to 2780)
	50-69 years	47·2 (37·8 to 58·5)	43·6 (34·8 to 53·6)	-7·70 (-12·8 to -2·87)	37·8 (30·3 to 46·8)	1030 (675 to 2690)	960 (616 to 2530)	-8·11 (-20·6 to 8·25)	672 (439 to 1750)
	70+ years	49·2 (39·6 to 61·5)	41·9 (33·8 to 52·1)	-14·7 (-19·6 to -9·44)	39·4 (31·7 to 49·2)	569 (401 to 1250)	550 (367 to 1270)	-3·66 (-19·5 to 18·9)	370 (260 to 812)
Antigua and Barbuda	All Ages	17·2 (14·8 to 20·1)	15·2 (13·1 to 18·1)	-11·6 (-15·7 to -7·58)	13·8 (11·9 to 16·1)	0·475 (0·424 to 0·554)	0·382 (0·337 to 0·440)	-19·6 (-23·5 to -14·9)	0·309 (0·275 to 0·360)
	Under 5	9·98 (7·62 to 13·1)	9·01 (6·72 to 11·5)	-9·48 (-22·4 to 0·890)	7·99 (6·09 to 10·4)	0·00434 (0·00347 to 0·00527)	0·00237 (0·00198 to 0·00290)	-45·1 (-53·2 to -36·2)	0·00282 (0·00225 to 0·00343)
	5-14 years	8·02 (5·19 to 11·7)	7·05 (4·68 to 10·3)	-11·7 (-25·1 to 1·79)	6·41 (4·15 to 9·38)	0·00332 (0·00269 to 0·00397)	0·00178 (0·00150 to 0·00220)	-46·2 (-51·1 to -40·5)	0·00216 (0·00175 to 0·00258)
	15-49 years	19·0 (15·2 to 24·0)	16·5 (13·2 to 20·9)	-12·9 (-17·6 to -6·74)	15·2 (12·1 to 19·2)	0·186 (0·154 to 0·230)	0·129 (0·105 to 0·166)	-30·5 (-35·1 to -25·8)	0·121 (0·100 to 0·149)
	50-69 years	20·9 (15·1 to 28·3)	18·0 (13·3 to 24·5)	-13·8 (-21·8 to -5·41)	16·7 (12·1 to 22·6)	0·157 (0·138 to 0·185)	0·138 (0·119 to 0·156)	-11·9 (-18·1 to -5·85)	0·102 (0·0895 to 0·120)
	70+ years	20·5 (15·6 to 25·7)	17·2 (12·7 to 22·3)	-16·0 (-26·3 to -1·30)	16·4 (12·5 to 20·5)	0·125 (0·115 to 0·136)	0·110 (0·101 to 0·124)	-11·5 (-20·1 to -0·207)	0·0813 (0·0750 to 0·0886)
Bahamas	All Ages	15·0 (13·1 to 17·3)	13·9 (12·0 to 16·2)	-7·27 (-12·7 to -1·57)	12·0 (10·5 to 13·9)	9·70 (7·95 to 12·1)	9·58 (7·59 to 12·6)	-1·36 (-11·4 to 10·2)	6·31 (5·17 to 7·89)
	Under 5	6·40 (4·95 to 8·35)	5·49 (4·43 to 6·99)	-13·9 (-24·2 to -2·11)	5·12 (3·96 to 6·68)	0·0683 (0·0541 to 0·0899)	0·0487 (0·0369 to 0·0658)	-28·8 (-37·2 to -15·9)	0·0444 (0·0352 to 0·0585)
	5-14 years	6·11 (3·97 to 8·77)	4·98 (3·37 to 7·08)	-18·4 (-30·2 to -7·05)	4·89 (3·18 to 7·02)	0·0652 (0·0495 to 0·0886)	0·0546 (0·0435 to 0·0716)	-15·9 (-25·9 to -6·12)	0·0424 (0·0322 to 0·0576)
	15-49 years	16·3 (13·6 to 20·4)	14·6 (12·1 to 18·6)	-10·5 (-16·2 to -3·92)	13·0 (10·9 to 16·3)	5·48 (4·26 to 7·27)	5·16 (3·91 to 6·97)	-5·87 (-14·9 to 3·79)	3·56 (2·77 to 4·72)
	50-69 years	21·6 (16·0 to 27·5)	19·9 (14·4 to 26·0)	-7·94 (-16·1 to 2·63)	17·3 (12·8 to 22·0)	2·73 (2·25 to 3·29)	2·96 (2·35 to 3·87)	8·45 (-3·39 to 23·2)	1·77 (1·46 to 2·14)
	70+ years	21·8 (17·3 to 28·1)	18·9 (14·8 to 24·0)	-13·2 (-22·6 to -4·90)	17·5 (13·9 to 22·4)	1·37 (1·18 to 1·49)	1·36 (1·16 to 1·63)	-0·734 (-13·6 to 13·1)	0·888 (0·768 to 0·969)

eTable 3. Percent change from 2015 to 2021 in age-standardised all-form tuberculosis incidence rate per 100,000 population and in deaths due to all-form tuberculosis by age for 204 countries and territories.

Location	Age group	2015 Rate	2021 Rate	Incidence Percent Change	Incidence Milestone	2015 Deaths	2021 Deaths	Mortality Percent Change	Mortality Milestone
Barbados	All Ages	6·17 (5·37 to 7·23)	5·72 (4·91 to 6·65)	-7·28 (-12·8 to -1·88)	4·93 (4·29 to 5·79)	1·92 (1·62 to 2·23)	1·85 (1·46 to 2·30)	-3·94 (-18·1 to 13·2)	1·25 (1·05 to 1·45)
	Under 5	3·35 (2·48 to 4·46)	3·03 (2·23 to 3·89)	-9·44 (-21·9 to 3·58)	2·68 (1·98 to 3·57)	0·00824 (0·00613 to 0·0106)	0·00658 (0·00474 to 0·00892)	-20·2 (-31·8 to -6·39)	0·00536 (0·00398 to 0·00689)
	5-14 years	2·39 (1·54 to 3·50)	2·15 (1·35 to 3·24)	-9·99 (-20·7 to 2·19)	1·91 (1·23 to 2·80)	0·0101 (0·00843 to 0·0121)	0·00563 (0·00355 to 0·00749)	-44·1 (-60·4 to -25·0)	0·00654 (0·00548 to 0·00789)
	15-49 years	6·31 (5·07 to 7·96)	5·64 (4·53 to 7·13)	-10·6 (-17·0 to -4·40)	5·05 (4·06 to 6·37)	0·637 (0·498 to 0·803)	0·551 (0·402 to 0·743)	-13·5 (-24·5 to -2·34)	0·414 (0·324 to 0·522)
	50-69 years	7·51 (5·52 to 10·1)	6·87 (5·09 to 9·04)	-8·49 (-16·0 to 0·0000470)	6·01 (4·42 to 8·04)	0·651 (0·554 to 0·754)	0·622 (0·469 to 0·796)	-4·54 (-20·7 to 14·1)	0·423 (0·360 to 0·490)
	70+ years	8·77 (6·76 to 11·3)	8·04 (6·19 to 10·2)	-8·21 (-18·3 to 5·72)	7·02 (5·41 to 9·05)	0·619 (0·546 to 0·674)	0·664 (0·532 to 0·833)	7·38 (-11·2 to 30·6)	0·402 (0·355 to 0·438)
Belize	All Ages	28·1 (24·3 to 32·4)	24·9 (21·5 to 28·9)	-11·5 (-17·6 to -5·39)	22·5 (19·5 to 25·9)	19·0 (15·9 to 23·7)	17·2 (14·5 to 21·0)	-9·49 (-16·8 to -1·48)	12·3 (10·4 to 15·4)
	Under 5	9·41 (7·38 to 11·7)	7·48 (5·88 to 9·42)	-20·2 (-32·1 to -5·82)	7·53 (5·91 to 9·39)	0·363 (0·288 to 0·448)	0·258 (0·205 to 0·341)	-29·0 (-37·0 to -20·7)	0·236 (0·187 to 0·291)
	5-14 years	10·3 (6·95 to 14·6)	9·23 (6·13 to 13·0)	-9·94 (-24·5 to 4·47)	8·20 (5·56 to 11·7)	0·257 (0·212 to 0·308)	0·173 (0·135 to 0·219)	-32·6 (-40·0 to -26·5)	0·167 (0·138 to 0·200)
	15-49 years	30·7 (25·2 to 37·9)	26·3 (21·8 to 33·4)	-14·2 (-21·7 to -7·21)	24·5 (20·2 to 30·3)	11·2 (8·79 to 14·6)	9·48 (7·48 to 12·3)	-15·2 (-21·4 to -9·33)	7·27 (5·72 to 9·49)
	50-69 years	62·3 (47·7 to 77·9)	50·2 (36·6 to 63·8)	-19·5 (-26·8 to -10·7)	49·9 (38·2 to 62·3)	4·73 (4·15 to 5·63)	4·70 (3·95 to 5·60)	-0·663 (-11·4 to 10·4)	3·08 (2·69 to 3·66)
	70+ years	57·0 (43·6 to 74·1)	44·6 (35·5 to 57·2)	-21·5 (-30·3 to -12·2)	45·6 (34·9 to 59·3)	2·45 (2·26 to 2·61)	2·57 (2·24 to 2·94)	4·93 (-7·24 to 18·1)	1·59 (1·47 to 1·69)
Bermuda	All Ages	18·1 (15·4 to 22·2)	16·3 (13·9 to 19·7)	-10·0 (-14·6 to -4·24)	14·5 (12·3 to 17·7)	0·0635 (0·0544 to 0·0740)	0·0475 (0·0405 to 0·0556)	-25·1 (-31·6 to -17·8)	0·0412 (0·0353 to 0·0481)
	Under 5	8·16 (6·12 to 10·5)	7·28 (5·52 to 9·44)	-10·6 (-20·0 to 1·26)	6·53 (4·90 to 8·41)	0·000309 (0·000243 to 0·000389)	0·000140 (0·000105 to 0·000179)	-54·5 (-61·7 to -47·0)	0·000201 (0·000158 to 0·000253)
	5-14 years	6·42 (4·31 to 9·31)	5·65 (3·81 to 8·36)	-11·6 (-23·9 to 1·56)	5·14 (3·45 to 7·45)	0·000215 (0·000174 to 0·000277)	0·000124 (0·0000923 to 0·000170)	-42·4 (-50·1 to -33·3)	0·000140 (0·000113 to 0·000180)
	15-49 years	20·4 (16·5 to 27·0)	18·1 (14·4 to 23·7)	-11·3 (-18·1 to -4·56)	16·3 (13·2 to 21·6)	0·0165 (0·0126 to 0·0221)	0·0103 (0·00747 to 0·0141)	-37·4 (-42·2 to -32·5)	0·0107 (0·00819 to 0·0143)
	50-69 years	22·0 (16·1 to 29·8)	20·0 (14·9 to 27·0)	-9·09 (-16·5 to -0·968)	17·6 (12·9 to 23·9)	0·0196 (0·0162 to 0·0241)	0·0144 (0·0113 to 0·0177)	-26·2 (-33·0 to -19·1)	0·0127 (0·0105 to 0·0156)
	70+ years	14·1 (11·0 to 18·2)	12·6 (9·61 to 16·2)	-10·7 (-21·5 to 0·110)	11·3 (8·77 to 14·5)	0·0268 (0·0227 to 0·0306)	0·0224 (0·0194 to 0·0265)	-16·3 (-25·1 to -5·52)	0·0174 (0·0148 to 0·0199)
Cuba	All Ages	6·42 (5·68 to 7·40)	5·54 (4·82 to 6·44)	-13·8 (-20·0 to -9·17)	5·14 (4·54 to 5·92)	47·6 (44·3 to 52·1)	36·0 (31·2 to 39·8)	-24·2 (-33·8 to -14·0)	30·9 (28·8 to 33·9)
	Under 5	2·51 (1·98 to 3·19)	2·29 (1·80 to 2·90)	-8·58 (-20·3 to 4·24)	2·01 (1·59 to 2·55)	0·169 (0·152 to 0·190)	0·0880 (0·0695 to 0·107)	-47·9 (-57·0 to -38·7)	0·110 (0·0989 to 0·123)
	5-14 years	0·910 (0·603 to 1·26)	0·964 (0·630 to 1·41)	6·09 (-9·15 to 26·1)	0·728 (0·482 to 1·01)	0·155 (0·140 to 0·175)	0·0749 (0·0607 to 0·0855)	-51·7 (-59·2 to -44·8)	0·101 (0·0911 to 0·114)

eTable 3. Percent change from 2015 to 2021 in age-standardised all-form tuberculosis incidence rate per 100,000 population and in deaths due to all-form tuberculosis by age for 204 countries and territories.

Location	Age group	2015 Rate	2021 Rate	Incidence Percent Change	Incidence Milestone	2015 Deaths	2021 Deaths	Mortality Percent Change	Mortality Milestone
	15-49 years	6·81 (5·68 to 8·54)	5·89 (4·75 to 7·12)	-13·4 (-23·1 to -7·23)	5·45 (4·54 to 6·83)	12·9 (10·9 to 15·5)	8·06 (6·61 to 9·87)	-37·3 (-43·7 to -31·4)	8·36 (7·08 to 10·1)
	50-69 years	8·39 (6·31 to 10·9)	6·99 (5·27 to 9·00)	-16·7 (-23·4 to -10·3)	6·71 (5·05 to 8·68)	17·7 (16·0 to 19·5)	15·4 (12·9 to 17·7)	-12·9 (-26·5 to 1·62)	11·5 (10·4 to 12·7)
	70+ years	8·11 (6·52 to 10·0)	6·35 (5·13 to 7·85)	-21·5 (-29·9 to -10·5)	6·49 (5·21 to 8·03)	16·6 (15·3 to 18·0)	12·4 (10·6 to 13·9)	-25·6 (-36·7 to -16·0)	10·8 (9·97 to 11·7)
Dominica	All Ages	21·3 (18·7 to 24·4)	19·9 (17·4 to 22·8)	-6·57 (-11·3 to -0·779)	17·0 (15·0 to 19·5)	3·06 (2·46 to 3·90)	2·90 (2·21 to 3·71)	-5·24 (-16·9 to 9·33)	1·99 (1·60 to 2·53)
	Under 5	8·18 (6·53 to 10·2)	7·43 (5·88 to 9·32)	-8·96 (-19·2 to 3·29)	6·54 (5·22 to 8·20)	0·0494 (0·0383 to 0·0651)	0·0358 (0·0246 to 0·0489)	-27·7 (-41·8 to -13·1)	0·0321 (0·0249 to 0·0423)
	5-14 years	6·85 (4·85 to 9·64)	6·17 (4·36 to 8·74)	-9·79 (-19·7 to 1·97)	5·48 (3·88 to 7·72)	0·0345 (0·0274 to 0·0447)	0·0258 (0·0186 to 0·0334)	-25·2 (-36·7 to -11·0)	0·0224 (0·0178 to 0·0291)
	15-49 years	22·2 (18·2 to 27·6)	19·8 (16·4 to 24·2)	-10·7 (-16·2 to -4·25)	17·8 (14·6 to 22·1)	1·12 (0·838 to 1·53)	0·948 (0·672 to 1·30)	-15·4 (-25·5 to -3·25)	0·729 (0·545 to 0·997)
	50-69 years	31·6 (24·4 to 39·3)	28·0 (21·2 to 35·7)	-11·3 (-19·0 to -3·32)	25·3 (19·6 to 31·5)	1·08 (0·809 to 1·41)	1·12 (0·808 to 1·50)	3·02 (-15·2 to 26·8)	0·704 (0·526 to 0·917)
	70+ years	36·9 (29·5 to 46·3)	34·2 (27·0 to 42·8)	-7·40 (-16·6 to 1·36)	29·5 (23·6 to 37·0)	0·771 (0·631 to 0·926)	0·776 (0·569 to 1·00)	0·587 (-14·4 to 17·6)	0·501 (0·410 to 0·602)
Dominican Republic	All Ages	60·9 (53·4 to 70·1)	51·3 (45·0 to 60·0)	-15·7 (-21·4 to -11·2)	48·7 (42·7 to 56·0)	1160 (857 to 1530)	852 (587 to 1340)	-27·0 (-39·9 to -8·31)	755 (557 to 994)
	Under 5	18·4 (14·9 to 22·8)	14·5 (11·8 to 18·6)	-20·9 (-29·7 to -4·80)	14·7 (11·9 to 18·3)	17·5 (12·1 to 24·2)	13·3 (9·18 to 18·3)	-22·9 (-45·5 to 7·50)	11·4 (7·89 to 15·7)
	5-14 years	21·9 (14·7 to 30·4)	16·6 (11·7 to 23·2)	-23·6 (-34·1 to -9·88)	17·5 (11·7 to 24·3)	13·8 (10·4 to 17·4)	11·3 (7·74 to 17·2)	-17·5 (-34·0 to 2·91)	8·95 (6·78 to 11·3)
	15-49 years	70·6 (57·6 to 88·8)	58·2 (47·6 to 73·7)	-17·5 (-23·9 to -11·3)	56·5 (46·1 to 71·0)	605 (419 to 820)	353 (236 to 548)	-41·7 (-50·5 to -31·3)	394 (272 to 533)
	50-69 years	84·0 (64·3 to 106)	71·9 (54·6 to 90·6)	-14·4 (-21·8 to -6·33)	67·2 (51·4 to 84·8)	294 (210 to 414)	245 (156 to 403)	-17·1 (-36·9 to 12·3)	191 (136 to 269)
	70+ years	123 (99·3 to 155)	104 (83·4 to 129)	-15·6 (-23·1 to -7·03)	98·6 (79·5 to 124)	231 (176 to 314)	229 (156 to 393)	-1·54 (-22·9 to 37·2)	150 (114 to 204)
Grenada	All Ages	10·0 (8·45 to 11·8)	9·10 (7·79 to 10·5)	-9·18 (-13·9 to -4·06)	8·02 (6·76 to 9·45)	0·691 (0·613 to 0·785)	0·433 (0·376 to 0·493)	-37·4 (-43·6 to -30·3)	0·449 (0·398 to 0·510)
	Under 5	6·64 (4·89 to 8·43)	5·94 (4·57 to 7·83)	-10·3 (-21·0 to 1·29)	5·31 (3·91 to 6·75)	0·00741 (0·00575 to 0·00974)	0·00312 (0·00240 to 0·00401)	-57·8 (-63·1 to -50·7)	0·00482 (0·00374 to 0·00633)
	5-14 years	4·76 (3·07 to 6·88)	4·27 (2·70 to 6·46)	-10·2 (-22·3 to 2·91)	3·80 (2·46 to 5·50)	0·00649 (0·00566 to 0·00746)	0·00337 (0·00282 to 0·00396)	-48·0 (-55·3 to -41·4)	0·00422 (0·00368 to 0·00485)
	15-49 years	11·0 (8·90 to 13·8)	10·0 (8·04 to 12·7)	-8·61 (-15·0 to -3·11)	8·76 (7·12 to 11·0)	0·274 (0·227 to 0·328)	0·149 (0·122 to 0·179)	-45·4 (-50·9 to -39·2)	0·178 (0·148 to 0·213)
	50-69 years	12·5 (9·34 to 16·4)	10·9 (7·99 to 14·7)	-13·1 (-19·9 to -5·85)	10·0 (7·47 to 13·1)	0·251 (0·219 to 0·287)	0·160 (0·136 to 0·188)	-36·0 (-44·5 to -27·0)	0·163 (0·143 to 0·186)
	70+ years	12·8 (9·73 to 16·4)	10·2 (7·65 to 13·6)	-19·7 (-30·3 to -8·66)	10·2 (7·78 to 13·1)	0·153 (0·142 to 0·168)	0·116 (0·103 to 0·129)	-23·7 (-31·1 to -16·0)	0·0993 (0·0924 to 0·109)
Guyana	All Ages	63·4 (55·2 to 72·4)	48·5 (41·7 to 56·9)	-23·6 (-28·4 to -18·9)	50·7 (44·2 to 57·9)	107 (85·1 to 131)	72·9 (56·2 to 91·4)	-32·1 (-42·7 to -20·2)	69·8 (55·3 to 84·9)

eTable 3. Percent change from 2015 to 2021 in age-standardised all-form tuberculosis incidence rate per 100,000 population and in deaths due to all-form tuberculosis by age for 204 countries and territories.

Location	Age group	2015 Rate	2021 Rate	Incidence Percent Change	Incidence Milestone	2015 Deaths	2021 Deaths	Mortality Percent Change	Mortality Milestone
	Under 5	15·9 (13·2 to 20·3)	12·0 (9·47 to 15·3)	-24·4 (-34·6 to -13·5)	12·7 (10·6 to 16·2)	1·65 (1·25 to 2·11)	0·926 (0·668 to 1·22)	-43·8 (-53·1 to -35·9)	1·07 (0·815 to 1·37)
	5-14 years	15·6 (11·2 to 21·5)	12·8 (8·90 to 18·3)	-18·0 (-29·6 to -2·14)	12·5 (8·95 to 17·2)	1·20 (0·993 to 1·55)	0·662 (0·518 to 0·832)	-44·8 (-52·2 to -38·1)	0·779 (0·645 to 1·01)
	15-49 years	78·0 (65·2 to 94·2)	57·4 (48·2 to 70·5)	-26·3 (-32·4 to -19·2)	62·4 (52·1 to 75·4)	60·6 (47·0 to 77·3)	39·3 (30·0 to 51·0)	-35·2 (-43·4 to -26·2)	39·4 (30·5 to 50·2)
	50-69 years	101 (82·4 to 122)	79·1 (60·8 to 99·0)	-21·8 (-28·1 to -13·5)	80·9 (65·9 to 97·3)	33·0 (25·9 to 39·5)	23·7 (18·0 to 29·8)	-27·9 (-40·7 to -12·4)	21·4 (16·8 to 25·7)
	70+ years	82·7 (65·6 to 103)	60·5 (47·9 to 76·6)	-26·8 (-34·0 to -20·0)	66·2 (52·5 to 82·3)	11·0 (9·40 to 12·8)	8·31 (6·64 to 10·3)	-24·3 (-38·0 to -6·71)	7·14 (6·11 to 8·31)
Haiti	All Ages	101 (88·3 to 115)	87·8 (76·5 to 99·2)	-12·6 (-17·7 to -6·34)	80·4 (70·7 to 92·1)	2450 (1460 to 7000)	2170 (1270 to 6600)	-12·2 (-26·1 to 3·20)	1590 (948 to 4550)
	Under 5	64·8 (51·4 to 81·7)	54·0 (42·9 to 67·6)	-16·4 (-25·9 to -5·27)	51·8 (41·1 to 65·4)	290 (201 to 456)	225 (154 to 364)	-22·3 (-39·2 to -4·71)	189 (131 to 297)
	5-14 years	43·5 (30·7 to 62·2)	36·5 (24·5 to 51·2)	-15·8 (-24·9 to -3·23)	34·8 (24·5 to 49·7)	83·9 (50·3 to 187)	69·5 (43·9 to 157)	-16·2 (-30·4 to 2·63)	54·6 (32·7 to 122)
	15-49 years	118 (97·9 to 139)	101 (82·1 to 120)	-14·2 (-20·2 to -8·22)	94·3 (78·3 to 111)	1210 (690 to 3440)	1020 (590 to 3100)	-15·9 (-30·6 to -0·781)	788 (448 to 2240)
	50-69 years	156 (127 to 191)	144 (114 to 175)	-7·66 (-16·2 to -0·293)	124 (102 to 152)	606 (324 to 2270)	601 (329 to 2140)	-1·68 (-19·1 to 17·0)	394 (211 to 1480)
	70+ years	216 (172 to 276)	187 (145 to 231)	-13·2 (-21·6 to -3·24)	173 (138 to 220)	258 (135 to 933)	253 (127 to 928)	-2·37 (-23·9 to 24·4)	168 (87·9 to 607)
Jamaica	All Ages	5·04 (4·34 to 5·87)	4·52 (3·94 to 5·23)	-10·2 (-14·9 to -5·23)	4·03 (3·47 to 4·70)	27·2 (21·6 to 34·6)	24·4 (18·9 to 31·3)	-10·3 (-21·2 to 3·72)	17·7 (14·1 to 22·5)
	Under 5	2·67 (2·05 to 3·54)	2·36 (1·84 to 3·07)	-11·4 (-22·0 to 1·89)	2·13 (1·64 to 2·83)	0·213 (0·160 to 0·276)	0·145 (0·110 to 0·198)	-31·8 (-38·1 to -23·4)	0·138 (0·104 to 0·180)
	5-14 years	1·78 (1·17 to 2·45)	1·55 (1·01 to 2·22)	-12·9 (-23·9 to -0·381)	1·42 (0·932 to 1·96)	0·198 (0·156 to 0·258)	0·150 (0·116 to 0·192)	-24·3 (-32·0 to -16·6)	0·129 (0·101 to 0·168)
	15-49 years	5·16 (4·30 to 6·41)	4·50 (3·71 to 5·70)	-12·7 (-18·7 to -7·11)	4·13 (3·44 to 5·13)	12·5 (8·84 to 17·6)	10·9 (7·88 to 15·6)	-13·0 (-20·5 to -3·32)	8·13 (5·75 to 11·4)
	50-69 years	7·46 (5·68 to 9·60)	6·51 (4·93 to 8·43)	-12·7 (-21·7 to -4·75)	5·97 (4·54 to 7·68)	8·06 (6·46 to 10·1)	7·61 (5·73 to 9·64)	-5·41 (-18·9 to 15·1)	5·24 (4·20 to 6·59)
	70+ years	9·35 (7·62 to 11·6)	7·98 (6·50 to 10·2)	-14·6 (-22·0 to -6·08)	7·48 (6·09 to 9·27)	6·22 (5·52 to 6·91)	5·60 (4·53 to 6·79)	-9·83 (-26·8 to 7·00)	4·04 (3·59 to 4·49)
Puerto Rico	All Ages	3·59 (3·18 to 4·04)	3·46 (2·99 to 3·97)	-3·73 (-10·1 to 3·03)	2·87 (2·55 to 3·23)	34·8 (30·0 to 41·5)	21·4 (17·7 to 25·7)	-38·3 (-45·1 to -30·8)	22·6 (19·5 to 26·9)
	Under 5	1·70 (1·28 to 2·18)	1·50 (1·17 to 2·02)	-11·6 (-22·8 to 5·45)	1·36 (1·02 to 1·74)	0·134 (0·104 to 0·174)	0·0378 (0·0292 to 0·0508)	-71·8 (-74·4 to -68·8)	0·0870 (0·0673 to 0·113)
	5-14 years	1·21 (0·787 to 1·81)	1·07 (0·657 to 1·62)	-11·6 (-22·2 to 8·49)	0·972 (0·630 to 1·45)	0·101 (0·0783 to 0·133)	0·0377 (0·0267 to 0·0531)	-62·6 (-66·8 to -59·2)	0·0654 (0·0509 to 0·0867)
	15-49 years	3·02 (2·48 to 3·79)	2·94 (2·41 to 3·74)	-2·51 (-9·42 to 6·08)	2·42 (1·99 to 3·03)	10·5 (8·42 to 13·6)	5·98 (4·62 to 7·85)	-43·2 (-48·2 to -38·7)	6·85 (5·48 to 8·87)
	50-69 years	4·72 (3·58 to 5·94)	4·29 (3·16 to 5·52)	-8·87 (-17·0 to 0·252)	3·77 (2·86 to 4·75)	12·4 (10·3 to 15·4)	7·29 (5·76 to 9·16)	-41·1 (-48·0 to -34·5)	8·05 (6·72 to 10·0)

eTable 3. Percent change from 2015 to 2021 in age-standardised all-form tuberculosis incidence rate per 100,000 population and in deaths due to all-form tuberculosis by age for 204 countries and territories.

Location	Age group	2015 Rate	2021 Rate	Incidence Percent Change	Incidence Milestone	2015 Deaths	2021 Deaths	Mortality Percent Change	Mortality Milestone
	70+ years	6·68 (5·35 to 8·28)	5·44 (4·37 to 6·80)	-18·5 (-26·4 to -9·57)	5·34 (4·28 to 6·62)	11·6 (10·4 to 12·6)	8·09 (6·96 to 9·37)	-30·2 (-39·6 to -19·6)	7·55 (6·76 to 8·22)
Saint Kitts and Nevis	All Ages	12·0 (10·5 to 14·0)	11·3 (9·79 to 13·1)	-5·48 (-10·8 to 0·427)	9·58 (8·36 to 11·2)	1·24 (1·03 to 1·50)	1·23 (0·983 to 1·46)	-0·853 (-10·3 to 8·83)	0·805 (0·666 to 0·977)
	Under 5	4·97 (3·94 to 6·17)	4·55 (3·65 to 5·70)	-8·27 (-18·3 to 6·83)	3·98 (3·15 to 4·94)	0·0139 (0·0101 to 0·0186)	0·0124 (0·00864 to 0·0173)	-10·8 (-20·6 to -0·216)	0·00901 (0·00657 to 0·0121)
	5-14 years	3·51 (2·45 to 4·87)	2·67 (1·86 to 3·64)	-23·6 (-31·8 to -12·3)	2·80 (1·96 to 3·90)	0·00805 (0·00683 to 0·00957)	0·00592 (0·00487 to 0·00735)	-26·3 (-37·4 to -13·3)	0·00523 (0·00444 to 0·00622)
	15-49 years	12·6 (10·4 to 15·7)	11·1 (9·14 to 13·8)	-12·0 (-18·0 to -3·50)	10·1 (8·36 to 12·6)	0·488 (0·362 to 0·662)	0·441 (0·323 to 0·587)	-9·52 (-16·9 to 0·590)	0·317 (0·235 to 0·430)
	50-69 years	16·4 (12·5 to 20·5)	16·1 (12·0 to 20·3)	-1·80 (-11·0 to 7·31)	13·1 (9·97 to 16·4)	0·493 (0·405 to 0·571)	0·539 (0·433 to 0·623)	9·29 (-2·73 to 22·2)	0·320 (0·263 to 0·371)
	70+ years	20·5 (16·2 to 25·1)	17·7 (13·9 to 22·2)	-13·6 (-22·4 to -2·80)	16·4 (13·0 to 20·0)	0·235 (0·214 to 0·253)	0·229 (0·196 to 0·261)	-2·57 (-13·7 to 8·62)	0·153 (0·139 to 0·165)
Saint Lucia	All Ages	12·9 (11·2 to 15·0)	12·3 (10·6 to 14·4)	-4·48 (-12·5 to 1·45)	10·3 (8·97 to 12·0)	4·28 (3·81 to 4·83)	4·51 (3·71 to 5·38)	5·32 (-7·99 to 21·2)	2·78 (2·48 to 3·14)
	Under 5	4·96 (3·92 to 6·24)	4·36 (3·50 to 5·60)	-11·8 (-23·9 to 0·756)	3·97 (3·14 to 4·99)	0·0265 (0·0207 to 0·0329)	0·0172 (0·0130 to 0·0220)	-34·9 (-44·8 to -19·5)	0·0172 (0·0135 to 0·0214)
	5-14 years	4·42 (2·79 to 6·48)	3·81 (2·49 to 5·34)	-13·5 (-23·3 to -0·294)	3·53 (2·23 to 5·18)	0·0278 (0·0236 to 0·0338)	0·0225 (0·0184 to 0·0269)	-18·9 (-31·0 to -6·92)	0·0181 (0·0153 to 0·0220)
	15-49 years	12·6 (10·3 to 15·8)	11·7 (9·62 to 15·0)	-7·24 (-15·9 to 1·26)	10·1 (8·24 to 12·6)	1·77 (1·50 to 2·13)	1·65 (1·35 to 2·00)	-6·65 (-16·4 to 4·51)	1·15 (0·977 to 1·38)
	50-69 years	18·7 (13·7 to 23·6)	16·8 (12·8 to 21·5)	-10·1 (-18·0 to -0·873)	14·9 (11·0 to 18·9)	1·41 (1·25 to 1·58)	1·51 (1·19 to 1·82)	7·12 (-9·93 to 25·3)	0·918 (0·813 to 1·03)
	70+ years	23·4 (18·9 to 29·1)	21·1 (17·0 to 26·9)	-9·98 (-17·7 to -2·15)	18·7 (15·2 to 23·3)	1·04 (0·953 to 1·14)	1·30 (1·06 to 1·54)	24·8 (7·95 to 43·4)	0·678 (0·620 to 0·742)
Saint Vincent and the Grenadines	All Ages	17·3 (15·1 to 20·0)	15·0 (12·8 to 17·2)	-13·6 (-18·6 to -7·96)	13·8 (12·0 to 16·0)	3·41 (2·87 to 4·10)	2·70 (2·27 to 3·27)	-20·6 (-25·9 to -12·0)	2·22 (1·86 to 2·66)
	Under 5	7·31 (5·90 to 9·45)	6·58 (5·07 to 8·61)	-9·86 (-23·2 to 5·78)	5·85 (4·72 to 7·56)	0·0281 (0·0216 to 0·0369)	0·0145 (0·0105 to 0·0190)	-48·5 (-56·2 to -39·2)	0·0183 (0·0141 to 0·0240)
	5-14 years	7·11 (4·61 to 10·2)	5·93 (3·95 to 8·68)	-16·3 (-27·4 to -4·73)	5·69 (3·69 to 8·15)	0·0314 (0·0255 to 0·0384)	0·0213 (0·0171 to 0·0264)	-32·3 (-38·5 to -25·8)	0·0204 (0·0166 to 0·0250)
	15-49 years	18·4 (15·1 to 22·6)	15·5 (12·5 to 19·6)	-15·7 (-21·5 to -8·92)	14·7 (12·1 to 18·1)	1·60 (1·25 to 2·07)	1·12 (0·843 to 1·48)	-30·1 (-35·2 to -24·0)	1·04 (0·810 to 1·34)
	50-69 years	24·3 (18·0 to 31·7)	20·2 (15·0 to 26·2)	-16·6 (-24·2 to -8·40)	19·4 (14·4 to 25·3)	1·03 (0·871 to 1·24)	0·927 (0·787 to 1·13)	-10·0 (-18·5 to 4·00)	0·671 (0·566 to 0·806)
	70+ years	27·5 (21·4 to 35·4)	22·4 (17·3 to 28·4)	-18·4 (-27·9 to -6·53)	22·0 (17·1 to 28·3)	0·716 (0·651 to 0·787)	0·621 (0·559 to 0·694)	-13·2 (-22·0 to -2·20)	0·465 (0·423 to 0·512)
Suriname	All Ages	21·4 (18·6 to 24·6)	19·5 (17·2 to 22·5)	-8·94 (-13·4 to -2·73)	17·1 (14·9 to 19·7)	18·0 (13·7 to 23·0)	16·7 (12·1 to 22·7)	-6·96 (-21·7 to 17·7)	11·7 (8·93 to 14·9)
	Under 5	8·28 (6·68 to 10·2)	7·17 (5·84 to 8·83)	-13·2 (-22·6 to -4·09)	6·63 (5·35 to 8·16)	0·341 (0·248 to 0·469)	0·278 (0·193 to 0·383)	-18·3 (-33·5 to -1·21)	0·222 (0·161 to 0·305)
	5-14 years	6·70 (4·58 to 9·49)	6·00 (4·05 to 8·20)	-10·2 (-22·6 to 3·89)	5·36 (3·66 to 7·59)	0·208 (0·163 to 0·268)	0·188 (0·144 to 0·245)	-9·34 (-24·1 to 8·85)	0·135 (0·106 to 0·174)

eTable 3. Percent change from 2015 to 2021 in age-standardised all-form tuberculosis incidence rate per 100,000 population and in deaths due to all-form tuberculosis by age for 204 countries and territories.

Location	Age group	2015 Rate	2021 Rate	Incidence Percent Change	Incidence Milestone	2015 Deaths	2021 Deaths	Mortality Percent Change	Mortality Milestone
	15-49 years	26·1 (21·6 to 31·9)	23·3 (19·5 to 29·0)	-10·9 (-16·2 to -2·47)	20·9 (17·3 to 25·5)	9·41 (6·75 to 12·8)	8·25 (5·90 to 11·5)	-12·1 (-24·9 to 8·76)	6·12 (4·39 to 8·33)
	50-69 years	26·3 (20·4 to 33·3)	24·3 (18·1 to 29·9)	-7·83 (-15·2 to -0·256)	21·1 (16·3 to 26·6)	5·35 (4·19 to 6·74)	5·55 (4·14 to 7·71)	3·75 (-14·9 to 33·3)	3·48 (2·72 to 4·38)
	70+ years	30·1 (24·1 to 38·0)	26·5 (21·3 to 32·2)	-11·8 (-20·7 to -3·04)	24·0 (19·3 to 30·4)	2·66 (2·14 to 3·20)	2·43 (1·73 to 3·27)	-8·47 (-33·1 to 22·5)	1·73 (1·39 to 2·08)
Trinidad and Tobago	All Ages	14·5 (12·6 to 16·8)	12·5 (10·8 to 14·8)	-13·7 (-20·0 to -8·77)	11·6 (10·1 to 13·5)	27·0 (22·1 to 33·9)	26·2 (20·6 to 34·0)	-2·82 (-16·6 to 15·9)	17·5 (14·4 to 22·0)
	Under 5	4·86 (3·82 to 6·24)	4·28 (3·48 to 5·49)	-11·7 (-22·3 to -0·792)	3·89 (3·06 to 4·99)	0·220 (0·166 to 0·289)	0·156 (0·118 to 0·206)	-28·9 (-36·8 to -16·0)	0·143 (0·108 to 0·188)
	5-14 years	3·17 (2·06 to 4·47)	3·02 (2·02 to 4·22)	-4·51 (-16·9 to 8·39)	2·54 (1·65 to 3·58)	0·151 (0·117 to 0·200)	0·134 (0·107 to 0·169)	-11·0 (-20·1 to -1·58)	0·0979 (0·0761 to 0·130)
	15-49 years	15·8 (12·8 to 19·3)	13·5 (10·9 to 16·9)	-14·5 (-22·4 to -7·94)	12·6 (10·2 to 15·5)	12·5 (9·46 to 17·1)	11·7 (8·59 to 15·7)	-6·51 (-16·1 to 7·11)	8·15 (6·15 to 11·1)
	50-69 years	20·9 (15·9 to 26·0)	17·5 (13·1 to 21·4)	-16·5 (-26·2 to -8·88)	16·8 (12·7 to 20·8)	9·99 (8·45 to 12·2)	9·59 (7·54 to 12·1)	-3·96 (-22·7 to 17·7)	6·50 (5·49 to 7·96)
	70+ years	17·3 (13·2 to 21·4)	14·9 (11·8 to 19·1)	-13·8 (-24·4 to -3·41)	13·8 (10·6 to 17·1)	4·10 (3·75 to 4·59)	4·63 (3·65 to 5·74)	13·0 (-9·06 to 39·6)	2·66 (2·43 to 2·99)
United States Virgin Islands	All Ages	6·17 (5·41 to 7·13)	5·88 (5·04 to 6·79)	-4·62 (-9·04 to 0·295)	4·94 (4·33 to 5·71)	0·959 (0·724 to 1·22)	0·866 (0·601 to 1·19)	-9·83 (-26·9 to 5·56)	0·624 (0·470 to 0·791)
	Under 5	2·18 (1·68 to 2·82)	1·88 (1·53 to 2·36)	-13·2 (-22·2 to -1·90)	1·74 (1·34 to 2·26)	0·00362 (0·00254 to 0·00520)	0·00192 (0·00137 to 0·00267)	-46·2 (-61·0 to -24·8)	0·00235 (0·00165 to 0·00338)
	5-14 years	1·23 (0·807 to 1·74)	1·06 (0·712 to 1·52)	-13·2 (-24·7 to -1·03)	0·982 (0·645 to 1·40)	0·00287 (0·00218 to 0·00383)	0·00160 (0·00114 to 0·00218)	-44·2 (-55·3 to -32·5)	0·00187 (0·00142 to 0·00249)
	15-49 years	5·88 (4·79 to 7·24)	5·24 (4·43 to 6·62)	-10·7 (-18·0 to -5·07)	4·70 (3·83 to 5·80)	0·393 (0·287 to 0·529)	0·309 (0·215 to 0·444)	-21·5 (-34·8 to -8·63)	0·256 (0·187 to 0·344)
	50-69 years	8·36 (6·26 to 10·6)	7·68 (5·93 to 9·89)	-8·06 (-16·2 to -0·451)	6·69 (5·01 to 8·50)	0·305 (0·219 to 0·409)	0·264 (0·172 to 0·379)	-13·3 (-32·6 to 9·70)	0·198 (0·142 to 0·266)
	70+ years	9·56 (7·34 to 12·3)	8·70 (6·70 to 11·0)	-8·86 (-16·9 to -0·0647)	7·65 (5·87 to 9·81)	0·255 (0·196 to 0·314)	0·289 (0·200 to 0·404)	13·3 (-7·90 to 36·1)	0·165 (0·128 to 0·204)
Central Latin America	All Ages	22·2 (19·9 to 24·9)	22·3 (19·7 to 25·2)	0·0587 (-2·77 to 3·19)	17·8 (15·9 to 19·9)	7530 (6830 to 8320)	7720 (6690 to 9040)	2·45 (-4·96 to 12·1)	4890 (4440 to 5410)
	Under 5	5·32 (4·42 to 6·56)	4·46 (3·69 to 5·48)	-16·2 (-20·6 to -12·3)	4·26 (3·54 to 5·25)	128 (106 to 147)	92·6 (73·6 to 114)	-27·5 (-35·2 to -16·5)	83·0 (69·2 to 95·8)
	5-14 years	4·45 (3·14 to 6·11)	4·24 (2·85 to 6·03)	-4·79 (-12·5 to 2·06)	3·56 (2·51 to 4·89)	86·5 (76·2 to 98·7)	69·5 (58·9 to 80·4)	-19·6 (-25·6 to -12·5)	56·2 (49·6 to 64·1)
	15-49 years	23·4 (19·8 to 28·0)	23·1 (19·4 to 28·5)	-1·25 (-4·52 to 2·27)	18·7 (15·8 to 22·4)	3140 (2650 to 3780)	3060 (2500 to 3700)	-2·60 (-8·17 to 4·81)	2040 (1720 to 2450)
	50-69 years	41·0 (32·1 to 50·4)	38·6 (29·3 to 48·2)	-5·97 (-10·7 to -1·10)	32·8 (25·7 to 40·3)	2360 (2190 to 2530)	2600 (2250 to 3020)	10·2 (0·399 to 23·0)	1530 (1430 to 1640)
	70+ years	55·2 (45·3 to 67·8)	46·0 (37·1 to 57·3)	-16·7 (-19·5 to -14·0)	44·2 (36·2 to 54·2)	1820 (1680 to 1920)	1900 (1690 to 2170)	4·31 (-4·95 to 15·5)	1180 (1090 to 1250)
Colombia	All Ages	24·8 (21·8 to 28·2)	22·6 (20·0 to 26·5)	-8·77 (-13·1 to -3·98)	19·8 (17·5 to 22·5)	1180 (1050 to 1330)	1280 (1080 to 1510)	9·07 (-3·56 to 22·5)	764 (680 to 866)

eTable 3. Percent change from 2015 to 2021 in age-standardised all-form tuberculosis incidence rate per 100,000 population and in deaths due to all-form tuberculosis by age for 204 countries and territories.

Location	Age group	2015 Rate	2021 Rate	Incidence Percent Change	Incidence Milestone	2015 Deaths	2021 Deaths	Mortality Percent Change	Mortality Milestone
	Under 5	5·78 (4·73 to 6·97)	4·41 (3·47 to 5·66)	-23·6 (-32·8 to -14·6)	4·62 (3·78 to 5·57)	15·0 (11·7 to 19·6)	11·6 (8·38 to 15·4)	-22·2 (-38·7 to -4·40)	9·77 (7·60 to 12·7)
	5-14 years	3·54 (2·38 to 4·63)	2·89 (2·04 to 3·99)	-18·2 (-28·7 to -3·69)	2·83 (1·90 to 3·70)	9·21 (7·96 to 11·2)	7·59 (6·28 to 9·04)	-17·4 (-28·5 to -4·48)	5·98 (5·18 to 7·28)
	15-49 years	24·8 (21·2 to 30·8)	22·0 (18·0 to 27·3)	-11·3 (-17·5 to -4·48)	19·9 (16·9 to 24·6)	419 (340 to 542)	403 (310 to 544)	-3·76 (-12·4 to 4·12)	272 (221 to 352)
	50-69 years	41·0 (32·0 to 50·9)	36·2 (27·3 to 47·9)	-11·7 (-17·9 to -3·30)	32·8 (25·6 to 40·7)	373 (334 to 413)	441 (359 to 521)	18·2 (2·15 to 38·6)	243 (217 to 269)
	70+ years	65·0 (52·3 to 79·3)	52·4 (41·6 to 64·5)	-19·3 (-26·0 to -11·6)	52·0 (41·8 to 63·4)	359 (327 to 387)	418 (359 to 481)	16·5 (-0·640 to 33·5)	233 (213 to 252)
Costa Rica	All Ages	9·58 (8·39 to 11·0)	9·15 (7·95 to 10·5)	-4·44 (-8·92 to 3·32)	7·66 (6·71 to 8·79)	58·3 (53·7 to 63·6)	65·9 (58·9 to 72·9)	12·9 (2·25 to 21·4)	37·9 (34·9 to 41·3)
	Under 5	2·46 (1·91 to 3·17)	2·01 (1·57 to 2·54)	-17·8 (-28·9 to -5·59)	1·96 (1·53 to 2·54)	0·415 (0·351 to 0·496)	0·285 (0·228 to 0·353)	-31·4 (-41·0 to -22·7)	0·270 (0·228 to 0·323)
	5-14 years	1·58 (1·09 to 2·13)	1·38 (0·921 to 1·96)	-12·2 (-26·1 to 1·38)	1·26 (0·869 to 1·70)	0·358 (0·300 to 0·425)	0·306 (0·255 to 0·370)	-14·3 (-23·0 to -7·02)	0·232 (0·195 to 0·276)
	15-49 years	9·41 (7·80 to 11·6)	8·52 (7·15 to 10·7)	-9·33 (-15·8 to -1·68)	7·52 (6·24 to 9·30)	19·0 (16·0 to 22·8)	20·7 (17·3 to 24·8)	8·98 (0·961 to 16·3)	12·4 (10·4 to 14·8)
	50-69 years	16·1 (12·4 to 20·0)	15·2 (11·6 to 19·1)	-5·34 (-13·7 to 4·66)	12·9 (9·93 to 16·0)	20·1 (18·4 to 22·0)	24·5 (21·1 to 27·3)	21·6 (6·22 to 33·2)	13·1 (11·9 to 14·3)
	70+ years	24·2 (19·5 to 29·6)	21·0 (16·8 to 25·8)	-13·1 (-20·7 to -3·71)	19·3 (15·6 to 23·7)	18·4 (16·5 to 19·9)	20·1 (17·4 to 23·1)	9·22 (-3·35 to 21·0)	12·0 (10·7 to 12·9)
El Salvador	All Ages	44·4 (39·3 to 50·5)	38·8 (33·9 to 44·3)	-12·4 (-16·0 to -7·39)	35·5 (31·5 to 40·4)	304 (261 to 354)	253 (199 to 334)	-17·0 (-30·7 to 2·36)	197 (170 to 230)
	Under 5	10·6 (8·49 to 13·4)	8·24 (6·59 to 10·6)	-22·4 (-33·9 to -8·72)	8·52 (6·79 to 10·7)	4·65 (3·81 to 5·62)	2·60 (2·08 to 3·42)	-43·9 (-54·8 to -29·2)	3·02 (2·48 to 3·66)
	5-14 years	10·3 (7·21 to 14·5)	7·95 (5·28 to 11·1)	-23·0 (-32·5 to -11·4)	8·28 (5·77 to 11·6)	3·99 (3·28 to 4·83)	2·67 (2·13 to 3·47)	-33·0 (-45·0 to -20·3)	2·59 (2·13 to 3·14)
	15-49 years	50·0 (41·3 to 61·5)	42·4 (34·9 to 51·7)	-15·2 (-20·5 to -9·16)	40·0 (33·0 to 49·2)	128 (105 to 157)	94·8 (73·0 to 128)	-26·0 (-37·4 to -11·2)	83·2 (68·0 to 102)
	50-69 years	75·7 (58·6 to 92·8)	66·7 (50·2 to 83·9)	-12·0 (-18·2 to -5·05)	60·6 (46·9 to 74·2)	79·6 (66·9 to 93·7)	74·9 (54·1 to 105)	-6·06 (-22·5 to 23·1)	51·7 (43·5 to 60·9)
	70+ years	101 (83·2 to 124)	86·1 (71·1 to 107)	-14·9 (-21·2 to -8·07)	81·0 (66·5 to 98·9)	87·6 (72·5 to 101)	77·6 (58·9 to 99·3)	-11·4 (-29·2 to 8·32)	56·9 (47·1 to 65·9)
Guatemala	All Ages	21·1 (18·7 to 23·4)	19·0 (17·2 to 21·5)	-9·54 (-16·0 to -4·30)	16·8 (15·0 to 18·7)	605 (533 to 687)	581 (497 to 680)	-3·82 (-12·5 to 6·61)	393 (347 to 446)
	Under 5	8·22 (6·67 to 10·2)	6·07 (4·89 to 7·37)	-26·0 (-36·9 to -16·8)	6·57 (5·33 to 8·13)	24·6 (20·7 to 28·4)	13·7 (11·0 to 16·1)	-44·2 (-53·6 to -34·9)	16·0 (13·4 to 18·4)
	5-14 years	5·80 (4·11 to 7·66)	4·73 (3·43 to 6·42)	-18·3 (-29·3 to -3·08)	4·64 (3·29 to 6·13)	12·3 (10·5 to 14·5)	8·92 (7·33 to 10·4)	-27·4 (-33·0 to -21·5)	7·98 (6·80 to 9·42)
	15-49 years	23·9 (20·4 to 28·4)	20·7 (17·5 to 25·1)	-13·5 (-19·8 to -7·40)	19·2 (16·3 to 22·7)	294 (248 to 354)	274 (226 to 328)	-6·96 (-13·5 to 1·76)	191 (161 to 230)
	50-69 years	46·4 (38·6 to 56·5)	40·8 (32·1 to 50·8)	-12·2 (-21·6 to -5·38)	37·1 (30·9 to 45·2)	172 (153 to 195)	172 (147 to 201)	0·188 (-12·0 to 15·7)	112 (99·4 to 127)

eTable 3. Percent change from 2015 to 2021 in age-standardised all-form tuberculosis incidence rate per 100,000 population and in deaths due to all-form tuberculosis by age for 204 countries and territories.

Location	Age group	2015 Rate	2021 Rate	Incidence Percent Change	Incidence Milestone	2015 Deaths	2021 Deaths	Mortality Percent Change	Mortality Milestone
	70+ years	52·6 (42·0 to 64·3)	44·1 (35·1 to 54·2)	-16·0 (-22·4 to -6·56)	42·0 (33·6 to 51·4)	101 (94·7 to 108)	113 (98·4 to 128)	11·3 (-2·31 to 27·7)	65·8 (61·6 to 70·1)
Honduras	All Ages	38·6 (34·8 to 42·5)	34·3 (30·5 to 38·7)	-11·1 (-15·4 to -6·73)	30·9 (27·8 to 34·0)	780 (549 to 1020)	744 (579 to 982)	-4·29 (-20·0 to 17·3)	507 (357 to 662)
	Under 5	11·2 (8·94 to 13·7)	8·87 (7·16 to 10·6)	-20·3 (-29·7 to -7·77)	8·93 (7·15 to 10·9)	17·4 (13·2 to 22·9)	13·0 (9·91 to 17·5)	-24·8 (-37·6 to -6·83)	11·3 (8·61 to 14·9)
	5-14 years	9·95 (6·79 to 13·5)	7·73 (5·18 to 10·8)	-22·1 (-32·4 to -11·6)	7·96 (5·43 to 10·8)	11·6 (7·75 to 15·6)	8·54 (5·76 to 11·3)	-25·5 (-40·7 to -5·75)	7·51 (5·04 to 10·1)
	15-49 years	39·8 (32·9 to 47·2)	34·1 (28·6 to 40·5)	-14·2 (-19·7 to -8·35)	31·8 (26·4 to 37·7)	234 (160 to 318)	236 (160 to 327)	2·15 (-20·1 to 42·6)	152 (104 to 207)
	50-69 years	100 (82·2 to 123)	88·6 (70·5 to 109)	-11·8 (-19·3 to -3·33)	80·4 (65·7 to 98·6)	287 (214 to 365)	277 (202 to 381)	-3·31 (-20·1 to 21·6)	186 (139 to 237)
	70+ years	146 (117 to 183)	121 (96·6 to 154)	-16·9 (-24·3 to -8·29)	117 (93·8 to 146)	230 (155 to 311)	209 (146 to 273)	-8·51 (-26·7 to 5·59)	150 (101 to 202)
Mexico	All Ages	17·4 (15·4 to 19·9)	19·4 (16·8 to 22·7)	11·1 (7·21 to 15·9)	13·9 (12·3 to 15·9)	3170 (2910 to 3540)	3190 (2780 to 3670)	0·577 (-8·15 to 10·0)	2060 (1890 to 2300)
	Under 5	3·21 (2·63 to 4·09)	2·88 (2·35 to 3·64)	-10·4 (-15·5 to -3·55)	2·57 (2·10 to 3·27)	39·4 (32·5 to 45·9)	31·2 (24·7 to 38·4)	-20·8 (-30·9 to -4·91)	25·6 (21·1 to 29·9)
	5-14 years	3·37 (2·25 to 4·80)	3·79 (2·34 to 5·54)	12·1 (1·54 to 22·9)	2·69 (1·80 to 3·84)	33·3 (29·4 to 38·1)	28·2 (25·0 to 32·3)	-15·1 (-20·6 to -8·33)	21·6 (19·1 to 24·8)
	15-49 years	17·8 (15·0 to 21·8)	20·2 (16·5 to 25·1)	13·5 (7·46 to 19·2)	14·2 (12·0 to 17·4)	1440 (1250 to 1730)	1430 (1210 to 1710)	-0·857 (-7·42 to 6·58)	938 (810 to 1120)
	50-69 years	35·2 (26·9 to 44·3)	34·8 (26·4 to 45·8)	-1·25 (-6·80 to 5·09)	28·2 (21·5 to 35·4)	986 (927 to 1070)	1060 (917 to 1210)	7·41 (-4·69 to 20·0)	641 (603 to 693)
	70+ years	43·1 (34·3 to 54·3)	36·1 (28·2 to 46·1)	-16·4 (-19·3 to -12·7)	34·5 (27·5 to 43·4)	671 (640 to 696)	642 (577 to 709)	-4·26 (-14·0 to 6·01)	436 (416 to 452)
Nicaragua	All Ages	32·8 (29·0 to 37·2)	29·6 (25·7 to 33·8)	-9·98 (-14·9 to -4·99)	26·3 (23·2 to 29·7)	260 (237 to 297)	238 (186 to 302)	-8·21 (-23·3 to 10·4)	169 (154 to 193)
	Under 5	9·51 (7·41 to 11·8)	7·37 (5·97 to 9·16)	-22·3 (-33·6 to -12·3)	7·61 (5·93 to 9·43)	7·72 (5·80 to 9·52)	4·66 (3·42 to 6·06)	-39·5 (-50·2 to -22·4)	5·02 (3·77 to 6·19)
	5-14 years	7·59 (5·24 to 10·3)	6·07 (4·14 to 8·44)	-20·0 (-29·3 to -9·00)	6·07 (4·19 to 8·28)	3·68 (3·09 to 4·71)	1·89 (1·50 to 2·30)	-48·5 (-57·2 to -38·0)	2·39 (2·01 to 3·06)
	15-49 years	36·2 (29·6 to 44·1)	31·9 (25·9 to 39·8)	-11·8 (-18·8 to -4·58)	29·0 (23·7 to 35·3)	96·8 (83·9 to 110)	84·2 (64·9 to 104)	-13·0 (-27·6 to 3·75)	62·9 (54·6 to 71·6)
	50-69 years	68·5 (53·0 to 84·0)	59·2 (45·1 to 74·1)	-13·6 (-21·2 to -5·28)	54·8 (42·4 to 67·2)	80·1 (69·1 to 93·3)	79·2 (59·4 to 110)	-0·968 (-22·1 to 22·4)	52·0 (44·9 to 60·7)
	70+ years	95·0 (76·8 to 117)	78·2 (62·8 to 95·6)	-17·5 (-24·7 to -8·37)	76·0 (61·4 to 93·4)	71·4 (61·5 to 85·9)	68·4 (52·3 to 87·4)	-4·10 (-21·9 to 17·7)	46·4 (40·0 to 55·9)
Panama	All Ages	41·5 (37·1 to 47·4)	35·8 (31·4 to 42·1)	-13·9 (-18·4 to -8·82)	33·2 (29·7 to 37·9)	237 (207 to 271)	209 (168 to 246)	-11·6 (-24·9 to 0·967)	154 (135 to 176)
	Under 5	13·6 (11·1 to 17·1)	9·41 (7·55 to 11·9)	-30·6 (-39·6 to -21·2)	10·9 (8·85 to 13·7)	6·71 (5·34 to 7·77)	4·04 (3·12 to 5·03)	-39·7 (-48·5 to -29·2)	4·36 (3·47 to 5·05)
	5-14 years	9·96 (7·15 to 13·6)	8·01 (5·55 to 10·8)	-19·5 (-30·6 to -9·15)	7·97 (5·72 to 10·9)	2·94 (2·46 to 3·46)	2·12 (1·72 to 2·58)	-28·0 (-36·0 to -19·5)	1·91 (1·60 to 2·25)

eTable 3. Percent change from 2015 to 2021 in age-standardised all-form tuberculosis incidence rate per 100,000 population and in deaths due to all-form tuberculosis by age for 204 countries and territories.

Location	Age group	2015 Rate	2021 Rate	Incidence Percent Change	Incidence Milestone	2015 Deaths	2021 Deaths	Mortality Percent Change	Mortality Milestone
	15-49 years	48·7 (39·8 to 59·0)	40·8 (33·6 to 51·8)	-16·2 (-23·0 to -8·27)	39·0 (31·8 to 47·2)	98·5 (79·2 to 123)	83·5 (65·7 to 110)	-15·2 (-24·9 to -6·27)	64·0 (51·5 to 79·9)
	50-69 years	62·4 (47·7 to 76·3)	55·7 (41·0 to 69·9)	-10·7 (-18·3 to -0·751)	49·9 (38·2 to 61·1)	69·3 (62·0 to 77·0)	65·3 (51·9 to 79·3)	-5·65 (-23·3 to 13·0)	45·0 (40·3 to 50·1)
	70+ years	73·6 (59·1 to 91·1)	60·6 (47·7 to 74·5)	-17·5 (-27·7 to -9·95)	58·9 (47·3 to 72·8)	59·3 (53·3 to 65·2)	54·2 (44·0 to 62·8)	-8·44 (-25·6 to 7·79)	38·5 (34·6 to 42·4)
Venezuela (Bolivarian Republic of)	All Ages	26·3 (23·3 to 30·1)	27·2 (24·2 to 31·1)	3·48 (-2·14 to 9·78)	21·1 (18·6 to 24·0)	939 (846 to 1090)	1150 (912 to 1470)	22·8 (-0·977 to 53·9)	610 (550 to 706)
	Under 5	6·50 (5·27 to 8·24)	5·90 (4·74 to 7·48)	-9·00 (-21·9 to 5·14)	5·20 (4·22 to 6·59)	11·8 (10·3 to 13·9)	11·5 (8·84 to 14·7)	-2·50 (-20·5 to 21·1)	7·68 (6·69 to 9·07)
	5-14 years	4·88 (3·45 to 6·61)	4·87 (3·37 to 6·61)	0·0813 (-13·6 to 15·2)	3·90 (2·76 to 5·29)	9·21 (8·01 to 10·8)	9·27 (7·55 to 11·7)	0·592 (-13·2 to 19·0)	5·99 (5·21 to 7·03)
	15-49 years	30·2 (25·3 to 36·8)	30·4 (25·2 to 37·4)	0·891 (-5·77 to 8·19)	24·1 (20·2 to 29·4)	404 (336 to 515)	430 (318 to 568)	6·12 (-12·2 to 26·1)	263 (219 to 335)
	50-69 years	40·9 (31·6 to 49·4)	40·4 (30·4 to 50·0)	-1·29 (-9·60 to 6·49)	32·7 (25·3 to 39·6)	293 (268 to 329)	409 (303 to 534)	39·5 (8·35 to 81·6)	191 (174 to 214)
	70+ years	54·9 (44·7 to 66·1)	49·1 (39·0 to 62·1)	-10·6 (-17·6 to -2·89)	43·9 (35·8 to 52·9)	220 (200 to 238)	294 (234 to 377)	33·6 (6·59 to 73·1)	143 (130 to 155)
Tropical Latin America	All Ages	41·5 (36·4 to 48·3)	38·4 (33·3 to 45·3)	-7·60 (-11·3 to -2·79)	33·2 (29·1 to 38·6)	9010 (7850 to 10400)	8360 (7270 to 9580)	-7·19 (-9·90 to -3·70)	5850 (5100 to 6770)
	Under 5	6·98 (5·81 to 8·49)	6·38 (5·28 to 7·97)	-8·64 (-14·4 to -3·02)	5·58 (4·65 to 6·79)	168 (139 to 204)	113 (92·2 to 138)	-32·9 (-42·2 to -23·4)	109 (90·7 to 132)
	5-14 years	5·43 (3·71 to 7·65)	6·08 (3·99 to 8·72)	11·8 (-0·449 to 22·3)	4·34 (2·97 to 6·12)	68·9 (56·7 to 81·9)	49·7 (39·8 to 61·3)	-27·9 (-32·9 to -22·5)	44·8 (36·9 to 53·2)
	15-49 years	49·2 (41·6 to 60·3)	46·0 (37·8 to 57·2)	-6·47 (-12·1 to -0·802)	39·3 (33·3 to 48·2)	4200 (3390 to 5220)	3700 (3030 to 4500)	-11·9 (-14·6 to -8·66)	2730 (2210 to 3390)
	50-69 years	59·8 (46·0 to 74·8)	51·8 (38·6 to 68·5)	-13·6 (-19·8 to -6·78)	47·8 (36·8 to 59·8)	3120 (2820 to 3460)	3050 (2710 to 3450)	-1·99 (-5·73 to 2·44)	2020 (1830 to 2250)
	70+ years	58·9 (46·4 to 73·2)	46·9 (36·5 to 59·7)	-20·5 (-24·6 to -15·6)	47·2 (37·1 to 58·6)	1460 (1310 to 1530)	1450 (1320 to 1540)	-0·661 (-3·91 to 2·69)	946 (851 to 997)
Brazil	All Ages	41·7 (36·5 to 48·5)	38·6 (33·4 to 45·5)	-7·48 (-11·2 to -2·53)	33·4 (29·2 to 38·8)	8640 (7520 to 10000)	8030 (7010 to 9230)	-7·05 (-10·1 to -3·52)	5620 (4890 to 6530)
	Under 5	6·87 (5·72 to 8·38)	6·33 (5·24 to 7·94)	-7·91 (-13·9 to -2·21)	5·50 (4·57 to 6·71)	154 (128 to 188)	104 (85·1 to 128)	-32·7 (-42·5 to -22·6)	100 (83·1 to 122)
	5-14 years	5·33 (3·64 to 7·52)	6·07 (3·98 to 8·73)	13·6 (0·507 to 25·2)	4·27 (2·91 to 6·01)	64·5 (52·6 to 77·4)	46·1 (36·9 to 57·6)	-28·5 (-33·9 to -23·1)	41·9 (34·2 to 50·3)
	15-49 years	49·4 (41·8 to 60·6)	46·3 (37·9 to 57·6)	-6·25 (-12·1 to -0·370)	39·5 (33·4 to 48·4)	4040 (3260 to 5040)	3560 (2920 to 4350)	-11·8 (-14·7 to -8·76)	2630 (2120 to 3280)
	50-69 years	59·7 (45·9 to 74·7)	51·7 (38·5 to 68·6)	-13·5 (-20·1 to -6·52)	47·8 (36·7 to 59·8)	3000 (2710 to 3350)	2940 (2620 to 3330)	-1·90 (-5·77 to 2·02)	1950 (1760 to 2180)
	70+ years	58·7 (46·1 to 72·8)	46·6 (36·2 to 59·4)	-20·6 (-24·9 to -15·4)	46·9 (36·9 to 58·2)	1390 (1250 to 1470)	1380 (1260 to 1470)	-0·425 (-4·21 to 3·13)	901 (815 to 954)
Paraguay	All Ages	35·9 (31·2 to 41·4)	31·9 (27·6 to 37·1)	-11·3 (-16·4 to -5·50)	28·7 (24·9 to 33·1)	363 (311 to 425)	325 (254 to 408)	-10·2 (-25·6 to 9·20)	236 (202 to 276)

eTable 3. Percent change from 2015 to 2021 in age-standardised all-form tuberculosis incidence rate per 100,000 population and in deaths due to all-form tuberculosis by age for 204 countries and territories.

Location	Age group	2015 Rate	2021 Rate	Incidence Percent Change	Incidence Milestone	2015 Deaths	2021 Deaths	Mortality Percent Change	Mortality Milestone
	Under 5	9·52 (7·73 to 11·5)	7·50 (6·11 to 9·40)	-21·1 (-28·4 to -11·4)	7·62 (6·18 to 9·16)	13·7 (11·1 to 17·1)	8·90 (6·80 to 11·2)	-34·7 (-45·9 to -14·9)	8·90 (7·22 to 11·1)
	5-14 years	7·66 (5·44 to 10·6)	6·37 (4·25 to 8·98)	-16·5 (-28·1 to -5·40)	6·12 (4·35 to 8·50)	4·36 (3·66 to 5·21)	3·55 (2·82 to 4·36)	-18·4 (-33·3 to -0·918)	2·84 (2·38 to 3·38)
	15-49 years	42·8 (35·5 to 52·2)	37·3 (30·6 to 46·1)	-12·8 (-18·2 to -6·23)	34·2 (28·4 to 41·8)	159 (130 to 185)	136 (103 to 163)	-14·6 (-27·5 to 1·33)	103 (84·8 to 120)
	50-69 years	62·8 (47·6 to 77·7)	53·1 (38·8 to 66·0)	-15·4 (-22·9 to -7·62)	50·2 (38·1 to 62·2)	116 (97·9 to 144)	111 (85·1 to 149)	-3·99 (-25·2 to 22·4)	75·3 (63·7 to 93·8)
	70+ years	71·6 (57·9 to 87·7)	59·1 (47·6 to 73·2)	-17·4 (-24·1 to -10·0)	57·3 (46·3 to 70·2)	69·8 (58·6 to 81·6)	66·1 (49·6 to 84·2)	-5·08 (-24·7 to 16·4)	45·4 (38·1 to 53·1)
North Africa and Middle East	All Ages	31·5 (27·7 to 35·8)	27·1 (23·8 to 31·3)	-13·9 (-16·1 to -12·1)	25·2 (22·2 to 28·6)	22600 (18700 to 31700)	19600 (15600 to 26900)	-12·9 (-21·2 to -2·45)	14700 (12200 to 20600)
	Under 5	15·1 (12·5 to 18·5)	12·0 (9·39 to 15·0)	-20·8 (-26·0 to -15·6)	12·1 (9·97 to 14·8)	1660 (1260 to 2080)	1030 (767 to 1310)	-37·8 (-47·4 to -27·9)	1080 (821 to 1350)
	5-14 years	15·6 (11·4 to 21·9)	12·1 (8·62 to 16·8)	-22·3 (-28·3 to -16·2)	12·4 (9·11 to 17·5)	612 (481 to 829)	424 (337 to 582)	-30·5 (-38·5 to -20·7)	398 (313 to 539)
	15-49 years	31·3 (25·8 to 37·7)	26·5 (21·7 to 32·3)	-15·3 (-17·7 to -13·3)	25·1 (20·7 to 30·2)	7960 (6400 to 10200)	6740 (5370 to 8920)	-15·4 (-25·4 to -5·34)	5180 (4160 to 6630)
	50-69 years	54·5 (42·0 to 68·1)	46·8 (35·6 to 58·4)	-14·1 (-16·7 to -11·0)	43·6 (33·6 to 54·5)	5900 (4870 to 8700)	5790 (4500 to 8260)	-1·92 (-12·1 to 10·9)	3840 (3170 to 5650)
	70+ years	109 (87·6 to 134)	88·8 (71·3 to 111)	-18·4 (-21·6 to -15·6)	86·9 (70·1 to 108)	6410 (5020 to 10800)	5660 (4280 to 8480)	-11·5 (-21·7 to 0·809)	4170 (3260 to 7010)
Afghanistan	All Ages	130 (112 to 150)	107 (90·9 to 123)	-17·6 (-23·6 to -11·1)	104 (89·7 to 120)	7500 (5620 to 11600)	6590 (4970 to 9890)	-11·7 (-22·0 to 3·49)	4870 (3650 to 7520)
	Under 5	62·4 (50·1 to 77·6)	47·6 (37·2 to 60·7)	-23·7 (-34·3 to -13·7)	50·0 (40·1 to 62·1)	874 (621 to 1210)	644 (462 to 875)	-25·6 (-45·2 to -6·47)	568 (404 to 784)
	5-14 years	66·8 (47·8 to 96·0)	55·0 (37·6 to 77·4)	-17·3 (-32·5 to -2·94)	53·4 (38·2 to 76·8)	337 (243 to 543)	246 (172 to 377)	-26·5 (-41·4 to -10·8)	219 (158 to 353)
	15-49 years	157 (124 to 194)	129 (103 to 159)	-17·7 (-25·4 to -10·6)	126 (98·8 to 155)	3280 (2410 to 4720)	2930 (2170 to 4320)	-10·4 (-27·2 to 8·51)	2130 (1570 to 3070)
	50-69 years	389 (302 to 490)	273 (201 to 337)	-29·6 (-38·3 to -19·8)	311 (242 to 392)	1570 (1100 to 2810)	1670 (1160 to 2610)	7·59 (-11·2 to 31·6)	1020 (716 to 1830)
	70+ years	540 (418 to 675)	472 (370 to 613)	-12·5 (-21·4 to -0·914)	432 (334 to 540)	1430 (889 to 2940)	1100 (715 to 2090)	-22·4 (-35·5 to -4·84)	930 (578 to 1910)
Algeria	All Ages	47·2 (40·8 to 54·6)	41·3 (36·6 to 48·5)	-12·6 (-16·6 to -8·31)	37·8 (32·6 to 43·7)	1740 (1320 to 2260)	1610 (1260 to 2120)	-7·49 (-25·7 to 14·3)	1130 (856 to 1470)
	Under 5	20·9 (15·9 to 26·0)	15·4 (11·4 to 19·8)	-26·5 (-36·9 to -17·2)	16·7 (12·7 to 20·8)	89·7 (64·2 to 121)	41·7 (28·7 to 59·8)	-53·3 (-64·6 to -41·4)	58·3 (41·7 to 79·0)
	5-14 years	20·5 (14·4 to 29·9)	16·1 (11·0 to 22·7)	-21·1 (-30·6 to -9·81)	16·4 (11·5 to 23·9)	24·7 (18·4 to 33·3)	19·9 (14·5 to 27·9)	-19·3 (-32·8 to 3·40)	16·1 (11·9 to 21·6)
	15-49 years	42·9 (34·4 to 53·6)	37·1 (30·0 to 46·2)	-13·5 (-19·6 to -6·93)	34·3 (27·5 to 42·9)	450 (344 to 667)	386 (298 to 527)	-13·9 (-31·0 to 4·60)	292 (224 to 433)
	50-69 years	87·3 (64·6 to 113)	76·6 (59·3 to 96·7)	-12·0 (-18·4 to -4·21)	69·8 (51·7 to 90·3)	435 (322 to 596)	437 (315 to 615)	0·760 (-22·1 to 36·2)	283 (209 to 388)

eTable 3. Percent change from 2015 to 2021 in age-standardised all-form tuberculosis incidence rate per 100,000 population and in deaths due to all-form tuberculosis by age for 204 countries and territories.

Location	Age group	2015 Rate	2021 Rate	Incidence Percent Change	Incidence Milestone	2015 Deaths	2021 Deaths	Mortality Percent Change	Mortality Milestone
	70+ years	182 (144 to 221)	158 (124 to 199)	-13·2 (-19·7 to -4·94)	146 (116 to 177)	742 (543 to 1000)	723 (536 to 962)	-2·16 (-23·8 to 21·1)	482 (353 to 650)
Bahrain	All Ages	14·1 (12·0 to 17·4)	13·9 (11·7 to 16·7)	-0·973 (-8·00 to 6·10)	11·2 (9·57 to 13·9)	11·8 (10·1 to 14·2)	11·7 (9·47 to 16·2)	-0·899 (-14·1 to 17·6)	7·67 (6·56 to 9·22)
	Under 5	5·09 (4·11 to 6·50)	4·60 (3·43 to 5·91)	-9·54 (-26·5 to 3·33)	4·07 (3·28 to 5·20)	0·250 (0·209 to 0·299)	0·144 (0·107 to 0·186)	-42·7 (-52·3 to -30·8)	0·163 (0·136 to 0·195)
	5-14 years	5·68 (3·80 to 8·05)	5·31 (3·68 to 7·43)	-6·16 (-17·6 to 7·12)	4·54 (3·04 to 6·44)	0·132 (0·106 to 0·173)	0·0929 (0·0690 to 0·128)	-29·7 (-40·4 to -12·7)	0·0859 (0·0688 to 0·112)
	15-49 years	14·2 (11·5 to 18·0)	13·7 (10·9 to 16·8)	-3·18 (-11·9 to 5·71)	11·4 (9·23 to 14·4)	5·46 (4·55 to 6·87)	4·56 (3·51 to 6·70)	-16·7 (-30·3 to 1·51)	3·55 (2·96 to 4·46)
	50-69 years	23·8 (17·6 to 30·3)	22·0 (17·0 to 27·7)	-7·30 (-15·2 to -0·166)	19·0 (14·1 to 24·2)	3·32 (2·76 to 4·23)	3·88 (3·07 to 5·19)	17·1 (-8·74 to 42·2)	2·16 (1·79 to 2·75)
	70+ years	60·4 (47·7 to 75·0)	50·4 (39·7 to 64·0)	-16·4 (-26·3 to -6·41)	48·3 (38·1 to 60·0)	2·63 (2·21 to 3·17)	3·03 (2·29 to 4·13)	15·3 (-4·23 to 39·1)	1·71 (1·43 to 2·06)
Egypt	All Ages	11·2 (10·1 to 12·4)	8·59 (7·61 to 9·58)	-23·7 (-27·5 to -19·0)	9·00 (8·09 to 9·90)	1150 (1030 to 1280)	850 (679 to 1050)	-26·0 (-40·3 to -7·25)	748 (669 to 832)
	Under 5	5·76 (4·58 to 7·08)	3·79 (3·01 to 4·84)	-34·0 (-43·3 to -21·6)	4·61 (3·67 to 5·66)	62·9 (46·8 to 79·5)	27·5 (19·7 to 34·5)	-55·8 (-69·0 to -36·8)	40·9 (30·4 to 51·7)
	5-14 years	4·90 (3·38 to 6·99)	3·31 (2·37 to 4·61)	-32·3 (-40·2 to -20·6)	3·92 (2·71 to 5·59)	20·9 (16·5 to 26·3)	14·1 (11·0 to 18·5)	-31·5 (-49·9 to -9·26)	13·6 (10·7 to 17·1)
	15-49 years	12·0 (9·96 to 14·0)	9·25 (7·63 to 11·1)	-23·2 (-27·9 to -17·3)	9·63 (7·97 to 11·2)	377 (320 to 420)	283 (222 to 365)	-24·6 (-41·7 to -5·81)	245 (208 to 273)
	50-69 years	21·9 (18·0 to 26·2)	17·0 (14·1 to 21·2)	-22·2 (-28·6 to -15·7)	17·5 (14·4 to 20·9)	465 (415 to 533)	340 (259 to 432)	-26·8 (-43·3 to -5·76)	302 (269 to 346)
	70+ years	36·2 (29·3 to 43·3)	27·4 (21·9 to 34·4)	-24·3 (-33·5 to -13·8)	28·9 (23·4 to 34·7)	226 (196 to 275)	186 (144 to 236)	-17·2 (-32·8 to -1·54)	147 (127 to 179)
Iran (Islamic Republic of)	All Ages	16·3 (14·5 to 18·3)	15·6 (14·1 to 17·6)	-4·07 (-6·98 to -0·803)	13·0 (11·6 to 14·6)	1220 (1080 to 1310)	963 (830 to 1150)	-21·2 (-27·7 to -8·39)	793 (702 to 852)
	Under 5	6·55 (5·37 to 8·45)	4·46 (3·63 to 5·73)	-32·0 (-36·5 to -28·3)	5·24 (4·29 to 6·76)	43·0 (37·7 to 47·2)	12·2 (10·4 to 13·7)	-71·6 (-74·6 to -68·7)	28·0 (24·5 to 30·7)
	5-14 years	8·06 (5·70 to 11·3)	6·21 (4·33 to 8·77)	-22·9 (-27·3 to -19·1)	6·45 (4·56 to 9·01)	21·3 (19·4 to 22·9)	14·3 (12·6 to 16·0)	-32·6 (-38·0 to -25·0)	13·8 (12·6 to 14·9)
	15-49 years	15·5 (12·9 to 18·5)	14·9 (12·4 to 17·9)	-3·81 (-7·99 to 0·580)	12·4 (10·3 to 14·8)	431 (384 to 468)	332 (279 to 391)	-22·9 (-28·6 to -11·1)	280 (249 to 304)
	50-69 years	24·9 (19·5 to 30·8)	23·9 (18·9 to 29·6)	-4·12 (-7·54 to -0·860)	19·9 (15·6 to 24·7)	303 (273 to 330)	262 (224 to 318)	-13·8 (-21·2 to 1·74)	197 (177 to 214)
	70+ years	52·2 (42·7 to 64·4)	48·6 (40·0 to 59·2)	-6·81 (-10·3 to -2·63)	41·7 (34·2 to 51·5)	422 (363 to 463)	342 (282 to 422)	-19·0 (-27·9 to -4·04)	274 (236 to 301)
Iraq	All Ages	27·5 (23·6 to 32·3)	22·2 (19·0 to 25·8)	-19·4 (-23·3 to -15·9)	22·0 (18·9 to 25·8)	969 (764 to 1210)	859 (630 to 1140)	-11·2 (-27·3 to 10·5)	630 (497 to 785)
	Under 5	11·2 (8·57 to 14·0)	7·73 (5·86 to 9·74)	-30·8 (-40·4 to -18·7)	8·96 (6·86 to 11·2)	33·1 (24·7 to 43·7)	15·9 (11·2 to 23·4)	-51·5 (-63·9 to -32·3)	21·5 (16·1 to 28·4)
	5-14 years	13·4 (9·29 to 19·4)	8·33 (5·86 to 11·7)	-37·6 (-45·8 to -29·2)	10·7 (7·44 to 15·5)	29·5 (24·8 to 37·1)	11·3 (8·77 to 15·6)	-61·5 (-70·0 to -52·5)	19·2 (16·1 to 24·1)

eTable 3. Percent change from 2015 to 2021 in age-standardised all-form tuberculosis incidence rate per 100,000 population and in deaths due to all-form tuberculosis by age for 204 countries and territories.

Location	Age group	2015 Rate	2021 Rate	Incidence Percent Change	Incidence Milestone	2015 Deaths	2021 Deaths	Mortality Percent Change	Mortality Milestone
	15-49 years	28·0 (22·6 to 35·0)	21·4 (17·3 to 26·7)	-23·8 (-28·3 to -18·2)	22·4 (18·1 to 28·0)	340 (258 to 451)	244 (174 to 362)	-28·1 (-43·5 to -4·13)	221 (168 to 293)
	50-69 years	63·0 (47·3 to 77·9)	51·8 (39·3 to 66·3)	-17·7 (-23·8 to -10·4)	50·4 (37·8 to 62·3)	309 (237 to 400)	310 (214 to 439)	0·687 (-22·5 to 31·0)	201 (154 to 260)
	70+ years	107 (85·9 to 134)	91·5 (72·7 to 117)	-14·3 (-25·3 to -5·14)	85·6 (68·7 to 107)	258 (206 to 325)	278 (206 to 390)	7·90 (-15·5 to 42·9)	168 (134 to 211)
Jordan	All Ages	5·51 (4·75 to 6·41)	5·28 (4·55 to 6·09)	-4·12 (-9·63 to 4·37)	4·41 (3·80 to 5·13)	37·0 (31·4 to 44·6)	48·3 (36·2 to 61·1)	30·4 (2·43 to 65·1)	24·1 (20·4 to 29·0)
	Under 5	2·05 (1·43 to 2·75)	1·72 (1·25 to 2·39)	-15·8 (-27·6 to -5·23)	1·64 (1·14 to 2·20)	1·15 (0·905 to 1·50)	0·838 (0·643 to 1·12)	-27·0 (-39·8 to -9·75)	0·748 (0·588 to 0·978)
	5-14 years	2·60 (1·72 to 3·81)	2·40 (1·58 to 3·64)	-7·65 (-20·2 to 9·26)	2·08 (1·38 to 3·05)	0·747 (0·598 to 0·918)	0·737 (0·559 to 0·926)	-0·770 (-20·4 to 19·1)	0·485 (0·389 to 0·597)
	15-49 years	5·55 (4·48 to 6·90)	4·99 (3·94 to 6·22)	-10·0 (-16·2 to -2·15)	4·44 (3·58 to 5·52)	11·0 (9·42 to 13·0)	13·5 (10·3 to 17·6)	22·4 (-2·12 to 54·8)	7·16 (6·12 to 8·48)
	50-69 years	12·2 (9·11 to 15·4)	10·6 (8·05 to 13·2)	-12·7 (-18·2 to -5·96)	9·77 (7·29 to 12·3)	11·1 (9·21 to 14·1)	16·3 (12·2 to 21·9)	47·2 (12·6 to 91·0)	7·22 (5·99 to 9·18)
	70+ years	24·0 (18·8 to 30·5)	21·4 (16·2 to 27·1)	-10·8 (-20·9 to -1·57)	19·2 (15·0 to 24·4)	13·0 (10·6 to 16·2)	16·9 (12·3 to 21·2)	30·0 (2·21 to 58·4)	8·46 (6·86 to 10·5)
Kuwait	All Ages	20·7 (17·5 to 24·0)	18·0 (15·2 to 21·5)	-13·3 (-18·4 to -8·73)	16·6 (14·0 to 19·2)	35·6 (33·4 to 38·8)	41·2 (34·5 to 48·8)	16·0 (-2·70 to 39·7)	23·1 (21·7 to 25·2)
	Under 5	4·70 (3·58 to 5·70)	3·90 (2·93 to 4·91)	-16·9 (-31·0 to -5·64)	3·76 (2·86 to 4·56)	0·643 (0·575 to 0·713)	0·291 (0·245 to 0·354)	-54·6 (-62·2 to -44·5)	0·418 (0·374 to 0·464)
	5-14 years	5·03 (3·47 to 7·29)	4·81 (3·15 to 6·97)	-4·48 (-15·7 to 5·40)	4·03 (2·77 to 5·83)	0·454 (0·408 to 0·515)	0·333 (0·283 to 0·385)	-26·5 (-39·0 to -11·8)	0·295 (0·265 to 0·334)
	15-49 years	22·5 (17·9 to 27·7)	18·1 (14·5 to 23·1)	-19·8 (-25·9 to -13·6)	18·0 (14·4 to 22·2)	12·5 (11·8 to 13·6)	12·3 (10·4 to 14·8)	-1·66 (-16·2 to 20·6)	8·15 (7·65 to 8·85)
	50-69 years	33·1 (25·2 to 41·6)	28·6 (21·8 to 36·2)	-13·4 (-20·8 to -3·68)	26·5 (20·2 to 33·3)	9·28 (8·45 to 10·1)	12·1 (9·69 to 15·0)	30·8 (3·21 to 65·9)	6·03 (5·49 to 6·59)
	70+ years	60·8 (49·5 to 73·1)	58·3 (47·7 to 72·1)	-4·04 (-13·0 to 6·02)	48·6 (39·6 to 58·5)	12·7 (11·1 to 14·1)	16·2 (13·6 to 19·3)	27·9 (5·91 to 53·5)	8·24 (7·23 to 9·19)
Lebanon	All Ages	14·4 (12·7 to 16·7)	13·1 (11·6 to 15·2)	-8·83 (-13·8 to -3·84)	11·5 (10·1 to 13·3)	64·9 (56·6 to 76·5)	60·6 (49·2 to 73·1)	-6·48 (-21·2 to 9·68)	42·2 (36·8 to 49·7)
	Under 5	4·44 (3·52 to 5·50)	3·60 (2·70 to 4·60)	-18·8 (-33·4 to -6·28)	3·55 (2·82 to 4·40)	0·567 (0·400 to 0·764)	0·376 (0·270 to 0·531)	-33·4 (-48·7 to -19·2)	0·369 (0·260 to 0·497)
	5-14 years	3·97 (2·78 to 5·56)	3·26 (2·25 to 4·87)	-17·7 (-30·9 to -6·56)	3·18 (2·22 to 4·45)	0·363 (0·275 to 0·455)	0·282 (0·223 to 0·350)	-21·7 (-38·1 to -1·55)	0·236 (0·179 to 0·296)
	15-49 years	12·0 (9·59 to 15·0)	10·3 (8·24 to 12·7)	-14·0 (-22·3 to -7·20)	9·58 (7·67 to 12·0)	13·0 (10·7 to 15·4)	11·4 (9·19 to 14·0)	-11·6 (-27·7 to 7·21)	8·44 (6·99 to 10·0)
	50-69 years	26·4 (19·6 to 33·0)	22·6 (17·6 to 28·1)	-14·4 (-21·6 to -5·87)	21·1 (15·6 to 26·4)	16·6 (13·5 to 19·7)	14·8 (12·4 to 17·7)	-10·4 (-26·2 to 11·9)	10·8 (8·80 to 12·8)
	70+ years	51·1 (41·5 to 63·7)	46·6 (37·3 to 58·5)	-8·76 (-17·3 to -0·997)	40·9 (33·2 to 51·0)	34·4 (28·7 to 41·9)	33·6 (26·1 to 40·8)	-1·90 (-19·0 to 17·8)	22·3 (18·7 to 27·2)
Libya	All Ages	29·1 (25·4 to 34·0)	29·0 (25·2 to 34·2)	-0·104 (-4·42 to 5·94)	23·3 (20·3 to 27·2)	146 (103 to 197)	169 (106 to 247)	14·9 (-6·78 to 39·1)	95·0 (66·8 to 128)

eTable 3. Percent change from 2015 to 2021 in age-standardised all-form tuberculosis incidence rate per 100,000 population and in deaths due to all-form tuberculosis by age for 204 countries and territories.

Location	Age group	2015 Rate	2021 Rate	Incidence Percent Change	Incidence Milestone	2015 Deaths	2021 Deaths	Mortality Percent Change	Mortality Milestone
	Under 5	13·0 (10·2 to 16·4)	12·1 (9·61 to 15·4)	-6·80 (-18·3 to 6·67)	10·4 (8·17 to 13·1)	4·91 (2·81 to 7·35)	3·82 (1·98 to 5·83)	-22·6 (-38·1 to -2·51)	3·19 (1·82 to 4·77)
	5-14 years	11·7 (8·18 to 16·4)	10·9 (7·68 to 15·2)	-6·35 (-17·7 to 4·72)	9·35 (6·55 to 13·1)	1·91 (1·30 to 2·54)	1·64 (1·04 to 2·34)	-14·4 (-33·3 to 4·87)	1·24 (0·843 to 1·65)
	15-49 years	28·1 (22·6 to 34·3)	26·2 (21·3 to 32·3)	-6·86 (-13·3 to 0·137)	22·5 (18·1 to 27·4)	54·2 (36·4 to 78·4)	57·3 (33·8 to 87·1)	5·07 (-17·1 to 31·0)	35·3 (23·7 to 51·0)
	50-69 years	53·7 (41·6 to 66·8)	50·3 (38·9 to 64·1)	-6·17 (-13·8 to 1·49)	42·9 (33·3 to 53·4)	38·7 (25·4 to 53·9)	53·1 (31·9 to 83·7)	36·1 (8·72 to 73·2)	25·2 (16·5 to 35·1)
	70+ years	106 (85·6 to 132)	103 (80·1 to 126)	-3·18 (-12·5 to 7·55)	85·1 (68·4 to 105)	46·4 (33·0 to 63·7)	52·9 (34·2 to 76·2)	14·4 (-11·9 to 42·9)	30·1 (21·5 to 41·4)
Morocco	All Ages	83·4 (72·7 to 95·1)	69·8 (60·6 to 81·2)	-16·2 (-21·0 to -11·3)	66·7 (58·2 to 76·1)	4170 (2710 to 8680)	3480 (2160 to 7680)	-16·8 (-32·3 to 0·526)	2710 (1760 to 5640)
	Under 5	31·0 (24·2 to 38·6)	21·9 (16·6 to 27·6)	-29·2 (-40·2 to -18·2)	24·8 (19·4 to 30·8)	101 (73·4 to 142)	38·2 (25·2 to 59·9)	-62·3 (-71·8 to -51·0)	65·7 (47·7 to 92·6)
	5-14 years	35·9 (25·0 to 51·9)	26·1 (18·5 to 37·1)	-26·9 (-37·0 to -14·3)	28·7 (20·0 to 41·5)	53·1 (40·6 to 76·4)	27·1 (20·9 to 38·7)	-48·5 (-59·6 to -31·8)	34·5 (26·4 to 49·6)
	15-49 years	77·2 (63·2 to 92·3)	60·8 (48·5 to 74·5)	-21·3 (-27·6 to -14·1)	61·8 (50·5 to 73·9)	1080 (740 to 1810)	741 (524 to 1360)	-31·7 (-46·1 to -14·7)	704 (481 to 1180)
	50-69 years	144 (110 to 189)	125 (93·9 to 155)	-13·5 (-22·7 to -4·77)	116 (87·7 to 151)	1290 (768 to 2840)	1240 (724 to 2800)	-3·90 (-26·1 to 21·2)	839 (499 to 1850)
	70+ years	284 (222 to 354)	233 (183 to 290)	-17·7 (-24·3 to -10·3)	227 (177 to 284)	1640 (996 to 4040)	1440 (831 to 3450)	-12·2 (-28·0 to 12·5)	1060 (647 to 2630)
Oman	All Ages	8·59 (7·52 to 10·1)	7·28 (6·32 to 8·68)	-15·2 (-20·1 to -9·97)	6·87 (6·02 to 8·06)	22·4 (16·9 to 31·2)	17·8 (14·0 to 26·9)	-20·4 (-35·2 to 0·175)	14·5 (11·0 to 20·3)
	Under 5	3·87 (3·08 to 4·90)	3·11 (2·36 to 3·94)	-19·5 (-31·1 to -6·26)	3·09 (2·46 to 3·92)	0·617 (0·459 to 0·857)	0·338 (0·255 to 0·456)	-44·9 (-53·4 to -34·6)	0·401 (0·298 to 0·557)
	5-14 years	3·71 (2·57 to 5·33)	3·22 (2·18 to 4·68)	-13·1 (-24·7 to -2·11)	2·97 (2·05 to 4·26)	0·283 (0·219 to 0·385)	0·172 (0·129 to 0·235)	-38·8 (-50·4 to -16·9)	0·184 (0·143 to 0·250)
	15-49 years	8·18 (6·42 to 10·0)	6·77 (5·55 to 8·49)	-17·1 (-23·7 to -7·88)	6·54 (5·14 to 8·02)	8·23 (6·20 to 12·2)	6·57 (4·88 to 10·6)	-20·0 (-37·9 to -1·49)	5·35 (4·03 to 7·91)
	50-69 years	19·9 (15·0 to 25·5)	16·4 (12·3 to 20·9)	-17·5 (-23·7 to -10·7)	15·9 (12·0 to 20·4)	6·32 (4·48 to 8·53)	4·60 (3·36 to 6·84)	-26·5 (-43·6 to 6·86)	4·11 (2·91 to 5·55)
	70+ years	52·9 (41·8 to 66·5)	46·8 (36·2 to 58·2)	-11·5 (-20·1 to -2·66)	42·3 (33·4 to 53·2)	6·93 (4·61 to 9·95)	6·11 (4·26 to 9·31)	-11·6 (-27·7 to 15·0)	4·50 (3·00 to 6·47)
Palestine	All Ages	8·88 (7·65 to 10·3)	7·70 (6·59 to 8·89)	-13·3 (-17·5 to -8·45)	7·11 (6·12 to 8·21)	32·4 (24·7 to 42·7)	27·8 (19·5 to 38·8)	-14·3 (-24·9 to 2·59)	21·1 (16·1 to 27·8)
	Under 5	4·75 (3·67 to 5·98)	3·60 (2·82 to 4·56)	-24·1 (-32·8 to -11·9)	3·80 (2·93 to 4·79)	1·76 (1·21 to 2·53)	0·928 (0·646 to 1·51)	-47·3 (-59·9 to -30·9)	1·14 (0·784 to 1·64)
	5-14 years	4·20 (2·91 to 6·05)	3·37 (2·23 to 4·97)	-19·8 (-31·0 to -5·56)	3·36 (2·33 to 4·84)	1·06 (0·788 to 1·63)	0·521 (0·375 to 0·772)	-50·5 (-59·9 to -38·1)	0·689 (0·512 to 1·06)
	15-49 years	8·72 (7·05 to 10·8)	7·45 (5·81 to 9·39)	-14·6 (-21·0 to -9·42)	6·98 (5·64 to 8·65)	7·48 (5·57 to 11·2)	6·48 (4·46 to 10·4)	-13·3 (-25·7 to 1·80)	4·86 (3·62 to 7·28)
	50-69 years	21·9 (17·1 to 28·0)	18·1 (13·8 to 23·3)	-17·4 (-23·6 to -11·7)	17·5 (13·7 to 22·4)	9·73 (6·82 to 13·6)	9·08 (5·66 to 13·7)	-6·68 (-24·7 to 17·9)	6·32 (4·43 to 8·87)

eTable 3. Percent change from 2015 to 2021 in age-standardised all-form tuberculosis incidence rate per 100,000 population and in deaths due to all-form tuberculosis by age for 204 countries and territories.

Location	Age group	2015 Rate	2021 Rate	Incidence Percent Change	Incidence Milestone	2015 Deaths	2021 Deaths	Mortality Percent Change	Mortality Milestone
	70+ years	50·1 (40·0 to 63·9)	39·8 (31·3 to 50·3)	-20·4 (-27·7 to -13·2)	40·0 (32·0 to 51·1)	12·4 (9·46 to 16·2)	10·8 (8·01 to 14·8)	-12·5 (-27·2 to 13·5)	8·06 (6·15 to 10·5)
Qatar	All Ages	29·9 (25·6 to 36·5)	24·0 (20·6 to 29·3)	-19·6 (-25·2 to -12·2)	23·9 (20·5 to 29·2)	28·7 (22·7 to 36·0)	20·9 (14·5 to 28·4)	-27·0 (-42·9 to -11·4)	18·7 (14·7 to 23·4)
	Under 5	10·5 (8·10 to 13·2)	8·30 (6·30 to 11·0)	-21·2 (-30·1 to -10·3)	8·44 (6·48 to 10·5)	0·418 (0·322 to 0·535)	0·268 (0·190 to 0·367)	-35·8 (-48·6 to -21·2)	0·272 (0·209 to 0·348)
	5-14 years	10·9 (7·40 to 15·9)	9·44 (6·42 to 13·9)	-13·3 (-24·8 to -0·678)	8·74 (5·92 to 12·7)	0·236 (0·192 to 0·300)	0·157 (0·126 to 0·203)	-33·0 (-45·5 to -14·2)	0·153 (0·125 to 0·195)
	15-49 years	29·7 (24·3 to 37·7)	24·0 (19·0 to 30·9)	-19·1 (-25·9 to -9·29)	23·7 (19·4 to 30·2)	16·7 (12·7 to 21·0)	12·7 (8·84 to 17·6)	-23·9 (-42·4 to -2·64)	10·9 (8·26 to 13·7)
	50-69 years	55·7 (40·7 to 69·5)	43·7 (32·3 to 54·3)	-21·5 (-28·5 to -13·6)	44·6 (32·6 to 55·6)	7·41 (5·74 to 9·43)	4·59 (2·94 to 6·48)	-38·0 (-53·5 to -20·0)	4·82 (3·73 to 6·13)
	70+ years	185 (140 to 235)	143 (110 to 179)	-22·5 (-32·5 to -13·6)	148 (112 to 188)	3·94 (3·18 to 5·10)	3·23 (2·42 to 4·48)	-17·8 (-36·5 to 3·26)	2·56 (2·07 to 3·31)
Saudi Arabia	All Ages	45·6 (38·4 to 54·1)	38·1 (32·4 to 46·4)	-16·4 (-20·8 to -11·2)	36·5 (30·7 to 43·3)	1220 (993 to 1550)	1140 (841 to 1660)	-6·81 (-25·2 to 14·8)	793 (646 to 1010)
	Under 5	9·39 (7·27 to 11·7)	6·80 (5·09 to 8·70)	-27·3 (-38·8 to -16·1)	7·51 (5·81 to 9·34)	7·95 (5·88 to 10·1)	3·65 (2·55 to 5·32)	-54·1 (-63·6 to -42·6)	5·17 (3·82 to 6·57)
	5-14 years	13·0 (9·28 to 18·6)	9·73 (6·49 to 14·3)	-24·6 (-36·6 to -6·91)	10·4 (7·42 to 14·9)	6·44 (5·00 to 8·62)	3·97 (2·86 to 5·33)	-38·3 (-52·5 to -23·3)	4·18 (3·25 to 5·60)
	15-49 years	48·6 (37·9 to 60·9)	39·4 (31·5 to 49·4)	-18·8 (-23·6 to -14·2)	38·9 (30·3 to 48·7)	644 (503 to 861)	602 (423 to 902)	-6·98 (-27·8 to 17·6)	418 (327 to 560)
	50-69 years	90·6 (66·9 to 113)	70·8 (52·8 to 88·6)	-21·8 (-28·3 to -14·3)	72·5 (53·5 to 90·3)	327 (264 to 423)	324 (238 to 460)	-1·05 (-21·8 to 27·3)	213 (171 to 275)
	70+ years	177 (141 to 221)	130 (102 to 165)	-26·3 (-34·8 to -18·2)	142 (112 to 177)	235 (187 to 304)	208 (151 to 296)	-11·7 (-26·5 to 11·8)	153 (122 to 197)
Sudan	All Ages	48·1 (41·2 to 54·5)	39·1 (34·0 to 44·7)	-18·7 (-23·8 to -13·9)	38·5 (32·9 to 43·6)	1900 (1230 to 2690)	1540 (969 to 2270)	-18·8 (-29·6 to -5·09)	1240 (799 to 1750)
	Under 5	26·1 (20·2 to 32·8)	18·9 (14·7 to 23·8)	-27·1 (-38·3 to -15·2)	20·8 (16·1 to 26·3)	290 (196 to 408)	142 (84·3 to 204)	-51·2 (-60·1 to -39·5)	189 (127 to 265)
	5-14 years	25·0 (17·7 to 35·6)	19·2 (13·5 to 27·1)	-23·1 (-33·9 to -13·6)	20·0 (14·1 to 28·5)	69·7 (43·4 to 97·1)	46·4 (28·4 to 67·0)	-32·8 (-46·4 to -19·5)	45·3 (28·2 to 63·1)
	15-49 years	52·3 (42·0 to 63·0)	41·5 (33·1 to 50·2)	-20·7 (-26·2 to -15·5)	41·9 (33·6 to 50·4)	668 (393 to 1010)	561 (352 to 849)	-15·5 (-31·4 to 2·36)	434 (256 to 658)
	50-69 years	104 (80·2 to 130)	85·3 (64·7 to 106)	-17·9 (-25·6 to -9·53)	83·1 (64·1 to 104)	413 (242 to 603)	388 (241 to 604)	-5·45 (-21·8 to 11·4)	269 (158 to 392)
	70+ years	206 (167 to 256)	172 (137 to 210)	-16·4 (-24·0 to -8·40)	165 (133 to 205)	459 (267 to 700)	402 (245 to 594)	-11·8 (-25·8 to 9·90)	299 (173 to 455)
Syrian Arab Republic	All Ages	10·1 (8·79 to 12·0)	9·94 (8·59 to 11·5)	-1·37 (-8·74 to 4·67)	8·07 (7·03 to 9·63)	118 (87·6 to 149)	101 (73·6 to 138)	-13·3 (-34·2 to 8·35)	76·4 (57·0 to 96·9)
	Under 5	3·70 (2·96 to 4·78)	3·07 (2·36 to 4·01)	-16·8 (-27·7 to -4·12)	2·96 (2·37 to 3·83)	2·97 (2·06 to 4·31)	0·917 (0·597 to 1·38)	-69·0 (-76·2 to -58·0)	1·93 (1·34 to 2·80)
	5-14 years	5·17 (3·66 to 7·60)	4·68 (3·17 to 6·76)	-9·20 (-24·8 to 8·47)	4·14 (2·93 to 6·08)	3·47 (2·74 to 4·31)	1·42 (1·06 to 2·16)	-59·0 (-66·8 to -44·8)	2·26 (1·78 to 2·80)

eTable 3. Percent change from 2015 to 2021 in age-standardised all-form tuberculosis incidence rate per 100,000 population and in deaths due to all-form tuberculosis by age for 204 countries and territories.

Location	Age group	2015 Rate	2021 Rate	Incidence Percent Change	Incidence Milestone	2015 Deaths	2021 Deaths	Mortality Percent Change	Mortality Milestone
	15-49 years	10·5 (8·57 to 12·9)	9·36 (7·53 to 11·6)	-10·3 (-18·7 to -2·73)	8·36 (6·85 to 10·4)	30·8 (22·9 to 38·8)	21·2 (14·6 to 30·1)	-30·8 (-49·6 to -7·49)	20·0 (14·9 to 25·2)
	50-69 years	17·3 (13·3 to 21·5)	15·5 (11·6 to 19·7)	-10·6 (-17·1 to -4·86)	13·9 (10·6 to 17·2)	38·9 (27·0 to 51·8)	38·8 (26·4 to 56·5)	0·504 (-27·8 to 36·9)	25·3 (17·6 to 33·7)
	70+ years	31·9 (25·0 to 40·3)	27·0 (21·5 to 34·4)	-15·2 (-22·4 to -6·76)	25·5 (20·0 to 32·2)	41·4 (32·4 to 51·2)	39·1 (30·2 to 52·5)	-4·95 (-25·0 to 20·1)	26·9 (21·1 to 33·3)
Tunisia	All Ages	28·2 (25·0 to 32·8)	24·5 (21·6 to 28·8)	-13·1 (-17·6 to -9·26)	22·5 (20·0 to 26·2)	260 (180 to 377)	232 (158 to 403)	-10·7 (-27·9 to 11·9)	169 (117 to 245)
	Under 5	11·6 (8·89 to 14·7)	8·81 (6·87 to 11·4)	-23·6 (-33·9 to -12·7)	9·24 (7·12 to 11·7)	3·57 (2·38 to 5·59)	1·69 (1·08 to 2·76)	-52·6 (-64·2 to -35·6)	2·32 (1·55 to 3·63)
	5-14 years	9·55 (6·43 to 13·8)	7·57 (5·17 to 10·8)	-20·6 (-29·4 to -9·76)	7·64 (5·14 to 11·0)	1·94 (1·36 to 2·99)	1·23 (0·859 to 2·04)	-36·2 (-50·8 to -21·4)	1·26 (0·881 to 1·95)
	15-49 years	24·2 (19·6 to 29·7)	20·3 (16·3 to 25·7)	-16·1 (-21·5 to -9·75)	19·3 (15·6 to 23·7)	56·5 (36·2 to 86·9)	46·0 (31·8 to 77·2)	-18·6 (-33·9 to 4·17)	36·7 (23·5 to 56·5)
	50-69 years	45·0 (34·3 to 57·7)	38·6 (28·9 to 50·5)	-14·1 (-20·6 to -8·02)	36·0 (27·5 to 46·2)	73·2 (47·3 to 112)	70·5 (43·7 to 129)	-3·23 (-26·7 to 26·0)	47·6 (30·7 to 72·5)
	70+ years	97·2 (79·7 to 119)	79·3 (64·7 to 100)	-18·3 (-25·7 to -10·7)	77·7 (63·7 to 94·9)	125 (84·4 to 174)	113 (77·3 to 190)	-9·44 (-27·5 to 13·3)	81·2 (54·9 to 113)
Türkiye	All Ages	14·9 (13·0 to 17·5)	12·9 (11·2 to 15·4)	-13·5 (-19·2 to -8·29)	11·9 (10·4 to 14·0)	1030 (913 to 1190)	883 (708 to 1110)	-14·0 (-31·2 to 8·07)	669 (593 to 771)
	Under 5	5·16 (4·12 to 6·48)	3·67 (2·73 to 4·69)	-28·8 (-41·8 to -16·9)	4·13 (3·30 to 5·19)	17·7 (13·5 to 22·2)	7·46 (5·30 to 10·0)	-57·8 (-66·3 to -47·0)	11·5 (8·80 to 14·4)
	5-14 years	5·00 (3·42 to 6·95)	3·93 (2·72 to 5·61)	-21·1 (-30·9 to -10·0)	4·00 (2·74 to 5·56)	8·66 (7·12 to 10·4)	5·53 (4·62 to 6·78)	-35·9 (-46·7 to -25·1)	5·63 (4·63 to 6·76)
	15-49 years	12·0 (9·71 to 14·9)	9·92 (7·96 to 12·6)	-17·4 (-23·1 to -11·1)	9·61 (7·76 to 12·0)	192 (167 to 222)	147 (119 to 188)	-23·5 (-37·6 to -3·70)	125 (108 to 144)
	50-69 years	26·3 (18·1 to 34·5)	22·2 (15·7 to 28·9)	-15·6 (-23·3 to -5·67)	21·1 (14·4 to 27·6)	328 (272 to 394)	293 (229 to 381)	-10·4 (-29·9 to 19·9)	213 (177 to 256)
	70+ years	51·3 (40·1 to 63·8)	42·2 (32·4 to 53·9)	-17·8 (-25·4 to -9·69)	41·0 (32·0 to 51·0)	482 (401 to 573)	430 (332 to 544)	-10·6 (-30·6 to 10·1)	313 (261 to 372)
United Arab Emirates	All Ages	5·56 (4·66 to 6·67)	4·95 (4·13 to 5·99)	-10·8 (-19·5 to 1·23)	4·45 (3·73 to 5·34)	39·3 (17·7 to 60·2)	34·3 (15·0 to 52·7)	-11·1 (-30·5 to 12·2)	25·5 (11·5 to 39·1)
	Under 5	3·28 (2·60 to 4·07)	2·55 (1·98 to 3·21)	-22·1 (-32·8 to -8·11)	2·62 (2·08 to 3·26)	0·374 (0·169 to 0·815)	0·196 (0·0903 to 0·451)	-47·7 (-59·2 to -32·9)	0·243 (0·110 to 0·530)
	5-14 years	1·92 (1·23 to 2·74)	1·42 (0·900 to 2·06)	-25·8 (-36·7 to -15·4)	1·53 (0·981 to 2·20)	0·202 (0·0933 to 0·384)	0·135 (0·0570 to 0·235)	-31·7 (-47·1 to -3·88)	0·131 (0·0607 to 0·250)
	15-49 years	5·35 (4·20 to 6·65)	4·36 (3·51 to 5·58)	-18·4 (-28·9 to -5·88)	4·28 (3·36 to 5·32)	22·2 (8·53 to 36·7)	14·1 (5·71 to 22·9)	-35·4 (-50·5 to -15·0)	14·4 (5·54 to 23·8)
	50-69 years	12·0 (8·88 to 15·3)	9·62 (7·14 to 12·3)	-19·7 (-28·1 to -12·6)	9·59 (7·10 to 12·3)	8·18 (3·32 to 15·2)	9·17 (3·83 to 17·3)	14·4 (-15·1 to 48·8)	5·32 (2·16 to 9·89)
	70+ years	51·3 (38·6 to 64·3)	43·4 (34·3 to 54·0)	-15·3 (-24·6 to -6·01)	41·1 (30·9 to 51·4)	8·30 (3·82 to 11·4)	10·7 (4·84 to 14·7)	30·1 (1·85 to 62·2)	5·40 (2·48 to 7·41)
Yemen	All Ages	21·0 (18·3 to 23·6)	19·5 (17·0 to 22·3)	-7·34 (-12·1 to -1·83)	16·8 (14·7 to 18·9)	841 (520 to 1240)	947 (597 to 1400)	12·9 (-2·52 to 30·8)	547 (338 to 806)

eTable 3. Percent change from 2015 to 2021 in age-standardised all-form tuberculosis incidence rate per 100,000 population and in deaths due to all-form tuberculosis by age for 204 countries and territories.

Location	Age group	2015 Rate	2021 Rate	Incidence Percent Change	Incidence Milestone	2015 Deaths	2021 Deaths	Mortality Percent Change	Mortality Milestone
	Under 5	11·6 (9·04 to 14·5)	9·62 (7·75 to 12·1)	-17·0 (-30·1 to -5·41)	9·29 (7·23 to 11·6)	122 (77·5 to 173)	87·2 (58·3 to 129)	-27·8 (-43·5 to -9·08)	79·2 (50·4 to 112)
	5-14 years	9·08 (6·41 to 12·9)	7·78 (5·52 to 11·5)	-14·1 (-23·5 to -2·07)	7·26 (5·13 to 10·3)	28·9 (16·8 to 44·0)	28·6 (15·7 to 40·3)	-0·209 (-26·4 to 26·9)	18·8 (10·9 to 28·6)
	15-49 years	23·2 (19·1 to 28·0)	20·9 (16·9 to 25·1)	-9·72 (-15·4 to -2·45)	18·6 (15·2 to 22·4)	252 (157 to 396)	300 (192 to 499)	19·0 (-2·47 to 44·1)	164 (102 to 257)
	50-69 years	55·9 (43·0 to 69·4)	51·9 (39·2 to 65·9)	-7·02 (-14·8 to 1·45)	44·7 (34·4 to 55·5)	229 (129 to 364)	279 (155 to 431)	22·6 (-0·985 to 46·9)	149 (83·8 to 237)
	70+ years	103 (82·3 to 130)	95·8 (73·4 to 119)	-7·04 (-15·6 to 3·37)	82·5 (65·8 to 104)	209 (126 to 331)	253 (163 to 369)	21·9 (3·76 to 44·7)	136 (81·9 to 215)
South Asia	All Ages	205 (177 to 235)	197 (170 to 227)	-3·69 (-5·62 to -1·50)	164 (142 to 188)	573000 (536000 to 611000)	509000 (458000 to 591000)	-11·1 (-20·3 to 0·223)	373000 (348000 to 397000)
	Under 5	48·2 (39·0 to 59·7)	39·5 (31·5 to 49·0)	-18·2 (-22·2 to -14·0)	38·6 (31·2 to 47·8)	17800 (15300 to 20200)	9970 (7950 to 12300)	-43·9 (-52·0 to -33·6)	11600 (9920 to 13100)
	5-14 years	47·7 (33·0 to 67·5)	38·3 (26·1 to 54·1)	-19·7 (-23·6 to -16·1)	38·2 (26·4 to 54·0)	8230 (7250 to 9190)	5740 (4900 to 6740)	-30·2 (-38·6 to -20·1)	5350 (4710 to 5970)
	15-49 years	216 (181 to 273)	200 (169 to 252)	-7·01 (-9·77 to -4·52)	173 (145 to 218)	186000 (172000 to 202000)	166000 (149000 to 192000)	-10·9 (-21·9 to -0·102)	121000 (112000 to 131000)
	50-69 years	440 (331 to 561)	400 (303 to 507)	-9·01 (-11·7 to -5·97)	352 (265 to 449)	213000 (198000 to 229000)	181000 (159000 to 216000)	-14·9 (-25·4 to -4·17)	138000 (129000 to 149000)
	70+ years	560 (436 to 707)	532 (415 to 657)	-4·89 (-8·54 to -1·41)	448 (349 to 566)	148000 (137000 to 160000)	147000 (129000 to 171000)	-0·897 (-10·7 to 12·7)	96100 (89100 to 104000)
Bangladesh	All Ages	158 (134 to 182)	136 (116 to 157)	-13·8 (-20·4 to -8·46)	126 (107 to 146)	31100 (27100 to 35500)	28700 (23100 to 35400)	-7·76 (-22·0 to 12·4)	20200 (17600 to 23100)
	Under 5	47·0 (36·3 to 60·4)	32·3 (25·2 to 41·0)	-31·1 (-38·3 to -20·2)	37·6 (29·1 to 48·3)	1250 (875 to 1680)	494 (323 to 676)	-60·1 (-70·4 to -47·7)	812 (569 to 1090)
	5-14 years	64·2 (43·3 to 92·4)	46·3 (33·1 to 66·4)	-27·7 (-35·6 to -16·9)	51·4 (34·7 to 73·9)	1010 (801 to 1240)	625 (465 to 804)	-37·9 (-51·9 to -25·9)	657 (521 to 809)
	15-49 years	159 (128 to 194)	131 (106 to 168)	-17·2 (-25·1 to -10·4)	127 (102 to 156)	8740 (7270 to 10200)	7410 (5780 to 9160)	-15·0 (-29·4 to 5·16)	5680 (4730 to 6600)
	50-69 years	326 (238 to 419)	261 (197 to 343)	-19·8 (-29·0 to -13·1)	261 (191 to 335)	10400 (8620 to 12700)	9370 (7120 to 12700)	-9·58 (-29·6 to 14·6)	6740 (5600 to 8270)
	70+ years	454 (359 to 575)	374 (298 to 471)	-17·5 (-27·9 to -7·93)	363 (287 to 460)	9780 (7830 to 11600)	10800 (8140 to 13600)	11·0 (-8·20 to 36·4)	6360 (5090 to 7510)
Bhutan	All Ages	120 (101 to 142)	109 (93·8 to 131)	-9·00 (-13·9 to -1·99)	96·1 (80·6 to 114)	125 (69·4 to 204)	108 (59·1 to 193)	-14·0 (-25·5 to 0·897)	81·2 (45·1 to 133)
	Under 5	24·8 (18·3 to 31·9)	19·3 (14·5 to 24·7)	-22·1 (-31·0 to -10·8)	19·8 (14·6 to 25·5)	2·94 (1·46 to 4·77)	1·60 (0·859 to 2·76)	-45·1 (-57·7 to -25·6)	1·91 (0·947 to 3·10)
	5-14 years	26·3 (18·3 to 40·0)	21·0 (13·5 to 31·2)	-20·0 (-30·3 to -11·1)	21·1 (14·6 to 32·0)	1·06 (0·601 to 1·83)	0·675 (0·376 to 1·17)	-35·7 (-51·6 to -12·8)	0·691 (0·390 to 1·19)
	15-49 years	119 (94·7 to 148)	104 (85·6 to 129)	-12·4 (-19·3 to -2·60)	94·9 (75·7 to 118)	33·2 (18·1 to 58·4)	27·4 (14·6 to 49·9)	-17·1 (-34·5 to 4·13)	21·6 (11·8 to 38·0)

eTable 3. Percent change from 2015 to 2021 in age-standardised all-form tuberculosis incidence rate per 100,000 population and in deaths due to all-form tuberculosis by age for 204 countries and territories.

Location	Age group	2015 Rate	2021 Rate	Incidence Percent Change	Incidence Milestone	2015 Deaths	2021 Deaths	Mortality Percent Change	Mortality Milestone
	50-69 years	267 (197 to 357)	229 (173 to 303)	-14·0 (-21·4 to -3·87)	214 (158 to 286)	38·8 (21·0 to 72·5)	33·8 (18·8 to 67·9)	-13·1 (-27·6 to 7·75)	25·2 (13·7 to 47·1)
	70+ years	388 (294 to 502)	324 (252 to 406)	-16·3 (-26·4 to -2·86)	311 (235 to 402)	48·9 (25·7 to 78·4)	44·3 (23·4 to 73·8)	-9·66 (-23·3 to 13·2)	31·8 (16·7 to 50·9)
India	All Ages	214 (185 to 247)	214 (184 to 248)	-0·209 (-2·55 to 2·22)	171 (148 to 198)	458000 (419000 to 496000)	405000 (351000 to 500000)	-11·5 (-20·8 to 2·56)	297000 (272000 to 322000)
	Under 5	39·2 (31·9 to 48·4)	33·4 (26·2 to 42·3)	-14·7 (-20·7 to -9·10)	31·3 (25·5 to 38·7)	9590 (7730 to 11800)	4770 (3590 to 6590)	-50·1 (-62·4 to -29·8)	6230 (5030 to 7690)
	5-14 years	41·0 (28·2 to 57·9)	33·5 (22·8 to 47·1)	-18·5 (-24·7 to -13·6)	32·8 (22·6 to 46·3)	4580 (3910 to 5380)	3140 (2460 to 3890)	-31·4 (-41·7 to -16·9)	2980 (2540 to 3490)
	15-49 years	225 (188 to 284)	216 (182 to 271)	-3·94 (-7·07 to -0·992)	180 (150 to 227)	147000 (135000 to 160000)	131000 (115000 to 161000)	-10·8 (-21·8 to 2·74)	95700 (87800 to 104000)
	50-69 years	456 (343 to 583)	424 (323 to 538)	-6·82 (-10·3 to -3·43)	365 (275 to 466)	177000 (161000 to 194000)	148000 (124000 to 186000)	-16·7 (-27·1 to -3·45)	115000 (105000 to 126000)
	70+ years	565 (436 to 717)	555 (432 to 688)	-1·70 (-5·85 to 2·70)	452 (349 to 574)	119000 (108000 to 130000)	118000 (103000 to 144000)	-0·691 (-11·1 to 15·2)	77300 (70100 to 84800)
Nepal	All Ages	164 (143 to 187)	140 (121 to 164)	-14·7 (-19·6 to -9·10)	131 (115 to 150)	8060 (5600 to 10300)	7010 (4730 to 10000)	-12·9 (-30·1 to 6·70)	5240 (3640 to 6720)
	Under 5	41·6 (31·8 to 52·2)	30·8 (24·4 to 38·3)	-25·8 (-32·5 to -17·9)	33·3 (25·4 to 41·8)	125 (87·2 to 175)	65·4 (44·2 to 92·9)	-47·6 (-59·8 to -34·1)	81·4 (56·7 to 114)
	5-14 years	34·7 (25·1 to 47·7)	26·8 (18·1 to 37·8)	-22·8 (-32·3 to -12·7)	27·8 (20·0 to 38·2)	83·0 (57·2 to 109)	50·3 (36·1 to 66·7)	-39·0 (-50·2 to -24·3)	53·9 (37·2 to 70·8)
	15-49 years	153 (126 to 196)	124 (105 to 157)	-18·9 (-25·8 to -12·4)	122 (101 to 157)	2080 (1530 to 2790)	1660 (1170 to 2480)	-20·0 (-32·0 to -2·01)	1350 (995 to 1810)
	50-69 years	425 (326 to 546)	348 (275 to 452)	-18·1 (-24·5 to -9·06)	340 (261 to 437)	3080 (2030 to 4210)	2700 (1740 to 4060)	-12·3 (-34·0 to 11·7)	2010 (1320 to 2730)
	70+ years	639 (513 to 792)	521 (403 to 656)	-18·5 (-26·3 to -9·98)	511 (410 to 634)	2690 (1810 to 3590)	2540 (1710 to 3610)	-5·02 (-27·6 to 23·3)	1750 (1180 to 2340)
Pakistan	All Ages	187 (164 to 211)	150 (131 to 170)	-19·8 (-24·6 to -16·8)	149 (131 to 169)	76300 (56000 to 95200)	68400 (48800 to 85900)	-9·66 (-29·0 to 14·0)	49600 (36400 to 61900)
	Under 5	88·9 (70·9 to 111)	66·5 (53·8 to 82·4)	-25·2 (-30·1 to -20·0)	71·1 (56·7 to 88·9)	6820 (4760 to 8680)	4640 (3270 to 5970)	-31·3 (-46·3 to -16·4)	4430 (3100 to 5640)
	5-14 years	74·6 (51·3 to 108)	57·5 (39·2 to 82·7)	-22·9 (-29·9 to -14·8)	59·7 (41·0 to 86·2)	2550 (2040 to 3110)	1920 (1540 to 2320)	-24·0 (-39·9 to -2·78)	1660 (1320 to 2020)
	15-49 years	208 (174 to 262)	163 (136 to 205)	-21·4 (-26·1 to -17·4)	166 (139 to 210)	28200 (21000 to 35800)	25500 (17900 to 32800)	-8·98 (-32·7 to 18·0)	18300 (13700 to 23300)
	50-69 years	412 (314 to 529)	332 (252 to 424)	-19·2 (-24·0 to -13·5)	329 (251 to 423)	22200 (16000 to 29100)	21400 (14300 to 27900)	-2·74 (-30·0 to 30·5)	14400 (10400 to 18900)
	70+ years	614 (481 to 764)	491 (392 to 619)	-20·0 (-26·0 to -13·9)	491 (385 to 611)	16500 (11300 to 21400)	15000 (10200 to 19200)	-7·99 (-25·3 to 16·5)	10700 (7340 to 13900)
Southeast Asia, East Asia, and Oceania	All Ages	97·5 (88·6 to 106)	94·3 (85·7 to 105)	-3·29 (-5·04 to -1·91)	78·0 (70·9 to 85·1)	265000 (248000 to 286000)	237000 (215000 to 281000)	-10·7 (-18·5 to 0·693)	173000 (161000 to 186000)

eTable 3. Percent change from 2015 to 2021 in age-standardised all-form tuberculosis incidence rate per 100,000 population and in deaths due to all-form tuberculosis by age for 204 countries and territories.

Location	Age group	2015 Rate	2021 Rate	Incidence Percent Change	Incidence Milestone	2015 Deaths	2021 Deaths	Mortality Percent Change	Mortality Milestone
	Under 5	39·1 (32·6 to 48·3)	33·8 (28·3 to 41·9)	-13·6 (-16·3 to -11·8)	31·3 (26·1 to 38·6)	6020 (5020 to 6970)	3520 (2790 to 4370)	-41·5 (-48·0 to -34·5)	3910 (3260 to 4530)
	5-14 years	30·4 (21·7 to 41·4)	26·4 (18·7 to 36·0)	-13·1 (-15·3 to -10·5)	24·3 (17·3 to 33·1)	2810 (2540 to 3070)	1790 (1570 to 2040)	-36·4 (-42·7 to -30·6)	1830 (1650 to 2000)
	15-49 years	84·6 (72·5 to 97·1)	82·1 (69·3 to 95·3)	-3·05 (-5·00 to -0·904)	67·7 (58·0 to 77·7)	77800 (72200 to 84400)	59700 (53700 to 68700)	-23·2 (-30·0 to -13·4)	50500 (46900 to 54900)
	50-69 years	154 (127 to 184)	142 (117 to 174)	-7·39 (-9·32 to -5·46)	123 (102 to 148)	92300 (85400 to 99900)	90400 (81200 to 108000)	-1·95 (-11·9 to 12·5)	60000 (55500 to 64900)
	70+ years	244 (205 to 285)	207 (173 to 247)	-15·4 (-17·9 to -13·2)	195 (164 to 228)	86500 (78700 to 95000)	81500 (73200 to 97300)	-5·82 (-14·0 to 5·77)	56300 (51100 to 61800)
East Asia	All Ages	53·9 (48·9 to 59·0)	49·6 (44·4 to 55·4)	-8·04 (-11·1 to -5·43)	43·1 (39·1 to 47·2)	58500 (52700 to 65600)	50400 (42700 to 62700)	-13·8 (-29·4 to 3·30)	38000 (34200 to 42700)
	Under 5	17·7 (14·7 to 21·7)	14·3 (12·1 to 17·7)	-19·2 (-24·3 to -15·0)	14·1 (11·8 to 17·4)	806 (723 to 905)	334 (274 to 404)	-58·6 (-64·0 to -51·7)	524 (470 to 588)
	5-14 years	11·3 (8·09 to 15·3)	8·76 (6·29 to 12·3)	-22·4 (-27·2 to -17·2)	9·02 (6·47 to 12·2)	215 (192 to 240)	135 (115 to 165)	-37·1 (-43·7 to -28·5)	140 (125 to 156)
	15-49 years	43·7 (38·1 to 50·9)	40·3 (34·1 to 47·6)	-7·86 (-11·6 to -2·07)	34·9 (30·5 to 40·7)	13900 (12300 to 15500)	9710 (8230 to 12200)	-29·9 (-41·3 to -16·8)	9010 (7980 to 10100)
	50-69 years	81·8 (66·9 to 97·5)	71·3 (57·6 to 87·2)	-12·9 (-17·0 to -9·35)	65·4 (53·5 to 78·0)	20600 (18200 to 22900)	19400 (16300 to 24400)	-5·49 (-25·5 to 15·6)	13400 (11900 to 14900)
	70+ years	145 (118 to 172)	117 (94·1 to 143)	-19·0 (-23·1 to -15·3)	116 (94·4 to 137)	23000 (20800 to 26400)	20700 (17300 to 26500)	-9·74 (-24·0 to 6·98)	14900 (13500 to 17200)
China	All Ages	48·8 (44·2 to 53·6)	44·9 (40·1 to 50·0)	-7·90 (-11·1 to -4·92)	39·0 (35·4 to 42·9)	45500 (39900 to 54300)	38700 (31300 to 52100)	-14·9 (-33·5 to 4·37)	29600 (26000 to 35300)
	Under 5	15·4 (12·8 to 19·0)	12·6 (10·6 to 15·7)	-17·7 (-23·7 to -13·2)	12·3 (10·2 to 15·2)	672 (595 to 773)	264 (216 to 330)	-60·6 (-66·2 to -54·4)	437 (387 to 502)
	5-14 years	9·88 (7·05 to 13·3)	7·88 (5·67 to 11·0)	-20·3 (-26·1 to -14·2)	7·90 (5·64 to 10·6)	159 (142 to 184)	103 (87·3 to 128)	-35·6 (-43·7 to -25·0)	104 (92·3 to 120)
	15-49 years	39·5 (34·6 to 46·6)	36·2 (30·7 to 43·0)	-8·40 (-12·5 to -2·36)	31·6 (27·7 to 37·3)	10800 (9390 to 12600)	7150 (5760 to 9690)	-33·9 (-47·3 to -18·3)	7030 (6100 to 8210)
	50-69 years	74·5 (60·8 to 88·4)	64·6 (52·0 to 78·7)	-13·4 (-17·6 to -9·65)	59·6 (48·7 to 70·7)	15600 (13300 to 18600)	14400 (11100 to 19500)	-7·07 (-30·1 to 17·9)	10100 (8620 to 12100)
	70+ years	130 (105 to 156)	108 (86·4 to 132)	-16·9 (-20·7 to -12·8)	104 (84·0 to 125)	18300 (16100 to 22000)	16700 (13600 to 22600)	-8·54 (-26·4 to 11·8)	11900 (10500 to 14300)
Democratic People's Republic of Korea	All Ages	328 (298 to 360)	306 (275 to 341)	-6·63 (-11·8 to -1·44)	262 (238 to 288)	12200 (8870 to 16400)	11000 (7710 to 15200)	-9·56 (-21·5 to 5·68)	7910 (5770 to 10700)
	Under 5	127 (104 to 157)	98·7 (82·2 to 125)	-22·2 (-32·9 to -11·1)	102 (83·1 to 126)	132 (85·8 to 184)	68·3 (43·9 to 98·9)	-47·9 (-61·8 to -34·4)	85·5 (55·8 to 120)
	5-14 years	75·8 (50·7 to 111)	57·2 (37·7 to 81·2)	-24·2 (-33·7 to -7·31)	60·6 (40·5 to 88·9)	54·5 (40·8 to 83·1)	31·7 (22·0 to 45·1)	-41·4 (-54·2 to -26·6)	35·4 (26·5 to 54·0)
	15-49 years	273 (222 to 312)	242 (198 to 281)	-11·3 (-18·1 to -3·85)	218 (178 to 249)	2970 (2060 to 4480)	2500 (1770 to 3730)	-15·0 (-30·6 to 5·28)	1930 (1340 to 2910)
	50-69 years	576 (475 to 687)	516 (431 to 620)	-10·5 (-18·6 to -3·49)	461 (380 to 550)	4910 (3470 to 6760)	4890 (3290 to 6720)	-0·132 (-16·5 to 16·4)	3190 (2260 to 4390)

eTable 3. Percent change from 2015 to 2021 in age-standardised all-form tuberculosis incidence rate per 100,000 population and in deaths due to all-form tuberculosis by age for 204 countries and territories.

Location	Age group	2015 Rate	2021 Rate	Incidence Percent Change	Incidence Milestone	2015 Deaths	2021 Deaths	Mortality Percent Change	Mortality Milestone
	70+ years	826 (668 to 971)	730 (583 to 897)	-11·6 (-23·3 to 0·263)	660 (534 to 777)	4110 (2790 to 5490)	3500 (2460 to 4630)	-14·6 (-30·2 to 0·724)	2670 (1820 to 3570)
Taiwan (Province of China)	All Ages	53·7 (48·8 to 59·1)	44·5 (38·7 to 50·1)	-17·2 (-23·7 to -10·9)	43·0 (39·1 to 47·3)	803 (744 to 850)	688 (618 to 748)	-14·4 (-19·7 to -8·57)	522 (484 to 552)
	Under 5	10·2 (8·26 to 12·6)	14·9 (11·9 to 18·7)	45·8 (26·1 to 68·9)	8·20 (6·61 to 10·1)	2·60 (2·28 to 2·90)	1·45 (1·22 to 1·66)	-44·2 (-54·6 to -32·7)	1·69 (1·48 to 1·89)
	5-14 years	4·08 (2·81 to 5·86)	9·77 (6·43 to 14·4)	140 (93·9 to 191)	3·26 (2·25 to 4·69)	0·923 (0·831 to 1·03)	0·677 (0·590 to 0·782)	-26·6 (-36·9 to -19·2)	0·600 (0·540 to 0·672)
	15-49 years	29·0 (25·3 to 33·2)	30·2 (24·1 to 36·6)	4·00 (-6·84 to 18·1)	23·2 (20·3 to 26·5)	75·9 (67·9 to 84·2)	54·2 (48·1 to 61·0)	-28·6 (-33·4 to -23·1)	49·4 (44·2 to 54·7)
	50-69 years	65·4 (53·9 to 77·1)	53·1 (41·2 to 67·5)	-18·8 (-30·4 to -6·60)	52·3 (43·1 to 61·7)	155 (144 to 167)	139 (125 to 151)	-10·4 (-17·9 to -2·90)	101 (93·7 to 109)
	70+ years	247 (204 to 297)	124 (95·1 to 154)	-49·6 (-55·6 to -43·2)	197 (164 to 238)	569 (511 to 610)	493 (431 to 543)	-13·4 (-19·0 to -6·89)	370 (332 to 396)
Oceania	All Ages	114 (102 to 125)	108 (96·2 to 120)	-5·60 (-9·01 to -1·11)	91·3 (81·9 to 100)	3280 (2760 to 3950)	3390 (2840 to 4000)	3·56 (-9·77 to 18·9)	2130 (1790 to 2570)
	Under 5	51·0 (41·0 to 63·7)	43·2 (34·4 to 52·9)	-15·0 (-24·6 to -7·10)	40·8 (32·8 to 51·0)	264 (162 to 399)	218 (115 to 324)	-17·1 (-33·8 to -2·45)	172 (105 to 259)
	5-14 years	51·9 (36·2 to 71·4)	45·3 (31·8 to 63·2)	-12·6 (-20·4 to -3·48)	41·6 (29·0 to 57·1)	102 (74·3 to 132)	93·2 (71·8 to 128)	-8·23 (-22·9 to 13·9)	66·3 (48·3 to 85·6)
	15-49 years	126 (107 to 145)	119 (101 to 137)	-5·52 (-11·1 to 0·447)	101 (85·6 to 116)	1290 (1090 to 1570)	1280 (1010 to 1560)	-0·386 (-13·7 to 18·8)	838 (705 to 1020)
	50-69 years	225 (188 to 267)	217 (181 to 258)	-3·62 (-8·51 to 3·18)	180 (150 to 213)	1010 (817 to 1210)	1140 (914 to 1460)	12·6 (-3·69 to 30·0)	659 (531 to 785)
	70+ years	410 (347 to 455)	379 (321 to 421)	-7·47 (-12·8 to -1·60)	328 (278 to 364)	613 (506 to 750)	661 (531 to 839)	8·19 (-10·4 to 27·3)	398 (329 to 488)
American Samoa	All Ages	14·1 (12·3 to 15·5)	15·0 (13·2 to 16·6)	6·68 (2·15 to 12·3)	11·3 (9·80 to 12·4)	1·07 (0·962 to 1·22)	1·12 (0·966 to 1·39)	4·59 (-5·79 to 18·3)	0·699 (0·625 to 0·791)
	Under 5	4·71 (3·65 to 6·24)	4·30 (3·35 to 5·64)	-8·38 (-18·3 to 0·352)	3·76 (2·92 to 4·99)	0·00986 (0·00808 to 0·0127)	0·00591 (0·00463 to 0·00769)	-39·9 (-51·3 to -26·6)	0·00641 (0·00525 to 0·00823)
	5-14 years	3·47 (2·39 to 5·17)	3·20 (2·17 to 4·73)	-7·80 (-24·3 to 7·11)	2·78 (1·91 to 4·14)	0·0148 (0·0116 to 0·0183)	0·0111 (0·00816 to 0·0142)	-24·6 (-38·3 to -7·91)	0·00963 (0·00755 to 0·0119)
	15-49 years	15·6 (12·3 to 18·4)	15·7 (13·0 to 18·3)	0·924 (-4·06 to 8·41)	12·5 (9·80 to 14·7)	0·323 (0·260 to 0·387)	0·299 (0·226 to 0·392)	-7·37 (-23·7 to 9·49)	0·210 (0·169 to 0·252)
	50-69 years	25·5 (20·9 to 30·7)	25·1 (20·3 to 31·2)	-1·33 (-8·76 to 6·15)	20·4 (16·7 to 24·6)	0·412 (0·350 to 0·478)	0·464 (0·391 to 0·579)	12·8 (-0·178 to 31·6)	0·268 (0·228 to 0·310)
	70+ years	39·4 (30·8 to 48·4)	38·1 (30·0 to 46·5)	-3·30 (-13·1 to 4·77)	31·5 (24·6 to 38·7)	0·315 (0·267 to 0·371)	0·344 (0·278 to 0·430)	9·38 (-7·58 to 32·9)	0·205 (0·174 to 0·241)
Cook Islands	All Ages	14·9 (13·3 to 16·6)	14·7 (12·9 to 16·6)	-1·70 (-6·38 to 4·53)	11·9 (10·6 to 13·3)	0·304 (0·266 to 0·355)	0·277 (0·233 to 0·351)	-8·98 (-22·2 to 4·49)	0·198 (0·173 to 0·231)
	Under 5	4·00 (3·14 to 5·21)	3·62 (2·77 to 4·68)	-9·37 (-17·2 to 2·65)	3·20 (2·51 to 4·17)	0·00102 (0·000742 to 0·00141)	0·00103 (0·000686 to 0·00147)	0·0447 (-22·7 to 22·5)	0·000666 (0·000483 to 0·000919)

eTable 3. Percent change from 2015 to 2021 in age-standardised all-form tuberculosis incidence rate per 100,000 population and in deaths due to all-form tuberculosis by age for 204 countries and territories.

Location	Age group	2015 Rate	2021 Rate	Incidence Percent Change	Incidence Milestone	2015 Deaths	2021 Deaths	Mortality Percent Change	Mortality Milestone
	5-14 years	3·51 (2·38 to 4·99)	3·13 (2·06 to 4·50)	-10·9 (-21·4 to 1·98)	2·81 (1·91 to 4·00)	0·00112 (0·000862 to 0·00147)	0·000824 (0·000610 to 0·00109)	-26·1 (-39·1 to -9·94)	0·000727 (0·000560 to 0·000956)
	15-49 years	13·4 (10·8 to 16·3)	12·7 (10·3 to 15·7)	-5·34 (-10·0 to 1·07)	10·7 (8·67 to 13·1)	0·0550 (0·0438 to 0·0661)	0·0429 (0·0330 to 0·0564)	-22·0 (-36·8 to -6·27)	0·0358 (0·0285 to 0·0430)
	50-69 years	23·3 (17·7 to 29·1)	21·8 (16·1 to 27·2)	-6·76 (-14·0 to 1·97)	18·7 (14·2 to 23·2)	0·110 (0·0951 to 0·131)	0·104 (0·0833 to 0·139)	-4·86 (-19·6 to 17·5)	0·0714 (0·0618 to 0·0853)
	70+ years	36·7 (28·8 to 47·1)	33·1 (26·2 to 41·4)	-9·70 (-18·6 to -2·10)	29·3 (23·0 to 37·7)	0·137 (0·116 to 0·166)	0·127 (0·105 to 0·156)	-6·82 (-23·4 to 7·14)	0·0891 (0·0756 to 0·108)
Fiji	All Ages	36·4 (32·5 to 39·9)	33·8 (30·2 to 37·2)	-7·12 (-11·6 to -2·91)	29·1 (26·0 to 31·9)	52·6 (42·4 to 66·7)	47·3 (37·0 to 61·4)	-9·98 (-23·5 to 9·82)	34·2 (27·6 to 43·4)
	Under 5	14·1 (11·1 to 18·2)	12·1 (9·52 to 15·5)	-14·2 (-25·2 to -2·21)	11·3 (8·86 to 14·6)	1·19 (0·926 to 1·57)	0·860 (0·627 to 1·14)	-27·2 (-41·3 to -6·36)	0·770 (0·602 to 1·02)
	5-14 years	19·6 (13·0 to 28·4)	17·9 (12·5 to 24·6)	-8·05 (-20·0 to 5·85)	15·7 (10·4 to 22·7)	1·14 (0·885 to 1·48)	1·08 (0·833 to 1·42)	-4·60 (-26·1 to 16·8)	0·740 (0·576 to 0·961)
	15-49 years	34·0 (27·8 to 39·8)	31·1 (25·3 to 35·1)	-8·66 (-13·9 to -1·54)	27·2 (22·2 to 31·8)	14·3 (11·7 to 18·7)	11·9 (9·23 to 15·8)	-16·2 (-32·2 to 3·30)	9·27 (7·60 to 12·2)
	50-69 years	66·4 (54·6 to 79·9)	61·3 (50·6 to 73·0)	-7·62 (-14·8 to -0·236)	53·2 (43·7 to 63·9)	22·1 (17·7 to 27·8)	20·0 (14·8 to 28·2)	-9·19 (-26·7 to 17·4)	14·4 (11·5 to 18·0)
	70+ years	106 (86·4 to 126)	94·6 (74·1 to 112)	-11·1 (-20·4 to -2·64)	85·2 (69·1 to 100)	13·9 (11·2 to 17·9)	13·4 (10·5 to 17·6)	-3·17 (-21·2 to 20·6)	9·04 (7·28 to 11·6)
Guam	All Ages	40·2 (35·3 to 45·4)	42·1 (37·1 to 47·5)	4·74 (-0·917 to 9·32)	32·2 (28·2 to 36·3)	8·90 (7·92 to 10·1)	9·20 (7·71 to 10·7)	3·36 (-10·0 to 18·3)	5·79 (5·15 to 6·57)
	Under 5	11·2 (8·91 to 14·2)	10·2 (8·23 to 13·0)	-8·51 (-18·0 to 1·89)	8·95 (7·13 to 11·4)	0·0948 (0·0790 to 0·114)	0·0679 (0·0534 to 0·0859)	-28·3 (-38·7 to -15·8)	0·0617 (0·0514 to 0·0739)
	5-14 years	15·5 (10·7 to 21·4)	15·3 (10·1 to 21·8)	-0·976 (-14·7 to 14·2)	12·4 (8·59 to 17·1)	0·0846 (0·0704 to 0·100)	0·105 (0·0831 to 0·129)	24·5 (3·52 to 49·1)	0·0550 (0·0458 to 0·0651)
	15-49 years	38·7 (32·6 to 47·0)	38·2 (31·2 to 46·0)	-1·26 (-7·45 to 6·17)	31·0 (26·1 to 37·6)	2·42 (2·13 to 2·80)	2·49 (2·07 to 2·92)	2·66 (-10·2 to 17·2)	1·58 (1·38 to 1·82)
	50-69 years	63·4 (48·4 to 79·1)	64·1 (50·1 to 80·1)	1·21 (-6·41 to 7·73)	50·7 (38·7 to 63·2)	3·70 (3·17 to 4·36)	3·82 (3·13 to 4·72)	3·52 (-12·8 to 23·0)	2·41 (2·06 to 2·84)
	70+ years	89·6 (72·4 to 109)	86·9 (71·0 to 107)	-2·87 (-10·2 to 4·89)	71·7 (58·0 to 87·3)	2·60 (2·18 to 2·99)	2·71 (2·22 to 3·27)	4·62 (-10·9 to 20·2)	1·69 (1·42 to 1·94)
Kiribati	All Ages	309 (279 to 341)	293 (264 to 325)	-5·15 (-8·32 to -0·773)	248 (223 to 273)	89·7 (67·5 to 115)	85·9 (63·1 to 121)	-4·34 (-18·0 to 9·71)	58·3 (43·8 to 75·0)
	Under 5	114 (87·2 to 145)	98·5 (77·4 to 126)	-13·7 (-24·0 to -4·33)	91·5 (69·7 to 116)	3·05 (2·09 to 4·02)	2·20 (1·46 to 3·44)	-27·7 (-45·8 to -1·68)	1·99 (1·36 to 2·61)
	5-14 years	164 (115 to 232)	147 (97·9 to 204)	-10·6 (-22·3 to 0·0187)	131 (91·9 to 186)	2·30 (1·63 to 2·89)	2·09 (1·57 to 2·74)	-8·60 (-29·6 to 22·7)	1·49 (1·06 to 1·88)
	15-49 years	328 (282 to 374)	309 (258 to 353)	-5·79 (-10·9 to -0·0556)	262 (226 to 299)	33·8 (23·8 to 44·0)	31·1 (21·1 to 44·6)	-8·08 (-25·0 to 9·32)	22·0 (15·4 to 28·6)
	50-69 years	601 (506 to 702)	581 (486 to 665)	-3·20 (-8·50 to 2·78)	481 (405 to 562)	32·9 (24·5 to 43·5)	33·7 (24·3 to 49·0)	2·47 (-13·1 to 18·3)	21·4 (15·9 to 28·3)
	70+ years	1080 (949 to 1200)	973 (850 to 1080)	-10·0 (-17·4 to -3·18)	866 (759 to 958)	17·7 (13·2 to 21·9)	16·8 (13·4 to 21·3)	-4·72 (-17·3 to 9·77)	11·5 (8·56 to 14·3)

eTable 3. Percent change from 2015 to 2021 in age-standardised all-form tuberculosis incidence rate per 100,000 population and in deaths due to all-form tuberculosis by age for 204 countries and territories.

Location	Age group	2015 Rate	2021 Rate	Incidence Percent Change	Incidence Milestone	2015 Deaths	2021 Deaths	Mortality Percent Change	Mortality Milestone
Marshall Islands	All Ages	357 (316 to 402)	342 (299 to 384)	-4·25 (-8·84 to 0·196)	286 (253 to 321)	35·0 (21·9 to 46·5)	33·4 (20·2 to 45·0)	-4·89 (-16·5 to 6·44)	22·8 (14·2 to 30·2)
	Under 5	120 (93·2 to 150)	104 (80·5 to 133)	-12·9 (-24·0 to 2·13)	95·8 (74·6 to 120)	0·664 (0·410 to 0·925)	0·481 (0·313 to 0·668)	-27·0 (-41·6 to -8·84)	0·431 (0·266 to 0·601)
	5-14 years	166 (113 to 228)	144 (97·3 to 200)	-12·8 (-23·3 to 1·32)	132 (90·6 to 182)	0·728 (0·483 to 0·958)	0·547 (0·341 to 0·717)	-24·2 (-41·3 to -5·65)	0·473 (0·314 to 0·623)
	15-49 years	391 (324 to 452)	358 (295 to 417)	-8·48 (-13·6 to -3·53)	313 (259 to 362)	14·7 (9·57 to 19·4)	13·1 (8·13 to 18·2)	-11·5 (-23·3 to 0·732)	9·59 (6·22 to 12·6)
	50-69 years	729 (595 to 866)	679 (560 to 803)	-6·81 (-12·9 to -1·28)	583 (476 to 693)	14·0 (8·43 to 18·6)	13·4 (7·51 to 18·8)	-4·26 (-18·5 to 12·3)	9·08 (5·48 to 12·1)
	70+ years	1120 (926 to 1270)	1010 (838 to 1170)	-9·39 (-16·3 to -1·66)	895 (741 to 1010)	4·94 (3·09 to 6·59)	5·91 (3·62 to 8·29)	20·2 (-2·52 to 40·5)	3·21 (2·01 to 4·29)
Micronesia (Federated States of)	All Ages	80·4 (72·1 to 88·1)	78·1 (69·9 to 86·0)	-2·78 (-7·38 to 1·39)	64·3 (57·7 to 70·4)	12·9 (9·27 to 16·6)	12·8 (9·10 to 17·4)	-0·653 (-13·8 to 15·7)	8·36 (6·02 to 10·8)
	Under 5	25·4 (19·6 to 31·9)	22·8 (18·0 to 29·5)	-9·82 (-22·0 to 0·994)	20·3 (15·7 to 25·5)	0·208 (0·145 to 0·278)	0·160 (0·111 to 0·213)	-22·6 (-41·4 to -3·62)	0·135 (0·0940 to 0·181)
	5-14 years	34·2 (23·3 to 49·0)	29·6 (19·7 to 39·9)	-13·2 (-24·1 to -3·17)	27·4 (18·6 to 39·2)	0·233 (0·168 to 0·304)	0·165 (0·125 to 0·220)	-28·7 (-40·8 to -11·0)	0·152 (0·109 to 0·198)
	15-49 years	85·0 (69·5 to 98·3)	79·0 (64·7 to 90·5)	-7·06 (-12·3 to -1·34)	68·0 (55·6 to 78·6)	4·69 (3·24 to 6·16)	4·33 (3·07 to 5·82)	-7·40 (-24·3 to 14·6)	3·05 (2·11 to 4·01)
	50-69 years	157 (126 to 191)	151 (125 to 185)	-4·11 (-10·3 to 3·48)	126 (101 to 153)	5·28 (3·65 to 7·37)	5·58 (3·68 to 8·01)	5·82 (-9·36 to 28·1)	3·43 (2·37 to 4·79)
	70+ years	251 (211 to 286)	225 (185 to 265)	-10·3 (-17·2 to -1·47)	201 (169 to 228)	2·44 (1·84 to 3·14)	2·53 (1·88 to 3·41)	3·92 (-16·6 to 24·3)	1·59 (1·20 to 2·04)
Nauru	All Ages	159 (140 to 179)	140 (124 to 157)	-11·6 (-15·8 to -6·73)	127 (112 to 143)	3·38 (2·14 to 4·86)	2·61 (1·83 to 3·59)	-22·2 (-33·3 to -6·89)	2·20 (1·39 to 3·16)
	Under 5	58·3 (44·8 to 72·0)	47·7 (37·3 to 60·2)	-17·9 (-28·9 to -6·72)	46·7 (35·9 to 57·6)	0·104 (0·0632 to 0·142)	0·0670 (0·0429 to 0·0964)	-35·2 (-47·0 to -18·0)	0·0677 (0·0411 to 0·0920)
	5-14 years	75·9 (53·6 to 106)	62·6 (43·8 to 88·1)	-17·2 (-25·7 to -6·15)	60·7 (42·9 to 84·8)	0·0808 (0·0542 to 0·106)	0·0563 (0·0391 to 0·0736)	-29·6 (-43·1 to -8·73)	0·0525 (0·0352 to 0·0690)
	15-49 years	179 (150 to 207)	158 (129 to 181)	-12·0 (-17·5 to -5·95)	143 (120 to 166)	1·37 (0·882 to 1·88)	1·05 (0·747 to 1·54)	-22·9 (-34·9 to -6·37)	0·889 (0·573 to 1·22)
	50-69 years	337 (276 to 408)	296 (241 to 357)	-12·1 (-18·2 to -4·13)	270 (221 to 326)	1·21 (0·688 to 1·72)	0·916 (0·552 to 1·26)	-23·1 (-39·3 to -9·14)	0·784 (0·447 to 1·12)
	70+ years	485 (416 to 555)	410 (338 to 484)	-15·4 (-24·9 to -7·08)	388 (333 to 444)	0·625 (0·370 to 1·20)	0·525 (0·357 to 0·935)	-14·7 (-31·6 to 12·1)	0·407 (0·240 to 0·779)
Niue	All Ages	37·0 (32·9 to 41·2)	35·5 (31·6 to 39·1)	-4·11 (-8·01 to 0·497)	29·6 (26·4 to 33·0)	0·119 (0·0923 to 0·149)	0·108 (0·0806 to 0·138)	-9·78 (-21·5 to 3·42)	0·0777 (0·0600 to 0·0968)
	Under 5	13·0 (10·1 to 16·5)	12·0 (9·37 to 15·4)	-7·44 (-18·9 to 7·45)	10·4 (8·08 to 13·2)	0·00150 (0·00105 to 0·00201)	0·00132 (0·000865 to 0·00178)	-11·8 (-30·5 to 11·9)	0·000974 (0·000685 to 0·00131)
	5-14 years	14·9 (10·3 to 20·3)	13·5 (9·17 to 18·4)	-8·83 (-21·9 to 5·80)	11·9 (8·25 to 16·2)	0·00134 (0·000957 to 0·00182)	0·00108 (0·000780 to 0·00144)	-19·0 (-34·4 to -1·17)	0·000870 (0·000622 to 0·00118)

eTable 3. Percent change from 2015 to 2021 in age-standardised all-form tuberculosis incidence rate per 100,000 population and in deaths due to all-form tuberculosis by age for 204 countries and territories.

Location	Age group	2015 Rate	2021 Rate	Incidence Percent Change	Incidence Milestone	2015 Deaths	2021 Deaths	Mortality Percent Change	Mortality Milestone
	15-49 years	30·0 (25·1 to 35·3)	28·2 (23·1 to 33·4)	-6·06 (-12·3 to -0·0433)	24·0 (20·0 to 28·2)	0·0199 (0·0133 to 0·0280)	0·0175 (0·0118 to 0·0243)	-11·8 (-26·3 to 10·4)	0·0129 (0·00864 to 0·0182)
	50-69 years	59·2 (47·5 to 72·1)	57·4 (46·6 to 70·5)	-2·93 (-10·5 to 4·39)	47·4 (38·0 to 57·7)	0·0435 (0·0308 to 0·0592)	0·0438 (0·0300 to 0·0602)	1·26 (-21·7 to 18·5)	0·0282 (0·0200 to 0·0385)
	70+ years	93·2 (74·4 to 112)	86·1 (68·7 to 103)	-7·54 (-15·9 to -1·38)	74·6 (59·5 to 90·0)	0·0533 (0·0409 to 0·0646)	0·0440 (0·0341 to 0·0548)	-17·3 (-29·9 to -1·65)	0·0346 (0·0266 to 0·0420)
Northern Mariana Islands	All Ages	94·5 (82·1 to 106)	95·5 (82·0 to 106)	1·11 (-4·13 to 6·93)	75·6 (65·6 to 84·4)	4·57 (3·61 to 6·02)	4·64 (3·55 to 5·88)	1·82 (-15·0 to 21·7)	2·97 (2·35 to 3·91)
	Under 5	28·2 (22·1 to 36·7)	25·6 (19·9 to 33·4)	-9·13 (-19·5 to 5·38)	22·6 (17·7 to 29·4)	0·0311 (0·0235 to 0·0411)	0·0191 (0·0143 to 0·0253)	-38·4 (-49·3 to -25·9)	0·0202 (0·0153 to 0·0267)
	5-14 years	30·7 (21·2 to 43·1)	27·7 (19·2 to 38·4)	-9·32 (-21·4 to 4·17)	24·5 (17·0 to 34·5)	0·0404 (0·0308 to 0·0496)	0·0244 (0·0156 to 0·0332)	-39·3 (-57·4 to -22·9)	0·0262 (0·0200 to 0·0322)
	15-49 years	87·6 (73·2 to 105)	82·6 (69·7 to 98·3)	-5·60 (-12·2 to 0·728)	70·1 (58·6 to 84·1)	1·16 (0·860 to 1·59)	0·888 (0·688 to 1·20)	-23·4 (-36·2 to -1·30)	0·756 (0·559 to 1·04)
	50-69 years	167 (126 to 205)	163 (122 to 202)	-1·82 (-8·94 to 4·97)	133 (101 to 164)	2·25 (1·74 to 2·96)	2·28 (1·67 to 2·94)	1·36 (-16·0 to 26·4)	1·47 (1·13 to 1·93)
	70+ years	265 (207 to 327)	238 (185 to 301)	-10·0 (-18·2 to -0·245)	212 (166 to 262)	1·08 (0·868 to 1·39)	1·43 (0·990 to 2·24)	32·9 (4·51 to 94·5)	0·700 (0·564 to 0·906)
Palau	All Ages	45·0 (39·5 to 50·3)	46·2 (41·0 to 52·5)	2·55 (-3·13 to 7·23)	36·0 (31·6 to 40·2)	1·51 (1·23 to 1·82)	1·54 (1·21 to 1·98)	1·84 (-12·0 to 22·3)	0·983 (0·802 to 1·18)
	Under 5	12·4 (9·89 to 16·0)	11·5 (9·21 to 14·8)	-6·70 (-15·2 to 5·16)	9·89 (7·91 to 12·8)	0·0128 (0·00980 to 0·0172)	0·00942 (0·00737 to 0·0120)	-25·8 (-39·8 to -10·0)	0·00832 (0·00637 to 0·0112)
	5-14 years	12·6 (8·63 to 18·2)	11·7 (7·97 to 17·2)	-6·51 (-16·4 to 7·62)	10·1 (6·91 to 14·6)	0·00748 (0·00591 to 0·00902)	0·00635 (0·00486 to 0·00792)	-14·7 (-30·7 to 4·87)	0·00486 (0·00384 to 0·00587)
	15-49 years	44·5 (37·2 to 52·2)	43·8 (36·4 to 52·9)	-1·54 (-8·38 to 5·34)	35·6 (29·7 to 41·8)	0·518 (0·411 to 0·645)	0·445 (0·359 to 0·568)	-13·7 (-30·0 to 6·03)	0·337 (0·267 to 0·419)
	50-69 years	66·1 (52·5 to 82·6)	64·1 (51·4 to 79·8)	-2·95 (-11·8 to 5·05)	52·9 (42·0 to 66·1)	0·626 (0·472 to 0·788)	0·675 (0·489 to 0·990)	8·18 (-13·5 to 32·6)	0·407 (0·307 to 0·512)
	70+ years	108 (86·8 to 134)	97·4 (78·5 to 121)	-9·66 (-19·0 to 0·256)	86·3 (69·4 to 107)	0·348 (0·284 to 0·437)	0·401 (0·311 to 0·525)	15·7 (-2·06 to 42·5)	0·226 (0·185 to 0·284)
Papua New Guinea	All Ages	129 (115 to 142)	119 (106 to 133)	-7·12 (-11·0 to -1·83)	103 (92·0 to 113)	2770 (2300 to 3380)	2900 (2380 to 3470)	4·85 (-9·90 to 21·3)	1800 (1500 to 2200)
	Under 5	57·5 (46·1 to 71·6)	47·6 (37·6 to 58·6)	-17·0 (-27·2 to -8·76)	46·0 (36·8 to 57·3)	241 (146 to 368)	201 (102 to 300)	-16·5 (-33·6 to -1·15)	157 (94·8 to 239)
	5-14 years	57·7 (40·1 to 79·4)	49·3 (34·8 to 69·2)	-14·3 (-23·2 to -4·03)	46·1 (32·1 to 63·5)	88·8 (62·4 to 116)	81·9 (62·0 to 115)	-7·35 (-23·5 to 17·1)	57·7 (40·6 to 75·4)
	15-49 years	143 (120 to 164)	133 (112 to 153)	-6·79 (-13·2 to 0·0402)	114 (96·3 to 131)	1100 (911 to 1360)	1100 (847 to 1370)	0·689 (-13·7 to 21·3)	714 (592 to 884)
	50-69 years	270 (226 to 321)	254 (214 to 303)	-5·59 (-11·3 to 2·50)	216 (181 to 257)	835 (655 to 1010)	958 (749 to 1270)	15·0 (-3·08 to 34·7)	543 (426 to 658)
	70+ years	533 (451 to 594)	486 (411 to 540)	-8·69 (-14·6 to -1·78)	426 (361 to 475)	505 (402 to 629)	554 (430 to 722)	10·1 (-10·6 to 32·3)	328 (261 to 409)
Samoa	All Ages	42·8 (38·3 to 47·1)	40·9 (36·4 to 45·5)	-4·43 (-9·16 to 1·74)	34·3 (30·7 to 37·7)	15·0 (11·6 to 19·5)	14·2 (10·7 to 18·8)	-5·08 (-20·2 to 10·4)	9·75 (7·52 to 12·7)

eTable 3. Percent change from 2015 to 2021 in age-standardised all-form tuberculosis incidence rate per 100,000 population and in deaths due to all-form tuberculosis by age for 204 countries and territories.

Location	Age group	2015 Rate	2021 Rate	Incidence Percent Change	Incidence Milestone	2015 Deaths	2021 Deaths	Mortality Percent Change	Mortality Milestone
	Under 5	14·9 (11·7 to 19·4)	13·7 (10·9 to 17·0)	-8·22 (-20·7 to 2·96)	12·0 (9·39 to 15·5)	0·330 (0·226 to 0·467)	0·300 (0·198 to 0·449)	-8·07 (-33·0 to 24·6)	0·214 (0·147 to 0·303)
	5-14 years	17·4 (12·0 to 24·4)	15·2 (11·0 to 21·7)	-12·4 (-25·9 to 8·98)	13·9 (9·59 to 19·5)	0·245 (0·178 to 0·351)	0·215 (0·158 to 0·292)	-11·1 (-30·7 to 13·5)	0·159 (0·116 to 0·228)
	15-49 years	44·4 (36·1 to 52·3)	42·4 (34·7 to 50·0)	-4·63 (-10·4 to 1·11)	35·6 (28·9 to 41·9)	4·10 (2·92 to 5·65)	3·82 (2·65 to 4·92)	-6·15 (-26·4 to 18·0)	2·67 (1·90 to 3·67)
	50-69 years	93·5 (76·9 to 115)	90·3 (74·8 to 109)	-3·32 (-10·7 to 2·73)	74·8 (61·5 to 91·8)	5·47 (3·99 to 7·19)	5·39 (3·90 to 7·64)	-0·842 (-21·2 to 22·3)	3·56 (2·59 to 4·67)
	70+ years	146 (119 to 174)	136 (110 to 163)	-7·18 (-16·3 to 4·42)	117 (95·1 to 139)	4·85 (3·70 to 6·72)	4·45 (3·23 to 6·10)	-7·88 (-22·6 to 13·0)	3·15 (2·41 to 4·37)
Solomon Islands	All Ages	64·6 (58·9 to 71·8)	63·3 (56·6 to 70·6)	-2·04 (-6·65 to 2·94)	51·7 (47·1 to 57·4)	83·6 (62·2 to 110)	84·9 (66·5 to 109)	2·02 (-12·3 to 22·9)	54·3 (40·4 to 71·4)
	Under 5	27·0 (21·6 to 34·1)	24·7 (19·2 to 30·7)	-8·25 (-18·4 to 5·02)	21·6 (17·3 to 27·3)	2·97 (2·15 to 3·95)	2·41 (1·74 to 3·25)	-17·7 (-36·0 to 9·21)	1·93 (1·40 to 2·57)
	5-14 years	28·3 (18·7 to 39·9)	27·2 (18·1 to 38·1)	-3·61 (-17·9 to 9·74)	22·6 (14·9 to 31·9)	2·32 (1·60 to 3·31)	1·97 (1·37 to 3·01)	-14·2 (-33·4 to 5·50)	1·51 (1·04 to 2·15)
	15-49 years	72·6 (62·2 to 84·6)	70·1 (57·8 to 80·4)	-3·44 (-9·77 to 3·43)	58·0 (49·7 to 67·7)	35·3 (25·0 to 47·4)	35·7 (26·2 to 44·4)	1·63 (-15·8 to 24·2)	22·9 (16·2 to 30·8)
	50-69 years	146 (119 to 175)	138 (111 to 166)	-5·71 (-11·4 to 2·29)	117 (95·3 to 140)	25·0 (17·5 to 34·2)	27·0 (20·5 to 37·6)	9·06 (-10·6 to 37·3)	16·2 (11·4 to 22·2)
	70+ years	255 (215 to 289)	243 (204 to 273)	-4·44 (-13·1 to 4·76)	204 (172 to 231)	18·0 (13·4 to 22·3)	17·8 (14·1 to 22·0)	-0·708 (-15·7 to 17·6)	11·7 (8·74 to 14·5)
Tokelau	All Ages	39·2 (34·9 to 43·1)	35·6 (32·3 to 39·4)	-9·03 (-13·5 to -4·73)	31·3 (27·9 to 34·5)	0·102 (0·0726 to 0·143)	0·0852 (0·0653 to 0·117)	-16·1 (-29·5 to 1·68)	0·0663 (0·0472 to 0·0928)
	Under 5	10·3 (8·27 to 13·5)	9·11 (7·15 to 11·7)	-11·3 (-20·1 to -1·67)	8·23 (6·61 to 10·8)	0·000688 (0·000476 to 0·000987)	0·000592 (0·000430 to 0·000903)	-13·3 (-29·7 to 11·4)	0·000447 (0·000309 to 0·000642)
	5-14 years	12·5 (8·53 to 18·1)	10·7 (7·28 to 15·1)	-14·4 (-23·7 to -3·65)	10·0 (6·83 to 14·5)	0·000896 (0·000613 to 0·00118)	0·000739 (0·000533 to 0·000979)	-16·8 (-33·1 to 3·05)	0·000583 (0·000399 to 0·000766)
	15-49 years	35·0 (29·2 to 40·3)	31·9 (26·4 to 37·7)	-8·90 (-14·3 to -2·80)	28·0 (23·3 to 32·2)	0·0194 (0·0134 to 0·0293)	0·0172 (0·0129 to 0·0241)	-10·2 (-25·9 to 9·40)	0·0126 (0·00869 to 0·0190)
	50-69 years	69·9 (55·1 to 85·0)	63·8 (51·7 to 78·3)	-8·70 (-13·9 to -2·64)	55·9 (44·1 to 68·0)	0·0337 (0·0233 to 0·0484)	0·0290 (0·0203 to 0·0415)	-13·2 (-30·4 to 7·80)	0·0219 (0·0152 to 0·0315)
	70+ years	112 (90·8 to 135)	98·9 (81·0 to 120)	-11·3 (-17·7 to -1·18)	89·3 (72·7 to 108)	0·0475 (0·0336 to 0·0671)	0·0376 (0·0275 to 0·0498)	-20·2 (-33·7 to -3·11)	0·0309 (0·0218 to 0·0436)
Tonga	All Ages	32·6 (28·8 to 35·8)	30·8 (27·0 to 33·8)	-5·55 (-10·8 to 0·261)	26·1 (23·1 to 28·6)	5·79 (4·52 to 7·52)	5·01 (3·81 to 6·44)	-13·2 (-27·6 to 0·962)	3·76 (2·94 to 4·89)
	Under 5	11·5 (9·12 to 14·7)	9·78 (7·76 to 12·9)	-14·4 (-23·5 to -2·15)	9·17 (7·29 to 11·7)	0·124 (0·0940 to 0·154)	0·0836 (0·0609 to 0·111)	-32·0 (-47·1 to -14·1)	0·0803 (0·0611 to 0·100)
	5-14 years	13·0 (9·01 to 18·0)	11·4 (7·84 to 16·0)	-12·6 (-25·4 to 0·859)	10·4 (7·21 to 14·4)	0·0867 (0·0649 to 0·114)	0·0678 (0·0463 to 0·0902)	-21·5 (-38·2 to -0·000516)	0·0564 (0·0422 to 0·0742)
	15-49 years	32·7 (26·6 to 38·6)	30·6 (24·3 to 36·1)	-6·25 (-13·7 to -0·243)	26·1 (21·3 to 30·9)	1·31 (0·951 to 1·74)	1·08 (0·743 to 1·44)	-17·2 (-33·5 to -0·684)	0·851 (0·618 to 1·13)

eTable 3. Percent change from 2015 to 2021 in age-standardised all-form tuberculosis incidence rate per 100,000 population and in deaths due to all-form tuberculosis by age for 204 countries and territories.

Location	Age group	2015 Rate	2021 Rate	Incidence Percent Change	Incidence Milestone	2015 Deaths	2021 Deaths	Mortality Percent Change	Mortality Milestone
	50-69 years	70·4 (56·9 to 84·6)	65·2 (53·1 to 79·2)	-7·36 (-13·0 to -2·45)	56·3 (45·5 to 67·7)	2·02 (1·46 to 2·93)	1·85 (1·34 to 2·49)	-8·05 (-24·9 to 12·5)	1·31 (0·947 to 1·90)
	70+ years	111 (89·4 to 135)	103 (82·9 to 123)	-7·39 (-17·2 to 0·0863)	88·8 (71·5 to 108)	2·25 (1·80 to 3·05)	1·93 (1·54 to 2·54)	-13·6 (-27·6 to 2·79)	1·46 (1·17 to 1·98)
Tuvalu	All Ages	229 (205 to 252)	211 (190 to 232)	-7·68 (-11·5 to -2·96)	183 (164 to 202)	5·08 (3·31 to 6·25)	4·57 (3·18 to 5·60)	-9·81 (-24·9 to 5·05)	3·30 (2·15 to 4·06)
	Under 5	73·5 (57·6 to 94·1)	62·7 (49·0 to 78·8)	-14·4 (-25·0 to -2·54)	58·8 (46·1 to 75·3)	0·0857 (0·0605 to 0·115)	0·0634 (0·0457 to 0·0849)	-25·7 (-39·0 to -7·22)	0·0557 (0·0393 to 0·0746)
	5-14 years	99·6 (65·5 to 143)	83·6 (58·5 to 120)	-15·9 (-26·3 to -2·73)	79·7 (52·4 to 115)	0·0840 (0·0620 to 0·113)	0·0610 (0·0460 to 0·0788)	-27·0 (-39·9 to -11·9)	0·0546 (0·0403 to 0·0732)
	15-49 years	220 (179 to 254)	201 (163 to 233)	-8·51 (-14·4 to -2·91)	176 (143 to 203)	1·34 (0·849 to 1·73)	1·20 (0·828 to 1·58)	-9·50 (-27·4 to 9·95)	0·871 (0·552 to 1·12)
	50-69 years	436 (361 to 537)	408 (340 to 489)	-6·16 (-12·7 to -1·14)	349 (288 to 429)	2·04 (1·35 to 2·61)	1·81 (1·21 to 2·39)	-10·7 (-27·4 to 4·22)	1·32 (0·876 to 1·70)
	70+ years	671 (566 to 777)	600 (489 to 697)	-10·6 (-17·8 to -3·70)	537 (453 to 622)	1·54 (1·02 to 1·98)	1·42 (0·991 to 1·78)	-6·45 (-24·4 to 13·1)	0·998 (0·665 to 1·29)
Vanuatu	All Ages	53·5 (48·3 to 59·9)	52·8 (47·2 to 58·5)	-1·22 (-5·43 to 2·86)	42·8 (38·6 to 47·9)	32·5 (18·9 to 47·0)	35·0 (21·5 to 49·0)	8·21 (-6·45 to 22·5)	21·1 (12·3 to 30·5)
	Under 5	21·1 (17·0 to 27·2)	19·9 (15·8 to 24·9)	-5·44 (-16·9 to 6·10)	16·9 (13·6 to 21·8)	1·05 (0·628 to 1·58)	0·974 (0·563 to 1·46)	-6·61 (-25·8 to 9·91)	0·683 (0·408 to 1·03)
	5-14 years	24·5 (16·2 to 34·7)	24·0 (16·7 to 33·4)	-1·65 (-14·3 to 9·04)	19·6 (13·0 to 27·7)	0·825 (0·519 to 1·13)	0·779 (0·520 to 1·05)	-4·59 (-22·7 to 19·2)	0·536 (0·337 to 0·737)
	15-49 years	59·0 (49·6 to 68·5)	56·9 (47·7 to 64·9)	-3·45 (-10·5 to 2·56)	47·2 (39·6 to 54·8)	12·1 (6·93 to 17·5)	13·0 (7·63 to 18·8)	7·55 (-9·33 to 27·1)	7·88 (4·51 to 11·4)
	50-69 years	119 (97·5 to 141)	115 (94·1 to 138)	-3·87 (-8·84 to 2·41)	95·4 (78·0 to 113)	11·6 (6·01 to 17·6)	12·8 (6·84 to 19·4)	11·6 (-8·76 to 33·7)	7·52 (3·90 to 11·4)
	70+ years	187 (158 to 212)	173 (142 to 194)	-7·28 (-15·0 to -0·374)	149 (127 to 169)	6·95 (4·46 to 9·54)	7·49 (5·18 to 10·9)	8·39 (-7·27 to 28·8)	4·52 (2·90 to 6·20)
Southeast Asia	All Ages	192 (174 to 211)	188 (172 to 208)	-2·01 (-3·57 to -0·392)	154 (139 to 169)	204000 (186000 to 221000)	183000 (165000 to 217000)	-9·98 (-19·0 to 5·04)	132000 (121000 to 144000)
	Under 5	68·2 (56·7 to 84·6)	61·2 (50·9 to 76·4)	-10·2 (-13·8 to -7·36)	54·5 (45·3 to 67·7)	4950 (4040 to 5810)	2970 (2310 to 3660)	-40·0 (-47·4 to -32·7)	3220 (2630 to 3780)
	5-14 years	56·4 (39·8 to 77·3)	54·4 (38·5 to 74·9)	-3·66 (-6·60 to -0·00996)	45·1 (31·8 to 61·8)	2490 (2220 to 2740)	1560 (1360 to 1770)	-37·4 (-44·0 to -31·5)	1620 (1440 to 1780)
	15-49 years	172 (146 to 197)	159 (133 to 184)	-7·79 (-9·23 to -6·43)	138 (116 to 158)	62600 (57700 to 68900)	48700 (43800 to 56000)	-22·2 (-30·8 to -11·0)	40700 (37500 to 44800)
	50-69 years	384 (314 to 464)	366 (300 to 444)	-4·68 (-6·77 to -2·71)	307 (251 to 371)	70700 (64100 to 77300)	69800 (61600 to 84500)	-0·988 (-13·0 to 17·8)	45900 (41700 to 50300)
	70+ years	625 (536 to 723)	573 (487 to 671)	-8·43 (-10·9 to -6·12)	500 (429 to 578)	62900 (55000 to 69400)	60100 (53200 to 71700)	-4·42 (-13·6 to 11·9)	40900 (35800 to 45100)
Cambodia	All Ages	298 (269 to 329)	252 (225 to 281)	-15·6 (-19·4 to -12·2)	239 (215 to 263)	7570 (5300 to 9670)	6900 (4650 to 9440)	-8·88 (-19·8 to 9·15)	4920 (3450 to 6290)
	Under 5	99·3 (81·3 to 122)	74·3 (59·6 to 91·7)	-25·0 (-34·7 to -13·1)	79·4 (65·1 to 98·0)	229 (128 to 347)	132 (76·5 to 215)	-41·8 (-54·8 to -27·0)	149 (83·3 to 225)

eTable 3. Percent change from 2015 to 2021 in age-standardised all-form tuberculosis incidence rate per 100,000 population and in deaths due to all-form tuberculosis by age for 204 countries and territories.

Location	Age group	2015 Rate	2021 Rate	Incidence Percent Change	Incidence Milestone	2015 Deaths	2021 Deaths	Mortality Percent Change	Mortality Milestone
	5-14 years	91·0 (62·8 to 125)	63·8 (42·4 to 90·4)	-29·8 (-39·4 to -20·0)	72·8 (50·3 to 100)	141 (94·2 to 194)	83·0 (55·6 to 121)	-41·0 (-51·3 to -24·6)	91·4 (61·2 to 126)
	15-49 years	289 (241 to 331)	234 (195 to 276)	-19·0 (-24·8 to -14·1)	231 (193 to 265)	2390 (1750 to 3180)	1920 (1320 to 2720)	-19·6 (-32·7 to -1·91)	1550 (1130 to 2070)
	50-69 years	701 (565 to 847)	594 (472 to 719)	-15·2 (-23·6 to -9·25)	561 (452 to 677)	2890 (1980 to 3990)	2840 (1830 to 3870)	-1·43 (-15·7 to 15·3)	1880 (1290 to 2590)
	70+ years	993 (794 to 1200)	800 (652 to 943)	-19·4 (-27·5 to -8·77)	794 (635 to 957)	1930 (1220 to 2510)	1920 (1330 to 2720)	-0·198 (-17·3 to 19·1)	1260 (790 to 1630)
Indonesia	All Ages	190 (171 to 208)	178 (161 to 197)	-6·17 (-8·56 to -4·18)	152 (137 to 167)	102000 (87000 to 117000)	93100 (80700 to 112000)	-8·09 (-22·1 to 14·2)	66200 (56600 to 75800)
	Under 5	73·7 (60·7 to 90·7)	59·2 (49·0 to 72·3)	-19·5 (-23·6 to -14·8)	58·9 (48·6 to 72·6)	2470 (1950 to 3060)	1520 (1130 to 1960)	-38·4 (-48·4 to -26·4)	1600 (1270 to 1990)
	5-14 years	56·3 (38·7 to 78·8)	44·1 (30·9 to 62·2)	-21·8 (-25·6 to -18·6)	45·1 (30·9 to 63·0)	1120 (935 to 1310)	688 (582 to 832)	-38·5 (-49·8 to -28·2)	730 (608 to 849)
	15-49 years	151 (125 to 172)	129 (107 to 148)	-14·7 (-16·6 to -13·0)	121 (100 to 137)	30400 (26600 to 35800)	24400 (20800 to 29900)	-19·5 (-34·5 to -1·57)	19800 (17300 to 23300)
	50-69 years	438 (358 to 532)	400 (325 to 483)	-8·71 (-11·5 to -6·19)	350 (287 to 425)	37200 (31200 to 43400)	37300 (31100 to 45400)	0·806 (-17·8 to 29·9)	24200 (20300 to 28200)
	70+ years	806 (668 to 941)	745 (623 to 884)	-7·51 (-11·4 to -3·87)	644 (534 to 753)	30600 (25600 to 34800)	29300 (25300 to 35400)	-3·79 (-20·5 to 21·3)	19900 (16600 to 22600)
Lao People's Democratic Republic	All Ages	128 (113 to 148)	108 (92·0 to 125)	-15·8 (-20·5 to -11·1)	102 (90·6 to 119)	2220 (1440 to 2970)	1880 (1240 to 2600)	-15·5 (-27·9 to -0·988)	1440 (935 to 1930)
	Under 5	51·4 (40·9 to 63·1)	38·2 (30·1 to 47·6)	-25·6 (-37·6 to -12·5)	41·1 (32·7 to 50·5)	122 (80·5 to 167)	76·0 (53·2 to 115)	-37·0 (-56·6 to -14·1)	79·2 (52·3 to 109)
	5-14 years	41·3 (27·7 to 59·9)	30·6 (21·6 to 44·0)	-25·7 (-37·2 to -15·0)	33·0 (22·1 to 48·0)	47·9 (32·4 to 69·4)	31·0 (20·2 to 43·6)	-34·9 (-50·7 to -19·2)	31·1 (21·1 to 45·1)
	15-49 years	119 (95·8 to 147)	95·8 (78·2 to 121)	-19·3 (-24·4 to -14·4)	95·0 (76·6 to 118)	801 (537 to 1090)	618 (391 to 906)	-22·7 (-35·0 to -6·41)	520 (349 to 707)
	50-69 years	344 (256 to 440)	284 (218 to 358)	-17·3 (-23·8 to -9·11)	275 (205 to 352)	737 (470 to 1020)	677 (416 to 952)	-7·81 (-26·3 to 13·5)	479 (305 to 664)
	70+ years	529 (422 to 662)	428 (341 to 544)	-19·0 (-25·5 to -12·2)	423 (338 to 530)	514 (305 to 690)	473 (300 to 664)	-7·52 (-26·6 to 13·6)	334 (198 to 448)
Malaysia	All Ages	65·4 (57·5 to 74·6)	64·8 (57·2 to 72·5)	-0·862 (-5·25 to 3·44)	52·3 (46·0 to 59·7)	2260 (2000 to 2570)	2350 (2020 to 2820)	3·81 (-5·62 to 17·8)	1470 (1300 to 1670)
	Under 5	13·1 (10·5 to 17·0)	11·6 (9·42 to 14·7)	-11·4 (-23·5 to 0·589)	10·5 (8·44 to 13·6)	13·5 (10·3 to 16·2)	8·77 (6·68 to 11·0)	-34·8 (-46·8 to -23·6)	8·78 (6·71 to 10·5)
	5-14 years	13·7 (9·18 to 19·6)	11·9 (8·15 to 16·9)	-13·5 (-23·1 to -2·91)	11·0 (7·34 to 15·7)	18·2 (14·1 to 22·8)	13·0 (10·3 to 16·1)	-28·3 (-38·9 to -17·4)	11·9 (9·19 to 14·9)
	15-49 years	62·1 (51·2 to 75·8)	59·7 (49·3 to 73·0)	-3·74 (-10·0 to 2·23)	49·7 (40·9 to 60·6)	705 (586 to 859)	696 (568 to 884)	-1·29 (-10·9 to 13·8)	458 (381 to 558)
	50-69 years	136 (104 to 168)	129 (97·6 to 159)	-5·00 (-12·9 to 1·50)	109 (83·2 to 134)	838 (728 to 962)	826 (675 to 990)	-1·32 (-16·1 to 16·7)	545 (473 to 625)
	70+ years	207 (166 to 256)	187 (150 to 235)	-9·95 (-18·6 to -1·56)	166 (133 to 205)	687 (583 to 793)	803 (673 to 1000)	17·1 (1·62 to 31·1)	446 (379 to 515)

eTable 3. Percent change from 2015 to 2021 in age-standardised all-form tuberculosis incidence rate per 100,000 population and in deaths due to all-form tuberculosis by age for 204 countries and territories.

Location	Age group	2015 Rate	2021 Rate	Incidence Percent Change	Incidence Milestone	2015 Deaths	2021 Deaths	Mortality Percent Change	Mortality Milestone
Maldives	All Ages	51·7 (45·2 to 59·5)	49·3 (43·4 to 56·2)	-4·62 (-9·48 to 0·813)	41·4 (36·2 to 47·6)	18·5 (16·5 to 20·8)	17·6 (13·8 to 23·5)	-4·53 (-20·0 to 19·4)	12·0 (10·7 to 13·5)
	Under 5	17·1 (13·5 to 21·0)	14·3 (11·4 to 17·8)	-16·2 (-26·5 to -6·16)	13·7 (10·8 to 16·8)	0·410 (0·323 to 0·536)	0·205 (0·143 to 0·275)	-49·7 (-63·0 to -38·0)	0·266 (0·210 to 0·348)
	5-14 years	16·8 (11·7 to 24·4)	12·8 (8·86 to 17·7)	-23·6 (-33·1 to -13·8)	13·5 (9·39 to 19·5)	0·267 (0·204 to 0·333)	0·161 (0·129 to 0·228)	-38·9 (-53·0 to -6·43)	0·174 (0·132 to 0·216)
	15-49 years	45·6 (37·2 to 57·0)	41·8 (34·4 to 51·1)	-8·22 (-15·3 to -0·394)	36·5 (29·8 to 45·6)	6·35 (5·52 to 7·39)	6·76 (4·93 to 8·94)	6·74 (-21·6 to 36·4)	4·12 (3·59 to 4·80)
	50-69 years	117 (84·9 to 142)	108 (84·3 to 133)	-7·49 (-15·4 to 1·59)	93·5 (67·9 to 114)	4·58 (3·73 to 5·74)	4·90 (3·44 to 6·62)	7·22 (-15·9 to 40·6)	2·98 (2·42 to 3·73)
	70+ years	236 (188 to 302)	221 (182 to 270)	-6·12 (-15·6 to 1·24)	189 (151 to 241)	6·86 (5·95 to 7·84)	5·60 (4·43 to 7·25)	-18·3 (-30·8 to -1·51)	4·46 (3·87 to 5·10)
Mauritius	All Ages	14·6 (12·9 to 16·8)	16·0 (14·0 to 18·6)	10·0 (4·86 to 15·6)	11·7 (10·3 to 13·4)	16·9 (15·6 to 18·2)	16·3 (14·8 to 17·7)	-3·51 (-10·5 to 1·29)	11·0 (10·1 to 11·8)
	Under 5	5·85 (4·39 to 7·48)	5·73 (4·44 to 7·68)	-1·94 (-12·3 to 9·71)	4·68 (3·51 to 5·99)	0·0685 (0·0588 to 0·0771)	0·0490 (0·0418 to 0·0574)	-28·2 (-38·1 to -14·6)	0·0445 (0·0382 to 0·0501)
	5-14 years	3·85 (2·37 to 5·70)	3·71 (2·33 to 5·47)	-3·18 (-15·4 to 8·08)	3·08 (1·89 to 4·56)	0·0604 (0·0511 to 0·0718)	0·0445 (0·0353 to 0·0561)	-26·4 (-37·6 to -18·4)	0·0393 (0·0332 to 0·0466)
	15-49 years	14·0 (11·6 to 17·1)	14·7 (12·1 to 18·2)	5·35 (-1·63 to 12·0)	11·2 (9·30 to 13·7)	5·45 (4·81 to 6·25)	4·97 (4·24 to 5·81)	-8·79 (-15·8 to -3·80)	3·54 (3·13 to 4·06)
	50-69 years	20·8 (15·7 to 25·6)	22·3 (17·1 to 27·8)	7·31 (-3·31 to 17·3)	16·6 (12·6 to 20·5)	7·19 (6·56 to 7·86)	7·01 (6·31 to 7·82)	-2·39 (-12·0 to 5·34)	4·67 (4·26 to 5·11)
	70+ years	28·3 (22·4 to 35·9)	28·6 (22·4 to 35·9)	1·08 (-8·74 to 12·5)	22·7 (17·9 to 28·7)	4·09 (3·74 to 4·43)	4·19 (3·81 to 4·58)	2·38 (-5·29 to 10·3)	2·66 (2·43 to 2·88)
Myanmar	All Ages	219 (200 to 243)	173 (158 to 192)	-21·1 (-24·4 to -18·0)	176 (160 to 194)	22100 (18800 to 26500)	17000 (13300 to 22300)	-22·9 (-33·0 to -6·94)	14300 (12200 to 17200)
	Under 5	71·9 (57·3 to 90·1)	53·6 (42·8 to 67·2)	-25·1 (-35·5 to -10·6)	57·5 (45·9 to 72·1)	922 (570 to 1230)	533 (334 to 763)	-41·8 (-57·4 to -23·8)	599 (370 to 803)
	5-14 years	69·2 (46·2 to 98·3)	47·2 (31·4 to 66·6)	-31·7 (-39·7 to -22·3)	55·3 (37·0 to 78·6)	379 (268 to 492)	203 (145 to 278)	-46·3 (-56·8 to -32·9)	246 (174 to 320)
	15-49 years	215 (183 to 248)	162 (137 to 188)	-24·6 (-28·2 to -19·9)	172 (146 to 199)	7900 (6440 to 9500)	4990 (3900 to 6700)	-36·9 (-46·2 to -23·4)	5130 (4190 to 6180)
	50-69 years	424 (342 to 509)	338 (277 to 407)	-20·2 (-25·4 to -15·3)	339 (274 to 407)	7070 (5560 to 8900)	6200 (4530 to 8410)	-12·4 (-29·2 to 12·1)	4600 (3610 to 5780)
	70+ years	665 (560 to 750)	504 (400 to 583)	-24·1 (-31·8 to -16·7)	532 (448 to 600)	5790 (4640 to 7280)	5120 (3700 to 7000)	-11·8 (-26·7 to 9·07)	3770 (3020 to 4730)
Philippines	All Ages	265 (237 to 294)	311 (275 to 351)	17·2 (14·8 to 20·5)	212 (190 to 235)	28000 (26200 to 30100)	26200 (21500 to 30800)	-6·54 (-21·8 to 14·5)	18200 (17100 to 19600)
	Under 5	103 (85·8 to 131)	116 (93·6 to 149)	11·7 (7·06 to 16·7)	82·8 (68·6 to 105)	851 (681 to 1030)	509 (394 to 656)	-39·9 (-52·9 to -17·9)	553 (442 to 671)
	5-14 years	85·6 (62·3 to 118)	123 (87·8 to 170)	43·2 (37·0 to 49·9)	68·5 (49·8 to 94·2)	610 (540 to 696)	420 (368 to 478)	-31·0 (-40·9 to -19·7)	397 (351 to 452)
	15-49 years	272 (226 to 317)	297 (241 to 345)	9·18 (5·94 to 13·0)	217 (181 to 254)	8360 (7770 to 9000)	7410 (6000 to 8780)	-11·3 (-26·4 to 8·61)	5440 (5050 to 5850)

eTable 3. Percent change from 2015 to 2021 in age-standardised all-form tuberculosis incidence rate per 100,000 population and in deaths due to all-form tuberculosis by age for 204 countries and territories.

Location	Age group	2015 Rate	2021 Rate	Incidence Percent Change	Incidence Milestone	2015 Deaths	2021 Deaths	Mortality Percent Change	Mortality Milestone
	50-69 years	583 (472 to 722)	650 (515 to 828)	11·5 (7·67 to 14·5)	466 (378 to 578)	11000 (10200 to 11900)	10700 (8580 to 13100)	-2·41 (-20·1 to 22·1)	7160 (6620 to 7740)
	70+ years	747 (620 to 849)	872 (720 to 1000)	16·7 (12·4 to 21·0)	598 (496 to 679)	7180 (6690 to 7680)	7080 (6010 to 8140)	-1·28 (-15·5 to 17·8)	4660 (4350 to 4990)
Seychelles	All Ages	36·0 (31·8 to 41·1)	35·2 (30·7 to 40·0)	-2·01 (-8·01 to 3·02)	28·8 (25·4 to 32·9)	4·37 (3·99 to 4·89)	3·69 (3·16 to 4·44)	-15·6 (-26·5 to -5·39)	2·84 (2·59 to 3·18)
	Under 5	8·54 (6·69 to 10·3)	8·05 (6·49 to 10·1)	-5·51 (-17·1 to 9·05)	6·83 (5·35 to 8·26)	0·0292 (0·0226 to 0·0348)	0·0247 (0·0196 to 0·0312)	-15·1 (-27·7 to 4·26)	0·0190 (0·0147 to 0·0226)
	5-14 years	5·76 (3·97 to 8·27)	5·36 (3·49 to 7·65)	-6·74 (-20·6 to 2·43)	4·61 (3·18 to 6·61)	0·0262 (0·0191 to 0·0329)	0·0109 (0·00868 to 0·0139)	-57·9 (-68·0 to -42·9)	0·0170 (0·0124 to 0·0214)
	15-49 years	34·5 (29·0 to 41·4)	32·5 (26·7 to 38·8)	-5·79 (-12·8 to -0·236)	27·6 (23·2 to 33·1)	1·47 (1·25 to 1·72)	1·08 (0·869 to 1·31)	-26·4 (-37·1 to -13·5)	0·958 (0·814 to 1·12)
	50-69 years	63·6 (50·3 to 79·6)	61·0 (46·5 to 75·5)	-3·99 (-9·90 to 3·94)	50·9 (40·3 to 63·7)	1·58 (1·33 to 1·89)	1·40 (1·11 to 1·76)	-11·3 (-25·8 to 3·85)	1·03 (0·863 to 1·23)
	70+ years	86·9 (68·9 to 109)	80·5 (61·1 to 99·6)	-7·23 (-17·2 to 3·74)	69·5 (55·1 to 87·1)	1·26 (1·13 to 1·45)	1·17 (0·991 to 1·41)	-7·22 (-20·4 to 4·53)	0·819 (0·733 to 0·943)
Sri Lanka	All Ages	50·8 (44·5 to 58·8)	51·2 (45·6 to 58·3)	0·861 (-4·34 to 5·92)	40·7 (35·6 to 47·1)	1070 (907 to 1280)	909 (620 to 1300)	-14·9 (-39·5 to 11·4)	694 (590 to 830)
	Under 5	11·3 (8·91 to 14·0)	10·4 (8·05 to 13·1)	-8·07 (-21·3 to 7·50)	9·05 (7·13 to 11·2)	5·88 (4·80 to 6·93)	3·05 (2·22 to 4·08)	-48·1 (-59·7 to -31·4)	3·82 (3·12 to 4·50)
	5-14 years	9·23 (6·47 to 13·1)	8·30 (5·58 to 12·0)	-10·0 (-19·9 to 1·03)	7·38 (5·17 to 10·5)	5·43 (4·25 to 6·71)	3·64 (2·54 to 5·09)	-32·8 (-49·9 to -14·5)	3·53 (2·76 to 4·36)
	15-49 years	42·2 (35·1 to 51·4)	41·9 (34·6 to 51·4)	-0·786 (-8·04 to 6·79)	33·8 (28·0 to 41·1)	191 (163 to 232)	169 (117 to 237)	-11·5 (-37·1 to 18·8)	124 (106 to 151)
	50-69 years	102 (75·6 to 126)	95·9 (75·2 to 118)	-5·83 (-13·7 to 1·42)	81·6 (60·5 to 101)	457 (366 to 548)	354 (222 to 536)	-22·5 (-49·6 to 5·19)	297 (238 to 356)
	70+ years	139 (110 to 178)	125 (94·6 to 158)	-9·92 (-17·8 to -2·04)	111 (88·3 to 143)	409 (334 to 505)	380 (267 to 513)	-7·04 (-33·9 to 24·2)	266 (217 to 329)
Thailand	All Ages	173 (153 to 201)	174 (152 to 198)	0·201 (-4·61 to 5·81)	139 (122 to 161)	12700 (10800 to 14900)	12700 (10600 to 15000)	0·702 (-14·8 to 29·2)	8250 (7030 to 9680)
	Under 5	27·1 (21·3 to 35·1)	23·5 (18·0 to 29·8)	-12·9 (-22·3 to -0·774)	21·6 (17·0 to 28·1)	43·4 (36·3 to 49·4)	25·6 (21·1 to 31·2)	-41·1 (-49·1 to -30·8)	28·2 (23·6 to 32·1)
	5-14 years	44·6 (30·4 to 61·7)	39·1 (26·7 to 54·8)	-12·0 (-22·7 to -0·734)	35·7 (24·3 to 49·3)	39·7 (34·1 to 46·5)	27·5 (21·9 to 35·7)	-30·6 (-45·0 to -14·1)	25·8 (22·2 to 30·2)
	15-49 years	151 (123 to 182)	143 (114 to 175)	-5·01 (-11·6 to 0·253)	121 (98·7 to 146)	4160 (3030 to 5590)	3180 (2210 to 4180)	-23·1 (-31·4 to -12·2)	2700 (1970 to 3640)
	50-69 years	247 (184 to 306)	233 (179 to 286)	-5·31 (-13·1 to 1·51)	197 (148 to 245)	3480 (2880 to 4130)	3880 (2970 to 5020)	11·8 (-9·50 to 42·8)	2260 (1870 to 2680)
	70+ years	410 (332 to 513)	368 (300 to 462)	-10·2 (-18·8 to -0·382)	328 (266 to 410)	4960 (4060 to 5800)	5610 (4310 to 6960)	13·8 (-8·63 to 52·5)	3220 (2640 to 3770)
Timor-Leste	All Ages	262 (237 to 288)	264 (241 to 290)	0·822 (-2·49 to 5·51)	210 (189 to 230)	608 (414 to 954)	649 (446 to 976)	7·17 (-6·60 to 22·3)	395 (269 to 620)
	Under 5	113 (88·7 to 141)	110 (89·7 to 138)	-2·66 (-13·9 to 8·96)	90·6 (70·9 to 113)	37·4 (24·8 to 50·8)	34·4 (24·6 to 47·9)	-7·01 (-28·9 to 21·8)	24·3 (16·1 to 33·0)

eTable 3. Percent change from 2015 to 2021 in age-standardised all-form tuberculosis incidence rate per 100,000 population and in deaths due to all-form tuberculosis by age for 204 countries and territories.

Location	Age group	2015 Rate	2021 Rate	Incidence Percent Change	Incidence Milestone	2015 Deaths	2021 Deaths	Mortality Percent Change	Mortality Milestone
	5-14 years	90·8 (62·1 to 129)	89·8 (59·8 to 128)	-0·870 (-11·5 to 10·9)	72·6 (49·7 to 103)	13·8 (10·2 to 18·4)	12·1 (9·32 to 16·5)	-11·5 (-28·5 to 9·90)	8·97 (6·60 to 12·0)
	15-49 years	216 (178 to 250)	218 (179 to 251)	1·12 (-4·46 to 7·04)	173 (142 to 200)	135 (91·9 to 170)	144 (98·6 to 197)	6·94 (-10·4 to 29·4)	87·7 (59·7 to 111)
	50-69 years	831 (683 to 1010)	795 (650 to 963)	-4·37 (-9·58 to 2·99)	665 (546 to 810)	226 (153 to 364)	227 (151 to 342)	1·24 (-14·9 to 25·2)	147 (99·6 to 237)
	70+ years	1250 (1020 to 1440)	1230 (1010 to 1390)	-1·94 (-8·51 to 5·52)	1000 (818 to 1150)	196 (129 to 348)	232 (157 to 407)	18·8 (-1·18 to 40·0)	127 (83·9 to 227)
Viet Nam	All Ages	178 (163 to 195)	164 (149 to 180)	-7·77 (-11·9 to -3·18)	143 (130 to 156)	25000 (21400 to 29500)	21100 (17100 to 28100)	-15·7 (-25·8 to -0·657)	16300 (13900 to 19200)
	Under 5	40·9 (33·1 to 51·5)	33·0 (26·2 to 41·0)	-19·2 (-30·3 to -9·89)	32·8 (26·5 to 41·2)	254 (199 to 328)	128 (86·7 to 176)	-49·4 (-62·9 to -33·6)	165 (129 to 213)
	5-14 years	31·3 (21·8 to 44·4)	22·9 (15·9 to 32·0)	-26·9 (-35·4 to -17·2)	25·1 (17·5 to 35·5)	111 (89·6 to 139)	75·4 (55·6 to 100)	-31·6 (-45·1 to -10·1)	71·9 (58·2 to 90·4)
	15-49 years	168 (146 to 189)	150 (127 to 171)	-11·1 (-16·7 to -5·56)	135 (117 to 151)	7480 (5950 to 8990)	5090 (4130 to 6340)	-31·7 (-42·4 to -18·9)	4860 (3870 to 5840)
	50-69 years	315 (261 to 365)	297 (243 to 353)	-5·57 (-12·9 to 2·65)	252 (209 to 292)	6580 (5370 to 8280)	6710 (4760 to 9610)	1·65 (-15·1 to 22·5)	4280 (3490 to 5380)
	70+ years	628 (552 to 698)	539 (476 to 599)	-14·1 (-22·4 to -8·55)	502 (442 to 558)	10600 (8270 to 13000)	9110 (6900 to 11700)	-13·9 (-26·7 to 1·32)	6880 (5370 to 8430)
Sub-Saharan Africa	All Ages	333 (297 to 370)	265 (235 to 297)	-20·3 (-21·6 to -19·3)	266 (238 to 296)	646000 (557000 to 727000)	533000 (456000 to 609000)	-17·6 (-23·7 to -10·0)	420000 (362000 to 473000)
	Under 5	187 (156 to 227)	133 (111 to 163)	-28·7 (-30·8 to -26·1)	149 (125 to 182)	76700 (59000 to 90300)	44800 (31200 to 57800)	-41·7 (-50·0 to -32·0)	49800 (38300 to 58700)
	5-14 years	93·0 (64·9 to 128)	66·4 (45·9 to 92·6)	-28·7 (-31·1 to -26·9)	74·4 (51·9 to 102)	19900 (16700 to 22800)	12600 (10300 to 15100)	-36·6 (-42·2 to -31·5)	12900 (10900 to 14800)
	15-49 years	435 (366 to 515)	342 (289 to 406)	-21·4 (-22·8 to -20·2)	348 (293 to 412)	295000 (255000 to 341000)	240000 (204000 to 280000)	-18·5 (-24·2 to -11·7)	192000 (166000 to 221000)
	50-69 years	724 (581 to 897)	595 (468 to 741)	-17·9 (-19·7 to -15·7)	579 (465 to 717)	166000 (144000 to 187000)	154000 (130000 to 178000)	-7·32 (-14·9 to 2·15)	108000 (93300 to 122000)
	70+ years	1010 (836 to 1200)	831 (689 to 992)	-17·8 (-19·7 to -15·7)	808 (669 to 961)	89000 (77800 to 97200)	81400 (70200 to 91700)	-8·50 (-15·0 to 2·47)	57900 (50600 to 63200)
Central Sub-Saharan Africa	All Ages	396 (355 to 438)	325 (289 to 363)	-17·9 (-22·2 to -14·6)	317 (284 to 350)	96900 (75600 to 123000)	78600 (57000 to 105000)	-19·0 (-29·8 to -8·09)	63000 (49100 to 79700)
	Under 5	325 (264 to 394)	241 (195 to 298)	-25·7 (-31·6 to -18·7)	260 (211 to 315)	15500 (10500 to 22100)	7170 (4190 to 10300)	-53·7 (-61·8 to -44·7)	10100 (6810 to 14300)
	5-14 years	136 (95·9 to 187)	101 (68·8 to 138)	-25·8 (-32·2 to -18·7)	109 (76·7 to 149)	3260 (2500 to 4250)	2250 (1740 to 3140)	-30·7 (-40·1 to -19·4)	2120 (1630 to 2760)
	15-49 years	462 (383 to 531)	376 (311 to 441)	-18·8 (-24·0 to -13·9)	370 (307 to 424)	38800 (30100 to 49700)	31700 (22700 to 42300)	-18·3 (-28·8 to -7·27)	25200 (19600 to 32300)
	50-69 years	951 (777 to 1150)	836 (677 to 1050)	-12·2 (-16·5 to -6·17)	761 (622 to 922)	28300 (20300 to 36500)	27300 (18300 to 37400)	-3·51 (-17·8 to 12·3)	18400 (13200 to 23700)
	70+ years	1180 (976 to 1360)	968 (795 to 1150)	-18·0 (-24·9 to -11·4)	944 (781 to 1090)	11100 (7590 to 14600)	10100 (7130 to 13900)	-8·38 (-22·3 to 6·70)	7190 (4930 to 9470)

eTable 3. Percent change from 2015 to 2021 in age-standardised all-form tuberculosis incidence rate per 100,000 population and in deaths due to all-form tuberculosis by age for 204 countries and territories.

Location	Age group	2015 Rate	2021 Rate	Incidence Percent Change	Incidence Milestone	2015 Deaths	2021 Deaths	Mortality Percent Change	Mortality Milestone
Angola	All Ages	328 (293 to 366)	272 (242 to 304)	-17·0 (-21·6 to -12·0)	262 (234 to 293)	16600 (11700 to 21300)	15600 (11700 to 20400)	-5·66 (-18·3 to 10·3)	10800 (7590 to 13800)
	Under 5	287 (234 to 356)	211 (168 to 261)	-26·5 (-35·3 to -17·3)	230 (187 to 285)	3420 (2510 to 4400)	1990 (1300 to 2720)	-41·6 (-53·2 to -27·0)	2230 (1630 to 2860)
	5-14 years	107 (77·9 to 149)	80·8 (56·5 to 110)	-24·2 (-35·3 to -13·6)	85·6 (62·3 to 119)	571 (424 to 740)	446 (307 to 596)	-21·7 (-35·9 to -5·82)	371 (276 to 481)
	15-49 years	405 (337 to 461)	340 (282 to 404)	-16·0 (-22·0 to -8·87)	324 (269 to 369)	6700 (4660 to 9320)	6890 (4920 to 9520)	3·13 (-10·6 to 22·9)	4360 (3030 to 6060)
	50-69 years	751 (606 to 923)	679 (544 to 840)	-9·47 (-14·7 to -3·62)	600 (485 to 739)	4250 (2970 to 5800)	4570 (3300 to 5980)	8·69 (-12·8 to 36·9)	2760 (1930 to 3770)
	70+ years	887 (731 to 1030)	787 (629 to 935)	-11·2 (-20·7 to 1·13)	710 (585 to 826)	1650 (1060 to 2290)	1700 (1190 to 2270)	3·66 (-14·3 to 23·0)	1070 (690 to 1490)
Central African Republic	All Ages	678 (605 to 747)	608 (549 to 678)	-10·4 (-14·7 to -6·99)	543 (484 to 598)	11100 (8570 to 13500)	9500 (6730 to 12700)	-14·1 (-25·9 to -0·864)	7180 (5570 to 8750)
	Under 5	538 (434 to 652)	434 (348 to 547)	-19·1 (-27·4 to -9·06)	430 (347 to 521)	1630 (1190 to 2090)	1060 (744 to 1500)	-34·5 (-48·8 to -16·7)	1060 (774 to 1360)
	5-14 years	223 (162 to 314)	207 (140 to 299)	-7·24 (-19·6 to 5·89)	178 (129 to 251)	335 (258 to 411)	264 (189 to 338)	-21·0 (-32·6 to -5·83)	217 (168 to 267)
	15-49 years	812 (683 to 925)	715 (607 to 817)	-12·0 (-17·4 to -7·08)	650 (546 to 740)	5040 (3880 to 6310)	4180 (3110 to 5660)	-17·1 (-27·9 to -5·54)	3270 (2520 to 4100)
	50-69 years	1530 (1290 to 1790)	1400 (1180 to 1660)	-8·82 (-15·5 to -2·58)	1230 (1030 to 1430)	3220 (2390 to 4270)	3210 (2100 to 4280)	-0·335 (-14·6 to 14·5)	2100 (1550 to 2770)
	70+ years	1840 (1630 to 2060)	1670 (1390 to 1880)	-9·28 (-20·9 to -0·960)	1470 (1300 to 1650)	831 (553 to 1100)	781 (513 to 1020)	-5·86 (-17·7 to 7·79)	540 (359 to 716)
Congo	All Ages	327 (293 to 366)	281 (241 to 318)	-14·2 (-17·7 to -10·4)	262 (234 to 293)	3180 (2250 to 4480)	2800 (2000 to 4180)	-12·0 (-23·2 to 4·06)	2070 (1460 to 2920)
	Under 5	215 (172 to 262)	159 (127 to 200)	-26·1 (-35·3 to -17·0)	172 (138 to 209)	269 (178 to 362)	129 (86·7 to 182)	-52·0 (-60·7 to -38·1)	175 (115 to 235)
	5-14 years	103 (72·5 to 146)	78·4 (55·9 to 110)	-23·5 (-34·3 to -14·5)	82·1 (58·0 to 117)	81·9 (54·8 to 115)	57·2 (37·9 to 82·2)	-30·1 (-40·2 to -16·2)	53·3 (35·6 to 74·9)
	15-49 years	395 (329 to 463)	329 (273 to 400)	-16·6 (-21·7 to -9·97)	316 (264 to 371)	1620 (1070 to 2430)	1380 (961 to 2040)	-14·9 (-26·3 to -0·359)	1050 (698 to 1580)
	50-69 years	670 (545 to 812)	591 (468 to 728)	-11·8 (-17·7 to -4·23)	536 (436 to 649)	856 (584 to 1230)	914 (630 to 1400)	6·91 (-9·02 to 27·8)	557 (379 to 801)
	70+ years	721 (572 to 849)	617 (488 to 747)	-14·4 (-23·1 to -4·98)	577 (458 to 679)	348 (256 to 469)	318 (230 to 425)	-8·43 (-21·8 to 10·4)	226 (166 to 305)
Democratic Republic of the Congo	All Ages	412 (368 to 454)	335 (294 to 377)	-18·7 (-24·2 to -14·0)	329 (294 to 363)	64900 (48800 to 89800)	49600 (33100 to 73700)	-23·7 (-34·9 to -11·9)	42200 (31700 to 58400)
	Under 5	337 (269 to 409)	249 (202 to 310)	-26·0 (-32·6 to -17·5)	269 (215 to 327)	10000 (6420 to 16000)	3930 (2190 to 6570)	-61·1 (-71·2 to -46·8)	6530 (4170 to 10400)
	5-14 years	145 (100 to 200)	105 (71·0 to 145)	-27·4 (-36·7 to -18·2)	116 (80·2 to 160)	2230 (1690 to 3170)	1460 (1090 to 2270)	-34·5 (-48·1 to -20·5)	1450 (1100 to 2060)
	15-49 years	470 (386 to 544)	376 (307 to 444)	-20·0 (-26·6 to -13·4)	376 (309 to 435)	24900 (19000 to 34500)	18800 (12900 to 28000)	-24·6 (-36·7 to -10·3)	16200 (12400 to 22400)

eTable 3. Percent change from 2015 to 2021 in age-standardised all-form tuberculosis incidence rate per 100,000 population and in deaths due to all-form tuberculosis by age for 204 countries and territories.

Location	Age group	2015 Rate	2021 Rate	Incidence Percent Change	Incidence Milestone	2015 Deaths	2021 Deaths	Mortality Percent Change	Mortality Milestone
	50-69 years	1010 (818 to 1220)	885 (706 to 1130)	-12·8 (-18·2 to -5·00)	812 (654 to 979)	19700 (13500 to 27000)	18300 (11500 to 27800)	-7·18 (-23·1 to 11·5)	12800 (8790 to 17500)
	70+ years	1300 (1070 to 1500)	1040 (856 to 1250)	-19·8 (-27·9 to -12·2)	1040 (858 to 1200)	8080 (5320 to 11300)	7170 (4690 to 10500)	-10·9 (-28·1 to 6·22)	5250 (3460 to 7330)
Equatorial Guinea	All Ages	191 (165 to 219)	174 (146 to 201)	-8·84 (-14·3 to -3·76)	153 (132 to 175)	324 (203 to 527)	332 (205 to 511)	2·83 (-14·8 to 23·1)	210 (132 to 343)
	Under 5	154 (127 to 192)	126 (105 to 156)	-17·8 (-26·8 to -9·80)	123 (102 to 154)	39·5 (22·7 to 62·7)	28·2 (15·5 to 46·2)	-28·6 (-40·2 to -11·7)	25·7 (14·7 to 40·7)
	5-14 years	66·2 (45·6 to 91·2)	61·2 (41·5 to 83·8)	-7·39 (-19·6 to 4·99)	53·0 (36·5 to 72·9)	14·2 (8·32 to 21·6)	13·6 (7·89 to 24·8)	-4·89 (-24·5 to 30·9)	9·24 (5·41 to 14·0)
	15-49 years	241 (198 to 291)	211 (170 to 261)	-12·3 (-17·3 to -5·88)	193 (159 to 233)	171 (100 to 290)	182 (106 to 283)	6·57 (-11·2 to 27·8)	111 (65·2 to 188)
	50-69 years	401 (312 to 505)	378 (287 to 476)	-5·58 (-12·7 to 0·998)	321 (250 to 404)	63·1 (42·0 to 102)	74·9 (49·4 to 111)	19·3 (-2·41 to 52·4)	41·0 (27·3 to 66·2)
	70+ years	441 (346 to 555)	402 (322 to 512)	-8·83 (-18·5 to 1·48)	353 (277 to 444)	35·8 (25·3 to 52·9)	34·0 (22·6 to 49·2)	-4·85 (-22·1 to 18·9)	23·3 (16·4 to 34·4)
Gabon	All Ages	275 (243 to 311)	220 (193 to 256)	-20·1 (-24·2 to -14·8)	220 (194 to 249)	848 (543 to 1160)	714 (466 to 1050)	-15·8 (-26·4 to 0·364)	551 (353 to 756)
	Under 5	176 (142 to 216)	116 (90·0 to 144)	-34·0 (-40·4 to -27·6)	140 (113 to 173)	58·9 (39·6 to 81·4)	22·0 (13·7 to 33·2)	-62·6 (-71·5 to -52·1)	38·3 (25·7 to 52·9)
	5-14 years	91·1 (61·5 to 124)	66·0 (46·3 to 91·0)	-27·4 (-35·2 to -18·4)	72·9 (49·2 to 98·8)	21·0 (13·0 to 30·9)	13·6 (8·48 to 19·3)	-34·8 (-48·7 to -20·0)	13·6 (8·44 to 20·1)
	15-49 years	325 (266 to 390)	252 (211 to 314)	-22·4 (-27·3 to -16·3)	260 (213 to 312)	403 (247 to 580)	323 (208 to 480)	-19·5 (-31·5 to -1·92)	262 (161 to 377)
	50-69 years	537 (425 to 673)	452 (339 to 558)	-15·7 (-23·7 to -8·03)	430 (340 to 538)	253 (152 to 362)	260 (157 to 374)	3·05 (-12·6 to 25·3)	164 (98·9 to 236)
	70+ years	542 (431 to 654)	444 (328 to 557)	-18·0 (-25·5 to -7·79)	434 (345 to 523)	113 (70·1 to 150)	95·4 (61·8 to 135)	-14·8 (-28·2 to 6·00)	73·2 (45·6 to 97·4)
Eastern Sub-Saharan Africa	All Ages	330 (294 to 373)	270 (238 to 305)	-18·3 (-19·9 to -17·0)	264 (235 to 298)	269000 (224000 to 305000)	226000 (188000 to 264000)	-15·8 (-22·6 to -6·61)	175000 (145000 to 198000)
	Under 5	169 (141 to 208)	124 (103 to 153)	-26·7 (-28·8 to -24·0)	135 (113 to 166)	28200 (22100 to 33900)	16300 (11900 to 21600)	-42·1 (-49·3 to -29·5)	18400 (14400 to 22000)
	5-14 years	90·9 (64·5 to 128)	66·5 (45·1 to 94·6)	-26·9 (-30·2 to -24·4)	72·7 (51·6 to 102)	8030 (6600 to 9360)	5120 (4190 to 6190)	-36·3 (-42·6 to -30·1)	5220 (4290 to 6080)
	15-49 years	432 (360 to 529)	343 (285 to 422)	-20·7 (-22·4 to -19·1)	346 (288 to 423)	126000 (104000 to 147000)	105000 (83600 to 124000)	-16·8 (-23·2 to -8·91)	81800 (67300 to 95700)
	50-69 years	795 (632 to 988)	656 (521 to 818)	-17·5 (-19·6 to -15·6)	636 (506 to 790)	67800 (55900 to 77200)	63200 (50400 to 75000)	-6·74 (-14·9 to 4·73)	44000 (36400 to 50200)
	70+ years	1200 (993 to 1420)	1020 (850 to 1210)	-14·8 (-17·5 to -12·4)	959 (794 to 1140)	38900 (32100 to 44000)	36900 (30100 to 43500)	-5·13 (-13·4 to 8·19)	25300 (20800 to 28600)
Burundi	All Ages	370 (322 to 419)	276 (242 to 318)	-25·4 (-29·5 to -21·2)	296 (258 to 335)	8640 (7200 to 10700)	7760 (5740 to 9920)	-10·3 (-23·8 to 3·22)	5620 (4680 to 6950)
	Under 5	205 (170 to 256)	138 (112 to 172)	-32·4 (-41·5 to -25·4)	164 (136 to 205)	1110 (766 to 1470)	530 (295 to 837)	-52·4 (-65·5 to -36·2)	720 (498 to 959)

eTable 3. Percent change from 2015 to 2021 in age-standardised all-form tuberculosis incidence rate per 100,000 population and in deaths due to all-form tuberculosis by age for 204 countries and territories.

Location	Age group	2015 Rate	2021 Rate	Incidence Percent Change	Incidence Milestone	2015 Deaths	2021 Deaths	Mortality Percent Change	Mortality Milestone
	5-14 years	103 (71·9 to 144)	72·5 (49·5 to 104)	-29·6 (-38·2 to -18·8)	82·7 (57·5 to 115)	234 (185 to 285)	165 (125 to 215)	-29·3 (-43·8 to -16·1)	152 (120 to 185)
	15-49 years	469 (382 to 592)	339 (281 to 426)	-27·7 (-32·9 to -22·3)	375 (306 to 474)	3450 (2870 to 4400)	3020 (2270 to 3900)	-12·5 (-26·7 to 4·05)	2240 (1870 to 2860)
	50-69 years	975 (758 to 1220)	786 (617 to 998)	-19·3 (-26·4 to -11·4)	780 (606 to 978)	2480 (1930 to 3050)	2570 (1760 to 3470)	3·51 (-13·4 to 21·9)	1610 (1250 to 1980)
	70+ years	1520 (1230 to 1800)	1270 (1000 to 1550)	-16·3 (-25·2 to -7·49)	1210 (980 to 1440)	1370 (1050 to 1720)	1480 (975 to 2020)	8·18 (-8·58 to 24·7)	888 (684 to 1120)
Comoros	All Ages	237 (210 to 273)	215 (188 to 255)	-9·52 (-15·2 to -3·69)	190 (168 to 218)	327 (238 to 420)	309 (222 to 427)	-5·54 (-17·8 to 10·6)	213 (155 to 273)
	Under 5	95·7 (74·4 to 122)	78·8 (59·6 to 98·2)	-17·4 (-28·8 to -4·17)	76·5 (59·5 to 97·4)	19·2 (12·3 to 27·8)	11·6 (6·36 to 17·7)	-38·5 (-52·7 to -22·9)	12·5 (7·97 to 18·1)
	5-14 years	33·4 (23·0 to 49·5)	27·6 (19·8 to 41·4)	-17·5 (-28·0 to -6·57)	26·7 (18·4 to 39·6)	3·32 (2·23 to 4·84)	2·37 (1·60 to 3·46)	-27·9 (-44·0 to -4·65)	2·16 (1·45 to 3·15)
	15-49 years	249 (208 to 307)	211 (174 to 273)	-15·3 (-22·6 to -8·56)	199 (166 to 246)	96·9 (68·7 to 132)	85·6 (58·7 to 128)	-11·6 (-25·9 to 3·70)	63·0 (44·6 to 86·1)
	50-69 years	598 (472 to 739)	527 (402 to 678)	-11·9 (-22·0 to -0·980)	478 (377 to 591)	106 (74·4 to 143)	107 (75·2 to 154)	1·95 (-16·8 to 24·3)	68·7 (48·3 to 92·7)
	70+ years	955 (762 to 1190)	843 (629 to 1050)	-11·7 (-20·9 to -0·284)	764 (609 to 952)	102 (71·7 to 130)	102 (71·3 to 141)	-0·107 (-14·0 to 20·8)	66·5 (46·6 to 84·4)
Djibouti	All Ages	276 (238 to 320)	220 (189 to 257)	-20·2 (-25·2 to -13·6)	221 (191 to 256)	567 (307 to 778)	507 (277 to 737)	-10·7 (-26·0 to 4·21)	369 (199 to 506)
	Under 5	115 (89·3 to 145)	82·5 (67·4 to 103)	-27·8 (-36·8 to -17·2)	91·7 (71·4 to 116)	43·1 (27·4 to 61·6)	26·0 (16·5 to 38·8)	-39·2 (-53·3 to -21·8)	28·0 (17·8 to 40·0)
	5-14 years	52·4 (35·0 to 74·9)	40·7 (27·9 to 57·9)	-22·1 (-31·1 to -8·76)	41·9 (28·0 to 59·9)	6·52 (3·88 to 9·29)	5·61 (3·25 to 8·78)	-14·2 (-29·9 to 3·45)	4·24 (2·52 to 6·04)
	15-49 years	316 (257 to 396)	241 (199 to 305)	-23·7 (-30·6 to -16·8)	252 (205 to 317)	258 (134 to 376)	208 (115 to 310)	-19·4 (-32·3 to -6·30)	167 (87·2 to 244)
	50-69 years	675 (517 to 855)	538 (404 to 690)	-20·3 (-27·2 to -9·75)	540 (414 to 684)	179 (88·3 to 265)	178 (94·6 to 263)	0·104 (-21·1 to 22·1)	117 (57·4 to 172)
	70+ years	949 (742 to 1170)	763 (584 to 974)	-19·5 (-28·9 to -10·2)	759 (594 to 940)	80·9 (41·9 to 111)	89·0 (49·5 to 121)	10·3 (-12·4 to 35·2)	52·6 (27·3 to 72·1)
Eritrea	All Ages	472 (413 to 530)	415 (357 to 477)	-12·2 (-17·7 to -6·89)	378 (330 to 424)	5160 (3370 to 7310)	4830 (3010 to 7520)	-6·25 (-18·3 to 8·71)	3350 (2190 to 4750)
	Under 5	271 (217 to 333)	227 (180 to 277)	-16·1 (-26·0 to -5·06)	217 (174 to 266)	397 (245 to 626)	301 (187 to 468)	-23·7 (-39·3 to -0·670)	258 (159 to 407)
	5-14 years	93·1 (62·7 to 133)	76·1 (50·8 to 109)	-18·2 (-28·2 to -9·40)	74·5 (50·2 to 106)	78·5 (42·9 to 119)	58·9 (35·4 to 104)	-25·1 (-35·7 to -12·0)	51·0 (27·9 to 77·1)
	15-49 years	569 (466 to 685)	486 (391 to 606)	-14·6 (-21·9 to -7·41)	455 (373 to 548)	2240 (1390 to 3350)	2020 (1190 to 3430)	-9·78 (-25·0 to 5·46)	1460 (907 to 2180)
	50-69 years	1230 (975 to 1510)	1080 (844 to 1370)	-12·3 (-19·9 to -4·42)	988 (780 to 1210)	1670 (1100 to 2370)	1700 (1080 to 2480)	1·59 (-13·7 to 19·0)	1090 (713 to 1540)
	70+ years	1700 (1330 to 2060)	1500 (1160 to 1860)	-12·1 (-19·4 to -0·432)	1360 (1060 to 1650)	768 (474 to 1130)	755 (460 to 1120)	-1·33 (-16·7 to 17·6)	499 (308 to 737)

eTable 3. Percent change from 2015 to 2021 in age-standardised all-form tuberculosis incidence rate per 100,000 population and in deaths due to all-form tuberculosis by age for 204 countries and territories.

Location	Age group	2015 Rate	2021 Rate	Incidence Percent Change	Incidence Milestone	2015 Deaths	2021 Deaths	Mortality Percent Change	Mortality Milestone
Ethiopia	All Ages	232 (205 to 258)	205 (184 to 229)	-11·5 (-14·3 to -8·56)	186 (164 to 206)	40000 (35800 to 44000)	34000 (28900 to 40300)	-15·0 (-25·3 to 1·39)	26000 (23300 to 28600)
	Under 5	126 (101 to 158)	101 (80·3 to 126)	-20·0 (-24·5 to -14·3)	101 (80·4 to 126)	3780 (2920 to 4740)	2420 (1780 to 3110)	-35·8 (-49·3 to -20·7)	2460 (1900 to 3080)
	5-14 years	60·2 (40·9 to 85·0)	48·1 (32·2 to 68·4)	-20·1 (-24·9 to -15·3)	48·2 (32·7 to 68·0)	908 (706 to 1100)	566 (432 to 724)	-37·5 (-48·3 to -21·2)	590 (459 to 718)
	15-49 years	279 (228 to 328)	238 (194 to 282)	-14·8 (-17·7 to -11·5)	223 (183 to 262)	14600 (12900 to 16600)	12100 (9890 to 14400)	-17·2 (-29·2 to -2·01)	9480 (8380 to 10800)
	50-69 years	645 (522 to 804)	576 (470 to 706)	-10·7 (-14·8 to -7·62)	516 (418 to 643)	10500 (9080 to 11800)	9590 (7940 to 11800)	-8·04 (-23·9 to 13·5)	6800 (5900 to 7660)
	70+ years	1090 (899 to 1270)	993 (825 to 1160)	-8·49 (-13·5 to -3·07)	868 (719 to 1010)	10300 (9070 to 11600)	9340 (7950 to 11200)	-9·24 (-23·3 to 9·99)	6700 (5900 to 7550)
Kenya	All Ages	321 (281 to 365)	229 (199 to 260)	-28·6 (-29·8 to -27·4)	257 (225 to 292)	43100 (27500 to 52700)	33100 (20000 to 43700)	-23·1 (-33·7 to -9·95)	28000 (17900 to 34200)
	Under 5	115 (93·5 to 142)	67·9 (55·7 to 84·4)	-40·7 (-42·0 to -39·4)	91·6 (74·8 to 113)	2380 (1440 to 3080)	1180 (717 to 1550)	-50·4 (-56·1 to -44·1)	1550 (939 to 2000)
	5-14 years	87·6 (59·4 to 123)	52·1 (34·8 to 73·6)	-40·5 (-41·8 to -38·7)	70·1 (47·5 to 98·5)	1050 (658 to 1340)	542 (304 to 679)	-48·6 (-54·8 to -41·9)	685 (428 to 870)
	15-49 years	413 (344 to 501)	278 (229 to 341)	-32·7 (-33·9 to -31·5)	330 (275 to 401)	21000 (13300 to 25800)	14400 (8760 to 19000)	-31·5 (-40·3 to -20·6)	13700 (8620 to 16700)
	50-69 years	735 (582 to 915)	515 (409 to 640)	-29·9 (-31·4 to -28·5)	588 (466 to 732)	12400 (7810 to 15100)	10800 (6580 to 13500)	-12·3 (-26·8 to 6·66)	8030 (5080 to 9790)
	70+ years	1270 (1030 to 1520)	993 (803 to 1200)	-21·8 (-23·9 to -20·0)	1020 (827 to 1210)	6270 (3780 to 8590)	6180 (3750 to 8730)	-1·18 (-19·2 to 22·3)	4080 (2460 to 5580)
Madagascar	All Ages	255 (225 to 290)	211 (182 to 241)	-17·2 (-21·8 to -12·7)	204 (180 to 232)	11300 (8470 to 14800)	10600 (7660 to 14600)	-5·76 (-21·8 to 11·9)	7330 (5510 to 9600)
	Under 5	143 (114 to 176)	107 (85·4 to 134)	-24·7 (-33·0 to -13·5)	114 (90·9 to 141)	1370 (887 to 1930)	785 (457 to 1240)	-42·7 (-58·1 to -22·5)	892 (576 to 1250)
	5-14 years	51·3 (33·9 to 72·5)	39·4 (26·4 to 56·4)	-23·1 (-32·8 to -10·4)	41·1 (27·1 to 58·0)	212 (159 to 292)	164 (113 to 238)	-22·5 (-42·0 to -0·0775)	138 (103 to 190)
	15-49 years	321 (269 to 407)	256 (212 to 333)	-20·3 (-25·4 to -13·9)	257 (215 to 325)	4350 (3230 to 5700)	4220 (3060 to 5880)	-2·96 (-19·0 to 17·8)	2830 (2100 to 3700)
	50-69 years	698 (546 to 873)	580 (449 to 731)	-16·9 (-24·2 to -8·58)	559 (436 to 699)	3590 (2550 to 4950)	3720 (2600 to 5350)	4·04 (-18·8 to 22·6)	2330 (1660 to 3220)
	70+ years	1010 (793 to 1210)	829 (628 to 1040)	-17·8 (-28·1 to -8·09)	807 (634 to 968)	1750 (1300 to 2420)	1740 (1280 to 2430)	-0·831 (-18·3 to 24·6)	1140 (845 to 1580)
Malawi	All Ages	411 (355 to 466)	352 (304 to 409)	-14·4 (-19·7 to -9·32)	329 (284 to 373)	15200 (11700 to 18400)	14300 (10600 to 18000)	-5·65 (-16·5 to 5·90)	9880 (7590 to 12000)
	Under 5	252 (210 to 308)	171 (138 to 221)	-32·1 (-41·4 to -22·0)	202 (168 to 246)	1870 (1440 to 2420)	654 (455 to 969)	-65·1 (-73·5 to -55·9)	1210 (934 to 1570)
	5-14 years	154 (112 to 211)	120 (84·0 to 166)	-22·0 (-32·0 to -11·1)	123 (89·7 to 169)	796 (643 to 1010)	506 (354 to 726)	-36·6 (-53·2 to -18·9)	517 (418 to 659)
	15-49 years	536 (432 to 658)	448 (367 to 561)	-16·3 (-22·3 to -10·2)	429 (346 to 527)	7590 (5980 to 9590)	7690 (5810 to 9720)	1·57 (-12·5 to 14·3)	4930 (3890 to 6230)

eTable 3. Percent change from 2015 to 2021 in age-standardised all-form tuberculosis incidence rate per 100,000 population and in deaths due to all-form tuberculosis by age for 204 countries and territories.

Location	Age group	2015 Rate	2021 Rate	Incidence Percent Change	Incidence Milestone	2015 Deaths	2021 Deaths	Mortality Percent Change	Mortality Milestone
	50-69 years	894 (687 to 1110)	793 (607 to 1020)	-11·1 (-20·7 to -2·76)	715 (550 to 884)	3340 (2370 to 4370)	3840 (2770 to 5180)	14·9 (-0·528 to 34·9)	2170 (1540 to 2840)
	70+ years	1210 (962 to 1500)	1070 (836 to 1370)	-10·8 (-17·9 to -2·36)	964 (770 to 1200)	1610 (1100 to 2120)	1660 (1180 to 2310)	3·56 (-12·4 to 21·9)	1050 (715 to 1380)
Mozambique	All Ages	618 (526 to 705)	525 (454 to 608)	-15·1 (-19·7 to -10·2)	495 (420 to 564)	44900 (38700 to 51400)	35800 (28400 to 42500)	-20·3 (-30·6 to -10·8)	29200 (25200 to 33400)
	Under 5	285 (227 to 366)	207 (169 to 251)	-27·1 (-33·9 to -15·8)	228 (182 to 292)	4940 (4050 to 6010)	2780 (2020 to 3630)	-43·6 (-54·8 to -27·9)	3210 (2630 to 3900)
	5-14 years	165 (115 to 238)	137 (91·3 to 195)	-17·1 (-26·2 to -6·43)	132 (92·4 to 191)	1650 (1390 to 1920)	1460 (1150 to 1790)	-11·1 (-24·3 to 3·34)	1070 (901 to 1250)
	15-49 years	899 (749 to 1100)	758 (624 to 956)	-15·6 (-20·5 to -9·57)	719 (599 to 876)	24500 (21600 to 27600)	20700 (16500 to 24100)	-15·5 (-25·8 to -6·09)	15900 (14000 to 18000)
	50-69 years	1350 (1070 to 1650)	1170 (910 to 1440)	-13·8 (-20·7 to -5·82)	1080 (855 to 1320)	9690 (7530 to 12000)	7300 (5410 to 9420)	-24·6 (-37·3 to -9·43)	6300 (4900 to 7780)
	70+ years	1930 (1520 to 2270)	1660 (1340 to 2010)	-14·3 (-23·2 to -4·84)	1550 (1220 to 1810)	4130 (3140 to 5100)	3540 (2440 to 4350)	-14·2 (-32·2 to 2·82)	2690 (2040 to 3320)
Rwanda	All Ages	228 (196 to 265)	194 (166 to 229)	-15·2 (-19·9 to -9·87)	183 (157 to 212)	5510 (4290 to 6920)	5130 (3850 to 6920)	-7·14 (-18·4 to 8·93)	3580 (2790 to 4500)
	Under 5	93·4 (72·5 to 115)	66·6 (52·4 to 83·3)	-28·5 (-36·3 to -19·1)	74·7 (58·0 to 91·8)	368 (242 to 510)	203 (143 to 287)	-44·5 (-55·7 to -30·1)	239 (157 to 332)
	5-14 years	60·0 (42·2 to 83·4)	37·1 (24·9 to 54·2)	-38·0 (-45·1 to -29·6)	48·0 (33·7 to 66·8)	158 (118 to 203)	55·4 (38·2 to 75·8)	-64·9 (-73·2 to -56·2)	103 (76·9 to 132)
	15-49 years	286 (236 to 370)	235 (189 to 303)	-17·9 (-23·8 to -11·9)	229 (189 to 296)	2500 (1970 to 3180)	2220 (1690 to 3020)	-11·2 (-21·4 to 1·80)	1620 (1280 to 2070)
	50-69 years	526 (405 to 667)	463 (349 to 575)	-12·1 (-18·7 to -3·84)	421 (324 to 534)	1620 (1220 to 2130)	1720 (1210 to 2480)	5·49 (-14·2 to 33·4)	1060 (790 to 1380)
	70+ years	780 (624 to 959)	676 (536 to 845)	-13·1 (-22·2 to -5·26)	624 (499 to 767)	868 (646 to 1110)	938 (670 to 1310)	8·33 (-16·2 to 37·0)	564 (420 to 722)
Somalia	All Ages	436 (382 to 500)	368 (318 to 425)	-15·5 (-19·6 to -9·86)	348 (305 to 400)	18200 (11600 to 25800)	18800 (11700 to 26400)	3·54 (-7·81 to 16·4)	11800 (7560 to 16700)
	Under 5	230 (179 to 281)	178 (136 to 227)	-22·6 (-31·6 to -13·9)	184 (143 to 225)	2920 (1880 to 4540)	2330 (1410 to 3640)	-20·2 (-37·2 to 3·12)	1900 (1220 to 2950)
	5-14 years	101 (70·8 to 147)	81·5 (54·9 to 115)	-19·5 (-28·3 to -7·98)	81·2 (56·7 to 117)	380 (209 to 629)	332 (175 to 557)	-12·5 (-28·2 to 10·1)	247 (136 to 409)
	15-49 years	572 (459 to 725)	487 (399 to 608)	-14·8 (-20·8 to -7·32)	457 (367 to 580)	6880 (4090 to 10000)	8050 (4610 to 11600)	17·2 (-2·05 to 39·7)	4470 (2660 to 6530)
	50-69 years	1490 (1160 to 1930)	1360 (1030 to 1760)	-8·88 (-16·9 to -1·43)	1200 (925 to 1540)	5940 (3700 to 8550)	5720 (3630 to 8280)	-3·49 (-17·6 to 10·1)	3860 (2400 to 5560)
	70+ years	1960 (1490 to 2490)	1760 (1340 to 2310)	-10·3 (-19·1 to -2·43)	1570 (1190 to 2000)	2040 (1120 to 3110)	2390 (1340 to 3800)	17·6 (1·19 to 35·2)	1330 (728 to 2020)
South Sudan	All Ages	349 (306 to 399)	339 (293 to 385)	-2·58 (-7·45 to 3·52)	279 (245 to 319)	6840 (5040 to 9270)	6400 (4360 to 8980)	-6·31 (-19·5 to 8·30)	4440 (3280 to 6020)
	Under 5	197 (160 to 243)	184 (146 to 228)	-6·60 (-15·7 to 4·38)	158 (128 to 194)	1360 (944 to 1870)	997 (691 to 1500)	-26·6 (-43·5 to -5·37)	887 (614 to 1210)

eTable 3. Percent change from 2015 to 2021 in age-standardised all-form tuberculosis incidence rate per 100,000 population and in deaths due to all-form tuberculosis by age for 204 countries and territories.

Location	Age group	2015 Rate	2021 Rate	Incidence Percent Change	Incidence Milestone	2015 Deaths	2021 Deaths	Mortality Percent Change	Mortality Milestone
	5-14 years	84·1 (56·5 to 117)	82·3 (53·6 to 117)	-2·09 (-13·2 to 12·8)	67·3 (45·2 to 93·3)	147 (100 to 202)	122 (79·2 to 176)	-16·7 (-32·8 to 4·38)	95·7 (65·3 to 131)
	15-49 years	462 (384 to 570)	429 (353 to 533)	-7·12 (-14·1 to 1·32)	370 (307 to 456)	2700 (1870 to 3750)	2360 (1600 to 3380)	-12·1 (-27·0 to 4·51)	1750 (1220 to 2440)
	50-69 years	922 (720 to 1170)	879 (666 to 1100)	-4·71 (-12·1 to 3·08)	738 (576 to 935)	1640 (1120 to 2330)	1980 (1280 to 2840)	21·1 (-1·60 to 45·8)	1070 (731 to 1520)
	70+ years	1320 (1050 to 1600)	1200 (969 to 1490)	-8·77 (-17·0 to 7·80)	1060 (844 to 1280)	987 (718 to 1410)	942 (655 to 1420)	-4·50 (-20·4 to 18·1)	642 (466 to 917)
Uganda	All Ages	358 (321 to 401)	285 (246 to 321)	-20·3 (-26·0 to -13·4)	286 (256 to 321)	26500 (22300 to 30900)	22100 (18200 to 27700)	-16·5 (-28·0 to -2·99)	17200 (14500 to 20100)
	Under 5	182 (144 to 228)	133 (107 to 166)	-26·6 (-35·7 to -15·5)	145 (115 to 182)	2640 (1750 to 3660)	1790 (1170 to 2730)	-32·3 (-46·0 to -16·6)	1720 (1130 to 2380)
	5-14 years	107 (80·3 to 143)	67·2 (45·7 to 94·2)	-37·4 (-47·4 to -23·7)	85·7 (64·2 to 114)	1030 (778 to 1290)	429 (303 to 587)	-58·4 (-66·0 to -46·9)	671 (506 to 836)
	15-49 years	520 (444 to 599)	406 (321 to 482)	-21·9 (-29·5 to -12·5)	416 (355 to 479)	14100 (11600 to 17000)	11700 (9330 to 15300)	-16·9 (-30·7 to -4·20)	9160 (7530 to 11000)
	50-69 years	766 (650 to 895)	633 (522 to 768)	-17·3 (-25·0 to -9·02)	613 (520 to 716)	5600 (4210 to 6810)	5400 (3820 to 7150)	-3·65 (-19·7 to 15·2)	3640 (2740 to 4430)
	70+ years	1170 (1050 to 1280)	1020 (891 to 1140)	-12·4 (-23·0 to -3·93)	935 (839 to 1020)	3130 (2520 to 3940)	2830 (2130 to 3700)	-9·48 (-22·4 to 10·6)	2030 (1640 to 2560)
United Republic of Tanzania	All Ages	282 (245 to 332)	195 (171 to 224)	-30·9 (-35·4 to -27·4)	226 (196 to 265)	30600 (20900 to 39300)	23700 (16700 to 32300)	-22·5 (-34·0 to -10·7)	19900 (13600 to 25600)
	Under 5	147 (118 to 184)	96·7 (77·9 to 123)	-34·3 (-41·5 to -25·2)	118 (94·8 to 148)	3580 (2490 to 4600)	1860 (1250 to 2520)	-47·9 (-57·5 to -37·2)	2330 (1620 to 2990)
	5-14 years	78·1 (55·1 to 115)	47·3 (31·9 to 69·7)	-39·3 (-46·8 to -31·4)	62·4 (44·0 to 91·6)	891 (547 to 1290)	461 (300 to 673)	-48·1 (-56·5 to -40·2)	579 (355 to 840)
	15-49 years	369 (301 to 477)	243 (202 to 304)	-33·9 (-39·4 to -28·8)	295 (241 to 382)	14700 (9320 to 20000)	10800 (7210 to 15800)	-26·3 (-37·2 to -15·3)	9560 (6060 to 13000)
	50-69 years	606 (460 to 761)	444 (340 to 562)	-26·6 (-32·6 to -20·4)	485 (368 to 609)	6950 (4600 to 9200)	6490 (4450 to 9230)	-6·49 (-24·2 to 13·4)	4520 (2990 to 5980)
	70+ years	1000 (790 to 1250)	761 (612 to 942)	-24·0 (-32·5 to -15·4)	802 (632 to 999)	4480 (3170 to 5760)	4070 (2830 to 5380)	-8·78 (-27·1 to 10·9)	2910 (2060 to 3750)
Zambia	All Ages	465 (394 to 536)	382 (326 to 446)	-17·7 (-23·3 to -12·6)	372 (315 to 429)	11800 (7990 to 16000)	8680 (5580 to 12900)	-27·2 (-37·5 to -13·3)	7690 (5190 to 10400)
	Under 5	214 (170 to 265)	139 (111 to 179)	-35·1 (-44·0 to -25·9)	171 (136 to 212)	1430 (1010 to 1980)	471 (280 to 712)	-67·2 (-75·0 to -58·0)	929 (654 to 1280)
	5-14 years	153 (103 to 217)	106 (72·6 to 153)	-30·1 (-38·3 to -18·5)	122 (82·5 to 173)	471 (299 to 623)	240 (142 to 376)	-49·5 (-59·7 to -29·2)	306 (194 to 405)
	15-49 years	659 (541 to 837)	540 (437 to 686)	-18·1 (-24·9 to -11·5)	527 (433 to 670)	6840 (4460 to 9500)	5130 (3060 to 7930)	-25·6 (-36·1 to -10·4)	4450 (2900 to 6170)
	50-69 years	909 (700 to 1140)	760 (581 to 978)	-16·4 (-25·1 to -7·78)	727 (560 to 913)	2070 (1370 to 2890)	2000 (1210 to 2900)	-4·01 (-20·2 to 15·4)	1350 (893 to 1880)
	70+ years	1230 (957 to 1510)	1010 (776 to 1270)	-17·5 (-24·0 to -8·18)	981 (766 to 1210)	1020 (660 to 1370)	837 (523 to 1200)	-18·0 (-33·1 to 0·953)	663 (429 to 892)

eTable 3. Percent change from 2015 to 2021 in age-standardised all-form tuberculosis incidence rate per 100,000 population and in deaths due to all-form tuberculosis by age for 204 countries and territories.

Location	Age group	2015 Rate	2021 Rate	Incidence Percent Change	Incidence Milestone	2015 Deaths	2021 Deaths	Mortality Percent Change	Mortality Milestone
Southern Sub-Saharan Africa	All Ages	969 (853 to 1100)	857 (752 to 967)	-11·6 (-13·7 to -9·46)	775 (682 to 877)	117000 (107000 to 125000)	95600 (84500 to 105000)	-18·4 (-22·5 to -13·0)	76100 (69400 to 81500)
	Under 5	301 (248 to 378)	225 (185 to 281)	-25·4 (-28·7 to -22·1)	241 (198 to 302)	4240 (3670 to 4880)	2730 (2270 to 3420)	-35·7 (-43·4 to -24·7)	2760 (2390 to 3170)
	5-14 years	269 (192 to 362)	182 (127 to 246)	-32·5 (-35·7 to -29·0)	215 (153 to 289)	3420 (3070 to 3690)	1460 (1240 to 1690)	-57·3 (-62·4 to -52·9)	2230 (1990 to 2400)
	15-49 years	1360 (1150 to 1590)	1220 (1030 to 1410)	-10·4 (-12·8 to -7·47)	1090 (919 to 1270)	71100 (63400 to 77800)	57000 (48700 to 62400)	-19·9 (-24·3 to -15·3)	46200 (41200 to 50600)
	50-69 years	1020 (837 to 1210)	891 (733 to 1050)	-12·6 (-15·1 to -10·5)	816 (670 to 967)	29900 (27200 to 32400)	26900 (24300 to 30300)	-10·2 (-14·7 to -4·77)	19400 (17700 to 21100)
	70+ years	886 (747 to 1000)	778 (667 to 885)	-12·2 (-14·7 to -8·83)	709 (598 to 802)	8410 (7590 to 8960)	7530 (6750 to 8540)	-10·4 (-15·7 to -0·569)	5460 (4930 to 5820)
Botswana	All Ages	758 (642 to 908)	610 (520 to 716)	-19·5 (-24·7 to -12·8)	606 (513 to 727)	3220 (2340 to 3870)	2660 (1850 to 3360)	-17·5 (-28·7 to -5·15)	2090 (1520 to 2510)
	Under 5	226 (183 to 285)	181 (145 to 228)	-19·7 (-27·9 to -10·5)	181 (146 to 228)	71·7 (46·2 to 104)	52·0 (29·2 to 78·1)	-27·2 (-44·9 to -4·29)	46·6 (30·1 to 67·5)
	5-14 years	181 (127 to 259)	111 (78·0 to 156)	-38·5 (-45·5 to -29·7)	145 (102 to 208)	46·2 (33·6 to 56·2)	17·5 (12·5 to 23·9)	-62·1 (-69·1 to -54·4)	30·0 (21·8 to 36·5)
	15-49 years	1030 (842 to 1320)	810 (663 to 997)	-21·6 (-27·5 to -13·8)	827 (674 to 1050)	2110 (1550 to 2650)	1590 (1080 to 2030)	-24·9 (-35·6 to -11·9)	1370 (1010 to 1720)
	50-69 years	967 (712 to 1260)	826 (619 to 1050)	-14·5 (-20·5 to -5·92)	774 (569 to 1010)	811 (573 to 1060)	833 (565 to 1110)	2·65 (-15·4 to 21·0)	527 (372 to 691)
	70+ years	711 (525 to 922)	612 (460 to 851)	-13·8 (-24·4 to -1·63)	569 (420 to 738)	181 (134 to 231)	172 (125 to 217)	-4·77 (-20·6 to 12·0)	118 (86·9 to 150)
Eswatini	All Ages	1040 (879 to 1230)	806 (688 to 965)	-22·5 (-27·3 to -15·9)	831 (703 to 983)	2960 (2540 to 3490)	1960 (1610 to 2330)	-33·5 (-43·2 to -24·3)	1920 (1650 to 2270)
	Under 5	373 (299 to 471)	256 (203 to 323)	-31·2 (-41·2 to -23·7)	298 (239 to 377)	162 (123 to 200)	60·2 (46·4 to 82·5)	-62·7 (-71·3 to -53·4)	105 (80·2 to 130)
	5-14 years	342 (235 to 467)	250 (169 to 352)	-27·0 (-34·4 to -18·7)	274 (188 to 373)	109 (89·0 to 136)	47·6 (33·5 to 61·0)	-55·8 (-72·6 to -40·0)	70·8 (57·9 to 88·6)
	15-49 years	1500 (1230 to 1900)	1130 (933 to 1440)	-24·2 (-30·1 to -17·4)	1200 (983 to 1520)	1980 (1700 to 2480)	1310 (1080 to 1570)	-33·6 (-44·6 to -22·2)	1290 (1100 to 1610)
	50-69 years	1330 (999 to 1670)	1050 (795 to 1340)	-20·8 (-28·5 to -12·8)	1060 (799 to 1330)	592 (459 to 756)	452 (310 to 633)	-23·8 (-37·8 to -6·45)	385 (298 to 492)
	70+ years	1000 (692 to 1490)	806 (576 to 1180)	-19·2 (-30·8 to -4·90)	804 (554 to 1190)	112 (83·7 to 142)	93·1 (66·7 to 123)	-16·8 (-33·3 to -2·07)	73·0 (54·4 to 92·0)
Lesotho	All Ages	1220 (1060 to 1400)	1050 (929 to 1210)	-13·7 (-18·1 to -8·24)	976 (852 to 1120)	7980 (6910 to 9250)	6010 (5140 to 6880)	-24·5 (-33·3 to -16·3)	5190 (4490 to 6010)
	Under 5	499 (403 to 622)	480 (397 to 589)	-3·62 (-13·7 to 5·40)	400 (322 to 497)	288 (212 to 355)	355 (269 to 443)	23·9 (6·74 to 53·4)	187 (138 to 231)
	5-14 years	392 (272 to 545)	338 (229 to 474)	-13·8 (-24·1 to -2·46)	314 (218 to 436)	208 (179 to 247)	129 (99·6 to 158)	-37·2 (-59·3 to -22·7)	135 (116 to 161)
	15-49 years	1600 (1310 to 1970)	1330 (1100 to 1660)	-16·7 (-22·2 to -10·7)	1280 (1050 to 1580)	4630 (3880 to 5630)	3370 (2870 to 3970)	-26·8 (-36·7 to -14·6)	3010 (2520 to 3660)

eTable 3. Percent change from 2015 to 2021 in age-standardised all-form tuberculosis incidence rate per 100,000 population and in deaths due to all-form tuberculosis by age for 204 countries and territories.

Location	Age group	2015 Rate	2021 Rate	Incidence Percent Change	Incidence Milestone	2015 Deaths	2021 Deaths	Mortality Percent Change	Mortality Milestone
	50-69 years	2000 (1590 to 2500)	1710 (1330 to 2130)	-14·5 (-22·7 to -5·93)	1600 (1270 to 2000)	2390 (1890 to 2800)	1760 (1330 to 2290)	-26·8 (-36·4 to -14·8)	1560 (1230 to 1820)
	70+ years	1630 (1160 to 2160)	1440 (1040 to 2030)	-11·2 (-24·3 to 4·60)	1300 (925 to 1730)	466 (345 to 580)	401 (311 to 502)	-13·7 (-26·3 to -2·84)	303 (224 to 377)
Namibia	All Ages	673 (568 to 784)	549 (474 to 635)	-18·4 (-22·7 to -12·6)	538 (454 to 627)	2730 (2060 to 3440)	2080 (1560 to 2660)	-23·7 (-34·1 to -11·9)	1770 (1340 to 2240)
	Under 5	264 (204 to 330)	194 (160 to 247)	-26·6 (-35·1 to -16·1)	212 (163 to 264)	108 (81·3 to 148)	56·9 (41·8 to 78·8)	-47·3 (-57·2 to -33·4)	70·4 (52·8 to 96·2)
	5-14 years	213 (148 to 300)	148 (106 to 207)	-30·2 (-38·4 to -21·3)	170 (118 to 240)	86·8 (66·6 to 108)	37·3 (27·7 to 52·2)	-56·9 (-65·2 to -44·2)	56·4 (43·3 to 70·3)
	15-49 years	922 (745 to 1150)	740 (630 to 924)	-19·7 (-25·8 to -12·6)	738 (596 to 921)	1590 (1130 to 2110)	1120 (827 to 1440)	-29·5 (-39·2 to -19·7)	1030 (734 to 1370)
	50-69 years	948 (719 to 1170)	808 (603 to 1010)	-14·8 (-22·0 to -6·79)	759 (575 to 934)	734 (557 to 921)	681 (511 to 881)	-7·16 (-21·3 to 8·71)	477 (362 to 599)
	70+ years	743 (597 to 921)	628 (493 to 795)	-15·4 (-23·0 to -7·77)	595 (478 to 737)	208 (165 to 283)	185 (136 to 255)	-10·9 (-26·0 to 3·85)	135 (107 to 184)
South Africa	All Ages	1050 (921 to 1190)	960 (836 to 1090)	-8·55 (-11·1 to -5·99)	840 (737 to 950)	76900 (67800 to 81700)	62800 (53300 to 68200)	-18·3 (-21·7 to -13·0)	50000 (44000 to 53100)
	Under 5	296 (241 to 364)	222 (178 to 277)	-25·0 (-27·9 to -21·6)	237 (193 to 291)	2090 (1840 to 2410)	1250 (1020 to 1550)	-40·4 (-48·5 to -30·1)	1360 (1200 to 1570)
	5-14 years	274 (197 to 370)	180 (125 to 237)	-34·5 (-37·7 to -31·4)	219 (158 to 296)	1890 (1720 to 2040)	697 (609 to 785)	-63·0 (-66·7 to -59·1)	1230 (1120 to 1320)
	15-49 years	1480 (1240 to 1720)	1380 (1150 to 1600)	-6·64 (-9·58 to -3·21)	1180 (992 to 1370)	48100 (40700 to 51600)	38800 (31500 to 42600)	-19·2 (-23·7 to -14·1)	31200 (26500 to 33600)
	50-69 years	965 (800 to 1140)	852 (712 to 1000)	-11·6 (-14·1 to -9·12)	772 (640 to 914)	19400 (18000 to 20600)	17200 (15400 to 18700)	-11·3 (-16·1 to -4·42)	12600 (11700 to 13400)
	70+ years	828 (708 to 914)	734 (642 to 811)	-11·2 (-13·8 to -8·06)	662 (566 to 732)	5510 (5130 to 5880)	4880 (4480 to 5530)	-11·5 (-17·0 to -2·74)	3580 (3330 to 3820)
Zimbabwe	All Ages	705 (604 to 819)	548 (476 to 623)	-22·2 (-27·8 to -17·0)	564 (483 to 656)	23300 (18700 to 26500)	20000 (15500 to 24400)	-14·1 (-22·5 to -5·17)	15100 (12100 to 17200)
	Under 5	300 (247 to 389)	213 (175 to 271)	-29·0 (-38·4 to -21·2)	240 (198 to 311)	1520 (1150 to 1990)	956 (686 to 1320)	-36·8 (-50·0 to -21·7)	987 (747 to 1290)
	5-14 years	253 (178 to 363)	178 (123 to 243)	-29·6 (-38·9 to -19·7)	202 (143 to 290)	1090 (949 to 1280)	535 (386 to 679)	-50·8 (-64·0 to -40·0)	708 (617 to 833)
	15-49 years	976 (791 to 1240)	739 (619 to 902)	-24·2 (-29·9 to -18·6)	781 (633 to 989)	12700 (10800 to 14800)	10800 (8730 to 13000)	-15·4 (-24·5 to -6·04)	8280 (7010 to 9590)
	50-69 years	1200 (887 to 1560)	1030 (740 to 1340)	-13·8 (-21·2 to -5·57)	957 (709 to 1250)	6010 (4340 to 7670)	5960 (4060 to 7750)	-0·930 (-10·9 to 9·66)	3910 (2820 to 4980)
	70+ years	1280 (975 to 1590)	1090 (836 to 1410)	-14·5 (-24·9 to -3·46)	1020 (780 to 1270)	1930 (1320 to 2370)	1800 (1200 to 2270)	-6·76 (-20·3 to 7·28)	1250 (856 to 1540)
Western Sub-Saharan Africa	All Ages	201 (179 to 223)	147 (131 to 164)	-26·6 (-28·6 to -24·8)	161 (143 to 179)	163000 (135000 to 195000)	132000 (102000 to 163000)	-19·1 (-29·0 to -10·2)	106000 (87400 to 127000)
	Under 5	150 (123 to 181)	103 (84·0 to 128)	-31·3 (-34·8 to -27·3)	120 (98·6 to 145)	28700 (20800 to 38000)	18600 (11700 to 27300)	-35·8 (-47·0 to -24·1)	18700 (13500 to 24700)

eTable 3. Percent change from 2015 to 2021 in age-standardised all-form tuberculosis incidence rate per 100,000 population and in deaths due to all-form tuberculosis by age for 204 countries and territories.

Location	Age group	2015 Rate	2021 Rate	Incidence Percent Change	Incidence Milestone	2015 Deaths	2021 Deaths	Mortality Percent Change	Mortality Milestone
	5-14 years	59·3 (40·4 to 82·8)	42·9 (30·2 to 60·7)	-27·7 (-31·5 to -23·4)	47·5 (32·3 to 66·2)	5210 (4040 to 6390)	3810 (2810 to 4850)	-27·0 (-35·3 to -17·9)	3390 (2630 to 4150)
	15-49 years	232 (193 to 284)	167 (139 to 207)	-28·1 (-30·1 to -26·0)	186 (155 to 228)	59000 (46100 to 76200)	46800 (35400 to 61000)	-20·7 (-29·1 to -12·7)	38400 (30000 to 49600)
	50-69 years	509 (397 to 635)	390 (296 to 488)	-23·5 (-26·3 to -19·1)	407 (318 to 508)	39800 (32800 to 48000)	36400 (28100 to 45100)	-8·76 (-21·6 to 3·84)	25900 (21300 to 31200)
	70+ years	854 (691 to 1050)	655 (528 to 815)	-23·3 (-25·9 to -20·7)	684 (553 to 843)	30600 (26000 to 34600)	26900 (22100 to 32300)	-12·3 (-21·4 to -1·38)	19900 (16900 to 22500)
Benin	All Ages	130 (114 to 144)	94·3 (81·9 to 105)	-27·3 (-31·6 to -22·8)	104 (90·9 to 115)	2660 (1990 to 3560)	2200 (1560 to 3100)	-17·5 (-30·1 to -2·53)	1730 (1290 to 2310)
	Under 5	104 (84·1 to 127)	70·7 (57·4 to 89·8)	-32·1 (-40·7 to -22·0)	83·5 (67·3 to 102)	484 (297 to 794)	267 (134 to 594)	-46·1 (-59·7 to -28·1)	314 (193 to 516)
	5-14 years	41·6 (28·9 to 59·0)	28·4 (18·5 to 39·7)	-31·6 (-39·2 to -24·0)	33·3 (23·1 to 47·2)	86·2 (57·2 to 126)	62·2 (38·6 to 100)	-28·0 (-42·6 to -15·4)	56·1 (37·2 to 81·9)
	15-49 years	147 (120 to 179)	105 (87·0 to 131)	-28·1 (-33·5 to -21·9)	117 (96·2 to 143)	946 (701 to 1260)	809 (578 to 1120)	-14·5 (-29·2 to 1·07)	615 (456 to 821)
	50-69 years	346 (272 to 433)	264 (196 to 339)	-23·8 (-30·5 to -17·0)	277 (217 to 346)	607 (447 to 772)	593 (414 to 877)	-2·37 (-21·5 to 21·6)	395 (291 to 502)
	70+ years	561 (442 to 686)	418 (331 to 514)	-25·4 (-34·2 to -16·9)	449 (354 to 549)	537 (413 to 678)	467 (356 to 621)	-12·9 (-27·6 to 7·28)	349 (269 to 441)
Burkina Faso	All Ages	194 (172 to 215)	136 (119 to 152)	-29·9 (-33·4 to -25·5)	155 (138 to 172)	7820 (6290 to 9030)	6510 (4980 to 7970)	-16·8 (-29·5 to -5·74)	5080 (4090 to 5870)
	Under 5	162 (131 to 199)	112 (89·3 to 138)	-30·9 (-39·1 to -21·2)	130 (105 to 159)	1380 (939 to 2000)	973 (612 to 1570)	-29·6 (-42·8 to -8·57)	898 (610 to 1300)
	5-14 years	50·2 (34·0 to 71·0)	34·1 (23·3 to 48·5)	-31·9 (-40·3 to -21·4)	40·2 (27·2 to 56·8)	236 (183 to 289)	171 (127 to 214)	-27·4 (-38·3 to -14·7)	153 (119 to 188)
	15-49 years	219 (179 to 267)	147 (122 to 185)	-33·1 (-38·3 to -26·3)	175 (143 to 214)	2670 (2140 to 3220)	2090 (1520 to 2650)	-21·5 (-35·9 to -8·25)	1730 (1390 to 2090)
	50-69 years	518 (412 to 636)	387 (300 to 484)	-25·2 (-32·3 to -17·6)	415 (330 to 509)	2010 (1600 to 2420)	1850 (1420 to 2330)	-8·07 (-22·1 to 7·68)	1310 (1040 to 1570)
	70+ years	839 (667 to 1060)	634 (502 to 779)	-24·4 (-32·3 to -16·7)	671 (533 to 846)	1520 (1320 to 1790)	1420 (1090 to 1800)	-6·47 (-23·6 to 9·68)	988 (855 to 1160)
Cabo Verde	All Ages	96·2 (85·2 to 110)	84·5 (74·2 to 97·0)	-12·2 (-17·0 to -7·07)	77·0 (68·2 to 87·7)	65·4 (48·6 to 101)	64·5 (44·0 to 114)	-1·88 (-18·1 to 16·0)	42·5 (31·6 to 65·8)
	Under 5	51·4 (40·8 to 64·1)	40·8 (32·4 to 52·0)	-20·5 (-28·5 to -11·3)	41·1 (32·7 to 51·3)	0·948 (0·628 to 2·01)	0·522 (0·342 to 0·935)	-44·0 (-58·8 to -30·5)	0·616 (0·408 to 1·30)
	5-14 years	23·6 (16·3 to 33·7)	18·4 (12·5 to 26·4)	-21·8 (-32·3 to -11·4)	18·9 (13·1 to 27·0)	0·638 (0·438 to 1·02)	0·429 (0·300 to 0·657)	-32·5 (-44·3 to -22·8)	0·415 (0·285 to 0·664)
	15-49 years	91·3 (76·4 to 113)	71·4 (58·3 to 88·3)	-21·7 (-25·8 to -16·6)	73·1 (61·1 to 90·6)	22·1 (14·4 to 36·5)	16·4 (10·5 to 28·7)	-25·7 (-39·2 to -10·9)	14·4 (9·33 to 23·7)
	50-69 years	197 (148 to 259)	186 (140 to 241)	-5·50 (-14·4 to 3·76)	157 (118 to 207)	16·4 (11·4 to 27·3)	22·4 (14·5 to 42·6)	36·1 (5·55 to 72·5)	10·6 (7·44 to 17·8)
	70+ years	329 (261 to 399)	294 (242 to 362)	-10·4 (-18·8 to -0·451)	263 (209 to 319)	25·4 (19·0 to 37·0)	24·8 (17·5 to 41·5)	-2·76 (-19·1 to 19·1)	16·5 (12·3 to 24·1)

eTable 3. Percent change from 2015 to 2021 in age-standardised all-form tuberculosis incidence rate per 100,000 population and in deaths due to all-form tuberculosis by age for 204 countries and territories.

Location	Age group	2015 Rate	2021 Rate	Incidence Percent Change	Incidence Milestone	2015 Deaths	2021 Deaths	Mortality Percent Change	Mortality Milestone
Cameroon	All Ages	225 (198 to 255)	144 (127 to 164)	-35·9 (-40·0 to -32·0)	180 (158 to 204)	13000 (8100 to 19300)	8420 (5060 to 12900)	-35·3 (-44·1 to -22·8)	8470 (5260 to 12500)
	Under 5	156 (125 to 195)	98·3 (79·0 to 123)	-36·9 (-45·1 to -27·4)	125 (99·7 to 156)	1370 (895 to 2030)	676 (439 to 1160)	-51·1 (-59·0 to -39·8)	892 (582 to 1320)
	5-14 years	68·0 (47·5 to 93·9)	45·5 (31·7 to 63·4)	-33·0 (-39·3 to -24·0)	54·4 (38·0 to 75·1)	365 (217 to 541)	246 (160 to 371)	-32·4 (-40·9 to -19·9)	237 (141 to 351)
	15-49 years	275 (231 to 339)	168 (140 to 202)	-38·7 (-42·4 to -34·1)	220 (185 to 271)	6770 (4000 to 10600)	4070 (2440 to 6610)	-39·7 (-48·5 to -28·7)	4400 (2600 to 6910)
	50-69 years	528 (402 to 664)	354 (265 to 461)	-32·9 (-39·0 to -23·8)	423 (321 to 531)	3080 (1800 to 4700)	2260 (1280 to 3590)	-26·6 (-38·8 to -12·4)	2000 (1170 to 3060)
	70+ years	793 (639 to 1000)	538 (422 to 671)	-32·0 (-37·8 to -24·5)	634 (512 to 802)	1440 (902 to 2060)	1170 (750 to 1730)	-18·5 (-32·9 to -5·01)	936 (586 to 1340)
Chad	All Ages	230 (203 to 254)	187 (165 to 209)	-18·8 (-23·9 to -13·1)	184 (162 to 203)	7860 (5950 to 9530)	7440 (5620 to 9670)	-5·38 (-19·7 to 6·91)	5110 (3870 to 6200)
	Under 5	194 (153 to 239)	154 (118 to 191)	-20·7 (-32·4 to -9·24)	155 (123 to 191)	1650 (1170 to 2080)	1390 (930 to 1930)	-15·7 (-37·5 to 5·17)	1070 (761 to 1350)
	5-14 years	86·7 (58·9 to 123)	70·3 (47·9 to 94·8)	-18·8 (-28·7 to -7·82)	69·4 (47·2 to 98·4)	318 (228 to 413)	291 (192 to 383)	-8·39 (-27·3 to 11·2)	207 (148 to 268)
	15-49 years	265 (220 to 322)	214 (179 to 270)	-19·1 (-25·0 to -12·5)	212 (176 to 258)	2680 (2030 to 3480)	2560 (1880 to 3540)	-4·43 (-22·2 to 9·54)	1750 (1320 to 2260)
	50-69 years	634 (504 to 807)	543 (424 to 672)	-14·3 (-22·9 to -5·82)	507 (403 to 645)	1890 (1310 to 2340)	1960 (1400 to 2600)	3·52 (-13·7 to 21·2)	1230 (852 to 1520)
	70+ years	1030 (808 to 1270)	855 (690 to 1040)	-16·7 (-24·6 to -6·88)	823 (646 to 1020)	1320 (916 to 1710)	1240 (856 to 1590)	-5·43 (-21·6 to 9·69)	858 (596 to 1110)
Côte d'Ivoire	All Ages	206 (179 to 234)	145 (128 to 165)	-29·2 (-33·3 to -25·5)	164 (143 to 187)	10100 (7250 to 13700)	6680 (4490 to 9510)	-33·9 (-43·6 to -22·3)	6570 (4710 to 8890)
	Under 5	144 (113 to 175)	98·6 (80·5 to 124)	-31·5 (-40·0 to -21·3)	115 (90·6 to 140)	1230 (873 to 1910)	544 (347 to 920)	-56·0 (-65·2 to -43·0)	798 (567 to 1240)
	5-14 years	67·1 (46·9 to 92·3)	50·0 (34·7 to 71·3)	-25·3 (-35·1 to -12·9)	53·7 (37·6 to 73·8)	342 (243 to 501)	212 (147 to 312)	-38·0 (-46·2 to -26·0)	223 (158 to 326)
	15-49 years	242 (203 to 300)	165 (137 to 200)	-31·9 (-36·5 to -27·9)	194 (162 to 240)	4730 (3270 to 6730)	2910 (1950 to 4160)	-38·4 (-48·8 to -27·6)	3080 (2120 to 4380)
	50-69 years	483 (367 to 604)	359 (273 to 460)	-25·6 (-32·0 to -17·9)	387 (294 to 483)	2520 (1720 to 3480)	1970 (1260 to 2930)	-22·3 (-33·8 to -8·95)	1640 (1110 to 2260)
	70+ years	722 (574 to 905)	540 (431 to 676)	-25·1 (-32·5 to -19·1)	577 (460 to 724)	1270 (918 to 1610)	1050 (765 to 1520)	-17·3 (-31·3 to -0·301)	828 (597 to 1050)
Gambia	All Ages	232 (203 to 272)	192 (166 to 220)	-17·2 (-25·3 to -9·71)	185 (163 to 218)	751 (581 to 1110)	753 (526 to 1140)	0·0903 (-13·7 to 17·8)	488 (378 to 723)
	Under 5	184 (144 to 227)	149 (116 to 190)	-18·3 (-35·6 to 0·858)	147 (115 to 182)	62·9 (45·4 to 98·6)	38·9 (25·4 to 61·9)	-38·2 (-50·2 to -23·8)	40·9 (29·5 to 64·1)
	5-14 years	58·1 (44·7 to 73·1)	53·1 (35·6 to 77·1)	-8·57 (-32·8 to 26·2)	46·5 (35·7 to 58·5)	17·8 (12·6 to 24·8)	15·3 (10·2 to 22·8)	-13·6 (-30·9 to 7·91)	11·5 (8·18 to 16·1)
	15-49 years	262 (207 to 327)	210 (170 to 262)	-19·5 (-27·5 to -10·3)	209 (166 to 262)	319 (226 to 481)	333 (219 to 508)	4·34 (-10·0 to 24·4)	208 (147 to 313)

eTable 3. Percent change from 2015 to 2021 in age-standardised all-form tuberculosis incidence rate per 100,000 population and in deaths due to all-form tuberculosis by age for 204 countries and territories.

Location	Age group	2015 Rate	2021 Rate	Incidence Percent Change	Incidence Milestone	2015 Deaths	2021 Deaths	Mortality Percent Change	Mortality Milestone
	50-69 years	626 (473 to 771)	519 (385 to 667)	-17·0 (-24·5 to -8·28)	501 (378 to 617)	183 (132 to 274)	206 (136 to 329)	12·3 (-7·15 to 32·1)	119 (85·7 to 178)
	70+ years	897 (695 to 1100)	757 (600 to 952)	-15·5 (-26·4 to -6·89)	718 (556 to 884)	168 (115 to 239)	160 (106 to 246)	-4·93 (-24·2 to 16·0)	110 (75·0 to 155)
Ghana	All Ages	246 (219 to 277)	186 (162 to 213)	-24·2 (-28·8 to -18·9)	196 (175 to 221)	14700 (11800 to 18500)	12100 (9210 to 16100)	-18·1 (-29·3 to -3·60)	9580 (7680 to 12000)
	Under 5	122 (99·1 to 146)	83·4 (67·0 to 104)	-31·7 (-40·5 to -22·2)	97·8 (79·3 to 117)	911 (640 to 1530)	341 (220 to 686)	-63·2 (-71·5 to -50·7)	592 (416 to 992)
	5-14 years	70·0 (49·2 to 100)	50·0 (34·2 to 69·6)	-28·4 (-37·8 to -18·3)	56·0 (39·3 to 80·3)	336 (254 to 439)	204 (151 to 288)	-39·4 (-48·1 to -31·2)	219 (165 to 285)
	15-49 years	281 (231 to 346)	206 (172 to 258)	-26·4 (-31·4 to -20·1)	224 (185 to 277)	6730 (5150 to 8520)	5320 (4030 to 7250)	-21·1 (-30·6 to -10·7)	4370 (3350 to 5540)
	50-69 years	584 (463 to 718)	465 (359 to 581)	-20·5 (-28·2 to -12·6)	467 (371 to 574)	4120 (3100 to 5450)	3760 (2780 to 5310)	-8·44 (-25·3 to 12·0)	2680 (2010 to 3540)
	70+ years	895 (709 to 1120)	720 (581 to 895)	-19·4 (-28·3 to -10·2)	716 (567 to 897)	2640 (2080 to 3310)	2450 (1740 to 3350)	-7·30 (-25·1 to 12·9)	1720 (1350 to 2150)
Guinea	All Ages	200 (177 to 223)	144 (128 to 161)	-27·8 (-32·1 to -23·3)	160 (142 to 178)	5550 (4280 to 6720)	4260 (3130 to 5740)	-23·4 (-33·3 to -10·9)	3610 (2780 to 4370)
	Under 5	148 (117 to 180)	101 (78·9 to 126)	-32·0 (-38·6 to -20·9)	119 (93·7 to 144)	732 (492 to 1080)	390 (230 to 707)	-47·3 (-60·0 to -28·5)	476 (320 to 705)
	5-14 years	53·4 (37·5 to 76·0)	38·1 (26·9 to 54·9)	-28·7 (-38·6 to -19·2)	42·8 (30·0 to 60·8)	152 (118 to 191)	119 (82·2 to 167)	-21·9 (-33·8 to -8·70)	99·0 (76·8 to 124)
	15-49 years	227 (186 to 281)	164 (137 to 206)	-27·6 (-33·0 to -22·2)	181 (149 to 225)	2060 (1540 to 2650)	1650 (1230 to 2120)	-19·6 (-31·7 to -5·00)	1340 (1000 to 1720)
	50-69 years	509 (415 to 620)	397 (307 to 491)	-22·1 (-28·8 to -15·5)	407 (332 to 496)	1400 (1040 to 1720)	1190 (846 to 1600)	-15·1 (-28·4 to 0·551)	909 (674 to 1120)
	70+ years	854 (683 to 1040)	639 (516 to 763)	-25·1 (-31·9 to -15·8)	683 (547 to 830)	1210 (918 to 1560)	909 (650 to 1270)	-24·7 (-36·8 to -6·59)	784 (597 to 1010)
Guinea-Bissau	All Ages	277 (241 to 314)	235 (208 to 269)	-15·1 (-19·4 to -10·3)	222 (193 to 251)	1410 (1060 to 1830)	1390 (1020 to 1810)	-1·02 (-12·9 to 14·5)	914 (687 to 1190)
	Under 5	194 (156 to 232)	155 (126 to 190)	-20·0 (-27·6 to -10·2)	155 (125 to 185)	143 (93·6 to 215)	132 (64·5 to 194)	-7·94 (-37·9 to 17·3)	92·8 (60·8 to 139)
	5-14 years	86·6 (60·5 to 118)	69·9 (48·9 to 99·4)	-19·0 (-29·5 to -6·56)	69·3 (48·4 to 94·1)	47·7 (32·5 to 62·7)	34·9 (22·4 to 47·9)	-26·2 (-43·9 to 14·2)	31·0 (21·1 to 40·8)
	15-49 years	336 (274 to 410)	291 (242 to 359)	-13·5 (-20·3 to -6·80)	269 (219 to 328)	697 (496 to 983)	728 (480 to 1040)	4·82 (-11·8 to 26·2)	453 (323 to 639)
	50-69 years	701 (567 to 854)	589 (456 to 733)	-16·0 (-23·1 to -6·81)	561 (454 to 683)	359 (261 to 445)	353 (256 to 437)	-1·34 (-16·6 to 16·1)	233 (170 to 289)
	70+ years	1010 (815 to 1230)	823 (655 to 1010)	-18·5 (-27·9 to -9·28)	809 (652 to 986)	159 (122 to 191)	142 (111 to 171)	-10·7 (-25·1 to 4·41)	103 (79·1 to 124)
Liberia	All Ages	146 (130 to 167)	115 (101 to 130)	-21·2 (-25·6 to -16·5)	117 (104 to 134)	1070 (716 to 1660)	907 (575 to 1580)	-16·3 (-28·3 to -2·33)	698 (465 to 1080)
	Under 5	105 (85·6 to 130)	78·2 (62·6 to 98·5)	-25·3 (-34·4 to -15·9)	83·9 (68·5 to 104)	128 (82·4 to 212)	63·8 (34·0 to 125)	-50·5 (-65·6 to -30·3)	82·9 (53·5 to 138)

eTable 3. Percent change from 2015 to 2021 in age-standardised all-form tuberculosis incidence rate per 100,000 population and in deaths due to all-form tuberculosis by age for 204 countries and territories.

Location	Age group	2015 Rate	2021 Rate	Incidence Percent Change	Incidence Milestone	2015 Deaths	2021 Deaths	Mortality Percent Change	Mortality Milestone
	5-14 years	40·3 (27·7 to 57·5)	31·6 (21·8 to 43·8)	-21·5 (-30·4 to -9·27)	32·3 (22·2 to 46·0)	28·3 (19·9 to 43·9)	23·6 (15·9 to 43·4)	-17·0 (-35·1 to 5·51)	18·4 (13·0 to 28·6)
	15-49 years	168 (138 to 209)	128 (105 to 156)	-23·3 (-29·2 to -18·0)	134 (111 to 168)	433 (282 to 692)	384 (232 to 667)	-12·0 (-25·9 to 7·13)	281 (183 to 450)
	50-69 years	393 (300 to 495)	316 (244 to 401)	-19·3 (-26·1 to -10·8)	314 (240 to 396)	260 (174 to 389)	252 (157 to 432)	-3·70 (-18·1 to 16·6)	169 (113 to 253)
	70+ years	637 (509 to 795)	495 (386 to 618)	-22·2 (-31·1 to -13·2)	510 (407 to 636)	225 (153 to 361)	183 (115 to 340)	-19·0 (-33·0 to -0·223)	146 (99·4 to 234)
Mali	All Ages	162 (143 to 180)	127 (110 to 143)	-21·7 (-26·8 to -17·0)	129 (114 to 144)	6580 (5130 to 8690)	5750 (4280 to 8090)	-12·8 (-23·9 to -1·02)	4280 (3330 to 5650)
	Under 5	121 (95·0 to 142)	95·4 (77·8 to 120)	-20·9 (-32·3 to -10·8)	96·6 (76·0 to 113)	862 (621 to 1330)	687 (443 to 1120)	-20·7 (-35·5 to -2·60)	560 (404 to 866)
	5-14 years	45·1 (30·3 to 63·9)	35·2 (24·9 to 50·7)	-21·6 (-31·0 to -10·3)	36·1 (24·3 to 51·1)	168 (123 to 238)	142 (99·4 to 201)	-15·6 (-28·5 to -4·71)	109 (79·9 to 155)
	15-49 years	180 (149 to 223)	138 (113 to 171)	-23·4 (-29·2 to -17·4)	144 (119 to 178)	2140 (1620 to 2910)	1760 (1340 to 2480)	-17·6 (-28·5 to -2·99)	1390 (1050 to 1890)
	50-69 years	483 (378 to 604)	401 (299 to 499)	-16·9 (-24·9 to -8·14)	386 (302 to 483)	1820 (1340 to 2500)	1670 (1220 to 2380)	-8·05 (-22·5 to 14·9)	1180 (870 to 1620)
	70+ years	864 (663 to 1050)	706 (562 to 872)	-18·1 (-26·3 to -8·89)	691 (531 to 843)	1600 (1150 to 2090)	1490 (1020 to 2100)	-6·90 (-21·0 to 10·8)	1040 (746 to 1360)
Mauritania	All Ages	75·4 (66·6 to 84·8)	59·2 (52·7 to 67·2)	-21·5 (-26·9 to -16·2)	60·3 (53·3 to 67·8)	492 (348 to 759)	438 (293 to 689)	-11·3 (-25·7 to 8·06)	320 (226 to 493)
	Under 5	60·2 (48·1 to 74·3)	43·9 (34·1 to 54·9)	-27·0 (-36·7 to -17·2)	48·2 (38·5 to 59·4)	47·1 (31·0 to 75·1)	25·5 (16·3 to 45·7)	-45·8 (-56·2 to -31·2)	30·6 (20·2 to 48·8)
	5-14 years	22·5 (15·6 to 31·8)	16·6 (11·4 to 23·3)	-26·2 (-34·0 to -15·6)	18·0 (12·5 to 25·4)	12·0 (8·60 to 16·2)	8·43 (5·90 to 11·8)	-29·3 (-46·0 to -9·67)	7·80 (5·59 to 10·5)
	15-49 years	69·5 (56·7 to 85·8)	53·2 (42·5 to 66·2)	-23·4 (-29·1 to -17·3)	55·6 (45·4 to 68·7)	113 (74·5 to 164)	102 (65·8 to 163)	-9·58 (-28·7 to 10·9)	73·4 (48·4 to 107)
	50-69 years	222 (170 to 278)	183 (142 to 233)	-17·4 (-26·8 to -10·3)	177 (136 to 223)	144 (96·3 to 230)	141 (87·5 to 226)	-2·26 (-22·8 to 23·8)	93·9 (62·6 to 150)
	70+ years	393 (309 to 491)	310 (247 to 382)	-21·1 (-28·8 to -12·4)	315 (247 to 392)	175 (128 to 280)	160 (105 to 257)	-8·93 (-25·1 to 13·5)	114 (83·1 to 182)
Niger	All Ages	182 (159 to 205)	149 (131 to 166)	-18·3 (-23·3 to -13·6)	146 (127 to 164)	7420 (5360 to 10600)	6890 (4750 to 10000)	-7·31 (-19·2 to 8·80)	4820 (3490 to 6910)
	Under 5	151 (120 to 185)	122 (97·9 to 152)	-18·8 (-29·6 to -5·87)	120 (96·0 to 148)	1940 (1170 to 3390)	1480 (899 to 2750)	-23·7 (-39·8 to -7·04)	1260 (762 to 2200)
	5-14 years	62·0 (42·1 to 87·1)	49·8 (33·7 to 73·1)	-19·4 (-30·6 to -5·04)	49·6 (33·7 to 69·7)	286 (210 to 395)	231 (158 to 362)	-19·0 (-35·3 to 3·60)	186 (137 to 257)
	15-49 years	207 (169 to 252)	165 (133 to 206)	-20·5 (-26·0 to -15·1)	166 (135 to 202)	2000 (1460 to 2780)	1850 (1250 to 2640)	-7·97 (-22·0 to 13·1)	1300 (952 to 1800)
	50-69 years	557 (439 to 693)	482 (364 to 604)	-13·3 (-20·3 to -4·38)	445 (351 to 554)	1900 (1320 to 2720)	1930 (1220 to 2700)	1·67 (-14·8 to 27·5)	1230 (855 to 1770)
	70+ years	897 (695 to 1110)	758 (605 to 968)	-15·4 (-22·5 to -6·97)	717 (556 to 892)	1300 (882 to 1920)	1400 (911 to 1960)	8·42 (-10·5 to 27·8)	843 (573 to 1250)

eTable 3. Percent change from 2015 to 2021 in age-standardised all-form tuberculosis incidence rate per 100,000 population and in deaths due to all-form tuberculosis by age for 204 countries and territories.

Location	Age group	2015 Rate	2021 Rate	Incidence Percent Change	Incidence Milestone	2015 Deaths	2021 Deaths	Mortality Percent Change	Mortality Milestone
Nigeria	All Ages	203 (180 to 225)	147 (130 to 163)	-27·5 (-29·5 to -25·6)	162 (144 to 180)	73200 (59500 to 88300)	60100 (42800 to 76400)	-17·9 (-32·2 to -2·49)	47600 (38700 to 57400)
	Under 5	158 (130 to 193)	102 (83·8 to 128)	-35·4 (-38·2 to -31·8)	127 (104 to 154)	16600 (11700 to 21500)	10900 (6700 to 15900)	-34·6 (-48·6 to -18·7)	10800 (7640 to 14000)
	5-14 years	59·0 (40·3 to 82·7)	41·4 (29·4 to 58·7)	-29·9 (-34·1 to -25·8)	47·2 (32·2 to 66·1)	2550 (1800 to 3250)	1860 (1260 to 2520)	-27·0 (-40·9 to -10·5)	1660 (1170 to 2110)
	15-49 years	234 (195 to 285)	169 (140 to 209)	-27·8 (-29·8 to -25·5)	187 (156 to 228)	22500 (17200 to 28400)	19100 (13200 to 25800)	-15·0 (-30·4 to 2·81)	14600 (11200 to 18500)
	50-69 years	505 (389 to 630)	378 (284 to 478)	-25·3 (-27·6 to -21·7)	404 (312 to 504)	16700 (12800 to 20700)	15600 (11000 to 19900)	-5·67 (-27·2 to 22·1)	10800 (8330 to 13500)
	70+ years	911 (726 to 1120)	687 (550 to 860)	-24·6 (-27·5 to -21·3)	729 (581 to 894)	14900 (11600 to 17500)	12600 (9890 to 15100)	-15·0 (-27·1 to -0·652)	9680 (7570 to 11400)
Sao Tome and Principe	All Ages	74·7 (66·3 to 83·5)	61·3 (54·0 to 70·3)	-17·9 (-22·9 to -12·8)	59·7 (53·0 to 66·8)	22·1 (16·7 to 32·8)	18·7 (13·2 to 30·8)	-15·8 (-28·8 to 0·0106)	14·4 (10·8 to 21·3)
	Under 5	45·9 (36·0 to 57·9)	34·5 (27·5 to 43·6)	-24·8 (-32·5 to -14·4)	36·8 (28·8 to 46·3)	0·476 (0·285 to 0·989)	0·180 (0·101 to 0·447)	-62·8 (-73·2 to -50·2)	0·309 (0·185 to 0·643)
	5-14 years	14·6 (9·83 to 20·5)	11·1 (7·64 to 15·7)	-23·8 (-32·9 to -13·9)	11·7 (7·87 to 16·4)	0·261 (0·187 to 0·392)	0·138 (0·0930 to 0·233)	-46·9 (-60·8 to -24·0)	0·170 (0·122 to 0·255)
	15-49 years	75·4 (62·2 to 93·8)	58·6 (48·5 to 71·0)	-22·2 (-29·3 to -15·8)	60·4 (49·8 to 75·1)	6·21 (4·51 to 9·32)	5·21 (3·18 to 8·95)	-16·8 (-34·8 to 6·08)	4·04 (2·93 to 6·06)
	50-69 years	225 (171 to 279)	182 (142 to 238)	-19·1 (-26·7 to -10·5)	180 (137 to 223)	7·27 (5·22 to 11·0)	7·22 (5·06 to 12·3)	-0·803 (-17·4 to 21·6)	4·72 (3·39 to 7·18)
	70+ years	382 (294 to 472)	292 (234 to 362)	-23·5 (-31·2 to -15·6)	306 (236 to 378)	7·86 (5·49 to 11·6)	5·92 (3·97 to 9·82)	-24·8 (-36·4 to -11·3)	5·11 (3·57 to 7·52)
Senegal	All Ages	140 (122 to 159)	108 (93·8 to 123)	-22·9 (-27·3 to -18·2)	112 (97·3 to 128)	3760 (3040 to 4680)	3140 (2310 to 4040)	-16·6 (-27·5 to -3·58)	2450 (1980 to 3040)
	Under 5	89·9 (70·6 to 113)	63·8 (50·8 to 80·8)	-28·8 (-38·7 to -18·8)	71·9 (56·5 to 90·7)	303 (219 to 469)	134 (89·7 to 244)	-56·0 (-65·7 to -44·1)	197 (142 to 305)
	5-14 years	36·0 (24·3 to 50·0)	27·2 (18·6 to 38·2)	-24·4 (-33·1 to -13·5)	28·8 (19·5 to 40·0)	81·1 (61·0 to 108)	59·5 (41·0 to 81·7)	-26·5 (-38·0 to -11·4)	52·7 (39·6 to 69·9)
	15-49 years	152 (126 to 190)	112 (92·7 to 141)	-26·5 (-31·5 to -20·3)	121 (101 to 152)	1300 (1000 to 1690)	1020 (758 to 1450)	-21·7 (-32·9 to -5·33)	847 (653 to 1100)
	50-69 years	366 (274 to 447)	296 (228 to 377)	-19·0 (-26·1 to -11·2)	293 (219 to 357)	1080 (819 to 1420)	989 (714 to 1310)	-8·25 (-22·6 to 7·66)	700 (532 to 925)
	70+ years	614 (496 to 769)	490 (386 to 588)	-20·2 (-29·6 to -13·4)	492 (397 to 615)	998 (781 to 1260)	940 (654 to 1300)	-5·95 (-21·8 to 12·8)	649 (508 to 820)
Sierra Leone	All Ages	268 (236 to 305)	199 (178 to 224)	-25·8 (-30·0 to -21·4)	215 (189 to 244)	3530 (2810 to 4320)	2850 (2130 to 3690)	-19·7 (-30·1 to -5·14)	2300 (1830 to 2810)
	Under 5	215 (173 to 264)	152 (123 to 193)	-28·9 (-38·1 to -16·4)	172 (138 to 211)	632 (473 to 883)	397 (271 to 609)	-37·4 (-51·2 to -17·1)	411 (308 to 574)
	5-14 years	74·2 (50·0 to 105)	53·2 (36·7 to 75·5)	-28·0 (-37·6 to -18·2)	59·4 (40·0 to 83·8)	95·2 (73·8 to 122)	68·0 (49·9 to 87·7)	-28·5 (-41·6 to -10·9)	61·9 (48·0 to 79·4)
	15-49 years	300 (253 to 374)	216 (177 to 272)	-28·1 (-34·5 to -21·9)	240 (202 to 299)	1310 (1010 to 1700)	1040 (779 to 1420)	-20·4 (-30·9 to -6·98)	852 (656 to 1110)

eTable 3. Percent change from 2015 to 2021 in age-standardised all-form tuberculosis incidence rate per 100,000 population and in deaths due to all-form tuberculosis by age for 204 countries and territories.

Location	Age group	2015 Rate	2021 Rate	Incidence Percent Change	Incidence Milestone	2015 Deaths	2021 Deaths	Mortality Percent Change	Mortality Milestone
	50-69 years	675 (525 to 832)	520 (397 to 655)	-22·9 (-29·7 to -15·3)	540 (420 to 665)	816 (580 to 1040)	746 (526 to 1020)	-8·67 (-23·6 to 11·8)	531 (377 to 678)
	70+ years	1010 (800 to 1270)	771 (596 to 975)	-23·9 (-31·2 to -16·7)	811 (640 to 1010)	681 (531 to 862)	590 (441 to 775)	-13·2 (-26·2 to 6·02)	443 (345 to 560)
Togo	All Ages	228 (202 to 260)	171 (150 to 196)	-25·2 (-29·6 to -20·3)	183 (162 to 208)	3390 (2640 to 4610)	2560 (1880 to 3850)	-24·6 (-36·4 to -12·3)	2200 (1710 to 2990)
	Under 5	145 (114 to 174)	106 (85·6 to 133)	-27·1 (-35·6 to -17·1)	116 (91·6 to 140)	271 (179 to 417)	130 (72·6 to 237)	-52·9 (-64·5 to -37·7)	176 (117 to 271)
	5-14 years	66·6 (45·4 to 94·6)	50·0 (35·1 to 69·6)	-24·9 (-35·0 to -13·7)	53·3 (36·3 to 75·7)	91·4 (66·1 to 131)	61·7 (44·9 to 87·7)	-32·3 (-46·4 to -18·8)	59·4 (43·0 to 85·3)
	15-49 years	268 (225 to 330)	188 (156 to 232)	-29·9 (-34·6 to -24·1)	215 (180 to 264)	1630 (1180 to 2310)	1080 (759 to 1600)	-33·5 (-44·8 to -21·8)	1060 (766 to 1500)
	50-69 years	563 (436 to 701)	436 (323 to 565)	-22·7 (-30·3 to -16·1)	450 (349 to 561)	947 (714 to 1370)	854 (566 to 1380)	-10·1 (-26·5 to 9·09)	616 (464 to 892)
	70+ years	765 (598 to 958)	600 (461 to 756)	-21·5 (-29·1 to -12·3)	612 (478 to 767)	455 (359 to 589)	439 (325 to 641)	-3·64 (-20·9 to 16·2)	296 (234 to 383)

eTable 4. Risk deleted deaths due to all-form tuberculosis for alcohol use, smoking, and diabetes, and all three risk factors combined by age groups for 204 countries and territories (2020) with percent change between 2015 observed deaths and 2020 all-risk deleted deaths.

Location	Age group	2015 Observed deaths	2020 Observed deaths	2020 Smoking deleted deaths	2020 Alcohol deleted deaths	2020 Diabetes deleted deaths	2020 All-risk deleted deaths	Percent Change
**Global**	**All Ages**	**1570000 (1450000 to 1700000)**	**1390000 (1280000 to 1540000)**	**1220000 (1050000 to 1390000)**	**1250000 (889000 to 1550000)**	**1240000 (1130000 to 1390000)**	**999000 (703000 to 1230000)**	**-36·5 (-54·8 to -21·5)**
	**15-49 years**	**593000 (534000 to 650000)**	**503000 (455000 to 549000)**	**457000 (396000 to 510000)**	**453000 (323000 to 566000)**	**492000 (444000 to 538000)**	**408000 (297000 to 499000)**	**-31·0 (-48·8 to -15·9)**
	**50-69 years**	**499000 (467000 to 528000)**	**459000 (421000 to 515000)**	**382000 (313000 to 456000)**	**403000 (255000 to 523000)**	**399000 (361000 to 462000)**	**297000 (185000 to 389000)**	**-40·5 (-62·6 to -22·3)**
	**70+ years**	**347000 (322000 to 367000)**	**335000 (307000 to 378000)**	**294000 (253000 to 336000)**	**303000 (207000 to 379000)**	**257000 (230000 to 309000)**	**204000 (129000 to 260000)**	**-41·0 (-62·0 to -25·4)**
Central Europe, Eastern Europe, and Central Asia	All Ages	26900 (25900 to 28600)	16400 (15600 to 17600)	11900 (9140 to 15200)	13000 (6220 to 20800)	15100 (14200 to 16500)	9040 (4520 to 14500)	-66·3 (-83·0 to -45·4)
	15-49 years	12400 (11600 to 13900)	7160 (6570 to 8070)	5220 (4090 to 6710)	5670 (2500 to 9260)	7000 (6410 to 7930)	4230 (2090 to 6690)	-66·0 (-83·2 to -45·2)
	50-69 years	11000 (10800 to 11300)	6610 (6340 to 7040)	4310 (3060 to 5900)	5000 (1750 to 8580)	5870 (5520 to 6320)	2970 (989 to 5560)	-73·1 (-91·0 to -49·4)
	70+ years	2680 (2550 to 2760)	2240 (2100 to 2330)	1950 (1680 to 2190)	1940 (1190 to 2560)	1850 (1710 to 2020)	1420 (882 to 1920)	-47·1 (-67·5 to -30·2)
Central Asia	All Ages	6430 (6060 to 6820)	4520 (4150 to 4980)	3480 (2870 to 4240)	3740 (1910 to 5210)	4160 (3770 to 4630)	2740 (1500 to 3870)	-57·3 (-76·4 to -39·3)
	15-49 years	3260 (3060 to 3470)	2160 (1980 to 2370)	1640 (1360 to 2000)	1720 (760 to 2640)	2110 (1930 to 2310)	1310 (626 to 1990)	-59·7 (-80·7 to -38·2)
	50-69 years	2100 (1980 to 2230)	1590 (1440 to 1750)	1140 (869 to 1460)	1280 (545 to 1840)	1360 (1220 to 1530)	805 (330 to 1220)	-61·6 (-84·2 to -40·7)
	70+ years	522 (479 to 560)	411 (374 to 453)	358 (307 to 414)	376 (251 to 453)	336 (301 to 380)	271 (176 to 340)	-48·1 (-65·5 to -35·5)
Armenia	All Ages	76·5 (69·5 to 83·3)	44·0 (40·6 to 48·3)	28·7 (20·8 to 38·6)	36·3 (17·1 to 52·7)	39·9 (36·5 to 44·5)	22·4 (11·7 to 34·2)	-70·7 (-84·2 to -57·2)
	15-49 years	26·0 (24·2 to 28·0)	18·2 (16·8 to 20·1)	11·8 (8·85 to 15·8)	14·4 (5·78 to 22·8)	17·8 (16·4 to 19·7)	9·67 (4·60 to 15·4)	-62·8 (-81·6 to -41·9)
	50-69 years	34·6 (31·5 to 37·7)	15·4 (13·9 to 17·1)	8·73 (5·33 to 13·0)	12·3 (4·84 to 18·6)	13·3 (11·9 to 15·1)	6·22 (2·50 to 10·6)	-82·0 (-92·4 to -70·5)
	70+ years	14·3 (12·4 to 16·1)	9·45 (8·20 to 10·7)	7·23 (5·23 to 9·02)	8·61 (5·40 to 10·7)	7·86 (6·70 to 9·17)	5·63 (3·41 to 7·65)	-60·7 (-75·1 to -48·0)
Azerbaijan	All Ages	678 (539 to 934)	444 (347 to 655)	301 (208 to 418)	345 (133 to 638)	409 (313 to 594)	223 (92·7 to 409)	-66·9 (-87·7 to -47·6)
	15-49 years	357 (277 to 486)	242 (180 to 366)	162 (105 to 236)	179 (42·0 to 377)	237 (175 to 358)	120 (27·9 to 241)	-66·5 (-92·5 to -38·2)
	50-69 years	224 (167 to 329)	126 (87·5 to 210)	76·0 (45·0 to 125)	96·9 (34·3 to 172)	107 (73·5 to 183)	50·6 (16·8 to 101)	-77·1 (-93·7 to -60·9)
	70+ years	55·5 (41·6 to 75·5)	48·2 (36·2 to 69·2)	36·9 (25·2 to 52·4)	42·6 (26·1 to 64·2)	38·5 (28·8 to 55·4)	26·5 (16·4 to 42·6)	-51·8 (-72·4 to -32·7)
Georgia	All Ages	199 (180 to 219)	124 (110 to 137)	79·0 (53·4 to 107)	96·4 (38·8 to 156)	107 (95·5 to 121)	55·1 (24·0 to 93·2)	-72·3 (-87·8 to -53·8)

eTable 4. Risk deleted deaths due to all-form tuberculosis for alcohol use, smoking, and diabetes, and all three risk factors combined by age groups for 204 countries and territories (2020) with percent change between 2015 observed deaths and 2020 all-risk deleted deaths.

Location	Age group	2015 Observed deaths	2020 Observed deaths	2020 Smoking deleted deaths	2020 Alcohol deleted deaths	2020 Diabetes deleted deaths	2020 All-risk deleted deaths	Percent Change
	15-49 years	71·0 (66·5 to 76·2)	39·5 (36·4 to 43·1)	24·8 (17·4 to 33·5)	29·9 (10·5 to 53·4)	38·3 (35·4 to 42·0)	19·4 (7·01 to 34·7)	-72·7 (-90·1 to -52·1)
	50-69 years	87·2 (74·5 to 99·3)	52·7 (44·9 to 60·4)	29·5 (16·6 to 44·3)	39·4 (12·6 to 68·2)	43·7 (37·4 to 51·2)	18·8 (5·20 to 35·4)	-78·3 (-93·8 to -58·7)
	70+ years	38·5 (33·2 to 44·2)	30·1 (26·0 to 34·2)	23·3 (17·7 to 28·8)	25·7 (13·3 to 35·4)	23·3 (20·2 to 27·8)	15·6 (7·83 to 23·4)	-59·4 (-79·5 to -42·7)
Kazakhstan	All Ages	826 (774 to 889)	571 (515 to 620)	411 (313 to 530)	443 (155 to 701)	512 (465 to 562)	290 (102 to 468)	-64·8 (-87·7 to -40·5)
	15-49 years	451 (416 to 491)	273 (245 to 296)	186 (136 to 243)	200 (50·9 to 350)	264 (238 to 287)	134 (30·1 to 244)	-70·3 (-93·4 to -43·5)
	50-69 years	277 (255 to 309)	200 (174 to 224)	134 (92·4 to 182)	152 (42·3 to 242)	166 (145 to 190)	85·2 (19·0 to 146)	-69·1 (-93·3 to -45·7)
	70+ years	65·6 (61·5 to 73·2)	78·1 (70·7 to 85·7)	70·6 (61·9 to 80·7)	69·8 (42·7 to 90·7)	61·6 (55·6 to 69·2)	50·6 (29·8 to 65·5)	-23·0 (-53·1 to -1·01)
Kyrgyzstan	All Ages	470 (421 to 513)	344 (291 to 405)	236 (178 to 333)	286 (144 to 399)	325 (276 to 383)	193 (105 to 302)	-58·8 (-77·1 to -35·3)
	15-49 years	262 (233 to 289)	179 (152 to 210)	128 (102 to 172)	148 (75·8 to 212)	176 (149 to 205)	109 (60·6 to 164)	-58·4 (-76·3 to -36·8)
	50-69 years	155 (138 to 170)	127 (104 to 151)	74·7 (45·7 to 120)	102 (40·9 to 149)	115 (93·0 to 137)	55·0 (20·3 to 101)	-64·4 (-87·0 to -33·9)
	70+ years	31·4 (28·2 to 35·1)	23·0 (19·1 to 27·6)	18·2 (14·5 to 24·6)	21·2 (14·1 to 27·6)	20·1 (16·6 to 24·1)	14·9 (9·65 to 20·7)	-52·5 (-69·1 to -34·9)
Mongolia	All Ages	549 (387 to 753)	400 (296 to 578)	255 (148 to 376)	290 (33·3 to 527)	384 (283 to 546)	179 (-0·503 to 371)	-67·2 (-99·6 to -36·2)
	15-49 years	322 (226 to 453)	232 (169 to 360)	147 (87·1 to 228)	162 (2·21 to 330)	227 (164 to 345)	100 (-13·4 to 243)	-68·7 (-103 to -33·8)
	50-69 years	171 (116 to 232)	127 (90·1 to 175)	71·7 (33·7 to 113)	89·5 (1·33 to 163)	118 (82·1 to 160)	45·4 (-13·8 to 106)	-73·0 (-107 to -40·4)
	70+ years	28·8 (20·9 to 38·2)	21·7 (16·4 to 28·7)	17·7 (11·8 to 24·2)	18·8 (10·2 to 27·4)	19·9 (14·8 to 25·9)	14·2 (7·60 to 20·7)	-50·4 (-74·5 to -30·7)
Tajikistan	All Ages	881 (683 to 1120)	769 (571 to 960)	675 (501 to 854)	707 (463 to 929)	724 (538 to 918)	597 (404 to 774)	-32·1 (-51·5 to -13·4)
	15-49 years	401 (286 to 526)	325 (235 to 426)	280 (191 to 366)	285 (148 to 395)	318 (229 to 418)	245 (130 to 326)	-38·2 (-62·8 to -14·6)
	50-69 years	238 (181 to 304)	244 (171 to 320)	201 (138 to 261)	223 (131 to 309)	215 (150 to 281)	164 (91·4 to 225)	-31·0 (-57·8 to -3·62)
	70+ years	68·6 (49·0 to 91·8)	55·8 (36·8 to 75·7)	51·3 (32·0 to 70·2)	55·1 (36·6 to 75·3)	48·2 (31·9 to 66·3)	43·9 (27·5 to 61·0)	-35·7 (-49·8 to -19·7)
Turkmenistan	All Ages	486 (456 to 520)	397 (320 to 487)	314 (248 to 395)	323 (162 to 485)	376 (301 to 461)	249 (133 to 379)	-48·7 (-72·3 to -22·8)
	15-49 years	297 (276 to 327)	237 (188 to 293)	186 (144 to 236)	189 (87·9 to 297)	232 (184 to 287)	148 (72·4 to 232)	-49·9 (-74·9 to -22·4)
	50-69 years	131 (122 to 140)	117 (92·1 to 148)	87·4 (62·9 to 113)	93·4 (40·7 to 143)	104 (81·3 to 132)	63·6 (27·0 to 102)	-51·4 (-79·7 to -22·1)

eTable 4. Risk deleted deaths due to all-form tuberculosis for alcohol use, smoking, and diabetes, and all three risk factors combined by age groups for 204 countries and territories (2020) with percent change between 2015 observed deaths and 2020 all-risk deleted deaths.

Location	Age group	2015 Observed deaths	2020 Observed deaths	2020 Smoking deleted deaths	2020 Alcohol deleted deaths	2020 Diabetes deleted deaths	2020 All-risk deleted deaths	Percent Change
	70+ years	23·8 (22·0 to 25·7)	19·7 (16·4 to 23·4)	17·5 (14·3 to 21·2)	17·7 (11·5 to 22·8)	16·8 (13·6 to 20·2)	13·5 (8·74 to 18·5)	-43·0 (-63·9 to -23·7)
Uzbekistan	All Ages	2270 (2100 to 2460)	1420 (1280 to 1600)	1180 (987 to 1450)	1210 (700 to 1590)	1290 (1150 to 1490)	934 (562 to 1290)	-58·7 (-75·1 to -41·4)
	15-49 years	1070 (1000 to 1160)	618 (561 to 698)	511 (435 to 620)	515 (283 to 721)	602 (542 to 679)	428 (251 to 607)	-60·1 (-76·5 to -41·9)
	50-69 years	783 (715 to 856)	577 (504 to 657)	454 (352 to 589)	476 (232 to 669)	481 (418 to 565)	316 (150 to 478)	-59·4 (-81·4 to -36·0)
	70+ years	195 (177 to 212)	125 (111 to 141)	115 (97·9 to 132)	116 (81·1 to 138)	99·4 (84·4 to 116)	85·6 (59·1 to 103)	-56·0 (-70·6 to -46·0)
Central Europe	All Ages	2730 (2640 to 2850)	1970 (1850 to 2080)	1470 (1170 to 1800)	1490 (485 to 2600)	1670 (1550 to 1780)	964 (315 to 1760)	-64·7 (-88·5 to -34·2)
	15-49 years	667 (630 to 713)	430 (394 to 460)	283 (204 to 384)	313 (81·2 to 614)	414 (377 to 441)	205 (53·5 to 422)	-69·2 (-91·7 to -35·1)
	50-69 years	1200 (1150 to 1270)	803 (751 to 851)	542 (376 to 721)	580 (129 to 1090)	675 (626 to 725)	335 (65·0 to 691)	-72·0 (-94·6 to -40·3)
	70+ years	839 (791 to 880)	715 (667 to 757)	630 (544 to 704)	580 (261 to 873)	558 (514 to 620)	405 (178 to 614)	-51·7 (-78·9 to -26·3)
Albania	All Ages	42·6 (31·4 to 59·0)	35·0 (24·5 to 53·0)	22·6 (12·6 to 37·2)	28·3 (11·2 to 48·4)	31·2 (21·6 to 47·1)	16·7 (4·92 to 33·8)	-60·5 (-86·6 to -36·4)
	15-49 years	8·61 (6·56 to 12·1)	5·50 (3·74 to 8·21)	3·11 (1·79 to 5·09)	4·11 (0·977 to 7·75)	5·37 (3·62 to 8·00)	2·32 (0·225 to 5·03)	-72·8 (-97·2 to -46·5)
	50-69 years	14·3 (9·94 to 20·7)	8·70 (5·44 to 14·3)	4·66 (1·98 to 8·41)	6·55 (1·75 to 11·9)	7·81 (4·85 to 12·7)	3·17 (0·250 to 7·51)	-77·6 (-97·8 to -55·2)
	70+ years	18·7 (13·7 to 26·3)	20·1 (13·8 to 31·2)	14·2 (8·26 to 22·9)	17·0 (7·43 to 29·3)	17·3 (11·9 to 26·1)	10·6 (3·85 to 20·3)	-43·2 (-77·1 to -12·1)
Bosnia and Herzegovina	All Ages	167 (147 to 197)	124 (104 to 147)	92·4 (68·3 to 115)	102 (54·6 to 148)	94·7 (79·1 to 115)	59·5 (33·7 to 89·5)	-64·1 (-80·8 to -44·6)
	15-49 years	23·9 (20·0 to 29·2)	13·3 (11·1 to 16·3)	8·16 (5·32 to 11·7)	10·1 (3·70 to 17·5)	12·8 (10·7 to 15·7)	6·18 (2·02 to 11·0)	-73·9 (-92·3 to -52·5)
	50-69 years	57·1 (48·3 to 69·2)	39·1 (30·9 to 49·2)	23·9 (14·8 to 34·8)	29·5 (9·94 to 50·8)	29·6 (23·8 to 38·9)	13·7 (3·51 to 27·2)	-75·8 (-93·9 to -52·0)
	70+ years	85·0 (74·2 to 101)	71·1 (60·0 to 84·8)	59·7 (46·1 to 72·5)	61·8 (39·2 to 82·7)	51·7 (42·7 to 61·2)	39·0 (26·3 to 56·0)	-53·9 (-71·1 to -37·6)
Bulgaria	All Ages	123 (114 to 132)	88·7 (83·0 to 95·6)	64·9 (48·4 to 80·3)	65·5 (15·6 to 117)	71·8 (63·7 to 78·9)	39·0 (9·67 to 76·4)	-68·3 (-92·4 to -36·4)
	15-49 years	21·7 (19·8 to 23·8)	14·7 (13·1 to 16·1)	7·92 (4·48 to 12·2)	10·2 (1·09 to 21·8)	14·1 (12·5 to 15·5)	5·28 (0·00696 to 13·0)	-75·8 (-100 to -40·3)
	50-69 years	58·0 (52·8 to 63·9)	35·8 (32·9 to 40·4)	22·8 (14·5 to 31·3)	25·5 (4·90 to 48·9)	29·3 (25·8 to 33·5)	13·5 (2·29 to 30·2)	-76·8 (-96·1 to -46·1)
	70+ years	42·6 (38·9 to 46·0)	37·8 (34·8 to 40·8)	33·9 (28·9 to 37·9)	29·5 (9·95 to 46·6)	28·1 (24·0 to 31·8)	19·9 (6·12 to 32·9)	-53·2 (-85·8 to -21·9)
Croatia	All Ages	77·6 (71·2 to 84·7)	57·4 (51·4 to 63·2)	43·6 (31·0 to 54·9)	44·9 (15·7 to 70·7)	44·9 (38·9 to 50·9)	26·9 (8·32 to 47·5)	-65·3 (-89·5 to -37·4)

eTable 4. Risk deleted deaths due to all-form tuberculosis for alcohol use, smoking, and diabetes, and all three risk factors combined by age groups for 204 countries and territories (2020) with percent change between 2015 observed deaths and 2020 all-risk deleted deaths.

Location	Age group	2015 Observed deaths	2020 Observed deaths	2020 Smoking deleted deaths	2020 Alcohol deleted deaths	2020 Diabetes deleted deaths	2020 All-risk deleted deaths	Percent Change
	15-49 years	11·0 (10·3 to 11·9)	5·66 (5·04 to 6·38)	3·54 (2·15 to 4·90)	4·17 (0·998 to 7·57)	5·45 (4·87 to 6·21)	2·58 (0·574 to 5·01)	-76·5 (-94·8 to -53·0)
	50-69 years	27·1 (24·7 to 29·5)	16·9 (15·1 to 19·0)	10·8 (6·57 to 15·4)	12·2 (2·30 to 22·7)	13·5 (11·4 to 15·6)	6·19 (0·804 to 13·4)	-77·2 (-97·1 to -50·0)
	70+ years	39·1 (35·1 to 43·0)	34·6 (29·9 to 38·9)	28·9 (22·1 to 35·5)	28·3 (12·1 to 41·2)	25·6 (21·6 to 30·6)	17·9 (6·90 to 28·5)	-54·3 (-83·1 to -26·7)
Czechia	All Ages	61·6 (57·0 to 66·1)	34·4 (31·3 to 37·4)	26·7 (20·9 to 32·2)	25·8 (8·02 to 46·8)	26·3 (23·9 to 29·6)	15·6 (4·87 to 29·0)	-74·6 (-92·4 to -50·2)
	15-49 years	10·5 (9·46 to 11·5)	5·35 (4·81 to 6·24)	3·58 (2·50 to 4·81)	3·94 (0·887 to 7·92)	5·14 (4·57 to 6·02)	2·66 (0·676 to 5·21)	-74·6 (-93·5 to -47·8)
	50-69 years	23·3 (21·0 to 26·0)	9·39 (8·22 to 10·6)	6·02 (3·88 to 8·34)	6·62 (1·18 to 13·9)	7·24 (6·21 to 8·40)	3·34 (0·542 to 7·37)	-85·6 (-97·8 to -66·7)
	70+ years	27·4 (24·6 to 29·7)	19·4 (17·5 to 21·3)	16·9 (14·0 to 18·9)	15·0 (5·36 to 25·3)	13·7 (12·2 to 15·8)	9·36 (3·08 to 15·9)	-65·7 (-88·6 to -37·9)
Hungary	All Ages	117 (110 to 124)	65·8 (60·3 to 70·2)	51·5 (41·8 to 62·6)	50·5 (16·3 to 84·7)	51·6 (46·2 to 57·3)	31·3 (10·6 to 56·7)	-73·2 (-90·8 to -51·4)
	15-49 years	21·8 (19·9 to 23·8)	10·3 (9·19 to 11·4)	7·13 (5·21 to 9·25)	7·67 (2·02 to 13·8)	9·91 (8·74 to 11·0)	5·29 (1·44 to 10·0)	-75·7 (-93·1 to -53·4)
	50-69 years	49·9 (45·6 to 54·8)	26·5 (23·9 to 29·3)	18·4 (12·6 to 24·2)	19·4 (4·41 to 35·2)	20·8 (18·4 to 23·7)	10·7 (2·12 to 22·8)	-78·5 (-95·4 to -54·4)
	70+ years	44·0 (40·2 to 47·5)	28·4 (25·3 to 31·6)	25·3 (21·2 to 29·4)	22·8 (9·42 to 34·4)	20·2 (17·6 to 23·1)	14·7 (5·60 to 23·0)	-66·6 (-87·2 to -47·3)
Montenegro	All Ages	8·51 (6·91 to 10·9)	7·28 (5·65 to 9·53)	5·00 (3·42 to 7·24)	5·65 (1·81 to 10·4)	5·80 (4·36 to 7·80)	3·15 (0·982 to 5·97)	-63·0 (-88·4 to -34·9)
	15-49 years	1·54 (1·17 to 1·96)	1·12 (0·840 to 1·51)	0·611 (0·334 to 0·984)	0·815 (0·132 to 1·67)	1·07 (0·808 to 1·44)	0·434 (0·0175 to 0·965)	-71·7 (-98·7 to -39·0)
	50-69 years	3·74 (2·95 to 4·94)	2·92 (2·17 to 3·96)	1·69 (1·02 to 2·71)	2·16 (0·484 to 4·33)	2·32 (1·68 to 3·23)	1·01 (0·146 to 2·24)	-73·0 (-95·8 to -45·3)
	70+ years	3·17 (2·60 to 3·94)	3·20 (2·42 to 4·00)	2·65 (1·94 to 3·54)	2·64 (1·20 to 4·34)	2·37 (1·80 to 3·09)	1·67 (0·757 to 2·55)	-47·4 (-75·9 to -19·4)
North Macedonia	All Ages	58·7 (49·4 to 70·9)	34·3 (26·9 to 45·1)	24·0 (16·3 to 32·4)	26·6 (9·47 to 46·2)	26·0 (19·4 to 35·9)	14·2 (4·47 to 25·4)	-75·9 (-92·4 to -58·2)
	15-49 years	10·6 (8·80 to 12·3)	4·77 (3·62 to 6·75)	2·60 (1·51 to 4·20)	3·47 (0·784 to 6·48)	4·56 (3·44 to 6·43)	1·84 (0·260 to 4·05)	-82·5 (-97·6 to -61·0)
	50-69 years	24·4 (19·7 to 31·4)	12·3 (9·13 to 17·9)	7·35 (4·17 to 11·6)	9·18 (2·35 to 18·1)	9·29 (6·61 to 14·2)	4·11 (0·682 to 8·54)	-83·2 (-97·2 to -66·4)
	70+ years	23·3 (19·6 to 28·0)	17·0 (13·9 to 21·2)	13·9 (10·6 to 18·2)	13·8 (6·00 to 22·0)	11·9 (9·39 to 15·4)	8·03 (3·25 to 13·2)	-65·5 (-85·6 to -45·6)
Poland	All Ages	632 (612 to 652)	462 (438 to 487)	347 (259 to 431)	345 (101 to 629)	376 (345 to 405)	214 (53·9 to 419)	-66·1 (-91·5 to -33·3)
	15-49 years	140 (132 to 152)	97·1 (91·2 to 105)	66·9 (45·4 to 89·2)	70·2 (17·0 to 143)	92·3 (86·2 to 100)	47·5 (11·1 to 101)	-66·0 (-92·2 to -27·2)
	50-69 years	303 (293 to 313)	202 (190 to 214)	135 (83·4 to 184)	143 (22·4 to 286)	162 (147 to 176)	76·6 (-2·52 to 176)	-74·7 (-101 to -41·4)

eTable 4. Risk deleted deaths due to all-form tuberculosis for alcohol use, smoking, and diabetes, and all three risk factors combined by age groups for 204 countries and territories (2020) with percent change between 2015 observed deaths and 2020 all-risk deleted deaths.

Location	Age group	2015 Observed deaths	2020 Observed deaths	2020 Smoking deleted deaths	2020 Alcohol deleted deaths	2020 Diabetes deleted deaths	2020 All-risk deleted deaths	Percent Change
	70+ years	188 (177 to 197)	162 (150 to 172)	144 (122 to 161)	132 (59·8 to 196)	122 (110 to 135)	89·3 (39·7 to 138)	-52·5 (-78·8 to -26·1)
Romania	All Ages	1180 (1130 to 1280)	859 (769 to 943)	634 (493 to 781)	633 (184 to 1170)	771 (696 to 861)	428 (124 to 816)	-63·9 (-89·7 to -29·9)
	15-49 years	367 (339 to 407)	237 (210 to 263)	152 (106 to 205)	169 (33·7 to 346)	228 (202 to 254)	108 (19·4 to 231)	-70·5 (-94·5 to -34·5)
	50-69 years	531 (493 to 580)	374 (335 to 411)	255 (175 to 333)	266 (55·5 to 518)	330 (296 to 369)	163 (28·6 to 343)	-69·2 (-94·5 to -34·3)
	70+ years	269 (249 to 289)	234 (208 to 256)	213 (186 to 233)	184 (71·3 to 294)	199 (173 to 219)	143 (53·8 to 230)	-46·6 (-80·7 to -15·7)
Serbia	All Ages	162 (139 to 185)	130 (105 to 160)	102 (74·1 to 138)	105 (49·5 to 170)	102 (80·3 to 129)	66·0 (32·3 to 110)	-59·3 (-79·2 to -32·4)
	15-49 years	30·7 (26·8 to 34·8)	22·8 (19·2 to 28·2)	16·8 (12·6 to 22·8)	18·7 (9·86 to 30·3)	22·2 (18·5 to 27·5)	14·1 (8·30 to 21·9)	-53·7 (-73·3 to -27·0)
	50-69 years	65·2 (56·0 to 74·3)	49·2 (39·7 to 59·0)	34·5 (23·8 to 46·6)	38·1 (14·6 to 66·3)	39·0 (31·2 to 48·3)	22·1 (8·98 to 38·0)	-66·1 (-85·4 to -42·6)
	70+ years	64·5 (52·9 to 75·9)	57·1 (43·8 to 72·7)	49·7 (36·5 to 65·7)	47·0 (22·4 to 74·7)	39·5 (29·1 to 53·7)	28·6 (12·3 to 48·8)	-55·7 (-80·4 to -24·0)
Slovakia	All Ages	40·2 (34·3 to 47·9)	32·3 (25·9 to 38·8)	24·9 (19·3 to 30·9)	24·1 (6·71 to 44·0)	27·9 (21·8 to 34·1)	16·3 (4·29 to 29·8)	-59·6 (-88·2 to -23·8)
	15-49 years	7·92 (6·41 to 9·53)	5·47 (4·17 to 6·94)	3·57 (2·35 to 5·20)	3·74 (0·355 to 8·04)	5·28 (4·01 to 6·70)	2·37 (0·0884 to 5·79)	-69·9 (-98·9 to -27·5)
	50-69 years	17·8 (14·2 to 22·3)	12·3 (9·35 to 16·7)	8·39 (5·41 to 11·9)	8·69 (1·27 to 17·3)	10·5 (7·64 to 14·6)	5·08 (0·451 to 11·0)	-71·5 (-97·4 to -41·3)
	70+ years	14·3 (11·7 to 17·1)	14·3 (11·4 to 17·4)	12·7 (9·95 to 15·7)	11·5 (4·86 to 18·1)	11·9 (8·98 to 14·6)	8·62 (3·51 to 13·4)	-39·4 (-75·2 to -2·42)
Slovenia	All Ages	15·2 (14·0 to 16·3)	8·10 (7·16 to 8·78)	6·65 (5·25 to 7·86)	6·61 (3·11 to 9·69)	6·74 (5·97 to 7·42)	4·53 (1·94 to 6·86)	-70·2 (-87·4 to -53·6)
	15-49 years	2·10 (1·94 to 2·30)	0·717 (0·639 to 0·808)	0·452 (0·290 to 0·629)	0·553 (0·179 to 0·899)	0·693 (0·609 to 0·785)	0·343 (0·0892 to 0·610)	-83·7 (-95·8 to -71·1)
	50-69 years	5·51 (5·14 to 5·96)	2·19 (1·94 to 2·38)	1·40 (0·837 to 1·92)	1·65 (0·446 to 2·80)	1·84 (1·62 to 2·03)	0·859 (0·101 to 1·72)	-84·4 (-98·2 to -67·5)
	70+ years	7·53 (6·70 to 8·16)	5·16 (4·53 to 5·68)	4·77 (4·04 to 5·27)	4·38 (2·33 to 6·00)	4·18 (3·61 to 4·62)	3·30 (1·66 to 4·59)	-56·1 (-78·5 to -39·2)
Eastern Europe	All Ages	17700 (16800 to 19100)	9950 (9270 to 11000)	6940 (5210 to 9090)	7800 (3390 to 12900)	9310 (8660 to 10300)	5330 (2340 to 9020)	-69·9 (-86·6 to -48·8)
	15-49 years	8520 (7680 to 9710)	4570 (4040 to 5300)	3300 (2480 to 4290)	3630 (1650 to 6060)	4480 (3940 to 5210)	2710 (1400 to 4450)	-68·2 (-83·5 to -48·6)
	50-69 years	7730 (7510 to 7950)	4220 (3960 to 4580)	2630 (1760 to 3700)	3140 (932 to 5790)	3830 (3590 to 4210)	1830 (461 to 3710)	-76·4 (-93·9 to -52·2)
	70+ years	1320 (1260 to 1360)	1110 (1040 to 1160)	961 (822 to 1080)	985 (613 to 1270)	952 (870 to 1030)	743 (482 to 986)	-43·6 (-63·5 to -27·1)
Belarus	All Ages	391 (362 to 428)	252 (222 to 284)	170 (121 to 227)	192 (69·4 to 332)	238 (207 to 269)	127 (51·5 to 228)	-67·5 (-87·5 to -40·8)

eTable 4. Risk deleted deaths due to all-form tuberculosis for alcohol use, smoking, and diabetes, and all three risk factors combined by age groups for 204 countries and territories (2020) with percent change between 2015 observed deaths and 2020 all-risk deleted deaths.

Location	Age group	2015 Observed deaths	2020 Observed deaths	2020 Smoking deleted deaths	2020 Alcohol deleted deaths	2020 Diabetes deleted deaths	2020 All-risk deleted deaths	Percent Change
	15-49 years	162 (147 to 181)	88·6 (76·1 to 102)	62·1 (45·8 to 80·1)	68·3 (26·5 to 122)	87·0 (74·5 to 99·9)	49·4 (22·1 to 84·6)	-69·4 (-86·0 to -46·3)
	50-69 years	188 (173 to 208)	133 (115 to 150)	81·9 (49·9 to 116)	97·0 (24·0 to 184)	123 (107 to 140)	56·8 (13·9 to 117)	-69·8 (-93·0 to -38·0)
	70+ years	39·0 (36·3 to 41·7)	29·3 (26·1 to 33·3)	24·7 (20·7 to 29·6)	25·6 (12·9 to 33·9)	26·4 (23·5 to 30·3)	19·7 (10·6 to 26·6)	-49·3 (-73·6 to -32·5)
Estonia	All Ages	45·3 (41·4 to 48·9)	26·5 (23·5 to 30·2)	21·2 (17·7 to 26·3)	21·8 (11·3 to 33·6)	24·4 (21·3 to 28·5)	16·8 (9·66 to 26·4)	-62·9 (-78·3 to -41·6)
	15-49 years	18·9 (15·4 to 22·0)	9·98 (7·69 to 13·0)	8·61 (6·30 to 11·2)	8·73 (5·78 to 13·0)	9·84 (7·53 to 12·8)	7·74 (5·23 to 11·2)	-59·3 (-69·2 to -43·2)
	50-69 years	18·1 (16·7 to 19·4)	11·2 (10·3 to 12·3)	7·80 (5·87 to 10·4)	8·47 (2·95 to 15·3)	10·1 (9·06 to 11·3)	5·55 (1·70 to 10·2)	-69·4 (-90·7 to -41·7)
	70+ years	8·00 (7·21 to 8·65)	5·17 (4·56 to 5·73)	4·61 (3·87 to 5·25)	4·47 (2·29 to 6·21)	4·32 (3·68 to 4·87)	3·38 (1·78 to 4·63)	-57·8 (-78·4 to -40·2)
Latvia	All Ages	95·1 (85·4 to 106)	55·2 (48·9 to 62·7)	43·5 (35·9 to 52·4)	44·1 (20·7 to 72·5)	50·7 (44·8 to 58·3)	33·4 (16·1 to 54·9)	-64·9 (-82·7 to -42·2)
	15-49 years	36·0 (30·4 to 43·9)	20·6 (16·7 to 26·1)	16·8 (12·3 to 22·2)	17·2 (9·95 to 28·4)	20·2 (16·4 to 25·7)	14·5 (9·51 to 22·2)	-59·8 (-72·7 to -38·7)
	50-69 years	43·3 (39·5 to 47·5)	23·5 (21·3 to 26·0)	16·5 (12·7 to 21·4)	17·4 (5·21 to 32·8)	21·0 (18·8 to 23·7)	11·4 (3·13 to 22·2)	-73·6 (-92·7 to -47·7)
	70+ years	15·2 (14·0 to 16·3)	10·9 (9·91 to 11·7)	9·79 (8·47 to 11·0)	9·26 (4·39 to 12·7)	9·28 (8·34 to 10·2)	7·21 (3·39 to 9·94)	-52·7 (-77·9 to -33·2)
Lithuania	All Ages	210 (198 to 224)	105 (97·1 to 115)	77·2 (59·9 to 96·6)	79·3 (26·9 to 137)	96·8 (89·6 to 106)	55·6 (19·0 to 99·5)	-73·4 (-90·8 to -51·7)
	15-49 years	69·5 (64·8 to 75·1)	34·7 (31·5 to 39·3)	24·4 (18·2 to 32·1)	25·5 (7·20 to 47·8)	33·8 (30·8 to 38·3)	18·2 (5·48 to 34·4)	-73·8 (-92·1 to -49·0)
	50-69 years	102 (94·1 to 109)	46·5 (42·8 to 51·7)	31·8 (22·8 to 42·5)	33·6 (7·55 to 64·5)	42·2 (38·7 to 46·9)	21·4 (3·64 to 43·1)	-79·0 (-96·3 to -56·9)
	70+ years	37·2 (34·0 to 40·4)	23·2 (20·7 to 25·6)	20·5 (17·2 to 23·2)	19·8 (8·92 to 27·5)	20·5 (18·3 to 22·7)	15·6 (7·27 to 21·8)	-58·0 (-80·8 to -40·3)
Republic of Moldova	All Ages	276 (256 to 293)	144 (132 to 154)	94·5 (66·1 to 127)	107 (30·7 to 207)	132 (122 to 143)	67·0 (22·7 to 133)	-75·8 (-92·0 to -54·0)
	15-49 years	114 (104 to 124)	54·6 (49·1 to 61·5)	35·2 (25·4 to 47·6)	40·4 (11·7 to 79·8)	53·0 (47·3 to 59·9)	26·5 (9·28 to 51·8)	-76·9 (-91·8 to -56·9)
	50-69 years	138 (127 to 149)	70·5 (64·1 to 76·7)	43·1 (25·7 to 62·9)	50·7 (10·3 to 103)	63·0 (56·6 to 68·9)	28·1 (6·04 to 64·8)	-79·7 (-95·8 to -55·7)
	70+ years	21·6 (19·8 to 23·3)	17·4 (16·2 to 18·9)	15·1 (12·7 to 17·2)	15·0 (7·04 to 21·0)	15·0 (13·5 to 16·7)	11·3 (5·51 to 16·0)	-47·7 (-75·2 to -25·5)
Russian Federation	All Ages	12600 (11900 to 13400)	6460 (5990 to 6990)	4420 (3200 to 5850)	5060 (2180 to 8440)	6030 (5570 to 6590)	3390 (1480 to 5640)	-73·0 (-88·0 to -54·9)
	15-49 years	6320 (5760 to 7130)	3100 (2720 to 3510)	2170 (1610 to 2840)	2430 (971 to 4170)	3040 (2670 to 3450)	1760 (797 to 2930)	-72·2 (-87·4 to -53·9)
	50-69 years	5250 (5170 to 5370)	2520 (2400 to 2650)	1530 (936 to 2210)	1880 (555 to 3370)	2280 (2160 to 2420)	1060 (296 to 2060)	-79·8 (-94·3 to -60·7)

eTable 4. Risk deleted deaths due to all-form tuberculosis for alcohol use, smoking, and diabetes, and all three risk factors combined by age groups for 204 countries and territories (2020) with percent change between 2015 observed deaths and 2020 all-risk deleted deaths.

Location	Age group	2015 Observed deaths	2020 Observed deaths	2020 Smoking deleted deaths	2020 Alcohol deleted deaths	2020 Diabetes deleted deaths	2020 All-risk deleted deaths	Percent Change
	70+ years	879 (834 to 903)	797 (737 to 835)	692 (576 to 782)	714 (452 to 904)	679 (621 to 736)	536 (351 to 694)	-39·1 (-60·5 to -22·2)
Ukraine	All Ages	4120 (3780 to 4600)	2910 (2470 to 3500)	2110 (1490 to 2830)	2300 (939 to 3800)	2730 (2280 to 3320)	1640 (588 to 2750)	-60·2 (-84·6 to -32·6)
	15-49 years	1800 (1540 to 2150)	1260 (1050 to 1550)	987 (698 to 1270)	1040 (571 to 1580)	1230 (1020 to 1520)	839 (437 to 1270)	-53·4 (-75·0 to -31·3)
	50-69 years	1990 (1810 to 2160)	1410 (1130 to 1690)	923 (529 to 1400)	1050 (243 to 1980)	1290 (1020 to 1540)	643 (61·1 to 1330)	-67·7 (-96·9 to -31·7)
	70+ years	318 (295 to 340)	226 (191 to 260)	194 (155 to 234)	197 (95·7 to 271)	198 (162 to 228)	150 (72·9 to 211)	-52·8 (-78·1 to -34·0)
High-income	All Ages	13200 (11800 to 14300)	12500 (11000 to 13600)	10900 (8970 to 12400)	10200 (4570 to 15200)	10400 (9190 to 11700)	7520 (3320 to 11200)	-43·2 (-74·0 to -15·3)
	15-49 years	1800 (1440 to 2120)	1500 (1200 to 1810)	1330 (1030 to 1610)	1320 (805 to 1860)	1480 (1180 to 1780)	1180 (763 to 1560)	-34·4 (-53·1 to -16·1)
	50-69 years	2700 (2490 to 2920)	2350 (2120 to 2540)	1850 (1450 to 2240)	1880 (841 to 2910)	2050 (1810 to 2300)	1360 (640 to 2090)	-49·7 (-75·5 to -22·1)
	70+ years	8660 (7320 to 9370)	8630 (7120 to 9510)	7660 (5910 to 8830)	6950 (2920 to 10500)	6860 (5680 to 7980)	4930 (1890 to 7650)	-43·1 (-77·7 to -14·4)
Australasia	All Ages	93·8 (84·7 to 101)	81·2 (72·7 to 88·7)	73·5 (62·0 to 83·2)	62·7 (22·1 to 104)	70·3 (62·8 to 78·4)	49·5 (16·4 to 83·8)	-47·2 (-82·2 to -12·8)
	15-49 years	7·82 (6·73 to 9·43)	6·17 (5·16 to 7·58)	5·30 (4·10 to 6·71)	5·12 (2·46 to 7·95)	6·03 (5·02 to 7·51)	4·44 (2·29 to 6·77)	-43·4 (-69·2 to -15·4)
	50-69 years	21·8 (20·3 to 23·9)	18·1 (16·4 to 20·1)	15·1 (12·2 to 17·5)	13·8 (4·83 to 23·4)	16·1 (14·4 to 18·0)	10·4 (3·46 to 18·4)	-52·4 (-84·0 to -15·1)
	70+ years	63·7 (55·4 to 69·1)	56·6 (48·3 to 61·7)	52·8 (44·5 to 59·2)	43·5 (13·4 to 72·5)	47·9 (41·1 to 53·1)	34·4 (9·36 to 58·5)	-46·0 (-84·8 to -10·5)
Australia	All Ages	77·2 (69·5 to 83·9)	75·7 (67·6 to 82·8)	68·8 (58·1 to 77·9)	58·4 (20·4 to 96·9)	65·7 (58·5 to 73·3)	46·4 (15·3 to 78·7)	-39·9 (-79·9 to -0·204)
	15-49 years	6·12 (5·25 to 7·36)	5·59 (4·69 to 6·88)	4·80 (3·71 to 6·09)	4·63 (2·22 to 7·23)	5·47 (4·56 to 6·82)	4·02 (2·07 to 6·15)	-34·5 (-64·6 to -1·17)
	50-69 years	17·1 (15·7 to 18·8)	16·4 (14·8 to 18·2)	13·7 (11·1 to 15·9)	12·5 (4·35 to 21·2)	14·6 (13·1 to 16·5)	9·48 (3·18 to 16·9)	-44·4 (-81·1 to -1·33)
	70+ years	53·7 (46·7 to 58·7)	53·5 (45·5 to 58·3)	50·0 (42·1 to 56·1)	41·1 (12·6 to 68·6)	45·4 (38·8 to 50·2)	32·6 (8·91 to 55·7)	-39·2 (-82·8 to 1·13)
New Zealand	All Ages	16·6 (15·3 to 17·6)	5·49 (4·97 to 5·89)	4·71 (3·83 to 5·29)	4·33 (1·65 to 6·85)	4·62 (4·21 to 5·11)	3·14 (1·09 to 5·05)	-81·0 (-93·3 to -68·8)
	15-49 years	1·70 (1·47 to 2·03)	0·579 (0·475 to 0·720)	0·495 (0·383 to 0·617)	0·490 (0·241 to 0·745)	0·562 (0·458 to 0·706)	0·420 (0·224 to 0·631)	-75·4 (-86·0 to -65·4)
	50-69 years	4·75 (4·49 to 5·02)	1·73 (1·59 to 1·84)	1·36 (1·03 to 1·66)	1·34 (0·459 to 2·19)	1·47 (1·35 to 1·61)	0·914 (0·275 to 1·59)	-80·7 (-94·3 to -66·5)
	70+ years	10·0 (8·94 to 10·9)	3·14 (2·68 to 3·46)	2·81 (2·29 to 3·17)	2·46 (0·791 to 3·86)	2·55 (2·25 to 2·86)	1·77 (0·449 to 2·87)	-82·3 (-95·4 to -70·6)
High-income Asia Pacific	All Ages	6210 (5260 to 6870)	6200 (5080 to 6930)	5320 (4050 to 6360)	5010 (2050 to 7700)	5050 (4170 to 5910)	3530 (1320 to 5440)	-43·3 (-78·4 to -15·5)

eTable 4. Risk deleted deaths due to all-form tuberculosis for alcohol use, smoking, and diabetes, and all three risk factors combined by age groups for 204 countries and territories (2020) with percent change between 2015 observed deaths and 2020 all-risk deleted deaths.

Location	Age group	2015 Observed deaths	2020 Observed deaths	2020 Smoking deleted deaths	2020 Alcohol deleted deaths	2020 Diabetes deleted deaths	2020 All-risk deleted deaths	Percent Change
	15-49 years	252 (229 to 281)	177 (159 to 196)	134 (108 to 164)	136 (52·0 to 219)	171 (153 to 188)	103 (41·2 to 167)	-59·2 (-83·2 to -32·1)
	50-69 years	827 (766 to 919)	657 (593 to 734)	483 (359 to 617)	498 (157 to 846)	572 (516 to 656)	327 (92·2 to 556)	-60·4 (-89·0 to -32·4)
	70+ years	5120 (4220 to 5700)	5360 (4290 to 6040)	4700 (3510 to 5580)	4370 (1860 to 6600)	4300 (3450 to 5110)	3090 (1200 to 4770)	-39·7 (-76·2 to -11·0)
Brunei Darussalam	All Ages	18·0 (15·7 to 21·5)	17·9 (15·0 to 21·4)	15·5 (12·0 to 19·2)	17·6 (14·2 to 21·3)	14·4 (11·6 to 17·3)	12·3 (9·01 to 15·3)	-31·5 (-47·1 to -13·5)
	15-49 years	3·61 (3·09 to 4·33)	3·64 (2·97 to 4·35)	3·02 (2·34 to 3·74)	3·54 (2·86 to 4·24)	3·44 (2·81 to 4·15)	2·80 (2·10 to 3·46)	-22·4 (-44·3 to -0·316)
	50-69 years	4·69 (3·85 to 5·64)	5·07 (4·07 to 6·28)	3·99 (2·73 to 5·15)	4·96 (3·69 to 6·24)	3·81 (2·93 to 4·90)	2·94 (1·78 to 3·93)	-37·2 (-58·9 to -11·1)
	70+ years	9·45 (7·98 to 11·4)	8·97 (7·14 to 11·3)	8·26 (6·51 to 10·3)	8·90 (7·06 to 11·3)	6·91 (5·23 to 8·70)	6·38 (4·79 to 7·98)	-32·3 (-46·4 to -16·0)
Japan	All Ages	3450 (2880 to 3800)	3700 (2980 to 4140)	3300 (2460 to 3920)	3010 (1310 to 4470)	2990 (2390 to 3480)	2190 (892 to 3380)	-36·9 (-74·0 to -8·93)
	15-49 years	55·2 (49·6 to 60·9)	45·5 (40·6 to 49·3)	37·7 (31·2 to 44·2)	37·4 (17·8 to 56·6)	44·5 (39·4 to 48·4)	31·3 (15·7 to 47·3)	-43·3 (-69·3 to -15·2)
	50-69 years	279 (260 to 301)	220 (202 to 235)	170 (134 to 206)	176 (72·0 to 273)	197 (174 to 215)	127 (52·3 to 201)	-54·7 (-80·3 to -28·6)
	70+ years	3120 (2540 to 3450)	3430 (2710 to 3860)	3090 (2290 to 3670)	2800 (1220 to 4140)	2740 (2150 to 3220)	2020 (822 to 3140)	-35·2 (-73·5 to -7·31)
Republic of Korea	All Ages	2670 (2290 to 3030)	2430 (2030 to 2830)	1950 (1490 to 2420)	1930 (668 to 2970)	2000 (1680 to 2380)	1290 (427 to 2040)	-51·6 (-84·4 to -24·9)
	15-49 years	185 (163 to 214)	123 (108 to 141)	89·1 (67·6 to 113)	90·4 (27·7 to 155)	118 (104 to 133)	64·7 (20·7 to 113)	-65·1 (-88·7 to -37·8)
	50-69 years	522 (461 to 618)	418 (349 to 499)	297 (204 to 405)	305 (71·4 to 554)	360 (299 to 429)	188 (31·8 to 359)	-63·8 (-94·2 to -33·8)
	70+ years	1960 (1630 to 2280)	1890 (1540 to 2250)	1560 (1180 to 1960)	1530 (567 to 2290)	1520 (1240 to 1860)	1040 (377 to 1610)	-47·0 (-80·9 to -18·6)
Singapore	All Ages	70·2 (64·3 to 76·3)	56·2 (51·6 to 61·6)	51·1 (43·9 to 56·8)	52·4 (38·2 to 60·4)	44·9 (39·3 to 49·7)	38·4 (27·1 to 45·9)	-45·3 (-60·3 to -36·3)
	15-49 years	7·31 (6·44 to 8·23)	5·10 (4·54 to 5·80)	4·46 (3·69 to 5·16)	4·54 (2·78 to 5·92)	4·89 (4·32 to 5·59)	3·87 (2·36 to 5·05)	-47·1 (-65·2 to -34·3)
	50-69 years	21·0 (18·4 to 23·8)	13·7 (12·2 to 15·3)	11·7 (9·75 to 13·8)	12·4 (8·13 to 15·4)	11·5 (10·0 to 13·3)	9·01 (5·59 to 11·7)	-57·1 (-71·5 to -46·1)
	70+ years	41·5 (36·3 to 46·0)	37·1 (32·1 to 41·6)	34·6 (28·6 to 39·0)	35·2 (27·9 to 40·8)	28·2 (24·0 to 32·3)	25·2 (18·4 to 29·9)	-39·1 (-54·2 to -30·1)
High-income North America	All Ages	950 (869 to 1050)	1050 (965 to 1150)	891 (745 to 1020)	868 (425 to 1250)	829 (742 to 942)	597 (312 to 870)	-37·2 (-67·4 to -8·77)
	15-49 years	156 (130 to 194)	174 (150 to 210)	147 (116 to 176)	145 (74·9 to 219)	168 (143 to 204)	122 (68·5 to 178)	-21·9 (-53·9 to 15·1)
	50-69 years	333 (298 to 377)	363 (329 to 409)	288 (223 to 348)	297 (144 to 444)	296 (257 to 343)	204 (104 to 304)	-38·8 (-67·1 to -11·3)

eTable 4. Risk deleted deaths due to all-form tuberculosis for alcohol use, smoking, and diabetes, and all three risk factors combined by age groups for 204 countries and territories (2020) with percent change between 2015 observed deaths and 2020 all-risk deleted deaths.

Location	Age group	2015 Observed deaths	2020 Observed deaths	2020 Smoking deleted deaths	2020 Alcohol deleted deaths	2020 Diabetes deleted deaths	2020 All-risk deleted deaths	Percent Change
	70+ years	456 (396 to 488)	509 (442 to 546)	451 (354 to 508)	421 (191 to 599)	360 (311 to 416)	266 (111 to 396)	-41·7 (-75·1 to -14·9)
Canada	All Ages	126 (112 to 142)	124 (113 to 140)	107 (88·7 to 124)	102 (45·7 to 151)	103 (89·7 to 119)	74·7 (34·6 to 112)	-40·6 (-72·2 to -12·5)
	15-49 years	19·2 (15·0 to 25·6)	20·0 (16·0 to 25·9)	17·7 (13·3 to 22·9)	17·6 (10·2 to 26·2)	19·8 (15·8 to 25·7)	15·9 (9·81 to 22·9)	-17·5 (-41·3 to 7·98)
	50-69 years	35·6 (29·9 to 42·2)	32·8 (27·9 to 38·5)	26·5 (20·1 to 32·4)	26·7 (11·7 to 42·0)	29·1 (24·4 to 35·2)	20·0 (9·02 to 31·4)	-43·9 (-72·6 to -16·0)
	70+ years	70·4 (62·2 to 76·7)	71·1 (62·0 to 77·1)	62·4 (48·5 to 71·1)	57·7 (22·4 to 84·9)	53·6 (46·8 to 61·2)	38·3 (13·2 to 58·9)	-45·5 (-81·1 to -17·7)
Greenland	All Ages	2·71 (2·23 to 3·15)	2·64 (2·23 to 3·13)	1·99 (1·45 to 2·50)	2·09 (0·869 to 3·28)	2·20 (1·81 to 2·62)	1·32 (0·588 to 2·16)	-51·0 (-80·1 to -19·9)
	15-49 years	0·418 (0·338 to 0·491)	0·329 (0·266 to 0·390)	0·275 (0·211 to 0·333)	0·289 (0·183 to 0·390)	0·325 (0·264 to 0·385)	0·249 (0·171 to 0·335)	-40·2 (-56·3 to -22·0)
	50-69 years	1·25 (1·02 to 1·46)	1·27 (1·06 to 1·48)	0·890 (0·627 to 1·18)	1·02 (0·477 to 1·64)	1·12 (0·922 to 1·32)	0·658 (0·354 to 1·04)	-47·3 (-72·3 to -14·1)
	70+ years	1·01 (0·775 to 1·27)	1·02 (0·799 to 1·34)	0·803 (0·516 to 1·08)	0·765 (0·126 to 1·30)	0·735 (0·521 to 1·02)	0·395 (-0·0562 to 0·808)	-60·5 (-105 to -22·5)
United States of America	All Ages	822 (752 to 901)	925 (849 to 1010)	782 (654 to 897)	763 (378 to 1100)	724 (648 to 824)	521 (275 to 760)	-36·6 (-66·7 to -7·98)
	15-49 years	137 (115 to 168)	154 (133 to 183)	129 (102 to 155)	127 (64·4 to 193)	148 (127 to 177)	106 (57·8 to 156)	-22·4 (-55·6 to 16·1)
	50-69 years	296 (265 to 333)	329 (299 to 368)	261 (202 to 317)	269 (131 to 401)	266 (231 to 307)	183 (93·7 to 272)	-38·1 (-66·5 to -10·6)
	70+ years	384 (332 to 412)	437 (377 to 470)	388 (305 to 438)	362 (168 to 514)	306 (264 to 355)	227 (96·5 to 337)	-40·9 (-74·0 to -14·5)
Southern Latin America	All Ages	2230 (1880 to 2490)	2000 (1700 to 2230)	1790 (1480 to 2030)	1720 (976 to 2360)	1800 (1500 to 2020)	1430 (890 to 1900)	-36·1 (-59·1 to -15·6)
	15-49 years	929 (705 to 1090)	802 (615 to 959)	737 (544 to 898)	729 (474 to 960)	794 (609 to 951)	678 (459 to 860)	-27·2 (-42·8 to -12·1)
	50-69 years	711 (620 to 791)	666 (575 to 743)	553 (441 to 656)	559 (299 to 813)	594 (510 to 673)	435 (265 to 617)	-39·0 (-62·5 to -14·0)
	70+ years	554 (503 to 588)	509 (462 to 539)	478 (418 to 519)	407 (163 to 604)	384 (347 to 434)	290 (107 to 444)	-47·8 (-80·3 to -21·2)
Argentina	All Ages	1460 (1210 to 1660)	1330 (1100 to 1530)	1190 (949 to 1370)	1160 (695 to 1580)	1220 (1010 to 1420)	985 (641 to 1290)	-32·6 (-54·2 to -13·4)
	15-49 years	666 (499 to 797)	578 (435 to 711)	535 (388 to 669)	528 (342 to 705)	573 (431 to 705)	494 (332 to 639)	-25·9 (-41·7 to -11·5)
	50-69 years	472 (408 to 528)	449 (383 to 511)	372 (289 to 444)	384 (217 to 544)	408 (344 to 470)	304 (194 to 417)	-35·7 (-58·6 to -13·0)
	70+ years	296 (273 to 316)	287 (264 to 311)	266 (229 to 289)	232 (100 to 337)	224 (201 to 255)	170 (70·4 to 252)	-42·8 (-75·0 to -16·0)
Chile	All Ages	682 (610 to 733)	592 (517 to 637)	536 (463 to 604)	492 (251 to 722)	506 (435 to 560)	392 (223 to 564)	-42·6 (-66·7 to -19·5)

eTable 4. Risk deleted deaths due to all-form tuberculosis for alcohol use, smoking, and diabetes, and all three risk factors combined by age groups for 204 countries and territories (2020) with percent change between 2015 observed deaths and 2020 all-risk deleted deaths.

Location	Age group	2015 Observed deaths	2020 Observed deaths	2020 Smoking deleted deaths	2020 Alcohol deleted deaths	2020 Diabetes deleted deaths	2020 All-risk deleted deaths	Percent Change
	15-49 years	234 (185 to 262)	198 (157 to 226)	180 (139 to 212)	178 (120 to 236)	196 (155 to 224)	164 (116 to 211)	-30·2 (-48·2 to -12·8)
	50-69 years	209 (186 to 226)	191 (167 to 208)	162 (134 to 187)	154 (74·2 to 241)	163 (140 to 181)	116 (60·5 to 179)	-44·4 (-69·6 to -14·6)
	70+ years	231 (205 to 249)	198 (176 to 217)	190 (169 to 211)	155 (53·5 to 240)	142 (125 to 161)	107 (32·0 to 172)	-53·6 (-85·5 to -27·3)
Uruguay	All Ages	86·9 (76·5 to 101)	75·3 (66·1 to 86·5)	63·8 (52·4 to 76·6)	62·7 (30·9 to 91·7)	66·1 (56·6 to 76·9)	48·3 (25·9 to 68·6)	-44·5 (-69·8 to -20·6)
	15-49 years	29·1 (22·3 to 38·5)	25·5 (19·8 to 33·3)	22·4 (16·7 to 29·3)	22·7 (14·4 to 32·6)	25·2 (19·5 to 32·9)	20·3 (13·4 to 28·3)	-30·6 (-49·2 to -12·2)
	50-69 years	30·3 (26·6 to 34·8)	25·5 (22·3 to 29·8)	19·4 (14·2 to 24·6)	20·7 (9·93 to 31·3)	22·5 (19·2 to 26·3)	14·6 (7·44 to 22·7)	-51·8 (-75·0 to -27·7)
	70+ years	26·7 (23·2 to 29·4)	23·7 (20·3 to 26·3)	21·5 (18·4 to 24·4)	18·8 (6·74 to 29·3)	17·9 (15·3 to 20·7)	12·9 (4·17 to 20·7)	-51·7 (-84·9 to -25·7)
Western Europe	All Ages	3730 (3350 to 4100)	3190 (2830 to 3460)	2800 (2370 to 3140)	2530 (1040 to 3930)	2680 (2370 to 3030)	1920 (789 to 2970)	-48·7 (-77·8 to -19·3)
	15-49 years	450 (357 to 585)	344 (267 to 451)	302 (227 to 395)	304 (179 to 449)	339 (263 to 447)	273 (170 to 384)	-39·7 (-55·4 to -22·4)
	50-69 years	805 (720 to 933)	642 (565 to 742)	509 (398 to 633)	514 (219 to 821)	569 (498 to 678)	381 (179 to 616)	-52·8 (-76·6 to -25·0)
	70+ years	2460 (2130 to 2620)	2190 (1880 to 2360)	1980 (1570 to 2210)	1710 (597 to 2700)	1770 (1520 to 1990)	1250 (420 to 1980)	-49·2 (-83·3 to -17·3)
Andorra	All Ages	0·109 (0·0882 to 0·135)	0·105 (0·0790 to 0·141)	0·0851 (0·0564 to 0·119)	0·0776 (0·0147 to 0·141)	0·0883 (0·0648 to 0·122)	0·0527 (0·00729 to 0·0966)	-51·4 (-93·7 to -10·5)
	15-49 years	0·0140 (0·0108 to 0·0172)	0·0113 (0·00859 to 0·0145)	0·00798 (0·00463 to 0·0114)	0·00834 (0·00214 to 0·0166)	0·0108 (0·00821 to 0·0141)	0·00592 (0·00142 to 0·0113)	-57·4 (-88·3 to -14·9)
	50-69 years	0·0260 (0·0201 to 0·0330)	0·0250 (0·0174 to 0·0344)	0·0169 (0·00898 to 0·0264)	0·0174 (0·00157 to 0·0379)	0·0215 (0·0150 to 0·0303)	0·0101 (-0·000164 to 0·0228)	-60·7 (-102 to -12·1)
	70+ years	0·0687 (0·0518 to 0·0861)	0·0691 (0·0506 to 0·0946)	0·0601 (0·0422 to 0·0831)	0·0517 (0·0121 to 0·0886)	0·0559 (0·0404 to 0·0796)	0·0365 (0·00686 to 0·0646)	-46·6 (-91·6 to -7·84)
Austria	All Ages	68·3 (61·6 to 74·2)	59·0 (52·4 to 63·8)	49·0 (40·4 to 57·4)	45·9 (16·8 to 74·8)	54·3 (47·8 to 59·0)	35·7 (13·1 to 56·5)	-47·9 (-80·3 to -14·0)
	15-49 years	7·18 (5·69 to 9·31)	5·50 (4·14 to 7·24)	4·65 (3·30 to 6·33)	4·82 (2·58 to 7·25)	5·45 (4·10 to 7·19)	4·22 (2·43 to 6·12)	-41·7 (-60·3 to -23·8)
	50-69 years	17·6 (16·0 to 19·5)	12·6 (11·4 to 14·1)	8·54 (5·90 to 11·4)	9·44 (2·61 to 17·3)	11·8 (10·6 to 13·3)	6·15 (1·64 to 11·6)	-65·2 (-90·4 to -33·1)
	70+ years	43·4 (38·2 to 46·9)	40·8 (35·1 to 44·6)	35·7 (27·5 to 40·8)	31·5 (10·7 to 51·0)	37·0 (31·7 to 40·7)	25·2 (8·21 to 41·2)	-42·1 (-80·9 to -5·34)
Belgium	All Ages	83·1 (74·0 to 91·7)	64·2 (56·3 to 71·1)	53·9 (42·2 to 63·3)	49·9 (16·8 to 81·8)	55·8 (48·6 to 62·6)	37·1 (12·0 to 60·8)	-55·6 (-84·5 to -25·2)
	15-49 years	8·97 (6·95 to 11·8)	6·38 (4·75 to 8·71)	5·61 (3·94 to 7·75)	5·68 (3·23 to 8·29)	6·30 (4·68 to 8·62)	5·10 (3·08 to 7·26)	-43·5 (-57·6 to -27·6)
	50-69 years	17·7 (15·7 to 20·5)	14·6 (12·9 to 16·9)	10·7 (7·35 to 13·8)	11·1 (3·38 to 20·2)	13·0 (11·1 to 15·4)	7·56 (2·23 to 13·6)	-57·5 (-86·5 to -22·6)

eTable 4. Risk deleted deaths due to all-form tuberculosis for alcohol use, smoking, and diabetes, and all three risk factors combined by age groups for 204 countries and territories (2020) with percent change between 2015 observed deaths and 2020 all-risk deleted deaths.

Location	Age group	2015 Observed deaths	2020 Observed deaths	2020 Smoking deleted deaths	2020 Alcohol deleted deaths	2020 Diabetes deleted deaths	2020 All-risk deleted deaths	Percent Change
	70+ years	56·2 (49·2 to 62·1)	43·0 (36·8 to 47·6)	37·4 (27·3 to 43·2)	32·9 (10·2 to 54·4)	36·2 (30·8 to 40·9)	24·2 (6·69 to 41·3)	-57·1 (-87·8 to -25·0)
Cyprus	All Ages	6·83 (5·74 to 8·26)	6·53 (5·54 to 7·73)	5·14 (3·97 to 6·78)	5·13 (1·89 to 8·31)	4·94 (4·10 to 6·05)	3·11 (1·07 to 5·34)	-54·6 (-83·8 to -27·2)
	15-49 years	0·854 (0·666 to 1·14)	0·791 (0·602 to 1·05)	0·652 (0·437 to 0·888)	0·692 (0·413 to 1·06)	0·780 (0·591 to 1·04)	0·591 (0·377 to 0·889)	-31·2 (-52·0 to -9·38)
	50-69 years	1·57 (1·27 to 1·95)	1·44 (1·18 to 1·80)	0·927 (0·582 to 1·37)	1·09 (0·315 to 2·04)	1·16 (0·940 to 1·48)	0·587 (0·152 to 1·15)	-62·8 (-89·9 to -33·1)
	70+ years	4·39 (3·56 to 5·38)	4·27 (3·56 to 5·19)	3·54 (2·68 to 4·78)	3·32 (1·10 to 5·22)	2·98 (2·38 to 3·70)	1·91 (0·438 to 3·22)	-56·6 (-89·4 to -29·2)
Denmark	All Ages	24·6 (22·2 to 27·0)	20·5 (18·2 to 22·1)	16·2 (12·5 to 19·3)	15·8 (5·05 to 25·9)	18·3 (16·0 to 20·0)	11·3 (3·41 to 18·6)	-54·1 (-85·4 to -19·5)
	15-49 years	2·13 (1·75 to 2·60)	1·66 (1·38 to 2·06)	1·42 (1·06 to 1·83)	1·43 (0·799 to 2·14)	1·64 (1·36 to 2·03)	1·25 (0·750 to 1·77)	-41·3 (-61·5 to -19·3)
	50-69 years	6·42 (5·83 to 7·11)	5·13 (4·63 to 5·72)	3·68 (2·58 to 4·77)	3·93 (1·29 to 6·66)	4·69 (4·20 to 5·23)	2·67 (0·848 to 4·62)	-58·4 (-85·9 to -24·9)
	70+ years	16·0 (13·8 to 17·5)	13·7 (11·6 to 14·9)	11·0 (7·96 to 13·1)	10·4 (2·89 to 17·0)	11·9 (10·1 to 13·4)	7·37 (1·84 to 12·4)	-54·2 (-88·8 to -18·0)
Finland	All Ages	55·6 (47·1 to 60·2)	48·5 (41·3 to 52·8)	43·8 (36·3 to 49·4)	39·1 (16·1 to 57·6)	37·7 (31·8 to 42·7)	27·7 (10·8 to 41·1)	-50·2 (-80·6 to -25·6)
	15-49 years	4·12 (3·61 to 4·51)	3·91 (3·40 to 4·27)	3·33 (2·71 to 3·84)	3·26 (1·63 to 4·73)	3·79 (3·30 to 4·16)	2·80 (1·53 to 4·01)	-32·1 (-60·8 to -2·50)
	50-69 years	10·6 (9·87 to 11·5)	8·42 (7·75 to 9·19)	6·64 (5·03 to 8·07)	6·44 (1·99 to 10·5)	6·93 (6·20 to 7·65)	4·27 (1·30 to 7·39)	-59·8 (-86·8 to -31·6)
	70+ years	40·7 (33·0 to 45·2)	36·1 (29·1 to 39·8)	33·8 (27·2 to 38·0)	29·3 (12·4 to 43·1)	26·9 (21·7 to 31·2)	20·5 (7·81 to 30·8)	-49·6 (-80·5 to -25·7)
France	All Ages	942 (822 to 1050)	821 (709 to 913)	748 (610 to 838)	658 (271 to 1010)	750 (633 to 850)	556 (233 to 851)	-41·0 (-75·0 to -12·1)
	15-49 years	99·2 (77·6 to 130)	84·4 (64·3 to 112)	75·0 (55·1 to 99·3)	75·4 (43·9 to 110)	83·5 (63·6 to 111)	68·5 (42·0 to 95·3)	-31·2 (-48·6 to -15·3)
	50-69 years	174 (147 to 215)	139 (115 to 172)	119 (89·8 to 148)	119 (65·5 to 178)	132 (110 to 166)	101 (60·5 to 145)	-41·8 (-63·4 to -20·8)
	70+ years	665 (560 to 731)	595 (489 to 654)	551 (435 to 617)	461 (149 to 750)	531 (443 to 592)	383 (120 to 620)	-42·5 (-82·8 to -8·47)
Germany	All Ages	504 (453 to 549)	505 (439 to 557)	434 (350 to 497)	385 (131 to 639)	395 (351 to 452)	264 (92·0 to 436)	-47·6 (-81·0 to -9·15)
	15-49 years	52·1 (43·1 to 65·1)	43·4 (35·2 to 54·8)	36·0 (26·6 to 46·2)	36·3 (18·6 to 60·2)	42·5 (34·4 to 53·9)	30·9 (17·5 to 48·4)	-40·8 (-63·9 to -12·8)
	50-69 years	121 (108 to 139)	115 (99·3 to 133)	87·7 (66·0 to 109)	88·5 (31·6 to 153)	98·0 (84·3 to 119)	61·7 (24·7 to 109)	-49·2 (-78·2 to -11·2)
	70+ years	330 (284 to 364)	346 (297 to 381)	309 (238 to 353)	259 (75·4 to 433)	253 (212 to 291)	171 (48·4 to 292)	-48·2 (-85·2 to -8·12)
Greece	All Ages	118 (105 to 126)	112 (102 to 121)	90·8 (70·2 to 106)	91·3 (42·1 to 133)	97·0 (86·8 to 106)	65·8 (31·3 to 97·2)	-44·4 (-73·2 to -15·2)

eTable 4. Risk deleted deaths due to all-form tuberculosis for alcohol use, smoking, and diabetes, and all three risk factors combined by age groups for 204 countries and territories (2020) with percent change between 2015 observed deaths and 2020 all-risk deleted deaths.

Location	Age group	2015 Observed deaths	2020 Observed deaths	2020 Smoking deleted deaths	2020 Alcohol deleted deaths	2020 Diabetes deleted deaths	2020 All-risk deleted deaths	Percent Change
	15-49 years	15·1 (13·5 to 16·5)	11·6 (10·6 to 12·8)	9·74 (8·51 to 11·4)	10·3 (7·49 to 13·8)	11·5 (10·4 to 12·6)	8·87 (7·06 to 11·5)	-41·1 (-54·5 to -22·6)
	50-69 years	22·7 (21·0 to 24·5)	22·7 (20·9 to 25·3)	15·6 (11·2 to 21·1)	17·9 (7·49 to 28·0)	20·2 (18·5 to 23·0)	11·5 (5·62 to 19·4)	-49·2 (-75·6 to -15·7)
	70+ years	80·1 (67·9 to 87·6)	77·6 (67·3 to 84·9)	65·3 (48·6 to 76·1)	63·0 (26·8 to 93·1)	65·2 (55·9 to 72·3)	45·2 (18·2 to 68·3)	-43·6 (-76·8 to -12·0)
Iceland	All Ages	1·64 (1·41 to 1·84)	1·53 (1·32 to 1·73)	1·32 (1·02 to 1·52)	1·21 (0·411 to 1·89)	1·31 (1·12 to 1·51)	0·899 (0·266 to 1·40)	-45·4 (-83·5 to -15·5)
	15-49 years	0·183 (0·145 to 0·248)	0·207 (0·160 to 0·278)	0·190 (0·142 to 0·254)	0·189 (0·119 to 0·268)	0·205 (0·159 to 0·276)	0·175 (0·114 to 0·239)	-4·20 (-24·6 to 12·7)
	50-69 years	0·314 (0·281 to 0·344)	0·295 (0·271 to 0·328)	0·225 (0·170 to 0·277)	0·225 (0·0646 to 0·379)	0·260 (0·237 to 0·296)	0·154 (0·0396 to 0·268)	-51·1 (-86·7 to -15·5)
	70+ years	1·14 (0·933 to 1·29)	1·02 (0·833 to 1·17)	0·898 (0·653 to 1·05)	0·794 (0·224 to 1·29)	0·836 (0·682 to 0·970)	0·564 (0·108 to 0·917)	-50·6 (-90·6 to -20·6)
Ireland	All Ages	29·7 (26·7 to 31·8)	25·5 (22·5 to 27·8)	21·9 (17·7 to 24·9)	20·5 (8·58 to 30·3)	22·1 (18·9 to 24·2)	15·6 (6·43 to 23·0)	-47·5 (-77·6 to -20·8)
	15-49 years	5·36 (4·43 to 6·01)	4·33 (3·56 to 4·87)	4·00 (3·13 to 4·58)	3·92 (2·80 to 4·96)	4·28 (3·52 to 4·82)	3·67 (2·75 to 4·55)	-31·7 (-46·0 to -18·1)
	50-69 years	6·57 (6·03 to 7·08)	5·33 (4·86 to 5·76)	4·17 (3·26 to 5·11)	4·13 (1·54 to 6·91)	4·79 (4·36 to 5·29)	3·00 (1·13 to 5·06)	-54·4 (-82·4 to -24·0)
	70+ years	17·6 (14·8 to 19·1)	15·7 (13·3 to 17·7)	13·6 (10·0 to 15·8)	12·3 (4·44 to 18·7)	12·9 (11·1 to 14·8)	8·81 (2·73 to 13·9)	-50·0 (-84·8 to -21·1)
Israel	All Ages	30·3 (27·1 to 33·3)	29·6 (26·6 to 32·4)	25·8 (21·6 to 29·2)	26·6 (17·4 to 33·5)	22·6 (19·6 to 26·0)	17·9 (10·9 to 23·7)	-40·9 (-62·3 to -22·8)
	15-49 years	3·89 (3·07 to 4·92)	3·51 (2·81 to 4·44)	3·05 (2·27 to 3·84)	3·18 (2·03 to 4·38)	3·43 (2·75 to 4·38)	2·77 (1·85 to 3·79)	-29·1 (-49·8 to -13·3)
	50-69 years	7·24 (6·62 to 8·01)	6·25 (5·68 to 6·89)	4·73 (3·63 to 5·94)	5·32 (2·77 to 7·30)	4·88 (4·34 to 5·58)	3·20 (1·51 to 4·72)	-55·7 (-78·2 to -36·8)
	70+ years	18·9 (15·9 to 20·8)	19·7 (16·5 to 21·7)	17·9 (14·4 to 20·2)	17·9 (12·2 to 22·3)	14·1 (11·5 to 16·5)	11·7 (7·22 to 15·5)	-37·9 (-61·2 to -21·7)
Italy	All Ages	388 (351 to 416)	305 (272 to 327)	267 (212 to 298)	238 (85·5 to 382)	259 (234 to 292)	179 (65·0 to 289)	-53·9 (-83·2 to -24·1)
	15-49 years	38·8 (33·9 to 45·7)	22·2 (18·7 to 27·1)	18·3 (14·1 to 22·2)	18·2 (8·35 to 29·4)	21·8 (18·2 to 26·7)	15·2 (7·61 to 23·7)	-60·8 (-80·0 to -40·7)
	50-69 years	72·4 (66·3 to 80·2)	52·5 (49·0 to 57·0)	39·9 (31·0 to 49·3)	40·4 (14·4 to 69·9)	46·3 (42·0 to 51·0)	28·0 (9·99 to 48·9)	-61·3 (-85·4 to -31·2)
	70+ years	276 (237 to 301)	230 (193 to 249)	208 (169 to 233)	179 (62·7 to 287)	191 (162 to 218)	135 (47·1 to 216)	-51·1 (-83·3 to -20·5)
Luxembourg	All Ages	1·62 (1·44 to 1·81)	1·47 (1·31 to 1·64)	1·27 (1·02 to 1·47)	1·13 (0·356 to 1·90)	1·10 (0·966 to 1·29)	0·733 (0·212 to 1·30)	-54·9 (-86·5 to -21·6)
	15-49 years	0·194 (0·151 to 0·265)	0·154 (0·116 to 0·215)	0·135 (0·0947 to 0·186)	0·135 (0·0727 to 0·205)	0·151 (0·113 to 0·212)	0·120 (0·0687 to 0·177)	-38·8 (-59·4 to -19·2)
	50-69 years	0·354 (0·311 to 0·394)	0·292 (0·259 to 0·330)	0·217 (0·151 to 0·274)	0·221 (0·0624 to 0·403)	0·239 (0·201 to 0·281)	0·139 (0·0381 to 0·255)	-60·6 (-88·7 to -25·7)

eTable 4. Risk deleted deaths due to all-form tuberculosis for alcohol use, smoking, and diabetes, and all three risk factors combined by age groups for 204 countries and territories (2020) with percent change between 2015 observed deaths and 2020 all-risk deleted deaths.

Location	Age group	2015 Observed deaths	2020 Observed deaths	2020 Smoking deleted deaths	2020 Alcohol deleted deaths	2020 Diabetes deleted deaths	2020 All-risk deleted deaths	Percent Change
	70+ years	1·07 (0·924 to 1·19)	1·02 (0·874 to 1·14)	0·911 (0·723 to 1·05)	0·766 (0·195 to 1·31)	0·711 (0·599 to 0·855)	0·471 (0·0723 to 0·870)	-56·1 (-92·9 to -20·9)
Malta	All Ages	0·926 (0·846 to 1·01)	0·767 (0·696 to 0·860)	0·660 (0·541 to 0·773)	0·611 (0·226 to 0·939)	0·586 (0·508 to 0·677)	0·401 (0·122 to 0·673)	-56·8 (-86·2 to -29·3)
	15-49 years	0·116 (0·0926 to 0·151)	0·100 (0·0815 to 0·129)	0·0847 (0·0623 to 0·112)	0·0873 (0·0472 to 0·127)	0·0982 (0·0793 to 0·127)	0·0754 (0·0441 to 0·109)	-35·0 (-57·1 to -12·7)
	50-69 years	0·230 (0·207 to 0·256)	0·155 (0·140 to 0·180)	0·111 (0·0736 to 0·144)	0·118 (0·0353 to 0·199)	0·126 (0·111 to 0·148)	0·0700 (0·0153 to 0·130)	-69·5 (-93·0 to -44·6)
	70+ years	0·578 (0·512 to 0·630)	0·510 (0·435 to 0·573)	0·461 (0·366 to 0·529)	0·403 (0·134 to 0·628)	0·359 (0·298 to 0·424)	0·252 (0·0542 to 0·433)	-56·5 (-90·5 to -28·4)
Monaco	All Ages	0·623 (0·523 to 0·761)	0·587 (0·477 to 0·727)	0·494 (0·376 to 0·632)	0·487 (0·177 to 0·793)	0·511 (0·408 to 0·637)	0·362 (0·132 to 0·591)	-41·9 (-79·2 to -11·7)
	15-49 years	0·0484 (0·0365 to 0·0619)	0·0396 (0·0284 to 0·0511)	0·0309 (0·0221 to 0·0422)	0·0334 (0·0180 to 0·0554)	0·0385 (0·0276 to 0·0498)	0·0263 (0·0154 to 0·0427)	-45·7 (-66·1 to -19·6)
	50-69 years	0·129 (0·0995 to 0·165)	0·119 (0·0923 to 0·153)	0·0872 (0·0602 to 0·125)	0·0971 (0·0403 to 0·156)	0·107 (0·0826 to 0·137)	0·0665 (0·0300 to 0·112)	-48·2 (-75·5 to -15·8)
	70+ years	0·445 (0·368 to 0·567)	0·428 (0·340 to 0·541)	0·375 (0·285 to 0·491)	0·356 (0·116 to 0·582)	0·365 (0·281 to 0·467)	0·268 (0·0839 to 0·447)	-39·7 (-81·7 to -11·6)
Netherlands	All Ages	108 (94·2 to 121)	91·1 (80·7 to 103)	79·1 (66·2 to 91·0)	73·2 (32·0 to 114)	80·2 (70·7 to 90·6)	57·4 (25·1 to 88·0)	-46·7 (-74·9 to -17·3)
	15-49 years	12·2 (8·97 to 16·9)	9·81 (7·23 to 13·7)	9·05 (6·47 to 12·6)	9·03 (5·52 to 13·2)	9·72 (7·15 to 13·6)	8·44 (5·34 to 11·9)	-31·3 (-45·3 to -18·5)
	50-69 years	24·8 (21·4 to 30·0)	19·7 (16·8 to 23·5)	16·0 (12·2 to 19·8)	16·0 (6·80 to 26·5)	18·1 (15·0 to 21·7)	12·6 (5·60 to 19·7)	-49·5 (-75·0 to -21·4)
	70+ years	70·2 (59·3 to 76·8)	61·2 (51·4 to 66·4)	53·7 (40·7 to 62·3)	47·8 (18·0 to 76·1)	52·1 (43·5 to 58·0)	36·1 (13·2 to 56·0)	-48·7 (-81·2 to -16·3)
Norway	All Ages	44·3 (38·7 to 48·1)	45·2 (39·7 to 49·2)	40·8 (33·4 to 46·8)	37·1 (17·8 to 53·3)	38·7 (33·4 to 43·1)	29·0 (13·2 to 42·2)	-34·7 (-69·1 to -3·99)
	15-49 years	2·37 (1·73 to 3·14)	2·36 (1·78 to 3·06)	2·19 (1·59 to 2·86)	2·20 (1·39 to 3·02)	2·34 (1·75 to 3·04)	2·06 (1·35 to 2·78)	-13·1 (-28·6 to 0·714)
	50-69 years	6·47 (6·04 to 7·07)	6·12 (5·56 to 6·70)	5·10 (4·20 to 5·98)	4·95 (2·30 to 7·40)	5·50 (4·94 to 6·18)	3·82 (1·76 to 5·91)	-41·1 (-70·7 to -7·17)
	70+ years	35·4 (29·9 to 38·6)	36·6 (31·0 to 40·1)	33·5 (26·6 to 38·2)	29·9 (13·4 to 43·0)	30·8 (26·0 to 34·4)	23·0 (9·60 to 34·0)	-35·0 (-72·3 to -3·89)
Portugal	All Ages	309 (272 to 353)	271 (240 to 310)	247 (209 to 282)	221 (104 to 335)	222 (191 to 260)	170 (85·0 to 260)	-45·0 (-70·8 to -17·6)
	15-49 years	64·4 (47·5 to 88·8)	50·7 (35·9 to 69·9)	46·9 (32·3 to 64·5)	47·3 (30·9 to 69·3)	50·1 (35·4 to 69·3)	44·4 (29·5 to 64·2)	-31·3 (-41·8 to -20·0)
	50-69 years	83·2 (71·7 to 102)	68·6 (57·8 to 82·7)	56·6 (43·5 to 69·8)	55·5 (26·1 to 90·2)	59·5 (48·4 to 72·1)	42·4 (23·9 to 66·5)	-49·2 (-70·8 to -20·8)
	70+ years	161 (143 to 177)	151 (132 to 169)	143 (121 to 161)	117 (40·7 to 188)	112 (93·9 to 130)	82·6 (26·1 to 135)	-48·5 (-83·3 to -16·7)
San Marino	All Ages	0·129 (0·102 to 0·167)	0·128 (0·0898 to 0·169)	0·114 (0·0801 to 0·155)	0·105 (0·0340 to 0·176)	0·110 (0·0766 to 0·146)	0·0814 (0·0269 to 0·143)	-37·6 (-79·4 to -0·684)

eTable 4. Risk deleted deaths due to all-form tuberculosis for alcohol use, smoking, and diabetes, and all three risk factors combined by age groups for 204 countries and territories (2020) with percent change between 2015 observed deaths and 2020 all-risk deleted deaths.

Location	Age group	2015 Observed deaths	2020 Observed deaths	2020 Smoking deleted deaths	2020 Alcohol deleted deaths	2020 Diabetes deleted deaths	2020 All-risk deleted deaths	Percent Change
	15-49 years	0·00849 (0·00668 to 0·0105)	0·00696 (0·00505 to 0·00897)	0·00561 (0·00359 to 0·00798)	0·00576 (0·00195 to 0·0101)	0·00678 (0·00487 to 0·00880)	0·00465 (0·00164 to 0·00839)	-45·6 (-78·2 to -10·1)
	50-69 years	0·0210 (0·0162 to 0·0278)	0·0194 (0·0131 to 0·0269)	0·0149 (0·00869 to 0·0214)	0·0153 (0·00382 to 0·0284)	0·0172 (0·0115 to 0·0238)	0·0106 (0·00265 to 0·0202)	-49·6 (-87·2 to -0·256)
	70+ years	0·0997 (0·0759 to 0·131)	0·102 (0·0680 to 0·134)	0·0937 (0·0643 to 0·128)	0·0836 (0·0275 to 0·141)	0·0862 (0·0570 to 0·116)	0·0659 (0·0212 to 0·116)	-34·4 (-79·0 to 2·26)
Spain	All Ages	435 (395 to 493)	361 (322 to 401)	317 (264 to 364)	295 (133 to 444)	280 (243 to 333)	207 (93·2 to 317)	-52·6 (-77·4 to -28·3)
	15-49 years	56·4 (42·3 to 76·1)	43·8 (31·7 to 60·5)	39·3 (27·3 to 54·7)	39·9 (24·3 to 60·1)	43·2 (31·2 to 59·8)	36·4 (22·6 to 53·2)	-35·7 (-48·7 to -20·0)
	50-69 years	103 (89·9 to 123)	75·9 (63·7 to 88·5)	59·9 (44·4 to 72·8)	61·6 (27·3 to 97·5)	65·3 (53·9 to 77·9)	44·5 (23·6 to 70·5)	-56·9 (-76·2 to -33·4)
	70+ years	274 (237 to 301)	240 (202 to 264)	217 (173 to 245)	192 (77·9 to 288)	170 (145 to 208)	124 (45·8 to 199)	-54·6 (-82·5 to -29·2)
Sweden	All Ages	72·9 (64·4 to 80·3)	60·2 (53·3 to 65·3)	52·6 (41·6 to 60·4)	48·4 (22·2 to 71·6)	50·1 (44·4 to 56·4)	35·9 (15·7 to 54·2)	-50·8 (-78·1 to -22·8)
	15-49 years	3·12 (2·09 to 4·42)	2·41 (1·57 to 3·43)	2·34 (1·50 to 3·32)	2·31 (1·44 to 3·32)	2·40 (1·56 to 3·42)	2·25 (1·40 to 3·22)	-28·2 (-38·3 to -19·7)
	50-69 years	8·18 (7·27 to 9·24)	5·91 (5·23 to 6·79)	4·64 (3·43 to 5·77)	4·72 (1·97 to 7·37)	5·24 (4·44 to 6·08)	3·42 (1·40 to 5·61)	-58·2 (-81·3 to -31·4)
	70+ years	61·4 (52·4 to 67·4)	51·7 (44·6 to 56·2)	45·5 (35·3 to 52·4)	41·3 (18·4 to 62·0)	42·4 (36·6 to 47·7)	30·1 (12·1 to 45·5)	-51·0 (-80·2 to -22·3)
Switzerland	All Ages	39·8 (35·3 to 43·3)	34·5 (30·2 to 38·1)	29·7 (24·2 to 34·5)	27·4 (10·8 to 42·2)	27·1 (23·6 to 31·2)	18·9 (7·37 to 30·2)	-52·6 (-80·7 to -23·9)
	15-49 years	4·14 (3·16 to 5·43)	3·03 (2·24 to 4·15)	2·69 (1·88 to 3·69)	2·70 (1·58 to 4·10)	2·98 (2·19 to 4·11)	2·44 (1·50 to 3·55)	-41·4 (-56·3 to -24·0)
	50-69 years	8·70 (7·83 to 10·1)	5·83 (5·05 to 7·04)	4·44 (3·28 to 5·58)	4·47 (1·50 to 7·85)	4·85 (4·14 to 6·03)	2·96 (0·970 to 5·24)	-66·0 (-88·1 to -41·0)
	70+ years	26·9 (22·8 to 29·2)	25·6 (21·1 to 28·1)	22·4 (17·0 to 25·5)	20·1 (7·95 to 31·5)	19·1 (15·8 to 22·0)	13·4 (5·11 to 21·6)	-50·2 (-80·4 to -18·0)
United Kingdom	All Ages	462 (424 to 496)	321 (294 to 344)	275 (232 to 312)	253 (100 to 402)	262 (239 to 294)	180 (68·7 to 287)	-61·0 (-84·5 to -35·2)
	15-49 years	69·3 (57·0 to 86·7)	43·2 (34·5 to 55·2)	37·4 (28·2 to 48·2)	36·6 (19·2 to 57·0)	42·3 (33·7 to 54·4)	32·1 (18·0 to 48·1)	-53·9 (-72·1 to -34·1)
	50-69 years	112 (104 to 122)	76·2 (71·2 to 83·5)	60·1 (47·5 to 71·8)	58·8 (20·8 to 102)	65·1 (58·8 to 72·5)	40·9 (14·0 to 70·8)	-63·3 (-86·8 to -35·5)
	70+ years	278 (249 to 294)	200 (179 to 212)	176 (143 to 197)	155 (57·6 to 248)	153 (137 to 172)	105 (35·3 to 170)	-62·2 (-87·2 to -35·9)
Latin America and Caribbean	All Ages	25800 (22600 to 31200)	24700 (21200 to 29200)	22400 (18700 to 27200)	21300 (12900 to 29600)	22100 (18500 to 26800)	17600 (10700 to 24500)	-31·6 (-56·4 to -9·35)
	15-49 years	11400 (9440 to 14200)	10300 (8370 to 12800)	9550 (7610 to 12100)	8820 (5350 to 12900)	10100 (8180 to 12500)	8170 (5190 to 11700)	-28·4 (-54·5 to -4·74)
	50-69 years	7960 (7130 to 9610)	8140 (7040 to 9660)	7070 (5800 to 8740)	6890 (3990 to 9720)	7050 (5930 to 8510)	5320 (3080 to 7570)	-33·2 (-59·6 to -9·66)

eTable 4. Risk deleted deaths due to all-form tuberculosis for alcohol use, smoking, and diabetes, and all three risk factors combined by age groups for 204 countries and territories (2020) with percent change between 2015 observed deaths and 2020 all-risk deleted deaths.

Location	Age group	2015 Observed deaths	2020 Observed deaths	2020 Smoking deleted deaths	2020 Alcohol deleted deaths	2020 Diabetes deleted deaths	2020 All-risk deleted deaths	Percent Change
	70+ years	5290 (4810 to 5960)	5390 (4830 to 6100)	4930 (4230 to 5690)	4700 (2780 to 6300)	4090 (3530 to 4810)	3280 (1830 to 4510)	-38·1 (-63·9 to -19·3)
Andean Latin America	All Ages	5200 (4510 to 6100)	4880 (4030 to 6040)	4490 (3580 to 5590)	4080 (2220 to 6400)	4360 (3520 to 5350)	3380 (1830 to 5180)	-35·1 (-64·6 to -3·09)
	15-49 years	2050 (1700 to 2430)	1850 (1500 to 2320)	1720 (1350 to 2160)	1510 (732 to 2490)	1830 (1480 to 2300)	1400 (697 to 2250)	-31·6 (-65·2 to 8·65)
	50-69 years	1450 (1240 to 1800)	1440 (1150 to 1810)	1270 (970 to 1610)	1180 (545 to 1920)	1280 (989 to 1600)	932 (442 to 1500)	-35·8 (-67·8 to -2·55)
	70+ years	1440 (1260 to 1730)	1410 (1160 to 1810)	1320 (1060 to 1670)	1220 (644 to 1810)	1080 (871 to 1410)	866 (441 to 1290)	-40·3 (-68·2 to -20·4)
Bolivia (Plurinational State of)	All Ages	1780 (1160 to 2620)	1550 (1070 to 2120)	1420 (947 to 1920)	1310 (645 to 2130)	1330 (905 to 1800)	1040 (515 to 1690)	-41·6 (-66·2 to -16·4)
	15-49 years	570 (354 to 823)	483 (324 to 685)	452 (300 to 640)	396 (185 to 734)	474 (318 to 671)	367 (172 to 671)	-35·5 (-67·1 to 4·77)
	50-69 years	575 (384 to 854)	512 (352 to 679)	444 (290 to 591)	423 (182 to 719)	426 (293 to 578)	308 (131 to 525)	-46·4 (-73·6 to -19·0)
	70+ years	532 (366 to 752)	485 (342 to 684)	459 (315 to 649)	425 (206 to 645)	358 (250 to 508)	299 (146 to 455)	-43·8 (-65·5 to -26·6)
Ecuador	All Ages	1110 (947 to 1290)	652 (545 to 797)	612 (499 to 742)	579 (361 to 763)	585 (487 to 701)	492 (312 to 653)	-55·6 (-70·4 to -45·5)
	15-49 years	531 (423 to 653)	303 (241 to 386)	289 (229 to 361)	262 (157 to 369)	300 (240 to 381)	250 (154 to 349)	-53·0 (-70·6 to -39·4)
	50-69 years	267 (232 to 310)	161 (133 to 197)	145 (115 to 180)	141 (85·9 to 189)	142 (116 to 176)	116 (72·7 to 153)	-56·8 (-72·1 to -45·4)
	70+ years	266 (233 to 290)	170 (142 to 197)	159 (133 to 191)	156 (108 to 195)	124 (101 to 152)	106 (66·5 to 137)	-60·0 (-75·0 to -47·9)
Peru	All Ages	2310 (1940 to 2660)	2680 (2010 to 3560)	2450 (1900 to 3260)	2190 (1010 to 3720)	2450 (1840 to 3230)	1840 (859 to 3140)	-19·9 (-61·2 to 24·1)
	15-49 years	945 (782 to 1130)	1060 (800 to 1340)	979 (753 to 1240)	850 (357 to 1550)	1050 (792 to 1330)	784 (336 to 1410)	-16·9 (-61·9 to 36·3)
	50-69 years	607 (494 to 708)	770 (549 to 1030)	686 (504 to 929)	611 (261 to 1060)	708 (503 to 945)	508 (219 to 878)	-15·9 (-61·9 to 33·0)
	70+ years	646 (543 to 786)	758 (578 to 1010)	698 (532 to 955)	637 (304 to 992)	595 (437 to 771)	461 (201 to 747)	-28·6 (-68·6 to 5·35)
Caribbean	All Ages	4060 (2780 to 8610)	3490 (2340 to 8060)	3260 (2210 to 7520)	3060 (1730 to 7670)	3220 (2150 to 7300)	2680 (1550 to 6590)	-34·1 (-54·8 to -15·6)
	15-49 years	2030 (1340 to 4280)	1590 (1040 to 3780)	1500 (966 to 3560)	1370 (728 to 3730)	1560 (1020 to 3720)	1290 (698 to 3390)	-36·8 (-60·2 to -14·7)
	50-69 years	1030 (675 to 2690)	989 (644 to 2560)	897 (581 to 2410)	844 (435 to 2270)	862 (559 to 2240)	682 (360 to 1790)	-34·0 (-57·2 to -11·2)
	70+ years	569 (401 to 1250)	564 (388 to 1240)	519 (360 to 1150)	492 (250 to 1130)	445 (301 to 935)	360 (176 to 794)	-36·3 (-61·4 to -14·5)
Antigua and Barbuda	All Ages	0·475 (0·424 to 0·554)	0·375 (0·318 to 0·433)	0·340 (0·286 to 0·398)	0·319 (0·173 to 0·455)	0·312 (0·255 to 0·368)	0·246 (0·136 to 0·340)	-48·3 (-69·5 to -27·9)

eTable 4. Risk deleted deaths due to all-form tuberculosis for alcohol use, smoking, and diabetes, and all three risk factors combined by age groups for 204 countries and territories (2020) with percent change between 2015 observed deaths and 2020 all-risk deleted deaths.

Location	Age group	2015 Observed deaths	2020 Observed deaths	2020 Smoking deleted deaths	2020 Alcohol deleted deaths	2020 Diabetes deleted deaths	2020 All-risk deleted deaths	Percent Change
	15-49 years	0·186 (0·154 to 0·230)	0·136 (0·108 to 0·174)	0·127 (0·101 to 0·159)	0·120 (0·0759 to 0·171)	0·133 (0·106 to 0·172)	0·113 (0·0738 to 0·155)	-39·4 (-56·8 to -22·7)
	50-69 years	0·157 (0·138 to 0·185)	0·130 (0·109 to 0·151)	0·112 (0·0886 to 0·136)	0·106 (0·0486 to 0·160)	0·104 (0·0838 to 0·121)	0·0747 (0·0343 to 0·116)	-52·3 (-77·4 to -28·0)
	70+ years	0·125 (0·115 to 0·136)	0·105 (0·0939 to 0·117)	0·0965 (0·0850 to 0·111)	0·0878 (0·0457 to 0·121)	0·0709 (0·0583 to 0·0863)	0·0541 (0·0244 to 0·0823)	-56·6 (-81·0 to -34·8)
Bahamas	All Ages	9·70 (7·95 to 12·1)	9·66 (7·71 to 12·6)	8·96 (6·94 to 11·6)	8·62 (5·81 to 12·2)	8·77 (6·76 to 11·5)	7·42 (5·21 to 10·4)	-23·6 (-45·3 to -6·19)
	15-49 years	5·48 (4·26 to 7·27)	5·23 (3·99 to 7·07)	4·93 (3·67 to 6·75)	4·72 (3·14 to 6·68)	5·14 (3·89 to 6·98)	4·44 (3·03 to 6·32)	-19·2 (-38·4 to -0·980)
	50-69 years	2·73 (2·25 to 3·29)	2·94 (2·37 to 3·81)	2·64 (2·00 to 3·31)	2·58 (1·60 to 3·79)	2·54 (1·99 to 3·30)	2·08 (1·41 to 3·00)	-24·0 (-47·2 to -5·75)
	70+ years	1·37 (1·18 to 1·49)	1·37 (1·19 to 1·64)	1·27 (1·08 to 1·50)	1·21 (0·716 to 1·66)	0·976 (0·784 to 1·17)	0·799 (0·462 to 1·09)	-41·4 (-66·4 to -23·4)
Barbados	All Ages	1·92 (1·62 to 2·23)	1·92 (1·55 to 2·36)	1·78 (1·36 to 2·18)	1·61 (0·830 to 2·48)	1·56 (1·22 to 1·98)	1·24 (0·651 to 1·86)	-35·9 (-64·4 to -9·83)
	15-49 years	0·637 (0·498 to 0·803)	0·579 (0·434 to 0·772)	0·545 (0·404 to 0·737)	0·501 (0·287 to 0·749)	0·565 (0·423 to 0·760)	0·469 (0·271 to 0·687)	-26·5 (-50·2 to -2·70)
	50-69 years	0·651 (0·554 to 0·754)	0·669 (0·522 to 0·832)	0·615 (0·471 to 0·778)	0·556 (0·273 to 0·895)	0·547 (0·413 to 0·691)	0·429 (0·226 to 0·668)	-34·2 (-63·4 to -5·93)
	70+ years	0·619 (0·546 to 0·674)	0·662 (0·529 to 0·823)	0·603 (0·470 to 0·763)	0·544 (0·212 to 0·815)	0·439 (0·318 to 0·570)	0·325 (0·0998 to 0·510)	-47·4 (-83·3 to -18·0)
Belize	All Ages	19·0 (15·9 to 23·7)	17·2 (14·6 to 20·9)	15·7 (12·8 to 19·4)	15·0 (9·39 to 20·5)	15·7 (12·9 to 19·3)	12·8 (8·41 to 17·3)	-32·5 (-54·7 to -14·4)
	15-49 years	11·2 (8·79 to 14·6)	9·51 (7·51 to 12·3)	8·94 (6·88 to 11·8)	8·37 (5·25 to 11·7)	9·38 (7·37 to 12·2)	7·87 (5·06 to 11·1)	-29·8 (-49·4 to -10·8)
	50-69 years	4·73 (4·15 to 5·63)	4·63 (4·03 to 5·45)	3·99 (3·22 to 4·66)	3·97 (2·30 to 5·54)	3·98 (3·34 to 4·77)	3·03 (1·84 to 4·12)	-35·9 (-59·8 to -16·8)
	70+ years	2·45 (2·26 to 2·61)	2·55 (2·27 to 2·87)	2·29 (1·96 to 2·65)	2·21 (1·25 to 2·93)	1·87 (1·62 to 2·20)	1·46 (0·727 to 2·01)	-40·2 (-71·0 to -17·8)
Bermuda	All Ages	0·0635 (0·0544 to 0·0740)	0·0453 (0·0384 to 0·0540)	0·0420 (0·0352 to 0·0501)	0·0400 (0·0261 to 0·0547)	0·0396 (0·0331 to 0·0477)	0·0331 (0·0222 to 0·0440)	-47·8 (-64·7 to -34·9)
	15-49 years	0·0165 (0·0126 to 0·0221)	0·00980 (0·00696 to 0·0135)	0·00926 (0·00662 to 0·0127)	0·00894 (0·00573 to 0·0132)	0·00966 (0·00686 to 0·0134)	0·00847 (0·00543 to 0·0126)	-48·8 (-57·8 to -37·8)
	50-69 years	0·0196 (0·0162 to 0·0241)	0·0140 (0·0110 to 0·0175)	0·0125 (0·00973 to 0·0158)	0·0122 (0·00762 to 0·0175)	0·0127 (0·00994 to 0·0161)	0·0101 (0·00653 to 0·0141)	-48·2 (-65·7 to -32·2)
	70+ years	0·0268 (0·0227 to 0·0306)	0·0212 (0·0179 to 0·0243)	0·0199 (0·0168 to 0·0232)	0·0186 (0·0113 to 0·0246)	0·0170 (0·0144 to 0·0199)	0·0142 (0·00819 to 0·0188)	-47·0 (-67·9 to -32·7)
Cuba	All Ages	47·6 (44·3 to 52·1)	37·4 (34·0 to 41·0)	29·6 (23·7 to 35·2)	31·2 (15·4 to 44·4)	30·5 (26·9 to 34·4)	20·8 (10·6 to 30·5)	-56·2 (-78·0 to -38·4)
	15-49 years	12·9 (10·9 to 15·5)	8·50 (7·04 to 10·3)	7·16 (5·51 to 8·87)	7·26 (4·40 to 10·5)	8·21 (6·80 to 10·0)	6·12 (4·06 to 8·69)	-52·5 (-68·8 to -35·9)
	50-69 years	17·7 (16·0 to 19·5)	15·9 (14·0 to 17·8)	11·3 (7·93 to 14·4)	12·9 (5·38 to 19·7)	12·9 (11·2 to 14·7)	7·72 (3·25 to 12·4)	-56·5 (-81·8 to -32·3)

eTable 4. Risk deleted deaths due to all-form tuberculosis for alcohol use, smoking, and diabetes, and all three risk factors combined by age groups for 204 countries and territories (2020) with percent change between 2015 observed deaths and 2020 all-risk deleted deaths.

Location	Age group	2015 Observed deaths	2020 Observed deaths	2020 Smoking deleted deaths	2020 Alcohol deleted deaths	2020 Diabetes deleted deaths	2020 All-risk deleted deaths	Percent Change
	70+ years	16·6 (15·3 to 18·0)	12·8 (11·5 to 14·0)	11·0 (9·15 to 12·7)	10·9 (5·92 to 14·4)	9·15 (7·73 to 10·5)	6·81 (3·39 to 9·46)	-59·1 (-79·7 to -44·0)
Dominica	All Ages	3·06 (2·46 to 3·90)	2·94 (2·25 to 3·73)	2·70 (1·97 to 3·55)	2·48 (1·35 to 3·91)	2·38 (1·83 to 3·07)	1·88 (1·02 to 2·91)	-38·9 (-65·3 to -14·6)
	15-49 years	1·12 (0·838 to 1·53)	0·974 (0·711 to 1·35)	0·916 (0·640 to 1·27)	0·851 (0·459 to 1·32)	0·949 (0·696 to 1·32)	0·792 (0·438 to 1·23)	-29·6 (-53·8 to -6·74)
	50-69 years	1·08 (0·809 to 1·41)	1·12 (0·823 to 1·48)	1·00 (0·689 to 1·37)	0·933 (0·466 to 1·55)	0·907 (0·648 to 1·16)	0·695 (0·367 to 1·10)	-36·2 (-65·4 to -7·31)
	70+ years	0·771 (0·631 to 0·926)	0·779 (0·563 to 0·982)	0·724 (0·527 to 0·933)	0·627 (0·252 to 0·953)	0·463 (0·332 to 0·573)	0·328 (0·0562 to 0·551)	-57·6 (-93·1 to -32·3)
Dominican Republic	All Ages	1160 (857 to 1530)	900 (635 to 1390)	797 (567 to 1300)	777 (390 to 1360)	841 (590 to 1310)	652 (302 to 1130)	-44·3 (-66·4 to -22·5)
	15-49 years	605 (419 to 820)	372 (250 to 551)	339 (232 to 519)	306 (106 to 574)	366 (246 to 541)	277 (96·1 to 493)	-54·6 (-80·2 to -32·1)
	50-69 years	294 (210 to 414)	263 (176 to 420)	224 (147 to 381)	227 (121 to 405)	242 (159 to 383)	181 (88·6 to 321)	-38·8 (-60·2 to -11·0)
	70+ years	231 (176 to 314)	239 (167 to 377)	209 (146 to 330)	218 (125 to 367)	208 (149 to 325)	168 (96·0 to 284)	-28·0 (-52·2 to -0·274)
Grenada	All Ages	0·691 (0·613 to 0·785)	0·442 (0·390 to 0·497)	0·406 (0·347 to 0·460)	0·368 (0·186 to 0·515)	0·367 (0·318 to 0·423)	0·284 (0·144 to 0·394)	-58·9 (-78·1 to -43·9)
	15-49 years	0·274 (0·227 to 0·328)	0·154 (0·128 to 0·183)	0·143 (0·118 to 0·173)	0·131 (0·0705 to 0·188)	0·150 (0·124 to 0·180)	0·120 (0·0672 to 0·168)	-56·2 (-73·7 to -43·0)
	50-69 years	0·251 (0·219 to 0·287)	0·163 (0·140 to 0·187)	0·144 (0·123 to 0·169)	0·134 (0·0645 to 0·186)	0·133 (0·112 to 0·155)	0·0998 (0·0510 to 0·137)	-60·1 (-79·2 to -44·7)
	70+ years	0·153 (0·142 to 0·168)	0·118 (0·107 to 0·132)	0·111 (0·0977 to 0·125)	0·0954 (0·0367 to 0·134)	0·0769 (0·0648 to 0·0916)	0·0571 (0·0169 to 0·0862)	-62·6 (-89·0 to -43·9)
Guyana	All Ages	107 (85·1 to 131)	79·7 (63·5 to 98·4)	72·0 (55·9 to 90·0)	68·6 (41·6 to 97·8)	70·5 (55·7 to 87·5)	56·5 (37·5 to 78·2)	-47·3 (-63·8 to -29·1)
	15-49 years	60·6 (47·0 to 77·3)	43·1 (33·1 to 55·4)	39·1 (30·4 to 50·6)	37·5 (23·5 to 53·7)	41·8 (32·1 to 54·3)	33·9 (22·1 to 47·0)	-44·0 (-60·1 to -25·5)
	50-69 years	33·0 (25·9 to 39·5)	25·8 (20·2 to 32·1)	22·7 (17·3 to 27·8)	21·6 (12·4 to 31·7)	20·6 (15·8 to 25·5)	15·7 (9·27 to 22·5)	-52·2 (-69·9 to -32·4)
	70+ years	11·0 (9·40 to 12·8)	9·01 (7·18 to 11·0)	8·40 (6·74 to 10·4)	7·69 (4·37 to 10·7)	6·30 (4·89 to 8·14)	5·05 (2·73 to 7·09)	-53·9 (-73·5 to -37·1)
Haiti	All Ages	2450 (1460 to 7000)	2220 (1290 to 6650)	2130 (1250 to 6290)	1960 (1100 to 6430)	2050 (1180 to 6000)	1760 (1010 to 5540)	-27·4 (-48·4 to -8·51)
	15-49 years	1210 (690 to 3440)	1050 (610 to 3220)	1010 (583 to 3020)	924 (481 to 3180)	1040 (598 to 3160)	880 (466 to 2910)	-26·8 (-50·7 to -5·56)
	50-69 years	606 (324 to 2270)	607 (333 to 2160)	571 (318 to 2050)	517 (243 to 1920)	521 (280 to 1880)	425 (210 to 1510)	-28·7 (-53·6 to -4·69)
	70+ years	258 (135 to 933)	255 (131 to 905)	247 (125 to 864)	214 (94·2 to 849)	186 (91·6 to 652)	152 (63·6 to 585)	-40·4 (-65·0 to -13·3)
Jamaica	All Ages	27·2 (21·6 to 34·6)	24·4 (18·3 to 31·4)	22·4 (16·8 to 28·8)	22·2 (14·9 to 30·6)	22·4 (16·6 to 29·3)	19·2 (13·1 to 27·1)	-29·6 (-45·4 to -9·92)

eTable 4. Risk deleted deaths due to all-form tuberculosis for alcohol use, smoking, and diabetes, and all three risk factors combined by age groups for 204 countries and territories (2020) with percent change between 2015 observed deaths and 2020 all-risk deleted deaths.

Location	Age group	2015 Observed deaths	2020 Observed deaths	2020 Smoking deleted deaths	2020 Alcohol deleted deaths	2020 Diabetes deleted deaths	2020 All-risk deleted deaths	Percent Change
	15-49 years	12·5 (8·84 to 17·6)	11·0 (7·83 to 15·7)	10·4 (7·39 to 14·9)	10·3 (6·77 to 15·0)	10·9 (7·78 to 15·6)	9·78 (6·53 to 14·4)	-21·9 (-33·4 to -6·09)
	50-69 years	8·06 (6·46 to 10·1)	7·59 (5·52 to 9·95)	6·71 (4·87 to 8·75)	6·80 (4·53 to 9·74)	6·90 (4·96 to 9·12)	5·65 (3·74 to 8·19)	-30·0 (-46·4 to -7·49)
	70+ years	6·22 (5·52 to 6·91)	5·53 (4·42 to 6·67)	5·00 (3·77 to 6·16)	4·83 (3·06 to 6·88)	4·33 (3·25 to 5·36)	3·43 (2·02 to 4·93)	-44·7 (-69·4 to -21·9)
Puerto Rico	All Ages	34·8 (30·0 to 41·5)	21·7 (18·4 to 26·1)	19·7 (16·0 to 23·5)	19·2 (11·8 to 25·3)	17·7 (14·6 to 21·9)	14·6 (9·70 to 19·5)	-58·0 (-72·8 to -46·9)
	15-49 years	10·5 (8·42 to 13·6)	6·24 (4·96 to 8·17)	5·76 (4·43 to 7·45)	5·53 (3·61 to 7·68)	6·03 (4·75 to 7·97)	5·06 (3·45 to 6·96)	-52·1 (-65·7 to -40·8)
	50-69 years	12·4 (10·3 to 15·4)	7·46 (6·13 to 9·36)	6·58 (5·01 to 8·26)	6·61 (4·21 to 9·01)	6·23 (4·85 to 8·13)	5·11 (3·58 to 6·95)	-58·8 (-71·5 to -48·4)
	70+ years	11·6 (10·4 to 12·6)	7·92 (7·07 to 8·78)	7·27 (6·20 to 8·10)	6·98 (4·16 to 8·98)	5·39 (4·59 to 6·33)	4·38 (2·54 to 5·85)	-62·3 (-78·2 to -49·7)
Saint Kitts and Nevis	All Ages	1·24 (1·03 to 1·50)	1·23 (0·986 to 1·48)	1·16 (0·926 to 1·38)	1·13 (0·718 to 1·48)	1·06 (0·839 to 1·32)	0·931 (0·618 to 1·23)	-24·8 (-47·7 to -8·89)
	15-49 years	0·488 (0·362 to 0·662)	0·454 (0·336 to 0·617)	0·437 (0·316 to 0·601)	0·428 (0·291 to 0·615)	0·446 (0·329 to 0·610)	0·409 (0·277 to 0·592)	-16·1 (-32·1 to -4·56)
	50-69 years	0·493 (0·405 to 0·571)	0·532 (0·433 to 0·611)	0·491 (0·396 to 0·575)	0·476 (0·281 to 0·628)	0·440 (0·350 to 0·521)	0·370 (0·219 to 0·491)	-24·9 (-51·8 to -4·91)
	70+ years	0·235 (0·214 to 0·253)	0·226 (0·198 to 0·253)	0·213 (0·184 to 0·242)	0·203 (0·119 to 0·250)	0·157 (0·132 to 0·183)	0·133 (0·0775 to 0·173)	-43·5 (-67·7 to -26·3)
Saint Lucia	All Ages	4·28 (3·81 to 4·83)	4·57 (3·88 to 5·35)	4·16 (3·45 to 4·98)	3·77 (1·98 to 5·74)	3·88 (3·26 to 4·53)	2·97 (1·62 to 4·50)	-30·6 (-61·1 to 1·62)
	15-49 years	1·77 (1·50 to 2·13)	1·69 (1·40 to 2·02)	1·54 (1·24 to 1·89)	1·39 (0·690 to 2·25)	1·64 (1·35 to 1·97)	1·25 (0·666 to 2·00)	-29·5 (-61·3 to 7·70)
	50-69 years	1·41 (1·25 to 1·58)	1·52 (1·26 to 1·78)	1·32 (1·06 to 1·61)	1·23 (0·585 to 1·89)	1·24 (1·03 to 1·50)	0·902 (0·478 to 1·38)	-36·2 (-66·8 to -4·41)
	70+ years	1·04 (0·953 to 1·14)	1·32 (1·15 to 1·52)	1·25 (1·07 to 1·44)	1·12 (0·602 to 1·58)	0·958 (0·790 to 1·14)	0·777 (0·411 to 1·10)	-25·6 (-59·5 to 5·45)
Saint Vincent and the Grenadines	All Ages	3·41 (2·87 to 4·10)	2·75 (2·33 to 3·35)	2·51 (2·10 to 3·12)	2·31 (1·32 to 3·41)	2·39 (1·92 to 2·98)	1·90 (1·20 to 2·70)	-44·5 (-64·4 to -22·5)
	15-49 years	1·60 (1·25 to 2·07)	1·16 (0·879 to 1·52)	1·08 (0·818 to 1·45)	1·01 (0·624 to 1·47)	1·13 (0·851 to 1·50)	0·943 (0·605 to 1·35)	-41·4 (-56·9 to -21·9)
	50-69 years	1·03 (0·871 to 1·24)	0·925 (0·779 to 1·12)	0·812 (0·655 to 0·981)	0·754 (0·388 to 1·17)	0·773 (0·632 to 0·971)	0·579 (0·344 to 0·853)	-44·0 (-67·8 to -18·5)
	70+ years	0·716 (0·651 to 0·787)	0·624 (0·563 to 0·686)	0·584 (0·509 to 0·656)	0·504 (0·220 to 0·740)	0·441 (0·374 to 0·508)	0·335 (0·148 to 0·501)	-53·2 (-79·5 to -30·1)
Suriname	All Ages	18·0 (13·7 to 23·0)	17·6 (13·5 to 22·6)	15·8 (11·4 to 20·8)	15·8 (9·85 to 22·7)	15·6 (11·7 to 20·6)	13·1 (8·22 to 18·7)	-27·2 (-44·8 to -7·38)
	15-49 years	9·41 (6·75 to 12·8)	8·61 (6·21 to 11·9)	7·98 (5·69 to 11·2)	7·90 (4·86 to 11·7)	8·41 (6·03 to 11·7)	7·31 (4·63 to 11·0)	-22·4 (-39·7 to -3·56)
	50-69 years	5·35 (4·19 to 6·74)	5·79 (4·36 to 7·56)	4·89 (3·49 to 6·52)	5·06 (3·03 to 7·35)	4·87 (3·64 to 6·45)	3·79 (2·29 to 5·64)	-29·3 (-48·5 to -5·29)

eTable 4. Risk deleted deaths due to all-form tuberculosis for alcohol use, smoking, and diabetes, and all three risk factors combined by age groups for 204 countries and territories (2020) with percent change between 2015 observed deaths and 2020 all-risk deleted deaths.

Location	Age group	2015 Observed deaths	2020 Observed deaths	2020 Smoking deleted deaths	2020 Alcohol deleted deaths	2020 Diabetes deleted deaths	2020 All-risk deleted deaths	Percent Change
	70+ years	2·66 (2·14 to 3·20)	2·66 (2·09 to 3·22)	2·43 (1·90 to 3·03)	2·35 (1·40 to 3·23)	1·87 (1·41 to 2·38)	1·52 (0·828 to 2·25)	-42·9 (-68·6 to -19·0)
Trinidad and Tobago	All Ages	27·0 (22·1 to 33·9)	26·7 (21·2 to 32·9)	24·1 (18·6 to 31·2)	23·4 (15·3 to 32·5)	23·6 (18·5 to 29·4)	19·3 (13·5 to 26·3)	-28·6 (-49·9 to -8·86)
	15-49 years	12·5 (9·46 to 17·1)	12·0 (8·87 to 16·0)	11·2 (8·13 to 15·1)	10·9 (7·08 to 15·4)	11·8 (8·70 to 15·8)	10·2 (6·65 to 14·4)	-18·9 (-36·1 to -1·34)
	50-69 years	9·99 (8·45 to 12·2)	9·79 (7·85 to 12·0)	8·31 (6·27 to 10·7)	8·29 (4·73 to 12·3)	8·17 (6·44 to 10·0)	6·19 (4·11 to 8·66)	-38·2 (-60·2 to -15·0)
	70+ years	4·10 (3·75 to 4·59)	4·64 (3·89 to 5·61)	4·24 (3·46 to 5·19)	3·96 (2·21 to 5·56)	3·32 (2·67 to 4·00)	2·62 (1·42 to 3·68)	-35·9 (-64·6 to -11·2)
United States Virgin Islands	All Ages	0·959 (0·724 to 1·22)	0·875 (0·613 to 1·19)	0·781 (0·540 to 1·05)	0·736 (0·286 to 1·28)	0·671 (0·453 to 0·965)	0·511 (0·211 to 0·858)	-47·0 (-77·7 to -22·4)
	15-49 years	0·393 (0·287 to 0·529)	0·320 (0·225 to 0·457)	0·305 (0·212 to 0·430)	0·297 (0·177 to 0·452)	0·311 (0·216 to 0·449)	0·279 (0·168 to 0·418)	-29·3 (-48·4 to -13·1)
	50-69 years	0·305 (0·219 to 0·409)	0·268 (0·178 to 0·383)	0·218 (0·140 to 0·314)	0·208 (0·0332 to 0·441)	0·187 (0·117 to 0·289)	0·113 (0·00233 to 0·246)	-63·2 (-99·8 to -30·5)
	70+ years	0·255 (0·196 to 0·314)	0·283 (0·199 to 0·386)	0·255 (0·171 to 0·348)	0·227 (0·0544 to 0·389)	0·170 (0·110 to 0·235)	0·115 (0·00309 to 0·219)	-55·4 (-98·8 to -22·5)
Central Latin America	All Ages	7530 (6830 to 8320)	7730 (6860 to 8590)	7100 (6060 to 8050)	6750 (4360 to 9080)	6700 (5890 to 7620)	5460 (3460 to 7170)	-27·6 (-53·7 to -5·76)
	15-49 years	3140 (2650 to 3780)	3070 (2540 to 3690)	2870 (2330 to 3430)	2660 (1590 to 3640)	2980 (2470 to 3610)	2460 (1530 to 3320)	-21·6 (-49·8 to 2·87)
	50-69 years	2360 (2190 to 2530)	2590 (2330 to 2870)	2290 (1880 to 2570)	2230 (1400 to 3030)	2150 (1910 to 2430)	1680 (1030 to 2260)	-29·0 (-56·5 to -5·60)
	70+ years	1820 (1680 to 1920)	1910 (1760 to 2100)	1760 (1540 to 1970)	1690 (1050 to 2160)	1390 (1210 to 1610)	1150 (660 to 1520)	-36·9 (-62·4 to -19·3)
Colombia	All Ages	1180 (1050 to 1330)	1310 (1140 to 1520)	1220 (1060 to 1420)	1170 (759 to 1490)	1140 (969 to 1340)	958 (606 to 1220)	-18·5 (-45·6 to -0·432)
	15-49 years	419 (340 to 542)	420 (328 to 556)	398 (307 to 524)	374 (240 to 523)	414 (323 to 550)	354 (234 to 489)	-15·6 (-42·4 to 2·36)
	50-69 years	373 (334 to 413)	447 (394 to 509)	402 (339 to 465)	393 (248 to 509)	390 (341 to 452)	314 (195 to 401)	-15·7 (-46·8 to 6·66)
	70+ years	359 (327 to 387)	420 (376 to 456)	398 (348 to 436)	379 (255 to 454)	314 (271 to 360)	268 (168 to 342)	-25·2 (-53·2 to -8·55)
Costa Rica	All Ages	58·3 (53·7 to 63·6)	64·8 (58·7 to 71·5)	57·3 (48·5 to 65·9)	55·6 (32·1 to 74·2)	54·7 (48·7 to 62·0)	42·5 (25·3 to 58·0)	-27·2 (-55·9 to -2·79)
	15-49 years	19·0 (16·0 to 22·8)	20·0 (16·8 to 23·8)	18·3 (15·0 to 22·0)	17·5 (11·3 to 24·5)	19·5 (16·4 to 23·3)	15·9 (10·7 to 21·3)	-16·3 (-42·5 to 5·35)
	50-69 years	20·1 (18·4 to 22·0)	23·8 (21·3 to 26·4)	19·9 (15·6 to 23·4)	19·9 (10·6 to 27·6)	19·9 (17·3 to 22·8)	14·4 (7·74 to 20·9)	-28·6 (-60·5 to 0·167)
	70+ years	18·4 (16·5 to 19·9)	20·4 (18·1 to 22·7)	18·5 (15·7 to 20·7)	17·6 (10·4 to 23·6)	14·7 (12·5 to 17·4)	11·5 (6·29 to 16·6)	-37·4 (-66·2 to -14·3)
El Salvador	All Ages	304 (261 to 354)	259 (214 to 311)	238 (193 to 297)	230 (148 to 320)	225 (184 to 271)	186 (120 to 249)	-38·9 (-59·5 to -24·0)

eTable 4. Risk deleted deaths due to all-form tuberculosis for alcohol use, smoking, and diabetes, and all three risk factors combined by age groups for 204 countries and territories (2020) with percent change between 2015 observed deaths and 2020 all-risk deleted deaths.

Location	Age group	2015 Observed deaths	2020 Observed deaths	2020 Smoking deleted deaths	2020 Alcohol deleted deaths	2020 Diabetes deleted deaths	2020 All-risk deleted deaths	Percent Change
	15-49 years	128 (105 to 157)	95·9 (77·0 to 124)	88·4 (67·7 to 110)	84·4 (51·6 to 117)	93·6 (74·8 to 120)	77·2 (48·1 to 105)	-39·8 (-56·9 to -22·8)
	50-69 years	79·6 (66·9 to 93·7)	77·4 (61·4 to 99·9)	67·8 (52·4 to 90·6)	67·5 (41·4 to 95·6)	65·1 (51·0 to 83·3)	50·8 (31·6 to 69·9)	-36·3 (-61·1 to -16·3)
	70+ years	87·6 (72·5 to 101)	80·1 (64·9 to 101)	76·3 (61·3 to 98·4)	72·5 (49·6 to 97·4)	60·6 (48·0 to 80·5)	52·7 (33·3 to 76·9)	-40·0 (-64·8 to -25·0)
Guatemala	All Ages	605 (533 to 687)	585 (519 to 659)	545 (461 to 612)	526 (354 to 657)	512 (448 to 590)	435 (285 to 544)	-28·1 (-50·8 to -12·9)
	15-49 years	294 (248 to 354)	274 (229 to 322)	261 (214 to 303)	246 (165 to 314)	268 (223 to 316)	231 (159 to 291)	-21·4 (-46·4 to -4·10)
	50-69 years	172 (153 to 195)	173 (152 to 194)	155 (129 to 177)	153 (96·2 to 193)	140 (120 to 161)	113 (69·1 to 146)	-34·2 (-57·5 to -17·9)
	70+ years	101 (94·7 to 108)	114 (101 to 126)	104 (89·6 to 117)	103 (66·6 to 123)	79·6 (70·1 to 91·3)	66·0 (40·0 to 85·6)	-34·8 (-60·2 to -17·1)
Honduras	All Ages	780 (549 to 1020)	753 (575 to 990)	663 (474 to 886)	679 (374 to 956)	632 (474 to 825)	511 (281 to 707)	-34·4 (-54·0 to -13·6)
	15-49 years	234 (160 to 318)	235 (154 to 319)	215 (138 to 290)	213 (133 to 298)	229 (151 to 311)	192 (123 to 268)	-17·1 (-42·1 to 20·5)
	50-69 years	287 (214 to 365)	281 (206 to 379)	233 (152 to 320)	248 (123 to 352)	226 (164 to 299)	169 (79·6 to 252)	-40·9 (-61·8 to -15·2)
	70+ years	230 (155 to 311)	215 (149 to 280)	192 (128 to 256)	195 (99·6 to 276)	155 (103 to 198)	127 (62·0 to 181)	-44·7 (-64·2 to -29·0)
Mexico	All Ages	3170 (2910 to 3540)	3190 (2880 to 3570)	2950 (2550 to 3330)	2730 (1560 to 3730)	2770 (2410 to 3170)	2220 (1250 to 3060)	-29·9 (-58·9 to -2·17)
	15-49 years	1440 (1250 to 1730)	1430 (1220 to 1700)	1340 (1110 to 1600)	1210 (661 to 1750)	1380 (1170 to 1640)	1110 (633 to 1580)	-22·8 (-55·7 to 9·06)
	50-69 years	986 (927 to 1070)	1050 (946 to 1160)	947 (796 to 1060)	891 (499 to 1230)	860 (751 to 986)	671 (378 to 957)	-32·0 (-60·3 to -4·09)
	70+ years	671 (640 to 696)	650 (584 to 700)	602 (535 to 659)	562 (328 to 722)	469 (394 to 538)	378 (211 to 520)	-43·7 (-67·7 to -21·5)
Nicaragua	All Ages	260 (237 to 297)	240 (201 to 284)	209 (167 to 263)	204 (115 to 295)	204 (169 to 245)	154 (84·6 to 211)	-40·8 (-67·7 to -16·4)
	15-49 years	96·8 (83·9 to 110)	84·7 (71·1 to 101)	73·6 (59·1 to 92·8)	68·5 (29·9 to 111)	82·2 (69·2 to 97·8)	58·5 (25·6 to 89·6)	-39·6 (-73·6 to -5·11)
	50-69 years	80·1 (69·1 to 93·3)	78·6 (63·3 to 99·1)	65·7 (51·8 to 85·9)	65·8 (35·4 to 99·8)	62·6 (49·0 to 80·0)	44·4 (22·9 to 65·3)	-44·4 (-70·7 to -19·0)
	70+ years	71·4 (61·5 to 85·9)	69·1 (56·6 to 84·1)	62·8 (48·1 to 80·2)	62·3 (41·5 to 83·1)	52·1 (41·7 to 63·8)	43·4 (29·0 to 59·1)	-39·1 (-58·6 to -16·6)
Panama	All Ages	237 (207 to 271)	216 (192 to 254)	201 (175 to 227)	190 (121 to 252)	189 (163 to 223)	157 (103 to 204)	-33·5 (-56·1 to -15·6)
	15-49 years	98·5 (79·2 to 123)	86·0 (70·4 to 112)	81·9 (65·1 to 104)	76·4 (49·1 to 103)	84·5 (69·1 to 110)	72·4 (47·9 to 96·5)	-26·5 (-49·0 to -8·43)
	50-69 years	69·3 (62·0 to 77·0)	67·4 (59·8 to 76·5)	60·5 (51·8 to 68·8)	57·8 (35·4 to 80·6)	57·3 (50·0 to 66·9)	45·4 (28·7 to 60·0)	-34·4 (-59·4 to -13·8)

eTable 4. Risk deleted deaths due to all-form tuberculosis for alcohol use, smoking, and diabetes, and all three risk factors combined by age groups for 204 countries and territories (2020) with percent change between 2015 observed deaths and 2020 all-risk deleted deaths.

Location	Age group	2015 Observed deaths	2020 Observed deaths	2020 Smoking deleted deaths	2020 Alcohol deleted deaths	2020 Diabetes deleted deaths	2020 All-risk deleted deaths	Percent Change
	70+ years	59·3 (53·3 to 65·2)	56·0 (48·8 to 61·1)	51·6 (44·7 to 57·6)	48·7 (28·6 to 63·2)	40·7 (34·7 to 46·7)	32·9 (18·6 to 45·4)	-44·4 (-69·5 to -26·7)
Venezuela (Bolivarian Republic of)	All Ages	939 (846 to 1090)	1110 (878 to 1420)	1010 (792 to 1250)	974 (616 to 1430)	973 (770 to 1230)	788 (503 to 1110)	-16·1 (-43·6 to 14·2)
	15-49 years	404 (336 to 515)	422 (329 to 566)	393 (299 to 516)	373 (226 to 536)	414 (321 to 558)	346 (217 to 482)	-14·5 (-40·7 to 13·0)
	50-69 years	293 (268 to 329)	386 (298 to 494)	339 (264 to 424)	331 (197 to 513)	329 (254 to 418)	253 (154 to 372)	-13·7 (-45·1 to 21·5)
	70+ years	220 (200 to 238)	283 (228 to 368)	256 (208 to 332)	248 (148 to 354)	208 (164 to 266)	167 (98·1 to 241)	-24·0 (-57·7 to 6·39)
Tropical Latin America	All Ages	9010 (7850 to 10400)	8560 (7470 to 9830)	7570 (6280 to 8750)	7390 (4310 to 9920)	7790 (6670 to 9000)	6130 (3790 to 7920)	-32·0 (-55·6 to -11·2)
	15-49 years	4200 (3390 to 5220)	3750 (3100 to 4570)	3460 (2770 to 4240)	3270 (2100 to 4470)	3680 (3030 to 4500)	3020 (2020 to 4030)	-28·2 (-51·0 to -7·81)
	50-69 years	3120 (2820 to 3460)	3120 (2770 to 3530)	2610 (2070 to 3060)	2650 (1450 to 3650)	2770 (2380 to 3150)	2030 (1170 to 2790)	-34·9 (-60·4 to -13·6)
	70+ years	1460 (1310 to 1530)	1510 (1370 to 1600)	1330 (1110 to 1500)	1300 (756 to 1670)	1170 (1060 to 1300)	904 (492 to 1200)	-37·9 (-64·6 to -18·5)
Brazil	All Ages	8640 (7520 to 10000)	8220 (7190 to 9490)	7290 (6030 to 8440)	7120 (4170 to 9510)	7490 (6390 to 8680)	5910 (3680 to 7630)	-31·7 (-54·9 to -11·2)
	15-49 years	4040 (3260 to 5040)	3610 (2990 to 4420)	3340 (2680 to 4090)	3150 (2030 to 4310)	3540 (2920 to 4350)	2910 (1960 to 3890)	-28·0 (-50·7 to -7·67)
	50-69 years	3000 (2710 to 3350)	3010 (2660 to 3420)	2520 (2000 to 2950)	2560 (1410 to 3520)	2670 (2290 to 3040)	1970 (1140 to 2690)	-34·5 (-59·6 to -13·7)
	70+ years	1390 (1250 to 1470)	1440 (1310 to 1530)	1280 (1070 to 1430)	1250 (736 to 1590)	1120 (1010 to 1240)	872 (483 to 1150)	-37·1 (-63·6 to -17·6)
Paraguay	All Ages	363 (311 to 425)	333 (264 to 409)	283 (208 to 358)	275 (138 to 443)	302 (239 to 377)	220 (115 to 355)	-39·6 (-66·3 to -10·4)
	15-49 years	159 (130 to 185)	138 (107 to 163)	125 (92·1 to 153)	117 (64·2 to 180)	136 (106 to 161)	107 (59·6 to 161)	-32·8 (-57·5 to -3·80)
	50-69 years	116 (97·9 to 144)	112 (87·0 to 148)	89·8 (62·4 to 122)	89·5 (34·5 to 159)	99·0 (77·1 to 133)	65·2 (27·2 to 121)	-43·9 (-74·4 to -10·0)
	70+ years	69·8 (58·6 to 81·6)	67·5 (51·1 to 81·9)	53·9 (35·4 to 71·3)	53·4 (17·9 to 87·7)	51·8 (40·4 to 66·1)	32·4 (8·94 to 58·9)	-53·6 (-86·6 to -24·6)
North Africa and Middle East	All Ages	22600 (18700 to 31700)	20200 (16300 to 28300)	17700 (13800 to 23900)	19900 (16000 to 28200)	16500 (12800 to 23500)	14300 (11100 to 19500)	-36·4 (-45·9 to -26·9)
	15-49 years	7960 (6400 to 10200)	6930 (5530 to 9100)	6020 (4840 to 7730)	6800 (5350 to 8950)	6560 (5260 to 8660)	5610 (4440 to 7270)	-29·4 (-39·1 to -18·0)
	50-69 years	5900 (4870 to 8700)	5790 (4590 to 8450)	4790 (3540 to 6700)	5700 (4490 to 8320)	4330 (3250 to 6440)	3500 (2460 to 5130)	-40·6 (-53·4 to -26·5)
	70+ years	6410 (5020 to 10800)	5870 (4500 to 8980)	5290 (3910 to 8130)	5820 (4480 to 8970)	4050 (2980 to 6550)	3620 (2600 to 5800)	-43·7 (-54·2 to -33·2)
Afghanistan	All Ages	7500 (5620 to 11600)	6790 (5120 to 10300)	6160 (4670 to 9060)	6760 (5120 to 10200)	5650 (4180 to 8530)	5070 (3830 to 7320)	-31·8 (-42·9 to -19·6)

eTable 4. Risk deleted deaths due to all-form tuberculosis for alcohol use, smoking, and diabetes, and all three risk factors combined by age groups for 204 countries and territories (2020) with percent change between 2015 observed deaths and 2020 all-risk deleted deaths.

Location	Age group	2015 Observed deaths	2020 Observed deaths	2020 Smoking deleted deaths	2020 Alcohol deleted deaths	2020 Diabetes deleted deaths	2020 All-risk deleted deaths	Percent Change
	15-49 years	3280 (2410 to 4720)	3030 (2200 to 4440)	2700 (2070 to 3820)	3000 (2200 to 4360)	2840 (2090 to 4170)	2520 (1930 to 3560)	-22·8 (-37·3 to -7·79)
	50-69 years	1570 (1100 to 2810)	1630 (1140 to 2680)	1400 (931 to 2250)	1620 (1130 to 2630)	1100 (696 to 1850)	908 (578 to 1430)	-41·4 (-60·7 to -24·8)
	70+ years	1430 (889 to 2940)	1170 (765 to 2230)	1090 (696 to 2080)	1170 (764 to 2220)	735 (422 to 1540)	676 (395 to 1380)	-52·3 (-62·7 to -36·6)
Algeria	All Ages	1740 (1320 to 2260)	1620 (1260 to 2090)	1370 (982 to 1810)	1580 (1190 to 2040)	1290 (992 to 1700)	1070 (772 to 1400)	-38·2 (-49·4 to -24·7)
	15-49 years	450 (344 to 667)	388 (307 to 524)	327 (254 to 442)	370 (257 to 516)	371 (293 to 497)	301 (219 to 405)	-32·6 (-47·1 to -16·9)
	50-69 years	435 (322 to 596)	437 (317 to 587)	354 (239 to 493)	423 (306 to 583)	330 (239 to 462)	260 (176 to 380)	-39·9 (-54·3 to -18·7)
	70+ years	742 (543 to 1000)	729 (538 to 991)	619 (417 to 822)	719 (523 to 973)	525 (387 to 702)	443 (294 to 582)	-40·1 (-52·9 to -24·7)
Bahrain	All Ages	11·8 (10·1 to 14·2)	11·4 (9·26 to 15·3)	9·17 (7·01 to 12·6)	10·9 (8·05 to 14·1)	9·12 (7·36 to 12·4)	7·04 (4·84 to 9·81)	-40·4 (-57·9 to -24·2)
	15-49 years	5·46 (4·55 to 6·87)	4·59 (3·61 to 6·57)	3·83 (2·85 to 5·60)	4·30 (3·06 to 5·83)	4·38 (3·43 to 6·23)	3·47 (2·44 to 4·96)	-36·5 (-55·1 to -21·3)
	50-69 years	3·32 (2·76 to 4·23)	3·63 (2·95 to 4·75)	2·68 (1·84 to 3·66)	3·46 (2·40 to 4·36)	2·59 (2·02 to 3·52)	1·81 (1·10 to 2·62)	-45·5 (-63·7 to -23·2)
	70+ years	2·63 (2·21 to 3·17)	2·91 (2·26 to 3·84)	2·41 (1·79 to 3·26)	2·84 (2·25 to 3·69)	1·89 (1·45 to 2·57)	1·51 (0·989 to 2·04)	-42·6 (-59·5 to -24·8)
Egypt	All Ages	1150 (1030 to 1280)	883 (749 to 1030)	695 (556 to 853)	870 (727 to 1010)	740 (618 to 868)	574 (442 to 727)	-50·0 (-59·1 to -37·6)
	15-49 years	377 (320 to 420)	294 (242 to 358)	232 (193 to 280)	287 (233 to 343)	286 (235 to 347)	222 (180 to 271)	-40·9 (-52·0 to -26·4)
	50-69 years	465 (415 to 533)	350 (290 to 418)	264 (198 to 334)	345 (282 to 415)	279 (228 to 332)	207 (149 to 259)	-55·3 (-65·2 to -41·9)
	70+ years	226 (196 to 275)	192 (159 to 235)	153 (114 to 193)	191 (159 to 234)	128 (103 to 165)	98·1 (70·4 to 133)	-56·4 (-67·7 to -41·6)
Iran (Islamic Republic of)	All Ages	1220 (1080 to 1310)	986 (866 to 1170)	868 (729 to 1050)	960 (831 to 1150)	832 (738 to 1000)	717 (593 to 878)	-41·2 (-49·8 to -29·6)
	15-49 years	431 (384 to 468)	338 (290 to 397)	301 (251 to 355)	326 (256 to 386)	333 (284 to 391)	288 (227 to 340)	-33·2 (-42·7 to -22·4)
	50-69 years	303 (273 to 330)	263 (230 to 314)	214 (165 to 262)	254 (213 to 310)	216 (192 to 267)	171 (133 to 218)	-43·5 (-55·7 to -29·0)
	70+ years	422 (363 to 463)	354 (298 to 428)	323 (254 to 395)	348 (291 to 430)	252 (211 to 325)	227 (180 to 295)	-46·2 (-56·0 to -33·1)
Iraq	All Ages	969 (764 to 1210)	894 (664 to 1180)	706 (503 to 973)	883 (657 to 1150)	654 (490 to 873)	516 (365 to 684)	-46·7 (-61·0 to -29·4)
	15-49 years	340 (258 to 451)	256 (184 to 355)	205 (142 to 302)	251 (175 to 352)	231 (164 to 326)	184 (127 to 268)	-45·7 (-59·5 to -26·4)
	50-69 years	309 (237 to 400)	314 (217 to 427)	232 (151 to 342)	310 (211 to 424)	216 (150 to 294)	158 (104 to 235)	-48·6 (-66·7 to -29·2)

eTable 4. Risk deleted deaths due to all-form tuberculosis for alcohol use, smoking, and diabetes, and all three risk factors combined by age groups for 204 countries and territories (2020) with percent change between 2015 observed deaths and 2020 all-risk deleted deaths.

Location	Age group	2015 Observed deaths	2020 Observed deaths	2020 Smoking deleted deaths	2020 Alcohol deleted deaths	2020 Diabetes deleted deaths	2020 All-risk deleted deaths	Percent Change
	70+ years	258 (206 to 325)	294 (221 to 406)	238 (173 to 336)	292 (214 to 398)	177 (135 to 241)	143 (97·4 to 203)	-44·4 (-60·9 to -23·8)
Jordan	All Ages	37·0 (31·4 to 44·6)	47·0 (36·0 to 57·9)	35·8 (27·0 to 46·5)	46·2 (35·7 to 57·7)	36·1 (27·9 to 45·6)	27·2 (19·8 to 35·9)	-26·6 (-44·8 to -0·314)
	15-49 years	11·0 (9·42 to 13·0)	13·2 (10·4 to 16·7)	9·78 (7·38 to 12·8)	12·9 (10·2 to 16·6)	12·5 (9·88 to 15·7)	9·15 (6·55 to 12·0)	-16·8 (-35·1 to 12·1)
	50-69 years	11·1 (9·21 to 14·1)	15·4 (11·9 to 19·8)	10·7 (7·55 to 14·7)	15·1 (11·3 to 19·7)	11·0 (8·37 to 14·6)	7·57 (5·17 to 10·3)	-31·6 (-53·5 to -0·905)
	70+ years	13·0 (10·6 to 16·2)	16·7 (12·5 to 20·9)	13·6 (9·58 to 17·6)	16·5 (12·3 to 20·9)	10·9 (8·01 to 14·3)	8·77 (6·05 to 12·3)	-32·6 (-50·7 to -8·39)
Kuwait	All Ages	35·6 (33·4 to 38·8)	39·2 (35·2 to 42·9)	31·3 (24·3 to 37·7)	39·0 (35·1 to 42·8)	29·3 (25·9 to 33·1)	22·9 (17·2 to 28·5)	-35·4 (-52·3 to -19·0)
	15-49 years	12·5 (11·8 to 13·6)	11·9 (10·8 to 13·0)	9·01 (6·97 to 11·0)	11·8 (10·7 to 12·9)	11·1 (10·1 to 12·2)	8·43 (6·58 to 10·2)	-32·7 (-49·3 to -17·3)
	50-69 years	9·28 (8·45 to 10·1)	11·6 (10·3 to 13·1)	8·96 (6·65 to 11·3)	11·5 (10·3 to 12·9)	8·64 (7·55 to 9·91)	6·64 (4·86 to 8·49)	-28·3 (-48·5 to -8·18)
	70+ years	12·7 (11·1 to 14·1)	15·1 (13·0 to 16·8)	12·7 (9·79 to 15·4)	15·1 (13·0 to 16·8)	8·96 (7·19 to 11·1)	7·26 (5·07 to 9·97)	-42·6 (-59·1 to -21·7)
Lebanon	All Ages	64·9 (56·6 to 76·5)	60·5 (50·8 to 72·6)	47·8 (33·4 to 62·2)	57·6 (46·1 to 71·9)	49·1 (41·1 to 60·5)	37·9 (27·3 to 51·3)	-41·4 (-56·0 to -21·7)
	15-49 years	13·0 (10·7 to 15·4)	11·6 (9·44 to 13·8)	9·12 (6·81 to 12·0)	10·8 (8·19 to 13·5)	11·2 (9·17 to 13·3)	8·36 (6·01 to 11·4)	-35·4 (-50·1 to -12·4)
	50-69 years	16·6 (13·5 to 19·7)	14·7 (12·6 to 17·6)	9·68 (6·19 to 13·4)	13·7 (10·5 to 17·1)	11·4 (9·52 to 13·9)	7·19 (4·79 to 10·8)	-56·4 (-74·0 to -37·7)
	70+ years	34·4 (28·7 to 41·9)	33·5 (26·7 to 42·0)	28·3 (19·7 to 37·5)	32·4 (25·0 to 41·3)	25·8 (20·4 to 33·9)	21·7 (15·5 to 29·4)	-36·8 (-51·2 to -16·3)
Libya	All Ages	146 (103 to 197)	164 (104 to 234)	136 (84·5 to 203)	161 (99·9 to 235)	133 (82·7 to 193)	109 (66·4 to 158)	-25·9 (-40·9 to -6·71)
	15-49 years	54·2 (36·4 to 78·4)	56·4 (34·1 to 85·0)	45·4 (26·8 to 73·3)	54·9 (34·2 to 83·4)	54·0 (32·4 to 82·3)	42·8 (24·5 to 68·5)	-21·7 (-40·0 to 1·10)
	50-69 years	38·7 (25·4 to 53·9)	49·9 (30·4 to 76·9)	40·5 (23·8 to 60·9)	49·1 (29·6 to 76·5)	38·7 (23·0 to 60·9)	31·0 (17·7 to 46·7)	-20·2 (-39·0 to 2·29)
	70+ years	46·4 (33·0 to 63·7)	51·6 (34·6 to 73·2)	44·7 (30·8 to 68·6)	51·1 (34·4 to 72·9)	34·4 (21·7 to 52·8)	29·2 (18·6 to 44·4)	-36·8 (-53·0 to -17·3)
Morocco	All Ages	4170 (2710 to 8680)	3620 (2280 to 7990)	3320 (2110 to 6980)	3580 (2250 to 7990)	2820 (1670 to 6310)	2560 (1530 to 5690)	-38·8 (-51·0 to -24·7)
	15-49 years	1080 (740 to 1810)	780 (554 to 1430)	691 (489 to 1170)	755 (538 to 1340)	723 (500 to 1340)	624 (425 to 1080)	-42·2 (-57·3 to -26·0)
	50-69 years	1290 (768 to 2840)	1270 (747 to 2890)	1120 (656 to 2440)	1260 (746 to 2880)	961 (501 to 2260)	844 (441 to 1890)	-34·9 (-52·7 to -15·3)
	70+ years	1640 (996 to 4040)	1490 (857 to 3580)	1430 (825 to 3320)	1490 (854 to 3580)	1060 (585 to 2690)	1020 (545 to 2500)	-38·4 (-51·1 to -20·8)
Oman	All Ages	22·4 (16·9 to 31·2)	17·8 (14·3 to 26·4)	15·8 (11·7 to 24·0)	17·4 (13·5 to 26·3)	14·6 (11·6 to 21·8)	12·6 (9·22 to 19·7)	-43·6 (-54·8 to -30·9)

eTable 4. Risk deleted deaths due to all-form tuberculosis for alcohol use, smoking, and diabetes, and all three risk factors combined by age groups for 204 countries and territories (2020) with percent change between 2015 observed deaths and 2020 all-risk deleted deaths.

Location	Age group	2015 Observed deaths	2020 Observed deaths	2020 Smoking deleted deaths	2020 Alcohol deleted deaths	2020 Diabetes deleted deaths	2020 All-risk deleted deaths	Percent Change
	15-49 years	8·23 (6·20 to 12·2)	6·39 (4·89 to 10·1)	5·44 (3·66 to 8·73)	6·10 (4·15 to 9·85)	6·15 (4·72 to 9·83)	5·02 (3·29 to 8·21)	-39·0 (-54·4 to -25·1)
	50-69 years	6·32 (4·48 to 8·53)	4·29 (3·22 to 6·12)	3·76 (2·69 to 5·49)	4·20 (3·20 to 5·98)	3·37 (2·52 to 4·68)	2·90 (2·01 to 4·28)	-53·8 (-65·1 to -36·7)
	70+ years	6·93 (4·61 to 9·95)	6·57 (4·27 to 9·72)	6·07 (3·88 to 9·10)	6·52 (4·16 to 9·70)	4·50 (2·89 to 6·95)	4·11 (2·59 to 6·11)	-40·5 (-52·9 to -23·9)
Palestine	All Ages	32·4 (24·7 to 42·7)	28·0 (19·6 to 37·8)	22·7 (16·7 to 33·0)	27·0 (19·2 to 38·1)	22·4 (15·4 to 31·0)	17·6 (12·5 to 26·1)	-45·8 (-62·5 to -29·3)
	15-49 years	7·48 (5·57 to 11·2)	6·46 (4·49 to 10·5)	5·08 (3·56 to 8·04)	6·01 (3·93 to 9·50)	6·25 (4·31 to 10·2)	4·65 (3·03 to 7·38)	-37·4 (-56·9 to -19·1)
	50-69 years	9·73 (6·82 to 13·6)	8·89 (5·64 to 13·3)	6·54 (4·23 to 10·2)	8·50 (5·44 to 13·4)	6·80 (4·19 to 10·1)	4·81 (3·01 to 7·91)	-50·4 (-67·8 to -29·3)
	70+ years	12·4 (9·46 to 16·2)	10·9 (8·16 to 14·5)	9·34 (7·13 to 12·3)	10·7 (8·06 to 14·5)	7·61 (5·48 to 10·2)	6·41 (4·52 to 8·97)	-48·1 (-62·3 to -34·0)
Qatar	All Ages	28·7 (22·7 to 36·0)	20·7 (14·5 to 27·7)	16·3 (10·9 to 22·7)	19·6 (12·9 to 27·1)	16·6 (11·7 to 22·5)	12·2 (6·99 to 17·9)	-57·5 (-71·8 to -42·6)
	15-49 years	16·7 (12·7 to 21·0)	12·8 (8·96 to 16·9)	10·0 (6·29 to 14·5)	12·0 (7·42 to 16·6)	12·2 (8·56 to 16·2)	8·96 (5·11 to 13·1)	-46·4 (-64·3 to -27·8)
	50-69 years	7·41 (5·74 to 9·43)	4·34 (2·80 to 6·20)	3·25 (2·06 to 4·72)	4·16 (2·65 to 5·98)	2·87 (1·85 to 4·15)	1·97 (1·04 to 3·01)	-73·4 (-84·1 to -61·2)
	70+ years	3·94 (3·18 to 5·10)	3·04 (2·30 to 4·20)	2·58 (1·72 to 3·63)	2·97 (2·30 to 4·05)	1·15 (0·685 to 2·00)	0·803 (0·281 to 1·57)	-79·5 (-92·3 to -63·3)
Saudi Arabia	All Ages	1220 (993 to 1550)	1150 (857 to 1650)	990 (724 to 1450)	1140 (853 to 1610)	967 (724 to 1380)	826 (603 to 1210)	-32·5 (-46·5 to -15·9)
	15-49 years	644 (503 to 861)	609 (427 to 911)	518 (356 to 804)	601 (423 to 882)	567 (397 to 849)	478 (332 to 744)	-26·0 (-41·2 to -6·25)
	50-69 years	327 (264 to 423)	319 (236 to 449)	266 (193 to 370)	316 (235 to 443)	247 (182 to 346)	205 (149 to 291)	-37·3 (-53·8 to -18·7)
	70+ years	235 (187 to 304)	213 (159 to 301)	197 (149 to 274)	212 (159 to 297)	145 (109 to 207)	134 (102 to 194)	-43·1 (-54·9 to -25·6)
Sudan	All Ages	1900 (1230 to 2690)	1600 (1010 to 2310)	1450 (936 to 2070)	1600 (1010 to 2310)	1400 (880 to 2000)	1270 (818 to 1810)	-32·8 (-42·5 to -19·6)
	15-49 years	668 (393 to 1010)	579 (365 to 859)	525 (339 to 768)	579 (365 to 859)	566 (356 to 835)	513 (331 to 752)	-22·7 (-35·6 to -7·10)
	50-69 years	413 (242 to 603)	393 (241 to 600)	340 (215 to 526)	393 (241 to 600)	315 (193 to 474)	273 (172 to 411)	-33·4 (-46·6 to -18·0)
	70+ years	459 (267 to 700)	418 (257 to 612)	377 (238 to 545)	418 (257 to 612)	303 (181 to 439)	274 (168 to 385)	-39·8 (-51·3 to -25·1)
Syrian Arab Republic	All Ages	118 (87·6 to 149)	104 (76·3 to 137)	86·0 (63·4 to 120)	101 (74·6 to 135)	84·1 (61·1 to 115)	68·4 (51·1 to 93·6)	-41·5 (-56·0 to -24·6)
	15-49 years	30·8 (22·9 to 38·8)	22·0 (15·9 to 31·0)	18·1 (13·0 to 25·0)	21·2 (15·3 to 30·0)	21·2 (15·2 to 30·1)	17·0 (12·1 to 24·2)	-44·4 (-59·2 to -25·2)
	50-69 years	38·9 (27·0 to 51·8)	39·1 (27·3 to 55·9)	30·2 (21·5 to 44·2)	38·0 (26·9 to 54·6)	31·5 (21·9 to 45·6)	23·7 (16·5 to 34·9)	-38·6 (-57·0 to -15·2)

eTable 4. Risk deleted deaths due to all-form tuberculosis for alcohol use, smoking, and diabetes, and all three risk factors combined by age groups for 204 countries and territories (2020) with percent change between 2015 observed deaths and 2020 all-risk deleted deaths.

Location	Age group	2015 Observed deaths	2020 Observed deaths	2020 Smoking deleted deaths	2020 Alcohol deleted deaths	2020 Diabetes deleted deaths	2020 All-risk deleted deaths	Percent Change
	70+ years	41·4 (32·4 to 51·2)	39·7 (31·1 to 52·4)	35·0 (26·3 to 48·6)	39·1 (29·5 to 52·1)	28·6 (21·7 to 39·9)	25·0 (18·5 to 35·2)	-39·2 (-52·6 to -21·5)
Tunisia	All Ages	260 (180 to 377)	236 (167 to 395)	185 (118 to 327)	224 (154 to 382)	183 (127 to 317)	138 (85·6 to 254)	-46·6 (-62·1 to -27·2)
	15-49 years	56·5 (36·2 to 86·9)	47·4 (33·4 to 79·6)	37·9 (25·9 to 64·4)	43·3 (28·4 to 74·6)	45·7 (32·2 to 76·6)	34·2 (23·1 to 58·6)	-39·1 (-54·4 to -18·9)
	50-69 years	73·2 (47·3 to 112)	70·6 (44·6 to 122)	52·0 (29·9 to 98·8)	66·5 (40·5 to 118)	53·9 (34·1 to 97·6)	38·0 (20·9 to 73·6)	-47·5 (-66·4 to -20·7)
	70+ years	125 (84·4 to 174)	115 (77·9 to 188)	92·1 (57·3 to 155)	111 (73·9 to 185)	80·2 (53·5 to 142)	62·7 (37·7 to 113)	-49·6 (-65·2 to -33·0)
Turkey	All Ages	1030 (913 to 1190)	907 (725 to 1120)	726 (528 to 938)	844 (616 to 1110)	719 (575 to 916)	536 (347 to 752)	-47·8 (-65·8 to -27·3)
	15-49 years	192 (167 to 222)	150 (123 to 189)	109 (78·1 to 151)	133 (81·6 to 191)	145 (119 to 183)	94·6 (54·9 to 146)	-50·6 (-71·6 to -29·5)
	50-69 years	328 (272 to 394)	297 (236 to 382)	216 (147 to 296)	272 (182 to 374)	245 (195 to 312)	165 (98·7 to 247)	-49·4 (-70·4 to -23·7)
	70+ years	482 (401 to 573)	445 (345 to 545)	386 (286 to 496)	425 (308 to 550)	314 (238 to 420)	261 (176 to 374)	-45·7 (-62·2 to -28·7)
United Arab Emirates	All Ages	39·3 (17·7 to 60·2)	34·5 (14·9 to 52·5)	29·3 (12·9 to 45·4)	31·8 (13·4 to 50·6)	29·0 (12·6 to 43·7)	22·8 (9·98 to 38·2)	-40·3 (-64·9 to -7·01)
	15-49 years	22·2 (8·53 to 36·7)	16·1 (6·58 to 25·8)	13·2 (5·50 to 22·5)	14·3 (5·38 to 24·9)	15·1 (6·12 to 23·9)	11·2 (4·27 to 20·3)	-48·3 (-73·4 to -15·6)
	50-69 years	8·18 (3·32 to 15·2)	8·03 (3·29 to 15·8)	6·24 (2·57 to 12·1)	7·23 (2·80 to 13·5)	5·52 (2·18 to 11·5)	3·69 (1·26 to 7·02)	-53·1 (-82·2 to -20·5)
	70+ years	8·30 (3·82 to 11·4)	10·1 (4·41 to 14·0)	9·44 (4·27 to 13·3)	9·82 (4·36 to 14·0)	8·08 (3·57 to 11·1)	7·53 (3·40 to 10·6)	-7·60 (-34·2 to 26·1)
Yemen	All Ages	841 (520 to 1240)	949 (582 to 1420)	786 (491 to 1210)	935 (579 to 1410)	846 (514 to 1270)	694 (439 to 1080)	-17·2 (-34·2 to 1·05)
	15-49 years	252 (157 to 396)	298 (186 to 491)	238 (141 to 410)	290 (184 to 482)	291 (182 to 480)	228 (135 to 398)	-9·31 (-34·0 to 18·2)
	50-69 years	229 (129 to 364)	276 (150 to 428)	209 (117 to 333)	272 (148 to 428)	237 (124 to 369)	177 (97·3 to 282)	-22·2 (-44·0 to 2·33)
	70+ years	209 (126 to 331)	252 (160 to 373)	216 (138 to 338)	250 (159 to 373)	194 (117 to 297)	165 (98·2 to 261)	-20·5 (-39·2 to -2·19)
South Asia	All Ages	573000 (536000 to 611000)	523000 (460000 to 598000)	448000 (367000 to 534000)	471000 (314000 to 594000)	448000 (388000 to 525000)	351000 (223000 to 454000)	-38·8 (-60·2 to -21·2)
	15-49 years	186000 (172000 to 202000)	169000 (148000 to 188000)	149000 (127000 to 172000)	150000 (97900 to 191000)	163000 (143000 to 181000)	129000 (84500 to 168000)	-30·7 (-54·7 to -10·0)
	50-69 years	213000 (198000 to 229000)	189000 (164000 to 224000)	154000 (120000 to 193000)	168000 (102000 to 218000)	158000 (134000 to 193000)	116000 (65900 to 157000)	-45·7 (-67·7 to -27·3)
	70+ years	148000 (137000 to 160000)	148000 (130000 to 170000)	128000 (104000 to 152000)	137000 (96600 to 168000)	110000 (92400 to 136000)	88800 (56400 to 116000)	-40·0 (-60·6 to -22·7)
Bangladesh	All Ages	31100 (27100 to 35500)	29600 (24000 to 35500)	24600 (18700 to 31400)	29000 (22900 to 35500)	25500 (20500 to 31400)	20900 (15900 to 27200)	-32·8 (-49·6 to -14·8)

eTable 4. Risk deleted deaths due to all-form tuberculosis for alcohol use, smoking, and diabetes, and all three risk factors combined by age groups for 204 countries and territories (2020) with percent change between 2015 observed deaths and 2020 all-risk deleted deaths.

Location	Age group	2015 Observed deaths	2020 Observed deaths	2020 Smoking deleted deaths	2020 Alcohol deleted deaths	2020 Diabetes deleted deaths	2020 All-risk deleted deaths	Percent Change
	15-49 years	8740<br>(7270 to 10200)	7700<br>(6160 to 9580)	6590<br>(5040 to 8710)	7480<br>(5690 to 9310)	7460<br>(6010 to 9310)	6240<br>(4790 to 8290)	-28·4<br>(-44·5 to -9·23)
	50-69 years	10400<br>(8620 to 12700)	9580<br>(7290 to 12700)	7480<br>(5350 to 10500)	9370<br>(7110 to 12500)	8420<br>(6510 to 11200)	6460<br>(4610 to 9080)	-37·6<br>(-54·7 to -19·3)
	70+ years	9780<br>(7830 to 11600)	11000<br>(8540 to 13600)	9290<br>(6940 to 12000)	10900<br>(8540 to 13600)	8320<br>(6520 to 10600)	6940<br>(5000 to 9020)	-28·9<br>(-51·2 to -7·86)
Bhutan	All Ages	125<br>(69·4 to 204)	110<br>(61·2 to 199)	100<br>(57·2 to 181)	106<br>(55·6 to 197)	93·4<br>(51·5 to 168)	82·6<br>(44·8 to 152)	-33·8<br>(-49·5 to -18·5)
	15-49 years	33·2<br>(18·1 to 58·4)	28·1<br>(15·2 to 51·8)	25·7<br>(14·1 to 49·5)	26·7<br>(13·4 to 49·6)	27·4<br>(14·7 to 50·5)	23·8<br>(12·4 to 45·0)	-27·7<br>(-47·4 to -7·08)
	50-69 years	38·8<br>(21·0 to 72·5)	34·5<br>(18·6 to 70·0)	31·0<br>(17·2 to 63·0)	33·0<br>(16·7 to 67·0)	29·2<br>(15·9 to 57·9)	25·0<br>(13·1 to 50·0)	-35·3<br>(-52·8 to -16·6)
	70+ years	48·9<br>(25·7 to 78·4)	44·9<br>(23·6 to 73·4)	41·3<br>(22·4 to 70·4)	44·2<br>(22·9 to 73·2)	34·3<br>(18·3 to 57·5)	31·3<br>(16·9 to 51·9)	-36·0<br>(-48·3 to -17·3)
India	All Ages	458000<br>(419000 to 496000)	416000<br>(354000 to 495000)	357000<br>(287000 to 442000)	368000<br>(224000 to 493000)	355000<br>(301000 to 426000)	273000<br>(160000 to 374000)	-40·3<br>(-64·4 to -22·4)
	15-49 years	147000<br>(135000 to 160000)	133000<br>(114000 to 158000)	118000<br>(98600 to 143000)	116000<br>(68700 to 158000)	128000<br>(110000 to 152000)	100000<br>(60100 to 138000)	-31·9<br>(-59·3 to -11·1)
	50-69 years	177000<br>(161000 to 194000)	155000<br>(132000 to 188000)	127000<br>(96900 to 163000)	136000<br>(74400 to 187000)	129000<br>(109000 to 160000)	94000<br>(49400 to 133000)	-47·0<br>(-71·9 to -28·5)
	70+ years	119000<br>(108000 to 130000)	118000<br>(101000 to 141000)	103000<br>(82300 to 127000)	108000<br>(71000 to 141000)	88500<br>(72400 to 111000)	70300<br>(41900 to 97900)	-40·9<br>(-62·8 to -23·0)
Nepal	All Ages	8060<br>(5600 to 10300)	7270<br>(4930 to 10100)	5920<br>(3900 to 8440)	6380<br>(3460 to 9360)	6120<br>(4300 to 8650)	4430<br>(2330 to 6880)	-45·1<br>(-68·6 to -23·9)
	15-49 years	2080<br>(1530 to 2790)	1740<br>(1230 to 2620)	1530<br>(1050 to 2270)	1490<br>(730 to 2340)	1690<br>(1200 to 2540)	1290<br>(639 to 2030)	-38·1<br>(-64·8 to -14·4)
	50-69 years	3080<br>(2030 to 4210)	2790<br>(1790 to 4090)	2150<br>(1350 to 3320)	2390<br>(1140 to 3870)	2360<br>(1530 to 3520)	1570<br>(691 to 2610)	-49·0<br>(-75·4 to -20·2)
	70+ years	2690<br>(1810 to 3590)	2620<br>(1800 to 3610)	2110<br>(1220 to 3140)	2380<br>(1390 to 3420)	1950<br>(1320 to 2680)	1440<br>(766 to 2260)	-46·3<br>(-69·0 to -23·1)
Pakistan	All Ages	76300<br>(56000 to 95200)	70500<br>(50200 to 88800)	60900<br>(44300 to 78700)	67800<br>(47600 to 87200)	61800<br>(44400 to 79600)	52000<br>(35700 to 69200)	-31·1<br>(-52·7 to -8·21)
	15-49 years	28200<br>(21000 to 35800)	26000<br>(18100 to 33400)	23000<br>(16000 to 29800)	24700<br>(16100 to 33100)	25100<br>(17500 to 32400)	21200<br>(14200 to 28500)	-23·9<br>(-51·9 to 2·09)
	50-69 years	22200<br>(16000 to 29100)	21600<br>(14700 to 29600)	17200<br>(12000 to 25100)	20600<br>(13200 to 28300)	17800<br>(12000 to 25100)	13600<br>(7990 to 20000)	-38·0<br>(-64·1 to -7·46)
	70+ years	16500<br>(11300 to 21400)	15600<br>(11000 to 19900)	13600<br>(9880 to 18000)	15300<br>(10800 to 19500)	11700<br>(8120 to 15600)	10000<br>(6920 to 13600)	-38·6<br>(-56·3 to -16·2)
Southeast Asia, East Asia, and Oceania	All Ages	265000<br>(248000 to 286000)	240000<br>(219000 to 273000)	191000<br>(159000 to 230000)	215000<br>(144000 to 276000)	209000<br>(185000 to 241000)	151000<br>(95900 to 203000)	-42·9<br>(-63·0 to -24·4)
	15-49 years	77800<br>(72200 to 84400)	62000<br>(56500 to 70500)	51200<br>(45600 to 60000)	55400<br>(38000 to 70400)	60800<br>(55000 to 69200)	45700<br>(31900 to 60900)	-41·1<br>(-60·1 to -24·7)
	50-69 years	92300<br>(85400 to 99900)	90700<br>(80000 to 104000)	67100<br>(52500 to 85800)	80100<br>(49600 to 106000)	79000<br>(68100 to 92500)	52800<br>(31300 to 74500)	-42·8<br>(-66·0 to -18·8)

eTable 4. Risk deleted deaths due to all-form tuberculosis for alcohol use, smoking, and diabetes, and all three risk factors combined by age groups for 204 countries and territories (2020) with percent change between 2015 observed deaths and 2020 all-risk deleted deaths.

Location	Age group	2015 Observed deaths	2020 Observed deaths	2020 Smoking deleted deaths	2020 Alcohol deleted deaths	2020 Diabetes deleted deaths	2020 All-risk deleted deaths	Percent Change
	70+ years	86500 (78700 to 95000)	81700 (74000 to 93800)	67000 (55000 to 78500)	73500 (50100 to 95700)	63000 (55400 to 73600)	47000 (28700 to 62300)	-45·7 (-65·8 to -26·8)
East Asia	All Ages	58500 (52700 to 65600)	51000 (43700 to 59900)	37500 (27000 to 50600)	42400 (21500 to 63900)	44200 (37500 to 52400)	27900 (14800 to 43900)	-52·3 (-73·8 to -26·8)
	15-49 years	13900 (12300 to 15500)	10200 (8410 to 12200)	7750 (5850 to 9960)	8490 (4580 to 13000)	9870 (8090 to 11800)	6550 (3760 to 10100)	-52·6 (-71·7 to -27·9)
	50-69 years	20600 (18200 to 22900)	19500 (16200 to 22700)	13100 (8460 to 18800)	15800 (7150 to 24300)	17000 (14000 to 20000)	9730 (4320 to 16700)	-52·7 (-78·4 to -24·0)
	70+ years	23000 (20800 to 26400)	20800 (18100 to 24700)	16100 (11600 to 20900)	17600 (8740 to 26100)	16800 (14300 to 20100)	11000 (5760 to 16800)	-52·0 (-74·6 to -27·4)
China	All Ages	45500 (39900 to 54300)	39300 (32300 to 48500)	28400 (18500 to 38800)	32500 (14900 to 52700)	33900 (27300 to 41500)	20900 (9530 to 33700)	-54·0 (-78·6 to -27·1)
	15-49 years	10800 (9390 to 12600)	7560 (6060 to 9380)	5740 (4030 to 7550)	6310 (3380 to 10400)	7310 (5810 to 9040)	4880 (2660 to 7580)	-54·8 (-73·4 to -31·8)
	50-69 years	15600 (13300 to 18600)	14500 (11600 to 17700)	9600 (5420 to 14100)	11700 (4750 to 19800)	12600 (9800 to 15500)	7060 (2150 to 12700)	-54·5 (-85·9 to -22·7)
	70+ years	18300 (16100 to 22000)	16800 (14000 to 20800)	12600 (8480 to 17200)	14000 (6410 to 22200)	13500 (11000 to 16700)	8540 (3700 to 13400)	-53·3 (-78·8 to -26·1)
Democratic People's Republic of Korea	All Ages	12200 (8870 to 16400)	11100 (8030 to 15100)	8560 (5700 to 12400)	9390 (4830 to 13600)	9820 (7090 to 13400)	6580 (3360 to 10000)	-45·7 (-70·9 to -23·0)
	15-49 years	2970 (2060 to 4480)	2570 (1780 to 3890)	1960 (1270 to 3080)	2130 (918 to 3270)	2500 (1730 to 3780)	1630 (739 to 2620)	-44·1 (-72·8 to -12·7)
	50-69 years	4910 (3470 to 6760)	4830 (3280 to 6580)	3430 (2080 to 5350)	4010 (1880 to 6030)	4290 (2960 to 5900)	2600 (1200 to 4260)	-46·7 (-73·6 to -21·4)
	70+ years	4110 (2790 to 5490)	3570 (2570 to 4760)	3060 (2030 to 4370)	3150 (1930 to 4480)	2920 (2110 to 3970)	2230 (1290 to 3330)	-45·4 (-66·7 to -27·2)
Taiwan (Province of China)	All Ages	803 (744 to 850)	682 (629 to 729)	543 (427 to 647)	587 (329 to 748)	545 (488 to 596)	377 (202 to 511)	-53·1 (-74·6 to -36·5)
	15-49 years	75·9 (67·9 to 84·2)	55·7 (49·8 to 62·4)	43·5 (35·0 to 53·5)	47·0 (23·3 to 66·0)	54·2 (48·5 to 60·7)	37·1 (19·7 to 51·5)	-51·2 (-71·6 to -33·6)
	50-69 years	155 (144 to 167)	144 (132 to 156)	103 (78·2 to 133)	117 (49·8 to 166)	121 (109 to 135)	71·5 (31·5 to 108)	-53·9 (-79·5 to -30·8)
	70+ years	569 (511 to 610)	480 (427 to 519)	394 (311 to 470)	421 (250 to 528)	368 (312 to 412)	266 (149 to 356)	-53·2 (-73·8 to -38·1)
Oceania	All Ages	3280 (2760 to 3950)	3410 (2820 to 4010)	2910 (2380 to 3600)	3180 (2390 to 3910)	2920 (2460 to 3420)	2340 (1760 to 2940)	-28·4 (-48·2 to -11·3)
	15-49 years	1290 (1090 to 1570)	1290 (1020 to 1570)	1060 (846 to 1340)	1170 (781 to 1520)	1230 (987 to 1500)	939 (660 to 1220)	-27·0 (-50·5 to -8·28)
	50-69 years	1010 (817 to 1210)	1130 (904 to 1450)	903 (685 to 1190)	1050 (774 to 1380)	880 (716 to 1140)	662 (438 to 913)	-34·4 (-57·9 to -12·0)
	70+ years	613 (506 to 750)	666 (536 to 827)	612 (496 to 769)	632 (492 to 789)	473 (381 to 608)	415 (307 to 523)	-32·0 (-50·1 to -14·4)
American Samoa	All Ages	1·07 (0·962 to 1·22)	1·17 (1·01 to 1·44)	0·968 (0·755 to 1·18)	1·15 (0·942 to 1·44)	0·882 (0·720 to 1·12)	0·717 (0·516 to 0·957)	-33·1 (-52·9 to -13·9)

eTable 4. Risk deleted deaths due to all-form tuberculosis for alcohol use, smoking, and diabetes, and all three risk factors combined by age groups for 204 countries and territories (2020) with percent change between 2015 observed deaths and 2020 all-risk deleted deaths.

Location	Age group	2015 Observed deaths	2020 Observed deaths	2020 Smoking deleted deaths	2020 Alcohol deleted deaths	2020 Diabetes deleted deaths	2020 All-risk deleted deaths	Percent Change
	15-49 years	0·323 (0·260 to 0·387)	0·323 (0·262 to 0·410)	0·266 (0·202 to 0·348)	0·312 (0·239 to 0·390)	0·294 (0·229 to 0·375)	0·238 (0·165 to 0·305)	-25·8 (-50·0 to -1·11)
	50-69 years	0·412 (0·350 to 0·478)	0·487 (0·415 to 0·596)	0·373 (0·266 to 0·497)	0·474 (0·393 to 0·584)	0·333 (0·258 to 0·443)	0·252 (0·165 to 0·369)	-38·8 (-59·6 to -17·2)
	70+ years	0·315 (0·267 to 0·371)	0·349 (0·279 to 0·444)	0·314 (0·248 to 0·394)	0·344 (0·267 to 0·438)	0·240 (0·175 to 0·327)	0·213 (0·153 to 0·287)	-32·4 (-47·9 to -14·1)
Cook Islands	All Ages	0·304 (0·266 to 0·355)	0·282 (0·237 to 0·351)	0·237 (0·189 to 0·297)	0·221 (0·0793 to 0·373)	0·198 (0·166 to 0·259)	0·133 (0·0444 to 0·213)	-56·2 (-84·1 to -29·7)
	15-49 years	0·0550 (0·0438 to 0·0661)	0·0449 (0·0346 to 0·0578)	0·0372 (0·0267 to 0·0478)	0·0346 (0·0140 to 0·0619)	0·0409 (0·0311 to 0·0520)	0·0272 (0·0119 to 0·0464)	-50·5 (-77·2 to -17·4)
	50-69 years	0·110 (0·0951 to 0·131)	0·108 (0·0869 to 0·143)	0·0826 (0·0558 to 0·113)	0·0802 (0·0220 to 0·148)	0·0681 (0·0539 to 0·0941)	0·0385 (0·00637 to 0·0739)	-64·9 (-94·2 to -35·4)
	70+ years	0·137 (0·116 to 0·166)	0·127 (0·106 to 0·152)	0·115 (0·0947 to 0·140)	0·104 (0·0413 to 0·156)	0·0867 (0·0709 to 0·107)	0·0655 (0·0245 to 0·0936)	-52·1 (-79·2 to -28·2)
Fiji	All Ages	52·6 (42·4 to 66·7)	48·2 (37·7 to 63·6)	41·1 (30·9 to 54·4)	43·3 (27·0 to 62·5)	37·3 (29·4 to 49·7)	29·0 (18·7 to 40·9)	-44·8 (-61·8 to -27·7)
	15-49 years	14·3 (11·7 to 18·7)	12·2 (9·48 to 16·0)	10·6 (7·75 to 13·7)	10·6 (6·32 to 15·2)	11·4 (8·85 to 15·1)	8·72 (5·44 to 12·6)	-38·7 (-60·8 to -13·1)
	50-69 years	22·1 (17·7 to 27·8)	20·3 (15·3 to 28·1)	16·0 (11·2 to 21·8)	18·1 (10·8 to 27·6)	14·4 (11·1 to 19·4)	10·2 (5·79 to 15·4)	-53·6 (-71·1 to -35·4)
	70+ years	13·9 (11·2 to 17·9)	13·6 (10·4 to 17·9)	12·5 (9·62 to 16·1)	12·6 (8·40 to 17·0)	9·36 (7·32 to 12·4)	7·98 (5·36 to 11·0)	-42·5 (-58·2 to -21·4)
Guam	All Ages	8·90 (7·92 to 10·1)	9·22 (7·82 to 10·6)	7·74 (6·28 to 9·31)	8·01 (4·64 to 10·8)	7·11 (6·11 to 8·39)	5·22 (3·30 to 7·51)	-41·3 (-65·9 to -16·6)
	15-49 years	2·42 (2·13 to 2·80)	2·50 (2·11 to 2·89)	2·13 (1·71 to 2·59)	2·17 (1·35 to 2·91)	2·34 (1·98 to 2·70)	1·78 (1·19 to 2·47)	-26·6 (-51·3 to -4·13)
	50-69 years	3·70 (3·17 to 4·36)	3·87 (3·20 to 4·65)	3·00 (2·23 to 3·88)	3·28 (1·68 to 4·78)	2·90 (2·40 to 3·47)	1·92 (1·08 to 3·05)	-48·0 (-73·3 to -20·2)
	70+ years	2·60 (2·18 to 2·99)	2·65 (2·15 to 3·18)	2·41 (1·92 to 2·91)	2·36 (1·35 to 3·21)	1·67 (1·35 to 2·24)	1·33 (0·708 to 1·97)	-48·7 (-73·9 to -27·2)
Kiribati	All Ages	89·7 (67·5 to 115)	86·1 (64·8 to 118)	61·9 (43·7 to 82·8)	82·4 (61·8 to 111)	68·9 (51·2 to 91·5)	48·5 (34·1 to 69·7)	-46·0 (-59·9 to -29·7)
	15-49 years	33·8 (23·8 to 44·0)	31·3 (21·6 to 43·6)	23·4 (15·8 to 32·3)	29·4 (19·3 to 41·4)	29·0 (20·1 to 40·7)	20·7 (13·3 to 30·6)	-38·9 (-54·7 to -24·0)
	50-69 years	32·9 (24·5 to 43·5)	33·6 (24·7 to 47·0)	20·9 (13·1 to 30·2)	32·2 (24·0 to 43·6)	23·8 (17·2 to 34·0)	14·2 (8·73 to 21·9)	-56·7 (-73·4 to -34·3)
	70+ years	17·7 (13·2 to 21·9)	16·7 (13·2 to 21·1)	13·1 (9·58 to 16·9)	16·4 (12·3 to 20·6)	11·7 (9·35 to 14·9)	9·14 (6·70 to 11·8)	-48·1 (-61·0 to -34·3)
Marshall Islands	All Ages	35·0 (21·9 to 46·5)	33·7 (20·7 to 45·8)	28·4 (17·5 to 39·8)	30·4 (16·3 to 43·9)	23·4 (14·6 to 32·1)	18·0 (9·74 to 25·7)	-48·7 (-65·5 to -35·3)
	15-49 years	14·7 (9·57 to 19·4)	13·3 (8·34 to 18·4)	11·1 (6·96 to 15·2)	11·7 (6·34 to 17·2)	12·1 (7·68 to 16·7)	9·02 (4·77 to 13·5)	-38·8 (-60·1 to -21·3)
	50-69 years	14·0 (8·43 to 18·6)	13·6 (7·76 to 19·2)	11·1 (6·56 to 16·4)	12·3 (6·33 to 18·7)	7·79 (4·50 to 11·5)	5·82 (2·89 to 8·89)	-58·3 (-74·9 to -44·1)

eTable 4. Risk deleted deaths due to all-form tuberculosis for alcohol use, smoking, and diabetes, and all three risk factors combined by age groups for 204 countries and territories (2020) with percent change between 2015 observed deaths and 2020 all-risk deleted deaths.

Location	Age group	2015 Observed deaths	2020 Observed deaths	2020 Smoking deleted deaths	2020 Alcohol deleted deaths	2020 Diabetes deleted deaths	2020 All-risk deleted deaths	Percent Change
	70+ years	4·94 (3·09 to 6·59)	5·69 (3·55 to 8·00)	5·11 (3·28 to 7·21)	5·26 (2·88 to 7·55)	2·49 (1·52 to 3·85)	2·04 (1·06 to 3·06)	-58·5 (-75·9 to -42·5)
Micronesia (Federated States of)	All Ages	12·9 (9·27 to 16·6)	12·8 (9·02 to 17·1)	10·0 (6·84 to 13·5)	11·5 (6·96 to 16·8)	10·3 (7·31 to 13·9)	7·47 (4·23 to 10·5)	-41·8 (-65·5 to -23·8)
	15-49 years	4·69 (3·24 to 6·16)	4·40 (3·09 to 5·87)	3·49 (2·38 to 4·61)	3·86 (2·06 to 5·61)	4·12 (2·87 to 5·48)	2·93 (1·55 to 4·10)	-37·3 (-66·0 to -18·9)
	50-69 years	5·28 (3·65 to 7·37)	5·61 (3·68 to 7·91)	4·05 (2·52 to 5·87)	5·05 (2·80 to 7·73)	4·14 (2·74 to 5·99)	2·74 (1·46 to 4·23)	-47·8 (-72·1 to -24·9)
	70+ years	2·44 (1·84 to 3·14)	2·43 (1·82 to 3·20)	2·16 (1·61 to 2·89)	2·29 (1·66 to 3·18)	1·72 (1·27 to 2·31)	1·46 (0·989 to 2·00)	-40·2 (-55·1 to -24·5)
Nauru	All Ages	3·38 (2·14 to 4·86)	2·69 (1·91 to 3·65)	2·26 (1·52 to 3·12)	2·36 (1·35 to 3·52)	2·36 (1·67 to 3·22)	1·80 (1·07 to 2·64)	-46·6 (-63·7 to -30·3)
	15-49 years	1·37 (0·882 to 1·88)	1·08 (0·772 to 1·58)	0·868 (0·570 to 1·23)	0·896 (0·413 to 1·54)	1·02 (0·724 to 1·49)	0·692 (0·319 to 1·09)	-49·3 (-74·9 to -24·1)
	50-69 years	1·21 (0·688 to 1·72)	0·947 (0·571 to 1·30)	0·749 (0·427 to 1·07)	0·828 (0·426 to 1·28)	0·740 (0·468 to 1·03)	0·534 (0·278 to 0·807)	-55·4 (-70·2 to -38·8)
	70+ years	0·625 (0·370 to 1·20)	0·531 (0·356 to 0·962)	0·509 (0·343 to 0·927)	0·508 (0·338 to 0·880)	0·468 (0·306 to 0·864)	0·441 (0·289 to 0·781)	-28·5 (-42·0 to -12·7)
Niue	All Ages	0·119 (0·0923 to 0·149)	0·109 (0·0827 to 0·140)	0·0933 (0·0695 to 0·121)	0·0943 (0·0508 to 0·140)	0·0807 (0·0610 to 0·105)	0·0604 (0·0343 to 0·0893)	-49·5 (-69·9 to -27·6)
	15-49 years	0·0199 (0·0133 to 0·0280)	0·0178 (0·0120 to 0·0248)	0·0152 (0·00988 to 0·0210)	0·0147 (0·00685 to 0·0248)	0·0164 (0·0111 to 0·0230)	0·0118 (0·00611 to 0·0194)	-40·2 (-68·9 to -7·45)
	50-69 years	0·0435 (0·0308 to 0·0592)	0·0441 (0·0305 to 0·0610)	0·0341 (0·0230 to 0·0481)	0·0365 (0·0167 to 0·0613)	0·0309 (0·0218 to 0·0424)	0·0203 (0·00995 to 0·0333)	-53·3 (-78·7 to -24·3)
	70+ years	0·0533 (0·0409 to 0·0646)	0·0451 (0·0353 to 0·0558)	0·0415 (0·0306 to 0·0524)	0·0406 (0·0235 to 0·0545)	0·0310 (0·0236 to 0·0388)	0·0259 (0·0142 to 0·0360)	-51·5 (-70·7 to -35·4)
Northern Mariana Islands	All Ages	4·57 (3·61 to 6·02)	4·40 (3·16 to 5·70)	3·49 (2·34 to 4·54)	3·83 (1·99 to 5·50)	3·15 (2·18 to 4·07)	2·18 (1·07 to 3·33)	-52·0 (-76·9 to -26·9)
	15-49 years	1·16 (0·860 to 1·59)	0·899 (0·625 to 1·24)	0·758 (0·513 to 1·05)	0·790 (0·447 to 1·19)	0·825 (0·566 to 1·14)	0·632 (0·383 to 0·932)	-45·2 (-67·1 to -23·5)
	50-69 years	2·25 (1·74 to 2·96)	2·25 (1·60 to 2·90)	1·60 (0·978 to 2·27)	1·89 (0·788 to 2·91)	1·45 (1·00 to 1·93)	0·803 (0·180 to 1·45)	-64·0 (-91·3 to -36·7)
	70+ years	1·08 (0·868 to 1·39)	1·21 (0·890 to 1·62)	1·09 (0·749 to 1·49)	1·11 (0·706 to 1·53)	0·832 (0·577 to 1·18)	0·699 (0·399 to 1·06)	-34·7 (-62·8 to -5·19)
Palau	All Ages	1·51 (1·23 to 1·82)	1·52 (1·20 to 1·94)	1·27 (0·979 to 1·71)	1·29 (0·639 to 1·87)	1·16 (0·927 to 1·49)	0·835 (0·417 to 1·27)	-44·6 (-72·3 to -21·7)
	15-49 years	0·518 (0·411 to 0·645)	0·454 (0·364 to 0·587)	0·381 (0·298 to 0·512)	0·373 (0·188 to 0·571)	0·416 (0·331 to 0·537)	0·294 (0·156 to 0·469)	-42·8 (-72·5 to -19·1)
	50-69 years	0·626 (0·472 to 0·788)	0·655 (0·483 to 0·939)	0·512 (0·364 to 0·754)	0·547 (0·238 to 0·858)	0·463 (0·340 to 0·674)	0·305 (0·124 to 0·493)	-51·0 (-80·3 to -25·3)
	70+ years	0·348 (0·284 to 0·437)	0·391 (0·309 to 0·500)	0·357 (0·281 to 0·446)	0·353 (0·205 to 0·475)	0·262 (0·204 to 0·345)	0·219 (0·127 to 0·295)	-36·8 (-59·6 to -14·9)
Papua New Guinea	All Ages	2770 (2300 to 3380)	2910 (2360 to 3470)	2500 (2020 to 3110)	2710 (2010 to 3330)	2510 (2080 to 2990)	2030 (1520 to 2540)	-26·5 (-46·9 to -9·01)

eTable 4. Risk deleted deaths due to all-form tuberculosis for alcohol use, smoking, and diabetes, and all three risk factors combined by age groups for 204 countries and territories (2020) with percent change between 2015 observed deaths and 2020 all-risk deleted deaths.

Location	Age group	2015 Observed deaths	2020 Observed deaths	2020 Smoking deleted deaths	2020 Alcohol deleted deaths	2020 Diabetes deleted deaths	2020 All-risk deleted deaths	Percent Change
	15-49 years	1100 (911 to 1360)	1110 (864 to 1380)	915 (707 to 1160)	1010 (673 to 1330)	1060 (836 to 1320)	811 (563 to 1080)	-26·0 (-49·3 to -6·46)
	50-69 years	835 (655 to 1010)	944 (739 to 1250)	767 (582 to 1020)	878 (636 to 1180)	746 (594 to 999)	569 (381 to 803)	-31·5 (-56·3 to -7·78)
	70+ years	505 (402 to 629)	558 (433 to 715)	517 (404 to 667)	530 (406 to 673)	398 (311 to 526)	351 (256 to 452)	-30·0 (-49·1 to -10·7)
Samoa	All Ages	15·0 (11·6 to 19·5)	14·2 (10·8 to 18·6)	11·1 (7·81 to 15·2)	12·9 (9·20 to 18·4)	11·0 (8·38 to 14·7)	7·96 (5·56 to 11·4)	-46·4 (-62·2 to -27·7)
	15-49 years	4·10 (2·92 to 5·65)	3·85 (2·69 to 4·92)	3·08 (2·20 to 4·11)	3·41 (2·24 to 5·17)	3·54 (2·47 to 4·54)	2·58 (1·72 to 3·94)	-36·2 (-55·5 to -9·13)
	50-69 years	5·47 (3·99 to 7·19)	5·41 (3·97 to 7·46)	3·74 (2·25 to 5·73)	4·85 (3·16 to 7·19)	3·84 (2·69 to 5·33)	2·40 (1·41 to 3·93)	-55·6 (-74·5 to -32·4)
	70+ years	4·85 (3·70 to 6·72)	4·45 (3·22 to 6·21)	3·78 (2·77 to 5·34)	4·15 (3·02 to 5·79)	3·07 (2·23 to 4·40)	2·45 (1·70 to 3·44)	-49·0 (-64·5 to -34·9)
Solomon Islands	All Ages	83·6 (62·2 to 110)	85·5 (66·2 to 111)	69·3 (51·4 to 89·7)	81·4 (62·6 to 109)	75·3 (58·0 to 98·2)	58·9 (43·7 to 78·8)	-29·2 (-43·7 to -11·8)
	15-49 years	35·3 (25·0 to 47·4)	36·1 (26·7 to 45·1)	29·2 (21·2 to 38·6)	33·6 (24·4 to 44·6)	34·6 (25·6 to 43·3)	26·4 (18·7 to 36·9)	-24·7 (-43·6 to -2·43)
	50-69 years	25·0 (17·5 to 34·2)	27·1 (20·2 to 37·3)	19·5 (13·4 to 27·1)	25·9 (19·4 to 37·2)	21·9 (16·1 to 30·4)	15·2 (10·4 to 21·7)	-38·8 (-54·5 to -15·5)
	70+ years	18·0 (13·4 to 22·3)	17·9 (14·2 to 22·3)	16·2 (12·4 to 20·1)	17·5 (13·5 to 22·3)	14·4 (11·0 to 18·4)	12·9 (9·68 to 16·3)	-28·2 (-41·5 to -12·9)
Tokelau	All Ages	0·102 (0·0726 to 0·143)	0·0865 (0·0655 to 0·115)	0·0740 (0·0549 to 0·0980)	0·0776 (0·0454 to 0·109)	0·0612 (0·0463 to 0·0828)	0·0480 (0·0280 to 0·0673)	-52·8 (-69·2 to -37·6)
	15-49 years	0·0194 (0·0134 to 0·0293)	0·0175 (0·0131 to 0·0241)	0·0147 (0·0105 to 0·0206)	0·0152 (0·00730 to 0·0234)	0·0160 (0·0120 to 0·0221)	0·0119 (0·00639 to 0·0180)	-38·3 (-64·8 to -14·3)
	50-69 years	0·0337 (0·0233 to 0·0484)	0·0305 (0·0222 to 0·0428)	0·0234 (0·0152 to 0·0342)	0·0265 (0·0130 to 0·0414)	0·0180 (0·0128 to 0·0259)	0·0116 (0·00441 to 0·0203)	-65·3 (-86·2 to -45·0)
	70+ years	0·0475 (0·0336 to 0·0671)	0·0372 (0·0279 to 0·0489)	0·0346 (0·0253 to 0·0453)	0·0346 (0·0226 to 0·0455)	0·0259 (0·0187 to 0·0355)	0·0231 (0·0145 to 0·0317)	-51·0 (-62·6 to -39·8)
Tonga	All Ages	5·79 (4·52 to 7·52)	5·12 (3·95 to 6·56)	4·01 (2·91 to 5·29)	4·85 (3·63 to 6·36)	3·93 (2·95 to 5·12)	2·96 (2·10 to 4·05)	-48·5 (-62·5 to -34·8)
	15-49 years	1·31 (0·951 to 1·74)	1·11 (0·769 to 1·48)	0·916 (0·667 to 1·26)	1·03 (0·679 to 1·41)	1·03 (0·719 to 1·39)	0·799 (0·561 to 1·12)	-38·4 (-53·4 to -23·1)
	50-69 years	2·02 (1·46 to 2·93)	1·87 (1·34 to 2·56)	1·34 (0·831 to 1·82)	1·76 (1·24 to 2·44)	1·37 (0·971 to 1·88)	0·932 (0·549 to 1·33)	-53·3 (-70·9 to -37·2)
	70+ years	2·25 (1·80 to 3·05)	1·98 (1·56 to 2·62)	1·60 (1·20 to 2·12)	1·90 (1·51 to 2·39)	1·36 (1·06 to 1·79)	1·07 (0·755 to 1·48)	-52·0 (-67·0 to -37·5)
Tuvalu	All Ages	5·08 (3·31 to 6·25)	4·64 (3·21 to 5·76)	3·81 (2·55 to 5·05)	4·23 (2·56 to 5·61)	3·79 (2·68 to 4·74)	2·86 (1·72 to 3·94)	-43·5 (-61·2 to -26·9)
	15-49 years	1·34 (0·849 to 1·73)	1·22 (0·825 to 1·60)	1·00 (0·633 to 1·39)	1·08 (0·571 to 1·49)	1·17 (0·797 to 1·54)	0·862 (0·480 to 1·23)	-35·3 (-57·6 to -9·29)
	50-69 years	2·04 (1·35 to 2·61)	1·87 (1·25 to 2·46)	1·42 (0·870 to 2·12)	1·69 (1·00 to 2·44)	1·47 (0·998 to 2·02)	1·01 (0·576 to 1·57)	-50·2 (-70·7 to -32·3)

eTable 4. Risk deleted deaths due to all-form tuberculosis for alcohol use, smoking, and diabetes, and all three risk factors combined by age groups for 204 countries and territories (2020) with percent change between 2015 observed deaths and 2020 all-risk deleted deaths.

Location	Age group	2015 Observed deaths	2020 Observed deaths	2020 Smoking deleted deaths	2020 Alcohol deleted deaths	2020 Diabetes deleted deaths	2020 All-risk deleted deaths	Percent Change
	70+ years	1·54 (1·02 to 1·98)	1·42 (0·964 to 1·79)	1·26 (0·864 to 1·61)	1·33 (0·891 to 1·81)	1·03 (0·673 to 1·30)	0·859 (0·525 to 1·16)	-43·6 (-58·0 to -25·1)
Vanuatu	All Ages	32·5 (18·9 to 47·0)	34·8 (21·2 to 48·8)	30·7 (19·4 to 42·7)	31·1 (19·0 to 45·4)	28·2 (17·1 to 39·8)	22·5 (13·7 to 33·7)	-29·9 (-52·9 to -8·33)
	15-49 years	12·1 (6·93 to 17·5)	12·8 (7·54 to 18·5)	11·5 (7·19 to 15·8)	11·1 (6·36 to 16·4)	12·2 (7·21 to 17·6)	9·65 (5·71 to 14·4)	-19·3 (-45·3 to 7·50)
	50-69 years	11·6 (6·01 to 17·6)	12·6 (6·65 to 19·3)	10·6 (5·89 to 15·2)	11·2 (6·39 to 17·1)	9·55 (4·93 to 14·5)	7·30 (4·16 to 11·7)	-35·5 (-61·0 to -11·3)
	70+ years	6·95 (4·46 to 9·54)	7·48 (5·08 to 10·4)	6·65 (4·57 to 9·67)	6·80 (3·91 to 10·2)	4·59 (3·02 to 6·40)	3·61 (1·66 to 5·64)	-47·3 (-73·5 to -26·7)
Southeast Asia	All Ages	204000 (186000 to 221000)	186000 (167000 to 212000)	151000 (123000 to 180000)	169000 (119000 to 209000)	162000 (141000 to 188000)	121000 (82300 to 158000)	-40·4 (-59·4 to -23·1)
	15-49 years	62600 (57700 to 68900)	50600 (45300 to 58300)	42400 (37200 to 49600)	45700 (32600 to 57100)	49700 (44300 to 57400)	38300 (27900 to 49700)	-38·8 (-57·7 to -23·1)
	50-69 years	70700 (64100 to 77300)	70100 (61500 to 81000)	53000 (40400 to 67200)	63200 (41700 to 81200)	61100 (52400 to 72200)	42400 (25600 to 58300)	-39·9 (-62·7 to -17·8)
	70+ years	62900 (55000 to 69400)	60200 (53700 to 69300)	50300 (40700 to 58900)	55400 (40300 to 69100)	45800 (39600 to 54400)	35500 (23900 to 45500)	-43·5 (-62·1 to -26·8)
Cambodia	All Ages	7570 (5300 to 9670)	6990 (4810 to 9600)	5320 (3770 to 7770)	5750 (2800 to 9480)	6190 (4240 to 8570)	3990 (1920 to 6940)	-46·7 (-76·9 to -21·8)
	15-49 years	2390 (1750 to 3180)	1990 (1420 to 2800)	1650 (1200 to 2350)	1610 (686 to 2710)	1960 (1400 to 2760)	1350 (618 to 2290)	-42·9 (-75·5 to -14·1)
	50-69 years	2890 (1980 to 3990)	2840 (1850 to 3870)	1990 (1320 to 3260)	2270 (926 to 3920)	2500 (1620 to 3420)	1440 (572 to 2740)	-49·3 (-82·7 to -21·5)
	70+ years	1930 (1220 to 2510)	1920 (1330 to 2610)	1440 (911 to 2190)	1630 (809 to 2630)	1490 (1050 to 2120)	965 (419 to 1790)	-49·6 (-77·7 to -26·4)
Indonesia	All Ages	102000 (87000 to 117000)	94500 (78300 to 110000)	75800 (54300 to 93000)	92600 (76500 to 107000)	82800 (66300 to 98200)	65500 (43400 to 81800)	-35·4 (-54·1 to -15·5)
	15-49 years	30400 (26600 to 35800)	25400 (21000 to 30800)	20800 (16800 to 26800)	24500 (20000 to 30000)	24900 (20400 to 30300)	19900 (14400 to 25900)	-34·3 (-51·2 to -14·9)
	50-69 years	37200 (31200 to 43400)	37400 (30100 to 44200)	28300 (18200 to 36100)	36600 (29400 to 43500)	32900 (25300 to 39600)	24600 (14100 to 32000)	-33·7 (-58·0 to -10·3)
	70+ years	30600 (25600 to 34800)	29400 (24500 to 35300)	24300 (18100 to 29500)	29100 (24200 to 34400)	22700 (17900 to 28500)	18700 (12200 to 24000)	-38·7 (-58·6 to -21·2)
Lao People's Democratic Republic	All Ages	2220 (1440 to 2970)	1930 (1280 to 2630)	1580 (1020 to 2300)	1570 (690 to 2530)	1690 (1100 to 2320)	1150 (525 to 1820)	-47·8 (-74·7 to -22·8)
	15-49 years	801 (537 to 1090)	647 (414 to 937)	557 (366 to 819)	511 (196 to 861)	636 (408 to 919)	442 (179 to 722)	-44·5 (-74·5 to -17·5)
	50-69 years	737 (470 to 1020)	687 (423 to 960)	516 (309 to 778)	539 (174 to 973)	575 (357 to 830)	347 (114 to 594)	-52·5 (-82·5 to -21·5)
	70+ years	514 (305 to 690)	478 (300 to 657)	389 (229 to 582)	401 (183 to 624)	359 (223 to 503)	249 (105 to 378)	-51·1 (-75·8 to -28·8)
Malaysia	All Ages	2260 (2000 to 2570)	2150 (1850 to 2570)	1800 (1490 to 2200)	2010 (1520 to 2520)	1750 (1520 to 2150)	1400 (965 to 1830)	-38·1 (-57·6 to -23·9)

eTable 4. Risk deleted deaths due to all-form tuberculosis for alcohol use, smoking, and diabetes, and all three risk factors combined by age groups for 204 countries and territories (2020) with percent change between 2015 observed deaths and 2020 all-risk deleted deaths.

Location	Age group	2015 Observed deaths	2020 Observed deaths	2020 Smoking deleted deaths	2020 Alcohol deleted deaths	2020 Diabetes deleted deaths	2020 All-risk deleted deaths	Percent Change
	15-49 years	705 (586 to 859)	660 (538 to 822)	581 (453 to 739)	622 (470 to 812)	645 (522 to 804)	542 (409 to 709)	-23·1 (-41·3 to -9·01)
	50-69 years	838 (728 to 962)	790 (657 to 942)	638 (497 to 802)	733 (513 to 911)	650 (549 to 804)	492 (309 to 654)	-41·2 (-62·2 to -24·5)
	70+ years	687 (583 to 793)	675 (563 to 834)	567 (441 to 715)	640 (492 to 795)	440 (345 to 569)	346 (223 to 458)	-49·6 (-66·9 to -33·8)
Maldives	All Ages	18·5 (16·5 to 20·8)	16·7 (14·1 to 20·8)	12·8 (9·74 to 17·2)	15·8 (11·7 to 20·9)	13·6 (11·4 to 17·2)	9·88 (6·62 to 14·0)	-46·5 (-62·8 to -28·6)
	15-49 years	6·35 (5·52 to 7·39)	6·55 (5·20 to 8·35)	5·47 (4·27 to 6·88)	6·16 (4·19 to 8·11)	6·44 (5·13 to 8·20)	5·11 (3·52 to 6·69)	-19·2 (-43·0 to 5·37)
	50-69 years	4·58 (3·73 to 5·74)	4·48 (3·44 to 5·85)	3·25 (2·16 to 4·76)	4·21 (2·94 to 5·66)	3·75 (2·92 to 4·99)	2·55 (1·60 to 3·97)	-44·2 (-63·7 to -15·3)
	70+ years	6·86 (5·95 to 7·84)	5·26 (4·35 to 6·57)	3·69 (2·34 to 5·09)	5·07 (3·68 to 6·60)	3·05 (2·32 to 4·23)	1·83 (0·800 to 3·04)	-73·4 (-88·6 to -55·7)
Mauritius	All Ages	16·9 (15·6 to 18·2)	16·5 (15·0 to 17·9)	13·1 (11·0 to 15·9)	14·1 (7·77 to 18·3)	13·5 (12·0 to 15·0)	9·50 (5·70 to 12·9)	-43·7 (-65·8 to -23·2)
	15-49 years	5·45 (4·81 to 6·25)	5·36 (4·51 to 6·22)	4·54 (3·69 to 5·55)	4·68 (2·85 to 6·17)	5·11 (4·28 to 5·95)	3·91 (2·58 to 5·15)	-28·3 (-49·8 to -9·41)
	50-69 years	7·19 (6·56 to 7·86)	7·02 (6·30 to 7·76)	5·17 (4·13 to 6·59)	5·81 (2·80 to 8·04)	5·49 (4·83 to 6·18)	3·49 (1·72 to 5·14)	-51·4 (-76·1 to -27·1)
	70+ years	4·09 (3·74 to 4·43)	3·99 (3·60 to 4·31)	3·30 (2·69 to 3·86)	3·47 (2·12 to 4·34)	2·73 (2·41 to 3·12)	1·98 (1·12 to 2·75)	-51·6 (-72·1 to -32·2)
Myanmar	All Ages	22100 (18800 to 26500)	17700 (14000 to 22900)	15100 (11700 to 19100)	15500 (9420 to 22300)	15000 (12000 to 19100)	11500 (6900 to 16400)	-47·7 (-68·7 to -27·5)
	15-49 years	7900 (6440 to 9500)	5380 (4290 to 7190)	4830 (3760 to 6550)	4680 (2980 to 6660)	5270 (4170 to 6960)	4190 (2770 to 5980)	-46·8 (-64·8 to -30·7)
	50-69 years	7070 (5560 to 8900)	6310 (4710 to 8450)	5110 (3650 to 6820)	5420 (2820 to 8300)	5200 (3840 to 6860)	3680 (1820 to 5730)	-47·8 (-73·8 to -21·4)
	70+ years	5790 (4640 to 7280)	5180 (3820 to 6930)	4380 (3140 to 5820)	4620 (2810 to 6450)	3710 (2690 to 5060)	2830 (1500 to 4090)	-51·1 (-72·7 to -29·5)
Philippines	All Ages	28000 (26200 to 30100)	26200 (23700 to 28500)	20600 (15600 to 24800)	20700 (8290 to 33000)	23100 (20600 to 25700)	14500 (5880 to 23400)	-48·1 (-79·0 to -12·2)
	15-49 years	8360 (7770 to 9000)	7280 (6580 to 7890)	5730 (4520 to 6870)	5510 (1720 to 9590)	7150 (6480 to 7750)	4350 (1400 to 7730)	-47·8 (-83·1 to -3·83)
	50-69 years	11000 (10200 to 11900)	10700 (9520 to 11800)	7740 (5350 to 9760)	8250 (2620 to 13900)	9400 (8250 to 10600)	5270 (1630 to 9160)	-52·0 (-85·5 to -14·1)
	70+ years	7180 (6690 to 7680)	7220 (6520 to 7850)	6130 (4980 to 7030)	5970 (2870 to 8540)	5510 (4950 to 6280)	3890 (1760 to 5780)	-45·7 (-75·2 to -17·3)
Seychelles	All Ages	4·37 (3·99 to 4·89)	3·84 (3·36 to 4·64)	2·99 (2·25 to 3·83)	3·24 (1·70 to 4·53)	3·02 (2·59 to 3·67)	2·01 (1·05 to 2·86)	-54·0 (-76·0 to -34·0)
	15-49 years	1·47 (1·25 to 1·72)	1·28 (1·06 to 1·49)	1·05 (0·796 to 1·31)	1·08 (0·615 to 1·55)	1·21 (0·994 to 1·42)	0·869 (0·514 to 1·21)	-40·9 (-65·3 to -18·3)
	50-69 years	1·58 (1·33 to 1·89)	1·55 (1·26 to 1·96)	1·13 (0·766 to 1·56)	1·28 (0·601 to 1·84)	1·18 (0·924 to 1·52)	0·723 (0·337 to 1·10)	-54·2 (-78·9 to -30·3)

eTable 4. Risk deleted deaths due to all-form tuberculosis for alcohol use, smoking, and diabetes, and all three risk factors combined by age groups for 204 countries and territories (2020) with percent change between 2015 observed deaths and 2020 all-risk deleted deaths.

Location	Age group	2015 Observed deaths	2020 Observed deaths	2020 Smoking deleted deaths	2020 Alcohol deleted deaths	2020 Diabetes deleted deaths	2020 All-risk deleted deaths	Percent Change
	70+ years	1·26 (1·13 to 1·45)	0·975 (0·845 to 1·17)	0·766 (0·592 to 0·942)	0·846 (0·446 to 1·15)	0·587 (0·472 to 0·736)	0·378 (0·152 to 0·572)	-69·9 (-87·5 to -54·1)
Sri Lanka	All Ages	1070 (907 to 1280)	866 (512 to 1390)	758 (439 to 1190)	734 (329 to 1200)	670 (326 to 1100)	495 (141 to 871)	-53·9 (-85·7 to -23·3)
	15-49 years	191 (163 to 232)	164 (107 to 250)	141 (92·5 to 217)	135 (54·3 to 226)	157 (101 to 240)	113 (43·7 to 188)	-40·8 (-75·6 to -4·74)
	50-69 years	457 (366 to 548)	349 (200 to 573)	296 (163 to 476)	287 (114 to 497)	267 (129 to 450)	185 (47·5 to 344)	-59·6 (-88·6 to -28·1)
	70+ years	409 (334 to 505)	346 (205 to 523)	314 (161 to 476)	305 (142 to 481)	239 (77·0 to 388)	189 (30·9 to 329)	-53·9 (-92·2 to -24·9)
Thailand	All Ages	12700 (10800 to 14900)	12700 (10400 to 15000)	11200 (9010 to 13500)	11300 (7440 to 14700)	10700 (8850 to 12600)	8700 (5960 to 11600)	-31·2 (-53·5 to -7·14)
	15-49 years	4160 (3030 to 5590)	3340 (2360 to 4380)	3170 (2210 to 4210)	3140 (2120 to 4180)	3320 (2340 to 4350)	3000 (2020 to 4020)	-27·7 (-40·0 to -14·6)
	50-69 years	3480 (2880 to 4130)	3850 (3040 to 4780)	3260 (2530 to 4030)	3350 (1990 to 4590)	3410 (2660 to 4230)	2630 (1690 to 3560)	-24·3 (-53·7 to 3·38)
	70+ years	4960 (4060 to 5800)	5440 (4300 to 6650)	4700 (3550 to 5830)	4740 (2910 to 6480)	3940 (3190 to 4950)	3010 (1690 to 4530)	-38·9 (-66·5 to -8·32)
Timor-Leste	All Ages	608 (414 to 954)	647 (451 to 960)	538 (374 to 744)	570 (326 to 793)	565 (395 to 841)	426 (253 to 613)	-29·1 (-52·4 to -7·58)
	15-49 years	135 (91·9 to 170)	143 (96·4 to 193)	123 (81·3 to 162)	125 (75·9 to 174)	141 (94·9 to 189)	108 (68·2 to 151)	-19·3 (-46·7 to 4·36)
	50-69 years	226 (153 to 364)	227 (150 to 329)	173 (110 to 247)	192 (93·2 to 297)	193 (130 to 283)	127 (64·8 to 208)	-42·5 (-69·2 to -16·9)
	70+ years	196 (129 to 348)	230 (155 to 395)	195 (131 to 303)	206 (116 to 318)	184 (123 to 324)	143 (81·3 to 221)	-25·7 (-50·2 to -0·991)
Viet Nam	All Ages	25000 (21400 to 29500)	21800 (17900 to 28100)	17600 (14200 to 22500)	18100 (10200 to 26900)	18800 (15600 to 24200)	13200 (8440 to 19900)	-47·2 (-66·2 to -22·1)
	15-49 years	7480 (5950 to 8990)	5520 (4500 to 6800)	4750 (3940 to 5980)	4780 (3290 to 6880)	5470 (4470 to 6740)	4230 (3100 to 5710)	-43·2 (-57·9 to -21·6)
	50-69 years	6580 (5370 to 8280)	6790 (4940 to 9770)	4870 (3290 to 7440)	5390 (2430 to 9240)	5990 (4360 to 8530)	3580 (1790 to 5870)	-45·7 (-70·6 to -11·1)
	70+ years	10600 (8270 to 13000)	9230 (7080 to 11900)	7770 (5810 to 9970)	7710 (4240 to 11600)	7140 (5500 to 8990)	5150 (3040 to 8010)	-51·2 (-70·9 to -30·3)
Sub-Saharan Africa	All Ages	646000 (557000 to 727000)	549000 (476000 to 624000)	520000 (446000 to 589000)	498000 (359000 to 626000)	516000 (442000 to 590000)	449000 (323000 to 553000)	-30·6 (-49·1 to -18·3)
	15-49 years	295000 (255000 to 341000)	246000 (210000 to 288000)	234000 (199000 to 270000)	225000 (165000 to 284000)	243000 (208000 to 285000)	214000 (160000 to 263000)	-27·2 (-45·0 to -14·8)
	50-69 years	166000 (144000 to 187000)	156000 (133000 to 179000)	142000 (118000 to 163000)	136000 (86600 to 179000)	142000 (120000 to 165000)	115000 (72400 to 149000)	-30·6 (-56·7 to -10·6)
	70+ years	89000 (77800 to 97200)	83500 (72400 to 92600)	79300 (68000 to 88300)	73200 (44000 to 92800)	66400 (57500 to 78600)	55500 (32100 to 71700)	-37·7 (-63·1 to -21·3)
Central Sub-Saharan Africa	All Ages	96900 (75600 to 123000)	80800 (59600 to 106000)	75700 (56200 to 101000)	70900 (40500 to 93700)	73700 (54000 to 97000)	61400 (36500 to 79300)	-36·3 (-59·7 to -22·0)

eTable 4. Risk deleted deaths due to all-form tuberculosis for alcohol use, smoking, and diabetes, and all three risk factors combined by age groups for 204 countries and territories (2020) with percent change between 2015 observed deaths and 2020 all-risk deleted deaths.

Location	Age group	2015 Observed deaths	2020 Observed deaths	2020 Smoking deleted deaths	2020 Alcohol deleted deaths	2020 Diabetes deleted deaths	2020 All-risk deleted deaths	Percent Change
	15-49 years	38800 (30100 to 49700)	32400 (23700 to 42600)	30200 (22300 to 40300)	28000 (16000 to 38800)	31500 (23000 to 41400)	25600 (14900 to 34400)	-33·5 (-59·8 to -17·8)
	50-69 years	28300 (20300 to 36500)	27500 (18700 to 37300)	25100 (16800 to 35100)	23400 (11200 to 32700)	23900 (16100 to 32700)	18700 (8980 to 25700)	-33·6 (-64·5 to -13·2)
	70+ years	11100 (7590 to 14600)	10200 (7300 to 13900)	9800 (7060 to 13300)	8860 (4390 to 12000)	7720 (5540 to 10500)	6470 (3180 to 8950)	-40·9 (-70·7 to -23·0)
Angola	All Ages	16600 (11700 to 21300)	15900 (12100 to 20800)	14400 (10800 to 19200)	13700 (8460 to 20400)	14700 (11200 to 19400)	11900 (7610 to 17200)	-28·1 (-54·4 to -5·08)
	15-49 years	6700 (4660 to 9320)	6930 (4910 to 9810)	6350 (4490 to 9010)	5920 (3430 to 9120)	6820 (4820 to 9660)	5440 (3280 to 8420)	-18·3 (-50·3 to 10·1)
	50-69 years	4250 (2970 to 5800)	4580 (3360 to 6010)	3850 (2780 to 5230)	3690 (1540 to 5730)	3940 (2810 to 5160)	2740 (1180 to 4270)	-34·7 (-70·8 to -2·16)
	70+ years	1650 (1060 to 2290)	1700 (1160 to 2270)	1560 (1110 to 2070)	1430 (669 to 2090)	1310 (891 to 1810)	1030 (484 to 1450)	-37·2 (-70·9 to -12·9)
Central African Republic	All Ages	11100 (8570 to 13500)	9870 (7260 to 12900)	9250 (6950 to 12000)	8980 (5820 to 12700)	9150 (6830 to 12100)	7900 (5260 to 10500)	-28·5 (-49·4 to -14·0)
	15-49 years	5040 (3880 to 6310)	4360 (3300 to 5770)	4080 (3080 to 5260)	3940 (2560 to 5500)	4250 (3210 to 5630)	3630 (2420 to 5020)	-27·9 (-49·4 to -12·9)
	50-69 years	3220 (2390 to 4270)	3260 (2160 to 4250)	2960 (1990 to 3920)	2870 (1480 to 4240)	2850 (1860 to 3750)	2310 (1190 to 3320)	-28·3 (-60·1 to -7·19)
	70+ years	831 (553 to 1100)	815 (538 to 1070)	777 (520 to 1010)	729 (407 to 1020)	617 (403 to 819)	529 (276 to 771)	-36·1 (-62·4 to -17·9)
Congo	All Ages	3180 (2250 to 4480)	2880 (2070 to 4280)	2680 (1940 to 3780)	2470 (1300 to 4150)	2660 (1900 to 3990)	2170 (1170 to 3560)	-31·8 (-57·7 to -11·5)
	15-49 years	1620 (1070 to 2430)	1430 (985 to 2130)	1340 (928 to 1980)	1240 (652 to 2150)	1400 (970 to 2090)	1160 (631 to 1940)	-28·5 (-53·6 to -7·98)
	50-69 years	856 (584 to 1230)	915 (639 to 1380)	831 (578 to 1230)	761 (356 to 1350)	805 (565 to 1220)	621 (296 to 1030)	-27·5 (-61·7 to 2·47)
	70+ years	348 (256 to 469)	324 (233 to 428)	299 (209 to 395)	261 (91·0 to 434)	238 (170 to 321)	178 (59·7 to 291)	-48·8 (-81·0 to -20·4)
Democratic Republic of the Congo	All Ages	64900 (48800 to 89800)	51100 (35000 to 75400)	48300 (33500 to 71800)	44700 (24200 to 63100)	46200 (31400 to 68000)	38700 (21300 to 54400)	-40·1 (-66·2 to -26·5)
	15-49 years	24900 (19000 to 34500)	19200 (13300 to 27900)	17900 (12400 to 25900)	16400 (8720 to 22900)	18500 (12700 to 27100)	15000 (7940 to 21100)	-39·4 (-69·4 to -22·5)
	50-69 years	19700 (13500 to 27000)	18400 (11800 to 27600)	17100 (11000 to 26200)	15700 (7700 to 23100)	16000 (10500 to 23800)	12800 (6420 to 18700)	-34·5 (-67·6 to -15·5)
	70+ years	8080 (5320 to 11300)	7240 (4830 to 10400)	7040 (4750 to 10200)	6330 (3180 to 9080)	5460 (3650 to 8100)	4660 (2330 to 6820)	-41·5 (-73·3 to -25·8)
Equatorial Guinea	All Ages	324 (203 to 527)	339 (211 to 527)	323 (199 to 509)	301 (168 to 515)	314 (195 to 492)	271 (154 to 463)	-16·5 (-41·6 to 5·88)
	15-49 years	171 (100 to 290)	184 (106 to 291)	177 (101 to 284)	166 (88·4 to 287)	180 (103 to 284)	157 (85·2 to 269)	-8·05 (-33·4 to 16·5)
	50-69 years	63·1 (42·0 to 102)	75·4 (49·0 to 112)	68·9 (45·4 to 109)	62·2 (28·1 to 112)	64·1 (40·9 to 97·2)	49·6 (23·6 to 89·1)	-21·4 (-62·1 to 10·0)

eTable 4. Risk deleted deaths due to all-form tuberculosis for alcohol use, smoking, and diabetes, and all three risk factors combined by age groups for 204 countries and territories (2020) with percent change between 2015 observed deaths and 2020 all-risk deleted deaths.

Location	Age group	2015 Observed deaths	2020 Observed deaths	2020 Smoking deleted deaths	2020 Alcohol deleted deaths	2020 Diabetes deleted deaths	2020 All-risk deleted deaths	Percent Change
	70+ years	35·8 (25·3 to 52·9)	34·4 (22·7 to 49·0)	32·7 (21·5 to 47·2)	27·5 (7·78 to 51·5)	24·7 (16·3 to 35·8)	18·9 (4·18 to 37·1)	-47·2 (-86·7 to -20·0)
Gabon	All Ages	848 (543 to 1160)	755 (487 to 1100)	709 (465 to 1000)	653 (332 to 1100)	682 (435 to 994)	569 (304 to 903)	-33·1 (-55·7 to -10·4)
	15-49 years	403 (247 to 580)	340 (214 to 500)	321 (205 to 455)	303 (165 to 508)	331 (208 to 489)	284 (162 to 451)	-29·6 (-48·7 to -8·91)
	50-69 years	253 (152 to 362)	273 (165 to 400)	251 (153 to 368)	227 (107 to 406)	235 (145 to 342)	185 (91·9 to 313)	-26·7 (-57·4 to 2·80)
	70+ years	113 (70·1 to 150)	96·1 (63·1 to 136)	91·8 (59·1 to 128)	76·9 (24·8 to 133)	70·1 (44·7 to 96·3)	54·0 (17·1 to 93·7)	-52·1 (-83·7 to -22·8)
Eastern Sub-Saharan Africa	All Ages	269000 (224000 to 305000)	234000 (190000 to 270000)	221000 (180000 to 258000)	214000 (155000 to 272000)	223000 (183000 to 259000)	194000 (142000 to 243000)	-27·7 (-46·4 to -14·9)
	15-49 years	126000 (104000 to 147000)	108000 (87800 to 128000)	103000 (84900 to 121000)	99800 (74300 to 125000)	107000 (87200 to 127000)	95200 (70800 to 117000)	-24·3 (-42·3 to -11·9)
	50-69 years	67800 (55900 to 77200)	64200 (50800 to 75000)	58600 (45900 to 69900)	56800 (36300 to 74600)	60500 (48100 to 71600)	49300 (31900 to 63600)	-27·2 (-52·7 to -8·15)
	70+ years	38900 (32100 to 44000)	37800 (31200 to 44300)	35600 (29100 to 41600)	33500 (21500 to 42400)	30900 (25900 to 37700)	25900 (15800 to 33500)	-33·5 (-58·9 to -15·2)
Burundi	All Ages	8640 (7200 to 10700)	7750 (5870 to 9750)	7320 (5600 to 9260)	6540 (3530 to 9050)	7290 (5600 to 9290)	5880 (3180 to 8080)	-31·6 (-65·1 to -6·46)
	15-49 years	3450 (2870 to 4400)	3010 (2330 to 3780)	2840 (2200 to 3610)	2480 (1250 to 3490)	2980 (2310 to 3750)	2340 (1190 to 3270)	-31·7 (-68·3 to -3·87)
	50-69 years	2480 (1930 to 3050)	2530 (1790 to 3410)	2320 (1640 to 3170)	2050 (902 to 3150)	2350 (1680 to 3140)	1760 (766 to 2670)	-28·3 (-70·4 to 3·75)
	70+ years	1370 (1050 to 1720)	1450 (986 to 1930)	1400 (932 to 1880)	1250 (646 to 1780)	1200 (832 to 1660)	1010 (508 to 1490)	-25·8 (-62·1 to -3·39)
Comoros	All Ages	327 (238 to 420)	311 (226 to 431)	284 (201 to 399)	301 (205 to 425)	276 (200 to 384)	245 (166 to 355)	-25·1 (-43·9 to -10·6)
	15-49 years	96·9 (68·7 to 132)	86·8 (59·6 to 129)	78·8 (52·0 to 116)	83·2 (49·8 to 127)	85·4 (58·7 to 127)	74·5 (44·3 to 112)	-23·1 (-46·4 to -5·00)
	50-69 years	106 (74·4 to 143)	106 (74·7 to 154)	96·5 (65·3 to 143)	103 (67·0 to 154)	95·8 (66·5 to 138)	84·5 (54·1 to 129)	-19·9 (-38·5 to -0·414)
	70+ years	102 (71·7 to 130)	103 (72·1 to 140)	94·2 (63·3 to 129)	100 (67·7 to 136)	80·2 (55·4 to 114)	71·2 (46·7 to 103)	-30·4 (-48·4 to -12·6)
Djibouti	All Ages	567 (307 to 778)	517 (284 to 751)	455 (252 to 653)	512 (274 to 743)	484 (265 to 695)	425 (231 to 603)	-25·1 (-37·5 to -10·4)
	15-49 years	258 (134 to 376)	214 (118 to 316)	192 (107 to 288)	212 (114 to 312)	212 (117 to 314)	188 (103 to 281)	-26·9 (-38·7 to -12·9)
	50-69 years	179 (88·3 to 265)	177 (92·4 to 261)	149 (81·5 to 216)	176 (89·8 to 257)	165 (85·8 to 238)	138 (73·6 to 201)	-22·4 (-39·9 to -0·952)
	70+ years	80·9 (41·9 to 111)	90·0 (49·9 to 126)	79·2 (44·5 to 109)	89·6 (48·6 to 125)	71·8 (40·9 to 99·2)	62·9 (34·5 to 87·0)	-22·0 (-41·0 to -2·33)
Eritrea	All Ages	5160 (3370 to 7310)	4860 (3080 to 7460)	4530 (2910 to 6840)	4560 (2700 to 6770)	4590 (2900 to 7030)	4030 (2410 to 6030)	-21·8 (-36·6 to -3·51)

eTable 4. Risk deleted deaths due to all-form tuberculosis for alcohol use, smoking, and diabetes, and all three risk factors combined by age groups for 204 countries and territories (2020) with percent change between 2015 observed deaths and 2020 all-risk deleted deaths.

Location	Age group	2015 Observed deaths	2020 Observed deaths	2020 Smoking deleted deaths	2020 Alcohol deleted deaths	2020 Diabetes deleted deaths	2020 All-risk deleted deaths	Percent Change
	15-49 years	2240 (1390 to 3350)	2040 (1210 to 3360)	1860 (1090 to 3080)	1880 (963 to 2900)	2020 (1200 to 3300)	1700 (893 to 2590)	-23·9 (-43·3 to -4·28)
	50-69 years	1670 (1100 to 2370)	1680 (1100 to 2460)	1550 (1020 to 2230)	1570 (954 to 2350)	1570 (1010 to 2260)	1360 (814 to 2020)	-18·7 (-37·9 to 2·29)
	70+ years	768 (474 to 1130)	755 (465 to 1130)	737 (458 to 1110)	729 (422 to 1060)	622 (384 to 923)	587 (345 to 862)	-23·4 (-36·0 to -5·13)
Ethiopia	All Ages	40000 (35800 to 44000)	35000 (30700 to 40000)	33800 (29000 to 38900)	31100 (20300 to 39000)	32800 (28500 to 37900)	28200 (18100 to 35600)	-29·4 (-54·6 to -12·2)
	15-49 years	14600 (12900 to 16600)	12400 (10500 to 14700)	12000 (9880 to 14100)	10900 (6700 to 14000)	12300 (10400 to 14600)	10500 (6430 to 13600)	-28·1 (-56·5 to -8·01)
	50-69 years	10500 (9080 to 11800)	9730 (8290 to 11500)	9220 (7490 to 11000)	8390 (4600 to 11400)	9240 (7890 to 11000)	7580 (4000 to 10200)	-27·4 (-61·7 to -1·74)
	70+ years	10300 (9070 to 11600)	9630 (7960 to 11300)	9330 (7630 to 10900)	8560 (5650 to 10900)	8000 (6440 to 9740)	6910 (4360 to 8910)	-32·8 (-57·5 to -14·7)
Kenya	All Ages	43100 (27500 to 52700)	35100 (20400 to 43800)	32500 (19000 to 40700)	30300 (17800 to 43700)	33100 (19300 to 41300)	26900 (16000 to 39000)	-37·4 (-61·3 to -17·6)
	15-49 years	21000 (13300 to 25800)	15600 (9090 to 19300)	14700 (8740 to 18300)	13900 (8520 to 18800)	15500 (9020 to 19200)	13100 (8210 to 18000)	-37·6 (-56·8 to -20·6)
	50-69 years	12400 (7810 to 15100)	11200 (6620 to 13900)	9960 (5990 to 12900)	9240 (4390 to 14100)	10500 (6260 to 13100)	7870 (3620 to 11700)	-36·0 (-71·1 to -8·29)
	70+ years	6270 (3780 to 8590)	6320 (3610 to 8830)	5840 (3360 to 8080)	5220 (2160 to 8430)	5110 (2910 to 7210)	3920 (1460 to 6430)	-37·1 (-75·4 to -8·63)
Madagascar	All Ages	11300 (8470 to 14800)	10800 (7690 to 14600)	10200 (7260 to 13700)	9870 (6130 to 14000)	10200 (7340 to 13900)	8840 (5470 to 12600)	-21·5 (-43·1 to -4·19)
	15-49 years	4350 (3230 to 5700)	4260 (3020 to 5810)	3990 (2760 to 5350)	3810 (2130 to 5320)	4220 (2980 to 5750)	3540 (1980 to 5040)	-18·4 (-45·9 to 3·37)
	50-69 years	3590 (2550 to 4950)	3720 (2550 to 5250)	3470 (2420 to 4920)	3360 (1930 to 4770)	3470 (2410 to 4950)	2930 (1710 to 4250)	-18·0 (-44·0 to 4·90)
	70+ years	1750 (1300 to 2420)	1770 (1290 to 2490)	1700 (1210 to 2360)	1650 (1090 to 2340)	1450 (1020 to 2020)	1310 (810 to 1830)	-25·1 (-46·5 to -2·48)
Malawi	All Ages	15200 (11700 to 18400)	14600 (10800 to 18000)	13700 (10100 to 16700)	13700 (9810 to 17800)	14200 (10600 to 17400)	12700 (9290 to 16000)	-16·4 (-30·6 to -3·12)
	15-49 years	7590 (5980 to 9590)	7670 (5730 to 9530)	7360 (5580 to 9200)	7230 (5370 to 9170)	7640 (5710 to 9500)	6980 (5280 to 8720)	-7·73 (-24·3 to 9·06)
	50-69 years	3340 (2370 to 4370)	3800 (2690 to 5040)	3460 (2440 to 4560)	3500 (2240 to 5010)	3680 (2650 to 4850)	3130 (2080 to 4400)	-6·27 (-28·6 to 16·7)
	70+ years	1610 (1100 to 2120)	1710 (1200 to 2440)	1530 (1080 to 2050)	1550 (869 to 2230)	1450 (1060 to 2050)	1200 (686 to 1700)	-25·0 (-49·1 to -3·84)
Mozambique	All Ages	44900 (38700 to 51400)	37500 (30000 to 44500)	35700 (28800 to 42900)	35500 (27900 to 42600)	36100 (29200 to 43000)	32900 (25800 to 40200)	-26·6 (-38·7 to -15·1)
	15-49 years	24500 (21600 to 27600)	21200 (16900 to 24500)	20500 (16400 to 24000)	20200 (16000 to 24000)	21100 (16900 to 24300)	19600 (15500 to 23300)	-20·1 (-33·1 to -8·55)
	50-69 years	9690 (7530 to 12000)	7680 (5700 to 9710)	6930 (5120 to 9040)	6950 (4490 to 9000)	7160 (5420 to 9120)	5900 (3790 to 8000)	-39·0 (-58·8 to -23·0)

eTable 4. Risk deleted deaths due to all-form tuberculosis for alcohol use, smoking, and diabetes, and all three risk factors combined by age groups for 204 countries and territories (2020) with percent change between 2015 observed deaths and 2020 all-risk deleted deaths.

Location	Age group	2015 Observed deaths	2020 Observed deaths	2020 Smoking deleted deaths	2020 Alcohol deleted deaths	2020 Diabetes deleted deaths	2020 All-risk deleted deaths	Percent Change
	70+ years	4130 (3140 to 5100)	3670 (2550 to 4620)	3430 (2310 to 4320)	3410 (2120 to 4490)	2950 (2030 to 3830)	2560 (1600 to 3410)	-37·9 (-59·3 to -21·3)
Rwanda	All Ages	5510 (4290 to 6920)	5180 (3940 to 6900)	4620 (3340 to 5940)	4460 (2480 to 6620)	4940 (3730 to 6640)	3870 (2240 to 5670)	-29·8 (-55·1 to -6·88)
	15-49 years	2500 (1970 to 3180)	2250 (1720 to 3060)	2110 (1630 to 2780)	1950 (1200 to 2840)	2240 (1710 to 3040)	1840 (1180 to 2680)	-26·0 (-49·5 to -4·25)
	50-69 years	1620 (1220 to 2130)	1710 (1230 to 2460)	1440 (933 to 1990)	1430 (650 to 2320)	1640 (1180 to 2350)	1180 (574 to 1910)	-27·4 (-60·8 to 5·85)
	70+ years	868 (646 to 1110)	923 (679 to 1290)	763 (494 to 1070)	785 (347 to 1200)	769 (549 to 1050)	546 (232 to 839)	-37·0 (-69·7 to -4·86)
Somalia	All Ages	18200 (11600 to 25800)	18800 (11700 to 26400)	17400 (11000 to 24600)	18800 (11700 to 26400)	17600 (11000 to 24500)	16300 (10300 to 23100)	-10·3 (-22·0 to 1·50)
	15-49 years	6880 (4090 to 10000)	7840 (4520 to 11500)	7140 (4270 to 10400)	7840 (4520 to 11500)	7730 (4460 to 11300)	7040 (4210 to 10200)	2·68 (-12·1 to 21·4)
	50-69 years	5940 (3700 to 8550)	5750 (3530 to 8280)	5150 (3280 to 7330)	5750 (3530 to 8280)	5270 (3250 to 7600)	4720 (2960 to 6740)	-20·3 (-34·9 to -9·05)
	70+ years	2040 (1120 to 3110)	2390 (1310 to 3760)	2240 (1260 to 3410)	2390 (1310 to 3760)	1820 (955 to 2840)	1690 (909 to 2540)	-16·8 (-32·4 to -0·486)
South Sudan	All Ages	6840 (5040 to 9270)	6470 (4470 to 8940)	6040 (4240 to 8510)	6420 (4470 to 8940)	6120 (4200 to 8480)	5690 (4000 to 8100)	-16·7 (-28·6 to -3·45)
	15-49 years	2700 (1870 to 3750)	2390 (1640 to 3380)	2240 (1570 to 3210)	2370 (1630 to 3380)	2370 (1630 to 3350)	2210 (1560 to 3180)	-17·8 (-31·3 to -3·08)
	50-69 years	1640 (1120 to 2330)	1930 (1270 to 2730)	1720 (1170 to 2530)	1910 (1250 to 2730)	1810 (1200 to 2540)	1600 (1080 to 2380)	-2·38 (-21·1 to 16·7)
	70+ years	987 (718 to 1410)	973 (689 to 1420)	900 (652 to 1300)	968 (683 to 1420)	774 (539 to 1180)	711 (501 to 1090)	-27·9 (-43·9 to -6·14)
Uganda	All Ages	26500 (22300 to 30900)	23000 (19200 to 28400)	22200 (18500 to 27200)	20600 (14500 to 27600)	22000 (18400 to 27200)	19300 (13900 to 25100)	-27·0 (-48·0 to -10·3)
	15-49 years	14100 (11600 to 17000)	12200 (10000 to 15800)	11900 (9780 to 15300)	11100 (8560 to 14400)	12200 (9970 to 15700)	10900 (8420 to 13900)	-22·6 (-42·6 to -4·30)
	50-69 years	5600 (4210 to 6810)	5510 (3990 to 7110)	5160 (3730 to 6690)	4680 (2550 to 7300)	5170 (3730 to 6640)	4180 (2350 to 6380)	-25·2 (-61·8 to 1·25)
	70+ years	3130 (2520 to 3940)	2900 (2190 to 3700)	2780 (2090 to 3600)	2470 (1250 to 3780)	2330 (1720 to 3030)	1910 (931 to 2870)	-38·9 (-71·6 to -14·0)
United Republic of Tanzania	All Ages	30600 (20900 to 39300)	24900 (16700 to 34000)	23400 (15700 to 31400)	22500 (13700 to 32500)	23900 (16200 to 32800)	20500 (12700 to 29600)	-33·0 (-49·3 to -16·9)
	15-49 years	14700 (9320 to 20000)	11400 (7330 to 16400)	10800 (6920 to 15300)	10500 (6330 to 15000)	11300 (7310 to 16400)	10000 (6110 to 14200)	-31·8 (-44·9 to -16·8)
	50-69 years	6950 (4600 to 9200)	6640 (4350 to 9330)	5980 (3880 to 8200)	5730 (3210 to 8920)	6360 (4140 to 8970)	5040 (2850 to 7750)	-27·6 (-52·5 to -1·18)
	70+ years	4480 (3170 to 5760)	4180 (2970 to 5570)	3910 (2730 to 5370)	3520 (1400 to 5340)	3510 (2500 to 4760)	2790 (1100 to 4420)	-38·0 (-70·3 to -8·14)
Zambia	All Ages	11800 (7990 to 16000)	9130 (5910 to 13300)	8920 (5750 to 13100)	8700 (5360 to 13000)	8820 (5690 to 13000)	8300 (5130 to 12300)	-30·4 (-43·4 to -17·6)

eTable 4. Risk deleted deaths due to all-form tuberculosis for alcohol use, smoking, and diabetes, and all three risk factors combined by age groups for 204 countries and territories (2020) with percent change between 2015 observed deaths and 2020 all-risk deleted deaths.

Location	Age group	2015 Observed deaths	2020 Observed deaths	2020 Smoking deleted deaths	2020 Alcohol deleted deaths	2020 Diabetes deleted deaths	2020 All-risk deleted deaths	Percent Change
	15-49 years	6840 (4460 to 9500)	5380 (3170 to 8200)	5310 (3140 to 8150)	5190 (3010 to 7940)	5360 (3160 to 8180)	5130 (2980 to 7810)	-25·7 (-38·8 to -10·4)
	50-69 years	2070 (1370 to 2890)	2030 (1290 to 2910)	1950 (1210 to 2780)	1900 (1020 to 2950)	1940 (1240 to 2810)	1760 (973 to 2640)	-15·3 (-33·7 to 5·65)
	70+ years	1020 (660 to 1370)	879 (542 to 1230)	823 (504 to 1130)	774 (337 to 1220)	684 (421 to 923)	568 (235 to 890)	-44·6 (-71·2 to -23·6)
Southern Sub-Saharan Africa	All Ages	117000 (107000 to 125000)	95700 (85600 to 104000)	89900 (78900 to 97300)	88200 (68600 to 104000)	91200 (81600 to 99900)	80800 (66100 to 93900)	-31·0 (-43·1 to -21·6)
	15-49 years	71100 (63400 to 77800)	56600 (49000 to 62000)	54400 (46800 to 59000)	53400 (44300 to 61600)	56300 (48800 to 61700)	51700 (43100 to 58900)	-27·3 (-36·9 to -19·1)
	50-69 years	29900 (27200 to 32400)	27000 (24300 to 30000)	24100 (21000 to 27600)	23800 (16400 to 30300)	24500 (22000 to 27400)	20000 (14600 to 25300)	-33·0 (-50·5 to -18·8)
	70+ years	8410 (7590 to 8960)	7680 (6910 to 8550)	6990 (6000 to 7950)	6570 (3740 to 8720)	5960 (5290 to 6920)	4710 (2640 to 6220)	-44·1 (-69·4 to -27·7)
Botswana	All Ages	3220 (2340 to 3870)	2740 (1870 to 3470)	2600 (1790 to 3270)	2610 (1680 to 3350)	2650 (1800 to 3360)	2440 (1590 to 3120)	-24·5 (-37·6 to -12·1)
	15-49 years	2110 (1550 to 2650)	1670 (1110 to 2110)	1610 (1070 to 2030)	1600 (1030 to 2040)	1660 (1110 to 2100)	1560 (1000 to 1960)	-26·4 (-38·5 to -14·4)
	50-69 years	811 (573 to 1060)	826 (559 to 1090)	762 (510 to 1010)	773 (503 to 1110)	778 (522 to 1040)	691 (454 to 982)	-15·0 (-33·5 to 5·55)
	70+ years	181 (134 to 231)	171 (127 to 214)	153 (113 to 196)	157 (92·7 to 209)	141 (102 to 178)	120 (75·2 to 161)	-33·6 (-49·6 to -13·9)
Eswatini	All Ages	2960 (2540 to 3490)	2060 (1700 to 2440)	2010 (1660 to 2340)	1950 (1600 to 2350)	1990 (1650 to 2340)	1860 (1540 to 2230)	-37·0 (-49·8 to -26·9)
	15-49 years	1980 (1700 to 2480)	1370 (1140 to 1650)	1350 (1120 to 1600)	1310 (1070 to 1610)	1370 (1140 to 1640)	1290 (1050 to 1580)	-34·3 (-47·5 to -21·3)
	50-69 years	592 (459 to 756)	464 (323 to 634)	443 (305 to 613)	422 (250 to 630)	423 (281 to 584)	374 (225 to 568)	-37·0 (-56·3 to -18·7)
	70+ years	112 (83·7 to 142)	97·8 (70·9 to 130)	92·3 (65·7 to 121)	86·7 (43·7 to 125)	74·7 (51·9 to 101)	63·1 (32·6 to 95·2)	-43·9 (-68·3 to -25·6)
Lesotho	All Ages	7980 (6910 to 9250)	6300 (5410 to 7170)	5660 (4880 to 6520)	5740 (4490 to 6980)	6040 (5210 to 6880)	5080 (4170 to 6100)	-36·1 (-50·2 to -23·6)
	15-49 years	4630 (3880 to 5630)	3530 (3040 to 4190)	3250 (2890 to 3870)	3250 (2610 to 3900)	3510 (3030 to 4170)	3040 (2540 to 3640)	-33·9 (-45·6 to -21·5)
	50-69 years	2390 (1890 to 2800)	1850 (1400 to 2380)	1560 (1180 to 2100)	1610 (966 to 2310)	1690 (1270 to 2180)	1290 (809 to 1870)	-46·0 (-68·3 to -25·9)
	70+ years	466 (345 to 580)	421 (330 to 533)	356 (257 to 471)	372 (219 to 512)	338 (264 to 425)	257 (151 to 381)	-44·8 (-67·2 to -26·1)
Namibia	All Ages	2730 (2060 to 3440)	2180 (1640 to 2800)	2070 (1550 to 2600)	1950 (1220 to 2730)	2080 (1550 to 2640)	1800 (1180 to 2460)	-33·9 (-52·0 to -20·9)
	15-49 years	1590 (1130 to 2110)	1190 (878 to 1530)	1160 (856 to 1450)	1100 (755 to 1500)	1190 (873 to 1520)	1070 (742 to 1430)	-32·4 (-47·4 to -19·7)
	50-69 years	734 (557 to 921)	696 (515 to 884)	640 (473 to 842)	598 (318 to 879)	646 (474 to 830)	526 (303 to 741)	-28·3 (-54·1 to -7·93)

eTable 4. Risk deleted deaths due to all-form tuberculosis for alcohol use, smoking, and diabetes, and all three risk factors combined by age groups for 204 countries and territories (2020) with percent change between 2015 observed deaths and 2020 all-risk deleted deaths.

Location	Age group	2015 Observed deaths	2020 Observed deaths	2020 Smoking deleted deaths	2020 Alcohol deleted deaths	2020 Diabetes deleted deaths	2020 All-risk deleted deaths	Percent Change
	70+ years	208 (165 to 283)	191 (143 to 260)	166 (117 to 224)	150 (50·1 to 261)	145 (105 to 202)	99·6 (29·2 to 172)	-52·2 (-85·6 to -25·0)
South Africa	All Ages	76900 (67800 to 81700)	61700 (54000 to 66100)	58700 (50400 to 64000)	57000 (44400 to 68000)	58900 (51000 to 63600)	52800 (43000 to 61800)	-31·4 (-42·6 to -22·6)
	15-49 years	48100 (40700 to 51600)	37600 (31500 to 40800)	36400 (30200 to 39800)	35700 (29100 to 41400)	37400 (31300 to 40700)	34800 (28800 to 40000)	-27·6 (-36·0 to -20·1)
	50-69 years	19400 (18000 to 20600)	17100 (15900 to 18400)	15500 (13900 to 17000)	15000 (10200 to 19000)	15600 (14300 to 17000)	12800 (9320 to 15800)	-33·9 (-49·7 to -19·6)
	70+ years	5510 (5130 to 5880)	4950 (4580 to 5530)	4650 (4200 to 5320)	4220 (2380 to 5700)	3840 (3480 to 4520)	3110 (1740 to 4100)	-43·6 (-67·9 to -26·9)
Zimbabwe	All Ages	23300 (18700 to 26500)	20700 (16000 to 24800)	18900 (15000 to 22100)	19000 (13700 to 23900)	19600 (15400 to 23300)	16800 (12800 to 20100)	-27·7 (-41·6 to -16·4)
	15-49 years	12700 (10800 to 14800)	11300 (9180 to 13200)	10600 (8800 to 12200)	10400 (7890 to 12600)	11200 (9120 to 13100)	9870 (7730 to 11700)	-22·3 (-36·9 to -9·39)
	50-69 years	6010 (4340 to 7670)	6050 (4180 to 7800)	5220 (3710 to 6840)	5410 (3300 to 7700)	5450 (3820 to 7220)	4360 (2800 to 6370)	-27·6 (-47·1 to -10·9)
	70+ years	1930 (1320 to 2370)	1840 (1230 to 2310)	1570 (942 to 1990)	1580 (766 to 2410)	1430 (943 to 1830)	1060 (509 to 1550)	-45·4 (-69·0 to -25·0)
Western Sub-Saharan Africa	All Ages	163000 (135000 to 195000)	138000 (113000 to 171000)	133000 (108000 to 165000)	125000 (77700 to 174000)	128000 (103000 to 161000)	112000 (69000 to 157000)	-31·7 (-52·7 to -15·1)
	15-49 years	59000 (46100 to 76200)	48800 (37100 to 63500)	46700 (34900 to 60300)	44000 (26700 to 64300)	48200 (36700 to 62900)	41900 (25300 to 60800)	-29·2 (-51·2 to -11·6)
	50-69 years	39800 (32800 to 48000)	37100 (29200 to 45500)	34500 (26500 to 42800)	31800 (16300 to 47500)	33600 (26400 to 41700)	27000 (13100 to 40400)	-32·5 (-64·6 to -7·83)
	70+ years	30600 (26000 to 34600)	27900 (22900 to 32900)	27000 (21700 to 32200)	24200 (13100 to 33400)	21900 (17200 to 26900)	18500 (9220 to 26000)	-39·9 (-67·3 to -21·4)
Benin	All Ages	2660 (1990 to 3560)	2300 (1640 to 3140)	2200 (1560 to 3000)	2070 (1100 to 3090)	2110 (1470 to 2950)	1830 (983 to 2810)	-31·4 (-54·8 to -12·7)
	15-49 years	946 (701 to 1260)	844 (606 to 1170)	808 (572 to 1110)	749 (415 to 1130)	833 (596 to 1160)	713 (400 to 1080)	-24·7 (-52·5 to 0·364)
	50-69 years	607 (447 to 772)	606 (424 to 882)	560 (372 to 813)	522 (235 to 790)	540 (381 to 799)	432 (186 to 678)	-29·1 (-62·9 to -4·97)
	70+ years	537 (413 to 678)	481 (364 to 630)	466 (349 to 609)	436 (225 to 598)	369 (278 to 500)	323 (149 to 455)	-40·0 (-64·6 to -19·4)
Burkina Faso	All Ages	7820 (6290 to 9030)	6790 (5280 to 8190)	6460 (4980 to 7980)	5780 (3000 to 8150)	6260 (4760 to 7690)	5120 (2780 to 7250)	-34·5 (-63·5 to -12·7)
	15-49 years	2670 (2140 to 3220)	2180 (1660 to 2680)	2020 (1540 to 2480)	1800 (776 to 2560)	2150 (1630 to 2640)	1660 (731 to 2370)	-37·6 (-70·9 to -13·7)
	50-69 years	2010 (1600 to 2420)	1900 (1440 to 2380)	1750 (1330 to 2290)	1520 (513 to 2360)	1710 (1300 to 2170)	1270 (454 to 1970)	-36·9 (-77·1 to -6·50)
	70+ years	1520 (1320 to 1790)	1490 (1160 to 1880)	1460 (1140 to 1830)	1240 (525 to 1850)	1180 (908 to 1520)	964 (389 to 1430)	-36·6 (-73·4 to -10·5)
Cabo Verde	All Ages	65·4 (48·6 to 101)	67·0 (46·3 to 115)	63·7 (45·3 to 110)	57·1 (27·9 to 125)	55·6 (39·3 to 94·2)	45·2 (22·3 to 99·5)	-31·2 (-62·4 to -2·60)

eTable 4. Risk deleted deaths due to all-form tuberculosis for alcohol use, smoking, and diabetes, and all three risk factors combined by age groups for 204 countries and territories (2020) with percent change between 2015 observed deaths and 2020 all-risk deleted deaths.

Location	Age group	2015 Observed deaths	2020 Observed deaths	2020 Smoking deleted deaths	2020 Alcohol deleted deaths	2020 Diabetes deleted deaths	2020 All-risk deleted deaths	Percent Change
	15-49 years	22·1 (14·4 to 36·5)	17·0 (11·0 to 29·5)	16·0 (10·7 to 27·3)	14·6 (7·46 to 30·8)	16·6 (10·7 to 28·6)	13·5 (7·10 to 28·7)	-39·0 (-64·0 to -15·5)
	50-69 years	16·4 (11·4 to 27·3)	22·7 (15·0 to 42·4)	20·8 (14·1 to 38·1)	18·5 (7·41 to 43·0)	19·4 (12·8 to 35·7)	14·7 (6·09 to 34·9)	-10·4 (-60·2 to 39·5)
	70+ years	25·4 (19·0 to 37·0)	26·2 (18·7 to 42·6)	25·7 (18·4 to 42·0)	23·0 (11·6 to 46·5)	18·6 (13·2 to 30·2)	16·0 (8·06 to 31·4)	-37·3 (-64·2 to -9·85)
Cameroon	All Ages	13000 (8100 to 19300)	9050 (5480 to 14000)	8700 (5200 to 13200)	8060 (4030 to 14700)	8550 (5200 to 13300)	7410 (3780 to 13200)	-43·4 (-61·7 to -23·6)
	15-49 years	6770 (4000 to 10600)	4370 (2620 to 7060)	4220 (2550 to 6660)	3960 (2010 to 7230)	4330 (2590 to 7010)	3820 (1980 to 6890)	-43·8 (-60·6 to -24·4)
	50-69 years	3080 (1800 to 4700)	2360 (1340 to 3720)	2200 (1210 to 3400)	2000 (839 to 3950)	2180 (1250 to 3420)	1740 (754 to 3250)	-43·8 (-68·9 to -20·2)
	70+ years	1440 (902 to 2060)	1230 (778 to 1860)	1190 (739 to 1780)	1010 (310 to 1880)	949 (590 to 1460)	759 (217 to 1430)	-47·9 (-81·7 to -22·9)
Chad	All Ages	7860 (5950 to 9530)	7660 (5710 to 9850)	7280 (5460 to 9210)	6940 (4440 to 9600)	7120 (5290 to 9090)	6190 (4080 to 8560)	-21·0 (-48·3 to -5·40)
	15-49 years	2680 (2030 to 3480)	2660 (1960 to 3620)	2520 (1840 to 3460)	2370 (1440 to 3420)	2630 (1930 to 3570)	2230 (1370 to 3220)	-16·5 (-49·0 to 3·92)
	50-69 years	1890 (1310 to 2340)	1990 (1360 to 2640)	1830 (1310 to 2400)	1720 (870 to 2570)	1790 (1230 to 2380)	1430 (681 to 2140)	-24·3 (-62·0 to 0·544)
	70+ years	1320 (916 to 1710)	1260 (860 to 1610)	1190 (838 to 1510)	1110 (567 to 1610)	962 (645 to 1250)	800 (402 to 1200)	-39·2 (-70·1 to -20·8)
Côte d'Ivoire	All Ages	10100 (7250 to 13700)	7100 (4780 to 9830)	6620 (4510 to 9220)	6060 (2950 to 10600)	6590 (4420 to 9250)	5330 (2660 to 9180)	-47·5 (-68·8 to -27·0)
	15-49 years	4730 (3270 to 6730)	3120 (2090 to 4490)	2890 (1910 to 4120)	2680 (1250 to 4700)	3070 (2050 to 4450)	2490 (1210 to 4250)	-47·6 (-68·7 to -27·3)
	50-69 years	2520 (1720 to 3480)	2030 (1280 to 2970)	1820 (1200 to 2660)	1640 (657 to 3140)	1830 (1160 to 2670)	1350 (542 to 2410)	-46·9 (-75·8 to -22·2)
	70+ years	1270 (918 to 1610)	1100 (808 to 1560)	1050 (753 to 1510)	884 (303 to 1490)	834 (582 to 1210)	639 (196 to 1090)	-50·1 (-84·1 to -25·1)
Gambia	All Ages	751 (581 to 1110)	778 (549 to 1160)	730 (512 to 1110)	699 (382 to 1080)	714 (496 to 1060)	610 (339 to 973)	-19·0 (-46·1 to 0·133)
	15-49 years	319 (226 to 481)	338 (226 to 507)	318 (214 to 489)	309 (181 to 483)	334 (224 to 503)	290 (174 to 462)	-9·20 (-34·2 to 10·0)
	50-69 years	183 (132 to 274)	207 (139 to 323)	186 (124 to 292)	178 (81·5 to 284)	186 (123 to 291)	146 (67·2 to 249)	-20·1 (-59·8 to 6·83)
	70+ years	168 (115 to 239)	169 (112 to 255)	162 (108 to 243)	149 (78·2 to 242)	130 (84·0 to 200)	110 (54·4 to 180)	-35·2 (-62·4 to -15·0)
Ghana	All Ages	14700 (11800 to 18500)	12700 (9760 to 16900)	12100 (9260 to 16000)	11200 (6540 to 16900)	11700 (8970 to 15700)	9950 (5900 to 14800)	-32·7 (-54·1 to -14·3)
	15-49 years	6730 (5150 to 8520)	5650 (4330 to 7630)	5460 (4130 to 7430)	5100 (2830 to 7610)	5590 (4270 to 7570)	4910 (2750 to 7370)	-27·2 (-45·7 to -9·96)
	50-69 years	4120 (3100 to 5450)	3880 (2880 to 5430)	3600 (2570 to 5050)	3260 (1650 to 5310)	3490 (2600 to 4910)	2760 (1420 to 4390)	-33·2 (-62·1 to -7·43)

eTable 4. Risk deleted deaths due to all-form tuberculosis for alcohol use, smoking, and diabetes, and all three risk factors combined by age groups for 204 countries and territories (2020) with percent change between 2015 observed deaths and 2020 all-risk deleted deaths.

Location	Age group	2015 Observed deaths	2020 Observed deaths	2020 Smoking deleted deaths	2020 Alcohol deleted deaths	2020 Diabetes deleted deaths	2020 All-risk deleted deaths	Percent Change
	70+ years	2640 (2080 to 3310)	2530 (1820 to 3330)	2400 (1710 to 3150)	2160 (1170 to 3240)	1990 (1450 to 2760)	1620 (843 to 2380)	-39·0 (-67·5 to -17·8)
Guinea	All Ages	5550 (4280 to 6720)	4550 (3320 to 6060)	4290 (3210 to 5710)	4300 (3140 to 6120)	4240 (3090 to 5680)	3830 (2860 to 5430)	-31·1 (-45·6 to -18·5)
	15-49 years	2060 (1540 to 2650)	1740 (1330 to 2240)	1660 (1260 to 2140)	1660 (1180 to 2220)	1730 (1310 to 2220)	1580 (1150 to 2090)	-23·2 (-38·7 to -9·57)
	50-69 years	1400 (1040 to 1720)	1240 (882 to 1650)	1120 (817 to 1420)	1140 (746 to 1620)	1140 (814 to 1520)	966 (639 to 1340)	-30·9 (-49·5 to -14·7)
	70+ years	1210 (918 to 1560)	974 (714 to 1320)	923 (671 to 1250)	908 (635 to 1260)	786 (528 to 1090)	697 (452 to 1000)	-42·3 (-57·7 to -24·7)
Guinea-Bissau	All Ages	1410 (1060 to 1830)	1420 (1050 to 1870)	1370 (1020 to 1840)	1300 (879 to 1750)	1340 (981 to 1800)	1200 (816 to 1650)	-14·6 (-36·5 to 3·79)
	15-49 years	697 (496 to 983)	744 (500 to 1080)	723 (484 to 1060)	690 (451 to 975)	738 (495 to 1070)	668 (439 to 965)	-3·95 (-26·8 to 15·4)
	50-69 years	359 (261 to 445)	361 (266 to 444)	340 (244 to 421)	317 (169 to 439)	326 (241 to 402)	272 (150 to 380)	-24·2 (-55·8 to -0·742)
	70+ years	159 (122 to 191)	147 (113 to 176)	143 (109 to 175)	128 (62·7 to 170)	109 (82·5 to 139)	92·4 (44·7 to 130)	-41·9 (-70·4 to -22·6)
Liberia	All Ages	1070 (716 to 1660)	925 (595 to 1610)	871 (567 to 1530)	781 (351 to 1660)	825 (534 to 1440)	661 (291 to 1380)	-39·0 (-67·3 to -15·3)
	15-49 years	433 (282 to 692)	388 (235 to 677)	364 (224 to 645)	319 (123 to 698)	378 (231 to 658)	294 (115 to 633)	-32·4 (-65·5 to 1·32)
	50-69 years	260 (174 to 389)	252 (155 to 429)	228 (147 to 384)	205 (68·5 to 465)	216 (134 to 364)	159 (52·4 to 351)	-39·4 (-77·7 to -8·36)
	70+ years	225 (153 to 361)	187 (119 to 338)	183 (117 to 331)	160 (69·7 to 314)	134 (85·9 to 245)	111 (40·5 to 229)	-51·1 (-80·8 to -32·8)
Mali	All Ages	6580 (5130 to 8690)	6000 (4470 to 8370)	5560 (4100 to 7800)	5660 (3920 to 8150)	5480 (4090 to 7760)	4840 (3370 to 6900)	-26·6 (-44·9 to -13·0)
	15-49 years	2140 (1620 to 2910)	1830 (1400 to 2520)	1720 (1260 to 2360)	1750 (1150 to 2490)	1790 (1360 to 2460)	1610 (1060 to 2260)	-24·6 (-41·4 to -9·98)
	50-69 years	1820 (1340 to 2500)	1720 (1250 to 2390)	1520 (1050 to 2120)	1590 (985 to 2280)	1540 (1110 to 2190)	1270 (781 to 1730)	-29·8 (-53·0 to -7·91)
	70+ years	1600 (1150 to 2090)	1550 (1060 to 2080)	1410 (985 to 1920)	1420 (839 to 2020)	1250 (886 to 1720)	1050 (569 to 1530)	-34·6 (-60·7 to -16·0)
Mauritania	All Ages	492 (348 to 759)	441 (299 to 683)	411 (279 to 641)	441 (299 to 683)	406 (272 to 639)	377 (253 to 598)	-23·6 (-34·4 to -6·53)
	15-49 years	113 (74·5 to 164)	102 (65·8 to 158)	88·5 (56·5 to 143)	102 (65·8 to 158)	100 (64·7 to 156)	87·2 (55·5 to 141)	-22·9 (-35·8 to -7·91)
	50-69 years	144 (96·3 to 230)	141 (88·8 to 226)	128 (82·5 to 204)	141 (88·8 to 226)	130 (80·1 to 212)	118 (75·1 to 190)	-18·4 (-33·8 to 0·880)
	70+ years	175 (128 to 280)	162 (111 to 259)	158 (109 to 254)	162 (111 to 259)	140 (94·3 to 227)	136 (92·1 to 222)	-22·9 (-35·8 to -3·56)
Niger	All Ages	7420 (5360 to 10600)	7090 (4910 to 10100)	6840 (4750 to 9730)	7000 (4800 to 10100)	6550 (4450 to 9380)	6240 (4280 to 9000)	-15·9 (-27·4 to -0·873)

eTable 4. Risk deleted deaths due to all-form tuberculosis for alcohol use, smoking, and diabetes, and all three risk factors combined by age groups for 204 countries and territories (2020) with percent change between 2015 observed deaths and 2020 all-risk deleted deaths.

Location	Age group	2015 Observed deaths	2020 Observed deaths	2020 Smoking deleted deaths	2020 Alcohol deleted deaths	2020 Diabetes deleted deaths	2020 All-risk deleted deaths	Percent Change
	15-49 years	2000 (1460 to 2780)	1870 (1280 to 2640)	1760 (1220 to 2500)	1840 (1260 to 2640)	1840 (1250 to 2590)	1700 (1180 to 2430)	-15·2 (-30·1 to 4·31)
	50-69 years	1900 (1320 to 2720)	1930 (1230 to 2670)	1840 (1170 to 2570)	1890 (1190 to 2660)	1730 (1110 to 2410)	1610 (1040 to 2300)	-15·0 (-32·0 to 7·31)
	70+ years	1300 (882 to 1920)	1440 (931 to 2000)	1390 (909 to 1940)	1420 (918 to 2000)	1140 (733 to 1630)	1090 (706 to 1580)	-15·5 (-31·1 to 1·28)
Nigeria	All Ages	73200 (59500 to 88300)	62500 (47500 to 78400)	60900 (46300 to 76500)	56000 (34100 to 80600)	58300 (44100 to 74400)	51300 (31200 to 74200)	-29·9 (-53·6 to -9·84)
	15-49 years	22500 (17200 to 28400)	19600 (13600 to 26300)	19000 (13200 to 26000)	17600 (10200 to 26500)	19400 (13400 to 26100)	17000 (9830 to 25500)	-24·4 (-50·7 to -0·742)
	50-69 years	16700 (12800 to 20700)	15800 (11500 to 19700)	15000 (10700 to 19100)	13200 (5450 to 20500)	14500 (10600 to 18600)	11600 (4520 to 18700)	-30·3 (-70·5 to 5·23)
	70+ years	14900 (11600 to 17500)	13100 (10400 to 15900)	12800 (10300 to 15700)	11100 (5350 to 15800)	10400 (8020 to 13300)	8670 (3990 to 12900)	-41·8 (-71·9 to -20·9)
Sao Tome and Principe	All Ages	22·1 (16·7 to 32·8)	19·1 (13·7 to 31·6)	18·0 (12·7 to 29·7)	15·4 (6·09 to 29·4)	17·0 (12·4 to 27·7)	13·0 (5·03 to 25·3)	-41·6 (-75·6 to -10·5)
	15-49 years	6·21 (4·51 to 9·32)	5·30 (3·23 to 8·85)	4·95 (3·06 to 8·30)	4·23 (1·40 to 8·60)	5·19 (3·18 to 8·67)	3·89 (1·28 to 7·73)	-38·3 (-75·3 to 0·850)
	50-69 years	7·27 (5·22 to 11·0)	7·24 (5·06 to 12·1)	6·64 (4·63 to 11·0)	5·76 (2·06 to 12·0)	6·52 (4·62 to 10·9)	4·78 (1·71 to 10·3)	-34·5 (-75·3 to 5·62)
	70+ years	7·86 (5·49 to 11·6)	6·19 (4·15 to 9·75)	6·05 (4·07 to 9·55)	5·09 (2·27 to 9·79)	4·89 (3·32 to 7·51)	3·95 (1·69 to 7·86)	-49·9 (-78·4 to -28·0)
Senegal	All Ages	3760 (3040 to 4680)	3300 (2520 to 4240)	3080 (2330 to 3920)	3220 (2410 to 4210)	2820 (2090 to 3670)	2570 (1830 to 3390)	-31·8 (-46·7 to -21·2)
	15-49 years	1300 (1000 to 1690)	1080 (815 to 1520)	984 (740 to 1340)	1050 (775 to 1490)	1050 (794 to 1480)	941 (670 to 1300)	-27·8 (-42·6 to -11·4)
	50-69 years	1080 (819 to 1420)	1020 (742 to 1370)	915 (674 to 1210)	987 (714 to 1330)	846 (614 to 1180)	740 (512 to 992)	-31·3 (-48·5 to -16·2)
	70+ years	998 (781 to 1260)	977 (714 to 1340)	953 (698 to 1310)	960 (700 to 1340)	691 (473 to 978)	663 (452 to 951)	-33·8 (-47·2 to -17·7)
Sierra Leone	All Ages	3530 (2810 to 4320)	2950 (2190 to 3800)	2770 (2060 to 3670)	2630 (1580 to 3600)	2730 (2030 to 3560)	2300 (1430 to 3200)	-35·1 (-59·2 to -18·0)
	15-49 years	1310 (1010 to 1700)	1080 (813 to 1490)	1000 (725 to 1420)	951 (559 to 1380)	1070 (800 to 1470)	882 (534 to 1300)	-32·6 (-57·2 to -13·6)
	50-69 years	816 (580 to 1040)	757 (539 to 1020)	669 (466 to 915)	632 (279 to 998)	679 (475 to 941)	504 (211 to 793)	-38·1 (-72·8 to -12·5)
	70+ years	681 (531 to 862)	608 (463 to 790)	588 (445 to 762)	533 (283 to 715)	471 (360 to 635)	400 (208 to 543)	-41·1 (-69·8 to -21·9)
Togo	All Ages	3390 (2640 to 4610)	2700 (2010 to 3970)	2450 (1810 to 3720)	2430 (1380 to 3910)	2550 (1890 to 3780)	2110 (1240 to 3330)	-38·1 (-60·9 to -19·9)
	15-49 years	1630 (1180 to 2310)	1160 (824 to 1680)	1080 (776 to 1600)	1050 (598 to 1670)	1150 (817 to 1670)	973 (566 to 1510)	-40·1 (-60·0 to -25·3)
	50-69 years	947 (714 to 1370)	874 (591 to 1390)	735 (481 to 1160)	757 (372 to 1340)	812 (552 to 1300)	597 (299 to 1070)	-37·2 (-68·5 to -9·13)

eTable 4. Risk deleted deaths due to all-form tuberculosis for alcohol use, smoking, and diabetes, and all three risk factors combined by age groups for 204 countries and territories (2020) with percent change between 2015 observed deaths and 2020 all-risk deleted deaths.

Location	Age group	2015 Observed deaths	2020 Observed deaths	2020 Smoking deleted deaths	2020 Alcohol deleted deaths	2020 Diabetes deleted deaths	2020 All-risk deleted deaths	Percent Change
	70+ years	455 (359 to 589)	445 (335 to 638)	415 (316 to 606)	403 (230 to 621)	372 (279 to 536)	314 (180 to 472)	-31·2 (-60·3 to -11·4)

eTable 5. Risk deleted deaths due to all-form tuberculosis for alcohol use, smoking, and diabetes, and all three risk factors combined by age groups for 204 countries and territories in (2021) with percent change between 2015 observed deaths and 2021 all-risk deleted deaths.

Location	Age group	2015 Observed deaths	2021 Observed deaths	2021 Smoking deleted deaths	2021 Alcohol deleted deaths	2021 Diabetes deleted deaths	2021 All-risk deleted deaths	Percent Change
**Global**	**All Ages**	**1570000 (1450000 to 1700000)**	**1350000 (1230000 to 1520000)**	**1190000 (1020000 to 1380000)**	**1210000 (869000 to 1540000)**	**1200000 (1080000 to 1370000)**	**966000 (684000 to 1210000)**	**-38·6 (-56·4 to -24·0)**
	**15-49 years**	**593000 (534000 to 650000)**	**491000 (439000 to 546000)**	**446000 (392000 to 498000)**	**442000 (318000 to 562000)**	**481000 (428000 to 535000)**	**398000 (290000 to 495000)**	**-32·8 (-50·1 to -18·7)**
	**50-69 years**	**499000 (467000 to 528000)**	**448000 (404000 to 510000)**	**372000 (307000 to 447000)**	**393000 (252000 to 523000)**	**388000 (347000 to 447000)**	**288000 (179000 to 388000)**	**-42·3 (-63·5 to -24·2)**
	**70+ years**	**347000 (322000 to 367000)**	**331000 (302000 to 379000)**	**290000 (250000 to 339000)**	**299000 (207000 to 381000)**	**252000 (226000 to 309000)**	**200000 (126000 to 264000)**	**-42·4 (-62·8 to -25·8)**
Central Europe, Eastern Europe, and Central Asia	All Ages	26900 (25900 to 28600)	15900 (14600 to 17300)	11400 (8770 to 14700)	12500 (5280 to 20200)	14500 (13300 to 16000)	8600 (4030 to 13700)	-68·0 (-85·2 to -49·2)
	15-49 years	12400 (11600 to 13900)	6900 (6260 to 7800)	4990 (3960 to 6530)	5440 (2270 to 8890)	6740 (6090 to 7620)	4020 (1950 to 6540)	-67·7 (-84·3 to -48·7)
	50-69 years	11000 (10800 to 11300)	6410 (5850 to 7030)	4160 (2720 to 5800)	4840 (1450 to 8530)	5640 (5060 to 6310)	2820 (741 to 5060)	-74·4 (-93·4 to -53·7)
	70+ years	2680 (2550 to 2760)	2150 (2040 to 2270)	1870 (1640 to 2110)	1860 (1100 to 2470)	1760 (1650 to 1940)	1360 (805 to 1850)	-49·3 (-70·4 to -32·4)
Central Asia	All Ages	6430 (6060 to 6820)	4430 (3960 to 4950)	3400 (2780 to 4190)	3650 (1800 to 5030)	4070 (3610 to 4560)	2650 (1410 to 3860)	-58·7 (-78·0 to -39·4)
	15-49 years	3260 (3060 to 3470)	2120 (1870 to 2400)	1600 (1310 to 1980)	1680 (714 to 2530)	2070 (1830 to 2340)	1280 (589 to 1990)	-60·8 (-81·8 to -39·2)
	50-69 years	2100 (1980 to 2230)	1570 (1370 to 1770)	1110 (838 to 1470)	1260 (498 to 1790)	1330 (1160 to 1500)	770 (285 to 1210)	-63·3 (-86·4 to -41·3)
	70+ years	522 (479 to 560)	400 (358 to 449)	351 (305 to 405)	367 (248 to 436)	329 (291 to 372)	268 (178 to 341)	-48·5 (-65·5 to -34·8)
Armenia	All Ages	76·5 (69·5 to 83·3)	40·1 (35·5 to 45·3)	25·5 (18·8 to 34·3)	32·8 (14·3 to 47·9)	36·0 (31·8 to 41·1)	19·5 (9·17 to 30·5)	-74·4 (-87·8 to -60·7)
	15-49 years	26·0 (24·2 to 28·0)	16·5 (14·8 to 18·3)	10·6 (7·93 to 14·0)	13·0 (4·99 to 20·8)	16·0 (14·3 to 17·9)	8·60 (3·89 to 13·3)	-66·9 (-84·5 to -50·8)
	50-69 years	34·6 (31·5 to 37·7)	14·4 (12·6 to 16·4)	7·74 (4·63 to 12·0)	11·3 (3·76 to 17·5)	12·2 (10·4 to 14·2)	5·22 (1·36 to 10·1)	-84·9 (-95·8 to -71·1)
	70+ years	14·3 (12·4 to 16·1)	8·51 (7·41 to 9·57)	6·43 (4·70 to 8·05)	7·72 (4·44 to 9·55)	7·00 (5·95 to 8·21)	4·93 (2·62 to 6·85)	-65·6 (-80·3 to -50·2)
Azerbaijan	All Ages	678 (539 to 934)	416 (307 to 612)	281 (187 to 407)	322 (120 to 604)	383 (283 to 560)	206 (83·6 to 372)	-69·5 (-88·7 to -50·1)
	15-49 years	357 (277 to 486)	228 (159 to 337)	153 (95·0 to 223)	168 (40·3 to 351)	222 (154 to 329)	113 (28·1 to 222)	-68·3 (-92·4 to -43·5)
	50-69 years	224 (167 to 329)	121 (79·5 to 190)	69·7 (38·2 to 113)	90·7 (27·1 to 168)	101 (65·6 to 165)	43·9 (8·50 to 90·4)	-80·1 (-96·9 to -62·6)
	70+ years	55·5 (41·6 to 75·5)	43·1 (31·1 to 62·0)	33·1 (22·6 to 46·6)	38·1 (22·5 to 58·8)	34·3 (24·9 to 48·8)	23·7 (13·8 to 37·3)	-56·7 (-76·1 to -39·8)
Georgia	All Ages	199 (180 to 219)	114 (99·9 to 131)	71·4 (46·6 to 102)	88·0 (32·2 to 146)	98·0 (85·9 to 114)	48·5 (17·9 to 81·2)	-75·6 (-91·1 to -59·3)

eTable 5. Risk deleted deaths due to all-form tuberculosis for alcohol use, smoking, and diabetes, and all three risk factors combined by age groups for 204 countries and territories in (2021) with percent change between 2015 observed deaths and 2021 all-risk deleted deaths.

Location	Age group	2015 Observed deaths	2021 Observed deaths	2021 Smoking deleted deaths	2021 Alcohol deleted deaths	2021 Diabetes deleted deaths	2021 All-risk deleted deaths	Percent Change
	15-49 years	71·0 (66·5 to 76·2)	36·7 (32·8 to 40·9)	22·7 (15·5 to 31·8)	27·6 (8·93 to 49·1)	35·7 (31·7 to 40·0)	17·5 (5·74 to 30·8)	-75·4 (-91·8 to -57·0)
	50-69 years	87·2 (74·5 to 99·3)	48·6 (40·9 to 57·7)	26·1 (13·4 to 42·2)	35·6 (9·84 to 64·3)	39·7 (33·8 to 47·8)	15·6 (2·27 to 30·6)	-82·0 (-97·4 to -64·5)
	70+ years	38·5 (33·2 to 44·2)	27·8 (23·6 to 31·8)	21·4 (16·0 to 27·1)	23·7 (12·0 to 32·8)	21·4 (18·0 to 25·8)	14·3 (7·05 to 21·2)	-62·9 (-81·3 to -47·7)
Kazakhstan	All Ages	826 (774 to 889)	571 (501 to 635)	408 (299 to 535)	439 (148 to 709)	510 (449 to 574)	285 (80·1 to 461)	-65·4 (-90·3 to -43·2)
	15-49 years	451 (416 to 491)	275 (243 to 302)	186 (132 to 252)	200 (48·3 to 357)	266 (235 to 294)	133 (22·2 to 243)	-70·3 (-95·1 to -45·2)
	50-69 years	277 (255 to 309)	200 (169 to 230)	133 (87·5 to 183)	151 (44·6 to 249)	165 (139 to 192)	82·4 (12·9 to 143)	-70·1 (-95·6 to -45·7)
	70+ years	65·6 (61·5 to 73·2)	76·9 (68·4 to 85·7)	69·7 (60·6 to 80·4)	68·8 (42·2 to 89·3)	60·7 (53·2 to 69·5)	50·1 (30·1 to 65·5)	-23·7 (-53·5 to -0·955)
Kyrgyzstan	All Ages	470 (421 to 513)	347 (297 to 412)	236 (176 to 337)	287 (144 to 402)	327 (282 to 385)	193 (104 to 304)	-58·8 (-77·8 to -35·7)
	15-49 years	262 (233 to 289)	183 (158 to 216)	130 (102 to 178)	150 (76·4 to 213)	179 (155 to 211)	110 (61·5 to 166)	-57·9 (-76·6 to -34·8)
	50-69 years	155 (138 to 170)	127 (107 to 154)	74·1 (44·7 to 118)	102 (40·0 to 149)	114 (92·1 to 137)	53·9 (19·2 to 99·0)	-65·0 (-87·8 to -35·0)
	70+ years	31·4 (28·2 to 35·1)	22·7 (19·0 to 27·6)	18·3 (14·3 to 25·0)	21·1 (14·4 to 27·2)	19·9 (16·6 to 23·9)	15·1 (10·2 to 21·3)	-52·0 (-67·9 to -34·9)
Mongolia	All Ages	549 (387 to 753)	379 (264 to 559)	231 (126 to 350)	266 (9·95 to 495)	362 (251 to 523)	153 (-24·7 to 343)	-72·0 (-104 to -38·4)
	15-49 years	322 (226 to 453)	215 (149 to 344)	129 (70·5 to 197)	145 (-15·5 to 294)	210 (146 to 327)	82·0 (-33·7 to 206)	-74·4 (-112 to -37·1)
	50-69 years	171 (116 to 232)	123 (80·4 to 172)	65·4 (26·7 to 107)	83·8 (-5·83 to 160)	114 (73·2 to 156)	37·9 (-24·3 to 99·3)	-77·5 (-116 to -41·5)
	70+ years	28·8 (20·9 to 38·2)	22·1 (16·2 to 29·0)	18·0 (12·0 to 24·4)	19·2 (9·91 to 27·2)	20·2 (14·7 to 26·5)	14·5 (7·63 to 21·2)	-49·3 (-73·3 to -27·6)
Tajikistan	All Ages	881 (683 to 1120)	756 (563 to 970)	663 (487 to 871)	693 (452 to 913)	711 (533 to 926)	582 (387 to 781)	-33·7 (-54·0 to -15·9)
	15-49 years	401 (286 to 526)	327 (231 to 437)	281 (189 to 382)	286 (144 to 404)	319 (225 to 428)	245 (125 to 343)	-38·2 (-65·1 to -13·6)
	50-69 years	238 (181 to 304)	241 (171 to 329)	198 (132 to 268)	219 (127 to 311)	211 (148 to 284)	159 (86·3 to 226)	-32·8 (-59·3 to -6·22)
	70+ years	68·6 (49·0 to 91·8)	51·6 (33·9 to 71·5)	47·6 (30·0 to 66·4)	51·0 (33·8 to 71·3)	44·6 (28·9 to 62·1)	40·9 (25·8 to 57·9)	-40·1 (-55·3 to -23·0)
Turkmenistan	All Ages	486 (456 to 520)	388 (300 to 492)	306 (235 to 387)	316 (154 to 493)	366 (283 to 462)	242 (125 to 379)	-50·2 (-73·9 to -23·7)
	15-49 years	297 (276 to 327)	232 (179 to 296)	181 (138 to 231)	184 (84·2 to 301)	226 (174 to 288)	145 (68·9 to 237)	-51·2 (-76·1 to -22·0)
	50-69 years	131 (122 to 140)	115 (85·8 to 149)	85·5 (61·7 to 112)	91·6 (37·0 to 149)	101 (75·8 to 131)	61·4 (24·0 to 102)	-53·1 (-82·0 to -21·8)

eTable 5. Risk deleted deaths due to all-form tuberculosis for alcohol use, smoking, and diabetes, and all three risk factors combined by age groups for 204 countries and territories in (2021) with percent change between 2015 observed deaths and 2021 all-risk deleted deaths.

Location	Age group	2015 Observed deaths	2021 Observed deaths	2021 Smoking deleted deaths	2021 Alcohol deleted deaths	2021 Diabetes deleted deaths	2021 All-risk deleted deaths	Percent Change
	70+ years	23·8 (22·0 to 25·7)	19·6 (16·0 to 23·8)	17·5 (14·4 to 21·4)	17·7 (11·2 to 23·5)	16·6 (13·2 to 20·2)	13·5 (8·49 to 18·7)	-43·1 (-63·5 to -22·8)
Uzbekistan	All Ages	2270 (2100 to 2460)	1420 (1230 to 1670)	1180 (966 to 1450)	1200 (674 to 1630)	1280 (1120 to 1520)	923 (535 to 1260)	-59·2 (-76·6 to -41·5)
	15-49 years	1070 (1000 to 1160)	613 (538 to 724)	506 (414 to 619)	509 (267 to 730)	597 (525 to 709)	421 (238 to 596)	-60·7 (-78·1 to -43·5)
	50-69 years	783 (715 to 856)	577 (485 to 689)	452 (342 to 588)	474 (211 to 672)	476 (400 to 575)	310 (143 to 474)	-60·2 (-82·2 to -37·4)
	70+ years	195 (177 to 212)	128 (109 to 149)	119 (100 to 140)	120 (87·1 to 143)	104 (87·3 to 123)	91·3 (65·0 to 111)	-53·1 (-67·6 to -41·8)
Central Europe	All Ages	2730 (2640 to 2850)	1860 (1700 to 2020)	1390 (1100 to 1730)	1410 (447 to 2440)	1570 (1420 to 1710)	892 (277 to 1680)	-67·3 (-89·8 to -37·0)
	15-49 years	667 (630 to 713)	409 (364 to 452)	267 (194 to 363)	297 (60·3 to 577)	392 (350 to 434)	193 (37·2 to 411)	-71·1 (-94·2 to -37·2)
	50-69 years	1200 (1150 to 1270)	766 (694 to 836)	513 (354 to 691)	551 (105 to 1030)	639 (575 to 706)	311 (48·4 to 676)	-74·0 (-95·9 to -41·9)
	70+ years	839 (791 to 880)	672 (618 to 726)	591 (515 to 661)	541 (235 to 816)	517 (465 to 578)	371 (150 to 582)	-55·8 (-82·0 to -31·7)
Albania	All Ages	42·6 (31·4 to 59·0)	33·0 (22·7 to 50·5)	21·1 (11·7 to 34·3)	26·7 (10·5 to 45·9)	29·2 (19·9 to 43·7)	15·4 (4·66 to 29·5)	-63·6 (-89·1 to -39·6)
	15-49 years	8·61 (6·56 to 12·1)	5·16 (3·39 to 7·99)	2·93 (1·60 to 4·69)	3·86 (0·977 to 7·70)	5·04 (3·31 to 7·75)	2·19 (0·342 to 4·62)	-74·2 (-96·3 to -51·5)
	50-69 years	14·3 (9·94 to 20·7)	8·56 (5·26 to 14·2)	4·53 (1·87 to 8·09)	6·44 (1·62 to 11·7)	7·64 (4·60 to 12·5)	3·03 (0·234 to 7·17)	-78·6 (-98·7 to -57·9)
	70+ years	18·7 (13·7 to 26·3)	18·7 (12·4 to 28·9)	13·0 (7·73 to 21·1)	15·8 (7·01 to 27·3)	15·9 (10·5 to 24·4)	9·58 (3·72 to 17·3)	-48·6 (-80·9 to -22·2)
Bosnia and Herzegovina	All Ages	167 (147 to 197)	111 (88·7 to 134)	80·5 (50·3 to 109)	89·4 (39·5 to 138)	82·3 (63·1 to 103)	48·8 (17·9 to 80·8)	-70·6 (-90·3 to -52·6)
	15-49 years	23·9 (20·0 to 29·2)	11·7 (8·37 to 15·2)	6·83 (3·14 to 10·4)	8·63 (2·14 to 16·2)	11·2 (8·03 to 14·7)	4·95 (0·512 to 9·79)	-79·0 (-98·0 to -57·3)
	50-69 years	57·1 (48·3 to 69·2)	34·9 (27·1 to 45·7)	20·4 (9·24 to 31·6)	25·6 (5·20 to 47·5)	25·6 (18·6 to 35·1)	10·4 (-0·986 to 24·1)	-81·6 (-102 to -60·6)
	70+ years	85·0 (74·2 to 101)	63·5 (51·8 to 75·5)	52·8 (38·4 to 68·1)	54·6 (29·9 to 76·3)	45·0 (34·6 to 54·7)	32·9 (18·0 to 47·6)	-61·1 (-81·4 to -45·0)
Bulgaria	All Ages	123 (114 to 132)	83·6 (74·7 to 93·8)	60·5 (44·9 to 77·0)	60·7 (11·9 to 116)	67·0 (57·1 to 75·3)	35·0 (5·38 to 68·1)	-71·5 (-95·7 to -42·8)
	15-49 years	21·7 (19·8 to 23·8)	13·9 (11·9 to 15·7)	7·30 (3·89 to 11·0)	9·42 (0·456 to 22·0)	13·3 (11·3 to 15·0)	4·70 (-0·584 to 11·8)	-78·4 (-103 to -46·2)
	50-69 years	58·0 (52·8 to 63·9)	34·4 (29·7 to 40·2)	21·8 (13·6 to 30·6)	24·2 (3·73 to 48·4)	28·1 (23·9 to 32·6)	12·6 (1·23 to 27·6)	-78·2 (-97·9 to -49·7)
	70+ years	42·6 (38·9 to 46·0)	35·0 (31·7 to 38·6)	31·1 (26·2 to 35·7)	26·8 (7·71 to 44·4)	25·3 (21·0 to 29·0)	17·3 (3·83 to 29·1)	-59·3 (-91·1 to -31·2)
Croatia	All Ages	77·6 (71·2 to 84·7)	53·5 (46·9 to 59·9)	40·3 (28·5 to 51·5)	41·6 (13·1 to 67·0)	41·6 (35·6 to 48·0)	24·5 (7·06 to 43·0)	-68·5 (-90·6 to -43·1)

eTable 5. Risk deleted deaths due to all-form tuberculosis for alcohol use, smoking, and diabetes, and all three risk factors combined by age groups for 204 countries and territories in (2021) with percent change between 2015 observed deaths and 2021 all-risk deleted deaths.

Location	Age group	2015 Observed deaths	2021 Observed deaths	2021 Smoking deleted deaths	2021 Alcohol deleted deaths	2021 Diabetes deleted deaths	2021 All-risk deleted deaths	Percent Change
	15-49 years	11·0 (10·3 to 11·9)	5·54 (4·76 to 6·32)	3·52 (2·21 to 4·84)	4·13 (0·965 to 7·59)	5·34 (4·63 to 6·11)	2·61 (0·631 to 4·76)	-76·2 (-94·3 to -55·4)
	50-69 years	27·1 (24·7 to 29·5)	15·8 (13·6 to 18·1)	9·98 (6·10 to 14·9)	11·3 (1·51 to 21·4)	12·5 (10·4 to 14·2)	5·46 (0·262 to 12·1)	-79·9 (-99·1 to -53·7)
	70+ years	39·1 (35·1 to 43·0)	31·9 (27·5 to 36·5)	26·5 (19·9 to 32·4)	25·9 (10·4 to 38·2)	23·5 (19·9 to 28·3)	16·2 (6·16 to 25·7)	-58·7 (-83·9 to -34·8)
Czechia	All Ages	61·6 (57·0 to 66·1)	32·8 (29·4 to 36·5)	25·2 (19·3 to 30·4)	24·4 (6·57 to 45·2)	24·8 (22·3 to 27·5)	14·4 (4·06 to 27·5)	-76·6 (-93·5 to -51·8)
	15-49 years	10·5 (9·46 to 11·5)	5·15 (4·40 to 5·99)	3·41 (2·26 to 4·60)	3·78 (0·909 to 7·62)	4·93 (4·21 to 5·78)	2·52 (0·630 to 5·06)	-76·0 (-93·9 to -49·3)
	50-69 years	23·3 (21·0 to 26·0)	9·40 (8·04 to 10·9)	6·13 (3·96 to 8·38)	6·73 (1·58 to 14·3)	7·30 (6·29 to 8·35)	3·53 (0·734 to 7·68)	-84·8 (-96·8 to -65·3)
	70+ years	27·4 (24·6 to 29·7)	18·0 (16·1 to 19·7)	15·5 (12·9 to 17·7)	13·7 (4·12 to 24·2)	12·4 (10·8 to 14·1)	8·13 (1·97 to 15·0)	-70·2 (-92·8 to -42·7)
Hungary	All Ages	117 (110 to 124)	62·4 (55·0 to 69·1)	48·6 (36·6 to 58·2)	47·5 (14·6 to 81·3)	48·4 (42·4 to 54·4)	28·9 (9·60 to 52·4)	-75·3 (-91·8 to -53·7)
	15-49 years	21·8 (19·9 to 23·8)	10·0 (8·55 to 11·6)	6·89 (4·79 to 9·17)	7·43 (2·05 to 13·9)	9·62 (8·17 to 11·2)	5·07 (1·38 to 10·0)	-76·7 (-93·2 to -53·1)
	50-69 years	49·9 (45·6 to 54·8)	25·7 (22·3 to 29·3)	18·0 (12·0 to 23·4)	18·9 (4·84 to 35·1)	20·1 (17·5 to 22·7)	10·5 (2·94 to 21·7)	-79·0 (-94·2 to -54·9)
	70+ years	44·0 (40·2 to 47·5)	26·2 (22·6 to 29·6)	23·2 (18·8 to 27·1)	20·7 (7·97 to 32·8)	18·2 (15·1 to 21·0)	12·8 (4·36 to 21·4)	-70·9 (-90·0 to -52·2)
Montenegro	All Ages	8·51 (6·91 to 10·9)	6·49 (5·02 to 8·51)	4·28 (2·83 to 6·31)	4·92 (1·19 to 9·68)	5·03 (3·94 to 6·68)	2·47 (0·307 to 4·94)	-70·9 (-96·4 to -40·1)
	15-49 years	1·54 (1·17 to 1·96)	1·05 (0·781 to 1·44)	0·564 (0·292 to 0·900)	0·761 (0·101 to 1·62)	1·00 (0·749 to 1·37)	0·392 (-0·00529 to 0·914)	-74·4 (-100 to -42·0)
	50-69 years	3·74 (2·95 to 4·94)	2·52 (1·95 to 3·48)	1·33 (0·658 to 2·28)	1·78 (0·114 to 4·05)	1·92 (1·43 to 2·69)	0·644 (-0·183 to 1·71)	-82·7 (-105 to -53·5)
	70+ years	3·17 (2·60 to 3·94)	2·88 (2·23 to 3·60)	2·36 (1·71 to 3·15)	2·34 (0·970 to 4·00)	2·06 (1·60 to 2·69)	1·40 (0·535 to 2·28)	-55·8 (-83·0 to -30·3)
North Macedonia	All Ages	58·7 (49·4 to 70·9)	33·0 (24·6 to 44·1)	23·0 (15·3 to 33·0)	25·4 (8·36 to 45·2)	24·8 (18·2 to 34·2)	13·2 (3·40 to 24·6)	-77·5 (-94·2 to -60·5)
	15-49 years	10·6 (8·80 to 12·3)	4·70 (3·50 to 6·72)	2·57 (1·46 to 3·97)	3·41 (0·773 to 6·74)	4·49 (3·34 to 6·37)	1·81 (0·235 to 3·80)	-82·8 (-97·8 to -62·8)
	50-69 years	24·4 (19·7 to 31·4)	12·1 (8·81 to 17·4)	7·17 (4·08 to 11·8)	8·92 (2·05 to 17·5)	9·05 (6·18 to 13·5)	3·91 (0·436 to 8·38)	-84·0 (-98·1 to -67·7)
	70+ years	23·3 (19·6 to 28·0)	16·1 (12·3 to 20·2)	13·1 (9·89 to 17·4)	12·9 (5·09 to 20·7)	11·1 (8·46 to 14·0)	7·32 (2·57 to 12·1)	-68·5 (-88·5 to -48·7)
Poland	All Ages	632 (612 to 652)	441 (405 to 476)	330 (237 to 421)	332 (73·3 to 600)	356 (316 to 401)	202 (34·2 to 403)	-67·9 (-94·5 to -35·9)
	15-49 years	140 (132 to 152)	94·0 (86·1 to 104)	64·9 (42·0 to 88·0)	69·0 (11·6 to 137)	89·2 (80·6 to 99·4)	46·6 (7·77 to 101)	-66·6 (-94·3 to -27·4)
	50-69 years	303 (293 to 313)	194 (175 to 212)	129 (78·8 to 182)	138 (8·55 to 275)	154 (131 to 179)	72·7 (-3·17 to 169)	-75·9 (-101 to -43·8)

eTable 5. Risk deleted deaths due to all-form tuberculosis for alcohol use, smoking, and diabetes, and all three risk factors combined by age groups for 204 countries and territories in (2021) with percent change between 2015 observed deaths and 2021 all-risk deleted deaths.

Location	Age group	2015 Observed deaths	2021 Observed deaths	2021 Smoking deleted deaths	2021 Alcohol deleted deaths	2021 Diabetes deleted deaths	2021 All-risk deleted deaths	Percent Change
	70+ years	188 (177 to 197)	153 (139 to 163)	136 (114 to 155)	124 (52·4 to 187)	113 (98·9 to 129)	82·5 (31·1 to 126)	-56·1 (-83·4 to -32·4)
Romania	All Ages	1180 (1130 to 1280)	816 (697 to 939)	599 (460 to 746)	598 (151 to 1120)	728 (618 to 829)	398 (97·3 to 774)	-66·3 (-91·5 to -33·5)
	15-49 years	367 (339 to 407)	223 (191 to 259)	142 (97·9 to 194)	159 (23·8 to 325)	215 (184 to 249)	99·8 (12·0 to 221)	-72·7 (-96·5 to -38·4)
	50-69 years	531 (493 to 580)	359 (305 to 418)	243 (169 to 319)	254 (41·2 to 505)	315 (266 to 362)	153 (21·4 to 323)	-71·2 (-95·9 to -36·7)
	70+ years	269 (249 to 289)	221 (193 to 246)	201 (175 to 223)	173 (60·5 to 282)	186 (159 to 212)	133 (44·2 to 220)	-50·4 (-83·3 to -19·9)
Serbia	All Ages	162 (139 to 185)	125 (95·9 to 157)	97·5 (70·2 to 134)	99·8 (40·1 to 167)	96·7 (77·0 to 126)	61·4 (24·9 to 104)	-62·1 (-84·3 to -35·4)
	15-49 years	30·7 (26·8 to 34·8)	22·0 (18·3 to 27·2)	16·1 (12·2 to 21·4)	18·0 (9·43 to 29·6)	21·4 (17·6 to 26·6)	13·5 (8·12 to 21·4)	-55·9 (-72·9 to -28·0)
	50-69 years	65·2 (56·0 to 74·3)	45·0 (34·6 to 55·5)	30·6 (19·2 to 43·8)	33·9 (9·66 to 62·1)	34·8 (26·4 to 44·4)	18·2 (4·76 to 33·5)	-72·1 (-92·5 to -47·4)
	70+ years	64·5 (52·9 to 75·9)	56·8 (41·9 to 74·3)	49·7 (35·9 to 66·4)	46·8 (21·3 to 75·1)	39·4 (29·3 to 53·7)	28·6 (11·4 to 47·6)	-55·7 (-81·8 to -26·4)
Slovakia	All Ages	40·2 (34·3 to 47·9)	31·7 (25·8 to 38·8)	24·3 (18·4 to 31·2)	23·6 (6·06 to 44·1)	27·1 (21·7 to 33·2)	15·6 (3·77 to 28·6)	-61·2 (-90·4 to -27·0)
	15-49 years	7·92 (6·41 to 9·53)	5·39 (4·12 to 7·07)	3·50 (2·26 to 5·06)	3·67 (0·308 to 8·24)	5·20 (3·92 to 6·89)	2·30 (-0·0243 to 5·53)	-70·7 (-100 to -29·6)
	50-69 years	17·8 (14·2 to 22·3)	12·2 (9·11 to 16·2)	8·32 (5·39 to 12·0)	8·64 (1·35 to 17·5)	10·4 (7·58 to 14·1)	4·99 (0·462 to 10·6)	-72·0 (-96·9 to -42·3)
	70+ years	14·3 (11·7 to 17·1)	13·9 (11·2 to 17·7)	12·3 (9·78 to 15·4)	11·1 (4·48 to 18·2)	11·4 (8·71 to 14·2)	8·14 (3·30 to 13·1)	-42·8 (-78·4 to -6·24)
Slovenia	All Ages	15·2 (14·0 to 16·3)	7·54 (6·50 to 8·39)	6·14 (4·81 to 7·31)	6·08 (2·60 to 9·37)	6·17 (5·23 to 7·01)	4·05 (1·54 to 6·36)	-73·4 (-90·1 to -57·2)
	15-49 years	2·10 (1·94 to 2·30)	0·693 (0·602 to 0·793)	0·443 (0·298 to 0·613)	0·535 (0·165 to 0·886)	0·670 (0·582 to 0·763)	0·337 (0·0915 to 0·600)	-83·9 (-95·4 to -70·9)
	50-69 years	5·51 (5·14 to 5·96)	2·14 (1·83 to 2·42)	1·36 (0·781 to 1·90)	1·59 (0·408 to 2·84)	1·78 (1·52 to 2·01)	0·817 (0·0635 to 1·65)	-85·2 (-98·8 to -69·3)
	70+ years	7·53 (6·70 to 8·16)	4·69 (3·98 to 5·19)	4·31 (3·63 to 4·89)	3·92 (1·97 to 5·67)	3·70 (3·06 to 4·26)	2·87 (1·29 to 4·24)	-61·9 (-83·3 to -44·4)
Eastern Europe	All Ages	17700 (16800 to 19100)	9560 (8510 to 10700)	6640 (4830 to 8730)	7490 (3070 to 12600)	8910 (7870 to 9900)	5060 (2360 to 8590)	-71·4 (-86·8 to -53·4)
	15-49 years	8520 (7680 to 9710)	4370 (3760 to 5030)	3130 (2370 to 4180)	3460 (1610 to 5760)	4280 (3660 to 4950)	2550 (1320 to 4140)	-70·0 (-84·1 to -52·9)
	50-69 years	7730 (7510 to 7950)	4070 (3600 to 4680)	2540 (1540 to 3690)	3030 (806 to 5630)	3670 (3140 to 4300)	1740 (381 to 3410)	-77·5 (-95·1 to -56·8)
	70+ years	1320 (1260 to 1360)	1070 (986 to 1160)	931 (797 to 1080)	956 (550 to 1220)	917 (822 to 1010)	718 (415 to 986)	-45·5 (-68·3 to -26·9)
Belarus	All Ages	391 (362 to 428)	245 (205 to 295)	164 (117 to 229)	185 (67·4 to 313)	230 (191 to 277)	122 (48·6 to 211)	-68·9 (-88·5 to -45·3)

eTable 5. Risk deleted deaths due to all-form tuberculosis for alcohol use, smoking, and diabetes, and all three risk factors combined by age groups for 204 countries and territories in (2021) with percent change between 2015 observed deaths and 2021 all-risk deleted deaths.

Location	Age group	2015 Observed deaths	2021 Observed deaths	2021 Smoking deleted deaths	2021 Alcohol deleted deaths	2021 Diabetes deleted deaths	2021 All-risk deleted deaths	Percent Change
	15-49 years	162 (147 to 181)	88·3 (70·5 to 104)	61·8 (46·5 to 81·6)	67·7 (26·2 to 119)	86·7 (69·2 to 103)	48·9 (21·7 to 82·1)	-69·7 (-86·3 to -48·5)
	50-69 years	188 (173 to 208)	127 (102 to 155)	77·3 (46·8 to 119)	91·6 (21·4 to 164)	117 (94·8 to 142)	52·4 (11·4 to 105)	-72·1 (-94·4 to -44·3)
	70+ years	39·0 (36·3 to 41·7)	28·6 (24·3 to 33·3)	24·1 (19·6 to 30·3)	25·0 (12·1 to 33·4)	25·7 (22·3 to 30·5)	19·1 (9·86 to 25·2)	-50·9 (-75·5 to -35·9)
Estonia	All Ages	45·3 (41·4 to 48·9)	25·3 (21·9 to 29·1)	20·2 (16·4 to 25·6)	20·8 (10·6 to 30·8)	23·1 (20·0 to 27·3)	15·8 (9·14 to 23·7)	-65·1 (-80·4 to -45·8)
	15-49 years	18·9 (15·4 to 22·0)	9·58 (7·32 to 12·8)	8·23 (5·83 to 11·1)	8·35 (5·36 to 12·7)	9·44 (7·17 to 12·7)	7·38 (4·76 to 10·9)	-61·2 (-71·0 to -48·4)
	50-69 years	18·1 (16·7 to 19·4)	10·3 (9·05 to 11·9)	7·07 (5·03 to 9·96)	7·70 (2·64 to 13·5)	9·17 (8·04 to 10·4)	4·86 (1·74 to 9·01)	-73·2 (-90·8 to -49·7)
	70+ years	8·00 (7·21 to 8·65)	5·24 (4·61 to 6·03)	4·71 (3·96 to 5·58)	4·57 (2·39 to 6·06)	4·36 (3·86 to 5·12)	3·46 (1·92 to 4·61)	-56·8 (-75·9 to -41·4)
Latvia	All Ages	95·1 (85·4 to 106)	55·2 (48·6 to 62·7)	43·6 (35·7 to 53·0)	44·1 (21·7 to 71·3)	50·5 (44·4 to 58·5)	33·3 (17·9 to 54·1)	-64·9 (-81·0 to -42·9)
	15-49 years	36·0 (30·4 to 43·9)	20·7 (16·5 to 26·1)	17·0 (12·4 to 22·2)	17·3 (10·5 to 28·1)	20·3 (16·2 to 25·6)	14·7 (9·79 to 21·9)	-59·3 (-70·7 to -39·8)
	50-69 years	43·3 (39·5 to 47·5)	23·8 (21·4 to 27·2)	16·9 (12·9 to 22·1)	17·7 (5·91 to 32·3)	21·2 (19·1 to 24·1)	11·6 (3·97 to 21·8)	-73·1 (-90·4 to -48·5)
	70+ years	15·2 (14·0 to 16·3)	10·5 (9·32 to 11·6)	9·42 (8·02 to 11·0)	8·87 (3·96 to 12·3)	8·79 (7·66 to 10·0)	6·77 (2·98 to 9·52)	-55·6 (-80·6 to -35·4)
Lithuania	All Ages	210 (198 to 224)	98·3 (87·9 to 111)	71·9 (56·7 to 92·8)	73·8 (23·9 to 128)	90·2 (81·3 to 102)	50·9 (16·7 to 92·3)	-75·7 (-91·9 to -55·6)
	15-49 years	69·5 (64·8 to 75·1)	31·9 (28·0 to 36·6)	22·3 (16·4 to 29·9)	23·2 (6·58 to 43·8)	31·0 (27·3 to 35·7)	16·4 (5·49 to 31·4)	-76·3 (-92·2 to -54·6)
	50-69 years	102 (94·1 to 109)	44·3 (39·5 to 50·8)	30·1 (20·8 to 41·2)	31·7 (6·67 to 61·8)	39·9 (35·5 to 45·5)	19·7 (3·59 to 40·1)	-80·6 (-96·4 to -59·7)
	70+ years	37·2 (34·0 to 40·4)	21·7 (19·0 to 24·2)	19·2 (16·0 to 22·0)	18·5 (8·17 to 25·4)	19·0 (16·6 to 21·4)	14·5 (6·60 to 20·2)	-61·1 (-82·6 to -47·1)
Republic of Moldova	All Ages	276 (256 to 293)	138 (124 to 154)	90·5 (62·9 to 121)	103 (27·7 to 194)	126 (113 to 141)	63·5 (20·0 to 125)	-77·0 (-93·0 to -54·8)
	15-49 years	114 (104 to 124)	53·2 (46·9 to 60·7)	34·2 (24·6 to 45·2)	39·4 (11·9 to 76·0)	51·5 (45·5 to 58·7)	25·8 (9·78 to 49·9)	-77·5 (-91·8 to -56·9)
	50-69 years	138 (127 to 149)	67·0 (59·2 to 75·9)	40·4 (23·7 to 60·5)	47·8 (7·66 to 96·0)	59·1 (52·2 to 66·7)	25·6 (3·53 to 59·3)	-81·6 (-97·5 to -57·3)
	70+ years	21·6 (19·8 to 23·3)	17·0 (15·3 to 18·7)	14·8 (12·7 to 17·0)	14·7 (7·44 to 21·0)	14·6 (12·8 to 16·4)	11·2 (5·80 to 15·4)	-47·9 (-74·0 to -29·1)
Russian Federation	All Ages	12600 (11900 to 13400)	6140 (5610 to 6840)	4170 (2890 to 5620)	4790 (2040 to 8130)	5710 (5210 to 6440)	3150 (1290 to 5380)	-74·9 (-89·4 to -57·0)
	15-49 years	6320 (5760 to 7130)	2930 (2590 to 3440)	2010 (1470 to 2650)	2280 (927 to 3950)	2860 (2530 to 3380)	1620 (789 to 2800)	-74·4 (-87·7 to -57·1)
	50-69 years	5250 (5170 to 5370)	2410 (2220 to 2670)	1450 (879 to 2160)	1790 (499 to 3310)	2160 (1980 to 2470)	991 (191 to 1980)	-81·1 (-96·4 to -62·3)

eTable 5. Risk deleted deaths due to all-form tuberculosis for alcohol use, smoking, and diabetes, and all three risk factors combined by age groups for 204 countries and territories in (2021) with percent change between 2015 observed deaths and 2021 all-risk deleted deaths.

Location	Age group	2015 Observed deaths	2021 Observed deaths	2021 Smoking deleted deaths	2021 Alcohol deleted deaths	2021 Diabetes deleted deaths	2021 All-risk deleted deaths	Percent Change
	70+ years	879 (834 to 903)	766 (718 to 818)	664 (569 to 748)	686 (420 to 866)	649 (593 to 715)	512 (319 to 669)	-41·8 (-63·4 to -23·9)
Ukraine	All Ages	4120 (3780 to 4600)	2860 (2090 to 3810)	2090 (1380 to 3060)	2270 (875 to 3960)	2680 (1950 to 3630)	1620 (603 to 2960)	-60·7 (-85·4 to -30·3)
	15-49 years	1800 (1540 to 2150)	1240 (897 to 1590)	969 (641 to 1320)	1020 (511 to 1610)	1210 (872 to 1570)	823 (435 to 1300)	-54·2 (-75·0 to -29·2)
	50-69 years	1990 (1810 to 2160)	1390 (972 to 1970)	913 (404 to 1490)	1040 (184 to 2110)	1260 (828 to 1870)	635 (53·7 to 1450)	-68·0 (-97·3 to -30·5)
	70+ years	318 (295 to 340)	226 (171 to 286)	195 (143 to 260)	198 (85·4 to 283)	196 (143 to 258)	151 (60·3 to 233)	-52·5 (-80·7 to -28·9)
High-income	All Ages	13200 (11800 to 14300)	12700 (11200 to 13900)	11000 (9040 to 12500)	10300 (4510 to 15400)	10400 (9210 to 11800)	7490 (3220 to 11300)	-43·4 (-74·8 to -14·9)
	15-49 years	1800 (1440 to 2120)	1470 (1170 to 1780)	1290 (1000 to 1550)	1290 (795 to 1820)	1440 (1140 to 1750)	1150 (754 to 1530)	-36·1 (-53·9 to -17·7)
	50-69 years	2700 (2490 to 2920)	2330 (2090 to 2520)	1830 (1430 to 2220)	1860 (826 to 2890)	2020 (1770 to 2260)	1330 (622 to 2080)	-50·7 (-76·1 to -22·8)
	70+ years	8660 (7320 to 9370)	8820 (7290 to 9740)	7820 (6100 to 9040)	7070 (2980 to 10800)	6940 (5780 to 8110)	4970 (1900 to 7770)	-42·7 (-77·6 to -12·9)
Australasia	All Ages	93·8 (84·7 to 101)	83·2 (74·8 to 90·9)	75·5 (64·2 to 85·5)	64·3 (23·3 to 107)	71·9 (63·7 to 80·2)	50·8 (17·3 to 86·0)	-45·9 (-81·6 to -10·8)
	15-49 years	7·82 (6·73 to 9·43)	6·16 (5·09 to 7·55)	5·30 (4·10 to 6·76)	5·12 (2·50 to 7·93)	6·02 (4·98 to 7·47)	4·45 (2·33 to 6·74)	-43·3 (-68·7 to -15·4)
	50-69 years	21·8 (20·3 to 23·9)	18·1 (16·3 to 19·9)	15·1 (12·1 to 17·5)	13·7 (4·74 to 23·4)	16·0 (14·2 to 17·9)	10·3 (3·35 to 18·4)	-52·7 (-84·5 to -15·2)
	70+ years	63·7 (55·4 to 69·1)	58·7 (50·6 to 64·2)	54·8 (46·7 to 61·3)	45·2 (14·7 to 75·5)	49·6 (42·4 to 55·0)	35·7 (10·3 to 60·6)	-44·0 (-83·3 to -7·53)
Australia	All Ages	77·2 (69·5 to 83·9)	77·5 (69·4 to 84·8)	70·5 (60·1 to 79·9)	59·8 (21·5 to 99·7)	67·1 (59·3 to 74·8)	47·5 (16·2 to 80·8)	-38·6 (-79·2 to 2·04)
	15-49 years	6·12 (5·25 to 7·36)	5·58 (4·63 to 6·85)	4·80 (3·71 to 6·12)	4·62 (2·27 to 7·21)	5·46 (4·54 to 6·78)	4·02 (2·12 to 6·13)	-34·4 (-63·7 to -1·16)
	50-69 years	17·1 (15·7 to 18·8)	16·4 (14·7 to 18·1)	13·8 (11·0 to 16·0)	12·4 (4·34 to 21·3)	14·6 (12·9 to 16·4)	9·46 (3·13 to 16·9)	-44·6 (-81·4 to -1·23)
	70+ years	53·7 (46·7 to 58·7)	55·2 (47·5 to 60·4)	51·7 (44·0 to 58·1)	42·4 (13·7 to 71·4)	46·8 (39·9 to 52·1)	33·7 (9·73 to 57·5)	-37·2 (-81·3 to 4·26)
New Zealand	All Ages	16·6 (15·3 to 17·6)	5·71 (5·22 to 6·19)	4·92 (4·07 to 5·56)	4·52 (1·74 to 7·12)	4·81 (4·36 to 5·29)	3·31 (1·16 to 5·24)	-80·0 (-92·9 to -67·6)
	15-49 years	1·70 (1·47 to 2·03)	0·578 (0·470 to 0·724)	0·496 (0·384 to 0·621)	0·492 (0·233 to 0·741)	0·562 (0·450 to 0·710)	0·423 (0·212 to 0·629)	-75·2 (-86·3 to -65·6)
	50-69 years	4·75 (4·49 to 5·02)	1·68 (1·54 to 1·79)	1·31 (0·985 to 1·59)	1·29 (0·404 to 2·12)	1·42 (1·29 to 1·54)	0·861 (0·215 to 1·53)	-81·9 (-95·5 to -67·5)
	70+ years	10·0 (8·94 to 10·9)	3·42 (2·95 to 3·77)	3·08 (2·51 to 3·46)	2·70 (0·927 to 4·17)	2·80 (2·49 to 3·12)	1·99 (0·568 to 3·12)	-80·2 (-94·2 to -68·0)
High-income Asia Pacific	All Ages	6210 (5260 to 6870)	6460 (5280 to 7290)	5550 (4270 to 6610)	5220 (2210 to 8080)	5220 (4300 to 6130)	3660 (1440 to 5660)	-41·2 (-76·4 to -12·5)

eTable 5. Risk deleted deaths due to all-form tuberculosis for alcohol use, smoking, and diabetes, and all three risk factors combined by age groups for 204 countries and territories in (2021) with percent change between 2015 observed deaths and 2021 all-risk deleted deaths.

Location	Age group	2015 Observed deaths	2021 Observed deaths	2021 Smoking deleted deaths	2021 Alcohol deleted deaths	2021 Diabetes deleted deaths	2021 All-risk deleted deaths	Percent Change
	15-49 years	252 (229 to 281)	170 (154 to 188)	128 (101 to 158)	129 (43·2 to 212)	164 (148 to 181)	96·2 (32·3 to 160)	-61·8 (-86·8 to -35·8)
	50-69 years	827 (766 to 919)	660 (590 to 742)	485 (363 to 625)	499 (161 to 853)	571 (502 to 659)	325 (96·0 to 561)	-60·7 (-88·6 to -32·7)
	70+ years	5120 (4220 to 5700)	5620 (4510 to 6380)	4930 (3740 to 5870)	4580 (2030 to 6960)	4480 (3600 to 5340)	3230 (1320 to 4990)	-37·0 (-73·7 to -6·71)
Brunei Darussalam	All Ages	18·0 (15·7 to 21·5)	17·6 (14·6 to 21·0)	15·2 (11·9 to 19·2)	17·3 (13·7 to 20·6)	14·0 (11·3 to 17·0)	11·9 (8·74 to 15·1)	-33·8 (-48·1 to -13·0)
	15-49 years	3·61 (3·09 to 4·33)	3·36 (2·70 to 4·07)	2·73 (2·13 to 3·47)	3·25 (2·67 to 4·01)	3·16 (2·55 to 3·84)	2·51 (1·89 to 3·22)	-30·4 (-49·6 to -6·19)
	50-69 years	4·69 (3·85 to 5·64)	5·00 (4·01 to 6·29)	3·89 (2·68 to 5·29)	4·89 (3·68 to 6·15)	3·68 (2·88 to 4·84)	2·78 (1·72 to 3·93)	-40·5 (-61·5 to -14·4)
	70+ years	9·45 (7·98 to 11·4)	9·09 (7·19 to 11·1)	8·36 (6·49 to 10·6)	9·02 (7·09 to 11·1)	6·97 (5·33 to 8·68)	6·43 (4·76 to 8·23)	-31·8 (-45·9 to -13·3)
Japan	All Ages	3450 (2880 to 3800)	3880 (3140 to 4340)	3470 (2590 to 4110)	3170 (1420 to 4670)	3130 (2520 to 3650)	2300 (967 to 3510)	-33·5 (-70·9 to -4·90)
	15-49 years	55·2 (49·6 to 60·9)	43·2 (38·7 to 46·6)	35·5 (29·3 to 42·2)	35·2 (15·9 to 54·4)	42·2 (37·6 to 45·7)	29·1 (13·9 to 44·8)	-47·3 (-73·0 to -19·7)
	50-69 years	279 (260 to 301)	211 (196 to 224)	163 (129 to 199)	169 (70·4 to 261)	188 (168 to 205)	121 (51·4 to 190)	-56·7 (-80·6 to -31·9)
	70+ years	3120 (2540 to 3450)	3630 (2880 to 4070)	3270 (2430 to 3870)	2960 (1330 to 4350)	2890 (2290 to 3390)	2150 (902 to 3280)	-31·2 (-70·0 to -1·92)
Republic of Korea	All Ages	2670 (2290 to 3030)	2500 (2080 to 2940)	2010 (1530 to 2520)	1980 (663 to 3130)	2030 (1710 to 2460)	1310 (433 to 2090)	-51·1 (-83·8 to -22·9)
	15-49 years	185 (163 to 214)	119 (104 to 135)	85·4 (65·6 to 110)	86·6 (21·7 to 151)	114 (100 to 131)	61·1 (14·2 to 110)	-67·0 (-92·2 to -41·4)
	50-69 years	522 (461 to 618)	430 (360 to 510)	307 (213 to 416)	313 (78·0 to 573)	368 (304 to 438)	192 (37·3 to 369)	-63·0 (-93·2 to -32·1)
	70+ years	1960 (1630 to 2280)	1950 (1590 to 2330)	1620 (1230 to 2020)	1580 (582 to 2430)	1550 (1280 to 1900)	1050 (394 to 1650)	-46·3 (-80·0 to -15·6)
Singapore	All Ages	70·2 (64·3 to 76·3)	56·2 (50·9 to 62·3)	51·0 (43·3 to 57·2)	52·3 (37·8 to 60·4)	44·5 (39·2 to 50·3)	37·8 (26·4 to 45·6)	-46·1 (-61·8 to -37·0)
	15-49 years	7·31 (6·44 to 8·23)	4·79 (4·19 to 5·49)	4·13 (3·32 to 4·84)	4·21 (2·45 to 5·57)	4·58 (4·02 to 5·29)	3·53 (1·96 to 4·66)	-51·8 (-71·1 to -38·8)
	50-69 years	21·0 (18·4 to 23·8)	13·1 (11·7 to 14·7)	11·1 (9·03 to 13·2)	11·8 (7·30 to 14·9)	10·8 (9·38 to 12·6)	8·33 (4·84 to 11·0)	-60·3 (-75·4 to -49·9)
	70+ years	41·5 (36·3 to 46·0)	37·9 (32·5 to 42·8)	35·4 (28·8 to 40·1)	36·0 (27·9 to 41·9)	28·8 (24·5 to 33·4)	25·6 (18·5 to 30·8)	-38·2 (-54·6 to -28·5)
High-income North America	All Ages	950 (869 to 1050)	1080 (1000 to 1180)	915 (770 to 1040)	889 (431 to 1280)	845 (753 to 959)	608 (315 to 886)	-36·0 (-67·3 to -7·04)
	15-49 years	156 (130 to 194)	173 (148 to 210)	146 (114 to 177)	145 (77·0 to 218)	167 (142 to 205)	122 (71·3 to 178)	-21·8 (-52·1 to 13·6)
	50-69 years	333 (298 to 377)	369 (335 to 415)	294 (226 to 354)	302 (150 to 451)	300 (259 to 349)	207 (110 to 310)	-37·9 (-65·3 to -9·82)

eTable 5. Risk deleted deaths due to all-form tuberculosis for alcohol use, smoking, and diabetes, and all three risk factors combined by age groups for 204 countries and territories in (2021) with percent change between 2015 observed deaths and 2021 all-risk deleted deaths.

Location	Age group	2015 Observed deaths	2021 Observed deaths	2021 Smoking deleted deaths	2021 Alcohol deleted deaths	2021 Diabetes deleted deaths	2021 All-risk deleted deaths	Percent Change
	70+ years	456 (396 to 488)	531 (468 to 565)	470 (379 to 528)	437 (193 to 625)	373 (330 to 428)	274 (112 to 410)	-39·8 (-75·5 to -11·8)
Canada	All Ages	126 (112 to 142)	127 (116 to 144)	109 (89·9 to 127)	104 (45·1 to 156)	104 (91·0 to 121)	74·9 (32·9 to 114)	-40·4 (-73·6 to -10·7)
	15-49 years	19·2 (15·0 to 25·6)	20·3 (16·0 to 26·3)	18·1 (13·4 to 23·4)	17·9 (10·4 to 26·7)	20·0 (15·8 to 26·0)	16·2 (10·0 to 23·5)	-15·8 (-39·7 to 9·46)
	50-69 years	35·6 (29·9 to 42·2)	33·5 (28·5 to 39·6)	27·1 (20·6 to 33·2)	27·2 (11·8 to 42·9)	29·5 (24·5 to 35·3)	20·3 (9·06 to 32·2)	-43·2 (-72·5 to -13·4)
	70+ years	70·4 (62·2 to 76·7)	72·9 (62·9 to 80·1)	63·8 (49·9 to 72·5)	58·8 (21·4 to 87·9)	53·8 (46·0 to 61·5)	37·9 (11·4 to 58·8)	-46·1 (-83·7 to -15·4)
Greenland	All Ages	2·71 (2·23 to 3·15)	2·47 (2·13 to 2·92)	1·82 (1·36 to 2·38)	1·93 (0·664 to 3·01)	2·02 (1·64 to 2·46)	1·14 (0·296 to 1·86)	-57·7 (-88·6 to -27·0)
	15-49 years	0·418 (0·338 to 0·491)	0·314 (0·260 to 0·367)	0·262 (0·204 to 0·314)	0·276 (0·175 to 0·375)	0·310 (0·258 to 0·364)	0·237 (0·163 to 0·311)	-43·2 (-58·5 to -27·7)
	50-69 years	1·25 (1·02 to 1·46)	1·18 (0·965 to 1·38)	0·795 (0·555 to 1·08)	0·924 (0·350 to 1·53)	1·02 (0·828 to 1·21)	0·556 (0·225 to 0·889)	-55·4 (-82·1 to -24·0)
	70+ years	1·01 (0·775 to 1·27)	0·964 (0·770 to 1·28)	0·749 (0·507 to 1·04)	0·709 (0·0649 to 1·20)	0·672 (0·460 to 0·954)	0·330 (-0·147 to 0·701)	-66·9 (-114 to -30·9)
United States of America	All Ages	822 (752 to 901)	949 (881 to 1030)	804 (674 to 920)	783 (385 to 1120)	739 (660 to 840)	532 (282 to 775)	-35·2 (-66·3 to -6·13)
	15-49 years	137 (115 to 168)	152 (131 to 183)	128 (100 to 154)	127 (66·1 to 190)	146 (125 to 177)	106 (60·3 to 154)	-22·5 (-53·7 to 14·4)
	50-69 years	296 (265 to 333)	335 (305 to 375)	266 (204 to 322)	274 (138 to 408)	270 (234 to 313)	186 (100 to 278)	-37·2 (-64·4 to -9·10)
	70+ years	384 (332 to 412)	457 (402 to 488)	405 (329 to 456)	378 (171 to 538)	318 (282 to 367)	236 (101 to 350)	-38·6 (-74·0 to -10·6)
Southern Latin America	All Ages	2230 (1880 to 2490)	1930 (1630 to 2140)	1730 (1420 to 1980)	1650 (926 to 2300)	1720 (1430 to 1950)	1360 (837 to 1860)	-39·2 (-62·0 to -17·8)
	15-49 years	929 (705 to 1090)	780 (591 to 934)	715 (519 to 856)	707 (456 to 932)	772 (584 to 927)	657 (441 to 836)	-29·4 (-44·0 to -13·3)
	50-69 years	711 (620 to 791)	645 (549 to 728)	533 (415 to 634)	538 (276 to 791)	571 (483 to 658)	414 (244 to 598)	-41·9 (-65·1 to -16·6)
	70+ years	554 (503 to 588)	488 (442 to 519)	457 (402 to 498)	385 (140 to 584)	360 (313 to 408)	265 (82·3 to 421)	-52·2 (-84·6 to -25·3)
Argentina	All Ages	1460 (1210 to 1660)	1270 (1040 to 1440)	1130 (894 to 1330)	1100 (649 to 1490)	1160 (941 to 1350)	921 (594 to 1230)	-37·0 (-58·1 to -17·4)
	15-49 years	666 (499 to 797)	557 (413 to 681)	514 (369 to 631)	508 (323 to 669)	552 (409 to 677)	474 (314 to 612)	-29·0 (-43·9 to -13·6)
	50-69 years	472 (408 to 528)	426 (356 to 494)	350 (265 to 418)	361 (196 to 512)	384 (320 to 456)	282 (172 to 396)	-40·4 (-62·6 to -18·0)
	70+ years	296 (273 to 316)	267 (244 to 291)	246 (212 to 270)	213 (82·7 to 314)	203 (175 to 232)	149 (50·7 to 232)	-49·7 (-82·0 to -23·2)
Chile	All Ages	682 (610 to 733)	591 (515 to 649)	536 (459 to 599)	490 (243 to 736)	502 (422 to 555)	386 (214 to 569)	-43·5 (-68·2 to -18·0)

eTable 5. Risk deleted deaths due to all-form tuberculosis for alcohol use, smoking, and diabetes, and all three risk factors combined by age groups for 204 countries and territories in (2021) with percent change between 2015 observed deaths and 2021 all-risk deleted deaths.

Location	Age group	2015 Observed deaths	2021 Observed deaths	2021 Smoking deleted deaths	2021 Alcohol deleted deaths	2021 Diabetes deleted deaths	2021 All-risk deleted deaths	Percent Change
	15-49 years	234 (185 to 262)	198 (156 to 224)	179 (135 to 208)	177 (120 to 241)	195 (154 to 222)	163 (114 to 215)	-30·4 (-46·4 to -9·82)
	50-69 years	209 (186 to 226)	193 (169 to 214)	163 (135 to 193)	155 (72·9 to 243)	164 (138 to 184)	116 (59·1 to 180)	-44·4 (-69·8 to -13·7)
	70+ years	231 (205 to 249)	196 (176 to 216)	188 (169 to 208)	152 (47·2 to 241)	138 (119 to 157)	102 (25·8 to 170)	-56·0 (-88·8 to -28·3)
Uruguay	All Ages	86·9 (76·5 to 101)	76·3 (67·1 to 86·7)	65·0 (53·5 to 76·7)	64·0 (32·8 to 92·1)	67·2 (57·4 to 76·9)	49·8 (27·9 to 69·7)	-42·8 (-67·4 to -18·1)
	15-49 years	29·1 (22·3 to 38·5)	25·1 (19·5 to 32·3)	22·1 (16·6 to 28·8)	22·3 (14·4 to 31·7)	24·7 (19·1 to 32·0)	20·0 (13·5 to 28·0)	-31·4 (-49·0 to -13·4)
	50-69 years	30·3 (26·6 to 34·8)	26·2 (22·8 to 30·0)	20·0 (14·8 to 25·0)	21·3 (10·6 to 32·1)	23·1 (19·5 to 26·6)	15·2 (8·12 to 23·6)	-49·8 (-72·5 to -24·2)
	70+ years	26·7 (23·2 to 29·4)	24·6 (21·1 to 27·5)	22·4 (19·3 to 25·3)	19·8 (8·02 to 30·2)	18·8 (16·3 to 21·6)	14·0 (5·63 to 21·6)	-47·6 (-80·0 to -20·8)
Western Europe	All Ages	3730 (3350 to 4100)	3100 (2760 to 3380)	2720 (2310 to 3050)	2440 (939 to 3850)	2590 (2300 to 2910)	1820 (686 to 2880)	-51·3 (-80·7 to -21·4)
	15-49 years	450 (357 to 585)	341 (264 to 449)	300 (225 to 394)	301 (177 to 447)	336 (260 to 444)	270 (169 to 383)	-40·3 (-55·7 to -22·9)
	50-69 years	805 (720 to 933)	636 (559 to 732)	504 (391 to 623)	508 (217 to 808)	562 (493 to 659)	376 (177 to 616)	-53·3 (-76·8 to -25·5)
	70+ years	2460 (2130 to 2620)	2120 (1820 to 2280)	1910 (1500 to 2130)	1620 (500 to 2630)	1680 (1450 to 1900)	1160 (321 to 1900)	-52·9 (-87·2 to -20·3)
Andorra	All Ages	0·109 (0·0882 to 0·135)	0·107 (0·0805 to 0·140)	0·0859 (0·0564 to 0·122)	0·0784 (0·0153 to 0·144)	0·0889 (0·0647 to 0·119)	0·0526 (0·00834 to 0·0993)	-51·4 (-92·6 to -7·67)
	15-49 years	0·0140 (0·0108 to 0·0172)	0·0110 (0·00826 to 0·0142)	0·00773 (0·00443 to 0·0112)	0·00809 (0·00161 to 0·0161)	0·0106 (0·00787 to 0·0138)	0·00568 (0·000857 to 0·0111)	-59·2 (-92·3 to -14·8)
	50-69 years	0·0260 (0·0201 to 0·0330)	0·0256 (0·0178 to 0·0349)	0·0173 (0·00954 to 0·0271)	0·0179 (0·00184 to 0·0383)	0·0220 (0·0152 to 0·0300)	0·0103 (-0·000801 to 0·0245)	-60·1 (-103 to -6·39)
	70+ years	0·0687 (0·0518 to 0·0861)	0·0700 (0·0522 to 0·0944)	0·0608 (0·0434 to 0·0844)	0·0523 (0·0120 to 0·0905)	0·0562 (0·0402 to 0·0774)	0·0366 (0·00684 to 0·0646)	-46·4 (-90·8 to -5·12)
Austria	All Ages	68·3 (61·6 to 74·2)	55·2 (49·3 to 59·8)	45·3 (36·8 to 53·8)	42·0 (13·2 to 71·2)	50·3 (44·7 to 54·9)	31·9 (9·73 to 52·7)	-53·5 (-85·6 to -19·5)
	15-49 years	7·18 (5·69 to 9·31)	5·32 (4·03 to 7·13)	4·49 (3·17 to 6·21)	4·66 (2·46 to 7·00)	5·28 (3·99 to 7·09)	4·06 (2·31 to 6·01)	-43·9 (-62·2 to -26·3)
	50-69 years	17·6 (16·0 to 19·5)	12·2 (11·1 to 13·8)	8·16 (5·52 to 11·0)	9·05 (2·24 to 16·9)	11·4 (10·3 to 12·9)	5·76 (1·25 to 11·2)	-67·4 (-92·7 to -35·2)
	70+ years	43·4 (38·2 to 46·9)	37·5 (32·3 to 41·3)	32·5 (24·5 to 37·6)	28·2 (7·66 to 47·7)	33·6 (29·0 to 37·2)	22·0 (5·12 to 37·6)	-49·6 (-88·0 to -13·2)
Belgium	All Ages	83·1 (74·0 to 91·7)	62·6 (55·0 to 69·6)	52·4 (41·2 to 61·2)	48·3 (15·3 to 80·3)	53·9 (47·3 to 61·1)	35·4 (10·3 to 59·2)	-57·6 (-86·7 to -26·2)
	15-49 years	8·97 (6·95 to 11·8)	6·60 (4·95 to 8·94)	5·85 (4·20 to 7·97)	5·91 (3·51 to 8·49)	6·51 (4·88 to 8·85)	5·35 (3·35 to 7·47)	-40·7 (-54·5 to -25·6)
	50-69 years	17·7 (15·7 to 20·5)	15·3 (13·4 to 17·6)	11·4 (8·19 to 14·6)	11·8 (4·06 to 20·7)	13·7 (11·8 to 16·3)	8·31 (2·92 to 14·1)	-53·2 (-82·3 to -19·9)

eTable 5. Risk deleted deaths due to all-form tuberculosis for alcohol use, smoking, and diabetes, and all three risk factors combined by age groups for 204 countries and territories in (2021) with percent change between 2015 observed deaths and 2021 all-risk deleted deaths.

Location	Age group	2015 Observed deaths	2021 Observed deaths	2021 Smoking deleted deaths	2021 Alcohol deleted deaths	2021 Diabetes deleted deaths	2021 All-risk deleted deaths	Percent Change
	70+ years	56·2 (49·2 to 62·1)	40·5 (34·8 to 44·9)	34·9 (25·3 to 40·7)	30·3 (7·61 to 52·2)	33·5 (28·3 to 37·7)	21·5 (4·04 to 38·7)	-61·9 (-92·7 to -29·0)
Cyprus	All Ages	6·83 (5·74 to 8·26)	6·59 (5·50 to 7·83)	5·15 (3·93 to 6·90)	5·13 (1·79 to 8·36)	4·92 (4·11 to 5·96)	3·03 (0·995 to 5·24)	-55·8 (-85·6 to -27·7)
	15-49 years	0·854 (0·666 to 1·14)	0·802 (0·606 to 1·08)	0·660 (0·441 to 0·900)	0·700 (0·409 to 1·10)	0·790 (0·597 to 1·06)	0·596 (0·376 to 0·881)	-30·5 (-52·5 to -7·88)
	50-69 years	1·57 (1·27 to 1·95)	1·39 (1·12 to 1·74)	0·866 (0·539 to 1·28)	1·03 (0·249 to 1·95)	1·09 (0·883 to 1·39)	0·520 (0·0918 to 1·07)	-67·0 (-94·0 to -38·4)
	70+ years	4·39 (3·56 to 5·38)	4·38 (3·58 to 5·36)	3·61 (2·69 to 4·99)	3·38 (1·06 to 5·35)	3·02 (2·50 to 3·72)	1·89 (0·421 to 3·20)	-56·9 (-89·8 to -29·3)
Denmark	All Ages	24·6 (22·2 to 27·0)	20·7 (18·2 to 22·3)	16·3 (12·7 to 19·5)	15·9 (5·12 to 26·3)	18·4 (16·1 to 20·0)	11·4 (3·44 to 18·7)	-53·7 (-85·3 to -18·9)
	15-49 years	2·13 (1·75 to 2·60)	1·59 (1·29 to 2·00)	1·36 (1·00 to 1·76)	1·36 (0·737 to 2·07)	1·57 (1·27 to 1·98)	1·19 (0·686 to 1·70)	-44·3 (-64·4 to -23·9)
	50-69 years	6·42 (5·83 to 7·11)	5·03 (4·50 to 5·62)	3·62 (2·52 to 4·67)	3·86 (1·28 to 6·65)	4·60 (4·14 to 5·12)	2·63 (0·840 to 4·52)	-59·1 (-86·1 to -27·1)
	70+ years	16·0 (13·8 to 17·5)	14·0 (12·0 to 15·3)	11·3 (8·32 to 13·4)	10·7 (2·96 to 17·6)	12·2 (10·5 to 13·6)	7·58 (1·91 to 12·7)	-52·9 (-88·0 to -17·2)
Finland	All Ages	55·6 (47·1 to 60·2)	48·8 (41·5 to 53·4)	44·2 (36·0 to 49·9)	39·3 (15·9 to 58·1)	37·7 (31·7 to 42·3)	27·6 (10·5 to 41·2)	-50·3 (-80·5 to -25·0)
	15-49 years	4·12 (3·61 to 4·51)	3·65 (3·18 to 4·01)	3·09 (2·49 to 3·60)	3·02 (1·43 to 4·50)	3·54 (3·06 to 3·90)	2·57 (1·33 to 3·80)	-37·7 (-65·7 to -7·61)
	50-69 years	10·6 (9·87 to 11·5)	8·22 (7·57 to 8·89)	6·50 (4·98 to 7·81)	6·28 (2·03 to 10·4)	6·74 (6·07 to 7·37)	4·16 (1·36 to 7·15)	-60·8 (-86·7 to -32·2)
	70+ years	40·7 (33·0 to 45·2)	36·9 (29·7 to 40·7)	34·5 (27·6 to 38·6)	29·9 (12·6 to 43·9)	27·3 (22·0 to 31·6)	20·8 (7·96 to 31·0)	-48·9 (-80·5 to -24·3)
France	All Ages	942 (822 to 1050)	794 (689 to 881)	720 (593 to 809)	626 (231 to 991)	718 (613 to 808)	522 (194 to 816)	-44·7 (-79·5 to -14·9)
	15-49 years	99·2 (77·6 to 130)	83·2 (62·9 to 110)	74·1 (54·0 to 98·5)	74·4 (43·6 to 111)	82·4 (62·3 to 109)	67·6 (41·7 to 97·0)	-32·1 (-49·3 to -15·8)
	50-69 years	174 (147 to 215)	134 (111 to 164)	114 (86·3 to 143)	114 (62·2 to 172)	128 (105 to 156)	96·8 (57·6 to 136)	-44·3 (-65·6 to -22·9)
	70+ years	665 (560 to 731)	573 (475 to 630)	529 (418 to 590)	434 (120 to 726)	505 (430 to 566)	354 (88·4 to 594)	-46·9 (-87·3 to -11·8)
Germany	All Ages	504 (453 to 549)	494 (426 to 545)	423 (343 to 486)	372 (113 to 629)	381 (329 to 434)	249 (75·7 to 426)	-50·5 (-84·3 to -12·6)
	15-49 years	52·1 (43·1 to 65·1)	42·7 (34·7 to 54·0)	35·5 (26·0 to 46·0)	35·8 (18·2 to 59·5)	41·9 (33·9 to 53·1)	30·5 (17·1 to 48·0)	-41·6 (-65·1 to -14·3)
	50-69 years	121 (108 to 139)	112 (97·1 to 129)	85·0 (62·7 to 107)	85·7 (28·6 to 149)	95·0 (80·8 to 114)	58·6 (21·7 to 104)	-51·8 (-80·9 to -14·7)
	70+ years	330 (284 to 364)	338 (290 to 373)	302 (233 to 344)	250 (61·7 to 425)	243 (205 to 282)	159 (34·7 to 284)	-51·7 (-89·4 to -12·4)
Greece	All Ages	118 (105 to 126)	111 (101 to 119)	90·1 (69·7 to 106)	90·2 (41·0 to 133)	95·6 (85·4 to 104)	64·6 (30·0 to 95·1)	-45·4 (-74·3 to -15·9)

eTable 5. Risk deleted deaths due to all-form tuberculosis for alcohol use, smoking, and diabetes, and all three risk factors combined by age groups for 204 countries and territories in (2021) with percent change between 2015 observed deaths and 2021 all-risk deleted deaths.

Location	Age group	2015 Observed deaths	2021 Observed deaths	2021 Smoking deleted deaths	2021 Alcohol deleted deaths	2021 Diabetes deleted deaths	2021 All-risk deleted deaths	Percent Change
	15-49 years	15·1 (13·5 to 16·5)	11·1 (10·1 to 12·1)	9·25 (8·11 to 10·9)	9·79 (7·09 to 12·8)	10·9 (9·90 to 12·0)	8·39 (6·66 to 10·4)	-44·3 (-56·6 to -31·1)
	50-69 years	22·7 (21·0 to 24·5)	22·2 (20·8 to 24·8)	15·2 (11·1 to 20·3)	17·5 (7·32 to 27·4)	19·7 (18·0 to 22·2)	11·2 (5·27 to 18·6)	-50·6 (-76·9 to -18·5)
	70+ years	80·1 (67·9 to 87·6)	77·6 (67·6 to 85·3)	65·4 (48·9 to 75·5)	62·7 (25·9 to 93·5)	64·8 (55·6 to 71·7)	44·8 (17·4 to 67·5)	-44·1 (-77·9 to -11·4)
Iceland	All Ages	1·64 (1·41 to 1·84)	1·63 (1·43 to 1·83)	1·41 (1·10 to 1·63)	1·30 (0·471 to 2·03)	1·39 (1·23 to 1·60)	0·973 (0·322 to 1·49)	-40·8 (-79·8 to -10·7)
	15-49 years	0·183 (0·145 to 0·248)	0·203 (0·155 to 0·266)	0·186 (0·139 to 0·244)	0·185 (0·115 to 0·263)	0·200 (0·153 to 0·263)	0·171 (0·111 to 0·234)	-6·60 (-28·0 to 11·4)
	50-69 years	0·314 (0·281 to 0·344)	0·297 (0·266 to 0·332)	0·226 (0·167 to 0·277)	0·226 (0·0610 to 0·391)	0·262 (0·234 to 0·299)	0·153 (0·0352 to 0·267)	-51·4 (-88·2 to -16·0)
	70+ years	1·14 (0·933 to 1·29)	1·12 (0·936 to 1·27)	0·996 (0·751 to 1·16)	0·884 (0·291 to 1·40)	0·927 (0·768 to 1·07)	0·643 (0·177 to 1·01)	-43·6 (-84·6 to -12·7)
Ireland	All Ages	29·7 (26·7 to 31·8)	24·4 (21·6 to 26·7)	20·8 (16·1 to 23·7)	19·2 (6·98 to 29·3)	20·9 (18·3 to 22·9)	14·3 (4·78 to 22·0)	-52·0 (-83·2 to -23·9)
	15-49 years	5·36 (4·43 to 6·01)	4·20 (3·44 to 4·79)	3·88 (3·04 to 4·51)	3·79 (2·59 to 5·02)	4·15 (3·40 to 4·72)	3·55 (2·55 to 4·53)	-33·9 (-49·8 to -17·4)
	50-69 years	6·57 (6·03 to 7·08)	5·07 (4·54 to 5·52)	3·91 (2·90 to 4·90)	3·87 (1·26 to 6·66)	4·52 (4·06 to 4·99)	2·73 (0·844 to 4·75)	-58·5 (-86·7 to -27·4)
	70+ years	17·6 (14·8 to 19·1)	15·0 (12·7 to 17·0)	12·9 (9·37 to 15·1)	11·5 (3·28 to 17·8)	12·1 (10·1 to 13·8)	7·88 (1·50 to 13·1)	-55·3 (-91·4 to -24·2)
Israel	All Ages	30·3 (27·1 to 33·3)	29·3 (26·3 to 32·3)	25·5 (21·2 to 28·9)	26·2 (17·0 to 33·0)	22·1 (18·8 to 25·4)	17·3 (10·5 to 23·4)	-42·9 (-64·3 to -24·3)
	15-49 years	3·89 (3·07 to 4·92)	3·43 (2·73 to 4·34)	2·96 (2·18 to 3·73)	3·10 (1·98 to 4·27)	3·35 (2·67 to 4·26)	2·67 (1·77 to 3·70)	-31·5 (-52·3 to -15·1)
	50-69 years	7·24 (6·62 to 8·01)	6·06 (5·47 to 6·77)	4·55 (3·41 to 5·78)	5·12 (2·59 to 7·10)	4·67 (4·04 to 5·37)	2·99 (1·36 to 4·59)	-58·6 (-80·7 to -38·6)
	70+ years	18·9 (15·9 to 20·8)	19·7 (16·5 to 21·8)	17·8 (14·4 to 20·2)	17·8 (12·1 to 22·1)	13·9 (11·2 to 16·2)	11·4 (7·00 to 15·5)	-39·4 (-63·1 to -21·7)
Italy	All Ages	388 (351 to 416)	289 (256 to 312)	252 (198 to 282)	223 (74·1 to 364)	243 (218 to 275)	165 (53·4 to 271)	-57·5 (-86·1 to -28·3)
	15-49 years	38·8 (33·9 to 45·7)	22·2 (18·7 to 26·8)	18·4 (14·1 to 22·1)	18·3 (8·70 to 29·1)	21·8 (18·2 to 26·3)	15·3 (7·98 to 23·9)	-60·6 (-79·0 to -40·3)
	50-69 years	72·4 (66·3 to 80·2)	52·7 (49·2 to 56·6)	40·4 (31·1 to 49·7)	40·8 (14·9 to 70·0)	46·6 (42·5 to 51·3)	28·8 (10·6 to 49·1)	-60·3 (-84·5 to -30·8)
	70+ years	276 (237 to 301)	214 (177 to 233)	192 (154 to 217)	164 (50·0 to 269)	175 (146 to 201)	121 (34·6 to 201)	-56·4 (-87·7 to -26·4)
Luxembourg	All Ages	1·62 (1·44 to 1·81)	1·42 (1·23 to 1·60)	1·21 (0·947 to 1·41)	1·07 (0·300 to 1·82)	1·04 (0·889 to 1·24)	0·673 (0·155 to 1·24)	-58·6 (-90·1 to -26·0)
	15-49 years	0·194 (0·151 to 0·265)	0·157 (0·119 to 0·219)	0·137 (0·0983 to 0·189)	0·137 (0·0741 to 0·209)	0·154 (0·116 to 0·216)	0·122 (0·0702 to 0·181)	-37·5 (-58·3 to -17·5)
	50-69 years	0·354 (0·311 to 0·394)	0·300 (0·263 to 0·345)	0·224 (0·156 to 0·283)	0·228 (0·0686 to 0·404)	0·245 (0·210 to 0·295)	0·144 (0·0432 to 0·262)	-59·2 (-87·1 to -25·6)

eTable 5. Risk deleted deaths due to all-form tuberculosis for alcohol use, smoking, and diabetes, and all three risk factors combined by age groups for 204 countries and territories in (2021) with percent change between 2015 observed deaths and 2021 all-risk deleted deaths.

Location	Age group	2015 Observed deaths	2021 Observed deaths	2021 Smoking deleted deaths	2021 Alcohol deleted deaths	2021 Diabetes deleted deaths	2021 All-risk deleted deaths	Percent Change
	70+ years	1·07 (0·924 to 1·19)	0·956 (0·791 to 1·09)	0·846 (0·660 to 0·986)	0·702 (0·132 to 1·24)	0·642 (0·518 to 0·791)	0·403 (0·0100 to 0·800)	-62·4 (-98·9 to -26·6)
Malta	All Ages	0·926 (0·846 to 1·01)	0·819 (0·750 to 0·915)	0·711 (0·595 to 0·825)	0·658 (0·270 to 1·00)	0·626 (0·547 to 0·720)	0·438 (0·165 to 0·721)	-52·7 (-81·5 to -24·3)
	15-49 years	0·116 (0·0926 to 0·151)	0·0968 (0·0777 to 0·125)	0·0816 (0·0589 to 0·109)	0·0841 (0·0444 to 0·124)	0·0949 (0·0755 to 0·123)	0·0723 (0·0410 to 0·106)	-37·7 (-59·6 to -15·2)
	50-69 years	0·230 (0·207 to 0·256)	0·164 (0·150 to 0·192)	0·122 (0·0830 to 0·155)	0·129 (0·0489 to 0·207)	0·136 (0·119 to 0·162)	0·0813 (0·0302 to 0·144)	-64·6 (-86·2 to -39·2)
	70+ years	0·578 (0·512 to 0·630)	0·555 (0·481 to 0·620)	0·504 (0·408 to 0·578)	0·442 (0·166 to 0·681)	0·393 (0·334 to 0·462)	0·282 (0·0842 to 0·480)	-51·4 (-85·2 to -21·1)
Monaco	All Ages	0·623 (0·523 to 0·761)	0·584 (0·472 to 0·720)	0·492 (0·367 to 0·633)	0·484 (0·172 to 0·790)	0·507 (0·406 to 0·624)	0·358 (0·130 to 0·596)	-42·5 (-79·5 to -15·1)
	15-49 years	0·0484 (0·0365 to 0·0619)	0·0385 (0·0276 to 0·0511)	0·0301 (0·0220 to 0·0418)	0·0324 (0·0175 to 0·0532)	0·0375 (0·0268 to 0·0497)	0·0256 (0·0148 to 0·0414)	-47·1 (-66·1 to -23·3)
	50-69 years	0·129 (0·0995 to 0·165)	0·120 (0·0930 to 0·153)	0·0884 (0·0620 to 0·126)	0·0979 (0·0419 to 0·155)	0·108 (0·0835 to 0·137)	0·0677 (0·0310 to 0·111)	-47·2 (-76·7 to -9·71)
	70+ years	0·445 (0·368 to 0·567)	0·426 (0·337 to 0·536)	0·373 (0·273 to 0·489)	0·353 (0·111 to 0·584)	0·362 (0·279 to 0·451)	0·265 (0·0803 to 0·449)	-40·5 (-82·3 to -13·8)
Netherlands	All Ages	108 (94·2 to 121)	89·9 (80·0 to 101)	77·9 (64·8 to 89·4)	71·8 (30·0 to 112)	78·7 (69·2 to 89·0)	55·7 (23·1 to 86·8)	-48·3 (-76·7 to -17·8)
	15-49 years	12·2 (8·97 to 16·9)	9·51 (7·01 to 13·3)	8·78 (6·26 to 12·1)	8·75 (5·34 to 12·8)	9·42 (6·94 to 13·2)	8·18 (5·17 to 11·7)	-33·4 (-47·3 to -21·5)
	50-69 years	24·8 (21·4 to 30·0)	19·7 (16·8 to 23·6)	16·1 (12·2 to 20·0)	16·1 (6·81 to 26·3)	18·1 (15·0 to 21·7)	12·6 (5·68 to 19·9)	-49·3 (-74·8 to -20·6)
	70+ years	70·2 (59·3 to 76·8)	60·3 (51·0 to 65·6)	52·7 (40·0 to 61·2)	46·6 (16·4 to 76·3)	50·9 (43·5 to 56·8)	34·6 (11·8 to 55·0)	-50·7 (-83·3 to -16·7)
Norway	All Ages	44·3 (38·7 to 48·1)	46·4 (40·3 to 50·5)	42·0 (34·8 to 47·7)	38·2 (18·3 to 54·4)	39·7 (34·2 to 44·3)	29·9 (13·6 to 42·6)	-32·7 (-67·8 to -2·40)
	15-49 years	2·37 (1·73 to 3·14)	2·30 (1·71 to 3·01)	2·13 (1·53 to 2·79)	2·14 (1·33 to 2·97)	2·28 (1·69 to 2·98)	2·00 (1·30 to 2·70)	-15·7 (-31·0 to -2·89)
	50-69 years	6·47 (6·04 to 7·07)	6·09 (5·58 to 6·66)	5·07 (4·14 to 5·89)	4·91 (2·26 to 7·36)	5·46 (4·90 to 6·07)	3·77 (1·72 to 5·74)	-41·7 (-71·3 to -8·80)
	70+ years	35·4 (29·9 to 38·6)	37·9 (32·1 to 41·5)	34·7 (27·8 to 39·2)	31·0 (14·0 to 44·1)	31·9 (26·6 to 35·8)	24·0 (10·3 to 35·0)	-32·2 (-70·5 to -0·959)
Portugal	All Ages	309 (272 to 353)	270 (241 to 306)	246 (208 to 281)	219 (102 to 336)	219 (187 to 256)	167 (82·7 to 259)	-46·0 (-71·6 to -17·2)
	15-49 years	64·4 (47·5 to 88·8)	50·6 (35·5 to 70·9)	46·8 (32·1 to 65·4)	47·1 (30·4 to 69·4)	50·0 (34·9 to 70·2)	44·3 (29·1 to 64·4)	-31·5 (-43·1 to -18·1)
	50-69 years	83·2 (71·7 to 102)	68·6 (58·1 to 81·4)	56·9 (43·5 to 69·9)	55·7 (25·9 to 91·2)	59·6 (48·3 to 72·1)	42·7 (23·8 to 68·7)	-48·8 (-70·9 to -18·5)
	70+ years	161 (143 to 177)	150 (131 to 167)	141 (120 to 160)	115 (37·9 to 189)	109 (91·7 to 128)	79·4 (23·6 to 132)	-50·6 (-85·0 to -17·0)
San Marino	All Ages	0·129 (0·102 to 0·167)	0·125 (0·0798 to 0·171)	0·111 (0·0678 to 0·161)	0·101 (0·0280 to 0·182)	0·106 (0·0643 to 0·147)	0·0768 (0·0184 to 0·147)	-41·2 (-86·6 to -1·21)

eTable 5. Risk deleted deaths due to all-form tuberculosis for alcohol use, smoking, and diabetes, and all three risk factors combined by age groups for 204 countries and territories in (2021) with percent change between 2015 observed deaths and 2021 all-risk deleted deaths.

Location	Age group	2015 Observed deaths	2021 Observed deaths	2021 Smoking deleted deaths	2021 Alcohol deleted deaths	2021 Diabetes deleted deaths	2021 All-risk deleted deaths	Percent Change
	15-49 years	0·00849 (0·00668 to 0·0105)	0·00655 (0·00465 to 0·00901)	0·00521 (0·00330 to 0·00778)	0·00536 (0·00158 to 0·00988)	0·00637 (0·00443 to 0·00872)	0·00425 (0·00117 to 0·00807)	-50·3 (-83·5 to -12·7)
	50-69 years	0·0210 (0·0162 to 0·0278)	0·0188 (0·0123 to 0·0274)	0·0143 (0·00799 to 0·0219)	0·0146 (0·00238 to 0·0272)	0·0166 (0·0107 to 0·0245)	0·00986 (0·00106 to 0·0202)	-53·3 (-94·9 to -2·35)
	70+ years	0·0997 (0·0759 to 0·131)	0·0996 (0·0608 to 0·137)	0·0915 (0·0547 to 0·132)	0·0811 (0·0231 to 0·146)	0·0833 (0·0478 to 0·116)	0·0625 (0·0157 to 0·118)	-37·8 (-85·6 to 2·42)
Spain	All Ages	435 (395 to 493)	350 (312 to 389)	306 (257 to 351)	283 (119 to 433)	271 (235 to 321)	196 (80·6 to 307)	-54·9 (-80·5 to -29·1)
	15-49 years	56·4 (42·3 to 76·1)	44·4 (32·1 to 61·5)	39·9 (27·6 to 55·6)	40·4 (24·5 to 61·5)	43·7 (31·6 to 60·8)	37·0 (22·9 to 54·6)	-34·8 (-47·7 to -18·8)
	50-69 years	103 (89·9 to 123)	77·8 (65·5 to 90·3)	61·9 (46·6 to 75·0)	63·4 (29·3 to 99·0)	67·6 (55·5 to 79·7)	46·8 (25·9 to 74·1)	-54·6 (-73·9 to -30·6)
	70+ years	274 (237 to 301)	227 (191 to 249)	204 (161 to 229)	178 (59·7 to 277)	159 (133 to 193)	111 (29·3 to 186)	-59·4 (-89·3 to -32·0)
Sweden	All Ages	72·9 (64·4 to 80·3)	56·5 (48·0 to 63·3)	49·0 (38·4 to 57·9)	45·0 (16·8 to 67·9)	46·3 (39·2 to 54·1)	32·4 (11·5 to 52·7)	-55·5 (-84·3 to -28·3)
	15-49 years	3·12 (2·09 to 4·42)	2·35 (1·50 to 3·39)	2·27 (1·43 to 3·28)	2·24 (1·37 to 3·29)	2·33 (1·49 to 3·38)	2·18 (1·33 to 3·20)	-30·4 (-41·3 to -21·6)
	50-69 years	8·18 (7·27 to 9·24)	5·66 (4·82 to 6·65)	4·42 (3·05 to 5·68)	4·50 (1·55 to 7·32)	4·99 (4·11 to 5·94)	3·22 (1·28 to 5·39)	-60·7 (-84·6 to -33·7)
	70+ years	61·4 (52·4 to 67·4)	48·4 (40·1 to 54·2)	42·2 (32·0 to 50·5)	38·1 (13·2 to 59·1)	38·9 (33·3 to 46·4)	26·9 (8·40 to 44·3)	-56·2 (-86·3 to -28·5)
Switzerland	All Ages	39·8 (35·3 to 43·3)	33·4 (29·0 to 36·8)	28·5 (23·0 to 32·9)	26·2 (9·85 to 41·7)	25·9 (22·3 to 29·5)	17·7 (6·30 to 29·4)	-55·6 (-83·3 to -26·2)
	15-49 years	4·14 (3·16 to 5·43)	3·03 (2·24 to 4·14)	2·70 (1·90 to 3·67)	2·70 (1·58 to 4·08)	2·98 (2·18 to 4·09)	2·45 (1·50 to 3·53)	-41·3 (-56·6 to -22·5)
	50-69 years	8·70 (7·83 to 10·1)	6·25 (5·48 to 7·47)	4·85 (3·57 to 6·00)	4·88 (1·91 to 8·35)	5·26 (4·61 to 6·44)	3·37 (1·39 to 5·69)	-61·4 (-82·9 to -35·9)
	70+ years	26·9 (22·8 to 29·2)	24·0 (19·4 to 26·4)	20·8 (15·3 to 24·0)	18·5 (6·56 to 30·3)	17·5 (14·2 to 20·6)	11·8 (3·72 to 20·2)	-56·1 (-85·7 to -23·9)
United Kingdom	All Ages	462 (424 to 496)	315 (290 to 339)	269 (228 to 307)	245 (92·4 to 402)	255 (230 to 287)	172 (60·6 to 282)	-62·7 (-86·3 to -36·3)
	15-49 years	69·3 (57·0 to 86·7)	42·8 (34·2 to 54·6)	37·0 (28·2 to 48·1)	36·2 (18·7 to 56·4)	41·9 (33·3 to 53·7)	31·7 (17·5 to 48·1)	-54·5 (-72·7 to -34·3)
	50-69 years	112 (104 to 122)	76·0 (70·1 to 83·4)	59·9 (46·6 to 72·9)	58·4 (20·7 to 103)	64·8 (58·4 to 73·1)	40·6 (14·0 to 71·0)	-63·6 (-86·8 to -35·3)
	70+ years	278 (249 to 294)	194 (175 to 206)	170 (139 to 191)	149 (50·1 to 244)	147 (132 to 165)	98·2 (27·7 to 165)	-64·7 (-89·9 to -37·7)
Latin America and Caribbean	All Ages	25800 (22600 to 31200)	24200 (20600 to 29100)	21900 (18400 to 26400)	20800 (12300 to 29700)	21500 (17900 to 26100)	17100 (10100 to 24200)	-33·7 (-58·7 to -11·3)
	15-49 years	11400 (9440 to 14200)	10100 (8190 to 12500)	9380 (7530 to 11900)	8650 (5180 to 12800)	9870 (8000 to 12200)	8010 (5020 to 11500)	-29·8 (-55·5 to -6·97)
	50-69 years	7960 (7130 to 9610)	8040 (6850 to 9630)	6960 (5690 to 8580)	6770 (3840 to 9940)	6920 (5780 to 8420)	5180 (2910 to 7480)	-34·9 (-61·8 to -9·68)

eTable 5. Risk deleted deaths due to all-form tuberculosis for alcohol use, smoking, and diabetes, and all three risk factors combined by age groups for 204 countries and territories in (2021) with percent change between 2015 observed deaths and 2021 all-risk deleted deaths.

Location	Age group	2015 Observed deaths	2021 Observed deaths	2021 Smoking deleted deaths	2021 Alcohol deleted deaths	2021 Diabetes deleted deaths	2021 All-risk deleted deaths	Percent Change
	70+ years	5290 (4810 to 5960)	5230 (4650 to 6090)	4760 (4060 to 5650)	4520 (2590 to 6200)	3900 (3340 to 4710)	3080 (1630 to 4220)	-41·8 (-67·9 to -22·0)
Andean Latin America	All Ages	5200 (4510 to 6100)	4710 (3820 to 6040)	4320 (3330 to 5540)	3910 (2070 to 6230)	4180 (3340 to 5340)	3210 (1680 to 5140)	-38·3 (-67·5 to -5·71)
	15-49 years	2050 (1700 to 2430)	1790 (1440 to 2260)	1670 (1310 to 2120)	1460 (703 to 2390)	1770 (1420 to 2240)	1350 (666 to 2220)	-34·0 (-66·7 to 5·53)
	50-69 years	1450 (1240 to 1800)	1420 (1120 to 1810)	1250 (919 to 1600)	1150 (523 to 1960)	1250 (969 to 1620)	906 (414 to 1540)	-37·6 (-69·7 to -1·85)
	70+ years	1440 (1260 to 1730)	1340 (1080 to 1740)	1240 (946 to 1610)	1140 (578 to 1780)	1000 (792 to 1350)	793 (379 to 1270)	-45·3 (-72·7 to -21·6)
Bolivia (Plurinational State of)	All Ages	1780 (1160 to 2620)	1500 (1030 to 2080)	1380 (934 to 1840)	1270 (610 to 2100)	1280 (877 to 1760)	996 (484 to 1650)	-44·2 (-68·7 to -16·9)
	15-49 years	570 (354 to 823)	473 (324 to 684)	443 (296 to 631)	386 (181 to 720)	464 (318 to 672)	357 (168 to 658)	-37·3 (-69·0 to 2·97)
	50-69 years	575 (384 to 854)	503 (346 to 680)	435 (281 to 573)	414 (182 to 713)	416 (287 to 565)	299 (128 to 516)	-47·9 (-75·2 to -18·9)
	70+ years	532 (366 to 752)	464 (317 to 657)	437 (297 to 617)	403 (188 to 635)	337 (226 to 480)	277 (130 to 429)	-47·9 (-69·8 to -28·2)
Ecuador	All Ages	1110 (947 to 1290)	621 (514 to 752)	582 (476 to 701)	550 (341 to 738)	555 (445 to 674)	465 (299 to 610)	-58·0 (-72·7 to -46·5)
	15-49 years	531 (423 to 653)	290 (230 to 369)	276 (217 to 351)	251 (152 to 349)	287 (227 to 366)	239 (149 to 329)	-55·0 (-71·4 to -40·9)
	50-69 years	267 (232 to 310)	154 (125 to 191)	139 (112 to 169)	135 (78·7 to 191)	135 (107 to 168)	109 (68·1 to 146)	-59·2 (-74·4 to -45·7)
	70+ years	266 (233 to 290)	159 (130 to 195)	149 (124 to 178)	146 (101 to 186)	115 (90·3 to 141)	98·7 (62·9 to 127)	-62·9 (-76·9 to -51·5)
Peru	All Ages	2310 (1940 to 2660)	2580 (1920 to 3520)	2360 (1730 to 3220)	2090 (937 to 3620)	2350 (1720 to 3180)	1750 (784 to 3090)	-24·0 (-64·7 to 24·4)
	15-49 years	945 (782 to 1130)	1030 (765 to 1360)	946 (703 to 1260)	819 (352 to 1500)	1020 (760 to 1350)	755 (331 to 1370)	-20·0 (-63·5 to 35·8)
	50-69 years	607 (494 to 708)	762 (515 to 1050)	679 (470 to 947)	602 (249 to 1070)	699 (463 to 963)	498 (208 to 901)	-17·6 (-65·3 to 36·9)
	70+ years	646 (543 to 786)	714 (513 to 1010)	654 (464 to 949)	591 (260 to 969)	551 (388 to 762)	417 (165 to 725)	-35·4 (-75·8 to -2·03)
Caribbean	All Ages	4060 (2780 to 8610)	3380 (2230 to 7970)	3150 (2050 to 7400)	2940 (1610 to 7270)	3100 (2040 to 7190)	2560 (1400 to 6230)	-37·0 (-58·1 to -19·2)
	15-49 years	2030 (1340 to 4280)	1530 (996 to 3680)	1450 (925 to 3460)	1320 (674 to 3510)	1510 (975 to 3620)	1230 (639 to 3260)	-39·6 (-63·0 to -16·9)
	50-69 years	1030 (675 to 2690)	960 (616 to 2530)	869 (546 to 2360)	814 (397 to 2140)	833 (525 to 2220)	652 (323 to 1700)	-37·1 (-61·0 to -15·3)
	70+ years	569 (401 to 1250)	550 (367 to 1270)	505 (343 to 1180)	476 (236 to 1080)	429 (281 to 971)	343 (167 to 747)	-39·4 (-62·5 to -16·2)
Antigua and Barbuda	All Ages	0·475 (0·424 to 0·554)	0·382 (0·337 to 0·440)	0·347 (0·293 to 0·394)	0·325 (0·170 to 0·453)	0·319 (0·270 to 0·375)	0·252 (0·137 to 0·347)	-46·9 (-69·3 to -26·2)

eTable 5. Risk deleted deaths due to all-form tuberculosis for alcohol use, smoking, and diabetes, and all three risk factors combined by age groups for 204 countries and territories in (2021) with percent change between 2015 observed deaths and 2021 all-risk deleted deaths.

Location	Age group	2015 Observed deaths	2021 Observed deaths	2021 Smoking deleted deaths	2021 Alcohol deleted deaths	2021 Diabetes deleted deaths	2021 All-risk deleted deaths	Percent Change
	15-49 years	0·186 (0·154 to 0·230)	0·129 (0·105 to 0·166)	0·121 (0·0965 to 0·152)	0·114 (0·0701 to 0·162)	0·127 (0·102 to 0·163)	0·107 (0·0680 to 0·147)	-42·7 (-61·1 to -25·5)
	50-69 years	0·157 (0·138 to 0·185)	0·138 (0·119 to 0·156)	0·120 (0·0968 to 0·141)	0·114 (0·0540 to 0·167)	0·112 (0·0922 to 0·132)	0·0823 (0·0415 to 0·123)	-47·4 (-71·4 to -23·4)
	70+ years	0·125 (0·115 to 0·136)	0·110 (0·101 to 0·124)	0·102 (0·0894 to 0·118)	0·0931 (0·0473 to 0·124)	0·0763 (0·0635 to 0·0923)	0·0592 (0·0268 to 0·0843)	-52·5 (-78·6 to -31·6)
Bahamas	All Ages	9·70 (7·95 to 12·1)	9·58 (7·59 to 12·6)	8·87 (6·85 to 11·5)	8·53 (5·66 to 12·1)	8·66 (6·68 to 11·5)	7·32 (5·11 to 10·3)	-24·7 (-46·6 to -7·83)
	15-49 years	5·48 (4·26 to 7·27)	5·16 (3·91 to 6·97)	4·86 (3·63 to 6·61)	4·65 (3·11 to 6·62)	5·06 (3·82 to 6·84)	4·37 (2·99 to 6·22)	-20·4 (-40·0 to -2·68)
	50-69 years	2·73 (2·25 to 3·29)	2·96 (2·35 to 3·87)	2·66 (2·00 to 3·29)	2·60 (1·59 to 3·81)	2·55 (1·99 to 3·37)	2·08 (1·40 to 3·00)	-24·0 (-47·5 to -4·67)
	70+ years	1·37 (1·18 to 1·49)	1·36 (1·16 to 1·63)	1·25 (1·05 to 1·50)	1·18 (0·683 to 1·65)	0·949 (0·760 to 1·15)	0·771 (0·426 to 1·09)	-43·5 (-68·7 to -24·6)
Barbados	All Ages	1·92 (1·62 to 2·23)	1·85 (1·46 to 2·30)	1·70 (1·28 to 2·13)	1·53 (0·741 to 2·42)	1·48 (1·13 to 1·89)	1·14 (0·558 to 1·78)	-40·6 (-69·5 to -12·7)
	15-49 years	0·637 (0·498 to 0·803)	0·551 (0·402 to 0·743)	0·519 (0·373 to 0·708)	0·476 (0·265 to 0·729)	0·538 (0·393 to 0·731)	0·445 (0·250 to 0·669)	-30·3 (-53·3 to -5·86)
	50-69 years	0·651 (0·554 to 0·754)	0·622 (0·469 to 0·796)	0·566 (0·414 to 0·726)	0·504 (0·219 to 0·855)	0·494 (0·355 to 0·638)	0·373 (0·171 to 0·622)	-42·9 (-72·8 to -12·1)
	70+ years	0·619 (0·546 to 0·674)	0·664 (0·532 to 0·833)	0·604 (0·462 to 0·768)	0·540 (0·193 to 0·822)	0·433 (0·316 to 0·575)	0·315 (0·0722 to 0·509)	-49·1 (-88·4 to -16·1)
Belize	All Ages	19·0 (15·9 to 23·7)	17·2 (14·5 to 21·0)	15·7 (12·9 to 19·4)	15·0 (9·24 to 20·6)	15·7 (12·9 to 19·4)	12·8 (8·33 to 17·3)	-32·8 (-54·7 to -14·5)
	15-49 years	11·2 (8·79 to 14·6)	9·48 (7·48 to 12·3)	8·91 (6·84 to 11·7)	8·32 (5·17 to 11·6)	9·35 (7·34 to 12·1)	7·82 (4·91 to 10·9)	-30·2 (-50·8 to -10·8)
	50-69 years	4·73 (4·15 to 5·63)	4·70 (3·95 to 5·60)	4·05 (3·16 to 4·80)	4·03 (2·44 to 5·71)	4·03 (3·37 to 4·84)	3·07 (1·88 to 4·21)	-35·2 (-57·9 to -16·1)
	70+ years	2·45 (2·26 to 2·61)	2·57 (2·24 to 2·94)	2·31 (1·96 to 2·71)	2·22 (1·26 to 2·94)	1·88 (1·58 to 2·25)	1·46 (0·722 to 2·02)	-40·2 (-71·1 to -19·1)
Bermuda	All Ages	0·0635 (0·0544 to 0·0740)	0·0475 (0·0405 to 0·0556)	0·0441 (0·0375 to 0·0522)	0·0421 (0·0274 to 0·0578)	0·0415 (0·0346 to 0·0495)	0·0348 (0·0233 to 0·0464)	-45·2 (-63·1 to -31·4)
	15-49 years	0·0165 (0·0126 to 0·0221)	0·0103 (0·00747 to 0·0141)	0·00973 (0·00701 to 0·0130)	0·00937 (0·00606 to 0·0134)	0·0102 (0·00736 to 0·0139)	0·00884 (0·00579 to 0·0127)	-46·6 (-55·9 to -34·4)
	50-69 years	0·0196 (0·0162 to 0·0241)	0·0144 (0·0113 to 0·0177)	0·0129 (0·0101 to 0·0163)	0·0126 (0·00792 to 0·0179)	0·0131 (0·0101 to 0·0164)	0·0105 (0·00697 to 0·0143)	-46·2 (-63·4 to -30·1)
	70+ years	0·0268 (0·0227 to 0·0306)	0·0224 (0·0194 to 0·0265)	0·0212 (0·0181 to 0·0253)	0·0198 (0·0122 to 0·0267)	0·0180 (0·0155 to 0·0218)	0·0151 (0·00875 to 0·0206)	-43·6 (-64·7 to -28·7)
Cuba	All Ages	47·6 (44·3 to 52·1)	36·0 (31·2 to 39·8)	28·5 (22·9 to 35·7)	29·9 (15·1 to 42·0)	29·2 (25·1 to 33·6)	19·8 (9·86 to 29·7)	-58·4 (-79·7 to -40·8)
	15-49 years	12·9 (10·9 to 15·5)	8·06 (6·61 to 9·87)	6·78 (5·24 to 8·50)	6·83 (4·15 to 9·76)	7·78 (6·32 to 9·52)	5·75 (3·82 to 8·06)	-55·4 (-70·7 to -38·5)
	50-69 years	17·7 (16·0 to 19·5)	15·4 (12·9 to 17·7)	11·0 (7·73 to 15·0)	12·4 (5·28 to 18·5)	12·6 (10·7 to 14·7)	7·48 (3·15 to 12·2)	-57·8 (-82·7 to -34·3)

eTable 5. Risk deleted deaths due to all-form tuberculosis for alcohol use, smoking, and diabetes, and all three risk factors combined by age groups for 204 countries and territories in (2021) with percent change between 2015 observed deaths and 2021 all-risk deleted deaths.

Location	Age group	2015 Observed deaths	2021 Observed deaths	2021 Smoking deleted deaths	2021 Alcohol deleted deaths	2021 Diabetes deleted deaths	2021 All-risk deleted deaths	Percent Change
	70+ years	16·6 (15·3 to 18·0)	12·4 (10·6 to 13·9)	10·6 (8·72 to 12·8)	10·5 (5·44 to 14·0)	8·70 (7·28 to 10·4)	6·37 (3·09 to 8·86)	-61·7 (-81·7 to -47·6)
Dominica	All Ages	3·06 (2·46 to 3·90)	2·90 (2·21 to 3·71)	2·67 (1·95 to 3·52)	2·44 (1·31 to 3·82)	2·35 (1·82 to 3·01)	1·85 (0·992 to 2·82)	-39·9 (-66·7 to -16·5)
	15-49 years	1·12 (0·838 to 1·53)	0·948 (0·672 to 1·30)	0·891 (0·605 to 1·24)	0·828 (0·443 to 1·27)	0·923 (0·656 to 1·27)	0·770 (0·422 to 1·18)	-31·5 (-55·2 to -8·80)
	50-69 years	1·08 (0·809 to 1·41)	1·12 (0·808 to 1·50)	0·996 (0·700 to 1·37)	0·927 (0·462 to 1·55)	0·898 (0·651 to 1·17)	0·684 (0·357 to 1·09)	-37·1 (-65·4 to -7·74)
	70+ years	0·771 (0·631 to 0·926)	0·776 (0·569 to 1·00)	0·722 (0·528 to 0·932)	0·627 (0·253 to 0·936)	0·463 (0·334 to 0·593)	0·330 (0·0604 to 0·541)	-57·4 (-92·6 to -32·6)
Dominican Republic	All Ages	1160 (857 to 1530)	852 (587 to 1340)	752 (517 to 1220)	730 (347 to 1320)	792 (534 to 1240)	606 (259 to 1130)	-48·3 (-71·0 to -24·0)
	15-49 years	605 (419 to 820)	353 (236 to 548)	321 (216 to 522)	288 (87·2 to 574)	347 (232 to 538)	260 (73·3 to 496)	-57·5 (-83·0 to -34·4)
	50-69 years	294 (210 to 414)	245 (156 to 403)	207 (134 to 345)	209 (102 to 386)	223 (139 to 364)	164 (70·5 to 317)	-44·7 (-67·5 to -16·5)
	70+ years	231 (176 to 314)	229 (156 to 393)	199 (134 to 346)	207 (111 to 376)	197 (129 to 335)	158 (82·6 to 295)	-32·6 (-56·9 to -1·72)
Grenada	All Ages	0·691 (0·613 to 0·785)	0·433 (0·376 to 0·493)	0·396 (0·332 to 0·458)	0·357 (0·175 to 0·508)	0·356 (0·308 to 0·416)	0·273 (0·134 to 0·386)	-60·4 (-79·7 to -44·6)
	15-49 years	0·274 (0·227 to 0·328)	0·149 (0·122 to 0·179)	0·139 (0·111 to 0·169)	0·126 (0·0687 to 0·178)	0·145 (0·119 to 0·175)	0·116 (0·0660 to 0·161)	-57·6 (-75·3 to -44·2)
	50-69 years	0·251 (0·219 to 0·287)	0·160 (0·136 to 0·188)	0·142 (0·117 to 0·165)	0·132 (0·0623 to 0·185)	0·130 (0·111 to 0·156)	0·0973 (0·0491 to 0·135)	-61·1 (-80·0 to -44·7)
	70+ years	0·153 (0·142 to 0·168)	0·116 (0·103 to 0·129)	0·109 (0·0946 to 0·125)	0·0928 (0·0326 to 0·135)	0·0740 (0·0613 to 0·0894)	0·0536 (0·0128 to 0·0842)	-64·9 (-92·0 to -45·7)
Guyana	All Ages	107 (85·1 to 131)	72·9 (56·2 to 91·4)	65·8 (49·9 to 82·1)	62·6 (36·9 to 89·7)	64·2 (49·0 to 81·1)	51·3 (32·2 to 70·9)	-52·3 (-68·3 to -34·3)
	15-49 years	60·6 (47·0 to 77·3)	39·3 (30·0 to 51·0)	35·6 (27·4 to 46·7)	34·1 (20·5 to 49·2)	38·1 (28·8 to 49·7)	30·8 (19·1 to 43·1)	-49·2 (-65·1 to -31·7)
	50-69 years	33·0 (25·9 to 39·5)	23·7 (18·0 to 29·8)	20·8 (15·4 to 26·6)	19·8 (10·7 to 29·4)	18·7 (14·0 to 23·5)	14·2 (8·11 to 20·6)	-56·7 (-73·4 to -36·1)
	70+ years	11·0 (9·40 to 12·8)	8·31 (6·64 to 10·3)	7·74 (6·21 to 9·78)	7·10 (4·10 to 10·0)	5·76 (4·45 to 7·66)	4·61 (2·50 to 6·51)	-57·9 (-76·6 to -40·8)
Haiti	All Ages	2450 (1460 to 7000)	2170 (1270 to 6600)	2080 (1230 to 6180)	1900 (1060 to 6120)	2000 (1160 to 5930)	1700 (962 to 5270)	-29·6 (-50·6 to -8·96)
	15-49 years	1210 (690 to 3440)	1020 (590 to 3100)	980 (567 to 2910)	893 (444 to 3010)	1010 (578 to 3050)	847 (430 to 2790)	-29·4 (-52·5 to -7·88)
	50-69 years	606 (324 to 2270)	601 (329 to 2140)	564 (315 to 2020)	508 (235 to 1810)	513 (276 to 1870)	416 (199 to 1440)	-30·2 (-56·0 to -5·59)
	70+ years	258 (135 to 933)	253 (127 to 928)	244 (124 to 894)	210 (94·4 to 821)	183 (87·7 to 675)	147 (63·0 to 559)	-41·9 (-67·4 to -14·1)
Jamaica	All Ages	27·2 (21·6 to 34·6)	24·4 (18·9 to 31·3)	22·4 (17·1 to 28·5)	22·2 (14·4 to 30·3)	22·4 (17·2 to 29·2)	19·2 (13·0 to 26·5)	-29·6 (-43·0 to -12·3)

eTable 5. Risk deleted deaths due to all-form tuberculosis for alcohol use, smoking, and diabetes, and all three risk factors combined by age groups for 204 countries and territories in (2021) with percent change between 2015 observed deaths and 2021 all-risk deleted deaths.

Location	Age group	2015 Observed deaths	2021 Observed deaths	2021 Smoking deleted deaths	2021 Alcohol deleted deaths	2021 Diabetes deleted deaths	2021 All-risk deleted deaths	Percent Change
	15-49 years	12·5 (8·84 to 17·6)	10·9 (7·88 to 15·6)	10·3 (7·30 to 14·8)	10·1 (6·72 to 14·8)	10·8 (7·81 to 15·5)	9·67 (6·54 to 14·2)	-22·8 (-33·2 to -8·45)
	50-69 years	8·06 (6·46 to 10·1)	7·61 (5·73 to 9·64)	6·74 (4·99 to 8·55)	6·82 (4·34 to 9·80)	6·91 (5·22 to 8·91)	5·67 (3·80 to 8·11)	-29·7 (-44·4 to -9·11)
	70+ years	6·22 (5·52 to 6·91)	5·60 (4·53 to 6·79)	5·07 (4·03 to 6·13)	4·90 (3·08 to 6·75)	4·41 (3·51 to 5·44)	3·52 (2·21 to 4·87)	-43·3 (-65·8 to -23·2)
Puerto Rico	All Ages	34·8 (30·0 to 41·5)	21·4 (17·7 to 25·7)	19·5 (15·8 to 23·6)	19·0 (12·0 to 25·8)	17·5 (14·0 to 21·8)	14·4 (9·61 to 19·3)	-58·6 (-72·5 to -46·4)
	15-49 years	10·5 (8·42 to 13·6)	5·98 (4·62 to 7·85)	5·52 (4·11 to 7·29)	5·29 (3·32 to 7·47)	5·77 (4·42 to 7·64)	4·83 (3·17 to 6·67)	-54·2 (-66·9 to -42·6)
	50-69 years	12·4 (10·3 to 15·4)	7·29 (5·76 to 9·16)	6·44 (4·94 to 8·26)	6·47 (4·12 to 8·81)	6·10 (4·61 to 8·01)	5·01 (3·42 to 6·97)	-59·6 (-71·7 to -48·9)
	70+ years	11·6 (10·4 to 12·6)	8·09 (6·96 to 9·37)	7·44 (6·20 to 8·82)	7·14 (4·50 to 9·40)	5·53 (4·52 to 6·80)	4·49 (2·63 to 6·08)	-61·3 (-78·5 to -46·3)
Saint Kitts and Nevis	All Ages	1·24 (1·03 to 1·50)	1·23 (0·983 to 1·46)	1·16 (0·912 to 1·37)	1·12 (0·698 to 1·47)	1·06 (0·823 to 1·27)	0·924 (0·595 to 1·21)	-25·4 (-49·6 to -7·24)
	15-49 years	0·488 (0·362 to 0·662)	0·441 (0·323 to 0·587)	0·425 (0·305 to 0·567)	0·416 (0·279 to 0·574)	0·434 (0·315 to 0·580)	0·397 (0·266 to 0·552)	-18·6 (-34·7 to -4·97)
	50-69 years	0·493 (0·405 to 0·571)	0·539 (0·433 to 0·623)	0·497 (0·390 to 0·594)	0·481 (0·274 to 0·644)	0·443 (0·352 to 0·528)	0·372 (0·213 to 0·490)	-24·4 (-53·8 to -2·58)
	70+ years	0·235 (0·214 to 0·253)	0·229 (0·196 to 0·261)	0·216 (0·184 to 0·246)	0·206 (0·122 to 0·256)	0·160 (0·134 to 0·191)	0·136 (0·0778 to 0·177)	-42·0 (-66·6 to -24·7)
Saint Lucia	All Ages	4·28 (3·81 to 4·83)	4·51 (3·71 to 5·38)	4·09 (3·26 to 5·03)	3·70 (1·85 to 5·73)	3·80 (3·12 to 4·54)	2·89 (1·51 to 4·52)	-32·6 (-62·8 to 2·79)
	15-49 years	1·77 (1·50 to 2·13)	1·65 (1·35 to 2·00)	1·50 (1·18 to 1·87)	1·35 (0·623 to 2·23)	1·60 (1·31 to 1·95)	1·21 (0·601 to 1·97)	-31·6 (-62·7 to 6·34)
	50-69 years	1·41 (1·25 to 1·58)	1·51 (1·19 to 1·82)	1·32 (0·993 to 1·65)	1·22 (0·550 to 1·94)	1·23 (0·959 to 1·51)	0·889 (0·431 to 1·43)	-37·1 (-68·5 to 0·236)
	70+ years	1·04 (0·953 to 1·14)	1·30 (1·06 to 1·54)	1·23 (0·983 to 1·46)	1·09 (0·563 to 1·62)	0·933 (0·743 to 1·15)	0·748 (0·379 to 1·10)	-28·5 (-62·3 to 3·74)
Saint Vincent and the Grenadines	All Ages	3·41 (2·87 to 4·10)	2·70 (2·27 to 3·27)	2·48 (2·06 to 3·09)	2·27 (1·27 to 3·40)	2·34 (1·90 to 2·91)	1·86 (1·15 to 2·66)	-45·6 (-65·9 to -23·9)
	15-49 years	1·60 (1·25 to 2·07)	1·12 (0·843 to 1·48)	1·05 (0·782 to 1·41)	0·979 (0·593 to 1·43)	1·10 (0·820 to 1·46)	0·913 (0·574 to 1·31)	-43·2 (-58·9 to -23·8)
	50-69 years	1·03 (0·871 to 1·24)	0·927 (0·787 to 1·13)	0·814 (0·672 to 0·997)	0·754 (0·381 to 1·18)	0·772 (0·630 to 0·963)	0·576 (0·337 to 0·848)	-44·2 (-68·4 to -18·9)
	70+ years	0·716 (0·651 to 0·787)	0·621 (0·559 to 0·694)	0·580 (0·517 to 0·656)	0·499 (0·215 to 0·744)	0·438 (0·380 to 0·507)	0·332 (0·141 to 0·497)	-53·6 (-80·5 to -30·8)
Suriname	All Ages	18·0 (13·7 to 23·0)	16·7 (12·1 to 22·7)	14·9 (10·2 to 20·7)	14·9 (8·57 to 22·2)	14·7 (10·1 to 20·4)	12·2 (7·09 to 18·3)	-32·1 (-54·2 to -10·4)
	15-49 years	9·41 (6·75 to 12·8)	8·25 (5·90 to 11·5)	7·65 (5·28 to 10·5)	7·56 (4·48 to 11·7)	8·06 (5·70 to 11·3)	6·99 (4·29 to 10·7)	-25·8 (-44·9 to -7·88)
	50-69 years	5·35 (4·19 to 6·74)	5·55 (4·14 to 7·71)	4·63 (3·00 to 6·65)	4·81 (2·57 to 7·03)	4·60 (3·23 to 6·67)	3·50 (1·80 to 5·31)	-34·6 (-60·7 to -6·98)

eTable 5. Risk deleted deaths due to all-form tuberculosis for alcohol use, smoking, and diabetes, and all three risk factors combined by age groups for 204 countries and territories in (2021) with percent change between 2015 observed deaths and 2021 all-risk deleted deaths.

Location	Age group	2015 Observed deaths	2021 Observed deaths	2021 Smoking deleted deaths	2021 Alcohol deleted deaths	2021 Diabetes deleted deaths	2021 All-risk deleted deaths	Percent Change
	70+ years	2·66 (2·14 to 3·20)	2·43 (1·73 to 3·27)	2·19 (1·51 to 2·97)	2·11 (0·880 to 3·03)	1·62 (0·971 to 2·24)	1·26 (0·429 to 1·97)	-52·5 (-82·5 to -24·1)
Trinidad and Tobago	All Ages	27·0 (22·1 to 33·9)	26·2 (20·6 to 34·0)	23·5 (18·3 to 30·5)	22·9 (14·5 to 31·7)	23·0 (18·0 to 29·8)	18·7 (12·3 to 25·4)	-30·7 (-51·6 to -10·1)
	15-49 years	12·5 (9·46 to 17·1)	11·7 (8·59 to 15·7)	10·9 (7·97 to 14·8)	10·6 (6·58 to 14·9)	11·5 (8·45 to 15·5)	9·94 (6·23 to 13·9)	-20·9 (-37·9 to -2·78)
	50-69 years	9·99 (8·45 to 12·2)	9·59 (7·54 to 12·1)	8·11 (6·06 to 10·4)	8·08 (4·51 to 11·8)	7·95 (6·21 to 10·2)	5·97 (3·84 to 8·38)	-40·3 (-62·1 to -16·7)
	70+ years	4·10 (3·75 to 4·59)	4·63 (3·65 to 5·74)	4·21 (3·31 to 5·20)	3·92 (2·19 to 5·49)	3·27 (2·60 to 4·14)	2·55 (1·43 to 3·54)	-37·8 (-64·6 to -11·9)
United States Virgin Islands	All Ages	0·959 (0·724 to 1·22)	0·866 (0·601 to 1·19)	0·774 (0·534 to 1·03)	0·726 (0·282 to 1·30)	0·661 (0·450 to 0·963)	0·502 (0·195 to 0·855)	-47·9 (-78·6 to -22·0)
	15-49 years	0·393 (0·287 to 0·529)	0·309 (0·215 to 0·444)	0·295 (0·202 to 0·417)	0·287 (0·168 to 0·444)	0·300 (0·207 to 0·436)	0·270 (0·159 to 0·411)	-31·7 (-51·1 to -14·4)
	50-69 years	0·305 (0·219 to 0·409)	0·264 (0·172 to 0·379)	0·215 (0·136 to 0·307)	0·205 (0·0346 to 0·442)	0·184 (0·117 to 0·291)	0·112 (0·00451 to 0·240)	-63·5 (-98·7 to -32·3)
	70+ years	0·255 (0·196 to 0·314)	0·289 (0·200 to 0·404)	0·260 (0·170 to 0·361)	0·231 (0·0513 to 0·408)	0·173 (0·119 to 0·241)	0·117 (0·000410 to 0·228)	-54·5 (-99·9 to -19·5)
Central Latin America	All Ages	7530 (6830 to 8320)	7720 (6690 to 9040)	7070 (6010 to 8260)	6730 (4260 to 9570)	6640 (5720 to 7810)	5400 (3340 to 7410)	-28·4 (-53·7 to -6·93)
	15-49 years	3140 (2650 to 3780)	3060 (2500 to 3700)	2860 (2350 to 3450)	2660 (1560 to 3800)	2970 (2440 to 3600)	2460 (1510 to 3470)	-21·8 (-48·9 to 2·88)
	50-69 years	2360 (2190 to 2530)	2600 (2250 to 3020)	2300 (1930 to 2730)	2230 (1360 to 3250)	2150 (1850 to 2520)	1670 (984 to 2310)	-29·3 (-56·8 to -5·20)
	70+ years	1820 (1680 to 1920)	1900 (1690 to 2170)	1750 (1520 to 2010)	1670 (1010 to 2200)	1360 (1150 to 1580)	1110 (614 to 1460)	-38·9 (-64·9 to -22·3)
Colombia	All Ages	1180 (1050 to 1330)	1280 (1080 to 1510)	1190 (1020 to 1450)	1140 (769 to 1530)	1110 (923 to 1320)	926 (610 to 1210)	-21·3 (-45·1 to -0·949)
	15-49 years	419 (340 to 542)	403 (310 to 544)	382 (297 to 509)	359 (227 to 510)	398 (307 to 538)	339 (222 to 476)	-19·1 (-43·8 to 1·89)
	50-69 years	373 (334 to 413)	441 (359 to 521)	397 (328 to 496)	387 (260 to 523)	382 (315 to 449)	307 (208 to 414)	-17·7 (-44·0 to 6·07)
	70+ years	359 (327 to 387)	418 (359 to 481)	396 (338 to 461)	376 (249 to 465)	307 (262 to 370)	260 (163 to 325)	-27·4 (-54·4 to -10·5)
Costa Rica	All Ages	58·3 (53·7 to 63·6)	65·9 (58·9 to 72·9)	58·4 (49·1 to 67·4)	56·7 (33·2 to 76·7)	55·3 (49·0 to 63·2)	43·2 (25·3 to 57·6)	-25·9 (-55·8 to -2·43)
	15-49 years	19·0 (16·0 to 22·8)	20·7 (17·3 to 24·8)	19·1 (15·4 to 22·8)	18·3 (12·4 to 25·5)	20·2 (16·9 to 24·2)	16·8 (11·8 to 22·3)	-11·9 (-37·7 to 10·6)
	50-69 years	20·1 (18·4 to 22·0)	24·5 (21·1 to 27·3)	20·6 (15·9 to 24·5)	20·6 (10·8 to 28·8)	20·4 (17·3 to 23·5)	14·9 (8·21 to 20·9)	-25·9 (-59·3 to -0·448)
	70+ years	18·4 (16·5 to 19·9)	20·1 (17·4 to 23·1)	18·2 (15·6 to 20·7)	17·2 (9·42 to 24·0)	14·1 (12·2 to 16·8)	11·0 (5·42 to 15·4)	-40·4 (-70·8 to -17·0)
El Salvador	All Ages	304 (261 to 354)	253 (199 to 334)	232 (179 to 309)	224 (138 to 328)	217 (170 to 289)	179 (113 to 249)	-41·2 (-60·5 to -23·9)

eTable 5. Risk deleted deaths due to all-form tuberculosis for alcohol use, smoking, and diabetes, and all three risk factors combined by age groups for 204 countries and territories in (2021) with percent change between 2015 observed deaths and 2021 all-risk deleted deaths.

Location	Age group	2015 Observed deaths	2021 Observed deaths	2021 Smoking deleted deaths	2021 Alcohol deleted deaths	2021 Diabetes deleted deaths	2021 All-risk deleted deaths	Percent Change
	15-49 years	128 (105 to 157)	94·8 (73·0 to 128)	87·3 (66·5 to 117)	83·3 (49·4 to 120)	92·5 (71·4 to 124)	76·0 (46·1 to 105)	-40·8 (-57·9 to -20·9)
	50-69 years	79·6 (66·9 to 93·7)	74·9 (54·1 to 105)	65·6 (47·2 to 93·7)	65·1 (38·3 to 98·0)	62·3 (45·9 to 87·9)	48·5 (29·7 to 68·9)	-39·2 (-62·0 to -18·4)
	70+ years	87·6 (72·5 to 101)	77·6 (58·9 to 99·3)	73·8 (56·0 to 96·1)	70·0 (46·9 to 98·7)	57·0 (43·3 to 74·6)	49·3 (31·5 to 68·9)	-43·8 (-65·8 to -28·0)
Guatemala	All Ages	605 (533 to 687)	581 (497 to 680)	541 (451 to 624)	522 (346 to 677)	508 (424 to 598)	431 (285 to 551)	-28·8 (-52·1 to -11·5)
	15-49 years	294 (248 to 354)	274 (226 to 328)	260 (211 to 311)	245 (165 to 324)	267 (218 to 321)	230 (160 to 298)	-22·0 (-46·7 to -2·44)
	50-69 years	172 (153 to 195)	172 (147 to 201)	155 (125 to 180)	153 (95·3 to 202)	140 (115 to 166)	114 (68·5 to 146)	-33·8 (-57·8 to -16·4)
	70+ years	101 (94·7 to 108)	113 (98·4 to 128)	103 (88·4 to 119)	102 (66·8 to 130)	77·9 (65·8 to 93·1)	64·5 (37·7 to 82·4)	-36·3 (-62·8 to -18·7)
Honduras	All Ages	780 (549 to 1020)	744 (579 to 982)	653 (465 to 865)	669 (374 to 967)	622 (480 to 815)	501 (281 to 705)	-35·6 (-55·5 to -14·4)
	15-49 years	234 (160 to 318)	236 (160 to 327)	215 (143 to 302)	213 (130 to 304)	230 (155 to 318)	192 (120 to 272)	-16·7 (-45·1 to 29·2)
	50-69 years	287 (214 to 365)	277 (202 to 381)	229 (150 to 321)	244 (125 to 356)	222 (159 to 298)	165 (81·9 to 250)	-42·3 (-63·2 to -15·7)
	70+ years	230 (155 to 311)	209 (146 to 273)	187 (127 to 257)	190 (101 to 281)	150 (98·7 to 198)	122 (63·3 to 180)	-46·9 (-65·8 to -29·3)
Mexico	All Ages	3170 (2910 to 3540)	3190 (2780 to 3670)	2950 (2490 to 3410)	2720 (1560 to 3910)	2750 (2330 to 3230)	2210 (1250 to 3130)	-30·3 (-59·8 to -5·84)
	15-49 years	1440 (1250 to 1730)	1430 (1210 to 1710)	1340 (1120 to 1590)	1210 (670 to 1830)	1380 (1160 to 1650)	1120 (645 to 1670)	-22·4 (-52·8 to 6·70)
	50-69 years	986 (927 to 1070)	1060 (917 to 1210)	951 (772 to 1120)	894 (489 to 1290)	858 (716 to 1020)	668 (337 to 949)	-32·3 (-64·6 to -5·25)
	70+ years	671 (640 to 696)	642 (577 to 709)	594 (505 to 672)	554 (302 to 735)	455 (374 to 536)	364 (189 to 495)	-45·8 (-71·5 to -26·5)
Nicaragua	All Ages	260 (237 to 297)	238 (186 to 302)	208 (158 to 267)	202 (112 to 295)	202 (157 to 250)	151 (83·3 to 211)	-41·9 (-67·4 to -16·5)
	15-49 years	96·8 (83·9 to 110)	84·2 (64·9 to 104)	73·0 (56·7 to 94·6)	68·0 (30·6 to 109)	81·6 (63·3 to 101)	57·9 (26·6 to 87·8)	-40·2 (-73·6 to -8·12)
	50-69 years	80·1 (69·1 to 93·3)	79·2 (59·4 to 110)	66·1 (48·1 to 91·2)	66·3 (34·6 to 102)	62·7 (46·2 to 85·0)	44·3 (22·7 to 65·8)	-44·6 (-70·9 to -15·4)
	70+ years	71·4 (61·5 to 85·9)	68·4 (52·3 to 87·4)	62·0 (46·1 to 81·1)	61·5 (39·1 to 86·9)	50·9 (38·1 to 65·5)	42·1 (26·7 to 58·4)	-41·0 (-62·6 to -15·9)
Panama	All Ages	237 (207 to 271)	209 (168 to 246)	194 (156 to 231)	183 (116 to 250)	182 (145 to 218)	150 (98·1 to 200)	-36·6 (-57·7 to -15·7)
	15-49 years	98·5 (79·2 to 123)	83·5 (65·7 to 110)	79·5 (61·9 to 103)	73·9 (47·8 to 101)	82·0 (64·4 to 108)	70·0 (45·7 to 94·0)	-29·1 (-48·8 to -9·20)
	50-69 years	69·3 (62·0 to 77·0)	65·3 (51·9 to 79·3)	58·5 (46·3 to 72·0)	55·8 (32·3 to 77·4)	55·1 (43·0 to 66·8)	43·3 (27·2 to 59·9)	-37·4 (-61·9 to -10·5)

eTable 5. Risk deleted deaths due to all-form tuberculosis for alcohol use, smoking, and diabetes, and all three risk factors combined by age groups for 204 countries and territories in (2021) with percent change between 2015 observed deaths and 2021 all-risk deleted deaths.

Location	Age group	2015 Observed deaths	2021 Observed deaths	2021 Smoking deleted deaths	2021 Alcohol deleted deaths	2021 Diabetes deleted deaths	2021 All-risk deleted deaths	Percent Change
	70+ years	59·3 (53·3 to 65·2)	54·2 (44·0 to 62·8)	49·8 (39·7 to 58·6)	46·9 (26·0 to 61·1)	38·4 (28·1 to 47·2)	30·7 (16·3 to 42·3)	-48·2 (-73·3 to -27·2)
Venezuela (Bolivarian Republic of)	All Ages	939 (846 to 1090)	1150 (912 to 1470)	1050 (811 to 1310)	1010 (633 to 1450)	999 (772 to 1250)	806 (508 to 1130)	-14·2 (-43·5 to 16·1)
	15-49 years	404 (336 to 515)	430 (318 to 568)	401 (295 to 530)	381 (233 to 545)	422 (313 to 560)	353 (223 to 498)	-12·8 (-38·9 to 13·6)
	50-69 years	293 (268 to 329)	409 (303 to 534)	358 (266 to 465)	349 (198 to 524)	345 (257 to 446)	264 (157 to 388)	-10·1 (-45·2 to 25·2)
	70+ years	220 (200 to 238)	294 (234 to 377)	266 (208 to 349)	257 (146 to 365)	212 (164 to 273)	168 (90·3 to 238)	-23·6 (-61·1 to 5·91)
Tropical Latin America	All Ages	9010 (7850 to 10400)	8360 (7270 to 9580)	7380 (6100 to 8710)	7200 (4260 to 9660)	7580 (6540 to 8770)	5920 (3670 to 7680)	-34·3 (-58·8 to -14·4)
	15-49 years	4200 (3390 to 5220)	3700 (3030 to 4500)	3410 (2710 to 4190)	3220 (2030 to 4390)	3620 (2970 to 4430)	2970 (1970 to 3960)	-29·3 (-52·1 to -9·83)
	50-69 years	3120 (2820 to 3460)	3050 (2710 to 3450)	2540 (1990 to 3070)	2570 (1380 to 3560)	2690 (2340 to 3090)	1960 (1110 to 2700)	-37·3 (-63·5 to -17·3)
	70+ years	1460 (1310 to 1530)	1450 (1320 to 1540)	1270 (1070 to 1420)	1240 (678 to 1590)	1100 (1010 to 1250)	835 (415 to 1140)	-42·7 (-70·8 to -23·4)
Brazil	All Ages	8640 (7520 to 10000)	8030 (7010 to 9230)	7100 (5900 to 8400)	6930 (4120 to 9280)	7290 (6290 to 8460)	5710 (3560 to 7410)	-34·0 (-58·2 to -14·5)
	15-49 years	4040 (3260 to 5040)	3560 (2920 to 4350)	3290 (2620 to 4050)	3110 (1970 to 4230)	3490 (2870 to 4280)	2860 (1910 to 3820)	-29·1 (-51·9 to -9·79)
	50-69 years	3000 (2710 to 3350)	2940 (2620 to 3330)	2450 (1930 to 2960)	2490 (1350 to 3440)	2600 (2260 to 2990)	1890 (1080 to 2610)	-37·0 (-62·7 to -17·3)
	70+ years	1390 (1250 to 1470)	1380 (1260 to 1470)	1220 (1040 to 1360)	1180 (662 to 1510)	1060 (966 to 1190)	804 (409 to 1100)	-42·0 (-69·7 to -22·5)
Paraguay	All Ages	363 (311 to 425)	325 (254 to 408)	276 (196 to 354)	268 (130 to 439)	290 (221 to 367)	211 (107 to 336)	-42·1 (-68·5 to -12·6)
	15-49 years	159 (130 to 185)	136 (103 to 163)	122 (87·8 to 151)	115 (60·5 to 180)	134 (101 to 160)	105 (57·3 to 155)	-34·4 (-60·2 to -3·88)
	50-69 years	116 (97·9 to 144)	111 (85·1 to 149)	88·6 (58·4 to 122)	88·3 (34·0 to 159)	95·7 (71·5 to 131)	63·2 (25·6 to 115)	-45·6 (-76·4 to -14·6)
	70+ years	69·8 (58·6 to 81·6)	66·1 (49·6 to 84·2)	52·9 (34·0 to 69·3)	52·3 (17·0 to 85·6)	48·3 (35·9 to 63·4)	30·5 (7·79 to 51·7)	-56·4 (-88·4 to -26·5)
North Africa and Middle East	All Ages	22600 (18700 to 31700)	19600 (15600 to 26900)	17200 (13200 to 23800)	19400 (15400 to 26900)	16000 (12200 to 22300)	13800 (10600 to 19100)	-38·8 (-48·7 to -29·0)
	15-49 years	7960 (6400 to 10200)	6740 (5370 to 8920)	5840 (4640 to 7460)	6610 (5150 to 8750)	6360 (5070 to 8430)	5430 (4240 to 6970)	-31·8 (-42·0 to -18·8)
	50-69 years	5900 (4870 to 8700)	5790 (4500 to 8260)	4790 (3480 to 6680)	5700 (4400 to 8240)	4300 (3210 to 6320)	3470 (2410 to 5060)	-41·1 (-54·5 to -26·1)
	70+ years	6410 (5020 to 10800)	5660 (4280 to 8480)	5090 (3730 to 7720)	5610 (4260 to 8470)	3870 (2860 to 6180)	3440 (2490 to 5580)	-46·4 (-56·5 to -35·0)
Afghanistan	All Ages	7500 (5620 to 11600)	6590 (4970 to 9890)	5950 (4420 to 8720)	6560 (4970 to 9800)	5440 (4070 to 8290)	4870 (3700 to 7090)	-34·6 (-46·5 to -21·0)

eTable 5. Risk deleted deaths due to all-form tuberculosis for alcohol use, smoking, and diabetes, and all three risk factors combined by age groups for 204 countries and territories in (2021) with percent change between 2015 observed deaths and 2021 all-risk deleted deaths.

Location	Age group	2015 Observed deaths	2021 Observed deaths	2021 Smoking deleted deaths	2021 Alcohol deleted deaths	2021 Diabetes deleted deaths	2021 All-risk deleted deaths	Percent Change
	15-49 years	3280 (2410 to 4720)	2930 (2170 to 4320)	2620 (1980 to 3690)	2910 (2160 to 4280)	2740 (2030 to 4050)	2430 (1860 to 3440)	-25·5 (-39·8 to -8·74)
	50-69 years	1570 (1100 to 2810)	1670 (1160 to 2610)	1430 (940 to 2190)	1660 (1150 to 2590)	1130 (725 to 1830)	930 (586 to 1450)	-39·9 (-59·2 to -20·2)
	70+ years	1430 (889 to 2940)	1100 (715 to 2090)	1020 (659 to 1930)	1090 (714 to 2080)	673 (402 to 1400)	616 (379 to 1280)	-56·5 (-66·5 to -40·3)
Algeria	All Ages	1740 (1320 to 2260)	1610 (1260 to 2120)	1350 (972 to 1810)	1560 (1150 to 2060)	1270 (973 to 1710)	1050 (752 to 1380)	-39·7 (-52·3 to -24·5)
	15-49 years	450 (344 to 667)	386 (298 to 527)	324 (247 to 439)	367 (256 to 515)	368 (286 to 498)	298 (211 to 407)	-33·3 (-49·6 to -16·9)
	50-69 years	435 (322 to 596)	437 (315 to 615)	352 (238 to 501)	422 (299 to 601)	327 (234 to 472)	256 (172 to 385)	-40·8 (-58·2 to -18·2)
	70+ years	742 (543 to 1000)	723 (536 to 962)	611 (417 to 804)	712 (525 to 943)	513 (387 to 698)	430 (301 to 574)	-41·8 (-55·1 to -24·0)
Bahrain	All Ages	11·8 (10·1 to 14·2)	11·7 (9·47 to 16·2)	9·25 (6·91 to 12·9)	11·2 (8·20 to 14·8)	9·16 (7·28 to 12·6)	6·89 (4·55 to 9·92)	-41·6 (-60·3 to -22·8)
	15-49 years	5·46 (4·55 to 6·87)	4·56 (3·51 to 6·70)	3·77 (2·75 to 5·65)	4·27 (2·94 to 6·00)	4·34 (3·34 to 6·31)	3·40 (2·34 to 4·94)	-37·8 (-57·6 to -22·1)
	50-69 years	3·32 (2·76 to 4·23)	3·88 (3·07 to 5·19)	2·76 (1·74 to 3·96)	3·69 (2·56 to 4·74)	2·65 (2·05 to 3·74)	1·73 (0·957 to 2·69)	-47·7 (-69·7 to -17·8)
	70+ years	2·63 (2·21 to 3·17)	3·03 (2·29 to 4·13)	2·49 (1·82 to 3·49)	2·96 (2·29 to 3·87)	1·93 (1·42 to 2·66)	1·52 (0·991 to 2·14)	-42·1 (-61·1 to -19·7)
Egypt	All Ages	1150 (1030 to 1280)	850 (679 to 1050)	667 (524 to 863)	837 (654 to 1030)	708 (571 to 901)	546 (405 to 702)	-52·4 (-63·6 to -37·7)
	15-49 years	377 (320 to 420)	283 (222 to 365)	222 (178 to 287)	276 (218 to 354)	275 (217 to 354)	213 (163 to 273)	-43·3 (-56·3 to -24·4)
	50-69 years	465 (415 to 533)	340 (259 to 432)	255 (188 to 348)	335 (248 to 424)	268 (204 to 347)	198 (135 to 257)	-57·2 (-68·6 to -42·3)
	70+ years	226 (196 to 275)	186 (144 to 236)	147 (106 to 190)	185 (144 to 235)	123 (96·2 to 163)	94·0 (65·4 to 129)	-58·2 (-69·2 to -44·0)
Iran (Islamic Republic of)	All Ages	1220 (1080 to 1310)	963 (830 to 1150)	845 (693 to 1040)	937 (801 to 1150)	809 (690 to 980)	694 (565 to 882)	-43·2 (-51·7 to -30·6)
	15-49 years	431 (384 to 468)	332 (279 to 391)	294 (241 to 350)	321 (247 to 386)	327 (274 to 385)	282 (216 to 343)	-34·6 (-44·6 to -22·0)
	50-69 years	303 (273 to 330)	262 (224 to 318)	212 (166 to 275)	254 (211 to 316)	215 (182 to 265)	170 (131 to 224)	-44·0 (-56·2 to -29·7)
	70+ years	422 (363 to 463)	342 (282 to 422)	312 (238 to 392)	336 (270 to 422)	241 (197 to 314)	215 (167 to 284)	-49·0 (-60·2 to -34·0)
Iraq	All Ages	969 (764 to 1210)	859 (630 to 1140)	675 (473 to 940)	849 (618 to 1110)	621 (463 to 835)	485 (341 to 664)	-49·8 (-64·3 to -31·7)
	15-49 years	340 (258 to 451)	244 (174 to 362)	195 (135 to 295)	239 (166 to 345)	220 (156 to 326)	174 (121 to 259)	-48·6 (-62·8 to -28·3)
	50-69 years	309 (237 to 400)	310 (214 to 439)	229 (144 to 345)	306 (204 to 436)	212 (146 to 295)	155 (99·9 to 236)	-49·5 (-68·7 to -26·9)

eTable 5. Risk deleted deaths due to all-form tuberculosis for alcohol use, smoking, and diabetes, and all three risk factors combined by age groups for 204 countries and territories in (2021) with percent change between 2015 observed deaths and 2021 all-risk deleted deaths.

Location	Age group	2015 Observed deaths	2021 Observed deaths	2021 Smoking deleted deaths	2021 Alcohol deleted deaths	2021 Diabetes deleted deaths	2021 All-risk deleted deaths	Percent Change
	70+ years	258 (206 to 325)	278 (206 to 390)	223 (160 to 322)	276 (201 to 384)	162 (120 to 222)	129 (84·3 to 186)	-50·0 (-65·9 to -31·1)
Jordan	All Ages	37·0 (31·4 to 44·6)	48·3 (36·2 to 61·1)	36·5 (26·5 to 49·0)	47·5 (35·5 to 60·8)	36·7 (28·0 to 48·1)	27·4 (19·3 to 36·9)	-26·0 (-47·1 to 6·90)
	15-49 years	11·0 (9·42 to 13·0)	13·5 (10·3 to 17·6)	9·88 (7·37 to 13·6)	13·2 (9·99 to 17·1)	12·7 (9·80 to 16·5)	9·22 (6·55 to 12·7)	-16·1 (-35·7 to 17·3)
	50-69 years	11·1 (9·21 to 14·1)	16·3 (12·2 to 21·9)	11·3 (7·60 to 16·1)	16·0 (11·7 to 21·6)	11·6 (8·57 to 16·0)	7·96 (5·32 to 11·6)	-28·1 (-53·8 to 10·2)
	70+ years	13·0 (10·6 to 16·2)	16·9 (12·3 to 21·2)	13·7 (9·32 to 18·2)	16·7 (12·2 to 21·2)	10·8 (7·88 to 14·9)	8·64 (5·86 to 12·3)	-33·6 (-54·0 to -5·98)
Kuwait	All Ages	35·6 (33·4 to 38·8)	41·2 (34·5 to 48·8)	32·7 (25·3 to 41·2)	41·0 (34·6 to 48·6)	30·6 (25·5 to 38·4)	23·7 (17·7 to 31·9)	-33·4 (-51·5 to -8·12)
	15-49 years	12·5 (11·8 to 13·6)	12·3 (10·4 to 14·8)	9·29 (7·29 to 11·7)	12·2 (10·4 to 14·7)	11·5 (9·77 to 13·7)	8·66 (6·82 to 11·0)	-30·8 (-46·1 to -10·9)
	50-69 years	9·28 (8·45 to 10·1)	12·1 (9·69 to 15·0)	9·20 (6·86 to 11·8)	12·1 (9·68 to 14·9)	8·87 (7·17 to 11·5)	6·65 (4·74 to 9·11)	-28·2 (-49·1 to -0·801)
	70+ years	12·7 (11·1 to 14·1)	16·2 (13·6 to 19·3)	13·6 (10·3 to 17·5)	16·2 (13·6 to 19·3)	9·60 (7·67 to 13·0)	7·74 (5·32 to 11·2)	-38·8 (-59·2 to -12·0)
Lebanon	All Ages	64·9 (56·6 to 76·5)	60·6 (49·2 to 73·1)	47·7 (33·2 to 61·7)	57·6 (45·1 to 73·0)	48·8 (40·5 to 60·4)	37·6 (26·9 to 49·9)	-41·9 (-57·6 to -20·2)
	15-49 years	13·0 (10·7 to 15·4)	11·4 (9·19 to 14·0)	8·96 (6·52 to 11·6)	10·6 (8·20 to 13·5)	11·0 (8·86 to 13·4)	8·21 (6·01 to 11·1)	-36·4 (-50·1 to -13·9)
	50-69 years	16·6 (13·5 to 19·7)	14·8 (12·4 to 17·7)	9·76 (6·09 to 13·8)	13·8 (10·7 to 17·3)	11·4 (9·68 to 14·0)	7·23 (4·68 to 10·7)	-56·1 (-74·2 to -35·7)
	70+ years	34·4 (28·7 to 41·9)	33·6 (26·1 to 40·8)	28·3 (19·6 to 37·0)	32·6 (24·4 to 40·4)	25·7 (20·6 to 33·4)	21·5 (15·1 to 28·6)	-37·3 (-53·4 to -15·3)
Libya	All Ages	146 (103 to 197)	169 (106 to 247)	140 (84·4 to 211)	166 (99·9 to 244)	136 (84·7 to 199)	111 (67·1 to 165)	-24·2 (-40·3 to -4·73)
	15-49 years	54·2 (36·4 to 78·4)	57·3 (33·8 to 87·1)	45·9 (26·9 to 73·6)	55·8 (33·9 to 86·1)	54·8 (32·3 to 84·5)	43·2 (24·6 to 69·2)	-20·8 (-40·6 to 2·32)
	50-69 years	38·7 (25·4 to 53·9)	53·1 (31·9 to 83·7)	43·1 (25·0 to 66·2)	52·2 (31·1 to 83·3)	41·3 (24·1 to 65·2)	33·1 (18·6 to 51·2)	-15·0 (-35·4 to 7·16)
	70+ years	46·4 (33·0 to 63·7)	52·9 (34·2 to 76·2)	45·8 (31·0 to 71·3)	52·4 (34·1 to 75·7)	34·9 (22·4 to 52·3)	29·6 (19·1 to 44·7)	-36·0 (-54·2 to -15·5)
Morocco	All Ages	4170 (2710 to 8680)	3480 (2160 to 7680)	3180 (2020 to 6740)	3440 (2130 to 7680)	2690 (1570 to 5990)	2440 (1430 to 5410)	-41·7 (-54·1 to -27·1)
	15-49 years	1080 (740 to 1810)	741 (524 to 1360)	654 (464 to 1110)	716 (508 to 1290)	685 (477 to 1280)	588 (400 to 1020)	-45·5 (-60·0 to -28·4)
	50-69 years	1290 (768 to 2840)	1240 (724 to 2800)	1090 (651 to 2330)	1230 (722 to 2800)	924 (495 to 2190)	809 (434 to 1840)	-37·4 (-54·7 to -17·0)
	70+ years	1640 (996 to 4040)	1440 (831 to 3450)	1370 (785 to 3210)	1430 (829 to 3450)	1020 (550 to 2570)	974 (512 to 2400)	-40·9 (-55·4 to -24·1)
Oman	All Ages	22·4 (16·9 to 31·2)	17·8 (14·0 to 26·9)	15·8 (11·5 to 24·1)	17·4 (13·4 to 26·8)	14·6 (11·4 to 22·3)	12·6 (8·90 to 20·0)	-43·8 (-56·1 to -29·1)

eTable 5. Risk deleted deaths due to all-form tuberculosis for alcohol use, smoking, and diabetes, and all three risk factors combined by age groups for 204 countries and territories in (2021) with percent change between 2015 observed deaths and 2021 all-risk deleted deaths.

Location	Age group	2015 Observed deaths	2021 Observed deaths	2021 Smoking deleted deaths	2021 Alcohol deleted deaths	2021 Diabetes deleted deaths	2021 All-risk deleted deaths	Percent Change
	15-49 years	8·23 (6·20 to 12·2)	6·57 (4·88 to 10·6)	5·62 (3·75 to 9·05)	6·28 (4·29 to 10·2)	6·31 (4·69 to 10·2)	5·19 (3·38 to 8·39)	-36·9 (-53·2 to -22·0)
	50-69 years	6·32 (4·48 to 8·53)	4·60 (3·36 to 6·84)	4·04 (2·82 to 6·44)	4·51 (3·31 to 6·77)	3·63 (2·61 to 5·54)	3·14 (2·17 to 5·03)	-49·9 (-62·4 to -28·5)
	70+ years	6·93 (4·61 to 9·95)	6·11 (4·26 to 9·31)	5·61 (3·76 to 8·70)	6·05 (4·12 to 9·30)	4·11 (2·80 to 6·27)	3·73 (2·52 to 5·73)	-45·9 (-58·1 to -29·3)
Palestine	All Ages	32·4 (24·7 to 42·7)	27·8 (19·5 to 38·8)	22·6 (16·3 to 32·8)	26·8 (18·7 to 38·6)	22·2 (15·3 to 31·4)	17·5 (12·1 to 26·0)	-45·9 (-60·2 to -30·4)
	15-49 years	7·48 (5·57 to 11·2)	6·48 (4·46 to 10·4)	5·15 (3·58 to 8·07)	6·04 (4·02 to 9·48)	6·28 (4·27 to 10·2)	4·71 (3·13 to 7·42)	-36·6 (-53·0 to -18·4)
	50-69 years	9·73 (6·82 to 13·6)	9·08 (5·66 to 13·7)	6·72 (4·30 to 10·4)	8·69 (5·53 to 13·8)	6·95 (4·26 to 10·4)	4·98 (3·11 to 8·09)	-48·6 (-65·4 to -28·3)
	70+ years	12·4 (9·46 to 16·2)	10·8 (8·01 to 14·8)	9·25 (6·82 to 12·5)	10·6 (7·87 to 14·8)	7·56 (5·52 to 10·4)	6·38 (4·65 to 9·11)	-48·3 (-61·3 to -31·8)
Qatar	All Ages	28·7 (22·7 to 36·0)	20·9 (14·5 to 28·4)	16·5 (11·0 to 23·4)	19·9 (12·9 to 28·3)	16·7 (11·6 to 23·1)	12·2 (7·13 to 18·1)	-57·4 (-71·6 to -41·3)
	15-49 years	16·7 (12·7 to 21·0)	12·7 (8·84 to 17·6)	9·97 (6·33 to 14·5)	11·9 (7·58 to 17·2)	12·0 (8·38 to 16·8)	8·91 (5·27 to 13·4)	-46·6 (-64·8 to -27·6)
	50-69 years	7·41 (5·74 to 9·43)	4·59 (2·94 to 6·48)	3·41 (2·16 to 5·10)	4·40 (2·74 to 6·10)	2·95 (1·90 to 4·29)	1·98 (0·976 to 3·23)	-73·2 (-85·4 to -60·9)
	70+ years	3·94 (3·18 to 5·10)	3·23 (2·42 to 4·48)	2·74 (1·81 to 4·06)	3·15 (2·40 to 4·44)	1·26 (0·724 to 2·31)	0·894 (0·314 to 1·79)	-77·2 (-91·8 to -58·9)
Saudi Arabia	All Ages	1220 (993 to 1550)	1140 (841 to 1660)	981 (702 to 1440)	1130 (837 to 1620)	958 (709 to 1390)	816 (582 to 1200)	-33·3 (-48·1 to -14·4)
	15-49 years	644 (503 to 861)	602 (423 to 902)	510 (342 to 800)	594 (414 to 872)	559 (393 to 838)	470 (318 to 739)	-27·3 (-43·5 to -6·19)
	50-69 years	327 (264 to 423)	324 (238 to 460)	272 (193 to 388)	322 (237 to 460)	252 (184 to 365)	210 (150 to 302)	-35·8 (-52·1 to -14·2)
	70+ years	235 (187 to 304)	208 (151 to 296)	192 (142 to 268)	207 (151 to 293)	140 (105 to 201)	129 (97·0 to 184)	-45·3 (-57·7 to -24·4)
Sudan	All Ages	1900 (1230 to 2690)	1540 (969 to 2270)	1390 (890 to 2050)	1540 (969 to 2270)	1340 (844 to 1940)	1210 (770 to 1770)	-35·8 (-46·7 to -21·9)
	15-49 years	668 (393 to 1010)	561 (352 to 849)	508 (324 to 754)	561 (352 to 849)	548 (343 to 829)	497 (316 to 739)	-25·1 (-39·0 to -7·91)
	50-69 years	413 (242 to 603)	388 (241 to 604)	335 (213 to 531)	388 (241 to 604)	311 (194 to 475)	269 (170 to 410)	-34·3 (-48·8 to -17·3)
	70+ years	459 (267 to 700)	402 (245 to 594)	361 (221 to 528)	402 (245 to 594)	289 (170 to 424)	261 (154 to 374)	-42·7 (-54·3 to -25·2)
Syrian Arab Republic	All Ages	118 (87·6 to 149)	101 (73·6 to 138)	84·2 (60·7 to 122)	98·9 (71·0 to 138)	82·0 (57·9 to 115)	66·7 (48·4 to 91·7)	-42·9 (-57·6 to -23·9)
	15-49 years	30·8 (22·9 to 38·8)	21·2 (14·6 to 30·1)	17·5 (12·0 to 25·2)	20·4 (14·5 to 29·2)	20·4 (14·0 to 29·2)	16·4 (11·3 to 23·4)	-46·3 (-61·9 to -24·7)
	50-69 years	38·9 (27·0 to 51·8)	38·8 (26·4 to 56·5)	29·9 (20·6 to 46·7)	37·6 (25·8 to 57·0)	31·0 (20·8 to 46·2)	23·3 (15·7 to 34·1)	-39·6 (-58·1 to -11·9)

eTable 5. Risk deleted deaths due to all-form tuberculosis for alcohol use, smoking, and diabetes, and all three risk factors combined by age groups for 204 countries and territories in (2021) with percent change between 2015 observed deaths and 2021 all-risk deleted deaths.

Location	Age group	2015 Observed deaths	2021 Observed deaths	2021 Smoking deleted deaths	2021 Alcohol deleted deaths	2021 Diabetes deleted deaths	2021 All-risk deleted deaths	Percent Change
	70+ years	41·4 (32·4 to 51·2)	39·1 (30·2 to 52·5)	34·5 (25·8 to 48·8)	38·5 (29·1 to 52·1)	28·3 (20·9 to 39·1)	24·7 (18·5 to 35·4)	-39·9 (-53·3 to -21·3)
Tunisia	All Ages	260 (180 to 377)	232 (158 to 403)	182 (120 to 322)	220 (149 to 392)	179 (120 to 318)	135 (83·7 to 251)	-48·0 (-63·3 to -26·7)
	15-49 years	56·5 (36·2 to 86·9)	46·0 (31·8 to 77·2)	36·6 (25·4 to 62·9)	41·9 (26·8 to 71·2)	44·3 (30·7 to 74·3)	33·0 (22·0 to 56·7)	-41·3 (-56·6 to -20·5)
	50-69 years	73·2 (47·3 to 112)	70·5 (43·7 to 129)	51·8 (32·1 to 98·2)	66·4 (39·7 to 126)	53·5 (33·2 to 99·2)	37·6 (21·4 to 75·3)	-47·9 (-67·8 to -21·3)
	70+ years	125 (84·4 to 174)	113 (77·3 to 190)	90·5 (57·9 to 152)	109 (73·2 to 187)	78·4 (51·6 to 137)	61·2 (36·8 to 110)	-50·8 (-65·0 to -32·1)
Turkey	All Ages	1030 (913 to 1190)	883 (708 to 1110)	705 (519 to 935)	821 (607 to 1120)	697 (570 to 913)	518 (339 to 771)	-49·5 (-67·0 to -29·0)
	15-49 years	192 (167 to 222)	147 (119 to 188)	106 (74·6 to 148)	130 (80·4 to 192)	142 (115 to 182)	91·9 (54·9 to 144)	-52·0 (-71·8 to -31·4)
	50-69 years	328 (272 to 394)	293 (229 to 381)	213 (141 to 302)	268 (181 to 378)	240 (187 to 321)	163 (99·7 to 255)	-50·2 (-70·2 to -23·6)
	70+ years	482 (401 to 573)	430 (332 to 544)	373 (275 to 490)	410 (304 to 542)	302 (234 to 405)	251 (171 to 358)	-47·9 (-63·5 to -29·6)
United Arab Emirates	All Ages	39·3 (17·7 to 60·2)	34·3 (15·0 to 52·7)	28·6 (12·9 to 44·6)	31·3 (13·4 to 50·3)	28·2 (12·4 to 43·8)	21·5 (9·54 to 36·1)	-43·6 (-68·1 to -13·8)
	15-49 years	22·2 (8·53 to 36·7)	14·1 (5·71 to 22·9)	11·2 (4·61 to 18·5)	12·4 (4·60 to 21·4)	13·1 (5·30 to 20·8)	9·07 (3·34 to 16·5)	-58·1 (-81·1 to -33·3)
	50-69 years	8·18 (3·32 to 15·2)	9·17 (3·83 to 17·3)	7·08 (2·99 to 13·5)	8·22 (3·18 to 15·2)	6·24 (2·44 to 12·3)	4·11 (1·31 to 8·24)	-47·7 (-79·6 to -9·21)
	70+ years	8·30 (3·82 to 11·4)	10·7 (4·84 to 14·7)	10·0 (4·66 to 13·9)	10·4 (4·79 to 14·7)	8·58 (3·94 to 11·6)	8·00 (3·74 to 11·1)	-1·89 (-28·9 to 28·9)
Yemen	All Ages	841 (520 to 1240)	947 (597 to 1400)	781 (489 to 1190)	933 (590 to 1400)	840 (524 to 1280)	685 (435 to 1070)	-18·1 (-37·0 to 1·96)
	15-49 years	252 (157 to 396)	300 (192 to 499)	238 (144 to 416)	291 (190 to 489)	293 (186 to 488)	228 (138 to 403)	-9·20 (-34·2 to 21·2)
	50-69 years	229 (129 to 364)	279 (155 to 431)	211 (118 to 344)	275 (153 to 430)	239 (129 to 367)	178 (99·4 to 287)	-21·4 (-45·1 to 3·56)
	70+ years	209 (126 to 331)	253 (163 to 369)	216 (137 to 335)	251 (162 to 367)	193 (112 to 297)	164 (93·7 to 255)	-21·1 (-42·7 to 3·27)
South Asia	All Ages	573000 (536000 to 611000)	509000 (458000 to 591000)	436000 (363000 to 525000)	459000 (307000 to 589000)	435000 (381000 to 519000)	339000 (224000 to 447000)	-40·8 (-60·4 to -25·1)
	15-49 years	186000 (172000 to 202000)	166000 (149000 to 192000)	147000 (128000 to 172000)	147000 (95600 to 189000)	160000 (143000 to 185000)	127000 (83700 to 163000)	-31·7 (-55·3 to -13·2)
	50-69 years	213000 (198000 to 229000)	181000 (159000 to 216000)	147000 (115000 to 188000)	160000 (101000 to 214000)	151000 (128000 to 189000)	110000 (67700 to 153000)	-48·4 (-67·8 to -30·2)
	70+ years	148000 (137000 to 160000)	147000 (129000 to 171000)	126000 (103000 to 154000)	135000 (99600 to 169000)	108000 (91800 to 134000)	86800 (57800 to 114000)	-41·4 (-59·2 to -25·0)
Bangladesh	All Ages	31100 (27100 to 35500)	28700 (23100 to 35400)	23800 (18100 to 30600)	28200 (22000 to 34800)	24600 (19800 to 31000)	20000 (14700 to 26400)	-35·6 (-52·9 to -16·5)

eTable 5. Risk deleted deaths due to all-form tuberculosis for alcohol use, smoking, and diabetes, and all three risk factors combined by age groups for 204 countries and territories in (2021) with percent change between 2015 observed deaths and 2021 all-risk deleted deaths.

Location	Age group	2015 Observed deaths	2021 Observed deaths	2021 Smoking deleted deaths	2021 Alcohol deleted deaths	2021 Diabetes deleted deaths	2021 All-risk deleted deaths	Percent Change
	15-49 years	8740 (7270 to 10200)	7410 (5780 to 9160)	6330 (4690 to 8350)	7190 (5530 to 8950)	7170 (5600 to 8900)	5990 (4530 to 7920)	-31·2 (-49·0 to -13·2)
	50-69 years	10400 (8620 to 12700)	9370 (7120 to 12700)	7290 (5230 to 10400)	9150 (6720 to 12400)	8210 (6310 to 11200)	6270 (4350 to 9050)	-39·4 (-58·6 to -22·6)
	70+ years	9780 (7830 to 11600)	10800 (8140 to 13600)	9070 (6610 to 11800)	10700 (8100 to 13500)	8060 (6120 to 10500)	6650 (4590 to 8980)	-31·8 (-55·3 to -9·06)
Bhutan	All Ages	125 (69·4 to 204)	108 (59·1 to 193)	98·3 (55·2 to 176)	104 (53·7 to 191)	91·0 (49·4 to 159)	80·5 (43·2 to 144)	-35·4 (-50·9 to -19·5)
	15-49 years	33·2 (18·1 to 58·4)	27·4 (14·6 to 49·9)	24·9 (13·6 to 47·8)	25·9 (13·0 to 48·9)	26·6 (14·2 to 48·6)	23·1 (11·8 to 43·6)	-29·6 (-49·2 to -8·06)
	50-69 years	38·8 (21·0 to 72·5)	33·8 (18·8 to 67·9)	30·3 (17·3 to 60·5)	32·3 (16·1 to 64·1)	28·4 (15·7 to 55·4)	24·3 (12·7 to 47·9)	-36·9 (-56·1 to -17·0)
	70+ years	48·9 (25·7 to 78·4)	44·3 (23·4 to 73·8)	40·8 (22·4 to 70·9)	43·6 (22·8 to 73·6)	33·7 (17·9 to 56·7)	30·7 (16·5 to 53·2)	-37·1 (-50·6 to -17·7)
India	All Ages	458000 (419000 to 496000)	405000 (351000 to 500000)	348000 (287000 to 437000)	359000 (226000 to 480000)	344000 (292000 to 433000)	265000 (167000 to 368000)	-42·2 (-62·5 to -26·3)
	15-49 years	147000 (135000 to 160000)	131000 (115000 to 161000)	116000 (99800 to 144000)	114000 (69600 to 152000)	127000 (110000 to 156000)	99100 (61800 to 131000)	-32·7 (-57·8 to -13·5)
	50-69 years	177000 (161000 to 194000)	148000 (124000 to 186000)	121000 (94500 to 157000)	129000 (75700 to 179000)	122000 (100000 to 163000)	88700 (52200 to 130000)	-50·0 (-69·5 to -32·2)
	70+ years	119000 (108000 to 130000)	118000 (103000 to 144000)	102000 (83100 to 129000)	108000 (74500 to 141000)	87300 (72200 to 111000)	69200 (44300 to 94200)	-41·9 (-60·0 to -25·5)
Nepal	All Ages	8060 (5600 to 10300)	7010 (4730 to 10000)	5680 (3800 to 8440)	6140 (3310 to 9310)	5870 (4130 to 8340)	4220 (2200 to 6740)	-47·7 (-70·8 to -27·3)
	15-49 years	2080 (1530 to 2790)	1660 (1170 to 2480)	1460 (1020 to 2230)	1410 (700 to 2230)	1600 (1140 to 2390)	1220 (607 to 1960)	-41·5 (-67·1 to -14·9)
	50-69 years	3080 (2030 to 4210)	2700 (1740 to 4060)	2070 (1330 to 3300)	2310 (1120 to 3860)	2270 (1480 to 3440)	1510 (659 to 2610)	-51·2 (-76·9 to -23·9)
	70+ years	2690 (1810 to 3590)	2540 (1710 to 3610)	2040 (1190 to 3050)	2300 (1340 to 3290)	1890 (1250 to 2610)	1380 (699 to 2160)	-48·6 (-72·0 to -24·2)
Pakistan	All Ages	76300 (56000 to 95200)	68400 (48800 to 85900)	59000 (43100 to 78800)	65700 (46300 to 85600)	59800 (41900 to 77500)	50200 (35000 to 70700)	-33·5 (-55·1 to -4·96)
	15-49 years	28200 (21000 to 35800)	25500 (17900 to 32800)	22500 (16100 to 29800)	24100 (16200 to 32400)	24600 (17300 to 31900)	20800 (13700 to 28600)	-25·6 (-50·8 to 4·77)
	50-69 years	22200 (16000 to 29100)	21400 (14300 to 27900)	17000 (11100 to 25200)	20300 (13100 to 28000)	17600 (11200 to 24600)	13400 (7640 to 21600)	-38·8 (-65·1 to -0·735)
	70+ years	16500 (11300 to 21400)	15000 (10200 to 19200)	13000 (9170 to 17800)	14700 (9930 to 19000)	11100 (7360 to 15300)	9490 (6280 to 14100)	-41·7 (-60·2 to -14·5)
Southeast Asia, East Asia, and Oceania	All Ages	265000 (248000 to 286000)	237000 (215000 to 281000)	188000 (151000 to 227000)	211000 (148000 to 286000)	206000 (185000 to 245000)	148000 (97000 to 204000)	-44·1 (-64·1 to -24·4)
	15-49 years	77800 (72200 to 84400)	59700 (53700 to 68700)	49000 (41500 to 57300)	53100 (37900 to 69400)	58400 (52500 to 67400)	43700 (30900 to 58100)	-43·7 (-62·0 to -24·5)
	50-69 years	92300 (85400 to 99900)	90400 (81200 to 108000)	66700 (49900 to 83900)	79600 (52600 to 110000)	78700 (69200 to 94000)	52300 (30800 to 76400)	-43·3 (-66·9 to -18·9)

eTable 5. Risk deleted deaths due to all-form tuberculosis for alcohol use, smoking, and diabetes, and all three risk factors combined by age groups for 204 countries and territories in (2021) with percent change between 2015 observed deaths and 2021 all-risk deleted deaths.

Location	Age group	2015 Observed deaths	2021 Observed deaths	2021 Smoking deleted deaths	2021 Alcohol deleted deaths	2021 Diabetes deleted deaths	2021 All-risk deleted deaths	Percent Change
	70+ years	86500 (78700 to 95000)	81500 (73200 to 97300)	66900 (52400 to 83300)	73300 (52200 to 100000)	63100 (55400 to 77200)	47000 (30300 to 66800)	-45·7 (-65·4 to -24·8)
East Asia	All Ages	58500 (52700 to 65600)	50400 (42700 to 62700)	37100 (25700 to 49600)	41700 (20100 to 66800)	44000 (36600 to 56000)	27700 (13200 to 45400)	-52·5 (-77·0 to -25·1)
	15-49 years	13900 (12300 to 15500)	9710 (8230 to 12200)	7370 (5490 to 9810)	8060 (4150 to 13000)	9400 (7910 to 11800)	6210 (3660 to 10000)	-55·0 (-74·4 to -32·6)
	50-69 years	20600 (18200 to 22900)	19400 (16300 to 24400)	13200 (8340 to 19000)	15800 (6790 to 25900)	17100 (14000 to 22000)	9820 (3740 to 16900)	-52·2 (-81·5 to -19·3)
	70+ years	23000 (20800 to 26400)	20700 (17300 to 26500)	16000 (11200 to 21300)	17400 (8690 to 27400)	17000 (14000 to 22700)	11200 (5100 to 18300)	-51·3 (-76·8 to -20·6)
China	All Ages	45500 (39900 to 54300)	38700 (31300 to 52100)	28000 (18000 to 40800)	31800 (13500 to 55700)	33800 (26400 to 47000)	20800 (8350 to 36900)	-54·2 (-81·1 to -22·8)
	15-49 years	10800 (9390 to 12600)	7150 (5760 to 9690)	5430 (3750 to 7890)	5950 (3080 to 10500)	6910 (5500 to 9420)	4600 (2570 to 7640)	-57·4 (-75·7 to -36·2)
	50-69 years	15600 (13300 to 18600)	14400 (11100 to 19500)	9590 (5380 to 14700)	11600 (4380 to 21200)	12700 (9670 to 17700)	7110 (2180 to 13800)	-54·2 (-86·4 to -16·8)
	70+ years	18300 (16100 to 22000)	16700 (13600 to 22600)	12600 (8070 to 18000)	13900 (6090 to 23600)	13800 (10700 to 19600)	8750 (3240 to 15300)	-52·3 (-81·1 to -16·6)
Democratic People's Republic of Korea	All Ages	12200 (8870 to 16400)	11000 (7710 to 15200)	8490 (5380 to 12700)	9310 (4720 to 13600)	9710 (6800 to 13400)	6490 (3280 to 10000)	-46·4 (-71·2 to -23·0)
	15-49 years	2970 (2060 to 4480)	2500 (1770 to 3730)	1900 (1250 to 3010)	2060 (877 to 3220)	2430 (1710 to 3610)	1580 (701 to 2570)	-45·9 (-73·9 to -14·9)
	50-69 years	4910 (3470 to 6760)	4890 (3290 to 6720)	3480 (2080 to 5510)	4060 (1900 to 6280)	4340 (2920 to 6080)	2650 (1260 to 4380)	-45·7 (-73·2 to -20·1)
	70+ years	4110 (2790 to 5490)	3500 (2460 to 4630)	3010 (1960 to 4170)	3090 (1830 to 4300)	2840 (1990 to 3740)	2170 (1170 to 3230)	-47·0 (-68·6 to -29·2)
Taiwan (Province of China)	All Ages	803 (744 to 850)	688 (618 to 748)	549 (435 to 666)	591 (319 to 753)	546 (487 to 613)	379 (190 to 514)	-52·8 (-75·2 to -36·7)
	15-49 years	75·9 (67·9 to 84·2)	54·2 (48·1 to 61·0)	42·1 (33·5 to 52·0)	45·6 (22·3 to 64·8)	52·7 (46·8 to 59·5)	35·8 (19·9 to 50·5)	-52·9 (-72·2 to -36·2)
	50-69 years	155 (144 to 167)	139 (125 to 151)	97·6 (69·8 to 128)	111 (40·8 to 161)	115 (101 to 130)	64·8 (19·7 to 102)	-58·2 (-87·8 to -35·5)
	70+ years	569 (511 to 610)	493 (431 to 543)	407 (324 to 492)	433 (244 to 535)	377 (328 to 426)	276 (149 to 366)	-51·4 (-72·4 to -36·9)
Oceania	All Ages	3280 (2760 to 3950)	3390 (2840 to 4000)	2880 (2360 to 3630)	3160 (2380 to 3910)	2890 (2430 to 3380)	2300 (1740 to 2970)	-29·7 (-48·3 to -11·9)
	15-49 years	1290 (1090 to 1570)	1280 (1010 to 1560)	1050 (822 to 1350)	1160 (771 to 1510)	1230 (969 to 1490)	925 (641 to 1230)	-28·0 (-51·4 to -7·91)
	50-69 years	1010 (817 to 1210)	1140 (914 to 1460)	910 (692 to 1220)	1060 (791 to 1420)	886 (729 to 1130)	663 (434 to 940)	-34·3 (-57·3 to -11·8)
	70+ years	613 (506 to 750)	661 (531 to 839)	605 (488 to 763)	626 (488 to 781)	462 (369 to 611)	402 (298 to 509)	-34·0 (-52·5 to -16·1)
American Samoa	All Ages	1·07 (0·962 to 1·22)	1·12 (0·966 to 1·39)	0·918 (0·718 to 1·14)	1·09 (0·911 to 1·38)	0·827 (0·697 to 1·06)	0·661 (0·503 to 0·866)	-38·5 (-50·5 to -24·2)

eTable 5. Risk deleted deaths due to all-form tuberculosis for alcohol use, smoking, and diabetes, and all three risk factors combined by age groups for 204 countries and territories in (2021) with percent change between 2015 observed deaths and 2021 all-risk deleted deaths.

Location	Age group	2015 Observed deaths	2021 Observed deaths	2021 Smoking deleted deaths	2021 Alcohol deleted deaths	2021 Diabetes deleted deaths	2021 All-risk deleted deaths	Percent Change
	15-49 years	0·323 (0·260 to 0·387)	0·299 (0·226 to 0·392)	0·243 (0·180 to 0·320)	0·287 (0·219 to 0·386)	0·270 (0·206 to 0·348)	0·215 (0·165 to 0·287)	-33·5 (-47·7 to -17·2)
	50-69 years	0·412 (0·350 to 0·478)	0·464 (0·391 to 0·579)	0·349 (0·245 to 0·473)	0·451 (0·366 to 0·564)	0·307 (0·250 to 0·399)	0·225 (0·145 to 0·326)	-45·4 (-61·8 to -25·8)
	70+ years	0·315 (0·267 to 0·371)	0·344 (0·278 to 0·430)	0·308 (0·247 to 0·393)	0·339 (0·268 to 0·430)	0·232 (0·177 to 0·301)	0·205 (0·149 to 0·265)	-34·9 (-47·5 to -21·3)
Cook Islands	All Ages	0·304 (0·266 to 0·355)	0·277 (0·233 to 0·351)	0·232 (0·186 to 0·296)	0·215 (0·0688 to 0·365)	0·192 (0·159 to 0·253)	0·127 (0·0390 to 0·208)	-58·1 (-86·1 to -32·9)
	15-49 years	0·0550 (0·0438 to 0·0661)	0·0429 (0·0330 to 0·0564)	0·0356 (0·0253 to 0·0467)	0·0330 (0·0133 to 0·0592)	0·0391 (0·0301 to 0·0509)	0·0260 (0·0115 to 0·0447)	-52·6 (-77·6 to -20·3)
	50-69 years	0·110 (0·0951 to 0·131)	0·104 (0·0833 to 0·139)	0·0787 (0·0538 to 0·108)	0·0759 (0·0141 to 0·142)	0·0635 (0·0491 to 0·0892)	0·0335 (0·00158 to 0·0679)	-69·5 (-98·4 to -41·4)
	70+ years	0·137 (0·116 to 0·166)	0·127 (0·105 to 0·156)	0·116 (0·0945 to 0·143)	0·104 (0·0407 to 0·155)	0·0871 (0·0701 to 0·109)	0·0661 (0·0248 to 0·0960)	-51·7 (-80·6 to -27·3)
Fiji	All Ages	52·6 (42·4 to 66·7)	47·3 (37·0 to 61·4)	40·2 (30·2 to 52·5)	42·4 (25·8 to 61·7)	36·3 (28·6 to 48·1)	28·1 (17·6 to 40·1)	-46·5 (-64·3 to -27·3)
	15-49 years	14·3 (11·7 to 18·7)	11·9 (9·23 to 15·8)	10·3 (7·67 to 13·6)	10·3 (6·05 to 15·0)	11·2 (8·64 to 14·8)	8·51 (5·31 to 12·4)	-40·2 (-62·0 to -14·7)
	50-69 years	22·1 (17·7 to 27·8)	20·0 (14·8 to 28·2)	15·7 (11·0 to 21·7)	17·7 (10·1 to 27·3)	14·1 (10·7 to 19·5)	9·93 (5·44 to 14·7)	-54·9 (-73·8 to -37·7)
	70+ years	13·9 (11·2 to 17·9)	13·4 (10·5 to 17·6)	12·3 (9·49 to 15·9)	12·4 (8·14 to 16·9)	9·13 (7·01 to 12·4)	7·75 (5·12 to 10·6)	-44·2 (-61·2 to -24·2)
Guam	All Ages	8·90 (7·92 to 10·1)	9·20 (7·71 to 10·7)	7·71 (6·15 to 9·29)	7·96 (4·56 to 10·9)	7·02 (5·88 to 8·38)	5·13 (3·05 to 7·44)	-42·3 (-68·5 to -21·2)
	15-49 years	2·42 (2·13 to 2·80)	2·49 (2·07 to 2·92)	2·12 (1·69 to 2·56)	2·15 (1·33 to 2·87)	2·33 (1·93 to 2·71)	1·77 (1·18 to 2·44)	-27·0 (-52·1 to -4·65)
	50-69 years	3·70 (3·17 to 4·36)	3·82 (3·13 to 4·72)	2·96 (2·13 to 3·89)	3·23 (1·62 to 4·77)	2·83 (2·28 to 3·44)	1·86 (0·968 to 2·96)	-49·6 (-76·8 to -24·6)
	70+ years	2·60 (2·18 to 2·99)	2·71 (2·22 to 3·27)	2·46 (1·92 to 2·96)	2·41 (1·38 to 3·32)	1·68 (1·30 to 2·23)	1·33 (0·674 to 1·94)	-48·8 (-75·5 to -27·5)
Kiribati	All Ages	89·7 (67·5 to 115)	85·9 (63·1 to 121)	61·8 (44·1 to 83·1)	82·3 (61·2 to 114)	68·7 (50·0 to 94·6)	48·3 (34·2 to 69·7)	-46·2 (-60·7 to -30·4)
	15-49 years	33·8 (23·8 to 44·0)	31·1 (21·1 to 44·6)	23·3 (15·8 to 33·1)	29·2 (19·3 to 42·3)	28·9 (19·5 to 41·6)	20·6 (13·3 to 30·5)	-39·1 (-55·1 to -21·9)
	50-69 years	32·9 (24·5 to 43·5)	33·7 (24·3 to 49·0)	21·0 (13·1 to 30·8)	32·3 (23·5 to 45·1)	23·7 (16·8 to 33·9)	14·2 (8·51 to 22·3)	-56·9 (-74·2 to -33·7)
	70+ years	17·7 (13·2 to 21·9)	16·8 (13·4 to 21·3)	13·3 (9·56 to 17·1)	16·5 (12·7 to 20·9)	11·8 (9·23 to 15·3)	9·27 (6·80 to 12·3)	-47·4 (-61·8 to -32·3)
Marshall Islands	All Ages	35·0 (21·9 to 46·5)	33·4 (20·2 to 45·0)	28·2 (17·4 to 40·0)	30·2 (16·0 to 44·0)	23·0 (14·3 to 31·2)	17·6 (9·57 to 25·1)	-49·7 (-67·2 to -36·5)
	15-49 years	14·7 (9·57 to 19·4)	13·1 (8·13 to 18·2)	10·9 (6·87 to 15·0)	11·5 (6·14 to 16·9)	11·8 (7·48 to 16·5)	8·81 (4·62 to 13·2)	-40·2 (-61·9 to -23·4)
	50-69 years	14·0 (8·43 to 18·6)	13·4 (7·51 to 18·8)	11·0 (6·48 to 16·4)	12·2 (6·01 to 18·8)	7·57 (4·31 to 11·2)	5·63 (2·66 to 8·63)	-59·6 (-77·1 to -43·1)

eTable 5. Risk deleted deaths due to all-form tuberculosis for alcohol use, smoking, and diabetes, and all three risk factors combined by age groups for 204 countries and territories in (2021) with percent change between 2015 observed deaths and 2021 all-risk deleted deaths.

Location	Age group	2015 Observed deaths	2021 Observed deaths	2021 Smoking deleted deaths	2021 Alcohol deleted deaths	2021 Diabetes deleted deaths	2021 All-risk deleted deaths	Percent Change
	70+ years	4·94 (3·09 to 6·59)	5·91 (3·62 to 8·29)	5·31 (3·34 to 7·60)	5·47 (3·06 to 7·92)	2·61 (1·50 to 3·94)	2·14 (1·09 to 3·14)	-56·3 (-73·7 to -37·0)
Micronesia (Federated States of)	All Ages	12·9 (9·27 to 16·6)	12·8 (9·10 to 17·4)	10·0 (6·89 to 13·6)	11·5 (6·89 to 16·7)	10·3 (7·31 to 14·0)	7·47 (4·22 to 10·6)	-41·8 (-65·6 to -23·3)
	15-49 years	4·69 (3·24 to 6·16)	4·33 (3·07 to 5·82)	3·45 (2·36 to 4·63)	3·81 (2·03 to 5·54)	4·05 (2·85 to 5·46)	2·90 (1·54 to 4·10)	-38·1 (-66·5 to -18·2)
	50-69 years	5·28 (3·65 to 7·37)	5·58 (3·68 to 8·01)	4·01 (2·50 to 5·80)	5·01 (2·78 to 7·73)	4·09 (2·72 to 5·84)	2·69 (1·39 to 4·15)	-48·8 (-72·9 to -22·5)
	70+ years	2·44 (1·84 to 3·14)	2·53 (1·88 to 3·41)	2·26 (1·66 to 3·11)	2·39 (1·71 to 3·38)	1·82 (1·32 to 2·48)	1·55 (1·04 to 2·19)	-36·3 (-50·1 to -19·3)
Nauru	All Ages	3·38 (2·14 to 4·86)	2·61 (1·83 to 3·59)	2·19 (1·47 to 3·14)	2·29 (1·30 to 3·44)	2·28 (1·65 to 3·17)	1·74 (1·02 to 2·60)	-48·3 (-65·2 to -32·3)
	15-49 years	1·37 (0·882 to 1·88)	1·05 (0·747 to 1·54)	0·843 (0·551 to 1·18)	0·869 (0·400 to 1·49)	0·987 (0·711 to 1·45)	0·672 (0·313 to 1·07)	-50·7 (-75·8 to -25·9)
	50-69 years	1·21 (0·688 to 1·72)	0·916 (0·552 to 1·26)	0·724 (0·419 to 1·04)	0·799 (0·405 to 1·24)	0·713 (0·449 to 1·01)	0·511 (0·265 to 0·785)	-57·2 (-71·7 to -40·1)
	70+ years	0·625 (0·370 to 1·20)	0·525 (0·357 to 0·935)	0·504 (0·340 to 0·915)	0·503 (0·339 to 0·852)	0·461 (0·313 to 0·840)	0·434 (0·295 to 0·761)	-29·5 (-43·9 to -10·7)
Niue	All Ages	0·119 (0·0923 to 0·149)	0·108 (0·0806 to 0·138)	0·0917 (0·0679 to 0·119)	0·0926 (0·0501 to 0·138)	0·0792 (0·0598 to 0·103)	0·0591 (0·0334 to 0·0873)	-50·5 (-70·4 to -28·1)
	15-49 years	0·0199 (0·0133 to 0·0280)	0·0175 (0·0118 to 0·0243)	0·0149 (0·00955 to 0·0207)	0·0144 (0·00688 to 0·0241)	0·0160 (0·0111 to 0·0225)	0·0116 (0·00598 to 0·0190)	-41·3 (-69·9 to -8·54)
	50-69 years	0·0435 (0·0308 to 0·0592)	0·0438 (0·0300 to 0·0602)	0·0338 (0·0218 to 0·0468)	0·0362 (0·0164 to 0·0609)	0·0305 (0·0214 to 0·0422)	0·0199 (0·00905 to 0·0328)	-54·1 (-78·8 to -24·7)
	70+ years	0·0533 (0·0409 to 0·0646)	0·0440 (0·0341 to 0·0548)	0·0405 (0·0296 to 0·0504)	0·0396 (0·0232 to 0·0538)	0·0303 (0·0226 to 0·0374)	0·0253 (0·0151 to 0·0352)	-52·6 (-70·4 to -35·0)
Northern Mariana Islands	All Ages	4·57 (3·61 to 6·02)	4·64 (3·55 to 5·88)	3·70 (2·62 to 4·90)	4·04 (2·14 to 5·64)	3·33 (2·48 to 4·43)	2·33 (1·19 to 3·64)	-48·6 (-73·6 to -18·1)
	15-49 years	1·16 (0·860 to 1·59)	0·888 (0·688 to 1·20)	0·753 (0·561 to 1·03)	0·781 (0·432 to 1·11)	0·817 (0·628 to 1·10)	0·630 (0·369 to 0·865)	-45·3 (-69·6 to -25·0)
	50-69 years	2·25 (1·74 to 2·96)	2·28 (1·67 to 2·94)	1·59 (0·974 to 2·22)	1·89 (0·720 to 2·91)	1·42 (0·999 to 1·97)	0·741 (0·0657 to 1·52)	-66·7 (-97·1 to -33·9)
	70+ years	1·08 (0·868 to 1·39)	1·43 (0·990 to 2·24)	1·31 (0·875 to 2·10)	1·32 (0·900 to 2·14)	1·05 (0·706 to 1·78)	0·912 (0·598 to 1·54)	-14·9 (-41·4 to 38·5)
Palau	All Ages	1·51 (1·23 to 1·82)	1·54 (1·21 to 1·98)	1·28 (0·984 to 1·74)	1·30 (0·628 to 1·87)	1·17 (0·925 to 1·53)	0·837 (0·406 to 1·29)	-44·4 (-73·0 to -19·5)
	15-49 years	0·518 (0·411 to 0·645)	0·445 (0·359 to 0·568)	0·373 (0·288 to 0·501)	0·364 (0·182 to 0·560)	0·407 (0·327 to 0·520)	0·286 (0·146 to 0·458)	-44·3 (-74·3 to -20·6)
	50-69 years	0·626 (0·472 to 0·788)	0·675 (0·489 to 0·990)	0·529 (0·370 to 0·795)	0·564 (0·242 to 0·871)	0·476 (0·343 to 0·711)	0·314 (0·126 to 0·520)	-49·5 (-80·3 to -22·4)
	70+ years	0·348 (0·284 to 0·437)	0·401 (0·311 to 0·525)	0·364 (0·284 to 0·461)	0·360 (0·206 to 0·493)	0·266 (0·203 to 0·367)	0·221 (0·120 to 0·308)	-36·3 (-60·8 to -10·1)
Papua New Guinea	All Ages	2770 (2300 to 3380)	2900 (2380 to 3470)	2480 (2010 to 3150)	2700 (1990 to 3340)	2480 (2060 to 2950)	1990 (1500 to 2590)	-27·8 (-47·0 to -9·58)

eTable 5. Risk deleted deaths due to all-form tuberculosis for alcohol use, smoking, and diabetes, and all three risk factors combined by age groups for 204 countries and territories in (2021) with percent change between 2015 observed deaths and 2021 all-risk deleted deaths.

Location	Age group	2015 Observed deaths	2021 Observed deaths	2021 Smoking deleted deaths	2021 Alcohol deleted deaths	2021 Diabetes deleted deaths	2021 All-risk deleted deaths	Percent Change
	15-49 years	1100 (911 to 1360)	1100 (847 to 1370)	907 (693 to 1180)	1000 (650 to 1320)	1060 (814 to 1310)	800 (547 to 1090)	-27·0 (-51·2 to -6·04)
	50-69 years	835 (655 to 1010)	958 (749 to 1270)	775 (582 to 1060)	890 (652 to 1210)	753 (609 to 982)	571 (378 to 825)	-31·2 (-55·3 to -6·35)
	70+ years	505 (402 to 629)	554 (430 to 722)	510 (399 to 659)	525 (401 to 673)	388 (300 to 528)	340 (246 to 441)	-32·2 (-52·2 to -12·4)
Samoa	All Ages	15·0 (11·6 to 19·5)	14·2 (10·7 to 18·8)	11·1 (7·78 to 15·3)	12·9 (9·15 to 18·5)	10·9 (8·17 to 14·8)	7·92 (5·51 to 11·1)	-46·7 (-62·9 to -28·6)
	15-49 years	4·10 (2·92 to 5·65)	3·82 (2·65 to 4·92)	3·05 (2·15 to 4·08)	3·38 (2·19 to 5·14)	3·50 (2·45 to 4·49)	2·56 (1·69 to 3·82)	-36·7 (-56·2 to -9·62)
	50-69 years	5·47 (3·99 to 7·19)	5·39 (3·90 to 7·64)	3·71 (2·15 to 5·60)	4·84 (3·14 to 7·07)	3·80 (2·67 to 5·34)	2·35 (1·35 to 3·72)	-56·5 (-76·0 to -36·2)
	70+ years	4·85 (3·70 to 6·72)	4·45 (3·23 to 6·10)	3·79 (2·76 to 5·36)	4·17 (3·06 to 5·70)	3·10 (2·20 to 4·36)	2·50 (1·74 to 3·60)	-48·1 (-64·2 to -32·7)
Solomon Islands	All Ages	83·6 (62·2 to 110)	84·9 (66·5 to 109)	68·6 (52·0 to 89·5)	80·8 (62·5 to 107)	74·6 (58·3 to 95·3)	58·1 (44·4 to 78·8)	-30·1 (-44·2 to -9·49)
	15-49 years	35·3 (25·0 to 47·4)	35·7 (26·2 to 44·4)	28·8 (21·4 to 37·7)	33·2 (24·2 to 44·1)	34·2 (25·0 to 42·5)	26·0 (18·9 to 36·2)	-25·9 (-45·7 to -0·534)
	50-69 years	25·0 (17·5 to 34·2)	27·0 (20·5 to 37·6)	19·3 (13·1 to 27·8)	25·8 (18·9 to 36·9)	21·7 (16·1 to 30·2)	15·0 (10·0 to 22·1)	-39·7 (-56·8 to -14·5)
	70+ years	18·0 (13·4 to 22·3)	17·8 (14·1 to 22·0)	16·1 (12·4 to 20·0)	17·4 (13·6 to 22·0)	14·3 (11·3 to 17·7)	12·8 (9·96 to 16·4)	-28·6 (-42·3 to -11·6)
Tokelau	All Ages	0·102 (0·0726 to 0·143)	0·0852 (0·0653 to 0·117)	0·0724 (0·0532 to 0·0976)	0·0760 (0·0456 to 0·107)	0·0590 (0·0441 to 0·0835)	0·0455 (0·0262 to 0·0644)	-55·2 (-71·2 to -39·4)
	15-49 years	0·0194 (0·0134 to 0·0293)	0·0172 (0·0129 to 0·0241)	0·0144 (0·0103 to 0·0206)	0·0149 (0·00716 to 0·0221)	0·0157 (0·0115 to 0·0221)	0·0116 (0·00621 to 0·0171)	-39·8 (-66·1 to -14·3)
	50-69 years	0·0337 (0·0233 to 0·0484)	0·0290 (0·0203 to 0·0415)	0·0216 (0·0140 to 0·0315)	0·0248 (0·0112 to 0·0379)	0·0158 (0·0112 to 0·0231)	0·00925 (0·00286 to 0·0173)	-72·5 (-91·8 to -52·2)
	70+ years	0·0475 (0·0336 to 0·0671)	0·0376 (0·0275 to 0·0498)	0·0350 (0·0255 to 0·0463)	0·0350 (0·0229 to 0·0473)	0·0262 (0·0188 to 0·0367)	0·0234 (0·0147 to 0·0326)	-50·4 (-61·5 to -37·9)
Tonga	All Ages	5·79 (4·52 to 7·52)	5·01 (3·81 to 6·44)	3·93 (2·87 to 5·14)	4·75 (3·48 to 6·13)	3·84 (2·87 to 5·10)	2·90 (2·08 to 3·98)	-49·6 (-63·8 to -35·0)
	15-49 years	1·31 (0·951 to 1·74)	1·08 (0·743 to 1·44)	0·889 (0·650 to 1·23)	0·999 (0·656 to 1·38)	0·999 (0·700 to 1·35)	0·776 (0·535 to 1·08)	-40·1 (-55·8 to -25·3)
	50-69 years	2·02 (1·46 to 2·93)	1·85 (1·34 to 2·49)	1·32 (0·833 to 1·82)	1·74 (1·21 to 2·43)	1·35 (0·955 to 1·88)	0·921 (0·566 to 1·33)	-53·8 (-72·4 to -37·5)
	70+ years	2·25 (1·80 to 3·05)	1·93 (1·54 to 2·54)	1·57 (1·16 to 2·10)	1·86 (1·44 to 2·38)	1·33 (1·03 to 1·78)	1·05 (0·742 to 1·48)	-53·0 (-67·0 to -38·2)
Tuvalu	All Ages	5·08 (3·31 to 6·25)	4·57 (3·18 to 5·60)	3·75 (2·54 to 4·89)	4·16 (2·51 to 5·50)	3·73 (2·67 to 4·68)	2·82 (1·71 to 3·90)	-44·4 (-62·2 to -25·9)
	15-49 years	1·34 (0·849 to 1·73)	1·20 (0·828 to 1·58)	0·987 (0·623 to 1·35)	1·06 (0·583 to 1·47)	1·15 (0·801 to 1·51)	0·850 (0·485 to 1·20)	-36·1 (-58·8 to -11·6)
	50-69 years	2·04 (1·35 to 2·61)	1·81 (1·21 to 2·39)	1·37 (0·856 to 2·06)	1·64 (0·968 to 2·38)	1·42 (0·972 to 1·94)	0·969 (0·545 to 1·54)	-52·4 (-73·2 to -34·8)

eTable 5. Risk deleted deaths due to all-form tuberculosis for alcohol use, smoking, and diabetes, and all three risk factors combined by age groups for 204 countries and territories in (2021) with percent change between 2015 observed deaths and 2021 all-risk deleted deaths.

Location	Age group	2015 Observed deaths	2021 Observed deaths	2021 Smoking deleted deaths	2021 Alcohol deleted deaths	2021 Diabetes deleted deaths	2021 All-risk deleted deaths	Percent Change
	70+ years	1·54 (1·02 to 1·98)	1·42 (0·991 to 1·78)	1·27 (0·890 to 1·62)	1·34 (0·916 to 1·81)	1·04 (0·697 to 1·31)	0·872 (0·548 to 1·17)	-42·7 (-56·9 to -22·4)
Vanuatu	All Ages	32·5 (18·9 to 47·0)	35·0 (21·5 to 49·0)	30·9 (19·6 to 42·5)	31·2 (18·9 to 45·7)	28·3 (17·1 to 39·6)	22·4 (13·4 to 33·6)	-29·9 (-54·1 to -6·50)
	15-49 years	12·1 (6·93 to 17·5)	13·0 (7·63 to 18·8)	11·6 (7·28 to 16·3)	11·3 (6·41 to 16·6)	12·3 (7·31 to 17·9)	9·75 (5·72 to 14·6)	-18·4 (-45·3 to 11·2)
	50-69 years	11·6 (6·01 to 17·6)	12·8 (6·84 to 19·4)	10·8 (6·05 to 15·8)	11·4 (6·43 to 17·9)	9·69 (5·05 to 14·6)	7·40 (4·09 to 12·3)	-34·5 (-61·8 to -8·15)
	70+ years	6·95 (4·46 to 9·54)	7·49 (5·18 to 10·9)	6·66 (4·62 to 9·54)	6·79 (3·83 to 10·3)	4·53 (2·99 to 6·79)	3·53 (1·57 to 5·41)	-48·4 (-75·9 to -26·4)
Southeast Asia	All Ages	204000 (186000 to 221000)	183000 (165000 to 217000)	148000 (118000 to 179000)	166000 (123000 to 212000)	159000 (141000 to 188000)	118000 (81800 to 155000)	-41·8 (-61·5 to -24·1)
	15-49 years	62600 (57700 to 68900)	48700 (43800 to 56000)	40600 (34400 to 47600)	43900 (32400 to 56100)	47800 (42900 to 55100)	36600 (26700 to 47400)	-41·5 (-59·2 to -23·1)
	50-69 years	70700 (64100 to 77300)	69800 (61600 to 84500)	52600 (39000 to 67700)	62800 (43200 to 83800)	60600 (52500 to 73300)	41800 (25800 to 58100)	-40·7 (-63·9 to -18·7)
	70+ years	62900 (55000 to 69400)	60100 (53200 to 71700)	50300 (39800 to 61700)	55300 (42100 to 71000)	45600 (39600 to 55200)	35400 (23800 to 48000)	-43·6 (-62·6 to -26·4)
Cambodia	All Ages	7570 (5300 to 9670)	6900 (4650 to 9440)	5220 (3660 to 7700)	5640 (2670 to 9420)	6070 (4100 to 8390)	3870 (1790 to 6870)	-48·4 (-78·8 to -20·8)
	15-49 years	2390 (1750 to 3180)	1920 (1320 to 2720)	1580 (1110 to 2270)	1540 (659 to 2710)	1890 (1310 to 2680)	1290 (592 to 2260)	-45·5 (-76·9 to -14·9)
	50-69 years	2890 (1980 to 3990)	2840 (1830 to 3870)	1980 (1300 to 3270)	2260 (883 to 4060)	2470 (1610 to 3430)	1410 (494 to 2710)	-50·4 (-83·5 to -20·0)
	70+ years	1930 (1220 to 2510)	1920 (1330 to 2720)	1440 (875 to 2270)	1620 (780 to 2680)	1490 (1060 to 2140)	955 (391 to 1800)	-50·1 (-79·0 to -25·6)
Indonesia	All Ages	102000 (87000 to 117000)	93100 (80700 to 112000)	74300 (56000 to 95000)	91200 (77400 to 108000)	81300 (68400 to 98000)	64000 (43900 to 85200)	-36·9 (-57·1 to -19·2)
	15-49 years	30400 (26600 to 35800)	24400 (20800 to 29900)	19800 (15200 to 25800)	23600 (19300 to 29200)	23900 (20300 to 29500)	19000 (13500 to 25100)	-37·3 (-55·8 to -17·5)
	50-69 years	37200 (31200 to 43400)	37300 (31100 to 45400)	28100 (19200 to 38100)	36500 (30000 to 44200)	32700 (26800 to 40400)	24300 (15400 to 33800)	-34·5 (-58·6 to -11·9)
	70+ years	30600 (25600 to 34800)	29300 (25300 to 35400)	24200 (18500 to 30500)	28900 (24900 to 35400)	22500 (17800 to 27500)	18500 (13100 to 23800)	-39·2 (-57·7 to -21·6)
Lao People's Democratic Republic	All Ages	2220 (1440 to 2970)	1880 (1240 to 2600)	1530 (996 to 2190)	1510 (635 to 2470)	1630 (1070 to 2250)	1100 (479 to 1760)	-50·1 (-76·1 to -23·1)
	15-49 years	801 (537 to 1090)	618 (391 to 906)	529 (342 to 790)	483 (175 to 823)	607 (384 to 888)	415 (158 to 698)	-47·9 (-77·7 to -20·1)
	50-69 years	737 (470 to 1020)	677 (416 to 952)	506 (296 to 737)	529 (161 to 969)	563 (348 to 806)	336 (104 to 598)	-54·0 (-84·4 to -20·3)
	70+ years	514 (305 to 690)	473 (300 to 664)	384 (232 to 560)	396 (169 to 616)	354 (219 to 507)	244 (95·9 to 377)	-52·0 (-75·6 to -29·0)
Malaysia	All Ages	2260 (2000 to 2570)	2350 (2020 to 2820)	2000 (1670 to 2420)	2220 (1640 to 2800)	1950 (1690 to 2390)	1590 (1090 to 2010)	-29·8 (-49·8 to -14·7)

eTable 5. Risk deleted deaths due to all-form tuberculosis for alcohol use, smoking, and diabetes, and all three risk factors combined by age groups for 204 countries and territories in (2021) with percent change between 2015 observed deaths and 2021 all-risk deleted deaths.

Location	Age group	2015 Observed deaths	2021 Observed deaths	2021 Smoking deleted deaths	2021 Alcohol deleted deaths	2021 Diabetes deleted deaths	2021 All-risk deleted deaths	Percent Change
	15-49 years	705 (586 to 859)	696 (568 to 884)	616 (484 to 783)	657 (484 to 860)	680 (556 to 866)	577 (436 to 752)	-18·2 (-36·5 to -3·60)
	50-69 years	838 (728 to 962)	826 (675 to 990)	674 (533 to 860)	769 (524 to 974)	690 (570 to 870)	531 (349 to 693)	-36·5 (-58·1 to -19·4)
	70+ years	687 (583 to 793)	803 (673 to 1000)	690 (552 to 872)	767 (582 to 955)	556 (426 to 749)	457 (293 to 599)	-33·3 (-56·1 to -12·4)
Maldives	All Ages	18·5 (16·5 to 20·8)	17·6 (13·8 to 23·5)	13·5 (9·13 to 18·5)	16·8 (12·3 to 22·5)	14·4 (11·1 to 19·6)	10·5 (6·90 to 14·7)	-43·2 (-62·7 to -19·7)
	15-49 years	6·35 (5·52 to 7·39)	6·76 (4·93 to 8·94)	5·60 (3·88 to 7·47)	6·35 (4·21 to 8·67)	6·64 (4·83 to 8·79)	5·24 (3·58 to 7·10)	-17·3 (-41·4 to 10·5)
	50-69 years	4·58 (3·73 to 5·74)	4·90 (3·44 to 6·62)	3·58 (2·14 to 5·06)	4·62 (3·31 to 6·59)	4·11 (2·82 to 5·62)	2·84 (1·62 to 4·29)	-37·9 (-62·3 to -8·88)
	70+ years	6·86 (5·95 to 7·84)	5·60 (4·43 to 7·25)	4·00 (2·50 to 5·48)	5·41 (4·10 to 7·24)	3·29 (2·43 to 4·66)	2·05 (0·857 to 3·40)	-70·2 (-87·7 to -53·4)
Mauritius	All Ages	16·9 (15·6 to 18·2)	16·3 (14·8 to 17·7)	12·9 (10·8 to 15·7)	13·9 (7·69 to 18·4)	13·2 (11·7 to 15·0)	9·31 (5·36 to 12·8)	-44·8 (-67·9 to -25·6)
	15-49 years	5·45 (4·81 to 6·25)	4·97 (4·24 to 5·81)	4·18 (3·39 to 5·16)	4·32 (2·61 to 5·76)	4·74 (4·02 to 5·53)	3·59 (2·35 to 4·81)	-34·2 (-55·1 to -18·0)
	50-69 years	7·19 (6·56 to 7·86)	7·01 (6·31 to 7·82)	5·15 (4·11 to 6·64)	5·80 (2·77 to 8·06)	5·50 (4·78 to 6·21)	3·49 (1·66 to 5·16)	-51·5 (-76·9 to -28·3)
	70+ years	4·09 (3·74 to 4·43)	4·19 (3·81 to 4·58)	3·47 (2·87 to 4·02)	3·64 (2·20 to 4·60)	2·91 (2·43 to 3·42)	2·13 (1·18 to 3·01)	-47·9 (-70·8 to -25·6)
Myanmar	All Ages	22100 (18800 to 26500)	17000 (13300 to 22300)	14500 (11000 to 18700)	14900 (8820 to 21600)	14300 (11200 to 18500)	10900 (6370 to 15800)	-50·5 (-70·6 to -31·7)
	15-49 years	7900 (6440 to 9500)	4990 (3900 to 6700)	4460 (3440 to 6050)	4300 (2660 to 6220)	4870 (3810 to 6470)	3830 (2450 to 5550)	-51·4 (-68·9 to -35·0)
	50-69 years	7070 (5560 to 8900)	6200 (4530 to 8410)	5010 (3510 to 6960)	5310 (2750 to 8190)	5070 (3620 to 6820)	3560 (1760 to 5540)	-49·6 (-75·0 to -24·3)
	70+ years	5790 (4640 to 7280)	5120 (3700 to 7000)	4330 (3100 to 5790)	4560 (2650 to 6410)	3660 (2530 to 4960)	2790 (1500 to 4070)	-51·8 (-72·8 to -30·3)
Philippines	All Ages	28000 (26200 to 30100)	26200 (21500 to 30800)	20700 (15100 to 26700)	20600 (7490 to 33200)	23000 (18400 to 27600)	14500 (4330 to 23700)	-48·1 (-84·5 to -15·1)
	15-49 years	8360 (7770 to 9000)	7410 (6000 to 8780)	5910 (4470 to 7590)	5630 (1740 to 9890)	7280 (5860 to 8600)	4510 (1270 to 7910)	-45·9 (-84·8 to -2·80)
	50-69 years	11000 (10200 to 11900)	10700 (8580 to 13100)	7800 (5120 to 10800)	8190 (2350 to 14000)	9370 (7140 to 11600)	5270 (957 to 9610)	-52·1 (-91·5 to -14·9)
	70+ years	7180 (6690 to 7680)	7080 (6010 to 8140)	6040 (4700 to 7500)	5830 (2740 to 8420)	5400 (4330 to 6590)	3810 (1290 to 5700)	-46·9 (-81·8 to -22·3)
Seychelles	All Ages	4·37 (3·99 to 4·89)	3·69 (3·16 to 4·44)	2·83 (2·10 to 3·74)	3·09 (1·62 to 4·45)	2·85 (2·37 to 3·55)	1·83 (0·979 to 2·67)	-58·0 (-78·0 to -39·1)
	15-49 years	1·47 (1·25 to 1·72)	1·08 (0·869 to 1·31)	0·856 (0·629 to 1·16)	0·886 (0·454 to 1·34)	1·02 (0·807 to 1·24)	0·679 (0·362 to 0·992)	-53·8 (-76·1 to -34·7)
	50-69 years	1·58 (1·33 to 1·89)	1·40 (1·11 to 1·76)	0·981 (0·630 to 1·37)	1·13 (0·452 to 1·74)	1·02 (0·792 to 1·34)	0·556 (0·193 to 0·914)	-64·8 (-88·5 to -41·9)

eTable 5. Risk deleted deaths due to all-form tuberculosis for alcohol use, smoking, and diabetes, and all three risk factors combined by age groups for 204 countries and territories in (2021) with percent change between 2015 observed deaths and 2021 all-risk deleted deaths.

Location	Age group	2015 Observed deaths	2021 Observed deaths	2021 Smoking deleted deaths	2021 Alcohol deleted deaths	2021 Diabetes deleted deaths	2021 All-risk deleted deaths	Percent Change
	70+ years	1·26 (1·13 to 1·45)	1·17 (0·991 to 1·41)	0·955 (0·768 to 1·19)	1·04 (0·622 to 1·39)	0·776 (0·642 to 0·974)	0·562 (0·345 to 0·784)	-55·3 (-72·3 to -38·7)
Sri Lanka	All Ages	1070 (907 to 1280)	909 (620 to 1300)	802 (526 to 1140)	777 (401 to 1250)	711 (468 to 1060)	536 (233 to 871)	-49·8 (-77·1 to -19·7)
	15-49 years	191 (163 to 232)	169 (117 to 237)	146 (98·6 to 213)	140 (67·1 to 233)	162 (113 to 227)	118 (53·8 to 187)	-37·9 (-71·1 to 3·43)
	50-69 years	457 (366 to 548)	354 (222 to 536)	302 (173 to 463)	292 (124 to 491)	272 (161 to 423)	191 (62·4 to 341)	-58·2 (-85·5 to -27·9)
	70+ years	409 (334 to 505)	380 (267 to 513)	347 (241 to 472)	337 (202 to 522)	271 (176 to 392)	220 (105 to 344)	-46·2 (-72·6 to -18·2)
Thailand	All Ages	12700 (10800 to 14900)	12700 (10600 to 15000)	11200 (9010 to 14000)	11300 (7520 to 15400)	10700 (8810 to 12800)	8670 (6010 to 12000)	-31·4 (-53·7 to -6·28)
	15-49 years	4160 (3030 to 5590)	3180 (2210 to 4180)	3020 (2090 to 3980)	2990 (2040 to 4060)	3160 (2190 to 4150)	2860 (1930 to 3840)	-31·2 (-41·6 to -20·2)
	50-69 years	3480 (2880 to 4130)	3880 (2970 to 5020)	3290 (2550 to 4250)	3380 (2010 to 4780)	3420 (2590 to 4440)	2630 (1700 to 3750)	-24·2 (-54·9 to 8·25)
	70+ years	4960 (4060 to 5800)	5610 (4310 to 6960)	4860 (3590 to 6160)	4910 (3000 to 7160)	4070 (3190 to 5240)	3130 (1870 to 4650)	-36·4 (-64·6 to -1·09)
Timor-Leste	All Ages	608 (414 to 954)	649 (446 to 976)	538 (369 to 744)	570 (328 to 782)	565 (385 to 831)	423 (253 to 624)	-29·5 (-53·7 to -4·16)
	15-49 years	135 (91·9 to 170)	144 (98·6 to 197)	123 (84·7 to 165)	125 (78·1 to 177)	142 (97·2 to 194)	108 (69·0 to 153)	-19·3 (-48·1 to 7·65)
	50-69 years	226 (153 to 364)	227 (151 to 342)	173 (111 to 250)	191 (92·3 to 296)	192 (128 to 287)	126 (63·2 to 210)	-43·0 (-70·7 to -15·1)
	70+ years	196 (129 to 348)	232 (157 to 407)	196 (132 to 308)	207 (115 to 312)	184 (122 to 325)	142 (80·0 to 221)	-26·2 (-51·7 to 2·50)
Viet Nam	All Ages	25000 (21400 to 29500)	21100 (17100 to 28100)	17000 (13700 to 21900)	17500 (9710 to 26400)	18200 (14700 to 23800)	12600 (7930 to 19200)	-49·6 (-67·9 to -24·1)
	15-49 years	7480 (5950 to 8990)	5090 (4130 to 6340)	4340 (3540 to 5450)	4370 (2800 to 6610)	5040 (4090 to 6250)	3830 (2690 to 5430)	-48·6 (-64·4 to -28·0)
	50-69 years	6580 (5370 to 8280)	6710 (4760 to 9610)	4770 (3190 to 7370)	5280 (2340 to 9250)	5850 (4190 to 8320)	3430 (1660 to 5790)	-48·0 (-72·7 to -12·1)
	70+ years	10600 (8270 to 13000)	9110 (6900 to 11700)	7690 (5740 to 10000)	7620 (4170 to 11500)	7070 (5420 to 9090)	5130 (3020 to 7940)	-51·4 (-71·3 to -30·6)
Sub-Saharan Africa	All Ages	646000 (557000 to 727000)	533000 (456000 to 609000)	504000 (430000 to 581000)	481000 (343000 to 613000)	500000 (422000 to 573000)	432000 (312000 to 543000)	-33·2 (-50·9 to -19·4)
	15-49 years	295000 (255000 to 341000)	240000 (204000 to 280000)	229000 (193000 to 264000)	219000 (160000 to 278000)	238000 (202000 to 277000)	208000 (155000 to 258000)	-29·3 (-46·2 to -16·0)
	50-69 years	166000 (144000 to 187000)	154000 (130000 to 178000)	140000 (117000 to 162000)	133000 (81900 to 177000)	140000 (118000 to 163000)	113000 (69700 to 147000)	-32·1 (-58·7 to -13·4)
	70+ years	89000 (77800 to 97200)	81400 (70200 to 91700)	77200 (66300 to 87800)	70900 (41000 to 90700)	64100 (55500 to 75900)	53200 (29400 to 68500)	-40·3 (-67·3 to -22·1)
Central Sub-Saharan Africa	All Ages	96900 (75600 to 123000)	78600 (57000 to 105000)	73500 (53300 to 99500)	68500 (37800 to 91600)	71400 (51300 to 95500)	59000 (33600 to 77500)	-38·9 (-63·0 to -23·1)

eTable 5. Risk deleted deaths due to all-form tuberculosis for alcohol use, smoking, and diabetes, and all three risk factors combined by age groups for 204 countries and territories in (2021) with percent change between 2015 observed deaths and 2021 all-risk deleted deaths.

Location	Age group	2015 Observed deaths	2021 Observed deaths	2021 Smoking deleted deaths	2021 Alcohol deleted deaths	2021 Diabetes deleted deaths	2021 All-risk deleted deaths	Percent Change
	15-49 years	38800 (30100 to 49700)	31700 (22700 to 42300)	29500 (21100 to 39600)	27300 (15100 to 38400)	30800 (22000 to 41100)	24900 (14100 to 34200)	-35·4 (-61·7 to -18·9)
	50-69 years	28300 (20300 to 36500)	27300 (18300 to 37400)	24800 (16500 to 35100)	23100 (10600 to 32500)	23600 (15500 to 32800)	18300 (8380 to 24900)	-34·8 (-67·6 to -12·2)
	70+ years	11100 (7590 to 14600)	10100 (7130 to 13900)	9690 (6950 to 13300)	8720 (4260 to 11700)	7580 (5380 to 10300)	6320 (3060 to 8800)	-42·3 (-73·6 to -23·6)
Angola	All Ages	16600 (11700 to 21300)	15600 (11700 to 20400)	14100 (10500 to 18800)	13400 (8120 to 19900)	14400 (10800 to 18900)	11500 (7260 to 16900)	-30·0 (-55·6 to -7·01)
	15-49 years	6700 (4660 to 9320)	6890 (4920 to 9520)	6310 (4440 to 8720)	5880 (3380 to 9140)	6780 (4820 to 9400)	5400 (3210 to 8380)	-18·8 (-51·2 to 9·75)
	50-69 years	4250 (2970 to 5800)	4570 (3300 to 5980)	3830 (2840 to 5230)	3660 (1490 to 5630)	3910 (2780 to 5180)	2690 (1110 to 4230)	-35·8 (-72·6 to -0·592)
	70+ years	1650 (1060 to 2290)	1700 (1190 to 2270)	1560 (1110 to 2060)	1430 (672 to 2100)	1300 (888 to 1800)	1020 (484 to 1480)	-37·8 (-70·8 to -12·2)
Central African Republic	All Ages	11100 (8570 to 13500)	9500 (6730 to 12700)	8880 (6430 to 11700)	8620 (5270 to 12300)	8770 (6300 to 11800)	7530 (4710 to 10300)	-31·9 (-54·5 to -17·1)
	15-49 years	5040 (3880 to 6310)	4180 (3110 to 5660)	3900 (2900 to 5130)	3760 (2350 to 5370)	4060 (3040 to 5520)	3450 (2210 to 4930)	-31·4 (-54·3 to -14·1)
	50-69 years	3220 (2390 to 4270)	3210 (2100 to 4280)	2910 (1950 to 3890)	2830 (1400 to 4170)	2800 (1810 to 3760)	2260 (1110 to 3260)	-29·8 (-61·2 to -7·98)
	70+ years	831 (553 to 1100)	781 (513 to 1020)	744 (495 to 957)	696 (380 to 996)	581 (387 to 771)	494 (258 to 729)	-40·3 (-65·4 to -21·4)
Congo	All Ages	3180 (2250 to 4480)	2800 (2000 to 4180)	2600 (1910 to 3730)	2390 (1250 to 4100)	2570 (1830 to 3890)	2080 (1120 to 3460)	-34·5 (-60·5 to -11·4)
	15-49 years	1620 (1070 to 2430)	1380 (961 to 2040)	1290 (911 to 1950)	1190 (632 to 2100)	1350 (945 to 2010)	1110 (615 to 1870)	-31·4 (-56·0 to -8·86)
	50-69 years	856 (584 to 1230)	914 (630 to 1400)	827 (574 to 1240)	756 (350 to 1330)	799 (553 to 1230)	612 (292 to 1030)	-28·5 (-62·6 to 3·98)
	70+ years	348 (256 to 469)	318 (230 to 425)	293 (208 to 390)	254 (85·5 to 429)	232 (164 to 315)	172 (54·0 to 283)	-50·5 (-82·8 to -21·0)
Democratic Republic of the Congo	All Ages	64900 (48800 to 89800)	49600 (33100 to 73700)	46900 (31700 to 70600)	43100 (22600 to 61200)	44700 (29300 to 67000)	37000 (19800 to 53400)	-42·7 (-70·0 to -26·9)
	15-49 years	24900 (19000 to 34500)	18800 (12900 to 28000)	17500 (11900 to 26000)	16000 (8250 to 22200)	18100 (12400 to 27200)	14500 (7500 to 20500)	-41·2 (-71·6 to -24·2)
	50-69 years	19700 (13500 to 27000)	18300 (11500 to 27800)	17000 (10700 to 26300)	15500 (7340 to 22600)	15800 (9980 to 24300)	12500 (6060 to 18200)	-35·7 (-71·1 to -13·8)
	70+ years	8080 (5320 to 11300)	7170 (4690 to 10500)	6970 (4610 to 10200)	6240 (3140 to 8870)	5370 (3580 to 7990)	4560 (2230 to 6670)	-42·7 (-76·7 to -25·9)
Equatorial Guinea	All Ages	324 (203 to 527)	332 (205 to 511)	316 (195 to 497)	294 (159 to 510)	306 (189 to 474)	263 (146 to 456)	-18·8 (-44·1 to 5·03)
	15-49 years	171 (100 to 290)	182 (106 to 283)	174 (101 to 276)	163 (85·7 to 280)	177 (103 to 275)	154 (82·6 to 263)	-9·80 (-35·8 to 17·4)
	50-69 years	63·1 (42·0 to 102)	74·9 (49·4 to 111)	68·4 (46·1 to 107)	61·7 (26·8 to 113)	63·4 (40·3 to 97·0)	48·8 (23·2 to 89·0)	-22·6 (-60·4 to 9·91)

eTable 5. Risk deleted deaths due to all-form tuberculosis for alcohol use, smoking, and diabetes, and all three risk factors combined by age groups for 204 countries and territories in (2021) with percent change between 2015 observed deaths and 2021 all-risk deleted deaths.

Location	Age group	2015 Observed deaths	2021 Observed deaths	2021 Smoking deleted deaths	2021 Alcohol deleted deaths	2021 Diabetes deleted deaths	2021 All-risk deleted deaths	Percent Change
	70+ years	35·8 (25·3 to 52·9)	34·0 (22·6 to 49·2)	32·3 (21·2 to 46·9)	27·1 (7·67 to 50·4)	24·2 (16·4 to 36·3)	18·4 (4·21 to 34·9)	-48·5 (-86·8 to -19·7)
Gabon	All Ages	848 (543 to 1160)	714 (466 to 1050)	669 (442 to 956)	615 (306 to 1050)	641 (415 to 949)	531 (279 to 847)	-37·5 (-59·8 to -14·3)
	15-49 years	403 (247 to 580)	323 (208 to 480)	305 (198 to 432)	288 (157 to 476)	315 (202 to 469)	269 (154 to 418)	-33·2 (-52·1 to -11·3)
	50-69 years	253 (152 to 362)	260 (157 to 374)	238 (146 to 343)	215 (98·6 to 381)	221 (135 to 318)	172 (83·6 to 292)	-31·8 (-62·4 to -0·575)
	70+ years	113 (70·1 to 150)	95·4 (61·8 to 135)	91·1 (57·8 to 128)	76·6 (25·1 to 139)	69·8 (44·3 to 97·9)	54·0 (17·1 to 93·6)	-52·0 (-82·7 to -22·0)
Eastern Sub-Saharan Africa	All Ages	269000 (224000 to 305000)	226000 (188000 to 264000)	213000 (177000 to 250000)	206000 (149000 to 265000)	215000 (179000 to 252000)	186000 (135000 to 239000)	-30·7 (-49·5 to -16·0)
	15-49 years	126000 (104000 to 147000)	105000 (83600 to 124000)	99700 (80300 to 116000)	96300 (72600 to 122000)	104000 (83100 to 123000)	91700 (69200 to 115000)	-27·1 (-45·0 to -13·4)
	50-69 years	67800 (55900 to 77200)	63200 (50400 to 75000)	57600 (45100 to 68800)	55500 (35900 to 75100)	59300 (47800 to 71600)	48000 (31200 to 62800)	-29·1 (-54·2 to -7·36)
	70+ years	38900 (32100 to 44000)	36900 (30100 to 43500)	34700 (28100 to 40800)	32500 (20000 to 42400)	29900 (25100 to 36500)	24800 (14700 to 33300)	-36·2 (-61·9 to -17·2)
Burundi	All Ages	8640 (7200 to 10700)	7760 (5740 to 9920)	7320 (5460 to 9380)	6510 (3390 to 9090)	7270 (5460 to 9440)	5820 (3040 to 8140)	-32·3 (-66·6 to -6·23)
	15-49 years	3450 (2870 to 4400)	3020 (2270 to 3900)	2850 (2150 to 3620)	2480 (1200 to 3510)	2990 (2250 to 3870)	2330 (1140 to 3290)	-31·8 (-69·2 to -2·56)
	50-69 years	2480 (1930 to 3050)	2570 (1760 to 3470)	2350 (1640 to 3250)	2070 (858 to 3240)	2370 (1650 to 3230)	1770 (716 to 2740)	-28·1 (-73·3 to 4·93)
	70+ years	1370 (1050 to 1720)	1480 (975 to 2020)	1430 (921 to 1940)	1270 (661 to 1900)	1220 (814 to 1690)	1020 (510 to 1540)	-24·8 (-62·6 to -2·54)
Comoros	All Ages	327 (238 to 420)	309 (222 to 427)	282 (196 to 394)	299 (202 to 416)	274 (195 to 378)	243 (163 to 350)	-25·9 (-44·8 to -10·8)
	15-49 years	96·9 (68·7 to 132)	85·6 (58·7 to 128)	77·7 (50·5 to 115)	82·0 (48·2 to 128)	84·2 (57·8 to 125)	73·4 (42·9 to 113)	-24·2 (-49·2 to -5·47)
	50-69 years	106 (74·4 to 143)	107 (75·2 to 154)	97·4 (65·3 to 143)	104 (65·7 to 152)	96·5 (67·0 to 140)	85·2 (53·8 to 129)	-19·1 (-38·5 to 0·962)
	70+ years	102 (71·7 to 130)	102 (71·3 to 141)	93·4 (62·6 to 131)	99·4 (66·8 to 138)	79·2 (54·5 to 114)	70·1 (45·6 to 104)	-31·4 (-50·5 to -12·0)
Djibouti	All Ages	567 (307 to 778)	507 (277 to 737)	446 (249 to 644)	503 (268 to 728)	473 (258 to 680)	414 (226 to 595)	-26·9 (-41·6 to -9·51)
	15-49 years	258 (134 to 376)	208 (115 to 310)	186 (103 to 281)	206 (110 to 303)	206 (114 to 308)	183 (98·7 to 274)	-29·1 (-42·2 to -14·5)
	50-69 years	179 (88·3 to 265)	178 (94·6 to 263)	150 (84·5 to 221)	177 (93·3 to 259)	165 (86·8 to 241)	139 (76·3 to 202)	-21·9 (-41·4 to 4·38)
	70+ years	80·9 (41·9 to 111)	89·0 (49·5 to 121)	77·8 (44·4 to 107)	88·5 (49·1 to 119)	70·1 (39·0 to 96·4)	61·0 (33·1 to 86·6)	-24·3 (-44·9 to 1·64)
Eritrea	All Ages	5160 (3370 to 7310)	4830 (3010 to 7520)	4500 (2850 to 6870)	4530 (2690 to 6660)	4550 (2820 to 7060)	3980 (2400 to 5880)	-22·7 (-38·5 to -3·69)

eTable 5. Risk deleted deaths due to all-form tuberculosis for alcohol use, smoking, and diabetes, and all three risk factors combined by age groups for 204 countries and territories in (2021) with percent change between 2015 observed deaths and 2021 all-risk deleted deaths.

Location	Age group	2015 Observed deaths	2021 Observed deaths	2021 Smoking deleted deaths	2021 Alcohol deleted deaths	2021 Diabetes deleted deaths	2021 All-risk deleted deaths	Percent Change
	15-49 years	2240 (1390 to 3350)	2020 (1190 to 3430)	1840 (1080 to 3120)	1860 (963 to 2820)	1990 (1170 to 3370)	1680 (891 to 2500)	-25·0 (-46·2 to -2·70)
	50-69 years	1670 (1100 to 2370)	1700 (1080 to 2480)	1560 (1010 to 2260)	1580 (960 to 2330)	1570 (992 to 2270)	1360 (817 to 1990)	-18·5 (-38·0 to 3·94)
	70+ years	768 (474 to 1130)	755 (460 to 1120)	737 (453 to 1100)	728 (425 to 1050)	618 (390 to 909)	583 (346 to 859)	-23·8 (-37·1 to -5·31)
Ethiopia	All Ages	40000 (35800 to 44000)	34000 (28900 to 40300)	32700 (28000 to 39100)	30000 (19100 to 40000)	31800 (26700 to 38200)	27100 (17200 to 36300)	-32·0 (-58·7 to -7·85)
	15-49 years	14600 (12900 to 16600)	12100 (9890 to 14400)	11600 (9530 to 13900)	10500 (6400 to 14000)	12000 (9780 to 14300)	10100 (6170 to 13400)	-30·5 (-57·9 to -5·78)
	50-69 years	10500 (9080 to 11800)	9590 (7940 to 11800)	9090 (7500 to 11300)	8240 (4530 to 11400)	9110 (7520 to 11400)	7440 (4030 to 10400)	-28·6 (-62·8 to 2·68)
	70+ years	10300 (9070 to 11600)	9340 (7950 to 11200)	9050 (7460 to 10900)	8260 (5020 to 11100)	7720 (6330 to 9340)	6620 (3840 to 8810)	-35·6 (-64·6 to -12·9)
Kenya	All Ages	43100 (27500 to 52700)	33100 (20000 to 43700)	30500 (18500 to 39500)	28100 (16100 to 41100)	31100 (18900 to 40900)	24700 (14200 to 35500)	-42·4 (-66·3 to -19·8)
	15-49 years	21000 (13300 to 25800)	14400 (8760 to 19000)	13500 (8200 to 17700)	12600 (7650 to 17600)	14300 (8680 to 18900)	11900 (7330 to 16400)	-43·3 (-62·4 to -24·5)
	50-69 years	12400 (7810 to 15100)	10800 (6580 to 13500)	9580 (6060 to 12000)	8760 (3980 to 13100)	10100 (6240 to 12700)	7380 (3500 to 11100)	-39·8 (-72·2 to -7·73)
	70+ years	6270 (3780 to 8590)	6180 (3750 to 8730)	5690 (3440 to 8050)	5000 (2280 to 8100)	4930 (2910 to 7260)	3690 (1480 to 6050)	-40·7 (-76·9 to -8·58)
Madagascar	All Ages	11300 (8470 to 14800)	10600 (7660 to 14600)	10000 (6970 to 13800)	9680 (5850 to 13900)	10000 (7280 to 13800)	8640 (5170 to 12500)	-23·3 (-45·9 to -6·08)
	15-49 years	4350 (3230 to 5700)	4220 (3060 to 5880)	3940 (2720 to 5480)	3760 (2060 to 5470)	4170 (3010 to 5810)	3490 (1910 to 5100)	-19·5 (-47·0 to 2·01)
	50-69 years	3590 (2550 to 4950)	3720 (2600 to 5350)	3480 (2410 to 4960)	3350 (1910 to 4820)	3460 (2450 to 4960)	2920 (1690 to 4320)	-18·2 (-45·7 to 4·99)
	70+ years	1750 (1300 to 2420)	1740 (1280 to 2430)	1670 (1180 to 2310)	1620 (1040 to 2380)	1420 (1000 to 1950)	1270 (763 to 1820)	-27·1 (-48·2 to -3·93)
Malawi	All Ages	15200 (11700 to 18400)	14300 (10600 to 18000)	13500 (9930 to 16600)	13400 (9560 to 17800)	13900 (10400 to 17500)	12500 (9160 to 16000)	-17·9 (-33·6 to -2·92)
	15-49 years	7590 (5980 to 9590)	7690 (5810 to 9720)	7380 (5640 to 9300)	7250 (5410 to 9460)	7660 (5790 to 9680)	7000 (5320 to 9090)	-7·36 (-26·4 to 11·7)
	50-69 years	3340 (2370 to 4370)	3840 (2770 to 5180)	3500 (2490 to 4700)	3530 (2270 to 5180)	3710 (2710 to 4970)	3170 (2130 to 4490)	-5·20 (-28·5 to 19·1)
	70+ years	1610 (1100 to 2120)	1660 (1180 to 2310)	1480 (1030 to 2000)	1500 (829 to 2210)	1390 (1010 to 1950)	1150 (633 to 1650)	-28·5 (-52·7 to -6·53)
Mozambique	All Ages	44900 (38700 to 51400)	35800 (28400 to 42500)	34100 (27000 to 41000)	33800 (26100 to 40600)	34400 (27500 to 40800)	31300 (24500 to 38600)	-30·3 (-42·9 to -17·9)
	15-49 years	24500 (21600 to 27600)	20700 (16500 to 24100)	20000 (16000 to 23500)	19700 (15600 to 23300)	20600 (16400 to 23900)	19000 (15100 to 22700)	-22·2 (-35·3 to -10·4)
	50-69 years	9690 (7530 to 12000)	7300 (5410 to 9420)	6570 (4880 to 8680)	6570 (4150 to 8500)	6780 (5110 to 8840)	5540 (3470 to 7660)	-42·7 (-62·7 to -25·9)

eTable 5. Risk deleted deaths due to all-form tuberculosis for alcohol use, smoking, and diabetes, and all three risk factors combined by age groups for 204 countries and territories in (2021) with percent change between 2015 observed deaths and 2021 all-risk deleted deaths.

Location	Age group	2015 Observed deaths	2021 Observed deaths	2021 Smoking deleted deaths	2021 Alcohol deleted deaths	2021 Diabetes deleted deaths	2021 All-risk deleted deaths	Percent Change
	70+ years	4130 (3140 to 5100)	3540 (2440 to 4350)	3290 (2210 to 4000)	3270 (2010 to 4320)	2820 (1920 to 3610)	2430 (1490 to 3290)	-41·1 (-63·3 to -23·7)
Rwanda	All Ages	5510 (4290 to 6920)	5130 (3850 to 6920)	4560 (3250 to 5880)	4400 (2370 to 6580)	4880 (3660 to 6620)	3800 (2130 to 5650)	-31·1 (-57·3 to -7·12)
	15-49 years	2500 (1970 to 3180)	2220 (1690 to 3020)	2080 (1590 to 2730)	1920 (1180 to 2790)	2210 (1680 to 3010)	1820 (1150 to 2660)	-27·2 (-51·5 to -2·97)
	50-69 years	1620 (1220 to 2130)	1720 (1210 to 2480)	1440 (915 to 1990)	1430 (628 to 2320)	1630 (1150 to 2370)	1170 (554 to 1920)	-27·8 (-62·2 to 6·21)
	70+ years	868 (646 to 1110)	938 (670 to 1310)	777 (484 to 1080)	799 (366 to 1240)	781 (546 to 1070)	554 (236 to 882)	-35·9 (-69·3 to -1·80)
Somalia	All Ages	18200 (11600 to 25800)	18800 (11700 to 26400)	17300 (11000 to 24600)	18800 (11700 to 26400)	17600 (11000 to 24600)	16200 (10300 to 22900)	-10·7 (-23·7 to 1·32)
	15-49 years	6880 (4090 to 10000)	8050 (4610 to 11600)	7310 (4350 to 10500)	8050 (4610 to 11600)	7930 (4550 to 11400)	7200 (4290 to 10300)	5·22 (-14·4 to 26·0)
	50-69 years	5940 (3700 to 8550)	5720 (3630 to 8280)	5120 (3370 to 7310)	5720 (3630 to 8280)	5210 (3350 to 7600)	4670 (3090 to 6650)	-21·0 (-35·0 to -5·49)
	70+ years	2040 (1120 to 3110)	2390 (1340 to 3800)	2230 (1280 to 3380)	2390 (1340 to 3800)	1790 (999 to 2810)	1660 (955 to 2540)	-18·4 (-34·8 to -1·38)
South Sudan	All Ages	6840 (5040 to 9270)	6400 (4360 to 8980)	5960 (4130 to 8490)	6350 (4290 to 8980)	6040 (4140 to 8460)	5600 (3880 to 8020)	-17·9 (-30·6 to -3·04)
	15-49 years	2700 (1870 to 3750)	2360 (1600 to 3380)	2210 (1530 to 3210)	2330 (1600 to 3380)	2340 (1590 to 3340)	2170 (1520 to 3180)	-18·9 (-33·8 to -2·58)
	50-69 years	1640 (1120 to 2330)	1980 (1280 to 2840)	1770 (1190 to 2590)	1960 (1280 to 2840)	1850 (1210 to 2630)	1630 (1100 to 2410)	-0·0854 (-18·4 to 22·8)
	70+ years	987 (718 to 1410)	942 (655 to 1420)	868 (613 to 1270)	937 (645 to 1420)	738 (511 to 1150)	676 (470 to 1020)	-31·4 (-49·9 to -8·67)
Uganda	All Ages	26500 (22300 to 30900)	22100 (18200 to 27700)	21400 (17400 to 26400)	19800 (13700 to 27000)	21200 (17500 to 26500)	18500 (13200 to 24600)	-30·2 (-52·1 to -12·7)
	15-49 years	14100 (11600 to 17000)	11700 (9330 to 15300)	11400 (9030 to 14800)	10600 (7880 to 13800)	11600 (9280 to 15200)	10300 (7770 to 13200)	-26·4 (-48·2 to -6·31)
	50-69 years	5600 (4210 to 6810)	5400 (3820 to 7150)	5060 (3550 to 6770)	4560 (2440 to 7160)	5060 (3570 to 6670)	4070 (2260 to 6270)	-27·2 (-62·6 to 2·19)
	70+ years	3130 (2520 to 3940)	2830 (2130 to 3700)	2710 (1990 to 3530)	2400 (1170 to 3680)	2260 (1710 to 3050)	1840 (855 to 2870)	-41·1 (-73·2 to -14·8)
United Republic of Tanzania	All Ages	30600 (20900 to 39300)	23700 (16700 to 32300)	22200 (15400 to 29500)	21300 (13200 to 30700)	22700 (16100 to 31100)	19300 (12100 to 27500)	-36·9 (-53·1 to -20·9)
	15-49 years	14700 (9320 to 20000)	10800 (7210 to 15800)	10200 (6790 to 14500)	9940 (5770 to 14200)	10800 (7180 to 15700)	9460 (5640 to 13400)	-35·7 (-48·8 to -20·2)
	50-69 years	6950 (4600 to 9200)	6490 (4450 to 9230)	5830 (3810 to 8010)	5570 (3130 to 8600)	6200 (4220 to 8920)	4870 (2850 to 7510)	-29·9 (-54·2 to -3·79)
	70+ years	4480 (3170 to 5760)	4070 (2830 to 5380)	3800 (2630 to 5230)	3410 (1350 to 5180)	3390 (2420 to 4560)	2670 (1060 to 4240)	-40·4 (-71·9 to -10·7)
Zambia	All Ages	11800 (7990 to 16000)	8680 (5580 to 12900)	8470 (5420 to 12700)	8250 (4960 to 12400)	8360 (5350 to 12500)	7840 (4750 to 11700)	-34·3 (-48·1 to -20·2)

eTable 5. Risk deleted deaths due to all-form tuberculosis for alcohol use, smoking, and diabetes, and all three risk factors combined by age groups for 204 countries and territories in (2021) with percent change between 2015 observed deaths and 2021 all-risk deleted deaths.

Location	Age group	2015 Observed deaths	2021 Observed deaths	2021 Smoking deleted deaths	2021 Alcohol deleted deaths	2021 Diabetes deleted deaths	2021 All-risk deleted deaths	Percent Change
	15-49 years	6840 (4460 to 9500)	5130 (3060 to 7930)	5070 (3030 to 7820)	4950 (2900 to 7630)	5110 (3050 to 7910)	4880 (2870 to 7500)	-29·2 (-43·1 to -13·1)
	50-69 years	2070 (1370 to 2890)	2000 (1210 to 2900)	1910 (1130 to 2750)	1860 (1000 to 2910)	1900 (1160 to 2770)	1720 (942 to 2610)	-17·3 (-37·1 to 5·05)
	70+ years	1020 (660 to 1370)	837 (523 to 1200)	780 (479 to 1110)	731 (301 to 1190)	639 (401 to 906)	523 (201 to 872)	-49·0 (-76·0 to -27·1)
Southern Sub-Saharan Africa	All Ages	117000 (107000 to 125000)	95600 (84500 to 105000)	89700 (78100 to 98300)	87900 (68200 to 106000)	91100 (80900 to 99400)	80500 (65600 to 94900)	-31·3 (-43·2 to -20·7)
	15-49 years	71100 (63400 to 77800)	57000 (48700 to 62400)	54700 (46300 to 59500)	53700 (44100 to 62400)	56700 (48400 to 62000)	51800 (42600 to 59300)	-27·1 (-37·2 to -17·6)
	50-69 years	29900 (27200 to 32400)	26900 (24300 to 30300)	24000 (20700 to 27800)	23600 (15700 to 30800)	24400 (22000 to 27600)	19900 (14200 to 25500)	-33·5 (-51·7 to -18·8)
	70+ years	8410 (7590 to 8960)	7530 (6750 to 8540)	6840 (5890 to 7900)	6410 (3490 to 8660)	5810 (5070 to 6850)	4540 (2350 to 6220)	-46·0 (-71·7 to -29·1)
Botswana	All Ages	3220 (2340 to 3870)	2660 (1850 to 3360)	2520 (1760 to 3170)	2530 (1660 to 3250)	2580 (1790 to 3250)	2360 (1590 to 3010)	-26·9 (-39·7 to -14·6)
	15-49 years	2110 (1550 to 2650)	1590 (1080 to 2030)	1530 (1050 to 1930)	1520 (994 to 1940)	1580 (1080 to 2020)	1480 (956 to 1870)	-30·3 (-42·5 to -16·6)
	50-69 years	811 (573 to 1060)	833 (565 to 1110)	769 (514 to 1020)	780 (500 to 1120)	784 (529 to 1050)	696 (457 to 990)	-14·3 (-33·5 to 6·41)
	70+ years	181 (134 to 231)	172 (125 to 217)	154 (111 to 196)	158 (91·7 to 211)	141 (100 to 185)	120 (73·8 to 162)	-33·5 (-50·0 to -15·0)
Eswatini	All Ages	2960 (2540 to 3490)	1960 (1610 to 2330)	1920 (1560 to 2230)	1850 (1490 to 2300)	1890 (1560 to 2240)	1760 (1430 to 2170)	-40·2 (-52·8 to -30·1)
	15-49 years	1980 (1700 to 2480)	1310 (1080 to 1570)	1290 (1060 to 1530)	1250 (978 to 1560)	1300 (1080 to 1560)	1230 (969 to 1530)	-37·5 (-50·5 to -24·5)
	50-69 years	592 (459 to 756)	452 (310 to 633)	431 (290 to 605)	411 (240 to 619)	411 (265 to 579)	364 (218 to 557)	-38·7 (-56·8 to -17·6)
	70+ years	112 (83·7 to 142)	93·1 (66·7 to 123)	87·6 (61·4 to 115)	82·0 (41·3 to 121)	69·9 (48·0 to 93·6)	58·4 (29·6 to 87·3)	-48·0 (-72·2 to -29·4)
Lesotho	All Ages	7980 (6910 to 9250)	6010 (5140 to 6880)	5370 (4570 to 6120)	5450 (4240 to 6560)	5750 (4940 to 6560)	4800 (3930 to 5800)	-39·6 (-54·0 to -27·4)
	15-49 years	4630 (3880 to 5630)	3370 (2870 to 3970)	3080 (2710 to 3630)	3090 (2510 to 3740)	3350 (2860 to 3950)	2880 (2420 to 3450)	-37·4 (-51·0 to -24·1)
	50-69 years	2390 (1890 to 2800)	1760 (1330 to 2290)	1470 (1070 to 2020)	1520 (883 to 2220)	1590 (1200 to 2110)	1200 (734 to 1790)	-49·8 (-71·1 to -28·4)
	70+ years	466 (345 to 580)	401 (311 to 502)	336 (242 to 444)	352 (197 to 480)	319 (248 to 395)	238 (132 to 357)	-48·6 (-70·8 to -29·5)
Namibia	All Ages	2730 (2060 to 3440)	2080 (1560 to 2660)	1960 (1490 to 2470)	1850 (1130 to 2600)	1970 (1480 to 2520)	1700 (1100 to 2340)	-37·6 (-55·9 to -24·9)
	15-49 years	1590 (1130 to 2110)	1120 (827 to 1440)	1080 (806 to 1360)	1020 (705 to 1400)	1110 (822 to 1430)	996 (700 to 1340)	-37·1 (-52·0 to -25·6)
	50-69 years	734 (557 to 921)	681 (511 to 881)	626 (452 to 835)	583 (312 to 859)	631 (471 to 827)	511 (291 to 721)	-30·3 (-56·6 to -10·1)

eTable 5. Risk deleted deaths due to all-form tuberculosis for alcohol use, smoking, and diabetes, and all three risk factors combined by age groups for 204 countries and territories in (2021) with percent change between 2015 observed deaths and 2021 all-risk deleted deaths.

Location	Age group	2015 Observed deaths	2021 Observed deaths	2021 Smoking deleted deaths	2021 Alcohol deleted deaths	2021 Diabetes deleted deaths	2021 All-risk deleted deaths	Percent Change
	70+ years	208 (165 to 283)	185 (136 to 255)	161 (112 to 221)	145 (44·2 to 254)	140 (103 to 192)	94·8 (24·6 to 166)	-54·5 (-87·5 to -28·3)
South Africa	All Ages	76900 (67800 to 81700)	62800 (53300 to 68200)	59700 (50200 to 65500)	58000 (45500 to 69800)	60000 (50700 to 65600)	53700 (44000 to 64000)	-30·2 (-41·9 to -18·7)
	15-49 years	48100 (40700 to 51600)	38800 (31500 to 42600)	37600 (30000 to 41300)	36800 (29400 to 43200)	38700 (31300 to 42400)	35900 (29000 to 41800)	-25·4 (-33·9 to -15·3)
	50-69 years	19400 (18000 to 20600)	17200 (15400 to 18700)	15600 (13900 to 17200)	15000 (10100 to 19400)	15600 (13800 to 17200)	12900 (9370 to 16400)	-33·5 (-51·7 to -17·8)
	70+ years	5510 (5130 to 5880)	4880 (4480 to 5530)	4570 (4040 to 5250)	4140 (2190 to 5610)	3760 (3350 to 4500)	3030 (1540 to 4130)	-45·0 (-71·6 to -27·0)
Zimbabwe	All Ages	23300 (18700 to 26500)	20000 (15500 to 24400)	18200 (14500 to 21500)	18300 (12900 to 23200)	18900 (14900 to 22900)	16100 (12200 to 19800)	-30·7 (-45·2 to -19·2)
	15-49 years	12700 (10800 to 14800)	10800 (8730 to 13000)	10100 (8310 to 11900)	9940 (7460 to 12200)	10700 (8670 to 12900)	9380 (7250 to 11400)	-26·3 (-40·2 to -14·1)
	50-69 years	6010 (4340 to 7670)	5960 (4060 to 7750)	5130 (3600 to 6720)	5310 (3210 to 7550)	5360 (3720 to 7150)	4270 (2740 to 6200)	-29·2 (-48·6 to -12·6)
	70+ years	1930 (1320 to 2370)	1800 (1200 to 2270)	1520 (910 to 1940)	1530 (743 to 2350)	1380 (924 to 1750)	1000 (478 to 1480)	-48·2 (-71·4 to -28·2)
Western Sub-Saharan Africa	All Ages	163000 (135000 to 195000)	132000 (102000 to 163000)	127000 (101000 to 160000)	119000 (75700 to 164000)	122000 (93000 to 154000)	106000 (68500 to 147000)	-35·3 (-55·9 to -19·9)
	15-49 years	59000 (46100 to 76200)	46800 (35400 to 61000)	44700 (34000 to 58400)	42100 (26500 to 63100)	46200 (34900 to 60400)	40000 (25200 to 58900)	-32·4 (-53·6 to -14·8)
	50-69 years	39800 (32800 to 48000)	36400 (28100 to 45100)	33800 (26200 to 42900)	31100 (15700 to 45400)	32900 (24700 to 41500)	26300 (13200 to 38100)	-34·2 (-65·4 to -8·81)
	70+ years	30600 (26000 to 34600)	26900 (22100 to 32300)	26000 (21400 to 31500)	23300 (12200 to 32600)	20900 (16600 to 26500)	17500 (8610 to 24300)	-43·2 (-71·1 to -23·7)
Benin	All Ages	2660 (1990 to 3560)	2200 (1560 to 3100)	2100 (1480 to 2910)	1970 (1050 to 3020)	2010 (1380 to 2890)	1740 (933 to 2710)	-34·9 (-58·2 to -14·2)
	15-49 years	946 (701 to 1260)	809 (578 to 1120)	774 (540 to 1060)	715 (384 to 1100)	799 (569 to 1110)	679 (368 to 1040)	-28·3 (-55·2 to -0·911)
	50-69 years	607 (447 to 772)	593 (414 to 877)	548 (366 to 797)	509 (228 to 771)	529 (365 to 791)	421 (178 to 646)	-30·9 (-63·7 to -3·23)
	70+ years	537 (413 to 678)	467 (356 to 621)	452 (339 to 604)	422 (220 to 609)	356 (255 to 495)	310 (146 to 440)	-42·3 (-66·6 to -20·9)
Burkina Faso	All Ages	7820 (6290 to 9030)	6510 (4980 to 7970)	6170 (4680 to 7650)	5500 (2750 to 7940)	5980 (4440 to 7410)	4830 (2520 to 6980)	-38·2 (-66·8 to -15·0)
	15-49 years	2670 (2140 to 3220)	2090 (1520 to 2650)	1930 (1420 to 2380)	1710 (703 to 2490)	2060 (1500 to 2620)	1560 (658 to 2260)	-41·2 (-73·9 to -15·0)
	50-69 years	2010 (1600 to 2420)	1850 (1420 to 2330)	1710 (1290 to 2220)	1470 (470 to 2380)	1660 (1250 to 2120)	1220 (412 to 1950)	-39·2 (-79·3 to -7·73)
	70+ years	1520 (1320 to 1790)	1420 (1090 to 1800)	1390 (1070 to 1760)	1170 (479 to 1810)	1110 (851 to 1450)	902 (345 to 1390)	-40·7 (-76·6 to -14·1)
Cabo Verde	All Ages	65·4 (48·6 to 101)	64·5 (44·0 to 114)	61·2 (43·0 to 108)	54·7 (25·6 to 124)	53·2 (37·4 to 92·2)	43·0 (20·5 to 97·3)	-34·7 (-65·7 to -5·48)

eTable 5. Risk deleted deaths due to all-form tuberculosis for alcohol use, smoking, and diabetes, and all three risk factors combined by age groups for 204 countries and territories in (2021) with percent change between 2015 observed deaths and 2021 all-risk deleted deaths.

Location	Age group	2015 Observed deaths	2021 Observed deaths	2021 Smoking deleted deaths	2021 Alcohol deleted deaths	2021 Diabetes deleted deaths	2021 All-risk deleted deaths	Percent Change
	15-49 years	22·1 (14·4 to 36·5)	16·4 (10·5 to 28·7)	15·4 (10·2 to 26·7)	14·0 (7·07 to 30·9)	15·9 (10·3 to 27·6)	12·9 (6·72 to 28·3)	-41·8 (-66·4 to -18·2)
	50-69 years	16·4 (11·4 to 27·3)	22·4 (14·5 to 42·6)	20·5 (13·7 to 38·2)	18·1 (6·53 to 43·7)	18·9 (12·3 to 35·5)	14·2 (5·39 to 35·2)	-13·8 (-65·7 to 39·0)
	70+ years	25·4 (19·0 to 37·0)	24·8 (17·5 to 41·5)	24·3 (17·3 to 40·7)	21·7 (11·1 to 44·9)	17·5 (12·4 to 28·6)	15·0 (7·96 to 30·0)	-41·2 (-67·4 to -14·9)
Cameroon	All Ages	13000 (8100 to 19300)	8420 (5060 to 12900)	8070 (4800 to 12200)	7430 (3640 to 13700)	7920 (4790 to 12300)	6800 (3410 to 12300)	-48·1 (-65·8 to -27·5)
	15-49 years	6770 (4000 to 10600)	4070 (2440 to 6610)	3920 (2340 to 6300)	3660 (1840 to 6860)	4030 (2420 to 6550)	3520 (1790 to 6430)	-48·1 (-64·1 to -28·7)
	50-69 years	3080 (1800 to 4700)	2260 (1280 to 3590)	2100 (1150 to 3290)	1900 (785 to 3780)	2080 (1190 to 3240)	1650 (700 to 3080)	-46·8 (-70·9 to -22·5)
	70+ years	1440 (902 to 2060)	1170 (750 to 1730)	1140 (712 to 1660)	956 (272 to 1780)	899 (552 to 1330)	711 (191 to 1340)	-51·2 (-84·2 to -25·7)
Chad	All Ages	7860 (5950 to 9530)	7440 (5620 to 9670)	7060 (5380 to 9090)	6710 (4150 to 9590)	6880 (5170 to 8890)	5950 (3760 to 8390)	-24·1 (-51·8 to -7·39)
	15-49 years	2680 (2030 to 3480)	2560 (1880 to 3540)	2410 (1770 to 3340)	2260 (1320 to 3340)	2520 (1850 to 3490)	2120 (1250 to 3140)	-20·5 (-54·0 to 2·37)
	50-69 years	1890 (1310 to 2340)	1960 (1400 to 2600)	1790 (1300 to 2360)	1680 (789 to 2550)	1740 (1240 to 2330)	1380 (600 to 2090)	-26·7 (-66·4 to -0·359)
	70+ years	1320 (916 to 1710)	1240 (856 to 1590)	1170 (833 to 1490)	1090 (561 to 1620)	932 (622 to 1220)	771 (372 to 1180)	-41·4 (-73·0 to -21·3)
Côte d'Ivoire	All Ages	10100 (7250 to 13700)	6680 (4490 to 9510)	6210 (4290 to 8920)	5660 (2680 to 9990)	6170 (4140 to 8880)	4940 (2380 to 8510)	-51·4 (-72·3 to -31·6)
	15-49 years	4730 (3270 to 6730)	2910 (1950 to 4160)	2690 (1770 to 3860)	2480 (1130 to 4410)	2870 (1920 to 4110)	2290 (1090 to 3940)	-51·7 (-72·3 to -31·1)
	50-69 years	2520 (1720 to 3480)	1970 (1260 to 2930)	1760 (1160 to 2650)	1580 (600 to 3030)	1760 (1130 to 2650)	1290 (486 to 2370)	-49·2 (-77·2 to -24·8)
	70+ years	1270 (918 to 1610)	1050 (765 to 1520)	1000 (716 to 1450)	839 (255 to 1450)	789 (558 to 1170)	597 (148 to 1040)	-53·4 (-87·2 to -27·6)
Gambia	All Ages	751 (581 to 1110)	753 (526 to 1140)	706 (482 to 1100)	675 (365 to 1040)	689 (473 to 1050)	586 (322 to 943)	-22·3 (-48·8 to -2·15)
	15-49 years	319 (226 to 481)	333 (219 to 508)	313 (207 to 491)	304 (179 to 479)	329 (217 to 504)	286 (172 to 463)	-10·6 (-35·3 to 11·1)
	50-69 years	183 (132 to 274)	206 (136 to 329)	185 (120 to 302)	177 (81·3 to 280)	185 (121 to 299)	145 (66·3 to 246)	-20·8 (-58·7 to 8·86)
	70+ years	168 (115 to 239)	160 (106 to 246)	153 (102 to 234)	140 (69·2 to 228)	121 (78·5 to 192)	101 (47·6 to 166)	-40·4 (-67·4 to -21·4)
Ghana	All Ages	14700 (11800 to 18500)	12100 (9210 to 16100)	11500 (8560 to 15300)	10500 (6080 to 16300)	11100 (8440 to 14900)	9330 (5430 to 14100)	-36·9 (-58·1 to -17·5)
	15-49 years	6730 (5150 to 8520)	5320 (4030 to 7250)	5130 (3810 to 7020)	4780 (2660 to 7100)	5250 (3970 to 7180)	4600 (2560 to 6950)	-31·9 (-50·1 to -14·4)
	50-69 years	4120 (3100 to 5450)	3760 (2780 to 5310)	3490 (2430 to 4980)	3150 (1570 to 5120)	3370 (2510 to 4790)	2650 (1340 to 4240)	-35·8 (-64·4 to -8·50)

eTable 5. Risk deleted deaths due to all-form tuberculosis for alcohol use, smoking, and diabetes, and all three risk factors combined by age groups for 204 countries and territories in (2021) with percent change between 2015 observed deaths and 2021 all-risk deleted deaths.

Location	Age group	2015 Observed deaths	2021 Observed deaths	2021 Smoking deleted deaths	2021 Alcohol deleted deaths	2021 Diabetes deleted deaths	2021 All-risk deleted deaths	Percent Change
	70+ years	2640 (2080 to 3310)	2450 (1740 to 3350)	2320 (1650 to 3170)	2070 (1090 to 3280)	1900 (1350 to 2700)	1530 (777 to 2430)	-42·3 (-69·8 to -16·2)
Guinea	All Ages	5550 (4280 to 6720)	4260 (3130 to 5740)	4010 (3000 to 5420)	4020 (2880 to 5790)	3960 (2890 to 5370)	3550 (2600 to 5110)	-36·1 (-50·0 to -23·0)
	15-49 years	2060 (1540 to 2650)	1650 (1230 to 2120)	1570 (1170 to 2030)	1570 (1110 to 2130)	1640 (1220 to 2110)	1490 (1070 to 2020)	-27·6 (-43·7 to -12·1)
	50-69 years	1400 (1040 to 1720)	1190 (846 to 1600)	1080 (777 to 1390)	1090 (709 to 1570)	1090 (773 to 1480)	917 (606 to 1300)	-34·3 (-52·3 to -16·9)
	70+ years	1210 (918 to 1560)	909 (650 to 1270)	860 (621 to 1210)	844 (570 to 1190)	724 (497 to 1020)	638 (423 to 935)	-47·2 (-62·0 to -29·0)
Guinea-Bissau	All Ages	1410 (1060 to 1830)	1390 (1020 to 1810)	1340 (986 to 1770)	1270 (831 to 1710)	1310 (955 to 1730)	1170 (771 to 1600)	-16·8 (-38·5 to 2·27)
	15-49 years	697 (496 to 983)	728 (480 to 1040)	706 (466 to 1010)	673 (449 to 952)	721 (476 to 1030)	651 (431 to 941)	-6·28 (-28·7 to 13·9)
	50-69 years	359 (261 to 445)	353 (256 to 437)	332 (236 to 417)	309 (168 to 433)	318 (230 to 396)	265 (150 to 373)	-26·2 (-55·7 to -3·59)
	70+ years	159 (122 to 191)	142 (111 to 171)	138 (107 to 167)	123 (58·0 to 164)	103 (78·1 to 131)	86·9 (39·2 to 119)	-45·3 (-74·0 to -27·5)
Liberia	All Ages	1070 (716 to 1660)	907 (575 to 1580)	854 (552 to 1510)	764 (340 to 1620)	806 (510 to 1410)	642 (273 to 1340)	-40·7 (-69·6 to -16·8)
	15-49 years	433 (282 to 692)	384 (232 to 667)	359 (219 to 644)	315 (122 to 689)	373 (228 to 652)	290 (113 to 624)	-33·4 (-67·4 to 1·01)
	50-69 years	260 (174 to 389)	252 (157 to 432)	228 (147 to 395)	205 (66·5 to 467)	215 (133 to 368)	158 (50·2 to 354)	-39·8 (-78·7 to -8·50)
	70+ years	225 (153 to 361)	183 (115 to 340)	179 (114 to 333)	156 (67·9 to 313)	130 (83·7 to 243)	107 (39·6 to 227)	-52·6 (-81·4 to -33·9)
Mali	All Ages	6580 (5130 to 8690)	5750 (4280 to 8090)	5310 (3870 to 7450)	5410 (3670 to 7860)	5220 (3920 to 7420)	4580 (3110 to 6530)	-30·6 (-49·3 to -14·7)
	15-49 years	2140 (1620 to 2910)	1760 (1340 to 2480)	1650 (1200 to 2330)	1680 (1090 to 2410)	1720 (1310 to 2410)	1540 (997 to 2160)	-27·8 (-45·6 to -10·9)
	50-69 years	1820 (1340 to 2500)	1670 (1220 to 2380)	1480 (1020 to 2090)	1540 (933 to 2220)	1490 (1070 to 2170)	1230 (727 to 1710)	-32·3 (-56·4 to -8·59)
	70+ years	1600 (1150 to 2090)	1490 (1020 to 2100)	1350 (965 to 1850)	1360 (790 to 2000)	1180 (868 to 1680)	979 (526 to 1440)	-38·7 (-65·5 to -18·4)
Mauritania	All Ages	492 (348 to 759)	438 (293 to 689)	408 (271 to 647)	438 (293 to 689)	400 (265 to 639)	372 (245 to 599)	-24·6 (-36·8 to -7·56)
	15-49 years	113 (74·5 to 164)	102 (65·8 to 163)	89·3 (57·1 to 141)	102 (65·8 to 163)	101 (64·8 to 161)	88·0 (56·3 to 139)	-22·0 (-38·4 to -4·15)
	50-69 years	144 (96·3 to 230)	141 (87·5 to 226)	129 (83·0 to 204)	141 (87·5 to 226)	130 (79·3 to 211)	119 (75·3 to 190)	-17·9 (-35·2 to 5·78)
	70+ years	175 (128 to 280)	160 (105 to 257)	156 (103 to 252)	160 (105 to 257)	135 (89·3 to 221)	131 (86·2 to 216)	-25·3 (-38·7 to -3·54)
Niger	All Ages	7420 (5360 to 10600)	6890 (4750 to 10000)	6630 (4590 to 9610)	6790 (4630 to 9980)	6330 (4270 to 9200)	6010 (4090 to 8810)	-19·1 (-30·6 to -3·39)

eTable 5. Risk deleted deaths due to all-form tuberculosis for alcohol use, smoking, and diabetes, and all three risk factors combined by age groups for 204 countries and territories in (2021) with percent change between 2015 observed deaths and 2021 all-risk deleted deaths.

Location	Age group	2015 Observed deaths	2021 Observed deaths	2021 Smoking deleted deaths	2021 Alcohol deleted deaths	2021 Diabetes deleted deaths	2021 All-risk deleted deaths	Percent Change
	15-49 years	2000 (1460 to 2780)	1850 (1250 to 2640)	1730 (1190 to 2470)	1810 (1240 to 2630)	1810 (1220 to 2590)	1660 (1160 to 2410)	-16·8 (-32·0 to 2·91)
	50-69 years	1900 (1320 to 2720)	1930 (1220 to 2700)	1830 (1180 to 2570)	1890 (1210 to 2650)	1720 (1080 to 2420)	1590 (1020 to 2280)	-15·9 (-35·3 to 8·53)
	70+ years	1300 (882 to 1920)	1400 (911 to 1960)	1360 (888 to 1890)	1380 (898 to 1950)	1090 (703 to 1540)	1040 (668 to 1490)	-19·2 (-35·5 to -2·28)
Nigeria	All Ages	73200 (59500 to 88300)	60100 (42800 to 76400)	58500 (42400 to 74600)	53800 (31100 to 75300)	55800 (39100 to 71200)	49000 (27500 to 69300)	-33·2 (-59·3 to -12·5)
	15-49 years	22500 (17200 to 28400)	19100 (13200 to 25800)	18500 (13000 to 25100)	17100 (10300 to 25700)	18900 (13000 to 25600)	16500 (9950 to 25200)	-26·5 (-52·3 to -3·50)
	50-69 years	16700 (12800 to 20700)	15600 (11000 to 19900)	14800 (10700 to 19200)	13100 (5570 to 19600)	14300 (9780 to 18600)	11400 (4520 to 17100)	-31·2 (-73·4 to 5·44)
	70+ years	14900 (11600 to 17500)	12600 (9890 to 15100)	12400 (9740 to 15000)	10700 (4600 to 15700)	9900 (7550 to 12600)	8210 (3650 to 12300)	-44·9 (-76·7 to -20·9)
Sao Tome and Principe	All Ages	22·1 (16·7 to 32·8)	18·7 (13·2 to 30·8)	17·6 (12·3 to 29·2)	15·0 (5·68 to 29·4)	16·5 (11·8 to 27·3)	12·6 (4·66 to 25·0)	-43·4 (-77·6 to -10·8)
	15-49 years	6·21 (4·51 to 9·32)	5·21 (3·18 to 8·95)	4·86 (3·04 to 8·49)	4·14 (1·38 to 8·56)	5·10 (3·14 to 8·82)	3·80 (1·28 to 7·68)	-39·6 (-75·5 to 2·78)
	50-69 years	7·27 (5·22 to 11·0)	7·22 (5·06 to 12·3)	6·62 (4·52 to 11·3)	5·73 (1·89 to 12·4)	6·47 (4·49 to 11·0)	4·73 (1·54 to 10·5)	-35·2 (-77·6 to 8·37)
	70+ years	7·86 (5·49 to 11·6)	5·92 (3·97 to 9·82)	5·78 (3·89 to 9·62)	4·86 (2·04 to 9·49)	4·64 (3·19 to 7·45)	3·75 (1·47 to 7·47)	-52·4 (-81·2 to -30·5)
Senegal	All Ages	3760 (3040 to 4680)	3140 (2310 to 4040)	2930 (2130 to 3750)	3070 (2200 to 4010)	2680 (1940 to 3520)	2440 (1690 to 3230)	-35·4 (-50·6 to -24·0)
	15-49 years	1300 (1000 to 1690)	1020 (758 to 1450)	930 (677 to 1280)	992 (717 to 1430)	999 (740 to 1420)	888 (619 to 1230)	-31·9 (-46·3 to -16·3)
	50-69 years	1080 (819 to 1420)	989 (714 to 1310)	889 (629 to 1170)	959 (674 to 1280)	827 (598 to 1130)	722 (494 to 962)	-33·0 (-50·4 to -15·4)
	70+ years	998 (781 to 1260)	940 (654 to 1300)	916 (639 to 1260)	922 (639 to 1300)	661 (443 to 942)	633 (419 to 910)	-36·7 (-52·3 to -20·6)
Sierra Leone	All Ages	3530 (2810 to 4320)	2850 (2130 to 3690)	2660 (1960 to 3520)	2520 (1510 to 3460)	2620 (1910 to 3480)	2190 (1340 to 3020)	-38·0 (-62·5 to -21·1)
	15-49 years	1310 (1010 to 1700)	1040 (779 to 1420)	969 (697 to 1370)	917 (522 to 1340)	1030 (768 to 1400)	848 (499 to 1240)	-35·1 (-61·3 to -17·2)
	50-69 years	816 (580 to 1040)	746 (526 to 1020)	659 (457 to 892)	623 (263 to 986)	667 (464 to 930)	495 (201 to 765)	-39·3 (-73·4 to -13·6)
	70+ years	681 (531 to 862)	590 (441 to 775)	570 (426 to 753)	517 (269 to 719)	453 (336 to 623)	384 (196 to 525)	-43·4 (-71·6 to -22·9)
Togo	All Ages	3390 (2640 to 4610)	2560 (1880 to 3850)	2310 (1690 to 3540)	2290 (1270 to 3810)	2410 (1750 to 3650)	1970 (1130 to 3220)	-42·1 (-65·0 to -23·2)
	15-49 years	1630 (1180 to 2310)	1080 (759 to 1600)	997 (720 to 1480)	969 (538 to 1560)	1070 (751 to 1580)	895 (509 to 1430)	-44·9 (-64·5 to -29·6)
	50-69 years	947 (714 to 1370)	854 (566 to 1380)	715 (464 to 1170)	737 (351 to 1340)	788 (519 to 1290)	575 (278 to 1060)	-39·5 (-71·0 to -11·2)

eTable 5. Risk deleted deaths due to all-form tuberculosis for alcohol use, smoking, and diabetes, and all three risk factors combined by age groups for 204 countries and territories in (2021) with percent change between 2015 observed deaths and 2021 all-risk deleted deaths.

Location	Age group	2015 Observed deaths	2021 Observed deaths	2021 Smoking deleted deaths	2021 Alcohol deleted deaths	2021 Diabetes deleted deaths	2021 All-risk deleted deaths	Percent Change
	70+ years	455 (359 to 589)	439 (325 to 641)	409 (303 to 614)	396 (229 to 609)	366 (267 to 525)	308 (169 to 468)	-32·5 (-60·7 to -11·8)

eTable 6. Observed and expected deaths due to tuberculosis without HIV coinfection in 2020 and 2021 by age for countries with available cause-specific vital registration data

Location	Year	Age group	Observed deaths	Expected deaths	Excess tuberculosis deaths	Observed to expected ratio
All locations (41) in 2020	2020	All-ages	45500	50900 (49700 to 52400)	-5340 (-6920 to -4070)	0.895 (0.868 to 0.918)
All locations (41) in 2020	2020	Under 65 years	27800	28800 (28000 to 30000)	-1000 (-2150 to -127)	0.965 (0.928 to 0.995)
All locations (41) in 2020	2020	65 years and greater	17700	22000 (21200 to 23000)	-4330 (-5310 to -3370)	0.803 (0.769 to 0.841)
All locations (20) in 2021	2021	All-ages	39000	39600 (38300 to 41100)	-657 (-2180 to 713)	0.983 (0.947 to 1.02)
All locations (20) in 2021	2021	Under 65 years	26300	24000 (23000 to 25200)	2290 (1100 to 3370)	1.10 (1.04 to 1.15)
All locations (20) in 2021	2021	65 years and greater	12600	15600 (14800 to 16500)	-2950 (-3920 to -2100)	0.811 (0.763 to 0.858)
Malaysia	2020	All-ages	886	999 (893 to 1120)	-113 (-229 to 12)	0.887 (0.787 to 1.01)
Malaysia	2020	Under 65 years	608	672 (591 to 763)	-64 (-166 to 37)	0.905 (0.78 to 1.06)
Malaysia	2020	65 years and greater	278	329 (277 to 391)	-51 (-111 to 6)	0.845 (0.699 to 1.02)
Philippines	2020	All-ages	19500	22700 (21400 to 24200)	-3240 (-4610 to -1920)	0.858 (0.809 to 0.910)
Philippines	2020	Under 65 years	12100	13100 (12300 to 13900)	-998 (-1810 to -228)	0.924 (0.87 to 0.982)
Philippines	2020	65 years and greater	7370	9680 (9030 to 10400)	-2310 (-2990 to -1630)	0.761 (0.709 to 0.819)
Philippines	2021	All-ages	20000	22100 (20400 to 23900)	-2120 (-4060 to -312)	0.904 (0.831 to 0.985)
Philippines	2021	Under 65 years	13200	12600 (11700 to 13600)	585 (-447 to 1550)	1.05 (0.967 to 1.13)
Philippines	2021	65 years and greater	6780	9530 (8720 to 10400)	-2760 (-3620 to -1930)	0.711 (0.651 to 0.779)
Armenia	2020	All-ages	46	25 (18 to 33)	21 (7 to 37)	1.88 (1.25 to 2.84)
Armenia	2020	Under 65 years	35	21 (17 to 25)	14 (4 to 29)	1.68 (1.16 to 2.46)
Armenia	2020	65 years and greater	11	4 (2 to 9)	7 (0 to 15)	2.50 (1.04 to 6.19)
Armenia	2021	All-ages	15	20 (14 to 30)	-5 (-16 to 8)	0.746 (0.393 to 1.49)
Armenia	2021	Under 65 years	9	18 (14 to 23)	-9 (-16 to 0)	0.499 (0.243 to 0.989)
Armenia	2021	65 years and greater	6	3 (1 to 8)	3 (-2 to 11)	1.91 (0.636 to 6.48)
Georgia	2020	All-ages	72	100 (78 to 129)	-28 (-61 to 2)	0.718 (0.508 to 1.02)
Georgia	2020	Under 65 years	41	53 (34 to 83)	-12 (-43 to 14)	0.774 (0.457 to 1.34)
Georgia	2020	65 years and greater	31	51 (39 to 66)	-20 (-40 to -3)	0.609 (0.375 to 0.935)
Kazakhstan	2020	All-ages	381	376 (351 to 402)	5 (-43 to 53)	1.01 (0.888 to 1.14)
Kazakhstan	2020	Under 65 years	314	320 (305 to 335)	-6 (-43 to 33)	0.982 (0.871 to 1.10)
Kazakhstan	2020	65 years and greater	67	57 (44 to 74)	10 (-10 to 33)	1.17 (0.843 to 1.68)
Kazakhstan	2021	All-ages	356	334 (306 to 364)	22 (-21 to 69)	1.07 (0.939 to 1.22)
Kazakhstan	2021	Under 65 years	293	281 (265 to 299)	12 (-27 to 52)	1.04 (0.908 to 1.19)
Kazakhstan	2021	65 years and greater	63	54 (39 to 76)	9 (-17 to 32)	1.16 (0.760 to 1.77)
Mongolia	2020	All-ages	215	241 (173 to 336)	-26 (-124 to 53)	0.891 (0.616 to 1.28)
Mongolia	2020	Under 65 years	181	214 (158 to 289)	-33 (-106 to 30)	0.848 (0.62 to 1.19)
Mongolia	2020	65 years and greater	34	29 (18 to 46)	5 (-14 to 23)	1.19 (0.660 to 2.10)
Mongolia	2021	All-ages	179	239 (153 to 372)	-60 (-190 to 27)	0.749 (0.476 to 1.17)
Mongolia	2021	Under 65 years	151	214 (142 to 321)	-63 (-165 to 13)	0.706 (0.462 to 1.09)
Mongolia	2021	65 years and greater	28	27 (14 to 51)	1 (-25 to 19)	1.05 (0.512 to 2.14)
Bosnia and Herzegovina	2020	All-ages	58	50 (36 to 68)	8 (-15 to 30)	1.16 (0.760 to 1.74)
Bosnia and Herzegovina	2020	Under 65 years	13	13 (8 to 22)	0 (-11 to 11)	1.02 (0.453 to 2.17)
Bosnia and Herzegovina	2020	65 years and greater	45	37 (30 to 46)	8 (-7 to 24)	1.21 (0.832 to 1.72)
Bulgaria	2020	All-ages	74	90 (77 to 104)	-16 (-36 to 7)	0.823 (0.635 to 1.08)
Bulgaria	2020	Under 65 years	50	54 (44 to 65)	-4 (-20 to 14)	0.933 (0.672 to 1.28)

eTable 6. Observed and expected deaths due to tuberculosis without HIV coinfection in 2020 and 2021 by age for countries with available cause-specific vital registration data

Location	Year	Age group	Observed deaths	Expected deaths	Excess tuberculosis deaths	Observed to expected ratio
Bulgaria	2020	65 years and greater	24	36 (26 to 50)	-12 (-27 to 3)	0.66 (0.408 to 1.10)
Croatia	2020	All-ages	26	40 (22 to 74)	-14 (-44 to 7)	0.647 (0.332 to 1.30)
Croatia	2020	Under 65 years	6	15 (8 to 26)	-9 (-20 to 2)	0.403 (0.144 to 1.19)
Croatia	2020	65 years and greater	20	26 (13 to 49)	-6 (-30 to 11)	0.783 (0.357 to 1.75)
Czechia	2020	All-ages	24	21 (15 to 30)	3 (-8 to 16)	1.13 (0.689 to 1.90)
Czechia	2020	Under 65 years	9	13 (8 to 21)	-4 (-13 to 5)	0.690 (0.320 to 1.54)
Czechia	2020	65 years and greater	15	9 (6 to 12)	6 (-1 to 16)	1.68 (0.933 to 3.05)
Czechia	2021	All-ages	36	18 (12 to 27)	18 (5 to 34)	2.05 (1.22 to 3.57)
Czechia	2021	Under 65 years	14	12 (7 to 22)	2 (-8 to 13)	1.16 (0.559 to 2.61)
Czechia	2021	65 years and greater	22	7 (4 to 10)	15 (7 to 27)	3.28 (1.82 to 5.64)
Poland	2020	All-ages	481	467 (426 to 513)	14 (-45 to 73)	1.03 (0.912 to 1.17)
Poland	2020	Under 65 years	288	277 (243 to 316)	11 (-39 to 55)	1.04 (0.872 to 1.21)
Poland	2020	65 years and greater	193	189 (177 to 203)	4 (-26 to 34)	1.02 (0.867 to 1.18)
Poland	2021	All-ages	457	450 (399 to 506)	7 (-60 to 71)	1.02 (0.882 to 1.17)
Poland	2021	Under 65 years	306	264 (224 to 313)	42 (-15 to 97)	1.16 (0.950 to 1.42)
Poland	2021	65 years and greater	151	184 (168 to 201)	-33 (-59 to -4)	0.822 (0.69 to 0.979)
Serbia	2020	All-ages	43	51 (34 to 78)	-8 (-35 to 15)	0.836 (0.501 to 1.41)
Serbia	2020	Under 65 years	16	25 (19 to 33)	-9 (-18 to 3)	0.645 (0.376 to 1.17)
Serbia	2020	65 years and greater	27	27 (15 to 49)	0 (-23 to 18)	0.985 (0.510 to 1.92)
Serbia	2021	All-ages	36	46 (27 to 79)	-10 (-43 to 13)	0.78 (0.425 to 1.44)
Serbia	2021	Under 65 years	21	24 (17 to 35)	-3 (-17 to 10)	0.865 (0.495 to 1.54)
Serbia	2021	65 years and greater	15	23 (11 to 48)	-8 (-34 to 8)	0.648 (0.267 to 1.62)
Latvia	2020	All-ages	58	61 (47 to 80)	-3 (-26 to 18)	0.948 (0.647 to 1.35)
Latvia	2020	Under 65 years	32	33 (23 to 48)	-1 (-19 to 17)	0.959 (0.588 to 1.66)
Latvia	2020	65 years and greater	26	29 (23 to 37)	-3 (-14 to 11)	0.896 (0.578 to 1.44)
Latvia	2021	All-ages	62	57 (41 to 80)	5 (-21 to 28)	1.09 (0.708 to 1.64)
Latvia	2021	Under 65 years	41	29 (18 to 47)	12 (-8 to 31)	1.39 (0.815 to 2.49)
Latvia	2021	65 years and greater	21	30 (22 to 40)	-9 (-21 to 5)	0.708 (0.414 to 1.18)
Lithuania	2020	All-ages	101	92 (79 to 108)	9 (-16 to 34)	1.09 (0.830 to 1.40)
Lithuania	2020	Under 65 years	72	60 (52 to 68)	12 (-5 to 32)	1.21 (0.927 to 1.56)
Lithuania	2020	65 years and greater	29	33 (25 to 45)	-4 (-18 to 11)	0.875 (0.561 to 1.40)
Lithuania	2021	All-ages	97	79 (65 to 97)	18 (-7 to 42)	1.22 (0.917 to 1.60)
Lithuania	2021	Under 65 years	74	50 (42 to 59)	24 (5 to 43)	1.47 (1.09 to 1.95)
Lithuania	2021	65 years and greater	23	30 (20 to 43)	-7 (-22 to 8)	0.775 (0.449 to 1.34)
Russia	2020	All-ages	6660	6200 (5890 to 6520)	467 (105 to 813)	1.08 (1.02 to 1.14)
Russia	2020	Under 65 years	5570	5090 (4800 to 5410)	480 (121 to 806)	1.09 (1.02 to 1.17)
Russia	2020	65 years and greater	1090	1150 (1090 to 1220)	-61 (-149 to 32)	0.947 (0.875 to 1.03)
Russia	2021	All-ages	6120	5330 (5000 to 5690)	795 (408 to 1180)	1.15 (1.07 to 1.23)
Russia	2021	Under 65 years	5020	4310 (3990 to 4640)	715 (359 to 1040)	1.17 (1.08 to 1.26)
Russia	2021	65 years and greater	1100	1110 (1030 to 1180)	-3 (-106 to 96)	0.997 (0.91 to 1.09)
Ukraine	2020	All-ages	180	75 (6 to 943)	106 (-808 to 184)	2.42 (0.173 to 30.2)

eTable 6. Observed and expected deaths due to tuberculosis without HIV coinfection in 2020 and 2021 by age for countries with available cause-specific vital registration data

Location	Year	Age group	Observed deaths	Expected deaths	Excess tuberculosis deaths	Observed to expected ratio
Ukraine	2020	Under 65 years	154	66 (5 to 846)	88 (-655 to 159)	2.33 (0.192 to 31.7)
Ukraine	2020	65 years and greater	26	9 (1 to 99)	18 (-105 to 33)	3.05 (0.194 to 35.3)
Ukraine	2021	All-ages	187	31 (1 to 677)	156 (-321 to 203)	6.10 (0.365 to 157)
Ukraine	2021	Under 65 years	165	27 (1 to 607)	138 (-375 to 179)	6.11 (0.306 to 116)
Ukraine	2021	65 years and greater	22	4 (0 to 73)	18 (-52 to 31)	5.97 (0.306 to 138)
Japan	2020	All-ages	2560	3250 (2690 to 3930)	-691 (-1430 to -106)	0.787 (0.638 to 0.962)
Japan	2020	Under 65 years	75	76 (58 to 99)	-1 (-29 to 25)	0.984 (0.682 to 1.39)
Japan	2020	65 years and greater	2480	3150 (2620 to 3790)	-667 (-1310 to -94)	0.788 (0.655 to 0.964)
Republic of Korea	2020	All-ages	1440	1590 (1460 to 1740)	-154 (-314 to -9)	0.903 (0.820 to 0.994)
Republic of Korea	2020	Under 65 years	256	259 (229 to 293)	-3 (-46 to 40)	0.987 (0.838 to 1.17)
Republic of Korea	2020	65 years and greater	1180	1370 (1230 to 1520)	-184 (-341 to -19)	0.865 (0.770 to 0.985)
Republic of Korea	2021	All-ages	1510	1460 (1310 to 1630)	51 (-126 to 229)	1.03 (0.923 to 1.17)
Republic of Korea	2021	Under 65 years	272	225 (193 to 263)	47 (4 to 93)	1.21 (1.02 to 1.46)
Republic of Korea	2021	65 years and greater	1240	1280 (1120 to 1470)	-45 (-248 to 133)	0.965 (0.829 to 1.12)
Australia	2020	All-ages	32	48 (40 to 57)	-16 (-29 to -1)	0.665 (0.453 to 0.985)
Australia	2020	Under 65 years	7	11 (5 to 23)	-4 (-18 to 5)	0.619 (0.215 to 1.70)
Australia	2020	65 years and greater	25	37 (27 to 50)	-12 (-27 to 4)	0.679 (0.415 to 1.12)
Australia	2021	All-ages	48	48 (38 to 60)	0 (-17 to 18)	1.01 (0.696 to 1.41)
Australia	2021	Under 65 years	9	11 (4 to 29)	-2 (-20 to 9)	0.79 (0.255 to 2.55)
Australia	2021	65 years and greater	39	36 (24 to 54)	3 (-18 to 22)	1.07 (0.644 to 1.80)
Austria	2020	All-ages	40	33 (19 to 55)	7 (-17 to 26)	1.22 (0.668 to 2.16)
Austria	2020	Under 65 years	10	6 (3 to 11)	4 (-3 to 13)	1.76 (0.752 to 4.38)
Austria	2020	65 years and greater	30	27 (17 to 44)	3 (-16 to 20)	1.10 (0.602 to 2.07)
Austria	2021	All-ages	30	30 (15 to 58)	0 (-27 to 18)	1.01 (0.481 to 2.02)
Austria	2021	Under 65 years	11	5 (2 to 12)	6 (-2 to 15)	2.25 (0.802 to 6.40)
Austria	2021	65 years and greater	19	25 (14 to 47)	-6 (-29 to 9)	0.750 (0.342 to 1.60)
Finland	2020	All-ages	21	29 (22 to 38)	-8 (-20 to 5)	0.715 (0.423 to 1.17)
Finland	2020	Under 65 years	4	0 (0 to 2)	4 (1 to 11)	18.9 (2.13 to 194)
Finland	2020	65 years and greater	17	29 (21 to 38)	-12 (-24 to 1)	0.595 (0.327 to 1.07)
Germany	2020	All-ages	158	278 (262 to 294)	-120 (-150 to -90)	0.569 (0.473 to 0.67)
Germany	2020	Under 65 years	17	62 (43 to 92)	-45 (-75 to -24)	0.272 (0.148 to 0.495)
Germany	2020	65 years and greater	141	217 (199 to 236)	-76 (-106 to -46)	0.651 (0.538 to 0.779)
Greece	2020	All-ages	34	45 (34 to 58)	-11 (-27 to 6)	0.759 (0.493 to 1.17)
Greece	2020	Under 65 years	14	18 (12 to 27)	-4 (-14 to 8)	0.784 (0.420 to 1.56)
Greece	2020	65 years and greater	20	27 (21 to 35)	-7 (-18 to 5)	0.734 (0.445 to 1.21)
Italy	2020	All-ages	226	257 (245 to 270)	-31 (-62 to 2)	0.88 (0.764 to 1.01)
Italy	2020	Under 65 years	62	74 (61 to 91)	-12 (-34 to 11)	0.835 (0.607 to 1.17)
Italy	2020	65 years and greater	164	185 (172 to 200)	-21 (-48 to 9)	0.884 (0.75 to 1.05)
Netherlands	2020	All-ages	32	19 (14 to 25)	13 (3 to 28)	1.72 (1.10 to 2.68)
Netherlands	2020	Under 65 years	8	6 (3 to 14)	2 (-7 to 11)	1.23 (0.423 to 3.65)
Netherlands	2020	65 years and greater	24	13 (7 to 23)	11 (-1 to 24)	1.85 (0.949 to 3.62)

eTable 6. Observed and expected deaths due to tuberculosis without HIV coinfection in 2020 and 2021 by age for countries with available cause-specific vital registration data

Location	Year	Age group	Observed deaths	Expected deaths	Excess tuberculosis deaths	Observed to expected ratio
Spain	2020	All-ages	194	220 (199 to 242)	-26 (-55 to 11)	0.884 (0.762 to 1.05)
Spain	2020	Under 65 years	55	60 (43 to 83)	-5 (-33 to 20)	0.917 (0.599 to 1.45)
Spain	2020	65 years and greater	139	161 (146 to 177)	-22 (-48 to 8)	0.865 (0.716 to 1.05)
United Kingdom	2020	All-ages	192	200 (181 to 220)	-8 (-40 to 29)	0.962 (0.816 to 1.15)
United Kingdom	2020	Under 65 years	71	52 (51 to 54)	19 (4 to 37)	1.36 (1.07 to 1.71)
United Kingdom	2020	65 years and greater	121	147 (129 to 168)	-26 (-57 to 1)	0.821 (0.654 to 1.00)
United Kingdom	2021	All-ages	185	181 (159 to 204)	4 (-30 to 41)	1.02 (0.845 to 1.25)
United Kingdom	2021	Under 65 years	63	48 (46 to 49)	15 (1 to 32)	1.32 (1.03 to 1.68)
United Kingdom	2021	65 years and greater	122	133 (112 to 157)	-11 (-41 to 22)	0.919 (0.722 to 1.18)
Argentina	2020	All-ages	562	595 (551 to 642)	-33 (-97 to 38)	0.945 (0.847 to 1.07)
Argentina	2020	Under 65 years	349	359 (318 to 406)	-11 (-71 to 45)	0.971 (0.826 to 1.14)
Argentina	2020	65 years and greater	213	237 (217 to 258)	-23 (-57 to 12)	0.901 (0.772 to 1.05)
Chile	2020	All-ages	250	283 (251 to 319)	-33 (-78 to 12)	0.884 (0.745 to 1.05)
Chile	2020	Under 65 years	98	126 (114 to 140)	-28 (-51 to -5)	0.775 (0.629 to 0.961)
Chile	2020	65 years and greater	152	158 (136 to 184)	-6 (-38 to 28)	0.960 (0.778 to 1.20)
Chile	2021	All-ages	288	257 (221 to 299)	31 (-23 to 80)	1.12 (0.921 to 1.35)
Chile	2021	Under 65 years	158	122 (108 to 139)	36 (6 to 66)	1.29 (1.05 to 1.58)
Chile	2021	65 years and greater	130	139 (115 to 168)	-9 (-43 to 25)	0.935 (0.726 to 1.2)
Uruguay	2020	All-ages	71	63 (46 to 87)	8 (-21 to 33)	1.12 (0.744 to 1.67)
Uruguay	2020	Under 65 years	38	36 (25 to 53)	2 (-18 to 20)	1.04 (0.644 to 1.68)
Uruguay	2020	65 years and greater	33	27 (21 to 36)	6 (-8 to 20)	1.21 (0.758 to 1.86)
United States of America	2020	All-ages	624	571 (532 to 613)	53 (-10 to 112)	1.09 (0.983 to 1.20)
United States of America	2020	Under 65 years	241	189 (151 to 235)	52 (2 to 100)	1.28 (1.01 to 1.63)
United States of America	2020	65 years and greater	383	383 (349 to 421)	0 (-57 to 49)	0.999 (0.861 to 1.14)
Cuba	2020	All-ages	35	27 (22 to 34)	8 (-4 to 23)	1.29 (0.876 to 1.93)
Cuba	2020	Under 65 years	23	17 (13 to 23)	6 (-4 to 18)	1.32 (0.822 to 2.13)
Cuba	2020	65 years and greater	12	10 (7 to 14)	2 (-4 to 11)	1.23 (0.663 to 2.36)
Ecuador	2020	All-ages	410	473 (416 to 536)	-63 (-137 to 8)	0.868 (0.739 to 1.02)
Ecuador	2020	Under 65 years	285	297 (269 to 327)	-12 (-54 to 34)	0.960 (0.832 to 1.12)
Ecuador	2020	65 years and greater	125	174 (144 to 209)	-49 (-90 to -9)	0.720 (0.558 to 0.938)
Ecuador	2021	All-ages	542	476 (405 to 560)	66 (-26 to 152)	1.14 (0.951 to 1.36)
Ecuador	2021	Under 65 years	388	303 (268 to 344)	85 (29 to 139)	1.28 (1.09 to 1.51)
Ecuador	2021	65 years and greater	154	169 (133 to 215)	-15 (-66 to 27)	0.911 (0.684 to 1.19)
Peru	2020	All-ages	1360	2180 (1820 to 2600)	-812 (-1230 to -450)	0.627 (0.523 to 0.758)
Peru	2020	Under 65 years	816	1200 (1020 to 1420)	-387 (-599 to -182)	0.678 (0.570 to 0.817)
Peru	2020	65 years and greater	549	963 (779 to 1190)	-414 (-645 to -226)	0.570 (0.455 to 0.713)
Colombia	2020	All-ages	1010	1210 (1120 to 1310)	-204 (-318 to -92)	0.832 (0.753 to 0.918)
Colombia	2020	Under 65 years	554	623 (536 to 723)	-69 (-179 to 30)	0.890 (0.748 to 1.05)
Colombia	2020	65 years and greater	456	586 (570 to 603)	-130 (-171 to -81)	0.778 (0.711 to 0.861)
Colombia	2021	All-ages	1190	1250 (1130 to 1380)	-62 (-204 to 76)	0.951 (0.848 to 1.07)
Colombia	2021	Under 65 years	703	638 (527 to 774)	65 (-83 to 190)	1.10 (0.889 to 1.35)

eTable 6. Observed and expected deaths due to tuberculosis without HIV coinfection in 2020 and 2021 by age for countries with available cause-specific vital registration data

Location	Year	Age group	Observed deaths	Expected deaths	Excess tuberculosis deaths	Observed to expected ratio
Colombia	2021	65 years and greater	485	604 (583 to 626)	-119 (-166 to -73)	0.803 (0.73 to 0.877)
Costa Rica	2020	All-ages	35	42 (35 to 50)	-7 (-21 to 9)	0.836 (0.557 to 1.24)
Costa Rica	2020	Under 65 years	23	24 (16 to 36)	-1 (-14 to 13)	0.942 (0.541 to 1.68)
Costa Rica	2020	65 years and greater	12	18 (11 to 28)	-6 (-17 to 6)	0.677 (0.321 to 1.43)
Guatemala	2020	All-ages	183	290 (256 to 329)	-107 (-150 to -66)	0.630 (0.524 to 0.755)
Guatemala	2020	Under 65 years	146	207 (164 to 262)	-61 (-118 to -11)	0.706 (0.534 to 0.942)
Guatemala	2020	65 years and greater	37	83 (70 to 98)	-46 (-65 to -25)	0.444 (0.305 to 0.653)
Mexico	2020	All-ages	2110	2230 (2100 to 2370)	-121 (-292 to 32)	0.946 (0.878 to 1.02)
Mexico	2020	Under 65 years	1560	1590 (1540 to 1650)	-28 (-115 to 73)	0.982 (0.928 to 1.05)
Mexico	2020	65 years and greater	546	642 (563 to 731)	-96 (-191 to -8)	0.85 (0.733 to 0.987)
Mexico	2021	All-ages	2360	2230 (2060 to 2420)	125 (-65 to 326)	1.06 (0.973 to 1.16)
Mexico	2021	Under 65 years	1750	1620 (1550 to 1690)	129 (25 to 242)	1.08 (1.01 to 1.15)
Mexico	2021	65 years and greater	610	615 (521 to 726)	-5 (-132 to 96)	0.992 (0.815 to 1.18)
Nicaragua	2020	All-ages	96	97 (83 to 113)	-1 (-23 to 26)	0.992 (0.782 to 1.29)
Nicaragua	2020	Under 65 years	62	74 (58 to 95)	-12 (-37 to 10)	0.837 (0.595 to 1.17)
Nicaragua	2020	65 years and greater	34	23 (17 to 33)	11 (-3 to 25)	1.45 (0.907 to 2.26)
Brazil	2020	All-ages	4830	4840 (4640 to 5060)	-16 (-272 to 210)	0.997 (0.946 to 1.04)
Brazil	2020	Under 65 years	3380	3260 (3130 to 3390)	125 (-44 to 301)	1.04 (0.987 to 1.10)
Brazil	2020	65 years and greater	1450	1580 (1490 to 1690)	-138 (-265 to -13)	0.913 (0.842 to 0.991)
Brazil	2021	All-ages	5320	4840 (4580 to 5110)	480 (165 to 780)	1.10 (1.03 to 1.17)
Brazil	2021	Under 65 years	3700	3240 (3070 to 3400)	462 (266 to 672)	1.14 (1.08 to 1.22)
Brazil	2021	65 years and greater	1620	1590 (1470 to 1730)	26 (-128 to 176)	1.02 (0.923 to 1.12)
Paraguay	2020	All-ages	214	227 (184 to 279)	-13 (-72 to 41)	0.945 (0.736 to 1.21)
Paraguay	2020	Under 65 years	145	141 (110 to 180)	4 (-41 to 43)	1.03 (0.766 to 1.37)
Paraguay	2020	65 years and greater	69	86 (69 to 107)	-17 (-41 to 8)	0.804 (0.593 to 1.11)
